# Research trends on neuroinflammation in Parkinson’s disease: an exploratory analysis

**DOI:** 10.3389/fnagi.2026.1699038

**Published:** 2026-02-04

**Authors:** Yan-Jun Chen, Ming-Rong Xie, Fang Liu, Sheng-Qiang Zhou, Bo Li

**Affiliations:** 1 The First Hospital of Hunan University of Chinese Medicine, Changsha, China; 2 Graduate School of Hunan University of Chinese Medicine, Changsha, China; 3 The First Clinical College of Nanjing University of Chinese Medicine, Nanjing, China; 4 National TCM Master Liu Zuyi Inheritance Studio, Hunan Provincial Hospital of Integrated Traditional Chinese and Western Medicine (The Affiliated Hospital of Hunan Academy of Traditional Chinese Medicine), Changsha, China

**Keywords:** alpha-synuclein, gut microbiota, microglia, neuroinflammation, oxidative stress, Parkinson’s disease

## Abstract

**Background:**

Neuroinflammation is a core mechanism in the pathogenesis of Parkinson’s disease (PD). Research on PD and neuroinflammation has been steadily increasing. This study aims to explore and analyze the current status, hotspots, and future directions of neuroinflammation-associated research in PD.

**Method:**

Data were retrieved from the Web of Science and PubMed databases. CiteSpace, VOSviewer, and the R package bibliometrix were used for data analysis and visualization.

**Results:**

Publications on neuroinflammation in PD showed an upward trend. China had the most publications, while Norway exhibited the greatest influence. Shanghai Jiao Tong University was the most prolific institution, whereas Arizona State University demonstrated the strongest influence. The International *Journal of Molecular Sciences* was the most prolific journal in this field, and *Cell* had the strongest impact. The most productive author was Hong, Jau-Shyong, and the most influential author was Block, Michelle L. High-frequency keywords included PD, neuroinflammation, microglia, oxidative stress, alpha-synuclein, neurodegeneration, inflammation, and mouse models. Gut microbiota has become a recent focus of research.

**Conclusion:**

Over the past two decades, research on neuroinflammation in PD has been continuously increasing. The research hotspots included microglia, oxidative stress, *α*-synuclein, brain-gut axis, and gut microbiota. This field is developing toward a multi-dimensional and in-depth exploration of mechanisms.

## 1 Introduction

Neuroinflammation refers to the response of reactive central nervous system (CNS) components to disruptions in homeostasis resulting from endogenous or exogenous stimuli. The primary function of this response is to protect the CNS and facilitate tissue repair. However, when the inflammatory reaction becomes excessive, it can induce neuronal damage and accelerate neurodegenerative processes, thereby playing a significant role in various neurological disorders (Ransohoff, 2016). In recent years, neuroinflammation has emerged as a prominent research focus within neuroscience due to its implications in the pathogenesis, progression, and potential therapeutic targeting of numerous neurodegenerative diseases (Zhang et al., 2023).

Parkinson’s disease (PD) is a progressive neurodegenerative disorder characterized by resting tremor, bradykinesia, rigidity, and postural instability (Kalia and Lang, 2015). The core pathological features of PD include the loss of dopaminergic neurons in the substantia nigra of the midbrain and the formation of Lewy bodies (Surmeier, 2018). Lewy bodies are primarily composed of aggregated misfolded *α*-synuclein protein. The abnormal accumulation of this protein not only disrupts neuronal function but also exacerbates disease progression by triggering neuroinflammation and impairing synaptic transmission (Burré et al., 2024). The pathogenesis involves a complex interplay of multiple factors, including genetic mutations, mitochondrial dysfunction, oxidative stress, and neuroinflammation (Ebadpour et al., 2024). Neuroinflammation plays a pivotal role in both the pathogenesis and progression of PD. Studies have demonstrated a marked inflammatory response in the brains of PD patients, evidenced by microglial activation (McGeer et al., 1988) and the release of inflammatory factors (Williams-Gray et al., 2016). These responses may not only directly damage neurons but also exacerbate neuronal injury and death by altering neurotransmitter release and compromising blood–brain barrier (BBB) integrity, ultimately leading to irreversible neurological damage (Tansey and Goldberg, 2010). Furthermore, neuroinflammation may influence the expression and regulation of PD-associated genes, thereby contributing to disease mechanisms (Pajares et al., 2020). Consequently, suppressing neuroinflammation has been proposed as a potential therapeutic strategy for PD. In recent years, growing attention has been directed toward understanding the role of neuroinflammation in PD and exploring ways to modulate neuroinflammatory processes to alleviate symptoms and alter disease progression. These investigations have not only advanced our understanding of PD pathogenesis but also provided a theoretical foundation for the development of novel treatments and pharmacological agents.

Bibliometric analysis is a scientific methodology that identifies developmental trends and research hotspots within a particular field through quantitative examination of published academic literature (Ninkov et al., 2022). Given the large number of published relevant literature and the continuous increase, conducting bibliometric and quantitative analysis on the research related to neuroinflammation in PD is both feasible and of great significance. This study aims to summarize existing research findings and advances in this area through a bibliometric analysis, thereby offering valuable insights and guidance for future investigations.

## 2 Methods

### 2.1 Data retrieval

**Figure 1:**
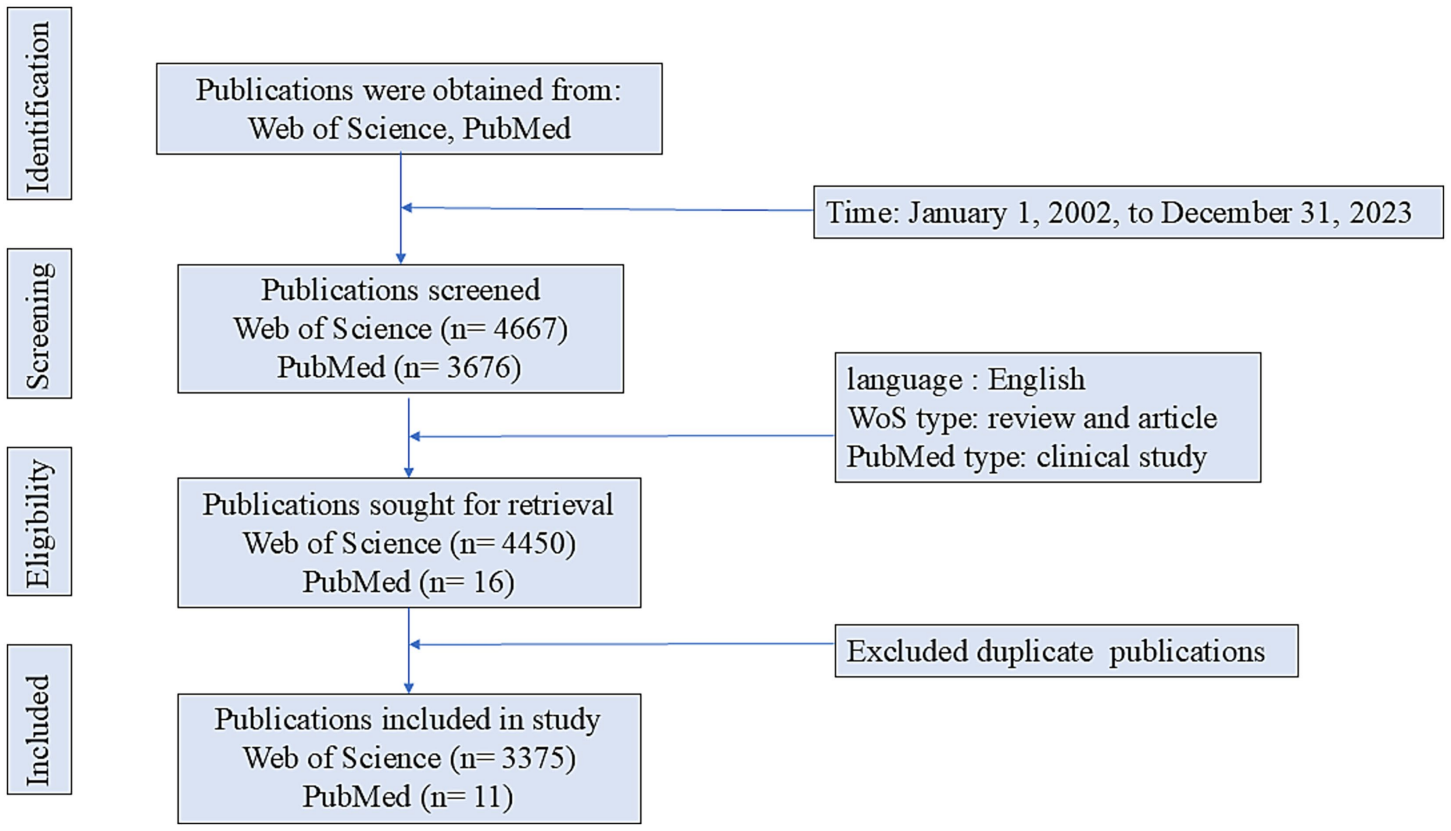
Flowchart of literature search.

The data for the bibliometric analysis were primarily retrieved from the Web of Science Core Collection database. This WoS dataset formed the basis for all visual analyses, including those of annual publication trends, countries, institutions, journals, authors, references, and keywords. Additionally, a supplementary search was performed in the PubMed database. The objective of this search was not to expand the bibliometric dataset, but to identify a select number of highly relevant clinical studies that could provide further context for discussing the research topic. It should be noted that publications retrieved from PubMed were not incorporated into the WoS dataset for formal bibliometric computations (e.g., co-occurrence analysis, clustering). Instead, they were cited individually to support specific clinical points in the discussion. The specific search strategy used in WoS was (((TS = (neurological inflammation)) OR TS = (neurogenic inflammation)) OR TS = (neuroinflammation)) AND TS = (Parkinson’s disease). The search period spanned from January 1, 2002, to December 31, 2023. The publication language was restricted to English, and only articles and reviews were included. The PubMed search strategy was (((neurogenic inflammation[Title/Abstract]) OR (neurological inflammation[Title/Abstract])) OR (neuroinflammation[Title/Abstract]))) AND (Parkinson’s disease[Title/Abstract]). Publications were limited to the English language, and the study types were restricted to clinical studies. Two investigators independently screened the literature and excluded records irrelevant to the research topic. Ultimately, 3,375 publications from WoS and 11 clinical studies from PubMed were included in the analysis (Figure 1).

### 2.2 Data analysis

Data were visualized using CiteSpace, VOSviewer, and the R package “bibliometrix,” consistent with previously described methodologies (Chen et al., 2025a; Chen et al., 2025b). CiteSpace is a software tool designed for scientific citation analysis; it can reveal the structure and development trends within a research field, as well as evaluate the importance and influence of nodes in the literature network (Chen, 2004). VOSviewer enables multidimensional bibliometric analysis and offers multiple visualization modes for interpretation, making it widely valuable in bibliometric studies (van Eck and Waltman, 2010). Bibliometrix facilitates the graphical representation of bibliographic data, aiding researchers in intuitively understanding the characteristics and patterns within the literature dataset (Aria and Cuccurullo, 2017). The burst detection function and Bradford’s Law analysis are built-in analytical features of CiteSpace and bibliometrix, respectively. According to the official CiteSpace documentation and conventions, the default value of *γ* is generally set to 1.0. The bibliometrix R package includes built-in functions for conducting Bradford’s Law analysis, enabling users to directly generate a list of core journals related to the research topic along with the corresponding distribution graph.

## 3 Results

### 3.1 Publication growth trend

**Figure 2:**
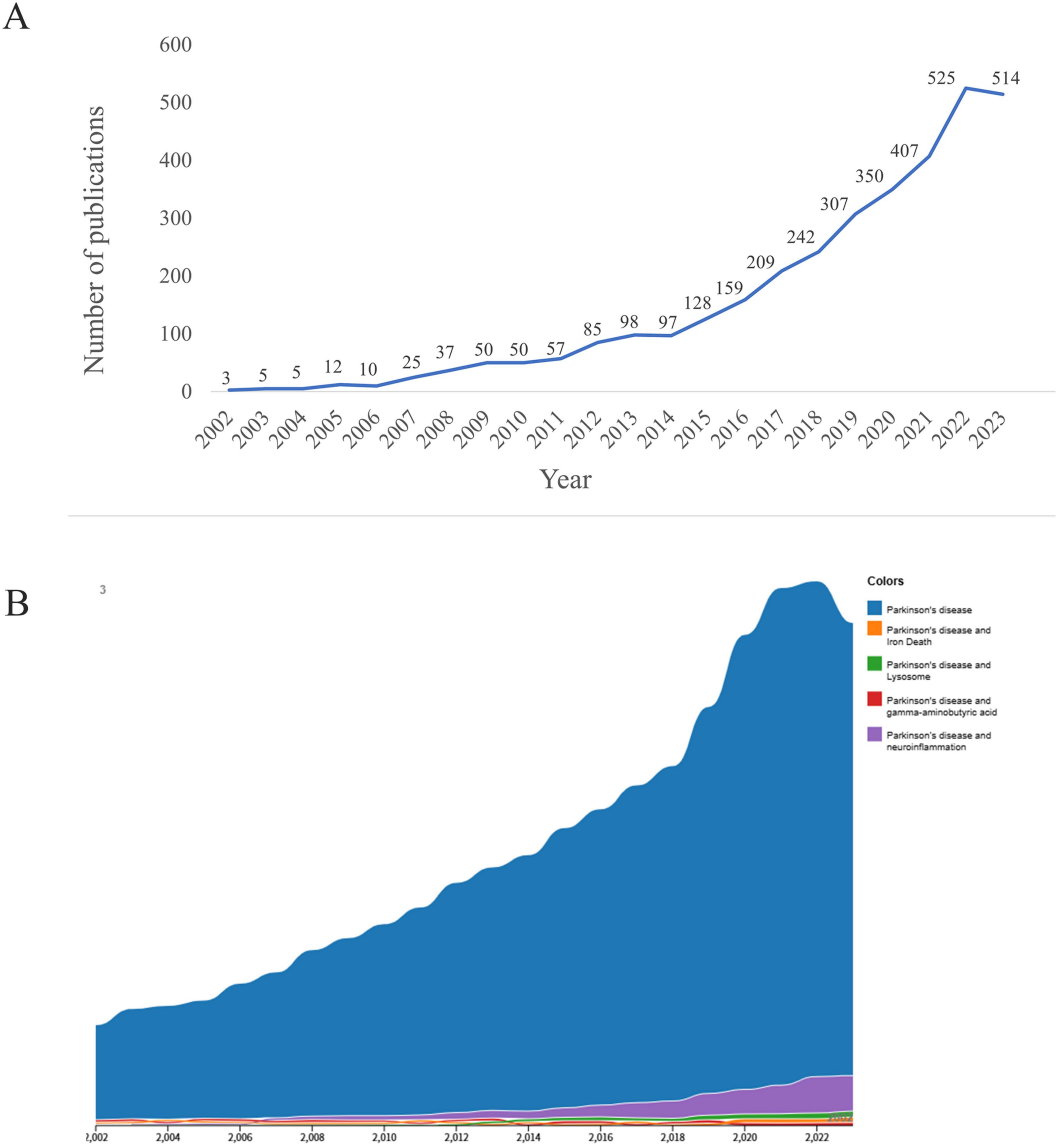
Analysis of publications. **(A)** Trends in PD and neuroinflammation publications from 2002 to 2023. **(B)** Proportion of research topics in PD publications.

The number of published papers reflects the level of research attention on a particular topic. Between 2002 and 2023, the number of publications related to PD and neuroinflammation increased gradually (Figures 2A,B). A rapid increase was observed since 2015, peaking at 525 publications in 2022. This indicates a growing interest in the research topic over time.

### 3.2 Geographic distribution and country

**Figure 3:**
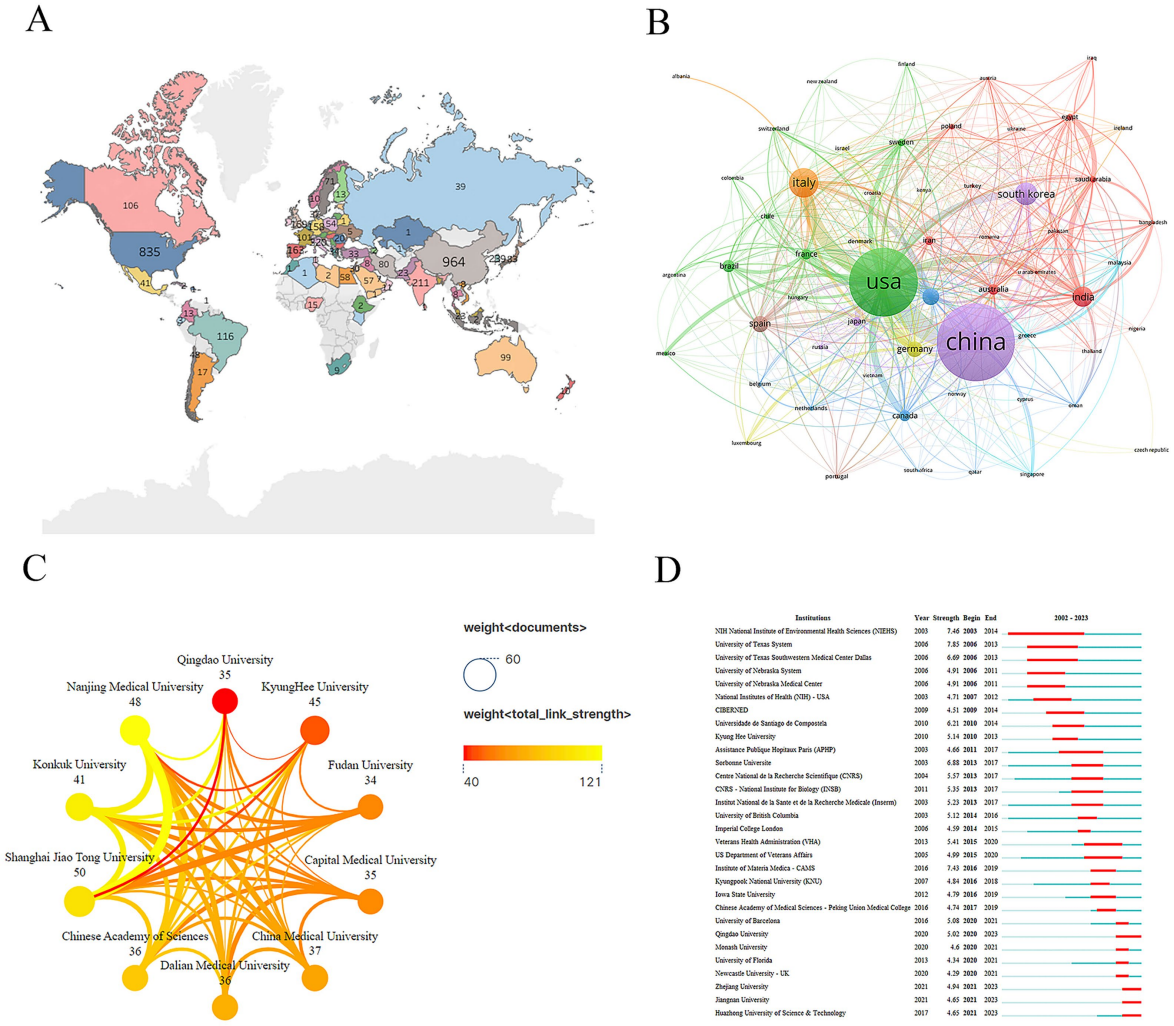
Analysis of countries and organizations. **(A)** The geographical distribution of different countries. **(B)** Network of collaboration between countries. **(C)** Graph of network collaboration among the top 10 institutions. **(D)** Organizations with the strongest citation bursts.

The geographical distribution analysis revealed that studies on PD and neuroinflammation were predominantly conducted in Asia, Europe, and North America, with relatively fewer contributions from Africa (Figure 3A; Table 1). In terms of the number of publications, China ranked first with 964 papers, followed by the United States (835 papers) and Italy (320 papers). In terms of publications’ influence, Norway had the highest quality (average number of citations: 126.3), followed by Chile (average number of citations: 89.65) and Sweden (average number of citations: 82.62) (Table 2). In the international collaboration network, the United States and China exhibited a relatively large node scale and a considerable number of connections, indicating their roles as central hubs. Italy, South Korea, and India also emerged as significant contributors in this field of research (Figure 3B). This collaborative pattern suggests that international collaboration may play an important role in the dissemination and innovation of neuroinflammation and PD research.

**Table 1:** Publications from each continent.

Rank	Continent	Number of publications
1	Asia	1,877
2	Europe	1,462
3	North America	986
4	South America	184
5	Oceania	109
6	Africa	94

**Table 2:** The top 20 countries in terms of the total number of papers and the average citations.

Rank	Total number of papers	Average citations
1	China (964)	Norway (126.3)
2	USA (835)	Chile (89.65)
3	Italy (320)	Sweden (82.62)
4	South Korea (239)	Mexico (79.89)
5	India (211)	USA (79.23)
6	United Kingdom (169)	Serbia (78.5)
7	Spain (163)	France (77.42)
8	Germany (158)	Denmark (76.57)
9	Brazil (116)	Ireland (67.2)
10	Canada (106)	Russia (67.19)
11	France (101)	United Kingdom (66.6)
12	Australia (99)	Cyprus (66.5)
13	Japan (83)	Israel (66.07)
14	Iran (80)	Switzerland (65.2)
15	Sweden (71)	Greece (64.265)
16	Egypt (58)	Hungary (62.5)
17	Saudi Arabia (57)	Sri Lanka (59)
18	Poland (54)	Portugal (58.84)
19	Chile (48)	Belgium (57.49)
20	Netherlands (43)	Romania (55.8)

### 3.3 Research organizations

A large number of organizations contributed to research on PD and neuroinflammation. In terms of the number of publications, Shanghai Jiao Tong University had the highest number of publications (58), followed by Kyung Hee University (45) and Nanjing Medical University (40) (Table 3). Among the top ten institutions, eight were from China and two from South Korea. The thickness of connecting lines in the collaboration network indicated the strength of institutional partnerships. Nanjing Medical University showed close collaboration with Konkuk University and Shanghai Jiao Tong University (Figure 3C). In terms of the influence of publications, Arizona State University has the highest influence (average number of citations: 761), followed by the Salk Institute for Biological Studies (average number of citations: 731) and the Instituto Nacional de Pediatría (average number of citations: 608) (Table 3). Recently, the emerging institutions included Qingdao University, Zhejiang University, Jiangnan University, and Huazhong University of Science and Technology (Figure 3D).

**Table 3:** The top 10 institutions in terms of the total number of papers and the average citations.

Rank	Total number of papers	Average citations
1	Shanghai Jiao Tong University (50)	Arizona State University (761)
2	Nanjing Medical University (48)	Salk Institute for Biological Studies (731)
3	KyungHee University (45)	Instituto Nacional de Pediatría (608)
4	Konkuk University (41)	California Institute of Technology (573.5)
5	China Medical University (37)	Biogen Inc. (504.33)
6	Chinese Academy of Sciences (36)	University of Wisconsin (452.4)
6	Dalian Medical University (36)	National Institute of Environmental Health Sciences (411.2)
8	Capital Medical University and Qingdao University (35)	Foundation for Research and Technology - Hellas (376.67)
9	Fudan University (34)	Hanyang University (351.33)
10	Harvard Medical School (32)	University of Montana (322.86)

### 3.4 Journals and co-cited journals

**Figure 4:**
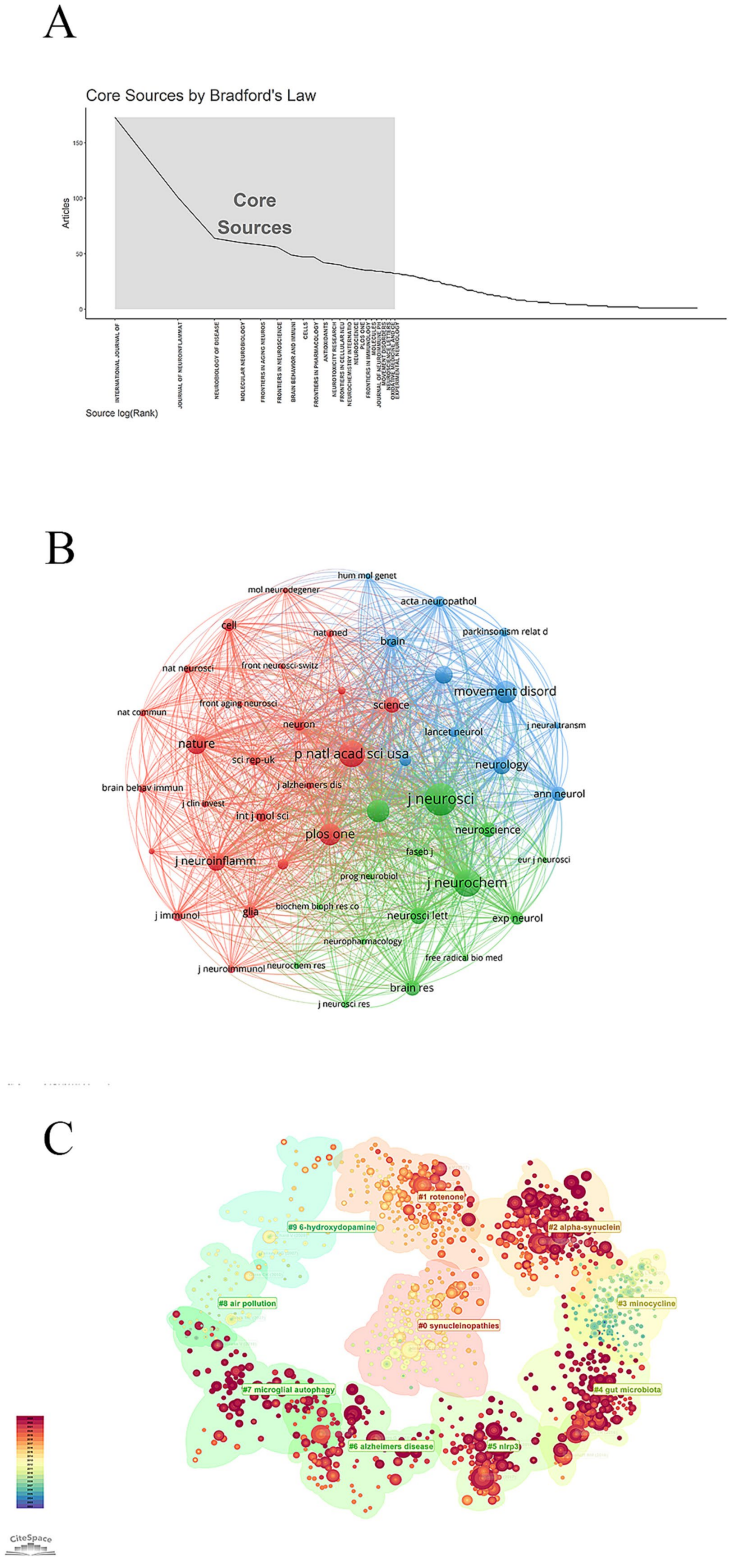
Analysis of journals and references. **(A)** Core journal. **(B)** Graph of network collaboration among co-cited journals. **(C)** Reference cluster analysis.

Based on Bradford’s Law (Bradford, 1934), 22 core journals were identified (Figure 4A). These journals specialized in neuroscience, neurobiology, immunology, pharmacology, and interdisciplinary areas, focusing primarily on the nervous system and related disorders. The top three journals by publication volume were the *International Journal of Molecular Sciences* (173 publications), the *Journal of Neuroinflammation* (101 publications), and *Neurobiology of Disease* (64 publications). In terms of journal influence, *Cell* ranked first (average number of citations: 2421), followed by *Science* (average number of citations: 1285), and *Lancet Neurology* (average number of citations: 970) (Table 4).

**Table 4:** The top 10 journals in terms of the total number of papers and the average citations.

Rank	Total number of papers	Average citations
1	International Journal of Molecular Sciences (173)	Cell (2421)
2	Journal of Neuroinflammation (101)	Science (1285)
3	Neurobiology of Disease (64)	Lancet Neurology (970)
4	Molecular Neurobiology (60)	Toxicologic Pathology (686)
5	Frontiers in Aging Neuroscience (58)	Nature Neuroscience (674.67)
6	Frontiers in Neuroscience (56)	Nature Clinical Practice Neurology (618)
7	Brain Behavior and Immunity (49)	Journal of Pharmacology and Experimental Therapeutics (507)
8	Cells (47)	Trends in Neurosciences (478.34)
9	Frontiers in Pharmacology (47)	Journal of Controlled Release (473.4)
10	Antioxidants (42)	Annals of Neurology (328)

The co-cited journal network was shown in Figure 4B. Nodes were color-coded by cluster: red nodes primarily represented immune and neural basis research, green nodes indicated neuropathology and movement disorders, and blue nodes corresponded to neurochemistry and neuroscience. The top three co-cited journals were the *Journal of Neuroscience* (7,593 citations), the *Journal of Neurochemistry* (6,338 citations), and *Proceedings of the National Academy of Sciences of the United States of America* (6,279 citations) (Table 5).

**Table 5:** The top 10 co-cited journals.

Rank	Source	Citations	IF	JCR
1	Journal of Neuroscience	7,593	4.4	Q1
2	Journal of Neurochemistry	6,338	4.2	Q2
3	Proceedings of the National Academy of Sciences of The United States of America	6,279	9.4	Q1
4	Movement Disorders	5,293	7.4	Q1
5	Journal of Biological Chemistry	5,248	4	Q2
6	Plos One	5,190	2.9	Q1
7	Nature	4,685	50.5	Q1
8	Journal of Neuroinflammation	4,348	9.3	Q1
9	Neurology	4,218	7.7	Q1
10	Neurobiology of Disease	4,201	5.1	Q1

### 3.5 Co-cited references

Co-citation frequency reflects the academic influence of publications. Table 6 listed the top ten co-cited references. The most cited paper was “*Reactive microglia are positive for HLA-DR in the substantia nigra of Parkinson’s and Alzheimer’s disease brains*” (566 citations), published in *Neurology*, followed by “*Neuroinflammation in Parkinson’s disease: a target for neuroprotection” (471 citations) and “Parkinson’s disease: Mechanisms and models*” (283 citations). The co-citation reference clustering showed a temporal evolution in research themes, transitioning from light green (older) to dark red (recent) (Figure 4C). Earlier research emphasized “air pollution,” “6-hydroxydopamine,” and “minocycline,” while recent studies have shifted toward “alpha-synuclein,” “gut microbiota,” “nlrp3,” and “microglial autophagy.”

**Table 6:** Top 10 co-cited references.

Rank	Title	Type	Citation times	Year	Journal	IF	JCR
1	Reactive microglia are positive for HLA-DR in the substantia nigra of Parkinson’s and Alzheimer’s disease brains. Neurology. 1988 Aug;38(8):1285–91. doi: 10.1212/wnl.38.8.1285 (McGeer et al., 1988).	Article	566	1988	Neurology	7.7	Q1
2	Neuroinflammation in Parkinson’s disease: a target for neuroprotection? Lancet Neurol. 2009 Apr;8(4):382–97. doi: 10.1016/S1474-4422(09)70062-6 (Hirsch and Hunot, 2009)	Review	471	2009	Lancet Neurology	46.5	Q1
3	Parkinson’s disease: mechanisms and models. Neuron. 2003 Sep 11;39(6):889–909. doi: 10.1016/s0896-6273(03)00568-3 (Dauer and Przedborski, 2003)	Review	283	2003	Neuron	14.7	Q1
4	Staging of brain pathology related to sporadic Parkinson’s disease. Neurobiol. Aging. 2003 Mar-Apr;24(2):197–211. doi: 10.1016/s0197-4580(02)00065-9 (Braak et al., 2003a)	Review	282	2003	Neurobiology Of Aging	3.7	Q2
5	Infiltration of CD4 + lymphocytes into the brain contributes to neurodegeneration in a mouse model of Parkinson disease. J. Clin. Invest. 2009 Jan;119(1):182–92. doi: 10.1172/JCI36470 (Brochard et al., 2009)	Article	265	2009	Journal Of Clinical Investigation	13.3	Q1
6	*In vivo* imaging of microglial activation with [11C](R)-PK11195 PET in idiopathic Parkinson’s disease. Neurobiol. Dis. 2006 Feb;21(2):404–12. doi: 10.1016/j.nbd.2005.08.002 (Gerhard et al., 2006)	Article	260	2006	Neurobiology Of Disease	5.1	Q1
7	Microglia-mediated neurotoxicity: uncovering the molecular mechanisms. Nat. Rev. Neurosci. 2007 Jan;8(1):57–69. doi: 10.1038/nrn2038 (Block et al., 2007)	Review	255	2007	Nature Reviews Neuroscience	28.7	Q1
8	Aggregated alpha-synuclein activates microglia: a process leading to disease progression in Parkinson’s disease. FASEB J. 2005 Apr;19(6):533–42. doi: 10.1096/fj.04-2751com (Zhang et al., 2005)	Article	249	2005	Faseb Journal	4.4	Q2
9	Mechanisms underlying inflammation in neurodegeneration. Cell. 2010 Mar 19;140(6):918–34. doi: 10.1016/j.cell.2010.02.016 (Glass et al., 2010)	Review	245	2010	Cell	45.5	Q1
10	Parkinson disease. Nat Rev. Dis Primers. 2017 Mar 23;3:17013. doi: 10.1038/nrdp.2017.13 (Poewe et al., 2017)	Article	238	2017	Nature Reviews Disease Primers	76.9	Q1

### 3.6 Authors and co-cited authors

**Figure 5:**
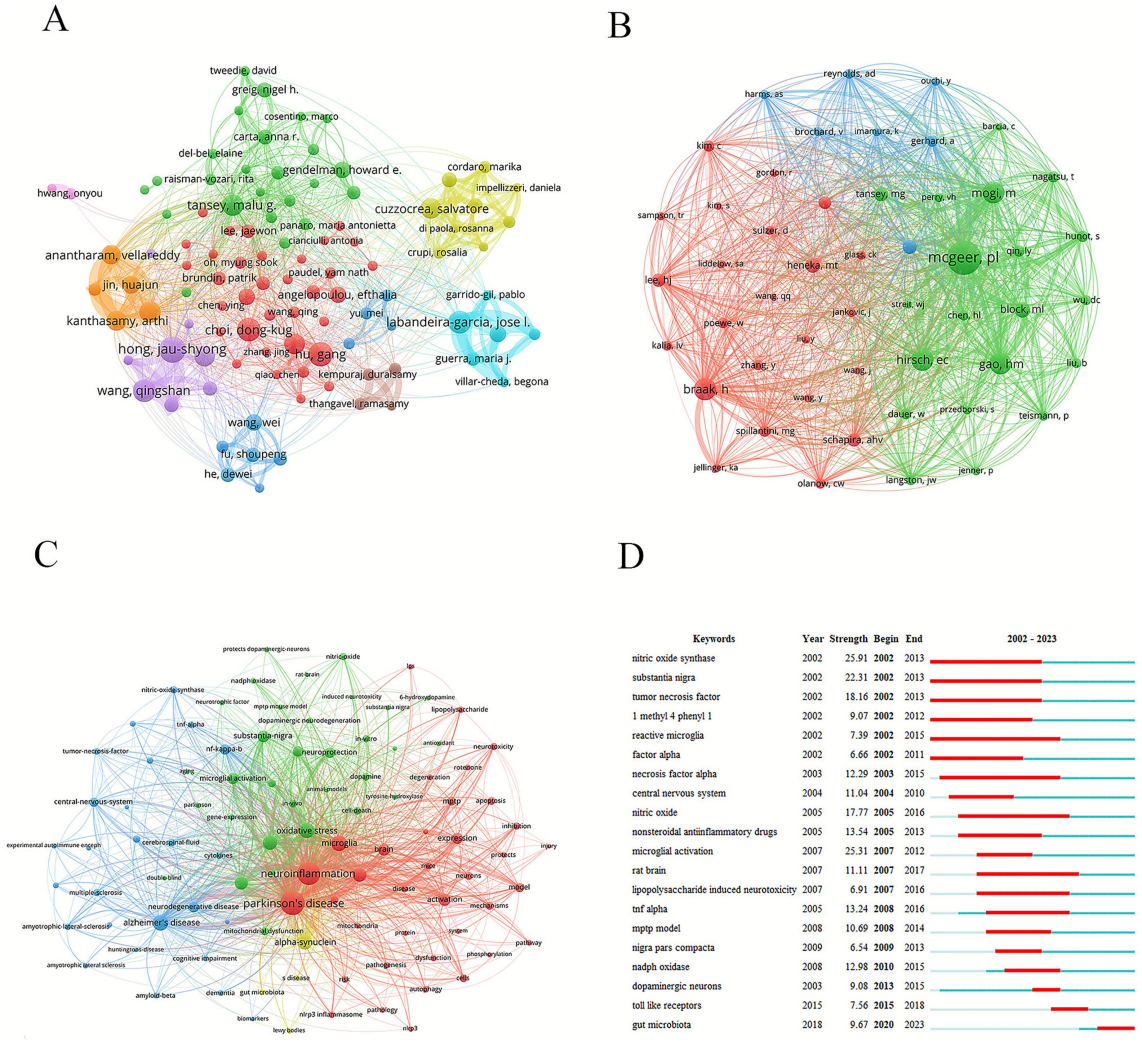
Analysis of authors and keywords. **(A)** Graph of network collaboration among authors. **(B)** Graph of network collaboration among co-cited authors. **(C)** Keyword network diagram. **(D)** Keywords with the strongest citation bursts.

The most prolific author was Hong, Jau-Shyong (27 papers), followed by Hu Gang (25 papers) and Choi, Dong-Kug (24 papers). The nodes with different colors represented different research teams (Figure 5A). Thicker lines indicated stronger collaborations. The red node team mainly comprised Hu, Gang, Choi, Dong-Kug, and Lu Ming. The orange node team mainly comprised Kanthasamy, Anumantha G, Kanthasamy, Arthi, and Jin, Huajun. The purple node team mainly comprised Hong, Jau-Shyong, Wang, Qingshan, and Zhang, Feng. In terms of the influence of the papers, Block, Michelle L. ranked first (average number of citations: 798.75), followed by Winner, Beate (average number of citations: 496.83), and Shannon, Kathleen M. (average number of citations: 496.8) (Table 7).

**Table 7:** Top 10 authors in terms of the total number of papers and the average citations.

Rank	Total number of papers	Average citations
1	Hong, Jau-Shyong (27)	Block, Michelle L. (798.75)
2	Hu, Gang (25)	Winner, Beate (496.84)
3	Choi, Dong-Kug (24)	Shannon, Kathleen M. (496.8)
4	José Luis Labandeira García (23)	Wu, Xuefei. (440.5)
5	Wang, Qingshan (23)	Hirsch, Etienne C. (371.8)
6	Tansey, Malu Gamez (22)	Tang, Yu (362)
7	Cuzzocrea, Salvatore (21)	Calderon-Garciduenas, Lilian (352.67)
8	Lu, Ming (21)	Mouradian, M. Maral (340)
9	Zhang, Feng (21)	Hunot, Stephane (328.6)
10	Kanthasamy, Arthi (20)	Haney, Matthew J. (320.6)

Among the top ten co-cited authors, five were cited more than 500 times. Dr. Patrick L. McGeer was the most cited (1,726 citations), followed by Dr. Heiko Braak (764 citations) and Dr. Etienne C. Hirsch (751 citations) (Table 8; Figure 5B). The work of these highly cited authors has substantially influenced the field and supported its development.

**Table 8:** Top 10 co-cited authors.

Rank	Author	Citations	Country	Institution
1	Mcgeer, Patrick L.	1,276	Canada	University of British Columbia
2	Braak, Heiko	764	Germany	Ulm University
3	Hirsch, Etienne C.	751	France	Sorbonne University
4	Mogi, M	740	Japan	Matsumoto Dental University
5	Gao, Hongming	736	China	Harbin Institute of Technology
6	Block, Michelle L.	496	USA	Indiana University
7	Heneka, Michael T.	452	Germany	University of Bonn
8	Tansey, Malu Gamez	445	USA	University of Florida
9	Schapira, Anthony H. V.	399	UK	Institute of Neurology, University College London
10	Lee, Hyuk-Joon	394	South Korea	Seoul National University

### 3.7 Keywords

Keyword analysis helps researchers to quickly understand the research topics and focus on a certain field. Keyword nodes with different colors represented different clustering directions. Green nodes focused on microglia and oxidative stress involved in the neuroinflammatory response. The blue nodes were related to neurodegenerative diseases, such as Alzheimer’s and Huntington’s disease. The red nodes were mainly concentrated on neurotoxicity and pathological processes (Figure 5C). In this study, in addition to PD (2,332 times) and neuroinflammation (2,059 times), high-frequency keywords included microglia (842 times), oxidative stress (798 times), alpha-synuclein (741 times), neurodegeneration (707 times), inflammation (629 times), and mouse model (554 times). Analyzing the outbreak of keywords can reveal the current research hotspots and future development trends. “Reactive microglia,” “nitric oxide synthase,” “substantia nigra,” and “necrosis factor alpha” were the longer-lasting burst words of the past two decades. “Gut microbiota” was the most impactful keyword from 2020 to 2023 (Figure 5D).

## 4 Discussion

### 4.1 General information

From 2002 to 2023, the number of related publications showed an upward trend. This phenomenon may be attributed to the increasing severity of population aging, drawing greater research attention to the mechanisms of PD. China had the most publications, while Norway exhibited the greatest influence. The Chinese government has made continuous and substantial financial investments in the fields of brain science and neurodegenerative diseases. Moreover, China has a large number of researchers and PD patients. With its scale and systematic investment, it has dominated the quantity of scientific research outputs. Norway leads in terms of the average number of citations per paper. It relies on a few but extremely outstanding research centers, led by thought leaders in the field, through in-depth interdisciplinary cooperation, focusing on solving fundamental and pioneering scientific problems, thereby generating highly influential results. Shanghai Jiao Tong University was the most prolific institution, whereas Arizona State University demonstrated the strongest influence. The International *Journal of Molecular Sciences* was the most prolific journal in this field, and *Cell* had the strongest impact. The most productive author was Hong, Jau-Shyong, and the most influential author was Block, Michelle L.

### 4.2 Hotspots and frontiers

Keywords represent the core content of publications and can reveal research hotspots and trends. In addition to “PD” and “neuroinflammation,” high-frequency keywords included microglia, oxidative stress, alpha-synuclein, neurodegeneration, inflammation, and mouse models. Microglia, as immune cells within the CNS, exhibit both pro-inflammatory and anti-inflammatory functions and play a crucial role in maintaining tissue homeostasis (Kwon and Koh, 2020). Overactivation of microglia can damage surrounding healthy neural tissues, leading to progressive neuronal loss (Subhramanyam et al., 2019). In PD, reactive microglia can exhibit a signature associated with the production of pro-inflammatory cytokines such as IL-1β and TNF-*α*, which may exacerbate neuronal damage. Conversely, in certain contexts, microglia may adopt a state that supports tissue repair and the clearance of cellular debris (Paolicelli et al., 2022). Oxidative stress arises from an imbalance between the production of oxidizing substances, such as reactive oxygen species (ROS) and reactive nitrogen species (RNS), and the body’s antioxidant defense mechanisms. In dopaminergic neurons, oxidative stress originates from multiple sources, including dopamine metabolism (Segura-Aguilar et al., 2014), mitochondrial dysfunction (Schapira, 2008), and neuroinflammation (Block et al., 2007), resulting in substantial ROS accumulation. A tight interplay exists between oxidative stress and neuroinflammation in PD (Taylor et al., 2013), potentially forming a vicious cycle: oxidative stress can trigger neuroinflammation, while neuroinflammation may further exacerbate oxidative stress, thereby accelerating neuronal damage and death (Niranjan, 2014). A hallmark pathological feature of PD is the accumulation of alpha-synuclein, which both aggravates and potentially initiates neuroinflammation (Johnson et al., 2019). Alpha-synuclein participates in oxidative stress responses by impairing mitochondrial complex I activity, leading to excessive ROS production (Devi et al., 2008). Moreover, it acts on astrocytes, microglia, and lymphocytes, eliciting neuroinflammatory responses (Zella et al., 2019). Commonly used mouse models of PD involve neuroinflammation induction via 1-methyl-4-phenyl-1,2,3,6-tetrahydropyridine (MPTP), 6-hydroxydopamine (6-OHDA), paraquat, or lipopolysaccharide (LPS). MPTP is converted to MPP^+^ in the brain, where it inhibits mitochondrial complex I function and causes dopaminergic neuronal death (Przedborski and Jackson-Lewis, 1998). 6-OHDA generates abundant ROS and exerts neurotoxicity upon uptake into dopaminergic neurons (Cohen and Heikkila, 1974). Paraquat is a potent inducer of oxidative stress and neuroinflammatory responses, leading to dopaminergic neurodegeneration (Berry et al., 2010). LPS triggers microglial activation, promoting the release of pro-inflammatory mediators that drive neuroinflammation (Elin and Wolff, 1976). These keywords reflect the specific mechanisms of neuroinflammation in PD, and the animal models used to induce neuroinflammation have garnered significant research interest.

Gut microbiota has emerged as a frequently identified burst keyword in recent years, representing a key frontier in PD research. This trend directly corresponds to the core “gut-brain axis” hypothesis, which offers novel perspectives on PD pathogenesis, preclinical diagnosis, and therapeutic intervention. The Braak hypothesis posits that PD pathology may originate in the enteric nervous system and subsequently propagate retrogradely to the brainstem and substantia nigra via the vagus nerve (Braak et al., 2003b). Specific gut microorganisms and their metabolites can induce the misfolding and aggregation of *α*-synuclein within intestinal epithelial and enteric neurons. This pathological α-synuclein can subsequently behave in a “prion-like” manner, spreading trans-synaptically through the vagus nerve to the brain (Holmqvist et al., 2014). A landmark study demonstrated that transferring gut microbiota from PD patients to model mice significantly exacerbated motor deficits and α-synuclein pathology, whereas germ-free mice exhibited markedly reduced pathology (Sampson et al., 2016), indicating that the gut microbiota is necessary for pathogenesis. Dysbiosis of the gut microbiota can increase intestinal barrier permeability, permitting bacterial endotoxins (e.g., LPS) to enter the systemic circulation. LPS can activate microglia in both the gut and the brain, triggering a robust release of pro-inflammatory cytokines that exacerbates neuroinflammation and promotes dopaminergic neuronal death (Qin et al., 2007). Furthermore, microbiota-derived short-chain fatty acids (SCFAs), such as butyrate, possess anti-inflammatory properties (Silva et al., 2020), and a reduction in their levels can contribute to a pro-inflammatory state. A healthy gut microbiota produces greater quantities of SCFAs, which can enter the bloodstream, reach the brain, and modulate microglial and astrocytic functions, thereby exerting anti-inflammatory and neuroprotective effects that help preserve dopaminergic neurons (Sun et al., 2018). The gut microbiota and the brain engage in bidirectional communication via neural, immune, and endocrine pathways. SCFAs not only regulate immune responses but also influence vagal nerve activity, gut hormone secretion, and the tryptophan metabolic pathway. Alterations in these pathways collectively impact neuronal survival, synaptic plasticity, and neurotransmitter balance (Fülling et al., 2019). Clinically, gastrointestinal symptoms such as constipation frequently manifest several years or even decades before motor symptom onset in PD patients (Savica et al., 2009). Analysis of the fecal microbiome can identify PD-specific microbial signatures (Hopfner et al., 2017), which hold promise as non-invasive diagnostic or early-warning biomarkers, particularly in the prodromal stage. Consequently, correcting gut microbiota dysbiosis represents a promising therapeutic strategy for PD. Current interventions include: (i) Probiotics/prebiotics: Aimed at restoring a healthy microbial balance, improving intestinal barrier integrity, and alleviating constipation (Barichella et al., 2016). (ii) Fecal microbiota transplantation (FMT): This procedure involves transferring microbiota from healthy donors to PD patients to fundamentally reshape their gut microecology. Small-scale clinical studies are currently evaluating its safety and efficacy (Kuai et al., 2021). (iii) Dietary interventions: High-fiber diets can stimulate the growth of beneficial SCFA-producing bacteria and represent a feasible adjunctive therapy (Kwon et al., 2024).

### 4.3 Clinical study

Clinical studies showed that neuroinflammation plays a key role in the occurrence and development of PD. Bartel’s team used the first-generation TSPO tracer [^11^C]PK11195 to study microglial activation in PD patients and attempted to evaluate the anti-inflammatory effect of the COX-2 inhibitor celecoxib (Bartels et al., 2010). The study found that although the binding potential in certain brain regions of PD patients showed an increasing trend, the difference was not significant; moreover, the binding value increased after celecoxib treatment. The authors concluded that [^11^C]PK11195 had limitations in quantifying neuroinflammation and that more superior tracers needed to be developed. Jucaite’s team used the second-generation TSPO tracer [^11^C]PBR28 to assess the effect of the MPO inhibitor AZD3241 on neuroinflammation in PD patients (Jucaite et al., 2015). This randomized placebo-controlled study demonstrated that after 8 weeks of AZD3241 treatment, TSPO binding in multiple brain regions decreased significantly, indicating its potential to inhibit microglial activation along with a favorable safety profile. Terada’s team conducted a longitudinal study using another second-generation TSPO tracer, [^11^C]DPA713, to observe the effect of adding zonisamide to dopamine replacement therapy on neuroinflammation in early-stage PD patients (Terada et al., 2024). The study found that although neuroinflammation spread throughout the brain with disease progression, the zonisamide group exhibited lower levels of neuroinflammation and better attention scores, suggesting anti-inflammatory and neuroprotective effects. Collectively, these three studies indicate that neuroinflammation is an important factor in PD progression and can be monitored via TSPO PET imaging. From first- to second-generation TSPO tracers (such as PBR28 and DPA713), the sensitivity and reliability of imaging have improved. Different anti-inflammatory strategies (COX-2 inhibition, MPO inhibition, and multimodal drugs such as zonisamide) have shown varying degrees of anti-inflammatory effects, among which AZD3241 and zonisamide demonstrated promising potential in clinical trials, supporting the further development of disease-modifying treatment strategies targeting neuroinflammation in PD.

Therapies targeting neuroinflammation are still in the clinical trial stage, and no anti-neuroinflammatory drugs have been officially approved for PD treatment. As an adjunct treatment, ultramicronized palmitoylethanolamide significantly improves both motor and non-motor symptoms in PD patients and reduces MDS-UPDRS scores (Brotini et al., 2017). Its mechanism may involve inhibition of microglia- and mast cell-mediated neuroinflammation, and it has a good safety profile. Sargramostim has been studied in PD patients for its long-term safety and immunomodulatory effects. A reduced dose of 3 μg/kg/day decreased adverse reactions while increasing the number and function of regulatory T cells (Tregs), thereby improving immune balance (Olson et al., 2021). Although it did not significantly improve motor scores, it provides biomarker support for immunotherapy. As an adjunct therapy, celecoxib significantly reduces inflammatory markers (TNF-*α*, TLR-4, α-synuclein), enhances neurotrophic factor BDNF and antioxidant factor Nrf2, and improves UPDRS scores, supporting its therapeutic effects via inhibition of COX-2-mediated neuroinflammation (Khrieba et al., 2024). These drugs highlight the central role of immune regulation in PD treatment and provide experimental and clinical evidence for the development of novel adjuvant therapies.

### 4.4 Challenges and future directions

At present, several practical challenges in neuroinflammation and PD research hinder its clinical translation. One major issue is the “double-edged sword” nature of neuroinflammation: while the initial inflammatory response has beneficial effects by eliminating threats and promoting repair, its chronic phase often exacerbates neurodegeneration due to destructive effects (Tansey et al., 2022). This implies that completely suppressing neuroinflammation may not be an optimal strategy. Instead, designing intervention strategies that precisely modulate its beneficial and detrimental aspects to steer it toward a protective direction remains highly complex. Preclinical studies largely rely on animal or cellular models (Dovonou et al., 2023); however, it remains uncertain whether these models fully recapitulate the complex neuroinflammatory processes of human PD. Furthermore, many potential anti-inflammatory compounds have difficulty crossing the BBB, failing to reach therapeutically effective concentrations in the CNS. PD also exhibits considerable heterogeneity—clinical manifestations, disease progression, and treatment responses vary greatly among patients (Espay et al., 2017). Such variability may stem from differences in the type and extent of neuroinflammation. Consequently, a universal anti-inflammatory treatment strategy may not be effective for all patients. There is a growing urgency to develop individualized treatment strategies, although this endeavor also poses significant challenges.

Despite these challenges, emerging technologies and novel concepts are providing unprecedented opportunities in the field. Future treatment strategies will likely focus on modulating specific activation states of microglia and astrocytes rather than employing broad suppression or activation, thereby preserving their physiological functions while reducing neurotoxicity. Another promising approach involves transplanting stem cell-derived dopaminergic neurons (Piao et al., 2021) or cells with anti-inflammatory/neuroprotective functions (Xiong et al., 2021) to replace lost neurons and modulate the local inflammatory microenvironment (Barker et al., 2015). Exosomes, as natural nanovesicles, can carry various therapeutic molecules across the BBB and target specific cell types (Alvarez-Erviti et al., 2011). They may not only serve as delivery vehicles but also convey bioactive substances with therapeutic potential to regulate neuroinflammation and promote neural repair. Identifying biomarkers that reflect neuroinflammatory activity is crucial for enabling early diagnosis, disease staging, and treatment evaluation—key steps toward personalized medicine (Tolosa et al., 2021). For example, detecting *α*-synuclein fibrils in cerebrospinal fluid may allow treatment interventions to begin at the preclinical stage. Single-cell transcriptomics and other high-resolution technologies can reveal gene expression changes in specific cell types within the brains of PD patients, helping to identify distinct cellular subsets and molecular pathways involved in neuroinflammation (Smajić et al., 2022). Through these strategies, modulating neuroinflammation may eventually slow the progression of PD.

### 4.5 Limitations

The data for this study were sourced from the WoS and PubMed databases. While these are widely recognized as authoritative biomedical literature databases with extensive coverage, they do not encompass all relevant databases, such as Scopus and Embase. Additionally, limiting the search language to English improved data manageability and consistency but systematically excluded significant non-English literature published in various journals. Another important consideration is the potential citation bias introduced by the inclusion of review articles, which accounted for 37.7% (1,273 out of 3,375) of the analyzed dataset. Reviews tend to cite and synthesize well-established concepts and theoretical frameworks that have gained consensus in the field and, due to their comprehensive and instructive nature, are often cited more frequently. This citation pattern may have influenced the observed research hotspots and knowledge structure. Specifically, prominent themes identified in our network, such as “neuroinflammation and its interaction with α-synuclein,” “microglial activation,” and “the gut–brain axis,” undoubtedly represent deeply rooted and critical directions in PD neuroinflammation research. However, their central position and close co-occurrence relationship may be magnified to a certain extent by the high proportion of citations in review articles. This is because one of the core tasks of review is to sort out and consolidate these widely accepted consensus themes. In contrast, emerging, highly specialized, or contested original research may appear less significant in co-citation and keyword clustering analyses due to their lower frequency of inclusion in reviews. Therefore, the resulting knowledge graph more accurately reflects the core conceptual framework that is systematically discussed and cited within the field. This interpretation does not undermine the value of the present study—which lies precisely in revealing this core framework—but serves to caution readers that identifying research frontiers should also involve examining recent original articles. Future studies could develop algorithmic tools capable of stratifying literature by type to better distinguish between well-established consensus and emerging evidence. Due to the data export format employed in this analysis, we were unable to utilize more advanced standardized impact metrics such as Field-Weighted Citation Impact (FWCI) and g-index to accurately compare the influence of the research outcomes. Future studies that can obtain more granular structured data should incorporate indicators such as FWCI for supplementary analysis. This approach would represent a meaningful direction for further investigation.

## 5 Conclusion

This study delineated the pattern of neuroinflammation in PD through quantitative analysis. This field is undergoing rapid development and has attracted growing academic attention. The distribution of research is uneven, and international collaboration serves as an important driving force for progress. Research institutions are relatively concentrated, yet their influence requires further enhancement. A diversity of journals is involved, and interdisciplinary characteristics are evident. Research focus has shifted from environmental factors to molecular mechanisms and the gut–brain axis. Future research should build upon existing collaborative networks, emphasize research quality, promote interdisciplinary integration, deepen mechanistic investigations, and optimize research models and translational applications, ultimately providing new insights for the prevention and treatment of PD.

## Data Availability

The raw data supporting the conclusions of this article will be made available by the authors, without undue reservation.
