# Supplementary material for: Research trends on neuroinflammation in Parkinson’s disease: an exploratory analysis
Source: Front Aging Neurosci. 2026 Feb 4;18:1699038. doi: 10.3389/fnagi.2026.1699038 (PMC12913463; doi:10.3389/fnagi.2026.1699038)
Supplement: Supplementary file 1 [file Data_Sheet_1.DOCX]

FN Clarivate Analytics Web of Science

VR 1.0

PT J

AU Amanollahi, M

Jameie, M

Heidari, A

Rezaei, N

AF Amanollahi, Mobina

Jameie, Melika

Heidari, Arash

Rezaei, Nima

TI The Dialogue Between Neuroinflammation and Adult Neurogenesis:

Mechanisms Involved and Alterations in Neurological Diseases

SO MOLECULAR NEUROBIOLOGY

LA English

DT Review

DE Neuroinflammatory diseases; Neuroinflammation; Neurogenesis;

Hippocampus; Lateral Ventricles; Neuroglia; Cytokines; Chemokines

ID NEURAL STEM-CELLS; TRAUMATIC BRAIN-INJURY; NECROSIS-FACTOR-ALPHA;

NF-KAPPA-B; SPONTANEOUS RECURRENT SEIZURES; INDUCED RAT MODEL; IN-VIVO

MODELS; HIPPOCAMPAL NEUROGENESIS; SUBVENTRICULAR ZONE;

alzheimer's disease

AB Adult neurogenesis occurs mainly in the subgranular zone of the hippocampal dentate gyrus and the subventricular zone of the lateral ventricles. Evidence supports the critical role of adult neurogenesis in various conditions, including cognitive dysfunction, Alzheimer's disease (AD), and Parkinson's disease (PD). Several factors can alter adult neurogenesis, including genetic, epigenetic, age, physical activity, diet, sleep status, sex hormones, and central nervous system (CNS) disorders, exerting either pro-neurogenic or anti-neurogenic effects. Compelling evidence suggests that any insult or injury to the CNS, such as traumatic brain injury (TBI), infectious diseases, or neurodegenerative disorders, can provoke an inflammatory response in the CNS. This inflammation could either promote or inhibit neurogenesis, depending on various factors, such as chronicity and severity of the inflammation and underlying neurological disorders. Notably, neuroinflammation, driven by different immune components such as activated glia, cytokines, chemokines, and reactive oxygen species, can regulate every step of adult neurogenesis, including cell proliferation, differentiation, migration, survival of newborn neurons, maturation, synaptogenesis, and neuritogenesis. Therefore, this review aims to present recent findings regarding the effects of various components of the immune system on adult neurogenesis and to provide a better understanding of the role of neuroinflammation and neurogenesis in the context of neurological disorders, including AD, PD, ischemic stroke (IS), seizure/epilepsy, TBI, sleep deprivation, cognitive impairment, and anxiety- and depressive-like behaviors. For each disorder, some of the most recent therapeutic candidates, such as curcumin, ginseng, astragaloside, boswellic acids, andrographolide, caffeine, royal jelly, estrogen, metformin, and minocycline, have been discussed based on the available preclinical and clinical evidence.

C1 [Amanollahi, Mobina; Heidari, Arash; Rezaei, Nima] Universal Sci Educ & Res Network USERN, Network Immun Infect Malignancy & Autoimmun NIIMA, Tehran, Iran.

[Amanollahi, Mobina] Univ Tehran Med Sci, Sch Med, Tehran, Iran.

[Jameie, Melika] Univ Tehran Med Sci, Iranian Ctr Neurol Res, Neurosci Inst, Tehran, Iran.

[Jameie, Melika] Iran Univ Med Sci, Neurosci Res Ctr, Tehran, Iran.

[Heidari, Arash; Rezaei, Nima] Univ Tehran Med Sci, Res Ctr Immunodeficiencies, Childrens Med Ctr, Tehran, Iran.

[Heidari, Arash] Univ Tehran Med Sci, Students Sci Res Ctr, Tehran, Iran.

[Rezaei, Nima] Univ Tehran Med Sci, Sch Med, Dept Immunol, Tehran, Iran.

C3 Universal Scientific Education & Research Network (USERN); Tehran

University of Medical Sciences; Tehran University of Medical Sciences;

Iran University of Medical Sciences; Tehran University of Medical

Sciences; Tehran University of Medical Sciences; Tehran University of

Medical Sciences

RP Rezaei, N (corresponding author), Universal Sci Educ & Res Network USERN, Network Immun Infect Malignancy & Autoimmun NIIMA, Tehran, Iran.; Rezaei, N (corresponding author), Univ Tehran Med Sci, Res Ctr Immunodeficiencies, Childrens Med Ctr, Tehran, Iran.; Rezaei, N (corresponding author), Univ Tehran Med Sci, Sch Med, Dept Immunol, Tehran, Iran.

EM cameliaa1999@gmail.com; ms-jameie@farabi.tums.ac.ir;

heidaryarash@yahoo.com; rezaei_nima@tums.ac.ir

RI Rezaei, Navid/AAF-9880-2019; Jameie, Melika/GRR-4350-2022; Rezaei,

Nima/B-4245-2008

OI Rezaei, Navid/0000-0002-8570-8301; Jameie, Melika/0000-0002-2028-9935;

Rezaei, Nima/0000-0002-3836-1827; Heidari, Arash/0000-0001-8290-2363

CR Acosta SA, 2013, PLOS ONE, V8, DOI 10.1371/journal.pone.0053376

Ahmed S, 2021, BRAIN BEHAV IMMUN, V91, P142, DOI 10.1016/j.bbi.2020.09.017

Ahmed T, 2010, NEUROSCIENCE, V169, P1296, DOI 10.1016/j.neuroscience.2010.05.078

Al-Onaizi M, 2020, INT J MOL SCI, V21, DOI 10.3390/ijms21186875

Ameen AM, 2017, CAN J PHYSIOL PHARM, V95, P819, DOI 10.1139/cjpp-2016-0158

Anusha C, 2017, CHEM-BIOL INTERACT, V269, P67, DOI 10.1016/j.cbi.2017.03.016

Araki T, 2021, EUR J NEUROSCI, V54, P5880, DOI 10.1111/ejn.14969

Arredondo SB, 2021, SCI REP-UK, V11, DOI 10.1038/s41598-021-01977-x

Arvidsson A, 2002, NAT MED, V8, P963, DOI 10.1038/nm747

Augusto-Oliveira M, 2019, CELLS-BASEL, V8, DOI 10.3390/cells8101293

Ayoub R, 2020, NAT MED, V26, P1285, DOI 10.1038/s41591-020-0985-2

Bachstetter AD, 2011, NEUROBIOL AGING, V32, P2030, DOI 10.1016/j.neurobiolaging.2009.11.022

Baglietto-Vargas D, 2017, SCI REP-UK, V7, DOI 10.1038/s41598-017-10353-7

Baker SA, 2004, EUR J NEUROSCI, V20, P575, DOI 10.1111/j.1460-9568.2004.03486.x

Barkho BZ, 2006, STEM CELLS DEV, V15, P407, DOI 10.1089/scd.2006.15.407

Bassani TB, 2017, BEHAV BRAIN RES, V335, P41, DOI 10.1016/j.bbr.2017.08.014

Bassani TB, 2018, MOL NEUROBIOL, V55, P4280, DOI 10.1007/s12035-017-0645-9

Bassett B, 2021, BRAIN BEHAV IMMUN, V91, P519, DOI 10.1016/j.bbi.2020.11.009

Belenguer G, 2021, CELL STEM CELL, V28, P285, DOI 10.1016/j.stem.2020.10.016

Belmadani A, 2006, J NEUROSCI, V26, P3182, DOI 10.1523/JNEUROSCI.0156-06.2006

Bernardino L, 2005, CNS NEUROL DISORD-DR, V4, P349, DOI 10.2174/1568007054546171

Bernardino L, 2008, STEM CELLS, V26, P2361, DOI 10.1634/stemcells.2007-0914

Bhattarai P, 2016, CELL REP, V17, P941, DOI 10.1016/j.celrep.2016.09.075

Boche D, 2006, NEUROBIOL DIS, V22, P638, DOI 10.1016/j.nbd.2006.01.004

Boldrini M, 2018, CELL STEM CELL, V22, P589, DOI 10.1016/j.stem.2018.03.015

Bongartz H, 2021, J NEUROCHEM, V157, P532, DOI 10.1111/jnc.15305

Borsini A, 2015, TRENDS NEUROSCI, V38, P145, DOI 10.1016/j.tins.2014.12.006

Bowen KK, 2011, NEUROREPORT, V22, P126, DOI 10.1097/WNR.0b013e3283430a44

Bramlett HM, 2015, J NEUROTRAUM, V32, P1834, DOI 10.1089/neu.2014.3352

Braun H, 2002, J NEUROTRAUM, V19, P975, DOI 10.1089/089771502320317122

Braun SMG, 2014, DEVELOPMENT, V141, P1983, DOI 10.1242/dev.104596

Brill MS, 2009, NAT NEUROSCI, V12, P1524, DOI 10.1038/nn.2416

Burmeister AR, 2018, FRONT CELL NEUROSCI, V12, DOI 10.3389/fncel.2018.00458

Burns JM, 2006, J EXP MED, V203, P2201, DOI 10.1084/jem.20052144

Bye N, 2011, J NEUROSCI RES, V89, P986, DOI 10.1002/jnr.22635

Cacci E, 2005, J NEUROSCI RES, V80, P789, DOI 10.1002/jnr.20531

Calabrese V, 2018, FREE RADICAL BIO MED, V115, P80, DOI 10.1016/j.freeradbiomed.2017.10.379

CAMPBELL IL, 1993, P NATL ACAD SCI USA, V90, P10061, DOI 10.1073/pnas.90.21.10061

Cao X, 2013, STEM CELLS, V31, P1633, DOI 10.1002/stem.1408

Cassé F, 2018, FRONT CELL NEUROSCI, V12, DOI 10.3389/fncel.2018.00432

Cha HJ, 2007, J BIOL CHEM, V282, P22984, DOI 10.1074/jbc.M700735200

CHAO CC, 1993, J IMMUNOL, V151, P1473

Chen GL, 2018, CELL MOL NEUROBIOL, V38, P1465, DOI 10.1007/s10571-018-0616-3

Chen JF, 2018, NEURAL REGEN RES, V13, P717, DOI 10.4103/1673-5374.230300

Chen Q, 2015, STEM CELLS, V33, P2574, DOI 10.1002/stem.2022

Chen ZG, 2013, BRAIN BEHAV IMMUN, V30, P45, DOI 10.1016/j.bbi.2013.01.083

Cheng X, 2017, FRONT NEUROSCI-SWITZ, V11, DOI 10.3389/fnins.2017.00590

Cherry JD, 2014, J NEUROINFLAMM, V11, DOI 10.1186/1742-2094-11-98

Chesnokova V, 2016, BRAIN BEHAV IMMUN, V58, P1, DOI 10.1016/j.bbi.2016.01.017

Chintamen S, 2021, FRONT CELL NEUROSCI, V14, DOI 10.3389/fncel.2020.571071

Chisholm SP, 2012, J NEUROSCI, V32, P1146, DOI 10.1523/JNEUROSCI.5343-11.2012

Cho KO, 2015, NAT COMMUN, V6, DOI 10.1038/ncomms7606

Choi P, 1998, CLIN EXP IMMUNOL, V113, P317

Choi SH, 2008, NEURON, V59, P568, DOI 10.1016/j.neuron.2008.07.033

Costa IM, 2019, CURR NEUROPHARMACOL, V17, P648, DOI 10.2174/1570159X16666180911123341

Covacu R, 2009, J IMMUNOL, V182, P6889, DOI 10.4049/jimmunol.0802907

Cuartero MI, 2021, FRONT CELL DEV BIOL, V9, DOI 10.3389/fcell.2021.657846

Cui LY, 2019, BRAIN RES, V1714, P81, DOI 10.1016/j.brainres.2019.01.024

Curtis E, 2018, CELL STEM CELL, V22, P941, DOI 10.1016/j.stem.2018.05.014

Das S, 2008, J NEUROSCI RES, V86, P1199, DOI 10.1002/jnr.21585

Dash PK, 2001, J NEUROSCI RES, V63, P313, DOI 10.1002/1097-4547(20010215)63:4<313::AID-JNR1025>3.3.CO;2-W

De Simoni MG, 2000, EUR J NEUROSCI, V12, P2623, DOI 10.1046/j.1460-9568.2000.00140.x

Desplats P, 2012, J BIOL CHEM, V287, P31691, DOI 10.1074/jbc.M112.354522

Dheen ST, 2007, CURR MED CHEM, V14, P1189, DOI 10.2174/092986707780597961

Dillen Y, 2020, TRANSL STROKE RES, V11, P60, DOI 10.1007/s12975-019-00717-8

Dinel AL, 2014, J NEUROINFLAMM, V11, DOI 10.1186/s12974-014-0155-x

Donovan MH, 2006, J COMP NEUROL, V495, P70, DOI 10.1002/cne.20840

Picanço LCD, 2018, CURR MED CHEM, V25, P3141, DOI 10.2174/0929867323666161213101126

Dziembowska M, 2005, GLIA, V50, P258, DOI 10.1002/glia.20170

Efferth T, 2022, SEMIN CANCER BIOL, V80, P39, DOI 10.1016/j.semcancer.2020.01.015

Ehninger D, 2008, CELL TISSUE RES, V331, P243, DOI 10.1007/s00441-007-0478-3

Ekdahl CT, 2009, NEUROSCIENCE, V158, P1021, DOI 10.1016/j.neuroscience.2008.06.052

Ekdahl CT, 2003, P NATL ACAD SCI USA, V100, P13632, DOI 10.1073/pnas.2234031100

Elyasi L, 2021, J BASIC CLIN PHYSIOL, V32, P11, DOI 10.1515/jbcpp-2019-0270

Endesfelder S, 2018, NEUROTOX RES, V34, P173, DOI 10.1007/s12640-018-9872-8

Endesfelder S, 2017, NEUROTOX RES, V32, P460, DOI 10.1007/s12640-017-9768-z

Erta M, 2012, INT J BIOL SCI, V8, P1254, DOI 10.7150/ijbs.4679

Eshraghi M, 2022, PHARMACOL THERAPEUT, V237, DOI 10.1016/j.pharmthera.2022.108171

Eshraghi M, 2021, INT J MOL SCI, V22, DOI 10.3390/ijms22073330

Eyo UB, 2014, J NEUROSCI, V34, P10528, DOI 10.1523/JNEUROSCI.0416-14.2014

Fabel K, 2003, EUR J NEUROSCI, V18, P2803, DOI 10.1111/j.1460-9568.2003.03041.x

Fan LW, 2017, NEURAL REGEN RES, V12, P366, DOI 10.4103/1673-5374.202926

Fawley LC, 2020, BRAIN BEHAV IMMUN, V83, P172, DOI 10.1016/j.bbi.2019.10.007

Flynn G, 2003, J NEUROIMMUNOL, V136, P84, DOI 10.1016/S0165-5728(03)00009-2

Fuster-Matanzo A, 2013, MEDIAT INFLAMM, V2013, DOI 10.1155/2013/260925

Gadani SP, 2012, J IMMUNOL, V189, P4213, DOI 10.4049/jimmunol.1202246

Gage FH, 2019, SCIENCE, V364, P827, DOI 10.1126/science.aav6885

Galgano M, 2017, CELL TRANSPLANT, V26, P1118, DOI 10.1177/0963689717714102

Gao X, 2013, EXP NEUROL, V239, P38, DOI 10.1016/j.expneurol.2012.09.012

Gao XA, 2011, J NEUROPATH EXP NEUR, V70, P183, DOI 10.1097/NEN.0b013e31820c6878

Ge SY, 2008, J PHYSIOL-LONDON, V586, P3759, DOI 10.1113/jphysiol.2008.155655

Gemma C, 2007, EUR J NEUROSCI, V26, P2795, DOI 10.1111/j.1460-9568.2007.05875.x

Gemma C, 2013, FRONT CELL NEUROSCI, V7, DOI 10.3389/fncel.2013.00229

Gerhard A, 2006, NEUROBIOL DIS, V21, P404, DOI 10.1016/j.nbd.2005.08.002

Ghaddar B, 2021, CELLS-BASEL, V10, DOI 10.3390/cells10020391

Ghavami S, 2014, PROG NEUROBIOL, V112, P24, DOI 10.1016/j.pneurobio.2013.10.004

Ghosal K, 2010, PLOS ONE, V5, DOI 10.1371/journal.pone.0011866

Gilhus NE, 2019, NAT REV NEUROL, V15, P429, DOI 10.1038/s41582-019-0227-8

Giunta B, 2012, J NEUROINFLAMM, V9, DOI 10.1186/1742-2094-9-185

Glick D, 2010, J PATHOL, V221, P3, DOI 10.1002/path.2697

Gong XM, 2006, CELL BIOL INT, V30, P466, DOI 10.1016/j.cellbi.2006.01.007

GOULD E, 1992, J NEUROSCI, V12, P3642

Griffiths BB, 2020, NEURAL REGEN RES, V15, P973, DOI 10.4103/1673-5374.270292

Guo YJ, 2013, NEUROCHEM INT, V63, P498, DOI 10.1016/j.neuint.2013.08.003

Gupta N, 2012, SCI TRANSL MED, V4, DOI 10.1126/scitranslmed.3004373

Guzman-Marin R, 2005, EUR J NEUROSCI, V22, P2111, DOI 10.1111/j.1460-9568.2005.04376.x

Gyengesi E, 2019, FRONT NEUROSCI-SWITZ, V13, DOI 10.3389/fnins.2019.00303

Habash T, 2015, EXP NEUROL, V273, P177, DOI 10.1016/j.expneurol.2015.08.016

Han JJ, 2016, NEURON, V91, P79, DOI 10.1016/j.neuron.2016.05.034

HANDA S S, 1992, Fitoterapia, V63, P3

Harrison JK, 1998, P NATL ACAD SCI USA, V95, P10896, DOI 10.1073/pnas.95.18.10896

Hasebe K, 2022, ACTA NEUROPSYCHIATR, V34, P220, DOI 10.1017/neu.2021.44

Hauck L, 2007, CIRC RES, V100, P50, DOI 10.1161/01.RES.0000254704.92532.b9

Haughey NJ, 2002, J NEUROCHEM, V83, P1509, DOI 10.1046/j.1471-4159.2002.01267.x

Hayden MS, 2014, SEMIN IMMUNOL, V26, P253, DOI 10.1016/j.smim.2014.05.004

He XJ, 2006, J NEUROPATH EXP NEUR, V65, P873, DOI 10.1097/01.jnen.0000235115.29440.ce

Heidari A, 2022, J NEUROINFLAMM, V19, DOI 10.1186/s12974-022-02496-w

Heidari A, 2021, ACTA NEUROBIOL EXP, V81, P249, DOI 10.21307/ane-2021-023

Heidari A, 2021, CURR PHARM DESIGN, V27, P4650, DOI 10.2174/1381612827666210816114003

Heneka MT, 2015, LANCET NEUROL, V14, P388, DOI 10.1016/S1474-4422(15)70016-5

Hensley K, 2010, J ALZHEIMERS DIS, V21, P1, DOI 10.3233/JAD-2010-1414

Hernandez GD, 2020, ALZH DEMENT-TRCI, V6, DOI 10.1002/trc2.12107

Hernandez-Ontiveros DG, 2013, FRONT NEUROL, V4, DOI 10.3389/fneur.2013.00030

HIRANO T, 1985, P NATL ACAD SCI USA, V82, P5490, DOI 10.1073/pnas.82.16.5490

Hirsch Etienne C, 2012, Parkinsonism Relat Disord, V18 Suppl 1, pS210, DOI 10.1016/S1353-8020(11)70065-7

Hiyama A, 2013, ARTHRITIS RES THER, V15, DOI 10.1186/ar4379

Höglinger GU, 2004, NAT NEUROSCI, V7, P726, DOI 10.1038/nn1265

Hollands C, 2017, MOL NEURODEGENER, V12, DOI 10.1186/s13024-017-0207-7

Huang F, 2018, MOLECULES, V23, DOI 10.3390/molecules23092178

Hueston CM, 2018, BRAIN BEHAV IMMUN, V74, P252, DOI 10.1016/j.bbi.2018.09.015

Hui L, 2007, BRAIN BEHAV IMMUN, V21, P308, DOI 10.1016/j.bbi.2006.09.005

Hurley MJ, 2013, BRAIN, V136, P2077, DOI 10.1093/brain/awt134

Ikonomidou C, 2010, EPILEPSY RES, V88, P11, DOI 10.1016/j.eplepsyres.2009.09.019

Imitola J, 2004, P NATL ACAD SCI USA, V101, P18117, DOI 10.1073/pnas.0408258102

Iosif RE, 2008, J CEREBR BLOOD F MET, V28, P1574, DOI 10.1038/jcbfm.2008.47

Iosif RE, 2006, J NEUROSCI, V26, P9703, DOI 10.1523/JNEUROSCI.2723-06.2006

Iqbal H, 2020, J GINSENG RES, V44, P593, DOI 10.1016/j.jgr.2019.05.007

Islam MM, 2015, BBA-GENE REGUL MECH, V1849, P210, DOI 10.1016/j.bbagrm.2014.06.001

Islam O, 2009, MOL BIOL CELL, V20, P188, DOI 10.1091/mbc.E08-05-0463

Jäkel S, 2017, FRONT CELL NEUROSCI, V11, DOI 10.3389/fncel.2017.00024

Jameie B., 2016, INT CLIN NEUROSCIENC, V3, P99, DOI DOI 10.22037/ICNJ.V3I2.13766

Jameie SB, 2021, BASIC CLIN NEUROSCI, V12, P383, DOI 10.32598/bcn.2021.2634.1

Jameie SB, 2019, J CHEM NEUROANAT, V100, DOI 10.1016/j.jchemneu.2019.101656

Jessberger S, 2007, J NEUROSCI, V27, P9400, DOI 10.1523/JNEUROSCI.2002-07.2007

Jessberger S, 2015, CSH PERSPECT BIOL, V7, DOI 10.1101/cshperspect.a020677

Jia CH, 2020, EXP NEUROL, V323, DOI 10.1016/j.expneurol.2019.113088

Jiang S, 2013, J INNATE IMMUN, V5, P471, DOI 10.1159/000346707

Jin K, 2003, MOL CELL NEUROSCI, V24, P171, DOI 10.1016/S1044-7431(03)00159-3

Jin KL, 2004, P NATL ACAD SCI USA, V101, P343, DOI 10.1073/pnas.2634794100

Jin KL, 2006, P NATL ACAD SCI USA, V103, P13198, DOI 10.1073/pnas.0603512103

Jin XM, 2012, PLOS ONE, V7, DOI [10.1371/journal.pone.0041892, 10.1371/journal.pone.0040637]

Merino JJ, 2015, J CELL PHYSIOL, V230, P27, DOI 10.1002/jcp.24695

Jung JS, 2010, J NEUROCHEM, V115, P1668, DOI 10.1111/j.1471-4159.2010.07075.x

Jung KH, 2004, EUR J NEUROSCI, V19, P3219, DOI 10.1111/j.0953-816X.2004.03412.x

Kalueff AV, 2004, NEUROSCI LETT, V365, P106, DOI 10.1016/j.neulet.2004.04.061

Kato H, 1996, BRAIN RES, V734, P203, DOI 10.1016/0006-8993(96)00636-1

Kempermann G, 2018, CELL STEM CELL, V23, P25, DOI 10.1016/j.stem.2018.04.004

Khodaverdiloo A, 2021, BRAIN BEHAV, V11, DOI 10.1002/brb3.2423

Kim JK, 2021, J GINSENG RES, V45, P717, DOI 10.1016/j.jgr.2021.03.009

Kim Miri, 2018, Exp Mol Med, V50, P1, DOI 10.1038/s12276-018-0033-1

Kim SJ, 2008, J BIOL CHEM, V283, P14497, DOI 10.1074/jbc.M708373200

Kirby ED, 2013, ELIFE, V2, DOI 10.7554/eLife.00362

Kiyota T, 2012, GENE THER, V19, P724, DOI 10.1038/gt.2011.126

Klein C, 2016, BEHAV BRAIN RES, V307, P227, DOI 10.1016/j.bbr.2016.02.040

Ko CH, 2018, IRAN J BASIC MED SCI, V21, P1174, DOI 10.22038/IJBMS.2018.30371.7322

Kodali M, 2021, AGING CELL, V20, DOI 10.1111/acel.13277

Koh SH, 2015, J CLIN NEUROL, V11, P297, DOI 10.3988/jcn.2015.11.4.297

Kolodziej A, 2008, J NEUROSCI, V28, P4488, DOI 10.1523/JNEUROSCI.4721-07.2008

Komleva YK, 2022, CELL MOL NEUROBIOL, V42, P1355, DOI 10.1007/s10571-020-01021-y

Kong XJ, 2019, BRAIN BEHAV IMMUN, V79, P159, DOI 10.1016/j.bbi.2019.01.027

Koo JW, 2008, P NATL ACAD SCI USA, V105, P751, DOI 10.1073/pnas.0708092105

Kozareva Danka A, 2019, Neuronal Signal, V3, pNS20180177, DOI 10.1042/NS20180177

Kozareva DA, 2019, J NEUROIMMUNOL, V331, P87, DOI 10.1016/j.jneuroim.2017.08.008

Krathwohl MD, 2004, STEM CELLS, V22, P109, DOI 10.1634/stemcells.22-1-109

Kumar A, 2015, PHARMACOL REP, V67, P195, DOI 10.1016/j.pharep.2014.09.004

Kurkowska-Jastrzebska I, 1999, EXP NEUROL, V156, P50, DOI 10.1006/exnr.1998.6993

LANGSTON JW, 1985, TRENDS NEUROSCI, V8, P79

Lazarov O, 2010, EXP NEUROL, V223, P267, DOI 10.1016/j.expneurol.2009.08.009

Lee H, 2013, HUM GENE THER, V24, P655, DOI 10.1089/hum.2013.001

Lee SW, 2013, BRAIN BEHAV IMMUN, V30, P33, DOI 10.1016/j.bbi.2012.09.010

Lehtimäki KA, 2003, MOL BRAIN RES, V110, P253, DOI 10.1016/S0169-328X(02)00654-X

Leng FD, 2021, NAT REV NEUROL, V17, P157, DOI 10.1038/s41582-020-00435-y

Leuner B, 2010, ANNU REV PSYCHOL, V61, P111, DOI 10.1146/annurev.psych.093008.100359

Li L, 2021, INT IMMUNOPHARMACOL, V92, DOI 10.1016/j.intimp.2020.107335

Li YJ, 2021, ANGEW CHEM INT EDIT, V60, P5083, DOI 10.1002/anie.202010391

Li ZC, 2013, BMC IMMUNOL, V14, DOI 10.1186/1471-2172-14-20

Liang HX, 2019, P NATL ACAD SCI USA, V116, P13621, DOI 10.1073/pnas.1811825116

Lim J, 2018, ARCH PHARM RES, V41, P943, DOI 10.1007/s12272-018-1063-x

Liu Q, 2014, SCI REP-UK, V4, DOI 10.1038/srep07554

Liu XS, 2007, J NEUROSCI RES, V85, P2120, DOI 10.1002/jnr.21359

Lledo PM, 2006, NAT REV NEUROSCI, V7, P179, DOI 10.1038/nrn1867

Lobo-Silva D, 2016, J NEUROINFLAMM, V13, DOI 10.1186/s12974-016-0763-8

López-Armas G, 2016, OXID MED CELL LONGEV, V2016, DOI 10.1155/2016/2136902

Lopez-Toledano MA, 2007, J ALZHEIMERS DIS, V12, P229

Lorenzen K, 2021, MOL BIOL REP, V48, P563, DOI 10.1007/s11033-020-06092-0

Lotocki G, 2009, J NEUROTRAUM, V26, P1123, DOI 10.1089/neu.2008.0802

Lu M, 2002, P NATL ACAD SCI USA, V99, P7090, DOI 10.1073/pnas.092013799

Lu ZJ, 2010, FASEB J, V24, P1925, DOI 10.1096/fj.09-150573

Luarte A, 2017, STEM CELLS INT, V2017, DOI 10.1155/2017/1719050

Lull ME, 2010, NEUROTHERAPEUTICS, V7, P354, DOI 10.1016/j.nurt.2010.05.014

Luo C., 2016, Neurogenesis, V3, pe1235525, DOI DOI 10.1080/23262133.2016.1235525

Luo C, 2016, GLIA, V64, P1508, DOI 10.1002/glia.23018

Ma L, 2012, BRAIN RES, V1469, P103, DOI 10.1016/j.brainres.2012.05.058

Ma LK, 2019, FRONT PHARMACOL, V10, DOI 10.3389/fphar.2019.00097

Macas J, 2006, J NEUROSCI, V26, P13114, DOI 10.1523/JNEUROSCI.4667-06.2006

MacEwan DJ, 2002, BRIT J PHARMACOL, V135, P855, DOI 10.1038/sj.bjp.0704549

Maiese K, 2020, EXPERT REV CLIN PHAR, V13, P23, DOI 10.1080/17512433.2020.1698288

Malhotra K, 2018, J NEUROL, V265, P1871, DOI 10.1007/s00415-018-8935-3

Manivannan S, 2021, NEUROPHARMACOLOGY, V183, DOI 10.1016/j.neuropharm.2020.108400

Mantovani A, 2013, J PATHOL, V229, P176, DOI 10.1002/path.4133

Mao ZF, 2020, FASEB J, V34, P10998, DOI 10.1096/fj.202000146RR

Vega-Rivera NM, 2020, NEUROSCIENCE, V440, P316, DOI 10.1016/j.neuroscience.2020.05.014

Martinelli S, 2021, NAT COMMUN, V12, DOI 10.1038/s41467-021-24810-5

Marxreiter F, 2013, CELL MOL LIFE SCI, V70, P459, DOI 10.1007/s00018-012-1062-x

Matsuda T, 2015, NAT COMMUN, V6, DOI 10.1038/ncomms7514

Matsui TK, 2018, BIOCHEM BIOPH RES CO, V503, P1880, DOI 10.1016/j.bbrc.2018.07.130

MCGEER PL, 1988, NEUROLOGY, V38, P1285, DOI 10.1212/WNL.38.8.1285

Michaluk P, 2007, CELL DEATH DIFFER, V14, P1255, DOI 10.1038/sj.cdd.4402141

Ming GL, 2011, NEURON, V70, P687, DOI 10.1016/j.neuron.2011.05.001

Minger SL, 2007, REGEN MED, V2, P69, DOI 10.2217/17460751.2.1.69

Mishra A, 2021, METAB BRAIN DIS, V36, P1591, DOI 10.1007/s11011-021-00806-4

Mishra SK, 2018, NEUROCHEM INT, V113, P56, DOI 10.1016/j.neuint.2017.11.012

Mizuno T, 2003, BRAIN RES, V979, P65, DOI 10.1016/S0006-8993(03)02867-1

Mo MS, 2019, J NEUROSCI, V39, P9453, DOI 10.1523/JNEUROSCI.0487-19.2019

Molyneaux KA, 2003, DEVELOPMENT, V130, P4279, DOI 10.1242/dev.00640

Mongiat LA, 2011, EUR J NEUROSCI, V33, P1055, DOI 10.1111/j.1460-9568.2011.07603.x

Monje ML, 2003, SCIENCE, V302, P1760, DOI 10.1126/science.1088417

Moreno-Jiménez EP, 2019, NAT MED, V25, P554, DOI 10.1038/s41591-019-0375-9

Morisse MC, 2018, EXPERT REV NEUROTHER, V18, P729, DOI 10.1080/14737175.2018.1510321

Morteau O., 2006, ENCY RESP MED, P356

Mosser DM, 2008, NAT REV IMMUNOL, V8, P958, DOI 10.1038/nri2448

Mosser DM, 2003, J LEUKOCYTE BIOL, V73, P209, DOI 10.1189/jlb.0602325

Mu YL, 2011, MOL NEURODEGENER, V6, DOI 10.1186/1750-1326-6-85

Mueller AD, 2014, BRAIN BEHAV IMMUN, V35, P182, DOI 10.1016/j.bbi.2013.10.001

Naseri A., 2014, RAZI J MED SCI, V21, P95

National Center for Biotechnology Information, 2022, ANDR

Navarro-Sanchis C, 2017, FRONT NEURAL CIRCUIT, V11, DOI 10.3389/fncir.2017.00074

Navidhamidi M, 2017, REV NEUROSCIENCE, V28, P307, DOI 10.1515/revneuro-2016-0059

Neo WH, 2014, J BIOL CHEM, V289, P20788, DOI 10.1074/jbc.M113.525493

Ng SY, 2019, FRONT CELL NEUROSCI, V13, DOI 10.3389/fncel.2019.00528

Nieto-Quero A, 2021, NEUROBIOL STRESS, V15, DOI 10.1016/j.ynstr.2021.100356

Nunan R, 2014, GLIA, V62, P1313, DOI 10.1002/glia.22682

Odaka H, 2017, NEURAL REGEN RES, V12, P1028, DOI 10.4103/1673-5374.211174

Oh J, 2010, J NEUROSCI RES, V88, P2798, DOI 10.1002/jnr.22447

Olesen LO, 2017, NEUROBIOL DIS, V104, P50, DOI 10.1016/j.nbd.2017.04.021

Orihuela R, 2016, BRIT J PHARMACOL, V173, P649, DOI 10.1111/bph.13139

Ou ZR, 2018, BRAIN BEHAV IMMUN, V69, P351, DOI 10.1016/j.bbi.2017.12.009

Pan WH, 1997, NEUROSCI BIOBEHAV R, V21, P603, DOI 10.1016/S0149-7634(96)00047-4

Panizzutti B, 2018, ACTA NEUROPSYCHIATR, V30, P334, DOI 10.1017/neu.2018.13

Parent JM, 1997, J NEUROSCI, V17, P3727

Park JH, 2010, EXP NEUROL, V222, P267, DOI 10.1016/j.expneurol.2010.01.004

Park SY, 2018, MOL BRAIN, V11, DOI 10.1186/s13041-018-0383-6

Patterson ZR, 2012, FRONT CELL NEUROSCI, V6, DOI 10.3389/fncel.2012.00058

Peng H, 2017, ALCOHOL, V62, P29, DOI 10.1016/j.alcohol.2017.02.175

Pereira L, 2015, FRONT CELL NEUROSCI, V9, DOI 10.3389/fncel.2015.00057

Perez-Asensio FJ, 2013, J CELL SCI, V126, P4208, DOI 10.1242/jcs.127803

Persidsky Y, 2006, J NEUROIMMUNE PHARM, V1, P223, DOI 10.1007/s11481-006-9025-3

Poewe W, 2017, NAT REV DIS PRIMERS, V3, DOI 10.1038/nrdp.2017.13

Ponomarev ED, 2007, J NEUROSCI, V27, P10714, DOI 10.1523/JNEUROSCI.1922-07.2007

Ponpuak M, 2015, CURR OPIN CELL BIOL, V35, P106, DOI 10.1016/j.ceb.2015.04.016

Pradillo JM, 2017, BRAIN BEHAV IMMUN, V61, P117, DOI 10.1016/j.bbi.2016.11.013

Pun RYK, 2012, NEURON, V75, P1022, DOI 10.1016/j.neuron.2012.08.002

Raballo R, 2000, J NEUROSCI, V20, P5012

Ramaswamy S, 2005, BRAIN RES, V1053, P38, DOI 10.1016/j.brainres.2005.06.042

Ransohoff RM, 2016, NAT NEUROSCI, V19, P987, DOI 10.1038/nn.4338

Rathbone CR, 2008, MUSCLE NERVE, V37, P84, DOI 10.1002/mus.20897

Ribeiro FF, 2021, ADV EXP MED BIOL, V1331, P77, DOI 10.1007/978-3-030-74046-7_7

Rodríguez JJ, 2009, NEUROREPORT, V20, P907, DOI 10.1097/WNR.0b013e32832be77d

Rodríguez-Iglesias N, 2019, FRONT CELL DEV BIOL, V7, DOI 10.3389/fcell.2019.00024

Rogers JT, 2011, J NEUROSCI, V31, P16241, DOI 10.1523/JNEUROSCI.3667-11.2011

Rolls A, 2007, NAT CELL BIOL, V9, P1081, DOI 10.1038/ncb1629

Rossi C, 2006, EUR J NEUROSCI, V24, P1850, DOI 10.1111/j.1460-9568.2006.05059.x

Rotelli AE, 2003, PHARMACOL RES, V48, P601, DOI 10.1016/S1043-6618(03)00225-1

Ryan SM, 2013, BRAIN BEHAV IMMUN, V33, P7, DOI 10.1016/j.bbi.2013.03.005

Sahab-Negah S, 2020, CELL MOL NEUROBIOL, V40, P283, DOI 10.1007/s10571-019-00733-0

Sahu S, 2013, EXP NEUROL, V248, P470, DOI 10.1016/j.expneurol.2013.07.021

Santos MCT, 2016, STEM CELLS, V34, P220, DOI 10.1002/stem.2204

Sato K, 2015, GLIA, V63, P1394, DOI 10.1002/glia.22858

Sawada M, 2006, J NEURAL TRANSM-SUPP, P373

Sayed AS, 2018, MOL NEUROBIOL, V55, P5798, DOI 10.1007/s12035-017-0801-2

Sayed AS, 2016, J MOL NEUROSCI, V59, P58, DOI 10.1007/s12031-016-0734-7

Schimmel Samantha J, 2017, Brain Circ, V3, P135, DOI 10.4103/bc.bc_18_17

Schmidt M, 2002, MOL CELL BIOL, V22, P7842, DOI 10.1128/MCB.22.22.7842-7852.2002

Schmidt SI, 2020, STEM CELL REP, V16, P281, DOI 10.1016/j.stemcr.2020.12.011

Scopa C, 2020, CELL DEATH DIFFER, V27, P934, DOI 10.1038/s41418-019-0409-3

Sellner S, 2016, ACTA NEUROPATHOL COM, V4, DOI 10.1186/s40478-016-0374-8

Shabab T, 2017, INT J NEUROSCI, V127, P624, DOI 10.1080/00207454.2016.1212854

Shapiro LA, 2005, NEUROSCIENCE, V136, P823, DOI 10.1016/j.neuroscience.2005.03.059

Shi RY, 2014, CNS NEUROSCI THER, V20, P1045, DOI 10.1111/cns.12325

Shi YH, 2020, FRONT PHARMACOL, V11, DOI 10.3389/fphar.2020.00285

Simon DW, 2017, NAT REV NEUROL, V13, P171, DOI 10.1038/nrneurol.2017.13

SINGH S, 1995, J BIOL CHEM, V270, P24995, DOI 10.1074/jbc.270.42.24995

Singh S, 2019, BEHAV PHARMACOL, V30, P688, DOI 10.1097/FBP.0000000000000516

Singh S, 2018, FREE RADICAL BIO MED, V129, P73, DOI 10.1016/j.freeradbiomed.2018.08.033

Singh S, 2018, MOL NEUROBIOL, V55, P6500, DOI 10.1007/s12035-017-0860-4

Sofroniew MV, 2010, ACTA NEUROPATHOL, V119, P7, DOI 10.1007/s00401-009-0619-8

Song SH, 2002, NATURE, V417, P39, DOI 10.1038/417039a

Sorrells SF, 2018, NATURE, V555, P377, DOI 10.1038/nature25975

Spittau B, 2017, NEURAL REGEN RES, V12, P1433, DOI 10.4103/1673-5374.215250

Stafstrom CE, 2015, CSH PERSPECT MED, V5, DOI 10.1101/cshperspect.a022426

Suh HS, 2013, J NEUROINFLAMM, V10, DOI 10.1186/1742-2094-10-37

Sultan S, 2015, NEURON, V88, DOI 10.1016/j.neuron.2015.10.037

Sun CR, 2013, J NEUROSCI, V33, P17314, DOI 10.1523/JNEUROSCI.2129-13.2013

Sun GC, 2020, BRAIN RES BULL, V162, P84, DOI 10.1016/j.brainresbull.2020.05.009

Sun L, 2020, CELL DEATH DISCOV, V6, DOI 10.1038/s41420-020-00298-8

Sung PS, 2020, INT J MOL SCI, V21, DOI 10.3390/ijms21030701

Switzer JA, 2011, STROKE, V42, P2633, DOI 10.1161/STROKEAHA.111.618215

Tang HL, 2021, PEERJ, V9, DOI 10.7717/peerj.10921

Tang HD, 2009, NEUROBIOL AGING, V30, P299, DOI 10.1016/j.neurobiolaging.2007.06.004

Tani M, 2010, J NEUROCHEM, V115, P854, DOI 10.1111/j.1471-4159.2010.06727.x

Tansey MG, 2007, EXP NEUROL, V208, P1, DOI 10.1016/j.expneurol.2007.07.004

Tavernier SJ, 2017, NAT CELL BIOL, V19, P698, DOI 10.1038/ncb3518

Tfilin M, 2019, J MOL NEUROSCI, V69, P254, DOI 10.1007/s12031-019-01354-4

Tiwari SK, 2014, ACS NANO, V8, P76, DOI 10.1021/nn405077y

Tobin MK, 2014, J CEREBR BLOOD F MET, V34, P1573, DOI 10.1038/jcbfm.2014.130

Todorova Antoniya, 2014, Pract Neurol, V14, P310, DOI 10.1136/practneurol-2013-000741

Tran PB, 2005, NEUROTOX RES, V8, P149, DOI 10.1007/BF03033826

Trevejo JM, 2001, P NATL ACAD SCI USA, V98, P12162, DOI 10.1073/pnas.211423598

Tripanichkul W, 2013, EUR REV MED PHARMACO, V17, P1360

Turnbull AV, 1999, PHYSIOL REV, V79, P1, DOI 10.1152/physrev.1999.79.1.1

Tyagi E, 2010, EUR J PHARMACOL, V640, P206, DOI 10.1016/j.ejphar.2010.04.041

Ueki T, 2003, J NEUROSCI, V23, P11732

Urrea C, 2007, RESTOR NEUROL NEUROS, V25, P65

Vainchtein ID, 2020, TRENDS NEUROSCI, V43, P144, DOI 10.1016/j.tins.2020.01.003

Vallières L, 2002, J NEUROSCI, V22, P486, DOI 10.1523/JNEUROSCI.22-02-00486.2002

van den Berge SA, 2011, BRAIN, V134, P3249, DOI 10.1093/brain/awr256

van Vliet EA, 2020, NEUROBIOL DIS, V145, DOI 10.1016/j.nbd.2020.105080

Varela-Nallar L, 2015, NEURAL PLAST, V2015, DOI 10.1155/2015/935403

Vawter MP, 1996, EXP NEUROL, V142, P313, DOI 10.1006/exnr.1996.0200

Victor Tanya R, 2020, Neuroimmunol Neuroinflamm, V7, P234, DOI 10.20517/2347-8659.2020.02

Virani SS, 2021, CIRCULATION, V143, pe254, DOI 10.1161/CIR.0000000000000950

Wadhwa M, 2019, BRAIN BEHAV IMMUN, V82, P129, DOI 10.1016/j.bbi.2019.08.004

Wadhwa M, 2017, J NEUROINFLAMM, V14, DOI 10.1186/s12974-017-0998-z

Walton NM, 2006, GLIA, V54, P815, DOI 10.1002/glia.20419

Wang B, 2015, METAB BRAIN DIS, V30, P355, DOI 10.1007/s11011-014-9523-6

Wang FM, 2011, BIOCHEM J, V433, P245, DOI 10.1042/BJ20101293

Wang J, 2021, MOL NEUROBIOL, V58, P1052, DOI 10.1007/s12035-020-02171-2

Wang XF, 2007, MOL CELL NEUROSCI, V36, P343, DOI 10.1016/j.mcn.2007.07.005

Wang XT, 2016, J NEUROTRAUM, V33, P721, DOI 10.1089/neu.2015.4097

Wang XL, 2012, PLOS ONE, V7, DOI 10.1371/journal.pone.0039159

Wang Y, 2016, CURR MOL MED, V16, P738, DOI 10.2174/1566524016666160829153453

Wang YL, 2013, AM J CHINESE MED, V41, P59, DOI 10.1142/S0192415X13500055

Wen PH, 2004, EXP NEUROL, V188, P224, DOI 10.1016/j.expneurol.2004.04.002

Wen SJ, 2021, NEURAL REGEN RES, V16, P1677, DOI 10.4103/1673-5374.306093

Wentz CT, 2009, NEUROPHARMACOLOGY, V56, P994, DOI 10.1016/j.neuropharm.2009.02.002

Whitney NP, 2009, J NEUROCHEM, V108, P1343, DOI 10.1111/j.1471-4159.2009.05886.x

Widera D, 2004, EUR J CELL BIOL, V83, P381, DOI 10.1078/0171-9335-00403

Willis EF, 2020, CELL, V180, P833, DOI 10.1016/j.cell.2020.02.013

Winner B, 2004, J NEUROPATH EXP NEUR, V63, P1155, DOI 10.1093/jnen/63.11.1155

Winner B, 2015, CSH PERSPECT BIOL, V7, DOI 10.1101/cshperspect.a021287

Wofford KL, 2019, NEURAL REGEN RES, V14, P1481, DOI 10.4103/1673-5374.255958

Wong G, 2004, EXP NEUROL, V187, P171, DOI 10.1016/j.expneurol.2004.01.009

Woodbury ME, 2014, J NEUROIMMUNE PHARM, V9, P92, DOI 10.1007/s11481-013-9501-5

Worlitzer MMA, 2012, STEM CELL RES THER, V3, DOI 10.1186/scrt124

Wu JP, 2000, NEUROSCI LETT, V292, P203, DOI 10.1016/S0304-3940(00)01472-5

Wu MD, 2013, BRAIN BEHAV IMMUN, V32, P9, DOI 10.1016/j.bbi.2013.03.003

Wu MD, 2012, BRAIN BEHAV IMMUN, V26, P292, DOI 10.1016/j.bbi.2011.09.012

Wu Q, 2018, INT J BIOCHEM CELL B, V94, P44, DOI 10.1016/j.biocel.2017.11.007

Wu YM, 2009, J NEUROCHEM, V109, P1157, DOI 10.1111/j.1471-4159.2009.06043.x

Xiong Y, 2018, CHIN J TRAUMATOL, V21, P137, DOI 10.1016/j.cjtee.2018.02.003

Yan YP, 2007, J CEREBR BLOOD F MET, V27, P1213, DOI 10.1038/sj.jcbfm.9600432

Yanagisawa M, 2001, NEUROSCI RES, V41, P391, DOI 10.1016/S0168-0102(01)00297-8

Yang F, 2010, J NEUROSCI RES, V88, P519, DOI 10.1002/jnr.22224

Yang SJ, 2018, FRONT IMMUNOL, V9, DOI 10.3389/fimmu.2018.00784

Yang XY, 2019, NEUROPHARMACOLOGY, V158, DOI 10.1016/j.neuropharm.2019.107748

Yao K, 2020, INFLAMMOPHARMACOLOGY, V28, P95, DOI 10.1007/s10787-019-00613-5

Yeganeh B., 2015, Autophagy: Cancer, Other Pathologies, Inflammation, Immunity, Infection, and Aging, P91, DOI DOI 10.1016/B978-0-12-801043-3.00005-4

Yli-Karjanmaa M, 2019, BRAIN BEHAV IMMUN, V82, P279, DOI 10.1016/j.bbi.2019.08.195

Yu SY, 2013, PROG NEURO-PSYCHOPH, V45, P47, DOI 10.1016/j.pnpbp.2013.05.001

Yuan JC, 2017, ONCOTARGET, V8, P19855, DOI 10.18632/oncotarget.15774

Zarkovic M, 2008, EUR J ENDOCRINOL, V159, P649, DOI 10.1530/EJE-08-0544

Zhang JQ, 2021, SCI ADV, V7, DOI 10.1126/sciadv.abb9888

Zhang JQ, 2017, BRAIN BEHAV IMMUN, V66, P111, DOI 10.1016/j.bbi.2017.07.012

Zhang L, 2021, NEUROPSYCH DIS TREAT, V17, P3819, DOI 10.2147/NDT.S334647

Zhang RL, 2006, J CEREBR BLOOD F MET, V26, P857, DOI 10.1038/sj.jcbfm.9600237

Zhang TT, 2016, FRONT MOL NEUROSCI, V9, DOI 10.3389/fnmol.2016.00101

Zhang Y, 2020, INT J NANOMED, V15, P6917, DOI 10.2147/IJN.S264498

Zhang YY, 2020, J IMMUNOL, V205, P502, DOI 10.4049/jimmunol.2000037

Zhang YJ, 2018, BRAIN RES, V1687, P20, DOI 10.1016/j.brainres.2018.02.030

Zhao C, 2009, NAT STRUCT MOL BIOL, V16, P365, DOI 10.1038/nsmb.1576

Zhao S, 2021, NEUROSCI BIOBEHAV R, V125, P148, DOI 10.1016/j.neubiorev.2021.02.027

Zhao XR, 2015, J NEUROSCI, V35, P11281, DOI 10.1523/JNEUROSCI.1685-15.2015

Zhu B, 2012, STEM CELLS, V30, P2571, DOI 10.1002/stem.1239

Zhu SY, 2021, J CANCER, V12, P54, DOI 10.7150/jca.49692

Zhu XJ, 2020, NEUROPHARMACOLOGY, V170, DOI 10.1016/j.neuropharm.2020.108044

Ziabreva I, 2006, J PSYCHOSOM RES, V61, P311, DOI 10.1016/j.jpsychores.2006.07.017

Ziv Y, 2006, NAT NEUROSCI, V9, P268, DOI 10.1038/nn1629

NR 387

TC 48

Z9 49

U1 6

U2 30

PU SPRINGER

PI NEW YORK

PA ONE NEW YORK PLAZA, SUITE 4600, NEW YORK, NY, UNITED STATES

SN 0893-7648

EI 1559-1182

J9 MOL NEUROBIOL

JI Mol. Neurobiol.

PD FEB

PY 2023

VL 60

IS 2

BP 923

EP 959

DI 10.1007/s12035-022-03102-z

EA NOV 2022

PG 37

WC Neurosciences

WE Science Citation Index Expanded (SCI-EXPANDED)

SC Neurosciences & Neurology

GA A3XK2

UT WOS:000884637800002

PM 36383328

DA 2024-08-12

ER

PT J

AU Wu, YY

Wang, WL

Qiu, XY

Lu, ZG

Ji, WH

Shen, J

Peng, H

Zhao, RC

Wang, JW

Zhang, TL

Yang, J

Zhang, X

AF Wu, Yanyue

Wang, Wenli

Qiu, Xinyu

Lu, Zhiguo

Ji, Weihong

Shen, Jie

Peng, Huan

Zhao, Ruichen

Wang, Jingwen

Zhang, Tianlu

Yang, Jun

Zhang, Xin

TI A STIR nucleic acid drug delivery system for stirring phenotypic switch

of microglia in Parkinson's disease treatments

SO NANO RESEARCH

LA English

DT Article

DE sequential targeting inflammation regulation; phenotypic switch of

microglia; targeted drug delivery; siRNA-based gene therapy; responsive

release; Parkinson's disease

ID NF-KAPPA-B; CELLULAR UPTAKE; NEUROINFLAMMATION; ACTIVATION

AB Neuroinflammation is one of the three important pathological features in neurodegenerative disease including Parkinson's disease (PD). The regulation of neuroinflammation can reduce the severity of neurological damage to alleviate diseases. Numerous studies have shown that the phenotype switch of microglia is tightly associated with the nuclear factor KB (NF -KB) mediated inflammatory pathway. Therefore, the small interfering RNA (siRNA) therapy for downregulating the expression of NF KB, provides a promising therapeutic strategy for Parkinson's disease treatments. Considering the brain delivery challenges of siRNA, a sequential targeting inflammation regulation (STIR) delivery system based on poly(amino acid)s is developed to improve the therapeutic effects of Parkinson's disease treatments. The STIR system sequentially targets the blood-brain barrier and the microglia to enhance the effective concentration of siRNA in the targeted microglia. The results demonstrate that the STIR nanoparticles can transform microglial phenotypes and regulate brain inflammation, thus achieving neuronal recovery and abnormal aggregation of a-synuclein protein (a-syn) reduction in the treatment of Parkinson's disease. Herein, this STIR delivery system provides a promising therapeutic platform in PD treatments and has great potential for other neurodegenerative disease' therapies.

C1 [Wu, Yanyue; Wang, Wenli; Qiu, Xinyu; Lu, Zhiguo; Ji, Weihong; Shen, Jie; Peng, Huan; Zhao, Ruichen; Wang, Jingwen; Zhang, Tianlu; Yang, Jun; Zhang, Xin] Chinese Acad Sci, Inst Proc Engn, State Key Lab Biochem Engn, Beijing 100190, China.

[Wu, Yanyue; Ji, Weihong; Shen, Jie; Peng, Huan; Zhao, Ruichen; Yang, Jun; Zhang, Xin] Univ Chinese Acad Sci, Sch Chem Engn, Beijing 100049, China.

C3 Chinese Academy of Sciences; Institute of Process Engineering, CAS;

Chinese Academy of Sciences; University of Chinese Academy of Sciences,

CAS

RP Yang, J; Zhang, X (corresponding author), Chinese Acad Sci, Inst Proc Engn, State Key Lab Biochem Engn, Beijing 100190, China.; Yang, J; Zhang, X (corresponding author), Univ Chinese Acad Sci, Sch Chem Engn, Beijing 100049, China.

EM jyang2012@ipe.ac.cn; xzhang@ipe.ac.cn

RI Lu, Zhiguo/KQV-3531-2024; shen, jie/JJF-0994-2023; Wu,

Yanyue/GSE-4487-2022; Zhang, Tianlu/GQP-6379-2022

FU National Natural Science Foundation of China [22075289, 21875254,

31771095, 52073287]

FX This work was supported by the National Natural Science Foundation of

China (Nos: 22075289, 21875254, 31771095, and 52073287).

CR Andreou KE, 2017, FRONT ONCOL, V7, DOI 10.3389/fonc.2017.00251

[Anonymous], 2016, MOL NEUROBIOL, V53, P1181

Bengoa-Vergniory N, 2017, ACTA NEUROPATHOL, V134, P819, DOI 10.1007/s00401-017-1755-1

Betarbet R, 2002, BRAIN PATHOL, V12, P499

Dorsey E. R., 2018, LANCET NEUROL, V17, P939

Du XY, 2020, INT J MOL SCI, V21, DOI 10.3390/ijms21228645

Dutta D, 2021, NAT COMMUN, V12, DOI 10.1038/s41467-021-25767-1

Ghosh A, 2007, P NATL ACAD SCI USA, V104, P18754, DOI 10.1073/pnas.0704908104

Huang Z. X., 2022, CHINESE SCI BULL, V2

Jeon S, 2018, NANOMATERIALS-BASEL, V8, DOI 10.3390/nano8121028

Ji WH, 2022, ADV MATER, V34, DOI 10.1002/adma.202105711

Ji XM, 2019, AMB EXPRESS, V9, DOI 10.1186/s13568-019-0869-3

Kanasty R, 2013, NAT MATER, V12, P967, DOI [10.1038/NMAT3765, 10.1038/nmat3765]

Khine YY, 2016, MACROMOL CHEM PHYS, V217, P2302, DOI 10.1002/macp.201600161

Kip E, 2022, AGEING RES REV, V78, DOI 10.1016/j.arr.2022.101618

Krashia P, 2019, NAT COMMUN, V10, DOI 10.1038/s41467-019-11928-w

Lee HJ, 2014, NAT REV NEUROL, V10, P92, DOI 10.1038/nrneurol.2013.275

Lee YJ, 2012, J NEUROINFLAMM, V9, DOI 10.1186/1742-2094-9-35

Li Y, 2018, NANO RES, V11, P5535, DOI 10.1007/s12274-018-2067-z

Li Y, 2018, J AM CHEM SOC, V140, P4164, DOI 10.1021/jacs.8b01641

Lianxu C, 2006, OSTEOARTHR CARTILAGE, V14, P367, DOI 10.1016/j.joca.2005.10.009

Liu RY, 2020, ADV SCI, V7, DOI 10.1002/advs.201901555

Liu Y. J., 2022, CHEM ENG J, V2

Morgan MJ, 2011, CELL RES, V21, P103, DOI 10.1038/cr.2010.178

Motawi TK, 2020, MOL CELL BIOCHEM, V465, P89, DOI 10.1007/s11010-019-03670-0

Patil S, 2007, BIOMATERIALS, V28, P4600, DOI 10.1016/j.biomaterials.2007.07.029

Peng H, 2022, ACS NANO, V16, P869, DOI 10.1021/acsnano.1c08473

Qiao CM, 2018, ADV MATER, V30, DOI 10.1002/adma.201705054

Refolo V, 2019, FRONT CELL NEUROSCI, V13, DOI 10.3389/fncel.2019.00263

Rizk M, 2017, PHARMACOGEN PERS MED, V10, P267, DOI 10.2147/PGPM.S87945

Samii A, 2004, LANCET, V363, P1783, DOI 10.1016/S0140-6736(04)16305-8

Santa-Cecília FV, 2016, NEUROTOX RES, V29, P447, DOI 10.1007/s12640-015-9592-2

Sharma A, 2018, J CONTROL RELEASE, V283, P175, DOI 10.1016/j.jconrel.2018.06.003

Siew JJ, 2018, FRONT MOL NEUROSCI, V11, DOI 10.3389/fnmol.2018.00158

Singh SS, 2020, NEUROTOX RES, V37, P491, DOI 10.1007/s12640-019-00147-2

Takeda K, 2005, INT IMMUNOL, V17, P1, DOI 10.1093/intimm/dxh186

Thawkar BS, 2019, J NEUROIMMUNOL, V326, P62, DOI 10.1016/j.jneuroim.2018.11.010

von Ehr A, 2020, FRONT CELL NEUROSCI, V14, DOI 10.3389/fncel.2020.00066

Xiang Xiujuan, 2022, Exploration (Beijing), V2, P20220008, DOI 10.1002/EXP.20220008

Xu HL, 2015, J CONTROL RELEASE, V199, P84, DOI 10.1016/j.jconrel.2014.12.012

Zhang Y, 2020, PHARMACEUTICS, V12, DOI 10.3390/pharmaceutics12010016

Zhang Yanbin, 2022, Exploration (Beijing), V2, P20220082, DOI 10.1002/EXP.20220082

NR 42

TC 4

Z9 4

U1 11

U2 37

PU TSINGHUA UNIV PRESS

PI BEIJING

PA B605D, XUE YAN BUILDING, BEIJING, 100084, China

SN 1998-0124

EI 1998-0000

J9 NANO RES

JI Nano Res.

PD MAY

PY 2023

VL 16

IS 5

BP 7216

EP 7226

DI 10.1007/s12274-022-5353-5

EA FEB 2023

PG 11

WC Chemistry, Physical; Nanoscience & Nanotechnology; Materials Science,

Multidisciplinary; Physics, Applied

WE Science Citation Index Expanded (SCI-EXPANDED)

SC Chemistry; Science & Technology - Other Topics; Materials Science;

Physics

GA L5NG1

UT WOS:000933469300003

DA 2024-08-12

ER

PT J

AU Di Filippo, M

Chiasserini, D

Tozzi, A

Picconi, B

Calabresi, P

AF Di Filippo, Massimiliano

Chiasserini, Davide

Tozzi, Alessandro

Picconi, Barbara

Calabresi, Paolo

TI Mitochondria and the Link Between Neuroinflammation and

Neurodegeneration

SO JOURNAL OF ALZHEIMERS DISEASE

LA English

DT Review

DE Inflammation; mitochondria; multiple sclerosis; neurodegeneration;

Parkinson's disease

ID MICROGLIAL NADPH OXIDASE; CENTRAL-NERVOUS-SYSTEM; COMPLEX I DEFICIENCY;

parkinson's disease; MULTIPLE-SCLEROSIS; alzheimer's disease;

HUNTINGTONS-DISEASE; NITRIC-OXIDE; SYNAPTIC PLASTICITY; CELL-DEATH

AB The innate immune response is thought to exert a dichotomous role in the brain. Indeed, although molecules of the innate immune response can promote repair mechanisms, during neuroinflammatory processes many harmful mediators are also released. Signs of neuroinflammation and neurodegeneration represent a ubiquitous pathological finding during the course of several different neurological diseases. Interestingly, it has been proposed that mitochondria may exert a crucial role in the pathogenesis of both inflammatory and neurodegenerative central nervous system disorders. In this review, we describe the mechanisms by which neuroinflammation and mitochondrial impairment may synergistically trigger a vicious cycle ultimately leading to neuronal death. In particular, we describe the close relationship existing among neuroinflammation, neurodegeneration, and mitochondrial impairment in three different widely-diffused neurological diseases in which these pathogenetic events coexist, namely multiple sclerosis, Parkinson's disease, and Alzheimer's disease.

C1 [Di Filippo, Massimiliano; Chiasserini, Davide; Tozzi, Alessandro; Calabresi, Paolo] Univ Perugia, Neurol Clin, I-06156 Perugia, Italy.

[Di Filippo, Massimiliano; Chiasserini, Davide; Tozzi, Alessandro; Picconi, Barbara; Calabresi, Paolo] IRCCS, Fdn S Lucia, Rome, Italy.

C3 University of Perugia; IRCCS Santa Lucia

RP Calabresi, P (corresponding author), Univ Perugia, Neurol Clin, Osped S Maria Misericordia, I-06156 Perugia, Italy.

EM calabre@unipg.it

RI Calabresi, Paolo/G-8496-2011; Tozzi, Alessandro/G-7254-2011; Tozzi,

Alessandro/AAB-8707-2022; Chiasserini, Davide/K-7074-2016; Picconi,

Barbara/G-8299-2011

OI Calabresi, Paolo/0000-0003-0326-5509; Tozzi,

Alessandro/0000-0003-3153-2424; Chiasserini, Davide/0000-0002-1169-3258;

Picconi, Barbara/0000-0001-6020-1021

FU European Community (EC) [222918]; Progetto Strategico

FX This review was supported by the European Community (EC) contract number

222918 (REPLACES) FP7-Thematic priority HEALTH (PC), and Progetto

Strategico 2007 (PC, BP).

CR Abou-Sleiman PM, 2006, NAT REV NEUROSCI, V7, P207, DOI 10.1038/nrn1868

Akiyama H, 2000, NEUROBIOL AGING, V21, P383, DOI 10.1016/S0197-4580(00)00124-X

Aktas O, 2005, NEURON, V46, P421, DOI 10.1016/j.neuron.2005.03.018

Aktas O, 2007, ARCH NEUROL-CHICAGO, V64, P185, DOI 10.1001/archneur.64.2.185

Allan SM, 2001, NAT REV NEUROSCI, V2, P734, DOI 10.1038/35094583

Babior BM, 2004, CURR OPIN IMMUNOL, V16, P42, DOI 10.1016/j.coi.2003.12.001

Bal-Price A, 2001, J NEUROSCI, V21, P6480

Balaban RS, 2005, CELL, V120, P483, DOI 10.1016/j.cell.2005.02.001

Bazzoni F, 1996, NEW ENGL J MED, V334, P1717, DOI 10.1056/NEJM199606273342607

Beal MF, 2005, ANN NEUROL, V58, P495, DOI 10.1002/ana.20624

Beattie EC, 2002, SCIENCE, V295, P2282, DOI 10.1126/science.1067859

Björkqvist M, 2009, NEURON, V64, P21, DOI 10.1016/j.neuron.2009.09.034

Blennow K, 2006, LANCET, V368, P387, DOI 10.1016/S0140-6736(20)32205-4

Block ML, 2007, NAT REV NEUROSCI, V8, P57, DOI 10.1038/nrn2038

Brochard V, 2009, J CLIN INVEST, V119, P182, DOI 10.1172/JCI36470

Brouillet E, 1999, PROG NEUROBIOL, V59, P427, DOI 10.1016/S0301-0082(99)00005-2

Browne SE, 1997, ANN NEUROL, V41, P646, DOI 10.1002/ana.410410514

Calabrese V, 2007, NAT REV NEUROSCI, V8, P766, DOI 10.1038/nrn2214

Calabresi P, 2007, TRENDS NEUROSCI, V30, P211, DOI 10.1016/j.tins.2007.03.001

Calabresi P, 2006, LANCET NEUROL, V5, P974, DOI 10.1016/S1474-4422(06)70600-7

Compston A, 2008, LANCET, V372, P1502, DOI 10.1016/S0140-6736(08)61620-7

Costa C, 2008, J NEUROSCI, V28, P8040, DOI 10.1523/JNEUROSCI.1947-08.2008

CSERR HF, 1992, IMMUNOL TODAY, V13, P507, DOI 10.1016/0167-5699(92)90027-5

Dantzer R, 2008, NAT REV NEUROSCI, V9, P46, DOI 10.1038/nrn2297

Dauer W, 2003, NEURON, V39, P889, DOI 10.1016/S0896-6273(03)00568-3

Davalos D, 2005, NAT NEUROSCI, V8, P752, DOI 10.1038/nn1472

Di Filippo M, 2010, J NEUROL NEUROSUR PS, V81, P204, DOI 10.1136/jnnp.2009.171769

Di Filippo M, 2008, TRENDS PHARMACOL SCI, V29, P402, DOI 10.1016/j.tips.2008.06.005

Di Filippo M, 2008, MOVEMENT DISORD, V23, P468, DOI 10.1002/mds.21871

Di Filippo M, 2006, EUR J PHARMACOL, V545, P65, DOI 10.1016/j.ejphar.2006.06.024

Dinis-Oliveira RJ, 2006, NEUROTOXICOLOGY, V27, P1110, DOI 10.1016/j.neuro.2006.05.012

Druzhyna NM, 2005, J BIOL CHEM, V280, P21673, DOI 10.1074/jbc.m411531200

Dutta R, 2006, ANN NEUROL, V59, P478, DOI 10.1002/ana.20736

Ferri A, 2008, NEUROBIOL DIS, V32, P454, DOI 10.1016/j.nbd.2008.08.004

Frank-Cannon TC, 2009, MOL NEURODEGENER, V4, DOI 10.1186/1750-1326-4-47

Fukui H, 2008, TRENDS NEUROSCI, V31, P251, DOI 10.1016/j.tins.2008.02.008

Gao HM, 2003, FASEB J, V17, DOI 10.1096/fj.03-0109fje

Gao HM, 2003, J NEUROSCI, V23, P6181

Gao HM, 2003, J NEUROSCI, V23, P1228, DOI 10.1523/JNEUROSCI.23-04-01228.2003

Glass CK, 2010, CELL, V140, P918, DOI 10.1016/j.cell.2010.02.016

Green DR, 2004, SCIENCE, V305, P626, DOI 10.1126/science.1099320

Gubellini P, 2010, BBA-MOL BASIS DIS, V1802, P151, DOI 10.1016/j.bbadis.2009.08.001

Hemmer B, 2002, NAT REV NEUROSCI, V3, P291, DOI 10.1038/nrn784

Heneka MT, 2007, J NEUROIMMUNOL, V184, P69, DOI 10.1016/j.jneuroim.2006.11.017

Hinchliffe P, 2005, SCIENCE, V309, P771, DOI 10.1126/science.1113988

Hirai K, 2001, J NEUROSCI, V21, P3017, DOI 10.1523/JNEUROSCI.21-09-03017.2001

Hirsch EC, 2009, LANCET NEUROL, V8, P382, DOI 10.1016/S1474-4422(09)70062-6

Huang Y, 2005, CELL MOL IMMUNOL, V2, P113

Imitola J, 2006, ARCH NEUROL-CHICAGO, V63, P25, DOI 10.1001/archneur.63.1.25

Jenner P, 1996, NEUROLOGY, V47, pS161, DOI 10.1212/WNL.47.6_Suppl_3.161S

Jenner P, 2006, NEUROLOGY, V66, pS24, DOI 10.1212/WNL.66.10_suppl_4.S24

Klein C, 2007, NEUROLOGY, V69, P2093, DOI 10.1212/01.wnl.0000271880.27321.a7

LANGSTON JW, 1983, SCIENCE, V219, P979, DOI 10.1126/science.6823561

Le WD, 2001, J NEUROSCI, V21, P8447, DOI 10.1523/JNEUROSCI.21-21-08447.2001

Lees AJ, 2009, LANCET, V373, P2055, DOI 10.1016/S0140-6736(09)60492-X

Levesque S, 2010, BRAIN, V133, P808, DOI 10.1093/brain/awp333

Long-Smith CM, 2009, PROG NEUROBIOL, V89, P277, DOI 10.1016/j.pneurobio.2009.08.001

Mahad D, 2008, BRAIN, V131, P1722, DOI 10.1093/brain/awn105

Mahad DJ, 2009, BRAIN, V132, P1161, DOI 10.1093/brain/awp046

Mao PZ, 2010, BBA-MOL BASIS DIS, V1802, P66, DOI 10.1016/j.bbadis.2009.07.002

Martino G, 2002, LANCET NEUROL, V1, P499, DOI 10.1016/S1474-4422(02)00223-5

Marzo I, 1998, SCIENCE, V281, P2027, DOI 10.1126/science.281.5385.2027

McGeer PL, 2008, MOVEMENT DISORD, V23, P474, DOI 10.1002/mds.21751

Meisel C, 2005, NAT REV NEUROSCI, V6, P775, DOI 10.1038/nrn1765

Merrill JE, 1996, TRENDS NEUROSCI, V19, P331, DOI 10.1016/0166-2236(96)10047-3

Moreira PI, 2010, BBA-MOL BASIS DIS, V1802, P2, DOI 10.1016/j.bbadis.2009.10.006

Napolitano M, 2004, NEUROBIOL DIS, V15, P407, DOI 10.1016/j.nbd.2003.11.021

Nguyen MD, 2002, NAT REV NEUROSCI, V3, P216, DOI 10.1038/nrn752

Nimmerjahn A, 2005, SCIENCE, V308, P1314, DOI 10.1126/science.1110647

PARKER WD, 1989, ANN NEUROL, V26, P719, DOI 10.1002/ana.410260606

Perier C, 2003, TRENDS NEUROSCI, V26, P345, DOI 10.1016/S0166-2236(03)00144-9

Picconi B, 2006, NEUROBIOL DIS, V22, P143, DOI 10.1016/j.nbd.2005.10.009

Picconi B, 2003, NAT NEUROSCI, V6, P501, DOI 10.1038/nn1040

Poyton RO, 2009, TRENDS ENDOCRIN MET, V20, P332, DOI 10.1016/j.tem.2009.04.001

Qin LY, 2007, GLIA, V55, P453, DOI 10.1002/glia.20467

Querfurth HW, 2010, NEW ENGL J MED, V362, P329, DOI 10.1056/NEJMra0909142

Reddy PH, 2008, TRENDS MOL MED, V14, P45, DOI 10.1016/j.molmed.2007.12.002

Reddy PH, 2009, EXP NEUROL, V218, P286, DOI 10.1016/j.expneurol.2009.03.042

Rivest S, 2009, NAT REV IMMUNOL, V9, P429, DOI 10.1038/nri2565

Ryu JK, 2003, NEUROBIOL DIS, V12, P121, DOI 10.1016/S0969-9961(03)00002-0

Saulle E, 2004, MOL CELL NEUROSCI, V25, P9, DOI 10.1016/j.mcn.2003.09.013

SCHAPIRA AHV, 1989, LANCET, V1, P1269, DOI 10.1016/S0140-6736(89)92366-0

SCHAPIRA AHV, 1990, J NEUROCHEM, V54, P823, DOI 10.1111/j.1471-4159.1990.tb02325.x

Schwarting RKW, 1996, PROG NEUROBIOL, V49, P215, DOI 10.1016/S0301-0082(96)00015-9

Shaikh SB, 2009, MOL NEURODEGENER, V4, DOI 10.1186/1750-1326-4-55

Sherer TB, 2003, NEUROSCI LETT, V341, P87, DOI 10.1016/S0304-3940(03)00172-1

Stewart VC, 2000, J NEUROCHEM, V75, P694, DOI 10.1046/j.1471-4159.2000.0750694.x

Stommel EW, 2007, NEUROSCIENCE, V146, P1013, DOI 10.1016/j.neuroscience.2007.02.036

Takeuchi H, 2006, J BIOL CHEM, V281, P21362, DOI 10.1074/jbc.M600504200

Trapp BD, 2009, LANCET NEUROL, V8, P280, DOI 10.1016/S1474-4422(09)70043-2

Volterra A, 2005, NAT REV NEUROSCI, V6, P626, DOI 10.1038/nrn1722

Wallace DC, 1999, SCIENCE, V283, P1482, DOI 10.1126/science.283.5407.1482

Waxman SG, 2006, NAT REV NEUROSCI, V7, P932, DOI 10.1038/nrn2023

Yamamoto M, 2007, AM J PATHOL, V170, P680, DOI 10.2353/ajpath.2007.060378

NR 94

TC 122

Z9 127

U1 0

U2 15

PU IOS PRESS

PI AMSTERDAM

PA NIEUWE HEMWEG 6B, 1013 BG AMSTERDAM, NETHERLANDS

SN 1387-2877

EI 1875-8908

J9 J ALZHEIMERS DIS

JI J. Alzheimers Dis.

PY 2010

VL 20

SU 2

SI SI

BP S369

EP S379

DI 10.3233/JAD-2010-100543

PG 11

WC Neurosciences

WE Science Citation Index Expanded (SCI-EXPANDED)

SC Neurosciences & Neurology

GA 633DL

UT WOS:000280479600010

PM 20463396

OA Green Published, hybrid

DA 2024-08-12

ER

PT J

AU Bao, XY

Zheng, ZZ

Lv, JC

Bao, JD

Chang, ST

Jiang, X

Xin, Y

AF Bao, Xueying

Zheng, Zhuangzhuang

Lv, Jincai

Bao, Jindian

Chang, Sitong

Jiang, Xin

Xin, Ying

TI Shikimic acid (SA) inhibits neuro-inflammation and exerts

neuroprotective effects in an LPS-induced <i>in vitro</i> and <i>in

vivo</i> model

SO FRONTIERS IN PHARMACOLOGY

LA English

DT Article

DE shikimic acid; parkinson's disease; microglia; neuro-inflammation;

neuroprotection

ID parkinson's disease; NEUROINFLAMMATION; MICROGLIA; DEMENTIA

AB Numerous studies have shown that neuroinflammation is involved in the process of neuronal damage in neurodegenerative disease such as Parkinson's disease (PD), for example, and that inhibiting neuroinflammation help improve PD. Shikimic acid (SA) has anti-inflammatory, analgesic and antioxidant activities in numerous diseases. However, its effect and mechanism in PD remain unclear. In this experiment, we found that SA inhibits production of pro-inflammatory mediators and ROS in LPS-induced BV2 cells. Mechanistic studies demonstrated that SA suppresses neuro-inflammation by activating the AKT/Nrf2 pathway and inhibiting the NF-kappa B pathway. Further in vivo study, we confirmed that SA ameliorated the neurological damage and behavioral deficits caused by LPS injection in mice. In summary, these study highlighted the beneficial role of SA as a novel therapy with potential PD drug by targeting neuro-inflammation.

C1 [Bao, Xueying; Zheng, Zhuangzhuang; Lv, Jincai; Bao, Jindian; Chang, Sitong; Jiang, Xin] First Hosp Jilin Univ, Dept Radiat Oncol, Changchun, China.

[Bao, Xueying; Zheng, Zhuangzhuang; Lv, Jincai; Bao, Jindian; Chang, Sitong; Jiang, Xin] First Hosp Jilin Univ, Jilin Prov Key Lab Radiat Oncol & Therapy, Changchun, China.

[Bao, Xueying; Zheng, Zhuangzhuang; Lv, Jincai; Bao, Jindian; Chang, Sitong; Jiang, Xin] Jilin Univ, Sch Publ Hlth, NHC Key Lab Radiobiol, Changchun, China.

[Xin, Ying] Jilin Univ, Key Lab Pathobiol, Minist Educ, Changchun, China.

[Xin, Ying] Jilin Univ, Coll Basic Med Sci, Changchun, China.

C3 Jilin University; Jilin University; Jilin University; Jilin University;

Jilin University

RP Jiang, X (corresponding author), First Hosp Jilin Univ, Dept Radiat Oncol, Changchun, China.; Jiang, X (corresponding author), First Hosp Jilin Univ, Jilin Prov Key Lab Radiat Oncol & Therapy, Changchun, China.; Jiang, X (corresponding author), Jilin Univ, Sch Publ Hlth, NHC Key Lab Radiobiol, Changchun, China.; Xin, Y (corresponding author), Jilin Univ, Key Lab Pathobiol, Minist Educ, Changchun, China.; Xin, Y (corresponding author), Jilin Univ, Coll Basic Med Sci, Changchun, China.

EM jiangx@jlu.edu.cn; xiny@jlu.edu.cn

RI Jiang, Xin/AHA-9454-2022

OI Jiang, Xin/0000-0002-4613-7438; Xin, Ying/0000-0001-7591-9423

FU The author(s) declare financial support was received for the research,

authorship, and/or publication of this article. This work was funded by

the Jilin Provincial Science and Technology Foundation (grant number

20230508064RC), the Achievement Transformati [20230508064RC]; Jilin

Provincial Science and Technology Foundation [CGZHYD202012-029];

Achievement Transformation Guiding Foundations of First Hospital of

Jilin University

FX The author(s) declare financial support was received for the research,

authorship, and/or publication of this article. This work was funded by

the Jilin Provincial Science and Technology Foundation (grant number

20230508064RC), the Achievement Transformation Guiding Foundations of

First Hospital of Jilin University (grant number CGZHYD202012-029).

CR Badanjak K, 2021, INT J MOL SCI, V22, DOI 10.3390/ijms22094676

Bencs V, 2020, ORVOSI HETILAP, V161, P727, DOI 10.1556/650.2020.31715

Calsolaro V, 2016, ALZHEIMERS DEMENT, V12, P719, DOI 10.1016/j.jalz.2016.02.010

Cerro AR, 2022, BIOINFORMATICS, V38, P4246, DOI 10.1093/bioinformatics/btac511

De Virgilio A, 2016, AUTOIMMUN REV, V15, P1005, DOI 10.1016/j.autrev.2016.07.022

Emre M, 2007, MOVEMENT DISORD, V22, P1689, DOI 10.1002/mds.21507

Feldman RA, 2022, ELIFE, V11, DOI 10.7554/eLife.81890

Gao XY, 2020, IMMUNOBIOLOGY, V225, DOI 10.1016/j.imbio.2020.151965

Grotemeyer A, 2022, FRONT IMMUNOL, V13, DOI 10.3389/fimmu.2022.878771

Han VX, 2021, NAT REV NEUROL, V17, P564, DOI 10.1038/s41582-021-00530-8

He DW, 2022, FRONT PHARMACOL, V12, DOI 10.3389/fphar.2021.787771

He DW, 2021, FRONT IMMUNOL, V12, DOI 10.3389/fimmu.2021.619761

Heidari A, 2022, J NEUROINFLAMM, V19, DOI 10.1186/s12974-022-02496-w

Hirsch EC, 2009, LANCET NEUROL, V8, P382, DOI 10.1016/S1474-4422(09)70062-6

Ho MS, 2019, ADV EXP MED BIOL, V1175, P335, DOI 10.1007/978-981-13-9913-8_13

Hou W, 2018, FUTURE MED CHEM, V10, P2253, DOI 10.4155/fmc-2018-0172

Jankovic J, 2020, J NEUROL NEUROSUR PS, V91, P795, DOI 10.1136/jnnp-2019-322338

Leng FD, 2021, NAT REV NEUROL, V17, P157, DOI 10.1038/s41582-020-00435-y

Liang H, 2018, J MUSCULOSKEL NEURON, V18, P551

Liu BB, 2020, BIOMED PHARMACOTHER, V125, DOI 10.1016/j.biopha.2020.109895

Liu L, 2021, CELL BIOL INT, V45, P422, DOI 10.1002/cbin.11505

Lu FF, 2019, NEUROSCI BULL, V35, P434, DOI 10.1007/s12264-018-0322-7

Catorce MN, 2016, CURR NEUROPHARMACOL, V14, P155, DOI 10.2174/1570159X14666151204122017

Pajares M, 2020, CELLS-BASEL, V9, DOI 10.3390/cells9071687

Patra JK, 2020, PHYTOTHER RES, V34, P1248, DOI 10.1002/ptr.6614

Peng X, 2019, CELL DEATH DIS, V10, DOI 10.1038/s41419-019-1483-7

Pottorf TS, 2022, CELLS-BASEL, V11, DOI 10.3390/cells11132083

Rabelo TK, 2016, INT IMMUNOPHARMACOL, V39, P97, DOI 10.1016/j.intimp.2016.07.016

Reich SG, 2019, MED CLIN N AM, V103, P337, DOI 10.1016/j.mcna.2018.10.014

Samii A, 2004, LANCET, V363, P1783, DOI 10.1016/S0140-6736(04)16305-8

Sampson TR, 2016, CELL, V167, P1469, DOI 10.1016/j.cell.2016.11.018

Sarkar S, 2020, J CLIN INVEST, V130, P4195, DOI 10.1172/JCI136174

Shi Y, 2021, FOOD CHEM, V342, DOI 10.1016/j.foodchem.2020.128359

Snyder A, 2021, J PARKINSON DIS, V11, P1995, DOI 10.3233/JPD-212705

Suna JY, 2016, PHARM BIOL, V54, P2282, DOI 10.3109/13880209.2016.1153663

Teleanu DM, 2022, INT J MOL SCI, V23, DOI 10.3390/ijms23115938

Williams GP, 2021, BRAIN, V144, P2047, DOI 10.1093/brain/awab103

Xing JF, 2012, DIGEST DIS SCI, V57, P2045, DOI 10.1007/s10620-012-2155-y

Xing JF, 2012, INFLAMMATION, V35, P1872, DOI 10.1007/s10753-012-9509-7

You HB, 2021, FRONT PHARMACOL, V12, DOI 10.3389/fphar.2021.634822

Zusso M, 2019, J NEUROINFLAMM, V16, DOI 10.1186/s12974-019-1538-9

NR 41

TC 3

Z9 3

U1 3

U2 6

PU FRONTIERS MEDIA SA

PI LAUSANNE

PA AVENUE DU TRIBUNAL FEDERAL 34, LAUSANNE, CH-1015, SWITZERLAND

EI 1663-9812

J9 FRONT PHARMACOL

JI Front. Pharmacol.

PD NOV 2

PY 2023

VL 14

AR 1265571

DI 10.3389/fphar.2023.1265571

PG 14

WC Pharmacology & Pharmacy

WE Science Citation Index Expanded (SCI-EXPANDED)

SC Pharmacology & Pharmacy

GA Y4KY6

UT WOS:001104979700001

PM 38026972

OA Green Published, gold

DA 2024-08-12

ER

PT J

AU Taing, K

Chen, L

Weng, HR

AF Taing, Kyle

Chen, Lawrence

Weng, Han-Rong

TI Emerging roles of GPR109A in regulation of neuroinflammation in

neurological diseases and pain

SO NEURAL REGENERATION RESEARCH

LA English

DT Review

DE beta-hydroxybutyrate; cytokines; HCAR2; lupus; neuroinflammation;

neuropathic; niacin; nociception; synaptic

ID PROTEIN-COUPLED RECEPTOR; SPINAL DORSAL-HORN; NICOTINIC-ACID; DIMETHYL

FUMARATE; BETA-HYDROXYBUTYRATE; OXIDATIVE STRESS; MONOMETHYL FUMARATE;

MOLECULAR-IDENTIFICATION; INTRACEREBRAL HEMORRHAGE; CENTRAL

SENSITIZATION

AB Neuroinflammation plays a critical role in the pathological process of multiple neurological disorders and pathological pain conditions. GPR109A, a Gi protein-coupled receptor, has emerged as an important therapeutic target for controlling inflammation in various tissues and organs. In this review, we summarized current data about the role of GPR109A in neuroinflammation. Specifically, we focused on the pharmacological features of GPR109A and signaling pathways used by GPR109A to ameliorate neuroinflammation and symptoms in Alzheimer's disease, Parkinson's disease, multiple sclerosis, stroke, and pathological pain conditions.

C1 [Taing, Kyle; Chen, Lawrence; Weng, Han-Rong] Calif Northstate Univ, Coll Med, Dept Basic Sci, Elk Grove, CA 95757 USA.

RP Weng, HR (corresponding author), Calif Northstate Univ, Coll Med, Dept Basic Sci, Elk Grove, CA 95757 USA.

EM han-rong.weng@cnsu.edu

FU NIH [NS107569]

FX This work was supported by the NIH RO1 grant (NS107569) to HRW.

CR Ahmad MA, 2022, INT J MOL SCI, V23, DOI 10.3390/ijms23020616

Ahmad M, 2014, CNS NEUROL DISORD-DR, V13, P1378, DOI 10.2174/1871527313666141023094720

Bachiller S, 2018, FRONT CELL NEUROSCI, V12, DOI 10.3389/fncel.2018.00488

Badanjak K, 2021, INT J MOL SCI, V22, DOI 10.3390/ijms22094676

Boatman PD, 2012, J MED CHEM, V55, P3644, DOI 10.1021/jm2010964

Boccella S, 2019, FASEB J, V33, P1062, DOI 10.1096/fj.201801033R

Brown AJ, 2003, J BIOL CHEM, V278, P11312, DOI 10.1074/jbc.M211609200

Cao DL, 2014, EXP NEUROL, V261, P328, DOI 10.1016/j.expneurol.2014.05.014

Chai JT, 2013, CURR ATHEROSCLER REP, V15, DOI 10.1007/s11883-013-0325-9

Chen G, 2018, NEURON, V100, P1292, DOI 10.1016/j.neuron.2018.11.009

Chen H, 2014, J CLIN INVEST, V124, P2188, DOI 10.1172/JCI72151

Chen JR, 2021, COMMUN BIOL, V4, DOI 10.1038/s42003-020-01564-2

Clausen BH, 2017, EXP NEUROL, V295, P144, DOI 10.1016/j.expneurol.2017.06.011

Cui X, 2010, STROKE, V41, P2044, DOI 10.1161/STROKEAHA.110.589333

Digby JE, 2012, ARTERIOSCL THROM VAS, V32, P669, DOI 10.1161/ATVBAHA.111.241836

Digby JE, 2010, ATHEROSCLEROSIS, V209, P89, DOI 10.1016/j.atherosclerosis.2009.08.045

DiSabato DJ, 2016, J NEUROCHEM, V139, P136, DOI 10.1111/jnc.13607

Dobbins RL, 2013, DIABETES OBES METAB, V15, P1013, DOI 10.1111/dom.12132

Elangovan S, 2014, CANCER RES, V74, P1166, DOI 10.1158/0008-5472.CAN-13-1451

Felizardo RJF, 2019, FASEB J, V33, P11894, DOI 10.1096/fj.201901080R

Fu SP, 2015, J NEUROINFLAMM, V12, DOI 10.1186/s12974-014-0230-3

Fu SP, 2014, MEDIAT INFLAMM, V2014, DOI 10.1155/2014/983401

Ghimire S, 2021, FRONT IMMUNOL, V12, DOI 10.3389/fimmu.2021.753287

Gille A, 2008, ANNU REV PHARMACOL, V48, P79, DOI 10.1146/annurev.pharmtox.48.113006.094746

Giri B, 2019, INT J MOL SCI, V20, DOI 10.3390/ijms20184559

Grace PM, 2014, NAT REV IMMUNOL, V14, P217, DOI 10.1038/nri3621

Gregus AM, 2021, PAIN, V162, P2186, DOI 10.1097/j.pain.0000000000002215

Guan ZH, 2016, NAT NEUROSCI, V19, P94, DOI 10.1038/nn.4189

Guo TZ, 2021, ANESTH ANALG, V132, P1475, DOI 10.1213/ANE.0000000000005440

Guo WJ, 2021, INT J BIOL SCI, V17, P4271, DOI 10.7150/ijbs.62380

Hanson J, 2010, J CLIN INVEST, V120, P2910, DOI 10.1172/JCI42273

Hartmann Andreas, 2004, Dialogues Clin Neurosci, V6, P281

Healy LM, 2022, NAT REV NEUROL, V18, P237, DOI 10.1038/s41582-022-00624-x

Israel JE, 2021, HEALTH PSYCHOL RES, V9, DOI 10.52965/001c.25535

Jadeja RN, 2019, AGING-US, V11, P386, DOI 10.18632/aging.101743

James SL, 2018, LANCET, V392, P1789, DOI [10.1016/s0140-6736(18)32335-3, 10.1016/S0140-6736(18)32335-3]

Jayaraj RL, 2019, J NEUROINFLAMM, V16, DOI 10.1186/s12974-019-1516-2

Jung JK, 2007, J MED CHEM, V50, P1445, DOI 10.1021/jm070022x

Kamanna VS, 2007, AM J CARDIOL, V100, p53N, DOI 10.1016/j.amjcard.2007.09.080

Kawasaki Y, 2008, J NEUROSCI, V28, P5189, DOI 10.1523/JNEUROSCI.3338-07.2008

Knopman DS, 2021, NAT REV DIS PRIMERS, V7, DOI 10.1038/s41572-021-00269-y

Knowles HJ, 2006, BIOCHEM PHARMACOL, V71, P646, DOI 10.1016/j.bcp.2005.11.019

Kobayashi K, 2008, J NEUROSCI, V28, P2892, DOI 10.1523/JNEUROSCI.5589-07.2008

Koppel SJ, 2018, NEUROCHEM INT, V117, P114, DOI 10.1016/j.neuint.2017.05.019

Kostylina G, 2008, CELL DEATH DIFFER, V15, P134, DOI 10.1038/sj.cdd.4402238

Lacagnina MJ, 2021, PAIN REP, V6, DOI 10.1097/PR9.0000000000000905

Lal R, 2021, EUR J PHARMACOL, V899, DOI 10.1016/j.ejphar.2021.174044

Lee AK, 2020, AGING DIS, V11, P777, DOI 10.14336/AD.2019.0926

Leng FD, 2021, NAT REV NEUROL, V17, P157, DOI 10.1038/s41582-020-00435-y

De Sousa RAL, 2022, METAB BRAIN DIS, V37, P851, DOI 10.1007/s11011-022-00953-2

Li JH, 2020, ANESTHESIOLOGY, V132, P343, DOI 10.1097/ALN.0000000000003077

Li Y, 2021, ACS CHEM NEUROSCI, V12, P1219, DOI 10.1021/acschemneuro.1c00046

Li Y, 2021, CURR NEUROVASC RES, V18, P456, DOI 10.2174/1567202618666211109105024

Lim JL, 2016, MICROVASC RES, V105, P61, DOI 10.1016/j.mvr.2015.12.003

Lin RH, 2016, J NEUROINFLAMM, V13, DOI 10.1186/s12974-016-0733-1

Liu FX, 2014, ANN CLIN LAB SCI, V44, P443

Maciejewski-Lenoir D, 2006, J INVEST DERMATOL, V126, P2637, DOI 10.1038/sj.jid.5700586

Mandrika I, 2010, BIOCHEM BIOPH RES CO, V395, P281, DOI 10.1016/j.bbrc.2010.04.011

Martin PM, 2009, MOL VIS, V15, P362

McCarty MF, 2015, MED HYPOTHESES, V85, P631, DOI 10.1016/j.mehy.2015.08.002

Muzio L, 2021, FRONT NEUROSCI-SWITZ, V15, DOI 10.3389/fnins.2021.742065

Nimmerjahn A, 2005, SCIENCE, V308, P1314, DOI 10.1126/science.1110647

Offermanns S, 2011, PHARMACOL REV, V63, P269, DOI 10.1124/pr.110.003301

Parodi B, 2015, ACTA NEUROPATHOL, V130, P279, DOI 10.1007/s00401-015-1422-3

Pike NB, 2005, J CLIN INVEST, V115, P3400, DOI 10.1172/JCI27160

Plaisance EP, 2009, AM J PHYSIOL-ENDOC M, V296, pE549, DOI 10.1152/ajpendo.91004.2008

Poewe W, 2017, NAT REV DIS PRIMERS, V3, DOI 10.1038/nrdp.2017.13

Qian J, 2017, BRIT J PHARMACOL, V174, P1961, DOI 10.1111/bph.13788

Rahman M, 2014, NAT COMMUN, V5, DOI 10.1038/ncomms4944

Rajman L, 2018, CELL METAB, V27, P529, DOI 10.1016/j.cmet.2018.02.011

Rezq S, 2016, J PHARMACOL EXP THER, V356, P456, DOI 10.1124/jpet.115.229146

Safari A, 2019, IRAN J NEUROL, V18, P108

SATO K, 1995, FASEB J, V9, P651, DOI 10.1096/fasebj.9.8.7768357

Schulze-Topphoff U, 2016, P NATL ACAD SCI USA, V113, P4777, DOI 10.1073/pnas.1603907113

Shehadah A, 2010, NEUROBIOL DIS, V40, P277, DOI 10.1016/j.nbd.2010.05.034

Shimazu T, 2013, SCIENCE, V339, P211, DOI 10.1126/science.1227166

Singh V, 2012, CELL HOST MICROBE, V12, P669, DOI 10.1016/j.chom.2012.09.012

Soga T, 2003, BIOCHEM BIOPH RES CO, V303, P364, DOI 10.1016/S0006-291X(03)00342-5

Sun JX, 2020, MEDIAT INFLAMM, V2020, DOI 10.1155/2020/7605160

Sun L, 2012, J NEUROSCI, V32, P11330, DOI 10.1523/JNEUROSCI.0677-12.2012

Suzuki M, 2002, JPN J PHARMACOL, V89, P36, DOI 10.1254/jjp.89.36

Taggart AKP, 2005, J BIOL CHEM, V280, P26649, DOI 10.1074/jbc.C500213200

Tang H, 2008, BIOCHEM BIOPH RES CO, V375, P562, DOI 10.1016/j.bbrc.2008.08.041

Taves S, 2013, NEURAL PLAST, V2013, DOI 10.1155/2013/753656

Thangaraju M, 2009, CANCER RES, V69, P2826, DOI 10.1158/0008-5472.CAN-08-4466

Thompson A, 2020, NAT REV NEUROL, V16, P589, DOI 10.1038/s41582-020-00421-4

Tschoe C, 2020, J STROKE, V22, P29

Tsuda M, 2013, J PHARMACOL SCI, V121, P89, DOI 10.1254/jphs.12R14CP

Tunaru S, 2003, NAT MED, V9, P352, DOI 10.1038/nm824

Tuteja S, 2017, PHARMACOGENET GENOM, V27, P285, DOI [10.1097/FPC.0000000000000289, 10.1097/fpc.0000000000000289]

Venci JV, 2013, ANN PHARMACOTHER, V47, P1697, DOI 10.1177/1060028013509232

Viatchenko-Karpinski V, 2022, GLIA, V70, P634, DOI 10.1002/glia.24130

Wakade C, 2018, J NEUROIMMUNOL, V320, P76, DOI 10.1016/j.jneuroim.2018.05.002

Wang XM, 2017, EXP ANIM TOKYO, V66, P177, DOI 10.1538/expanim.16-0090

Wise A, 2003, J BIOL CHEM, V278, P9869, DOI 10.1074/jbc.M210695200

Won YJ, 2013, J NEUROSCI, V33, P19314, DOI 10.1523/JNEUROSCI.3102-13.2013

Woolf CJ, 2011, PAIN, V152, pS2, DOI 10.1016/j.pain.2010.09.030

Wu YC, 2020, FASEB J, V34, P1412, DOI 10.1096/fj.201901984R

Yan X, 2017, J NEUROCHEM, V140, P963, DOI 10.1111/jnc.13952

Yan XS, 2015, J NEUROINFLAMM, V12, DOI 10.1186/s12974-014-0222-3

Yan XS, 2014, GLIA, V62, P1093, DOI 10.1002/glia.22665

Yan XS, 2013, J BIOL CHEM, V288, P30544, DOI 10.1074/jbc.M113.495465

Yao Y, 2016, TRANSL STROKE RES, V7, P535, DOI 10.1007/s12975-016-0496-0

Zellner C, 2005, HUM MUTAT, V25, P18, DOI 10.1002/humu.20121

Zhang SJ, 2021, ADV SCI, V8, DOI 10.1002/advs.202003410

Zhao XR, 2015, STROKE, V46, P1923, DOI 10.1161/STROKEAHA.115.009398

Zhu HN, 2020, EXP THER MED, V20, DOI 10.3892/etm.2020.9399

NR 107

TC 14

Z9 15

U1 5

U2 23

PU WOLTERS KLUWER MEDKNOW PUBLICATIONS

PI MUMBAI

PA WOLTERS KLUWER INDIA PVT LTD , A-202, 2ND FLR, QUBE, C T S NO 1498A-2

VILLAGE MAROL, ANDHERI EAST, MUMBAI, Maharashtra, INDIA

SN 1673-5374

EI 1876-7958

J9 NEURAL REGEN RES

JI Neural Regen. Res.

PD APR

PY 2023

VL 18

IS 4

BP 763

EP 768

DI 10.4103/1673-5374.354514

PG 6

WC Cell Biology; Neurosciences

WE Science Citation Index Expanded (SCI-EXPANDED)

SC Cell Biology; Neurosciences & Neurology

GA 7I2KX

UT WOS:000903723200010

PM 36204834

OA gold, Green Published

DA 2024-08-12

ER

PT J

AU Garodia, P

Hegde, M

Kunnumakkara, AB

Aggarwal, BB

AF Garodia, Prachi

Hegde, Mangala

Kunnumakkara, Ajaikumar B.

Aggarwal, Bharat B.

TI Curcumin, inflammation, and neurological disorders: How are they linked?

SO INTEGRATIVE MEDICINE RESEARCH

LA English

DT Review

DE Alzheimer's disease; Curcumin; Inflammation; Neurological disorders;

Parkinson's disease

ID TRAUMATIC BRAIN-INJURY; EXPERIMENTAL AUTOIMMUNE ENCEPHALOMYELITIS;

NEURAL PROGENITOR CELLS; CENTRAL-NERVOUS-SYSTEM; OXIDATIVE STRESS;

parkinson's disease; MULTIPLE-SCLEROSIS; COGNITIVE IMPAIRMENT;

alzheimer's disease; EXPERIMENTAL-MODEL

AB Background: Despite the extensive research in recent years, the current treatment modalities for neurological disorders are suboptimal. Curcumin, a polyphenol found in Curcuma genus, has been shown to mitigate the pathophysiology and clinical sequalae involved in neuroinflammation and neurodegenerative disease. Methods: We searched PubMed database for relevant publications on curcumin and its uses in treating neuro-logical diseases. We also reviewed relevant clinical trials which appeared on searching PubMed database using 'Curcumin and clinical trials'. Results: This review details the pleiotropic immunomodulatory functions and neuroprotective properties of cur -cumin, its derivatives and formulations in various preclinical and clinical investigations. The effects of curcumin on neurodegenerative disease such as Alzheimer's disease (AD), amyotrophic lateral sclerosis (ALS), brain tu-mors, epilepsy, Huntington's disorder (HD), ischemia, Parkinson's disease (PD), multiple sclerosis (MS), and trau-matic brain injury (TBI) with a major focus on associated signalling pathways have been thoroughly discussed. Conclusion: This review demonstrates curcumin can suppress spinal neuroinflammation by modulating diverse astroglia mediated cascades, ensuring the treatment of neurological disorders.

C1 [Garodia, Prachi] Integrat Res Ctr, Miami, FL 33199 USA.

[Hegde, Mangala; Kunnumakkara, Ajaikumar B.] Indian Inst Technol Guwahati, Dept Biosci & Bioengn, Gauhati, Assam, India.

[Aggarwal, Bharat B.] Inflammat Res Ctr, San Diego, CA 92109 USA.

C3 Indian Institute of Technology System (IIT System); Indian Institute of

Technology (IIT) - Guwahati

RP Garodia, P (corresponding author), Integrat Res Ctr, Miami, FL 33199 USA.; Aggarwal, BB (corresponding author), Inflammat Res Ctr, San Diego, CA 92109 USA.

EM prachigarodia@hotmail.com; bbaggarwal@gmail.com

RI Kunnumakkara, Ajaikumar B/AAH-5214-2019; Garodia, Prachi/AAN-9114-2021

OI Kunnumakkara, Ajaikumar B/0000-0001-9121-6816; Garodia,

Prachi/0000-0003-1799-8789; Hegde, Mangala/0000-0003-2926-896X

FU Department of Biotechnology (DBT), Government of India

[BT/556/NE/U-Excel/2016]

FX This project was supported by BT/556/NE/U-Excel/2016 grant awarded to

ABK by Department of Biotechnology (DBT), Government of India.

CR Abdolahi M, 2021, BMC RES NOTES, V14, DOI 10.1186/s13104-021-05700-x

Abdolahi M, 2019, ENDOCR METAB IMMUNE, V19, P874, DOI 10.2174/1871530319666190212170140

Abdolahi M, 2018, CNS NEUROL DISORD-DR, V17, P430, DOI 10.2174/1871527317666180625101643

Abdolahi M, 2017, IMMUNOGENETICS, V69, P371, DOI 10.1007/s00251-017-0992-8

Abdul-Latif R, 2021, J INTEGR MED-JIM, V19, P300, DOI 10.1016/j.joim.2021.03.007

Abdulmalek S, 2021, PHARMACEUTICS, V13, DOI 10.3390/pharmaceutics13111937

Aditi K, 2022, EXP BIOL MED, V247, P152, DOI 10.1177/15353702211046927

Agarwal NB, 2013, FUND CLIN PHARMACOL, V27, P169, DOI 10.1111/j.1472-8206.2011.01002.x

Agarwal NB, 2011, PHYTOMEDICINE, V18, P756, DOI 10.1016/j.phymed.2010.11.007

Ahmad M, 2013, SAUDI J BIOL SCI, V20, P155, DOI 10.1016/j.sjbs.2013.01.002

Ahmadi M, 2018, NEUROTHERAPEUTICS, V15, P430, DOI 10.1007/s13311-018-0606-7

Ahmadi N, 2021, BRAIN RES, V1766, DOI 10.1016/j.brainres.2021.147517

Ali OAMA, 2022, CNS NEUROL DISORD-DR, V21, P994, DOI 10.2174/1871527321666211221162104

Allison DJ, 2017, SPINAL CORD, V55, P26, DOI 10.1038/sc.2016.96

Amalraj A, 2019, Curcumin For Neurological and Psychiatric Disorders Elsevier, P105

Anand P, 2007, MOL PHARMACEUT, V4, P807, DOI 10.1021/mp700113r

Arun N, 2002, PLANT FOOD HUM NUTR, V57, P41, DOI 10.1023/A:1013106527829

Ascherio A, 2016, SEMIN NEUROL, V36, P103, DOI 10.1055/s-0036-1579693

Ashina M, 2021, LANCET, V397, P1505, DOI 10.1016/S0140-6736(20)32342-4

Atabaki R, 2020, INT IMMUNOPHARMACOL, V86, DOI 10.1016/j.intimp.2020.106720

Barzegarzadeh B, 2021, NEUROTOX RES, V39, P815, DOI 10.1007/s12640-020-00310-0

Benameur T, 2022, MOLECULES, V27, DOI 10.3390/molecules27010236

Benatar M, 2022, BRAIN, V145, P27, DOI 10.1093/brain/awab404

Bertoncello KT, 2018, SCI REP-UK, V8, DOI 10.1038/s41598-018-20897-x

Bharal N, 2008, PHYTOTHER RES, V22, P1660, DOI 10.1002/ptr.2551

Bhatia NK, 2015, BBA-PROTEINS PROTEOM, V1854, P426, DOI 10.1016/j.bbapap.2015.01.014

Bonilla L, 2022, EPILEPSIA OPEN, V7, pS121, DOI 10.1002/epi4.12567

Bonini NM, 2003, ANNU REV NEUROSCI, V26, P627, DOI 10.1146/annurev.neuro.26.041002.131425

Breijyeh Z, 2020, MOLECULES, V25, DOI 10.3390/molecules25245789

Buccarello L, 2020, BIOCHEM PHARMACOL, V178, DOI 10.1016/j.bcp.2020.114066

Bulboaca AE, 2019, MED SCI MONITOR, V25, P5087, DOI 10.12659/MSM.915607

Bulboaca AE, 2018, INT J NANOMED, V13, P3093, DOI 10.2147/IJN.S162087

Bulboaca AE, 2017, BIOMED RES INT, V2017, DOI 10.1155/2017/4754701

Buse DC, 2019, HEADACHE, V59, P306, DOI 10.1111/head.13459

Çakmak G, 2022, CASP J INTERN MED, V13, P16, DOI 10.22088/cjim.13.1.16

Carter RJ, 1999, J NEUROSCI, V19, P3248

Cavalcanti R. N., 2022, Uses and applications of extracts from natural sources, DOI [10.1039/9781839165894-00001, DOI 10.1039/9781839165894-00001]

Chen J, 2006, APOPTOSIS, V11, P943, DOI 10.1007/s10495-006-6715-5

Chevin M, 2022, BIOMEDICINES, V10, DOI 10.3390/biomedicines10112913

Chia R, 2018, LANCET NEUROL, V17, P94, DOI 10.1016/S1474-4422(17)30401-5

Chico L, 2018, CNS NEUROL DISORD-DR, V17, P767, DOI 10.2174/1871527317666180720162029

Chongtham A, 2016, SCI REP-UK, V6, DOI 10.1038/srep18736

Choudhary KM, 2013, EUR J PHARMACOL, V704, P33, DOI 10.1016/j.ejphar.2013.02.012

Comabella M, 2012, CLIN IMMUNOL, V142, P2, DOI 10.1016/j.clim.2011.03.004

Cores A, 2022, ANTIOXIDANTS-BASEL, V11, DOI 10.3390/antiox11010028

Cornelius C, 2013, ANTIOXID REDOX SIGN, V19, P836, DOI 10.1089/ars.2012.4981

Correale J, 2017, BRAIN, V140, P527, DOI 10.1093/brain/aww258

Cote B, 2022, MOLECULES, V27, DOI 10.3390/molecules27030781

Coulom H, 2004, J NEUROSCI, V24, P10993, DOI 10.1523/JNEUROSCI.2993-04.2004

Dai W, 2018, BRAIN RES BULL, V140, P65, DOI 10.1016/j.brainresbull.2018.03.020

de Guzman ACV, 2022, NANOMATERIALS-BASEL, V12, DOI 10.3390/nano12050758

De Lorenzi E, 2022, INT J MOL SCI, V23, DOI 10.3390/ijms23084381

Deng Isaac, 2022, Front Biosci (Schol Ed), V14, P13, DOI 10.31083/j.fbs1402013

Dibacto REK, 2022, HELIYON, V8, DOI 10.1016/j.heliyon.2022.e09896

Ding CY, 2022, FRONT PUBLIC HEALTH, V10, DOI 10.3389/fpubh.2022.952161

Djalali M, 2020, COMPLEMENT THER CLIN, V41, DOI 10.1016/j.ctcp.2020.101256

Djalali M, 2020, REP BIOCHEM MOL BIOL, V9, P1, DOI 10.29252/rbmb.9.1.1

Dobson R, 2019, EUR J NEUROL, V26, P27, DOI 10.1111/ene.13819

Dolati S, 2019, J NEUROIMMUNOL, V327, P15, DOI 10.1016/j.jneuroim.2019.01.007

Dolati S, 2018, PHARMACOL REP, V70, P1158, DOI 10.1016/j.pharep.2018.05.008

Dolati S, 2018, INT IMMUNOPHARMACOL, V61, P74, DOI 10.1016/j.intimp.2018.05.018

Dolati S, 2018, J CELL PHYSIOL, V233, P5222, DOI 10.1002/jcp.26301

Dong H, 2014, NEUROSCIENCE, V272, P141, DOI 10.1016/j.neuroscience.2014.04.032

Dong WW, 2018, TOXICOL APPL PHARM, V346, P28, DOI 10.1016/j.taap.2018.03.020

Dong XW, 2022, ENVIRON SCI POLLUT R, V29, P74619, DOI 10.1007/s11356-022-20943-2

Drion CM, 2022, EPILEPSY RES, V181, DOI 10.1016/j.eplepsyres.2022.106873

Drion CM, 2019, EPILEPSIA, V60, P605, DOI 10.1111/epi.14667

Du P, 2012, CHINESE MED J-PEKING, V125, P1975, DOI 10.3760/cma.j.issn.0366-6999.2012.11.025

Du P, 2009, CHINESE MED J-PEKING, V122, P1435, DOI 10.3760/cma.j.issn.0366-6999.2009.12.015

Du XX, 2012, NEUROSCI BULL, V28, P253, DOI 10.1007/s12264-012-1238-2

Duan WS, 2014, MOL NEUROBIOL, V49, P368, DOI 10.1007/s12035-013-8525-4

Dugger BN, 2017, CSH PERSPECT BIOL, V9, DOI 10.1101/cshperspect.a028035

El Nebrisi E, 2020, INT J MOL SCI, V21, DOI 10.3390/ijms21197329

ELBini-Dhouib I, 2022, INT J MOL SCI, V23, DOI 10.3390/ijms23158658

ELBini-Dhouib I, 2021, MOLECULES, V26, DOI 10.3390/molecules26103011

Esmaeilzadeh E, 2019, DRUG DEVELOP RES, V80, P629, DOI 10.1002/ddr.21540

Esquerda-Canals G, 2017, J ALZHEIMERS DIS, V57, P1171, DOI 10.3233/JAD-170045

Ezz HSA, 2011, NEUROCHEM RES, V36, P2195, DOI 10.1007/s11064-011-0544-9

Farkhondeh T, 2019, J CELL PHYSIOL, V234, P16953, DOI 10.1002/jcp.28411

Feldman EL, 2022, LANCET, V400, P1363, DOI 10.1016/S0140-6736(22)01272-7

Forouzanfar Fatemeh, 2021, Adv Exp Med Biol, V1291, P363, DOI 10.1007/978-3-030-56153-6_21

Gao CH, 2020, J NANOBIOTECHNOL, V18, DOI 10.1186/s12951-020-00626-1

Gao YY, 2016, J SURG RES, V206, P67, DOI 10.1016/j.jss.2016.07.014

Gharaibeh A, 2020, INT J MOL SCI, V21, DOI 10.3390/ijms21249542

Ghodsi H, 2022, CLIN NEUROL NEUROSUR, V218, DOI 10.1016/j.clineuro.2022.107300

Gilles C, 2000, Dialogues Clin Neurosci, V2, P247

Girisa S, 2021, ACS PHARMACOL TRANSL, V4, P647, DOI 10.1021/acsptsci.1c00017

Goldenberg Marvin M, 2012, P T, V37, P175

Greer JM, 2011, J NEUROIMMUNOL, V234, P7, DOI 10.1016/j.jneuroim.2011.03.003

Gupta Yogendra K, 2009, Indian J Physiol Pharmacol, V53, P39

Nguyen HD, 2022, INT IMMUNOPHARMACOL, V108, DOI 10.1016/j.intimp.2022.108736

Hamaguchi T, 2010, CNS NEUROSCI THER, V16, P285, DOI 10.1111/j.1755-5949.2010.00147.x

Han Y, 2021, J CELL MOL MED, V25, P8947, DOI 10.1111/jcmm.16855

Hatefi M, 2018, WORLD NEUROSURG, V114, pE785, DOI 10.1016/j.wneu.2018.03.081

He HJ, 2022, NEUROCHEM INT, V155, DOI 10.1016/j.neuint.2022.105297

He W, 2020, ANN TRANSL MED, V8, DOI 10.21037/atm.2019.12.147

Hegde M, 2023, CANCER METAST REV, V42, P765, DOI 10.1007/s10555-022-10068-w

Hickey MA, 2012, MOL NEURODEGENER, V7, DOI 10.1186/1750-1326-7-12

Honarvar NM, 2021, ENDOCR METAB IMMUNE, V21, P365, DOI 10.2174/1871530320666200729144430

Hui-Yin Y, 2018, INDIAN J PHARM EDUC, V52, P32, DOI 10.5530/ijper.52.1.4

Jacob J, 2019, J TRADIT COMPL MED, V9, P346, DOI 10.1016/j.jtcme.2018.06.001

Jahan AA, 2018, LIFE SCI, V211, P252, DOI 10.1016/j.lfs.2018.09.007

Jain S, 2019, CURR TREAT OPTION NE, V21, DOI 10.1007/s11940-019-0555-4

Jaisin Y, 2011, NEUROSCI LETT, V489, P192, DOI 10.1016/j.neulet.2010.12.014

Jaskulska A, 2021, INT J MOL SCI, V22, DOI 10.3390/ijms22010004

Jiang Z, 2015, NEUROSCIENCE, V310, P362, DOI 10.1016/j.neuroscience.2015.09.058

Jithavech P, 2022, PLOS ONE, V17, DOI 10.1371/journal.pone.0265689

Johnson DR, 2013, CANCER-AM CANCER SOC, V119, P3489, DOI 10.1002/cncr.28259

Jyoti A, 2009, EPILEPSY BEHAV, V14, P300, DOI 10.1016/j.yebeh.2008.11.011

Kanakasabai S, 2012, J NUTR BIOCHEM, V23, P1498, DOI 10.1016/j.jnutbio.2011.10.002

Kaur H, 2015, NEUROCHEM INT, V89, P40, DOI 10.1016/j.neuint.2015.07.009

Kaur H, 2014, PHARMACOL BIOCHEM BE, V125, P55, DOI 10.1016/j.pbb.2014.08.001

Khadrawy Yasser A, 2018, J Complement Integr Med, V15, DOI 10.1515/jcim-2017-0117

Khatri DK, 2016, PHARMACOL BIOCHEM BE, V150, P39, DOI 10.1016/j.pbb.2016.09.002

Khatri DK, 2015, ORIENTAL PHARM EXP M, V15, P327, DOI 10.1007/s13596-015-0202-x

Khayatan D, 2022, BIOMED PHARMACOTHER, V154, DOI 10.1016/j.biopha.2022.113621

Khyati, 2021, CHRONOBIOL INT, V38, P61, DOI 10.1080/07420528.2020.1842752

Kiasalari Z, 2013, PHARM BIOL, V51, P1572, DOI 10.3109/13880209.2013.803128

Kim MS, 2011, CHEM-BIOL INTERACT, V194, P139, DOI 10.1016/j.cbi.2011.10.001

Kim MS, 2001, TOXICOL APPL PHARM, V177, P121, DOI 10.1006/taap.2001.9301

Kim SJ, 2008, J BIOL CHEM, V283, P14497, DOI 10.1074/jbc.M708373200

KISO Y, 1983, PLANTA MED, V49, P185, DOI 10.1055/s-2007-969845

Klinger NV, 2016, OXID MED CELL LONGEV, V2016, DOI 10.1155/2016/9324085

Kobatake Y, 2021, VET SCI, V8, DOI 10.3390/vetsci8090192

Kou JJ, 2021, ACS OMEGA, V6, P6654, DOI 10.1021/acsomega.0c04810

Kremer B., 2002, HUNTINGTONS DIS 3, V3rd, P28

Kulkarni SK, 2010, INDIAN J PHARM SCI, V72, P149, DOI 10.4103/0250-474X.65012

Kumar A, 2021, PHYTOTHER RES, V35, P6768, DOI 10.1002/ptr.7264

Kumar V, 2019, EPILEPSY RES, V150, P7, DOI 10.1016/j.eplepsyres.2018.12.007

Kunnumakkara AB, 2017, BRIT J PHARMACOL, V174, P1325, DOI 10.1111/bph.13621

Laird MD, 2010, J NEUROCHEM, V113, P637, DOI 10.1111/j.1471-4159.2010.06630.x

Lazaro DF, 2017, EXP NEUROL, V298, P162, DOI 10.1016/j.expneurol.2017.05.007

Lee WCM, 2004, P NATL ACAD SCI USA, V101, P3224, DOI 10.1073/pnas.0400243101

Lees JR, 2008, J IMMUNOL, V180, P8066, DOI 10.4049/jimmunol.180.12.8066

Li JN, 2021, AM J TRANSL RES, V13, P13380

Li X, 2022, FRONT AGING NEUROSCI, V14, DOI 10.3389/fnagi.2022.937486

Lin MT, 2006, NATURE, V443, P787, DOI 10.1038/nature05292

Lin YW, 2022, ANTIOXIDANTS-BASEL, V11, DOI 10.3390/antiox11040727

Lin YT, 2018, NEURON, V98, P1141, DOI 10.1016/j.neuron.2018.05.008

Lione LA, 1999, J NEUROSCI, V19, P10428

Lipton RB, 2019, NEUROLOGY, V92, pE2250, DOI 10.1212/WNL.0000000000007452

Lipton RB, 2015, NEUROLOGY, V84, P688, DOI 10.1212/WNL.0000000000001256

Liu JQ, 2019, EUR REV MED PHARMACO, V23, P9108, DOI 10.26355/eurrev_201910_19314

Liu M, 2021, NEUROCHEM RES, V46, P3166, DOI 10.1007/s11064-021-03414-x

Liu ZH, 2011, PHARMACOL RES, V63, P439, DOI 10.1016/j.phrs.2011.01.004

Lu JS, 2012, BRAIN RES BULL, V89, P185, DOI 10.1016/j.brainresbull.2012.09.005

Lu LS, 2020, BIOMATERIALS, V245, DOI 10.1016/j.biomaterials.2020.119987

Ma QL, 2009, J NEUROSCI, V29, P9078, DOI 10.1523/JNEUROSCI.1071-09.2009

MACDONALD ME, 1993, CELL, V72, P971, DOI 10.1016/0092-8674(93)90585-E

Maiti P, 2021, ANTIOXIDANTS-BASEL, V10, DOI 10.3390/antiox10101592

Maiti P, 2021, ALZHEIMERS RES THER, V13, DOI 10.1186/s13195-021-00769-9

Makino J, 2012, FREE RADICAL RES, V46, P637, DOI 10.3109/10715762.2012.664841

Mangiarini L, 1996, CELL, V87, P493, DOI 10.1016/S0092-8674(00)81369-0

Manna J, 2021, ANTIOXIDANTS-BASEL, V10, DOI 10.3390/antiox10060899

Mansouri Z, 2012, J MOL NEUROSCI, V47, P234, DOI 10.1007/s12031-012-9727-3

Marsh JL, 2004, BIOESSAYS, V26, P485, DOI 10.1002/bies.20029

Marsh JL, 2003, HUM MOL GENET, V12, pR187, DOI 10.1093/hmg/ddg271

Mavaddatiyan L, 2021, VET RES FORUM, V12, P47, DOI 10.30466/vrf.2019.98789.2356

Mehla J, 2010, LIFE SCI, V87, P596, DOI 10.1016/j.lfs.2010.09.006

Michinaga S, 2021, INT J MOL SCI, V22, DOI 10.3390/ijms22126418

Mobahat M, 2023, CELL TISSUE BANK, V24, P75, DOI 10.1007/s10561-022-10008-6

Mohajeri M, 2015, NEUROPHARMACOLOGY, V99, P156, DOI 10.1016/j.neuropharm.2015.07.013

Mohseni M, 2021, PHYTOTHER RES, V35, P6862, DOI 10.1002/ptr.7273

Monroy A, 2013, BIOFACTORS, V39, P122, DOI 10.1002/biof.1063

Montoya A, 2006, J PSYCHIATR NEUROSCI, V31, P21

Moors T, 2016, MOVEMENT DISORD, V31, P791, DOI 10.1002/mds.26562

Mostafa DK, 2016, CAN J PHYSIOL PHARM, V94, P699, DOI 10.1139/cjpp-2015-0316

Motavaf Mahsa, 2020, Iran J Pharm Res, V19, P310, DOI 10.22037/ijpr.2020.112952.14033

Motavaf M, 2020, J TISSUE ENG REGEN M, V14, P1449, DOI 10.1002/term.3110

Motawi TK, 2020, MOL CELL BIOCHEM, V465, P89, DOI 10.1007/s11010-019-03670-0

Narouiepour A, 2022, SCI REP-UK, V12, DOI 10.1038/s41598-022-07367-1

Nascimento CP, 2022, FRONT CELL NEUROSCI, V16, DOI 10.3389/fncel.2022.884813

Natarajan C, 2002, J IMMUNOL, V168, P6506, DOI 10.4049/jimmunol.168.12.6506

Ngolab J, 2021, ALZH DEMENT-DADM, V13, DOI 10.1002/dad2.12199

Nguyen HD, 2023, TOXICOL ENV HEALTH, V15, P181, DOI 10.1007/s13530-023-00171-y

Nguyen HD, 2023, INT IMMUNOPHARMACOL, V115, DOI 10.1016/j.intimp.2023.109726

Nguyen HD, 2022, NEUROTOX RES, V40, P1272, DOI 10.1007/s12640-022-00535-1

Nguyen HD, 2022, INT IMMUNOPHARMACOL, V108, DOI 10.1016/j.intimp.2022.108901

Noor NA, 2022, METAB BRAIN DIS, V37, P343, DOI 10.1007/s11011-021-00897-z

Noseworthy JH, 2000, NEW ENGL J MED, V343, P938, DOI 10.1056/NEJM200009283431307

Ortiz-Ortiz MA, 2010, NEUROSCI LETT, V468, P120, DOI 10.1016/j.neulet.2009.10.081

Ostrom QT, 2015, NEURO-ONCOLOGY, V16, P1, DOI [10.1093/neuonc/nox158, 10.1093/neuonc/nou327, 10.1093/neuonc/nou223, 10.1093/neuonc/nov189]

Ouyang JP, 2019, PAIN RES MANAG, V2019, DOI 10.1155/2019/3173149

Pandareesh MD, 2016, NEUROCHEM RES, V41, P3113, DOI 10.1007/s11064-016-2034-6

Pandey N, 2008, ACTA NEUROPATHOL, V115, P479, DOI 10.1007/s00401-007-0332-4

Parohan M, 2021, NUTR NEUROSCI, V24, P317, DOI 10.1080/1028415X.2019.1627770

Peng H, 2022, ACS NANO, V16, P869, DOI 10.1021/acsnano.1c08473

Pratheeshkumar P, 2012, ANTI-CANCER AGENT ME, V12, P1159, DOI 10.2174/187152012803833035

Przedborski S, 2001, CLIN NEUROSCI RES, V1, P407, DOI 10.1016/S1566-2772(01)00019-6

Rajeswari A, 2006, Eur Rev Med Pharmacol Sci, V10, P157

Rajeswari A., 2008, Inflammopharmacology, V16, P96, DOI 10.1007/s10787-007-1614-0

Rakotoarisoa Miora, 2018, Medicines (Basel), V5, DOI 10.3390/medicines5040126

Ramires OV, 2021, NEUROTOX RES, V39, P787, DOI 10.1007/s12640-021-00362-w

Reeta KH, 2011, PHARMACOL BIOCHEM BE, V99, P399, DOI 10.1016/j.pbb.2011.05.011

Rezaie S, 2021, INT J PREVENTIVE MED, V12, DOI 10.4103/ijpvm.IJPVM_405_20

Ringman JM, 2012, ALZHEIMERS RES THER, V4, DOI 10.1186/alzrt146

Ruan YT, 2022, J NANOBIOTECHNOL, V20, DOI 10.1186/s12951-022-01524-4

SAGARA Y, 1992, J BIOL CHEM, V267, P1286

Saha L, 2016, INDIAN J EXP BIOL, V54, P133

Samini F, 2013, PHARMACOL BIOCHEM BE, V110, P238, DOI 10.1016/j.pbb.2013.07.019

Sandhir R, 2014, NEUROMOL MED, V16, P106, DOI 10.1007/s12017-013-8261-y

Sawada H, 2002, NEUROPHARMACOLOGY, V42, P1056, DOI 10.1016/S0028-3908(02)00049-7

Sedighiyan M, 2022, BMC RES NOTES, V15, DOI 10.1186/s13104-022-06074-4

Sehgal P, 2017, J BIOL CHEM, V292, P19656, DOI 10.1074/jbc.M117.796920

Shabnam B, 2020, The Chemistry and Bioactive Components of Turmeric, P150, DOI [10.1039/9781788015936-00150, DOI 10.1039/9781788015936-00150]

Shadnoush M, 2020, PHYTOTHER RES, V34, P3180, DOI 10.1002/ptr.6749

Sharifi-Rad M, 2020, J CLIN MED, V9, DOI 10.3390/jcm9041061

Sharma S, 2009, NEUROSCIENCE, V161, P1037, DOI 10.1016/j.neuroscience.2009.04.042

Shin HJ, 2007, NEUROSCI LETT, V416, P49, DOI 10.1016/j.neulet.2007.01.060

Singla RK, 2023, PHYTOMEDICINE, V108, DOI 10.1016/j.phymed.2022.154520

Small GW, 2018, AM J GERIAT PSYCHIAT, V26, P266, DOI 10.1016/j.jagp.2017.10.010

Song HC, 2020, FREE RADICAL RES, V54, P918, DOI 10.1080/10715762.2020.1791843

Song SL, 2016, PATHOL RES PRACT, V212, P247, DOI 10.1016/j.prp.2015.11.012

Soveyd N, 2017, CNS NEUROL DISORD-DR, V16, P1120, DOI 10.2174/1871527317666171213154749

Srinivasan M, 1972, Indian J Med Sci, V26, P269

Stupp R, 2005, NEW ENGL J MED, V352, P987, DOI 10.1056/NEJMoa043330

Stupp R, 2009, LANCET ONCOL, V10, P459, DOI 10.1016/S1470-2045(09)70025-7

Sun MJ, 2022, NEUROSCI LETT, V787, DOI 10.1016/j.neulet.2022.136821

Sun SC, 2017, NAT REV IMMUNOL, V17, P545, DOI 10.1038/nri.2017.52

Tabrizi SJ, 2022, LANCET NEUROL, V21, P645, DOI 10.1016/S1474-4422(22)00121-1

Tabrizi SJ, 2019, NEURON, V102, P899, DOI 10.1016/j.neuron.2019.05.001

Tadini-Buoninsegni F, 2018, FRONT MOL BIOSCI, V5, DOI 10.3389/fmolb.2018.00036

Tang KS, 2019, LIFE SCI, V233, DOI 10.1016/j.lfs.2019.116695

Tewari D, 2022, SEMIN CANCER BIOL, V80, P1, DOI 10.1016/j.semcancer.2019.12.008

Thapa K, 2021, J MOL NEUROSCI, V71, P1725, DOI 10.1007/s12031-021-01841-7

Theendakara V, 2018, MOL NEUROBIOL, V55, P5243, DOI 10.1007/s12035-017-0757-2

Thijs RD, 2019, LANCET, V393, P689, DOI 10.1016/S0140-6736(18)32596-0

Tian MY, 2021, J INTEGR NEUROSCI, V20, P895, DOI 10.31083/j.jin2004091

Tripanichkul W, 2013, EUR REV MED PHARMACO, V17, P1360

Tripanichkul W, 2012, INT J NEUROSCI, V122, P263, DOI 10.3109/00207454.2011.648760

Uddin MS, 2019, MOL NEUROBIOL, V56, P2450, DOI 10.1007/s12035-018-1237-z

Utomo RY, 2022, CHEM COMMUN, V58, P2576, DOI 10.1039/d1cc07000b

Vajragupta O, 2003, FREE RADICAL BIO MED, V35, P1632, DOI 10.1016/j.freeradbiomed.2003.09.011

Vasconcelos AR, 2019, FRONT PHARMACOL, V10, DOI 10.3389/fphar.2019.00033

Venkatesan N, 1998, BRIT J PHARMACOL, V124, P425, DOI 10.1038/sj.bjp.0701877

Venkatesan N, 2000, BRIT J PHARMACOL, V129, P231, DOI 10.1038/sj.bjp.0703067

Vos T, 2012, LANCET, V380, P2163, DOI 10.1016/S0140-6736(12)61729-2

Vos T, 2015, LANCET, V386, P743, DOI 10.1016/S0140-6736(15)60692-4

Wang J, 2017, CAN J PHYSIOL PHARM, V95, P501, DOI 10.1139/cjpp-2016-0154

Wang J, 2009, BIOCHEM PHARMACOL, V78, P178, DOI 10.1016/j.bcp.2009.03.031

Wang KR, 2021, SYNAPSE, V75, DOI 10.1002/syn.22196

Wang MS, 2010, BMC NEUROSCI, V11, DOI 10.1186/1471-2202-11-57

Wang YL, 2020, STEM CELLS INT, V2020, DOI 10.1155/2020/4636397

Wang YL, 2017, CELL PHYSIOL BIOCHEM, V43, P2226, DOI 10.1159/000484302

Weathers Shiao-Pei, 2014, F1000Prime Rep, V6, P46, DOI 10.12703/P6-46

Wei G, 2017, NEUROIMMUNOMODULAT, V24, P348, DOI 10.1159/000487998

Wong Michael, 2013, Biomed J, V36, P40, DOI 10.4103/2319-4170.110365

Wu AG, 2006, EXP NEUROL, V197, P309, DOI 10.1016/j.expneurol.2005.09.004

Wu AG, 2011, NEUROREHAB NEURAL RE, V25, P332, DOI 10.1177/1545968310397706

Xiao Y, 2021, BIOMED PHARMACOTHER, V139, DOI 10.1016/j.biopha.2021.111651

Xie L, 2011, INT IMMUNOPHARMACOL, V11, P323, DOI 10.1016/j.intimp.2010.08.013

Xie L, 2009, INT IMMUNOPHARMACOL, V9, P575, DOI 10.1016/j.intimp.2009.01.025

Xie ZC, 2006, EXP GERONTOL, V41, P346, DOI 10.1016/j.exger.2006.01.014

Xu CL, 2020, TRANSL NEUROSCI, V11, P319, DOI 10.1515/tnsci-2020-0140

Xu Y, 2020, BRAIN BEHAV, V10, DOI 10.1002/brb3.1655

Yallapu MM, 2015, AAPS J, V17, P1341, DOI 10.1208/s12248-015-9811-z

Yavarpour-Bali H, 2019, INT J NANOMED, V14, P4449, DOI 10.2147/IJN.S208332

Yenari MA, 2012, NAT REV NEUROSCI, V13, P267, DOI 10.1038/nrn3174

Yoon Woo Bin, 2018, Lab Anim Res, V34, P317

Yu S, 2010, REJUV RES, V13, P55, DOI 10.1089/rej.2009.0908

Yuan BY, 2019, J NEUROIMMUNOL, V337, DOI 10.1016/j.jneuroim.2019.577067

Zbarsky V, 2005, FREE RADICAL RES, V39, P1119, DOI 10.1080/10715760500233113

Zhang HQ, 2022, NANO LETT, V22, P2450, DOI 10.1021/acs.nanolett.2c00191

Zhou HY, 2022, NEUROTOX RES, V40, P449, DOI 10.1007/s12640-022-00481-y

Zhu HT, 2014, J NEUROINFLAMM, V11, DOI 10.1186/1742-2094-11-59

Zhu WT, 2015, BIO-MED MATER ENG, V26, pS841, DOI 10.3233/BME-151376

Gutierrez MEZ, 2021, NEURAL REGEN RES, V16, P783, DOI 10.4103/1673-5374.295339

Zielinska A, 2020, MEDICINA-LITHUANIA, V56, DOI 10.3390/medicina56070336

Zou ZY, 2017, J NEUROL NEUROSUR PS, V88, P540, DOI 10.1136/jnnp-2016-315018

Zuccato C, 2010, PHYSIOL REV, V90, P905, DOI 10.1152/physrev.00041.2009

Zussy C, 2022, ANTIOXIDANTS-BASEL, V11, DOI 10.3390/antiox11050838

NR 271

TC 7

Z9 8

U1 5

U2 5

PU ELSEVIER

PI AMSTERDAM

PA RADARWEG 29, 1043 NX AMSTERDAM, NETHERLANDS

SN 2213-4220

EI 2213-4239

J9 INTEGR MED RES

JI Integr. Med. Res.

PD SEP

PY 2023

VL 12

IS 3

AR 100968

DI 10.1016/j.imr.2023.100968

EA AUG 2023

PG 25

WC Integrative & Complementary Medicine

WE Science Citation Index Expanded (SCI-EXPANDED)

SC Integrative & Complementary Medicine

GA R5HK6

UT WOS:001064657400001

PM 37664456

OA Green Published, gold

DA 2024-08-12

ER

PT J

AU Espinosa-Oliva, AM

García-Miranda, P

Alonso-Bellido, IM

Carvajal, AE

González-Rodríguez, M

Carrillo-Jiménez, A

Temblador, AJ

Felices-Navarro, M

García-Domínguez, I

Roca-Ceballos, MA

Vázquez-Carretero, MD

García-Revilla, J

Santiago, M

Peral, MJ

Venero, JL

de Pablos, RM

AF Espinosa-Oliva, Ana M.

Garcia-Miranda, Pablo

Maria Alonso-Bellido, Isabel

Carvajal, Ana E.

Gonzalez-Rodriguez, Melania

Carrillo-Jimenez, Alejandro

Temblador, Arturo J.

Felices-Navarro, Manuel

Garcia-Dominguez, Irene

Angustias Roca-Ceballos, Maria

Vazquez-Carretero, Maria D.

Garcia-Revilla, Juan

Santiago, Marti

Peral, Maria J.

Luis Venero, Jose

de Pablos, Rocio M.

TI Galectin-3 Deletion Reduces LPS and Acute Colitis-Induced

Pro-Inflammatory Microglial Activation in the Ventral Mesencephalon

SO FRONTIERS IN PHARMACOLOGY

LA English

DT Article

DE galectin-3; microglia; Parkinson's disease; peripheral inflammation;

neuroinflammation

ID parkinson's disease; PERIPHERAL INFLAMMATION; GENE POLYMORPHISMS; GUT

MICROBIOTA; NEUROINFLAMMATION; ASSOCIATION; EXPRESSION; COLON;

PROLIFERATION; IL-1-BETA

AB Parkinson's disease is a highly prevalent neurological disorder for which there is currently no cure. Therefore, the knowledge of risk factors as well as the development of new putative molecular targets is mandatory. In this sense, peripheral inflammation, especially the originated in the colon, is emerging as a predisposing factor for suffering this disease. We have largely studied the pleiotropic roles of galectin-3 in driving microglia-associated immune responses. However, studies aimed at elucidating the role of galectin-3 in peripheral inflammation in terms of microglia polarization are lacking. To achieve this, we have evaluated the effect of galectin-3 deletion in two different models of acute peripheral inflammation: intraperitoneal injection of lipopolysaccharide or gut inflammation induced by oral administration of dextran sodium sulfate. We found that under peripheral inflammation the number of microglial cells and the expression levels of pro-inflammatory mediators take place specifically in the dopaminergic system, thus supporting causative links between Parkinson's disease and peripheral inflammation. Absence of galectin-3 highly reduced neuroinflammation in both models, suggesting an important central regulatory role of galectin-3 in driving microglial activation provoked by the peripheral inflammation. Thus, modulation of galectin-3 function emerges as a promising strategy to minimize undesired microglia polarization states.

C1 [Espinosa-Oliva, Ana M.; Maria Alonso-Bellido, Isabel; Carrillo-Jimenez, Alejandro; Felices-Navarro, Manuel; Garcia-Dominguez, Irene; Angustias Roca-Ceballos, Maria; Garcia-Revilla, Juan; Santiago, Marti; Luis Venero, Jose; de Pablos, Rocio M.] Univ Seville, Hosp Univ Virgen Rocio CSIC, Fac Farm, Inst Biomed Sevilla IBiS,Dept Bioquirn & Biol Mol, Seville, Spain.

[Garcia-Miranda, Pablo; Carvajal, Ana E.; Vazquez-Carretero, Maria D.; Peral, Maria J.] Univ Seville, Fac Farm, Dept Fisiol, Seville, Spain.

[Gonzalez-Rodriguez, Melania] Univ Castilla La Mancha, Ciudad Real Med Sch, Neuroplasti & Neurodegenerat Lab, CRIB, Ciudad Real, Spain.

[Temblador, Arturo J.] Rega Inst Med Res, Dept Microbiol Immunol & Transplantat, Lab Virol & Chemotherapy, Leuven, Belgium.

C3 Consejo Superior de Investigaciones Cientificas (CSIC); University of

Sevilla; CSIC-JA-USE - Instituto de Biomedicina de Sevilla (IBIS);

University of Sevilla; Universidad de Castilla-La Mancha; KU Leuven

RP de Pablos, RM (corresponding author), Univ Seville, Hosp Univ Virgen Rocio CSIC, Fac Farm, Inst Biomed Sevilla IBiS,Dept Bioquirn & Biol Mol, Seville, Spain.

EM depablos@us.es

RI Vazquez, Maria Dolores/K-8624-2014; Venero, Jose L/E-9979-2015;

Garcia-Miranda, Pablo/L-5213-2014; Gonzalez-Rodriguez,

Melania/M-7126-2016; Temblador Perez, Arturo Jesus/F-6461-2018; Peral,

Maria Jose/L-8573-2014; Garcia-Dominguez, Irene/J-8352-2018

OI Garcia-Miranda, Pablo/0000-0003-2588-6904; Gonzalez-Rodriguez,

Melania/0000-0002-5351-3008; Alonso Bellido, Isabel

Maria/0000-0002-7475-8653; Temblador Perez, Arturo

Jesus/0000-0003-3076-6317; Peral, Maria Jose/0000-0003-0343-7550;

Garcia-Dominguez, Irene/0000-0001-6668-1599

CR Alrafiah A, 2019, BIOMED RES INT, V2019, DOI 10.1155/2019/2690205

Araki Y, 2010, ONCOL REP, V24, P869, DOI 10.3892/or_00000932

Beier EE, 2017, NEUROBIOL DIS, V108, P115, DOI 10.1016/j.nbd.2017.08.009

Boza-Serrano A, 2019, ACTA NEUROPATHOL, V138, P251, DOI 10.1007/s00401-019-02013-z

Boza-Serrano A, 2014, ACTA NEUROPATHOL COM, V2, DOI 10.1186/s40478-014-0156-0

Braak H, 2003, NEUROBIOL AGING, V24, P197, DOI 10.1016/S0197-4580(02)00065-9

Braak H., 2002, Journal of neurology, V249, pIII/1, DOI [DOI 10.1007/S00415-002-1301-4, 10.1007/s00415-002-1301-4]

Braak H, 2007, ACTA NEUROPATHOL, V113, P421, DOI 10.1007/s00401-007-0193-x

Brudek T, 2019, J PARKINSON DIS, V9, pS331, DOI 10.3233/JPD-191729

Burguillos MA, 2015, CELL REP, V10, P1626, DOI 10.1016/j.celrep.2015.02.012

Buscarinu MC, 2017, MULT SCLER J, V23, P442, DOI 10.1177/1352458516652498

Butovsky O, 2014, NAT NEUROSCI, V17, P131, DOI 10.1038/nn.3599

Hernández-Romero MC, 2012, NEUROTOXICOLOGY, V33, P347, DOI 10.1016/j.neuro.2012.01.018

Carvajal AE, 2017, BBA-MOL BASIS DIS, V1863, P2126, DOI 10.1016/j.bbadis.2017.05.026

Carvajal AE, 2017, BBA-MOL BASIS DIS, V1863, P462, DOI 10.1016/j.bbadis.2016.11.028

Cengiz T, 2019, CLIN NEUROL NEUROSUR, V184, DOI 10.1016/j.clineuro.2019.105373

Clark IA, 2021, J ALZHEIMERS DIS, V79, P931, DOI 10.3233/JAD-201186

Colnot C, 1998, DEV DYNAM, V211, P306, DOI 10.1002/(SICI)1097-0177(199804)211:4<306::AID-AJA2>3.0.CO;2-L

COOPER HS, 1993, LAB INVEST, V69, P238

do Nascimento RD, 2020, LIFE SCI, V258, DOI 10.1016/j.lfs.2020.118129

deRijk MC, 1997, NEUROLOGY, V48, P1277, DOI 10.1212/WNL.48.5.1277

Díaz-Alvarez L, 2017, MEDIAT INFLAMM, V2017, DOI 10.1155/2017/9247574

Donaldson DS, 2016, J GEN VIROL, V97, P1725, DOI 10.1099/jgv.0.000507

Flavin WP, 2017, ACTA NEUROPATHOL, V134, P629, DOI 10.1007/s00401-017-1722-x

Friedman BA, 2018, CELL REP, V22, P832, DOI 10.1016/j.celrep.2017.12.066

Galatro TF, 2017, NAT NEUROSCI, V20, P1162, DOI 10.1038/nn.4597

García-Domínguez I, 2018, FRONT CELL NEUROSCI, V12, DOI 10.3389/fncel.2018.00398

García-Revilla J, 2019, J CLIN MED, V8, DOI 10.3390/jcm8101719

Garrido-Gil P, 2018, MOL NEUROBIOL, V55, P7297, DOI 10.1007/s12035-018-0937-8

Grabert K, 2016, NAT NEUROSCI, V19, P504, DOI 10.1038/nn.4222

Gundersen V, 2021, FRONT NEUROSCI-SWITZ, V14, DOI 10.3389/fnins.2020.580311

Heneka MT, 2015, LANCET NEUROL, V14, P388, DOI 10.1016/S1474-4422(15)70016-5

Herrera AJ, 2015, CURR TOP MED CHEM, V15, P2193, DOI 10.2174/1568026615666150610142027

Hickman SE, 2013, NAT NEUROSCI, V16, P1896, DOI 10.1038/nn.3554

Hirsch EC, 2009, LANCET NEUROL, V8, P382, DOI 10.1016/S1474-4422(09)70062-6

Jeon SB, 2010, J IMMUNOL, V185, P7037, DOI 10.4049/jimmunol.1000154

Jiang K, 2014, J BIOL CHEM, V289, P10510, DOI 10.1074/jbc.M113.538538

Kaushal Swati, 2021, CNS Neurol Disord Drug Targets, DOI 10.2174/1871527320666210310141015

Keren-Shaul H, 2017, CELL, V169, P1276, DOI 10.1016/j.cell.2017.05.018

Kim Young S, 2010, Curr Gastroenterol Rep, V12, P319, DOI 10.1007/s11894-010-0131-2

Kordower JH, 2013, BRAIN, V136, P2419, DOI 10.1093/brain/awt192

Krasemann S, 2017, IMMUNITY, V47, P566, DOI 10.1016/j.immuni.2017.08.008

Kumaresan M, 2021, CUREUS J MED SCIENCE, V13, DOI 10.7759/cureus.13275

Lalancette-Hébert M, 2012, J NEUROSCI, V32, P10383, DOI 10.1523/JNEUROSCI.1498-12.2012

Li N, 2021, EUR REV MED PHARMACO, V25, P890, DOI 10.26355/eurrev_202101_24657

Li Y, 2008, J IMMUNOL, V181, P2781, DOI 10.4049/jimmunol.181.4.2781

Lin JC, 2016, INFLAMM BOWEL DIS, V22, P1049, DOI 10.1097/MIB.0000000000000735

Lippert E, 2015, DIGESTION, V92, P45, DOI 10.1159/000431312

Markovic BS, 2016, J CROHNS COLITIS, V10, P593, DOI 10.1093/ecco-jcc/jjw013

Mathys H, 2017, CELL REP, V21, P366, DOI 10.1016/j.celrep.2017.09.039

Nalls MA, 2014, NAT GENET, V46, P989, DOI 10.1038/ng.3043

Peter I, 2018, JAMA NEUROL, V75, P939, DOI 10.1001/jamaneurol.2018.0605

Pröbstel AK, 2020, SCI IMMUNOL, V5, DOI 10.1126/sciimmunol.abc7191

Qin LY, 2007, GLIA, V55, P453, DOI 10.1002/glia.20467

Qin XY, 2016, JAMA NEUROL, V73, P1316, DOI 10.1001/jamaneurol.2016.2742

Rayaprolu S, 2013, MOL NEURODEGENER, V8, DOI 10.1186/1750-1326-8-19

Rooks MG, 2016, NAT REV IMMUNOL, V16, P341, DOI 10.1038/nri.2016.42

Satoh K, 2011, BRAIN RES, V1382, P266, DOI 10.1016/j.brainres.2011.01.049

Sciacchitano S, 2018, INT J MOL SCI, V19, DOI 10.3390/ijms19020379

Shimura T, 2004, CANCER RES, V64, P6363, DOI 10.1158/0008-5472.CAN-04-1816

Song SM, 2005, GASTROENTEROLOGY, V129, P1581, DOI 10.1053/j.gastro.2005.09.002

Tsai HF, 2016, J MOL MED, V94, P545, DOI 10.1007/s00109-015-1368-x

Viguier M, 2014, TISSUE BARRIERS, V2, DOI 10.4161/tisb.29103

Villarán RF, 2010, J NEUROCHEM, V114, P1687, DOI 10.1111/j.1471-4159.2010.06879.x

Villumsen M, 2019, GUT, V68, P18, DOI 10.1136/gutjnl-2017-315666

Volarevic V, 2019, CELLS-BASEL, V8, DOI 10.3390/cells8070709

Wan QY, 2020, GUT, V69, P193, DOI 10.1136/gutjnl-2018-317103

Weimers P, 2019, INFLAMM BOWEL DIS, V25, P111, DOI 10.1093/ibd/izy190

Wesley UV, 2013, BRAIN RES, V1496, P1, DOI 10.1016/j.brainres.2012.12.008

Yazar HO, 2019, J CLIN NEUROSCI, V70, P164, DOI 10.1016/j.jocn.2019.08.032

Yip PK, 2017, SCI REP-UK, V7, DOI 10.1038/srep41689

Yousefi A, 2018, ANN HEPATOL, V17, P1021, DOI 10.5604/01.3001.0012.7202

Zhang L, 2018, CELL PHYSIOL BIOCHEM, V51, P763, DOI 10.1159/000495331

Zhu F, 2019, DIGEST LIVER DIS, V51, P38, DOI 10.1016/j.dld.2018.09.017

NR 74

TC 8

Z9 9

U1 1

U2 6

PU FRONTIERS MEDIA SA

PI LAUSANNE

PA AVENUE DU TRIBUNAL FEDERAL 34, LAUSANNE, CH-1015, SWITZERLAND

EI 1663-9812

J9 FRONT PHARMACOL

JI Front. Pharmacol.

PD AUG 18

PY 2021

VL 12

DI 10.3389/fphar.2021.706439

PG 20

WC Pharmacology & Pharmacy

WE Science Citation Index Expanded (SCI-EXPANDED)

SC Pharmacology & Pharmacy

GA UM8FN

UT WOS:000693561900001

PM 34483912

OA gold, Green Published

DA 2024-08-12

ER

PT J

AU Hashioka, S

Inoue, K

Miyaoka, T

Hayashida, M

Wake, R

Oh-Nishi, A

Inagaki, M

AF Hashioka, Sadayuki

Inoue, Ken

Miyaoka, Tsuyoshi

Hayashida, Maiko

Wake, Rei

Oh-Nishi, Arata

Inagaki, Masatoshi

TI The Possible Causal Link of Periodontitis to Neuropsychiatric Disorders:

More Than Psychosocial Mechanisms

SO INTERNATIONAL JOURNAL OF MOLECULAR SCIENCES

LA English

DT Review

DE periodontitis; neuropsychiatric disorders; Alzheimer's disease;

Parkinson's disease; major depression; schizophrenia; neuroinflammation;

microglia

ID NUTRITION EXAMINATION SURVEY; alzheimer's disease; RISK-FACTOR; COGNITIVE

IMPAIRMENT; NATIONAL-HEALTH; ORAL INFECTIONS; ISCHEMIC-STROKE;

ASSOCIATION; BRAIN; DEPRESSION

AB Increasing evidence implies a possible causal link between periodontitis and neuropsychiatric disorders, such as Alzheimer's disease (AD) and major depression (MD). A possible mechanism underlying such a link can be explained by neuroinflammation induced by chronic systemic inflammation. This review article focuses on an overview of the biological and epidemiological evidence for a feasible causal link of periodontitis to neuropsychiatric disorders, including AD, MD, Parkinson's disease, and schizophrenia, as well as the neurological event, ischemic stroke. If there is such a link, a broad spectrum of neuropsychiatric disorders associated with neuroinflammation could be preventable and modifiable by simple daily dealings for oral hygiene. However, the notion that periodontitis is a risk factor for neuropsychiatric disorders remains to be effectively substantiated.

C1 [Hashioka, Sadayuki; Miyaoka, Tsuyoshi; Hayashida, Maiko; Wake, Rei; Oh-Nishi, Arata; Inagaki, Masatoshi] Shimane Univ, Dept Psychiat, 89-1 Enya Cho, Izumo, Shimane 6938501, Japan.

[Inoue, Ken] Kochi Univ, Hlth Serv Ctr, 2-5-1 Akebono Cho, Kochi 7808520, Japan.

C3 Shimane University; Kochi University

RP Hashioka, S (corresponding author), Shimane Univ, Dept Psychiat, 89-1 Enya Cho, Izumo, Shimane 6938501, Japan.

EM hashioka@f2.dion.ne.jp

FU JSPS KAKENHI [19K08018, 17K09194, 19K08046]; Grants-in-Aid for

Scientific Research [19K08046, 19K08018, 17K09194] Funding Source: KAKEN

FX \This study was supported by JSPS KAKENHI Grant Numbers 19K08018 (SH),

17K09194 (KI), 19K08046 (MH).

CR Bale Sue, 2004, Br J Nurs, V13, pS4

Beck J, 1996, J PERIODONTOL, V67, P1123, DOI 10.1902/jop.1996.67.10s.1123

Bhatia Anu, 2018, J Indian Soc Periodontol, V22, P442, DOI 10.4103/jisp.jisp_219_18

Bhatia A, 2015, J PERIODONTOL, V86, P927, DOI 10.1902/jop.2015.140706

BLUTHE RM, 1994, CR ACAD SCI III-VIE, V317, P499

Breivik T, 2015, OPEN DENT J, V9, P65, DOI 10.2174/1874210601509010065

Cademartori MG, 2018, CLIN ORAL INVEST, V22, P2685, DOI 10.1007/s00784-018-2611-y

Capuron L, 2011, PHARMACOL THERAPEUT, V130, P226, DOI 10.1016/j.pharmthera.2011.01.014

Chen CK, 2018, INT J ENV RES PUB HE, V15, DOI 10.3390/ijerph15081587

Chen CK, 2017, PEERJ, V5, DOI 10.7717/peerj.3647

Chen CK, 2017, ALZHEIMERS RES THER, V9, DOI 10.1186/s13195-017-0282-6

Chi L, 2019, NEUROREPORT, V30, P428, DOI 10.1097/WNR.0000000000001220

Choi S, 2019, J AM GERIATR SOC, V67, P1234, DOI 10.1111/jgs.15828

Crescenti A, 2009, PSYCHIAT RES, V165, P175, DOI 10.1016/j.psychres.2008.04.024

D'Mello C, 2017, CURR TOP BEHAV NEURO, V31, P73, DOI 10.1007/7854_2016_37

D'Mello C, 2009, J NEUROSCI, V29, P2089, DOI 10.1523/JNEUROSCI.3567-08.2009

daSilva AMM, 1996, J CLIN PERIODONTOL, V23, P789

Saintrain MVD, 2012, GERODONTOLOGY, V29, pE632, DOI 10.1111/j.1741-2358.2011.00535.x

Solis ACD, 2016, BRAIN BEHAV IMMUN, V53, P49, DOI 10.1016/j.bbi.2015.11.014

Delgado-Angulo EK, 2015, COMMUNITY DENT ORAL, V43, P540, DOI 10.1111/cdoe.12179

Ding Y, 2018, IMMUN AGEING, V15, DOI 10.1186/s12979-017-0110-7

Dominy SS, 2019, SCI ADV, V5, DOI 10.1126/sciadv.aau3333

Doorduin J, 2009, J NUCL MED, V50, P1801, DOI 10.2967/jnumed.109.066647

Dörfer CE, 2004, J CLIN PERIODONTOL, V31, P396, DOI 10.1111/j.1600-051X.2004.00579.x

Dumitrescu AL, 2016, FRONT PSYCHOL, V7, DOI 10.3389/fpsyg.2016.00347

Einarsdóttir ER, 2009, SPEC CARE DENT, V29, P123, DOI 10.1111/j.1754-4505.2009.00075.x

Eke PI, 2012, J DENT RES, V91, P914, DOI 10.1177/0022034512457373

Eltas A, 2013, INT J DENT HYG, V11, P78, DOI 10.1111/j.1601-5037.2012.00558.x

Cestari JAF, 2016, J ALZHEIMERS DIS, V52, P1479, DOI 10.3233/JAD-160212

Fillman SG, 2013, MOL PSYCHIATR, V18, P206, DOI 10.1038/mp.2012.110

Frister A, 2014, NEUROMOL MED, V16, P704, DOI 10.1007/s12017-014-8320-z

Gil-Montoya JA, 2015, J PERIODONTOL, V86, P244, DOI 10.1902/jop.2014.140340

Gomes C, 2018, MOL NEUROBIOL, V55, P2814, DOI 10.1007/s12035-017-0545-z

Grau AJ, 2004, STROKE, V35, P496, DOI 10.1161/01.STR.0000110789.20526.9D

Gurav AN, 2014, REV ASSOC MED BRAS, V60, P173, DOI 10.1590/1806-9282.60.02.015

Gürkan A, 2009, J CLIN PERIODONTOL, V36, P204, DOI 10.1111/j.1600-051X.2008.01379.x

Hashioka S, 2011, MINI-REV MED CHEM, V11, P555, DOI 10.2174/138955711795906888

Hashioka S, 2018, FRONT NEUROSCI-SWITZ, V12, DOI 10.3389/fnins.2018.00483

Hashioka Sadayuki, 2009, Central Nervous System Agents in Medicinal Chemistry, V9, P12

Hirotomi T, 2014, J CLIN PERIODONTOL, V41, P633, DOI 10.1111/jcpe.12267

Holmer J, 2018, J CLIN PERIODONTOL, V45, P1287, DOI 10.1111/jcpe.13016

Holmstrup P, 2017, J ORAL MICROBIOL, V9, DOI 10.1080/20002297.2017.1332710

Hsu CC, 2015, MEDICINE, V94, DOI 10.1097/MD.0000000000002347

Ide M, 2016, PLOS ONE, V11, DOI 10.1371/journal.pone.0151081

Ilievski V, 2018, PLOS ONE, V13, DOI 10.1371/journal.pone.0204941

Ishida N, 2017, NPJ AGING MECH DIS, V3, DOI 10.1038/s41514-017-0015-x

Jimenez M, 2009, ANN NEUROL, V66, P505, DOI 10.1002/ana.21742

Kamer AR, 2008, ALZHEIMERS DEMENT, V4, P242, DOI 10.1016/j.jalz.2007.08.004

Kamer AR, 2009, J NEUROIMMUNOL, V216, P92, DOI 10.1016/j.jneuroim.2009.08.013

Khambaty T, 2013, ANN BEHAV MED, V45, P393, DOI 10.1007/s12160-013-9471-0

Lafon A, 2014, ACTA ODONTOL SCAND, V72, P687, DOI 10.3109/00016357.2014.898089

Laforgia A, 2015, INT J MED SCI, V12, P832, DOI 10.7150/ijms.12317

Leira Y, 2017, EUR J EPIDEMIOL, V32, P43, DOI 10.1007/s10654-016-0170-6

Leira Y, 2017, NEUROEPIDEMIOLOGY, V48, P21, DOI 10.1159/000458411

LIU YC, 2013, MEDIAT INFLAMM, V2013, DOI DOI 10.1155/2013/407562

Liu Y, 2017, SCI REP-UK, V7, DOI 10.1038/s41598-017-07465-5

Maes M, 2011, PROG NEURO-PSYCHOPH, V35, P676, DOI 10.1016/j.pnpbp.2010.05.004

McGeer PL, 2011, FRONT PHARMACOL, V2, DOI 10.3389/fphar.2011.00077

MCGEER PL, 1987, NEUROSCI LETT, V79, P195, DOI 10.1016/0304-3940(87)90696-3

MCGEER PL, 1990, LANCET, V335, P1037, DOI 10.1016/0140-6736(90)91101-F

Moss ME, 1996, J PERIODONTOL, V67, P1060, DOI 10.1902/jop.1996.67.10s.1060

Muniz FWMG, 2018, J INVESTIG CLIN DENT, V9, DOI 10.1111/jicd.12291

Noble JM, 2014, PLOS ONE, V9, DOI 10.1371/journal.pone.0114959

Olsen I, 2015, J ORAL MICROBIOL, V7, DOI 10.3402/jom.v7.29143

Park SJ, 2014, J PUBLIC HEALTH DENT, V74, P127, DOI 10.1111/jphd.12036

Perry VH, 2004, BRAIN BEHAV IMMUN, V18, P407, DOI 10.1016/j.bbi.2004.01.004

Persson GR, 2003, J CLIN PERIODONTOL, V30, P691, DOI 10.1034/j.1600-051X.2003.00360.x

Poole S, 2013, J ALZHEIMERS DIS, V36, P665, DOI 10.3233/JAD-121918

Pradeep AR, 2010, J PERIODONTAL RES, V45, P223, DOI 10.1111/j.1600-0765.2009.01220.x

Riviere GR, 2002, ORAL MICROBIOL IMMUN, V17, P113, DOI 10.1046/j.0902-0055.2001.00100.x

Rolim TD, 2014, J ALZHEIMERS DIS, V38, P823, DOI 10.3233/JAD-131283

Román-Malo L, 2017, INT J MOL SCI, V18, DOI 10.3390/ijms18071438

Schwarz J, 2006, J NEUROL, V253, P608, DOI 10.1007/s00415-006-0068-4

Sim SJ, 2008, J PERIODONTOL, V79, P1652, DOI [10.1902/jop.2008.080015, 10.1902/jop.2008.080015 ]

Singhrao SK, 2019, J ORAL MICROBIOL, V11, DOI 10.1080/20002297.2018.1563405

Sochocka M, 2017, CURR NEUROPHARMACOL, V15, P996, DOI 10.2174/1570159X15666170313122937

Stein PS, 2012, ALZHEIMERS DEMENT, V8, P196, DOI 10.1016/j.jalz.2011.04.006

Su L, 2016, BRIT J PSYCHIAT, V209, P525, DOI 10.1192/bjp.bp.116.190165

Syrjälä AMH, 2012, GERODONTOLOGY, V29, P36, DOI 10.1111/j.1741-2358.2010.00396.x

Torres-Platas SG, 2014, BRAIN BEHAV IMMUN, V42, P50, DOI 10.1016/j.bbi.2014.05.007

van Berckel BN, 2008, BIOL PSYCHIAT, V64, P820, DOI 10.1016/j.biopsych.2008.04.025

Wu TJ, 2000, ARCH INTERN MED, V160, P2749, DOI 10.1001/archinte.160.18.2749

Wu Z, 2005, J NEUROIMMUNOL, V167, P90, DOI 10.1016/j.jneuroim.2005.06.025

Wu Z, 2017, BRAIN BEHAV IMMUN, V65, P350, DOI 10.1016/j.bbi.2017.06.002

Zhang J, 2018, J NEUROINFLAMM, V15, DOI 10.1186/s12974-017-1052-x

NR 85

TC 57

Z9 65

U1 2

U2 20

PU MDPI

PI BASEL

PA ST ALBAN-ANLAGE 66, CH-4052 BASEL, SWITZERLAND

EI 1422-0067

J9 INT J MOL SCI

JI Int. J. Mol. Sci.

PD AUG

PY 2019

VL 20

IS 15

AR 3723

DI 10.3390/ijms20153723

PG 12

WC Biochemistry & Molecular Biology; Chemistry, Multidisciplinary

WE Science Citation Index Expanded (SCI-EXPANDED); Social Science Citation Index (SSCI)

SC Biochemistry & Molecular Biology; Chemistry

GA IS8ET

UT WOS:000482383000116

PM 31366073

OA gold, Green Submitted, Green Published

DA 2024-08-12

ER

PT J

AU Shibu, MA

Bharath, M

Velmurugan, BK

AF Shibu, Marthandam Asokan

Bharath, Mahalakshmi

Velmurugan, Bharath Kumar

TI Regulating Inflammation Associated Ferroptosis- A Treatment Strategy for

parkinson's disease

SO CURRENT MEDICINAL CHEMISTRY

LA English

DT Review

DE Ferroptosis; Parkinson's disease; iron accumulation; inflammation;

neurodegeneration; drugs

ID CELL-DEATH; ALPHA-SYNUCLEIN; SUBSTANTIA-NIGRA; OXIDATIVE STRESS;

NEUROLOGICAL DISEASE; NEUROTROPHIC FACTORS; IRON; MECHANISMS; MODELS;

BRAIN

AB Ferroptosis plays a critical regulatory role as a new kind of cell death, initiating and developing an array of disorders like neurological diseases, acute injury of kidney, tumors, and ischemia, etc. Although selective deposition of iron is one of the pathogenic reasons for PD, it's underlying mechanism is still unknown. In this review, the role of neuroinflammation in Parkinson's disease (PD) leading to neurodegeneration has been discussed in detail. The accumulation of brain iron has been found in many chronic neurological disorders, including PD. We have also discussed the unique features of ferroptosis , and it links in aggravating the pathology of PD. Further, the concept of targeting ferroptosis for PD pathology and inducers and inhibitors, pharmacological drugs and clinical trials for PD candidates in phase IV stage in completed status are detailed in the respective sections.

C1 [Shibu, Marthandam Asokan] Hualien Tzu Chi Hosp, Buddhist Tzu Chi Med Fdn, Cardiovasc & Mitochondria Related Dis Res Ctr, Hualien, China.

[Bharath, Mahalakshmi] Vels Publisher, Dept Res & Dev, Bodinayakanur, Tamil Nadu, India.

[Velmurugan, Bharath Kumar] Asia Univ, Dept Med Lab Sci & Biotechnol, Taichung, China.

C3 Buddhist Tzu Chi General Hospital; Hualien Tzu Chi Hospital; Asia

University China

RP Velmurugan, BK (corresponding author), Asia Univ, Dept Med Lab Sci & Biotechnol, Taichung, China.

EM bharathvel@asia.edu.tw

RI ; Marthandam Asokan, Shibu/F-6137-2018

OI Bharath Kumar, V./0000-0002-4786-9913; Marthandam Asokan,

Shibu/0000-0001-6381-3525; B, Mahalakshmi/0000-0003-2917-7160

CR Alza NP, 2019, FRONT CELL NEUROSCI, V13, DOI 10.3389/fncel.2019.00175

Angeli JPF, 2014, NAT CELL BIOL, V16, P1180, DOI 10.1038/ncb3064

Aspelund A, 2015, J EXP MED, V212, P991, DOI 10.1084/jem.20142290

Athauda D, 2017, LANCET, V390, P1664, DOI 10.1016/S0140-6736(17)31585-4

Atik A, 2016, BRAIN PATHOL, V26, P410, DOI 10.1111/bpa.12370

Baruch K, 2016, NAT MED, V22, P135, DOI 10.1038/nm.4022

Bellinger FP, 2012, J PARKINSON DIS, V2, P115, DOI 10.3233/JPD-2012-11052

Bharath S, 2002, BIOCHEM PHARMACOL, V64, P1037, DOI 10.1016/S0006-2952(02)01174-7

Bové J, 2014, AUTOPHAGY, V10, P889, DOI 10.4161/auto.28286

Braak H, 2004, NEUROBIOL AGING, V25, P19, DOI 10.1016/j.neurobiolaging.2003.04.001

Büttner S, 2013, EMBO J, V32, P3041, DOI 10.1038/emboj.2013.228

Cassarino DS, 1997, BBA-MOL BASIS DIS, V1362, P77, DOI 10.1016/S0925-4439(97)00070-7

Cebrian C, 2015, CURR TOP BEHAV NEURO, V22, P237, DOI 10.1007/7854_2014_356

Chao YX, 2014, BIOMED RES INT, V2014, DOI 10.1155/2014/308654

Chen LY, 2015, ONCOL REP, V33, P1465, DOI 10.3892/or.2015.3712

Chen SL, 2012, J NEUROIMMUNE PHARM, V7, P444, DOI 10.1007/s11481-011-9337-9

Cheng HC, 2010, ANN NEUROL, V67, P715, DOI 10.1002/ana.21995

Choi ML, 2018, FEBS J, V285, P3631, DOI 10.1111/febs.14587

Costello DJ, 2004, J NEUROL NEUROSUR PS, V75, P631, DOI 10.1136/jnnp.2003.027441

Crichton RR, 2001, BIOMETALS, V14, P99, DOI 10.1023/A:1016710810701

Curtis ARJ, 2001, NAT GENET, V28, P350, DOI 10.1038/ng571

Da Mesquita S, 2018, NATURE, V560, P185, DOI 10.1038/s41586-018-0368-8

David KK, 2009, FRONT BIOSCI-LANDMRK, V14, P1116, DOI 10.2741/3297

Davies P, 2011, PLOS ONE, V6, DOI 10.1371/journal.pone.0015814

Deas E, 2016, ANTIOXID REDOX SIGN, V24, P376, DOI 10.1089/ars.2015.6343

Devos D, 2014, ANTIOXID REDOX SIGN, V21, P195, DOI 10.1089/ars.2013.5593

Dias V, 2013, J PARKINSON DIS, V3, P461, DOI 10.3233/JPD-130230

Dixon SJ, 2015, ACS CHEM BIOL, V10, P1604, DOI 10.1021/acschembio.5b00245

Dixon SJ, 2014, ELIFE, V3, DOI 10.7554/eLife.02523

Dixon SJ, 2014, NAT CHEM BIOL, V10, P9, DOI 10.1038/nchembio.1416

Dixon SJ, 2012, CELL, V149, P1060, DOI 10.1016/j.cell.2012.03.042

Do Van B, 2016, NEUROBIOL DIS, V94, P169, DOI 10.1016/j.nbd.2016.05.011

Doll S, 2017, NAT CHEM BIOL, V13, P91, DOI [10.1038/NCHEMBIO.2239, 10.1038/nchembio.2239]

Domanskyi A, 2015, HUM GENE THER, V26, P550, DOI 10.1089/hum.2015.065

Edwards TL, 2010, ANN HUM GENET, V74, P97, DOI 10.1111/j.1469-1809.2009.00560.x

Faucheux BA, 1999, LANCET, V353, P981, DOI 10.1016/S0140-6736(99)00641-8

Fink SL, 2008, P NATL ACAD SCI USA, V105, P4312, DOI 10.1073/pnas.0707370105

Fowler JS, 2002, J NUCL MED, V43, P1331

FRISCH SM, 1994, J CELL BIOL, V124, P619, DOI 10.1083/jcb.124.4.619

Fusco G, 2017, SCIENCE, V358, P1440, DOI 10.1126/science.aan6160

Galluzzi L, 2007, CELL DEATH DIFFER, V14, P1237, DOI 10.1038/sj.cdd.4402148

Galluzzi L, 2012, CELL DEATH DIFFER, V19, P107, DOI 10.1038/cdd.2011.96

Gao MH, 2016, CELL RES, V26, P1021, DOI 10.1038/cr.2016.95

Gao MH, 2015, MOL CELL, V59, P298, DOI 10.1016/j.molcel.2015.06.011

Glass CK, 2010, CELL, V140, P918, DOI 10.1016/j.cell.2010.02.016

Grammatopoulos TN, 2007, MOL NEURODEGENER, V2, DOI 10.1186/1750-1326-2-1

Greenshields AL, 2017, MOL CARCINOGEN, V56, P75, DOI 10.1002/mc.22474

Gryzik M, 2021, BBA-MOL CELL RES, V1868, DOI 10.1016/j.bbamcr.2020.118913

Ha Duy, 2012, Parkinsonism Relat Disord, V18 Suppl 1, pS218, DOI 10.1016/S1353-8020(11)70067-0

Hare DJ, 2014, CHEM SCI, V5, P2160, DOI 10.1039/c3sc53461h

Harris IS, 2015, CANCER CELL, V27, P211, DOI 10.1016/j.ccell.2014.11.019

Hassannia B, 2018, J CLIN INVEST, V128, P3341, DOI 10.1172/JCI99032

He Q, 2011, J NEURAL TRANSM, V118, P397, DOI 10.1007/s00702-010-0453-0

Hodgson RA, 2010, EXP NEUROL, V225, P384, DOI 10.1016/j.expneurol.2010.07.011

Hou W, 2016, AUTOPHAGY, V12, P1425, DOI 10.1080/15548627.2016.1187366

Hunter RL, 2007, J NEUROCHEM, V100, P1375, DOI 10.1111/j.1471-4159.2006.04327.x

Huppa JB, 2003, NAT REV IMMUNOL, V3, P973, DOI 10.1038/nri1245

JELLINGER K, 1992, J NEUROCHEM, V59, P1168, DOI 10.1111/j.1471-4159.1992.tb08362.x

Jellinger KA, 1999, J NEURAL TRANSM-SUPP, P1

Jellinger KA, 2010, J CELL MOL MED, V14, P457, DOI 10.1111/j.1582-4934.2010.01010.x

Jellinger KA, 2009, J NEURAL TRANSM, V116, P1111, DOI 10.1007/s00702-009-0240-y

JENNER P, 1992, ANN NEUROL, V32, pS82, DOI 10.1002/ana.410320714

Jóhannesson P, 2012, LAEKNABLADID, V98, P531, DOI 10.17992/lbl.2012.10.457

Kabiraj P, 2015, PROTEIN J, V34, P349, DOI 10.1007/s10930-015-9629-7

Kagan VE, 2017, NAT CHEM BIOL, V13, P81, DOI [10.1038/nchembio.2238, 10.1038/NCHEMBIO.2238]

Kannarkat GT, 2013, J PARKINSON DIS, V3, P493, DOI 10.3233/JPD-130250

Khandelwal PJ, 2011, J NEUROIMMUNOL, V238, P1, DOI 10.1016/j.jneuroim.2011.07.002

Kim HS, 2009, BEHAV BRAIN RES, V196, P168, DOI 10.1016/j.bbr.2008.09.040

Klionsky DJ, 2000, SCIENCE, V290, P1717, DOI 10.1126/science.290.5497.1717

Kosloski LM, 2010, J NEUROCHEM, V114, P1261, DOI 10.1111/j.1471-4159.2010.06834.x

Kubera M, 2009, PHARMACOL REP, V61, P1105, DOI 10.1016/S1734-1140(09)70173-2

Kumar MJ, 2004, MOL NEUROBIOL, V30, P77, DOI 10.1385/MN:30:1:077

Laurie C, 2007, J NEUROIMMUNOL, V183, P60, DOI 10.1016/j.jneuroim.2006.11.009

Lee FJS, 2001, FASEB J, V15, P916, DOI 10.1096/fj.00-0334com

Lee SS, 2003, NEUROBIOL AGING, V24, P687, DOI 10.1016/S0197-4580(02)00196-3

Li XP, 2012, NEUROSIGNALS, V20, P223, DOI 10.1159/000332954

Liu Y, 2015, BIOL PHARM BULL, V38, P1234, DOI 10.1248/bpb.b15-00048

Louyeau A, 2015, TRENDS IMMUNOL, V36, P569, DOI 10.1016/j.it.2015.08.006

Ludtmann MHR, 2018, NAT COMMUN, V9, DOI 10.1038/s41467-018-04422-2

Masliah E, 2005, NEURON, V46, P857, DOI 10.1016/j.neuron.2005.05.010

Masliah E, 2011, PLOS ONE, V6, DOI 10.1371/journal.pone.0019338

McNaught KSP, 2001, NEUROSCI LETT, V297, P191, DOI 10.1016/S0304-3940(00)01701-8

Miller RL, 2009, NEUROCHEM RES, V34, P55, DOI 10.1007/s11064-008-9656-2

MOGI M, 1994, NEUROSCI LETT, V165, P208, DOI 10.1016/0304-3940(94)90746-3

Monti DA, 2016, PLOS ONE, V11, DOI 10.1371/journal.pone.0157602

Moos T, 2004, ANN NY ACAD SCI, V1012, P14, DOI 10.1196/annals.1306.002

Napolitano M, 2002, NEUROBIOL DIS, V10, P387, DOI 10.1006/nbdi.2002.0525

Nasrolahi A, 2018, REV NEUROSCIENCE, V29, P475, DOI 10.1515/revneuro-2017-0040

Nnah IC, 2018, PHARMACEUTICALS-BASE, V11, DOI 10.3390/ph11040129

Ooko E, 2015, PHYTOMEDICINE, V22, P1045, DOI 10.1016/j.phymed.2015.08.002

Peng C, 2018, NATURE, V557, P558, DOI 10.1038/s41586-018-0104-4

Ramos JMP, 2019, FRONT IMMUNOL, V10, DOI 10.3389/fimmu.2019.00080

Peralta Ramos Javier Maria, 2017, Front Immunol, V8, P1666, DOI 10.3389/fimmu.2017.01666

Prinz M, 2017, NAT NEUROSCI, V20, P136, DOI 10.1038/nn.4475

RAUSCH WD, 1988, J NEUROCHEM, V50, P202, DOI 10.1111/j.1471-4159.1988.tb13250.x

Rees K, 2011, COCHRANE DB SYST REV, DOI 10.1002/14651858.CD008454.pub2

Reimao S, 2016, EUR J NEUROL, V23, P368, DOI 10.1111/ene.12838

Reksidler AB, 2007, EUR J PHARMACOL, V560, P163, DOI 10.1016/j.ejphar.2006.12.032

Richards RI, 2016, FRONT NEUROSCI-SWITZ, V10, DOI 10.3389/fnins.2016.00193

RINNE JO, 1989, ANN NEUROL, V26, P47, DOI 10.1002/ana.410260107

Rogers JT, 2002, J BIOL CHEM, V277, P45518, DOI 10.1074/jbc.M207435200

Schneeberger A, 2012, Parkinsonism Relat Disord, V18 Suppl 1, pS11, DOI 10.1016/S1353-8020(11)70006-2

Schneeberger A, 2010, HUM VACCINES, V6, P948, DOI 10.4161/hv.6.11.13217

Sehm T, 2016, ONCOTARGET, V7, P36021, DOI 10.18632/oncotarget.8651

Shaw AT, 2011, P NATL ACAD SCI USA, V108, P8773, DOI 10.1073/pnas.1105941108

Shimada K, 2016, NAT CHEM BIOL, V12, P497, DOI [10.1038/NCHEMBIO.2079, 10.1038/nchembio.2079]

Shimada K, 2016, CELL CHEM BIOL, V23, P225, DOI 10.1016/j.chembiol.2015.11.016

Sian-Hülsmann J, 2011, J NEUROCHEM, V118, P939, DOI 10.1111/j.1471-4159.2010.07132.x

Skouta R, 2014, J AM CHEM SOC, V136, P4551, DOI 10.1021/ja411006a

Spillantini MG, 1998, P NATL ACAD SCI USA, V95, P6469, DOI 10.1073/pnas.95.11.6469

Stockwell BR, 2017, CELL, V171, P273, DOI 10.1016/j.cell.2017.09.021

Takahashi Y, 2017, CHIN J CANCER, V36, DOI 10.1186/s40880-017-0216-5

Tansey MG, 2010, NEUROBIOL DIS, V37, P510, DOI 10.1016/j.nbd.2009.11.004

Tarze A, 2007, ONCOGENE, V26, P2606, DOI 10.1038/sj.onc.1210074

Thompson JW, 2012, J BIOL CHEM, V287, P7357, DOI 10.1074/jbc.M111.308684

Torii S, 2016, BIOCHEM J, V473, P769, DOI 10.1042/BJ20150658

Tribl GG, 2001, EXP GERONTOL, V36, P1761, DOI 10.1016/S0531-5565(01)00128-0

Tuo QZ, 2017, MOL PSYCHIATR, V22, P1520, DOI 10.1038/mp.2017.171

Vashisht AA, 2009, SCIENCE, V326, P718, DOI 10.1126/science.1176333

Wang L, 2008, CELL, V133, P693, DOI 10.1016/j.cell.2008.03.036

Ward R, 2014, LANCET NEUROL, V13, P1045, DOI 10.1016/S1474-4422(14)70117-6

Whitehead JP, 2011, INT J BIOCHEM CELL B, V43, P1071, DOI 10.1016/j.biocel.2011.04.017

Wu CH, 2018, SCI REP-UK, V8, DOI 10.1038/s41598-017-18935-1

Wu HM, 2009, NEUROPSYCHOPHARMACOL, V34, P2344, DOI 10.1038/npp.2009.64

Xie Y, 2016, CELL DEATH DIFFER, V23, P369, DOI 10.1038/cdd.2015.158

Yagoda N, 2007, NATURE, V447, P864, DOI 10.1038/nature05859

Yang WS, 2008, CHEM BIOL, V15, P234, DOI 10.1016/j.chembiol.2008.02.010

Yang WS, 2016, TRENDS CELL BIOL, V26, P165, DOI 10.1016/j.tcb.2015.10.014

Yang WS, 2014, CELL, V156, P317, DOI 10.1016/j.cell.2013.12.010

Yue X, 2014, NEUROSCIENCE, V258, P385, DOI 10.1016/j.neuroscience.2013.11.038

NR 130

TC 10

Z9 12

U1 1

U2 24

PU BENTHAM SCIENCE PUBL LTD

PI SHARJAH

PA EXECUTIVE STE Y-2, PO BOX 7917, SAIF ZONE, 1200 BR SHARJAH, U ARAB

EMIRATES

SN 0929-8673

EI 1875-533X

J9 CURR MED CHEM

JI Curr. Med. Chem.

PY 2021

VL 28

IS 33

BP 6895

EP 6914

DI 10.2174/0929867328666210419125032

PG 20

WC Biochemistry & Molecular Biology; Chemistry, Medicinal; Pharmacology &

Pharmacy

WE Science Citation Index Expanded (SCI-EXPANDED)

SC Biochemistry & Molecular Biology; Pharmacology & Pharmacy

GA WQ3IR

UT WOS:000713713500002

PM 33874863

DA 2024-08-12

ER

PT J

AU Cao, H

Han, XS

Jia, YL

Zhang, BH

AF Cao, Hua

Han, Xinsheng

Jia, Yonglin

Zhang, Baohua

TI Inhibition of long non-coding RNA HOXA11-AS against neuroinflammation in

Parkinson's disease model via targeting miR-124-3p mediated FSTL1/NF-κB

axis

SO AGING-US

LA English

DT Article

DE HOXA11-AS; Parkinson's disease; miR-124-3p; FSTL1; NF-kappa B

ID INFLAMMATION; PROGRESSION

AB Background: Studies have revealed that lncRNA HOXA11-AS contributes to regulating inflammation, while the role of HOXA11-AS in Parkinson's disease (PD) remains unclear.

Methods: Both in vivo and in vitro PD models were induced. Gain- or loss-assays of HOXA11-AS and miR-124-3p were conducted. The neurological functions, dopaminergic neurons damage, microglia activation of PD mice were measured. Afterwards, the expressions of inflammatory factors were examined with RT-PCR. Western blot was employed to detect the level of FSTL1, NF-kappa B and NLRP3 inflammasome. Meanwhile, bioinformatics analysis and dual-luciferase reporter assay were utilized to confirm the targeting relationships among miR-1243p, HOXA11-AS and FSTL1.

Results: HOXA11-AS promoted MPTP-mediated SH-SY5Y neuronal injury and LPS-induced microglia activation, while miR-124-3p had the opposite effects. Additionally, miR-124-3p was the target of HOXA11-AS and FSTL1. HOXA11-AS overexpression enhanced the expression of inflammatory factors and FSTL1, NF-kappa B and NLRP3 inflammasome, while inhibiting NF-KB weakened HOXA11-AS-mediated neuronal damage and microglia activation. Moreover, HOXA11-AS1 downregulation ameliorated MPTP-induced neurological damages and neuroinflammation in mice.

Conclusion: Inhibition of HOXA11-AS protects mice against PD through repressing neuroinflammation and neuronal apoptosis through miR-124-3p-FSTL1-NF-kappa B axis.

C1 [Cao, Hua; Han, Xinsheng; Jia, Yonglin; Zhang, Baohua] Kaifeng Cent Hosp, Dept Neurol, Kaifeng 475000, Henan, China.

RP Zhang, BH (corresponding author), Kaifeng Cent Hosp, Dept Neurol, Kaifeng 475000, Henan, China.

EM zhangbaohua83@163.com

CR Cai LJ, 2020, AGING-US, V12, P672, DOI 10.18632/aging.102649

Chen L, 2019, MOL MED REP, V20, P5345, DOI 10.3892/mmr.2019.10752

Cheng KY, 2017, J MOL HISTOL, V48, P63, DOI 10.1007/s10735-016-9706-z

Dong RF, 2018, J CELL BIOCHEM, V119, P269, DOI 10.1002/jcb.26170

Fang CC, 2020, PARKINSONS DIS-US, V2020, DOI 10.1155/2020/2076942

Fukae J, 2020, MOV DISORD CLIN PRAC, V7, P273, DOI 10.1002/mdc3.12908

Geng LJ, 2017, EXP BIOL MED, V242, P1757, DOI 10.1177/1535370217734492

Goldman JG, 2020, NEUROL CLIN, V38, P269, DOI 10.1016/j.ncl.2019.12.003

Guo JC, 2019, EXP MOL MED, V51, DOI 10.1038/s12276-019-0328-x

Guo JX, 2016, BIOCHEM BIOPH RES CO, V478, P1528, DOI 10.1016/j.bbrc.2016.08.138

Guo T, 2020, NEOPLASMA, V67, P492, DOI 10.4149/neo_2020_190722N653

Haque ME, 2020, MOVEMENT DISORD, V35, P20, DOI 10.1002/mds.27874

Jin QS, 2018, EUR REV MED PHARMACO, V22, P6912, DOI 10.26355/eurrev_201810_16161

Li HT, 2020, MOL GENET GENOM MED, V8, DOI 10.1002/mgg3.1189

Li R, 2020, ARTHRITIS RES THER, V22, DOI 10.1186/s13075-020-2146-x

Li XX, 2019, PROTEIN SCI, V28, P1819, DOI 10.1002/pro.3696

Li YH, 2020, J CELL MOL MED, V24, P238, DOI 10.1111/jcmm.14706

Lin QY, 2019, BIOL CHEM, V400, P1217, DOI 10.1515/hsz-2018-0431

Lin W, 2015, J MEDICAL REV, V21, P41

Vong LB, 2020, ACTA BIOMATER, V109, P220, DOI 10.1016/j.actbio.2020.03.021

Lu M, 2018, EUR REV MED PHARMACO, V22, P7908, DOI 10.26355/eurrev_201811_16417

Lu SN, 2018, CELL BIOL INT, V42, P1596, DOI 10.1002/cbin.11045

Lyu Y, 2019, ANIM MODEL EXP MED, V2, P239, DOI 10.1002/ame2.12093

Ma RJ, 2020, STEM CELLS DEV, V29, P599, DOI 10.1089/scd.2019.0174

Leo EEM, 2020, J FOOD BIOCHEM, V44, DOI 10.1111/jfbc.13207

Meng XY., 2017, STROKE NERVOUS DIS, V1, P78

Pisa D, 2020, INT J BIOL SCI, V16, P1135, DOI 10.7150/ijbs.42257

Qu Y, 2019, INFLAMMATION, V42, P1491, DOI 10.1007/s10753-019-01012-2

Richards EJ, 2015, ONCOTARGET, V6, P34745, DOI 10.18632/oncotarget.5784

Schmidt K, 2020, CELL REP, V30, P541, DOI 10.1016/j.celrep.2019.12.011

Sun Q, 2019, J CELL PHYSIOL, V234, P23379, DOI 10.1002/jcp.28907

Vuokila N, 2020, INT J MOL SCI, V21, DOI 10.3390/ijms21072418

Wang RL, 2020, CLIN EXP PHARMACOL P, V47, P372, DOI 10.1111/1440-1681.13212

Wang XH, 2020, EXP THER MED, V19, P931, DOI 10.3892/etm.2019.8297

Wang YX, 2020, NEUROBIOL AGING, V87, P1, DOI 10.1016/j.neurobiolaging.2019.12.013

Wei C, 2020, ONCOL REP, V43, P1737, DOI 10.3892/or.2020.7552

Xie SP, 2019, NEUROSCI LETT, V708, DOI 10.1016/j.neulet.2019.134340

Xu JN, 2020, ONCOL RES, V28, P311, DOI 10.3727/096504020X15801233454611

Yamada S, 2018, J NEUROINFLAMM, V15, DOI 10.1186/s12974-018-1332-0

Zhang L, 2014, LIFE SCI 2-BIOCH GEN, V6, P560

Zhu SB, 2020, AM J CANCER RES, V10, P284

NR 41

TC 25

Z9 27

U1 0

U2 14

PU IMPACT JOURNALS LLC

PI ORCHARD PARK

PA 6666 E QUAKER ST, STE 1, ORCHARD PARK, NY 14127 USA

SN 1945-4589

J9 AGING-US

JI Aging-US

PD APR 30

PY 2021

VL 13

IS 8

BP 11455

EP 11469

PG 15

WC Cell Biology; Geriatrics & Gerontology

WE Science Citation Index Expanded (SCI-EXPANDED)

SC Cell Biology; Geriatrics & Gerontology

GA RW1CE

UT WOS:000646260200015

PM 33839699

OA gold, Green Published

DA 2024-08-12

ER

PT J

AU Spielman, LJ

Little, JP

Klegeris, A

AF Spielman, Lindsay Joy

Little, Jonathan Peter

Klegeris, Andis

TI Physical activity and exercise attenuate neuroinflammation in

neurological diseases

SO BRAIN RESEARCH BULLETIN

LA English

DT Review

DE Alzheimer's disease; Exercise; Major depressive disorder; Parkinson's

disease; Schizophrenia; Sedentary lifestyle

ID ASTROCYTE-TARGETED EXPRESSION; C-REACTIVE PROTEIN; parkinson's disease;

alzheimer's disease; SKELETAL-MUSCLE; MICROGLIAL ACTIVATION; NEUROTROPHIC

FACTOR; TRANSGENIC MICE; ALPHA-SYNUCLEIN; ADIPOSE-TISSUE

AB Major depressive disorder (MDD), schizophrenia (SCH), Alzheimer's disease (AD), and Parkinson's disease (PD) are devastating neurological disorders, which increasingly contribute to global morbidity and mortality. Although the pathogenic mechanisms of these conditions are quite diverse, chronic neuroinflammation is one underlying feature shared by all these diseases. Even though the specific root causes of these diseases remain to be identified, evidence indicates that the observed neuroinflammation is initiated by unique pathological features associated with each specific disease. If the initial acute inflammation is not resolved, a chronic neuroinflammatory state develops and ultimately contributes to disease progression. Chronic neuroinflammation is characterized by adverse and non-specific activation of glial cells, which can lead to collateral damage of nearby neurons and other glia. This misdirected neuroinflammatory response is hypothesized to contribute to neuropathology in MDD, SCH, AD, and PD. Physical activity (PA), which is critical for maintenance of whole body and brain health, may also beneficially modify neuroimmune responses. Since PA has neuroimmune-modifying properties, and the common underlying feature of MDD, SCH, AD, and PD is chronic neuroinflammation, we hypothesize that PA could minimize brain diseases by modifying glia-mediated neuroinflammation. This review highlights current evidence supporting the disease-altering potential of PA and exercise through modifications of neuroimmune responses, specifically in MDD, SCH, AD and PD. (C) 2016 Elsevier Inc. All rights reserved.

C1 [Spielman, Lindsay Joy; Klegeris, Andis] Univ British Columbia, Dept Biol, Okanagan Campus,3187 Univ Way, Kelowna, BC V1V 1V7, Canada.

[Little, Jonathan Peter] Univ British Columbia, Sch Hlth & Exercise Sci, Okanagan Campus,1147 Res Rd, Kelowna, BC V1V 1V7, Canada.

C3 University of British Columbia; University of British Columbia

RP Klegeris, A (corresponding author), Univ British Columbia, Dept Biol, Okanagan Campus,3187 Univ Way, Kelowna, BC V1V 1V7, Canada.

EM lindsayspielman@live.ca; Jonathan.Little@ubc.ca; andis.klegeris@ubc.ca

RI Little, Jonathan P./T-4076-2019

OI Little, Jonathan P./0000-0002-9796-2008

FU Jack Brown and Family Alzheimer's Disease Research Foundation; Natural

Science and Engineering Research Council of Canada; University of

British Columbia Okanagan Campus; Canadian Institutes of Health Research

(CIHR) [MSH-141980]

FX AK is supported by grants from the Jack Brown and Family Alzheimer's

Disease Research Foundation, the Natural Science and Engineering

Research Council of Canada and the University of British Columbia

Okanagan Campus. JPL is supported by a Canadian Institutes of Health

Research (CIHR) New Investigator Salary Award (MSH-141980).

CR Adamson BC, 2015, ARCH PHYS MED REHAB, V96, P1329, DOI 10.1016/j.apmr.2015.01.005

Agudelo LZ, 2014, CELL, V159, P33, DOI 10.1016/j.cell.2014.07.051

Altshuler LL, 2010, BIPOLAR DISORD, V12, P541, DOI 10.1111/j.1399-5618.2010.00838.x

Annesi JJ, 2013, INT Q COMMUNITY HEAL, V33, P391, DOI 10.2190/IQ.33.4.f

Archer T, 2015, NEUROTOX RES, V28, P268, DOI 10.1007/s12640-015-9543-y

Arciniegas David B, 2015, Continuum (Minneap Minn), V21, P715, DOI 10.1212/01.CON.0000466662.89908.e7

Banati RB, 1998, MOVEMENT DISORD, V13, P221, DOI 10.1002/mds.870130205

Banks WA, 1995, NEUROIMMUNOMODULAT, V2, P241, DOI 10.1159/000097202

Barnes DE, 2011, LANCET NEUROL, V10, P819, DOI 10.1016/S1474-4422(11)70072-2

Belarbi K, 2011, NEUROBIOL DIS, V43, P486, DOI 10.1016/j.nbd.2011.04.022

Bennett MR, 2010, AUST NZ J PSYCHIAT, V44, P301, DOI 10.3109/00048670903555146

Bergman D, 2013, INTERN EMERG MED, V8, pS17, DOI 10.1007/s11739-013-0921-2

Blackburn SD, 2007, TRENDS MICROBIOL, V15, P143, DOI 10.1016/j.tim.2007.02.006

Block ML, 2007, NAT REV NEUROSCI, V8, P57, DOI 10.1038/nrn2038

Block ML, 2005, PROG NEUROBIOL, V76, P77, DOI 10.1016/j.pneurobio.2005.06.004

Bloem BR, 2015, MOVEMENT DISORD, V30, P1504, DOI 10.1002/mds.26363

Blumenthal JA, 2007, PSYCHOSOM MED, V69, P587, DOI 10.1097/PSY.0b013e318148c19a

BRETT FM, 1995, J NEUROPATH EXP NEUR, V54, P766, DOI 10.1097/00005072-199511000-00003

Brundage SI, 2008, J TRAUMA, V65, P367, DOI 10.1097/TA.0b013e3181801cc0

Bruun JM, 2006, AM J PHYSIOL-ENDOC M, V290, pE961, DOI 10.1152/ajpendo.00506.2005

Calabrese F, 2014, FRONT CELL NEUROSCI, V8, DOI 10.3389/fncel.2014.00430

Cameron B, 2010, NEUROBIOL DIS, V37, P503, DOI 10.1016/j.nbd.2009.10.006

Canning CG, 2015, NEUROLOGY, V84, P304, DOI 10.1212/WNL.0000000000001155

Carek PJ, 2011, INT J PSYCHIAT MED, V41, P15, DOI 10.2190/PM.41.1.c

CARLSSON A, 1963, ACTA PHARMACOL TOX, V20, P140

Centers for Disease Control and Prevention, 2015, DIV NUTR PHYS ACT OO

Colton CA, 2006, J NEUROINFLAMM, V3, DOI 10.1186/1742-2094-3-27

Comassi M, 2015, INTERN MED J, V45, P74, DOI 10.1111/imj.12625

Cotter D, 2001, ARCH GEN PSYCHIAT, V58, P545, DOI 10.1001/archpsyc.58.6.545

Cunningham C, 2013, GLIA, V61, P71, DOI 10.1002/glia.22350

Czlonkowska Anna, 2002, Med Sci Monit, V8, pRA165

Dantzer R, 2011, PSYCHONEUROENDOCRINO, V36, P426, DOI 10.1016/j.psyneuen.2010.09.012

Dayan P, 2008, PLOS COMPUT BIOL, V4, DOI 10.1371/journal.pcbi.0040004

de Andrade LP, 2013, J AM GERIATR SOC, V61, P1919, DOI 10.1111/jgs.12531

Dheen ST, 2007, CURR MED CHEM, V14, P1189, DOI 10.2174/092986707780597961

Dilger RN, 2008, J LEUKOCYTE BIOL, V84, P932, DOI 10.1189/jlb.0208108

Doose M, 2015, INT J PSYCHIAT CLIN, V19, P267, DOI 10.3109/13651501.2015.1082599

Dowlati Y, 2010, BIOL PSYCHIAT, V67, P446, DOI 10.1016/j.biopsych.2009.09.033

Dunn AL, 2005, AM J PREV MED, V28, P1, DOI 10.1016/j.amepre.2004.09.003

Dunner DL, 2007, J CLIN PSYCHIAT, V68, P619, DOI 10.4088/JCP.0307e06

Egan B, 2013, CELL METAB, V17, P162, DOI 10.1016/j.cmet.2012.12.012

Erickson KI, 2012, NEUROSCIENTIST, V18, P82, DOI 10.1177/1073858410397054

Eyre H, 2012, BRAIN BEHAV IMMUN, V26, P251, DOI 10.1016/j.bbi.2011.09.015

Falconer CL, 2014, NUTR METAB CARDIOVAS, V24, P956, DOI 10.1016/j.numecd.2014.03.009

Fattori E, 1995, EUR J NEUROSCI, V7, P2441, DOI 10.1111/j.1460-9568.1995.tb01042.x

Fillman SG, 2013, MOL PSYCHIATR, V18, P133, DOI 10.1038/mp.2012.199

Fiuza-Luces C, 2013, PHYSIOLOGY, V28, P330, DOI 10.1152/physiol.00019.2013

Flynn MG, 2006, EXERC SPORT SCI REV, V34, P176, DOI 10.1249/01.jes.0000240027.22749.14

Frank-Cannon TC, 2009, MOL NEURODEGENER, V4, DOI 10.1186/1750-1326-4-47

Frazzitta G, 2015, NEUROREHAB NEURAL RE, V29, P123, DOI 10.1177/1545968314542981

Frazzitta G, 2014, NEUROREHAB NEURAL RE, V28, P163, DOI 10.1177/1545968313508474

Funk JA, 2011, BRAIN BEHAV IMMUN, V25, P1063, DOI 10.1016/j.bbi.2011.03.012

Gaesser Glenn A, 2007, Curr Diab Rep, V7, P14, DOI 10.1007/s11892-007-0004-8

Garland T, 2011, J EXP BIOL, V214, P206, DOI 10.1242/jeb.048397

Giallauria Francesco, 2014, Monaldi Arch Chest Dis, V82, P61

Gibbons TE, 2014, BEHAV BRAIN RES, V272, P131, DOI 10.1016/j.bbr.2014.05.049

Giralt M, 2002, NEUROBIOL DIS, V9, P319, DOI 10.1006/nbdi.2002.0480

Gleeson M, 2012, SCAND J MED SCI SPOR, V22, P410, DOI 10.1111/j.1600-0838.2010.01272.x

Gleeson M, 2011, NAT REV IMMUNOL, V11, P607, DOI 10.1038/nri3041

Gong CY, 2011, MED HYPOTHESES, V77, P383, DOI 10.1016/j.mehy.2011.05.022

Guan ZW, 2006, BRAIN BEHAV IMMUN, V20, P64, DOI 10.1016/j.bbi.2005.04.005

Gyorkos AM, 2014, NEUROSCIENCE, V257, P111, DOI 10.1016/j.neuroscience.2013.10.068

Harizi H, 2008, TRENDS MOL MED, V14, P461, DOI 10.1016/j.molmed.2008.08.005

HAUW JJ, 1990, NEUROSCI LETT, V119, P182, DOI 10.1016/0304-3940(90)90829-X

Hoffman JM, 2010, PM&R, V2, P911, DOI 10.1016/j.pmrj.2010.06.008

Holloszy JO, 2005, J APPL PHYSIOL, V99, P338, DOI 10.1152/japplphysiol.00123.2005

Howes OD, 2009, SCHIZOPHRENIA BULL, V35, P549, DOI 10.1093/schbul/sbp006

Iida KT, 2001, J BIOL CHEM, V276, P32531, DOI 10.1074/jbc.M009894200

Javitt DC, 2007, INT REV NEUROBIOL, V78, P69, DOI 10.1016/S0074-7742(06)78003-5

Jessen KR, 2004, INT J BIOCHEM CELL B, V36, P1861, DOI 10.1016/j.biocel.2004.02.023

Johnson SS, 2015, AM J HEALTH PROMOT, V29, pTAHP4

Kamphuis W, 2012, PLOS ONE, V7, DOI 10.1371/journal.pone.0042823

Kanba S, 2014, JPN J NEUROPSYCHOPH, V34, P11

Kang EB, 2013, APOPTOSIS, V18, P1332, DOI 10.1007/s10495-013-0884-9

Kawanishi N, 2013, MED SCI SPORT EXER, V45, P1684, DOI 10.1249/MSS.0b013e31828ff9c6

Kawanishi N, 2010, EXERC IMMUNOL REV, V16, P105

Kegel ME, 2014, INT J TRYPTOPHAN RES, V7, P15, DOI [10.4137/IJTR.S16800, 10.4137/IJTRTR.S16800]

Keller P, 2003, BIOCHEM BIOPH RES CO, V310, P550, DOI 10.1016/j.bbrc.2003.09.048

Kemi OJ, 2002, J APPL PHYSIOL, V93, P1301, DOI 10.1152/japplphysiol.00231.2002

Kestler LP, 2001, BEHAV PHARMACOL, V12, P355, DOI 10.1097/00008877-200109000-00007

Kilmartin B, 2004, EUR J IMMUNOL, V34, P2041, DOI 10.1002/eji.200425108

Kim HJ, 2014, PSYCHIAT RES, V220, P792, DOI 10.1016/j.psychres.2014.09.020

Knab AM, 2009, PHYSIOL BEHAV, V98, P433, DOI 10.1016/j.physbeh.2009.07.006

Kohman RA, 2012, BRAIN BEHAV IMMUN, V26, P803, DOI 10.1016/j.bbi.2011.10.006

KORNHUBER HH, 1984, NERVENARZT, V55, P602

Kuo FC, 2013, PSYCHIAT RES, V209, P150, DOI 10.1016/j.psychres.2012.11.020

La Gerche A, 2015, PLOS ONE, V10, DOI 10.1371/journal.pone.0130031

Land Walter G, 2015, Sultan Qaboos Univ Med J, V15, pe9

LAPCHAK PA, 1993, NEUROSCIENCE, V53, P297, DOI 10.1016/0306-4522(93)90196-M

Lau YS, 2011, EUR J NEUROSCI, V33, P1264, DOI 10.1111/j.1460-9568.2011.07626.x

Law LLF, 2014, AGE AGEING, V43, P813, DOI 10.1093/ageing/afu055

Leem YH, 2011, BIOCHEM BIOPH RES CO, V406, P359, DOI 10.1016/j.bbrc.2011.02.046

Leza JC, 2015, NEUROSCI BIOBEHAV R, V55, P612, DOI 10.1016/j.neubiorev.2015.05.014

Liu WN, 2013, BEHAV BRAIN RES, V242, P110, DOI 10.1016/j.bbr.2012.12.041

Lull ME, 2010, NEUROTHERAPEUTICS, V7, P354, DOI 10.1016/j.nurt.2010.05.014

Lynch AM, 2010, AGING DIS, V1, P262

MAEDA Y, 1994, J EXP MED, V180, P2297, DOI 10.1084/jem.180.6.2297

MAES M, 1995, PROG NEURO-PSYCHOPH, V19, P11, DOI 10.1016/0278-5846(94)00101-M

Maes M, 2011, NEUROENDOCRINOL LETT, V32, P7

Mahlknecht P, 2013, J NEURAL TRANSM, V120, pS9, DOI 10.1007/s00702-013-1038-5

MANDYBUR TI, 1989, ACTA NEUROPATHOL, V78, P329, DOI 10.1007/BF00687764

Marques O, 2012, CELL DEATH DIS, V3, DOI 10.1038/cddis.2012.94

Martin SA, 2014, NEUROIMMUNOMODULAT, V21, P52, DOI 10.1159/000356144

Maynard CL, 2008, IMMUNOL REV, V226, P219, DOI 10.1111/j.1600-065X.2008.00711.x

McCarthy Michael, 2015, BMJ, V351, ph3785, DOI 10.1136/bmj.h3785

McFarlin BK, 2006, J GERONTOL A-BIOL, V61, P388, DOI 10.1093/gerona/61.4.388

Melancon MO, 2014, APPL PHYSIOL NUTR ME, V39, P1250, DOI 10.1139/apnm-2014-0092

Michigan A, 2011, MOL DIAGN THER, V15, P265, DOI 10.2165/11593400-000000000-00000

Minghetti L, 2005, BRAIN RES REV, V48, P251, DOI 10.1016/j.brainresrev.2004.12.015

Monteiro RS, 2015, MED HYPOTHESES, V85, P537, DOI 10.1016/j.mehy.2015.07.011

Moore SA, 2015, NEUROREHAB NEURAL RE, V29, P623, DOI 10.1177/1545968314562116

Mori T, 2010, GLIA, V58, P300, DOI 10.1002/glia.20924

Morris JK, 2011, EXP NEUROL, V231, P171, DOI 10.1016/j.expneurol.2011.06.005

Nagatsu T, 2000, J NEURAL TRANSM-SUPP, P143

Neefkes-Zonneveld CR, 2015, ARCH PHYS MED REHAB, V96, P30, DOI 10.1016/j.apmr.2014.07.006

Neubauer O, 2013, J APPL PHYSIOL, V114, P1677, DOI 10.1152/japplphysiol.00143.2013

Nichol KE, 2008, J NEUROINFLAMM, V5, DOI 10.1186/1742-2094-5-13

NIEMAN DC, 1990, J SPORT MED PHYS FIT, V30, P316

Noto C., 2015, WORLD J BIOL PSYCHIA

Nowotny K, 2015, BIOMOLECULES, V5, P194, DOI 10.3390/biom5010194

Nutt DJ, 2002, INT CLIN PSYCHOPHARM, V17, pS1

Oláh G, 2013, DRUG DES DEV THER, V7, P981, DOI 10.2147/DDDT.S44496

Osborn GG, 2010, J AM OSTEOPATH ASSOC, V110, pS16

Ouchi Y, 2009, PARKINSONISM RELAT D, V15, pS200, DOI 10.1016/S1353-8020(09)70814-4

Packer N, 2015, J SPORT MED PHYS FIT, V55, P368

Parajuli B, 2012, J NEUROINFLAMM, V9, DOI 10.1186/1742-2094-9-268

Pedersen BK, 2006, ESSAYS BIOCHEM, V42, P105, DOI 10.1042/bse0420105

Peixoto TCA, 2015, CAN J CARDIOL, V31, P308, DOI 10.1016/j.cjca.2014.11.014

Penkowa M, 2003, EXP NEUROL, V181, P130, DOI 10.1016/S0014-4886(02)00051-1

Piao CS, 2013, NEUROBIOL DIS, V54, P252, DOI 10.1016/j.nbd.2012.12.017

Pies RW, 2009, J AFFECT DISORDERS, V116, P1, DOI 10.1016/j.jad.2008.11.009

Ploeger HE, 2009, EXERC IMMUNOL REV, V15, P6

Powers MB, 2015, COGN BEHAV THERAPY, V44, P237, DOI 10.1080/16506073.2015.1047286

Prestes J, 2008, INT J SPORTS MED, V29, P1010, DOI 10.1055/s-2008-1038737

Puhl SL, 2015, AM J PHYSIOL-HEART C, V309, pH345, DOI 10.1152/ajpheart.00683.2014

Qian L, 2010, J NEURAL TRANSM, V117, P971, DOI 10.1007/s00702-010-0428-1

Qin LY, 2013, GLIA, V61, P855, DOI 10.1002/glia.22479

Radak Z, 2007, APPL PHYSIOL NUTR ME, V32, P942, DOI 10.1139/H07-081

Radák Z, 2013, FOOD CHEM TOXICOL, V61, P36, DOI 10.1016/j.fct.2013.01.047

Raedler TJ, 2011, CURR OPIN PSYCHIATR, V24, P519, DOI 10.1097/YCO.0b013e32834b9db6

Rajkowska G, 1999, BIOL PSYCHIAT, V45, P1085, DOI 10.1016/S0006-3223(99)00041-4

Rajkowska G, 2013, BIOL PSYCHIAT, V73, P613, DOI 10.1016/j.biopsych.2012.09.024

Rao JS, 2011, TRANSL PSYCHIAT, V1, DOI 10.1038/tp.2011.27

Rao JS, 2013, SCHIZOPHR RES, V147, P24, DOI 10.1016/j.schres.2013.02.017

Rees K., 2011, COCHRANE DB SYST REV, V9

Rethorst CD, 2013, MOL PSYCHIATR, V18, P1119, DOI 10.1038/mp.2012.125

Reynolds AD, 2008, J NEUROCHEM, V104, P1504, DOI 10.1111/j.1471-4159.2007.05087.x

Robinson E, 2015, J APPL PHYSIOL, V119, P508, DOI 10.1152/japplphysiol.00334.2015

Rohde T, 1997, AM J PHYSIOL-ENDOC M, V273, pE85, DOI 10.1152/ajpendo.1997.273.1.E85

Roig M, 2013, NEUROSCI BIOBEHAV R, V37, P1645, DOI 10.1016/j.neubiorev.2013.06.012

Rosety-Rodriguez M, 2014, ARCH PHYS MED REHAB, V95, P297, DOI 10.1016/j.apmr.2013.08.246

Rozovsky I, 1998, NEUROBIOL AGING, V19, P97, DOI 10.1016/S0197-4580(97)00169-3

Ruberg M, 1997, REV NEUROL, V153, P499

Sanacora G, 2012, NEUROPHARMACOLOGY, V62, P63, DOI 10.1016/j.neuropharm.2011.07.036

Santiago JA, 2013, TRENDS MOL MED, V19, P176, DOI 10.1016/j.molmed.2013.01.002

Scarmeas N, 2009, JAMA-J AM MED ASSOC, V302, P627, DOI 10.1001/jama.2009.1144

Selkirk GA, 2009, AM J PHYSIOL-REG I, V296, pR575, DOI 10.1152/ajpregu.90683.2008

Serrano-Pozo A, 2011, AM J PATHOL, V179, P1373, DOI 10.1016/j.ajpath.2011.05.047

Shockett PE, 2016, PHYSIOL REP, V4, DOI 10.14814/phy2.12672

Smith JA, 1996, AM J PHYSIOL-REG I, V270, pR838, DOI 10.1152/ajpregu.1996.270.4.R838

Sokolova A, 2009, BRAIN PATHOL, V19, P392, DOI 10.1111/j.1750-3639.2008.00188.x

Spielman LJ, 2014, J NEUROIMMUNOL, V273, P8, DOI 10.1016/j.jneuroim.2014.06.004

STAHL SM, 1984, J CLIN PSYCHIAT, V45, P37

Starkie R, 2003, FASEB J, V17, P884, DOI 10.1096/fj.02-0670fje

Steensberg A, 2003, AM J PHYSIOL-ENDOC M, V285, pE433, DOI 10.1152/ajpendo.00074.2003

Stewart LK, 2005, BRAIN BEHAV IMMUN, V19, P389, DOI 10.1016/j.bbi.2005.04.003

Stojanovic MD, 2012, J SPORT MED PHYS FIT, V52, P375

Stranska Zuzana, 2015, Vnitr Lek, V61, P365

Stubbs B, 2015, SCHIZOPHR RES, V168, P461, DOI 10.1016/j.schres.2015.07.003

Suijo K, 2013, INT J SPORTS MED, V34, P368, DOI 10.1055/s-0032-1323747

Swartz KR, 2001, BRAIN RES, V896, P86, DOI 10.1016/S0006-8993(01)02013-3

Tapia-Rojas C, 2016, BRAIN PATHOL, V26, P62, DOI 10.1111/bpa.12255

Teismann P, 2003, P NATL ACAD SCI USA, V100, P5473, DOI 10.1073/pnas.0837397100

Timmerman KL, 2008, J LEUKOCYTE BIOL, V84, P1271, DOI 10.1189/jlb.0408244

TISSOT R, 1975, Neuropsychobiology, V1, P243, DOI 10.1159/000117498

Torres-Platas SG, 2014, BRAIN BEHAV IMMUN, V42, P50, DOI 10.1016/j.bbi.2014.05.007

Tucsek Z, 2014, J GERONTOL A-BIOL, V69, P1212, DOI 10.1093/gerona/glt177

Uc EY, 2014, NEUROLOGY, V83, P413, DOI 10.1212/WNL.0000000000000644

Uher R, 2014, DEPRESS ANXIETY, V31, P459, DOI 10.1002/da.22217

Uysal N, 2015, BIOTECH HISTOCHEM, V90, P55, DOI 10.3109/10520295.2014.946968

Valkanova V, 2013, J AFFECT DISORDERS, V150, P736, DOI 10.1016/j.jad.2013.06.004

van der Sluijs KF, 2004, J IMMUNOL, V172, P7603, DOI 10.4049/jimmunol.172.12.7603

Varnum MM, 2012, ARCH IMMUNOL THER EX, V60, P251, DOI 10.1007/s00005-012-0181-2

Weinberg L, 2014, ACTA PSYCHOL, V153, P13, DOI 10.1016/j.actpsy.2014.06.011

Weisberg SP, 2003, J CLIN INVEST, V112, P1796, DOI 10.1172/JCI200319246

Wise R A, 1985, Psychiatr Med, V3, P445

Xu Lin, 2008, Zhong Nan Da Xue Xue Bao Yi Xue Ban, V33, P326

Yu Jia-Ling, 2014, Shengli Xuebao, V66, P559

Yu Q, 2013, EXP THER MED, V6, P1431, DOI 10.3892/etm.2013.1319

Zhang W, 2005, FASEB J, V19, P533, DOI 10.1096/fj.04-2751com

Zhao G, 2015, NEUROSCIENCE, V298, P357, DOI 10.1016/j.neuroscience.2015.04.038

Zinger A, 2011, PARKINSONS DIS-US, V2011, DOI 10.4061/2011/716859

Zoladz JA, 2014, J PHYSIOL PHARMACOL, V65, P441

Zoppini G, 2006, NUTR METAB CARDIOVAS, V16, P543, DOI 10.1016/j.numecd.2005.09.004

Zou YM, 2015, NEUROPSYCH DIS TREAT, V11, P695, DOI 10.2147/NDT.S79707

NR 195

TC 83

Z9 90

U1 2

U2 55

PU PERGAMON-ELSEVIER SCIENCE LTD

PI OXFORD

PA THE BOULEVARD, LANGFORD LANE, KIDLINGTON, OXFORD OX5 1GB, United Kingdom

SN 0361-9230

EI 1873-2747

J9 BRAIN RES BULL

JI Brain Res. Bull.

PD JUL

PY 2016

VL 125

BP 19

EP 29

DI 10.1016/j.brainresbull.2016.03.012

PG 11

WC Neurosciences

WE Science Citation Index Expanded (SCI-EXPANDED); Social Science Citation Index (SSCI)

SC Neurosciences & Neurology

GA DV3BR

UT WOS:000382796500002

PM 27021169

DA 2024-08-12

ER

PT J

AU Saha, S

Buttari, B

Profumo, E

Tucci, P

Saso, L

AF Saha, Sarmistha

Buttari, Brigitta

Profumo, Elisabetta

Tucci, Paolo

Saso, Luciano

TI A Perspective on Nrf2 Signaling Pathway for Neuroinflammation: A

Potential Therapeutic Target in Alzheimer's and Parkinson's Diseases

SO FRONTIERS IN CELLULAR NEUROSCIENCE

LA English

DT Review

DE Alzheimer's disease; Parkinson's disease; Nrf2 signaling pathway;

neuroinflammation; oxidative stress; Keap1

ID TRANSCRIPTION FACTOR NRF2; MITOCHONDRIAL COMPLEX-I; FUMARIC-ACID ESTERS;

PROTEIN-KINASE-C; OXIDATIVE STRESS; MOUSE MODEL; LIPID-PEROXIDATION;

CEREBROSPINAL-FLUID; SUBARACHNOID HEMORRHAGE; NF-E2-RELATED FACTOR-2

AB Neuroinflammation plays a pivotal role in Alzheimer's disease (AD) and Parkinson's disease (PD), the leading causes of dementia. These neurological disorders are characterized by the accumulation of misfolded proteins such as amyloid-ss (Ass), tau protein and alpha-synuclein, contributing to mitochondrial fragmentation, oxidative stress, and neuroinflammation. Misfolded proteins activate microglia, which induces neuroinflammation, expression of pro-inflammatory cytokines and subsequently facilitates synaptic damage and neuronal loss. So far, all the proposed drugs were based on the inhibition of protein aggregation and were failed in clinical trials. Therefore, the treatment options of dementia are still a challenging issue. Thus, it is worthwhile to study alternative therapeutic strategies. In this context, there is increasing data on the pivotal role of transcription factor NF- E2 p45-related factor 2 (Nrf2) on the redox homeostasis and anti-inflammatory functions in neurodegenerative disorders. Interestingly, Nrf2 signaling pathway has shown upregulation of antioxidant genes, inhibition of microglia-mediated inflammation, and improved mitochondrial function in neurodegenerative disease, suggesting Nrf2 activation could be a novel therapeutic approach to target pathogenesis. The present review will examine the correlation between Nrf2 signaling with neuroinflammation in AD and PD.

C1 [Saha, Sarmistha; Buttari, Brigitta; Profumo, Elisabetta] Italian Natl Inst Hlth, Dept Cardiovasc Endocrine Metabol Dis & Aging, Rome, Italy.

[Tucci, Paolo] Univ Foggia, Dept Clin & Expt Med, Foggia, Italy.

[Saso, Luciano] Sapienza Univ Rome, Dept Physiol & Pharmacol Vittorio Erspamer, Rome, Italy.

C3 Istituto Superiore di Sanita (ISS); University of Foggia; Sapienza

University Rome

RP Saha, S (corresponding author), Italian Natl Inst Hlth, Dept Cardiovasc Endocrine Metabol Dis & Aging, Rome, Italy.

EM sarmistha_pharmacol@yahoo.com

RI Tucci, Paolo/C-7197-2015; Saha, Sarmistha/ABH-8282-2020; saso,

luciano/F-6306-2012; Profumo, Elisabetta/B-4255-2015; Buttari,

Brigitta/I-3030-2014

OI Tucci, Paolo/0000-0001-7314-445X; saso, luciano/0000-0003-4530-8706;

Profumo, Elisabetta/0000-0002-7261-8884; Buttari,

Brigitta/0000-0002-0575-4106

CR Advedissian T, 2016, BIOCHEM BIOPH RES CO, V473, P87, DOI 10.1016/j.bbrc.2016.03.056

Ahuja M, 2016, J NEUROSCI, V36, P6332, DOI 10.1523/JNEUROSCI.0426-16.2016

Anandhan A, 2021, AGING DIS, V12, P964, DOI 10.14336/AD.2021.0511

Ansari MA, 2010, J NEUROPATH EXP NEUR, V69, P155, DOI 10.1097/NEN.0b013e3181cb5af4

Arlt S, 2002, CURR OPIN LIPIDOL, V13, P289, DOI 10.1097/00041433-200206000-00009

Ay M, 2017, J NEUROCHEM, V141, P766, DOI 10.1111/jnc.14033

Bahn G, 2019, P NATL ACAD SCI USA, V116, P12516, DOI 10.1073/pnas.1819541116

Bahn G, 2019, NEUROMOL MED, V21, P1, DOI 10.1007/s12017-018-08523-5

Barone MC, 2011, DIS MODEL MECH, V4, P701, DOI 10.1242/dmm.007575

Bloom DA, 2003, J BIOL CHEM, V278, P44675, DOI 10.1074/jbc.M307633200

BlumDegen D, 1995, NEUROSCI LETT, V202, P17, DOI 10.1016/0304-3940(95)12192-7

Branca C, 2017, HUM MOL GENET, V26, P4823, DOI 10.1093/hmg/ddx361

Brennan MS, 2017, SCI REP-UK, V7, DOI 10.1038/srep42054

Buendia I, 2016, PHARMACOL THERAPEUT, V157, P84, DOI 10.1016/j.pharmthera.2015.11.003

Canning P, 2015, FREE RADICAL BIO MED, V88, P101, DOI 10.1016/j.freeradbiomed.2015.05.034

Chambel SS, 2015, BIOMED RES INT, V2015, DOI 10.1155/2015/597134

Chen CM, 2009, NEUROBIOL DIS, V33, P429, DOI 10.1016/j.nbd.2008.11.011

Chhetri J, 2018, CURR ALZHEIMER RES, V15, P56, DOI 10.2174/1567205014666170203095802

Chinta SJ, 2013, J INTERN MED, V273, P429, DOI 10.1111/joim.12029

Chinta SJ, 2010, NEUROSCI LETT, V486, P235, DOI 10.1016/j.neulet.2010.09.061

Chowdhry S, 2013, ONCOGENE, V32, P3765, DOI 10.1038/onc.2012.388

Cicchetti F, 2002, EUR J NEUROSCI, V15, P991, DOI 10.1046/j.1460-9568.2002.01938.x

Ciechanover A, 2015, EXP MOL MED, V47, DOI 10.1038/emm.2014.117

Clements CM, 2006, P NATL ACAD SCI USA, V103, P15091, DOI 10.1073/pnas.0607260103

Cook AL, 2011, PLOS ONE, V6, DOI 10.1371/journal.pone.0021907

Cookson MR, 2010, MOVEMENT DISORD, V25, pS44, DOI 10.1002/mds.22713

Cuadrado A, 2019, NAT REV DRUG DISCOV, V18, P295, DOI 10.1038/s41573-018-0008-x

Cuadrado A, 2009, EXPERT OPIN THER TAR, V13, P319, DOI [10.1517/13543780802716501 , 10.1517/13543780802716501]

DEXTER DT, 1989, J NEUROCHEM, V52, P381, DOI 10.1111/j.1471-4159.1989.tb09133.x

Di Filippo M, 2010, J ALZHEIMERS DIS, V20, pS369, DOI 10.3233/JAD-2010-100543

Dias V, 2013, J PARKINSON DIS, V3, P461, DOI 10.3233/JPD-130230

Erejuwa OO, 2013, OXID MED CELL LONGEV, V2013, DOI 10.1155/2013/931251

Foresti R, 2013, PHARMACOL RES, V76, P132, DOI 10.1016/j.phrs.2013.07.010

Fragoulis A, 2012, ARTHRITIS RES THER, V14, DOI 10.1186/ar4059

Fulop GA, 2018, GEROSCIENCE, V40, P513, DOI 10.1007/s11357-018-0047-6

Gold R, 2012, CLIN IMMUNOL, V142, P44, DOI 10.1016/j.clim.2011.02.017

GRIFFIN WST, 1989, P NATL ACAD SCI USA, V86, P7611

Halle A, 2008, NAT IMMUNOL, V9, P857, DOI 10.1038/ni.1636

Hara T, 2006, NATURE, V441, P885, DOI 10.1038/nature04724

Hattingen E, 2009, BRAIN, V132, P3285, DOI 10.1093/brain/awp293

Hayes JD, 2010, ANTIOXID REDOX SIGN, V13, P1713, DOI 10.1089/ars.2010.3221

Hayes JD, 2009, TRENDS BIOCHEM SCI, V34, P176, DOI 10.1016/j.tibs.2008.12.008

Heiss EH, 2013, REDOX BIOL, V1, P359, DOI 10.1016/j.redox.2013.06.001

Heneka MT, 2015, NAT IMMUNOL, V16, P229, DOI 10.1038/ni.3102

Hoenen C, 2016, PLOS ONE, V11, DOI 10.1371/journal.pone.0162717

Hubbs AF, 2007, AM J PATHOL, V170, P2068, DOI 10.2353/ajpath.2007.060898

Ichimura Y, 2013, MOL CELL, V51, P618, DOI 10.1016/j.molcel.2013.08.003

Ikeyama S, 2002, FASEB J, V16, pA1163, DOI 10.1096/fj.01-0409fje

Im JY, 2012, HUM MOL GENET, V21, P3013, DOI 10.1093/hmg/dds131

Inoue K, 2012, MOL NEURODEGENER, V7, DOI 10.1186/1750-1326-7-48

Itoh K, 1999, GENE DEV, V13, P76, DOI 10.1101/gad.13.1.76

Jain AK, 2007, J BIOL CHEM, V282, P16502, DOI 10.1074/jbc.M611336200

Jarrott B, 2016, NEUROCHEM RES, V41, P523, DOI 10.1007/s11064-015-1661-7

Jazwa A, 2011, ANTIOXID REDOX SIGN, V14, P2347, DOI 10.1089/ars.2010.3731

Jiang DL, 2007, BIOCHEMISTRY-US, V46, P9270, DOI 10.1021/bi700508n

Jo C, 2014, NAT COMMUN, V5, DOI 10.1038/ncomms4496

Johnson JA, 2008, ANN NY ACAD SCI, V1147, P61, DOI 10.1196/annals.1427.036

Joshi G, 2015, NEUROBIOL AGING, V36, P664, DOI 10.1016/j.neurobiolaging.2014.09.004

Kaidery NA, 2013, ANTIOXID REDOX SIGN, V18, P139, DOI 10.1089/ars.2011.4491

Kärkkäinen V, 2014, STEM CELLS, V32, P1904, DOI 10.1002/stem.1666

Kerr JS, 2017, TRENDS NEUROSCI, V40, P151, DOI 10.1016/j.tins.2017.01.002

Keum YS, 2006, CANCER RES, V66, P8804, DOI 10.1158/0008-5472.CAN-05-3513

Keum YS, 2014, MOLECULES, V19, P10074, DOI 10.3390/molecules190710074

Khan A, 2018, FRONT PHARMACOL, V9, DOI 10.3389/fphar.2018.01383

Kim SK, 2008, FREE RADICAL BIO MED, V45, P537, DOI 10.1016/j.freeradbiomed.2008.05.011

Kim S, 2020, NEUROPHARMACOLOGY, V167, DOI 10.1016/j.neuropharm.2020.107989

Kobayashi EH, 2016, NAT COMMUN, V7, DOI 10.1038/ncomms11624

Komatsu M, 2010, NAT CELL BIOL, V12, P213, DOI 10.1038/ncb2021

Korczyn AD., 2001, J Neurol, V248, pIII1, DOI DOI 10.1007/PL00022916

Kraft AD, 2004, J NEUROSCI, V24, P1101, DOI 10.1523/JNEUROSCI.3817-03.2004

Kuter K, 2018, MOL NEUROBIOL, V55, P3049, DOI 10.1007/s12035-017-0529-z

Kwok JBJ, 2005, ANN NEUROL, V58, P829, DOI 10.1002/ana.20691

L'Episcopo F, 2011, NEUROBIOL DIS, V41, P508, DOI 10.1016/j.nbd.2010.10.023

L'Episcopo F, 2018, STEM CELLS, V36, P1179, DOI 10.1002/stem.2827

L'Episcopo F, 2013, J NEUROSCI, V33, P1462, DOI 10.1523/JNEUROSCI.3206-12.2013

Lastres-Becker I, 2016, ANTIOXID REDOX SIGN, V25, P61, DOI 10.1089/ars.2015.6549

Lastres-Becker I, 2014, BRAIN, V137, P78, DOI 10.1093/brain/awt323

Lee JM, 2003, J BIOL CHEM, V278, P12029, DOI 10.1074/jbc.M211558200

Lev N, 2015, PLOS ONE, V10, DOI 10.1371/journal.pone.0127549

Li CZ, 2017, NAT REV DRUG DISCOV, V16, P863, DOI 10.1038/nrd.2017.155

Licht-Murava A, 2016, SCI SIGNAL, V9, DOI 10.1126/scisignal.aah7102

Liddell JR, 2017, ANTIOXIDANTS-BASEL, V6, DOI 10.3390/antiox6030065

Liddelow SA, 2017, NATURE, V541, P481, DOI 10.1038/nature21029

Lim JH, 2008, PHARMACOL RES, V57, P325, DOI 10.1016/j.phrs.2008.03.004

Lin MS, 2015, J PHARMACOL SCI, V127, P203, DOI 10.1016/j.jphs.2014.12.010

Linker RA, 2011, BRAIN, V134, P678, DOI 10.1093/brain/awq386

Liu GH, 2008, BBA-MOL CELL RES, V1783, P713, DOI 10.1016/j.bbamcr.2008.01.002

Lok K, 2013, NEUROSCI LETT, V557, P84, DOI 10.1016/j.neulet.2013.10.051

Lu MC, 2016, MED RES REV, V36, P924, DOI 10.1002/med.21396

Mahul-Mellier AL, 2020, P NATL ACAD SCI USA, V117, P4971, DOI 10.1073/pnas.1913904117

Mao L, 2010, MEDIAT INFLAMM, V2010, DOI 10.1155/2010/238321

Marcotte D, 2013, BIOORGAN MED CHEM, V21, P4011, DOI 10.1016/j.bmc.2013.04.019

Martelli D, 2014, AUTON NEUROSCI-BASIC, V182, P65, DOI 10.1016/j.autneu.2013.12.007

Martin D, 2004, J BIOL CHEM, V279, P8919, DOI 10.1074/jbc.M309660200

Matigian N, 2010, DIS MODEL MECH, V3, P785, DOI 10.1242/dmm.005447

Miller DB, 2015, METABOLISM, V64, pS40, DOI 10.1016/j.metabol.2014.10.030

Moehle MS, 2015, NEUROSCIENCE, V302, P59, DOI 10.1016/j.neuroscience.2014.11.018

MOGI M, 1994, NEUROSCI LETT, V165, P208, DOI 10.1016/0304-3940(94)90746-3

Mogi M, 1999, NEUROSCI LETT, V268, P101, DOI 10.1016/S0304-3940(99)00388-2

Moon HE, 2015, EXP NEUROBIOL, V24, P103, DOI 10.5607/en.2015.24.2.103

Morroni F, 2013, NEUROTOXICOLOGY, V36, P63, DOI 10.1016/j.neuro.2013.03.004

Mota SI, 2015, BBA-MOL BASIS DIS, V1852, P1428, DOI 10.1016/j.bbadis.2015.03.015

Müftüoglu M, 2004, MOVEMENT DISORD, V19, P544, DOI 10.1002/mds.10695

Nakabeppu Y, 2007, J NEUROSCI RES, V85, P919, DOI 10.1002/jnr.21191

Nakaso K, 2003, FEBS LETT, V546, P181, DOI 10.1016/S0014-5793(03)00517-9

Nioi P, 2005, MOL CELL BIOL, V25, P10895, DOI 10.1128/MCB.25.24.10895-10906.2005

Numazawa S, 2003, AM J PHYSIOL-CELL PH, V285, pC334, DOI 10.1152/ajpcell.00043.2003

Ogura T, 2010, P NATL ACAD SCI USA, V107, P2842, DOI 10.1073/pnas.0914036107

Okamoto M, 2011, FASEB J, V25, P3570, DOI 10.1096/fj.11-184697

Oksanen M, 2020, GLIA, V68, P589, DOI 10.1002/glia.23741

Oksanen M, 2017, STEM CELL REP, V9, P1885, DOI 10.1016/j.stemcr.2017.10.016

Pajares M, 2020, CELLS-BASEL, V9, DOI 10.3390/cells9071687

Pajares M, 2018, AUTOPHAGY, V14, P1310, DOI 10.1080/15548627.2018.1474992

Pajares M, 2016, AUTOPHAGY, V12, P1902, DOI 10.1080/15548627.2016.1208889

Pan H, 2011, NEUROCHEM RES, V36, P2434, DOI 10.1007/s11064-011-0571-6

PAPPOLLA MA, 1992, AM J PATHOL, V140, P621

Parada E, 2014, J PINEAL RES, V56, P204, DOI 10.1111/jpi.12113

Park SY, 2013, INT IMMUNOPHARMACOL, V17, P415, DOI 10.1016/j.intimp.2013.06.032

Pedersen WA, 1998, ANN NEUROL, V44, P819, DOI 10.1002/ana.410440518

Pi JB, 2007, FREE RADICAL BIO MED, V42, P1797, DOI 10.1016/j.freeradbiomed.2007.03.001

Prasad KN, 2017, MECH AGEING DEV, V162, P63, DOI 10.1016/j.mad.2016.12.003

Rachakonda G, 2008, CHEM RES TOXICOL, V21, P705, DOI 10.1021/tx700302s

Rada P, 2012, MOL CELL BIOL, V32, P3486, DOI 10.1128/MCB.00180-12

Rada P, 2011, MOL CELL BIOL, V31, P1121, DOI 10.1128/MCB.01204-10

Ramsey CP, 2007, J NEUROPATH EXP NEUR, V66, P75, DOI 10.1097/nen.0b013e31802d6da9

Ren P, 2020, OXID MED CELL LONGEV, V2020, DOI 10.1155/2020/3050971

Riley BE, 2011, AUTOPHAGY, V7, P338, DOI 10.4161/auto.7.3.14780

Rojo AI, 2008, J NEUROCHEM, V105, P192, DOI 10.1111/j.1471-4159.2007.05124.x

Rojo AI, 2018, REDOX BIOL, V18, P173, DOI 10.1016/j.redox.2018.07.006

Rojo AI, 2017, REDOX BIOL, V13, P444, DOI 10.1016/j.redox.2017.07.006

Rojo AI, 2010, GLIA, V58, P588, DOI 10.1002/glia.20947

Ross D, 2017, FRONT PHYSIOL, V8, DOI 10.3389/fphys.2017.00595

Salazar M, 2006, J BIOL CHEM, V281, P14841, DOI 10.1074/jbc.M513737200

SantaCruz KS, 2004, NEUROBIOL AGING, V25, P63, DOI 10.1016/S0197-4580(03)00117-9

Sarlus H, 2017, J CLIN INVEST, V127, P3240, DOI 10.1172/JCI90606

Sayre LM, 2001, CURR MED CHEM, V8, P721, DOI 10.2174/0929867013372922

SCHAPIRA AHV, 1989, LANCET, V1, P1269, DOI 10.1016/S0140-6736(89)92366-0

SCHAPIRA AHV, 1990, J NEUROCHEM, V54, P823, DOI 10.1111/j.1471-4159.1990.tb02325.x

Schapira AHV, 2008, LANCET NEUROL, V7, P97, DOI 10.1016/S1474-4422(07)70327-7

Selley ML, 2002, NEUROBIOL AGING, V23, P383, DOI 10.1016/S0197-4580(01)00327-X

Serapide MF, 2020, FRONT AGING NEUROSCI, V12, DOI 10.3389/fnagi.2020.00024

Shi XW, 2011, J INVEST DERMATOL, V131, P2401, DOI 10.1038/jid.2011.222

Shi Y, 2018, NAT REV IMMUNOL, V18, P759, DOI 10.1038/s41577-018-0051-1

Shih HC, 2003, ANTICANCER RES, V23, P3749

Shih RH, 2015, FRONT MOL NEUROSCI, V8, DOI 10.3389/fnmol.2015.00077

Silva-Palacios A, 2018, AGEING RES REV, V47, P31, DOI 10.1016/j.arr.2018.06.003

Simard AR, 2006, MOL PSYCHIATR, V11, P327, DOI 10.1038/sj.mp.4001809

Sofroniew MV, 2010, ACTA NEUROPATHOL, V119, P7, DOI 10.1007/s00401-009-0619-8

Starhof C, 2018, J NEUROINFLAMM, V15, DOI 10.1186/s12974-018-1339-6

Stephenson J, 2018, IMMUNOLOGY, V154, P204, DOI 10.1111/imm.12922

Subramaniam SR, 2017, FRONT AGING NEUROSCI, V9, DOI 10.3389/fnagi.2017.00176

Sultana R, 2013, FREE RADICAL BIO MED, V62, P157, DOI 10.1016/j.freeradbiomed.2012.09.027

Sun Z, 2009, MOL CELL BIOL, V29, P2658, DOI 10.1128/MCB.01639-08

Suzuki T, 2015, FREE RADICAL BIO MED, V88, P93, DOI 10.1016/j.freeradbiomed.2015.06.006

Tang MP, 2018, NEUROBIOL AGING, V63, P128, DOI 10.1016/j.neurobiolaging.2017.12.001

Tarantini S, 2018, J GERONTOL A-BIOL, V73, P853, DOI 10.1093/gerona/glx177

Theodore M, 2008, J BIOL CHEM, V283, P8984, DOI 10.1074/jbc.M709040200

Trimmer PA, 2000, EXP NEUROL, V162, P37, DOI 10.1006/exnr.2000.7333

Trinh K, 2008, J NEUROSCI, V28, P465, DOI 10.1523/JNEUROSCI.4778-07.2008

Tysnes OB, 2017, J NEURAL TRANSM, V124, P901, DOI 10.1007/s00702-017-1686-y

van Muiswinkel FL, 2004, NEUROBIOL AGING, V25, P1253, DOI 10.1016/j.neurobiolaging.2003.12.010

Vranová HP, 2014, J NEUROL SCI, V343, P120, DOI 10.1016/j.jns.2014.05.052

Wakabayashi N, 2003, NAT GENET, V35, P238, DOI 10.1038/ng1248

Wang HY, 2013, CANCER RES, V73, P3097, DOI 10.1158/0008-5472.CAN-12-3386

Wei ZX, 2018, FRONT MOL NEUROSCI, V11, DOI 10.3389/fnmol.2018.00236

Wu Q, 2014, MAR DRUGS, V12, P6125, DOI 10.3390/md12126125

Wunder Christian, 2003, Current Drug Targets - Cardiovascular & Haematological Disorders, V3, P199

Yan W, 2008, NEUROSCI LETT, V431, P150, DOI 10.1016/j.neulet.2007.11.060

Yang LC, 2009, PLOS ONE, V4, DOI 10.1371/journal.pone.0005757

Yoo MS, 2003, MOL BRAIN RES, V110, P76, DOI 10.1016/S0169-328X(02)00586-7

Youdim MBH, 2004, ANN NY ACAD SCI, V1012, P306, DOI 10.1196/annals.1306.025

Yu II, 2019, Journal of Immunology, V202, P185, DOI DOI 10.1002/JCP.27185

Zhang HQ, 2015, FREE RADICAL BIO MED, V88, P314, DOI 10.1016/j.freeradbiomed.2015.05.036

Zhang J, 2010, CYTOKINE, V52, P252, DOI 10.1016/j.cyto.2010.08.011

Zhang M, 2019, EXP THER MED, V18, P467, DOI 10.3892/etm.2019.7632

Zhang XX, 2021, JPAD-J PREV ALZHEIM, V8, P313, DOI 10.14283/jpad.2021.15

Zhang ZJ, 2011, INT J MOL MED, V27, P195, DOI 10.3892/ijmm.2010.571

Zhou WB, 2005, J BIOL CHEM, V280, P43150, DOI 10.1074/jbc.M507124200

Zhou Y, 2019, FRONT NEUROSCI-SWITZ, V13, DOI 10.3389/fnins.2019.00852

Zipper LM, 2002, J BIOL CHEM, V277, P36544, DOI 10.1074/jbc.M206530200

NR 180

TC 69

Z9 71

U1 12

U2 81

PU FRONTIERS MEDIA SA

PI LAUSANNE

PA AVENUE DU TRIBUNAL FEDERAL 34, LAUSANNE, CH-1015, SWITZERLAND

EI 1662-5102

J9 FRONT CELL NEUROSCI

JI Front. Cell. Neurosci.

PD JAN 21

PY 2022

VL 15

AR 787258

DI 10.3389/fncel.2021.787258

PG 15

WC Neurosciences

WE Science Citation Index Expanded (SCI-EXPANDED)

SC Neurosciences & Neurology

GA YT6CY

UT WOS:000751446700001

PM 35126058

OA gold, Green Published

DA 2024-08-12

ER

PT J

AU Rehman, K

Irshad, K

Kamal, S

Imran, I

Akash, MSH

AF Rehman, Kanwal

Irshad, Kanwal

Kamal, Shagufta

Imran, Imran

Akash, Muhammad Sajid Hamid

TI Exposure of Environmental Contaminants and Development of Neurological

Disorders

SO CRITICAL REVIEWS IN EUKARYOTIC GENE EXPRESSION

LA English

DT Article

DE air pollutants; heavy metals; pesticides; neuroinflammation;

neurological diseases

ID OXYGEN SPECIES GENERATION; OXIDATIVE STRESS; HEAVY-METALS; ULTRAFINE

PARTICLES; parkinson's disease; PARTICULATE MATTER; alzheimer's disease;

INSTILLED FINE; AIR-POLLUTION; BRAIN

AB In this era of technology, neurological disorders are the most prevalent disorders in growing population. Alzheimer's and Parkinson's diseases are the most common neurological disorders which are manifested by any abnormality in the structure and functions of neurons present in brain and spinal cord. Exposure to environmental pollution is a serious issue which is associated with high morbidity and mortality rate in the worldwide. Air pollutants are the major contributors to induce the inflammation in lungs and brain which ultimately impairs the normal functioning of CNS. Air pollution persuades CNS pathology by inducing the oxidative stress, activation of microglial cells, neuroinflammation and alteration in permeability of blood brain barrier. Similarly, exposure of heavy metals also exhibits the major and long-lasting effects on brain and causes cognitive dysfunction. Likewise, pesticides have also major influence on the etiology of neurological disorders. Pesticides such as paraquat and rotenone are involved in the pathogenesis of parkinson's disease. Treatment strategy for environmental pollutants -induced neurological disorders is a challenging task because conventional therapeutics are effective but do not have optimum therapeutic efficacy against such type of disorders. This article addresses how the environmental pollutants are involved in the pathogenesis of neurological disorders and treatment strategies to reduce the occurrence of neurological disorders.

C1 [Rehman, Kanwal] Univ Agr Faisalabad, Dept Pharm, Faisalabad, Pakistan.

[Irshad, Kanwal; Akash, Muhammad Sajid Hamid] Govt Coll Univ Faisalabad, Dept Pharmaceut Chem, Faisalabad, Pakistan.

[Kamal, Shagufta] Govt Coll Univ Faisalabad, Dept Biochem, Faisalabad, Pakistan.

[Imran, Imran] Bahauddin Zakariya Univ, Dept Pharmacol, Multan, Pakistan.

C3 University of Agriculture Faisalabad; Government College University

Faisalabad; Government College University Faisalabad; Bahauddin Zakariya

University

RP Akash, MSH (corresponding author), Govt Coll Univ Faisalabad, Dept Pharmaceut Chem, Faisalabad, Pakistan.

EM sajidakash@gmail.com

RI Imran, Imran/AAL-5734-2020; Rehman, Kanwal/P-7727-2014; Akash, Muhammad

Sajid Hamid/C-3477-2018

OI Imran, Imran/0000-0003-1337-8574; Rehman, Kanwal/0000-0001-7873-6681;

Akash, Muhammad Sajid Hamid/0000-0002-9446-5233; Irshad,

Kanwal/0000-0003-1903-4502

FU Higher Education Commission (HEC) of Pakistan

[5661/Punjab/NRPU/RD/HEC/2016, 6429/Punjab/NRPU/RD/HEC/2016,

8365/Punjab/NRPU/RD/HEC/2017]

FX This work was supported by research grants

(5661/Punjab/NRPU/R&D/HEC/2016, 6429/Punjab/NRPU/R&D/HEC/2016, and

8365/Punjab/NRPU/R&D/HEC/2017) received from Higher Education Commission

(HEC) of Pakistan.

CR Abdollahi M, 2004, MED SCI MONITOR, V10, pRA141

Ahmad T, 2004, P 30 WEDC INT C LAO

Alshuaib WB, 1996, J EXP BIOL, V199, P537

[Anonymous], 2018, LANCET NEUROL, V17, P103, DOI 10.1016/S1474-4422(17)30462-3

[Anonymous], 1933, NATURE, V132, P167, DOI [10.1038/132167b0, DOI 10.1038/132167B0]

[Anonymous], 1980, International classification of impairments, disabilities, and handicaps

Antonio MT, 2003, TOXICOL LETT, V143, P331, DOI 10.1016/S0378-4274(03)00194-2

Anyanwu BO, 2018, TOXICS, V6, DOI 10.3390/toxics6040065

Arif IA, 2010, TOXICOL IND HEALTH, V26, P121, DOI 10.1177/0748233710362382

Aschner M, 2007, TOXICOL APPL PHARM, V221, P131, DOI 10.1016/j.taap.2007.03.001

Bachurin S, 2001, ANN NY ACAD SCI, V939, P425

Banks WA, 2010, BRAIN BEHAV IMMUN, V24, P102, DOI 10.1016/j.bbi.2009.09.001

Bhave A, 2011, NEUROEPIDEMIOLOGY, V37, P177, DOI 10.1159/000331873

Block ML, 2009, TRENDS NEUROSCI, V32, P506, DOI 10.1016/j.tins.2009.05.009

BONITHONKOPP C, 1986, BIOL RES PREG PERIN, V7, P37

Braak H, 2011, ACTA NEUROPATHOL, V121, P171, DOI 10.1007/s00401-010-0789-4

Branca JJV, 2018, NEURAL REGEN RES, V13, P1879, DOI 10.4103/1673-5374.239434

BROOKES N, 1989, J NEUROCHEM, V53, P1228, DOI 10.1111/j.1471-4159.1989.tb07419.x

Brown TP, 2006, ENVIRON HEALTH PERSP, V114, P156, DOI 10.1289/ehp.8095

Caito Samuel, 2015, Handb Clin Neurol, V131, P169, DOI 10.1016/B978-0-444-62627-1.00011-1

Calderón-Garcidueñas L, 2008, BRAIN COGNITION, V68, P117, DOI 10.1016/j.bandc.2008.04.008

Calderón-Garcidueñas L, 2017, ENVIRON RES, V158, P324, DOI 10.1016/j.envres.2017.06.026

Calderón-Garcidueñas L, 2015, J ALZHEIMERS DIS, V43, P1039, DOI 10.3233/JAD-141365

Calderón-Garcidueñas L, 2010, EXP TOXICOL PATHOL, V62, P91, DOI 10.1016/j.etp.2009.02.117

Calvert GM, 2004, AM J IND MED, V45, P14, DOI 10.1002/ajim.10309

Campbell A, 2005, NEUROTOXICOLOGY, V26, P133, DOI 10.1016/j.neuro.2004.08.003

Cannon JR, 2011, TOXICOL SCI, V124, P225, DOI 10.1093/toxsci/kfr239

Chesser AS, 2013, FRONT NEUROL, V4, DOI 10.3389/fneur.2013.00122

Clarkson TW, 2006, CRIT REV TOXICOL, V36, P609, DOI 10.1080/10408440600845619

Clarkson TW, 2003, NEW ENGL J MED, V349, P1731, DOI 10.1056/NEJMra022471

Costa LG, 1998, TOXICOL LETT, V103, P417, DOI 10.1016/S0378-4274(98)00248-3

Costa LG, 2017, NEUROTOXICOLOGY, V59, P133, DOI 10.1016/j.neuro.2015.11.008

Craig L, 2008, J TOXICOL ENV HEAL A, V71, P588, DOI 10.1080/15287390801997732

Cramer PE, 2012, SCIENCE, V335, P1503, DOI 10.1126/science.1217697

Cui ZR, 2005, EUR J PHARM BIOPHARM, V59, P263, DOI 10.1016/j.ejpb.2004.07.009

Cunningham C, 2009, BIOL PSYCHIAT, V65, P304, DOI 10.1016/j.biopsych.2008.07.024

D'Angiulli A, 2018, FRONT PUBLIC HEALTH, V6, DOI 10.3389/fpubh.2018.00095

Dantzer R, 2008, NAT REV NEUROSCI, V9, P46, DOI 10.1038/nrn2297

de Joode BV, 2001, LANCET, V357, P1014, DOI 10.1016/S0140-6736(00)04249-5

Domico LM, 2007, NEUROTOXICOLOGY, V28, P1079, DOI 10.1016/j.neuro.2007.04.008

Durczok A, 2005, POL J ENVIRON STUD, V14, P569

Farina M, 2011, LIFE SCI, V89, P555, DOI 10.1016/j.lfs.2011.05.019

Felix K, 2005, J BIOCHEM MOL TOXIC, V19, P67, DOI 10.1002/jbt.20062

Fleisher AS, 2008, ARCH NEUROL-CHICAGO, V65, P1031, DOI 10.1001/archneur.65.8.1031

Furuyama A, 2009, ARCH TOXICOL, V83, P429, DOI 10.1007/s00204-008-0371-1

Ortiz GG, 2016, FREE RADICALS AND DISEASES, P151, DOI 10.5772/63794

Garcia GJM, 2015, INHAL TOXICOL, V27, P394, DOI 10.3109/08958378.2015.1066904

Giasson BI, 2002, BIOCHEMISTRY-US, V41, P15376, DOI 10.1021/bi026813c

Guerra R, 2013, TOXICOL LETT, V222, P146, DOI 10.1016/j.toxlet.2013.07.012

Hahad O, 2020, INT J MOL SCI, V21, DOI 10.3390/ijms21124306

Hunter C, 1999, NEUROLOGY, V52, P271

Illum L, 2004, J PHARM PHARMACOL, V56, P3, DOI 10.1211/0022357022539

Insel TR, 2008, AM J PSYCHIAT, V165, P663, DOI 10.1176/appi.ajp.2008.08030366

Iqbal K, 2010, CURR ALZHEIMER RES, V7, P656, DOI 10.2174/156720510793611592

Jan AT, 2015, INT J MOL SCI, V16, P29592, DOI 10.3390/ijms161226183

Johansson C, 2007, NEUROTOX RES, V11, P241, DOI 10.1007/BF03033570

Kakkar P, 2005, ENVIRON TOXICOL PHAR, V19, P335, DOI 10.1016/j.etap.2004.09.003

Kanwar JR, 2012, INT J NANOMED, V7, P3259, DOI 10.2147/IJN.S30919

Karlsborg M, 2010, DAN MED BULL, V57

Kasdagli MI, 2019, INT J HYG ENVIR HEAL, V222, P402, DOI 10.1016/j.ijheh.2018.12.006

Keifer MC, 1997, OCCUP MED, V12, P291

Kim Y, 2013, NEUROTOXICOLOGY, V35, P15, DOI 10.1016/j.neuro.2012.11.006

Kirkham PA, 2013, CHEST, V144, P266, DOI 10.1378/chest.12-2664

KORPELA H, 1986, AM J OBSTET GYNECOL, V155, P1086, DOI 10.1016/0002-9378(86)90356-X

Kovacs GG, 2016, INT J MOL SCI, V17, DOI 10.3390/ijms17020189

LAFONCAZAL M, 1993, NATURE, V364, P535, DOI 10.1038/364535a0

Lawal HO, 2010, NEUROBIOL DIS, V40, P102, DOI 10.1016/j.nbd.2010.05.008

Le Couteur DG, 1999, BIOMED PHARMACOTHER, V53, P122, DOI 10.1016/S0753-3322(99)80077-8

Levine B, 2008, CELL, V132, P27, DOI 10.1016/j.cell.2007.12.018

Levine B, 2011, NATURE, V469, P323, DOI 10.1038/nature09782

Liazoghli D, 2005, AM J PATHOL, V166, P1499, DOI 10.1016/S0002-9440(10)62366-8

Liou HH, 2001, TOXICOL APPL PHARM, V172, P37, DOI 10.1006/taap.2001.9130

Lleó A, 2006, ANNU REV MED, V57, P513, DOI 10.1146/annurev.med.57.121304.131442

López E, 2006, FREE RADICAL BIO MED, V40, P940, DOI 10.1016/j.freeradbiomed.2005.10.062

Lucchini RG, 2012, NEUROTOXICOLOGY, V33, P838, DOI 10.1016/j.neuro.2011.12.001

Luo Y, 2002, P NATL ACAD SCI USA, V99, P12197, DOI 10.1073/pnas.182425199

Mazzoli-Rocha F, 2010, CELL BIOL TOXICOL, V26, P481, DOI 10.1007/s10565-010-9158-2

McBurney JW, 2017, INTEGRATIVE ENV MED

Méndez-Armenta M, 2007, ENVIRON TOXICOL PHAR, V23, P350, DOI 10.1016/j.etap.2006.11.009

Meng T, 2019, CELL STRESS, V3, P141, DOI 10.15698/cst2019.05.186

Mills NL, 2009, NAT CLIN PRACT CARD, V6, P36, DOI 10.1038/ncpcardio1399

Mizumura K, 2016, RESPIR INVESTIG, V54, P407, DOI 10.1016/j.resinv.2016.03.008

MIZUNO Y, 1987, J NEUROCHEM, V48, P1787, DOI 10.1111/j.1471-4159.1987.tb05737.x

Mori N, 2007, ARCH TOXICOL, V81, P769, DOI 10.1007/s00204-007-0209-2

MURRAY CJL, 1994, B WORLD HEALTH ORGAN, V72, P481

MURRAY RE, 1974, TOXICOL APPL PHARM, V27, P283, DOI 10.1016/0041-008X(74)90199-9

Nagahara AH, 2009, NAT MED, V15, P331, DOI 10.1038/nm.1912

Nakahira K, 2014, ANTIOXID REDOX SIGN, V20, P474, DOI 10.1089/ars.2013.5373

Namgung U, 2001, TOXICOL APPL PHARM, V174, P130, DOI 10.1006/taap.2001.9200

Neal AP, 2010, MOL NEUROBIOL, V42, P151, DOI 10.1007/s12035-010-8146-0

Nemmar A, 2002, CIRCULATION, V105, P411, DOI 10.1161/hc0402.104118

NICKLAS WJ, 1985, LIFE SCI, V36, P2503, DOI 10.1016/0024-3205(85)90146-8

Oberdörster G, 2009, J NANOSCI NANOTECHNO, V9, P4996, DOI 10.1166/jnn.2009.GR02

Oddo S, 2004, NEURON, V43, P321, DOI 10.1016/j.neuron.2004.07.003

Oerke EC, 2004, CROP PROT, V23, P275, DOI 10.1016/j.cropro.2003.10.001

ORRENIUS S, 1994, J NEURAL TRANSM-SUPP, P1

Pagonabarraga J, 2010, REV NEUROLOGIA, V51, P535, DOI 10.33588/rn.5109.2010528

Patlolla Anita K, 2005, Int J Environ Res Public Health, V2, P80, DOI 10.3390/ijerph2005010080

Power AG, 2010, PHILOS T R SOC B, V365, P2959, DOI 10.1098/rstb.2010.0143

Prakash C, 2016, J APPL TOXICOL, V36, P179, DOI 10.1002/jat.3256

Przedborski S, 2003, J CLIN INVEST, V111, P3, DOI 10.1172/JCI200317522

Radua J, 2018, WORLD PSYCHIATRY, V17, P49, DOI 10.1002/wps.20490

Ramachandiran S, 2007, TOXICOL SCI, V95, P163, DOI 10.1093/toxsci/kfl125

Rao M. V., 2004, Indian Journal of Experimental Biology, V42, P495

Rehman K, 2018, J CELL BIOCHEM, V119, P157, DOI 10.1002/jcb.26234

Reiter RJ, 2010, CURR NEUROPHARMACOL, V8, P194, DOI 10.2174/157015910792246236

Rigon AP, 2008, NEUROTOXICOLOGY, V29, P727, DOI 10.1016/j.neuro.2008.04.017

Rodríguez VM, 2002, NEUROTOXICOL TERATOL, V24, P743, DOI 10.1016/S0892-0362(02)00313-6

Rondi-Reig L, 2001, P NATL ACAD SCI USA, V98, P3543, DOI 10.1073/pnas.041620798

Rosado JL, 2007, ENVIRON HEALTH PERSP, V115, P1371, DOI 10.1289/ehp.9961

Saba S, 2020, CLIN EXP PHARMACOL P, V47, P1682, DOI 10.1111/1440-1681.13353

Sabir S, 2019, BIOMED PHARMACOTHER, V114, DOI 10.1016/j.biopha.2019.108802

Sankhla M S., 2017, Int J Innov Res Sci Eng Technol, V6, P7721, DOI DOI 10.15680/IJIRSET.2017.0605054

SARAFIAN TA, 1993, J NEUROCHEM, V61, P648

SCHEEPERS PTJ, 1992, INT ARCH OCC ENV HEA, V64, P163, DOI 10.1007/BF00380905

Schenk D, 1999, NATURE, V400, P173, DOI 10.1038/22124

Schmidt CW, 2007, ENVIRON HEALTH PERSP, V115, pA404, DOI 10.1289/ehp.115-a404

Schwartz BS, 2000, NEUROLOGY, V55, P1144, DOI 10.1212/WNL.55.8.1144

Semendeferi K, 2001, AM J PHYS ANTHROPOL, V114, P224, DOI 10.1002/1096-8644(200103)114:3<224::AID-AJPA1022>3.0.CO;2-I

Serrano-Pozo A, 2011, CSH PERSPECT MED, V1, DOI 10.1101/cshperspect.a006189

Shaw CA, 2013, IMMUNOL RES, V56, P304, DOI 10.1007/s12026-013-8403-1

Sheu JJ, 2015, J CLIN SLEEP MED, V11, P1403, DOI 10.5664/jcsm.5274

Shults CW, 1999, BIOFACTORS, V9, P267, DOI 10.1002/biof.5520090223

Sisó S, 2010, ACTA NEUROPATHOL, V120, P689, DOI 10.1007/s00401-010-0743-5

Siuly S, 2016, DATA SCI ENG, V1, P54, DOI 10.1007/s41019-016-0011-3

Suwannasual U, 2018, ENVIRON RES, V160, P449, DOI 10.1016/j.envres.2017.10.029

SUZUKI T, 1976, BRIT J IND MED, V33, P88

Teeling JL, 2009, NEUROSCIENCE, V158, P1062, DOI 10.1016/j.neuroscience.2008.07.031

Terzano C, 2010, EUR REV MED PHARMACO, V14, P809

Thrash Bessy, 2007, Proc West Pharmacol Soc, V50, P31

Tysnes OB, 2017, J NEURAL TRANSM, V124, P901, DOI 10.1007/s00702-017-1686-y

van der Mark M, 2012, ENVIRON HEALTH PERSP, V120, P340, DOI 10.1289/ehp.1103881

vanderWerf HMG, 1996, AGR ECOSYST ENVIRON, V60, P81, DOI 10.1016/S0167-8809(96)01096-1

Wang B, 2007, BIOL TRACE ELEM RES, V118, P233, DOI 10.1007/s12011-007-0028-6

Williams DR, 2006, INTERN MED J, V36, P652, DOI 10.1111/j.1445-5994.2006.01153.x

Wolfe DM, 2013, EUR J NEUROSCI, V37, P1949, DOI 10.1111/ejn.12169

Xie ZP, 2008, MOL BIOL CELL, V19, P3290, DOI 10.1091/mbc.E07-12-1292

Yang W, 2007, HUM MOL GENET, V16, P2900, DOI 10.1093/hmg/ddm249

Yu XD, 2011, NEUROTOXICOL TERATOL, V33, P437, DOI 10.1016/j.ntt.2011.05.010

Zhang ZG, 2015, ARCH IMMUNOL THER EX, V63, P333, DOI 10.1007/s00005-015-0351-0

NR 140

TC 8

Z9 8

U1 3

U2 24

PU BEGELL HOUSE INC

PI DANBURY

PA 50 NORTH ST, DANBURY, CT 06810 USA

SN 1045-4403

EI 2162-6502

J9 CRIT REV EUKAR GENE

JI Crit. Rev. Eukaryot. Gene Expr.

PY 2021

VL 31

IS 2

BP 35

EP 54

PG 20

WC Biotechnology & Applied Microbiology; Genetics & Heredity

WE Science Citation Index Expanded (SCI-EXPANDED)

SC Biotechnology & Applied Microbiology; Genetics & Heredity

GA RP8KB

UT WOS:000641970900004

PM 34347978

DA 2024-08-12

ER

PT J

AU Crispino, M

Trinchese, G

Penna, E

Cimmino, F

Catapano, A

Villano, I

Perrone-Capano, C

Mollica, MP

AF Crispino, Marianna

Trinchese, Giovanna

Penna, Eduardo

Cimmino, Fabiano

Catapano, Angela

Villano, Ines

Perrone-Capano, Carla

Mollica, Maria Pina

TI Interplay between Peripheral and Central Inflammation in

Obesity-Promoted Disorders: The Impact on Synaptic Mitochondrial

Functions

SO INTERNATIONAL JOURNAL OF MOLECULAR SCIENCES

LA English

DT Review

DE high fat diet; mitochondria; synaptic plasticity; inflammation;

neuroinflammation; neurological disorders; Alzheimer's disease;

Parkinson's disease; bipolar disorders and schizophrenia

ID HIGH-FAT DIET; LOCAL GENE-EXPRESSION; INSULIN-RESISTANCE; OXIDATIVE

STRESS; KAPPA-B; HYPOTHALAMIC SEROTONIN; COGNITIVE IMPAIRMENT;

alzheimer's disease; PREFRONTAL CORTEX; PROTEIN-SYNTHESIS

AB The metabolic dysfunctions induced by high fat diet (HFD) consumption are not limited to organs involved in energy metabolism but cause also a chronic low-grade systemic inflammation that affects the whole body including the central nervous system. The brain has been considered for a long time to be protected from systemic inflammation by the blood-brain barrier, but more recent data indicated an association between obesity and neurodegeneration. Moreover, obesity-related consequences, such as insulin and leptin resistance, mitochondrial dysfunction and reactive oxygen species (ROS) production, may anticipate and accelerate the physiological aging processes characterized by systemic inflammation and higher susceptibility to neurological disorders. Here, we discussed the link between obesity-related metabolic dysfunctions and neuroinflammation, with particular attention to molecules regulating the interplay between energetic impairment and altered synaptic plasticity, for instance AMP-activated protein kinase (AMPK) and Brain-derived neurotrophic factor (BDNF). The effects of HFD-induced neuroinflammation on neuronal plasticity may be mediated by altered brain mitochondrial functions. Since mitochondria play a key role in synaptic areas, providing energy to support synaptic plasticity and controlling ROS production, the negative effects of HFD may be more pronounced in synapses. In conclusion, it will be emphasized how HFD-induced metabolic alterations, systemic inflammation, oxidative stress, neuroinflammation and impaired brain plasticity are tightly interconnected processes, implicated in the pathogenesis of neurological diseases.

C1 [Crispino, Marianna; Trinchese, Giovanna; Penna, Eduardo; Cimmino, Fabiano; Catapano, Angela; Mollica, Maria Pina] Univ Naples Federico II, Dept Biol, I-80126 Naples, Italy.

[Catapano, Angela; Perrone-Capano, Carla] Univ Naples Federico II, Dept Pharm, I-80131 Naples, Italy.

[Villano, Ines] Univ Campania Luigi Vanvitelli, Dept Expt Med, I-80138 Naples, Italy.

[Perrone-Capano, Carla] Natl Res Council CNR, Inst Genet & Biophys Adriano Buzzati Traverso, I-80131 Naples, Italy.

C3 University of Naples Federico II; University of Naples Federico II;

Universita della Campania Vanvitelli; Consiglio Nazionale delle Ricerche

(CNR); Istituto di Genetica e Biofisica "Adriano Buzzati-Traverso"

(IGB-CNR)

RP Mollica, MP (corresponding author), Univ Naples Federico II, Dept Biol, I-80126 Naples, Italy.

EM crispino@unina.it; giovanna.trinchese@unina.it; eduardo.penna@unina.it;

fabiano.cimmino@unina.it; angelacatapano@me.com;

ines.villano@unicampania.it; perrone@unina.it; mariapia.mollica@unina.it

RI Perrone-Capano, Carla/AAX-3217-2020; Catapano, Angela/GXV-0918-2022;

Giovanna, Trinchese/AAC-5995-2022; Villano, Ines/ABU-5527-2022;

Crispino, Marianna/ABD-3702-2020; CIMMINO, FABIANO/AIA-1152-2022;

Perrone-Capano, Carla/O-7708-2015

OI Catapano, Angela/0000-0002-0429-8348; Giovanna,

Trinchese/0000-0002-4096-6876; Villano, Ines/0000-0002-1755-358X;

CIMMINO, FABIANO/0000-0001-5239-4329; Mollica, Maria

Pina/0000-0003-2726-7779; Perrone-Capano, Carla/0000-0002-6327-9753;

CRISPINO, Marianna/0000-0002-5797-8830

CR Aguer C, 2012, BEST PRACT RES CL EN, V26, P805, DOI 10.1016/j.beem.2012.06.001

Akira S, 2006, CELL, V124, P783, DOI 10.1016/j.cell.2006.02.015

Akira S, 2004, NAT REV IMMUNOL, V4, P499, DOI 10.1038/nri1391

Annunziata C, 2020, FASEB J, V34, P350, DOI 10.1096/fj.201901510RR

Arnold SE, 2014, NEUROBIOL DIS, V67, P79, DOI 10.1016/j.nbd.2014.03.011

Avena NM, 2014, EXP CLIN PSYCHOPHARM, V22, P460, DOI 10.1037/a0037223

Balaskó M, 2013, EXP GERONTOL, V48, P1180, DOI 10.1016/j.exger.2013.07.006

Barzilai N, 2012, DIABETES, V61, P1315, DOI 10.2337/db11-1300

Bertrand RL, 2011, ENDOCRINOLOGY, V152, P36, DOI 10.1210/en.2010-0377

Blennow K, 2006, LANCET, V368, P387, DOI 10.1016/S0140-6736(20)32205-4

BLUNDELL JE, 1986, APPETITE, V7, P39, DOI 10.1016/S0195-6663(86)80051-4

Boitard C, 2014, BRAIN BEHAV IMMUN, V40, P9, DOI 10.1016/j.bbi.2014.03.005

Boulos C, 2019, NUTRIENTS, V11, DOI 10.3390/nu11081896

Nakandakari SCBR, 2019, BRAIN BEHAV IMMUN, V79, P284, DOI 10.1016/j.bbi.2019.02.016

Bray GA, 2004, J CLIN ENDOCR METAB, V89, P2583, DOI 10.1210/jc.2004-0535

Briggs JA, 2015, NEURON, V88, P861, DOI 10.1016/j.neuron.2015.09.045

Cadonic C, 2016, MOL NEUROBIOL, V53, P6078, DOI 10.1007/s12035-015-9515-5

Cavaliere G, 2019, FRONT CELL NEUROSCI, V13, DOI 10.3389/fncel.2019.00509

Cavaliere G, 2018, FRONT PHYSIOL, V9, DOI 10.3389/fphys.2018.00818

Cavaliere G, 2016, PLOS ONE, V11, DOI 10.1371/journal.pone.0149033

Cefaliello C, 2020, MOL NEUROBIOL, V57, P1529, DOI 10.1007/s12035-019-01835-y

Chan DC, 2006, CELL, V125, P1241, DOI 10.1016/j.cell.2006.06.010

Chen C, 2019, BIOLOGY-BASEL, V8, DOI 10.3390/biology8020038

Cheng Y, 2018, FRONT NEUROSCI-SWITZ, V12, DOI 10.3389/fnins.2018.00163

Chinta SJ, 2010, NEUROSCI LETT, V486, P235, DOI 10.1016/j.neulet.2010.09.061

Cioni JM, 2019, CELL, V176, P56, DOI 10.1016/j.cell.2018.11.030

Citri A, 2008, NEUROPSYCHOPHARMACOL, V33, P18, DOI 10.1038/sj.npp.1301559

Clay HB, 2011, INT J DEV NEUROSCI, V29, P311, DOI 10.1016/j.ijdevneu.2010.08.007

Cohen JI, 2011, BMJ OPEN, V1, DOI 10.1136/bmjopen-2011-000175

Contreras A, 2017, NEUROREPORT, V28, P354, DOI 10.1097/WNR.0000000000000774

Cordner ZA, 2015, PHYSIOL BEHAV, V152, P363, DOI 10.1016/j.physbeh.2015.06.008

Crane JD, 2015, NAT MED, V21, P166, DOI 10.1038/nm.3766

Crispino M, 2020, INT J MOL SCI, V21, DOI 10.3390/ijms21020505

Crispino M, 2014, DEV NEUROBIOL, V74, P279, DOI 10.1002/dneu.22109

Crispino M, 2009, RESULTS PROBL CELL D, V48, P243, DOI 10.1007/400_2009_9

Cui YF, 2020, FRONT PHARMACOL, V11, DOI 10.3389/fphar.2020.00102

Currais A, 2015, AGING-US, V7, P937, DOI 10.18632/aging.100838

CURZON G, 1990, ANN NY ACAD SCI, V600, P521, DOI 10.1111/j.1749-6632.1990.tb16907.x

Dalvi PS, 2017, INT J OBESITY, V41, P149, DOI 10.1038/ijo.2016.183

Day EA, 2017, TRENDS ENDOCRIN MET, V28, P545, DOI 10.1016/j.tem.2017.05.004

Di Filippo M, 2010, J ALZHEIMERS DIS, V20, pS369, DOI 10.3233/JAD-2010-100543

Didier S, 2018, INT J MOL SCI, V19, DOI 10.3390/ijms19123716

Donovan MH, 2013, FRONT NEUROSCI-SWITZ, V7, DOI 10.3389/fnins.2013.00036

Dutheil S, 2016, NEUROPSYCHOPHARMACOL, V41, P1874, DOI 10.1038/npp.2015.357

Earls LR, 2014, AGEING RES REV, V17, P34, DOI 10.1016/j.arr.2014.03.004

Eckert GP, 2013, PROSTAG LEUKOTR ESS, V88, P105, DOI 10.1016/j.plefa.2012.05.006

Edelmann Elke, 2017, Front Synaptic Neurosci, V9, P7, DOI 10.3389/fnsyn.2017.00007

Eskelinen MH, 2008, INT J GERIATR PSYCH, V23, P741, DOI 10.1002/gps.1969

Eyman M, 2013, J NEUROSCI RES, V91, P20, DOI 10.1002/jnr.23133

Farrar AM, 2008, NEUROSCIENCE, V152, P321, DOI 10.1016/j.neuroscience.2007.12.034

Fattal O, 2006, PSYCHOSOMATICS, V47, P1, DOI 10.1176/appi.psy.47.1.1

Ferrara E, 2009, J NEUROSCI RES, V87, P1960, DOI 10.1002/jnr.22037

Forny-Germano L, 2019, FRONT NEUROSCI-SWITZ, V12, DOI 10.3389/fnins.2018.01027

Franco-Robles E, 2016, MOLECULES, V21, DOI 10.3390/molecules21080998

Frühbeck G, 2017, SCI REP-UK, V7, DOI 10.1038/s41598-017-06997-0

Gaidhu MP, 2009, J LIPID RES, V50, P704, DOI 10.1194/jlr.M800480-JLR200

Galic S, 2011, J CLIN INVEST, V121, P4903, DOI 10.1172/JCI58577

García-Alcocer G, 2010, NEUROSCI LETT, V486, P171, DOI 10.1016/j.neulet.2010.09.045

García-Escudero V, 2013, OXID MED CELL LONGEV, V2013, DOI 10.1155/2013/162152

Giacobbo BL, 2019, MOL NEUROBIOL, V56, P3295, DOI 10.1007/s12035-018-1283-6

Giuditta A, 2008, PHYSIOL REV, V88, P515, DOI 10.1152/physrev.00051.2006

Grassmann S, 2017, OBESITY, V25, P1776, DOI 10.1002/oby.21945

Grünewald A, 2019, PROG NEUROBIOL, V177, P73, DOI 10.1016/j.pneurobio.2018.09.003

Guillod-Maximin E, 2009, J ENDOCRINOL, V200, P93, DOI 10.1677/JOE-08-0348

Haleem DJ, 2021, NUTR NEUROSCI, V24, P226, DOI 10.1080/1028415X.2019.1619983

Haleem DJ, 2014, BRAIN RES, V1586, P73, DOI 10.1016/j.brainres.2014.06.022

Hao S, 2016, BRAIN BEHAV IMMUN, V51, P230, DOI 10.1016/j.bbi.2015.08.023

Hardie DG, 2014, J INTERN MED, V276, P543, DOI 10.1111/joim.12268

Hassan AM, 2019, NUTR NEUROSCI, V22, P877, DOI 10.1080/1028415X.2018.1465713

Hayden MS, 2008, CELL, V132, P344, DOI 10.1016/j.cell.2008.01.020

Hernández-Aguilera A, 2013, MEDIAT INFLAMM, V2013, DOI 10.1155/2013/135698

Herzig S, 2018, NAT REV MOL CELL BIO, V19, P121, DOI 10.1038/nrm.2017.95

Holt CE, 2019, NAT STRUCT MOL BIOL, V26, P557, DOI 10.1038/s41594-019-0263-5

Horvath TL, 2010, P NATL ACAD SCI USA, V107, P14875, DOI 10.1073/pnas.1004282107

Jackson J, 2019, FRONT NEUROSCI-SWITZ, V13, DOI 10.3389/fnins.2019.00735

Kandimalla R, 2018, HUM MOL GENET, V27, P30, DOI 10.1093/hmg/ddx381

Kanoski SE, 2007, BEHAV BRAIN RES, V182, P57, DOI 10.1016/j.bbr.2007.05.004

Kim HJ, 2011, J PROTEOME RES, V10, P722, DOI 10.1021/pr100892r

Kim JI, 2015, MOL CELL BIOL, V35, P1686, DOI 10.1128/MCB.01321-14

Kohjima M, 2010, ENDOCRINOLOGY, V151, P1532, DOI 10.1210/en.2009-1196

Kowianski Przemyslaw, 2018, Cell Mol Neurobiol, V38, P579, DOI 10.1007/s10571-017-0510-4

Kusminski CM, 2012, NAT MED, V18, P1539, DOI 10.1038/nm.2899

Kwak SH, 2012, OBESITY, V20, P233, DOI 10.1038/oby.2011.253

Labouesse MA, 2017, MOL PSYCHIATR, V22, P961, DOI 10.1038/mp.2016.193

Lama A, 2017, MOL NUTR FOOD RES, V61, DOI 10.1002/mnfr.201600418

Lane CA, 2018, EUR J NEUROL, V25, P59, DOI 10.1111/ene.13439

Larsen SB, 2018, CELL TISSUE RES, V373, P21, DOI 10.1007/s00441-017-2768-8

Layé S, 2010, PROSTAG LEUKOTR ESS, V82, P295, DOI 10.1016/j.plefa.2010.02.006

Leal G, 2014, NEUROPHARMACOLOGY, V76, P639, DOI 10.1016/j.neuropharm.2013.04.005

Leibowitz SF, 1998, BIOL PSYCHIAT, V44, P851, DOI 10.1016/S0006-3223(98)00186-3

LEIBOWITZ SF, 1988, CLIN NEUROPHARMACOL, V11, pS51

Li HZ, 2020, MITOCHONDRION, V53, P38, DOI 10.1016/j.mito.2020.04.012

Lindholm CR, 2013, J PHYSIOL BIOCHEM, V69, P165, DOI 10.1007/s13105-012-0199-2

Lionetti L, 2009, NUTR METAB CARDIOVAS, V19, P146, DOI 10.1016/j.numecd.2008.10.010

Liu Y, 2014, BEHAV BRAIN RES, V267, P178, DOI 10.1016/j.bbr.2014.02.040

Lizarbe B, 2019, FRONT NEUROSCI-SWITZ, V12, DOI 10.3389/fnins.2018.00985

López M, 2018, EUR J CLIN INVEST, V48, DOI 10.1111/eci.12996

Lopez-Esparza S, 2015, INT J DEV NEUROSCI, V42, P80, DOI 10.1016/j.ijdevneu.2015.02.010

Lores-Arnaiz S, 2016, NEUROCHEM RES, V41, P353, DOI 10.1007/s11064-015-1817-5

Ma ZA, 2012, CURR DIABETES REV, V8, P69

Maiti P, 2017, TRANSL NEURODEGENER, V6, DOI 10.1186/s40035-017-0099-z

Majd S, 2018, CURR ALZHEIMER RES, V15, P764, DOI 10.2174/1567205015666180223093020

Mattick JS, 2006, HUM MOL GENET, V15, pR17, DOI 10.1093/hmg/ddl046

McLean FH, 2019, NUTR METAB, V16, DOI 10.1186/s12986-019-0352-9

McLelland GL, 2014, EMBO J, V33, P282, DOI 10.1002/embj.201385902

Mi YS, 2017, FASEB J, V31, P4998, DOI 10.1096/fj.201700400RR

Minokoshi Y, 2004, NATURE, V428, P569, DOI 10.1038/nature02440

Minokoshi Y, 2002, NATURE, V415, P339, DOI 10.1038/415339a

Mlinar B, 2011, CLIN CHEM LAB MED, V49, P1925, DOI 10.1515/CCLM.2011.697

Mollica MP, 2011, NUTR METAB CARDIOVAS, V21, P222, DOI 10.1016/j.numecd.2010.10.012

Mollica MP, 2017, DIABETES, V66, P1405, DOI 10.2337/db16-0924

Murray S, 2019, FRONT NEUROSCI-SWITZ, V13, DOI 10.3389/fnins.2019.01108

Noble EE, 2011, AM J PHYSIOL-REG I, V300, pR1053, DOI 10.1152/ajpregu.00776.2010

Oh CM, 2015, NAT COMMUN, V6, DOI 10.1038/ncomms7794

Onyango IG, 2016, AGING DIS, V7, P201, DOI 10.14336/AD.2015.1007

Pan Q, 2019, METAB BRAIN DIS, V34, P747, DOI 10.1007/s11011-019-0392-x

Parimisetty A, 2016, J NEUROINFLAMM, V13, DOI 10.1186/s12974-016-0530-x

Pasinetti GM, 2008, J NEUROCHEM, V106, P1503, DOI 10.1111/j.1471-4159.2008.05454.x

Paulmann N, 2009, PLOS BIOL, V7, DOI 10.1371/journal.pbio.1000229

Penns E, 2019, FRONT MOL NEUROSCI, V12, DOI 10.3389/fnmol.2019.00195

Pereira C, 2018, INT J NEUROPSYCHOPH, V21, P550, DOI 10.1093/ijnp/pyy018

Pérez LM, 2016, J PHYSIOL-LONDON, V594, P3187, DOI 10.1113/JP271691

Posey KA, 2009, AM J PHYSIOL-ENDOC M, V296, pE1003, DOI 10.1152/ajpendo.90377.2008

Poulos SP, 2010, MOL CELL ENDOCRINOL, V323, P20, DOI 10.1016/j.mce.2009.12.011

Puig KL, 2012, PLOS ONE, V7, DOI 10.1371/journal.pone.0030378

Qu Y, 2019, NEURAL REGEN RES, V14, P2156, DOI 10.4103/1673-5374.262599

Rangaraju V, 2019, CELL, V176, P73, DOI 10.1016/j.cell.2018.12.013

Reddy PH, 2008, TRENDS MOL MED, V14, P45, DOI 10.1016/j.molmed.2007.12.002

Reilly SM, 2017, NAT REV ENDOCRINOL, V13, P633, DOI 10.1038/nrendo.2017.90

Rolo AP, 2012, FREE RADICAL BIO MED, V52, P59, DOI 10.1016/j.freeradbiomed.2011.10.003

Rull A, 2010, MEDIAT INFLAMM, V2010, DOI 10.1155/2010/326580

Sah SK, 2017, BIOCHEM BIOPH RES CO, V493, P731, DOI 10.1016/j.bbrc.2017.08.122

Salminen A, 2011, J MOL MED, V89, P667, DOI 10.1007/s00109-011-0748-0

Salt IP, 2012, EXPERT OPIN INV DRUG, V21, P1155, DOI 10.1517/13543784.2012.696609

Sandoval-Salazar C, 2016, BIOL RES, V49, DOI 10.1186/s40659-016-0075-6

Scarpace PJ, 2000, NEUROPHARMACOLOGY, V39, P1872, DOI 10.1016/S0028-3908(00)00014-9

Sethi JK, 2007, J LIPID RES, V48, P1253, DOI 10.1194/jlr.R700005-JLR200

Shapiro LA, 2011, AGING DIS, V2, P286

Shirihai OS, 2015, CIRC RES, V116, P1835, DOI 10.1161/CIRCRESAHA.116.306374

Simansky KJ, 1996, BEHAV BRAIN RES, V73, P37, DOI 10.1016/0166-4328(96)00066-6

Smalheiser NR, 2014, PHILOS T R SOC B, V369, DOI 10.1098/rstb.2013.0504

Spencer PS, 2015, ENVIRONMENTAL FACTORS IN NEURODEVELOPMENTAL AND NEURODEGENERATIVE DISORDERS, P211, DOI 10.1016/B978-0-12-800228-5.00011-X

Spillane M, 2013, CELL REP, V5, P1564, DOI 10.1016/j.celrep.2013.11.022

Spillantini MG, 1997, NATURE, V388, P839, DOI 10.1038/42166

Srinivasan S, 2008, CELL METAB, V7, P533, DOI 10.1016/j.cmet.2008.04.012

Stein CJ, 2004, J CLIN ENDOCR METAB, V89, P2522, DOI 10.1210/jc.2004-0288

Steinberg GR, 2006, CELL METAB, V4, P465, DOI 10.1016/j.cmet.2006.11.005

Stern JH, 2016, CELL METAB, V23, P770, DOI 10.1016/j.cmet.2016.04.011

Stranahan AM, 2008, HIPPOCAMPUS, V18, P1085, DOI 10.1002/hipo.20470

Subhramanyam CS, 2017, CURR MED CHEM, V24, P1983, DOI 10.2174/0929867324666170124151436

Sumara G, 2012, CELL METAB, V16, P588, DOI 10.1016/j.cmet.2012.09.014

Suzuki H, 1997, INT J VITAM NUTR RES, V67, P272

Swerdlow RH, 2014, BBA-MOL BASIS DIS, V1842, P1219, DOI 10.1016/j.bbadis.2013.09.010

Tang C, 2012, DIABETOLOGIA, V55, P1366, DOI 10.1007/s00125-012-2474-8

Tecott LH, 2007, CELL METAB, V6, P352, DOI 10.1016/j.cmet.2007.09.012

TECOTT LH, 1995, NATURE, V374, P542, DOI 10.1038/374542a0

Thaler JP, 2012, J CLIN INVEST, V122, P153, DOI 10.1172/JCI59660

Todorova V, 2017, CURR NEUROPHARMACOL, V15, P166, DOI 10.2174/1570159X14666160414111821

Trinchese G, 2020, CELLS-BASEL, V9, DOI 10.3390/cells9040823

Tsunekawa T, 2019, ISCIENCE, V20, P337, DOI 10.1016/j.isci.2019.09.032

Valassi E, 2008, NUTR METAB CARDIOVAS, V18, P158, DOI 10.1016/j.numecd.2007.06.004

Van Veldhoven PP, 2010, J LIPID RES, V51, P2863, DOI 10.1194/jlr.R005959

Vinuesa A, 2019, MOL NEUROBIOL, V56, P5075, DOI 10.1007/s12035-018-1435-8

Volpicelli F, 2014, FRONT BEHAV NEUROSCI, V8, DOI 10.3389/fnbeh.2014.00318

Wang BZ, 2019, AGING DIS, V10, P1058, DOI 10.14336/AD.2019.0102

Wang XK, 2012, EXP DIABETES RES, DOI 10.1155/2012/847246

Wang XL, 2014, BBA-MOL BASIS DIS, V1842, P1240, DOI 10.1016/j.bbadis.2013.10.015

Wang ZJ, 2020, NEUROSCIENCE, V424, P24, DOI 10.1016/j.neuroscience.2019.10.050

Watanabe H, 2010, ENDOCRINOLOGY, V151, P4776, DOI 10.1210/en.2009-1349

Wefelmeyer W, 2016, TRENDS NEUROSCI, V39, P656, DOI 10.1016/j.tins.2016.08.004

Wilkins HM, 2016, CURR TOP MED CHEM, V16, P849, DOI 10.2174/1568026615666150827095102

Winocur G, 2005, BEHAV NEUROSCI, V119, P1389, DOI 10.1037/0735-7044.119.5.1389

Wu AG, 2004, EUR J NEUROSCI, V19, P1699, DOI 10.1111/j.1460-9568.2004.03246.x

Wu HL, 2018, METAB BRAIN DIS, V33, P177, DOI 10.1007/s11011-017-0140-z

Xu AW, 2005, PLOS BIOL, V3, P2168, DOI 10.1371/journal.pbio.0030415

Yadav VK, 2011, J EXP MED, V208, P41, DOI 10.1084/jem.20101940

Yadav VK, 2009, CELL, V138, P976, DOI 10.1016/j.cell.2009.06.051

Yagita Y, 2017, J BIOL CHEM, V292, P691, DOI 10.1074/jbc.M116.760090

Yamauchi T, 2002, NAT MED, V8, P1288, DOI 10.1038/nm788

Yang ZG, 2010, J BIOL CHEM, V285, P19051, DOI 10.1074/jbc.M110.123620

Yoon G, 2019, FRONT GENET, V10, DOI 10.3389/fgene.2019.00083

Zemdegs J, 2016, BRIT J PHARMACOL, V173, P2095, DOI 10.1111/bph.13343

Zhang XQ, 2008, CELL, V135, P61, DOI 10.1016/j.cell.2008.07.043

Zhao ZM, 2020, NUTR NEUROSCI, V23, P971, DOI 10.1080/1028415X.2019.1581460

Zorzano A, 2009, INT J BIOCHEM CELL B, V41, P1846, DOI 10.1016/j.biocel.2009.02.004

NR 185

TC 44

Z9 45

U1 1

U2 10

PU MDPI

PI BASEL

PA ST ALBAN-ANLAGE 66, CH-4052 BASEL, SWITZERLAND

EI 1422-0067

J9 INT J MOL SCI

JI Int. J. Mol. Sci.

PD SEP

PY 2020

VL 21

IS 17

AR 5964

DI 10.3390/ijms21175964

PG 22

WC Biochemistry & Molecular Biology; Chemistry, Multidisciplinary

WE Science Citation Index Expanded (SCI-EXPANDED)

SC Biochemistry & Molecular Biology; Chemistry

GA NP7NF

UT WOS:000570359000001

PM 32825115

OA gold, Green Published

DA 2024-08-12

ER

PT J

AU Roohbakhsh, A

Etemad, L

Karimi, G

AF Roohbakhsh, Ali

Etemad, Leila

Karimi, Gholamreza

TI Resolvin D1: A key endogenous inhibitor of neuroinflammation

SO BIOFACTORS

LA English

DT Review

DE AT-RvD1; inflammation; memory; neuroinflammation; pain; resolvin D1;

resolvins; stroke

ID AMYLOID-BETA PHAGOCYTOSIS; LIPID MEDIATORS; MOUSE MODEL; FATTY-ACID;

INFLAMMATORY PAIN; MICROGLIAL CELLS; SPINAL-CORD; RESOLUTION; BRAIN;

ALZHEIMERS

AB After the initiation of inflammation, a series of processes start to resolve the inflammation. A group of endogenous lipid mediators, namely specialized pro-resolving lipid mediators is at the top list of inflammation resolution. Resolvin D1 (RvD1), is one of the lipid mediators with significant anti-inflammatory properties. It is produced from docosahexaenoic acid (omega-3 polyunsaturated fatty acid) in the body. In this article, we aimed to review the most recent findings concerning the pharmacological effects of RvD1 in the central nervous system with a focus on major neurological diseases and dysfunctions. A literature review of the past studies demonstrated that RvD1 plasma level changes during mania, depression, and Parkinson's disease. Furthermore, RVD1 and its epimer, aspirin-triggered RvD1 (AT-RvD1), have significant therapeutic effects on experimental models of ischemic and traumatic brain injuries, memory dysfunction, pain, depression, amyotrophic lateral sclerosis, and Alzheimer's and Parkinson's diseases. Interestingly, the beneficial effects of RvD1 and AT-RvD1 were mostly induced at nanomolar and micromolar concentrations implying the significant potency of these lipid mediators in treating diseases with inflammation.

C1 [Roohbakhsh, Ali; Etemad, Leila; Karimi, Gholamreza] Mashhad Univ Med Sci, Pharmaceut Res Ctr, Pharmaceut Technol Inst, POB 91775-1365, Mashhad, Razavi Khorasan, Iran.

[Karimi, Gholamreza] Mashhad Univ Med Sci, Sch Pharm, Dept Pharmacodynam & Toxicol, Mashhad, Razavi Khorasan, Iran.

C3 Mashhad University Medical Science; Mashhad University Medical Science

RP Karimi, G (corresponding author), Mashhad Univ Med Sci, Pharmaceut Res Ctr, Pharmaceut Technol Inst, POB 91775-1365, Mashhad, Razavi Khorasan, Iran.

EM karimig@mums.ac.ir

RI Etemad, leila/P-2952-2018; karimi, gholamreza/B-8726-2017

FU Mashhad University of Medical Sciences

FX Mashhad University of Medical Sciences

CR Alavi MS, 2019, LIFE SCI, V224, P33, DOI 10.1016/j.lfs.2019.03.045

Alhouayek M, 2014, TRENDS PHARMACOL SCI, V35, P284, DOI 10.1016/j.tips.2014.03.001

Arends S, 2020, PAIN REP, V5, DOI 10.1097/PR9.0000000000000810

Aronowski J, 2011, STROKE, V42, P1781, DOI 10.1161/STROKEAHA.110.596718

Bang S, 2010, BRIT J PHARMACOL, V161, P707, DOI 10.1111/j.1476-5381.2010.00909.x

Bartels T, 2020, SCIENCE, V370, P66, DOI 10.1126/science.abb8587

Basbaum AI, 2009, CELL, V139, P267, DOI 10.1016/j.cell.2009.09.028

Benito-León J, 2019, PLOS ONE, V14, DOI 10.1371/journal.pone.0222505

Bisicchia E, 2019, SEMIN CELL DEV BIOL, V94, P104, DOI 10.1016/j.semcdb.2019.01.011

Bisicchia E, 2018, MOL NEUROBIOL, V55, P6894, DOI 10.1007/s12035-018-0889-z

Chan MMY, 2010, J IMMUNOL, V184, P6418, DOI 10.4049/jimmunol.0903816

Chen C, 2010, NAT CHEM BIOL, V6, P401, DOI 10.1038/nchembio.375

Chen J J, 2020, J Biol Regul Homeost Agents, V34, DOI 10.23812/20-392-A

Chen WT, 2020, SHOCK, V54, P548, DOI 10.1097/SHK.0000000000001528

Cheng JK, 2008, NEUROCHEM RES, V33, P1970, DOI 10.1007/s11064-008-9711-z

Chiurchiù V, 2022, INT J MOL SCI, V23, DOI 10.3390/ijms23116235

Chiurchiù V, 2018, FRONT IMMUNOL, V9, DOI 10.3389/fimmu.2018.00038

Chiurchiù V, 2016, SCI TRANSL MED, V8, DOI 10.1126/scitranslmed.aaf7483

Clauw DJ, 2014, JAMA-J AM MED ASSOC, V311, P1547, DOI 10.1001/jama.2014.3266

Colas RA, 2014, AM J PHYSIOL-CELL PH, V307, pC39, DOI 10.1152/ajpcell.00024.2014

Derungs R, 2017, MOL PSYCHIATR, V22, P605, DOI 10.1038/mp.2016.112

Deyama S, 2017, INT J NEUROPSYCHOPH, V20, P575, DOI 10.1093/ijnp/pyx023

Do KV, 2023, CELL MOL NEUROBIOL, V43, P797, DOI 10.1007/s10571-022-01216-5

Dullemeijer C, 2007, AM J CLIN NUTR, V86, P1479, DOI 10.1093/ajcn/86.5.1479

Erttmann SF, 2019, NAT COMMUN, V10, DOI 10.1038/s41467-019-11169-x

Fiala M, 2015, FASEB J, V29, P2681, DOI 10.1096/fj.14-264218

Fiala M, 2010, J NEUROINFLAMM, V7, DOI 10.1186/1742-2094-7-76

Gilbert Kim, 2015, PharmaNutrition, V3, P78, DOI 10.1016/j.phanu.2015.05.003

Gilbert K, 2014, MAR DRUGS, V12, P5396, DOI 10.3390/md12115396

Gilroy DW, 1999, NAT MED, V5, P698, DOI 10.1038/9550

Green HF, 2019, J PARKINSON DIS, V9, P705, DOI 10.3233/JPD-191699

Groeger AL, 2010, NAT CHEM BIOL, V6, P433, DOI 10.1038/nchembio.367

Harrison JL, 2015, BRAIN BEHAV IMMUN, V47, P131, DOI 10.1016/j.bbi.2015.01.001

Headrick JP, 2017, J MOL CELL CARDIOL, V106, P14, DOI 10.1016/j.yjmcc.2017.03.007

Hickman S, 2018, NAT NEUROSCI, V21, P1359, DOI 10.1038/s41593-018-0242-x

Hopperton KE, 2018, BRAIN BEHAV IMMUN, V69, P74, DOI 10.1016/j.bbi.2017.11.002

Huang FP, 2002, J NEUROSURG, V96, P287, DOI 10.3171/jns.2002.96.2.0287

Huang LA, 2011, PAIN, V152, P557, DOI 10.1016/j.pain.2010.11.021

Hur J, 2018, FASEB J, V32, P4972, DOI 10.1096/fj.201800200R

Ikeda A, 2020, BRAIN RES BULL, V154, P61, DOI 10.1016/j.brainresbull.2019.11.002

Ikonomovic MD, 2008, J HISTOCHEM CYTOCHEM, V56, P1065, DOI 10.1369/jhc.2008.951855

Ishikawa Y, 2017, BEHAV BRAIN RES, V332, P233, DOI 10.1016/j.bbr.2017.06.010

Jiang BC, 2020, PHARMACOL THERAPEUT, V212, DOI 10.1016/j.pharmthera.2020.107581

Jung JS, 2020, KOREAN J PHYSIOL PHA, V24, P165, DOI 10.4196/kjpp.2020.24.2.165

Kendirlioglu BC., 2021, SO CLIN ISTANB EURAS, V32, P273

Kendirlioglu BK, 2020, NORD J PSYCHIAT, V74, P83, DOI 10.1080/08039488.2019.1673480

Khasabova IA, 2020, PROSTAG OTH LIPID M, V151, DOI 10.1016/j.prostaglandins.2020.106479

Kim JH, 2017, WORLD NEUROSURG, V102, P56, DOI 10.1016/j.wneu.2017.02.105

Kinney Jefferson W, 2018, Alzheimers Dement (N Y), V4, P575, DOI 10.1016/j.trci.2018.06.014

Kitsis P, 2022, LIFE-BASEL, V12, DOI 10.3390/life12020314

Klein CP, 2014, NEUROPHARMACOLOGY, V86, P57, DOI 10.1016/j.neuropharm.2014.05.043

Knott C, 2000, MOL CELL NEUROSCI, V16, P724, DOI 10.1006/mcne.2000.0914

Kohli P, 2009, BRIT J PHARMACOL, V158, P960, DOI 10.1111/j.1476-5381.2009.00290.x

Kooij G, 2020, HAEMATOLOGICA, V105, P2056, DOI 10.3324/haematol.2019.219519

Korn T, 2009, ANNU REV IMMUNOL, V27, P485, DOI 10.1146/annurev.immunol.021908.132710

Kotlega D, 2021, PROSTAG OTH LIPID M, V156, DOI [10.1016/j.prostaglandins.2021.106576, 10.1016/j.prostaglins.2021.106576]

Krashia P, 2019, NAT COMMUN, V10, DOI 10.1038/s41467-019-11928-w

Li LY, 2014, J NEUROINFLAMM, V11, DOI 10.1186/1742-2094-11-72

Liu GJ, 2021, MOL NEUROBIOL, V58, P1963, DOI 10.1007/s12035-020-02237-1

Liu GJ, 2020, J NEUROINFLAMM, V17, DOI 10.1186/s12974-020-01918-x

Liu Guanghao, 2012, Am J Neurodegener Dis, V1, P60

Liu P, 2019, NEURAL REGEN RES, V14, P1335, DOI 10.4103/1673-5374.253510

Liu W, 2019, EXP NEUROL, V320, DOI 10.1016/j.expneurol.2019.112982

Liu ZH, 2016, ANESTHESIOLOGY, V124, P934, DOI 10.1097/ALN.0000000000001010

Luo BW, 2016, J NEUROSCI, V36, P9590, DOI 10.1523/JNEUROSCI.0020-16.2016

Maciejewska-Markiewicz D, 2021, BIOMOLECULES, V11, DOI 10.3390/biom11070937

Martinon F, 2002, MOL CELL, V10, P417, DOI 10.1016/S1097-2765(02)00599-3

Mas E, 2012, CLIN CHEM, V58, P1476, DOI 10.1373/clinchem.2012.190199

Mathews SB, 2014, AM J GERIAT PSYCHIAT, V22, P465, DOI 10.1016/j.jagp.2012.08.012

Mawuenyega KG, 2010, SCIENCE, V330, P1774, DOI 10.1126/science.1197623

Meesawatsom P, 2020, J NEUROINFLAMM, V17, DOI 10.1186/s12974-020-01997-w

Meesawatsom P, 2016, J NEUROINFLAMM, V13, DOI 10.1186/s12974-016-0676-6

Miao ZJ, 2021, BIOMED RES INT, V2021, DOI 10.1155/2021/6657646

Minami Y, 2018, SCI REP-UK, V8, DOI 10.1038/s41598-018-21426-6

Mizwicki MT, 2013, J ALZHEIMERS DIS, V34, P155, DOI 10.3233/JAD-121735

Moller JT, 1998, LANCET, V351, P857, DOI 10.1016/S0140-6736(97)07382-0

Mostafa DG, 2020, J PHYSIOL BIOCHEM, V76, P393, DOI 10.1007/s13105-020-00744-2

Mostafa DG, 2020, NEUROCHEM RES, V45, P1576, DOI 10.1007/s11064-020-03022-1

Nolan JP, 2008, RESUSCITATION, V79, P350, DOI 10.1016/j.resuscitation.2008.09.017

Nowak JZ, 2014, EXPERT OPIN DRUG SAF, V13, P687, DOI 10.1517/14740338.2014.915939

Oehler B, 2017, FRONT MOL NEUROSCI, V10, DOI 10.3389/fnmol.2017.00242

Pajares M, 2020, CELLS-BASEL, V9, DOI 10.3390/cells9071687

Park CK, 2011, J NEUROSCI, V31, P18433, DOI 10.1523/JNEUROSCI.4192-11.2011

Payrits M, 2020, INT J MOL SCI, V21, DOI 10.3390/ijms21145019

Pellicanò M, 2012, J NEUROIMMUNOL, V242, P52, DOI 10.1016/j.jneuroim.2011.11.005

Podbielska M, 2021, INT J MOL SCI, V22, DOI 10.3390/ijms22147319

Poewe W, 2017, NAT REV DIS PRIMERS, V3, DOI 10.1038/nrdp.2017.13

Poisson LM, 2015, J BIOL CHEM, V290, P30697, DOI 10.1074/jbc.M115.679068

Prinz M, 2019, CELL, V179, P292, DOI 10.1016/j.cell.2019.08.053

Prüss H, 2013, PLOS ONE, V8, DOI 10.1371/journal.pone.0055859

Ramirez-Ramirez V, 2013, OXID MED CELL LONGEV, V2013, DOI 10.1155/2013/709493

Reale M, 2009, BRAIN BEHAV IMMUN, V23, P55, DOI 10.1016/j.bbi.2008.07.003

Recchiuti A, 2013, PROSTAG OTH LIPID M, V107, P64, DOI 10.1016/j.prostaglandins.2013.02.004

Ren HX, 2017, MOL NEUROBIOL, V54, P3317, DOI 10.1007/s12035-016-9931-1

Ren YZ, 2020, J NEUROCHEM, V154, P530, DOI 10.1111/jnc.14962

Rey C, 2016, BRAIN BEHAV IMMUN, V55, P249, DOI 10.1016/j.bbi.2015.12.013

Saito T, 2015, ANESTHESIOLOGY, V123, P1420, DOI 10.1097/ALN.0000000000000892

Sedeighzadeh SS, 2021, MEDIAT INFLAMM, V2021, DOI 10.1155/2021/6698049

Sekeljic V, 2012, BRAIN STRUCT FUNCT, V217, P411, DOI 10.1007/s00429-011-0336-7

Serhan CN, 2020, MOL ASPECTS MED, V74, DOI 10.1016/j.mam.2020.100894

Serhan CN, 2014, NATURE, V510, P92, DOI 10.1038/nature13479

Serhan CN, 2000, J EXP MED, V192, P1197, DOI 10.1084/jem.192.8.1197

Shalini SM, 2018, MOL NEUROBIOL, V55, P1537, DOI 10.1007/s12035-017-0413-x

Shati AA, 2022, CLIN EXP PHARMACOL P, V49, P275, DOI 10.1111/1440-1681.13596

Shati AA, 2021, HUM EXP TOXICOL, V40, pS215, DOI 10.1177/09603271211038739

Shevalye H, 2015, J NEUROPHYSIOL, V114, P199, DOI 10.1152/jn.00224.2015

Siegert E, 2017, BMC NEUROSCI, V18, DOI 10.1186/s12868-016-0312-5

Sta M, 2011, NEUROBIOL DIS, V42, P211, DOI 10.1016/j.nbd.2011.01.002

Stein C, 2009, BRAIN RES REV, V60, P90, DOI 10.1016/j.brainresrev.2008.12.017

Sun QM, 2021, FRONT PHARMACOL, V12, DOI 10.3389/fphar.2021.720249

Tao XS, 2021, FRONT PHYSIOL, V12, DOI 10.3389/fphys.2021.687046

Terrando N, 2013, FASEB J, V27, P3564, DOI 10.1096/fj.13-230276

Tiberi M, 2021, FRONT CELL NEUROSCI, V15, DOI 10.3389/fncel.2021.673549

Tiffany HL, 2001, J BIOL CHEM, V276, P23645, DOI 10.1074/jbc.M101031200

Tortelli R, 2020, FRONT NEUROL, V11, DOI 10.3389/fneur.2020.552295

Troletti CD, 2021, CELL REP, V35, DOI 10.1016/j.celrep.2021.109201

Trotta MC., 2021, INT J MOL SCI, V22

Varani K, 2010, FASEB J, V24, P587, DOI 10.1096/fj.09-141044

Viscomi MT, 2014, MOL NEUROBIOL, V50, P368, DOI 10.1007/s12035-013-8629-x

Vlad SC, 2008, NEUROLOGY, V70, P1672, DOI 10.1212/01.wnl.0000311269.57716.63

Voet S, 2019, TRENDS MOL MED, V25, P112, DOI 10.1016/j.molmed.2018.11.005

Voet S, 2018, NAT COMMUN, V9, DOI 10.1038/s41467-018-04376-5

Wang JCF, 2017, J PAIN, V18, P535, DOI 10.1016/j.jpain.2016.12.012

Wang XZ, 2015, J LIPID RES, V56, P674, DOI 10.1194/jlr.P055418

Wang XZ, 2015, ALZHEIMERS DEMENT, V11, P40, DOI 10.1016/j.jalz.2013.12.024

Wang X, 2019, MULT SCLER RELAT DIS, V27, P34, DOI 10.1016/j.msard.2018.09.040

Wei CC, 2021, FRONT PHARMACOL, V11, DOI 10.3389/fphar.2020.610734

Xu JY, 2017, MED SCI MONITOR, V23, P2684, DOI 10.12659/MSM.901995

Xu MX, 2013, CNS NEUROSCI THER, V19, P235, DOI 10.1111/cns.12069

Xu ZZ, 2010, NAT MED, V16, P592, DOI 10.1038/nm.2123

Zhu MQ, 2016, MOL NEUROBIOL, V53, P2733, DOI 10.1007/s12035-015-9544-0

NR 131

TC 15

Z9 15

U1 3

U2 15

PU WILEY

PI HOBOKEN

PA 111 RIVER ST, HOBOKEN 07030-5774, NJ USA

SN 0951-6433

EI 1872-8081

J9 BIOFACTORS

JI Biofactors

PD SEP

PY 2022

VL 48

IS 5

BP 1005

EP 1026

DI 10.1002/biof.1891

PG 22

WC Biochemistry & Molecular Biology; Endocrinology & Metabolism

WE Science Citation Index Expanded (SCI-EXPANDED)

SC Biochemistry & Molecular Biology; Endocrinology & Metabolism

GA 5H7MF

UT WOS:000867858200007

PM 36176016

DA 2024-08-12

ER

PT J

AU Eren, E

Özören, N

AF Eren, Elif

Ozoren, Nesrin

TI The NLRP3 inflammasome: a new player in neurological diseases

SO TURKISH JOURNAL OF BIOLOGY

LA English

DT Review

DE NLRP3; inflammasomes; Alzheimer's disease; Parkinson's disease

ID EXPERIMENTAL AUTOIMMUNE ENCEPHALOMYELITIS; NALP3 INFLAMMASOME;

FAMILY-MEMBER; AMYLOID-BETA; GASDERMIN D; ACTIVATION; PROTEIN; NLRC3;

REGULATOR; RECEPTOR

AB Inflammasomes are supramolecular protein complexes implicated in the detection of pathogens or danger-associated molecules and are responsible for mounting the first line of innate immune response to counteract these signals and restore tissue homeostasis. Among different inflammasomes identified so far, NLRP3 is of main interest since mutations in Nlrp3 gene are associated with autoinflammatory diseases such as Muckle-Wells syndrome, neonatal onset multisystem inflammatory disease, and familial cold urticaria/autoinflammatory syndrome. On the other hand, whereas other inflammasomes are mainly detectors of specific molecular motifs, NLRP3 is acting as a general sensor of cellular perturbations including potassium efflux, lysosomal damage, and ROS production. Besides this central role of NLRP3 in inflammation, recent publications show that the NLRP3 inflammasome is also involved in the physiopathology of several neurological disorders including Alzheimer's disease, Parkinson's disease, and multiple sclerosis. This review gives an overview of the established functions of the NLRP3 inflammasome in mediating inflammation in macrophages and describes its recently discovered roles in neurological disorders in promoting neuroinflammation, as well as modulating key proteins mediating the disorders. Finally, we discuss the targeting of NLRP3 in neurological diseases and present some examples of NLRP3 inhibitors that could be used in neurological disorder treatments.

C1 [Eren, Elif; Ozoren, Nesrin] Bogazici Univ, Dept Mol Biol & Genet, Apoptosis & Canc Immunol Lab AKIL, Istanbul, Turkey.

[Eren, Elif; Ozoren, Nesrin] Bogazici Univ, Ctr Life Sci & Technol, Istanbul, Turkey.

C3 Bogazici University; Bogazici University

RP Özören, N (corresponding author), Bogazici Univ, Dept Mol Biol & Genet, Apoptosis & Canc Immunol Lab AKIL, Istanbul, Turkey.; Özören, N (corresponding author), Bogazici Univ, Ctr Life Sci & Technol, Istanbul, Turkey.

RI Ozoren, Nesrin/AAS-3279-2020; Eren, Elif/A-6057-2016

OI Eren, Elif/0000-0002-0328-5609; OZOREN, NESRIN/0000-0002-8580-618X

FU Bogazici University [BAP 6526 (11B01D10), 7360 (13B01M3)]

FX Authors would like to thank Bogazici University Research Funding via

Projects BAP 6526 (11B01D10) and 7360 (13B01M3) to N.O.

CR Aganna E, 2002, ARTHRITIS RHEUM-US, V46, P2445, DOI 10.1002/art.10509

Allen IC, 2011, IMMUNITY, V34, P854, DOI 10.1016/j.immuni.2011.03.026

Andoh T, 2008, J IMMUNOL, V180, P2322, DOI 10.4049/jimmunol.180.4.2322

Bauernfeind FG, 2009, J IMMUNOL, V183, P787, DOI 10.4049/jimmunol.0901363

Blennow K, 2016, NAT REV DIS PRIMERS, V2, DOI 10.1038/nrdp.2016.84

Coll RC, 2019, NAT CHEM BIOL, V15, P556, DOI 10.1038/s41589-019-0277-7

Coll RC, 2015, NAT MED, V21, P248, DOI 10.1038/nm.3806

Conti BJ, 2005, J BIOL CHEM, V280, P18375, DOI 10.1074/jbc.M413169200

Coornaert B, 2009, J BIOL CHEM, V284, P8217, DOI 10.1074/jbc.R800032200

Dempsey C, 2017, BRAIN BEHAV IMMUN, V61, P306, DOI 10.1016/j.bbi.2016.12.014

Eitas TK, 2014, J BIOL CHEM, V289, P4173, DOI 10.1074/jbc.M113.533034

Eren E, 2017, J BIOL CHEM, V292, P12691, DOI 10.1074/jbc.M116.769695

Fan YX, 2018, FRONT CELL NEUROSCI, V12, DOI 10.3389/fncel.2018.00426

Farooqui AA, 2006, PHARMACOL REV, V58, P591, DOI 10.1124/pr.58.3.7

Filippi M, 2018, NAT REV DIS PRIMERS, V4, DOI [10.1038/s41572-018-0041-4, 10.1038/s41572-018-0046-z]

Flores J, 2018, NAT COMMUN, V9, DOI 10.1038/s41467-018-06449-x

Franklin BS, 2014, NAT IMMUNOL, V15, P727, DOI 10.1038/ni.2913

Freeman L, 2017, J EXP MED, V214, P1351, DOI 10.1084/jem.20150237

Gordon R, 2018, SCI TRANSL MED, V10, DOI 10.1126/scitranslmed.aah4066

Gris D, 2010, J IMMUNOL, V185, P974, DOI 10.4049/jimmunol.0904145

Guedes RP, 2014, J NEUROINFLAMM, V11, DOI 10.1186/1742-2094-11-122

Halle A, 2008, NAT IMMUNOL, V9, P857, DOI 10.1038/ni.1636

Hawkins PN, 2004, ARTHRITIS RHEUM-US, V50, P607, DOI 10.1002/art.20033

He WT, 2015, CELL RES, V25, P1285, DOI 10.1038/cr.2015.139

Heneka MT, 2013, NATURE, V493, P674, DOI 10.1038/nature11729

Hornung V, 2008, NAT IMMUNOL, V9, P847, DOI 10.1038/ni.1631

Hornung V, 2009, NATURE, V458, P514, DOI 10.1038/nature07725

Hsu LC, 2008, P NATL ACAD SCI USA, V105, P7803, DOI 10.1073/pnas.0802726105

Hu SF, 2018, PLOS PATHOG, V14, DOI 10.1371/journal.ppat.1007266

Ismael S, 2018, J NEUROTRAUM, V35, P1294, DOI 10.1089/neu.2017.5344

Iyer SS, 2013, IMMUNITY, V39, P311, DOI 10.1016/j.immuni.2013.08.001

Jorgensen I, 2016, EUR J IMMUNOL, V46, P2761, DOI 10.1002/eji.201646647

KOSTURA MJ, 1989, P NATL ACAD SCI USA, V86, P5227, DOI 10.1073/pnas.86.14.5227

Lee E, 2019, CELL DEATH DIFFER, V26, P213, DOI 10.1038/s41418-018-0124-5

Li HN, 2016, STRUCT MONIT MAINT, V3, P1, DOI 10.12989/smm.2016.3.1.001

Li XQ, 2019, NEUROPHARMACOLOGY, V160, DOI 10.1016/j.neuropharm.2019.05.038

Li X, 2019, IMMUNITY, V50, P591, DOI 10.1016/j.immuni.2019.02.009

Lim J, 2019, SCI REP-UK, V9, DOI 10.1038/s41598-019-44261-9

Liu X, 2016, NATURE, V535, P153, DOI 10.1038/nature18629

Ma A, 2012, NAT REV IMMUNOL, V12, P774, DOI 10.1038/nri3313

MAHMOUD S, 2019, CELLS-BASEL, V8, DOI DOI 10.3390/CELLS8050400

Mariathasan S, 2006, NATURE, V440, P228, DOI 10.1038/nature04515

Martinon F, 2002, MOL CELL, V10, P417, DOI 10.1016/S1097-2765(02)00599-3

Masters CL, 2015, NAT REV DIS PRIMERS, V1, DOI 10.1038/nrdp.2015.56

Maver A, 2017, SCI REP-UK, V7, DOI 10.1038/s41598-017-03536-9

McKenzie BA, 2018, P NATL ACAD SCI USA, V115, pE6065, DOI 10.1073/pnas.1722041115

Moore CB, 2008, NATURE, V451, P573, DOI 10.1038/nature06501

Muñoz-Planillo R, 2013, IMMUNITY, V38, P1142, DOI 10.1016/j.immuni.2013.05.016

Nimmerjahn A, 2005, SCIENCE, V308, P1314, DOI 10.1126/science.1110647

Panicker N, 2019, J EXP MED, V216, P1411, DOI 10.1084/jem.20182191

Park S, 2013, J IMMUNOL, V191, P4358, DOI 10.4049/jimmunol.1301170

Petrilli V, 2007, CELL DEATH DIFFER, V14, P1583, DOI 10.1038/sj.cdd.4402195

Poewe W, 2017, NAT REV DIS PRIMERS, V3, DOI 10.1038/nrdp.2017.13

Pontillo A, 2012, ALZ DIS ASSOC DIS, V26, P277, DOI 10.1097/WAD.0b013e318231a8ac

Saresella M, 2016, MOL NEURODEGENER, V11, DOI 10.1186/s13024-016-0088-1

Schneider M, 2012, NAT IMMUNOL, V13, P823, DOI 10.1038/ni.2378

Shi JJ, 2015, NATURE, V526, P660, DOI 10.1038/nature15514

Shiau CE, 2013, CELL REP, V5, P1342, DOI 10.1016/j.celrep.2013.11.004

Stancu IC, 2019, ACTA NEUROPATHOL, V137, P599, DOI 10.1007/s00401-018-01957-y

Stehlik C, 2003, J IMMUNOL, V171, P6154, DOI 10.4049/jimmunol.171.11.6154

Sutterwala FS, 2007, J EXP MED, V204, P3235, DOI 10.1084/jem.20071239

Tapia-Abelián A, 2019, NAT CHEM BIOL, V15, P560, DOI 10.1038/s41589-019-0278-6

Theus MH, 2017, J IMMUNOL, V199, P3547, DOI 10.4049/jimmunol.1700251

Tzeng TC, 2018, P NATL ACAD SCI USA, V115, P9002, DOI 10.1073/pnas.1801802115

Uchimura T, 2018, IMMUNITY, V49, P1049, DOI 10.1016/j.immuni.2018.10.008

Vande Walle L, 2019, PLOS BIOL, V17, DOI 10.1371/journal.pbio.3000354

Venegas C, 2017, NATURE, V552, P355, DOI 10.1038/nature25158

Voet S, 2018, NAT COMMUN, V9, DOI 10.1038/s41467-018-04376-5

Wang TN, 2019, J GENET GENOMICS, V46, P291, DOI 10.1016/j.jgg.2019.06.002

Wang W, 2016, P NATL ACAD SCI USA, V113, P9587, DOI 10.1073/pnas.1610099113

Xu H, 2014, NATURE, V513, P237, DOI 10.1038/nature13449

Xu X, 2018, NEUROBIOL DIS, V117, P15, DOI 10.1016/j.nbd.2018.05.016

Zhang L, 2014, IMMUNITY, V40, P329, DOI 10.1016/j.immuni.2014.01.010

Zhou RB, 2011, NATURE, V469, P221, DOI 10.1038/nature09663

NR 74

TC 32

Z9 35

U1 0

U2 6

PU Tubitak Scientific & Technological Research Council Turkey

PI ANKARA

PA ATATURK BULVARI NO 221, KAVAKLIDERE, TR-06100 ANKARA, Turkey

SN 1300-0152

EI 1303-6092

J9 TURK J BIOL

JI Turk. J. Biol.

PY 2019

VL 43

IS 6

BP 349

EP 359

DI 10.3906/biy-1909-31

PG 11

WC Biology

WE Science Citation Index Expanded (SCI-EXPANDED)

SC Life Sciences & Biomedicine - Other Topics

GA JY8FJ

UT WOS:000504643700001

PM 31892810

OA gold, Green Published

DA 2024-08-12

ER

PT J

AU Olsen, I

Kell, DB

Pretorius, E

AF Olsen, Ingar

Kell, Douglas B.

Pretorius, Etheresia

TI Is<i>Porphyromonas gingivalis</i>involved in Parkinson's disease?

SO EUROPEAN JOURNAL OF CLINICAL MICROBIOLOGY & INFECTIOUS DISEASES

LA English

DT Review

DE Systemic inflammation; Cytokines; Hypercoagulation; Amyloid formation;

Gingipains; LPS

ID PORPHYROMONAS-GINGIVALIS; CYSTEINE PROTEINASES; GUT; MICROBIOME;

ACTIVATION; BLOOD; NEUROINFLAMMATION; PERIODONTITIS; COMPLEMENT;

PATHOLOGY

AB Porphyromonas gingivalis, a major subgingival plaque bacterium in periodontitis, has recently attracted much attention as a possible microbial driver in Alzheimer's disease. In the present paper, another common neuroinflammatory disease, Parkinson's disease (PD), is discussed. A recent study found major virulence factors ofP. gingivalissuch as gingipain R1 (RgpA) and lipopolysaccharide in the blood circulation of a PD population. The current review reveals how features such as systemic inflammation, hypercoagulation, presence of amyloid fibrin(ogen) in plasma, and marked ultrastructural changes in platelets, probably induced byP. gingivalis, may affect the development of PD. Several other clinical studies have also demonstrated an association between periodontitis and PD. Even if the risk of periodontal diseases causing neurological disorders needs to be better substantiated, that should not keep us from trying to prevent them by performing careful daily dental hygiene.

C1 [Olsen, Ingar] Univ Oslo, Fac Dent, Dept Oral Biol, POB 1052 Blindern, N-0316 Oslo, Norway.

[Kell, Douglas B.] Univ Liverpool, Fac Hlth & Life Sci, Inst Integrat Biol, Dept Biochem, Liverpool, Merseyside, United Kingdom.

[Kell, Douglas B.; Pretorius, Etheresia] Stellenbosch Univ, Fac Sci, Dept Physiol Sci, Stellenbosch, South Africa.

C3 University of Oslo; University of Liverpool; Stellenbosch University

RP Olsen, I (corresponding author), Univ Oslo, Fac Dent, Dept Oral Biol, POB 1052 Blindern, N-0316 Oslo, Norway.

EM ingar.olsen@odont.uio.no

RI Pretorius, Etheresia/ACH-6368-2022; Kell, Douglas/E-8318-2011

OI Pretorius, Etheresia/0000-0002-9108-2384; Kell,

Douglas/0000-0001-5838-7963

FU University of Oslo (incl Oslo University Hospital); Biotechnology and

Biological Sciences Research Council [BB/L025752/1]; Medical Research

Council of South Africa (MRC); BBSRC [BB/L025752/1] Funding Source: UKRI

FX Open Access funding provided by University of Oslo (incl Oslo University

Hospital). This collaboration received financial support from the

Biotechnology and Biological Sciences Research Council (Grant

BB/L025752/1) as well as the Medical Research Council of South Africa

(MRC; Self-Initiated Research Program).

CR Aas JA, 2005, J CLIN MICROBIOL, V43, P5721, DOI 10.1128/JCM.43.11.5721-5732.2005

Adams B, 2019, FRONT AGING NEUROSCI, V11, DOI 10.3389/fnagi.2019.00210

Brodacki B, 2008, NEUROSCI LETT, V441, P158, DOI 10.1016/j.neulet.2008.06.040

Caputi V, 2018, INT J MOL SCI, V19, DOI 10.3390/ijms19061689

Caton JG, 2018, J CLIN PERIODONTOL, V45, pS1, DOI 10.1111/jcpe.12935

Cavaillon JM, 2018, TOXICON, V149, P45, DOI 10.1016/j.toxicon.2017.10.016

Chen CK, 2018, INT J ENV RES PUB HE, V15, DOI 10.3390/ijerph15081587

Chen CK, 2017, PEERJ, V5, DOI 10.7717/peerj.3647

Darveau RP, 2012, J DENT RES, V91, P816, DOI 10.1177/0022034512453589

Darveau RP, 1998, INFECT IMMUN, V66, P1660, DOI 10.1128/IAI.66.4.1660-1665.1998

De Chiara G, 2012, MOL NEUROBIOL, V46, P614, DOI 10.1007/s12035-012-8320-7

Dominy SS, 2019, SCI ADV, V5, DOI 10.1126/sciadv.aau3333

Duran-Pinedo AE, 2014, ISME J, V8, P1659, DOI 10.1038/ismej.2014.23

Friedland RP, 2017, PLOS PATHOG, V13, DOI 10.1371/journal.ppat.1006654

Guo YH, 2010, PERIODONTOL 2000, V54, P15, DOI 10.1111/j.1600-0757.2010.00377.x

Hajishengallis G, 2012, MOL ORAL MICROBIOL, V27, P409, DOI 10.1111/j.2041-1014.2012.00663.x

Hajishengallis G, 2012, NAT REV MICROBIOL, V10, P717, DOI 10.1038/nrmicro2873

Hasegawa S, 2015, PLOS ONE, V10, DOI 10.1371/journal.pone.0142164

Hashioka S, 2019, INT J MOL SCI, V20, DOI 10.3390/ijms20153723

Herrero ER, 2018, J DENT RES, V97, P547, DOI 10.1177/0022034517752675

Holmstrup P, 2017, J ORAL MICROBIOL, V9, DOI 10.1080/20002297.2017.1332710

IMAMURA T, 1995, INFECT IMMUN, V63, P4877, DOI 10.1128/IAI.63.12.4877-4882.1995

Imamura T, 1997, J BIOL CHEM, V272, P16062, DOI 10.1074/jbc.272.25.16062

Imamura T, 2001, J BIOL CHEM, V276, P18984, DOI 10.1074/jbc.M006760200

Kannarkat GT, 2013, J PARKINSON DIS, V3, P493, DOI 10.3233/JPD-130250

Kaur T, 2016, GERODONTOLOGY, V33, P434, DOI 10.1111/ger.12188

Kell DB, 2018, BIOL REV, V93, P1518, DOI 10.1111/brv.12407

Kell DB, 2015, INTEGR BIOL-UK, V7, P1339, DOI 10.1039/c5ib00158g

Lamont RJ, 2018, NAT REV MICROBIOL, V16, P745, DOI 10.1038/s41579-018-0089-x

Lotankar S, 2017, NEUROSCI BULL, V33, P585, DOI 10.1007/s12264-017-0183-5

More SV, 2013, MEDIAT INFLAMM, V2013, DOI 10.1155/2013/952375

Mulak A, 2015, WORLD J GASTROENTERO, V21, P10609, DOI 10.3748/wjg.v21.i37.10609

Nolan YM, 2013, TRENDS MOL MED, V19, P187, DOI 10.1016/j.molmed.2012.12.003

Olsen I., 2019, Curr. Oral Health Rep, V6, P120, DOI [10.1007/s40496-019-0212-8, DOI 10.1007/S40496-019-0212-8]

Olsen I, 2020, J ORAL MICROBIOL, V12, DOI 10.1080/20002297.2019.1676486

Olsen I, 2015, J ORAL MICROBIOL, V7, DOI 10.3402/jom.v7.29143

Olsen I, 2015, ACTA ODONTOL SCAND, V73, P563, DOI 10.3109/00016357.2015.1007480

Olumuyiwa-Akeredolu OO, 2019, NAT REV RHEUMATOL, V15, P237, DOI 10.1038/s41584-019-0187-9

Poole S, 2015, J ALZHEIMERS DIS, V43, P67, DOI 10.3233/JAD-140315

Potgieter M, 2015, FEMS MICROBIOL REV, V39, P567, DOI 10.1093/femsre/fuv013

Pretorius E, 2018, FRONT AGING NEUROSCI, V10, DOI 10.3389/fnagi.2018.00257

Pretorius E, 2018, PLOS ONE, V13, DOI 10.1371/journal.pone.0192121

Pretorius E, 2017, CARDIOVASC DIABETOL, V16, DOI 10.1186/s12933-017-0624-5

Pretorius E, 2017, SCI REP-UK, V7, DOI 10.1038/s41598-017-09860-4

Qin XY, 2016, JAMA NEUROL, V73, P1316, DOI 10.1001/jamaneurol.2016.2742

Reale M, 2009, BRAIN BEHAV IMMUN, V23, P55, DOI 10.1016/j.bbi.2008.07.003

Sampson TR, 2016, CELL, V167, P1469, DOI 10.1016/j.cell.2016.11.018

Scheperjans F, 2018, J PARKINSON DIS, V8, pS31, DOI 10.3233/JPD-181477

Schwarz J, 2006, J NEUROL, V253, P608, DOI 10.1007/s00415-006-0068-4

Slaney JM, 2008, FRONT BIOSCI-LANDMRK, V13, P188, DOI 10.2741/2669

Socransky SS, 2005, PERIODONTOL 2000, V38, P135, DOI 10.1111/j.1600-0757.2005.00107.x

Sun MF, 2018, BRAIN BEHAV IMMUN, V70, P48, DOI 10.1016/j.bbi.2018.02.005

Sun MF, 2018, AGEING RES REV, V45, P53, DOI 10.1016/j.arr.2018.04.004

Taylor JM, 2013, NEUROCHEM INT, V62, P803, DOI 10.1016/j.neuint.2012.12.016

Ton TGN, 2012, PARKINSONISM RELAT D, V18, P274, DOI 10.1016/j.parkreldis.2011.11.003

Tufekci KU, 2011, AUTOIMMUN DIS, V2011, DOI 10.4061/2011/807426

Weenen H, 2019, CHEMOSENS PERCEPT, V12, P1, DOI 10.1007/s12078-018-9246-x

Wong KT, 2010, NEUROEPIDEMIOLOGY, V34, P50, DOI 10.1159/000260070

Yang DM, 2019, FRONT NEUROL, V10, DOI 10.3389/fneur.2019.01155

Zlotnik Y, 2015, PARKINSONS DIS-US, V2015, DOI 10.1155/2015/379482

NR 60

TC 23

Z9 26

U1 0

U2 10

PU SPRINGER

PI NEW YORK

PA ONE NEW YORK PLAZA, SUITE 4600, NEW YORK, NY, UNITED STATES

SN 0934-9723

EI 1435-4373

J9 EUR J CLIN MICROBIOL

JI Eur. J. Clin. Microbiol. Infect. Dis.

PD NOV

PY 2020

VL 39

IS 11

BP 2013

EP 2018

DI 10.1007/s10096-020-03944-2

EA JUN 2020

PG 6

WC Infectious Diseases; Microbiology

WE Science Citation Index Expanded (SCI-EXPANDED)

SC Infectious Diseases; Microbiology

GA OA8XQ

UT WOS:000541409000001

PM 32564247

OA hybrid, Green Published

DA 2024-08-12

ER

PT J

AU Yang, LK

Guo, CF

Zhu, J

Feng, Y

Chen, WL

Feng, ZZ

Wang, D

Sun, SB

Lin, W

Wang, YH

AF Yang, Likun

Guo, Changfeng

Zhu, Jie

Feng, Yi

Chen, Weiliang

Feng, Zhizhong

Wang, Dan

Sun, Shibai

Lin, Wei

Wang, Yuhai

TI Increased Levels of Pro-Inflammatory and Anti-Inflammatory Cellular

Responses in Parkinson's Disease Patients: Search for a Disease

Indicator

SO MEDICAL SCIENCE MONITOR

LA English

DT Article

DE Biological Markers; Myeloid Cells; Neurogenic Inflammation; Parkinsonian

Disorders; T-Lymphocytes, Helper-Induc

ID CD4(+) T-CELLS; SUPPRESSOR-CELLS; TH17 CELLS; NEUROINFLAMMATION;

NEURODEGENERATION; MODEL; INFILTRATION; ACTIVATION; EXPRESSION;

MICROGLIA

AB Background: Parkinson's disease (PD) is the second most prevalent neurodegenerative disorder and it arises when most of the dopaminergic neurons of substantia nigra region die. Several mechanisms have been postulated as the causative event in PD pathology, and neuroinflammation is most crucial among them.

Material/Methods: We analyzed T-helper 17 (Th17) cells and myeloid-derived suppressor cells (MDSCs) from 80 PD patients to assess inflammatory processes and to find a cost-effective means to evaluate PD prognosis.

Results: We found significantly increased numbers of Th17 cells and MDSCs count in peripheral circulation in PD patients compared with controls (p<0.001). A positive correlation was found between Th17 cells and MDSCs in PD patients (r=0.421, p<0.05).

Conclusions: Our results show the effector role of Th17 cells and MDSCs in PD pathology and shows their utility as effective biomarkers for PD diagnosis.

C1 [Yang, Likun; Zhu, Jie; Feng, Yi; Chen, Weiliang; Feng, Zhizhong; Wang, Dan; Sun, Shibai; Lin, Wei; Wang, Yuhai] 101 Hosp Chinese PLA, Dept Neurosurg, Wuxi, Jiangsu, China.

[Guo, Changfeng] Tongji Univ, Shanghai Peoples Hosp 10, Dept Emergency Med, Sch Med, Shanghai, China.

C3 Tongji University

RP Lin, W; Wang, YH (corresponding author), 101 Hosp Chinese PLA, Dept Neurosurg, Wuxi, Jiangsu, China.

EM wumm514054@163.com; Woods.vuckovska580@hotmail.com

RI Lin, Wei/B-7995-2009; 朱洁, jie/HHZ-0832-2022

OI Lin, Wei/0000-0003-4075-7414;

CR Abrams SI, 2016, J INTERF CYTOK RES, V36, P442, DOI 10.1089/jir.2015.0174

Aktas O, 2007, ARCH NEUROL-CHICAGO, V64, P185, DOI 10.1001/archneur.64.2.185

Bahbouhi B, 2009, J LEUKOCYTE BIOL, V86, P1049, DOI 10.1189/jlb.1008666

Barnes PJ, 2016, J ALLERGY CLIN IMMUN, V138, P16, DOI 10.1016/j.jaci.2016.05.011

Basdeo SA, 2016, SPRINGERPLUS, V5, DOI 10.1186/s40064-016-2510-0

Brochard V, 2009, J CLIN INVEST, V119, P182, DOI 10.1172/JCI36470

Bueno V, 2014, AGE, V36, DOI 10.1007/s11357-014-9729-x

Chatterjee S, 2013, IMMUNOBIOLOGY, V218, P718, DOI 10.1016/j.imbio.2012.08.271

Chen YH, 2015, INT J CLIN EXP PATHO, V8, P2495

Fan K, 2015, J NEUROINFLAMM, V12, DOI 10.1186/s12974-015-0268-x

Gleason MK, 2014, BLOOD, V123, P3016, DOI 10.1182/blood-2013-10-533398

Hoechst B, 2011, BLOOD, V117, P6532, DOI 10.1182/blood-2010-11-317321

Holmgaard RB, 2015, CELL REP, V13, P412, DOI 10.1016/j.celrep.2015.08.077

Jha MK, 2013, EXPERT REV PROTEOMIC, V10, P43, DOI [10.1586/EPR.12.70, 10.1586/epr.12.70]

Ji JJ, 2016, CLIN SCI, V130, P1453, DOI 10.1042/CS20160311

Joniec-Maciejak I, 2014, PHARMACOL REP, V66, P660, DOI 10.1016/j.pharep.2014.03.008

Kannan AK, 2015, J NEUROSCI, V35, P221, DOI 10.1523/JNEUROSCI.1957-14.2015

Kim JH, 2016, J NEUROINFLAMM, V13, DOI 10.1186/s12974-016-0656-x

Landoni VI, 2016, CLIN SCI, V130, P259, DOI 10.1042/CS20150653

Lang AE, 1998, NEW ENGL J MED, V339, P1044, DOI 10.1056/NEJM199810083391506

Levite M, 2016, ACTA PHYSIOL, V216, P42, DOI 10.1111/apha.12476

Lim S, 2016, BRAIN PATHOL, V26, P404, DOI 10.1111/bpa.12371

Mehanna R, 2016, AM J CASE REP, V17, DOI 10.12659/AJCR.900537

Mifflin KA, 2017, J NEUROSCI RES, V95, P1282, DOI 10.1002/jnr.23844

Murphy AC, 2010, BRAIN BEHAV IMMUN, V24, P641, DOI 10.1016/j.bbi.2010.01.014

Obermeier B, 2016, HAND CLINIC, V133, P39, DOI 10.1016/B978-0-444-63432-0.00003-7

Ostanin DV, 2013, INFLAMM BOWEL DIS, V19, P2468, DOI 10.1097/MIB.0b013e3182902b11

Ren Y, 2016, MED SCI MONITOR, V22, P1733, DOI 10.12659/MSM.898400

Reynolds AD, 2010, J IMMUNOL, V184, P2261, DOI 10.4049/jimmunol.0901852

Saraiva C, 2016, J CONTROL RELEASE, V235, P34, DOI 10.1016/j.jconrel.2016.05.044

Sasaki T, 2016, EXP NEUROL, V275, P220, DOI 10.1016/j.expneurol.2015.11.003

Smith AR, 2014, J LEUKOCYTE BIOL, V96, P361, DOI 10.1189/jlb.3CE0414-205R

Vivekanantham S, 2015, INT J NEUROSCI, V125, P717, DOI 10.3109/00207454.2014.982795

Wu Li, 2016, Med Sci Monit, V22, P1553

YAHR MD, 1978, J NEURAL TRANSM, V43, P227, DOI 10.1007/BF01246959

NR 35

TC 23

Z9 24

U1 0

U2 2

PU INT SCIENTIFIC LITERATURE, INC

PI SMITHTOWN

PA 361 FOREST LANE, SMITHTOWN, NY 11787 USA

SN 1643-3750

J9 MED SCI MONITOR

JI Med. Sci. Monitor

PD JUN 18

PY 2017

VL 23

BP 2972

EP 2978

DI 10.12659/MSM.904240

PG 7

WC Medicine, Research & Experimental

WE Science Citation Index Expanded (SCI-EXPANDED)

SC Research & Experimental Medicine

GA EY1UY

UT WOS:000403753900001

PM 28624842

OA Green Published, Green Submitted

DA 2024-08-12

ER

PT J

AU Thakur, K

Khan, H

Grewal, AK

Singh, TG

AF Thakur, Kiran

Khan, Heena

Grewal, Amarjot Kaur

Singh, Thakur Gurjeet

TI Nuclear orphan receptors: A novel therapeutic agent in neuroinflammation

SO INTERNATIONAL IMMUNOPHARMACOLOGY

LA English

DT Review

DE Neuroinflammation; Neurodegeneration; Nuclear receptors; Orphan

receptors

ID PROTEIN-COUPLED RECEPTORS; parkinson's disease; HUNTINGTONS-DISEASE;

PPAR-GAMMA; GPR3; EXPRESSION; PIOGLITAZONE; INFLAMMATION; INTERPLAY;

NEURONS

AB Orphan receptors constitute a historically varied subsection of a superfamily of nuclear receptors. Nuclear receptors regulate gene expression in response to ligand signals and are particularly alluring therapeutic targets for chronic illnesses. Neuroinflammation and neurodegenerative disease have been linked to these orphan nuclear receptors. Preclinical and clinical evidence suggests that orphan receptors could serve as future targets in neuroinflammation, such as Parkinson's disease (PD), Alzheimer's Disease (AD), Huntington's Disease (HD), Multiple Sclerosis (MS), and Cerebral Ischemia. Given the therapeutic relevance of certain orphan receptors in a variety of disorders, their potential in neuroinflammation remains unproven. There is substantial evidence that ligand-activated transcription factors have great promise for preventing neurodegenerative and neurological disorders, with certain orphan nuclear receptors i.e., PPAR gamma, NR4As, and orphan GPCRs holding particularly high potential. Based on previous findings, we attempted to determine the contribution of PPAR, NR4As, and orphan GPCRs-regulated neuroinflammation to the pathogenesis of these disorders and their potential to become novel therapeutic targets.

C1 [Thakur, Kiran; Khan, Heena; Grewal, Amarjot Kaur; Singh, Thakur Gurjeet] Chitkara Univ, Chitkara Coll Pharm, Rajpura 140401, Punjab, India.

[Singh, Thakur Gurjeet] Chitkara Univ, Chitkara Coll Pharm, Rajpura, Punjab, India.

C3 Chitkara University, Punjab; Chitkara University, Punjab

RP Singh, TG (corresponding author), Chitkara Univ, Chitkara Coll Pharm, Rajpura, Punjab, India.

EM gurjeet.singh@chitkara.edu.in

RI Singh, Thakur Gurjeet/AGV-7671-2022

OI Singh, Thakur Gurjeet/0000-0003-2979-1590

CR Alavi MS, 2018, BIOMED PHARMACOTHER, V98, P222, DOI 10.1016/j.biopha.2017.12.056

Andersen KB, 2016, FRONT CELL NEUROSCI, V10, DOI 10.3389/fncel.2016.00164

Arefin TM, Doctoral dissertation

Behl T, 2021, MOLECULES, V26, DOI 10.3390/molecules26123724

Behl T, 2021, INT J MOL SCI, V22, DOI 10.3390/ijms22031413

Benoit G, 2006, PHARMACOL REV, V58, P798, DOI 10.1124/pr.58.4.10

Benoit ME, 2013, J BIOL CHEM, V288, P654, DOI 10.1074/jbc.M112.400168

Blumberg B, 1998, GENE DEV, V12, P3149, DOI 10.1101/gad.12.20.3149

Burgaz S, 2021, MOLECULES, V26, DOI 10.3390/molecules26247643

Carta AR, 2011, PARKINSONS DIS-US, V2011, DOI 10.4061/2011/689181

Celorrio M, 2017, NEUROPHARMACOLOGY, V125, P319, DOI 10.1016/j.neuropharm.2017.08.017

Chen LQ, 2020, CELL MOL BIOL LETT, V25, DOI 10.1186/s11658-020-00241-w

Chen WW, 2016, MOL MED REP, V13, P3391, DOI 10.3892/mmr.2016.4948

Chiang MC, 2010, HUM MOL GENET, V19, P4043, DOI 10.1093/hmg/ddq322

Chopra H, 2021, MOLECULES, V26, DOI 10.3390/molecules26164998

Collino Massimo, 2008, Ther Adv Cardiovasc Dis, V2, P179, DOI 10.1177/1753944708090924

Estrada SM, 2020, CELL DEATH DIS, V11, DOI 10.1038/s41419-019-2196-7

Fatemi I, 2021, ACTA NEUROPSYCHIATR, V33, P15, DOI 10.1017/neu.2020.30

Gheorghe G, 2020, DIAGNOSTICS, V10, DOI 10.3390/diagnostics10070483

Gilbert F, 2006, BIOL PSYCHIAT, V60, P538, DOI 10.1016/j.biopsych.2006.04.023

Grewal AK, 2019, CAN J PHYSIOL PHARM, V97, P1094, DOI 10.1139/cjpp-2019-0188

Hodges A, 2006, HUM MOL GENET, V15, P965, DOI 10.1093/hmg/ddl013

Huang YH, 2022, P NATL ACAD SCI USA, V119, DOI 10.1073/pnas.2204828119

Isawi IH, 2020, MOLECULES, V25, DOI 10.3390/molecules25030725

Jakaria M, 2019, MOL NEUROBIOL, V56, P5799, DOI 10.1007/s12035-019-1487-4

Jamwal S, 2021, PHARMACOL THERAPEUT, V219, DOI 10.1016/j.pharmthera.2020.107705

Jetten Anton M, 2009, Nucl Recept Signal, V7, pe003, DOI 10.1621/nrs.07003

Jin LH, 2010, ADV DRUG DELIVER REV, V62, P1218, DOI 10.1016/j.addr.2010.08.007

Kalra P, 2022, NEUROCHEM RES, V47, P825, DOI 10.1007/s11064-021-03500-0

Kelly ME, 2018, PATHOL ONCOL RES, V24, P815, DOI 10.1007/s12253-018-0440-6

Khan H, 2022, MITOCHONDRION, V66, P54, DOI 10.1016/j.mito.2022.08.001

Khan H, 2022, INFLAMMOPHARMACOLOGY, V30, P2051, DOI 10.1007/s10787-022-01072-1

Khan H, 2022, NEUROCHEM RES, V47, P3543, DOI 10.1007/s11064-022-03737-3

Khan H, 2022, INT IMMUNOPHARMACOL, V110, DOI 10.1016/j.intimp.2022.108951

Khan H, 2022, NEUROSCIENCE, V491, P23, DOI 10.1016/j.neuroscience.2022.03.016

Khan H, 2022, NEUROCHEM RES, V47, P1125, DOI 10.1007/s11064-021-03521-9

Khan H, 2021, PHARMACOL REP, V73, P1240, DOI 10.1007/s43440-021-00258-8

Kiaei M, 2008, PPAR RES, V2008, DOI 10.1155/2008/418765

Landreth G, 2007, CURR ALZHEIMER RES, V4, P159, DOI 10.2174/156720507780362092

Le Gras S, 2017, SCI REP-UK, V7, DOI 10.1038/srep42875

Lecourtier L, 2007, NEUROSCI BIOBEHAV R, V31, P658, DOI 10.1016/j.neubiorev.2007.01.004

Liu QD, 2020, NEUROPSYCH DIS TREAT, V16, P2285, DOI 10.2147/NDT.S265601

Lopes JP, 2015, J NEUROCHEM, V134, P135, DOI 10.1111/jnc.13109

Lyman M, 2014, NEUROSCI RES, V79, P1, DOI 10.1016/j.neures.2013.10.004

Mahmoudi S, 2009, NEUROBIOL DIS, V36, P213, DOI 10.1016/j.nbd.2009.07.017

Mannan A, 2021, NEUROCHEM RES, V46, P2800, DOI 10.1007/s11064-021-03402-1

Marazziti D, 2004, P NATL ACAD SCI USA, V101, P10189, DOI 10.1073/pnas.0403661101

Massart R, 2009, EUR J NEUROSCI, V30, P397, DOI 10.1111/j.1460-9568.2009.06842.x

McCrary MR, 2019, FASEB J, V33, P10680, DOI 10.1096/fj.201900070R

Montarolo F, 2015, INFLAMM RES, V64, P841, DOI 10.1007/s00011-015-0871-4

Morales P, 2018, DRUG METAB REV, V50, P74, DOI 10.1080/03602532.2018.1428616

Morató X, 2021, TRANSL NEURODEGENER, V10, DOI 10.1186/s40035-021-00232-7

Mouhi S, 2022, INT J MOL SCI, V23, DOI 10.3390/ijms23074028

Nagu P, 2021, REV NEUROSCIENCE, V32, P219, DOI 10.1515/revneuro-2020-0070

Napolitano M, 2011, BRAIN RES BULL, V85, P231, DOI 10.1016/j.brainresbull.2011.03.011

Nelson CD, 2013, PLOS ONE, V8, DOI 10.1371/journal.pone.0074680

Nolan YM, 2013, TRENDS MOL MED, V19, P187, DOI 10.1016/j.molmed.2012.12.003

Obeng S, 2021, J MED CHEM, V64, P6523, DOI 10.1021/acs.jmedchem.1c00028

Prabhakar NK, 2022, INT IMMUNOPHARMACOL, V108, DOI 10.1016/j.intimp.2022.108902

Raveney BJE, 2015, NAT COMMUN, V6, DOI 10.1038/ncomms9437

Rehni AK, 2008, BASIC CLIN PHARMACOL, V103, P262, DOI 10.1111/j.1742-7843.2008.00276.x

Rehni AK, 2008, PHARMACOL REP, V60, P904

Safe S, 2016, J STEROID BIOCHEM, V157, P48, DOI 10.1016/j.jsbmb.2015.04.016

Saijo K, 2010, ADV IMMUNOL, V106, P21, DOI 10.1016/S0065-2776(10)06002-5

Saijo K, 2009, CELL, V137, P47, DOI 10.1016/j.cell.2009.01.038

Saliba SW, 2018, J NEUROINFLAMM, V15, DOI 10.1186/s12974-018-1362-7

Schütz B, 2005, J NEUROSCI, V25, P7805, DOI 10.1523/JNEUROSCI.2038-05.2005

Sharma B., 2012, International Journal of Pharmacy and Pharmaceutical Sciences, V4, P57

Sharma T, 2021, NEUROSCI LETT, V749, DOI 10.1016/j.neulet.2021.135754

Sharma VK, 2020, LIFE SCI, V262, DOI 10.1016/j.lfs.2020.118401

Sharma VK, 2020, CURR DRUG TARGETS, V21, P433, DOI 10.2174/1389450120666191017114735

Shi YH, 2007, DRUG DISCOV TODAY, V12, P440, DOI 10.1016/j.drudis.2007.04.006

Singh S, 2021, MITOCHONDRION, V58, P213, DOI 10.1016/j.mito.2021.03.009

Singh V., 2021, Alzheimers Dement., V17

Slominski AT, 2017, SCI REP-UK, V7, DOI 10.1038/s41598-017-10202-7

Slominski AT, 2014, FASEB J, V28, P2775, DOI 10.1096/fj.13-242040

Soccio RE, 2020, LIVER: BIOLOGY AND PATHOBIOLOGY, 6TH EDITION, 6 EDITION, P337

Tanaka S, 2007, J BIOL CHEM, V282, P10506, DOI 10.1074/jbc.M700911200

Tanaka S, 2014, NEUROBIOL DIS, V68, P215, DOI 10.1016/j.nbd.2014.04.007

Tanaka S, 2009, NEUROSCI RES, V65, pS92, DOI 10.1016/j.neures.2009.09.383

Tang XL, 2012, ACTA PHARMACOL SIN, V33, P363, DOI 10.1038/aps.2011.210

Thapa K, 2022, EUR J PHARMACOL, V930, DOI 10.1016/j.ejphar.2022.175133

Thapa K, 2021, J MOL NEUROSCI, V71, P1725, DOI 10.1007/s12031-021-01841-7

Thapa K, 2021, LIFE SCI, V267, DOI 10.1016/j.lfs.2020.118975

Thathiah A, 2009, SCIENCE, V323, P946, DOI 10.1126/science.1160649

Uhlenbrock K, 2002, CELL SIGNAL, V14, P941, DOI 10.1016/S0898-6568(02)00041-4

Wang K, 2008, EXP BIOL MED, V233, P496, DOI 10.3181/0708-MR-231

Watkins LR, 2020, GENES-BASEL, V11, DOI 10.3390/genes11060694

Willems S, 2021, J MED CHEM, V64, P9592, DOI 10.1021/acs.jmedchem.1c00186

Xiang XT, 2022, PROG NEURO-PSYCHOPH, V112, DOI 10.1016/j.pnpbp.2021.110423

Yan JQ, 2020, AGING-US, V12, P8107, DOI 10.18632/aging.103128

Ye N, 2019, ACS CHEM NEUROSCI, V10, P190, DOI 10.1021/acschemneuro.8b00572

Yousefi H, 2022, FRONT ONCOL, V12, DOI 10.3389/fonc.2022.777824

Zaienne D, 2022, CHEMMEDCHEM, V17, DOI 10.1002/cmdc.202200259

Zhang YJ, 2019, BIOCHEM BIOPH RES CO, V518, P59, DOI 10.1016/j.bbrc.2019.08.008

Zhao LG, 2018, GENES DIS, V5, P342, DOI 10.1016/j.gendis.2018.04.008

NR 96

TC 0

Z9 0

U1 6

U2 12

PU ELSEVIER

PI AMSTERDAM

PA RADARWEG 29, 1043 NX AMSTERDAM, NETHERLANDS

SN 1567-5769

EI 1878-1705

J9 INT IMMUNOPHARMACOL

JI Int. Immunopharmacol.

PD NOV

PY 2023

VL 124

AR 110845

DI 10.1016/j.intimp.2023.110845

EA SEP 2023

PN A

PG 9

WC Immunology; Pharmacology & Pharmacy

WE Science Citation Index Expanded (SCI-EXPANDED)

SC Immunology; Pharmacology & Pharmacy

GA T5VS5

UT WOS:001078670900001

PM 37690241

DA 2024-08-12

ER

PT J

AU Thornton, E

Vink, R

AF Thornton, Emma

Vink, Robert

TI Treatment with a Substance P Receptor Antagonist Is Neuroprotective in

the Intrastriatal 6-Hydroxydopamine Model of Early Parkinson's Disease

SO PLOS ONE

LA English

DT Article

ID NEUROKININ-3 RECEPTORS; BASAL GANGLIA; IN-VIVO; MICROGLIAL ACTIVATION;

FUNCTIONAL DEFICITS; OXIDATIVE STRESS; DOPAMINE; DEGENERATION;

ASTROCYTES; NIGRA

AB Neuroinflammation and blood brain barrier (BBB) dysfunction have been implicated in the pathogenesis of Parkinson's disease (PD). The neuropeptide substance P (SP) is an important mediator of both neuroinflammation and BBB dysfunction through its NK1 receptor in a process known as neurogenic inflammation. Increased SP content has previously been reported following 6-OHDA treatment in vitro, with the levels of SP correlating with cell death. The present study used an in vivo 6-OHDA lesion model to determine if dopaminergic degeneration was associated with increased SP in the substantia nigra and whether this degeneration could be prevented by using a SP, NK1 receptor antagonist Unilateral, intrastriatal 6-OHDA lesions were induced and SP (10 mu g/2 mu L) or the NK1 receptor antagonists, N-acetyl-L-tryptophan (2 mu L at 50 nM) or L-333,060 (2 mu L at 100 nM), administered immediately after the neurotoxin. Nigral SP content was then determined using immunohistochemical and ELISA methods, neuroinflammation and barrier integrity was assessed using Iba-1, ED-1, GFAP and albumin immunohistochemistry, while dopaminergic cell loss was assessed with tyrosine hydroxylase immunohistochemistry. Motor function in all animals was assessed using the rotarod task. Intrastriatal 6-OHDA lesioning produced an early and sustained increase in ipsilateral nigral SP content, along with a breakdown of the BBB and activation of microglia and astrocytes. Further exacerbation of SP levels accelerated disease progression, whereas NK1 receptor antagonist treatment protected dopaminergic neurons, preserved barrier integrity, reduced neuroinflammation and significantly improved motor function. We propose that neurogenic inflammation contributes to dopaminergic degeneration in early experimental PD and demonstrate that an NK1 receptor antagonist may represent a novel neuroprotective therapy.

C1 [Thornton, Emma; Vink, Robert] Univ Adelaide, Sch Med Sci, Adelaide Ctr Neurosci Res, Adelaide, SA, Australia.

C3 University of Adelaide

RP Thornton, E (corresponding author), Univ Adelaide, Sch Med Sci, Adelaide Ctr Neurosci Res, Adelaide, SA, Australia.

EM Robert.Vink@adelaide.edu.au

RI Vink, Robert/J-7351-2012; Vink, Robert/S-5616-2019; Emery,

Kitty/B-5850-2011; Speller, Camilla/D-2830-2014

OI Vink, Robert/0000-0002-4885-0667; Vink, Robert/0000-0002-4885-0667;

Emery, Kitty/0000-0002-4031-1968; Speller, Camilla/0000-0001-7128-9903

FU Neurosurgical Research Foundation, Adelaide, Australia

FX This study was funded, in part, by the Neurosurgical Research

Foundation, Adelaide, Australia. The funders had no role in study

design, data collection and analysis, decision to publish, or

preparation of the manuscript. No additional external funding received

for this study.

CR Alves G, 2008, J NEUROL, V255, P18, DOI 10.1007/s00415-008-5004-3

BANNON MJ, 1995, BRAIN RES, V695, P19, DOI 10.1016/0006-8993(95)00791-N

Bartels AL, 2008, J NEURAL TRANSM, V115, P1001, DOI 10.1007/s00702-008-0030-y

BEAL MF, 1992, FASEB J, V6, P3338, DOI 10.1096/fasebj.6.15.1464368

Bevan MD, 1996, NEUROSCIENCE, V75, P5, DOI 10.1016/0306-4522(96)00377-6

Bishop C, 2004, BRAIN RES, V1023, P126, DOI 10.1016/j.brainres.2004.07.039

Block ML, 2006, FASEB J, V20, P251, DOI 10.1096/fj.05-4553com

Blum D, 2001, PROG NEUROBIOL, V65, P135, DOI 10.1016/S0301-0082(01)00003-X

Brahmachari S, 2006, J NEUROSCI, V26, P4930, DOI 10.1523/JNEUROSCI.5480-05.2006

Brochard V, 2009, J CLIN INVEST, V119, P182, DOI 10.1172/JCI36470

Carson MJ, 2006, CLIN NEUROSCI RES, V6, P237, DOI 10.1016/j.cnr.2006.09.004

Chauhan VS, 2008, J IMMUNOL, V180, P8241, DOI 10.4049/jimmunol.180.12.8241

Chen LW, 2004, CURR DRUG TARGETS, V5, P197, DOI 10.2174/1389450043490596

Chung YC, 2010, BMB REP, V43, P225, DOI 10.5483/BMBRep.2010.43.4.225

De Ceballos ML, 1999, PEPTIDES, V20, P249, DOI 10.1016/S0196-9781(98)00177-6

Depino AM, 2003, EUR J NEUROSCI, V18, P2731, DOI 10.1111/j.1460-9568.2003.03014.x

Deumens R, 2002, EXP NEUROL, V175, P303, DOI 10.1006/exnr.2002.7891

Donkin JJ, 2009, J CEREBR BLOOD F MET, V29, P1388, DOI 10.1038/jcbfm.2009.63

FERNANDEZ A, 1994, NEUROSCIENCE, V61, P73, DOI 10.1016/0306-4522(94)90061-2

Gao HM, 2002, J NEUROCHEM, V81, P1285, DOI 10.1046/j.1471-4159.2002.00928.x

HAMM RJ, 1994, J NEUROTRAUM, V11, P187, DOI 10.1089/neu.1994.11.187

Harrison S, 2001, INT J BIOCHEM CELL B, V33, P555, DOI 10.1016/S1357-2725(01)00031-0

Helps SC, 2012, APPL IMMUNOHISTO M M, V20, P82, DOI 10.1097/PAI.0b013e31821fc8cd

Hirsch EC, 2000, J NEUROL, V247, P58, DOI 10.1007/PL00007762

Ionov ID, 2008, INT J NEUROSCI, V118, P1763, DOI 10.1080/00207450802330561

Ji KA, 2008, GLIA, V56, P1039, DOI 10.1002/glia.20677

Kortekaas R, 2005, ANN NEUROL, V57, P176, DOI 10.1002/ana.20369

Lee CS, 1996, NEUROSCIENCE, V72, P641, DOI 10.1016/0306-4522(95)00571-4

Lévesque M, 2007, NEUROSCI RES, V57, P362, DOI 10.1016/j.neures.2006.11.002

Lin RCS, 1995, NEUROREPORT, V7, P310, DOI 10.1097/00001756-199512000-00074

Liu B, 2003, J PHARMACOL EXP THER, V304, P1, DOI 10.1124/jpet.102.035048

MANTYH PW, 1989, P NATL ACAD SCI USA, V86, P5193, DOI 10.1073/pnas.86.13.5193

Marriott I, 2004, FRONT BIOSCI-LANDMRK, V9, P2153, DOI 10.2741/1377

Marti M, 2005, J NEUROCHEM, V93, P195, DOI 10.1111/j.1471-4159.2005.03015.x

MAUBORGNE A, 1983, BRAIN RES, V268, P167, DOI 10.1016/0006-8993(83)90403-1

McGeer PL, 2008, MOVEMENT DISORD, V23, P474, DOI 10.1002/mds.21751

Mosley RL, 2006, CLIN NEUROSCI RES, V6, P261, DOI 10.1016/j.cnr.2006.09.006

Nimmo AJ, 2004, NEUROPEPTIDES, V38, P40, DOI 10.1016/j.npep.2003.12.003

NISBET AP, 1995, NEUROSCIENCE, V66, P361, DOI 10.1016/0306-4522(94)00606-6

Ogura T, 2005, NEUROSCI RES, V51, P299, DOI 10.1016/j.neures.2004.12.006

Paxinos G, 1998, RAT BRAIN IN STEREOTAXIC COORDINATES, FOURTH ED., pix

REID MS, 1990, EXP BRAIN RES, V82, P293

Rite I, 2007, J NEUROCHEM, V101, P1567, DOI 10.1111/j.1471-4159.2007.04567.x

Rock RB, 2006, J NEUROIMMUNE PHARM, V1, P117, DOI 10.1007/s11481-006-9012-8

Rodriguez MC, 1998, ANN NEUROL, V44, pS175, DOI 10.1002/ana.410440726

Schwarting RKW, 1996, PROG NEUROBIOL, V49, P215, DOI 10.1016/S0301-0082(96)00015-9

Shults CW, 2004, MITOCHONDRION, V4, P641, DOI 10.1016/j.mito.2004.07.028

SIVAM SP, 1991, NEUROPEPTIDES, V18, P201, DOI 10.1016/0143-4179(91)90148-C

Takagi S, 2007, J NEURAL TRANSM, V114, P1553, DOI 10.1007/s00702-007-0790-9

Teismann P, 2004, CELL TISSUE RES, V318, P149, DOI 10.1007/s00441-004-0944-0

TENOVUO O, 1984, BRAIN RES, V303, P113, DOI 10.1016/0006-8993(84)90217-8

Thornton E, 2010, NEUROSCI LETT, V481, P64, DOI 10.1016/j.neulet.2010.06.057

Turner RJ, 2011, BRAIN RES, V1393, P84, DOI 10.1016/j.brainres.2011.03.066

UKAI M, 1995, PHARMACOL BIOCHEM BE, V51, P705, DOI 10.1016/0091-3057(95)00010-T

Vink R, 2010, NEUROTHERAPEUTICS, V7, P74, DOI 10.1016/j.nurt.2009.10.018

Wojtera M, 2005, FOLIA NEUROPATHOL, V43, P311

NR 56

TC 74

Z9 83

U1 0

U2 5

PU PUBLIC LIBRARY SCIENCE

PI SAN FRANCISCO

PA 1160 BATTERY STREET, STE 100, SAN FRANCISCO, CA 94111 USA

SN 1932-6203

J9 PLOS ONE

JI PLoS One

PD APR 2

PY 2012

VL 7

IS 4

AR e34138

DI 10.1371/journal.pone.0034138

PG 8

WC Multidisciplinary Sciences

WE Science Citation Index Expanded (SCI-EXPANDED)

SC Science & Technology - Other Topics

GA 952GU

UT WOS:000304780500034

PM 22905156

OA gold, Green Published, Green Submitted

DA 2024-08-12

ER

PT J

AU Pathak, D

Sriram, K

AF Pathak, Dhruba

Sriram, Krishnan

TI Molecular Mechanisms Underlying Neuroinflammation Elicited by

Occupational Injuries and Toxicants

SO INTERNATIONAL JOURNAL OF MOLECULAR SCIENCES

LA English

DT Review

DE Alzheimer's disease; amyotrophic lateral sclerosis; astrocytes; cell

signaling; gliosis; hydrocarbons; inflammation; immune response; metals;

microglia; multiple sclerosis; nanoparticles; neuroinflammation;

neurological disorders; neurodegenerative disease; occupational injury;

Parkinson's disease; traumatic brain injury; workplace toxicants

ID FIBRILLARY ACIDIC PROTEIN; TUMOR-NECROSIS-FACTOR; NF-KAPPA-B; TRAUMATIC

BRAIN-INJURY; CREUTZFELDT-JAKOB-DISEASE; CENTRAL-NERVOUS-SYSTEM;

LONG-TERM EXPOSURE; AMYOTROPHIC-LATERAL-SCLEROSIS; ALPHA-SYNUCLEIN

AGGREGATION; AMYLOID PRECURSOR PROTEIN

AB Occupational injuries and toxicant exposures lead to the development of neuroinflammation by activating distinct mechanistic signaling cascades that ultimately culminate in the disruption of neuronal function leading to neurological and neurodegenerative disorders. The entry of toxicants into the brain causes the subsequent activation of glial cells, a response known as 'reactive gliosis'. Reactive glial cells secrete a wide variety of signaling molecules in response to neuronal perturbations and thus play a crucial role in the progression and regulation of central nervous system (CNS) injury. In parallel, the roles of protein phosphorylation and cell signaling in eliciting neuroinflammation are evolving. However, there is limited understanding of the molecular underpinnings associated with toxicant- or occupational injury-mediated neuroinflammation, gliosis, and neurological outcomes. The activation of signaling molecules has biological significance, including the promotion or inhibition of disease mechanisms. Nevertheless, the regulatory mechanisms of synergism or antagonism among intracellular signaling pathways remain elusive. This review highlights the research focusing on the direct interaction between the immune system and the toxicant- or occupational injury-induced gliosis. Specifically, the role of occupational injuries, e.g., trips, slips, and falls resulting in traumatic brain injury, and occupational toxicants, e.g., volatile organic compounds, metals, and nanoparticles/nanomaterials in the development of neuroinflammation and neurological or neurodegenerative disease are highlighted. Further, this review recapitulates the recent advancement related to the characterization of the molecular mechanisms comprising protein phosphorylation and cell signaling, culminating in neuroinflammation.

C1 [Pathak, Dhruba; Sriram, Krishnan] NIOSH, Hlth Effects Lab Div, Morgantown, WV 26505 USA.

C3 Centers for Disease Control & Prevention - USA; National Institute for

Occupational Safety & Health (NIOSH)

RP Sriram, K (corresponding author), NIOSH, Hlth Effects Lab Div, Morgantown, WV 26505 USA.

EM kos4@cdc.gov

RI Pathak, Dhruba/N-9597-2019

OI Sriram, Krishnan/0000-0001-7005-1872; Pathak, Dhruba/0000-0002-4005-2900

FU National Institute for Occupational Safety and Health [9390BN0];

[9390KK9]

FX The National Institute for Occupational Safety and Health provided

funding through the NORA (9390BN0 to K.S.) and NTRC (number 9390KK9 to

K.S.) projects. The APC charges were waived.

CR Aguzzi A, 2009, PHYSIOL REV, V89, P1105, DOI 10.1152/physrev.00006.2009

Ajtai BM, 1998, BRAIN RES, V802, P285, DOI 10.1016/S0006-8993(98)00622-2

Albini A, 2015, INT J NANOMED, V10, P6133, DOI 10.2147/IJN.S85275

Anderson T., 2006, PRACTICAL NEUROLOGY, V6, P342, DOI DOI 10.1136/JNNP.2006.106583

Anderson V.P., 2010, CHAIN STORAGE AGE, V86, P42

Anjum R, 2008, NAT REV MOL CELL BIO, V9, P747, DOI 10.1038/nrm2509

Anthonymuthu TS, 2016, BRAIN RES, V1640, P57, DOI 10.1016/j.brainres.2016.02.006

Antonini J.M., NEUROTOXICOLOGY

Aoki T, 1999, CLIN NEUROPATHOL, V18, P51

Apolloni S, 2014, DIS MODEL MECH, V7, P1101, DOI 10.1242/dmm.017038

Arnold SE, 1996, ACTA NEUROPATHOL, V91, P269, DOI 10.1007/s004010050425

ASCHNER M, 1994, BRAIN RES BULL, V33, P345, DOI 10.1016/0361-9230(94)90204-6

ASCHNER M, 1990, BRAIN RES BULL, V24, P857, DOI 10.1016/0361-9230(90)90152-P

Astillero-Lopez V, 2022, ALZHEIMERS DEMENT, V18, P2468, DOI 10.1002/alz.12580

Bader M, 1999, INT ARCH OCC ENV HEA, V72, P521, DOI 10.1007/s004200050410

Banati RB, 1998, MOVEMENT DISORD, V13, P221, DOI 10.1002/mds.870130205

Banerjee S, 2017, DRUGS, V77, P521, DOI 10.1007/s40265-017-0701-9

Beattie EC, 2002, SCIENCE, V295, P2282, DOI 10.1126/science.1067859

Beckley JT, 2011, NEUROPSYCHOPHARMACOL, V36, P1531, DOI 10.1038/npp.2011.38

Beltrao P, 2013, MOL SYST BIOL, V9, DOI 10.1002/msb.201304521

Ben Haim L, 2017, NAT REV NEUROSCI, V18, P31, DOI [10.1038/nrn.2016.159, 10.1038/nm.2016.159]

Ben Haim Lucile, 2015, Front Cell Neurosci, V9, P278, DOI 10.3389/fncel.2015.00278

Bendotti C, 2004, J NEUROPATH EXP NEUR, V63, P113, DOI 10.1093/jnen/63.2.113

Bhattacharjee S, 2014, FRONT AGING NEUROSCI, V6, DOI 10.3389/fnagi.2014.00062

Binukumar BK, 2011, NEUROMOL MED, V13, P251, DOI 10.1007/s12017-011-8156-8

Bishop A, 2005, TOXICOLOGY, V208, P193, DOI 10.1016/j.tox.2004.11.034

Blasko I, 2000, NEUROBIOL DIS, V7, P682, DOI 10.1006/nbdi.2000.0321

Boghdadi AG, 2020, J NEUROTRAUM, V37, P681, DOI 10.1089/neu.2019.6938

BONGCAMRUDLOFF E, 1991, CANCER RES, V51, P1553

Bottcher C., ACTA NEUROPATHOL COM

Bowler RM, 2003, INT J HYG ENVIR HEAL, V206, P517, DOI 10.1078/1438-4639-00249

Bowler RM, 2007, OCCUP ENVIRON MED, V64, P167, DOI 10.1136/oem.2006.028761

Bowler RM, 2007, NEUROTOXICOLOGY, V28, P298, DOI 10.1016/j.neuro.2006.11.001

Braak H, 2000, J NEUROL, V247, P3

Braak H, 2003, NEUROBIOL AGING, V24, P197, DOI 10.1016/S0197-4580(02)00065-9

Brea D, 2022, NATURE, V602, P217, DOI 10.1038/d41586-021-03802-x

Brenner M, 2021, ASN NEURO, V13, DOI 10.1177/1759091420981206

Brettschneider J, 2012, ACTA NEUROPATHOL, V123, P395, DOI 10.1007/s00401-011-0932-x

BROCK TO, 1987, J NEUROSCI, V7, P931

Burda JE, 2016, EXP NEUROL, V275, P305, DOI 10.1016/j.expneurol.2015.03.020

Burda JE, 2014, NEURON, V81, P229, DOI 10.1016/j.neuron.2013.12.034

Butler TL, 2002, BRAIN RES, V929, P252, DOI 10.1016/S0006-8993(01)03371-6

Cagnin A, 2001, LANCET, V358, P461, DOI 10.1016/S0140-6736(01)05625-2

Calabrese V, 2007, NAT REV NEUROSCI, V8, P766, DOI 10.1038/nrn2214

Campos-Ordonez T, 2015, CELL MOL NEUROBIOL, V35, P503, DOI 10.1007/s10571-014-0146-6

Carbonell WS, 2003, J NEUROTRAUM, V20, P327, DOI 10.1089/089771503765172282

Chai H, 2017, NEURON, V95, P531, DOI 10.1016/j.neuron.2017.06.029

Chakrabarty P, 2010, J IMMUNOL, V184, P5333, DOI 10.4049/jimmunol.0903382

Chan MH, 2008, TOXICOL SCI, V101, P286, DOI 10.1093/toxsci/kfm264

CHANDRA SV, 1981, CLIN TOXICOL, V18, P407, DOI 10.3109/15563658108990264

Chen C., 2022, BIOMASS BIOENERG, V8

Chen SY, 2015, J NEUROL SCI, V351, P36, DOI 10.1016/j.jns.2015.02.020

Chen WC, 2019, J NEUROINFLAMM, V16, DOI 10.1186/s12974-019-1572-7

Cheng Ying, 2021, Oxid Med Cell Longev, V2021, P5005136, DOI 10.1155/2021/5005136

Cherry JD, 2016, ACTA NEUROPATHOL COM, V4, DOI 10.1186/s40478-016-0382-8

Cheung WM, 1999, CHINESE J PHYSIOL, V42, P227

Chin-Chan M, 2015, FRONT CELL NEUROSCI, V9, DOI 10.3389/fncel.2015.00124

Cho J, 2020, SCI TOTAL ENVIRON, V737, DOI 10.1016/j.scitotenv.2020.140097

Chou TW, 2021, CELL DEATH DIS, V12, DOI 10.1038/s41419-021-04049-0

Cicognola C, 2021, ALZHEIMERS RES THER, V13, DOI 10.1186/s13195-021-00804-9

Combs CK, 2001, J NEUROSCI, V21, P1179

Conrad ME, 2000, AM J PHYSIOL-GASTR L, V279, pG767, DOI 10.1152/ajpgi.2000.279.4.G767

Cramer T, 2022, SCI ADV, V8, DOI 10.1126/sciadv.abj0112

Crossgrove JS, 2005, NEUROTOXICOLOGY, V26, P297, DOI 10.1016/j.neuro.2004.09.004

Crossgrove JS, 2003, NEUROTOXICOLOGY, V24, P3, DOI 10.1016/S0161-813X(02)00089-X

Cua DJ, 2003, NATURE, V421, P744, DOI 10.1038/nature01355

Cullen KM, 1997, NEUROREPORT, V8, P1961, DOI 10.1097/00001756-199705260-00033

Cunningham CL, 2013, J NEUROSCI, V33, P4216, DOI 10.1523/JNEUROSCI.3441-12.2013

Curran B, 2001, NEUROSCIENCE, V108, P83, DOI 10.1016/S0306-4522(01)00405-5

D'Erchia AM, 2017, SCI REP-UK, V7, DOI 10.1038/s41598-017-10488-7

DANZIGER N, 1995, J NEUROCHEM, V64, P1016

De Crescenzo LA, 2021, INJURY EPIDEMIOL, V8, DOI 10.1186/s40621-021-00308-y

Deininger MH, 2003, J MOL NEUROSCI, V20, P25, DOI 10.1385/JMN:20:1:25

Dejanovic B, 2022, NATURE AGING, V2, P837, DOI 10.1038/s43587-022-00281-1

DeKosky ST, 1996, ANN NEUROL, V39, P123, DOI 10.1002/ana.410390118

DELACOURTE A, 1990, NEUROLOGY, V40, P33, DOI 10.1212/WNL.40.1.33

DeMaagd George, 2015, P T, V40, P504

Dihné M, 2001, BRAIN RES, V889, P165, DOI 10.1016/S0006-8993(00)03129-2

Disdier C, 2017, SCI REP-UK, V7, DOI 10.1038/s41598-017-12404-5

Doble A, 1999, PHARMACOL THERAPEUT, V81, P163, DOI 10.1016/S0163-7258(98)00042-4

Dols-Icardo O., NEUROL-NEUROIMMUNOL

DONALDSON J, 1987, NEUROTOXICOLOGY, V8, P451

Donaldson K, 2006, TOXICOL SCI, V92, P5, DOI 10.1093/toxsci/kfj130

Donat CK, 2017, FRONT AGING NEUROSCI, V9, DOI 10.3389/fnagi.2017.00208

Dorsett CR, 2017, J NEUROTRAUM, V34, P263, DOI 10.1089/neu.2015.4373

Du S, 1999, EXP NEUROL, V157, P96, DOI 10.1006/exnr.1999.7041

Dugan LL, 2009, PLOS ONE, V4, DOI 10.1371/journal.pone.0005518

Ebadi M, 2003, ANTIOXID REDOX SIGN, V5, P319, DOI 10.1089/152308603322110896

Ekdahl CT, 2003, P NATL ACAD SCI USA, V100, P13632, DOI 10.1073/pnas.2234031100

Elder A, 2006, ENVIRON HEALTH PERSP, V114, P1172, DOI 10.1289/ehp.9030

ENDOH M, 1994, BRAIN RES, V651, P92, DOI 10.1016/0006-8993(94)90683-1

Estelles A, 1996, J BIOL CHEM, V271, P14800, DOI 10.1074/jbc.271.25.14800

Faul MX., 2010, Centers for Disease Control and Prevention, National Center for Injury Prevention and Control, DOI [10.15620/cdc.5571, DOI 10.15620/CDC.5571]

Ferrer I, 1998, NEUROPATH APPL NEURO, V24, P271

Fiedorowicz A, 2001, BRAIN RES, V912, P116, DOI 10.1016/S0006-8993(01)02675-0

Filous AR, 2016, PROG NEUROBIOL, V144, P173, DOI 10.1016/j.pneurobio.2016.03.009

Finney LA, 2003, SCIENCE, V300, P931, DOI 10.1126/science.1085049

Fitsanakis VA, 2007, HUM EXP TOXICOL, V26, P295, DOI 10.1177/0960327107070496

Fixemer S, 2022, ACTA NEUROPATHOL COM, V10, DOI 10.1186/s40478-022-01342-7

Flores-Cuadrado A, 2021, NPJ PARKINSONS DIS, V7, DOI 10.1038/s41531-020-00154-7

Fong TG, 2022, NAT REV NEUROL, V18, P579, DOI 10.1038/s41582-022-00698-7

Fracassi A, 2023, BRAIN PATHOL, V33, DOI 10.1111/bpa.13108

Frakes AE, 2014, NEURON, V81, P1009, DOI 10.1016/j.neuron.2014.01.013

Frischer JM, 2009, BRAIN, V132, P1175, DOI 10.1093/brain/awp070

Frohman EM, 2006, NEW ENGL J MED, V354, P942, DOI 10.1056/NEJMra052130

Frost JL, 2016, TRENDS CELL BIOL, V26, P587, DOI 10.1016/j.tcb.2016.02.006

Fugger L, 2009, NAT REV IMMUNOL, V9, P408, DOI 10.1038/nri2554

Fukui K, 1996, NEUROSCI LETT, V203, P85, DOI 10.1016/0304-3940(95)12270-2

Furman D, 2019, NAT MED, V25, P1822, DOI 10.1038/s41591-019-0675-0

Garcia SJ, 2002, DEV BRAIN RES, V133, P151, DOI 10.1016/S0165-3806(02)00283-3

Ghosh A, 2020, SCI TRANSL MED, V12, DOI 10.1126/scitranslmed.abb1206

Glass CK, 2010, CELL, V140, P918, DOI 10.1016/j.cell.2010.02.016

GOLDGABER D, 1989, P NATL ACAD SCI USA, V86, P7606, DOI 10.1073/pnas.86.19.7606

Gómez-Nicola D, 2013, J NEUROSCI, V33, P2481, DOI 10.1523/JNEUROSCI.4440-12.2013

Gonzalez-Cuyar LF, 2014, NEUROTOXICOLOGY, V45, P260, DOI 10.1016/j.neuro.2013.12.008

Goris A, 2007, J NEUROL, V254, P846, DOI 10.1007/s00415-006-0414-6

Gotohda Takako, 2002, Leg Med (Tokyo), V4, P21, DOI 10.1016/S1344-6223(01)00058-X

Gotohda Takako, 2000, Journal of Toxicological Sciences, V25, P223

Gray F, 1999, J NEUROPATH EXP NEUR, V58, P321, DOI 10.1097/00005072-199904000-00002

Greig FH, 2017, J AM HEART ASSOC, V6, DOI 10.1161/JAHA.117.006936

Greig FH, 2014, PHARMACOL THERAPEUT, V143, P265, DOI 10.1016/j.pharmthera.2014.03.006

Griemsmann S, 2015, CEREB CORTEX, V25, P3420, DOI 10.1093/cercor/bhu157

Griffin WST, 2006, J NEUROINFLAMM, V3, DOI 10.1186/1742-2094-3-5

GRIFFIN WST, 1989, P NATL ACAD SCI USA, V86, P7611

Grubman A, 2019, NAT NEUROSCI, V22, P2087, DOI 10.1038/s41593-019-0539-4

Guentchev M, 2002, NEUROBIOL DIS, V9, P275, DOI 10.1006/nbdi.2002.0477

Guilarte TR, 2013, FRONT AGING NEUROSCI, V5, DOI 10.3389/fnagi.2013.00023

Guo TT, 2020, MOL NEURODEGENER, V15, DOI 10.1186/s13024-020-00391-7

Haik S, 2014, LANCET NEUROL, V13, P150, DOI 10.1016/S1474-4422(13)70307-7

Hansen DV, 2018, J CELL BIOL, V217, P459, DOI 10.1083/jcb.201709069

HARPIN ML, 1990, J NEUROSCI RES, V27, P587, DOI 10.1002/jnr.490270420

Harry GJ, 1996, TOXICOL APPL PHARM, V139, P84, DOI 10.1006/taap.1996.0145

Hegarty SV, 2014, MOL NEUROBIOL, V50, P559, DOI 10.1007/s12035-014-8639-3

Hensley K, 1999, J NEUROCHEM, V72, P2053, DOI 10.1046/j.1471-4159.1999.0722053.x

Hickman SE, 2008, J NEUROSCI, V28, P8354, DOI 10.1523/JNEUROSCI.0616-08.2008

Hirsch EC, 2003, ANN NY ACAD SCI, V991, P214

Hishikawa N, 2001, NEUROPATH APPL NEURO, V27, P362, DOI 10.1046/j.1365-2990.2001.00345.x

Hoesel B, 2013, MOL CANCER, V12, DOI 10.1186/1476-4598-12-86

Hoozemans JJM, 2001, EXP GERONTOL, V36, P559, DOI 10.1016/S0531-5565(00)00226-6

Hou LY, 2017, TOXICOLOGY, V380, P1, DOI 10.1016/j.tox.2017.02.009

Hristovska I, 2022, NAT COMMUN, V13, DOI 10.1038/s41467-022-34035-9

Hsiao HY, 2013, HUM MOL GENET, V22, P1826, DOI 10.1093/hmg/ddt036

Hu CF, 2021, FRONT IMMUNOL, V12, DOI 10.3389/fimmu.2021.638381

Hu XY, 2021, SIGNAL TRANSDUCT TAR, V6, DOI 10.1038/s41392-021-00791-1

Huang CL, 2015, ENVIRON RES, V136, P253, DOI 10.1016/j.envres.2014.11.006

Huang YY, 2011, J NEUROSCI, V31, P18048, DOI 10.1523/JNEUROSCI.4067-11.2011

Hubbs A.F., 2023, HUSCHEK ROUSSEAUXS H, V3

Hubbs A.F., 2016, AM J PATHOL

Huh Y, 2003, NEUROSCI LETT, V349, P63, DOI 10.1016/S0304-3940(03)00743-2

Hunot S, 1997, P NATL ACAD SCI USA, V94, P7531, DOI 10.1073/pnas.94.14.7531

Hunter DD, 1999, AM J RESP CRIT CARE, V159, P1943, DOI 10.1164/ajrccm.159.6.9808078

Huynh H, 2022, TOXICOL PATHOL, V50, P763, DOI 10.1177/01926233221107607

Islam MT, 2017, NEUROL RES, V39, P73, DOI 10.1080/01616412.2016.1251711

Isono M, 2003, NEUROL RES, V25, P546, DOI 10.1179/016164103101201814

Jain M, 2021, ANN NEUROSCI, V28, P191, DOI 10.1177/09727531211070532

Jassam YN, 2017, NEURON, V95, P1246, DOI 10.1016/j.neuron.2017.07.010

Jiménez-Jiménez FJ, 2016, CURR MED CHEM, V23, P2666, DOI 10.2174/0929867323666160812151356

Jeffrey KL, 2007, NAT REV DRUG DISCOV, V6, P391, DOI 10.1038/nrd2289

Josephs KA, 2005, NEUROLOGY, V64, P2033, DOI 10.1212/01.WNL.0000167411.93483.A1

Jurga AM, 2021, BIOMOLECULES, V11, DOI 10.3390/biom11091361

Kacimi R, 2011, J INFLAMM-LOND, V8, DOI 10.1186/1476-9255-8-7

Kanski R, 2014, CELL MOL LIFE SCI, V71, P433, DOI 10.1007/s00018-013-1435-9

Katchanov J, 2003, BRAIN PATHOL, V13, P452

Kaur C, 2014, BRAIN STRUCT FUNCT, V219, P151, DOI 10.1007/s00429-012-0491-5

Kenkhuis B., ACTA NEUROPATHOL COM

Kim YT, 2022, SCI TOTAL ENVIRON, V816, DOI 10.1016/j.scitotenv.2021.151655

Kitazawa M, 2011, J IMMUNOL, V187, P6539, DOI 10.4049/jimmunol.1100620

Klein AL, 2021, PHARMACEUTICS, V13, DOI 10.3390/pharmaceutics13070948

Koczyk D, 2000, INT J DEV NEUROSCI, V18, P591, DOI 10.1016/S0736-5748(99)00111-2

Koistinaho M., P NATL ACAD SCI USA

Konda S, 2015, INJURY PREV, V21, P115, DOI 10.1136/injuryprev-2014-041323

Konishi H, 2020, EMBO J, V39, DOI 10.15252/embj.2020104464

Konnova EA., 2018, Parkinsons disease: pathogenesis and clinical aspects

Korn T, 2009, ANNU REV IMMUNOL, V27, P485, DOI 10.1146/annurev.immunol.021908.132710

Kotas ME, 2015, CELL, V160, P816, DOI 10.1016/j.cell.2015.02.010

Krum JM, 2002, EXP NEUROL, V174, P137, DOI 10.1006/exnr.2002.7867

Kuhlmann AC, 2000, J NEUROCHEM, V74, P1694, DOI 10.1046/j.1471-4159.2000.0741694.x

Kumar A, 2021, MOL PSYCHIATR, V26, P5833, DOI 10.1038/s41380-021-01101-5

Kwon HS, 2020, TRANSL NEURODEGENER, V9, DOI 10.1186/s40035-020-00221-2

Laabbar W, 2021, J CHEM NEUROANAT, V112, DOI 10.1016/j.jchemneu.2020.101915

Lall D, 2021, NEURON, V109, P2275, DOI 10.1016/j.neuron.2021.05.020

Langston JW, 1999, ANN NEUROL, V46, P598, DOI 10.1002/1531-8249(199910)46:4<598::AID-ANA7>3.0.CO;2-F

Laskawi R, 1997, EUR ARCH OTO-RHINO-L, V254, P81, DOI 10.1007/BF01526185

Leon LR, 2002, J APPL PHYSIOL, V92, P2648, DOI 10.1152/japplphysiol.01005.2001

Levesque S, 2011, ENVIRON HEALTH PERSP, V119, P1149, DOI 10.1289/ehp.1002986

Li JX, 2012, NAT CELL BIOL, V14, P999, DOI 10.1038/ncb2562

Li T, 2013, BRAIN, V136, P3578, DOI 10.1093/brain/awt287

Li WJ, 2010, NEUROSCI BULL, V26, P205, DOI 10.1007/s12264-010-1117-7

Li XL, 2018, TOXICOL IND HEALTH, V34, P409, DOI 10.1177/0748233718758233

Li YK, 2003, J NEUROSCI, V23, P1605

Liao HY, 2022, NEUROSCIENCE, V490, P250, DOI 10.1016/j.neuroscience.2022.02.015

Liao YF, 2004, J BIOL CHEM, V279, P49523, DOI 10.1074/jbc.M402034200

Lin BW, 1998, ACTA NEUROPATHOL, V95, P511, DOI 10.1007/s004010050832

Lin NH, 2021, FASEB J, V35, DOI 10.1096/fj.202100087R

Liu B, 2002, ANN NY ACAD SCI, V962, P318, DOI 10.1111/j.1749-6632.2002.tb04077.x

Liu HY, 2018, ONCOTARGET, V9, P2887, DOI [10.18632/oncotarget.20812, 10.18632/oncotarget.23208]

Liu LR, 2020, FRONT IMMUNOL, V11, DOI 10.3389/fimmu.2020.01024

Liu T, 2017, SIGNAL TRANSDUCT TAR, V2, DOI 10.1038/sigtrans.2017.23

Liu Y, 2019, ACCOUNTS CHEM RES, V52, P2026, DOI 10.1021/acs.accounts.9b00248

Liu Y, 2022, TOXICOL APPL PHARM, V451, DOI 10.1016/j.taap.2022.116190

Liu YD, 2015, CRIT REV IMMUNOL, V35, P505, DOI 10.1615/CritRevImmunol.2016015517

Llorens F, 2014, FRONT AGING NEUROSCI, V6, DOI 10.3389/fnagi.2014.00198

Lovell MA, 1998, J NEUROL SCI, V158, P47, DOI 10.1016/S0022-510X(98)00092-6

Lu KT, 2005, NEUROSCI LETT, V386, P40, DOI 10.1016/j.neulet.2005.05.057

Lucchini RG, 2007, AM J IND MED, V50, P788, DOI 10.1002/ajim.20494

Macchi ZA, 2022, J NEUROL SCI, V442, DOI 10.1016/j.jns.2022.120420

Madathil SK, 2018, FRONT NEUROL, V9, DOI 10.3389/fneur.2018.00964

Maniatis S, 2019, SCIENCE, V364, P89, DOI 10.1126/science.aav9776

MARTIN PM, 1995, J NEUROSCI METH, V58, P181, DOI 10.1016/0165-0270(94)00175-G

Mastrangelo L, 2017, ADV GENET, V98, P43, DOI 10.1016/bs.adgen.2017.08.001

Mastroeni D, 2018, NEUROBIOL AGING, V63, P12, DOI 10.1016/j.neurobiolaging.2017.10.019

Mayo Clinic, 2011, MC1298REV0111, pmc1298

McGeer PL, 2002, MUSCLE NERVE, V26, P459, DOI 10.1002/mus.10191

MCGEER PL, 1988, NEUROLOGY, V38, P1285, DOI 10.1212/WNL.38.8.1285

Mercer RR, 2013, PART FIBRE TOXICOL, V10, DOI 10.1186/1743-8977-10-38

MERGLER D, 1994, ENVIRON RES, V64, P151, DOI 10.1006/enrs.1994.1013

Mergler D, 1997, ENVIRON RES, V73, P92, DOI 10.1006/enrs.1997.3710

MERRILL JE, 1991, DEV NEUROSCI-BASEL, V13, P130, DOI 10.1159/000112150

Messing A, 2020, ASN NEURO, V12, DOI 10.1177/1759091420949680

Minter MR, 2016, SCI REP-UK, V6, DOI 10.1038/srep30028

Montes S, 2019, BRAIN RES BULL, V144, P58, DOI 10.1016/j.brainresbull.2018.11.007

Murakami M, 2012, FRONT IMMUNOL, V3, DOI 10.3389/fimmu.2012.00323

Muzio L, 2021, FRONT NEUROSCI-SWITZ, V15, DOI 10.3389/fnins.2021.742065

Narayanan SE, 2020, ENVIRON SCI POLLUT R, V27, P43542, DOI 10.1007/s11356-020-10477-w

NASKALI L, 1994, NEUROTOXICOLOGY, V15, P609

National Institute for Occupational Safety and Health (NIOSH), 2010, DHHS NIOSH PUBLICATI, V2010-116

Neidert N, 2018, FRONT IMMUNOL, V9, DOI 10.3389/fimmu.2018.01728

Netea MG, 2017, NAT IMMUNOL, V18, P826, DOI 10.1038/ni.3790

NIOSH, 2012, DHHS NIOSH PUBLICATI

Nourshargh S, 2014, IMMUNITY, V41, P694, DOI 10.1016/j.immuni.2014.10.008

Nowacki P, 2019, FOLIA NEUROPATHOL, V57, P249, DOI 10.5114/fn.2019.88453

O'Callaghan James P, 2005, Expert Opin Drug Saf, V4, P433, DOI 10.1517/14740338.4.3.433

O'Callaghan JP, 2004, J NEUROSCI METH, V135, P159, DOI 10.1016/j.jneumeth.2003.12.006

O'Shea JJ, 2008, IMMUNITY, V28, P477, DOI 10.1016/j.immuni.2008.03.002

Oberdörster G, 2002, J TOXICOL ENV HEAL A, V65, P1531, DOI 10.1080/00984100290071658

Oberdörster G, 2004, INHAL TOXICOL, V16, P437, DOI 10.1080/08958370490439597

Obst J, 2017, FRONT AGING NEUROSCI, V9, DOI 10.3389/fnagi.2017.00207

Oeckinghaus A, 2009, CSH PERSPECT BIOL, V1, DOI 10.1101/cshperspect.a000034

Ogbodo JO, 2022, FRONT IMMUNOL, V13, DOI 10.3389/fimmu.2022.928379

Ojala JO, 2008, J NEUROIMMUNOL, V205, P86, DOI 10.1016/j.jneuroim.2008.09.012

Olanow CW, 1996, NEUROLOGY, V46, P492, DOI 10.1212/WNL.46.2.492

ONeill LAJ, 1997, TRENDS NEUROSCI, V20, P252, DOI 10.1016/S0166-2236(96)01035-1

Opris RV, 2022, NANOMATERIALS-BASEL, V12, DOI 10.3390/nano12010058

Osborn LM, 2016, PROG NEUROBIOL, V144, P121, DOI 10.1016/j.pneurobio.2016.01.001

PANTER SS, 1985, NEUROCHEM RES, V10, P1567, DOI 10.1007/BF00988599

Paolicelli RC, 2011, SCIENCE, V333, P1456, DOI 10.1126/science.1202529

Park KM, 2010, CELL SIGNAL, V22, P977, DOI 10.1016/j.cellsig.2010.01.010

Park RM, 2005, AM J IND MED, V48, P63, DOI 10.1002/ajim.20178

Park RM, 2006, NEUROTOXICOLOGY, V27, P373, DOI 10.1016/j.neuro.2005.10.010

Park RM, 2013, SAF HEALTH WORK, V4, P123

Patchin ES, 2016, ENVIRON HEALTH PERSP, V124, P1870, DOI 10.1289/EHP234

Pek S, 2017, ACCIDENT ANAL PREV, V106, P202, DOI 10.1016/j.aap.2017.06.007

Peterson AB, 2022, J SAFETY RES, V83, P419, DOI 10.1016/j.jsr.2022.10.001

Petzold A, 2002, BRAIN, V125, P1462, DOI 10.1093/brain/awf165

Pezzoli G, 2000, NEUROLOGY, V55, P667, DOI 10.1212/WNL.55.5.667

Pezzoli G, 1996, ANN NEUROL, V40, P922, DOI 10.1002/ana.410400616

Pickering M, 2005, EXP PHYSIOL, V90, P663, DOI 10.1113/expphysiol.2005.030734

Platt B, 2001, BRAIN RES BULL, V55, P257, DOI 10.1016/S0361-9230(01)00511-1

Postolache TT, 2020, J ALZHEIMERS DIS, V74, P1, DOI 10.3233/JAD-191150

Quintanilla RA, 2004, EXP CELL RES, V295, P245, DOI 10.1016/j.yexcr.2004.01.002

Racette BA, 2005, NEUROLOGY, V64, P230, DOI 10.1212/01.WNL.0000149511.19487.44

Racette BA, 2001, NEUROLOGY, V56, P8, DOI 10.1212/WNL.56.1.8

Racette BA, 2017, NEUROLOGY, V88, P344, DOI 10.1212/WNL.0000000000003533

Radu R, 2022, FRONT ONCOL, V12, DOI 10.3389/fonc.2022.859247

Rahimi RA, 2018, ADV IMMUNOL, V138, P71, DOI 10.1016/bs.ai.2018.02.002

Rakers C, 2019, GLIA, V67, P619, DOI 10.1002/glia.23544

Ramos-García NA, 2020, INT J MOL SCI, V21, DOI 10.3390/ijms21061983

Ravula AR, 2022, EXP NEUROL, V349, DOI 10.1016/j.expneurol.2021.113938

Rawji KS, 2020, TRENDS NEUROSCI, V43, P596, DOI 10.1016/j.tins.2020.05.006

Reaney SH, 2006, TOXICOL SCI, V93, P114, DOI 10.1093/toxsci/kfl028

Renz BW, 2018, CANCER CELL, V34, P863, DOI 10.1016/j.ccell.2018.10.010

Richard AM, 2021, CHEM RES TOXICOL, V34, P189, DOI 10.1021/acs.chemrestox.0c00264

Riegel AC, 2007, NEUROPSYCHOPHARMACOL, V32, P1558, DOI 10.1038/sj.npp.1301273

Ringheim GE, 1998, MOL BRAIN RES, V55, P35, DOI 10.1016/S0169-328X(97)00356-2

Rodier J, 1955, BRIT J IND MED, V12, P21

ROELS H, 1987, AM J IND MED, V11, P307, DOI 10.1002/ajim.4700110308

ROSENGREN LE, 1985, SCAND J WORK ENV HEA, V11, P447, DOI 10.5271/sjweh.2201

ROSENGREN LE, 1986, BRIT J IND MED, V43, P291

Rossi F, 1996, BIOCHEM BIOPH RES CO, V225, P474, DOI 10.1006/bbrc.1996.1197

Roth TL, 2014, NATURE, V505, P223, DOI 10.1038/nature12808

Rothhammer V, 2019, NAT REV IMMUNOL, V19, P184, DOI 10.1038/s41577-019-0125-8

Ruiz-Hernandez A, 2015, CLIN EPIGENETICS, V7, DOI 10.1186/s13148-015-0055-7

Sabolek MK, 2019, ANN CLIN TRANSL NEUR, V6, P1151, DOI 10.1002/acn3.783

Safar JG, 2012, PRION, V6, P108, DOI 10.4161/pri.18666

Samiei F, 2020, ENVIRON SCI POLLUT R, V27, P12096, DOI 10.1007/s11356-020-07740-5

Schirmer L, 2019, NATURE, V573, P75, DOI 10.1038/s41586-019-1404-z

Schwarz SC, 1998, NEUROREPORT, V9, P3029, DOI 10.1097/00001756-199809140-00020

Scott G, 2018, BRAIN, V141, P459, DOI 10.1093/brain/awx339

Sears JM, 2013, J OCCUP ENVIRON MED, V55, P507, DOI 10.1097/JOM.0b013e31827ee018

Sedgwick JD, 2000, IMMUNOL TODAY, V21, P110, DOI 10.1016/S0167-5699(99)01573-X

Seif F, 2017, CELL COMMUN SIGNAL, V15, DOI 10.1186/s12964-017-0177-y

SELMAJ KW, 1990, J IMMUNOL, V144, P129

Seok SH, 2021, LIFE-BASEL, V11, DOI 10.3390/life11090957

Sheng JG, 2000, EXP NEUROL, V163, P388, DOI 10.1006/exnr.2000.7393

Sherer TB, 2003, NEUROSCI LETT, V341, P87, DOI 10.1016/S0304-3940(03)00172-1

Shi J, 2012, TOXICOL IND HEALTH, V28, P10, DOI 10.1177/0748233711401264

Shi XJ, 2021, NAT COMMUN, V12, DOI 10.1038/s41467-021-27248-x

Sian-Hülsmann J, 2011, J NEUROCHEM, V118, P939, DOI 10.1111/j.1471-4159.2010.07132.x

Simon DW, 2017, NAT REV NEUROL, V13, P572, DOI 10.1038/nrneurol.2017.116

Singh AK, 2011, TOXICOL SCI, V122, P526, DOI 10.1093/toxsci/kfr115

Singh-Bains MK, 2019, NEUROBIOL DIS, V132, DOI 10.1016/j.nbd.2019.104589

Sircar E, 2021, ARCH BIOCHEM BIOPHYS, V704, DOI 10.1016/j.abb.2021.108869

SJOGREN U, 1990, J ENDODONT, V16, P498

Smith HL, 2020, NEURON, V105, P855, DOI 10.1016/j.neuron.2019.12.014

Sofroniew MV, 2014, NEUROSCIENTIST, V20, P160, DOI 10.1177/1073858413504466

Sofroniew MV, 2009, TRENDS NEUROSCI, V32, P638, DOI 10.1016/j.tins.2009.08.002

Soltys Z, 2003, BRAIN RES, V987, P186, DOI 10.1016/S0006-8993(03)03327-4

Spiller KJ, 2018, NAT NEUROSCI, V21, P329, DOI 10.1038/s41593-018-0083-7

Spooren A, 2011, BRAIN RES REV, V67, P157, DOI 10.1016/j.brainresrev.2011.01.002

Sriram K, 2006, J NEUROCHEM, V96, P706, DOI 10.1111/j.1471-4159.2005.03566.x

Sriram K, 2004, J BIOL CHEM, V279, P19936, DOI 10.1074/jbc.M309304200

Sriram K, 2002, FASEB J, V16, P1474, DOI 10.1096/fj.02-0216fje

Sriram K., 2005, ROLE GLIA NEUROTOXIC, P141

Sriram K, 2006, FASEB J, V20, P670, DOI 10.1096/fj.05-5106com

Sriram K, 2020, TOXICOL APPL PHARM, V409, DOI 10.1016/j.taap.2020.115300

Sriram K, 2015, TOXICOLOGY, V328, P168, DOI 10.1016/j.tox.2014.12.015

Sriram K, 2014, INHAL TOXICOL, V26, P720, DOI 10.3109/08958378.2014.954654

Sriram K, 2012, TOXICOLOGY, V291, P73, DOI 10.1016/j.tox.2011.10.021

Sriram K, 2011, J TOXICOL ENV HEAL A, V74, P1405, DOI 10.1080/15287394.2011.606796

Sriram K, 2010, FASEB J, V24, P4989, DOI 10.1096/fj.10-163964

Sriram K, 2010, ARCH TOXICOL, V84, P521, DOI 10.1007/s00204-010-0525-9

Steinhäuser C, 2016, NEUROSCIENCE, V323, P157, DOI 10.1016/j.neuroscience.2014.12.047

Stellwagen D, 2006, NATURE, V440, P1054, DOI 10.1038/nature04671

Stellwagen D, 2005, J NEUROSCI, V25, P3219, DOI 10.1523/JNEUROSCI.4486-04.2005

Stoeck K, 2006, J NEUROIMMUNOL, V172, P175, DOI 10.1016/j.jneuroim.2005.10.008

Sugiyama M, 2013, NEUROPATHOLOGY, V33, P591, DOI 10.1111/neup.12027

Sutinen EM, 2012, J NEUROINFLAMM, V9, DOI 10.1186/1742-2094-9-199

Sutterwala FS, 2014, ANN NY ACAD SCI, V1319, P82, DOI 10.1111/nyas.12458

Svenson DW, 2022, J APPL TOXICOL, V42, P1168, DOI 10.1002/jat.4285

Sweeney MD, 2018, NAT REV NEUROL, V14, P133, DOI 10.1038/nrneurol.2017.188

Szpak GM, 2006, FOLIA NEUROPATHOL, V44, P202

Tachida Y, 2008, J NEUROCHEM, V104, P1387, DOI 10.1111/j.1471-4159.2007.05127.x

Takeuchi H, 2006, J BIOL CHEM, V281, P21362, DOI 10.1074/jbc.M600504200

Takizawa T, 2001, DEV CELL, V1, P749, DOI 10.1016/S1534-5807(01)00101-0

Tam OH, 2019, CELL REP, V29, P1164, DOI 10.1016/j.celrep.2019.09.066

Tassoni A, 2019, SCI REP-UK, V9, DOI 10.1038/s41598-019-46232-6

Terai K, 1996, BRAIN RES, V735, P159

Terry DP, 2018, PLOS ONE, V13, DOI 10.1371/journal.pone.0198128

Tesseur I, 2017, J NEUROSCI, V37, P4584, DOI 10.1523/JNEUROSCI.2952-16.2017

Thiruvengadam M, 2021, MICROMACHINES-BASEL, V12, DOI 10.3390/mi12121502

Thomas RS, 2018, ALTEX-ALTERN ANIM EX, V35, P163, DOI 10.14573/altex.1803011

Thomassen Y, 2001, J ENVIRON MONITOR, V3, P555, DOI 10.1039/b104479f

Thompson KH, 2003, SCIENCE, V300, P936, DOI 10.1126/science.1083004

Tiesman HM, 2011, AM J PREV MED, V41, P61, DOI 10.1016/j.amepre.2011.03.007

Tjalkens Ronald B, 2017, Adv Neurobiol, V18, P159, DOI 10.1007/978-3-319-60189-2_8

Tortarolo M, 2003, MOL CELL NEUROSCI, V23, P180, DOI 10.1016/S1044-7431(03)00022-8

Trager U, 2015, NEUROBIOL DIS, V73, P388, DOI 10.1016/j.nbd.2014.10.012

Trapp BD, 2008, ANNU REV NEUROSCI, V31, P247, DOI 10.1146/annurev.neuro.30.051606.094313

TROOST D, 1992, ACTA NEUROPATHOL, V84, P664, DOI 10.1007/BF00227744

Tsang AHK, 2009, BBA-MOL BASIS DIS, V1792, P643, DOI 10.1016/j.bbadis.2008.12.006

Turner MR, 2004, NEUROBIOL DIS, V15, P601, DOI 10.1016/j.nbd.2003.12.012

Tyburski AL, 2017, J HEADACHE PAIN, V18, DOI 10.1186/s10194-017-0726-1

Uttley L, 2020, LANCET INFECT DIS, V20, pE2, DOI 10.1016/S1473-3099(19)30615-2

Vaccari C, 2019, J TOXICOL ENV HEAL B, V22, P172, DOI 10.1080/10937404.2019.1659197

Vallabhapurapu S, 2009, ANNU REV IMMUNOL, V27, P693, DOI 10.1146/annurev.immunol.021908.132641

van Asperen JV, 2022, ASN NEURO, V14, DOI 10.1177/17590914221102065

Van Everbroeck B, 2004, ACTA NEUROPATHOL, V108, P194, DOI 10.1007/s00401-004-0879-2

Varacallo M., 2022, STATPEARLS

Vargas MR, 2008, J NEUROSCI, V28, P13574, DOI 10.1523/JNEUROSCI.4099-08.2008

Varlamova EG, 2021, INT J MOL SCI, V22, DOI 10.3390/ijms222312825

Vawter MP, 1996, EXP NEUROL, V142, P313, DOI 10.1006/exnr.1996.0200

Verboon LN, 2021, FRONT IMMUNOL, V12, DOI 10.3389/fimmu.2021.620698

Verina T, 2013, TOXICOL LETT, V217, P177, DOI 10.1016/j.toxlet.2012.12.006

Versijpt J.J., EUR NEUROL

Vezér T, 2007, AM J IND MED, V50, P841, DOI 10.1002/ajim.20485

VIJAYAN VK, 1991, EXP NEUROL, V112, P72, DOI 10.1016/0014-4886(91)90115-S

Wakabayashi K, 2000, ACTA NEUROPATHOL, V99, P14, DOI 10.1007/PL00007400

Wake H, 2009, J NEUROSCI, V29, P3974, DOI 10.1523/JNEUROSCI.4363-08.2009

Walker DG, 2017, FRONT AGING NEUROSCI, V9, DOI 10.3389/fnagi.2017.00244

Walton JR, 2009, J INORG BIOCHEM, V103, P1548, DOI 10.1016/j.jinorgbio.2009.07.027

Wang C., 2022, NAT COMMUN, V1969

Wang HX, 2016, J NEUROPATH EXP NEUR, V75, P183, DOI 10.1093/jnen/nlv019

Wang T, 2021, FOOD CHEM TOXICOL, V157, DOI 10.1016/j.fct.2021.112550

Wang Y, 2011, TOXICOL LETT, V205, P26, DOI 10.1016/j.toxlet.2011.05.001

Watson N, 2022, EUR J NEUROL, V29, P1222, DOI 10.1111/ene.15228

WEISS JH, 1993, NEURON, V10, P43, DOI 10.1016/0896-6273(93)90240-R

Wierzba-Bobrowicz T., 2002, FOLIA NEUROPATHOL, V40

Wilkaniec A, 2021, FRONT AGING NEUROSCI, V13, DOI 10.3389/fnagi.2021.591475

Wilson CM, 2001, NEUROLOGY, V57, P651, DOI 10.1212/WNL.57.4.651

Witcher KG, 2021, J NEUROSCI, V41, P1597, DOI 10.1523/JNEUROSCI.2469-20.2020

Witcher KG, 2015, TRENDS NEUROSCI, V38, P609, DOI 10.1016/j.tins.2015.08.002

Wojtulewicz K, 2020, INT J MOL SCI, V21, DOI 10.3390/ijms21186939

World Health Organization, 2017, Global action plan on the public health response to dementia 20172025, DOI DOI 10.1016/S1473-3099(09)70222-1

Wu T, 2019, CELL REP, V28, P2111, DOI 10.1016/j.celrep.2019.07.060

Yamaguchi H, 2002, ARCH ENVIRON HEALTH, V57, P232, DOI 10.1080/00039890209602942

YAMAGUCHI H, 1987, ACTA NEUROPATHOL, V72, P214, DOI 10.1007/BF00691092

Yan ZQ, 2018, CLIN IMMUNOL, V189, P4, DOI 10.1016/j.clim.2016.09.014

Yang JH, 2021, CELLS-BASEL, V10, DOI 10.3390/cells10102647

Yang ZH, 2015, TRENDS NEUROSCI, V38, P364, DOI 10.1016/j.tins.2015.04.003

Yeh H, 2019, TRENDS MOL MED, V25, P96, DOI 10.1016/j.molmed.2018.11.004

Yermakova AV, 1999, J NEUROPATH EXP NEUR, V58, P1135, DOI 10.1097/00005072-199911000-00003

Yu LT, 2020, FRONT CELL NEUROSCI, V14, DOI 10.3389/fncel.2020.00211

Yuan X, 2019, PART FIBRE TOXICOL, V16, DOI 10.1186/s12989-019-0299-z

Zanusso G., ANN NEUROL

Zatta P, 2009, TRENDS PHARMACOL SCI, V30, P346, DOI 10.1016/j.tips.2009.05.002

Ze YG, 2014, PLOS ONE, V9, DOI 10.1371/journal.pone.0092230

Zerr I, 2022, NEW ENGL J MED, V386, P1345, DOI 10.1056/NEJMra2119323

Zhang B, 2019, FRONT CELL NEUROSCI, V12, DOI 10.3389/fncel.2018.00531

Zhang C, 2018, CELL DEATH DIS, V9, DOI 10.1038/s41419-017-0091-7

Zhang DD, 2021, J NEUROINFLAMM, V18, DOI 10.1186/s12974-020-02065-z

Zhang G, 2013, NATURE, V497, P211, DOI 10.1038/nature12143

Zhang L, 2006, PHARMACOL THERAPEUT, V109, P33, DOI 10.1016/j.pharmthera.2005.05.007

Zhang Q, 2011, INT J IMMUNOPATH PH, V24, P79

Zhang W, 2002, CELL RES, V12, P9, DOI 10.1038/sj.cr.7290105

Zhao F, 2009, TOXICOL SCI, V107, P156, DOI 10.1093/toxsci/kfn213

Zhao J, 2011, J NEUROINFLAMM, V8, DOI 10.1186/1742-2094-8-150

Zhao YH, 2014, MOL NEUROBIOL, V50, P97, DOI 10.1007/s12035-013-8595-3

Zheng W, 2012, PHARMACOL THERAPEUT, V133, P177, DOI 10.1016/j.pharmthera.2011.10.006

Zhong Y., 2022, NATURE, V231

Zhu W, 2003, J GERONTOL A-BIOL, V58, P117

Zucconi GG, 2002, J COMP NEUROL, V446, P135, DOI 10.1002/cne.10197

US

NR 412

TC 22

Z9 22

U1 1

U2 9

PU MDPI

PI BASEL

PA ST ALBAN-ANLAGE 66, CH-4052 BASEL, SWITZERLAND

EI 1422-0067

J9 INT J MOL SCI

JI Int. J. Mol. Sci.

PD FEB

PY 2023

VL 24

IS 3

AR 2272

DI 10.3390/ijms24032272

PG 47

WC Biochemistry & Molecular Biology; Chemistry, Multidisciplinary

WE Science Citation Index Expanded (SCI-EXPANDED)

SC Biochemistry & Molecular Biology; Chemistry

GA 8U0AD

UT WOS:000929612600001

PM 36768596

OA Green Published, gold

DA 2024-08-12

ER

PT J

AU Russo, C

Valle, MS

Russo, A

Malaguarnera, L

AF Russo, Cristina

Valle, Maria Stella

Russo, Antonella

Malaguarnera, Lucia

TI The Interplay between Ghrelin and Microglia in Neuroinflammation:

Implications for Obesity and neurodegenerative disease

SO INTERNATIONAL JOURNAL OF MOLECULAR SCIENCES

LA English

DT Review

DE Ghrelin; Ghrelin receptor; microglia; inflammation; neuroinflammation;

neurodegenerative disease; Alzheimer's disease; Parkinson's disease

ID DIET-INDUCED OBESITY; BLOOD-BRAIN-BARRIER; DES-ACYL GHRELIN;

alzheimer's disease; parkinson's disease; SECRETAGOGUE RECEPTOR; MOUSE

MODEL; INSULIN; LEPTIN; ACTIVATION

AB Numerous studies have shown that microglia are capable of producing a wide range of chemokines to promote inflammatory processes within the central nervous system (CNS). These cells share many phenotypical and functional characteristics with macrophages, suggesting that microglia participate in innate immune responses in the brain. Neuroinflammation induces neurometabolic alterations and increases in energy consumption. Microglia may constitute an important therapeutic target in neuroinflammation. Recent research has attempted to clarify the role of Ghre signaling in microglia on the regulation of energy balance, obesity, neuroinflammation and the occurrence of neurodegenerative disease. These studies strongly suggest that Ghre modulates microglia activity and thus affects the pathophysiology of neurodegenerative disease. This review aims to summarize what is known from the current literature on the way in which Ghre modulates microglial activity during neuroinflammation and their impact on neurometabolic alterations in neurodegenerative disease. Understanding the role of Ghre in microglial activation/inhibition regulation could provide promising strategies for downregulating neuroinflammation and consequently for diminishing negative neurological outcomes.

C1 [Russo, Cristina; Malaguarnera, Lucia] Univ Catania, Sch Med, Dept Biomed & Biotechnol Sci, Sect Pathol, I-95123 Catania, Italy.

[Valle, Maria Stella] Univ Catania, Lab Neurobiomech, Dept Biomed & Biotechnol Sci, Sch Med, I-95123 Catania, Italy.

[Valle, Maria Stella; Russo, Antonella] Univ Catania, Dept Biomed & Biotechnol Sci, Sect Physiol, I-95123 Catania, Italy.

C3 University of Catania; University of Catania; University of Catania

RP Malaguarnera, L (corresponding author), Univ Catania, Sch Med, Dept Biomed & Biotechnol Sci, Sect Pathol, I-95123 Catania, Italy.

EM lucmal@unict.it

RI valle, maria/KII-1654-2024; Malaguarnera, Lucia/K-5530-2018

OI VALLE, Maria Stella/0000-0001-9728-3542; MALAGUARNERA,

Lucia/0000-0002-5171-7963; russo, antonella/0000-0002-1282-1629

CR Allen NJ, 2018, SCIENCE, V362, P181, DOI 10.1126/science.aat0473

Alvarez-Crespo M, 2012, PLOS ONE, V7, DOI 10.1371/journal.pone.0046321

Baatar D, 2011, MOL CELL ENDOCRINOL, V340, P44, DOI 10.1016/j.mce.2011.04.019

Ballard C, 2011, LANCET, V377, P1019, DOI 10.1016/S0140-6736(10)61349-9

Banerjee J, 2022, INT J MOL SCI, V23, DOI 10.3390/ijms23126380

Banks WA, 2002, J PHARMACOL EXP THER, V302, P822, DOI 10.1124/jpet.102.034827

Bayliss JA, 2016, J NEUROCHEM, V137, P460, DOI 10.1111/jnc.13576

Beach TG, 2020, PLOS ONE, V15, DOI 10.1371/journal.pone.0231720

Bomfim TR, 2012, J CLIN INVEST, V122, P1339, DOI 10.1172/JCI57256

Briggs DI, 2010, ENDOCRINOLOGY, V151, P4745, DOI 10.1210/en.2010-0556

Broglio F, 2003, J CLIN ENDOCR METAB, V88, P4268, DOI 10.1210/jc.2002-021940

Bulgarelli I, 2009, J NEUROSCI RES, V87, P2718, DOI 10.1002/jnr.22088

Burguillos MA, 2011, NATURE, V472, P319, DOI 10.1038/nature09788

Cai WJ, 2012, P NATL ACAD SCI USA, V109, P15888, DOI 10.1073/pnas.1205847109

Cecarini V, 2016, MOL NEUROBIOL, V53, P3168, DOI 10.1007/s12035-015-9227-x

Chaney A, 2019, J NEUROCHEM, V149, P438, DOI 10.1111/jnc.14615

Chang LL, 2019, LIFE SCI, V232, DOI 10.1016/j.lfs.2019.116654

Cheyuo C, 2011, SHOCK, V35, P258, DOI 10.1097/SHK.0b013e3181f48a37

Chhor V, 2013, BRAIN BEHAV IMMUN, V32, P70, DOI 10.1016/j.bbi.2013.02.005

Chorny A, 2008, J IMMUNOL, V180, P8369, DOI 10.4049/jimmunol.180.12.8369

Chow KBS, 2012, MOL CELL ENDOCRINOL, V348, P247, DOI 10.1016/j.mce.2011.08.034

Chung H, 2018, ENDOCR J, V65, P63, DOI 10.1507/endocrj.EJ17-0281

Collins RF, 2009, J BIOL CHEM, V284, P30288, DOI 10.1074/jbc.M109.045104

Cope EC, 2018, J NEUROSCI, V38, P8889, DOI 10.1523/JNEUROSCI.0789-18.2018

Cowley MA, 2003, NEURON, V37, P649, DOI 10.1016/S0896-6273(03)00063-1

Cui HX, 2017, NAT REV ENDOCRINOL, V13, P338, DOI 10.1038/nrendo.2016.222

Cui WG, 2020, FRONT NEUROSCI-SWITZ, V14, DOI 10.3389/fnins.2020.00444

De Luca SN, 2019, BRAIN BEHAV IMMUN, V77, P77, DOI 10.1016/j.bbi.2018.12.008

De Sousa VL, 2021, BRAIN BEHAV IMMUN, V95, P287, DOI 10.1016/j.bbi.2021.04.001

Delhanty PJD, 2010, AM J PHYSIOL-ENDOC M, V299, pE497, DOI 10.1152/ajpendo.00414.2009

Delporte C, 2013, SCIENTIFICA, V2013, DOI 10.1155/2013/518909

Deng B, 2015, INT J CLIN EXP MED, V8, P579

Dhurandhar EJ, 2013, PLOS ONE, V8, DOI 10.1371/journal.pone.0060437

Diano S, 2006, NAT NEUROSCI, V9, P381, DOI 10.1038/nn1656

Dinel AL, 2011, PLOS ONE, V6, DOI 10.1371/journal.pone.0024325

Ding SJ, 2019, BIOMOLECULES, V9, DOI 10.3390/biom9120850

Dixit VD, 2005, EXP GERONTOL, V40, P900, DOI 10.1016/j.exger.2005.09.003

Dixit VD, 2004, J CLIN INVEST, V114, P57, DOI 10.1172/JCI200421134

Dong XY, 2009, PEPTIDES, V30, P1203, DOI 10.1016/j.peptides.2009.03.001

Erdmann J, 2005, DIABETES, V54, P1371, DOI 10.2337/diabetes.54.5.1371

Fan Z, 2015, ALZHEIMERS DEMENT, V11, P608, DOI 10.1016/j.jalz.2014.06.016

FEARNLEY JM, 1991, BRAIN, V114, P2283, DOI 10.1093/brain/114.5.2283

Ferens DM, 2010, CLIN EXP PHARMACOL P, V37, P926, DOI 10.1111/j.1440-1681.2010.05409.x

Ferreira-Marques M, 2021, AGING-US, V13, P7872, DOI 10.18632/aging.202805

Fiszer U, 2010, ACTA NEUROL SCAND, V121, P230, DOI 10.1111/j.1600-0404.2009.01185.x

Fujita Y, 2019, FRONT NEUROSCI-SWITZ, V13, DOI 10.3389/fnins.2019.00828

Gao HM, 2002, J NEUROCHEM, V81, P1285, DOI 10.1046/j.1471-4159.2002.00928.x

Granado M, 2005, AM J PHYSIOL-ENDOC M, V288, pE486, DOI 10.1152/ajpendo.00196.2004

Gregor MF, 2011, ANNU REV IMMUNOL, V29, P415, DOI 10.1146/annurev-immunol-031210-101322

Haruwaka K, 2019, NAT COMMUN, V10, DOI 10.1038/s41467-019-13812-z

Hattori N, 2001, J CLIN ENDOCR METAB, V86, P4284, DOI 10.1210/jc.86.9.4284

Heneka MT, 2015, LANCET NEUROL, V14, P388, DOI 10.1016/S1474-4422(15)70016-5

Hirosumi J, 2002, NATURE, V420, P333, DOI 10.1038/nature01137

Hosoda H, 2000, BIOCHEM BIOPH RES CO, V279, P909, DOI 10.1006/bbrc.2000.4039

Hou ZC, 2006, REGUL PEPTIDES, V134, P126, DOI 10.1016/j.regpep.2006.02.005

Hu XM, 2015, NAT REV NEUROL, V11, P56, DOI 10.1038/nrneurol.2014.207

Jiao LL, 2021, NEUROBIOL AGING, V101, P70, DOI 10.1016/j.neurobiolaging.2021.01.011

Kaiser J, 2021, DIABETES, V70, P423, DOI 10.2337/db20-0231

Kam TI, 2020, NEUROBIOL DIS, V144, DOI 10.1016/j.nbd.2020.105028

Kiriyama Y, 2015, INT J MOL SCI, V16, P26797, DOI 10.3390/ijms161125990

Kirsz K, 2011, PEPTIDES, V32, P2256, DOI 10.1016/j.peptides.2011.04.010

Kohno D, 2003, DIABETES, V52, P948, DOI 10.2337/diabetes.52.4.948

Kojima M, 1999, NATURE, V402, P656, DOI 10.1038/45230

Kojima M, 2005, PHYSIOL REV, V85, P495, DOI 10.1152/physrev.00012.2004

Korbonits M, 2004, EUR J ENDOCRINOL, V151, pS67, DOI 10.1530/eje.0.151S067

Ku JM, 2016, CLIN SCI, V130, P1545, DOI 10.1042/CS20160077

Kulkarni VV, 2018, DEV NEUROBIOL, V78, P298, DOI 10.1002/dneu.22562

Kunath N, 2015, SCI REP-UK, V5, DOI 10.1038/srep11452

Lee JY, 2015, NEUROBIOL DIS, V82, P141, DOI 10.1016/j.nbd.2015.06.002

Lee JY, 2014, ENDOCRINOL METAB, V29, P371, DOI 10.3803/EnM.2014.29.3.371

Lee J, 2010, J ENDOCRINOL, V205, P262, DOI 10.1677/JOE-10-0040

Leung E, 2011, NEUROBIOL AGING, V32, P387, DOI 10.1016/j.neurobiolaging.2009.02.025

Lewitt MS, 2017, BIOCHEM INSIGHTS, V10, DOI 10.1177/1178626417703995

Li P, 2014, INT J OBESITY, V38, P724, DOI 10.1038/ijo.2013.140

Li QY, 2018, NAT REV IMMUNOL, V18, P225, DOI 10.1038/nri.2017.125

Liu F, 2019, FRONT PHARMACOL, V10, DOI 10.3389/fphar.2019.01320

Liu WF, 2020, HUM MOL GENET, V29, P3224, DOI 10.1093/hmg/ddaa209

Lockie SH, 2013, MOL METAB, V2, P329, DOI 10.1016/j.molmet.2013.08.003

Lv Y, 2018, BIOSCIENCE REP, V38, DOI 10.1042/BSR20181061

Mani BK, 2019, ENDOCRINOLOGY, V160, P1247, DOI 10.1210/en.2019-00074

Martins I, 2013, NEUROSCIENCE, V241, P41, DOI 10.1016/j.neuroscience.2013.02.062

McGrattan AM, 2019, CURR NUTR REP, V8, P53, DOI 10.1007/s13668-019-0271-4

Mendes NF, 2022, J NEUROINFLAMM, V19, DOI 10.1186/s12974-022-02519-6

Mendes NF, 2018, FRONT NEUROSCI-SWITZ, V12, DOI 10.3389/fnins.2018.00846

Miller AA, 2014, BRAIN BEHAV IMMUN, V42, P10, DOI 10.1016/j.bbi.2014.04.001

Mohammed MS, 2018, J HEALTHC ENG, V2018, DOI 10.1155/2018/1564748

Moon M, 2011, J ALZHEIMERS DIS, V23, P147, DOI 10.3233/JAD-2010-101263

Moon M, 2009, NEUROTOX RES, V15, P332, DOI 10.1007/s12640-009-9037-x

Morgan AH, 2018, NEUROPHARMACOLOGY, V136, P317, DOI 10.1016/j.neuropharm.2017.12.027

Müller TD, 2015, MOL METAB, V4, P437, DOI 10.1016/j.molmet.2015.03.005

Murray PJ, 2014, IMMUNITY, V41, P14, DOI 10.1016/j.immuni.2014.06.008

Naznin F, 2015, J ENDOCRINOL, V226, P81, DOI 10.1530/JOE-15-0139

Neher JJ, 2019, TRENDS IMMUNOL, V40, P358, DOI 10.1016/j.it.2019.02.001

O'Brien PD, 2017, LANCET NEUROL, V16, P465, DOI 10.1016/S1474-4422(17)30084-4

Orihuela R, 2016, BRIT J PHARMACOL, V173, P649, DOI 10.1111/bph.13139

Pagano G, 2018, NEUROLOGY, V90, pE1654, DOI 10.1212/WNL.0000000000005475

Page LC, 2018, DIABETES, V67, P1976, DOI 10.2337/db18-0451

Pan XD, 2011, MOL NEURODEGENER, V6, DOI 10.1186/1750-1326-6-45

Panza F, 2011, CURR ALZHEIMER RES, V8, P492, DOI 10.2174/156720511796391818

Patel Kalpesh, 2009, F1000 Biol Rep, V1, P42, DOI 10.3410/B1-42

PERRY G, 1991, J NEUROSCI, V11, P3679

Pinkney J, 2014, CURR OPIN CLIN NUTR, V17, P497, DOI 10.1097/MCO.0000000000000101

Qin C, 2019, NEUROSCI BULL, V35, P921, DOI 10.1007/s12264-019-00388-3

Reich N, 2020, FRONT NEUROSCI-SWITZ, V14, DOI 10.3389/fnins.2020.614828

Rezaeian F, 2012, AM J PHYSIOL-HEART C, V302, pH603, DOI 10.1152/ajpheart.00390.2010

Russo C, 2020, NEUROPEPTIDES, V79, DOI 10.1016/j.npep.2019.101997

Russo C, 2018, EXP BRAIN RES, V236, P2223, DOI 10.1007/s00221-018-5298-z

Russo C, 2017, NEUROSCI LETT, V653, P126, DOI 10.1016/j.neulet.2017.05.049

Santos VV, 2017, J NEUROENDOCRINOL, V29, DOI 10.1111/jne.12476

Schellekens H, 2013, FRONT NEUROSCI-SWITZ, V7, DOI 10.3389/fnins.2013.00148

Scrivo A, 2018, LANCET NEUROL, V17, P802, DOI 10.1016/S1474-4422(18)30238-2

Selkoe DJ, 2001, PHYSIOL REV, V81, P741, DOI 10.1152/physrev.2001.81.2.741

Seminara RS, 2018, CUREUS J MED SCIENCE, V10, DOI 10.7759/cureus.3285

Sergi D, 2019, FRONT AGING NEUROSCI, V11, DOI 10.3389/fnagi.2019.00302

Sheikh MH, 2022, FASEB J, V36, DOI 10.1096/fj.202101297R

Sofroniew MV, 2010, ACTA NEUROPATHOL, V119, P7, DOI 10.1007/s00401-009-0619-8

Solfrizzi V, 2017, J ALZHEIMERS DIS, V59, P815, DOI [10.3233/JAD-170248, 10.3233/jad-170248]

Spillantini MG, 1997, NATURE, V388, P839, DOI 10.1038/42166

Steculorum SM, 2015, J CLIN INVEST, V125, P846, DOI 10.1172/JCI73688

Stefanis L, 2012, CSH PERSPECT MED, V2, DOI 10.1101/cshperspect.a009399

Stillman CM, 2017, FRONT AGING NEUROSCI, V9, DOI 10.3389/fnagi.2017.00115

Stoyanova II, 2014, NEUROBIOL DIS, V72, P72, DOI [10.1016/j.nbd2014.08.026, 10.1016/j.nbd.2014.08.026]

Subhramanyam CS, 2019, SEMIN CELL DEV BIOL, V94, P112, DOI 10.1016/j.semcdb.2019.05.004

Taub DD, 2008, VITAM HORM, V77, P325, DOI 10.1016/S0083-6729(06)77014-5

Thaler JP, 2012, J CLIN INVEST, V122, P153, DOI 10.1172/JCI59660

Thomas K, 2019, SCI REP-UK, V9, DOI 10.1038/s41598-019-53578-4

Tian R, 2022, EUR J HISTOCHEM, V66, DOI 10.4081/ejh.2022.3350

Timper K, 2017, DIS MODEL MECH, V10, P679, DOI 10.1242/dmm.026609

Tong J, 2010, DIABETES, V59, P2145, DOI 10.2337/db10-0504

Travagli RA, 2006, ANNU REV PHYSIOL, V68, P279, DOI 10.1146/annurev.physiol.68.040504.094635

Tschöp M, 2000, NATURE, V407, P908, DOI 10.1038/35038090

Uranga RM, 2019, FRONT NEUROSCI-SWITZ, V13, DOI 10.3389/fnins.2019.00513

Vasek MJ, 2016, NATURE, V534, P538, DOI 10.1038/nature18283

Wang HQ, 2021, NEUROPEPTIDES, V85, DOI 10.1016/j.npep.2020.102112

Wang HQ, 2020, BRAIN RES, V1746, DOI 10.1016/j.brainres.2020.147023

Wang Q, 2017, LIFE SCI, V186, P50, DOI 10.1016/j.lfs.2017.08.004

Xiong W, 2021, FRONT IMMUNOL, V12, DOI 10.3389/fimmu.2021.714340

Xu Y, 2021, NEURAL REGEN RES, V16, P270, DOI 10.4103/1673-5374.290881

Yanagi S, 2018, CELL METAB, V27, P786, DOI 10.1016/j.cmet.2018.02.008

Yang YX, 2019, NEUROCHEM RES, V44, P811, DOI 10.1007/s11064-018-02714-z

Zhu MQ, 2018, THER ADV NEUROL DISO, V11, DOI 10.1177/1756286418791107

Zigman JM, 2016, TRENDS ENDOCRIN MET, V27, P54, DOI 10.1016/j.tem.2015.09.010

NR 142

TC 16

Z9 16

U1 4

U2 11

PU MDPI

PI BASEL

PA ST ALBAN-ANLAGE 66, CH-4052 BASEL, SWITZERLAND

EI 1422-0067

J9 INT J MOL SCI

JI Int. J. Mol. Sci.

PD NOV

PY 2022

VL 23

IS 21

AR 13432

DI 10.3390/ijms232113432

PG 20

WC Biochemistry & Molecular Biology; Chemistry, Multidisciplinary

WE Science Citation Index Expanded (SCI-EXPANDED)

SC Biochemistry & Molecular Biology; Chemistry

GA 6C8WA

UT WOS:000882285800001

PM 36362220

OA Green Published

DA 2024-08-12

ER

PT J

AU Benameur, T

Soleti, R

Porro, C

AF Benameur, Tarek

Soleti, Raffaella

Porro, Chiara

TI The Potential Neuroprotective Role of Free and Encapsulated Quercetin

Mediated by miRNA against Neurological Diseases

SO NUTRIENTS

LA English

DT Review

DE quercetin; natural flavonoid; neuroinflammation; microglial cells;

miRNA; neuroprotection; neurodegenerative disease; antioxidant;

nanodrug

ID NF-KAPPA-B; OXIDATIVE DAMAGE; SIGNALING PATHWAY; GENE-EXPRESSION; TAU

HYPERPHOSPHORYLATION; ISCHEMIA-REPERFUSION; alzheimer's disease; MOUSE

MODELS; M2 MICROGLIA; NITRIC-OXIDE

AB Chronic neuroinflammation is a pathological condition of numerous central nervous system (CNS) diseases such as Parkinson's disease, Alzheimer's disease, multiple sclerosis, amyotrophic lateral sclerosis and many others. Neuroinflammation is characterized by the microglia activation and concomitant production of pro-inflammatory cytokines leading to an increasing neuronal cell death. The decreased neuroinflammation could be obtained by using natural compounds, including flavonoids known to modulate the inflammatory responses. Among flavonoids, quercetin possess multiple pharmacological applications including anti-inflammatory, antitumoral, antiapoptotic and anti-thrombotic activities, widely demonstrated in both in vitro and in vivo studies. In this review, we describe the recent findings about the neuroprotective action of quercetin by acting with different mechanisms on the microglial cells of CNS. The ability of quercetin to influence microRNA expression represents an interesting skill in the regulation of inflammation, differentiation, proliferation, apoptosis and immune responses. Moreover, in order to enhance quercetin bioavailability and capacity to target the brain, we discuss an innovative drug delivery system. In summary, this review highlighted an important application of quercetin in the modulation of neuroinflammation and prevention of neurological disorders.

C1 [Benameur, Tarek] King Faisal Univ, Coll Med, Dept Biomed Sci, Al Hasa 31982, Saudi Arabia.

[Soleti, Raffaella] Univ Nantes, Univ Angers, INSERM, CRCINA,SFR ICAT, F-49800 Angers, France.

[Porro, Chiara] Univ Foggia, Dept Clin & Expt Med, I-71121 Foggia, Italy.

C3 King Faisal University; Nantes Universite; Institut National de la Sante

et de la Recherche Medicale (Inserm); Universite d'Angers; University of

Foggia

RP Porro, C (corresponding author), Univ Foggia, Dept Clin & Expt Med, I-71121 Foggia, Italy.

EM tbenameur@kfu.edu.sa; raffaella.soleti@univ-angers.fr;

chiara.porro@unifg.it

RI BENAMEUR, T/KIH-8629-2024; PORRO, CHIARA/G-7849-2018

OI SOLETI, RAFFAELLA/0000-0002-0271-0490; PORRO, CHIARA/0000-0002-7526-6968

FU University of Foggia

FX This study was published with a contribution from 5 x 1000 IRPEF funds

in favour of the University of Foggia, in memory of Gianluca Montel.

CR Aïd S, 2011, BIOCHIMIE, V93, P46, DOI 10.1016/j.biochi.2010.09.009

Andres S, 2018, MOL NUTR FOOD RES, V62, DOI 10.1002/mnfr.201700447

Ay M, 2017, J NEUROCHEM, V141, P766, DOI 10.1111/jnc.14033

Banzhaf-Strathmann J, 2014, EMBO J, V33, P1667, DOI 10.15252/embj.201387576

Batiha GE, 2020, FOODS, V9, DOI 10.3390/foods9030374

Baufeld C, 2018, J NEURAL TRANSM, V125, P809, DOI 10.1007/s00702-017-1795-7

Bilkei-Gorzo A, 2014, PHARMACOL THERAPEUT, V142, P244, DOI 10.1016/j.pharmthera.2013.12.009

Boesch-Saadatmandi C, 2012, PHARMACOL RES, V65, P523, DOI 10.1016/j.phrs.2012.02.007

Bureau G, 2008, J NEUROSCI RES, V86, P403, DOI 10.1002/jnr.21503

Chang WS, 2017, MED SCI MONITOR, V23, P2721, DOI 10.12659/MSM.905064

Chen JC, 2005, EUR J PHARMACOL, V521, P9, DOI 10.1016/j.ejphar.2005.08.005

Chen TJ, 2006, TOXICOLOGY, V223, P113, DOI 10.1016/j.tox.2006.03.007

Chen WW, 2016, MOL MED REP, V13, P3391, DOI 10.3892/mmr.2016.4948

Cherry JD, 2014, J NEUROINFLAMM, V11, DOI 10.1186/1742-2094-11-98

Cianciulli A, 2020, BIOMOLECULES, V10, DOI 10.3390/biom10010137

Cianciulli A, 2017, CYTOKINE GROWTH F R, V37, P67, DOI 10.1016/j.cytogfr.2017.07.005

Clarke LE, 2013, NAT REV NEUROSCI, V14, P311, DOI 10.1038/nrn3484

Colombo E, 2016, TRENDS IMMUNOL, V37, P608, DOI 10.1016/j.it.2016.06.006

Martins-Perles JVC, 2020, AUTON NEUROSCI-BASIC, V227, DOI 10.1016/j.autneu.2020.102675

Costa LG, 2016, OXID MED CELL LONGEV, V2016, DOI 10.1155/2016/2986796

Costa LG, 2013, NEUROCHEM RES, V38, P1809, DOI 10.1007/s11064-013-1085-1

Cox LM, 2018, NEUROTHERAPEUTICS, V15, P135, DOI 10.1007/s13311-017-0598-8

D'Andrea G, 2015, FITOTERAPIA, V106, P256, DOI 10.1016/j.fitote.2015.09.018

Dabeek WM, 2019, NUTRIENTS, V11, DOI 10.3390/nu11102288

Dajas F, 2003, BRAZ J MED BIOL RES, V36, P1613, DOI 10.1590/S0100-879X2003001200002

Das Sanchari, 2008, Curr Aging Sci, V1, P169

Derosa G, 2021, PHYTOTHER RES, V35, P1230, DOI 10.1002/ptr.6887

Dong FX, 2017, BIOCHEM BIOPH RES CO, V491, P636, DOI 10.1016/j.bbrc.2017.07.151

Ebrahimpour S, 2020, SCI REP-UK, V10, DOI 10.1038/s41598-020-71678-4

El Fatimy R, 2018, ACTA NEUROPATHOL, V136, P537, DOI 10.1007/s00401-018-1880-5

Fan H, 2019, J NEUROINFLAMM, V16, DOI 10.1186/s12974-019-1613-2

Almeida AF, 2018, COMPR REV FOOD SCI F, V17, P714, DOI 10.1111/1541-4337.12342

Gan L, 2014, BBA-MOL BASIS DIS, V1842, P1208, DOI 10.1016/j.bbadis.2013.12.011

García-Barrado MJ, 2020, PHARMACEUTICALS-BASE, V13, DOI 10.3390/ph13110342

Gay NJ, 2014, NAT REV IMMUNOL, V14, P546, DOI 10.1038/nri3713

Ghahremani S, 2018, J FOOD BIOCHEM, V42, DOI 10.1111/jfbc.12530

Ghosh A, 2013, PLOS ONE, V8, DOI [10.1371/journal.pone.0057735, 10.1371/journal.pone.0082529]

Ghosh A, 2009, LIFE SCI, V84, P75, DOI 10.1016/j.lfs.2008.11.001

Ghosh S, 2011, FREE RADICAL BIO MED, V51, P1893, DOI 10.1016/j.freeradbiomed.2011.08.019

González H, 2014, J NEUROIMMUNOL, V274, P1, DOI 10.1016/j.jneuroim.2014.07.012

Gregor MF, 2011, ANNU REV IMMUNOL, V29, P415, DOI 10.1146/annurev-immunol-031210-101322

Harwood M, 2007, FOOD CHEM TOXICOL, V45, P2179, DOI 10.1016/j.fct.2007.05.015

Hébert SS, 2010, HUM MOL GENET, V19, P3959, DOI 10.1093/hmg/ddq311

Hickman S, 2018, NAT NEUROSCI, V21, P1359, DOI 10.1038/s41593-018-0242-x

Hotamisligil GS, 2017, NATURE, V542, P177, DOI 10.1038/nature21363

Hu T, 2020, AGING-US, V12, P7015, DOI 10.18632/aging.103059

Jagannathan M, 2009, J IMMUNOL, V183, P7461, DOI 10.4049/jimmunol.0901517

Jeong E, 2011, YONSEI MED J, V52, P379, DOI [10.3349/ymj.2011.52.3.379, 10.3349/ymj.2011.52.3.429]

Jin Z, 2019, AM J TRANSL RES, V11, P4683

Kawabata K, 2015, FOOD FUNCT, V6, P1399, DOI [10.1039/c4fo01178c, 10.1039/C4FO01178C]

Kawai T, 2007, TRENDS MOL MED, V13, P460, DOI 10.1016/j.molmed.2007.09.002

Khan H, 2020, BIOMOLECULES, V10, DOI 10.3390/biom10010059

Konovalova J, 2019, INT J MOL SCI, V20, DOI 10.3390/ijms20236055

Kuipers EN, 2018, INT J MOL SCI, V19, DOI 10.3390/ijms19061786

Kwon HS, 2020, TRANSL NEURODEGENER, V9, DOI 10.1186/s40035-020-00221-2

Kyaw M, 2004, ACTA PHARMACOL SIN, V25, P977

Lakhanpal P, 2007, INTERNET J MED UPDAT, V2, P22

LAWSON LJ, 1990, NEUROSCIENCE, V39, P151, DOI 10.1016/0306-4522(90)90229-W

Le K, 2020, INFLAMM RES, V69, P1201, DOI 10.1007/s00011-020-01402-5

Lesjak M, 2018, J FUNCT FOODS, V40, P68, DOI 10.1016/j.jff.2017.10.047

Li Y, 2016, NUTRIENTS, V8, DOI 10.3390/nu8030167

Liu XY, 2016, AM J TRANSL RES, V8, P5169

Lv ML, 2018, MOL NUTR FOOD RES, V62, DOI 10.1002/mnfr.201800621

Ma Q, 2013, ANNU REV PHARMACOL, V53, P401, DOI 10.1146/annurev-pharmtox-011112-140320

MacVicar BA, 2015, CSH PERSPECT BIOL, V7, DOI 10.1101/cshperspect.a020388

Manach C, 1996, NUTR RES, V16, P517, DOI 10.1016/0271-5317(96)00032-2

Martel J, 2020, TRENDS BIOCHEM SCI, V45, P462, DOI 10.1016/j.tibs.2020.02.008

Nabavi SF, 2015, FOOD CHEM, V179, P305, DOI 10.1016/j.foodchem.2015.02.006

Nair MPN, 2009, AM J INFECT DIS, V5, P135, DOI 10.3844/ajidsp.2009.135.141

Nguyen T, 2009, J BIOL CHEM, V284, P13291, DOI 10.1074/jbc.R900010200

Nunomura A, 2020, OXID MED CELL LONGEV, V2020, DOI 10.1155/2020/2638130

Oksanen M, 2019, CELL MOL LIFE SCI, V76, P2739, DOI 10.1007/s00018-019-03111-7

Panaro MA, 2008, IMMUNOPHARM IMMUNOT, V30, P729, DOI [10.1080/08923970802278557, 10.1080/08923970802278557 ]

Panaro MA, 2020, BIOMOLECULES, V10, DOI 10.3390/biom10060901

Panaro MA, 2020, INT J MOL SCI, V21, DOI 10.3390/ijms21072299

Porro C, 2019, BIOLOGY-BASEL, V8, DOI 10.3390/biology8030051

Pricci M, 2009, TISSUE ENG PT A, V15, P137, DOI 10.1089/ten.tea.2007.0360

Qi Y, 2020, DRUG DELIV, V27, P745, DOI 10.1080/10717544.2020.1762262

Querfurth HW, 2010, NEW ENGL J MED, V362, P329, DOI 10.1056/NEJMra0909142

Ransohoff RM, 2016, NAT NEUROSCI, V19, P987, DOI 10.1038/nn.4338

Rogero MM, 2018, NUTRIENTS, V10, DOI 10.3390/nu10040432

Rothwell JA, 2013, DATABASE-OXFORD, DOI 10.1093/database/bat070

Santa-Maria I, 2015, J CLIN INVEST, V125, P681, DOI 10.1172/JCI78421

Schadich E, 2016, BIOMED RES INT, V2016, DOI 10.1155/2016/2173275

Schnare M, 2001, NAT IMMUNOL, V2, P947, DOI 10.1038/ni712

Selvakumar Kandaswamy, 2018, Interdiscip Toxicol, V11, P294, DOI 10.2478/intox-2018-0029

Serban MC, 2016, J AM HEART ASSOC, V5, DOI 10.1161/JAHA.115.002713

Shankar G Mohan, 2015, Enzymes, V37, P43, DOI 10.1016/bs.enz.2015.05.001

Sharma V, 2007, BRAIN RES BULL, V73, P55, DOI 10.1016/j.brainresbull.2007.01.016

Smith PY, 2015, HUM MOL GENET, V24, P6721, DOI 10.1093/hmg/ddv377

Sofroniew MV, 2009, TRENDS NEUROSCI, V32, P638, DOI 10.1016/j.tins.2009.08.002

Soleti R, 2018, ARCH BIOCHEM BIOPHYS, V644, P57, DOI 10.1016/j.abb.2018.03.004

Soleti R, 2012, FREE RADICAL BIO MED, V53, P2159, DOI 10.1016/j.freeradbiomed.2012.09.021

Subhramanyam CS, 2019, SEMIN CELL DEV BIOL, V94, P112, DOI 10.1016/j.semcdb.2019.05.004

Suganthy N, 2016, BIOMED PHARMACOTHER, V84, P892, DOI 10.1016/j.biopha.2016.10.011

Sun GY, 2015, PLOS ONE, V10, DOI 10.1371/journal.pone.0141509

Sun Y, 2017, CNS NEUROL DISORD-DR, V16, P326, DOI 10.2174/1871527316666170102120211

Sundaram MK, 2019, BIOSCIENCE REP, V39, DOI [10.1042/BSR20190720, 10.1042/bsr20190720]

Tang Y, 2016, MOL NEUROBIOL, V53, P1181, DOI 10.1007/s12035-014-9070-5

Trotta T, 2019, NUTR CANCER, V71, P1241, DOI 10.1080/01635581.2019.1604978

Trotta T, 2018, BIOCHEM PHARMACOL, V148, P184, DOI 10.1016/j.bcp.2017.12.020

van Meeteren ME, 2004, BIOCHEM PHARMACOL, V67, P967, DOI 10.1016/j.bcp.2003.10.018

Vomhof-DeKrey EE, 2012, J NUTR BIOCHEM, V23, P1201, DOI 10.1016/j.jnutbio.2012.03.005

Wang G, 2012, INT J NANOMED, V7, P271, DOI 10.2147/IJN.S26935

Wang M, 2019, BIOTECHNOL BIOTEC EQ, V33, P1000, DOI 10.1080/13102818.2019.1635528

Wang WY, 2016, TRENDS FOOD SCI TECH, V56, P21, DOI 10.1016/j.tifs.2016.07.004

Wang X, 2015, FEBS LETT, V589, P726, DOI 10.1016/j.febslet.2015.02.001

Wu MY, 2019, INT IMMUNOPHARMACOL, V74, DOI 10.1016/j.intimp.2019.105704

Xie J, 2020, BIOMED PHARMACOTHER, V127, DOI 10.1016/j.biopha.2020.110147

Yang J, 2017, NUTRIENTS, V9, DOI 10.3390/nu9070650

Yang SY, 2020, MOL NUTR FOOD RES, V64, DOI 10.1002/mnfr.201901019

Yardim A, 2020, NEUROTOXICOLOGY, V81, P137, DOI 10.1016/j.neuro.2020.10.001

Zaplatic E, 2019, LIFE SCI, V224, P109, DOI 10.1016/j.lfs.2019.03.055

Zhai SW, 2014, ISR J AQUACULT-BAMID, V66

Zhai SW, 2013, ITAL J ANIM SCI, V12, DOI 10.4081/ijas.2013.e85

Zhang FW, 2020, EXP THER MED, V20, DOI 10.3892/etm.2020.9410

Zhang M, 2011, ADV EXP MED BIOL, V701, P283, DOI 10.1007/978-1-4419-7756-4_38

Zhang X, 2016, CELL MOL NEUROBIOL, V36, P459, DOI 10.1007/s10571-016-0350-7

Zhang ZJ, 2011, INT J MOL MED, V27, P195, DOI 10.3892/ijmm.2010.571

Zhang ZY, 2020, OXID MED CELL LONGEV, V2020, DOI 10.1155/2020/6038919

Zhou L, 2013, VIROL J, V10, DOI 10.1186/1743-422X-10-172

NR 121

TC 42

Z9 42

U1 0

U2 18

PU MDPI

PI BASEL

PA ST ALBAN-ANLAGE 66, CH-4052 BASEL, SWITZERLAND

EI 2072-6643

J9 NUTRIENTS

JI Nutrients

PD APR

PY 2021

VL 13

IS 4

AR 1318

DI 10.3390/nu13041318

PG 16

WC Nutrition & Dietetics

WE Science Citation Index Expanded (SCI-EXPANDED)

SC Nutrition & Dietetics

GA RR9DD

UT WOS:000643388800001

PM 33923599

OA Green Published, gold

DA 2024-08-12

ER

PT J

AU Liu, Y

Liu, T

Zhou, YZ

Li, WJ

Wang, M

Song, NS

Zhang, WB

Jiang, JW

Yuan, ST

Ding, JH

Hu, G

Lu, M

AF Liu, Yang

Liu, Ting

Zhou, Yuanzhang

Li, Wenjie

Wang, Min

Song, Nanshan

Zhang, Wenbin

Jiang, Jingwei

Yuan, Shengtao

Ding, Jianhua

Hu, Gang

Lu, Ming

TI Impeding the combination of astrocytic ASCT2 and NLRP3 by talniflumate

alleviates neuroinflammation in experimental models of Parkinson's

disease

SO ACTA PHARMACEUTICA SINICA B

LA English

DT Article

DE ASCT2; NLRP3; Astrocytes; Inflammation; Neurodegeneration; Drug

screening; Talniflumate; parkinson's disease

ID AMINO-ACID TRANSPORTER; GLUTAMINE TRANSPORTERS; INFLAMMASOME;

INHIBITION; CLONING

AB Alanine-serine-cysteine transporter 2 (ASCT2) is reported to participate in the progression of tumors and metabolic diseases. It is also considered to play a crucial role in the glutamate-glutamine shuttle of neuroglial network. However, it remains unclear the involvement of ASCT2 in neurological diseases such as Parkinson's disease (PD). In this study, we demonstrated that high expression of ASCT2 in the plasma samples of PD patients and the midbrain of MPTP mouse models is positively correlated with dyskinesia. We further illustrated that ASCT2 expressed in astrocytes rather than neurons signifi-cantly upregulated in response to either MPP thorn or LPS/ATP challenge. Genetic ablation of astrocytic ASCT2 alleviated the neuroinflammation and rescued dopaminergic (DA) neuron damage in PD models in vitro and in vivo. Notably, the binding of ASCT2 to NLRP3 aggravates astrocytic inflammasome-triggered neuroinflammation. Then a panel of 2513 FDA-approved drugs were performed via virtual mo-lecular screening based on the target ASCT2 and we succeed in getting the drug talniflumate. It is vali-dated talniflumate impedes astrocytic inflammation and prevents degeneration of DA neurons in PD models. Collectively, these findings reveal the role of astrocytic ASCT2 in the pathogenesis of PD, broaden the therapeutic strategy and provide a promising candidate drug for PD treatment. 2023 Chinese Pharmaceutical Association and Institute of Materia Medica, Chinese Academy of Medical Sciences. Production and hosting by Elsevier B.V.

C1 [Liu, Ting; Zhou, Yuanzhang; Li, Wenjie; Song, Nanshan; Ding, Jianhua; Hu, Gang; Lu, Ming] Nanjing Med Univ, Dept Pharmacol, Jiangsu Key Lab Neurodegenerat, Nanjing 211166, China.

[Liu, Yang; Hu, Gang] Nanjing Univ Chinese Med, Sch Med & Holist Integrat Med, Dept Pharmacol, Nanjing 210023, China.

[Wang, Min] Nanjing Med Univ, Dept Geriatr, Affiliated Brain Hosp, Nanjing 210029, China.

[Zhang, Wenbin] Nanjing Med Univ, Dept Neurosurg, Affiliated Brain Hosp, Nanjing 210029, China.

[Jiang, Jingwei; Yuan, Shengtao] China Pharmaceut Univ, Jiangsu Key Lab Drug Screening, Nanjing 211198, China.

C3 Nanjing Medical University; Nanjing University of Chinese Medicine;

Nanjing Medical University; Nanjing Medical University; China

Pharmaceutical University

RP Hu, G; Lu, M (corresponding author), Nanjing Med Univ, Dept Pharmacol, Jiangsu Key Lab Neurodegenerat, Nanjing 211166, China.; Hu, G (corresponding author), Nanjing Univ Chinese Med, Sch Med & Holist Integrat Med, Dept Pharmacol, Nanjing 210023, China.

EM ghu@njmu.edu.cn; lum@njmu.edu.cn

RI li, bai/JNE-1502-2023; Li, Shiyu/KHE-1376-2024; xuan, li/JNI-7432-2023;

peng, jin/JRW-4493-2023; Li, Wenjie/AAC-2500-2021; lin,

lin/KCZ-0185-2024; Zhu, Li/JTT-9093-2023; Wang, Jiacheng/ABE-5948-2020;

Liu, Zhiyu/JNR-8043-2023; Wang, Han/JJF-2614-2023; shi,

chen/KEH-8339-2024; Li, Tingting/HKE-0812-2023

OI Li, Wenjie/0000-0002-1244-7657; Wang, Jiacheng/0000-0003-4327-1508; Liu,

Zhiyu/0000-0001-8351-1268; Liu, Yang/0000-0003-4243-2330

FU Experiment Center for Science and Technology, Nanjing University of

Chinese Medicine (Nanjing, China); National Key R&D Program of China

[2021ZD0202903]; National Natural Science Foundation of China [81922066,

82173797, 82003725, 81991523, 82003722]

FX We would like to thank Prof. Honghong Yao from the Department of

Pharmacology, Southeast University, for the gift of Nlrp3<SUP>-/-</SUP>

mice. We thank Dr. Lei Cao from the Department of Pharmacology, National

University of Singapore, for his helpful suggestions on the manuscript.

We also thank Yi Liu for the help in the experiment of UPLC-MS. The

authors are grateful for the support of the Experiment Center for

Science and Technology, Nanjing University of Chinese Medicine (Nanjing,

China) . The work was supported by grants from the National Key R & D

Program of China (No. 2021ZD0202903) and the National Natural Science

Foundation of China (Nos. 81922066, 82173797, 82003725, 81991523, and

82003722) .

CR Araque A, 2014, NEURON, V81, P728, DOI 10.1016/j.neuron.2014.02.007

Bröer A, 1999, J NEUROCHEM, V73, P2184

Bröer A, 2018, FRONT PHARMACOL, V9, DOI 10.3389/fphar.2018.00785

Clarke LE, 2013, NAT REV NEUROSCI, V14, P311, DOI 10.1038/nrn3484

Escartin C, 2021, NAT NEUROSCI, V24, P312, DOI 10.1038/s41593-020-00783-4

Freeman L, 2017, J EXP MED, V214, P1351, DOI 10.1084/jem.20150237

Freeman MR, 2010, SCIENCE, V330, P774, DOI 10.1126/science.1190928

Guo CS, 2016, IMMUNITY, V45, P802, DOI 10.1016/j.immuni.2016.09.008

Han XJ, 2019, AUTOPHAGY, V15, P1860, DOI 10.1080/15548627.2019.1596481

Heckel T, 2003, NEUROCHEM INT, V43, P289, DOI 10.1016/S0197-0186(03)00014-7

Heneka MT, 2018, NAT REV NEUROSCI, V19, P610, DOI 10.1038/s41583-018-0055-7

Kam TI, 2020, NEUROBIOL DIS, V144, DOI 10.1016/j.nbd.2020.105028

Kaushik DK, 2012, PLOS ONE, V7, DOI 10.1371/journal.pone.0032270

Kekuda R, 1996, J BIOL CHEM, V271, P18657, DOI 10.1074/jbc.271.31.18657

Knight Darryl, 2004, Curr Opin Investig Drugs, V5, P557

Lee NY, 2017, BIOCHEM BIOPH RES CO, V483, P135, DOI 10.1016/j.bbrc.2016.12.178

Leke R, 2016, ADV NEUROBIOL, V13, P223, DOI 10.1007/978-3-319-45096-4_8

Liu Y, 2018, EUR J PHARMACOL, V837, P81, DOI 10.1016/j.ejphar.2018.07.007

Mangan MSJ, 2018, NAT REV DRUG DISCOV, V17, P588, DOI [10.1038/nrd.2018.97, 10.1038/nrd.2018.149]

Masle-Farquhar E, 2017, FRONT IMMUNOL, V8, DOI 10.3389/fimmu.2017.00549

Maturana CJ, 2017, DEV NEUROBIOL, V77, P625, DOI 10.1002/dneu.22409

Maucler C, 2013, ACS CHEM NEUROSCI, V4, P772, DOI 10.1021/cn4000549

McGivan JD, 2007, FRONT BIOSCI, V12, P874, DOI 10.2741/2109

Ni F, 2019, NAT METAB, V1, P390, DOI 10.1038/s42255-019-0039-6

Przedborski S, 2017, NAT REV NEUROSCI, V18, P251, DOI 10.1038/nrn.2017.25

Scalise M, 2018, FRONT CELL DEV BIOL, V6, DOI 10.3389/fcell.2018.00096

Schulte ML, 2018, NAT MED, V24, P194, DOI 10.1038/nm.4464

Schwartz M, 2014, EMBO J, V33, P7, DOI 10.1002/embj.201386609

Shimizu K, 2014, BRIT J CANCER, V110, P2030, DOI 10.1038/bjc.2014.88

Song WX, 2020, ACTA PHARMACOL SIN B, V10, P61, DOI 10.1016/j.apsb.2019.12.006

Thundyil J, 2015, AGEING RES REV, V24, P17, DOI 10.1016/j.arr.2014.11.003

UtsunomiyaTate N, 1996, J BIOL CHEM, V271, P14883, DOI 10.1074/jbc.271.25.14883

Walker NM, 2006, J PHARMACOL EXP THER, V317, P275, DOI 10.1124/jpet.105.094847

Walsh JG, 2014, NAT REV NEUROSCI, V15, P84, DOI 10.1038/nrn3638

Wang JS, 2017, FRONT MOL NEUROSCI, V10, DOI 10.3389/fnmol.2017.00139

Wang L, 2019, PHYTOMEDICINE, V57, P117, DOI 10.1016/j.phymed.2018.12.011

Wei Y, 2020, NAT COMMUN, V11, DOI 10.1038/s41467-020-14788-x

Ye JX, 2018, J CANCER RES CLIN, V144, P821, DOI 10.1007/s00432-018-2605-9

Zhu JL, 2018, CELL DEATH DIFFER, V25, P2037, DOI 10.1038/s41418-018-0127-2

NR 39

TC 11

Z9 11

U1 4

U2 24

PU INST MATERIA MEDICA, CHINESE ACAD MEDICAL SCIENCES

PI BEIJING

PA C/O EDITORIAL BOARD OF ACTA PHARMACEUTICA SINICA, 1 XIANNONGTAN ST,

BEIJING, 100050, China

SN 2211-3835

EI 2211-3843

J9 ACTA PHARM SIN B

JI Acta Pharm. Sin. B

PD FEB

PY 2023

VL 13

IS 2

BP 662

EP 677

DI 10.1016/j.apsb.2022.07.021

EA FEB 2023

PG 16

WC Pharmacology & Pharmacy

WE Science Citation Index Expanded (SCI-EXPANDED)

SC Pharmacology & Pharmacy

GA 9Q0PG

UT WOS:000944675700001

PM 36873178

OA gold, Green Published

DA 2024-08-12

ER

PT J

AU Pottoo, FH

Ibrahim, AM

Alammar, A

Alsinan, R

Aleid, M

Alshehhi, A

Alshehri, M

Mishra, S

Alhajri, N

AF Pottoo, Faheem Hyder

Ibrahim, Abdallah Mohammad

Alammar, Ali

Alsinan, Rida

Aleid, Mahdi

Alshehhi, Ali

Alshehri, Muruj

Mishra, Supriya

Alhajri, Noora

TI Thymoquinone: Review of Its Potential in the Treatment of Neurological

Diseases

SO PHARMACEUTICALS

LA English

DT Review

DE thymoquinone; anti-inflammation; Alzheimer's disease; neuroinflammation;

Parkinson's disease; epilepsy; mitochondrial dysfunction

ID NIGELLA-SATIVA SEEDS; INDUCED alzheimer's disease; OXIDATIVE STRESS;

TOXICOLOGICAL PROPERTIES; IN-VITRO; NEURONS; OIL; INFLAMMATION; PATHWAY;

TOXICITY

AB Thymoquinone (TQ) possesses anticonvulsant, antianxiety, antidepressant, and antipsychotic properties. It could be utilized to treat drug misuse or dependence, and those with memory and cognitive impairment. TQ protects brain cells from oxidative stress, which is especially pronounced in memory-related regions. TQ exhibits antineurotoxin characteristics, implying its role in preventing neurodegenerative disorders such as Alzheimer's disease and Parkinson's disease. TQ's antioxidant and anti-inflammatory properties protect brain cells from damage and inflammation. Glutamate can trigger cell death by causing mitochondrial malfunction and the formation of reactive oxygen species (ROS). Reduction in ROS production can explain TQ effects in neuroinflammation. TQ can help prevent glutamate-induced apoptosis by suppressing mitochondrial malfunction. Several studies have demonstrated TQ's role in inhibiting Toll-like receptors (TLRs) and some inflammatory mediators, leading to reduced inflammation and neurotoxicity. Several studies did not show any signs of dopaminergic neuron loss after TQ treatment in various animals. TQ has been shown in clinical studies to block acetylcholinesterase (AChE) activity, which increases acetylcholine (ACh). As a result, fresh memories are programmed to preserve the effects. Treatment with TQ has been linked to better outcomes and decreased side effects than other drugs.

C1 [Pottoo, Faheem Hyder; Alammar, Ali; Alsinan, Rida; Aleid, Mahdi; Alshehri, Muruj] Imam Abdul Rahman Bin Faisal Univ, Coll Clin Pharm, Dept Pharmacol, Dammam 31441, Saudi Arabia.

[Ibrahim, Abdallah Mohammad] Imam Abdul Rahman Bin Faisal Univ, Dept Fundamentals Nursing, Coll Nursing, Dammam 31441, Saudi Arabia.

[Alshehhi, Ali] Khalifa Univ, Coll Med & Hlth Sci, POB 127788, Abu Dhabi, U Arab Emirates.

[Mishra, Supriya] SRM Inst Sci & Technol, SRM Modinagar Coll Pharm, Delhi NCR Campus, Ghaziabad 201204, UP, India.

[Alhajri, Noora] Sheikh Shakhbout Med City SSMC, Dept Med, POB 127788, Abu Dhabi, U Arab Emirates.

C3 Imam Abdulrahman Bin Faisal University; Imam Abdulrahman Bin Faisal

University; Khalifa University of Science & Technology; SRM Institute of

Science & Technology Delhi NCR (Ghaziabad)

RP Pottoo, FH (corresponding author), Imam Abdul Rahman Bin Faisal Univ, Coll Clin Pharm, Dept Pharmacol, Dammam 31441, Saudi Arabia.; Ibrahim, AM (corresponding author), Imam Abdul Rahman Bin Faisal Univ, Dept Fundamentals Nursing, Coll Nursing, Dammam 31441, Saudi Arabia.

EM fhpottoo@iau.edu.sa; amsudqi@iau.edu.sa; 2170005364@iau.edu.sa;

2170006017@iau.edu.sa; 2170000709@iau.edu.sa; 100042658@ku.ac.ae;

mneurodegenerative diseaselshehri@iau.edu.sa; supriya.initative@gmail.com;

nalhajri007@gmail.com

OI Pottoo, Dr. Faheem/0000-0003-2577-7505; ALAMMAR,

Ali/0000-0003-1145-6938; Alshehhi, Ali/0000-0002-2079-7155; Alhajri,

Noora/0000-0001-7205-7493

CR Abulfadl YS, 2018, HUM EXP TOXICOL, V37, P1092, DOI 10.1177/0960327118755256

Ahmad A, 2015, NAT PROD COMMUN, V10, P1395

Akgül B, 2021, DARU, V29, P85, DOI 10.1007/s40199-020-00385-2

Akhondian J, 2011, EPILEPSY RES, V93, P39, DOI 10.1016/j.eplepsyres.2010.10.010

Al Mamun A, 2022, NEUROCHEM RES, V47, P933, DOI 10.1007/s11064-021-03495-8

Al-Majed AA, 2006, EUR J PHARMACOL, V543, P40, DOI 10.1016/j.ejphar.2006.05.046

Alhebshi AH, 2013, BIOCHEM BIOPH RES CO, V433, P362, DOI 10.1016/j.bbrc.2012.11.139

Alhmied F, 2021, COMB CHEM HIGH T SCR, V24, P1644, DOI 10.2174/1386207323999201027225305

Ali BH, 2003, PHYTOTHER RES, V17, P299, DOI 10.1002/ptr.1309

Ali MY, 2021, BIOMED PHARMACOTHER, V134, DOI 10.1016/j.biopha.2020.111157

Alkharfy KM, 2015, EUR J DRUG METAB PH, V40, P319, DOI 10.1007/s13318-014-0207-8

Alkharfy KM, 2013, J LIQ CHROMATOGR R T, V36, P2242, DOI 10.1080/10826076.2012.717062

Alrafiah A, 2021, BIOMOLECULES, V11, DOI 10.3390/biom11020165

Alshyarba M, 2021, BIOTECHNOL APPL BIOC, V68, P1403, DOI 10.1002/bab.2062

[Anonymous], 2014, WORLD J NEUROSCI

Ardah MT, 2019, NEUROCHEM INT, V128, P115, DOI 10.1016/j.neuint.2019.04.014

Ayaz M, 2017, FRONT AGING NEUROSCI, V9, DOI 10.3389/fnagi.2017.00168

Badary OA, 1998, DRUG DEVELOP RES, V44, P56, DOI 10.1002/(SICI)1098-2299(199806/07)44:2/3<56::AID-DDR2>3.0.CO;2-9

Badary OA, 2003, DRUG CHEM TOXICOL, V26, P87, DOI 10.1081/DCT-120020404

Bargi R, 2021, JUNDISHAPUR J NAT PH, V16, DOI 10.5812/jjnpp.67765

Bargi R, 2017, CYTOKINE, V96, P173, DOI 10.1016/j.cyto.2017.04.015

Beyazcicek E, 2016, NEUROSCIENCES, V21, P131, DOI 10.17712/nsj.2016.2.20150781

Butt MS, 2021, FOOD SCI NUTR, V9, P1792, DOI 10.1002/fsn3.2070

Cascella M, 2018, FRONT AGING NEUROSCI, V10, DOI 10.3389/fnagi.2018.00016

Chen X, 2021, CELLS-BASEL, V10, DOI 10.3390/cells10061309

Cobourne-Duval MK, 2018, J NEUROIMMUNOL, V320, P87, DOI 10.1016/j.jneuroim.2018.04.018

Darakhshan S, 2015, PHARMACOL RES, V95-96, P138, DOI 10.1016/j.phrs.2015.03.011

Das S, 2022, J HERB MED, V32, DOI 10.1016/j.hermed.2021.100507

Ebrahimi SS, 2017, TOXICOL LETT, V276, P108, DOI 10.1016/j.toxlet.2017.05.018

El-Marasy S. A., 2012, Bulletin of the Faculty of Pharmacy, Cairo University, V50, P81, DOI 10.1016/j.bfopcu.2012.05.001

Elibol B, 2020, PHYTOMEDICINE, V79, DOI 10.1016/j.phymed.2020.153324

Elmaci I, 2016, BIOMED PHARMACOTHER, V83, P635, DOI 10.1016/j.biopha.2016.07.018

Fard AA, 2021, IRAN J BASIC MED SCI, V24, P30, DOI 10.22038/ijbms.2020.47479.10930

Farkhondeh T, 2018, DOSE-RESPONSE, V16, DOI 10.1177/1559325818761455

Farkhondeh T, 2017, BIOMED PHARMACOTHER, V96, P1440, DOI 10.1016/j.biopha.2017.12.013

Fattahl LIA, 2016, INT J STEM CELLS, V9, P230, DOI 10.15283/ijsc16021

Fouad IA, 2018, FRONT NEUROL, V9, DOI 10.3389/fneur.2018.00236

Ganguly U, 2021, OXID MED CELL LONGEV, V2021, DOI 10.1155/2021/7086512

Guler EM, 2021, TOXICOL REP, V8, P162, DOI 10.1016/j.toxrep.2020.12.026

Hajipour S, 2021, IRAN J BASIC MED SCI, V24, P881, DOI 10.22038/ijbms.2021.52824.11913

Hosseinzadeh H, 2007, PHYTOMEDICINE, V14, P621, DOI 10.1016/j.phymed.2006.12.005

Iranshahy M, 2022, PHYTOTHER RES, V36, P1952, DOI 10.1002/ptr.7425

Ismail N, 2008, J FOOD LIPIDS, V15, P519, DOI 10.1111/j.1745-4522.2008.00137.x

Jakaria M, 2018, OXID MED CELL LONGEV, V2018, DOI 10.1155/2018/1209801

Junaid M, 2021, MINI-REV MED CHEM, V21, P288, DOI 10.2174/1389557520666201005143818

Khader M, 2009, FOOD CHEM TOXICOL, V47, P129, DOI 10.1016/j.fct.2008.10.019

Khalilullah H., 2018, MOL THERAPEUTIC ACTI, P75, DOI [10.1007/978-981-10-8800-1_7, DOI 10.1007/978-981-10-8800-1_7]

Khan A, 2012, MOL CELL BIOCHEM, V369, P55, DOI 10.1007/s11010-012-1368-x

Kohandel Z, 2021, BIOMED PHARMACOTHER, V138, DOI 10.1016/j.biopha.2021.111492

Landucci E, 2021, MOLECULES, V26, DOI 10.3390/molecules26061592

Liang JF, 2021, MOL IMMUNOL, V135, P21, DOI 10.1016/j.molimm.2021.03.015

Malik S, 2021, DRUG DISCOV TODAY, V26, P2716, DOI 10.1016/j.drudis.2021.07.013

Malik T, 2016, NEUROCHEM RES, V41, P3386, DOI 10.1007/s11064-016-2073-z

Meral I, 2016, BIOTECH HISTOCHEM, V91, P493, DOI 10.1080/10520295.2016.1245866

Nampoothiri VPN, 2021, BIOCELL, V45, P1251, DOI 10.32604/biocell.2021.015090

Oskouei Z, 2021, PHYTOTHER RES, V35, P2252, DOI 10.1002/ptr.6982

Ozbolat G, 2021, BRATISL MED J, V122, P748, DOI 10.4149/BLL_2021_120

Pal RR, 2021, PHARMACEUTICS, V13, DOI 10.3390/pharmaceutics13060775

Poorgholam P, 2018, AVICENNA J PHYTOMEDI, V8, P188

Pottoo FH, 2021, PHARMACEUTICALS-BASE, V14, DOI 10.3390/ph14111132

Radad K, 2009, PHYTOTHER RES, V23, P696, DOI 10.1002/ptr.2708

Radad KS, 2015, NEUROSCIENCES, V20, P10

Roohbakhsh A, 2021, RES PHARM SCI, V16, P391, DOI 10.4103/1735-5362.319577

Sahak MKA, 2016, EVID-BASED COMPL ALT, V2016, DOI 10.1155/2016/6075679

Sahak MKA, 2013, EVID-BASED COMPL ALT, V2013, DOI 10.1155/2013/180598

Samad N, 2021, METAB BRAIN DIS, V36, P2535, DOI 10.1007/s11011-021-00789-2

Sandhua K.S., 2013, Int. J. Pharm. Pharm. Sci, V5, P884

Sarkar C, 2021, FOOD FUNCT, V12, P6167, DOI [10.1039/d1fo00401h, 10.1039/D1FO00401H]

Sedaghat R, 2014, IRAN J PHARM RES, V13, P227

Seghatoleslam Masoumeh, 2016, J Tradit Complement Med, V6, P262, DOI 10.1016/j.jtcme.2015.06.003

Song JX, 2012, J ETHNOPHARMACOL, V139, P698, DOI 10.1016/j.jep.2011.12.030

Talebi M, 2021, PHYTOTHER RES, V35, P1739, DOI 10.1002/ptr.6905

Turkeri ON, 2021, NEW TREND MED SCI, V2, P136

Uddin MN, 2021, CURR MOL PHARMACOL, V14, P1083, DOI 10.2174/1874467214666210105140944

Ustunova S, 2022, TOXICOL ENVIRON CHEM, V104, P176, DOI 10.1080/02772248.2022.2051509

Xiao XY, 2016, BIOMED RES INT, V2016, DOI 10.1155/2016/2571060

Yu TW, 2021, INT J MOL SCI, V22, DOI 10.3390/ijms22158208

Yusuf M, 2021, J DRUG DELIV SCI TEC, V61, DOI 10.1016/j.jddst.2020.102214

Zaher MF, 2019, J PHARM RES INT, V31, DOI 10.9734/JPRI/2019/v31i630358

NR 79

TC 24

Z9 24

U1 1

U2 13

PU MDPI

PI BASEL

PA ST ALBAN-ANLAGE 66, CH-4052 BASEL, SWITZERLAND

EI 1424-8247

J9 PHARMACEUTICALS-BASE

JI Pharmaceuticals

PD APR

PY 2022

VL 15

IS 4

AR 408

DI 10.3390/ph15040408

PG 11

WC Chemistry, Medicinal; Pharmacology & Pharmacy

WE Science Citation Index Expanded (SCI-EXPANDED)

SC Pharmacology & Pharmacy

GA 0Q8FJ

UT WOS:000785147100001

PM 35455405

OA Green Published, gold

DA 2024-08-12

ER

PT J

AU Sun, Y

Yang, YT

Liu, SS

Yang, SW

Chen, C

Lin, MY

Zeng, Q

Long, JP

Yao, J

Yi, F

Meng, L

Ai, QD

Chen, NH

AF Sun, Yang

Yang, Yantao

Liu, Shasha

Yang, Songwei

Chen, Chen

Lin, Meiyu

Zeng, Qi

Long, Junpeng

Yao, Jiao

Yi, Fan

Meng, Lei

Ai, Qidi

Chen, Naihong

TI New Therapeutic Approaches to and Mechanisms of Ginsenoside Rg1 against

Neurological Diseases

SO CELLS

LA English

DT Review

DE ginsenoside; Rg1; inflammation; oxidative stress

ID DEPRESSION-LIKE BEHAVIORS; OXIDATIVE STRESS; PANAX-GINSENG;

MOLECULAR-MECHANISMS; COGNITIVE IMPAIRMENT; MOUSE MODEL;

NEUROINFLAMMATION; APOPTOSIS; MICE; ACTIVATION

AB Neurological diseases, including Parkinson's disease (PD), Alzheimer's disease (AD), Huntington's disease (HD), stroke, cerebral infarction, ischemia-reperfusion injury, depression and, stress, have high incidence and morbidity and often lead to disability. However, there is no particularly effective medication against them. Therefore, finding drugs with a suitable efficacy, low toxicity and manageable effects to improve the quality of life of patients is an urgent problem. Ginsenoside Rg1 (Rg1) is the main active component of ginseng and has a variety of pharmacological effects. In this review, we focused on the therapeutic potential of Rg1 for improving neurological diseases. We introduce the mechanisms of Ginsenoside Rg1 in neurological diseases, including apoptosis, neuroinflammation, the microRNA (miRNA) family, the mitogen-activated protein kinase (MAPK) family, oxidative stress, nuclear factor-kappa B (NF-kappa B), and learning and memory of Rg1 in neurological diseases. In addition, Rg1 can also improve neurological diseases through the interaction of different signal pathways. The purpose of this review is to explore more in-depth ideas for the clinical treatment of neurological diseases (including PD, AD, HD, stroke, cerebral infarction, ischemia-reperfusion injury, depression, and stress). Therefore, Rg1 is expected to become a new therapeutic method for the clinical treatment of neurological diseases.

C1 [Sun, Yang; Yang, Yantao; Yang, Songwei; Lin, Meiyu; Zeng, Qi; Long, Junpeng; Yao, Jiao; Meng, Lei; Ai, Qidi; Chen, Naihong] Hunan Univ Chinese Med, Coll Pharm, Hunan Engn Technol Ctr Standardizat & Funct Chine, Changsha 410208, China.

[Liu, Shasha] Xiangtan Cent Hosp, Pharm Dept, Xiangtan 411100, China.

[Chen, Chen] Lanzhou Univ, Dept Pharm, Hosp 1, Lanzhou 730000, China.

[Yi, Fan] Beijing Technol & Business Univ, Key Lab Cosmet, China Natl Light Ind, Beijing 100048, China.

[Chen, Naihong] Chinese Acad Med Sci & Peking Union Med Coll, State Key Lab Bioact Subst & Funct Nat Med, Inst Mat Med, Beijing 100050, China.

[Chen, Naihong] Chinese Acad Med Sci & Peking Union Med Coll, Neurosci Ctr, Beijing 100050, China.

C3 Hunan University of Chinese Medicine; Lanzhou University; Beijing

Technology & Business University; Chinese Academy of Medical Sciences -

Peking Union Medical College; Peking Union Medical College; Chinese

Academy of Medical Sciences - Peking Union Medical College; Peking Union

Medical College

RP Ai, QD; Chen, NH (corresponding author), Hunan Univ Chinese Med, Coll Pharm, Hunan Engn Technol Ctr Standardizat & Funct Chine, Changsha 410208, China.; Chen, NH (corresponding author), Chinese Acad Med Sci & Peking Union Med Coll, State Key Lab Bioact Subst & Funct Nat Med, Inst Mat Med, Beijing 100050, China.; Chen, NH (corresponding author), Chinese Acad Med Sci & Peking Union Med Coll, Neurosci Ctr, Beijing 100050, China.

EM 13357329630@163.com; chennh@imm.ac.cn

RI Ai, Qidi/AGS-8732-2022

OI Chen, Nai-Hong/0000-0001-6354-7853; Ai, qidi/0000-0002-1647-2825; YI,

Fan/0000-0002-7476-616X; Yang, Songwei/0000-0003-4040-1215

FU Outstanding youth project of the Hunan Education Department [21B0354];

Changsha Natural Science Foundation [kq2202269, kq2014091]; Hunan

Natural Science Foundation [2021JJ30512, 2022JJ40456]; Research projects

of traditional Chinese medicine in Hunan Province [2021065]; Special

Scientific and Technological Projectfor Comprehensive Utilization of

Ampelopsis grossedentata Resources of Hunan Qiankun Biotechnology Co.,

Ltd. [212010]; Key Project of the Hunan University of Chinese Medicine

School level Scientific Research Fund [2019xjjj001]; Hunan University of

Chinese Medicine First-class Disciple Construction Project of Chinese

Material Medica; key discipline of biological engineering of the Hunan

University of Chinese Medicine [3]

FX This study was supported by the Outstanding youth project of the Hunan

Education Department (No. 21B0354); the Changsha Natural Science

Foundation (No. kq2202269, kq2014091); the Hunan Natural Science

Foundation (No. 2021JJ30512, 2022JJ40456); Research projects of

traditional Chinese medicine in Hunan Province (No. 2021065); the

Special Scientific and Technological Project for Comprehensive

Utilization of Ampelopsis grossedentata Resources of Hunan Qiankun

Biotechnology Co., Ltd. (No. 212010); the Key Project of the Hunan

University of Chinese Medicine School level Scientific Research Fund

(No. 2019xjjj001); the Hunan University of Chinese Medicine First-class

Disciple Construction Project of Chinese Material Medica; and the key

discipline of biological engineering of the Hunan University of Chinese

Medicine (2018) No. 3.

CR Abeysinghe AADT, 2020, LIFE SCI, V256, DOI 10.1016/j.lfs.2020.117996

Ahmed T, 2016, BRAIN RES BULL, V125, P30, DOI 10.1016/j.brainresbull.2016.04.002

Akiguchi I, 2017, NEUROPATHOLOGY, V37, P293, DOI 10.1111/neup.12373

Alolga RN, 2020, PHARMACOL RES, V152, DOI 10.1016/j.phrs.2020.104630

Baeg IH, 2013, J GINSENG RES, V37, P1, DOI 10.5142/jgr.2013.37.1

Breijyeh Z, 2020, MOLECULES, V25, DOI 10.3390/molecules25245789

Brinkmalm A, 2014, MOL NEURODEGENER, V9, DOI 10.1186/1750-1326-9-53

Calcia MA, 2016, PSYCHOPHARMACOLOGY, V233, P1637, DOI 10.1007/s00213-016-4218-9

Caputo M, 2019, PITUITARY, V22, P261, DOI 10.1007/s11102-019-00956-w

Chen F, 2021, PHARMACOL RES, V165, DOI 10.1016/j.phrs.2021.105469

Chen LB, 2018, NEUROCHEM RES, V43, P430, DOI 10.1007/s11064-017-2438-y

Chen WW, 2016, MOL MED REP, V13, P3391, DOI 10.3892/mmr.2016.4948

Chen XC, 2003, EUR J PHARMACOL, V473, P1, DOI 10.1016/S0014-2999(03)01945-9

Chu SF, 2019, ACTA PHARMACOL SIN, V40, P13, DOI 10.1038/s41401-018-0154-z

Dean J, 2017, ASIAN J PSYCHIATR, V27, P101, DOI 10.1016/j.ajp.2017.01.025

Fan CQ, 2018, FRONT IMMUNOL, V9, DOI 10.3389/fimmu.2018.02889

Fan CQ, 2018, NEUROPHARMACOLOGY, V131, P364, DOI 10.1016/j.neuropharm.2018.01.009

Fan XM, 2019, EUR J PHARMACOL, V854, P247, DOI 10.1016/j.ejphar.2019.04.027

Gao SC, 2019, BIOMED PHARMACOTHER, V114, DOI 10.1016/j.biopha.2019.108833

Gao Y, 2020, INT IMMUNOPHARMACOL, V87, DOI 10.1016/j.intimp.2020.106805

Gao Y, 2020, EUR J PHARMACOL, V866, DOI 10.1016/j.ejphar.2019.172801

Ghasemi R, 2013, MOL NEUROBIOL, V47, P1045, DOI 10.1007/s12035-013-8404-z

Glass CK, 2010, CELL, V140, P918, DOI 10.1016/j.cell.2010.02.016

Guo YQ, 2021, MOL MED REP, V23, DOI 10.3892/mmr.2021.11931

Hampel H, 2013, J ALZHEIMERS DIS, V33, pS79, DOI 10.3233/JAD-2012-129003

He CY, 2016, ACTA PHARM SIN B, V6, P593, DOI 10.1016/j.apsb.2016.05.001

He F, 2020, FRONT PHARMACOL, V10, DOI 10.3389/fphar.2019.01565

He JY, 2020, OXID MED CELL LONGEV, V2020, DOI 10.1155/2020/6137521

He YB, 2021, EXP THER MED, V21, DOI 10.3892/etm.2021.9984

Huang L, 2016, NEURAL REGEN RES, V11, P319, DOI 10.4103/1673-5374.177741

Huang QY, 2018, J NEUROINFLAMM, V15, DOI 10.1186/s12974-018-1387-y

Huang X, 2019, MOLECULES, V24, DOI 10.3390/molecules24162939

Iadecolar C, 2020, J CLIN INVEST, V130, P2777, DOI 10.1172/JCI135530

Im DS, 2020, BIOMOLECULES, V10, DOI 10.3390/biom10030444

Jankovic J, 2020, J NEUROL NEUROSUR PS, V91, P795, DOI 10.1136/jnnp-2019-322338

Jiang N, 2020, LIFE SCI, V252, DOI 10.1016/j.lfs.2020.117669

Jimenez-Sanchez M, 2017, CSH PERSPECT MED, V7, DOI 10.1101/cshperspect.a024240

Jin Z, 2019, BIOMED PHARMACOTHER, V120, DOI 10.1016/j.biopha.2019.109452

Kim JH, 2017, J GINSENG RES, V41, P435, DOI 10.1016/j.jgr.2016.08.004

Kim J, 2018, FOOD SCI BIOTECHNOL, V27, P227, DOI 10.1007/s10068-017-0255-3

Kim JH, 2018, J GINSENG RES, V42, P264, DOI 10.1016/j.jgr.2017.10.004

Kuriakose D, 2020, INT J MOL SCI, V21, DOI 10.3390/ijms21207609

Kustrimovic N, 2019, CURR MED CHEM, V26, P3719, DOI 10.2174/0929867325666181009161048

Labbadia J, 2013, TRENDS BIOCHEM SCI, V38, P378, DOI 10.1016/j.tibs.2013.05.003

Li H, 2014, MOL MED REP, V9, P395, DOI 10.3892/mmr.2013.1822

Li Y, 2020, OXID MED CELL LONGEV, V2020, DOI 10.1155/2020/2325391

Liang HY, 2021, AGING-US, V13, P7549, DOI 10.18632/aging.202619

Liu JQ, 2020, ACTA PHARMACOL SIN, V41, P523, DOI 10.1038/s41401-020-0358-x

Mandrekar-Colucci S, 2013, ASN NEURO, V5, DOI 10.1042/AN20130030

Mao YL, 2015, MEDIAT INFLAMM, V2015, DOI 10.1155/2015/372931

Mazon JN, 2017, LIFE SCI, V182, P22, DOI 10.1016/j.lfs.2017.06.002

McColgan P, 2018, EUR J NEUROL, V25, P24, DOI 10.1111/ene.13413

Ménard C, 2016, NEUROSCIENCE, V321, P138, DOI 10.1016/j.neuroscience.2015.05.053

Menozzi E, 2021, ANN MED, V53, P611, DOI 10.1080/07853890.2021.1890330

Menzies FM, 2015, NAT REV NEUROSCI, V16, P345, DOI 10.1038/nrn3961

Mogi M, 2007, NEUROSCI LETT, V414, P94, DOI 10.1016/j.neulet.2006.12.003

Nakhjavani M, 2020, MOLECULES, V25, DOI 10.3390/molecules25214905

Nie LL, 2017, OXID MED CELL LONGEV, V2017, DOI 10.1155/2017/6473506

Ohl K, 2016, EXP NEUROL, V277, P58, DOI 10.1016/j.expneurol.2015.11.010

Peng C, 2020, NAT REV NEUROL, V16, P199, DOI 10.1038/s41582-020-0333-7

Pisanu A, 2014, NEUROBIOL DIS, V71, P280, DOI 10.1016/j.nbd.2014.08.011

Quan Qk L.X., 2016, Chin. J. Integr. Med, DOI [10.1007/s11655-015-2301-4, DOI 10.1007/S11655-015-2301-4]

Rabbito A, 2020, INT J MOL SCI, V21, DOI 10.3390/ijms21061989

Ren TT, 2021, INFLAMM RES, V70, P1151, DOI 10.1007/s00011-021-01513-7

Sabouri-Rad S, 2017, CURR PHARM DESIGN, V23, P1649, DOI 10.2174/1381612822666161021152322

Shen F, 2021, NEURAL PLAST, V2021, DOI 10.1155/2021/2412220

Shen JY, 2017, INT J NANOMED, V12, P6477, DOI 10.2147/IJN.S139602

Shi R, 2018, NEUROCHEM RES, V43, P1529, DOI 10.1007/s11064-018-2567-y

Shim JW, 2018, INT J MOL SCI, V19, DOI 10.3390/ijms19010275

Song L, 2017, OXID MED CELL LONGEV, V2017, DOI 10.1155/2017/2163053

Strunk DR, 2013, COGNITIVE THER RES, V37, P1214, DOI 10.1007/s10608-013-9570-z

Stüve O, 2014, CLIN EXP IMMUNOL, V175, P333, DOI 10.1111/cei.12260

Sun MY, 2017, INT J MOL MED, V39, P507, DOI 10.3892/ijmm.2017.2857

Toman NG, 2019, CELL TRANSPLANT, V28, P1123, DOI 10.1177/0963689719863784

Vaváková M, 2015, OXID MED CELL LONGEV, V2015, DOI 10.1155/2015/898393

Walter C, 2016, NEUROPHARMACOLOGY, V108, P24, DOI 10.1016/j.neuropharm.2016.04.041

Wang HQ, 2021, FRONT PHARMACOL, V12, DOI 10.3389/fphar.2021.709019

Wang KZ, 2017, PHYTOTHER RES, V31, P81, DOI 10.1002/ptr.5733

Wang LM, 2020, FEMS MICROBIOL LETT, V367, DOI 10.1093/femsle/fnaa011

Wang QL, 2019, MEDIAT INFLAMM, V2019, DOI 10.1155/2019/6453296

Wang S, 2017, NEUROCHEM RES, V42, P583, DOI 10.1007/s11064-016-2113-8

Wang Y, 2016, INT J BIOL SCI, V12, P1341, DOI 10.7150/ijbs.15992

Wang YS, 2019, PHARMACOL RES, V150, DOI 10.1016/j.phrs.2019.104520

Wei XY, 2018, BIOFACTORS, V44, P327, DOI 10.1002/biof.1432

Wilkins HM, 2016, CURR TOP MED CHEM, V16, P849, DOI 10.2174/1568026615666150827095102

Wu JY, 2016, INT J MOL MED, V37, P781, DOI 10.3892/ijmm.2016.2485

Xie CL, 2015, SCI REP-UK, V5, DOI 10.1038/srep07790

Xie WJ, 2018, CELLS-BASEL, V7, DOI 10.3390/cells7120270

Xiong X, 2019, INT J BIOL MACROMOL, V126, P842, DOI 10.1016/j.ijbiomac.2018.12.266

Xu TZ, 2019, INT J MOL MED, V43, P717, DOI 10.3892/ijmm.2018.4005

Xu WQ, 2017, MOLECULES, V22, DOI 10.3390/molecules22091518

Xu ZM, 2018, INT J MOL SCI, V19, DOI 10.3390/ijms19113658

Xue QQ, 2021, J GINSENG RES, V45, P22, DOI 10.1016/j.jgr.2020.07.002

Yang QQ, 2019, GLIA, V67, P1017, DOI 10.1002/glia.23571

Yang X, 2021, ACTA PHARMACOL SIN, V42, P1409, DOI 10.1038/s41401-020-00558-4

Yang Y, 2022, CHEM BIOL DRUG DES, V99, P884, DOI 10.1111/cbdd.14041

Yang YJ, 2020, FRONT PHARMACOL, V11, DOI 10.3389/fphar.2020.00834

Yun TK, 2001, LANCET ONCOL, V2, P49, DOI 10.1016/S1470-2045(00)00196-0

Zhai KF, 2021, ACTA PHARM SIN B, V11, P3493, DOI 10.1016/j.apsb.2021.03.032

Zhang L, 2017, J ALZHEIMERS DIS, V60, P1241, DOI 10.3233/JAD-170020

Zhang ZR, 2021, MOL NEUROBIOL, V58, P1550, DOI 10.1007/s12035-020-02213-9

Zhao YF, 2022, INT J MOL MED, V49, DOI 10.3892/ijmm.2021.5070

Zheng X, 2014, NEUROSCIENCE, V256, P210, DOI 10.1016/j.neuroscience.2013.10.023

Zheng X, 2016, SCI REP-UK, V6, DOI 10.1038/srep19406

Zhong SJ, 2020, INT J MED SCI, V17, P1048, DOI 10.7150/ijms.43979

Zhou P, 2019, CELLS-BASEL, V8, DOI 10.3390/cells8030204

Zhou Q, 2014, THROMB RES, V133, P57, DOI 10.1016/j.thromres.2013.10.032

Zhu XZ, 2016, EUR J NEUROSCI, V44, P1878, DOI 10.1111/ejn.13255

Zuccato C, 2010, PHYSIOL REV, V90, P905, DOI 10.1152/physrev.00041.2009

Zusso M, 2019, J NEUROINFLAMM, V16, DOI 10.1186/s12974-019-1538-9

NR 110

TC 31

Z9 33

U1 9

U2 53

PU MDPI

PI BASEL

PA ST ALBAN-ANLAGE 66, CH-4052 BASEL, SWITZERLAND

EI 2073-4409

J9 CELLS-BASEL

JI Cells

PD AUG

PY 2022

VL 11

IS 16

AR 2529

DI 10.3390/cells11162529

PG 19

WC Cell Biology

WE Science Citation Index Expanded (SCI-EXPANDED)

SC Cell Biology

GA 4C0TS

UT WOS:000846178100001

PM 36010610

OA gold, Green Published

DA 2024-08-12

ER

PT J

AU Guo, TT

Zhang, Z

Sun, Y

Zhu, RY

Wang, FX

Ma, LJ

Jiang, L

Liu, HD

AF Guo, Tong-Tong

Zhang, Zheng

Sun, Yan

Zhu, Rui-Yang

Wang, Fei-Xia

Ma, Lian-Ju

Jiang, Lin

Liu, Han-Deng

TI Neuroprotective Effects of Sodium Butyrate by Restoring Gut Microbiota

and Inhibiting TLR4 Signaling in Mice with MPTP-Induced Parkinson's

Disease

SO NUTRIENTS

LA English

DT Article

DE sodium butyrate; microbiome-gut-brain axis; neuroinflammation; gut

microbial dysbiosis; Parkinson's disease

ID MOUSE MODEL; MOTOR DEFICITS; BRAIN AXIS; NEUROINFLAMMATION; MODULATION;

DYSBIOSIS; INFLAMMATION; PATHWAY

AB Parkinson's disease (PD) is a prevalent type of neurodegenerative disease. There is mounting evidence that the gut microbiota is involved in the pathogenesis of PD. Sodium butyrate (NaB) can regulate gut microbiota and improve brain functioning in neurological disorders. Hence, we examined whether the neuroprotective function of NaB on PD was mediated by the modulation of gut microbial dysbiosis and revealed its possible mechanisms. Mice were administered 1-methyl-4-phenyl-1,2,3,6-tetrahydropyridine (MPTP) for 7 consecutive days to construct the PD model. NaB gavage was given 2 h after the daily MPTP injections for 21 days. NaB improved the motor functioning of PD mice, increased striatal neurotransmitter levels, and reduced the death of dopaminergic neurons. The 16S rRNA sequencing analysis revealed that NaB restored the gut microbial dysbiosis. NaB also attenuated the intestinal barrier's disruption and reduced serum, colon, and striatal pro-inflammatory cytokines, along with inhibiting the overactivation of glial cells, suggesting an inhibitory effect on inflammation from NaB throughout the gut-brain axis of the PD mice. Mechanistic studies revealed that NaB treatment suppressed the TLR4/MyD88/NF-kB pathway in the colon and striatum. In summary, NaB had a neuroprotective impact on the PD mice, likely linked to its regulation of gut microbiota to inhibit gut-brain axis inflammation.

C1 [Guo, Tong-Tong; Wang, Fei-Xia; Ma, Lian-Ju; Jiang, Lin; Liu, Han-Deng] Chongqing Med Univ, Expt Teaching Ctr, Lab Tissue & Cell Biol, Chongqing 400016, China.

[Guo, Tong-Tong; Zhang, Zheng; Sun, Yan; Zhu, Rui-Yang; Wang, Fei-Xia; Liu, Han-Deng] Chongqing Med Univ, Dept Mol Med, Chongqing 400016, China.

[Guo, Tong-Tong; Zhang, Zheng; Sun, Yan; Zhu, Rui-Yang; Wang, Fei-Xia; Liu, Han-Deng] Chongqing Med Univ, Canc Res Ctr, Chongqing 400016, China.

C3 Chongqing Medical University; Chongqing Medical University; Chongqing

Medical University

RP Liu, HD (corresponding author), Chongqing Med Univ, Expt Teaching Ctr, Lab Tissue & Cell Biol, Chongqing 400016, China.; Liu, HD (corresponding author), Chongqing Med Univ, Dept Mol Med, Chongqing 400016, China.; Liu, HD (corresponding author), Chongqing Med Univ, Canc Res Ctr, Chongqing 400016, China.

EM hdliu@cqmu.edu.cn

RI Zhang, Zheng/ISA-8059-2023

CR Tan AH, 2022, NAT REV NEUROL, V18, P476, DOI 10.1038/s41582-022-00681-2

Armstrong MJ, 2020, JAMA-J AM MED ASSOC, V323, P548, DOI 10.1001/jama.2019.22360

Bartl M, 2022, NEUROBIOL DIS, V170, DOI 10.1016/j.nbd.2022.105744

Belarbi K, 2020, MOL NEURODEGENER, V15, DOI 10.1186/s13024-020-00408-1

Chen GX, 2018, EBIOMEDICINE, V30, P317, DOI 10.1016/j.ebiom.2018.03.030

Chidambaram SB, 2022, PHARMACOL THERAPEUT, V231, DOI 10.1016/j.pharmthera.2021.107988

Cirstea MS, 2020, MOVEMENT DISORD, V35, P1208, DOI 10.1002/mds.28052

Desai MS, 2016, CELL, V167, P1339, DOI 10.1016/j.cell.2016.10.043

Dong XL, 2020, INT J BIOL MACROMOL, V164, P994, DOI 10.1016/j.ijbiomac.2020.07.180

Dorsey ER, 2007, NEUROLOGY, V68, P384, DOI 10.1212/01.wnl.0000247740.47667.03

Drouin-Ouellet J, 2015, INT J NEUROPSYCHOPH, V18, DOI 10.1093/ijnp/pyu103

Fan HX, 2022, CNS NEUROSCI THER, V28, P1675, DOI 10.1111/cns.13916

Freedman SN, 2018, NEUROTHERAPEUTICS, V15, P109, DOI 10.1007/s13311-017-0588-x

Ganesh BP, 2013, PLOS ONE, V8, DOI 10.1371/journal.pone.0074963

Gorecki AM, 2021, TRANSL NEURODEGENER, V10, DOI 10.1186/s40035-021-00271-0

Gutiérrez-Repiso C, 2019, MOL NUTR FOOD RES, V63, DOI 10.1002/mnfr.201900167

Haavik J, 1998, MOL NEUROBIOL, V16, P285, DOI 10.1007/BF02741387

Haikal C, 2019, TRANSL NEURODEGENER, V8, DOI 10.1186/s40035-019-0175-7

Heeney DD, 2018, CURR OPIN BIOTECH, V49, P140, DOI 10.1016/j.copbio.2017.08.004

Hirayama M, 2021, ANN NUTR METAB, V77, P28, DOI 10.1159/000518147

Hirsch EC, 2009, LANCET NEUROL, V8, P382, DOI 10.1016/S1474-4422(09)70062-6

Hou YC, 2021, EXP GERONTOL, V150, DOI 10.1016/j.exger.2021.111376

Jang JH, 2020, BRAIN BEHAV IMMUN, V89, P641, DOI 10.1016/j.bbi.2020.08.015

Jaworska J, 2017, J NEUROINFLAMM, V14, DOI 10.1186/s12974-017-0807-8

Jiang PZ, 2017, SCI REP-UK, V7, DOI 10.1038/s41598-017-08149-w

Kakoty V, 2021, ACS CHEM NEUROSCI, V12, P2643, DOI 10.1021/acschemneuro.1c00144

Keshavarzian A, 2015, MOVEMENT DISORD, V30, P1351, DOI 10.1002/mds.26307

Knudsen KEB, 2003, P NUTR SOC, V62, P81, DOI 10.1079/PNS2002212

Lee SM, 2016, J STEROID BIOCHEM, V164, P265, DOI 10.1016/j.jsbmb.2015.08.018

Lin AQ, 2018, PARKINSONISM RELAT D, V53, P82, DOI 10.1016/j.parkreldis.2018.05.007

Lin TL, 2020, FRONT PHARMACOL, V11, DOI 10.3389/fphar.2020.00554

Liu JM, 2017, J NEUROL SCI, V381, P176, DOI 10.1016/j.jns.2017.08.3235

Liu J, 2019, J AGR FOOD CHEM, V67, P1674, DOI 10.1021/acs.jafc.8b06359

Lucas K, 2013, MOL NEUROBIOL, V48, P190, DOI 10.1007/s12035-013-8425-7

Ma XM, 2020, CELL BIOL TOXICOL, V36, P509, DOI 10.1007/s10565-020-09518-4

Menozzi E, 2021, ANN MED, V53, P611, DOI 10.1080/07853890.2021.1890330

Metta V, 2022, J NEUROL, V269, P1154, DOI 10.1007/s00415-021-10567-w

Miyazaki I, 2020, CELLS-BASEL, V9, DOI 10.3390/cells9122623

Paiva I, 2017, HUM MOL GENET, V26, P2231, DOI 10.1093/hmg/ddx114

Pavlov VA, 2015, IMMUNOL RES, V63, P38, DOI 10.1007/s12026-015-8718-1

Perez-Pardo P, 2019, GUT, V68, P829, DOI 10.1136/gutjnl-2018-316844

Poewe W, 2017, NAT REV DIS PRIMERS, V3, DOI 10.1038/nrdp.2017.13

Qiu J, 2020, PHARMACOLOGY, V105, P550, DOI 10.1159/000505132

Rahimifard M, 2017, AGEING RES REV, V36, P11, DOI 10.1016/j.arr.2017.02.004

Rocha DM, 2016, ATHEROSCLEROSIS, V244, P211, DOI 10.1016/j.atherosclerosis.2015.11.015

Romano S, 2021, NPJ PARKINSONS DIS, V7, DOI 10.1038/s41531-021-00156-z

Salama NN, 2006, ADV DRUG DELIVER REV, V58, P15, DOI 10.1016/j.addr.2006.01.003

Sampson TR, 2016, CELL, V167, P1469, DOI 10.1016/j.cell.2016.11.018

Scheperjans F, 2015, MOVEMENT DISORD, V30, P350, DOI 10.1002/mds.26069

Sharma S, 2015, BEHAV BRAIN RES, V291, P306, DOI 10.1016/j.bbr.2015.05.052

Shin NR, 2015, TRENDS BIOTECHNOL, V33, P496, DOI 10.1016/j.tibtech.2015.06.011

St Laurent R, 2013, NEUROSCIENCE, V246, P382, DOI 10.1016/j.neuroscience.2013.04.037

Sun J, 2021, BRAIN BEHAV IMMUN, V91, P703, DOI 10.1016/j.bbi.2020.10.014

Sun MF, 2018, BRAIN BEHAV IMMUN, V70, P48, DOI 10.1016/j.bbi.2018.02.005

Sun MF, 2018, AGEING RES REV, V45, P53, DOI 10.1016/j.arr.2018.04.004

Tritsch NX, 2012, NEURON, V76, P33, DOI 10.1016/j.neuron.2012.09.023

Valguarnera E, 2020, J MOL BIOL, V432, P765, DOI 10.1016/j.jmb.2019.12.003

van IJzendoorn SCD, 2019, J PARKINSON DIS, V9, pS323, DOI 10.3233/JPD-191707

Varesi A, 2022, INT J MOL SCI, V23, DOI 10.3390/ijms232012289

Vegas-Suarez S, 2019, INT REV NEUROBIOL, V146, P259, DOI 10.1016/bs.irn.2019.06.013

Vieira ELM, 2012, J NUTR BIOCHEM, V23, P430, DOI 10.1016/j.jnutbio.2011.01.007

Wang Q, 2021, BRAIN, V144, P2571, DOI 10.1093/brain/awab156

Xie WJ, 2020, NEUROTOXICOLOGY, V76, P200, DOI 10.1016/j.neuro.2019.11.009

Yang Y, 2016, CELL DEATH DIS, V7, DOI 10.1038/cddis.2016.140

Zhang Y, 2022, BRAIN RES, V1776, DOI 10.1016/j.brainres.2021.147749

Zhao JB, 2021, NUTRIENTS, V13, DOI 10.3390/nu13082756

Zhao Z, 2021, MICROBIOME, V9, DOI 10.1186/s40168-021-01107-9

Zhao Z, 2021, ACTA PHARM SIN B, V11, P2859, DOI 10.1016/j.apsb.2021.03.020

Zheng SY, 2021, AGEING RES REV, V69, DOI 10.1016/j.arr.2021.101347

Zhou D, 2017, WORLD J GASTROENTERO, V23, P60, DOI 10.3748/wjg.v23.i1.60

Zhou XT, 2021, OXID MED CELL LONGEV, V2021, DOI 10.1155/2021/9424582

Zhou ZL, 2019, NEUROTHERAPEUTICS, V16, P741, DOI 10.1007/s13311-019-00719-2

Zhu ML, 2022, FRONT IMMUNOL, V13, DOI 10.3389/fimmu.2022.937555

NR 73

TC 18

Z9 20

U1 15

U2 34

PU MDPI

PI BASEL

PA ST ALBAN-ANLAGE 66, CH-4052 BASEL, SWITZERLAND

EI 2072-6643

J9 NUTRIENTS

JI Nutrients

PD FEB

PY 2023

VL 15

IS 4

AR 930

DI 10.3390/nu15040930

PG 18

WC Nutrition & Dietetics

WE Science Citation Index Expanded (SCI-EXPANDED)

SC Nutrition & Dietetics

GA 9J2MA

UT WOS:000940026500001

PM 36839287

OA Green Published, gold

DA 2024-08-12

ER

PT J

AU Lu, BW

Baum, L

So, KF

Chiu, K

Xie, LK

AF Lu, Bing-Wen

Baum, Larry

So, Kwok-Fai

Chiu, Kin

Xie, Li-Ke

TI More than anti-malarial agents: therapeutic potential of artemisinins in

neurodegeneration

SO NEURAL REGENERATION RESEARCH

LA English

DT Review

DE artemisinin; inflammation; neuroinflammation; neurodegeneration;

Alzheimer's disease; Parkinson's disease; A beta; anti-oxidation;

neuroprotection; neural regeneration

ID alzheimer's disease; PC12 CELLS; IN-VITRO; NEUROINFLAMMATION; ARTESUNATE;

ACTIVATION; MECHANISMS; MODELS; NEUROPROTECTION; EXPOSURE

AB Artemisinin, also called qinghaosu, is originally derived from the sweet wormwood plant (Artemisia annua), which is used in traditional Chinese medicine. Artemisinin and its derivatives (artemisinins) have been widely used for many years as anti-malarial agents, with few adverse side effects. Interestingly, evidence has recently shown that artemisinins might have a therapeutic value for several other diseases beyond malaria, including cancers, inflammatory diseases, and autoimmune disorders. Neurodegeneration is a challenging age-associated neurological disorder characterized by deterioration of neuronal structures as well as functions, whereas neuroinflammation has been considered to be an underlying factor in the development of various neurodegenerative disorders, including Alzheimer's disease. Recently discovered properties of artemisinins suggested that they might be used to treat neurodegenerative disorders by decreasing oxidation, inflammation, and amyloid beta protein (A beta). In this review, we will introduce artemisinins and highlight the possible mechanisms of their neuroprotective activities, suggesting that artemisinins might have therapeutic potential in neurodegenerative disorders.

C1 [Lu, Bing-Wen; Xie, Li-Ke] China Acad Chinese Med Sci, Eye Hosp, Dept Ophthalmol, Beijing, China.

[Lu, Bing-Wen; So, Kwok-Fai; Chiu, Kin] Univ Hong Kong, Li Ka Shing Fac Med, Dept Ophthalmol, Hong Kong, China.

[Baum, Larry; So, Kwok-Fai; Chiu, Kin] Univ Hong Kong, State Key Lab Brain & Cognit Sci, Hong Kong, China.

[Baum, Larry; So, Kwok-Fai] Univ Hong Kong, Li Ka Shing Fac Med, Ctr Genom Sci, Hong Kong Li Ka Shing Fac Med, Hong Kong, China.

[Baum, Larry] Univ Hong Kong, Hong Kong Li Ka Shing Fac Med, Li Ka Shing Fac Med, Dept Psychiat, Hong Kong, China.

[So, Kwok-Fai] Jinan Univ, GHM Inst CNS Regenerat, Guangzhou, Guangdong, China.

C3 China Academy of Chinese Medical Sciences; Eye Hospital, CACMS;

University of Hong Kong; University of Hong Kong; University of Hong

Kong; University of Hong Kong; Jinan University

RP Xie, LK (corresponding author), China Acad Chinese Med Sci, Eye Hosp, Dept Ophthalmol, Beijing, China.; Chiu, K (corresponding author), Univ Hong Kong, Li Ka Shing Fac Med, Dept Ophthalmol, Hong Kong, China.; Chiu, K (corresponding author), Univ Hong Kong, State Key Lab Brain & Cognit Sci, Hong Kong, China.

EM datwai@hku.hk; bjxielike@sina.com

RI Hubsher, Marshall/Q-1825-2019; Baum, Lawrence/B-1005-2013; Hua,

QX/HGE-3640-2022; Chiu, Kin/ABI-7938-2020

OI Hubsher, Marshall/0000-0002-9775-0400; Baum,

Lawrence/0000-0002-5345-9355; Chiu, Kin/0000-0001-6581-9007; Bingwen,

Lu/0000-0001-5877-6459; Hubsher, Marshall/0000-0002-7315-8947; So,

Kwok-Fai/0000-0003-4039-4246

FU Natural Science Foundation of Beijing of China [7192235]

FX This work was supported by the Natural Science Foundation of Beijing of

China, No. 7192235 (to LKX). The funding body played no role in the

manuscript preparation other than providing funding.

CR Abdalkader M, 2018, FRONT NEUROSCI-SWITZ, V12, DOI 10.3389/fnins.2018.00466

Alexander RW, 2017, CLIN TRIAL DEGEN DIS, V2, P1

Artemisinin structure research collaboration group, 1977, KEXUE TONGBAO, V22, P142

Bartolotti N, 2016, MOL PSYCHIATR, V21, P1158, DOI 10.1038/mp.2016.111

Bohush A, 2018, INT J MOL SCI, V19, DOI 10.3390/ijms19102973

Bourne KZ, 2007, J NEUROSCI RES, V85, P1194, DOI 10.1002/jnr.21252

Braidy N, 2017, NEURAL REGEN RES, V12, P39, DOI 10.4103/1673-5374.198971

Caccamo A, 2011, J BIOL CHEM, V286, P8924, DOI 10.1074/jbc.M110.180638

Camara H, 2018, J NEUROSCI, V38, P9123, DOI 10.1523/JNEUROSCI.1592-18.2018

Chen WJ, 2016, BIOMED J, V39, P93, DOI 10.1016/j.bj.2016.04.002

Cheng C, 2011, PLOS ONE, V6, DOI 10.1371/journal.pone.0020932

Chong CM, 2016, REDOX BIOL, V9, P50, DOI 10.1016/j.redox.2016.06.002

De Felice FG, 2013, J CLIN INVEST, V123, P531, DOI 10.1172/JCI64595

Efferth T, 2017, PHYTOMEDICINE, V37, P58, DOI 10.1016/j.phymed.2017.11.003

Ettcheto M, 2018, MOL NEUROBIOL, V55, P4885, DOI 10.1007/s12035-017-0690-4

Feldmann KG, 2019, J NEUROCHEM, V148, P531, DOI 10.1111/jnc.14629

Feng X, 2017, LUPUS, V26, P62, DOI 10.1177/0961203316651738

Guo ZR, 2016, ACTA PHARM SIN B, V6, P115, DOI 10.1016/j.apsb.2016.01.008

He PD, 2018, J AGR FOOD CHEM, V66, P10205, DOI 10.1021/acs.jafc.8b03731

Horwood JM, 2006, EUR J NEUROSCI, V23, P3375, DOI 10.1111/j.1460-9568.2006.04859.x

Hou LF, 2009, INT IMMUNOPHARMACOL, V9, P1509, DOI 10.1016/j.intimp.2009.09.003

Kim MJ, 2017, NANOMED-NANOTECHNOL, V13, P2533, DOI 10.1016/j.nano.2017.06.022

Kreiner G, 2018, PHARMACOL REP, V70, P1105, DOI 10.1016/j.pharep.2018.09.006

Lafay-Chebassier C, 2005, J NEUROCHEM, V94, P215, DOI 10.1111/j.1471-4159.2005.03187.x

Li T, 2018, MOL MED REP, V17, P8385, DOI 10.3892/mmr.2018.8887

Lin SP, 2018, FRONT CELL NEUROSCI, V12, DOI 10.3389/fncel.2018.00108

Lin ZM, 2016, SCI REP-UK, V6, DOI 10.1038/srep38115

Lu H, 2018, MOL MED REP, V17, P6639, DOI 10.3892/mmr.2018.8666

McCauley ME, 2019, ACTA NEUROPATHOL, V137, P715, DOI 10.1007/s00401-018-1933-9

Morroni F, 2018, AGING DIS, V9, P605, DOI 10.14336/AD.2017.0903

Motaghinejad M, 2017, NEUROCHEM RES, V42, P2921, DOI 10.1007/s11064-017-2323-8

NAIR MSR, 1986, J NAT PROD, V49, P504, DOI 10.1021/np50045a021

Niranjan R, 2018, NEUROCHEM INT, V120, P13, DOI 10.1016/j.neuint.2018.07.003

Okorji UP, 2016, MOL NEUROBIOL, V53, P6426, DOI 10.1007/s12035-015-9543-1

Olanow CW, 2009, NEUROLOGY, V72, pS1, DOI 10.1212/WNL.0b013e3181a1d44c

Pinheiro LCS, 2018, AN ACAD BRAS CIENC, V90, P1251, DOI 10.1590/0001-3765201820170830

Qiang WJ, 2018, NEUROSCIENCE, V395, P1, DOI 10.1016/j.neuroscience.2018.10.041

Qin Y., 2017, ONCOTARGET, V8

Raffetin A, 2018, MED MALADIES INFECT, V48, P238, DOI 10.1016/j.medmal.2018.01.004

Sarina, 2013, BRAIN RES, V1490, P61, DOI 10.1016/j.brainres.2012.10.059

Shi CC, 2015, MEDIAT INFLAMM, V2015, DOI 10.1155/2015/435713

Singh S, 2015, MUTAT RES-GEN TOX EN, V777, P1, DOI 10.1016/j.mrgentox.2014.11.001

Subedi L, 2017, BMC NEUROSCI, V18, DOI 10.1186/s12868-017-0399-3

Sun C, 2017, BBA-MOL CELL RES, V1864, P1285, DOI 10.1016/j.bbamcr.2017.04.014

Sun ZQ, 2018, CHEM-BIOL INTERACT, V293, P11, DOI 10.1016/j.cbi.2018.07.011

Toosi A, 2019, NEUROPEPTIDES, V74, P88, DOI 10.1016/j.npep.2019.01.001

Tu YY, 2016, ANGEW CHEM INT EDIT, V55, P10210, DOI 10.1002/anie.201601967

Tu YY, 2011, NAT MED, V17, P1217, DOI 10.1038/nm.2471

Wang DT, 2015, SCI CHINA LIFE SCI, V58, P451, DOI 10.1007/s11427-014-4736-9

Wang JX, 2007, EUR J PHARMACOL, V564, P211, DOI 10.1016/j.ejphar.2007.01.092

Wong YK, 2017, MED RES REV, V37, P1492, DOI 10.1002/med.21446

Yan FX, 2017, ACS CHEM NEUROSCI, V8, P1713, DOI 10.1021/acschemneuro.7b00021

Yuan DS, 2017, FRONT PHARMACOL, V8, DOI 10.3389/fphar.2017.00737

Zeng ZW, 2017, REDOX BIOL, V12, P625, DOI 10.1016/j.redox.2017.04.003

Zhang FX, 2018, BIOMED PHARMACOTHER, V97, P1011, DOI 10.1016/j.biopha.2017.08.132

Zhao YG, 2012, J IMMUNOL, V189, P4417, DOI 10.4049/jimmunol.1200919

Zheng WH, 2016, FREE RADICAL BIO MED, V97, P158, DOI 10.1016/j.freeradbiomed.2016.05.023

Zhou WL, 2005, ACTA PHARMACOL SIN, V26, P1352, DOI 10.1111/j.1745-7254.2005.00232.x

NR 58

TC 16

Z9 18

U1 0

U2 41

PU WOLTERS KLUWER MEDKNOW PUBLICATIONS

PI MUMBAI

PA WOLTERS KLUWER INDIA PVT LTD , A-202, 2ND FLR, QUBE, C T S NO 1498A-2

VILLAGE MAROL, ANDHERI EAST, MUMBAI, 400059, INDIA

SN 1673-5374

EI 1876-7958

J9 NEURAL REGEN RES

JI Neural Regen. Res.

PD SEP

PY 2019

VL 14

IS 9

BP 1494

EP 1498

DI 10.4103/1673-5374.255960

PG 5

WC Cell Biology; Neurosciences

WE Science Citation Index Expanded (SCI-EXPANDED)

SC Cell Biology; Neurosciences & Neurology

GA HY3XE

UT WOS:000468060200004

PM 31089038

OA Green Published, gold

DA 2024-08-12

ER

PT J

AU Niaz, A

Karunia, J

Mandwie, M

Keay, KA

Musumeci, G

Al-Badri, G

Castorina, A

AF Niaz, Aram

Karunia, Jocelyn

Mandwie, Mawj

Keay, Kevin A.

Musumeci, Giuseppe

Al-Badri, Ghaith

Castorina, Alessandro

TI Robust Dopaminergic Differentiation and Enhanced LPS-Induced

Neuroinflammatory Response in Serum-Deprived Human SH-SY5Y Cells:

Implication for Parkinson's Disease

SO JOURNAL OF MOLECULAR NEUROSCIENCE

LA English

DT Article

DE Plasminogen activators; tPA; uPA; Inflammation; Neurodegenerative

diseases; Parkinson's disease; Dopamine; Neuroblastoma; nNOS; AKT

ID TISSUE-PLASMINOGEN ACTIVATOR; NEUROBLASTOMA-CELLS; TRANSCRIPTIONAL

REGULATION; SIGNALING PATHWAY; NUCLEAR FACTORS; MICROGLIA; SYSTEM; GENE;

RECEPTOR; INJURY

AB Parkinson's disease (PD) is a chronic neurodegenerative condition characterized by motor symptoms such as bradykinesia, resting tremor, and rigidity. PD diagnosis is based on medical history, review of signs, symptoms, neurological and physical examinations. Unfortunately, by the time the disease is diagnosed, dopamine (DA) neuronal loss is often extended, thereby resulting in ineffective therapies. Recent evidence suggests that neuroinflammation may be pivotal during PD onset and progression. However, suitable cellular models and biomarkers to detect early signs of neuroinflammation are still missing. In this study, we developed a well-differentiated DAergic neuronal cell line where we triggered a neuroinflammatory response to assess the temporal expression of the tissue- and urokinase plasminogen activators (tPA and uPA) and their endogenous inhibitor (PAI-1) along with that of pro-inflammatory mediators and the neuronal marker nNOS. Human neuroblastoma cells SH-SY5Y were differentiated into DAergic neuronal-like cells using a combination of 12-O-tetradecanoylphorbol-13-acetate (TPA) and serum depletion. Terminally-differentiated neurons were then exposed to lipopolysaccharide (LPS) for short (up to 24 h) or long term (up to 10 days) to mimic acute or chronic inflammation. Results demonstrated that uPA protein expression was stably upregulated during chronic inflammation, whereas the expression of nNOS protein better reflected the cellular response to acute inflammation. Additional studies revealed that the temporal induction of uPA was associated with increased AKT phosphorylation, but did not seem to involve cAMP-responsive element-binding protein (CREB) activation, nor the mitogen-activated protein kinase (MAPK) pathway. In conclusion, our in vitro data suggests that nNOS and uPA may serve as viable candidate biomarkers of acute and chronic neuroinflammation.

C1 [Niaz, Aram; Karunia, Jocelyn; Mandwie, Mawj; Al-Badri, Ghaith; Castorina, Alessandro] Univ Technol Sydney, Fac Sci, Sch Life Sci, Lab Cellular & Mol Neurosci LCMN, POB 123, Broadway, NSW 2007, Australia.

[Keay, Kevin A.; Castorina, Alessandro] Univ Sydney, Fac Med & Hlth, Sch Med Sci, Lab Neural Struct & Funct LNSF, Sydney, NSW 2006, Australia.

[Musumeci, Giuseppe] Univ Catania, Sch Med, Dept Biomed & Biotechnol Sci, Anat Histol & Movement Sci Sect, Via S Sofia 87, I-95123 Catania, Italy.

C3 University of Technology Sydney; University of Sydney; University of

Catania

RP Castorina, A (corresponding author), Univ Technol Sydney, Fac Sci, Sch Life Sci, Lab Cellular & Mol Neurosci LCMN, POB 123, Broadway, NSW 2007, Australia.; Castorina, A (corresponding author), Univ Sydney, Fac Med & Hlth, Sch Med Sci, Lab Neural Struct & Funct LNSF, Sydney, NSW 2006, Australia.

EM Alessandro.Castorina@UTS.edu.au

RI Castorina, Alessandro/C-5495-2013; Musumeci, Giuseppe/K-9955-2016

OI Castorina, Alessandro/0000-0001-7037-759X; Al-Badri,

Ghaith/0000-0003-3342-2376; Mandwie, Mawj/0000-0002-4165-4276; Musumeci,

Giuseppe/0000-0002-8260-8890; Keay, Kevin Anthony/0000-0003-3934-4380;

Niaz, Aram/0000-0001-6636-2555

FU UTS Microscopy Imaging Facility (MIF)

FX We would like to acknowledge A/Prof. Louise Cole and the UTS Microscopy

Imaging Facility (MIF) for the support provided for the acquisition and

processing of the immunofluorescent images shown in this study.

CR Andersen AD, 2017, ACTA NEUROL SCAND, V135, P34, DOI 10.1111/ane.12590

Aquilano K, 2006, FASEB J, V20, P1683, DOI 10.1096/fj.05-5225fje

Avwenagha O, 2003, J NEUROCYTOL, V32, P1077, DOI 10.1023/B:NEUR.0000021903.24849.6c

Baldelli S, 2011, INT J BIOCHEM CELL B, V43, P163, DOI 10.1016/j.biocel.2010.10.016

Baranes D, 1998, NEURON, V21, P813, DOI 10.1016/S0896-6273(00)80597-8

Bennur S, 2007, NEUROSCIENCE, V144, P8, DOI 10.1016/j.neuroscience.2006.08.075

Biosa A, 2019, NEUROTOX RES, V36, P746, DOI 10.1007/s12640-019-00078-y

Brown D, 2014, NEUROTOX RES, V26, P230, DOI 10.1007/s12640-014-9468-x

Brzozowski MJ, 2011, BRAIN RES, V1404, P21, DOI 10.1016/j.brainres.2011.05.063

Bucolo C, 2012, BIOCHEM PHARMACOL, V83, P680, DOI 10.1016/j.bcp.2011.11.031

Bugge TH, 1996, P NATL ACAD SCI USA, V93, P5899, DOI 10.1073/pnas.93.12.5899

Bukhari N, 2011, J NEUROSCI, V31, P14931, DOI 10.1523/JNEUROSCI.3339-11.2011

Cai LJ, 2019, INT IMMUNOPHARMACOL, V75, DOI 10.1016/j.intimp.2019.105734

Calabrese V, 2018, FREE RADICAL BIO MED, V115, P80, DOI 10.1016/j.freeradbiomed.2017.10.379

Castorina A, 2013, NEUROSCIENCE, V250, P546, DOI 10.1016/j.neuroscience.2013.07.053

Castorina A, 2019, NEUROPEPTIDES, V74, P60, DOI 10.1016/j.npep.2018.12.002

Castorina A, 2015, PLOS ONE, V10, DOI 10.1371/journal.pone.0117799

Castorina A, 2011, NEUROCHEM RES, V36, P426, DOI 10.1007/s11064-010-0359-0

Chahine Lama M, 2014, Parkinsonism Relat Disord, V20 Suppl 1, pS99, DOI 10.1016/S1353-8020(13)70025-7

Cheng Z, 2016, MOL SYST BIOL, V12, DOI 10.15252/msb.20156423

Costa M, 1998, EUR J BIOCHEM, V258, P123, DOI 10.1046/j.1432-1327.1998.2580123.x

Cunningham O, 2009, GLIA, V57, P1802, DOI 10.1002/glia.20892

de Pedro-Cuesta J, 2015, PLOS ONE, V10

FENG P, 1990, J BIOL CHEM, V265, P2022

Filograna R, 2015, PLOS ONE, V10, DOI 10.1371/journal.pone.0136769

Forster JI, 2016, J BIOMOL SCREEN, V21, P496, DOI 10.1177/1087057115625190

Franke TF, 2003, ONCOGENE, V22, P8983, DOI 10.1038/sj.onc.1207115

Gitler A.D., 2017, NEURODEGENERATIVE DI

Giunta S, 2010, NEUROPEPTIDES, V44, P45, DOI 10.1016/j.npep.2009.10.003

González H, 2014, J NEUROINFLAMM, V11, DOI 10.1186/s12974-014-0201-8

Gur-Wahnon D, 2013, J NEUROINFLAMM, V10, DOI 10.1186/1742-2094-10-124

Hong H, 2016, INT NEUROUROL J, V20, pS2, DOI 10.5213/inj.1632604.302

Jin XM, 2016, J BIOCHEM, V159, P491, DOI 10.1093/jb/mvw009

Justin A, 2020, NEUROTOX RES, V37, P508, DOI 10.1007/s12640-019-00132-9

Kavya R, 2006, NITRIC OXIDE-BIOL CH, V15, P280, DOI 10.1016/j.niox.2006.07.003

Kim EK, 2015, ARCH TOXICOL, V89, P867, DOI 10.1007/s00204-015-1472-2

Kovalevich J, 2013, METHODS MOL BIOL, V1078, P9, DOI 10.1007/978-1-62703-640-5_2

Lawrimore CJ, 2017, ALCOHOL CLIN EXP RES, V41, P939, DOI 10.1111/acer.13368

Lin YC, 2007, J PHARMACOL EXP THER, V323, P877, DOI 10.1124/jpet.107.125955

Liu YS, 2016, CELL, V165, P535, DOI 10.1016/j.cell.2016.03.014

Lo YC, 2008, TOXICOL APPL PHARM, V228, P247, DOI 10.1016/j.taap.2007.12.001

López-Carballo G, 2002, J BIOL CHEM, V277, P25297, DOI 10.1074/jbc.M201869200

Ma XH, 2018, BRAIN RES, V1690, P1, DOI 10.1016/j.brainres.2018.03.022

Mehra A, 2016, BBA-MOL BASIS DIS, V1862, P395, DOI 10.1016/j.bbadis.2015.10.011

Melchor JP, 2005, THROMB HAEMOSTASIS, V93, P655, DOI 10.1160/TH04-12-0838

Miller DB, 2015, METABOLISM, V64, pS40, DOI 10.1016/j.metabol.2014.10.030

Monaghan TK, 2008, J NEUROCHEM, V104, P74, DOI 10.1111/j.1471-4159.2007.05018.x

Nakano N, 2017, INT J MOL MED, V39, P253, DOI 10.3892/ijmm.2016.2833

OHLSSON M, 1993, MOL CELL BIOL, V13, P266, DOI 10.1128/MCB.13.1.266

Pang PT, 2004, SCIENCE, V306, P487, DOI 10.1126/science.1100135

Pawlak R, 2005, P NATL ACAD SCI USA, V102, P18201, DOI 10.1073/pnas.0509232102

Pineda D, 2012, GLIA, V60, P526, DOI 10.1002/glia.22284

Presgraves SP, 2004, NEUROTOX RES, V5, P579, DOI 10.1007/bf03033178

Rogove AD, 1999, J CELL SCI, V112, P4007

Rommelspacher H, 2012, CURR TOP NEUROTOX, V1, P165, DOI 10.1007/978-1-4614-1542-8_11

Schmittgen TD, 2008, NAT PROTOC, V3, P1101, DOI 10.1038/nprot.2008.73

Siao CJ, 2003, J NEUROSCI, V23, P3234

Son H, 2002, BIOCHEM BIOPH RES CO, V298, P262, DOI 10.1016/S0006-291X(02)02453-1

Sun Y, 2015, J RECEPT SIG TRANSD, V35, P600, DOI 10.3109/10799893.2015.1030412

Surmeier DJ, 2017, NAT REV NEUROSCI, V18, P101, DOI 10.1038/nrn.2016.178

Szepesi Z, 2018, FRONT CELL NEUROSCI, V12, DOI 10.3389/fncel.2018.00323

Titze-de-Almeida SS, 2014, NEUROCHEM RES, V39, P2452, DOI 10.1007/s11064-014-1449-1

Tjärnlund-Wolf A, 2014, GENE REGUL SYST BIO, V8, P113, DOI 10.4137/GRSB.S13387

van Horssen J., 2017, Neurosci Lett

Wu YC, 2011, ARCH NEUROL-CHICAGO, V68, P22, DOI 10.1001/archneurol.2010.321

Yan TX, 2019, EXP GERONTOL, V128, DOI 10.1016/j.exger.2019.110743

Zhang HA, 2012, BIOCHEM BIOPH RES CO, V421, P479, DOI 10.1016/j.bbrc.2012.04.021

Zhang X, 2020, J NEUROINFLAMM, V17, DOI 10.1186/s12974-020-1712-0

NR 68

TC 5

Z9 5

U1 0

U2 4

PU SPRINGERNATURE

PI LONDON

PA CAMPUS, 4 CRINAN ST, LONDON, N1 9XW, United Kingdom

SN 0895-8696

EI 1559-1166

J9 J MOL NEUROSCI

JI J. Mol. Neurosci.

PD MAR

PY 2021

VL 71

IS 3

BP 565

EP 582

DI 10.1007/s12031-020-01678-6

EA AUG 2020

PG 18

WC Biochemistry & Molecular Biology; Neurosciences

WE Science Citation Index Expanded (SCI-EXPANDED)

SC Biochemistry & Molecular Biology; Neurosciences & Neurology

GA QA9UW

UT WOS:000559492100001

PM 32789724

DA 2024-08-12

ER

PT J

AU Zingale, VD

Gugliandolo, A

Mazzon, E

AF Zingale, Valeria Domenica

Gugliandolo, Agnese

Mazzon, Emanuela

TI MiR-155: An Important Regulator of Neuroinflammation

SO INTERNATIONAL JOURNAL OF MOLECULAR SCIENCES

LA English

DT Review

DE miR-155; neuroinflammation; microRNAs; neurodegenerative disease;

multiple sclerosis; Alzheimer's disease; Parkinson's disease; ischemic

stroke; amyotrophic lateral sclerosis

ID GUALOU GUIZHI DECOCTION; TRAUMATIC BRAIN-INJURY; MULTIPLE-SCLEROSIS;

alzheimer's disease; INFLAMMATORY RESPONSE; IMMUNE-RESPONSE; MICRORNA

155; MICROGLIA; GENE; RNA

AB MicroRNAs (miRNAs) are small non-coding RNA molecules that regulate gene expression at the post-transcriptional level and that play an important role in many cellular processes, including modulation of inflammation. MiRNAs are present in high concentrations in the central nervous system (CNS) and are spatially and temporally expressed in a specific way. Therefore, an imbalance in the expression pattern of these small molecules can be involved in the development of neurological diseases. Generally, CNS responds to damage or disease through the activation of an inflammatory response, but many neurological disorders are characterized by uncontrolled neuroinflammation. Many studies support the involvement of miRNAs in the activation or inhibition of inflammatory signaling and in the promotion of uncontrolled neuroinflammation with pathological consequences. MiR-155 is a pro-inflammatory mediator of the CNS and plays an important regulatory role. The purpose of this review is to summarize how miR-155 is regulated and the pathological consequences of its deregulation during neuroinflammatory disorders, including multiple sclerosis, Alzheimer's disease and other neuroinflammatory disorders. Modulation of miRNAs' expression could be used as a therapeutic strategy in the treatment of pathological neuroinflammation.

C1 [Zingale, Valeria Domenica; Gugliandolo, Agnese; Mazzon, Emanuela] IRCCS Ctr Neurol Bonino Pulejo, Via Prov Palermo, I-98124 Messina, Italy.

C3 IRCCS Bonino Pulejo

RP Gugliandolo, A (corresponding author), IRCCS Ctr Neurol Bonino Pulejo, Via Prov Palermo, I-98124 Messina, Italy.

EM valeria.zingale@irccsme.it; agnese.gugliandolo@irccsme.it;

emanuela.mazzon@irccsme.it

RI Gugliandolo, Agnese/neurodegenerative disease-5466-2019

OI Gugliandolo, Agnese/0000-0002-7868-3353; mazzon,

emanuela/0000-0002-5073-717X

CR Aboulhoda BE, 2021, J CELL PHYSIOL, V236, P5994, DOI 10.1002/jcp.30283

Alexandrov PN, 2019, FOLIA NEUROPATHOL, V57, P211, DOI 10.5114/fn.2019.88449

Alexandrov Peter N, 2012, Int J Biochem Mol Biol, V3, P365

Aloi MS, 2021, GLIA, V69, P1736, DOI 10.1002/glia.23988

Anastasi F, 2021, INT J MOL SCI, V22, DOI 10.3390/ijms22062951

Appel SH, 2012, MOVEMENT DISORD, V27, P1075, DOI 10.1002/mds.25111

Azouz F, 2019, VIRUSES-BASEL, V11, DOI 10.3390/v11020162

Barbato C, 2008, MAMM GENOME, V19, P541, DOI 10.1007/s00335-008-9129-6

Becher B, 2017, NAT REV IMMUNOL, V17, P49, DOI 10.1038/nri.2016.123

Benilova I, 2012, NAT NEUROSCI, V15, P349, DOI 10.1038/nn.3028

Bhela S, 2014, J IMMUNOL, V192, P2734, DOI 10.4049/jimmunol.1302326

Biber K, 2007, TRENDS NEUROSCI, V30, P596, DOI 10.1016/j.tins.2007.08.007

Blennow K, 2016, NAT REV DIS PRIMERS, V2, DOI 10.1038/nrdp.2016.84

Boillee S, 2006, SCIENCE, V312, P1389, DOI 10.1126/science.1123511

Butovsky O, 2015, ANN NEUROL, V77, P75, DOI 10.1002/ana.24304

Caballero-Garrido E, 2015, J NEUROSCI, V35, P12446, DOI 10.1523/JNEUROSCI.1641-15.2015

Caggiu Elisa, 2018, eNeurologicalSci, V13, P1, DOI 10.1016/j.ensci.2018.09.002

CALNE DB, 1993, NEW ENGL J MED, V329, P1021

Cardoso AL, 2012, IMMUNOLOGY, V135, P73, DOI 10.1111/j.1365-2567.2011.03514.x

Pena-Philippides JC, 2016, J NEUROINFLAMM, V13, DOI 10.1186/s12974-016-0753-x

Cekanaviciute E, 2016, NEUROTHERAPEUTICS, V13, P685, DOI 10.1007/s13311-016-0477-8

Chakraborty C, 2021, J ADV RES, V28, P127, DOI 10.1016/j.jare.2020.08.012

Cheng XR, 2013, FRONT AGING NEUROSCI, V5, DOI 10.3389/fnagi.2013.00065

Dickey LL, 2017, J NEUROIMMUNOL, V308, P17, DOI 10.1016/j.jneuroim.2017.01.016

Dickey LL, 2016, J NEUROINFLAMM, V13, DOI 10.1186/s12974-016-0699-z

DiSabato DJ, 2016, J NEUROCHEM, V139, P136, DOI 10.1111/jnc.13607

Eis PS, 2005, P NATL ACAD SCI USA, V102, P3627, DOI 10.1073/pnas.0500613102

Elton TS, 2013, GENE, V532, P1, DOI 10.1016/j.gene.2012.12.009

Ertekin-Taner N, 2007, NEUROL CLIN, V25, P611, DOI 10.1016/j.ncl.2007.03.009

Esteller M, 2011, NAT REV GENET, V12, P861, DOI 10.1038/nrg3074

Filipowicz W, 2008, NAT REV GENET, V9, P102, DOI 10.1038/nrg2290

Freilich RW, 2013, PLOS ONE, V8, DOI 10.1371/journal.pone.0079416

Gaudet AD, 2018, NEUROSCIENTIST, V24, P221, DOI 10.1177/1073858417721150

Giuliani A, 2021, MULT SCLER RELAT DIS, V54, DOI 10.1016/j.msard.2021.103126

Gorski SA, 2017, NAT REV MOL CELL BIO, V18, P215, DOI 10.1038/nrm.2016.174

Guedes J, 2013, CLIN DEV IMMUNOL, DOI 10.1155/2013/186872

Guedes Joana R, 2016, Alzheimers Dement (Amst), V3, P7, DOI 10.1016/j.dadm.2015.11.004

Guedes JR, 2014, HUM MOL GENET, V23, P6286, DOI 10.1093/hmg/ddu348

Ha M, 2014, NAT REV MOL CELL BIO, V15, P509, DOI 10.1038/nrm3838

Harmon JL, 2017, J NEUROTRAUM, V34, P487, DOI 10.1089/neu.2015.4395

Harrison EB, 2017, FRONT MOL NEUROSCI, V10, DOI 10.3389/fnmol.2017.00228

He L, 2004, NAT REV GENET, V5, P522, DOI 10.1038/nrg1379

Henry RJ, 2019, NEUROTHERAPEUTICS, V16, P216, DOI 10.1007/s13311-018-0665-9

Hu HX, 2021, EVID-BASED COMPL ALT, V2021, DOI 10.1155/2021/2549076

Hu RZ, 2013, J IMMUNOL, V190, P5972, DOI 10.4049/jimmunol.1300351

Iadecola C, 2017, NEURON, V96, P17, DOI 10.1016/j.neuron.2017.07.030

Jablonski KA, 2016, PLOS ONE, V11, DOI 10.1371/journal.pone.0159724

Jankovic J, 2020, J NEUROL NEUROSUR PS, V91, P795, DOI 10.1136/jnnp-2019-322338

Jha NK, 2019, J NEUROCHEM, V150, P113, DOI 10.1111/jnc.14687

Johnston M, 2010, MOL BIOL CELL, V21, P1462, DOI 10.1091/mbc.E09-10-0885

Junker A, 2009, BRAIN, V132, P3342, DOI 10.1093/brain/awp300

Kabba JA, 2018, CELL MOL NEUROBIOL, V38, P53, DOI 10.1007/s10571-017-0504-2

Kamphuis WW, 2015, CNS NEUROL DISORD-DR, V14, P157, DOI 10.2174/1871527314666150116125246

Kasper LH, 2010, NEUROLOGY, V74, pS2, DOI 10.1212/WNL.0b013e3181c97c8f

Kempuraj D., 2016, J NEUROLOGY NEUROSUR, V1

Khan I, 2022, CELL MOL NEUROBIOL, V42, P2075, DOI 10.1007/s10571-021-01093-4

Kim Jong-Hyeon, 2014, Exp Neurobiol, V23, P148, DOI 10.5607/en.2014.23.2.148

Klein RS, 2017, NAT IMMUNOL, V18, P132, DOI 10.1038/ni.3656

Koval ED, 2013, HUM MOL GENET, V22, P4127, DOI 10.1093/hmg/ddt261

Kumar A, 2017, J NEUROINFLAMM, V14, DOI 10.1186/s12974-017-0819-4

Lagos-Quintana M, 2001, SCIENCE, V294, P853, DOI 10.1126/science.1064921

Lakhan SE, 2009, J TRANSL MED, V7, DOI 10.1186/1479-5876-7-97

LEE RC, 1993, CELL, V75, P843, DOI 10.1016/0092-8674(93)90529-Y

Li ZZ, 2017, AM J TRANSL RES, V9, P5653

Liang YY, 2021, FRONT CELL NEUROSCI, V15, DOI 10.3389/fncel.2021.785433

Lippai D, 2013, PLOS ONE, V8, DOI 10.1371/journal.pone.0070945

Liu DD, 2019, CURR ALZHEIMER RES, V16, P473, DOI 10.2174/1567205016666190503145207

Lopez-Ramirez MA, 2014, FASEB J, V28, P2551, DOI 10.1096/fj.13-248880

Lu LF, 2015, IMMUNITY, V43, P52, DOI 10.1016/j.immuni.2015.04.022

Lukiw WJ, 2012, NEUROREPORT, V23, P621, DOI [10.1097/00001756-201207110-00009, 10.1097/WNR.0b013e32835542b0]

Lund C, 2014, ACTA NEUROL SCAND, V130, P368, DOI 10.1111/ane.12276

Luo DL, 2020, MULT SCLER RELAT DIS, V41, DOI 10.1016/j.msard.2020.102044

Lv RX, 2020, ACS CHEM NEUROSCI, V11, P3259, DOI 10.1021/acschemneuro.0c00375

Lyman M, 2014, NEUROSCI RES, V79, P1, DOI 10.1016/j.neures.2013.10.004

Ma FY, 2017, PHARM BIOL, V55, P1263, DOI 10.1080/13880209.2017.1297838

Macrez R, 2011, LANCET NEUROL, V10, P471, DOI 10.1016/S1474-4422(11)70066-7

Mahesh G, 2019, J INTERF CYTOK RES, V39, P321, DOI 10.1089/jir.2018.0155

Majer A, 2017, PLOS ONE, V12, DOI 10.1371/journal.pone.0169081

Mameli G, 2016, PLOS ONE, V11, DOI 10.1371/journal.pone.0157153

Martinez-Nunez RT, 2011, J BIOL CHEM, V286, P1786, DOI 10.1074/jbc.M110.169367

McCoy CE, 2017, ADV EXP MED BIOL, V1024, P111, DOI 10.1007/978-981-10-5987-2_5

Michell-Robinson MA, 2016, ANN CLIN TRANSL NEUR, V3, P27, DOI 10.1002/acn3.270

Miller E, 2012, ADV EXP MED BIOL, V724, P222, DOI 10.1007/978-1-4614-0653-2_17

Moore CS, 2013, ANN NEUROL, V74, P709, DOI 10.1002/ana.23967

Murugaiyan G, 2011, J IMMUNOL, V187, P2213, DOI 10.4049/jimmunol.1003952

Mycko MP, 2012, P NATL ACAD SCI USA, V109, pE1248, DOI 10.1073/pnas.1114325109

Nagai M, 2007, NAT NEUROSCI, V10, P615, DOI 10.1038/nn1876

Natekar JP, 2020, VIRUSES-BASEL, V12, DOI 10.3390/v12010009

Nayak D, 2014, ANNU REV IMMUNOL, V32, P367, DOI 10.1146/annurev-immunol-032713-120240

O'Connell RM, 2007, P NATL ACAD SCI USA, V104, P1604, DOI 10.1073/pnas.0610731104

O'Connell RM, 2009, P NATL ACAD SCI USA, V106, P7113, DOI 10.1073/pnas.0902636106

Okun E, 2009, BRAIN RES REV, V59, P278, DOI 10.1016/j.brainresrev.2008.09.001

Paez-Colasante X, 2015, NAT REV NEUROL, V11, P266, DOI 10.1038/nrneurol.2015.57

Pan PF, 2019, TRANSPL P, V51, P3474, DOI 10.1016/j.transproceed.2019.08.042

Paraboschi EM, 2011, INT J MOL SCI, V12, P8695, DOI 10.3390/ijms12128695

Pareek S, 2014, J NEUROINFLAMM, V11, DOI 10.1186/1742-2094-11-97

Pilakka-Kanthikeel S, 2015, J NEUROINFLAMM, V12, DOI 10.1186/s12974-015-0285-9

Prajapati P, 2023, CELL MOL NEUROBIOL, V43, P423, DOI 10.1007/s10571-021-01164-6

Raikwar SP, 2021, CELL MOL NEUROBIOL, V41, P1687, DOI 10.1007/s10571-020-00937-9

Sadik NA, 2021, INT J GEN MED, V14, P1469, DOI 10.2147/IJGM.S295939

Saito Takashi, 2018, Clin Exp Neuroimmunol, V9, P211, DOI 10.1111/cen3.12475

Shabab T, 2017, INT J NEUROSCI, V127, P624, DOI 10.1080/00207454.2016.1212854

Sierksma A, 2018, MOL NEURODEGENER, V13, DOI 10.1186/s13024-018-0285-1

Singh J, 2016, J NEUROIMMUNE PHARM, V11, P84, DOI 10.1007/s11481-015-9630-0

Slota JA, 2019, NON-CODING RNA, V5, DOI 10.3390/ncrna5020035

Sofroniew MV, 2015, NAT REV NEUROSCI, V16, P249, DOI 10.1038/nrn3898

Song J, 2015, FRONT AGING NEUROSCI, V7, DOI 10.3389/fnagi.2015.00061

Su W, 2016, BRAIN BEHAV IMMUN, V52, P1, DOI 10.1016/j.bbi.2015.07.003

Su W, 2014, J IMMUNOL, V192, P358, DOI 10.4049/jimmunol.1301397

Tam W, 2001, GENE, V274, P157, DOI 10.1016/S0378-1119(01)00612-6

Tang Y, 2016, MOL NEUROBIOL, V53, P1181, DOI 10.1007/s12035-014-9070-5

Teter B, 2019, NEUROBIOL DIS, V127, P432, DOI 10.1016/j.nbd.2019.02.015

Thai TH, 2007, SCIENCE, V316, P604, DOI 10.1126/science.1141229

Thome AD, 2016, J NEUROSCI, V36, P2383, DOI 10.1523/JNEUROSCI.3900-15.2016

Thounaojam MC, 2013, MOL NEUROBIOL, V47, P1034, DOI 10.1007/s12035-013-8400-3

Thounaojam MC, 2014, J VIROL, V88, P4798, DOI 10.1128/JVI.02979-13

Tohidpour A, 2017, FRONT CELL INFECT MI, V7, DOI 10.3389/fcimb.2017.00276

Trejo-Lopez JA, 2022, NEUROTHERAPEUTICS, V19, P173, DOI 10.1007/s13311-021-01146-y

Tufekci KU, 2011, AUTOIMMUN DIS, V2011, DOI 10.4061/2011/807426

Varma-Doyle AV, 2021, J NEUROL SCI, V420, DOI 10.1016/j.jns.2020.117202

Vasile F, 2017, BRAIN STRUCT FUNCT, V222, P2017, DOI 10.1007/s00429-017-1383-5

Wang WX, 2021, NEURAL REGEN RES, V16, P514, DOI 10.4103/1673-5374.293149

Wang WX, 2015, EXP NEUROL, V265, P84, DOI 10.1016/j.expneurol.2014.12.018

Wen Y, 2015, MOL MED, V21, P197, DOI 10.2119/molmed.2014.00199

Winter J, 2009, NAT CELL BIOL, V11, P228, DOI 10.1038/ncb0309-228

Yang JB, 2021, ANN PALLIAT MED, V10, P1370, DOI 10.21037/apm-20-518

Zeng Y, 2004, NUCLEIC ACIDS RES, V32, P4776, DOI 10.1093/nar/gkh824

Zhang BH, 2007, J CELL PHYSIOL, V210, P279, DOI 10.1002/jcp.20869

Zhang HT, 2020, J NEUROSCI RES, V98, P2290, DOI 10.1002/jnr.24696

Zhang J, 2014, J NEUROIMMUNOL, V266, P56, DOI 10.1016/j.jneuroim.2013.09.019

Zheng XX, 2018, INFLAMMATION, V41, P11, DOI 10.1007/s10753-017-0658-6

NR 131

TC 58

Z9 61

U1 4

U2 42

PU MDPI

PI BASEL

PA ST ALBAN-ANLAGE 66, CH-4052 BASEL, SWITZERLAND

SN 1661-6596

EI 1422-0067

J9 INT J MOL SCI

JI Int. J. Mol. Sci.

PD JAN

PY 2022

VL 23

IS 1

AR 90

DI 10.3390/ijms23010090

PG 24

WC Biochemistry & Molecular Biology; Chemistry, Multidisciplinary

WE Science Citation Index Expanded (SCI-EXPANDED)

SC Biochemistry & Molecular Biology; Chemistry

GA YV3PM

UT WOS:000752643100001

PM 35008513

OA gold, Green Published

DA 2024-08-12

ER

PT J

AU Xiromerisiou, G

Marogianni, C

Androutsopoulou, A

Ntavaroukas, P

Mysiris, D

Papoutsopoulou, S

AF Xiromerisiou, Georgia

Marogianni, Chrysoula

Androutsopoulou, Anastasia

Ntavaroukas, Panagiotis

Mysiris, Dimitrios

Papoutsopoulou, Stamatia

TI Parkinson's Disease, It Takes Guts: The Correlation between Intestinal

Microbiome and Cytokine Network with Neurodegeneration

SO BIOLOGY-BASEL

LA English

DT Review

DE Parkinson's disease; cytokines; chemokines; gut; microbiota

ID CHAIN FATTY-ACIDS; ALPHA-SYNUCLEIN; BRAIN AXIS; MODEL;

NEUROINFLAMMATION; INFLAMMATION; FEATURES; INNATE

AB Simple Summary Parkinson's disease is a neurodegenerative disease of the central nervous system, characterized by movement problems and accompanied by behavioral changes such as depression and anxiety. It is a multifactorial condition that is affected by genetic alterations and environmental factors that progressively lead to the death of specialized neurons. This systematic review discusses the attractive hypothesis that gut intestinal dysbiosis is an initial step of a process that leads to Parkinson's. Gut microbiota alterations and their metabolites can cause intestinal inflammation, but they can also alter gut-brain communication and the brain barrier. This can lead to brain inflammation and the deterioration of brain cells, a process called neurodegeneration. Understanding the role of gut microbiota in the progression of Parkinson's could be a key element for research toward therapeutic approaches that could delay and even cure the disease. Parkinson's disease is a progressive neurodegenerative disorder with motor, physical and behavioral symptoms that can have a profound impact on the patient's quality of life. Most cases are idiopathic, and the exact mechanism of the disease's cause is unknown. The current hypothesis focuses on the gut-brain axis and states that gut microbiota dysbiosis can trigger inflammation and advances the development of Parkinson's disease. This systematic review presents the current knowledge of gut microbiota analysis and inflammation based on selected studies on Parkinson's patients and experimental animal models. Changes in gut microbiota correlate with Parkinson's disease, but only a few studies have considered inflammatory modulators as important triggers of the disease. Nevertheless, it is evident that proinflammatory cytokines and chemokines are induced in the gut, the circulation, and the brain before the development of the disease's neurological symptoms and exacerbate the disease. Increased levels of tumor necrosis factor, interleukin-1 beta, interleukin-6, interleukin-17A and interferon-gamma can correlate with altered gut microbiota. Instead, treatment of gut dysbiosis is accompanied by reduced levels of inflammatory mediators in specific tissues, such as the colon, brain and serum and/or cerebrospinal fluid. Deciphering the role of the immune responses and the mechanisms of the PD-associated gut microbiota will assist the interpretation of the pathogenesis of Parkinson's and will elucidate appropriate therapeutic strategies.

C1 [Xiromerisiou, Georgia; Marogianni, Chrysoula; Mysiris, Dimitrios] Univ Thessaly, Fac Life Sci, Dept Med, Larisa 41500, Greece.

[Androutsopoulou, Anastasia; Ntavaroukas, Panagiotis; Papoutsopoulou, Stamatia] Univ Thessaly, Fac Life Sci, Dept Biochem & Biotechnol, Larisa 41500, Greece.

C3 University of Thessaly; University of Thessaly

RP Papoutsopoulou, S (corresponding author), Univ Thessaly, Fac Life Sci, Dept Biochem & Biotechnol, Larisa 41500, Greece.

EM stapapou@uth.gr

RI Papoutsopoulou, Stamatia/HTR-2118-2023

OI Papoutsopoulou, Stamatia/0000-0001-6665-8508; Mysiris,

Dimitrios/0000-0001-7743-995X; Marogianni, Chrysoula/0000-0002-5979-9916

CR Aardoom MA, 2019, INT J MOL SCI, V20, DOI 10.3390/ijms20102529

Aho VTE, 2021, MOL NEURODEGENER, V16, DOI 10.1186/s13024-021-00427-6

Alshehri D, 2021, BOSNIAN J BASIC MED, V21, P270, DOI 10.17305/bjbms.2020.5016

Armstrong MJ, 2020, JAMA-J AM MED ASSOC, V323, P548, DOI 10.1001/jama.2019.22360

Barichella M, 2019, MOVEMENT DISORD, V34, P396, DOI 10.1002/mds.27581

Barnhart MM, 2006, ANNU REV MICROBIOL, V60, P131, DOI 10.1146/annurev.micro.60.080805.142106

Bedarf JR, 2017, GENOME MED, V9, DOI 10.1186/s13073-017-0428-y

Berlamont H, 2021, INT J MOL SCI, V22, DOI 10.3390/ijms222111328

Bessler H, 1999, BIOMED PHARMACOTHER, V53, P141, DOI 10.1016/S0753-3322(99)80079-1

Betarbet R, 2000, NAT NEUROSCI, V3, P1301, DOI 10.1038/81834

Bhattacharyya D, 2019, ACS CHEM NEUROSCI, V10, P2229, DOI 10.1021/acschemneuro.8b00733

Boertien JM, 2019, J PARKINSON DIS, V9, pS297, DOI 10.3233/JPD-191711

Braak H, 2003, J NEURAL TRANSM, V110, P517, DOI 10.1007/s00702-002-0808-2

Brown GC, 2019, J NEUROINFLAMM, V16, DOI 10.1186/s12974-019-1564-7

Bullich C, 2019, MOV DISORD CLIN PRAC, V6, P639, DOI 10.1002/mdc3.12840

Bussell R, 2003, J MOL BIOL, V329, P763, DOI 10.1016/S0022-2836(03)00520-5

Castillo-Alvarez F, 2022, NEUROLOGIA, V37, P492, DOI [10.1016/j.nrl.2019.03.017, 10.1016/j.nrleng.2019.03.026]

Chen H, 2008, AM J EPIDEMIOL, V167, P90, DOI 10.1093/aje/kwm260

Chen QQ, 2019, FRONT MOL NEUROSCI, V12, DOI 10.3389/fnmol.2019.00218

Chen SG, 2016, SCI REP-UK, V6, DOI 10.1038/srep34477

Chen SJ, 2022, NEUROLOGY, V98, pE848, DOI 10.1212/WNL.0000000000013225

Chen X, 2018, FRONT IMMUNOL, V9, DOI 10.3389/fimmu.2018.02122

Cheng L, 2020, NUCLEIC ACIDS RES, V48, pD554, DOI 10.1093/nar/gkz843

Cherny I, 2005, J MOL BIOL, V352, P245, DOI 10.1016/j.jmb.2005.07.028

Choi JG, 2018, SCI REP-UK, V8, DOI 10.1038/s41598-018-19646-x

Chorell E, 2015, PLOS ONE, V10, DOI 10.1371/journal.pone.0140194

Cui C, 2023, J NEUROIMMUNE PHARM, V18, P72, DOI 10.1007/s11481-021-10047-y

Dalile B, 2019, NAT REV GASTRO HEPAT, V16, P461, DOI 10.1038/s41575-019-0157-3

Danau A, 2021, INT J MOL SCI, V22, DOI 10.3390/ijms222111663

Deng H, 2018, AGEING RES REV, V42, P72, DOI 10.1016/j.arr.2017.12.007

Devos D, 2013, NEUROBIOL DIS, V50, P42, DOI 10.1016/j.nbd.2012.09.007

Domingo Aloysius, 2018, Handb Clin Neurol, V147, P211, DOI 10.1016/B978-0-444-63233-3.00014-2

Dong SY, 2022, BRAIN RES BULL, V183, P84, DOI 10.1016/j.brainresbull.2022.02.015

Dong XL, 2020, INT J BIOL MACROMOL, V164, P994, DOI 10.1016/j.ijbiomac.2020.07.180

Dorsey ER, 2007, NEUROLOGY, V68, P384, DOI 10.1212/01.wnl.0000247740.47667.03

Evans ML, 2015, MOL CELL, V57, P445, DOI 10.1016/j.molcel.2014.12.025

Fang X, 2019, J NEUROCHEM, V150, P441, DOI 10.1111/jnc.14694

Feng YK, 2020, J NEUROINFLAMM, V17, DOI 10.1186/s12974-020-02027-5

Fitzgerald E, 2019, FRONT NEUROSCI-SWITZ, V13, DOI 10.3389/fnins.2019.00369

Forsyth CB, 2011, PLOS ONE, V6, DOI 10.1371/journal.pone.0028032

Ghaisas S, 2019, NEUROTOXICOLOGY, V75, P186, DOI 10.1016/j.neuro.2019.09.004

Ghyselinck J, 2021, INT J PHARM-X, V3, DOI 10.1016/j.ijpx.2021.100087

Gorecki AM, 2020, FRONT AGING NEUROSCI, V12, DOI 10.3389/fnagi.2020.603849

Hasegawa S, 2015, PLOS ONE, V10, DOI 10.1371/journal.pone.0142164

Hawkes CH, 2007, NEUROPATH APPL NEURO, V33, P599, DOI 10.1111/j.1365-2990.2007.00874.x

Hooper LV, 2012, SCIENCE, V336, P1268, DOI 10.1126/science.1223490

Houser MC, 2018, MOVEMENT DISORD, V33, P793, DOI 10.1002/mds.27326

Jackson A, 2022, FRONT NEUROL, V13, DOI 10.3389/fneur.2022.882628

Jankovic J, 2008, J NEUROL NEUROSUR PS, V79, P368, DOI 10.1136/jnnp.2007.131045

Jankovic J, 2020, J NEUROL NEUROSUR PS, V91, P795, DOI 10.1136/jnnp-2019-322338

Joers V, 2020, NEUROBIOL DIS, V144, DOI 10.1016/j.nbd.2020.105027

Kannarkat GT, 2013, J PARKINSON DIS, V3, P493, DOI 10.3233/JPD-130250

Karunaratne TB, 2021, NUTRIENTS, V13, DOI 10.3390/nu13010028

Killinger BA, 2018, SCI TRANSL MED, V10, DOI 10.1126/scitranslmed.aar5280

Kim C, 2016, SCI REP-UK, V6, DOI 10.1038/srep30891

Kim S, 2019, NEURON, V103, P627, DOI 10.1016/j.neuron.2019.05.035

Klann EM, 2022, FRONT AGING NEUROSCI, V13, DOI 10.3389/fnagi.2021.782082

Klein C, 2012, CSH PERSPECT MED, V2, DOI 10.1101/cshperspect.a008888

Koutzoumis DN, 2020, EXP NEUROL, V325, DOI 10.1016/j.expneurol.2019.113159

Lai F, 2018, NEUROCHEM RES, V43, P1986, DOI 10.1007/s11064-018-2620-x

LANGSTON JW, 1984, ACTA NEUROL SCAND, V70, P49

Lee HS, 2021, GUT, V70, P408, DOI 10.1136/gutjnl-2020-322429

Lee JY, 2019, STEM CELL REV REP, V15, P690, DOI 10.1007/s12015-019-09906-2

Lee JY, 2019, J CELL MOL MED, V23, P5466, DOI 10.1111/jcmm.14429

Li JJ, 2022, CONTRAST MEDIA MOL I, V2022, DOI 10.1155/2022/8381870

Li PP, 2021, METABOLITES, V11, DOI 10.3390/metabo11010029

Li Z, 2022, NEUROSCIENCE, V480, P65, DOI 10.1016/j.neuroscience.2021.10.011

Liao JF, 2020, BRAIN BEHAV IMMUN, V90, P26, DOI 10.1016/j.bbi.2020.07.036

Lin CH, 2019, J NEUROINFLAMM, V16, DOI 10.1186/s12974-019-1528-y

Liu XR, 2021, NEUROCHEM RES, V46, P1514, DOI 10.1007/s11064-021-03293-2

Liu ZH, 2022, FRONT NUTR, V9, DOI 10.3389/fnut.2022.836500

Ma QL, 2013, BEHAV BRAIN FUNCT, V9, DOI 10.1186/1744-9081-9-19

MENDEZ JS, 1975, J NEUROSURG, V42, P166, DOI 10.3171/jns.1975.42.2.0166

Nagatsu T, 2000, ADV RES NEURODEGENER, V8, P277

Neurath MF, 2019, NAT IMMUNOL, V20, P970, DOI 10.1038/s41590-019-0415-0

Nuzum ND, 2020, NEUROSCI BIOBEHAV R, V112, P227, DOI 10.1016/j.neubiorev.2020.02.003

O'Donovan SM, 2020, NEUROGASTROENT MOTIL, V32, DOI 10.1111/nmo.13726

Page MJ, 2021, INT J SURG, V88, DOI [10.1186/s13643-021-01626-4, 10.1016/j.ijsu.2021.105906, 10.1016/j.jclinepi.2021.02.003]

Pal GD, 2015, FRONT NEUROSCI-SWITZ, V9, DOI 10.3389/fnins.2015.00306

Perez-Pardo P, 2019, GUT, V68, P829, DOI 10.1136/gutjnl-2018-316844

Qin LY, 2007, GLIA, V55, P453, DOI 10.1002/glia.20467

Qin XY, 2016, JAMA NEUROL, V73, P1316, DOI 10.1001/jamaneurol.2016.2742

Rocha JDB, 2015, NAT IMMUNOL, V16, P898, DOI 10.1038/ni.3255

Romano S, 2021, NPJ PARKINSONS DIS, V7, DOI 10.1038/s41531-021-00156-z

Sampson TR, 2016, CELL, V167, P1469, DOI 10.1016/j.cell.2016.11.018

Sandhu KV, 2017, TRANSL RES, V179, P223, DOI 10.1016/j.trsl.2016.10.002

Schapira AHV, 2017, NAT REV NEUROSCI, V18, P509, DOI 10.1038/nrn.2017.91

Scheperjans F, 2015, MOVEMENT DISORD, V30, P350, DOI 10.1002/mds.26069

Schonhoff AM, 2020, PROG BRAIN RES, V252, P169, DOI 10.1016/bs.pbr.2019.10.006

Singh Y, 2019, FRONT NEUROSCI-SWITZ, V13, DOI 10.3389/fnins.2019.01032

Smeland OB, 2021, BIOL PSYCHIAT, V89, P227, DOI 10.1016/j.biopsych.2020.01.026

Sommer F, 2013, NAT REV MICROBIOL, V11, P227, DOI 10.1038/nrmicro2974

Stokholm MG, 2016, ANN NEUROL, V79, P940, DOI 10.1002/ana.24648

Sun MF, 2018, BRAIN BEHAV IMMUN, V70, P48, DOI 10.1016/j.bbi.2018.02.005

Sun MF, 2018, AGEING RES REV, V45, P53, DOI 10.1016/j.arr.2018.04.004

Sun ZQ, 2022, FRONT CELL DEV BIOL, V9, DOI 10.3389/fcell.2021.808905

Sveinbjornsdottir S, 2016, J NEUROCHEM, V139, P318, DOI 10.1111/jnc.13691

Tan J, 2014, ADV IMMUNOL, V121, P91, DOI 10.1016/B978-0-12-800100-4.00003-9

Tsao SP, 2021, ANTIOXIDANTS-BASEL, V10, DOI 10.3390/antiox10111823

Unger MM, 2016, PARKINSONISM RELAT D, V32, P66, DOI 10.1016/j.parkreldis.2016.08.019

Van den Abbeele P, 2010, APPL ENVIRON MICROB, V76, P5237, DOI 10.1128/AEM.00759-10

Voigt RM, 2022, NEUROBIOL DIS, V170, DOI 10.1016/j.nbd.2022.105780

Wang L, 2022, J AGR FOOD CHEM, V70, P1163, DOI 10.1021/acs.jafc.1c07711

Wang N, 2022, FOOD FUNCT, V13, P2019, DOI [10.1039/D1FO02216D, 10.1039/d1fo02216d]

Xie WJ, 2020, NEUROTOXICOLOGY, V76, P200, DOI 10.1016/j.neuro.2019.11.009

Yamada-Fowler Naomi, 2015, Journal of Caffeine Research, V5, P3, DOI 10.1089/jcr.2014.0028

Yang DM, 2020, NEUROBIOL DIS, V135, DOI 10.1016/j.nbd.2019.104704

Yang DM, 2019, FRONT NEUROL, V10, DOI 10.3389/fneur.2019.01155

Yang XD, 2018, FRONT AGING NEUROSCI, V9, DOI 10.3389/fnagi.2017.00441

Yao J, 2022, CLIN IMMUNOL, V238, DOI 10.1016/j.clim.2022.109013

Zeng JQ, 2022, FRONT AGING NEUROSCI, V14, DOI 10.3389/fnagi.2022.955919

Zhang T, 2022, EXP THER MED, V23, DOI 10.3892/etm.2021.10974

Zhao Z, 2021, MICROBIOME, V9, DOI 10.1186/s40168-021-01107-9

Zhao Z, 2021, ACTA PHARM SIN B, V11, P2859, DOI 10.1016/j.apsb.2021.03.020

Zhong Z, 2021, NEUROCHEM RES, V46, P3050, DOI 10.1007/s11064-021-03411-0

Zhou XT, 2021, OXID MED CELL LONGEV, V2021, DOI 10.1155/2021/9424582

Zhou ZL, 2019, NEUROTHERAPEUTICS, V16, P741, DOI 10.1007/s13311-019-00719-2

Zhu ML, 2022, FRONT IMMUNOL, V13, DOI 10.3389/fimmu.2022.937555

NR 118

TC 2

Z9 3

U1 6

U2 16

PU MDPI

PI BASEL

PA ST ALBAN-ANLAGE 66, CH-4052 BASEL, SWITZERLAND

EI 2079-7737

J9 BIOLOGY-BASEL

JI Biology-Basel

PD JAN

PY 2023

VL 12

IS 1

AR 93

DI 10.3390/biology12010093

PG 13

WC Biology

WE Science Citation Index Expanded (SCI-EXPANDED)

SC Life Sciences & Biomedicine - Other Topics

GA 7X9DU

UT WOS:000914494400001

PM 36671785

OA Green Published, gold

DA 2024-08-12

ER

PT J

AU Shi, YJ

Wei, B

Li, LJ

Wang, B

Sun, M

AF Shi, Yajun

Wei, Bin

Li, Lingjun

Wang, Bin

Sun, Miao

TI Th17 cells and inflammation in neurological disorders: Possible

mechanisms of action

SO FRONTIERS IN IMMUNOLOGY

LA English

DT Review

DE Th17 cells; neurological disorders; IL-17A; neuroinflammation; immune

system; microglia

ID ROR-GAMMA-T; SUPPRESSES MICROGLIAL ACTIVITY; PATHOGENIC T(H)17 CELLS;

BLOOD-BRAIN-BARRIER; alzheimer's disease; MULTIPLE-SCLEROSIS;

AMYLOID-BETA; SERUM-LEVELS; DIFFERENTIATION; GENERATION

AB Neurological disorders (NDs) are one of the leading causes of global death. A sustained neuroinflammatory response has been reported to be associated with the pathogenesis of multiple NDs, including Parkinson's disease (PD), multiple sclerosis (MS), Alzheimer's disease (AD), amyotrophic lateral sclerosis (ALS), and major depressive disorder (MDD). Accumulating evidence shows that the recruitment of abundant lymphocytes in the central nervous system may contribute to promoting the development and progress of inflammation in neurological disorders. As one subset of T lymphocytes, CD4(+) T cells have a critical impact on the inflammation of neurological disorders. T helper (Th) 17 is one of the most studied CD4(+) Th subpopulations that produces cytokines (e.g., IL-17A, IL-23, IL-21, IL-6, and IFN-gamma), leading to the abnormal neuroinflammatory response including the excessive activation of microglia and the recruitment of other immune cell types. All these factors are involved in several neurological disorders. However, the possible mechanisms of Th17 cells and their associated cytokines in the immunopathology of the abovementioned neurological disorders have not been clarified completely. This review will summarize the mechanisms by which encephalitogenic inflammatory Th17 cells and their related cytokines strongly contribute to chronic neuroinflammation, thus perpetuating neurodegenerative processes in NDs. Finally, the potential therapeutic prospects of Th17 cells and their cytokines in NDs will also be discussed.

C1 [Shi, Yajun; Wei, Bin; Li, Lingjun; Wang, Bin; Sun, Miao] Soochow Univ, Affiliated Hosp 1, Inst Fetol, Suzhou, China.

C3 Soochow University - China

RP Wang, B; Sun, M (corresponding author), Soochow Univ, Affiliated Hosp 1, Inst Fetol, Suzhou, China.

EM binwang2233@suda.edu.cn; miaosunsuda@163.com

RI Shi, Yajun/GPS-8969-2022; li, chunlin/KFS-0761-2024

OI Shi, Yajun/0000-0002-4657-7337; Wang, Bin/0000-0002-6159-5242

FU National Key R&D Program of China [2019YFA0802600]; National Natural

Science Foundation of China [81974244, 81570960]

FX Funding This work was supported by grants from the National Key R&D

Program of China (2019YFA0802600) and the National Natural Science

Foundation of China (81974244 and 81570960).

CR Aboobacker S., 2022, STATPEARLS

Albeltagy ES, 2021, CLIN RHEUMATOL, V40, P895, DOI 10.1007/s10067-020-05326-7

Alvarez-Mon MA, 2021, J PERS MED, V11, DOI 10.3390/jpm11030220

[Anonymous], 2020, ALZHEIMERS DEMENT, V16, P391, DOI 10.1002/alz.12068

[Anonymous], 2019, Lancet Neurol, V18, P459, DOI [10.1016/S1474-4422(18)30499-X, DOI 10.1016/S1474-4422(18)30499-X]

Ben Hmid A, 2020, CYTOKINE, V134, DOI 10.1016/j.cyto.2020.155210

Berry SPDG, 2022, INT IMMUNOPHARMACOL, V102, DOI 10.1016/j.intimp.2021.108402

Bettelli E, 2006, NATURE, V441, P235, DOI 10.1038/nature04753

Beurel E, 2013, BIOL PSYCHIAT, V73, P622, DOI 10.1016/j.biopsych.2012.09.021

Brigas HC, 2021, CELL REP, V36, DOI 10.1016/j.celrep.2021.109574

Broux B, 2020, NEUROL-NEUROIMMUNOL, V7, DOI 10.1212/NXI.0000000000000870

Caputi V, 2018, INT J MOL SCI, V19, DOI 10.3390/ijms19061689

Caza T, 2015, BIOMED RES INT, V2015, DOI 10.1155/2015/521957

Cervellati C, 2020, ANTIOXID REDOX SIGN, V33, P191, DOI 10.1089/ars.2020.8076

Chen H, 2022, NUTRITION, V93, DOI 10.1016/j.nut.2021.111499

Chen JH, 2015, PLOS ONE, V10, DOI 10.1371/journal.pone.0116549

Chen JM, 2014, DEMENT GERIATR COGN, V38, P321, DOI 10.1159/000360606

Chen JJ, 2020, FRONT AGING NEUROSCI, V12, DOI 10.3389/fnagi.2020.566922

Chen XJ, 2018, CELL IMMUNOL, V329, P41, DOI 10.1016/j.cellimm.2018.04.011

Cheng JB, 2021, SCI ADV, V7, DOI 10.1126/sciadv.abe3600

Chong WP, 2020, IMMUNITY, V53, P384, DOI 10.1016/j.immuni.2020.06.022

Cobley JN, 2018, REDOX BIOL, V15, P490, DOI 10.1016/j.redox.2018.01.008

Cortese A, 2019, MULT SCLER RELAT DIS, V35, P193, DOI 10.1016/j.msard.2019.08.006

Davami MH, 2016, BASIC CLIN NEUROSCI, V7, P137, DOI 10.15412/J.BCN.03070207

Dong C, 2021, ANNU REV IMMUNOL, V39, P51, DOI 10.1146/annurev-immunol-061020-053702

Durelli L, 2009, ANN NEUROL, V65, P499, DOI 10.1002/ana.21652

Enevold C, 2022, JAMA DERMATOL, V158, P762, DOI 10.1001/jamadermatol.2022.1863

Faraco G, 2018, NAT NEUROSCI, V21, P240, DOI 10.1038/s41593-017-0059-z

Feng S, 2016, EUR REV MED PHARMACO, V20, P4032

Fung TC, 2017, NAT NEUROSCI, V20, P145, DOI 10.1038/nn.4476

Garofalo S, 2022, BRAIN BEHAV IMMUN, V105, P1, DOI 10.1016/j.bbi.2022.06.004

Gate D, 2021, SCIENCE, V374, P868, DOI 10.1126/science.abf7266

Geng J, 2017, NAT IMMUNOL, V18, P800, DOI 10.1038/ni.3748

Ghoreschi K, 2010, NATURE, V467, P967, DOI 10.1038/nature09447

Ghosh R, 2022, CRIT REV CL LAB SCI, V59, P19, DOI 10.1080/10408363.2021.1965535

Grigg JB, 2021, NATURE, V600, P707, DOI 10.1038/s41586-021-04136-4

Hampel H, 2021, BIOL PSYCHIAT, V89, P745, DOI 10.1016/j.biopsych.2020.02.001

Hartlehnert M, 2021, P NATL ACAD SCI USA, V118, DOI 10.1073/pnas.2023174118

Hassan W, 2022, CURR NEUROPHARMACOL, V20, P1046, DOI 10.2174/1570159X19666211111141246

Hinkle JT, 2022, P NATL ACAD SCI USA, V119, DOI 10.1073/pnas.2118819119

Huffman WJ, 2019, BRAIN STIMUL, V12, P19, DOI 10.1016/j.brs.2018.10.005

Islam SMT, 2021, IMMUNOLOGY, V164, P31, DOI 10.1111/imm.13335

Ivanov II, 2010, MUCOSAL IMMUNOL, V3, P209, DOI 10.1038/mi.2010.3

Ivanov II, 2006, CELL, V126, P1121, DOI 10.1016/j.cell.2006.07.035

Jiang Y, 2018, NAT COMMUN, V9, DOI 10.1038/s41467-018-03852-2

Jin MM, 2021, INT J MOL SCI, V22, DOI 10.3390/ijms22158042

Jin M, 2020, SCI REP-UK, V10, DOI 10.1038/s41598-020-62756-8

Kesika P, 2021, LIFE SCI, V264, DOI 10.1016/j.lfs.2020.118627

Khemka VK, 2014, AGING DIS, V5, P170, DOI 10.14336/AD.2014.0500170

Kim J, 2021, MOL BRAIN, V14, DOI 10.1186/s13041-020-00726-x

Komuczki J, 2019, IMMUNITY, V50, P1289, DOI 10.1016/j.immuni.2019.04.006

Korn T, 2007, NATURE, V448, P484, DOI 10.1038/nature05970

Krebs CF, 2020, SCI IMMUNOL, V5, DOI 10.1126/sciimmunol.aba4163

Kubick N, 2020, PHARMACEUTICS, V12, DOI 10.3390/pharmaceutics12090880

La Vitola P, 2021, NEUROPATH APPL NEURO, V47, P43, DOI 10.1111/nan.12644

Langrish CL, 2005, J EXP MED, V201, P233, DOI 10.1084/jem.20041257

Larochelle C, 2021, P NATL ACAD SCI USA, V118, DOI 10.1073/pnas.2025813118

Leonardi I, 2022, CELL, V185, P831, DOI 10.1016/j.cell.2022.01.017

Li YC, 2022, INT IMMUNOPHARMACOL, V108, DOI 10.1016/j.intimp.2022.108898

Li YF, 2017, MULT SCLER RELAT DIS, V18, P20, DOI 10.1016/j.msard.2017.09.003

Lim HW, 2015, J EXP MED, V212, P607, DOI 10.1084/jem.20132378

Liu WY, 2015, CNS NEUROSCI THER, V21, P568, DOI 10.1111/cns.12405

Liu XY, 2018, FRONT NEUROL, V9, DOI 10.3389/fneur.2018.00008

Liu Z, 2019, BRAIN BEHAV IMMUN, V81, P630, DOI 10.1016/j.bbi.2019.07.026

Lu MY, 2018, FRONT IMMUNOL, V9, DOI 10.3389/fimmu.2018.00415

Macaluso F, 2019, CLIN EXP RHEUMATOL, V37, P1096

Machhi J, 2021, J NEUROINFLAMM, V18, DOI 10.1186/s12974-021-02308-7

Manczak M, 2009, HUM MOL GENET, V18, P3876, DOI 10.1093/hmg/ddp331

Margolis KG, 2021, GASTROENTEROLOGY, V160, P1486, DOI 10.1053/j.gastro.2020.10.066

Marogianni C, 2020, INT J MOL SCI, V21, DOI 10.3390/ijms21228421

Marsh SE, 2016, P NATL ACAD SCI USA, V113, pE1316, DOI 10.1073/pnas.1525466113

Mathew AJ, 2020, RHEUMATOL THER, V7, P287, DOI 10.1007/s40744-020-00207-6

McGinley AM, 2020, IMMUNITY, V52, P342, DOI 10.1016/j.immuni.2020.01.002

Melnikov M, 2020, J NEUROIMMUNE PHARM, V15, P37, DOI 10.1007/s11481-019-09852-3

Monaghan KL, 2021, P NATL ACAD SCI USA, V118, DOI 10.1073/pnas.2116256118

Moser T, 2020, AUTOIMMUN REV, V19, DOI 10.1016/j.autrev.2020.102647

Nadeem A, 2017, CYTOKINE, V97, P14, DOI 10.1016/j.cyto.2017.05.018

Nakamura Y, 2019, J NEUROIMMUNOL, V335, DOI 10.1016/j.jneuroim.2019.577016

Neshan M, 2022, J ALZHEIMERS DIS, V85, P645, DOI 10.3233/JAD-210480

Novikova G, 2021, MOL NEURODEGENER, V16, DOI 10.1186/s13024-021-00449-0

Nurieva R, 2007, NATURE, V448, P480, DOI 10.1038/nature05969

Olsen I, 2016, J ORAL MICROBIOL, V8, DOI 10.3402/jom.v8.33029

Pandiyan P, 2019, FRONT IMMUNOL, V10, DOI 10.3389/fimmu.2019.00426

Park J, 2017, J LEUKOCYTE BIOL, V102, P1093, DOI 10.1189/jlb.1A0417-147RR

Park JC, 2020, BMB REP, V53, P10, DOI 10.5483/BMBRep.2020.53.1.309

Pastwinska J, 2022, INT J MOL SCI, V23, DOI 10.3390/ijms23031906

Picca A, 2021, BIOMOLECULES, V11, DOI 10.3390/biom11101508

Preuss C.V., 2022, Ixekizumab

Prots I, 2019, EXPERT OPIN THER TAR, V23, P309, DOI 10.1080/14728222.2019.1590336

Reddy PH, 2009, J NEUROCHEM, V111, P1514, DOI 10.1111/j.1471-4159.2009.06432.x

Regen T, 2021, ADV IMMUNOL, V149, P25, DOI 10.1016/bs.ai.2021.03.002

Rostami A, 2013, J NEUROL SCI, V333, P76, DOI 10.1016/j.jns.2013.03.002

Salinas TRW, 2021, P NATL ACAD SCI USA, V118, DOI 10.1073/pnas.2105927118

Sampson TR, 2016, CELL, V167, P1469, DOI 10.1016/j.cell.2016.11.018

Saresella M, 2013, CLIN IMMUNOL, V148, P79, DOI 10.1016/j.clim.2013.04.010

Solt Laura A, 2015, Diabetes Manag (Lond), V5, P247

Solt LA, 2011, NATURE, V472, P491, DOI 10.1038/nature10075

Sommer A, 2018, CELL STEM CELL, V23, P123, DOI 10.1016/j.stem.2018.06.015

Speakman EA, 2020, TRENDS IMMUNOL, V41, P61, DOI 10.1016/j.it.2019.11.007

Su RC, 2021, FRONT AGING NEUROSCI, V13, DOI 10.3389/fnagi.2021.769506

Tahmasebinia F, 2017, PROG NEURO-PSYCHOPH, V79, P408, DOI 10.1016/j.pnpbp.2017.07.023

Tian A, 2015, PLOS ONE, V10, DOI 10.1371/journal.pone.0141596

Town T, 2005, NEUROMOL MED, V7, P255, DOI 10.1385/NMM:7:3:255

Uyttenhove C, 2018, EUR J IMMUNOL, V48, P1883, DOI 10.1002/eji.201847580

van Langelaar J, 2018, BRAIN, V141, P1334, DOI 10.1093/brain/awy069

Van Zeller M, 2021, J ALZHEIMERS DIS, V83, P939, DOI 10.3233/JAD-210268

Vaz M, 2020, EUR J PHARMACOL, V887, DOI 10.1016/j.ejphar.2020.173554

Volpe E, 2016, FRONT IMMUNOL, V7, DOI 10.3389/fimmu.2016.00382

Wang TD, 2022, CELL DEATH DIFFER, V29, P1187, DOI 10.1038/s41418-021-00910-z

Wilck N, 2017, NATURE, V551, P585, DOI 10.1038/nature24628

Wouk J, 2021, ARCH VIROL, V166, P733, DOI 10.1007/s00705-021-04959-6

Wu B, 2021, IMMUNITY, V54, P308, DOI 10.1016/j.immuni.2020.12.010

Wu C, 2013, NATURE, V496, P513, DOI 10.1038/nature11984

Wu FJ, 2017, J LEUKOCYTE BIOL, V101, P1119, DOI 10.1189/jlb.3RU0816-352RR

Wu HJ, 2010, IMMUNITY, V32, P815, DOI 10.1016/j.immuni.2010.06.001

Wu ZR, 2022, CHEM SCI, V13, P1060, DOI 10.1039/d1sc05613a

Yan SF, 2022, CELL MOL LIFE SCI, V79, DOI 10.1007/s00018-021-04076-2

Yang LK, 2017, MED SCI MONITOR, V23, P2972, DOI 10.12659/MSM.904240

Yang T, 2020, FOOD FUNCT, V11, P6875, DOI [10.1039/C9FO03019K, 10.1039/c9fo03019k]

Yang XXO, 2007, J BIOL CHEM, V282, P9358, DOI 10.1074/jbc.C600321200

Zenaro E, 2017, NEUROBIOL DIS, V107, P41, DOI 10.1016/j.nbd.2016.07.007

Zenaro E, 2015, NAT MED, V21, P880, DOI 10.1038/nm.3913

Zeng JY, 2022, IRISH J MED SCI, V191, P1855, DOI 10.1007/s11845-021-02749-2

Zhao YL, 2021, AGEING RES REV, V72, DOI 10.1016/j.arr.2021.101504

NR 124

TC 28

Z9 28

U1 2

U2 13

PU FRONTIERS MEDIA SA

PI LAUSANNE

PA AVENUE DU TRIBUNAL FEDERAL 34, LAUSANNE, CH-1015, SWITZERLAND

SN 1664-3224

J9 FRONT IMMUNOL

JI Front. Immunol.

PD JUL 22

PY 2022

VL 13

AR 932152

DI 10.3389/fimmu.2022.932152

PG 12

WC Immunology

WE Science Citation Index Expanded (SCI-EXPANDED)

SC Immunology

GA 3O6YZ

UT WOS:000836981800001

PM 35935951

OA Green Published, gold

DA 2024-08-12

ER

PT J

AU Marc, MA

Jastrzab, R

Mytych, J

AF Marc, Malgorzata Anna

Jastrzab, Rafal

Mytych, Jennifer

TI Does the Gut Microbial Metabolome Really Matter? The Connection between

GUT Metabolome and Neurological Disorders

SO NUTRIENTS

LA English

DT Review

DE microbiome; metabolome; probiotics; neurodegeneration; Parkinson's

disease; Alzheimer's disease; gut microbiota

ID CHAIN FATTY-ACIDS; parkinson's disease; BRAIN AXIS; INTESTINAL

MICROBIOTA; MULTIPLE-SCLEROSIS; COGNITIVE DECLINE; ALPHA-SYNUCLEIN;

DIETARY PATTERNS; T-CELLS; PROBIOTICS

AB Herein we gathered updated knowledge regarding the alterations of gut microbiota (dysbiosis) and its correlation with human neurodegenerative and brain-related diseases, e.g., Alzheimer's and Parkinson's. This review underlines the importance of gut-derived metabolites and gut metabolic status as the main players in gut-brain crosstalk and their implications on the severity of neural conditions. Scientific evidence indicates that the administration of probiotic bacteria exerts beneficial and protective effects as reduced systemic inflammation, neuroinflammation, and inhibited neurodegeneration. The experimental results performed on animals, but also human clinical trials, show the importance of designing a novel microbiota-based probiotic dietary supplementation with the aim to prevent or ease the symptoms of Alzheimer's and Parkinson's diseases or other forms of dementia or neurodegeneration.

C1 [Marc, Malgorzata Anna; Jastrzab, Rafal; Mytych, Jennifer] Olimp Labs Sp Zoo, Res & Dev Ctr, Pustynia 84F, PL-39200 Debica, Poland.

RP Marc, MA (corresponding author), Olimp Labs Sp Zoo, Res & Dev Ctr, Pustynia 84F, PL-39200 Debica, Poland.

EM m.marc@olimp-labs.com

RI Marć, Małgorzata Anna/T-4449-2019

OI Marć, Małgorzata Anna/0000-0002-5183-801X; Jastrzab,

Rafal/0000-0001-8124-3626

FU National Centre for Research and Development (NCBiR, Poland)

[POIR.01.01.01-00-0985/17]

FX This research was funded by The National Centre for Research and

Development (NCBiR, Poland), under the grant number

POIR.01.01.01-00-0985/17.

CR Aarts E, 2017, PLOS ONE, V12, DOI 10.1371/journal.pone.0183509

Adams JB, 2011, BMC GASTROENTEROL, V11, DOI 10.1186/1471-230X-11-22

Ait-Belgnaoui A, 2014, NEUROGASTROENT MOTIL, V26, P510, DOI 10.1111/nmo.12295

Aizawa E, 2016, J AFFECT DISORDERS, V202, P254, DOI 10.1016/j.jad.2016.05.038

Ajslev TA, 2011, INT J OBESITY, V35, P522, DOI 10.1038/ijo.2011.27

Akbari E, 2016, FRONT AGING NEUROSCI, V8, DOI 10.3389/fnagi.2016.00256

Akkasheh G, 2016, NUTRITION, V32, P315, DOI 10.1016/j.nut.2015.09.003

Neto DPA, 2022, ISCIENCE, V25, DOI 10.1016/j.isci.2022.103908

Ansari F, 2020, CURR PHARM BIOTECHNO, V21, P555, DOI 10.2174/1389201021666200107113812

Aoyama M, 2010, NUTRITION, V26, P653, DOI 10.1016/j.nut.2009.07.006

Arteaga-Henríquez G, 2020, TRIALS, V21, DOI 10.1186/s13063-019-4040-x

Baganz NL, 2013, ACS CHEM NEUROSCI, V4, P48, DOI 10.1021/cn300186b

Bahari-Javan S, 2017, P NATL ACAD SCI USA, V114, pE4686, DOI 10.1073/pnas.1613842114

Barichella M, 2019, MOVEMENT DISORD, V34, P396, DOI 10.1002/mds.27581

Barrett E, 2012, J APPL MICROBIOL, V113, P411, DOI 10.1111/j.1365-2672.2012.05344.x

Bercik P, 2011, GASTROENTEROLOGY, V141, P599, DOI 10.1053/j.gastro.2011.04.052

Besser MJ, 2005, J NEUROIMMUNOL, V169, P161, DOI 10.1016/j.jneuroim.2005.07.013

Blacher E, 2019, NATURE, V572, P474, DOI 10.1038/s41586-019-1443-5

Blum HE, 2017, ADV MED SCI-POLAND, V62, P414, DOI 10.1016/j.advms.2017.04.005

Boulos C, 2019, NUTRIENTS, V11, DOI 10.3390/nu11081896

Braak H, 2006, NEUROSCI LETT, V396, P67, DOI 10.1016/j.neulet.2005.11.012

Braniste V, 2014, SCI TRANSL MED, V6, DOI 10.1126/scitranslmed.3009759

Bravo JA, 2011, P NATL ACAD SCI USA, V108, P16050, DOI 10.1073/pnas.1102999108

Brenner LA, 2020, FRONT NEUROL, V11, DOI 10.3389/fneur.2020.01015

Buffington SA, 2016, CELL, V165, P1762, DOI 10.1016/j.cell.2016.06.001

Camara-Lemarroy CR, 2018, WORLD J GASTROENTERO, V24, P4217, DOI 10.3748/wjg.v24.i37.4217

Canfora EE, 2015, NAT REV ENDOCRINOL, V11, P577, DOI 10.1038/nrendo.2015.128

Cannavale CN, 2023, NUTR NEUROSCI, V26, P265, DOI 10.1080/1028415X.2022.2046963

Caputi V, 2018, INT J MOL SCI, V19, DOI 10.3390/ijms19061689

Cardoza PA, 2022, NUTR REV, V80, P271, DOI 10.1093/nutrit/nuab022

Cattaneo A, 2017, NEUROBIOL AGING, V49, P60, DOI 10.1016/j.neurobiolaging.2016.08.019

Chen H, 2003, AM J EPIDEMIOL, V157, P1007, DOI 10.1093/aje/kwg073

Chen SJ, 2022, NEUROLOGY, V98, pE848, DOI 10.1212/WNL.0000000000013225

Chen YJ, 2021, NUTRIENTS, V13, DOI 10.3390/nu13062099

Christian LM, 2015, BRAIN BEHAV IMMUN, V45, P118, DOI 10.1016/j.bbi.2014.10.018

Chung CG, 2018, CELL MOL LIFE SCI, V75, P3159, DOI 10.1007/s00018-018-2854-4

Cirstea MS, 2020, MOVEMENT DISORD, V35, P1208, DOI 10.1002/mds.28052

Conlon MA, 2015, NUTRIENTS, V7, P17, DOI 10.3390/nu7010017

Cox LM, 2019, BRAIN RES, V1724, DOI 10.1016/j.brainres.2019.146385

Cryan JF, 2020, LANCET NEUROL, V19, P179, DOI 10.1016/S1474-4422(19)30356-4

Cryan JF, 2012, NAT REV NEUROSCI, V13, P701, DOI 10.1038/nrn3346

Dalile B, 2019, NAT REV GASTRO HEPAT, V16, P461, DOI 10.1038/s41575-019-0157-3

Davis EC, 2020, DIGEST DIS SCI, V65, P706, DOI 10.1007/s10620-020-06092-x

De Angelis M, 2013, PLOS ONE, V8, DOI 10.1371/journal.pone.0076993

de Lau LML, 2006, LANCET NEUROL, V5, P525, DOI 10.1016/S1474-4422(06)70471-9

De-Paula VDR, 2018, PHARMACOL RES, V136, P29, DOI 10.1016/j.phrs.2018.07.007

Devlin AS, 2016, CELL HOST MICROBE, V20, P709, DOI 10.1016/j.chom.2016.10.021

Di Paolo G, 2011, NAT REV NEUROSCI, V12, P284, DOI 10.1038/nrn3012

Dickerson F, 2018, BIPOLAR DISORD, V20, P614, DOI 10.1111/bdi.12652

Dickerson Faith B, 2014, Prim Care Companion CNS Disord, V16, DOI 10.4088/PCC.13m01579

Dinan TG, 2017, GASTROENTEROL CLIN N, V46, P77, DOI 10.1016/j.gtc.2016.09.007

Dinan TG, 2017, NAT REV GASTRO HEPAT, V14, P69, DOI 10.1038/nrgastro.2016.200

Dinan TG, 2017, J PHYSIOL-LONDON, V595, P489, DOI 10.1113/JP273106

Dingeo G, 2020, FOOD FUNCT, V11, P8444, DOI [10.1039/d0fo01483d, 10.1039/D0FO01483D]

Doifode T, 2021, PHARMACOL RES, V164, DOI 10.1016/j.phrs.2020.105314

Dopkins N, 2018, IMMUNOLOGY, V154, P178, DOI 10.1111/imm.12903

Drouin-Ouellet J, 2012, TRENDS PHARMACOL SCI, V33, P542, DOI 10.1016/j.tips.2012.07.002

Du G, 2021, FRONT IMMUNOL, V11, DOI 10.3389/fimmu.2020.603594

Duscha A, 2020, CELL, V180, P1067, DOI 10.1016/j.cell.2020.02.035

El Aidy S, 2014, FRONT MICROBIOL, V5, DOI 10.3389/fmicb.2014.00146

Erny D, 2015, NAT NEUROSCI, V18, P965, DOI 10.1038/nn.4030

Everard A, 2014, ISME J, V8, P2116, DOI 10.1038/ismej.2014.45

Faintuch J, 2019, MICROBIOME AND METABOLOME IN DIAGNOSIS, THERAPY, AND OTHER STRATEGIC APPLICATIONS, P1

Féart C, 2009, JAMA-J AM MED ASSOC, V302, P638, DOI 10.1001/jama.2009.1146

Fedeli C, 2019, AUTOPHAGY, V15, P2044, DOI 10.1080/15548627.2019.1596489

Ferrante RJ, 2000, J NEUROSCI, V20, P4389, DOI 10.1523/JNEUROSCI.20-12-04389.2000

Ferreira CM, 2014, J IMMUNOL RES, V2014, DOI 10.1155/2014/689492

Ferreira-Halder CV, 2017, BEST PRACT RES CL GA, V31, P643, DOI 10.1016/j.bpg.2017.09.011

Figueroa-Romero C, 2020, DIS MODEL MECH, V13, DOI 10.1242/dmm.041947

Floch M.H., 2016, The microbiota in gastrointestinal pathophysiology: Implications for human health, prebiotics, probiotics, and dysbiosis

Foster JA, 2013, TRENDS NEUROSCI, V36, P305, DOI 10.1016/j.tins.2013.01.005

Friedland RP, 2015, J ALZHEIMERS DIS, V45, P349, DOI 10.3233/JAD-142841

Fujii Y., 2019, MICROEXON PUBL, P147, DOI [10.15586/alzheimersdisease.2019.ch9, 10.15586/ALZHEIMERSDISEASE.2019.CH9, DOI 10.15586/ALZHEIMERSDISEASE.2019.CH9]

Fulceri F, 2016, DIGEST LIVER DIS, V48, P248, DOI 10.1016/j.dld.2015.11.026

Fuloria S, 2022, FRONT MICROBIOL, V13, DOI 10.3389/fmicb.2022.950913

Galland L, 2014, J MED FOOD, V17, P1261, DOI 10.1089/jmf.2014.7000

Galloway S, 2007, J NUTR BIOCHEM, V18, P279, DOI 10.1016/j.jnutbio.2006.07.003

Galloway S, 2009, LIPIDS HEALTH DIS, V8, DOI 10.1186/1476-511X-8-46

Galvagnion C, 2017, J PARKINSON DIS, V7, P433, DOI 10.3233/JPD-171103

Garcia CJ, 2022, BIOMOLECULES, V12, DOI 10.3390/biom12050687

Ghaisas S, 2016, PHARMACOL THERAPEUT, V158, P52, DOI 10.1016/j.pharmthera.2015.11.012

Ghezzi L, 2022, BIOMEDICINES, V10, DOI 10.3390/biomedicines10071486

Ghezzi L, 2021, J CLIN INVEST, V131, DOI 10.1172/JCI143774

Gill HS, 2001, AM J CLIN NUTR, V74, P833

Goyal D, 2021, PROG NEURO-PSYCHOPH, V106, DOI 10.1016/j.pnpbp.2020.110112

Greco V, 2019, ADV EXP MED BIOL, V1158, P71, DOI [10.1007/978-981-13-8367-0_5, 10.1007/978-981-113-S.367-0_5]

Guo X, 2016, J APPL MICROBIOL, V120, P452, DOI 10.1111/jam.13022

Hansen EB, 2018, CURR OPIN FOOD SCI, V19, P98, DOI 10.1016/j.cofs.2018.03.004

Haran JP, 2019, MBIO, V10, DOI 10.1128/mBio.00632-19

Hauss-Wegrzyniak B, 2000, NEUROREPORT, V11, P1759, DOI 10.1097/00001756-200006050-00032

He BK, 2019, FRONT IMMUNOL, V10, DOI 10.3389/fimmu.2019.00385

Hertel J, 2019, CELL REP, V29, P1767, DOI 10.1016/j.celrep.2019.10.035

Hill C, 2014, NAT REV GASTRO HEPAT, V11, P506, DOI 10.1038/nrgastro.2014.66

Hill-Burns EM, 2017, MOVEMENT DISORD, V32, P739, DOI 10.1002/mds.26942

Hilton D, 2014, ACTA NEUROPATHOL, V127, P235, DOI 10.1007/s00401-013-1214-6

Hirschberg S, 2019, INT J MOL SCI, V20, DOI 10.3390/ijms20123109

Ho L, 2018, EXPERT REV NEUROTHER, V18, P83, DOI 10.1080/14737175.2018.1400909

Hojsak I, 2023, J PEDIATR GASTR NUTR, V76, P102, DOI 10.1097/MPG.0000000000003568

Hooper LV, 2001, SCIENCE, V292, P1115, DOI 10.1126/science.1058709

Hori T, 2020, MICROORGANISMS, V8, DOI 10.3390/microorganisms8091401

Howarth GS, 2013, NUTRIENTS, V5, P58, DOI 10.3390/nu5010058

Hoyles L, 2018, MICROBIOME, V6, DOI 10.1186/s40168-018-0439-y

Huang YL, 2021, INT J MOL SCI, V22, DOI 10.3390/ijms222010903

Human Microbiome Project Consortium, 2012, Nature, V486, P207, DOI 10.1038/nature11234

Jangi S, 2016, NAT COMMUN, V7, DOI 10.1038/ncomms12015

Jia W, 2020, MED RES REV, V40, P1496, DOI 10.1002/med.21653

Jiang CM, 2017, J ALZHEIMERS DIS, V58, P1, DOI 10.3233/JAD-161141

Jiang HY, 2018, BEHAV BRAIN RES, V347, P408, DOI 10.1016/j.bbr.2018.03.036

Jiang HY, 2015, BRAIN BEHAV IMMUN, V48, P186, DOI 10.1016/j.bbi.2015.03.016

Joseph J, 2009, J NEUROSCI, V29, P12795, DOI 10.1523/JNEUROSCI.3520-09.2009

Juzwik CA, 2019, PROG NEUROBIOL, V182, DOI 10.1016/j.pneurobio.2019.101664

Kalia LV, 2015, LANCET, V386, P896, DOI 10.1016/S0140-6736(14)61393-3

Kang DW, 2013, PLOS ONE, V8, DOI 10.1371/journal.pone.0068322

Kashtanova DA, 2016, NUTRITION, V32, P620, DOI 10.1016/j.nut.2015.12.037

Kasubuchi M, 2015, NUTRIENTS, V7, P2839, DOI 10.3390/nu7042839

Kazemi A, 2019, CLIN NUTR, V38, P522, DOI 10.1016/j.clnu.2018.04.010

Keshavarzian A, 2015, MOVEMENT DISORD, V30, P1351, DOI 10.1002/mds.26307

Kesika P, 2021, LIFE SCI, V264, DOI 10.1016/j.lfs.2020.118627

Kessler RC, 2005, ARCH GEN PSYCHIAT, V62, P617, DOI 10.1001/archpsyc.62.6.617

Kiriyama Y, 2019, BIOMOLECULES, V9, DOI 10.3390/biom9060232

Kobayashi Y, 2019, JPAD-J PREV ALZHEIM, V6, P70, DOI 10.14283/jpad.2018.32

Kong G, 2021, NEUROBIOL DIS, V148, DOI 10.1016/j.nbd.2020.105199

Kong G, 2020, NEUROBIOL DIS, V135, DOI 10.1016/j.nbd.2018.09.001

Kong XJ, 2020, PILOT FEASIBILITY ST, V6, DOI 10.1186/s40814-020-0557-8

Konjevod M, 2021, J PHARMACEUT BIOMED, V194, DOI 10.1016/j.jpba.2020.113681

Krautkramer KA, 2016, MOL CELL, V64, P982, DOI 10.1016/j.molcel.2016.10.025

Kumperscak HG, 2020, FRONT PSYCHIATRY, V11, DOI 10.3389/fpsyt.2020.00181

Lamkanfi M, 2014, CELL, V157, P1013, DOI 10.1016/j.cell.2014.04.007

Le Chatelier E, 2013, NATURE, V500, P541, DOI 10.1038/nature12506

Lee JH, 2010, FEMS MICROBIOL REV, V34, P426, DOI 10.1111/j.1574-6976.2009.00204.x

Levy M, 2016, GENE DEV, V30, P1589, DOI 10.1101/gad.284091.116

Lew M, 2007, PHARMACOTHERAPY, V27, p155S, DOI 10.1592/phco.27.12part2.155S

Li PP, 2021, METABOLITES, V11, DOI 10.3390/metabo11010029

Li PY, 2018, AM J CLIN NUTR, V108, P603, DOI 10.1093/ajcn/nqy116

Lin AQ, 2018, PARKINSONISM RELAT D, V53, P82, DOI 10.1016/j.parkreldis.2018.05.007

Liu JM, 2015, BIOMED RES INT, V2015, DOI 10.1155/2015/412946

Liu WH, 2016, BEHAV BRAIN RES, V298, P202, DOI 10.1016/j.bbr.2015.10.046

Liu XR, 2021, CONTEMP CLIN TRIALS, V102, DOI 10.1016/j.cct.2021.106270

Lord C, 2020, NAT REV DIS PRIMERS, V6, DOI 10.1038/s41572-019-0138-4

Luan HM, 2019, MASS SPECTROM REV, V38, P22, DOI 10.1002/mas.21553

Lutgendorff F, 2008, CURR MOL MED, V8, P282, DOI 10.2174/156652408784533779

Ma QQ, 2019, J NEUROINFLAMM, V16, DOI 10.1186/s12974-019-1434-3

MahmoudianDehkordi S, 2019, ALZHEIMERS DEMENT, V15, P76, DOI 10.1016/j.jalz.2018.07.217

MARTEAU TM, 1992, BRIT J CLIN PSYCHOL, V31, P301, DOI 10.1111/j.2044-8260.1992.tb00997.x

Martin C.R., 2021, DIAGNOSIS MANAGEMENT

Mawe GM, 2013, NAT REV GASTRO HEPAT, V10, P473, DOI 10.1038/nrgastro.2013.105

Mayer EA, 2014, BIOESSAYS, V36, P933, DOI 10.1002/bies.201400075

McCourt Andrew C, 2015, PLoS Curr, V7, DOI 10.1371/currents.hd.858b4cc7f235df068387e9c20c436a79

Minato T, 2017, PLOS ONE, V12, DOI 10.1371/journal.pone.0187307

Mitchell RHB, 2014, J AM ACAD CHILD PSY, V53, P274, DOI 10.1016/j.jaac.2013.11.013

Miyake S, 2015, PLOS ONE, V10, DOI 10.1371/journal.pone.0137429

Möhle L, 2016, CELL REP, V15, P1945, DOI 10.1016/j.celrep.2016.04.074

Mogensen TH, 2009, CLIN MICROBIOL REV, V22, P240, DOI 10.1128/CMR.00046-08

Moya A, 2016, TRENDS MICROBIOL, V24, P402, DOI 10.1016/j.tim.2016.02.002

Mulak A, 2021, J ALZHEIMERS DIS, V84, P461, DOI 10.3233/JAD-210608

Mulak A, 2015, WORLD J GASTROENTERO, V21, P10609, DOI 10.3748/wjg.v21.i37.10609

Narengaowa, 2021, FRONT CELL NEUROSCI, V15, DOI 10.3389/fncel.2021.633735

Navarro-López V, 2022, GENES-BASEL, V13, DOI 10.3390/genes13050930

Nayak D, 2014, ANNU REV IMMUNOL, V32, P367, DOI 10.1146/annurev-immunol-032713-120240

Nayak Debasis, 2012, Intravital, V1, P95, DOI 10.4161/intv.22823

Nho K, 2019, ALZHEIMERS DEMENT, V15, P232, DOI 10.1016/j.jalz.2018.08.012

Nichols RG, 2021, HUM GENET, V140, P747, DOI 10.1007/s00439-020-02237-0

Obermeier B, 2013, NAT MED, V19, P1584, DOI 10.1038/nm.3407

Ochoa-Repáraz J, 2018, CSH PERSPECT MED, V8, DOI 10.1101/cshperspect.a029017

Padhi P, 2022, FRONT NEUROSCI-SWITZ, V16, DOI 10.3389/fnins.2022.836605

Venegas DP, 2019, FRONT IMMUNOL, V10, DOI 10.3389/fimmu.2019.00277

Park Ah-Mee, 2017, Clin Exp Neuroimmunol, V8, P215, DOI 10.1111/cen3.12401

Pasinetti G.M., 2022, HDB MICROBIOME GUT B

Patterson E, 2020, NEUROBIOL STRESS, V13, DOI 10.1016/j.ynstr.2020.100277

Pellegrini C, 2018, ACTA NEUROPATHOL, V136, P345, DOI 10.1007/s00401-018-1856-5

Perez-Pardo P, 2019, GUT, V68, P829, DOI 10.1136/gutjnl-2018-316844

Peterson DA, 2007, CELL HOST MICROBE, V2, P328, DOI 10.1016/j.chom.2007.09.013

Plaza-Díaz J, 2017, NUTRIENTS, V9, DOI 10.3390/nu9060555

Pouclet H, 2012, NEUROGASTROENT MOTIL, V24, pe202, DOI 10.1111/j.1365-2982.2012.01887.x

Prehn-Kristensen A, 2018, PLOS ONE, V13, DOI 10.1371/journal.pone.0200728

Qin JJ, 2010, NATURE, V464, P59, DOI 10.1038/nature08821

Radulescu CI, 2019, NEUROBIOL DIS, V127, P65, DOI 10.1016/j.nbd.2019.02.011

Rane P, 2012, NEUROPHARMACOLOGY, V62, P2409, DOI 10.1016/j.neuropharm.2012.01.026

Ravishankar R.V., 2014, BENEF MICROBES

Reitz C, 2014, BIOCHEM PHARMACOL, V88, P640, DOI 10.1016/j.bcp.2013.12.024

Roager HM, 2018, NAT COMMUN, V9, DOI 10.1038/s41467-018-05470-4

Rode J, 2022, FRONT NUTR, V9, DOI 10.3389/fnut.2022.827182

Romano S, 2021, NPJ PARKINSONS DIS, V7, DOI 10.1038/s41531-021-00156-z

Romo-Vaquero M, 2022, FOOD FUNCT, V13, P6306, DOI [10.1039/d2fo00552b, 10.1039/D2FO00552B]

Rosario D, 2021, CELL REP, V34, DOI 10.1016/j.celrep.2021.108807

Saccarello Alberto, 2020, Prim Care Companion CNS Disord, V22, DOI 10.4088/PCC.19m02578

Sakaguchi S, 2008, CELL, V133, P775, DOI 10.1016/j.cell.2008.05.009

Sampson TR, 2016, CELL, V167, P1469, DOI 10.1016/j.cell.2016.11.018

Samsel A, 2013, ENTROPY-SWITZ, V15, P1416, DOI 10.3390/e15041416

Sanborn V, 2022, APPL PHYSIOL NUTR ME, V47, P871, DOI 10.1139/apnm-2021-0557

Santocchi E, 2020, FRONT PSYCHIATRY, V11, DOI 10.3389/fpsyt.2020.550593

Santocchi E, 2016, BMC PSYCHIATRY, V16, DOI 10.1186/s12888-016-0887-5

Santoro A, 2018, CELL MOL LIFE SCI, V75, P129, DOI 10.1007/s00018-017-2674-y

Saransaari P, 2007, AMINO ACIDS, V32, P439, DOI 10.1007/s00726-006-0375-1

Schaub AC, 2022, TRANSL PSYCHIAT, V12, DOI 10.1038/s41398-022-01977-z

Scheperjans F, 2015, MOVEMENT DISORD, V30, P350, DOI 10.1002/mds.26069

Sharon G, 2019, CELL, V177, P1600, DOI 10.1016/j.cell.2019.05.004

Sherman HT, 2022, BMC PSYCHIATRY, V22, DOI 10.1186/s12888-022-04151-3

Singh A, 2019, MOLECULES, V24, DOI 10.3390/molecules24081583

Sofi F., 2013, Current Nutrition Reports, V2, P75, DOI DOI 10.1007/S13668-013-0041-7

Solch RJ, 2022, J NEUROL SCI, V434, DOI 10.1016/j.jns.2022.120166

Song JG, 2022, COMPUT STRUCT BIOTEC, V20, P1097, DOI 10.1016/j.csbj.2022.02.024

Spielman LJ, 2018, NEUROCHEM INT, V120, P149, DOI 10.1016/j.neuint.2018.08.005

Srivastav S, 2019, J NUTR BIOCHEM, V69, P73, DOI 10.1016/j.jnutbio.2019.03.021

Statovci D, 2017, FRONT IMMUNOL, V8, DOI 10.3389/fimmu.2017.00838

Steenbergen L, 2015, BRAIN BEHAV IMMUN, V48, P258, DOI 10.1016/j.bbi.2015.04.003

Stilling RM, 2014, GENES BRAIN BEHAV, V13, P69, DOI 10.1111/gbb.12109

Stockinger B, 2014, ANNU REV IMMUNOL, V32, P403, DOI 10.1146/annurev-immunol-032713-120245

Strandwitz P, 2018, BRAIN RES, V1693, P128, DOI 10.1016/j.brainres.2018.03.015

Sun J, 2016, BRAIN RES, V1642, P180, DOI 10.1016/j.brainres.2016.03.042

Sun MF, 2018, AGEING RES REV, V45, P53, DOI 10.1016/j.arr.2018.04.004

Szentirmai E, 2019, SCI REP-UK, V9, DOI 10.1038/s41598-019-43502-1

Tagliabue A, 2013, NUTR METAB CARDIOVAS, V23, P160, DOI 10.1016/j.numecd.2012.09.002

TAKAKI M, 1985, NEUROSCIENCE, V16, P223, DOI 10.1016/0306-4522(85)90059-4

Tamtaji OR, 2019, CLIN NUTR, V38, P2569, DOI 10.1016/j.clnu.2018.11.034

Tan AH, 2021, ANN NEUROL, V89, P546, DOI 10.1002/ana.25982

Tan AH, 2021, NEUROLOGY, V96, pE772, DOI 10.1212/WNL.0000000000010998

Tan J, 2014, ADV IMMUNOL, V121, P91, DOI 10.1016/B978-0-12-800100-4.00003-9

Tang WHW, 2013, NEW ENGL J MED, V368, P1575, DOI 10.1056/NEJMoa1109400

Taylor JD, 2015, FRONT CELL INFECT MI, V5, DOI 10.3389/fcimb.2015.00033

Tengeler AC, 2020, MICROBIOME, V8, DOI 10.1186/s40168-020-00816-x

Thaiss CA, 2016, NATURE, V535, P65, DOI 10.1038/nature18847

Tierney Mary C, 2017, Alzheimers Dement (N Y), V3, P660, DOI 10.1016/j.trci.2017.10.006

Tilocca B, 2020, INT J MOL SCI, V21, DOI 10.3390/ijms21114045

Tojo R, 2014, WORLD J GASTROENTERO, V20, P15163, DOI 10.3748/wjg.v20.i41.15163

Tolhurst G, 2012, DIABETES, V61, P364, DOI 10.2337/db11-1019

Tran SMS, 2021, NUTRIENTS, V13, DOI 10.3390/nu13030732

Unger MM, 2016, PARKINSONISM RELAT D, V32, P66, DOI 10.1016/j.parkreldis.2016.08.019

van de Rest O, 2015, ADV NUTR, V6, P154, DOI 10.3945/an.114.007617

van de Wouw M, 2018, J PHYSIOL-LONDON, V596, P4923, DOI 10.1113/JP276431

van der Burg JMM, 2011, NEUROBIOL DIS, V44, P1, DOI 10.1016/j.nbd.2011.05.006

Vanuytsel T, 2014, GUT, V63, P1293, DOI 10.1136/gutjnl-2013-305690

Vascellari S, 2020, MSYSTEMS, V5, DOI 10.1128/mSystems.00561-20

Verbeke KA, 2015, NUTR RES REV, V28, P42, DOI 10.1017/S0954422415000037

Vogt NM, 2017, SCI REP-UK, V7, DOI 10.1038/s41598-017-13601-y

Vulevic J, 2008, AM J CLIN NUTR, V88, P1438, DOI 10.3945/ajcn.2008.26242

Wallace CJK, 2017, ANN GEN PSYCHIATR, V16, DOI 10.1186/s12991-017-0138-2

Wallen ZD, 2021, BMC BIOINFORMATICS, V22, DOI 10.1186/s12859-021-04193-6

Wang HY, 2018, NEUROTHERAPEUTICS, V15, P807, DOI 10.1007/s13311-018-0627-2

Wang HY, 2016, J NEUROGASTROENTEROL, V22, P589, DOI 10.5056/jnm16018

Wang SS, 2018, J PAIN RES, V11, P1521, DOI 10.2147/JPR.S168008

Wang Y, 2019, CNS NEUROSCI THER, V25, P816, DOI 10.1111/cns.13116

Wasser CI, 2020, BRAIN COMMUN, V2, DOI 10.1093/braincomms/fcaa110

Wauters L, 2022, GUT MICROBES, V14, DOI 10.1080/19490976.2022.2031695

Westfall S, 2017, CELL MOL LIFE SCI, V74, P3769, DOI 10.1007/s00018-017-2550-9

Whitfield C, 2014, ANNU REV BIOCHEM, V83, P99, DOI 10.1146/annurev-biochem-060713-035600

Wolfe M.S., 2018, The molecular and cellular basis of neurodegenerative disease: underlying mechanisms

Wu GD, 2011, SCIENCE, V334, P105, DOI 10.1126/science.1208344

Wu JY, 2009, ADV EXP MED BIOL, V643, P169, DOI 10.1007/978-0-387-75681-3_17

Wu L, 2021, NUTRIENTS, V13, DOI 10.3390/nu13010228

Wu SI, 2022, FOOD FUNCT, V13, P8907, DOI [10.1039/d2fo01156e, 10.1039/D2FO01156E]

Wu SI, 2021, FRONT NUTR, V8, DOI 10.3389/fnut.2021.614105

Xia Y, 2022, An Integrated Analysis of Microbiomes and Metabolomics

Yanguas-Casás N, 2017, J CELL PHYSIOL, V232, P2231, DOI 10.1002/jcp.25742

Ye JZ, 2018, FRONT MICROBIOL, V9, DOI 10.3389/fmicb.2018.01967

Zhao YH, 2017, FRONT CELL INFECT MI, V7, DOI 10.3389/fcimb.2017.00318

Zhu GS, 2021, NUTRIENTS, V13, DOI 10.3390/nu13051602

Zhu SB, 2020, J NEUROINFLAMM, V17, DOI 10.1186/s12974-020-1705-z

NR 258

TC 15

Z9 15

U1 2

U2 18

PU MDPI

PI BASEL

PA ST ALBAN-ANLAGE 66, CH-4052 BASEL, SWITZERLAND

EI 2072-6643

J9 NUTRIENTS

JI Nutrients

PD OCT

PY 2022

VL 14

IS 19

AR 3967

DI 10.3390/nu14193967

PG 33

WC Nutrition & Dietetics

WE Science Citation Index Expanded (SCI-EXPANDED)

SC Nutrition & Dietetics

GA 5H9JN

UT WOS:000867987000001

PM 36235622

OA Green Published, gold

DA 2024-08-12

ER

PT J

AU Anwar, S

Pons, V

Rivest, S

AF Anwar, Shehata

Pons, Vincent

Rivest, Serge

TI Microglia Purinoceptor P2Y6: An Emerging Therapeutic Target in CNS

Diseases

SO CELLS

LA English

DT Review

DE microglia; phagocytosis; neuroinflammation; pro-inflammatory cytokines

ID MOLECULAR-MECHANISMS; NUCLEOTIDE RECEPTORS; AUTOPHAGY; PHAGOCYTOSIS;

UDP; INFLAMMATION; INHIBITION; ACTIVATION; CELLS; NEUROINFLAMMATION

AB The purinergic receptor P2Y6 is expressed in immune cells, including the microglia that are implicated in neurological disorders. Its ligand, UDP, is a signaling molecule that can serve as an "find-me" signal when released in significant quantities by damaged/dying cells. The binding of UDP by P2Y6R leads to the activation of different biochemical pathways, depending on the disease context and the pathological environment. Generally, P2Y6R stimulates phagocytosis. However, whether or not phagocytosis coincides with cell activation or the secretion of pro-inflammatory cytokines needs further investigation. The current review aims to discuss the various functions of P2Y6R in some CNS disorders. We present evidence that P2Y6R may have a detrimental or beneficial role in the nervous system, in the context of neurological pathologies, such as ischemic stroke, Alzheimer's disease, Parkinson's disease, radiation-induced brain injury, and neuropathic pain.

C1 [Anwar, Shehata; Pons, Vincent; Rivest, Serge] Laval Univ, Fac Med, CHU Quebec Res Ctr CHUL, Dept Mol Med,Neurosci Lab, Quebec City, PQ G1V 0A6, Canada.

[Anwar, Shehata] Beni Suef Univ, Fac Vet Med, Dept Pathol, Bani Suwayf 62511, Egypt.

C3 Laval University; Egyptian Knowledge Bank (EKB); Beni Suef University

RP Rivest, S (corresponding author), Laval Univ, Fac Med, CHU Quebec Res Ctr CHUL, Dept Mol Med,Neurosci Lab, Quebec City, PQ G1V 0A6, Canada.

EM shehata-ibrahim-anwar.shehata.1@ulaval.ca; vincent.pons.1@ulaval.ca;

serge.rivest@crchudequebec.ulaval.ca

RI Pons, Vincent/AAW-9222-2021

OI Pons, Vincent/0000-0002-1816-4308; Anwar, Shehata/0000-0002-6552-7511;

Rivest, Serge/0000-0002-6082-770X

FU Canadian Institutes in Health Research (CIHR) [331652]; les Fonds de

Recherche du Quebec-Sante (FRQS); Canada Research Chair in

Neuroimmunology

FX This work is supported by the Canadian Institutes in Health Research

(CIHR; grant no. 331652) and les Fonds de Recherche du Quebec-Sante

(FRQS) via the research center funding grant. Serge Rivest is supported

by the Canada Research Chair in Neuroimmunology.

CR Abbracchio MP, 2006, PHARMACOL REV, V58, P281, DOI 10.1124/pr.58.3.3

Ali S, 2018, PURINERG SIGNAL, V14, P371, DOI 10.1007/s11302-018-9618-3

Ampil FL, 2012, J ORAL MAXIL SURG, V70, P730, DOI 10.1016/j.joms.2011.03.037

Anwar S, 2020, EXPERT OPIN THER TAR, V24, P331, DOI 10.1080/14728222.2020.1738391

Bernier LP, 2013, GLIA, V61, P2038, DOI 10.1002/glia.22574

Bian J, 2019, PAIN RES MANAG, V2019, DOI 10.1155/2019/2612534

Burnstock G, 2012, BRIT J PHARMACOL, V167, P238, DOI 10.1111/j.1476-5381.2012.02008.x

BURNSTOCK G, 1985, GEN PHARMACOL, V16, P433, DOI 10.1016/0306-3623(85)90001-1

BURNSTOCK G, 1972, PHARMACOL REV, V24, P509

Burnstock G, 2004, INT REV CYTOL, V240, P31, DOI 10.1016/S0074-7696(04)40002-3

BURNSTOCK G, 1976, J THEOR BIOL, V62, P491, DOI 10.1016/0022-5193(76)90133-8

Burnstock G., 1978, CELL MEMBRAIN RECEPT, P107, DOI DOI 10.1016/0014-5793(79)81367-8

Burnstock Geoffrey, 2018, Brain Neurosci Adv, V2, p2398212818817494, DOI 10.1177/2398212818817494

Caggiu E, 2019, FRONT NEUROL, V10, DOI 10.3389/fneur.2019.00122

da Fonseca ACC, 2014, FRONT CELL NEUROSCI, V8, DOI 10.3389/fncel.2014.00362

Chen GF, 2017, ACTA PHARMACOL SIN, V38, P1205, DOI 10.1038/aps.2017.28

Chen J, 2014, FRONT IMMUNOL, V5, P1, DOI 10.3389/fimmu.2014.00656

Cieslak M, 2018, PURINERG SIGNAL, V14, P331, DOI 10.1007/s11302-018-9629-0

Colloca L, 2017, NAT REV DIS PRIMERS, V3, DOI 10.1038/nrdp.2017.2

Cox MA, 2005, BIOCHEM BIOPH RES CO, V330, P467, DOI 10.1016/j.bbrc.2005.03.004

Darios F, 2020, J MOL BIOL, V432, P2714, DOI 10.1016/j.jmb.2020.02.033

Dietrich J, 2008, ONCOLOGIST, V13, P1285, DOI 10.1634/theoncologist.2008-0130

Donkor ES, 2018, STROKE RES TREAT, V2018, DOI 10.1155/2018/3238165

Färber K, 2006, PFLUG ARCH EUR J PHY, V452, P615, DOI 10.1007/s00424-006-0064-7

Finnerup NB, 2015, LANCET NEUROL, V14, P162, DOI 10.1016/S1474-4422(14)70251-0

Fu RY, 2014, MOL NEUROBIOL, V49, P1422, DOI 10.1007/s12035-013-8620-6

Garaschuk O, 2019, METHODS MOL BIOL, V2034, P27, DOI 10.1007/978-1-4939-9658-2_3

Garcia RA, 2014, PLOS ONE, V9, DOI 10.1371/journal.pone.0111385

Ginhoux F, 2018, NAT NEUROSCI, V21, P897, DOI 10.1038/s41593-018-0176-3

Ginhoux F, 2013, FRONT CELL NEUROSCI, V7, DOI 10.3389/fncel.2013.00045

Glick D, 2010, J PATHOL, V221, P3, DOI 10.1002/path.2697

Greene-Schloesser D, 2012, FRONT ONCOL, V2, P1, DOI 10.3389/fonc.2012.00073

Guruswamy R, 2017, INT J MOL SCI, V18, DOI 10.3390/ijms18030496

Gutmann DH, 2019, NEURON, V104, P442, DOI 10.1016/j.neuron.2019.08.028

Harry GJ, 2008, EXPERT OPIN DRUG MET, V4, P1265, DOI 10.1517/17425255.4.10.1265

Haukedal H, 2019, J MOL BIOL, V431, P1818, DOI 10.1016/j.jmb.2019.02.004

Heneka MT, 2015, LANCET NEUROL, V14, P388, DOI 10.1016/S1474-4422(15)70016-5

Heneka MT, 2014, NAT REV IMMUNOL, V14, P463, DOI 10.1038/nri3705

Herrington FD, 2016, J BIOMOL SCREEN, V21, P223, DOI 10.1177/1087057115617456

Herz J, 2017, IMMUNITY, V46, P943, DOI 10.1016/j.immuni.2017.06.007

Inoue K, 2007, CELL ADHES MIGR, V1, P131, DOI 10.4161/cam.1.3.4937

Inoue K, 2009, INT REV NEUROBIOL, V85, P159, DOI 10.1016/S0074-7742(09)85012-5

Ito M, 2015, EUR J PHARMACOL, V749, P124, DOI 10.1016/j.ejphar.2014.12.024

Jacquel A, 2012, BLOOD, V119, P4527, DOI 10.1182/blood-2011-11-392167

Jacquel A, 2009, BLOOD, V114, P3633, DOI 10.1182/blood-2009-03-208843

Ji RR, 2018, ANESTHESIOLOGY, V129, P343, DOI 10.1097/ALN.0000000000002130

Kierdorf K, 2017, J CLIN INVEST, V127, P3201, DOI [10.1172/jci90602, 10.1172/JCI90602]

Kigerl KA, 2014, EXP NEUROL, V258, P5, DOI 10.1016/j.expneurol.2014.01.001

Kim SG, 2003, CELL MOL NEUROBIOL, V23, P401, DOI 10.1023/A:1023696806609

Kobayashi K, 2012, GLIA, V60, P1529, DOI 10.1002/glia.22373

Koizumi S, 2007, NATURE, V446, P1091, DOI 10.1038/nature05704

Korcok J, 2005, J BIOL CHEM, V280, P16909, DOI 10.1074/jbc.M410764200

Lawrence T, 2009, CSH PERSPECT BIOL, V1, DOI 10.1101/cshperspect.a001651

Lecours C, 2018, FRONT CELL NEUROSCI, V12, DOI 10.3389/fncel.2018.00282

Li S, 2018, INT J MOL SCI, V19, DOI 10.3390/ijms19072095

Liu B, 2003, J PHARMACOL EXP THER, V304, P1, DOI 10.1124/jpet.102.035048

Liu GD, 2009, NEUROSCI BULL, V25, P161, DOI 10.1007/s12264-009-0120-3

Liu T, 2017, SIGNAL TRANSDUCT TAR, V2, DOI 10.1038/sigtrans.2017.23

Lumniczky K, 2017, FRONT IMMUNOL, V8, DOI 10.3389/fimmu.2017.00517

LUTHJE J, 1989, KLIN WOCHENSCHR, V67, P317, DOI 10.1007/BF01741386

Madry C, 2015, J BIOL CHEM, V290, P12443, DOI 10.1074/jbc.R115.637157

Maiti P, 2017, TRANSL NEURODEGENER, V6, DOI 10.1186/s40035-017-0099-z

Maleki AF, 2019, FRONT CELL NEUROSCI, V13, DOI 10.3389/fncel.2019.00355

Mariani MM, 2009, J NEUROIMMUNE PHARM, V4, P448, DOI 10.1007/s11481-009-9170-6

McGeer PL, 2004, PARKINSONISM RELAT D, V10, pS3, DOI 10.1016/j.parkreldis.2004.01.005

Mhyre Timothy R, 2012, Subcell Biochem, V65, P389, DOI 10.1007/978-94-007-5416-4_16

Miller AM, 2009, GLIA, V57, P875, DOI 10.1002/glia.20813

Nau R, 2014, FRONT CELL NEUROSCI, V8, DOI 10.3389/fncel.2014.00138

Navone F, 2015, CELLS-BASEL, V4, P354, DOI 10.3390/cells4030354

Neher JJ, 2014, GLIA, V62, P1463, DOI 10.1002/glia.22693

Obba S, 2015, AUTOPHAGY, V11, P1114, DOI 10.1080/15548627.2015.1034406

Ohsawa K, 2007, GLIA, V55, P604, DOI 10.1002/glia.20489

Oliveira-Giacomelli A, 2018, FRONT PHARMACOL, V9, DOI 10.3389/fphar.2018.00325

Orihuela R, 2016, BRIT J PHARMACOL, V173, P649, DOI 10.1111/bph.13139

Park MH, 2016, CELLS-BASEL, V5, DOI 10.3390/cells5020015

Qin C, 2019, NEUROSCI BULL, V35, P921, DOI 10.1007/s12264-019-00388-3

Saul A, 2013, FRONT CELL NEUROSCI, V7, DOI 10.3389/fncel.2013.00250

Serrano-Pozo A, 2011, CSH PERSPECT MED, V1, DOI 10.1101/cshperspect.a006189

Skaper SD, 2018, FRONT CELL NEUROSCI, V12, DOI 10.3389/fncel.2018.00072

Tansey MG, 2010, NEUROBIOL DIS, V37, P510, DOI 10.1016/j.nbd.2009.11.004

Trautmann A, 2009, SCI SIGNAL, V2, DOI 10.1126/scisignal.256pe6

Tay TL, 2017, J PHYSIOL-LONDON, V595, P1929, DOI 10.1113/JP272134

Vernon PJ, 2013, ANTIOXID REDOX SIGN, V18, P677, DOI 10.1089/ars.2012.4810

Vieira RP, 2011, AM J RESP CRIT CARE, V184, P215, DOI 10.1164/rccm.201011-1762OC

Voet S, 2019, TRENDS MOL MED, V25, P112, DOI 10.1016/j.molmed.2018.11.005

Wang MM, 2018, ANN TRANSL MED, V6, DOI 10.21037/atm.2018.04.20

Wang WY, 2015, ANN TRANSL MED, V3, DOI 10.3978/j.issn.2305-5839.2015.03.49

Warny M, 2001, J BIOL CHEM, V276, P26051, DOI 10.1074/jbc.M102568200

Weinstein JR, 2010, FUTUR NEUROL, V5, P227, DOI 10.2217/FNL.10.1

Welsh LC, 2014, CLIN ONCOL-UK, V26, P765, DOI 10.1016/j.clon.2014.06.014

Wen RX, 2020, CNS NEUROSCI THER, V26, P416, DOI 10.1111/cns.13296

Wendt S, 2017, NEUROBIOL AGING, V58, P41, DOI 10.1016/j.neurobiolaging.2017.05.027

Wirawan E, 2012, CELL RES, V22, P43, DOI 10.1038/cr.2011.152

Woods LT, 2016, NEUROPHARMACOLOGY, V104, P169, DOI 10.1016/j.neuropharm.2015.10.031

Xu YT, 2016, MOL NEUROBIOL, V53, P3552, DOI 10.1007/s12035-015-9282-3

Yang XD, 2017, J NEUROINFLAMM, V14, DOI 10.1186/s12974-017-0795-8

Yang ZF, 2010, NAT CELL BIOL, V12, P814, DOI 10.1038/ncb0910-814

Yu WQ, 2013, N-S ARCH PHARMACOL, V386, P885, DOI 10.1007/s00210-013-0894-8

Zhang Z, 2011, J IMMUNOL, V186, P5376, DOI 10.4049/jimmunol.1002946

Zhao SC, 2017, ACTA PHARMACOL SIN, V38, P445, DOI 10.1038/aps.2016.162

Zhou MZ, 2020, DRUG DISCOV TODAY, V25, P568, DOI 10.1016/j.drudis.2019.12.015

NR 101

TC 32

Z9 35

U1 1

U2 23

PU MDPI

PI BASEL

PA ST ALBAN-ANLAGE 66, CH-4052 BASEL, SWITZERLAND

EI 2073-4409

J9 CELLS-BASEL

JI Cells

PD JUL

PY 2020

VL 9

IS 7

AR 1595

DI 10.3390/cells9071595

PG 13

WC Cell Biology

WE Science Citation Index Expanded (SCI-EXPANDED)

SC Cell Biology

GA MY8WX

UT WOS:000558701200001

PM 32630251

OA gold, Green Published

DA 2024-08-12

ER

PT J

AU Batista, CRA

Gomes, GF

Candelario-Jalil, E

Fiebich, BL

de Oliveira, ACP

AF Alvares Batista, Carla Ribeiro

Gomes, Giovanni Freitas

Candelario-Jalil, Eduardo

Fiebich, Bernd L.

Pinheiro de Oliveira, Antonio Carlos

TI Lipopolysaccharide-Induced Neuroinflammation as a Bridge to Understand

Neurodegeneration

SO INTERNATIONAL JOURNAL OF MOLECULAR SCIENCES

LA English

DT Review

DE lipopolysaccharide; inflammation; neurodegeneration; Alzheimer's

disease; Parkinson's disease; amyotrophic lateral sclerosis;

Huntington's disease

ID AMYOTROPHIC-LATERAL-SCLEROSIS; NITRIC-OXIDE SYNTHASE; KINASE-C-DELTA;

TRANSGENIC MOUSE MODEL; SINGLE INTRANIGRAL INJECTION; NIGRAL

DOPAMINERGIC-NEURONS; INDUCED parkinson's disease; AMYLOID PRECURSOR

PROTEIN; NECROSIS-FACTOR-ALPHA; TOLL-LIKE RECEPTORS

AB A large body of experimental evidence suggests that neuroinflammation is a key pathological event triggering and perpetuating the neurodegenerative process associated with many neurological diseases. Therefore, different stimuli, such as lipopolysaccharide (LPS), are used to model neuroinflammation associated with neurodegeneration. By acting at its receptors, LPS activates various intracellular molecules, which alter the expression of a plethora of inflammatory mediators. These factors, in turn, initiate or contribute to the development of neurodegenerative processes. Therefore, LPS is an important tool for the study of neuroinflammation associated with neurodegenerative disease. However, the serotype, route of administration, and number of injections of this toxin induce varied pathological responses. Thus, here, we review the use of LPS in various models of neurodegeneration as well as discuss the neuroinflammatory mechanisms induced by this toxin that could underpin the pathological events linked to the neurodegenerative process.

C1 [Alvares Batista, Carla Ribeiro; Gomes, Giovanni Freitas; Pinheiro de Oliveira, Antonio Carlos] Univ Fed Minas Gerais, Dept Pharmacol, Av Antonio Carlos 6627, BR-31270901 Belo Horizonte, MG, Brazil.

[Candelario-Jalil, Eduardo] Univ Florida, Dept Neurosci, Gainesville, FL 32610 USA.

[Fiebich, Bernd L.] Univ Freiburg, Neuroimmunol & Neurochem Res Grp, Dept Psychiat & Psychotherapy, Med Ctr,Fac Med, D-79104 Freiburg, Germany.

C3 Universidade Federal de Minas Gerais; State University System of

Florida; University of Florida; University of Freiburg

RP de Oliveira, ACP (corresponding author), Univ Fed Minas Gerais, Dept Pharmacol, Av Antonio Carlos 6627, BR-31270901 Belo Horizonte, MG, Brazil.; Fiebich, BL (corresponding author), Univ Freiburg, Neuroimmunol & Neurochem Res Grp, Dept Psychiat & Psychotherapy, Med Ctr,Fac Med, D-79104 Freiburg, Germany.

EM cacaribeiro@gmail.com; gvnngomes@gmail.com; ecandelario@ufl.edu;

bernd.fiebich@uniklinik-freiburg.de; antoniooliveira@icb.ufmg.br

RI Candelario-Jalil, Eduardo/I-1139-2019; Gomes, Giovanni

Freitas/ABE-7314-2021; de Oliveira, Antonio CP/F-1661-2013

OI Candelario-Jalil, Eduardo/0000-0003-3631-1989; Gomes, Giovanni

Freitas/0000-0002-4855-3056; Fiebich, Bernd L./0000-0002-9261-6771;

Batista, Carla/0000-0001-5826-4330

FU Fundacao de Amparo a Pesquisa do Estado de Minas Gerais (FAPEMIG)

[APQ-02044-15]; Conselho Nacional de Desenvolvimento Cientifico e

Tecnologico (CNPq) [424588/2016-1]; Coordenacao de Aperfeicoamento de

Pessoal de Nivel Superior (CAPES); CNPq [310347/2018-1]; University of

Freiburg Library

FX We thank Fundacao de Amparo a Pesquisa do Estado de Minas Gerais

(FAPEMIG-process number: APQ-02044-15), Conselho Nacional de

Desenvolvimento Cientifico e Tecnologico (CNPq-process number

424588/2016-1), and Coordenacao de Aperfeicoamento de Pessoal de Nivel

Superior (CAPES) for the financial support. A.C.P.d.O. acknowledge CNPq

for the productivity fellowship (310347/2018-1). The article processing

charge was funded by the University of Freiburg Library in the funding

program "Open Access Publishing".

CR Acosta C, 2008, J NEUROSCI RES, V86, P1077, DOI 10.1002/jnr.21565

Akundi RS, 2005, GLIA, V51, P199, DOI 10.1002/glia.20198

Alexianu ME, 2001, NEUROLOGY, V57, P1282, DOI 10.1212/WNL.57.7.1282

Alpizar YA, 2017, NAT COMMUN, V8, DOI 10.1038/s41467-017-01201-3

Anantharam V, 2002, J NEUROSCI, V22, P1738, DOI 10.1523/JNEUROSCI.22-05-01738.2002

Arai H, 2004, J BIOL CHEM, V279, P51647, DOI 10.1074/jbc.M407328200

Bachstetter AD, 2011, J NEUROINFLAMM, V8, DOI 10.1186/1742-2094-8-79

Bardou I, 2014, NEUROBIOL AGING, V35, P1065, DOI 10.1016/j.neurobiolaging.2013.11.006

BARGER S, 2004, J NEUROSCI RES, V62, P503, DOI DOI 10.1002/1097-4547(20001115)62:4

Barton SM, 2019, J ALZHEIMERS DIS, V67, P503, DOI 10.3233/JAD-180755

BAUER J, 1991, FEBS LETT, V285, P111, DOI 10.1016/0014-5793(91)80737-N

Bauer MKA, 1997, EUR J BIOCHEM, V243, P726, DOI 10.1111/j.1432-1033.1997.00726.x

Behairi N, 2016, J NEUROIMMUNOL, V300, P21, DOI 10.1016/j.jneuroim.2016.10.004

Beier EE, 2017, NEUROBIOL DIS, V108, P115, DOI 10.1016/j.nbd.2017.08.009

Bhat NR, 1998, J NEUROSCI, V18, P1633

Bhatia HS, 2017, SCI REP-UK, V7, DOI 10.1038/s41598-017-00225-5

Björkqvist M, 2008, J EXP MED, V205, P1869, DOI 10.1084/jem.20080178

Bonifati V, 2003, SCIENCE, V299, P256, DOI 10.1126/science.1077209

Boonen B, 2018, CELL CALCIUM, V73, P72, DOI 10.1016/j.ceca.2018.04.004

Bossu P, 2012, J NEUROINFLAMM, V9, DOI 10.1186/1742-2094-9-101

Bouchard J, 2012, J NEUROSCI, V32, P18259, DOI 10.1523/JNEUROSCI.4008-12.2012

BRAAK H, 1991, ACTA NEUROPATHOL, V82, P239, DOI 10.1007/BF00308809

Bronstein DM, 1995, BRAIN RES, V704, P112, DOI 10.1016/0006-8993(95)01189-7

BRUGG B, 1995, P NATL ACAD SCI USA, V92, P3032, DOI 10.1073/pnas.92.7.3032

BUGIANI O, 1980, EUR NEUROL, V19, P339, DOI 10.1159/000115172

Burguillos MA, 2011, NEUROBIOL DIS, V41, P177, DOI 10.1016/j.nbd.2010.09.005

Byrne S, 2011, J NEUROL NEUROSUR PS, V82, P623, DOI 10.1136/jnnp.2010.224501

Castaño A, 1998, J NEUROCHEM, V70, P1584

Castaño A, 2002, J NEUROCHEM, V81, P150, DOI 10.1046/j.1471-4159.2002.00799.x

Cazareth J, 2014, J NEUROINFLAMM, V11, DOI 10.1186/1742-2094-11-132

Charles T, 2001, J Neurosci Nurs, V33, P245

Chien CH, 2016, PLOS ONE, V11, DOI 10.1371/journal.pone.0151569

Chistyakov DV, 2018, INT J MOL SCI, V19, DOI 10.3390/ijms19010113

Choi DY, 2009, PLOS ONE, V4, DOI 10.1371/journal.pone.0005482

Cockerill I, 2018, ADV EXP MED BIOL, V1097, P261, DOI 10.1007/978-3-319-96445-4_14

Cookson MR, 2009, MOL NEURODEGENER, V4, DOI 10.1186/1750-1326-4-9

Correia AS, 2015, PLOS ONE, V10, DOI 10.1371/journal.pone.0140248

Couch Y, 2011, J NEUROINFLAMM, V8, DOI 10.1186/1742-2094-8-166

Cuenda A, 2007, BBA-MOL CELL RES, V1773, P1358, DOI 10.1016/j.bbamcr.2007.03.010

Czapski GA, 2007, J NEUROSCI RES, V85, P1694, DOI 10.1002/jnr.21294

Daher JPL, 2014, P NATL ACAD SCI USA, V111, P9289, DOI 10.1073/pnas.1403215111

Dalrymple A, 2007, J PROTEOME RES, V6, P2833, DOI 10.1021/pr0700753

Dauer W, 2003, NEURON, V39, P889, DOI 10.1016/S0896-6273(03)00568-3

Deng Xiaohua, 2014, Adv Alzheimer Dis, V3, P78

Dewil M, 2007, NEUROBIOL DIS, V26, P332, DOI 10.1016/j.nbd.2006.12.023

Erickson MA, 2012, BRAIN BEHAV IMMUN, V26, P1085, DOI 10.1016/j.bbi.2012.07.003

Erickson MA, 2012, J NEUROINFLAMM, V9, DOI 10.1186/1742-2094-9-150

Fiebich BL, 2018, FRONT CELL NEUROSCI, V12, DOI 10.3389/fncel.2018.00329

Fiebich BL, 2000, J IMMUNOL, V165, P5606, DOI 10.4049/jimmunol.165.10.5606

Fiebich BL, 1998, J NEUROIMMUNOL, V92, P170, DOI 10.1016/S0165-5728(98)00201-X

Fiebich BL, 2002, J NEUROIMMUNOL, V132, P18, DOI 10.1016/S0165-5728(02)00279-5

Fitzgerald KA, 2003, NAT IMMUNOL, V4, P491, DOI 10.1038/ni921

Flores-Martinez YM, 2018, J IMMUNOL RES, V2018, DOI 10.1155/2018/1838921

Folstein S E, 1991, Res Publ Assoc Res Nerv Ment Dis, V69, P181

Franciosi S, 2012, NEUROBIOL DIS, V45, P438, DOI 10.1016/j.nbd.2011.09.003

François A, 2013, J NEUROINFLAMM, V10, DOI 10.1186/1742-2094-10-151

Gao HM, 2002, J NEUROCHEM, V81, P1285, DOI 10.1046/j.1471-4159.2002.00928.x

Gao HM, 2011, ENVIRON HEALTH PERSP, V119, P807, DOI 10.1289/ehp.1003013

Gayle DA, 2002, DEV BRAIN RES, V133, P27, DOI 10.1016/S0165-3806(01)00315-7

Glass CK, 2010, CELL, V140, P918, DOI 10.1016/j.cell.2010.02.016

Go M, 2016, BIOCHEM BIOPH RES CO, V479, P331, DOI 10.1016/j.bbrc.2016.09.073

Gordon R, 2016, NEUROBIOL DIS, V93, P96, DOI 10.1016/j.nbd.2016.04.008

Graves MC, 2004, AMYOTROPH LATERAL SC, V5, P213, DOI 10.1080/14660820410020286

Gray P, 2011, J IMMUNOL, V187, P4331, DOI 10.4049/jimmunol.1100890

Gu C, 2018, FRONT CELL NEUROSCI, V12, DOI 10.3389/fncel.2018.00400

Gunawardena S, 2005, ARCH NEUROL-CHICAGO, V62, P46, DOI 10.1001/archneur.62.1.46

GURNEY ME, 1994, SCIENCE, V264, P1772, DOI 10.1126/science.8209258

Hauss-Wegrzyniak B, 1998, BRAIN RES, V780, P294, DOI 10.1016/S0006-8993(97)01215-8

Heppner FL, 2015, NAT REV NEUROSCI, V16, P358, DOI 10.1038/nrn3880

Herber DL, 2006, GLIA, V53, P382, DOI 10.1002/glia.20272

Herber DL, 2004, EXP NEUROL, V190, P245, DOI 10.1016/j.expneurol.2004.07.007

Herber DL, 2007, J NEUROIMMUNE PHARM, V2, P222, DOI 10.1007/s11481-007-9069-z

Herrera AJ, 2000, NEUROBIOL DIS, V7, P429, DOI 10.1006/nbdi.2000.0289

Hoozemans JJM, 2002, BRAIN RES, V951, P218, DOI 10.1016/S0006-8993(02)03164-5

Hsiao HY, 2013, HUM MOL GENET, V22, P1826, DOI 10.1093/hmg/ddt036

Huang WJ, 2016, EXP THER MED, V12, P1951, DOI 10.3892/etm.2016.3566

Huang YY, 2018, BRAIN BEHAV IMMUN, V71, P93, DOI 10.1016/j.bbi.2018.04.006

HUDSON AJ, 1981, BRAIN, V104, P217, DOI 10.1093/brain/104.2.217

Hunter R, 2017, NEUROSCI RES, V114, P62, DOI 10.1016/j.neures.2016.09.007

Hunter RL, 2008, NEUROSCI LETT, V432, P198, DOI 10.1016/j.neulet.2007.12.019

Hunter RL, 2007, J NEUROCHEM, V100, P1375, DOI 10.1111/j.1471-4159.2006.04327.x

Hunter RL, 2009, J NEUROSCI RES, V87, P1913, DOI 10.1002/jnr.22012

Iravani MM, 2005, EUR J NEUROSCI, V22, P317, DOI 10.1111/j.1460-9568.2005.04220.x

Iravani MM, 2002, NEUROSCIENCE, V110, P49, DOI 10.1016/S0306-4522(01)00562-0

Ittner LM, 2011, NAT REV NEUROSCI, V12, P67, DOI 10.1038/nrn2967

Jaeger LB, 2009, BRAIN BEHAV IMMUN, V23, P507, DOI 10.1016/j.bbi.2009.01.017

Jahn Holger, 2013, Dialogues Clin Neurosci, V15, P445

Jangula A, 2013, NEUROSCI LETT, V551, P23, DOI 10.1016/j.neulet.2013.06.058

JAVOYAGID F, 1980, NEUROLOGY, V30, P1326, DOI 10.1212/WNL.30.12.1326

Jeohn GH, 2002, ANN NY ACAD SCI, V962, P332, DOI 10.1111/j.1749-6632.2002.tb04078.x

Kanekiyo T, 2014, FRONT AGING NEUROSCI, V6, DOI 10.3389/fnagi.2014.00093

Katafuchi T, 2012, ANN NY ACAD SCI, V1262, P85, DOI 10.1111/j.1749-6632.2012.06641.x

Kaul S, 2003, EUR J NEUROSCI, V18, P1387, DOI 10.1046/j.1460-9568.2003.02864.x

Kempuraj D, 2016, J Neurol Neurosurg Spine, V1

Kim C, 2016, SCI REP-UK, V6, DOI 10.1038/srep30891

Kim CY, 2017, SEMIN NEUROL, V37, P135, DOI 10.1055/s-0037-1601567

Kim WG, 2000, J NEUROSCI, V20, P6309, DOI 10.1523/JNEUROSCI.20-16-06309.2000

Kitazawa M, 2005, J NEUROSCI, V25, P8843, DOI 10.1523/JNEUROSCI.2868-05.2005

L'Episcopo F, 2011, REJUV RES, V14, P411, DOI 10.1089/rej.2010.1134

Labbadia J, 2013, TRENDS BIOCHEM SCI, V38, P378, DOI 10.1016/j.tibs.2013.05.003

Lee DC, 2010, J NEUROINFLAMM, V7, DOI 10.1186/1742-2094-7-56

Lee J, 2002, NEUROMOL MED, V2, P29

Lee JW, 2008, J NEUROINFLAMM, V5, DOI 10.1186/1742-2094-5-37

Lee Sung Joong, 2002, Current Drug Targets - Inflammation and Allergy, V1, P181

Lee SW, 2018, ANIM CELLS SYST, V22, P219, DOI 10.1080/19768354.2018.1473291

LEIGH PN, 1991, BRAIN, V114, P775, DOI 10.1093/brain/114.2.775

Leow-Dyke S, 2012, J NEUROINFLAMM, V9, DOI 10.1186/1742-2094-9-230

Li SH, 2004, TRENDS GENET, V20, P146, DOI 10.1016/j.tig.2004.01.008

Liu J, 2017, FRONT IMMUNOL, V8, DOI 10.3389/fimmu.2017.01005

Liu M, 2011, PARKINSONS DIS-US, V2011, DOI 10.4061/2011/327089

Liu YX, 2008, NEUROTOXICOLOGY, V29, P864, DOI 10.1016/j.neuro.2008.02.014

Lu X, 2000, NEUROSCIENCE, V97, P285, DOI 10.1016/S0306-4522(00)00033-6

Lucin KM, 2009, NEURON, V64, P110, DOI 10.1016/j.neuron.2009.08.039

Lykhmus O, 2016, FRONT MOL NEUROSCI, V9, DOI 10.3389/fnmol.2016.00019

Lykhmus O, 2015, PLOS ONE, V10, DOI 10.1371/journal.pone.0122706

Lyon MS, 2019, MUSCLE NERVE, V59, P10, DOI 10.1002/mus.26289

Ma B, 2016, HUM MOL GENET, V25, P3515, DOI 10.1093/hmg/ddw194

Ma L, 2016, BRAIN RES BULL, V121, P192, DOI 10.1016/j.brainresbull.2016.02.001

MACDONALD ME, 1993, CELL, V72, P971, DOI 10.1016/0092-8674(93)90585-E

Madera-Salcedo IK, 2013, J IMMUNOL, V191, P3400, DOI 10.4049/jimmunol.1202658

Maher A, 2014, BRAIN RES BULL, V109, P109, DOI 10.1016/j.brainresbull.2014.10.007

Malm TM, 2008, GLIA, V56, P1767, DOI 10.1002/glia.20726

Mangano EN, 2009, NEUROBIOL AGING, V30, P1361, DOI 10.1016/j.neurobiolaging.2007.11.020

Marottoli FM, 2017, ASN NEURO, V9, DOI 10.1177/1759091417719201

Mata IF, 2012, MOVEMENT DISORD, V27, P1822, DOI 10.1002/mds.25226

McAlpine FE, 2009, NEUROBIOL DIS, V34, P163, DOI 10.1016/j.nbd.2009.01.006

MCGEER PL, 1988, NEUROLOGY, V38, P1285, DOI 10.1212/WNL.38.8.1285

MCMILLIAN M, 1995, BIOCHEM BIOPH RES CO, V215, P572, DOI 10.1006/bbrc.1995.2503

Meseguer V, 2014, NAT COMMUN, V5, DOI 10.1038/ncomms4125

Miklossy J, 2008, J ALZHEIMERS DIS, V13, P381

Min SS, 2009, BIOCHEM BIOPH RES CO, V383, P93, DOI 10.1016/j.bbrc.2009.03.133

Minghetti L, 2005, BRAIN RES REV, V48, P251, DOI 10.1016/j.brainresrev.2004.12.015

Moehle MS, 2012, J NEUROSCI, V32, P1602, DOI 10.1523/JNEUROSCI.5601-11.2012

Moon HE, 2015, EXP NEUROBIOL, V24, P103, DOI 10.5607/en.2015.24.2.103

MRAK RE, 1995, HUM PATHOL, V26, P816, DOI 10.1016/0046-8177(95)90001-2

Mrdjen D, 2018, IMMUNITY, V48, P380, DOI 10.1016/j.immuni.2018.01.011

Nalls MA, 2011, LANCET, V377, P641, DOI 10.1016/S0140-6736(10)62345-8

Nelson PT, 2012, J NEUROPATH EXP NEUR, V71, P362, DOI 10.1097/NEN.0b013e31825018f7

Ohgomori T, 2016, EUR J NEUROSCI, V43, P1340, DOI 10.1111/ejn.13227

Okun E, 2009, BRAIN RES REV, V59, P278, DOI 10.1016/j.brainresrev.2008.09.001

Ophir G, 2003, NEUROBIOL DIS, V12, P56, DOI 10.1016/S0969-9961(02)00005-0

Park BS, 2013, EXP MOL MED, V45, DOI 10.1038/emm.2013.97

Park JS, 2018, CURR NEUROL NEUROSCI, V18, DOI 10.1007/s11910-018-0829-3

Parkinson J, 2002, J NEUROPSYCH CLIN N, V14, P223, DOI 10.1176/appi.neuropsych.14.2.223

Perry VH, 2004, BRAIN BEHAV IMMUN, V18, P407, DOI 10.1016/j.bbi.2004.01.004

Philippens IH, 2017, J ALZHEIMERS DIS, V55, P101, DOI 10.3233/JAD-160673

Poewe W, 2017, NAT REV DIS PRIMERS, V3, DOI 10.1038/nrdp.2017.13

Qin LY, 2007, GLIA, V55, P453, DOI 10.1002/glia.20467

Qin LY, 2013, GLIA, V61, P855, DOI 10.1002/glia.22479

QUAN N, 1994, J NEUROIMMUNOL, V49, P125, DOI 10.1016/0165-5728(94)90188-0

Ransohoff RM, 2016, SCIENCE, V353, P777, DOI 10.1126/science.aag2590

Renoir T, 2015, J HUNTINGTONS DIS, V4, P297, DOI 10.3233/JHD-150153

Roe AD, 2011, EUR J NEUROSCI, V34, P448, DOI 10.1111/j.1460-9568.2011.07764.x

Rolls A, 2007, NAT CELL BIOL, V9, P1081, DOI 10.1038/ncb1629

Ruano D, 2006, NEUROSCIENCE, V140, P1157, DOI 10.1016/j.neuroscience.2006.02.073

Ruckdeschel K, 2004, J IMMUNOL, V173, P3320, DOI 10.4049/jimmunol.173.5.3320

Sai Y, 2012, NEUROSCI BIOBEHAV R, V36, P2034, DOI 10.1016/j.neubiorev.2012.06.007

Salter MW, 2017, NAT MED, V23, P1018, DOI 10.1038/nm.4397

Schmalz G, 2011, Adv Dent Res, V23, P302, DOI 10.1177/0022034511405391

Sharma N, 2017, INFLAMMOPHARMACOLOGY, V25, P351, DOI 10.1007/s10787-017-0346-z

Sharma N, 2016, MOL NEUROBIOL, V53, P3326, DOI 10.1007/s12035-015-9267-2

Sharma N, 2016, BEHAV BRAIN RES, V296, P177, DOI 10.1016/j.bbr.2015.09.012

Sheng JG, 2003, NEUROBIOL DIS, V14, P133, DOI 10.1016/S0969-9961(03)00069-X

Shi J, 2010, J IMMUNOL, V184, P7207, DOI 10.4049/jimmunol.0903487

Silvestroni A, 2009, NEUROREPORT, V20, P1098, DOI 10.1097/WNR.0b013e32832e34ee

Skelly DT, 2013, PLOS ONE, V8, DOI 10.1371/journal.pone.0069123

Sly LM, 2001, BRAIN RES BULL, V56, P581, DOI 10.1016/S0361-9230(01)00730-4

Small SA, 2011, NAT REV NEUROSCI, V12, P585, DOI 10.1038/nrn3085

Sugars KL, 2003, TRENDS GENET, V19, P233, DOI 10.1016/S0168-9525(03)00074-X

Tak PP, 2001, J CLIN INVEST, V107, P7, DOI 10.1172/JCI11830

Thygesen C, 2018, FRONT CELL NEUROSCI, V12, DOI 10.3389/fncel.2018.00397

Turner Ryan C, 2017, Neuroimmunol Neuroinflamm, V4, P6, DOI 10.20517/2347-8659.2016.40

Valente T, 2013, NEUROBIOL AGING, V34, P2110, DOI 10.1016/j.neurobiolaging.2013.02.007

van Duijn C. M., 2001, American Journal of Human Genetics, V69, P629, DOI 10.1086/322996

Wang D, 2016, CHIN J TRAUMATOL, V19, P193, DOI 10.1016/j.cjtee.2016.06.001

Wang LM, 2018, AM J NUCL MED MOLEC, V8, P86

Wu Dan, 2015, Shengli Xuebao, V67, P319

Wyss-Coray T, 2012, CSH PERSPECT MED, V2, DOI 10.1101/cshperspect.a006346

Xu W, 2017, CELL PHYSIOL BIOCHEM, V44, P2091, DOI 10.1159/000485947

Yang WW, 2017, CELL MOL LIFE SCI, V74, P1485, DOI 10.1007/s00018-016-2411-y

Yang XL, 2018, J BIOMED RES, V32, P136, DOI 10.7555/JBR.32.20170033

Yong VW, 2010, NEUROSCIENTIST, V16, P408, DOI 10.1177/1073858410371379

Yousif NM, 2018, GLIA, V66, P708, DOI 10.1002/glia.23276

Zakaria R, 2017, PHYSIOL RES, V66, P553, DOI 10.33549/physiolres.933480

Zhan XH, 2018, FRONT AGING NEUROSCI, V10, DOI 10.3389/fnagi.2018.00042

Zhan XH, 2016, NEUROLOGY, V87, P2324, DOI 10.1212/WNL.0000000000003391

Zhang DH, 2007, J PHARMACOL EXP THER, V322, P913, DOI 10.1124/jpet.107.124669

Zhang F, 2010, J PHARMACOL EXP THER, V333, P822, DOI 10.1124/jpet.110.165829

Zhang J, 2005, NEUROSCIENCE, V135, P829, DOI 10.1016/j.neuroscience.2005.06.049

Zhang W, 2005, FASEB J, V19, P533, DOI 10.1096/fj.04-2751com

Zhang W, 2018, MOL NEUROBIOL, V55, P619, DOI 10.1007/s12035-016-0308-2

Zhang Z, 2012, MOL MED REP, V5, P562, DOI 10.3892/mmr.2011.671

Zhao YF, 2018, AGING DIS, V9, P785, DOI 10.14336/AD.2017.1028

Zhao YH, 2018, MOL NEUROBIOL, V55, P9100, DOI 10.1007/s12035-018-1015-y

Zhao YH, 2017, FRONT IMMUNOL, V8, DOI 10.3389/fimmu.2017.01064

Zhao YH, 2017, FRONT CELL INFECT MI, V7, DOI 10.3389/fcimb.2017.00318

Zheng HF, 2013, PLOS ONE, V8, DOI 10.1371/journal.pone.0070472

Zhu Y, 2016, INT J MOL SCI, V17, DOI 10.3390/ijms17010025

Zughaier SM, 2005, INFECT IMMUN, V73, P2940, DOI 10.1128/IAI.73.5.2940-2950.2005

NR 199

TC 298

Z9 331

U1 16

U2 78

PU MDPI

PI BASEL

PA ST ALBAN-ANLAGE 66, CH-4052 BASEL, SWITZERLAND

EI 1422-0067

J9 INT J MOL SCI

JI Int. J. Mol. Sci.

PD MAY 1

PY 2019

VL 20

IS 9

AR 2293

DI 10.3390/ijms20092293

PG 31

WC Biochemistry & Molecular Biology; Chemistry, Multidisciplinary

WE Science Citation Index Expanded (SCI-EXPANDED)

SC Biochemistry & Molecular Biology; Chemistry

GA IA7RK

UT WOS:000469753500240

PM 31075861

OA Green Published, Green Submitted, gold

DA 2024-08-12

ER

PT J

AU Sampson, TR

Debelius, JW

Thron, T

Janssen, S

Shastri, GG

Ilhan, ZE

Challis, C

Schretter, CE

Rocha, S

Gradinaru, V

Chesselet, MF

Keshavarzian, A

Shannon, KM

Krajmalnik-Brown, R

Wittung-Stafshede, P

Knight, R

Mazmanian, SK

AF Sampson, Timothy R.

Debelius, Justine W.

Thron, Taren

Janssen, Stefan

Shastri, Gauri G.

Ilhan, Zehra Esra

Challis, Collin

Schretter, Catherine E.

Rocha, Sandra

Gradinaru, Viviana

Chesselet, Marie-Francoise

Keshavarzian, Ali

Shannon, Kathleen M.

Krajmalnik-Brown, Rosa

Wittung-Stafshede, Pernilla

Knight, Rob

Mazmanian, Sarkis K.

TI Gut Microbiota Regulate Motor Deficits and Neuroinflammation in a Model

of Parkinson's Disease

SO CELL

LA English

DT Article

ID CHAIN FATTY-ACIDS; ALPHA-SYNUCLEIN; BRAIN-DEVELOPMENT; GROWTH-FACTOR;

MICROGLIA; BEHAVIOR; SYSTEM; INFLAMMATION; METABOLITES; DIVERSITY

AB The intestinal microbiota influence neurodevelopment, modulate behavior, and contribute to neurological disorders. However, a functional link between gut bacteria and neurodegenerative disease remains unexplored. Synucleinopathies are characterized by aggregation of the protein alpha-synuclein (alpha Syn), often resulting in motor dysfunction as exemplified by Parkinson's disease (PD). Using mice that overexpress aSyn, we report herein that gut microbiota are required for motor deficits, microglia activation, and alpha Syn pathology. Antibiotic treatment ameliorates, while microbial re-colonization promotes, pathophysiology in adult animals, suggesting that postnatal signaling between the gut and the brain modulates disease. Indeed, oral administration of specific microbial metabolites to germ-free mice promotes neuroinflammation and motor symptoms. Remarkably, colonization of alpha Syn-overexpressing mice with microbiota from PD-affected patients enhances physical impairments compared to microbiota transplants from healthy human donors. These findings reveal that gut bacteria regulate movement disorders in mice and suggest that alterations in the human microbiome represent a risk factor for PD.

C1 [Sampson, Timothy R.; Thron, Taren; Shastri, Gauri G.; Challis, Collin; Schretter, Catherine E.; Gradinaru, Viviana; Mazmanian, Sarkis K.] CALTECH, Div Biol & Biol Engn, Pasadena, CA 91125 USA.

[Debelius, Justine W.; Janssen, Stefan; Knight, Rob] Univ Calif San Diego, Dept Pediat, San Diego, CA 92110 USA.

[Ilhan, Zehra Esra; Krajmalnik-Brown, Rosa] Arizona State Univ, Biodesign Inst, Swette Ctr Environm Biotechnol, Tempe, AZ 85287 USA.

[Rocha, Sandra; Wittung-Stafshede, Pernilla] Chalmers Univ Technol, Biol & Biol Engn Dept, S-41296 Gothenburg, Sweden.

[Chesselet, Marie-Francoise] UCLA, Dept Neurol, David Geffen Sch Med, Los Angeles, CA 90095 USA.

[Keshavarzian, Ali] Rush Univ, Div Gastroenterol, Dept Internal Med, Med Ctr, Chicago, IL 60612 USA.

[Shannon, Kathleen M.] Rush Univ, Sect Movement Disorders, Dept Neurol Sci, Med Ctr, Chicago, IL 60612 USA.

[Knight, Rob] Univ Calif San Diego, Dept Comp Sci & Engn, San Diego, CA 92093 USA.

[Shannon, Kathleen M.] Univ Wisconsin, Dept Neurol, Madison, WI 53705 USA.

C3 California Institute of Technology; University of California System;

University of California San Diego; Arizona State University; Arizona

State University-Tempe; Chalmers University of Technology; University of

California System; University of California Los Angeles; University of

California Los Angeles Medical Center; David Geffen School of Medicine

at UCLA; Rush University; Rush University; University of California

System; University of California San Diego; University of Wisconsin

System; University of Wisconsin Madison

RP Sampson, TR; Mazmanian, SK (corresponding author), CALTECH, Div Biol & Biol Engn, Pasadena, CA 91125 USA.

EM trsamps@caltech.edu; sarkis@caltech.edu

RI Mazmanian, Sarkis/ABD-7729-2021; Knight, Rob/D-1299-2010; Debelius,

Justine/ABE-7272-2021; wittung-stafshede, pernilla/AAV-4392-2021;

Sampson, Tim/B-9061-2013; Krajmalnik-Brown, Rosa/neurodegenerative disease-4803-2021; ,

Beloeil/I-5451-2014; Rocha, Sandra/Q-8427-2019; Rocha,

Sandra/G-6677-2013

OI Mazmanian, Sarkis/0000-0003-2713-1513; wittung-stafshede,

pernilla/0000-0003-1058-1964; Challis, Collin/0000-0003-4716-6086;

Sampson, Timothy/0000-0002-2486-8766; Rocha, Sandra/0000-0002-4530-3537;

Knight, Rob/0000-0002-0975-9019; Schretter,

Catherine/0000-0002-3957-6838; Ilhan, Zehra Esra/0000-0002-3387-9424;

Krajmalnik-Brown, Rosa/0000-0001-6064-3524; Shannon,

Kathleen/0000-0001-5171-6779; Janssen, Stefan/0000-0003-0955-0589

FU Caltech Beckman Institute; Arnold and Mabel Beckman Foundation; Knut and

Alice Wallenberg Foundation; Swedish Research Council; NIH [NS085910]

FX We thank E. Hsiao, M. Sampson, and the S.K.M. laboratory for helpful

critiques. We are grateful to K. Ly, A. Maskell, and M. Quintos for

animal husbandry and Y. Garcia-Flores, G. Ackermann, G. Humphrey, and H.

Derderian for technical support. Imaging and analysis was performed in

the Caltech Biological Imaging Facility, with the support of the Caltech

Beckman Institute and the Arnold and Mabel Beckman Foundation. T.R.S. is

a Larry L. Hillblom Foundation postdoctoral fellow. This project was

supported by funds from the Knut and Alice Wallenberg Foundation and

Swedish Research Council to P.W.-S.; a gift from Mrs. and Mr. Larry

Field to A. K.; the Heritage Medical Research Institute to V.G. and

S.K.M.; and NIH grant NS085910 to S.K.M.

CR Beilina A, 2016, J NEUROCHEM, V139, P91, DOI 10.1111/jnc.13266

Bercik P, 2011, GASTROENTEROLOGY, V141, P599, DOI 10.1053/j.gastro.2011.04.052

Braak H, 2003, J NEURAL TRANSM, V110, P517, DOI 10.1007/s00702-002-0808-2

Braniste V, 2014, SCI TRANSL MED, V6, DOI 10.1126/scitranslmed.3009759

Brettschneider J, 2015, NAT REV NEUROSCI, V16, P109, DOI 10.1038/nrn3887

Burke RE, 2008, ANN NEUROL, V64, P485, DOI 10.1002/ana.21541

Caporaso JG, 2010, NAT METHODS, V7, P335, DOI 10.1038/nmeth.f.303

Chesselet MF, 2012, NEUROTHERAPEUTICS, V9, P297, DOI 10.1007/s13311-012-0104-2

Chorell E, 2015, PLOS ONE, V10, DOI 10.1371/journal.pone.0140194

Clarke G, 2013, MOL PSYCHIATR, V18, P666, DOI 10.1038/mp.2012.77

Cleynen I, 2014, GUT, V63, P1265, DOI 10.1136/gutjnl-2012-303205

Del Tredici K, 2008, ACTA NEUROPATHOL, V115, P379, DOI 10.1007/s00401-008-0355-5

Devos D, 2013, NEUROBIOL DIS, V50, P42, DOI 10.1016/j.nbd.2012.09.007

Dinan TG, 2015, CURR OPIN CLIN NUTR, V18, P552, DOI 10.1097/MCO.0000000000000221

Eisenhofer G, 1997, J CLIN ENDOCR METAB, V82, P3864, DOI 10.1210/jc.82.11.3864

Erny D, 2015, NAT NEUROSCI, V18, P965, DOI 10.1038/nn.4030

Fleming SM, 2004, J NEUROSCI, V24, P9434, DOI 10.1523/JNEUROSCI.3080-04.2004

Gao B., 2016, ENV HLTH PERSPECT

Gao HM, 2011, ENVIRON HEALTH PERSP, V119, P807, DOI 10.1289/ehp.1003013

Gilbert JA, 2014, BMC BIOL, V12, DOI 10.1186/s12915-014-0069-1

Hasegawa S, 2015, PLOS ONE, V10, DOI 10.1371/journal.pone.0142164

Heijtza RD, 2011, P NATL ACAD SCI USA, V108, P3047, DOI 10.1073/pnas.1010529108

Hoban AE, 2016, TRANSL PSYCHIAT, V6, DOI 10.1038/tp.2016.42

Holmqvist S, 2014, ACTA NEUROPATHOL, V128, P805, DOI 10.1007/s00401-014-1343-6

Hooper LV, 2012, SCIENCE, V336, P1268, DOI 10.1126/science.1223490

Jenner P, 2008, NAT REV NEUROSCI, V9, P665, DOI 10.1038/nrn2471

Jo EJ, 2000, J BIOL CHEM, V275, P34328, DOI 10.1074/jbc.M004345200

Kannarkat GT, 2013, J PARKINSON DIS, V3, P493, DOI 10.3233/JPD-130250

Keshavarzian A, 2015, MOVEMENT DISORD, V30, P1351, DOI 10.1002/mds.26307

Kim C, 2013, NAT COMMUN, V4, DOI 10.1038/ncomms2534

Klucken J, 2006, ACTA NEUROPATHOL, V111, P101, DOI 10.1007/s00401-005-0027-7

Kohman RA, 2013, BEHAV BRAIN RES, V242, P17, DOI 10.1016/j.bbr.2012.12.032

Kopylova E, 2012, BIOINFORMATICS, V28, P3211, DOI 10.1093/bioinformatics/bts611

Kumar DKV, 2016, SCI TRANSL MED, V8, DOI 10.1126/scitranslmed.aaf1059

Langille MGI, 2013, NAT BIOTECHNOL, V31, P814, DOI 10.1038/nbt.2676

Ley RE, 2006, CELL, V124, P837, DOI 10.1016/j.cell.2006.02.017

Lin R, 2014, J DIGEST DIS, V15, P597, DOI 10.1111/1751-2980.12179

Lozupone C, 2005, APPL ENVIRON MICROB, V71, P8228, DOI 10.1128/AEM.71.12.8228-8235.2005

Mandal Siddhartha, 2015, Microbial Ecology in Health and Disease, V26, P27663, DOI 10.3402/mehd.v26.27663

Matcovitch-Natan O, 2016, SCIENCE, V353, DOI 10.1126/science.aad8670

Mayer EA, 2014, BIOESSAYS, V36, P933, DOI 10.1002/bies.201400075

McDonald D, 2012, ISME J, V6, P610, DOI 10.1038/ismej.2011.139

Minter MR, 2016, SCI REP-UK, V6, DOI 10.1038/srep30028

Möhle L, 2016, CELL REP, V15, P1945, DOI 10.1016/j.celrep.2016.04.074

MOGI M, 1994, NEUROSCI LETT, V180, P147, DOI 10.1016/0304-3940(94)90508-8

MOGI M, 1994, NEUROSCI LETT, V165, P208, DOI 10.1016/0304-3940(94)90746-3

Nalls MA, 2014, NAT GENET, V46, P989, DOI 10.1038/ng.3043

Neufeld KM, 2011, NEUROGASTROENT MOTIL, V23, DOI 10.1111/j.1365-2982.2010.01620.x

Prusiner SB, 2015, P NATL ACAD SCI USA, V112, pE5308, DOI 10.1073/pnas.1514475112

Ritz Beate R, 2016, Curr Environ Health Rep, V3, P40, DOI 10.1007/s40572-016-0083-2

Rockenstein E, 2002, J NEUROSCI RES, V68, P568, DOI 10.1002/jnr.10231

Rooks MG, 2016, NAT REV IMMUNOL, V16, P341, DOI 10.1038/nri.2016.42

Sacchettini JC, 2002, NAT REV DRUG DISCOV, V1, P267, DOI 10.1038/nrd769

Sanchez-Guajardo V, 2013, ASN NEURO, V5, P113, DOI 10.1042/AN20120066

Scheperjans F, 2015, MOVEMENT DISORD, V30, P350, DOI 10.1002/mds.26069

Schroeder B, 2016, NAT MED, V22, P1079, DOI 10.1038/nm.4185

Selkrig J, 2014, GUT MICROBES, V5, P369, DOI 10.4161/gmic.28681

Shannon KM, 2012, MOVEMENT DISORD, V27, P716, DOI 10.1002/mds.25020

Sharon G, 2016, CELL, V167, P915, DOI 10.1016/j.cell.2016.10.027

Smith PM, 2013, SCIENCE, V341, P569, DOI 10.1126/science.1241165

Soldner F, 2016, NATURE, V533, P95, DOI 10.1038/nature17939

Sudo N, 2004, J PHYSIOL-LONDON, V558, P263, DOI 10.1113/jphysiol.2004.063388

Unger MM, 2016, PARKINSONISM RELAT D, V32, P66, DOI 10.1016/j.parkreldis.2016.08.019

Valera E, 2016, MOVEMENT DISORD, V31, P225, DOI 10.1002/mds.26428

Vázquez-Baeza Y, 2013, GIGASCIENCE, V2, DOI 10.1186/2047-217X-2-16

Verbaan D, 2007, NEUROLOGY, V69, P333, DOI 10.1212/01.wnl.0000266593.50534.e8

Wall R, 2014, ADV EXP MED BIOL, V817, P221, DOI 10.1007/978-1-4939-0897-4_10

Walters W, 2016, MSYSTEMS, V1, DOI 10.1128/mSystems.00009-15

Wang L, 2012, NEUROGASTROENT MOTIL, V24, pe425, DOI 10.1111/j.1365-2982.2012.01974.x

Yano JM, 2015, CELL, V161, P264, DOI 10.1016/j.cell.2015.02.047

Zhang JP, 2014, J NEUROGENET, V28, P98, DOI 10.3109/01677063.2014.892486

NR 71

TC 2169

Z9 2451

U1 77

U2 1170

PU CELL PRESS

PI CAMBRIDGE

PA 50 HAMPSHIRE ST, FLOOR 5, CAMBRIDGE, MA 02139 USA

SN 0092-8674

EI 1097-4172

J9 CELL

JI Cell

PD DEC 1

PY 2016

VL 167

IS 6

BP 1469

EP +

DI 10.1016/j.cell.2016.11.018

PG 24

WC Biochemistry & Molecular Biology; Cell Biology

WE Science Citation Index Expanded (SCI-EXPANDED)

SC Biochemistry & Molecular Biology; Cell Biology

GA EE3EN

UT WOS:000389470500011

PM 27912057

OA Green Accepted, hybrid

DA 2024-08-12

ER

PT J

AU Hassanzadeh, K

Rahimmi, A

AF Hassanzadeh, Kambiz

Rahimmi, Arman

TI Oxidative stress and neuroinflammation in the story of Parkinson's

disease: Could targeting these pathways write a good ending?

SO JOURNAL OF CELLULAR PHYSIOLOGY

LA English

DT Review

DE antioxidants; neuroinflammation; oxidative stress; Parkinson's disease

ID NONSTEROIDAL ANTIINFLAMMATORY DRUGS; SUBSTANTIA-NIGRA;

DOPAMINERGIC-NEURONS; N-ACETYLCYSTEINE; CELL-DEATH; RISK; AUTOPHAGY;

INFLAMMATION; NEUROMELANIN; LRRK2

AB Although, current medications for Parkinson's disease can control and relief symptoms of the disease efficiently, they are unable to either prevent progression of the disease or maintain their controlling ability as a long-term medication. To find suitable adjuvant and/or alternative treatments, researchers have investigated antioxidative and anti-inflammatory approaches, since emerging evidence consider oxidative stress and neuroinflammation as leading causes of the development of Parkinson's disease. Here, how oxidative stress and neuroinflammation take part in Parkinson's disease pathogenesis was discussed based on featured studies in this context. Then, preclinical and clinical trial studies, which evaluated antioxidative and anti-inflammatory compounds' ability to treat Parkinson's disease, were reviewed.

C1 [Hassanzadeh, Kambiz] Kurdistan Univ Med Sci, Res Inst Hlth Dev, Cellular & Mol Res Ctr, Sanandaj, Iran.

[Hassanzadeh, Kambiz; Rahimmi, Arman] Kurdistan Univ Med Sci, Physiol & Pharmacol Dept, Sch Med, Sanandaj, Iran.

[Rahimmi, Arman] Kurdistan Univ Med Sci, Student Res Comm, Sanandaj, Iran.

C3 Kurdistan University of Medical Sciences; Kurdistan University of

Medical Sciences; Kurdistan University of Medical Sciences

RP Rahimmi, A (corresponding author), Kurdistan Univ Med Sci, Res Inst Hlth Dev, Cellular & Mol Res Ctr, Sanandaj, Iran.

EM a.rahimmi@yahoo.com

RI Hassanzadeh, Kambiz/Q-1241-2016; Rahimmi, Arman/AAT-5520-2020

OI Hassanzadeh, Kambiz/0000-0002-8737-312X; Rahimmi,

Arman/0000-0003-0720-3487

FU Vice Chancellor's office for Research Affairs of Kurdistan University of

Medical Sciences

FX The authors thank anonymous reviewers who helped in improving the

article by their valuable comments. We also thank the Vice Chancellor's

office for Research Affairs of Kurdistan University of Medical Sciences

for financial support.

CR AMES BN, 1993, P NATL ACAD SCI USA, V90, P7915, DOI 10.1073/pnas.90.17.7915

Anglade P, 1997, HISTOL HISTOPATHOL, V12, P25

[Anonymous], 2014, J MAZANDARAN U MED S

Arai H, 2006, HISTOL HISTOPATHOL, V21, P673, DOI 10.14670/HH-21.673

Arai H, 2004, J BIOL CHEM, V279, P51647, DOI 10.1074/jbc.M407328200

Martínez-Banaclocha MA, 2012, MED HYPOTHESES, V79, P8, DOI 10.1016/j.mehy.2012.03.021

Asanuma M, 2004, CURR PHARM DESIGN, V10, P695, DOI 10.2174/1381612043453072

Atashrazm F, 2016, CLIN PHARMACOL-ADV A, V8, P177, DOI 10.2147/CPAA.S102191

Beal MF, 2014, JAMA NEUROL, V71, P543, DOI 10.1001/jamaneurol.2014.131

Becker C, 2011, EUR J NEUROL, V18, P1336, DOI 10.1111/j.1468-1331.2011.03399.x

Betarbet R, 2000, NAT NEUROSCI, V3, P1301, DOI 10.1038/81834

Blesa J, 2015, FRONT NEUROANAT, V9, DOI 10.3389/fnana.2015.00091

Bornebroek M, 2007, NEUROEPIDEMIOLOGY, V28, P193, DOI 10.1159/000108110

Calabrese V, 2018, FREE RADICAL BIO MED, V115, P80, DOI 10.1016/j.freeradbiomed.2017.10.379

Castillo K, 2013, AUTOPHAGY, V9, P1308, DOI 10.4161/auto.25188

Chen CM, 2007, NUTRITION, V23, P589, DOI 10.1016/j.nut.2007.05.004

Chen HL, 2005, ANN NEUROL, V58, P963, DOI 10.1002/ana.20682

Chen HL, 2003, ARCH NEUROL-CHICAGO, V60, P1059, DOI 10.1001/archneur.60.8.1059

Chen ZH, 2016, J NEUROCHEM, V136, P10, DOI 10.1111/jnc.13062

Chinta SJ, 2018, CELL REP, V22, P930, DOI 10.1016/j.celrep.2017.12.092

Christensen K V, 2017, Prog Med Chem, V56, P37, DOI 10.1016/bs.pmch.2016.11.002

COYLE JT, 1993, SCIENCE, V262, P689, DOI 10.1126/science.7901908

Dauer W, 2003, NEURON, V39, P889, DOI 10.1016/S0896-6273(03)00568-3

Davie CA, 2008, BRIT MED BULL, V86, P109, DOI 10.1093/bmb/ldn013

Double KL, 2002, NEUROTOXICOL TERATOL, V24, P621, DOI 10.1016/S0892-0362(02)00218-0

Ebrahimi SS, 2017, TOXICOL LETT, V276, P108, DOI 10.1016/j.toxlet.2017.05.018

Etminan M, 2008, J CLIN NEUROSCI, V15, P576, DOI 10.1016/j.jocn.2007.02.095

Fedorow H, 2005, PROG NEUROBIOL, V75, P109, DOI 10.1016/j.pneurobio.2005.02.001

Finkel T, 2000, NATURE, V408, P239, DOI 10.1038/35041687

Franco-Iborra S, 2016, NEUROSCIENTIST, V22, P266, DOI 10.1177/1073858415574600

Fu SP, 2015, J NEUROINFLAMM, V12, DOI 10.1186/s12974-014-0230-3

Fujita KA, 2014, MOL NEUROBIOL, V49, P88, DOI 10.1007/s12035-013-8489-4

Gagne JJ, 2010, NEUROLOGY, V74, P995, DOI 10.1212/WNL.0b013e3181d5a4a3

Gao XA, 2011, NEUROLOGY, V76, P863, DOI 10.1212/WNL.0b013e31820f2d79

Gardet A, 2010, J IMMUNOL, V185, P5577, DOI 10.4049/jimmunol.1000548

Giordano S, 2014, REDOX BIOL, V2, P82, DOI 10.1016/j.redox.2013.12.013

Glass CK, 2010, CELL, V140, P918, DOI 10.1016/j.cell.2010.02.016

Guerreiro PS, 2016, MOL NEUROBIOL, V53, P3124, DOI 10.1007/s12035-015-9209-z

Gupta A, 2008, ANN NEUROL, V64, pS3, DOI 10.1002/ana.21573

Hirsch EC, 1998, ANN NEUROL, V44, pS115, DOI 10.1002/ana.410440717

Hirsch EC, 2009, LANCET NEUROL, V8, P382, DOI 10.1016/S1474-4422(09)70062-6

Hwang Onyou, 2013, Exp Neurobiol, V22, P11, DOI 10.5607/en.2013.22.1.11

Irrcher I, 2010, HUM MOL GENET, V19, P3734, DOI 10.1093/hmg/ddq288

Jackson-Lewis V, 2007, NAT PROTOC, V2, P141, DOI 10.1038/nprot.2006.342

Jankovic J, 2008, J NEUROL NEUROSUR PS, V79, P368, DOI 10.1136/jnnp.2007.131045

Jenner P, 2003, ANN NEUROL, V53, pS26, DOI 10.1002/ana.10483

Jin H, 2014, BBA-MOL BASIS DIS, V1842, P1282, DOI 10.1016/j.bbadis.2013.09.007

Karlsson O, 2013, J NEURAL TRANSM, V120, P1623, DOI 10.1007/s00702-013-1062-5

Khazdair MR, 2015, J TOXICOL-US, V2015, DOI 10.1155/2015/841823

Kitamura Y., 2009, CURR ANAESTHESIA CRI, V20, P142, DOI [10.1016/j.cacc.2008.12.007, DOI 10.1016/J.CACC.2008.12.007]

Kordower JH, 2000, SCIENCE, V290, P767, DOI 10.1126/science.290.5492.767

LANGSTON JW, 1983, SCIENCE, V219, P979, DOI 10.1126/science.6823561

Lee J, 2012, BIOCHEM J, V441, P523, DOI 10.1042/BJ20111451

Lehmann JM, 1997, J BIOL CHEM, V272, P3406, DOI 10.1074/jbc.272.6.3406

Lim KL, 2007, BMC BIOCHEM, V8, DOI 10.1186/1471-2091-8-S1-S13

de Maturana RL, 2016, J NEUROINFLAMM, V13, DOI 10.1186/s12974-016-0761-x

Malagelada C, 2010, J NEUROSCI, V30, P1166, DOI 10.1523/JNEUROSCI.3944-09.2010

Manthripragada AD, 2011, NEUROEPIDEMIOLOGY, V36, P155, DOI 10.1159/000325653

Martínez M, 1999, LIFE SCI, V64, P1253, DOI 10.1016/S0024-3205(98)00472-X

Mccoy MK, 2006, J NEUROSCI, V26, P9365, DOI 10.1523/JNEUROSCI.1504-06.2006

MCGEER PL, 1988, NEUROLOGY, V38, P1285, DOI 10.1212/WNL.38.8.1285

Mogi M, 2000, J NEURAL TRANSM, V107, P335, DOI 10.1007/s007020050028

Mortiboys H, 2008, ANN NEUROL, V64, P555, DOI 10.1002/ana.21492

Moussaud S, 2014, MOL NEURODEGENER, V9, DOI 10.1186/1750-1326-9-43

OGAWA N, 1994, EUR NEUROL, V34, P20, DOI 10.1159/000119538

Olanow CW, 2006, LANCET NEUROL, V5, P1013, DOI 10.1016/S1474-4422(06)70602-0

Pan T, 2009, NEUROSCIENCE, V164, P541, DOI 10.1016/j.neuroscience.2009.08.014

Parkinson J, 2002, J NEUROPSYCH CLIN N, V14, P223, DOI 10.1176/appi.neuropsych.14.2.223

Perier C, 2012, ANTIOXID REDOX SIGN, V16, P883, DOI 10.1089/ars.2011.4074

Powers KM, 2008, MOVEMENT DISORD, V23, P88, DOI 10.1002/mds.21782

Prasad KN, 1999, J AM COLL NUTR, V18, P413, DOI 10.1080/07315724.1999.10718878

Rahimmi A., 2014, Journal of Isfahan Medical School, V32, P1250

Rahimmi A, 2015, BRAIN RES BULL, V113, P34, DOI 10.1016/j.brainresbull.2015.02.007

Ramesh G., 2013, MEDIAT INFLAMM, V2013

Rascol O, 2003, ANN NEUROL, V53, pS3, DOI 10.1002/ana.10513

Riederer P, 2004, CURR MED CHEM, V11, P2033, DOI 10.2174/0929867043364775

Rocha NP, 2015, BIOMED RES INT, V2015, DOI 10.1155/2015/628192

Rodríguez-Navarro JA, 2010, NEUROBIOL DIS, V39, P423, DOI 10.1016/j.nbd.2010.05.014

Russo I, 2015, J NEUROINFLAMM, V12, DOI 10.1186/s12974-015-0449-7

Russo I, 2014, J NEUROINFLAMM, V11, DOI 10.1186/1742-2094-11-52

Samii A, 2009, DRUG AGING, V26, P769, DOI 10.2165/11316780-000000000-00000

Segura-Aguilar J, 2014, J NEUROCHEM, V129, P898, DOI 10.1111/jnc.12686

Shih RH, 2015, FRONT MOL NEUROSCI, V8, DOI 10.3389/fnmol.2015.00077

Shimoji M, 2009, NEUROTOX RES, V16, P318, DOI 10.1007/s12640-009-9076-3

Suchowersky O, 2006, NEUROLOGY, V66, P976, DOI 10.1212/01.wnl.0000206363.57955.1b

Surace MJ, 2012, CELL MOL LIFE SCI, V69, P2409, DOI 10.1007/s00018-012-1015-4

Tansey MG, 2010, NEUROBIOL DIS, V37, P510, DOI 10.1016/j.nbd.2009.11.004

Taymans JM, 2016, CURR NEUROPHARMACOL, V14, P214, DOI 10.2174/1570159X13666151030102847

Ton TG, 2006, MOVEMENT DISORD, V21, P964, DOI 10.1002/mds.20856

Tufekci KU, 2012, ADV PROTEIN CHEM STR, V88, P69, DOI 10.1016/B978-0-12-398314-5.00004-0

Uttara B, 2009, CURR NEUROPHARMACOL, V7, P65, DOI 10.2174/157015909787602823

Wahner AD, 2007, NEUROLOGY, V69, P1836, DOI 10.1212/01.wnl.0000279519.99344.ad

Wang HL, 2011, BBA-MOL BASIS DIS, V1812, P674, DOI 10.1016/j.bbadis.2011.03.007

WEINER RI, 1978, PHYSIOL REV, V58, P905, DOI 10.1152/physrev.1978.58.4.905

Xiong N, 2011, NEUROSCIENCE, V199, P292, DOI 10.1016/j.neuroscience.2011.10.031

Yacoubian TA, 2009, BBA-MOL BASIS DIS, V1792, P676, DOI 10.1016/j.bbadis.2008.09.009

Zecca L, 2008, J NEUROCHEM, V106, P1866, DOI 10.1111/j.1471-4159.2008.05541.x

Zucca FA, 2014, NEUROTOX RES, V25, P13, DOI 10.1007/s12640-013-9435-y

NR 98

TC 86

Z9 92

U1 3

U2 44

PU WILEY

PI HOBOKEN

PA 111 RIVER ST, HOBOKEN 07030-5774, NJ USA

SN 0021-9541

EI 1097-4652

J9 J CELL PHYSIOL

JI J. Cell. Physiol.

PD JAN

PY 2019

VL 234

IS 1

BP 23

EP 32

DI 10.1002/jcp.26865

PG 10

WC Cell Biology; Physiology

WE Science Citation Index Expanded (SCI-EXPANDED)

SC Cell Biology; Physiology

GA HC1BG

UT WOS:000451533300002

PM 30078201

OA Bronze

DA 2024-08-12

ER

PT J

AU Sian-Hülsmann, J

AF Sian-Hulsmann, Jeswinder

TI Wilful pathogens provoke a gut feeling in Parkinson's disease

SO JOURNAL OF NEURAL TRANSMISSION

LA English

DT Review

DE Parkinson's disease; Substantia nigra; Cytotoxic pathways; Inflammation;

Pathogens and oxidative stress

ID ALPHA-SYNUCLEIN; HELICOBACTER-PYLORI; OXIDATIVE STRESS; LEWY BODY;

NEUROINFLAMMATION; ASSOCIATION; MICROGLIA

AB Parkinson's disease is the second most common neurological disorder marked by characteristic poverty and dysfunction in movement. There are many mechanisms and factors which have been postulated to be associated with the neurodegenerative pathway(s) resulting in distinctive loss of neurons in the substantia nigra. Subsequently, the neuropathology is more widespread and exhibited in other areas of the brain, and enteric nervous system. Aggregates of misfolded alpha-synuclein or Lewy bodies are the hallmark of the illness and appear to be central in the whole cascade of cell destruction. There are many processes implicated in neuronal destruction including: oxidative stress, excitotoxicity, mitochondrial dysfunction, an imbalance in protein homeostasis and neuroinflammation. Interesting, inflammation induced by pathogens (including, bacteria and viruses) has been associated in the pathogenesis of the disease. Bacteria such as Helicobacter pylori and Helicobacter suis appear to colonise the gut, and elicit systemic immune responses, which is them transmitted via the gut-axis to the brain, where cytotoxic cytokines induce neuroinflammation and cell death. This conforms to the bottom-top hypothesis proposed by Braak. The gut is also implicated in two other theories postulated in the development and progression of the disorder, namely, the top-down and the threshold. There is a possibility that these theories may be inter-linked and operate together to certain degree. Ultimately specific trigger factors or conditions may govern the occurrences of these processes in genetically predisposed individuals. Nevertheless, the importance of pathogen-related gut infections cannot be overlooked, since it can result in dysbiosis of gut microbes, which may orchestrate alpha-synuclein pathology and eventually cell destruction.

C1 [Sian-Hulsmann, Jeswinder] Univ Nairobi, Dept Med Physiol, POB 30197, Nairobi 00100, Kenya.

C3 University of Nairobi

RP Sian-Hülsmann, J (corresponding author), Univ Nairobi, Dept Med Physiol, POB 30197, Nairobi 00100, Kenya.

EM j.sian@germanmedicalcenter.net

CR Al-Bachari S, 2020, FRONT PHYSIOL, V11, DOI 10.3389/fphys.2020.593026

Barnum CJ, 2012, CURR NEUROL NEUROSCI, V12, P350, DOI 10.1007/s11910-012-0283-6

Braak H, 2006, NEUROSCI LETT, V396, P67, DOI 10.1016/j.neulet.2005.11.012

Çamci G, 2016, J CLIN NEUROL, V12, P147, DOI 10.3988/jcn.2016.12.2.147

Campbell MC, 2020, ANN CLIN TRANSL NEUR, V7, P1272, DOI 10.1002/acn3.51102

Chao YX, 2014, BIOMED RES INT, V2014, DOI 10.1155/2014/308654

Colucci M, 2012, AUTON NEUROSCI-BASIC, V169, P77, DOI 10.1016/j.autneu.2012.04.005

Dardiotis E, 2018, CLIN NEUROL NEUROSUR, V175, P16, DOI 10.1016/j.clineuro.2018.09.039

De Pablo-Fernandez E, 2019, JAMA NEUROL, V76, P470, DOI 10.1001/jamaneurol.2018.4377

DEXTER DT, 1994, ANN NEUROL, V35, P38, DOI 10.1002/ana.410350107

Engelender S, 2017, TRENDS NEUROSCI, V40, P4, DOI 10.1016/j.tins.2016.10.008

EUGENIN E, 2010, HIVAIDS RES PALLIATI, DOI DOI 10.2147/HIV.S9186

Foffani G, 2018, NEURON, V99, P1116, DOI 10.1016/j.neuron.2018.07.028

Gao HM, 2008, J NEUROSCI, V28, P7687, DOI 10.1523/JNEUROSCI.0143-07.2008

Gao LB, 2009, CARCINOGENESIS, V30, P295, DOI 10.1093/carcin/bgn281

GIBB WRG, 1988, J NEUROL NEUROSUR PS, V51, P745, DOI 10.1136/jnnp.51.6.745

Gomez-Nicola D, 2015, NEUROSCIENTIST, V21, P169, DOI 10.1177/1073858414530512

Gorlé N, 2021, FRONT IMMUNOL, V11, DOI 10.3389/fimmu.2020.584165

Hirsch Etienne C, 2012, Parkinsonism Relat Disord, V18 Suppl 1, pS210, DOI 10.1016/S1353-8020(11)70065-7

Jenner, 1991, BRIT J PHARMACOL, V104, P281

Kettenmann H, 2011, PHYSIOL REV, V91, P461, DOI 10.1152/physrev.00011.2010

Koutsilieri E, 2002, J NEURAL TRANSM, V109, P767, DOI 10.1007/s007020200063

Lanas A, 2017, LANCET, V390, P613, DOI 10.1016/S0140-6736(16)32404-7

Leclair-Visonneau L, 2020, NEUROGASTROENT MOTIL, V32, DOI 10.1111/nmo.13777

Lee HJ, 2008, INT J BIOCHEM CELL B, V40, P1835, DOI 10.1016/j.biocel.2008.01.017

Lehnardt S, 2010, GLIA, V58, P253, DOI 10.1002/glia.20928

Limphaibool N, 2019, FRONT NEUROL, V10, DOI 10.3389/fneur.2019.00652

Liu BJ, 2017, NEUROLOGY, V88, P1996, DOI 10.1212/WNL.0000000000003961

Liu YP, 2017, FRONT IMMUNOL, V8, DOI 10.3389/fimmu.2017.00860

Mcgee DJ, 2018, J PARKINSON DIS, V8, P367, DOI 10.3233/JPD-181327

MCGEER PL, 1988, NEUROLOGY, V38, P1285, DOI 10.1212/WNL.38.8.1285

Mount MP, 2007, J NEUROSCI, V27, P3328, DOI 10.1523/JNEUROSCI.5321-06.2007

Nutt JG, 2016, MOVEMENT DISORD, V31, P957, DOI 10.1002/mds.26657

O'Donovan SM, 2020, NEUROGASTROENT MOTIL, V32, DOI 10.1111/nmo.13726

Pang SYY, 2019, TRANSL NEURODEGENER, V8, DOI 10.1186/s40035-019-0165-9

Phillips RJ, 2009, EXP NEUROL, V220, P109, DOI 10.1016/j.expneurol.2009.07.025

Przuntek H, 2004, J NEURAL TRANSM, V111, P201, DOI 10.1007/s00702-003-0102-y

RIEDERER P, 1989, J NEUROCHEM, V52, P515, DOI 10.1111/j.1471-4159.1989.tb09150.x

Rochette L, 2013, PHARMACOL THERAPEUT, V140, P239, DOI 10.1016/j.pharmthera.2013.07.004

SCHAPIRA AHV, 1989, LANCET, V1, P1269, DOI 10.1016/S0140-6736(89)92366-0

Sian-Hulsmann J, 2021, CELLS-BASEL, V10, DOI 10.3390/cells10030598

Sian-Hulsmann J, 2020, J NEURAL TRANSM, V127, P749, DOI 10.1007/s00702-020-02192-0

Stolzenberg E, 2017, J INNATE IMMUN, V9, P456, DOI 10.1159/000477990

Testerman TL, 2014, WORLD J GASTROENTERO, V20, P12781, DOI 10.3748/wjg.v20.i36.12781

van Vuuren MJ, 2021, BIOMOLECULES, V11, DOI 10.3390/biom11010030

Wu J, 2020, NITRIC OXIDE-BIOL CH, V102, P39, DOI 10.1016/j.niox.2020.06.002

Yamaoka Y, 2008, J MED MICROBIOL, V57, P545, DOI 10.1099/jmm.0.2008/000570-0

NR 47

TC 2

Z9 2

U1 0

U2 6

PU SPRINGER WIEN

PI WIEN

PA SACHSENPLATZ 4-6, PO BOX 89, A-1201 WIEN, AUSTRIA

SN 0300-9564

EI 1435-1463

J9 J NEURAL TRANSM

JI J. Neural Transm.

PD JUN

PY 2022

VL 129

IS 5-6

SI SI

BP 557

EP 562

DI 10.1007/s00702-021-02448-3

EA DEC 2021

PG 6

WC Clinical Neurology; Neurosciences

WE Science Citation Index Expanded (SCI-EXPANDED)

SC Neurosciences & Neurology

GA 2A5VQ

UT WOS:000731511800001

PM 34923593

OA Bronze, Green Published

DA 2024-08-12

ER

PT J

AU Niranjan, R

AF Niranjan, Rituraj

TI Recent advances in the mechanisms of neuroinflammation and their roles

in neurodegeneration

SO NEUROCHEMISTRY INTERNATIONAL

LA English

DT Review

DE Neuroinflammation; Monocytes and T cells; Inflammatory mediators; Matrix

mettaloproteinases; Autophagy

ID BLOOD-BRAIN-BARRIER; SPINAL-CORD-INJURY; REGULATORY T-CELLS; MOUSE

MODEL; MATRIX METALLOPROTEINASES; THERAPEUTIC TARGETS;

parkinson's disease; EPIGENETIC REGULATION; DIABETIC-RETINOPATHY;

UP-REGULATION

AB Neuroinflammation is associated with the pathogenesis of many neurological disorders including Parkinson's disease, Alzheimer's disease, Amyotrophic lateral sclerosis and Huntington disease. Current studies in this area have advanced the mechanism of neuroinflammation and its role in neurodegeneration. Studies from epidemiologic, clinical and animal models also contributed in the various new mechanisms of neuroinflammation. In this line, activation of monocytes is an important emerging mechanism that has a, profound role in neuroinflammation and neurodegeneration. Ion channels, matrix metalloproteases and microRNAs are also found to be the key players in the pathogenesis of neuroinflammation. In particular, microRNA-32 regulates microglia-mediated neuroinflammation and thus neurodegeneration. Notably, some important studies describe the role of Th17 cells in neuroinflammation, but, very little knowledge is available about their mechanism of action. Particularly, the role of autophagy gets emphasized, which plays a very critical role in protein aggregation and neurodegeneration. In this review, we highlight and discuss the mechanisms of these mediators of inflammation by which they contribute to the disease progression. In conclusion, we focus on the various newer molecular mechanisms that are associated with the basic understanding of neuroinflammation in neurodegeneration.

C1 [Niranjan, Rituraj] ICMR Vector Control Res Ctr, Unit Microbiol & Mol Biol, ICMR TSS, Pondicherry 605006, India.

C3 Indian Council of Medical Research (ICMR); ICMR - Vector Control

Research Center (VCRC)

RP Niranjan, R (corresponding author), ICMR Vector Control Res Ctr, Unit Microbiol & Mol Biol, ICMR TSS, Pondicherry 605006, India.

EM riturajniranjan@rediffmail.com

FU SERB (DST, India) [SB/YS/LS-198/2014]

FX The "young scientist grant No. SB/YS/LS-198/2014 to Dr. Rituraj Niranjan

by SERB (DST, India.) is gratefully acknowledged. The valuable

suggestions provided by Dr. Purushothaman Jambulingam at ICMR-Vector

Control Research Center, Puducherry are also gratefully acknowledged.

CR Adalid-Peralta L, 2012, J PARASITOL, V98, P142, DOI 10.1645/GE-2839.1

Adzemovic MZ, 2012, PLOS ONE, V7, DOI 10.1371/journal.pone.0039794

Agostinho P, 2010, CURR PHARM DESIGN, V16, P2766, DOI 10.2174/138161210793176572

Agrawal SM, 2011, BBA-MOL BASIS DIS, V1812, P213, DOI 10.1016/j.bbadis.2010.07.018

Ahmed ME, 2017, J ALZHEIMERS DIS, V60, P1143, DOI 10.3233/JAD-170634

Aïd S, 2011, BIOCHIMIE, V93, P46, DOI 10.1016/j.biochi.2010.09.009

Ajami B, 2011, NAT NEUROSCI, V14, P1142, DOI 10.1038/nn.2887

Aktas O, 2007, ARCH NEUROL-CHICAGO, V64, P185, DOI 10.1001/archneur.64.2.185

Alcendor DJ, 2012, J NEUROINFLAMM, V9, DOI 10.1186/1742-2094-9-95

Alexander JK, 2009, PROG BRAIN RES, V175, P125, DOI 10.1016/S0079-6123(09)17508-8

Ali S, 2014, EXP NEUROL, V261, P654, DOI 10.1016/j.expneurol.2014.08.017

Allaman I, 2011, TRENDS NEUROSCI, V34, P76, DOI 10.1016/j.tins.2010.12.001

Altman BJ, 2009, AUTOPHAGY, V5, P569, DOI 10.4161/auto.5.4.8254

Anglade P, 1997, HISTOL HISTOPATHOL, V12, P25

Appel SH, 2009, J CLIN INVEST, V119, P13, DOI 10.1172/JCI38096

Bailey ZS, 2016, FRONT MOL NEUROSCI, V9, DOI 10.3389/fnmol.2016.00064

Berson A, 2017, CURR BIOL, V27, P3579, DOI 10.1016/j.cub.2017.10.024

Bie B., NAT NEUROSCI, V17, P223

Böttger D, 2010, BRAIN RES, V1312, P108, DOI 10.1016/j.brainres.2009.11.062

Bosch ME, 2015, FRONT NEUROSCI-SWITZ, V9, DOI 10.3389/fnins.2015.00417

Bowman LR, 2016, PLOS NEGLECT TROP D, V10, DOI 10.1371/journal.pntd.0004551

Brkic M, 2015, MEDIAT INFLAMM, V2015, DOI 10.1155/2015/620581

Brown PN, 2013, CHEM COMMUN, V49, P4415, DOI 10.1039/c2cc36540e

Candelario-Jalil E, 2011, STROKE, V42, P1345, DOI 10.1161/STROKEAHA.110.600825

Cashman JR, 2012, NEURODEGENER DIS, V10, P274, DOI 10.1159/000333123

Chiu CC, 2016, J NEUROSCI METH, V272, P38, DOI 10.1016/j.jneumeth.2016.06.018

Christopher MA, 2017, EPIGENET CHROMATIN, V10, DOI 10.1186/s13072-017-0150-4

Connolly C, 2016, NEUROSCIENCE, V325, P74, DOI 10.1016/j.neuroscience.2016.03.031

Cordaro M, 2017, MOL NEUROBIOL, V54, P2415, DOI 10.1007/s12035-016-9827-0

Côté M, 2015, BRAIN BEHAV IMMUN, V46, P154, DOI 10.1016/j.bbi.2015.01.009

Curran C. S., J NEUROINFLAMMATION, V9, P11

Datta M, 2018, IMMUNITY, V48, P514, DOI 10.1016/j.immuni.2018.02.016

Viegas ATD, 2017, CURR PHARM DESIGN, V23, P669, DOI [10.2174/1381612823666170111, 10.2174/1381612823666170111094702]

De Filippis L, 2016, MOL BRAIN, V9, DOI 10.1186/s13041-016-0221-7

Dehghani R, 2018, REV NEUROSCIENCE, V29, P161, DOI 10.1515/revneuro-2017-0042

Delgado-Morales R, 2017, CLIN EPIGENETICS, V9, DOI 10.1186/s13148-017-0365-z

Denney L, 2012, J IMMUNOL, V189, P551, DOI 10.4049/jimmunol.1103608

Dong H, 2016, CELL MOL NEUROBIOL, V36, P639, DOI 10.1007/s10571-015-0244-0

Dong L, 2016, CNS NEUROSCI THER, V22, P661, DOI 10.1111/cns.12557

Downes CE, 2010, BRIT J PHARMACOL, V160, P1872, DOI 10.1111/j.1476-5381.2010.00864.x

Doyle KM, 2011, J CELL MOL MED, V15, P2025, DOI 10.1111/j.1582-4934.2011.01374.x

Drake C, 2011, BRAIN BEHAV IMMUN, V25, P1113, DOI 10.1016/j.bbi.2011.02.008

Duarte-Neves Joana, 2015, Hum Mol Genet, V24, P5451, DOI 10.1093/hmg/ddv271

Ellrichmann G., J NEUROINFLAMMATION, V9, P15

Fernández-Ruiz J, 2013, BRIT J CLIN PHARMACO, V75, P323, DOI 10.1111/j.1365-2125.2012.04341.x

Ferrante Margherita, 2017, Microrna, V6, P157, DOI 10.2174/2211536606666170811151503

Florczak-Rzepka M, 2012, CEREBROVASC DIS, V34, P249, DOI 10.1159/000341686

Fujimoto S, 2011, BIOCHEM BIOPH RES CO, V404, P223, DOI 10.1016/j.bbrc.2010.11.097

Garden GA, 2013, NEUROTHERAPEUTICS, V10, P782, DOI 10.1007/s13311-013-0207-4

Gawali NB, 2016, PHARMACOL BIOCHEM BE, V149, P1, DOI 10.1016/j.pbb.2016.07.004

Giaume C., 2017, NEUROSCI LETT

Gjoneska E, 2015, NATURE, V518, P365, DOI 10.1038/nature14252

González H, 2014, J NEUROINFLAMM, V11, DOI 10.1186/s12974-014-0201-8

Griñán-Ferré C, 2018, J ALZHEIMERS DIS, V62, P943, DOI 10.3233/JAD-170664

Guan Y, 2018, LIFE SCI, V202, P1, DOI 10.1016/j.lfs.2018.03.031

Guo ML, 2016, EPIGENETICS-US, V11, P819, DOI 10.1080/15592294.2016.1232233

Hajieva P, 2018, NEUROSCI LETT, V663, P29, DOI 10.1016/j.neulet.2017.09.033

Haorah J, 2008, GLIA, V56, P78, DOI 10.1002/glia.20596

Harms AS, 2014, MOVEMENT DISORD, V29, P1242, DOI 10.1002/mds.25993

Harms AS, 2018, EXP NEUROL, V300, P179, DOI 10.1016/j.expneurol.2017.11.010

He D, 2017, BIOCHEM BIOPH RES CO, V490, P941, DOI 10.1016/j.bbrc.2017.06.144

Hendriks JJA, 2005, BRAIN RES REV, V48, P185, DOI 10.1016/j.brainresrev.2004.12.008

Hinault MP, 2011, NEURODEGENER DIS, V8, P397, DOI 10.1159/000324514

Hoffman WH, 2012, EXP MOL PATHOL, V93, P273, DOI 10.1016/j.yexmp.2011.10.007

Hojati Z, 2017, ADV EXP MED BIOL, V958, P65, DOI 10.1007/978-3-319-47861-6_6

Holmoy T, 2008, EUR J NEUROL, V15, P360, DOI 10.1111/j.1468-1331.2008.02065.x

Hossain MM, 2017, TOXICOL SCI, V155, P112, DOI 10.1093/toxsci/kfw187

Hui CW, 2016, PLOS ONE, V11, DOI 10.1371/journal.pone.0147134

Irrcher I, 2009, SCI SIGNAL, V2, DOI 10.1126/scisignal.265pe21

Ji J, 2018, AGING CELL, V17, DOI 10.1111/acel.12774

Jia F, 2014, J NEUROTRAUM, V31, P1225, DOI 10.1089/neu.2013.3230

Jimenez-Pacheco A, 2017, ADV EXP MED BIOL, V978, P255, DOI 10.1007/978-3-319-53889-1_14

Jones KA, 2018, BRAIN BEHAV IMMUN, V67, P299, DOI 10.1016/j.bbi.2017.09.006

Kadiu I, 2005, NEUROTOX RES, V8, P25, DOI 10.1007/BF03033818

Kadlubowska J, 2016, CURR NEUROPHARMACOL, V14, P831, DOI 10.2174/1570159X14666160614095559

Karlmark KR, 2012, EUR J MICROBIOL IMMU, V2, P97, DOI 10.1556/EuJMI.2.2012.2.1

Karthikeyan A, 2016, CURR MED CHEM, V23, P3528, DOI 10.2174/0929867323666160814001040

Kaut O, 2017, NEUROGENETICS, V18, P7, DOI 10.1007/s10048-016-0497-x

Khoshnam SE, 2017, J NEUROPATH EXP NEUR, V76, P548, DOI 10.1093/jnen/nlx036

Kiguchi N, 2013, CYTOKINE, V64, P666, DOI 10.1016/j.cyto.2013.09.019

Kiguchi N, 2012, J PHARMACOL EXP THER, V340, P577, DOI 10.1124/jpet.111.187724

Kim CF, 2011, J PAIN, V12, P370, DOI 10.1016/j.jpain.2010.08.003

King IL, 2009, BLOOD, V113, P3190, DOI 10.1182/blood-2008-07-168575

Kohman Rachel A, 2012, Methods Mol Biol, V934, P193, DOI 10.1007/978-1-62703-071-7_11

Kozela E, 2011, BRIT J PHARMACOL, V163, P1507, DOI 10.1111/j.1476-5381.2011.01379.x

Kumar P, 2016, ADV PROTEIN CHEM STR, V103, P97, DOI 10.1016/bs.apcsb.2015.10.006

Lambert J, 2011, EVID-BASED COMPL ALT, V2011

Lee M, 2011, BRAIN RES, V1369, P21, DOI 10.1016/j.brainres.2010.10.084

Lewis CA, 2012, NEUROL RES INT, V2012, DOI 10.1155/2012/803701

Li DS, 2018, HUM CELL, V31, P106, DOI 10.1007/s13577-017-0187-5

Li XX, 2017, J NEUROENG REHABIL, V14, DOI 10.1186/s12984-016-0212-z

Liang PZ, 2015, NEUROSCI BULL, V31, P435, DOI 10.1007/s12264-015-1545-5

Lim HW, 2015, BRAIN RES BULL, V112, P25, DOI 10.1016/j.brainresbull.2015.01.004

Little J. P., J ALZHEIMERS DIS S2, V30, pS179

Liu HQ, 2012, J NEUROINFLAMM, V9, DOI 10.1186/1742-2094-9-119

Liu Y, 2013, NEUROBIOL AGING, V34, P1231, DOI 10.1016/j.neurobiolaging.2012.10.015

López-León M, 2017, AGEING RES REV, V40, P168, DOI 10.1016/j.arr.2017.09.002

Lopez-Ramirez MA, 2014, FASEB J, V28, P2551, DOI 10.1096/fj.13-248880

Lyck R, 2012, J VASC RES, V49, P497, DOI 10.1159/000341232

Machado A, 2011, PARKINSONS DIS-US, V2011, DOI 10.4061/2011/393769

Mairuae N, 2011, NEUROSCI LETT, V500, P36, DOI 10.1016/j.neulet.2011.06.001

Man SM, 2012, SCI TRANSL MED, V4, DOI 10.1126/scitranslmed.3003197

Mao SS, 2016, J NEUROINFLAMM, V13, DOI 10.1186/s12974-016-0685-5

Marchalant Y, 2009, NEUROBIOL DIS, V34, P300, DOI 10.1016/j.nbd.2009.01.014

Martins D, 2017, DIS MODEL MECH, V10, P1253, DOI 10.1242/dmm.028571

Massa MG, 2017, AM J PATHOL, V187, P1613, DOI 10.1016/j.ajpath.2017.03.006

Mateen BA, 2017, J NEUROTRAUM, V34, P2379, DOI 10.1089/neu.2017.5007

McKiernan RC, 2012, PLOS ONE, V7, DOI 10.1371/journal.pone.0035921

Medeiros MS, 2016, PLOS ONE, V11, DOI 10.1371/journal.pone.0146129

Mishra R, 2012, J NEUROINFLAMM, V9, DOI 10.1186/1742-2094-9-131

Molnarfi N, 2012, PLOS ONE, V7, DOI 10.1371/journal.pone.0049882

Muneer PMA, 2012, ARTERIOSCL THROM VAS, V32, P1167, DOI 10.1161/ATVBAHA.112.247668

Munoz P, 2012, PARKINSONS DIS-US, DOI [DOI 10.1155/2012/920953, 10.1155/2012/920953]

Naaldijk YM, 2016, BIOL CHEM, V397, P283, DOI 10.1515/hsz-2015-0257

Neal M, 2018, BBA-MOL BASIS DIS, V1864, P432, DOI 10.1016/j.bbadis.2017.11.004

Nicolia V, 2017, J NEUROPATH EXP NEUR, V76, P27, DOI 10.1093/jnen/nlw099

Niranjan R, 2018, MOL NEUROBIOL, V55, P8038, DOI 10.1007/s12035-018-0950-y

Niranjan R, 2017, FRONT IMMUNOL, V8, DOI 10.3389/fimmu.2017.00763

Niranjan R, 2016, INDIAN J GASTROENTER, V35, P72, DOI 10.1007/s12664-016-0629-0

Niranjan R, 2014, NEUROL SCI, V35, P409, DOI 10.1007/s10072-013-1530-6

Niranjan R, 2014, MOL NEUROBIOL, V49, P28, DOI 10.1007/s12035-013-8483-x

Niranjan R, 2013, MOL NEUROBIOL, V48, P412, DOI 10.1007/s12035-013-8428-4

Noori-Zadeh A, 2017, J NEUROL SCI, V375, P203, DOI 10.1016/j.jns.2017.01.060

Nuttall RK, 2007, GLIA, V55, P516, DOI 10.1002/glia.20478

Omran A, 2012, EPILEPSIA, V53, P1215, DOI 10.1111/j.1528-1167.2012.03540.x

Ortega-Ramírez A, 2017, MEDIAT INFLAMM, V2017, DOI 10.1155/2017/3728096

Periyasamy P, 2016, AUTOPHAGY, V12, P1310, DOI 10.1080/15548627.2016.1183844

Ponomarev ED, 2013, GLIA, V61, P91, DOI 10.1002/glia.22363

Puma C, 2001, J NEUROCHEM, V78, P960, DOI 10.1046/j.1471-4159.2001.00469.x

Quintana P, 2015, J PHYSIOL-LONDON, V593, P4373, DOI 10.1113/JP270701

Reynolds AD, 2010, J IMMUNOL, V184, P2261, DOI 10.4049/jimmunol.0901852

Robertson CL, 2015, PEDIATR CRIT CARE ME, V16, P236, DOI 10.1097/PCC.0000000000000323

Rosenkranz D, 2007, J NEUROIMMUNOL, V188, P117, DOI 10.1016/j.jneuroim.2007.05.011

Sajic M, 2012, MULT SCLER J, V18, P286, DOI 10.1177/1352458511424309

Saresella M, 2016, BRAIN BEHAV IMMUN, V57, P125, DOI 10.1016/j.bbi.2016.03.009

Saresella M, 2014, J ALZHEIMERS DIS, V38, P403, DOI 10.3233/JAD-131160

Saresella M, 2011, BRAIN BEHAV IMMUN, V25, P539, DOI 10.1016/j.bbi.2010.12.004

Sayyah Mohammad, 2012, Iranian Biomedical Journal, V16, P25, DOI 10.6091/IBJ.1030.2012

Scheld M, 2016, J NEUROSCI, V36, P1410, DOI 10.1523/JNEUROSCI.2456-15.2016

Schiffmann S, 2014, BIOCHEM PHARMACOL, V87, P625, DOI 10.1016/j.bcp.2013.12.006

Schmitt C, 2012, J NEUROINFLAMM, V9, DOI 10.1186/1742-2094-9-187

Segal B, 2010, SEMIN IMMUNOPATHOL, V32, P71, DOI 10.1007/s00281-009-0186-z

Sekeljic V, 2012, BRAIN STRUCT FUNCT, V217, P411, DOI 10.1007/s00429-011-0336-7

Shao ZQ, 2015, J NEUROL SCI, V349, P65, DOI 10.1016/j.jns.2014.12.027

Sharma S, 2016, NEUROPHARMACOLOGY, V105, P285, DOI 10.1016/j.neuropharm.2016.01.025

Shi GD, 2013, GLIA, V61, P504, DOI 10.1002/glia.22451

Skaper SD, 2011, CNS NEUROL DISORD-DR, V10, P44

Sommer A, 2017, MOL NEURODEGENER, V12, DOI 10.1186/s13024-017-0222-8

Srivastava IN, 2016, NEUROBIOL DIS, V85, P144, DOI 10.1016/j.nbd.2015.10.001

Staszewski O, 2014, CELL TISSUE RES, V356, P609, DOI 10.1007/s00441-014-1815-y

Sung H. Y., BIOCH BIOPHYS RES CO, V414, P700

Suzuki T, 2006, J NEUROSCI RES, V83, P1461, DOI 10.1002/jnr.20850

Tan C, 2016, MOL NEUROBIOL, V53, P3854, DOI 10.1007/s12035-015-9314-z

Tang L, 2014, J MOL NEUROSCI, V53, P103, DOI 10.1007/s12031-013-0204-4

Tang Y, 2017, GENET MOL RES, V16, DOI 10.4238/gmr16019166

Vafadari B, 2016, J NEUROCHEM, V139, P91, DOI 10.1111/jnc.13415

Venkateshaiah SU, 2018, J ALLERGY CLIN IMMUN, V141, P906, DOI 10.1016/j.jaci.2017.05.025

Veremeyko T, 2012, JOVE-J VIS EXP, DOI 10.3791/4097

Volpi C, 2012, NEUROPHARMACOLOGY, V63, P501, DOI 10.1016/j.neuropharm.2012.05.024

Vos CMP, 2000, J NEUROIMMUNOL, V109, P221, DOI 10.1016/S0165-5728(00)00308-8

Wattananit S, 2016, J NEUROSCI, V36, P4182, DOI 10.1523/JNEUROSCI.4317-15.2016

White EJ, 2017, J NEUROIMMUNOL, V306, P55, DOI 10.1016/j.jneuroim.2017.03.004

Williams DW, 2012, J LEUKOCYTE BIOL, V91, P401, DOI 10.1189/jlb.0811394

Woo MS, 2008, J NEUROCHEM, V106, P770, DOI 10.1111/j.1471-4159.2008.05430.x

Wu JQ, 2016, J PSYCHIATR RES, V76, P9, DOI 10.1016/j.jpsychires.2016.01.013

Yamano Y, 2009, PLOS ONE, V4, DOI 10.1371/journal.pone.0006517

Yang CM, 2015, MOL NEUROBIOL, V52, P303, DOI 10.1007/s12035-014-8838-y

Yao KR, 2010, J NEUROIMMUNE PHARM, V5, P469, DOI 10.1007/s11481-010-9232-9

Yeghiazaryan M, 2014, PLOS ONE, V9, DOI 10.1371/journal.pone.0099789

Yu JT, 2012, MOL NEUROBIOL, V45, P314, DOI 10.1007/s12035-012-8237-1

Zhang J, 2014, J NEUROSCI, V34, P14919, DOI 10.1523/JNEUROSCI.1165-14.2014

Zhang JX, 2013, PLOS ONE, V8, DOI [10.1371/journal.pone.0076378, 10.1371/journal.pone.0058115]

Zhou XG, 2011, BIOCHEM BIOPH RES CO, V411, P271, DOI 10.1016/j.bbrc.2011.06.117

Zhou Y, 2016, MOL NEURODEGENER, V11, DOI 10.1186/s13024-016-0094-3

Zorrilla-Zubilete MA, 2018, J NEUROCHEM, V144, P128, DOI 10.1111/jnc.14243

NR 175

TC 73

Z9 75

U1 7

U2 62

PU PERGAMON-ELSEVIER SCIENCE LTD

PI OXFORD

PA THE BOULEVARD, LANGFORD LANE, KIDLINGTON, OXFORD OX5 1GB, United Kingdom

SN 0197-0186

EI 1872-9754

J9 NEUROCHEM INT

JI Neurochem. Int.

PD NOV

PY 2018

VL 120

BP 13

EP 20

DI 10.1016/j.neuint.2018.07.003

PG 8

WC Biochemistry & Molecular Biology; Neurosciences

WE Science Citation Index Expanded (SCI-EXPANDED)

SC Biochemistry & Molecular Biology; Neurosciences & Neurology

GA GX5VR

UT WOS:000447819600002

PM 30016687

DA 2024-08-12

ER

PT J

AU Kim, N

Martínez, CC

Jang, DS

Lee, JK

Oh, MS

AF Kim, Namkwon

Martinez, Cindy Cruz

Jang, Dae Sik

Lee, Jong Kil

Oh, Myung Sook

TI Anti-neuroinflammatory effect of <i>Iresine</i> <i>celosia</i> on

lipopolysaccharide-stimulated microglial cells and mouse

SO BIOMEDICINE & PHARMACOTHERAPY

LA English

DT Article

DE Iresine celosia; Neuroinflammation; Microglia; Lipopolysaccharide;

Iresin

ID parkinson's disease; NEURODEGENERATION; INFLAMMATION; MECHANISMS;

EXPRESSION; PATHWAYS

AB Abnormal inflammatory response in the central nervous system plays a critical role in various neurological disorders such as Parkinson's disease, Alzheimer's disease and Huntington's disease. Therefore, modulation of abnormal neuroinflammation is thought to be a promising therapeutic strategy for these diseases. Based on this idea, we focused on finding a potential candidate material that would regulate excessive neuroinflammation. Iresine celosia has long been used as a traditional Mexican medicine to treat fever and oral disorders. In the present study, we evaluated the anti-neuroinflammatory effects of Iresine celosia extract (ICE) in lipopolysaccharide (LPS)-stimulated BV2 microglia cells and mice models. In BV2 microglia cells, ICE markedly inhibited production of nitric oxide and proinflammatory cytokines such as tumor necrosis factor-alpha, interleukin-1 beta, and interleukin-6 without causing cytotoxicity. ICE also ameliorated translocation of nuclear factor-kappa B from cytosol to nucleus by LPS. Moreover, ICE attenuated behavioral disturbances by inhibiting activation of microglia and astrocytes in LPS-treated mice. Collectively, these data indicate that ICE is a potential therapeutic agent for treating inflammation-related diseases.

C1 [Kim, Namkwon; Jang, Dae Sik; Oh, Myung Sook] Kyung Hee Univ, Grad Sch, Dept Life & Nanopharmaceut Sci, 26 Kyungheedae Ro, Seoul 02447, South Korea.

[Martinez, Cindy Cruz; Oh, Myung Sook] Kyung Hee Univ, Coll Pharm, Dept Oriental Pharmaceut Sci, 26 Kyungheedae Ro, Seoul 02447, South Korea.

[Lee, Jong Kil] Kyung Hee Univ, Coll Pharm, Dept Pharm, 26 Kyungheedae Ro, Seoul 02447, South Korea.

[Lee, Jong Kil; Oh, Myung Sook] Kyung Hee Univ, Kyung Hee East West Pharmaceut Res Inst, Seoul 02447, South Korea.

C3 Kyung Hee University; Kyung Hee University; Kyung Hee University; Kyung

Hee University

RP Oh, MS (corresponding author), Kyung Hee Univ, Grad Sch, Dept Life & Nanopharmaceut Sci, 26 Kyungheedae Ro, Seoul 02447, South Korea.; Lee, JK (corresponding author), Kyung Hee Univ, Coll Pharm, Dept Pharm, 26 Kyungheedae Ro, Seoul 02447, South Korea.

EM jklee3984@khu.ac.kr; msohok@khu.ac.kr

RI Oh, Myung Sook/AAH-9948-2020; Jang, Dae Sik/AAI-4526-2020; Lee, Jong

Kil/AAK-8231-2020

FU Medical Research Center Program through the National Research Foundation

of Korea - Ministry of Science and ICT [NRF-2017R1A5A2014768]

FX This work was also supported by the Medical Research Center Program

through the National Research Foundation of Korea funded by the Ministry

of Science and ICT (NRF-2017R1A5A2014768).

CR Amor S, 2010, IMMUNOLOGY, V129, P154, DOI 10.1111/j.1365-2567.2009.03225.x

[Anonymous], 2007, PLANTAS MED

Biber K, 2016, NAT REV DRUG DISCOV, V15, P110, DOI 10.1038/nrd.2015.14

BLASI E, 1990, J NEUROIMMUNOL, V27, P229, DOI 10.1016/0165-5728(90)90073-V

Block ML, 2005, PROG NEUROBIOL, V76, P77, DOI 10.1016/j.pneurobio.2005.06.004

Braak H, 2003, NEUROBIOL AGING, V24, P197, DOI 10.1016/S0197-4580(02)00065-9

Chen BC, 1999, IMMUNOLOGY, V97, P124, DOI 10.1046/j.1365-2567.1999.00747.x

Cunningham C, 2013, GLIA, V61, P71, DOI 10.1002/glia.22350

DENENBERG VH, 1969, ANN NY ACAD SCI, V159, P852, DOI 10.1111/j.1749-6632.1969.tb12983.x

Dipankar C, 2012, COLLOID SURFACE B, V98, P112, DOI 10.1016/j.colsurfb.2012.04.006

González H, 2014, J NEUROIMMUNOL, V274, P1, DOI 10.1016/j.jneuroim.2014.07.012

Hanada T, 2002, CYTOKINE GROWTH F R, V13, P413, DOI 10.1016/S1359-6101(02)00026-6

Hernandez F., 1959, HIST NATURAL NUEVA E

Hwang DS, 2018, ENVIRON TOXICOL, V33, P23, DOI 10.1002/tox.22463

Ju MS, 2010, FOOD CHEM TOXICOL, V48, P2037, DOI 10.1016/j.fct.2010.05.002

Kaminska B, 2005, BBA-PROTEINS PROTEOM, V1754, P253, DOI 10.1016/j.bbapap.2005.08.017

Kang A, 2011, J NEUROINFLAMM, V8, DOI 10.1186/1742-2094-8-100

Kim EK, 2015, ARCH TOXICOL, V89, P867, DOI 10.1007/s00204-015-1472-2

Kim N, 2018, BIOMOL THER, V26, P210, DOI 10.4062/biomolther.2018.008

Kim SH, 2004, NEUROBIOL AGING, V25, P431, DOI 10.1016/S0197-4580(03)00126-X

Krishna S, 2016, BEHAV BRAIN RES, V302, P279, DOI 10.1016/j.bbr.2016.01.038

Kumar A, 2014, FREE RADICAL BIO MED, V73, P51, DOI 10.1016/j.freeradbiomed.2014.04.014

Liu YS, 2007, NAT REV IMMUNOL, V7, P202, DOI 10.1038/nri2035

Martini A. C., 2018, J ALZHEIMERS DIS

Moon M, 2017, NUTRIENTS, V9, DOI 10.3390/nu9101057

O'Connor JC, 2009, MOL PSYCHIATR, V14, P511, DOI 10.1038/sj.mp.4002148

Ohsawa K, 2004, J NEUROCHEM, V88, P844, DOI 10.1046/j.1471-4159.2003.02213.x

Pawate S, 2004, J NEUROSCI RES, V77, P540, DOI 10.1002/jnr.20180

Paxinos G., 2013, PAXINOS FRANKLINS MO, VFourth

Porru D, 2017, J CLIN UROL, V10, P464, DOI 10.1177/2051415817698289

Querfurth HW, 2010, NEW ENGL J MED, V362, P329, DOI 10.1056/NEJMra0909142

Raponi E, 2007, GLIA, V55, P165, DOI 10.1002/glia.20445

Rios MY, 2005, MAGN RESON CHEM, V43, P339, DOI 10.1002/mrc.1550

Tai YJ, 2018, J MOL NEUROSCI, V64, P80, DOI 10.1007/s12031-017-1007-9

Teeling JL, 2009, NEUROSCIENCE, V158, P1062, DOI 10.1016/j.neuroscience.2008.07.031

Zhang FX, 2018, BIOMED PHARMACOTHER, V97, P1011, DOI 10.1016/j.biopha.2017.08.132

NR 36

TC 10

Z9 10

U1 0

U2 4

PU ELSEVIER FRANCE-EDITIONS SCIENTIFIQUES MEDICALES ELSEVIER

PI ISSY-LES-MOULINEAUX

PA 65 RUE CAMILLE DESMOULINS, CS50083, 92442 ISSY-LES-MOULINEAUX, FRANCE

SN 0753-3322

EI 1950-6007

J9 BIOMED PHARMACOTHER

JI Biomed. Pharmacother.

PD MAR

PY 2019

VL 111

BP 1359

EP 1366

DI 10.1016/j.biopha.2019.01.017

PG 8

WC Medicine, Research & Experimental; Pharmacology & Pharmacy

WE Science Citation Index Expanded (SCI-EXPANDED)

SC Research & Experimental Medicine; Pharmacology & Pharmacy

GA HL5AK

UT WOS:000458737800145

PM 30841450

OA gold

DA 2024-08-12

ER

PT J

AU Amruta, N

Chastain, WH

Paz, M

Solch, RJ

Murray-Brown, IC

Befeler, JB

Gressett, TE

Longo, MT

Engler-Chiurazzi, EB

Bix, G

AF Amruta, Narayanappa

Chastain, Wesley H.

Paz, Meshi

Solch, Rebecca J.

Murray-Brown, Isabel C.

Befeler, Jaime B.

Gressett, Timothy E.

Longo, Michele T.

Engler-Chiurazzi, Elizabeth B.

Bix, Gregory

TI SARS-CoV-2 mediated neuroinflammation and the impact of COVID-19 in

neurological disorders

SO CYTOKINE & GROWTH FACTOR REVIEWS

LA English

DT Review

DE COVID-19; SARS-CoV-2; Neuroinflammation; Integrins; Neurological

disorders; Fibrosis

ID PEPTIDE ATN-161 AC-PHSCN-NH2; CENTRAL-NERVOUS-SYSTEM;

parkinson's disease; CYTOKINE STORM; INTEGRIN; INFECTION; SARS; CELL;

METAANALYSIS; DEPRESSION

AB SARS-CoV-2 is a novel coronavirus that severely affects the respiratory system, is the cause of the COVID-19 pandemic, and is projected to result in the deaths of 2 million people worldwide. Recent reports suggest that SARS-CoV-2 also affects the central nervous system along with other organs. COVID-19-associated complications are observed in older people with underlying neurological conditions like stroke, Alzheimer?s disease, and parkinson's disease. Hence, we discuss SARS-CoV-2 viral replication and its inflammation-mediated infection. This review also focuses on COVID-19 associated neurological complications in individuals with those complications as well as other groups of people. Finally, we also briefly discuss the current therapies available to treat patients, as well as ongoing available treatments and vaccines for effective cures with a special focus on the therapeutic potential of a small 5 amino acid peptide (PHSCN), ATN-161, that inhibits SARS-CoV-2 spike protein binding to both integrin ?5131 and ?5131/hACE2.

C1 [Amruta, Narayanappa; Chastain, Wesley H.; Paz, Meshi; Murray-Brown, Isabel C.; Befeler, Jaime B.; Gressett, Timothy E.; Engler-Chiurazzi, Elizabeth B.; Bix, Gregory] Tulane Univ, Sch Med, Clin Neurosci Res Ctr, Dept Neurosurg, 1430 Tulane Ave, New Orleans, LA 70112 USA.

[Solch, Rebecca J.; Longo, Michele T.; Bix, Gregory] Tulane Univ, Sch Med, Dept Neurol, 1430 Tulane Ave, New Orleans, LA 70112 USA.

[Longo, Michele T.; Engler-Chiurazzi, Elizabeth B.; Bix, Gregory] Tulane Univ, Tulane Brain Inst, New Orleans, LA 70112 USA.

C3 Tulane University; Tulane University; Tulane University

RP Bix, G (corresponding author), Tulane Univ, Sch Med, Ctr Clin Neurosci, 131 S Robertson,Ste 1300,Room 1349, New Orleans, LA 70112 USA.

EM gbix@tulane.edu

RI Narayanappa, Amruta/GZA-4478-2022; Narayanappa, Amruta/ELQ-9260-2022;

Engler-Chiurazzi, Elizabeth/JKH-5253-2023

OI Narayanappa, Amruta/0000-0003-1388-9702; Engler-Chiurazzi,

Elizabeth/0000-0002-7656-1192; , Wesley Chastain/0000-0002-5360-4890;

Murray-Brown, Isabel/0000-0002-3077-5216; Ottaiano,

Rebecca/0000-0001-5752-9229

FU National Institutes of Health [R01 NS065842, R01 NS089515]; National

Institutes of Health/NIMH [K01 MH117343]

FX This work was funded by the National Institutes of HealthR01 NS065842

and National Institutes of HealthR01 NS089515 to G.J.B, National

Institutes of Health/NIMH K01 MH117343 to E.B.E-C.

CR Abbott NJ, 2010, NEUROBIOL DIS, V37, P13, DOI 10.1016/j.nbd.2009.07.030

Ahamed J, 2005, BLOOD, V105, P2384, DOI 10.1182/blood-2004-09-3422

Ahmed S, 2014, AGE AGEING, V43, P326, DOI 10.1093/ageing/afu022

Amruta N, 2020, J NEUROIMMUNOL, V346, DOI 10.1016/j.jneuroim.2020.577318

[Anonymous], NATURE, DOI DOI 10.1038/s41586-020-2312-y

[Anonymous], 2020, LANCET, DOI DOI 10.1016/S0140-6736(20)30920-X

Antonini A, 2020, MOVEMENT DISORD, V35, P905, DOI 10.1002/mds.28104

Antony AR, 2020, SEIZURE-EUR J EPILEP, V83, P234, DOI 10.1016/j.seizure.2020.10.014

Arbour N, 2000, J VIROL, V74, P8913, DOI 10.1128/JVI.74.19.8913-8921.2000

Astuti I, 2020, DIAB MET SYND CLIN R, V14, P407, DOI 10.1016/j.dsx.2020.04.020

Aznab M, 2020, INT J CLIN ONCOL, V25, P1581, DOI 10.1007/s10147-020-01734-6

Baig AM, 2020, ACS CHEM NEUROSCI, V11, P995, DOI 10.1021/acschemneuro.0c00122

Balestrino R, 2020, J NEUROL, V267, P2797, DOI 10.1007/s00415-020-09879-0

Beddingfield B.J., 2020, JACC BASIC TRANSL SC

Beddingfield BJ, 2021, JACC-BASIC TRANSL SC, V6, P1, DOI 10.1016/j.jacbts.2020.10.003

Besnard S, 2020, FRONT NEUROL, V11, DOI 10.3389/fneur.2020.582794

Bleau C, 2015, J VIROL, V89, P9896, DOI 10.1128/JVI.01501-15

Bombaci A, 2021, NEUROL SCI, V42, P9, DOI 10.1007/s10072-020-04783-x

Bradley BT, 2020, LANCET, V396, P320, DOI 10.1016/S0140-6736(20)31305-2

Brown EE, 2020, AM J GERIAT PSYCHIAT, V28, P712, DOI 10.1016/j.jagp.2020.04.010

Burke JF, 2020, NEUROSURGERY, V87, pE50, DOI 10.1093/neuros/nyaa116

Cabona C, 2021, NEUROL SCI, V42, P1231, DOI 10.1007/s10072-020-04997-z

Cafaro R P, 1960, J Am Dent Soc Anesthesiol, V7, P4

Campana P, 2020, CELLS-BASEL, V9, DOI 10.3390/cells9092046

Carfi A, 2020, JAMA-J AM MED ASSOC, V324, P603, DOI 10.1001/jama.2020.12603

Cashman R., 2020, NEUROONCOL ADV, V2

CECCACCI L, 1982, AM J OBSTET GYNECOL, V144, P105, DOI 10.1016/0002-9378(82)90405-7

Chua SE, 2004, CAN J PSYCHIAT, V49, P385, DOI 10.1177/070674370404900607

Cianfrocca ME, 2006, BRIT J CANCER, V94, P1621, DOI 10.1038/sj.bjc.6603171

Cilia R, 2020, MOVEMENT DISORD, V35, P1287, DOI 10.1002/mds.28170

Clarke NE, 2012, PLOS ONE, V7, DOI 10.1371/journal.pone.0034747

Cohen ME, 2020, LANCET NEUROL, V19, P804, DOI 10.1016/S1474-4422(20)30305-7

Consonni M, 2021, J NEUROL, V268, P420, DOI 10.1007/s00415-020-10080-6

Coperchini F, 2020, CYTOKINE GROWTH F R, V53, P25, DOI 10.1016/j.cytogfr.2020.05.003

Costela-Ruiz VJ, 2020, CYTOKINE GROWTH F R, V54, P62, DOI 10.1016/j.cytogfr.2020.06.001

Cox RJ, 2020, NAT REV IMMUNOL, V20, P581, DOI 10.1038/s41577-020-00436-4

Crisà FM, 2020, STEREOT FUNCT NEUROS, V98, P319, DOI 10.1159/000510271

Czeisler MÉ, 2020, MMWR-MORBID MORTAL W, V69, P1049, DOI [10.1101/2020.04.22.20076141v1, 10.15585/mmwr.mm6932a1]

Dakal TC, 2021, IMMUNOBIOLOGY, V226, DOI 10.1016/j.imbio.2020.152021

De Boni RB, 2020, J MED INTERNET RES, V22, DOI 10.2196/22835

De Lucia N, 2020, NEUROL SCI, V41, P2339, DOI 10.1007/s10072-020-04592-2

De Marchi F, 2021, ACTA NEUROL SCAND, V143, P489, DOI 10.1111/ane.13373

de Wit E, 2016, NAT REV MICROBIOL, V14, P523, DOI 10.1038/nrmicro.2016.81

Ding Q, 2020, J MED VIROL, V92, P1549, DOI 10.1002/jmv.25781

Doñate F, 2008, CLIN CANCER RES, V14, P2137, DOI 10.1158/1078-0432.CCR-07-4530

Dowlati Y, 2010, BIOL PSYCHIAT, V67, P446, DOI 10.1016/j.biopsych.2009.09.033

Dubé M, 2018, J VIROL, V92, DOI 10.1128/JVI.00404-18

Edwards DN, 2020, J CEREBR BLOOD F MET, V40, P1695, DOI 10.1177/0271678X19880161

Elibol E, 2021, EUR ARCH OTO-RHINO-L, V278, P1233, DOI 10.1007/s00405-020-06319-7

Elkhouly A, 2020, CUREUS J MED SCIENCE, V12, DOI 10.7759/cureus.8992

Ellul MA, 2020, LANCET NEUROL, V19, P767, DOI 10.1016/S1474-4422(20)30221-0

Esakandari H, 2020, BIOL PROCED ONLINE, V22, DOI 10.1186/s12575-020-00128-2

Escand K., 2020, COVID 19 FALSE DICHO

Fara A, 2020, OPEN BIOL, V10, DOI 10.1098/rsob.200160

Fasano A, 2020, PARKINSONISM RELAT D, V78, P134, DOI 10.1016/j.parkreldis.2020.08.012

Fehr AR, 2015, METHODS MOL BIOL, V1282, P1, DOI 10.1007/978-1-4939-2438-7_1

Figueiredo R, 2020, BMJ CASE REP, V13, DOI 10.1136/bcr-2020-237146

Filatov A, 2020, CUREUS J MED SCIENCE, V12, DOI 10.7759/cureus.7352

Fiolet T, 2021, CLIN MICROBIOL INFEC, V27, P19, DOI 10.1016/j.cmi.2020.08.022

Fotuhi M, 2020, J ALZHEIMERS DIS, V76, P3, DOI 10.3233/JAD-200581

Giacomelli A, 2020, CLIN INFECT DIS, V71, P889, DOI 10.1093/cid/ciaa330

Grant MC, 2020, PLOS ONE, V15, DOI 10.1371/journal.pone.0234765

Grifoni A, 2020, CELL, V181, P1489, DOI 10.1016/j.cell.2020.05.015

Guell K, 2014, EXPERT REV NEUROTHER, V14, P1287, DOI 10.1586/14737175.2014.964210

Guo DJ, 2020, PARKINSONS DIS-US, V2020, DOI 10.1155/2020/1216568

Gutiérrez-Ortiz C, 2020, NEUROLOGY, V95, pE601, DOI 10.1212/WNL.0000000000009619

Hainque E, 2020, PARKINSONISM RELAT D, V75, P126, DOI 10.1016/j.parkreldis.2020.05.008

Hatley RJD, 2018, ANGEW CHEM INT EDIT, V57, P3298, DOI 10.1002/anie.201707948

Helms J, 2020, NEW ENGL J MED, V382, P2268, DOI 10.1056/NEJMc2008597

Hoffmann M, 2020, CELL, V181, P271, DOI 10.1016/j.cell.2020.02.052

Hosseini M., 2020, ARCH ACAD EMERG MED, V8, pE62

Huang C., 2020, Lancet, V395, P506, DOI DOI 10.1016/S0140-6736(20)30183-5

Hulswit RJG, 2016, ADV VIRUS RES, V96, P29, DOI 10.1016/bs.aivir.2016.08.004

Hussein HAM, 2015, ARCH VIROL, V160, P2669, DOI 10.1007/s00705-015-2579-8

Ishibashi Y, 2001, MICROB PATHOGENESIS, V30, P279, DOI 10.1006/mpat.2001.0432

Janiri D, 2020, FRONT PSYCHIATRY, V11, DOI 10.3389/fpsyt.2020.581144

Jean SS, 2020, J MICROBIOL IMMUNOL, V53, P436, DOI 10.1016/j.jmii.2020.03.034

Kandikattu HK, 2020, CYTOKINE GROWTH F R, V54, P24, DOI 10.1016/j.cytogfr.2020.06.008

Kandikattu HK, 2019, CYTOKINE GROWTH F R, V47, P83, DOI 10.1016/j.cytogfr.2019.05.003

Kandikattu HK, 2017, PHYSIOL BEHAV, V175, P56, DOI 10.1016/j.physbeh.2017.03.035

Karimi N, 2020, IRAN RED CRESCENT ME, V22, DOI 10.5812/ircmj.102828

Kato V, 2020, ACTA NEUROL BELG, V120, P1051, DOI 10.1007/s13760-020-01412-4

Keni R, 2020, FRONT PUBLIC HEALTH, V8, DOI 10.3389/fpubh.2020.00216

Kessler RA, 2020, J NEURO-ONCOL, V148, P211, DOI 10.1007/s11060-020-03523-7

Khailany RA, 2020, GENE REP, V19, DOI 10.1016/j.genrep.2020.100682

Khaja M, 2020, AM J CASE REP, V21, DOI 10.12659/AJCR.927956

Khalili P, 2006, MOL CANCER THER, V5, P2271, DOI 10.1158/1535-7163.MCT-06-0100

Köhler O, 2014, JAMA PSYCHIAT, V71, P1381, DOI 10.1001/jamapsychiatry.2014.1611

Kubota T, 2021, CLIN NEUROL NEUROSUR, V200, DOI 10.1016/j.clineuro.2020.106349

Kumar S., 2019, CORONAVIRUS DIS, V2020

Kuo CL, 2020, J GERONTOL A-BIOL, V75, P2231, DOI 10.1093/gerona/glaa131

Kwong KCNK, 2020, J CLIN NEUROSCI, V77, P13, DOI 10.1016/j.jocn.2020.04.124

Lai CC, 2020, INT J ANTIMICROB AG, V55, DOI 10.1016/j.ijantimicag.2020.105924

Lam MHB, 2009, ARCH INTERN MED, V169, P2142, DOI 10.1001/archinternmed.2009.384

Lam S, 2020, EUR J PHARMACOL, V886, DOI 10.1016/j.ejphar.2020.173451

Lan J, 2020, NATURE, V581, P215, DOI 10.1038/s41586-020-2180-5

Lang FM, 2020, NAT REV IMMUNOL, V20, P507, DOI 10.1038/s41577-020-0357-7

Lau KK, 2004, EMERG INFECT DIS, V10, P342, DOI 10.3201/eid1002.030638

Lauer SA, 2020, ANN INTERN MED, V172, P577, DOI 10.7326/M20-0504

Lechien JR, 2020, EUR ARCH OTO-RHINO-L, V277, P2251, DOI 10.1007/s00405-020-05965-1

Li F, 2005, SCIENCE, V309, P1864, DOI 10.1126/science.1116480

Li F, 2016, ANNU REV VIROL, V3, P237, DOI 10.1146/annurev-virology-110615-042301

Li YY, 2016, INTERVIROLOGY, V59, P163, DOI 10.1159/000453066

Liotta EM, 2020, ANN CLIN TRANSL NEUR, V7, P2221, DOI 10.1002/acn3.51210

Liu YW, 2022, J AM STAT ASSOC, V117, P897, DOI 10.1080/01621459.2020.1831926

Loftis JM, 2010, NEUROBIOL DIS, V37, P519, DOI 10.1016/j.nbd.2009.11.015

Lu XJ, 2008, PERSPECT MED CHEM, V2, P57

Lv XL, 2019, VET MICROBIOL, V233, P147, DOI 10.1016/j.vetmic.2019.04.029

Mackay IM, 2015, VIROL J, V12, DOI 10.1186/s12985-015-0439-5

Mahmoudi S, 2020, J CLIN IMMUNOL, V40, P974, DOI 10.1007/s10875-020-00824-4

Mak WWS, 2009, PSYCHOL HEALTH, V24, P161, DOI 10.1080/08870440701447649

Maleki M, 2020, TRAVEL MED INFECT DI, V37, DOI 10.1016/j.tmaid.2020.101742

Mao L, 2020, JAMA NEUROL, V77, P683, DOI 10.1001/jamaneurol.2020.1127

Marshall M, 2020, NATURE, V585, P339, DOI 10.1038/d41586-020-02598-6

Masselli Elena, 2020, Advances in Biological Regulation, V77, P100737, DOI 10.1016/j.jbior.2020.100737

Mastroianni A, 2020, ECLINICALMEDICINE, V24, DOI 10.1016/j.eclinm.2020.100410

Mazza MG, 2020, BRAIN BEHAV IMMUN, V89, P594, DOI 10.1016/j.bbi.2020.07.037

McAloon C, 2020, BMJ OPEN, V10, DOI 10.1136/bmjopen-2020-039652

Ménard C, 2016, NEUROSCIENCE, V321, P138, DOI 10.1016/j.neuroscience.2015.05.053

Moldofsky H, 2011, BMC NEUROL, V11, DOI 10.1186/1471-2377-11-37

Mollica V, 2020, FUTURE ONCOL, V16, P2029, DOI 10.2217/fon-2020-0571

MORRIS AG, 1988, IMMUNOLOGY, P43

Mortaz E, 2020, BMC PULM MED, V20, DOI 10.1186/s12890-020-01310-8

Murray E.J, 2020, COVID 19 FALSE DICHO

Murta V, 2020, ASN NEURO, V12, DOI 10.1177/1759091420954960

Naish J, 1990, Nurs Stand, V4, P18

Nakagawa S, 2020, INFLAMM REGEN, V40, DOI 10.1186/s41232-020-00126-7

Napione L, 2007, AUTOIMMUN REV, V7, P18, DOI 10.1016/j.autrev.2007.03.007

Naqvi AA, 2020, BBA-MOL BASIS DIS, V1866, DOI 10.1016/j.bbadis.2020.165878

Natoli S, 2020, EUR J NEUROL, V27, P1764, DOI 10.1111/ene.14277

Netland J, 2008, J VIROL, V82, P7264, DOI 10.1128/JVI.00737-08

Neumann J, 2020, CLIN TRANSL IMMUNOL, V9, DOI 10.1002/cti2.1204

Paces J, 2020, PHYSIOL RES, V69, P379, DOI 10.33549/physiolres.934492

Pandey P, 2021, J BIOMOL STRUCT DYN, V39, P6306, DOI 10.1080/07391102.2020.1796811

Pappa S, 2020, BRAIN BEHAV IMMUN, V88, P901, DOI [10.1016/j.bbi.2020.11.023, 10.1016/j.bbi.2020.05.026]

Pessina F, 2020, NEUROSURG FOCUS, V49, DOI 10.3171/2020.9.FOCUS20704

Pezzini A, 2020, NAT REV NEUROL, V16, P636, DOI 10.1038/s41582-020-0398-3

Piano C, 2021, CLIN PARK RELAT DISO, V4, DOI DOI 10.1016/J.PRDOA.2020.100082

Povlow A, 2021, J EMERG MED, V60, P73, DOI 10.1016/j.jemermed.2020.10.010

Poyiadji N, 2020, RADIOLOGY, V296, pE119, DOI 10.1148/radiol.2020201187

Price SJ, 2020, BMJ OPEN, V10, DOI 10.1136/bmjopen-2020-040898

Ragia G, 2020, EUR J CLIN PHARMACOL, V76, P1623, DOI 10.1007/s00228-020-02963-4

Rahman AA, 2021, TRANSL STROKE RES, V12, P1, DOI 10.1007/s12975-020-00841-w

Ramadan N, 2019, GERMS, V9, P35, DOI 10.18683/germs.2019.1155

Ribeiro DE, 2021, MOL PSYCHIATR, V26, P1044, DOI 10.1038/s41380-020-00965-3

Roberts J, 2017, J CEREBR BLOOD F MET, V37, P85, DOI 10.1177/0271678X15616979

Santos-García D, 2020, MOVEMENT DISORD, V35, P1712, DOI 10.1002/mds.28261

Satarker S, 2020, ARCH MED RES, V51, P482, DOI 10.1016/j.arcmed.2020.05.012

Schellekens MMI, 2020, MOV DISORD CLIN PRAC, V7, P977, DOI 10.1002/mdc3.13088

Schirinzi T, 2020, MOV DISORD CLIN PRAC, V7, P797, DOI 10.1002/mdc3.13026

Scullen T, 2020, WORLD NEUROSURG, V141, pE437, DOI 10.1016/j.wneu.2020.05.192

Shah PB, 2021, NEUROLOGY, V96, P33, DOI 10.1212/WNL.0000000000010978

Shah VK, 2020, FRONT IMMUNOL, V11, DOI 10.3389/fimmu.2020.01949

Shereen MA, 2020, J ADV RES, V24, P91, DOI 10.1016/j.jare.2020.03.005

Solomon S, 2007, AR4 CLIMATE CHANGE 2007: THE PHYSICAL SCIENCE BASIS, P1

Song PP, 2020, CLIN CHIM ACTA, V509, P280, DOI 10.1016/j.cca.2020.06.017

Soy M, 2020, CLIN RHEUMATOL, V39, P2085, DOI 10.1007/s10067-020-05190-5

Spence JD, 2020, CEREBROVASC DIS, V49, P451, DOI 10.1159/000509581

Steardo L Jr, 2020, TRANSL PSYCHIAT, V10, DOI 10.1038/s41398-020-00949-5

Sun D, 2020, WORLD J PEDIATR, V16, P260, DOI 10.1007/s12519-020-00368-y

Sun PF, 2020, J MED VIROL, V92, P1721, DOI 10.1002/jmv.25804

Tai WB, 2020, CELL MOL IMMUNOL, V17, P613, DOI 10.1038/s41423-020-0400-4

Taquet M, 2021, LANCET PSYCHIAT, V8, P130, DOI 10.1016/S2215-0366(20)30462-4

Tay MZ, 2020, NAT REV IMMUNOL, V20, P363, DOI 10.1038/s41577-020-0311-8

Temesgen Z, 2020, MAYO CLIN PROC, V95, P2382, DOI 10.1016/j.mayocp.2020.08.038

Torales J, 2020, INT J SOC PSYCHIATR, V66, P317, DOI 10.1177/0020764020915212

Toscano G, 2020, NEW ENGL J MED, V382, P2574, DOI 10.1056/NEJMc2009191

Trejo-Gabriel-Galán JM, 2020, NEUROLOGIA, V35, P318, DOI 10.1016/j.nrl.2020.04.015

Tsai Li-Kai, 2005, Acta Neurol China, V14, P113

Tsai ST, 2020, FRONT NEUROL, V11, DOI 10.3389/fneur.2020.00498

Turski WA, 2020, RESTOR NEUROL NEUROS, V38, P343, DOI 10.3233/RNN-201042

V'kovski P, 2021, NAT REV MICROBIOL, V19, P155, DOI 10.1038/s41579-020-00468-6

van der Heide A, 2020, J PARKINSON DIS, V10, P1355, DOI 10.3233/JPD-202251

Vignatelli L., MOVEMENT DISORD

Vijayvargiya P, 2020, MAYO CLIN PROC, V95, P1454, DOI 10.1016/j.mayocp.2020.04.027

Vizheh M, 2020, J DIABETES METAB DIS, V19, P1967, DOI 10.1007/s40200-020-00643-9

Wajnberg A, 2020, SCIENCE, V370, P1227, DOI 10.1126/science.abd7728

Wang FZ, 2020, MED SCI MONITOR, V26, DOI 10.12659/MSM.928996

Wang HH, 2020, EUR J CLIN MICROBIOL, V39, P1629, DOI 10.1007/s10096-020-03899-4

Wang J, 2020, J LEUKOCYTE BIOL, V108, P17, DOI 10.1002/JLB.3COVR0520-272R

Wang L, 2020, J NEUROL, V267, P2777, DOI [10.1007/s00415-020-09974-2, DOI 10.1007/s00415-020-09974-2]

Wang QH, 2020, CELL, V181, P894, DOI 10.1016/j.cell.2020.03.045

Waterhouse A, 2018, NUCLEIC ACIDS RES, V46, pW296, DOI 10.1093/nar/gky427

WICKHAM TJ, 1994, J CELL BIOL, V127, P257, DOI 10.1083/jcb.127.1.257

Wohleb ES, 2016, NAT REV NEUROSCI, V17, P497, DOI 10.1038/nrn.2016.69

Wu YS, 2020, BRAIN BEHAV IMMUN, V87, P18, DOI 10.1016/j.bbi.2020.03.031

Xiao JQ, 2008, VIROLOGY, V370, P430, DOI 10.1016/j.virol.2007.09.012

Xiao XD, 2003, BIOCHEM BIOPH RES CO, V312, P1159, DOI 10.1016/j.bbrc.2003.11.054

Yang Y, 2020, J ALLERGY CLIN IMMUN, V146, P119, DOI 10.1016/j.jaci.2020.04.027

Yoshimoto FK, 2020, PROTEIN J, V39, P198, DOI 10.1007/s10930-020-09901-4

Yue W., 2020, RES SQUARE

Zárate S, 2004, J VIROL, V78, P10839, DOI 10.1128/JVI.78.20.10839-10847.2004

Zhang Q, 2020, MOVEMENT DISORD, V35, P1914, DOI 10.1002/mds.28325

Zhang W, 2020, CLIN IMMUNOL, V214, DOI 10.1016/j.clim.2020.108393

Zhou RH, 2020, IMMUNITY, V53, P864, DOI 10.1016/j.immuni.2020.07.026

Zhou ZQ, 2020, J NEUROL, V267, P2179, DOI 10.1007/s00415-020-09929-7

NR 196

TC 74

Z9 76

U1 0

U2 20

PU ELSEVIER SCI LTD

PI OXFORD

PA THE BOULEVARD, LANGFORD LANE, KIDLINGTON, OXFORD OX5 1GB, OXON, United Kingdom

SN 1359-6101

EI 1879-0305

J9 CYTOKINE GROWTH F R

JI Cytokine Growth Factor Rev.

PD APR

PY 2021

VL 58

BP 1

EP 15

DI 10.1016/j.cytogfr.2021.02.002

PG 15

WC Biochemistry & Molecular Biology; Cell Biology

WE Science Citation Index Expanded (SCI-EXPANDED); Social Science Citation Index (SSCI)

SC Biochemistry & Molecular Biology; Cell Biology

GA RG7KW

UT WOS:000635714000001

PM 33674185

OA Green Published

DA 2024-08-12

ER

PT J

AU Bonte, MA

El Idrissi, F

Gressier, B

Devos, D

Belarbi, K

AF Bonte, Marie-Amandine

El Idrissi, Fatima

Gressier, Bernard

Devos, David

Belarbi, Karim

TI Protein network exploration prioritizes targets for modulating

neuroinflammation in Parkinson's disease

SO INTERNATIONAL IMMUNOPHARMACOLOGY

LA English

DT Article

DE Caspase 1; Cytokines; Drug discovery; Heme Oxygenase-1; Microglia;

neurodegenerative disease; Neuroinflammation; parkinson's disease

ID DOPAMINERGIC-NEURONS; PHOSPHODIESTERASE INHIBITOR; ALPHA-SYNUCLEIN;

MICROGLIAL ACTIVATION; INFLAMMATORY MARKERS; alzheimer's disease;

OXIDATIVE STRESS; NEURODEGENERATION; IBUDILAST; MODEL

AB Parkinson's disease is a progressive neurodegenerative disease associated with a loss of dopaminergic neurons in the substantia nigra of the brain. Neuroinflammation, another hallmark of the disease, is thought to play an important role in the neurodegenerative process. While mitigating neuroinflammation could prove beneficial for Parkinson's disease, identifying the most relevant biological processes and pharmacological targets as well as drugs to modulate them remains highly challenging. The present study aimed to better understand the protein network behind neuroinflammation in Parkinson's disease and to prioritize possible targets for its pharmacological modulation. We first used text-mining to systematically collect the proteins significantly associated to Parkinson's disease neuroinflammation over the scientific literature. The functional interaction network formed by these proteins was then analyzed by integrating functional enrichment, network topology analysis and drug-protein interaction analysis. We identified 57 proteins significantly associated to neuroinflammation in Parkinson's disease. Toll-like Receptor Cascades as well as Interleukin 4, Interleukin 10 and Interleukin 13 signaling appeared as the most significantly enriched biological processes. Protein network analysis using STRING and CentiScaPe identified 8 proteins with the highest ability to control these biological processes underlying neuroinflammation, namely caspase 1, heme oxygenase 1, interleukin 1beta, interleukin 4, interleukin 6, interleukin 10, tumor necrosis factor alpha and toll-like receptor 4. These key proteins were indexed to be targetable by a total of 38 drugs including 27 small compounds 11 protein-based therapies. In conclusion, our study highlights key proteins in Parkinson's disease neuroinflammation as well as pharmacological compounds acting on them. As such, it may facilitate the prioritization of biomarkers for the development of diagnostic, target-engagement assessment and therapeutic tools against Parkinson's disease.

C1 [Bonte, Marie-Amandine; El Idrissi, Fatima; Gressier, Bernard; Devos, David; Belarbi, Karim] Univ Lille, Lille Neurosci & Cognit, CHU Lille, INSERM, F-59000 Lille, France.

[El Idrissi, Fatima; Gressier, Bernard; Belarbi, Karim] Univ Lille, Fac Pharm, Dept Pharmacol, Lille, France.

[Devos, David] I SITE ULNE, Dept Pharmacol Med, LiCEND, Lille, France.

C3 Institut National de la Sante et de la Recherche Medicale (Inserm);

Universite de Lille; CHU Lille; Universite de Lille

RP Belarbi, K (corresponding author), Univ Lille, Lille Neurosci & Cognit, CHU Lille, INSERM, 1 Pl Verdun, F-59006 Lille, France.

EM marieamandine.bonte.etu@univ-lille.fr;

fatima.elidrissi.etu@univ-lille.fr; bernard.gressier@univ-lille.fr;

david.devos@chru-lille.fr; karim.belarbi@inserm.fr

RI belarbi, karim/G-4467-2013; belarbi, karim/T-9461-2018; Devos,

David/E-4059-2013

OI belarbi, karim/0000-0002-0027-1172; Devos, David/0000-0002-2417-799X

CR Arimoto T, 2007, NEUROBIOL AGING, V28, P894, DOI 10.1016/j.neurobiolaging.2006.04.011

Ayuso P, 2015, MEDICINE, V94, DOI 10.1097/MD.0000000000000968

Ayuso P, 2014, FRONT CELL NEUROSCI, V8, DOI 10.3389/fncel.2014.00298

Banerjee S, 2008, J NEUROIMMUNE PHARM, V3, P154, DOI 10.1007/s11481-008-9105-7

Bassil F, 2016, P NATL ACAD SCI USA, V113, P9593, DOI 10.1073/pnas.1609291113

Belarbi K, 2017, MOL NEURODEGENER, V12, DOI 10.1186/s13024-017-0225-5

Belarbi K, 2012, J NEUROINFLAMM, V9, DOI 10.1186/1742-2094-9-23

Béraud D, 2013, J NEUROIMMUNE PHARM, V8, P94, DOI 10.1007/s11481-012-9401-0

Bindea G, 2009, BIOINFORMATICS, V25, P1091, DOI 10.1093/bioinformatics/btp101

Bok E, 2018, EXP NEUROBIOL, V27, P309, DOI 10.5607/en.2018.27.4.309

Brochard V, 2009, J CLIN INVEST, V119, P182, DOI 10.1172/JCI36470

Brodacki B, 2008, NEUROSCI LETT, V441, P158, DOI 10.1016/j.neulet.2008.06.040

Castellani R, 1996, BRAIN RES, V737, P195, DOI 10.1016/0006-8993(96)00729-9

Codolo G, 2013, PLOS ONE, V8, DOI 10.1371/journal.pone.0055375

Cotto Kelsy C, 2018, Nucleic Acids Res, V46, pD1068, DOI 10.1093/nar/gkx1143

Demb J, 2019, J GERIATR ONCOL, V10, P265, DOI 10.1016/j.jgo.2018.07.008

Devos D, 2014, ANTIOXID REDOX SIGN, V21, P195, DOI 10.1089/ars.2013.5593

Ekdahl CT, 2003, P NATL ACAD SCI USA, V100, P13632, DOI 10.1073/pnas.2234031100

Elkind MSV, 2010, INT J STROKE, V5, P117, DOI 10.1111/j.1747-4949.2010.00420.x

Fabiani C, 2019, FRONT CELL NEUROSCI, V13, DOI 10.3389/fncel.2019.00309

Fabregat A, 2018, NUCLEIC ACIDS RES, V46, pD649, DOI 10.1093/nar/gkx1132

Fabregat A, 2017, BMC BIOINFORMATICS, V18, DOI 10.1186/s12859-017-1559-2

Fellner L, 2013, GLIA, V61, P349, DOI 10.1002/glia.22437

Ferreira SA, 2018, FRONT CELL NEUROSCI, V12, DOI 10.3389/fncel.2018.00247

Flores J, 2020, NAT COMMUN, V11, DOI 10.1038/s41467-020-18405-9

Flores J, 2018, NAT COMMUN, V9, DOI 10.1038/s41467-018-06449-x

Gabbita SP, 2012, J NEUROINFLAMM, V9, DOI 10.1186/1742-2094-9-99

Gerhard A, 2006, NEUROBIOL DIS, V21, P404, DOI 10.1016/j.nbd.2005.08.002

Goedert M, 2017, J PARKINSON DIS, V7, pS53, DOI 10.3233/JPD-179005

Gordon R, 2018, SCI TRANSL MED, V10, DOI 10.1126/scitranslmed.aah4066

Gulisano W, 2018, NEUROPHARMACOLOGY, V138, P151, DOI 10.1016/j.neuropharm.2018.06.002

Hamza TH, 2010, NAT GENET, V42, P781, DOI 10.1038/ng.642

Hollenbach JA, 2019, P NATL ACAD SCI USA, V116, P7419, DOI 10.1073/pnas.1821778116

Hühner L, 2017, FRONT MOL NEUROSCI, V10, DOI 10.3389/fnmol.2017.00062

Johnston LC, 2008, MOL THER, V16, P1392, DOI 10.1038/mt.2008.113

Kaur Harjeet, 2016, eNeurologicalSci, V4, P42, DOI 10.1016/j.ensci.2016.06.003

Kim C, 2013, NAT COMMUN, V4, DOI 10.1038/ncomms2534

Lehnardt S, 2003, P NATL ACAD SCI USA, V100, P8514, DOI 10.1073/pnas.1432609100

Leitner GR, 2019, EXPERT OPIN THER TAR, V23, P865, DOI 10.1080/14728222.2019.1676416

Lever J, 2019, GENOME MED, V11, DOI 10.1186/s13073-019-0686-y

Mahoney-Sánchez L, 2021, PROG NEUROBIOL, V196, DOI 10.1016/j.pneurobio.2020.101890

McKenzie BA, 2018, P NATL ACAD SCI USA, V115, pE6065, DOI 10.1073/pnas.1722041115

Miklossy J, 2006, EXP NEUROL, V197, P275, DOI 10.1016/j.expneurol.2005.10.034

Mittelbronn M, 2001, ACTA NEUROPATHOL, V101, P249

Mizuno T, 2004, NEUROPHARMACOLOGY, V46, P404, DOI 10.1016/j.neuropharm.2003.09.009

Mori S, 2017, J NEUROINFLAMM, V14, DOI 10.1186/s12974-017-0862-1

Morrison BE, 2012, J IMMUNOL, V189, P5498, DOI 10.4049/jimmunol.1102150

O'Mara-Eves A, 2015, SYST REV-LONDON, V4, DOI 10.1186/2046-4053-4-5

Oprea TI, 2018, NAT REV DRUG DISCOV, V17, P317, DOI 10.1038/nrd.2018.14

Pan YY, 2019, HUM GENOMICS, V13, DOI 10.1186/s40246-019-0239-x

Pearson TA, 2003, CIRCULATION, V107, P499, DOI 10.1161/01.CIR.0000052939.59093.45

Peter I, 2018, JAMA NEUROL, V75, P939, DOI 10.1001/jamaneurol.2018.0605

Ransohoff RM, 2010, NATURE, V468, P253, DOI 10.1038/nature09615

Rastegar DA, 2019, NPJ PARKINSONS DIS, V5, DOI 10.1038/s41531-019-0086-4

Refolo V, 2019, FRONT CELL NEUROSCI, V13, DOI 10.3389/fncel.2019.00263

Russo I, 2015, J NEUROINFLAMM, V12, DOI 10.1186/s12974-015-0449-7

SAMPAIO EP, 1991, J EXP MED, V173, P699, DOI 10.1084/jem.173.3.699

Schett G, 2010, THER ADV MUSCULOSKEL, V2, P271, DOI 10.1177/1759720X10381432

Schipper HM, 1998, EXP NEUROL, V150, P60, DOI 10.1006/exnr.1997.6752

Schwartz M, 2016, TRENDS IMMUNOL, V37, P668, DOI 10.1016/j.it.2016.08.001

Schwenkgrub J, 2017, PLOS ONE, V12, DOI 10.1371/journal.pone.0182019

Schwenkgrub J, 2017, POSTEP HIG MED DOSW, V71, P137

Shabab T, 2017, INT J NEUROSCI, V127, P624, DOI 10.1080/00207454.2016.1212854

Shahmoradian SH, 2019, NAT NEUROSCI, V22, P1099, DOI 10.1038/s41593-019-0423-2

Singhal A, 2016, PLOS COMPUT BIOL, V12, DOI 10.1371/journal.pcbi.1005017

Spranger J, 2003, DIABETES, V52, P812, DOI 10.2337/diabetes.52.3.812

Stokholm MG, 2017, LANCET NEUROL, V16, P789, DOI 10.1016/S1474-4422(17)30173-4

Szklarczyk D, 2019, NUCLEIC ACIDS RES, V47, pD607, DOI 10.1093/nar/gky1131

Tarantino G, 2020, DIAGNOSTICS, V10, DOI 10.3390/diagnostics10060395

Voon V, 2017, LANCET NEUROL, V16, P238, DOI 10.1016/S1474-4422(17)30004-2

Wakita H, 2003, BRAIN RES, V992, P53, DOI 10.1016/j.brainres.2003.08.028

Wang W, 2016, P NATL ACAD SCI USA, V113, P9587, DOI 10.1073/pnas.1610099113

Wang ZC, 2015, BMC SYST BIOL, V9, DOI 10.1186/s12918-015-0173-z

Yan YQ, 2015, CELL, V160, P62, DOI 10.1016/j.cell.2014.11.047

Yi HA, 2005, CLIN EXP PHARMACOL P, V32, P1097, DOI 10.1111/j.1440-1681.2005.04305.x

Zeng FR, 2020, INT J INFECT DIS, V96, P467, DOI 10.1016/j.ijid.2020.05.055

Zhang W, 2005, FASEB J, V19, P533, DOI 10.1096/fj.04-2751com

NR 77

TC 10

Z9 11

U1 1

U2 10

PU ELSEVIER

PI AMSTERDAM

PA RADARWEG 29, 1043 NX AMSTERDAM, NETHERLANDS

SN 1567-5769

EI 1878-1705

J9 INT IMMUNOPHARMACOL

JI Int. Immunopharmacol.

PD JUN

PY 2021

VL 95

AR 107526

DI 10.1016/j.intimp.2021.107526

EA MAR 2021

PG 9

WC Immunology; Pharmacology & Pharmacy

WE Science Citation Index Expanded (SCI-EXPANDED)

SC Immunology; Pharmacology & Pharmacy

GA SJ5VU

UT WOS:000655603100009

PM 33756233

OA hybrid

DA 2024-08-12

ER

PT J

AU Dichev, V

Kazakova, M

Sarafian, V

AF Dichev, Valentin

Kazakova, Maria

Sarafian, Victoria

TI YKL-40 and neuron-specific enolase in neurodegeneration and

neuroinflammation

SO REVIEWS IN THE NEUROSCIENCES

LA English

DT Article

DE biomarkers; inflammation; neuronal degeneration

ID CEREBROSPINAL-FLUID BIOMARKERS; HUMAN CARTILAGE GLYCOPROTEIN;

MESSENGER-RNA EXPRESSION; MYELIN BASIC-PROTEIN; NORMAL HUMAN-TISSUES;

alzheimer's disease; GAMMA-ENOLASE; HUMAN-SERUM; CHITINASE; NSE

AB neurodegenerative disease comprise a large number of disorders with high impact on human health. Neurodegenerative processes are caused by various etiological factors and differ in their clinical presentation. Neuroinflammation is widely discussed as both a cause and a consequence in the manifestation of these disorders. The interplay between the two entities is considered as a major contributor to the ongoing disease progression. An attentive search and implementation of new and reliable markers specific for the processes of inflammation and degeneration is still needed. YKL-40 is a secreted glycoprotein produced by activated glial cells during neuroinflammation. Neuron-specific enolase (NSE), expressed mainly by neuronal cells, is a long-standing marker for neuronal damage. The aim of this review is to summarize, darify, and evaluate the potential significance and relationship between YKL-40 and NSE as biomarkers in the monitoring and prognosis of a set of neurological diseases, such as Alzheimer's disease, Parkinson's disease, Huntington's disease, and multiple sclerosis. YKL-40 appears to be a more reliable biomarker in neurological diseases than NSE. The more prominent expression pattern of YKL-40 could be explained with the more obvious involvement of glial cells in pathological processes accompanying each neurodegenerative disease, whereas reduced NSE levels are likely related to low metabolic activity and increased death of neurons.

C1 [Dichev, Valentin; Kazakova, Maria; Sarafian, Victoria] Med Univ Plovdiv, Dept Med Biol, Plovdiv 400, Bulgaria.

[Dichev, Valentin; Kazakova, Maria; Sarafian, Victoria] Med Univ Plovdiv, Res Inst, Plovdiv 4000, Bulgaria.

C3 Medical University Plovdiv; Medical University Plovdiv

RP Dichev, V (corresponding author), Med Univ Plovdiv, Dept Med Biol, Plovdiv 400, Bulgaria.; Dichev, V (corresponding author), Med Univ Plovdiv, Res Inst, Plovdiv 4000, Bulgaria.

EM valentin_d2@abv.bg; kazakova25@abv.bg; sarafian@abv.bg

OI Dichev, Valentin/0000-0001-5031-0136

FU National Science Fund [KP-06-PN-33/12]

FX The authors acknowledge the graphic work of Ms. Kristin Ozanian. The

review was supported by the National Science Fund project

KP-06-PN-33/12, Funder Id: http://dx.doi.org/10.13039/501100003336.

CR Abdo WF, 2004, MOVEMENT DISORD, V19, P571, DOI 10.1002/mds.10714

Mañé-Martínez MA, 2016, J NEUROIMMUNOL, V299, P112, DOI 10.1016/j.jneuroim.2016.08.004

Alcolea D, 2015, NEUROBIOL AGING, V36, P2018, DOI 10.1016/j.neurobiolaging.2015.03.001

Anderson KM, 2014, TRANSL NEURODEGENER, V3, DOI 10.1186/2047-9158-3-25

Antonell A, 2014, J ALZHEIMERS DIS, V42, P901, DOI 10.3233/JAD-140624

Baldacci F, 2017, ALZHEIMERS DEMENT, V13, P993, DOI 10.1016/j.jalz.2017.01.021

Bano D, 2011, CELL DEATH DIS, V2, DOI 10.1038/cddis.2011.112

BLENNOW K, 1994, J NEURAL TRANSM-PARK, V8, P183, DOI 10.1007/BF02260939

Bonneh-Barkay D, 2012, BRAIN PATHOL, V22, P530, DOI 10.1111/j.1750-3639.2011.00550.x

Bonneh-Barkay D, 2010, J NEUROINFLAMM, V7, DOI 10.1186/1742-2094-7-34

BROWN KW, 1980, CLIN CHIM ACTA, V101, P257, DOI 10.1016/0009-8981(80)90251-X

Bussink AP, 2007, GENETICS, V177, P959, DOI 10.1534/genetics.107.075846

Cantó E, 2015, BRAIN, V138, P918, DOI 10.1093/brain/awv017

Carabias C.S, 2019, WORLD NEUROSURG, VS1878-S8750, P32587

Charil A, 2007, J NEUROL SCI, V259, P7, DOI 10.1016/j.jns.2006.08.017

Chaves ML, 2010, J NEUROINFLAMM, V7, DOI 10.1186/1742-2094-7-6

Chitnis T, 2017, J CLIN INVEST, V127, P3577, DOI 10.1172/JCI90609

Ciancarelli I, 2015, FUNCT NEUROL, V30, P47

Ciancarelli I, 2014, BIOMARKERS, V19, P452, DOI 10.3109/1354750X.2014.935955

Comabella M, 2010, BRAIN, V133, P1082, DOI 10.1093/brain/awq035

Connor JR, 2000, OSTEOARTHR CARTILAGE, V8, P87, DOI 10.1053/joca.1999.0276

Coulson DTR, 2010, J ALZHEIMERS DIS, V22, P1111, DOI 10.3233/JAD-2010-101254

Craig-Schapiro R, 2010, BIOL PSYCHIAT, V68, P903, DOI 10.1016/j.biopsych.2010.08.025

Crews L, 2010, HUM MOL GENET, V19, pR12, DOI 10.1093/hmg/ddq160

CUNNINGHAM RT, 1994, CLIN CHIM ACTA, V230, P117, DOI 10.1016/0009-8981(94)90264-X

CUTLER NR, 1986, ARCH NEUROL-CHICAGO, V43, P153, DOI 10.1001/archneur.1986.00520020047017

Dayalu P, 2015, NEUROL CLIN, V33, P101, DOI 10.1016/j.ncl.2014.09.003

De Keyser J, 2008, J NEUROL SCI, V267, P3, DOI 10.1016/j.jns.2007.08.044

Gispert JD, 2016, NEUROBIOL AGING, V38, P47, DOI 10.1016/j.neurobiolaging.2015.10.022

Dugger BN, 2017, CSH PERSPECT BIOL, V9, DOI 10.1101/cshperspect.a028035

Francescone RA, 2011, J BIOL CHEM, V286, P15332, DOI 10.1074/jbc.M110.212514

Fusetti F, 2003, J BIOL CHEM, V278, P37753, DOI 10.1074/jbc.M303137200

Geng B, 2018, J EXP CLIN CANC RES, V37, DOI 10.1186/s13046-018-0876-2

Glass CK, 2010, CELL, V140, P918, DOI 10.1016/j.cell.2010.02.016

González H, 2014, J NEUROINFLAMM, V11, DOI 10.1186/s12974-014-0201-8

GU J, 1981, AM J PATHOL, V104, P63

Hafner A, 2012, BIOCHEM J, V443, P439, DOI 10.1042/BJ20111351

HAIMOTO H, 1985, LAB INVEST, V52, P257

Håkansson I, 2017, EUR J NEUROL, V24, P703, DOI 10.1111/ene.13274

Hall S, 2016, MOVEMENT DISORD, V31, P898, DOI 10.1002/mds.26578

Haque A, 2018, BRAIN SCI, V8, DOI 10.3390/brainsci8020033

He CH, 2013, CELL REP, V4, P830, DOI 10.1016/j.celrep.2013.07.032

Hein K, 2008, NEUROSCI LETT, V436, P72, DOI 10.1016/j.neulet.2008.02.064

Hinsinger G, 2015, MULT SCLER J, V21, P1251, DOI 10.1177/1352458514561906

Houston DR, 2003, J BIOL CHEM, V278, P30206, DOI 10.1074/jbc.M303371200

KAISER E, 1989, CLIN CHIM ACTA, V183, P13, DOI 10.1016/0009-8981(89)90268-4

KATO K, 1983, CLIN CHIM ACTA, V127, P353, DOI 10.1016/0009-8981(83)90162-6

Kazakova M., 2013, BIOMED REV, V24, P49, DOI [10.14748/bmr.v24.21, DOI 10.14748/BMR.V24.21]

Kinney Jefferson W, 2018, Alzheimers Dement (N Y), V4, P575, DOI 10.1016/j.trci.2018.06.014

Koch MW, 2015, J NEUROL SCI, V350, P46, DOI 10.1016/j.jns.2015.02.009

Kognole AA, 2017, J BIOL CHEM, V292, P2624, DOI 10.1074/jbc.M116.764985

Lamers KJB, 2003, BRAIN RES BULL, V61, P261, DOI 10.1016/S0361-9230(03)00089-3

Lautner Ronald, 2011, Int J Alzheimers Dis, V2011, P939426, DOI 10.4061/2011/939426

Lee CM, 2016, NAT COMMUN, V7, DOI 10.1038/ncomms12752

Lee CG, 2010, ALLERGY ASTHMA IMMUN, V2, P20, DOI 10.4168/aair.2010.2.1.20

Lee CG, 2009, J EXP MED, V206, P1149, DOI 10.1084/jem.20081271

Levin MC, 2014, DEGENER NEUROL NEURO, V4, P49, DOI 10.2147/DNND.S54391

Llorens F, 2017, MOL NEURODEGENER, V12, DOI 10.1186/s13024-017-0226-4

Llorens F, 2017, FRONT AGING NEUROSCI, V9, DOI 10.3389/fnagi.2017.00289

Lucas SM, 2006, BRIT J PHARMACOL, V147, pS232, DOI 10.1038/sj.bjp.0706400

Magdalinou NK, 2015, J NEUROL NEUROSUR PS, V86, P1240, DOI 10.1136/jnnp-2014-309562

Malmeström C, 2014, J NEUROIMMUNOL, V269, P87, DOI 10.1016/j.jneuroim.2014.02.004

MARANGOS PJ, 1978, J NEUROCHEM, V31, P727, DOI 10.1111/j.1471-4159.1978.tb07847.x

MARANGOS PJ, 1987, ANNU REV NEUROSCI, V10, P269, DOI 10.1146/annurev.ne.10.030187.001413

MARANGOS PJ, 1980, BRAIN RES, V190, P185, DOI 10.1016/0006-8993(80)91168-3

MARANGOS PJ, 1981, J NEUROCHEM, V37, P1338, DOI 10.1111/j.1471-4159.1981.tb04687.x

Nam SJ, 2016, KOREAN J INTERN MED, V31, P694, DOI 10.3904/kjim.2015.407

Ngernyuang N, 2018, NEOPLASIA, V20, P182, DOI 10.1016/j.neo.2017.11.011

Niemelä V, 2018, PLOS ONE, V13, DOI 10.1371/journal.pone.0193492

Noelker C, 2011, MOL DIAGN THER, V15, P83, DOI 10.2165/11534870-000000000-00000

Nooijen PTGA, 1997, DEMENT GERIATR COGN, V8, P169, DOI 10.1159/000106627

Nordengen K, 2019, J NEUROINFLAMM, V16, DOI 10.1186/s12974-019-1399-2

Ohno M, 2015, INT J MOL SCI, V16, P9922, DOI 10.3390/ijms16059922

Öhrfelt A, 2016, DEMENT GER COGN D EX, V6, P283, DOI 10.1159/000447239

OLIVA D, 1991, GENOMICS, V10, P157, DOI 10.1016/0888-7543(91)90496-2

Olsson B, 2013, MOVEMENT DISORD, V28, P1882, DOI 10.1002/mds.25589

Omerhoca Sami, 2018, Noro Psikiyatr Ars, V55, pS1, DOI 10.29399/npa.23418

Palumbo B, 2008, FUNCT NEUROL, V23, P93

Park HY, 2012, PLOS ONE, V7, DOI 10.1371/journal.pone.0051722

PARNETTI L, 1995, NEUROSCI LETT, V183, P43

Pouyafar A, 2018, BIOMED PHARMACOTHER, V100, P478, DOI 10.1016/j.biopha.2018.02.050

Prakash M, 2013, CELL SIGNAL, V25, P1567, DOI 10.1016/j.cellsig.2013.03.016

Querol-Vilaseca M, 2017, J NEUROINFLAMM, V14, DOI 10.1186/s12974-017-0893-7

Recklies AD, 2005, J BIOL CHEM, V280, P41213, DOI 10.1074/jbc.M510146200

Rehli M, 2003, J BIOL CHEM, V278, P44058, DOI 10.1074/jbc.M306792200

Rehli M, 1997, GENOMICS, V43, P221, DOI 10.1006/geno.1997.4778

Renkema GH, 1998, EUR J BIOCHEM, V251, P504, DOI 10.1046/j.1432-1327.1998.2510504.x

Ringsholt M, 2007, J MOL HISTOL, V38, P33, DOI 10.1007/s10735-006-9075-0

Rodrigues FB, 2016, PLOS ONE, V11, DOI 10.1371/journal.pone.0163479

Rubinsztein DC, 2006, NATURE, V443, P780, DOI 10.1038/nature05291

Schaf DV, 2005, PARKINSONISM RELAT D, V11, P39, DOI 10.1016/j.parkreldis.2004.07.002

SCHMECHEL D, 1978, SCIENCE, V199, P313, DOI 10.1126/science.339349

SCHMECHEL DE, 1987, NEUROSCI LETT, V76, P233, DOI 10.1016/0304-3940(87)90721-X

Schmidt FM, 2014, NEUROSCI LETT, V570, P81, DOI 10.1016/j.neulet.2014.04.007

Shao R, 2009, ONCOGENE, V28, P4456, DOI 10.1038/onc.2009.292

Shao R, 2015, ONCOTARGET, V6, P40507, DOI 10.18632/oncotarget.5943

Sladkova V, 2011, NEUROL RES, V33, P415, DOI 10.1179/016164110X12816242542535

Smith JA, 2012, BRAIN RES BULL, V87, P10, DOI 10.1016/j.brainresbull.2011.10.004

Spangenberg EE, 2017, BRAIN BEHAV IMMUN, V61, P1, DOI 10.1016/j.bbi.2016.07.003

Strimbu K, 2010, CURR OPIN HIV AIDS, V5, P463, DOI 10.1097/COH.0b013e32833ed177

SULKAVA R, 1988, J NEUROL NEUROSUR PS, V51, P549, DOI 10.1136/jnnp.51.4.549

Villar-Piqué A, 2019, J NEUROINFLAMM, V16, DOI 10.1186/s12974-019-1531-3

Vinther-Jensen T, 2014, PARKINSONISM RELAT D, V20, P1301, DOI 10.1016/j.parkreldis.2014.08.011

Vizin T, 2015, RADIOL ONCOL, V49, P217, DOI 10.1515/raon-2015-0035

Wenning GK, 1997, BAILLIERE CLIN NEUR, V6, P187

Wennström M, 2015, PLOS ONE, V10, DOI 10.1371/journal.pone.0135458

Yamanaka K, 2018, NEUROSCI RES, V126, P31, DOI 10.1016/j.neures.2017.09.011

Zeng XS, 2018, FRONT AGING NEUROSCI, V10, DOI 10.3389/fnagi.2018.00109

Zhou Y, 2018, J IMMUNOL, V200, P2140, DOI 10.4049/jimmunol.1701442

Zhou Y, 2015, J CLIN INVEST, V125, P3178, DOI 10.1172/JCI79792

NR 110

TC 20

Z9 21

U1 0

U2 5

PU WALTER DE GRUYTER GMBH

PI BERLIN

PA GENTHINER STRASSE 13, D-10785 BERLIN, GERMANY

SN 0334-1763

EI 1607-8470

J9 REV NEUROSCIENCE

JI Rev. Neurosci.

PD JUL

PY 2020

VL 31

IS 5

BP 539

EP 553

DI 10.1515/revneuro-2019-0100

PG 15

WC Neurosciences

WE Science Citation Index Expanded (SCI-EXPANDED)

SC Neurosciences & Neurology

GA MP1XN

UT WOS:000552004400004

PM 32045356

DA 2024-08-12

ER

PT J

AU Russo, C

Valle, MS

Casabona, A

Malaguarnera, L

AF Russo, Cristina

Valle, Maria Stella

Casabona, Antonino

Malaguarnera, Lucia

TI Chitinase Signature in the Plasticity of neurodegenerative disease

SO INTERNATIONAL JOURNAL OF MOLECULAR SCIENCES

LA English

DT Review

DE Alzheimer's disease; Parkinson's disease; amyotrophic lateral sclerosis;

multiple sclerosis; glycohydrolase family 18; neuroinflammation;

Chitotriosidase; chitinase-3-like 1

ID AMYOTROPHIC-LATERAL-SCLEROSIS; CEREBROSPINAL-FLUID; alzheimer's disease;

ALPHA-SYNUCLEIN; MULTIPLE-SCLEROSIS; TRANSCRIPTIONAL REGULATION; LOBAR

DEGENERATION; YKL-40; EXPRESSION; CHITOTRIOSIDASE

AB Several reports have pointed out that Chitinases are expressed and secreted by various cell types of central nervous system (CNS), including activated microglia and astrocytes. These cells play a key role in neuroinflammation and in the pathogenesis of many neurodegenerative disorders. Increased levels of Chitinases, in particular Chitotriosidase (CHIT-1) and chitinase-3-like protein 1 (CHI3L1), have been found increased in several neurodegenerative disorders. Although having important biological roles in inflammation, to date, the molecular mechanisms of Chitinase involvement in the pathogenesis of neurodegenerative disorders is not well-elucidated. Several studies showed that some Chitinases could be assumed as markers for diagnosis, prognosis, activity, and severity of a disease and therefore can be helpful in the choice of treatment. However, some studies showed controversial results. This review will discuss the potential of Chitinases in the pathogenesis of some neurodegenerative disorders, such as Alzheimer's disease, Parkinson's disease, amyotrophic lateral sclerosis, and multiple sclerosis, to understand their role as distinctive biomarkers of neuronal cell activity during neuroinflammatory processes. Knowledge of the role of Chitinases in neuronal cell activation could allow for the development of new methodologies for downregulating neuroinflammation and consequently for diminishing negative neurological disease outcomes.

C1 [Russo, Cristina; Malaguarnera, Lucia] Univ Catania, Sch Med, Dept Biomed & Biotechnol Sci, Sect Pathol, I-95123 Catania, Italy.

[Valle, Maria Stella; Casabona, Antonino] Univ Catania, Sch Med, Dept Biomed & Biotechnol Sci, Sect Physiol,Lab Neurobiomech, I-95123 Catania, Italy.

C3 University of Catania; University of Catania

RP Valle, MS (corresponding author), Univ Catania, Sch Med, Dept Biomed & Biotechnol Sci, Sect Physiol,Lab Neurobiomech, I-95123 Catania, Italy.

EM m.valle@unict.it

RI Malaguarnera, Lucia/K-5530-2018; valle, maria/KII-1654-2024

OI MALAGUARNERA, Lucia/0000-0002-5171-7963; VALLE, Maria

Stella/0000-0001-9728-3542; Casabona, Antonino/0000-0003-0180-4695

FU PNRR: "Health Extended Alliance for Innovative Therapies, Advanced

Lab-research, and Integrated Approaches of Precision" [CUP:

E63C22002080006]

FX This research was founded by PNRR: "Health Extended Alliance for

Innovative Therapies, Advanced Lab-research, and Integrated Approaches

of Precision" CUP: E63C22002080006.

CR Al-Chalabi A, 2016, LANCET NEUROL, V15, P1182, DOI 10.1016/S1474-4422(16)30199-5

Al-Chalabi A, 2013, NAT REV NEUROL, V9, P617, DOI 10.1038/nrneurol.2013.203

Alarcón TA, 2023, NEURAL REGEN RES, V18, P1450, DOI 10.4103/1673-5374.360264

Alcolea D, 2015, NEUROBIOL AGING, V36, P2018, DOI 10.1016/j.neurobiolaging.2015.03.001

Antonell A, 2014, J ALZHEIMERS DIS, V42, P901, DOI 10.3233/JAD-140624

Bartl M, 2021, PLOS ONE, V16, DOI 10.1371/journal.pone.0257372

Baxter PS, 2021, CELL REP, V34, DOI 10.1016/j.celrep.2021.108882

Beers DR, 2019, LANCET NEUROL, V18, P211, DOI 10.1016/S1474-4422(18)30394-6

Bellaver B, 2021, NEUROLOGY, V96, pE2944, DOI 10.1212/WNL.0000000000012109

Bi FF, 2013, P NATL ACAD SCI USA, V110, P4069, DOI 10.1073/pnas.1218497110

Bilican B, 2012, P NATL ACAD SCI USA, V109, P5803, DOI 10.1073/pnas.1202922109

Bjornevik K, 2023, NAT REV NEUROL, V19, P160, DOI 10.1038/s41582-023-00775-5

Boche D, 2013, NEUROPATH APPL NEURO, V39, P3, DOI 10.1111/nan.12011

Bolós M, 2017, MOL NEURODEGENER, V12, DOI 10.1186/s13024-017-0200-1

Bonneh-Barkay D, 2010, J NEUROINFLAMM, V7, DOI 10.1186/1742-2094-7-34

Bouvier DS, 2022, FRONT PHYSIOL, V13, DOI 10.3389/fphys.2022.814889

Brück D, 2016, NEUROBIOL DIS, V85, P262, DOI 10.1016/j.nbd.2015.03.003

Burrell JR, 2016, LANCET, V388, P919, DOI 10.1016/S0140-6736(16)00737-6

Carter SF, 2019, TRENDS MOL MED, V25, P77, DOI 10.1016/j.molmed.2018.11.006

Casal JA, 2003, CLIN BIOCHEM, V36, P553, DOI 10.1016/S0009-9120(03)00093-6

Cashion JM, 2023, NEUROBIOL DIS, V178, DOI 10.1016/j.nbd.2023.106028

Castellani RJ, 2007, NEUROTOX RES, V12, P269, DOI 10.1007/BF03033910

Castellani Rudy J., 2005, Current Alzheimer Research, V2, P419, DOI 10.2174/156720505774330555

Choi J, 2011, J NEUROL, V258, P2181, DOI 10.1007/s00415-011-6087-9

Chow HH, 2018, BRAIN BEHAV, V8, DOI 10.1002/brb3.875

Comabella M, 2010, BRAIN, V133, P1082, DOI 10.1093/brain/awq035

Cossburn MD, 2013, NEUROLOGY, V80, P55, DOI 10.1212/WNL.0b013e31827b5927

Costa J, 2021, DIAGNOSTICS, V11, DOI 10.3390/diagnostics11071210

Craig-Schapiro R, 2010, BIOL PSYCHIAT, V68, P903, DOI 10.1016/j.biopsych.2010.08.025

Da Silva CA, 2009, J IMMUNOL, V182, P3573, DOI 10.4049/jimmunol.0802113

de Boer EMJ, 2021, J NEUROL NEUROSUR PS, V92, P86, DOI 10.1136/jnnp-2020-322983

De Fino C, 2019, MULT SCLER RELAT DIS, V35, P176, DOI 10.1016/j.msard.2019.07.030

Degelman ML, 2017, MULT SCLER RELAT DIS, V17, P207, DOI 10.1016/j.msard.2017.07.020

Dettmer U, 2016, CURR OPIN NEUROBIOL, V36, P15, DOI 10.1016/j.conb.2015.07.007

Dharshini SAP, 2021, FRONT GENET, V12, DOI 10.3389/fgene.2021.639160

Di Rosa M, 2006, EUR J NEUROSCI, V23, P2648, DOI 10.1111/j.1460-9568.2006.04780.x

Di Rosa M, 2016, PATHOBIOLOGY, V83, P228, DOI 10.1159/000444855

Di Rosa M, 2016, PATHOBIOLOGY, V83, P211, DOI 10.1159/000443932

Di Rosa M, 2016, IMMUNOBIOLOGY, V221, P399, DOI 10.1016/j.imbio.2015.11.014

Di Rosa M, 2014, BONE, V61, P55, DOI 10.1016/j.bone.2014.01.005

Di Rosa M, 2013, MOL CELL BIOCHEM, V374, P73, DOI 10.1007/s11010-012-1506-5

Dichev V, 2020, REV NEUROSCIENCE, V31, P539, DOI 10.1515/revneuro-2019-0100

Dickson, 2017, CSH PERSPECT BIOL, V9, pa028035, DOI DOI 10.1101/CSHPERSPECT.A028035

Gispert JD, 2016, NEUROBIOL AGING, V38, P47, DOI 10.1016/j.neurobiolaging.2015.10.022

Duits FH, 2015, J ALZHEIMERS DIS, V48, P711, DOI 10.3233/JAD-143186

Escartin C, 2021, NAT NEUROSCI, V24, P312, DOI 10.1038/s41593-020-00783-4

Farhat SM, 2017, CURR DRUG TARGETS, V18, P1537, DOI 10.2174/1389450117666161005145143

Farina C, 2007, TRENDS IMMUNOL, V28, P138, DOI 10.1016/j.it.2007.01.005

Fellner L, 2011, ACTA NEUROPATHOL, V121, P675, DOI 10.1007/s00401-011-0833-z

Ferrari-Souza JP, 2022, MOL PSYCHIATR, V27, P4781, DOI 10.1038/s41380-022-01716-2

Gaur N, 2020, FRONT NEUROL, V11, DOI 10.3389/fneur.2020.00377

Gille B, 2019, J NEUROL NEUROSUR PS, V90, P1338, DOI 10.1136/jnnp-2018-319586

Gitler AD, 2017, DIS MODEL MECH, V10, P499, DOI 10.1242/dmm.030205

Gray E, 2020, ANN CLIN TRANSL NEUR, V7, P1296, DOI 10.1002/acn3.51114

Habib N, 2020, NAT NEUROSCI, V23, P701, DOI 10.1038/s41593-020-0624-8

Hansen DV, 2018, J CELL BIOL, V217, P459, DOI 10.1083/jcb.201709069

Hendrickx DAE, 2017, J NEUROIMMUNOL, V309, P12, DOI 10.1016/j.jneuroim.2017.04.007

Heneka MT, 2015, LANCET NEUROL, V14, P388, DOI 10.1016/S1474-4422(15)70016-5

Heywood WE, 2015, MOL NEURODEGENER, V10, DOI 10.1186/s13024-015-0059-y

Hinsinger G, 2015, MULT SCLER J, V21, P1251, DOI 10.1177/1352458514561906

Hol EM, 2015, CURR OPIN CELL BIOL, V32, P121, DOI 10.1016/j.ceb.2015.02.004

Huang C, 2011, PLOS GENET, V7, DOI 10.1371/journal.pgen.1002011

Huang J, 2022, MULT SCLER RELAT DIS, V68, DOI 10.1016/j.msard.2022.104250

Im JH, 2020, EXP NEUROL, V323, DOI 10.1016/j.expneurol.2019.113082

Irwin DJ, 2013, NAT REV NEUROSCI, V14, P626, DOI 10.1038/nrn3549

Iyer SS, 2012, CRIT REV IMMUNOL, V32, P23, DOI 10.1615/CritRevImmunol.v32.i1.30

Janelidze S, 2016, ANN CLIN TRANSL NEUR, V3, P12, DOI 10.1002/acn3.266

Johansen JS, 2006, DAN MED BULL, V53, P172

Kester MI, 2015, ALZHEIMERS RES THER, V7, DOI 10.1186/s13195-015-0142-1

Kim MN, 2012, BIOCHEM BIOPH RES CO, V421, P790, DOI 10.1016/j.bbrc.2012.04.085

Klegeris A, 2008, NEUROBIOL AGING, V29, P739, DOI 10.1016/j.neurobiolaging.2006.11.013

Kovacs GG, 2019, J CLIN PATHOL, V72, P725, DOI 10.1136/jclinpath-2019-205952

Kumar A, 2015, PHARMACOL REP, V67, P195, DOI 10.1016/j.pharep.2014.09.004

Lassmann H, 2019, FRONT IMMUNOL, V9, DOI 10.3389/fimmu.2018.03116

Lee CG, 2011, ANNU REV PHYSIOL, V73, P479, DOI 10.1146/annurev-physiol-012110-142250

Lee HJ, 2010, J BIOL CHEM, V285, P9262, DOI 10.1074/jbc.M109.081125

Li X, 2005, FEBS J, V272, P4211, DOI 10.1111/j.1742-4658.2005.04833.x

Li Y, 2023, J NEUROL, V270, P1487, DOI 10.1007/s00415-022-11476-2

Libreros S, 2012, INT J CANCER, V131, P377, DOI 10.1002/ijc.26379

Lindström V, 2017, MOL CELL NEUROSCI, V82, P143, DOI 10.1016/j.mcn.2017.04.009

Lu SZ, 2019, TRANSL NEURODEGENER, V8, DOI 10.1186/s40035-018-0143-7

Lv QK, 2023, INFLAMM RES, V72, P443, DOI 10.1007/s00011-022-01676-x

Malaguarnera L, 2006, CELL MOL LIFE SCI, V63, P3018, DOI 10.1007/s00018-006-6269-2

Malaguarnera L, 2006, GUT, V55, P1313, DOI 10.1136/gut.2005.075697

Mattsson N, 2011, NEUROMOL MED, V13, P151, DOI 10.1007/s12017-011-8147-9

McLarnon JG., 2016, J ALZHEIMERS DIS PAR, V6, P273, DOI [10.4172/2161-0460.1000273, DOI 10.4172/2161-0460.1000273]

Meade RM, 2019, MOL NEURODEGENER, V14, DOI 10.1186/s13024-019-0329-1

Morenas-Rodríguez E, 2019, SCI REP-UK, V9, DOI 10.1038/s41598-019-44173-8

Murphy GM, 2000, AM J PATHOL, V157, P895, DOI 10.1016/S0002-9440(10)64603-2

Neumann M, 2006, SCIENCE, V314, P130, DOI 10.1126/science.1134108

Novakova L, 2017, J NEUROCHEM, V141, P296, DOI 10.1111/jnc.13881

Oeckl P, 2019, J NEUROL NEUROSUR PS, V90, P4, DOI 10.1136/jnnp-2018-318868

Oldoni E, 2020, ANN NEUROL, V87, P633, DOI 10.1002/ana.25691

Olsson B, 2013, J ALZHEIMERS DIS, V33, P45, DOI 10.3233/JAD-2012-120787

Orihuela R, 2016, BRIT J PHARMACOL, V173, P649, DOI 10.1111/bph.13139

Pepe G, 2014, J NEUROINFLAMM, V11, DOI 10.1186/s12974-014-0211-6

Perez-Nievas BG, 2018, FRONT AGING NEUROSCI, V10, DOI 10.3389/fnagi.2018.00114

Petrzalka M, 2022, PLOS ONE, V17, DOI 10.1371/journal.pone.0270607

Pinteac R, 2021, NEUROL-NEUROIMMUNOL, V8, DOI 10.1212/NXI.0000000000000921

Ponath G, 2018, FRONT IMMUNOL, V9, DOI 10.3389/fimmu.2018.00217

Querol-Vilaseca M, 2017, J NEUROINFLAMM, V14, DOI 10.1186/s12974-017-0893-7

Rehli M, 2003, J BIOL CHEM, V278, P44058, DOI 10.1074/jbc.M306792200

Rehli M, 1997, GENOMICS, V43, P221, DOI 10.1006/geno.1997.4778

Rosén C, 2014, DEMENT GER COGN D EX, V4, P297, DOI 10.1159/000362164

Russo C, 2022, INT J MOL SCI, V23, DOI 10.3390/ijms232113432

Ryan M, 2019, JAMA NEUROL, V76, P1367, DOI 10.1001/jamaneurol.2019.2044

Saitgareeva AR, 2020, NEUROL SCI, V41, P3609, DOI 10.1007/s10072-020-04468-5

Samaha DY, 2023, Egypt J Immunol, V30, P42, DOI [10.55133/eji.300105, DOI 10.55133/EJI.300105]

Schneider R, 2021, NEUROL-NEUROIMMUNOL, V8, DOI 10.1212/NXI.0000000000000906

Sezgin M, 2019, SEMIN NEUROL, V39, P274, DOI 10.1055/s-0039-1678579

Steinacker P, 2021, AMYOTROPH LAT SCL FR, V22, P276, DOI 10.1080/21678421.2020.1861023

Steinacker P, 2018, J NEUROL NEUROSUR PS, V89, P239, DOI 10.1136/jnnp-2017-317138

Surendranathan A, 2015, PARKINSONISM RELAT D, V21, P1398, DOI 10.1016/j.parkreldis.2015.10.009

Sutphen CL, 2015, JAMA NEUROL, V72, P1029, DOI 10.1001/jamaneurol.2015.1285

Suzuki N, 2023, J HUM GENET, V68, P131, DOI 10.1038/s10038-022-01055-8

Swarup V, 2011, BRAIN, V134, P2610, DOI 10.1093/brain/awr159

Tan RH, 2017, ACTA NEUROPATHOL, V133, P177, DOI 10.1007/s00401-016-1666-6

Tang Y, 2016, MOL NEUROBIOL, V53, P1181, DOI 10.1007/s12035-014-9070-5

Thompson AG, 2019, J NEUROL NEUROSUR PS, V90, P1215, DOI 10.1136/jnnp-2019-320442

Tong JB, 2013, EMBO J, V32, P1917, DOI 10.1038/emboj.2013.122

Turano E, 2015, NEUROBIOL AGING, V36, P1780, DOI 10.1016/j.neurobiolaging.2014.12.033

Uchida A, 2012, BRAIN, V135, P833, DOI 10.1093/brain/awr348

Villar-Piqué A, 2019, J NEUROINFLAMM, V16, DOI 10.1186/s12974-019-1531-3

Vu L, 2020, J NEUROL NEUROSUR PS, V91, P350, DOI 10.1136/jnnp-2019-321916

Watabe-Rudolph M, 2012, NEUROLOGY, V78, P569, DOI 10.1212/WNL.0b013e318247caa1

Wattmo C, 2020, BMC NEUROL, V20, DOI 10.1186/s12883-019-1591-0

Wennström M, 2015, PLOS ONE, V10, DOI 10.1371/journal.pone.0135458

Wills-Karp M, 2010, MUCOSAL IMMUNOL, V3, P104, DOI 10.1038/mi.2009.138

Wu LS, 2012, J BIOL CHEM, V287, P27335, DOI 10.1074/jbc.M112.359000

Yu XY, 2021, NEUROSCI RES, V172, P73, DOI 10.1016/j.neures.2021.05.014

Zhang K, 2013, PLOS ONE, V8, DOI 10.1371/journal.pone.0072744

Zhao T, 2020, SIGNAL TRANSDUCT TAR, V5, DOI 10.1038/s41392-020-00303-7

Ziemssen T, 2017, AUTOIMMUN REV, V16, P355, DOI 10.1016/j.autrev.2017.02.007

Zrzavy T, 2017, BRAIN, V140, P1900, DOI 10.1093/brain/awx113

NR 134

TC 5

Z9 5

U1 1

U2 7

PU MDPI

PI BASEL

PA ST ALBAN-ANLAGE 66, CH-4052 BASEL, SWITZERLAND

SN 1661-6596

EI 1422-0067

J9 INT J MOL SCI

JI Int. J. Mol. Sci.

PD APR

PY 2023

VL 24

IS 7

AR 6301

DI 10.3390/ijms24076301

PG 18

WC Biochemistry & Molecular Biology; Chemistry, Multidisciplinary

WE Science Citation Index Expanded (SCI-EXPANDED)

SC Biochemistry & Molecular Biology; Chemistry

GA D7DR8

UT WOS:000970300700001

PM 37047273

OA Green Published, gold

DA 2024-08-12

ER

PT J

AU Kartik, S

Pal, R

Chaudhary, MJ

Nath, R

Kumar, M

Binwal, M

Bawankule, DU

AF Kartik, Shipra

Pal, Rishi

Chaudhary, Manju J.

Nath, Rajendra

Kumar, Madhu

Binwal, Monika

Bawankule, D. U.

TI Neuroprotective role of chloroquine via modulation of autophagy and

neuroinflammation in MPTP-induced Parkinson's disease

SO INFLAMMOPHARMACOLOGY

LA English

DT Article

DE Parkinson's disease; MPTP; Autophagy; Neuroinflammation; Chloroquine

ID CELL-DEATH; OXIDATIVE STRESS; MITOCHONDRIAL DYSFUNCTION; MECHANISMS;

INFLAMMATION; SUPPRESSION; APOPTOSIS; SYSTEM; ASSAY

AB Parkinson's disease (PD) is a neuro-motor ailment that strikes adults in their older life and results in both motor and non-motor impairments. In neuronal and glial cells, PD has recently been linked to a dysregulated autophagic system and cerebral inflammation. Chloroquine (CQ), an anti-malarial drug, has been demonstrated to suppress autophagy in a variety of diseases, including cerebral ischemia, Alzheimer's disease (AD), and Traumatic brain injury (TBI), while its involvement in PD is still unclear. BALB/c mice were randomly allocated to one of four groups: 1-methyl-4-phenyl-1, 2, 3, 6-tetrahydropyridine (MPTP), CQ treatment with or without MPTP, or control. The CQ treatment group received CQ (intraperitoneally, 8 mg/kg body weight) after 1 h of MPTP induction on day 1, and it lasted for 7 days. CQ therapy preserves dopamine levels stable, inhibits tyrosine hydroxylase (TH) positive dopaminergic cell death, and lowers oxidative stress. CQ reduces the behavioural, motor, and cognitive deficits caused by MPTP after injury. Furthermore, CQ therapy slowed aberrant neuronal autophagy (microtubule-associated protein-1 light chain 3B; LC3B & Beclin1) and lowered expression levels of the inflammatory cytokines interleukin 1 (IL-1 beta) and tumour necrosis factor (TNF-alpha) in the mice brain. In addition, CQ's antioxidant and anti-inflammatory effects were also tested in MPTP-mediated cell death in PC12 cells, demonstrating that CQ has a neurorestorative impact by successfully rescuing MPTP-induced ROS generation and cell loss. Our findings show that CQ's can help to prevent dopaminergic degeneration and improve neurological function after MPTP intoxication by lowering the harmful effects of neuronal autophagy and cerebral inflammation.

C1 [Kartik, Shipra; Pal, Rishi; Nath, Rajendra] King Georges Med Univ, Dept Pharmacol & Therapeut, Lucknow 226003, Uttar Pradesh, India.

[Chaudhary, Manju J.] Govt Med Coll, Dept Physiol, Tirwa Rd, Kannauj, Uttar Pradesh, India.

[Kumar, Madhu] King Georges Med Univ, Dept Pathol, Lucknow 226003, Uttar Pradesh, India.

[Binwal, Monika; Bawankule, D. U.] CSIR Cent Inst Med & Aromat Plants, Bioprospect & Prod Dev Div, Lucknow 226015, Uttar Pradesh, India.

C3 King George's Medical University; King George's Medical University;

Council of Scientific & Industrial Research (CSIR) - India; CSIR -

Central Institute of Medicinal & Aromatic Plants (CIMAP)

RP Pal, R (corresponding author), King Georges Med Univ, Dept Pharmacol & Therapeut, Lucknow 226003, Uttar Pradesh, India.

EM rishipal@kgmcindia.edu

RI Pal, Rishi/AAX-9512-2021; Pal, Rishi/HPE-4303-2023

OI Pal, Rishi/0000-0001-9813-6990; Kartik, Shipra/0000-0002-4775-3523

FU Indian Council of Medical Research (ICMR), New Delhi, India

[3/1/2/99/Neuro/2018-NCD-I]

FX The Indian Council of Medical Research (ICMR), New Delhi, India, awarded

Ms. Shipra Kartik a senior research fellowship

(Project-3/1/2/99/Neuro/2018-NCD-I).

CR An Lifeng, 2010, Zhongguo Zhong Yao Za Zhi, V35, P2021

Bae WY, 2018, INT J MOL SCI, V19, DOI 10.3390/ijms19020551

Baehrecke EH, 2005, NAT REV MOL CELL BIO, V6, P505, DOI 10.1038/nrm1666

BEERS RF, 1952, J BIOL CHEM, V195, P133

Brooks SP, 2009, NAT REV NEUROSCI, V10, P519, DOI 10.1038/nrn2652

Bursch W, 2001, CELL DEATH DIFFER, V8, P569, DOI 10.1038/sj.cdd.4400852

CALNE DB, 1983, LANCET, V2, P1457, DOI 10.1016/S0140-6736(83)90802-4

Canini F, 2009, NEUROSCI LETT, V457, P41, DOI 10.1016/j.neulet.2009.03.103

CARLBERG I, 1985, METHOD ENZYMOL, V113, P484

Castillo V, 2015, PLOS ONE, V10, DOI 10.1371/journal.pone.0136620

Castino R, 2008, J NEUROCHEM, V106, P1426, DOI 10.1111/j.1471-4159.2008.05488.x

Chen Y, 2008, CELL DEATH DIFFER, V15, P171, DOI 10.1038/sj.cdd.4402233

Cherra SJ, 2008, FUTUR NEUROL, V3, P309, DOI 10.2217/14796708.3.3.309

Cui CM, 2015, MOL MED REP, V12, P2323, DOI 10.3892/mmr.2015.3611

Dadakhujaev S, 2010, NEUROSCI LETT, V472, P47, DOI 10.1016/j.neulet.2010.01.053

Dagda RK, 2013, INT J MOL SCI, V14, P22163, DOI 10.3390/ijms141122163

DAMATO RJ, 1987, NATURE, V327, P324, DOI 10.1038/327324a0

Das G, 2012, CSH PERSPECT BIOL, V4, DOI 10.1101/cshperspect.a008813

Dauer W, 2003, NEURON, V39, P889, DOI 10.1016/S0896-6273(03)00568-3

Di Malta C, 2012, AUTOPHAGY, V8, P1871, DOI 10.4161/auto.22184

Dickson DW, 2007, AM J PATHOL, V170, P16, DOI 10.2353/ajpath.2007.061011

Dong XX, 2009, ACTA PHARMACOL SIN, V30, P379, DOI 10.1038/aps.2009.24

Du TT, 2010, INT J TOXICOL, V29, P625, DOI 10.1177/1091581810383705

Dupont N, 2011, EMBO J, V30, P4701, DOI 10.1038/emboj.2011.398

Ghavami S, 2014, PROG NEUROBIOL, V112, P24, DOI 10.1016/j.pneurobio.2013.10.004

Glass CK, 2010, CELL, V140, P918, DOI 10.1016/j.cell.2010.02.016

Hedya SA, 2019, BEHAV BRAIN RES, V365, P82, DOI 10.1016/j.bbr.2019.02.033

Hirata Y, 2011, J NEUROCHEM, V119, P839, DOI 10.1111/j.1471-4159.2011.07464.x

HOMEWOOD CA, 1972, NATURE, V235, P50, DOI 10.1038/235050a0

Hung KC, 2014, PLOS ONE, V9, DOI 10.1371/journal.pone.0091074

Islam MT, 2017, NEUROL RES, V39, P73, DOI 10.1080/01616412.2016.1251711

Janku F, 2011, NAT REV CLIN ONCOL, V8, P528, DOI 10.1038/nrclinonc.2011.71

Jiang PZ, 2018, ACTA NEUROPATHOL, V135, P13, DOI 10.1007/s00401-017-1788-5

Kim CH, 2015, P NATL ACAD SCI USA, V112, P8756, DOI 10.1073/pnas.1509742112

Kimura T, 2013, CANCER RES, V73, P3, DOI 10.1158/0008-5472.CAN-12-2464

Kuma A, 2004, NATURE, V432, P1032, DOI 10.1038/nature03029

Law BYK, 2014, SCI REP-UK, V4, DOI 10.1038/srep05510

Lin MT, 2006, NATURE, V443, P787, DOI 10.1038/nature05292

Liu CL, 2010, J NEUROCHEM, V115, P68, DOI 10.1111/j.1471-4159.2010.06905.x

Liu J, 2018, AUTOPHAGY, V14, P845, DOI 10.1080/15548627.2017.1390636

Lopez G, 2011, AUTOPHAGY, V7, P440, DOI 10.4161/auto.7.4.14680

Ma SM, 2017, PLOS ONE, V12, DOI 10.1371/journal.pone.0182921

Manna S, 2006, J VET SCI, V7, P133, DOI 10.4142/jvs.2006.7.2.133

MARKLUND S, 1974, EUR J BIOCHEM, V47, P469

McGeer PL, 2004, ANN NY ACAD SCI, V1035, P104, DOI 10.1196/annals.1332.007

Mizushima N, 2008, NATURE, V451, P1069, DOI 10.1038/nature06639

Moon HE, 2015, EXP NEUROBIOL, V24, P103, DOI 10.5607/en.2015.24.2.103

Nash Y, 2017, J NEUROCHEM, V143, P584, DOI 10.1111/jnc.14222

Niranjan R, 2014, MOL NEUROBIOL, V49, P28, DOI 10.1007/s12035-013-8483-x

NISHI K, 1989, BRAIN RES, V489, P157, DOI 10.1016/0006-8993(89)90018-8

Noldus LPJJ, 2001, BEHAV RES METH INS C, V33, P398, DOI 10.3758/BF03195394

Nopparat C, 2014, NEUROCHEM RES, V39, P225, DOI 10.1007/s11064-013-1208-8

OHKAWA H, 1979, ANAL BIOCHEM, V95, P351, DOI 10.1016/0003-2697(79)90738-3

Parekh P, 2019, ACS CHEM NEUROSCI, V10, P3914, DOI 10.1021/acschemneuro.9b00244

Rocha NP, 2015, BIOMED RES INT, V2015, DOI 10.1155/2015/628192

Rubinszfein DC, 2005, AUTOPHAGY, V1, P11, DOI 10.4161/auto.1.1.1513

SINHA AK, 1972, ANAL BIOCHEM, V47, P389, DOI 10.1016/0003-2697(72)90132-7

SLATER AFG, 1993, PHARMACOL THERAPEUT, V57, P203, DOI 10.1016/0163-7258(93)90056-J

Sotelo J, 2006, ANN INTERN MED, V144, P337, DOI 10.7326/0003-4819-144-5-200603070-00008

Soto C, 2018, NAT NEUROSCI, V21, P1332, DOI 10.1038/s41593-018-0235-9

Takacs-Vellai K, 2006, BIOESSAYS, V28, P1126, DOI 10.1002/bies.20489

Thome R, 2013, PLOS ONE, V8, DOI 10.1371/journal.pone.0065913

Wallace DJ, 2012, NAT REV RHEUMATOL, V8, P522, DOI 10.1038/nrrheum.2012.106

Wang QQ, 2015, TRANSL NEURODEGENER, V4, DOI 10.1186/s40035-015-0042-0

Weng MR, 2018, PARKINSONS DIS-US, V2018, DOI 10.1155/2018/9163040

Zhang JY, 2009, J ALZHEIMERS DIS, V16, P39, DOI 10.3233/JAD-2009-0908

Zhu HB, 2017, PLOS ONE, V12, DOI 10.1371/journal.pone.0183484

NR 67

TC 3

Z9 3

U1 5

U2 12

PU SPRINGER BASEL AG

PI BASEL

PA PICASSOPLATZ 4, BASEL, 4052, SWITZERLAND

SN 0925-4692

EI 1568-5608

J9 INFLAMMOPHARMACOLOGY

JI Inflammopharmacology

PD APR

PY 2023

VL 31

IS 2

BP 927

EP 941

DI 10.1007/s10787-023-01141-z

EA JAN 2023

PG 15

WC Immunology; Pharmacology & Pharmacy; Toxicology

WE Science Citation Index Expanded (SCI-EXPANDED)

SC Immunology; Pharmacology & Pharmacy; Toxicology

GA E3FU1

UT WOS:000921776700001

PM 36715843

DA 2024-08-12

ER

EF

FN Clarivate Analytics Web of Science

VR 1.0

PT J

AU Madeira, JM

Bajwa, E

Stuart, MJ

Hashioka, S

Klegeris, A

AF Madeira, Jocelyn M.

Bajwa, Ekta

Stuart, Maegan J.

Hashioka, Sadayuki

Klegeris, Andis

TI Gold drug auranofin could reduce neuroinflammation by inhibiting

microglia cytotoxic secretions and primed respiratory burst

SO JOURNAL OF NEUROIMMUNOLOGY

LA English

DT Article

DE Alzheimer's disease; Parkinson's disease; Neuroprotection;

Aurothiomalate; Aurothiosulfate; Respiratory burst priming

ID alzheimer's disease; PERITONEAL-MACROPHAGES; INTRACELLULAR CALCIUM;

RHEUMATOID-ARTHRITIS; CYTOKINE SECRETION; HUMAN NEUTROPHILS;

NERVOUS-SYSTEM; IN-VIVO; INFLAMMATION; ACTIVATION

AB Neuroinflammation contributes to the pathogenesis of neurological disorders. Anti-inflammatory treatments could potentially be used to slow down the progression of these diseases. We studied the anti-neuroinflammatory activity of gold compounds which have been used to treat rheumatoid arthritis. Non-toxic concentrations of auranofin (0.1-1 mu M) significantly reduced the cytotoxic secretions by primary human microglia and microglia-like THP-1 promonocytic cells. Auranofin inhibited primed NADPH-oxidase dependent respiratory burst and secretion of tumor necrosis factor (TNF)-alpha and nitric oxide by monocytic cells. It had a direct neuroprotective effect on SH-SY5Y neuronal cells. Auranofin could have a novel application in the treatment of neurodegenerative disease. (C) 2014 Elsevier B.V. All rights reserved.

C1 [Madeira, Jocelyn M.; Bajwa, Ekta; Stuart, Maegan J.; Klegeris, Andis] Univ British Columbia, Dept Biol, Kelowna, BC V1V 1V7, Canada.

[Hashioka, Sadayuki] Univ British Columbia, Dept Psychiat, Kinsmen Lab Neurol Res, Vancouver, BC V6T 1W5, Canada.

[Hashioka, Sadayuki] Shimane Univ, Fac Med, Dept Psychiat, Izumo, Shimane, Japan.

C3 University of British Columbia; University of British Columbia; Shimane

University

RP Klegeris, A (corresponding author), Univ British Columbia, Dept Biol, Okanagan Campus,3187 Univ Way, Kelowna, BC V1V 1V7, Canada.

EM andis.klegeris@ubc.ca

FU Grants-in-Aid for Scientific Research [24591721] Funding Source: KAKEN

CR ABRAMSON SL, 1990, J IMMUNOL, V144, P625

ANDERSON R, 1991, MOL PHARMACOL, V40, P427

Block ML, 2007, NAT REV NEUROSCI, V8, P57, DOI 10.1038/nrn2038

Brodie C, 1998, J NEUROIMMUNOL, V81, P20, DOI 10.1016/S0165-5728(97)00154-9

Brown GC, 2010, NITRIC OXIDE-BIOL CH, V23, P153, DOI 10.1016/j.niox.2010.06.001

BRUZE M, 1995, CONTACT DERMATITIS, V32, P5, DOI 10.1111/j.1600-0536.1995.tb00831.x

Cameron B, 2010, NEUROBIOL DIS, V37, P503, DOI 10.1016/j.nbd.2009.10.006

CHAMPION GD, 1990, BAILLIERE CLIN RHEUM, V4, P491, DOI 10.1016/S0950-3579(05)80005-6

Chomarat P, 1998, Int Rev Immunol, V17, P1, DOI 10.3109/08830189809084486

Cornelius C, 2013, ANTIOXID REDOX SIGN, V19, P836, DOI 10.1089/ars.2012.4981

Dajas F, 2003, NEUROTOX RES, V5, P425, DOI 10.1007/BF03033172

DECKER T, 1988, J IMMUNOL METHODS, V115, P61, DOI 10.1016/0022-1759(88)90310-9

DING AH, 1988, J IMMUNOL, V141, P2407

Ellingsen T, 2001, J RHEUMATOL, V28, P41

Frohman EM, 2006, NEW ENGL J MED, V354, P942, DOI 10.1056/NEJMra052130

Gao HM, 2012, TRENDS PHARMACOL SCI, V33, P295, DOI 10.1016/j.tips.2012.03.008

Gibbons HM, 2006, BRAIN RES, V1084, P1, DOI 10.1016/j.brainres.2006.02.032

Glennas A, 1997, BRIT J RHEUMATOL, V36, P870

Graham G G, 1994, Met Based Drugs, V1, P395

HANSEN MB, 1989, J IMMUNOL METHODS, V119, P203, DOI 10.1016/0022-1759(89)90397-9

Hashioka S, 2009, EXP NEUROL, V217, P177, DOI 10.1016/j.expneurol.2009.02.002

Heneka MT, 2010, J NEURAL TRANSM, V117, P919, DOI 10.1007/s00702-010-0438-z

Jeon KI, 2000, J IMMUNOL, V164, P5981, DOI 10.4049/jimmunol.164.11.5981

Kean WF, 1997, BRIT J RHEUMATOL, V36, P560

KEAN WF, 1990, BAILLIERE CLIN RHEUM, V4, P219, DOI 10.1016/S0950-3579(05)80019-6

Kim NH, 2007, IMMUNOLOGY, V122, P607, DOI 10.1111/j.1365-2567.2007.02679.x

Kim NH, 2010, J PHARMACOL SCI, V113, P246, DOI 10.1254/jphs.09330FP

Klegeris A, 2005, NEUROBIOL AGING, V26, P673, DOI 10.1016/j.neurobiolaging.2004.06.012

Klegeris A, 2003, BRIT J PHARMACOL, V139, P775, DOI 10.1038/sj.bjp.0705304

Klegeris A, 2007, CURR OPIN NEUROL, V20, P351, DOI 10.1097/WCO.0b013e3280adc943

Kortekaas R, 2005, ANN NEUROL, V57, P176, DOI 10.1002/ana.20369

Krause D, 2011, AM J PATHOL, V179, P1360, DOI 10.1016/j.ajpath.2011.05.048

Lee CW, 2013, FOOD CHEM TOXICOL, V60, P123, DOI 10.1016/j.fct.2013.07.029

Lee YM, 2010, ARCH PHARM RES, V33, P231, DOI 10.1007/s12272-010-0207-4

Li CY, 2011, CURR ALZHEIMER RES, V8, P67

Lu D, 2012, J CARDIOVASC PHARM, V59, P426, DOI 10.1097/FJC.0b013e318249509e

Lull ME, 2010, NEUROTHERAPEUTICS, V7, P354, DOI 10.1016/j.nurt.2010.05.014

Madeira JM, 2013, LIFE SCI, V92, P1072, DOI 10.1016/j.lfs.2013.04.005

Madeira J. M., 2012, Inflammopharmacology, V20, P297, DOI 10.1007/s10787-012-0149-1

Madeira Jocelyn., 2012, Microglia: Biology, Functions and Roles in Disease, P73

Makino J, 2012, FREE RADICAL RES, V46, P637, DOI 10.3109/10715762.2012.664841

MEANS AR, 1980, NATURE, V285, P73, DOI 10.1038/285073a0

MIRABELLI CK, 1985, CANCER RES, V45, P32

MOSMANN T, 1983, J IMMUNOL METHODS, V65, P55, DOI 10.1016/0022-1759(83)90303-4

Muranaka S, 2005, ANTIOXID REDOX SIGN, V7, P1367, DOI 10.1089/ars.2005.7.1367

Nakaya A, 2011, LEUKEMIA RES, V35, P243, DOI 10.1016/j.leukres.2010.05.011

Persidsky Y, 2006, J NEUROIMMUNE PHARM, V1, P223, DOI 10.1007/s11481-006-9025-3

Pocernich CB, 2012, BBA-MOL BASIS DIS, V1822, P625, DOI 10.1016/j.bbadis.2011.10.003

Polanski W, 2011, EXPERT REV NEUROTHER, V11, P845, DOI [10.1586/ERN.11.1, 10.1586/ern.11.1]

Ransohoff RM, 2012, J CLIN INVEST, V122, P1164, DOI 10.1172/JCI58644

Stern I, 2005, TOXICOL IN VITRO, V19, P365, DOI 10.1016/j.tiv.2004.11.001

VANDERVORT AL, 1992, J IMMUNOL, V149, P359

WALZ DT, 1983, AM J MED, V75, P90, DOI 10.1016/0002-9343(83)90481-3

WONG K, 1990, J BIOL CHEM, V265, P21454

Yamada R, 1999, EUR J PHARMACOL, V385, P71, DOI 10.1016/S0014-2999(99)00707-4

Yamamoto N, 2010, J NEUROSCI RES, V88, P1934, DOI 10.1002/jnr.22363

Yamashita M, 1997, EUR J PHARMACOL, V325, P221, DOI 10.1016/S0014-2999(97)00118-0

Zhang W, 2005, FASEB J, V19, P533, DOI 10.1096/fj.04-2751com

Zilka N, 2012, J NEUROINFLAMM, V9, DOI 10.1186/1742-2094-9-47

NR 59

TC 33

Z9 34

U1 0

U2 10

PU ELSEVIER

PI AMSTERDAM

PA RADARWEG 29, 1043 NX AMSTERDAM, NETHERLANDS

SN 0165-5728

EI 1872-8421

J9 J NEUROIMMUNOL

JI J. Neuroimmunol.

PD NOV 15

PY 2014

VL 276

IS 1-2

BP 71

EP 79

DI 10.1016/j.jneuroim.2014.08.615

PG 9

WC Immunology; Neurosciences

WE Science Citation Index Expanded (SCI-EXPANDED)

SC Immunology; Neurosciences & Neurology

GA AU3CI

UT WOS:000345490000009

PM 25175064

DA 2024-08-12

ER

PT J

AU Thomas, T

Timmer, M

Cesnulevicius, K

Hitti, E

Kotlyarov, A

Gaestel, M

AF Thomas, Tobias

Timmer, Marco

Cesnulevicius, Konstantin

Hitti, Edward

Kotlyarov, Alexey

Gaestel, Matthias

TI MAPKAP kinase 2-deficiency prevents neurons from cell death by reducing

neuroinflammation - relevance in a mouse model of Parkinson's disease

SO JOURNAL OF NEUROCHEMISTRY

LA English

DT Article

DE ablation; inflammation; neuroclegeneration; Parkinson's disease; protein

kinases

ID ACTIVATED PROTEIN-KINASE; NITRIC-OXIDE SYNTHASE; TUMOR-NECROSIS-FACTOR;

CEREBELLAR GRANULE NEURONS; DOPAMINERGIC-NEURONS; TNF-ALPHA; INDUCED

APOPTOSIS; SUBSTANTIA-NIGRA; BINDING PROTEIN; GENE-EXPRESSION

AB The inflammatory response in the brain is closely associated with the pathogenesis of degenerative neurological disorders. A role for the p38 stress-activated protein kinase/MAPK-activated protein kinase 2 (MK2) axis in inflammation and apoptosis is well documented. Here, we provide evidence that neurodegeneration can be prevented by eliminating MK2. In primary mesencephalic neuron-glia co-cultures dopaminergic neurons from MK2-deficient (MK2-/-) mice were significantly more resistant to lipopolysaccharide-induced neurotoxicity compared with cells from wild-type mice. This neuroprotection in MK2-deficient cultures was associated with a reduced inflammatory response, especially with reduced production of the inflammatory mediators tumor necrosis factor alpha, keratino-cyte-derived chemokine, interleukin-6, and nitric oxide (NO). Interestingly, in primary neuron-enriched cell cultures p38 MAPK, but not MK2, also participates in NO-mediated neuronal cell death. In the MPTP mouse model for Parkinson's disease, MK2-deficient mice show a reduced neuroinflammation and less degeneration of dopaminergic neurons in the substantia nigra after MPTP lesion compared with wild-type mice. In conclusion, our results reveal that MK2 does not directly participate in neuronal cell death, but indirectly contributes to neurodegeneration by the production of neurotoxic substances, such as NO or tumor necrosis factor alpha, from activated glia cells.

C1 [Thomas, Tobias; Hitti, Edward; Kotlyarov, Alexey; Gaestel, Matthias] Hannover Med Sch, Inst Biochem, D-30625 Hannover, Germany.

[Timmer, Marco; Cesnulevicius, Konstantin] Hannover Med Sch, Dept Neuroanat, D-3000 Hannover, Germany.

C3 Hannover Medical School; Hannover Medical School

RP Gaestel, M (corresponding author), Hannover Med Sch, Inst Biochem, Carl Neuberg Str 1, D-30625 Hannover, Germany.

EM gaestel.matthias@mh-hannover.de

RI Gaestel, Matthias/A-6560-2013

OI Gaestel, Matthias/0000-0002-4944-4652; Hitti, Edward/0000-0003-1587-2824

CR Barone FC, 2001, MED RES REV, V21, P129, DOI 10.1002/1098-1128(200103)21:2<129::AID-MED1003>3.0.CO;2-H

Bhat NR, 2002, J BIOL CHEM, V277, P29584, DOI 10.1074/jbc.M204994200

CHAO CC, 1993, J IMMUNOL, V151, P1473

Cheng AW, 2001, J BIOL CHEM, V276, P43320, DOI 10.1074/jbc.M107698200

Choi WS, 2004, J BIOL CHEM, V279, P20451, DOI 10.1074/jbc.M311164200

Clark AR, 2003, FEBS LETT, V546, P37, DOI 10.1016/S0014-5793(03)00439-3

Culbert AA, 2006, J BIOL CHEM, V281, P23658, DOI 10.1074/jbc.M513646200

DaSilva J, 1997, J BIOL CHEM, V272, P28373, DOI 10.1074/jbc.272.45.28373

Dean JLE, 2004, CELL SIGNAL, V16, P1113, DOI 10.1016/j.cellsig.2004.04.006

Du YS, 2001, P NATL ACAD SCI USA, V98, P14669, DOI 10.1073/pnas.251341998

Fechir M, 2005, MOL PHARMACOL, V67, P2148, DOI 10.1124/mol.104.008763

Ferger B, 2004, J NEUROCHEM, V89, P822, DOI 10.1111/j.1471-4159.2004.02399.x

Galea E, 1996, J NEUROIMMUNOL, V64, P19, DOI 10.1016/0165-5728(95)00143-3

Gao HM, 2003, J NEUROSCI, V23, P6181

Ghatan S, 2000, J CELL BIOL, V150, P335, DOI 10.1083/jcb.150.2.335

Harada J, 1999, JPN J PHARMACOL, V79, P369, DOI 10.1254/jjp.79.369

Harding TC, 2001, J BIOL CHEM, V276, P4531, DOI 10.1074/jbc.C000815200

Hitti E, 2006, MOL CELL BIOL, V26, P2399, DOI 10.1128/MCB.26.6.2399-2407.2006

Jeohn GH, 2002, ANN NY ACAD SCI, V962, P332, DOI 10.1111/j.1749-6632.2002.tb04078.x

Kanda H, 2004, J BIOCHEM, V136, P1, DOI 10.1093/jb/mvh098

Kontoyiannis D, 2002, J EXP MED, V196, P1563, DOI 10.1084/jem.20020281

Kotlyarov A, 1999, NAT CELL BIOL, V1, P94, DOI 10.1038/10061

Kotlyarov A, 2002, MOL CELL BIOL, V22, P4827, DOI 10.1128/MCB.22.13.4827-4835.2002

LEE SC, 1993, J IMMUNOL, V150, P2659

Lee SM, 2004, J NEUROCHEM, V91, P568, DOI 10.1111/j.1471-4159.2004.02780.x

Lehner MD, 2002, J IMMUNOL, V168, P4667, DOI 10.4049/jimmunol.168.9.4667

Liberatore GT, 1999, NAT MED, V5, P1403

Liu B, 2000, J PHARMACOL EXP THER, V293, P607

Matyszak MK, 1998, PROG NEUROBIOL, V56, P19, DOI 10.1016/S0301-0082(98)00014-8

Namgung U, 2000, J NEUROSCI, V20, P6442, DOI 10.1523/JNEUROSCI.20-17-06442.2000

Neininger A, 2002, J BIOL CHEM, V277, P3065, DOI 10.1074/jbc.C100685200

Olanow CW, 1999, ANNU REV NEUROSCI, V22, P123, DOI 10.1002/mds.23732

Onyango IG, 2005, MOL CELL NEUROSCI, V28, P452, DOI 10.1016/j.mcn.2004.10.006

Oorschot DE, 1996, J COMP NEUROL, V366, P580, DOI 10.1002/(SICI)1096-9861(19960318)366:4<580::AID-CNE3>3.0.CO;2-0

Ouyang MX, 2006, J NEUROCHEM, V97, P234, DOI 10.1111/j.1471-4159.2006.03730.x

PAKKENBERG B, 1991, J NEUROL NEUROSUR PS, V54, P30, DOI 10.1136/jnnp.54.1.30

Possel H, 2000, GLIA, V32, P51, DOI 10.1002/1098-1136(200010)32:1<51::AID-GLIA50>3.0.CO;2-4

Przedborski S, 1996, P NATL ACAD SCI USA, V93, P4565, DOI 10.1073/pnas.93.10.4565

Qin LY, 2004, J BIOL CHEM, V279, P1415, DOI 10.1074/jbc.M307657200

Rodriguez-Pascual F, 2000, J BIOL CHEM, V275, P26040, DOI 10.1074/jbc.M910460199

Rostworowski M, 1997, J NEUROSCI, V17, P3664

ROUSE J, 1994, CELL, V78, P1027, DOI 10.1016/0092-8674(94)90277-1

Ruano D, 2006, NEUROSCIENCE, V140, P1157, DOI 10.1016/j.neuroscience.2006.02.073

SHOHAMI E, 1994, J CEREBR BLOOD F MET, V14, P615, DOI 10.1038/jcbfm.1994.76

Sun AY, 2003, EXP NEUROL, V183, P394, DOI 10.1016/S0014-4886(03)00180-8

Teismann P, 2003, P NATL ACAD SCI USA, V100, P5473, DOI 10.1073/pnas.0837397100

Timmer M, 2007, J NEUROSCI, V27, P459, DOI 10.1523/JNEUROSCI.4493-06.2007

TIPTON KF, 1993, J NEUROCHEM, V61, P1191, DOI 10.1111/j.1471-4159.1993.tb13610.x

Vertii A, 2006, J BIOL CHEM, V281, P26966, DOI 10.1074/jbc.M602134200

Vezzani A, 2005, EPILEPSIA, V46, P1724, DOI 10.1111/j.1528-1167.2005.00298.x

Vila M, 2001, P NATL ACAD SCI USA, V98, P2837, DOI 10.1073/pnas.051633998

Volk H, 2005, J HISTOCHEM CYTOCHEM, V53, P517, DOI 10.1369/jhc.4A6451.2005

Wang XK, 2002, J BIOL CHEM, V277, P43968, DOI 10.1074/jbc.M206837200

WEST MJ, 1990, J COMP NEUROL, V296, P1, DOI 10.1002/cne.902960102

Wilms H, 2003, FASEB J, V17, P500, DOI 10.1096/fj.02-0314fje

Wu DC, 2002, J NEUROSCI, V22, P1763, DOI 10.1523/JNEUROSCI.22-05-01763.2002

Yamagishi S, 2001, J BIOL CHEM, V276, P5129, DOI 10.1074/jbc.M007258200

NR 57

TC 64

Z9 76

U1 0

U2 1

PU WILEY-BLACKWELL

PI MALDEN

PA COMMERCE PLACE, 350 MAIN ST, MALDEN 02148, MA USA

SN 0022-3042

J9 J NEUROCHEM

JI J. Neurochem.

PD JUN

PY 2008

VL 105

IS 5

BP 2039

EP 2052

DI 10.1111/j.1471-4159.2008.05310.x

PG 14

WC Biochemistry & Molecular Biology; Neurosciences

WE Science Citation Index Expanded (SCI-EXPANDED)

SC Biochemistry & Molecular Biology; Neurosciences & Neurology

GA 300OQ

UT WOS:000255835000039

PM 18298661

OA Bronze

DA 2024-08-12

ER

PT J

AU Mancini, M

Natoli, S

Gardoni, F

Di Luca, M

Pisani, A

AF Mancini, Maria

Natoli, Silvia

Gardoni, Fabrizio

Di Luca, Monica

Pisani, Antonio

TI Dopamine Transmission Imbalance in Neuroinflammation: Perspectives on

Long-Term COVID-19

SO INTERNATIONAL JOURNAL OF MOLECULAR SCIENCES

LA English

DT Review

DE dopamine; SARS-CoV-2; neuroinflammation; cytokines; Parkinson's disease;

dopamine release; interleukins; glia; alpha-synuclein; long-COVID;

post-acute sequelae

ID VESICULAR MONOAMINE TRANSPORTER-2; GLUTAMATE-DEPENDENT INHIBITION;

ALPHA-SYNUCLEIN EXPRESSION; INTERFERON-ALPHA; SUBSTANTIA-NIGRA;

TYROSINE-HYDROXYLASE; parkinson's disease; NITRIC-OXIDE; PRESYNAPTIC

REGULATION; BASAL GANGLIA

AB Dopamine (DA) is a key neurotransmitter in the basal ganglia, implicated in the control of movement and motivation. Alteration of DA levels is central in Parkinson's disease (PD), a common neurodegenerative disorder characterized by motor and non-motor manifestations and deposition of alpha-synuclein (alpha-syn) aggregates. Previous studies have hypothesized a link between PD and viral infections. Indeed, different cases of parkinsonism have been reported following COVID-19. However, whether SARS-CoV-2 may trigger a neurodegenerative process is still a matter of debate. Interestingly, evidence of brain inflammation has been described in postmortem samples of patients infected by SARS-CoV-2, which suggests immune-mediated mechanisms triggering the neurological sequelae. In this review, we discuss the role of proinflammatory molecules such as cytokines, chemokines, and oxygen reactive species in modulating DA homeostasis. Moreover, we review the existing literature on the possible mechanistic interplay between SARS-CoV-2-mediated neuroinflammation and nigrostriatal DAergic impairment, and the cross-talk with aberrant alpha-syn metabolism.

C1 [Mancini, Maria; Pisani, Antonio] Univ Pavia, Dept Brain & Behav Sci, I-27100 Pavia, Italy.

[Mancini, Maria; Pisani, Antonio] IRCCS Mondino Fdn, I-27100 Pavia, Italy.

[Natoli, Silvia] Univ Roma Tor Vergata, Dept Clin Sci & Translat Med, I-00133 Rome, Italy.

[Natoli, Silvia] IRCCS Maugeri Pavia, I-27100 Pavia, Italy.

[Gardoni, Fabrizio; Di Luca, Monica] Univ Milan, Dept Pharmacol & Biomol Sci Rodolfo Paoletti, I-20133 Milan, Italy.

C3 University of Pavia; IRCCS Fondazione Casimiro Mondino; University of

Rome Tor Vergata; University of Milan

RP Pisani, A (corresponding author), Univ Pavia, Dept Brain & Behav Sci, I-27100 Pavia, Italy.; Pisani, A (corresponding author), IRCCS Mondino Fdn, I-27100 Pavia, Italy.

EM maria.mancini@unipv.it; silvia.natoli@uniroma2.it;

fabrizio.gardoni@unimi.it; monica.diluca@unimi.it;

antonio.pisani@unipv.it

RI DiLuca, Monica mg/A-7632-2012; Gardoni, Fabrizio/A-3135-2009

OI Mancini, Maria/0000-0003-2181-7485; Natoli, Silvia/0000-0003-3758-5375;

Gardoni, Fabrizio/0000-0003-4598-5563

FU Fondazione Regionale per la Ricerca Biomedica (FRRB, Regione Lombardia),

Linking Park [3433068]

FX This work has been supported by Fondazione Regionale per la Ricerca

Biomedica (FRRB, Regione Lombardia), Linking Park, ID 3433068.

CR Adermark L, 2022, NEUROPSYCHOPHARMACOL, V47, P1493, DOI 10.1038/s41386-021-01232-x

Alam MM, 2022, CELL REP, V38, DOI 10.1016/j.celrep.2021.110090

Albensi BC, 2000, SYNAPSE, V35, P151

Albornoz EA, 2023, MOL PSYCHIATR, V28, P2878, DOI 10.1038/s41380-022-01831-0

ALONSO R, 1993, J NEUROCHEM, V61, P1284, DOI 10.1111/j.1471-4159.1993.tb13620.x

Amori L, 2009, NEUROSCIENCE, V159, P196, DOI 10.1016/j.neuroscience.2008.11.055

Anisman H, 1996, BRAIN RES, V731, P1

Ara J, 1998, P NATL ACAD SCI USA, V95, P7659, DOI 10.1073/pnas.95.13.7659

Asanuma M, 2014, PLOS ONE, V9, DOI 10.1371/journal.pone.0106362

Aviner R, 2020, CSH PERSPECT BIOL, V12, DOI 10.1101/cshperspect.a034090

Avital A, 2003, HIPPOCAMPUS, V13, P826, DOI 10.1002/hipo.10135

Avshalumov MV, 2008, J NEUROPHYSIOL, V100, P1590, DOI 10.1152/jn.90548.2008

Avshalumov MV, 2003, P NATL ACAD SCI USA, V100, P11729, DOI 10.1073/pnas.1834314100

Avshalumov MV, 2003, J NEUROSCI, V23, P2744

Bae EJ, 2022, EXP MOL MED, V54, P788, DOI 10.1038/s12276-022-00789-x

Balchin D, 2016, SCIENCE, V353, DOI 10.1126/science.aac4354

BAN E, 1991, NEUROSCIENCE, V43, P21, DOI 10.1016/0306-4522(91)90412-H

Barbut D, 2019, J PARKINSON DIS, V9, pS313, DOI 10.3233/JPD-191702

BARTFAI T, 1993, NEUROCHEM INT, V22, P435, DOI 10.1016/0197-0186(93)90038-7

Beatman EL, 2016, J VIROL, V90, P2767, DOI 10.1128/JVI.02949-15

Beckstead MJ, 2004, NEURON, V42, P939, DOI 10.1016/j.neuron.2004.05.019

Belarbi K, 2012, J NEUROINFLAMM, V9, DOI 10.1186/1742-2094-9-23

Bergquist F, 2002, BRAIN RES, V950, P245, DOI 10.1016/S0006-8993(02)03047-0

Bernard V, 1998, EUR J NEUROSCI, V10, P3721, DOI 10.1046/j.1460-9568.1998.00380.x

Bersano A, 2008, EUR J INTERN MED, V19, P370, DOI 10.1016/j.ejim.2007.10.003

Blauwendraat C, 2020, LANCET NEUROL, V19, P170, DOI 10.1016/S1474-4422(19)30287-X

Bojkova D, 2020, NATURE, V583, P469, DOI 10.1038/s41586-020-2332-7

Bopeththa B V K M, 2017, BMC Res Notes, V10, P655, DOI 10.1186/s13104-017-2954-5

Boura I, 2022, MOV DISORD CLIN PRAC, V9, P584, DOI 10.1002/mdc3.13461

Braida D, 2004, BEHAV BRAIN RES, V153, P423, DOI 10.1016/j.bbr.2003.12.018

Brimblecombe KR, 2015, J PHYSIOL-LONDON, V593, P929, DOI 10.1113/jphysiol.2014.285890

Brown JM, 2001, J PHARMACOL EXP THER, V296, P762

Bryclon L, 2008, BIOL PSYCHIAT, V63, P1022, DOI 10.1016/j.biopsych.2007.12.007

BULL DR, 1991, N-S ARCH PHARMACOL, V343, P260

Burre J, 2010, SCIENCE, V329, P1663, DOI 10.1126/science.1195227

Cadar D, 2021, MICROORGANISMS, V9, DOI 10.3390/microorganisms9081716

Calculli A, 2023, EUR J NEUROL, V30, P1272, DOI 10.1111/ene.15732

Cantuti-Castelvetri L, 2020, SCIENCE, V370, P856, DOI 10.1126/science.abd2985

Capuron L, 2003, BRAIN BEHAV IMMUN, V17, pS119

Capuron L, 2007, NEUROPSYCHOPHARMACOL, V32, P2384, DOI 10.1038/sj.npp.1301362

Capuron L, 2012, ARCH GEN PSYCHIAT, V69, P1044, DOI 10.1001/archgenpsychiatry.2011.2094

Carpenedo R, 2001, EUR J NEUROSCI, V13, P2141, DOI 10.1046/j.0953-816x.2001.01592.x

Cartier EA, 2010, J BIOL CHEM, V285, P1957, DOI 10.1074/jbc.M109.054510

Casals J, 1998, J NEURAL TRANSM, V105, P645, DOI 10.1007/s007020050086

Centonze D, 2009, J NEUROSCI, V29, P3442, DOI 10.1523/JNEUROSCI.5804-08.2009

Chen BT, 2006, J NEUROCHEM, V96, P645, DOI 10.1111/j.1471-4159.2005.03519.x

Chen BT, 2001, J NEUROSCI, V21, P7841, DOI 10.1523/JNEUROSCI.21-19-07841.2001

Chen CC, 2017, NATURE, V542, P43, DOI 10.1038/nature20818

Chen RR, 2021, FRONT NEUROL, V11, DOI 10.3389/fneur.2020.573095

Cohen ME, 2020, LANCET NEUROL, V19, P804, DOI 10.1016/S1474-4422(20)30305-7

Coutard B, 2020, ANTIVIR RES, V176, DOI 10.1016/j.antiviral.2020.104742

Cragg SJ, 2003, J NEUROSCI, V23, P4378

Crunfli F, 2022, P NATL ACAD SCI USA, V119, DOI 10.1073/pnas.2200960119

Cumiskey D, 2007, NEUROSCI LETT, V412, P206, DOI 10.1016/j.neulet.2006.11.007

Curran B, 2001, NEUROSCIENCE, V108, P83, DOI 10.1016/S0306-4522(01)00405-5

Di Filippo M, 2021, CELL REP, V37, DOI 10.1016/j.celrep.2021.110094

Di Filippo M, 2016, NEUROPHARMACOLOGY, V100, P98, DOI 10.1016/j.neuropharm.2015.06.009

Dickson DW, 2009, PARKINSONISM RELAT D, V15, pS1, DOI 10.1016/S1353-8020(09)70769-2

Dunn AJ, 2006, CLIN NEUROSCI RES, V6, P52, DOI 10.1016/j.cnr.2006.04.002

EIDELBERG D, 1994, J CEREBR BLOOD F MET, V14, P783, DOI 10.1038/jcbfm.1994.99

Ellul MA, 2020, LANCET NEUROL, V19, P767, DOI 10.1016/S1474-4422(20)30221-0

Emmi A, 2023, NPJ PARKINSONS DIS, V9, DOI 10.1038/s41531-023-00467-3

Fajgenbaum DC, 2020, NEW ENGL J MED, V383, P2255, DOI 10.1056/NEJMra2026131

Felger JC, 2016, MOL PSYCHIATR, V21, P1358, DOI 10.1038/mp.2015.168

Felger JC, 2007, BIOL PSYCHIAT, V62, P1324, DOI 10.1016/j.biopsych.2007.05.026

Felger JC, 2017, NEUROPSYCHOPHARMACOL, V42, P216, DOI 10.1038/npp.2016.143

Felger JC, 2015, INT J NEUROPSYCHOPH, V18, DOI 10.1093/ijnp/pyu084

Felger JC, 2013, NEUROPSYCHOPHARMACOL, V38, P2179, DOI 10.1038/npp.2013.115

Felger JC, 2013, BRAIN BEHAV IMMUN, V31, P153, DOI 10.1016/j.bbi.2012.10.010

Felger JC, 2012, FRONT NEUROENDOCRIN, V33, P315, DOI 10.1016/j.yfrne.2012.09.003

Fellner L, 2013, GLIA, V61, P349, DOI 10.1002/glia.22437

Filiano AJ, 2016, NATURE, V535, P425, DOI 10.1038/nature18626

Fountaine TM, 2007, J NEUROSCI RES, V85, P351, DOI 10.1002/jnr.21125

FREIDIN M, 1992, P NATL ACAD SCI USA, V89, P10440, DOI 10.1073/pnas.89.21.10440

Gao Nan, 2007, Neurosci Bull, V23, P53, DOI 10.1007/s12264-007-0008-z

Garay Paula A, 2010, Front Synaptic Neurosci, V2, P136, DOI 10.3389/fnsyn.2010.00136

Gentile A, 2015, NEUROBIOL DIS, V74, P347, DOI 10.1016/j.nbd.2014.11.022

George JM, 2002, GENOME BIOL, V3

German CL, 2015, PHARMACOL REV, V67, P1005, DOI 10.1124/pr.114.010397

Gitler AD, 2008, P NATL ACAD SCI USA, V105, P145, DOI 10.1073/pnas.0710685105

Goldsmith DR, 2016, BRAIN BEHAV IMMUN, V56, P281, DOI 10.1016/j.bbi.2016.03.025

Greenhalgh AD, 2020, NAT REV NEUROSCI, V21, P139, DOI 10.1038/s41583-020-0263-9

Greten-Harrison B, 2010, P NATL ACAD SCI USA, V107, P19573, DOI 10.1073/pnas.1005005107

Guan JX, 2012, MOVEMENT DISORD, V27, P156, DOI 10.1002/mds.23935

Guasp M, 2022, FRONT IMMUNOL, V13, DOI 10.3389/fimmu.2022.866153

Gulley JM, 2003, EUR J PHARMACOL, V479, P139, DOI 10.1016/j.ejphar.2003.08.064

Guyon A, 2008, EUR J NEUROSCI, V28, P862, DOI 10.1111/j.1460-9568.2008.06367.x

Guyon A, 2006, J NEUROCHEM, V96, P1540, DOI 10.1111/j.1471-4159.2006.03659.x

Habbas S, 2015, CELL, V163, P1730, DOI 10.1016/j.cell.2015.11.023

Haji N, 2012, EXP NEUROL, V237, P296, DOI 10.1016/j.expneurol.2012.07.010

Harms AS, 2021, ACTA NEUROPATHOL, V141, P527, DOI 10.1007/s00401-021-02268-5

Harschnitz O, 2021, NAT REV IMMUNOL, V21, P441, DOI 10.1038/s41577-020-00474-y

Hartung H, 2011, NEUROPSYCHOPHARMACOL, V36, P1811, DOI 10.1038/npp.2011.62

Hirsch EC, 2021, MOVEMENT DISORD, V36, P16, DOI 10.1002/mds.28075

Hoffman LA, 2017, BRAIN, V140, P2246, DOI 10.1093/brain/awx177

Hoffmann M, 2020, CELL, V181, P271, DOI 10.1016/j.cell.2020.02.052

Imbriani P, 2022, NEUROBIOL DIS, V173, DOI 10.1016/j.nbd.2022.105851

Iroegbu JD, 2020, NEUROL SCI, V41, P1329, DOI 10.1007/s10072-020-04469-4

Jacob F, 2020, CELL STEM CELL, V27, P937, DOI 10.1016/j.stem.2020.09.016

Jang H, 2009, BBA-MOL BASIS DIS, V1792, P714, DOI 10.1016/j.bbadis.2008.08.001

Jellinger KA, 2009, ACTA NEUROPATHOL, V118, P371, DOI 10.1007/s00401-009-0537-9

Juengling FD, 2000, PSYCHOPHARMACOLOGY, V152, P383, DOI 10.1007/s002130000549

Kaeser PS, 2014, ANNU REV PHYSIOL, V76, P333, DOI 10.1146/annurev-physiol-021113-170338

Kasen A, 2022, NEUROBIOL DIS, V166, DOI 10.1016/j.nbd.2022.105654

Kazumori H, 2004, AM J PHYSIOL-GASTR L, V286, pG508, DOI 10.1152/ajpgi.00269.2003

Khan S, 2021, ELIFE, V10, DOI 10.7554/eLife.68563

KINOUCHI K, 1991, BIOCHEM BIOPH RES CO, V181, P1532, DOI 10.1016/0006-291X(91)92113-X

Kirik D, 2002, J NEUROSCI, V22, P2780, DOI 10.1523/JNEUROSCI.22-07-02780.2002

Kiss JP, 1999, NEUROCHEM INT, V34, P345, DOI 10.1016/S0197-0186(99)00019-4

Kitagami T, 2003, BRAIN RES, V978, P104, DOI 10.1016/S0006-8993(03)02776-8

Kline EM, 2021, MOVEMENT DISORD, V36, P25, DOI 10.1002/mds.28411

KOSTIC VS, 1989, J NEUROL, V236, P421, DOI 10.1007/BF00314903

Krusz J C, 1987, Mov Disord, V2, P137, DOI 10.1002/mds.870020301

Kumai T, 2000, LIFE SCI, V67, P663, DOI 10.1016/S0024-3205(00)00660-3

Kumakura Y, 2006, J CEREBR BLOOD F MET, V26, P358, DOI 10.1038/sj.jcbfm.9600202

Kumakura Y, 2009, NEUROSCIENTIST, V15, P635, DOI 10.1177/1073858409338217

Labrie V, 2017, J INNATE IMMUN, V9, P437, DOI 10.1159/000479653

LAPCHAK PA, 1992, NEUROREPORT, V3, P165, DOI 10.1097/00001756-199202000-00011

Larsen KE, 2006, J NEUROSCI, V26, P11915, DOI 10.1523/JNEUROSCI.3821-06.2006

Lee HJ, 2008, BIOCHEM BIOPH RES CO, V372, P423, DOI 10.1016/j.bbrc.2008.05.045

Leta V, 2022, INT REV NEUROBIOL, P63, DOI 10.1016/bs.irn.2022.04.004

Leta V, 2022, J NEURAL TRANSM, V129, P1119, DOI 10.1007/s00702-022-02536-y

Li W, 2003, J NEUROCHEM, V86, P774, DOI 10.1046/j.1471-4159.2003.01890.x

Lin YF, 2004, P NATL ACAD SCI USA, V101, P7799, DOI 10.1073/pnas.0402496101

Liu CL, 2021, NAT REV NEUROSCI, V22, P345, DOI 10.1038/s41583-021-00455-7

Liu CL, 2019, CURR OPIN NEUROBIOL, V57, P46, DOI 10.1016/j.conb.2019.01.001

Liu DM, 2008, NEUROCHEM RES, V33, P1401, DOI 10.1007/s11064-008-9599-7

Majer M, 2008, BRAIN BEHAV IMMUN, V22, P870, DOI 10.1016/j.bbi.2007.12.009

Maleki AF, 2019, FRONT CELL NEUROSCI, V13, DOI 10.3389/fncel.2019.00355

Mander PK, 2006, J IMMUNOL, V176, P1046, DOI 10.4049/jimmunol.176.2.1046

Mandolesi G, 2013, J NEUROSCI, V33, P12105, DOI 10.1523/JNEUROSCI.5369-12.2013

Mandolesi G, 2012, NEUROBIOL DIS, V46, P414, DOI 10.1016/j.nbd.2012.02.005

MAROTEAUX L, 1988, J NEUROSCI, V8, P2804

Masliah E, 2000, SCIENCE, V287, P1265, DOI 10.1126/science.287.5456.1265

Matsuda W, 2009, J NEUROSCI, V29, P444, DOI 10.1523/JNEUROSCI.4029-08.2009

Mendez JA, 2011, J BIOL CHEM, V286, P23928, DOI 10.1074/jbc.M111.218032

Méndez-Guerrero A, 2020, NEUROLOGY, V95, pE2109, DOI 10.1212/WNL.0000000000010282

Mentis MJ, 2002, AM J PSYCHIAT, V159, P746, DOI 10.1176/appi.ajp.159.5.746

Merali Z, 1997, BRAIN RES, V761, P225, DOI 10.1016/S0006-8993(97)00312-0

Merello M, 2021, LANCET NEUROL, V20, P94, DOI 10.1016/S1474-4422(20)30442-7

MOHANKUMAR PS, 1991, LIFE SCI, V48, P925, DOI 10.1016/0024-3205(91)90040-I

Morassi M, 2021, J NEUROL, V268, P3980, DOI 10.1007/s00415-021-10560-3

Morón JA, 2003, J NEUROSCI, V23, P8480

Nemani VM, 2010, NEURON, V65, P66, DOI 10.1016/j.neuron.2009.12.023

Nirenberg MJ, 1996, J NEUROSCI, V16, P436

Nirenberg MJ, 1996, J NEUROSCI, V16, P4135

Normandin E, 2021, J NEUROL SCI, V430, DOI 10.1016/j.jns.2021.120023

Omiatek DM, 2013, SCI REP-UK, V3, DOI 10.1038/srep01447

PALIJ P, 1990, BRAIN RES, V509, P172, DOI 10.1016/0006-8993(90)90329-A

Pang ZPP, 2010, CURR OPIN CELL BIOL, V22, P496, DOI 10.1016/j.ceb.2010.05.001

Pasha Shaik Afsar, 2018, J Assoc Physicians India, V66, P92

Pellegrini L, 2020, CELL STEM CELL, V27, P951, DOI 10.1016/j.stem.2020.10.001

Peng XMM, 2005, J CELL SCI, V118, P3523, DOI 10.1242/jcs.02481

Pereira DB, 2012, FRONT BIOSCI-LANDMRK, V17, P2740, DOI 10.2741/4083

Perez RG, 2002, J NEUROSCI, V22, P3090, DOI 10.1523/JNEUROSCI.22-08-03090.2002

Petitto JM, 1997, J NEUROIMMUNOL, V73, P183, DOI 10.1016/S0165-5728(96)00196-8

Picconi B, 2005, NEUROTOXICOLOGY, V26, P779, DOI 10.1016/j.neuro.2005.02.002

Pilotto A, 2021, CLIN INFECT DIS, V73, pE3019, DOI 10.1093/cid/ciaa1933

Pothos EN, 2000, J NEUROSCI, V20, P7297

Pradhan S, 1999, NEUROLOGY, V53, P1781, DOI 10.1212/WNL.53.8.1781

Raison CL, 2009, BIOL PSYCHIAT, V65, P296, DOI 10.1016/j.biopsych.2008.08.010

Rassoulpour A, 2005, J NEUROCHEM, V93, P762, DOI 10.1111/j.1471-4159.2005.03134.x

REITE M, 1987, Biological Psychiatry, V22, P104, DOI 10.1016/0006-3223(87)90136-3

Rice ME, 2011, NEUROSCIENCE, V198, P112, DOI 10.1016/j.neuroscience.2011.08.066

Rice ME, 1997, J NEUROPHYSIOL, V77, P853, DOI 10.1152/jn.1997.77.2.853

Rossi S, 2012, MULT SCLER J, V18, P1633, DOI 10.1177/1352458512440207

Rossi S, 2012, ANN NEUROL, V71, P76, DOI 10.1002/ana.22512

Saha RN, 2006, ANTIOXID REDOX SIGN, V8, P929, DOI 10.1089/ars.2006.8.929

Sandoval V, 2003, J PHARMACOL EXP THER, V304, P1181, DOI 10.1124/jpet.102.045005

Sandoval V, 2002, J NEUROSCI, V22, P8705

Sarasombath Pichaya, 2002, Hawaii Med J, V61, P57

Sarkar S, 2020, FRONT IMMUNOL, V11, DOI 10.3389/fimmu.2020.00033

Scheiblich H, 2021, J IMMUNOL, V207, P2143, DOI 10.4049/jimmunol.2100035

Schmid AW, 2009, HIPPOCAMPUS, V19, P670, DOI 10.1002/hipo.20542

Schmitt KC, 2010, ANN NY ACAD SCI, V1187, P316, DOI 10.1111/j.1749-6632.2009.05148.x

Schmitz Y, 2003, J NEUROCHEM, V87, P273, DOI 10.1046/j.1471-4159.2003.02050.x

Schneider H, 1998, P NATL ACAD SCI USA, V95, P7778, DOI 10.1073/pnas.95.13.7778

Schwarcz R, 2017, NEUROPHARMACOLOGY, V112, P237, DOI 10.1016/j.neuropharm.2016.08.003

Semerdzhiev SA, 2022, ACS CHEM NEUROSCI, V13, P143, DOI 10.1021/acschemneuro.1c00666

Shameli A, 2016, IMMUNOBIOLOGY, V221, P333, DOI 10.1016/j.imbio.2015.10.002

Sheridan GK, 2013, OPEN BIOL, V3, DOI 10.1098/rsob.130181

SHINTANI F, 1993, J NEUROSCI, V13, P3574

Shuto H, 1997, BRAIN RES, V747, P348, DOI 10.1016/S0006-8993(96)01371-6

Simmons G, 2005, P NATL ACAD SCI USA, V102, P11876, DOI 10.1073/pnas.0505577102

Skrzydelski D, 2007, J NEUROCHEM, V102, P1175, DOI 10.1111/j.1471-4159.2007.04639.x

Smeyne RJ, 2021, J PARKINSON DIS, V11, P31, DOI 10.3233/JPD-202279

Somayaji M, 2020, P NATL ACAD SCI USA, V117, P32701, DOI 10.1073/pnas.2013652117

Song ER, 2021, J EXP MED, V218, DOI 10.1084/jem.20202135

SPETSIERIS PG, 1995, COMPUT MED IMAG GRAP, V19, P295, DOI 10.1016/0895-6111(95)00011-E

Spillantini MG, 1997, NATURE, V388, P839, DOI 10.1038/42166

Stein SR, 2022, NATURE, V612, P758, DOI 10.1038/s41586-022-05542-y

Steinman L, 2013, SCI SIGNAL, V6, DOI 10.1126/scisignal.2003898

Stolzenberg E, 2017, J INNATE IMMUN, V9, P456, DOI 10.1159/000477990

Sulzer D, 2007, TRENDS NEUROSCI, V30, P244, DOI 10.1016/j.tins.2007.03.009

Sulzer D, 2016, BASAL GANGLIA, V6, P123, DOI 10.1016/j.baga.2016.02.001

Szelényi J, 2001, BRAIN RES BULL, V54, P329, DOI 10.1016/S0361-9230(01)00428-2

TAKAO T, 1990, ENDOCRINOLOGY, V127, P3070, DOI 10.1210/endo-127-6-3070

Tanji K, 2002, PATHOL INT, V52, P572, DOI 10.1046/j.1440-1827.2002.01385.x

Taylor MP, 2015, TRENDS MICROBIOL, V23, P283, DOI 10.1016/j.tim.2015.01.002

Tehranian R, 2006, J NEUROCHEM, V99, P1188, DOI 10.1111/j.1471-4159.2006.04146.x

Tomlinson JJ, 2017, J NEURAL TRANSM, V124, P721, DOI 10.1007/s00702-017-1726-7

Tritsch NX, 2012, NEURON, V76, P33, DOI 10.1016/j.neuron.2012.09.023

Trudler D, 2021, P NATL ACAD SCI USA, V118, DOI 10.1073/pnas.2025847118

Vainchtein ID, 2020, TRENDS NEUROSCI, V43, P144, DOI 10.1016/j.tins.2020.01.003

Venda LL, 2010, TRENDS NEUROSCI, V33, P559, DOI 10.1016/j.tins.2010.09.004

Vumma R, 2017, NEUROPSYCHOBIOLOGY, V75, P178, DOI 10.1159/000485130

Wang K, 2020, SIGNAL TRANSDUCT TAR, V5, DOI 10.1038/s41392-020-00426-x

Wersinger C, 2003, NEUROSCI LETT, V340, P189, DOI 10.1016/S0304-3940(03)00097-1

Wichmann T, 1999, NATURE, V400, P621, DOI 10.1038/23148

Witkovsky P, 2009, NEUROSCIENCE, V164, P488, DOI 10.1016/j.neuroscience.2009.08.017

Wolters EC, 2009, PARKINSONISM RELAT D, V15, pS6, DOI 10.1016/S1353-8020(09)70770-9

Wu KE, 2020, CELL SYST, V11, P102, DOI 10.1016/j.cels.2020.06.008

Wu ZC, 2022, INT J MOL SCI, V23, DOI 10.3390/ijms23063394

Xanthos DN, 2014, NAT REV NEUROSCI, V15, P43, DOI 10.1038/nrn3617

Yamamoto T, 2011, NEUROSCI LETT, V487, P335, DOI 10.1016/j.neulet.2010.10.051

Yang HT, 2021, NAT REV MICROBIOL, V19, P685, DOI 10.1038/s41579-021-00630-8

Yang LL, 2020, CELL STEM CELL, V27, P125, DOI 10.1016/j.stem.2020.06.015

Yavich L, 2005, NEUROBIOL DIS, V20, P303, DOI 10.1016/j.nbd.2005.03.010

Yavich L, 2004, J NEUROSCI, V24, P11165, DOI 10.1523/JNEUROSCI.2559-04.2004

Yirmiya R, 2011, BRAIN BEHAV IMMUN, V25, P181, DOI 10.1016/j.bbi.2010.10.015

ZALCMAN S, 1994, BRAIN RES, V643, P40, DOI 10.1016/0006-8993(94)90006-X

Zhao YC, 2021, CELL RES, V31, P818, DOI 10.1038/s41422-021-00495-9

Zoller H, 2012, J INTERF CYTOK RES, V32, P216, DOI 10.1089/jir.2011.0093

NR 223

TC 2

Z9 2

U1 2

U2 2

PU MDPI

PI BASEL

PA ST ALBAN-ANLAGE 66, CH-4052 BASEL, SWITZERLAND

SN 1661-6596

EI 1422-0067

J9 INT J MOL SCI

JI Int. J. Mol. Sci.

PD MAR

PY 2023

VL 24

IS 6

AR 5618

DI 10.3390/ijms24065618

PG 22

WC Biochemistry & Molecular Biology; Chemistry, Multidisciplinary

WE Science Citation Index Expanded (SCI-EXPANDED)

SC Biochemistry & Molecular Biology; Chemistry

GA A8TY7

UT WOS:000957800300001

PM 36982693

OA Green Published, gold

DA 2024-08-12

ER

PT J

AU Pellegrini, C

Fornai, M

Antonioli, L

Blandizzi, C

Calderone, V

AF Pellegrini, Carolina

Fornai, Matteo

Antonioli, Luca

Blandizzi, Corrado

Calderone, Vincenzo

TI Phytochemicals as Novel Therapeutic Strategies for NLRP3

Inflammasome-Related Neurological, Metabolic, and Inflammatory Diseases

SO INTERNATIONAL JOURNAL OF MOLECULAR SCIENCES

LA English

DT Review

DE NLRP3 inflammasome; neurological diseases; psychiatric diseases;

metabolic diseases; inflammatory diseases; phytochemicals

ID COLLAGEN-INDUCED ARTHRITIS; NF-KAPPA-B; THIOREDOXIN-INTERACTING PROTEIN;

CHRONIC INTESTINAL INFLAMMATION; CRYSTAL-INDUCED INFLAMMATION; INDUCED

DEPRESSIVE BEHAVIOR; MURINE EXPERIMENTAL COLITIS; ADIPOSE-TISSUE;

RHEUMATOID-ARTHRITIS; OXIDATIVE STRESS

AB Several lines of evidence point out the relevance of nucleotide-binding oligomerization domain leucine-rich repeat and pyrin domain-containing protein 3 (NLRP3) inflammasome as a pivotal player in the pathophysiology of several neurological and psychiatric diseases (i.e., Parkinson's disease (PD), Alzheimer's disease (AD), multiple sclerosis (MS), amyotrophic lateral sclerosis, and major depressive disorder), metabolic disorders (i.e., obesity and type 2 diabetes) and chronic inflammatory diseases (i.e., intestinal inflammation, arthritis, and gout). Intensive research efforts are being made to achieve an integrated view about the pathophysiological role of NLRP3 inflammasome pathways in such disorders. Evidence is also emerging that the pharmacological modulation of NLRP3 inflammasome by phytochemicals could represent a promising molecular target for the therapeutic management of neurological, psychiatric, metabolic, and inflammatory diseases. The present review article has been intended to provide an integrated and critical overview of the available clinical and experimental evidence about the role of NLRP3 inflammasome in the pathophysiology of neurological, psychiatric, metabolic, and inflammatory diseases, including PD, AD, MS, depression, obesity, type 2 diabetes, arthritis, and intestinal inflammation. Special attention has been paid to highlight and critically discuss current scientific evidence on the effects of phytochemicals on NLRP3 inflammasome pathways and their potential in counteracting central neuroinflammation, metabolic alterations, and immune/inflammatory responses in such diseases.

C1 [Pellegrini, Carolina; Calderone, Vincenzo] Univ Pisa, Dept Pharm, I-56126 Pisa, Italy.

[Fornai, Matteo; Antonioli, Luca; Blandizzi, Corrado] Univ Pisa, Dept Clin & Expt Med, I-56126 Pisa, Italy.

C3 University of Pisa; University of Pisa

RP Pellegrini, C (corresponding author), Univ Pisa, Dept Pharm, I-56126 Pisa, Italy.

EM carolina.pellegrini87@gmail.com; mfornai74@gmail.com; lucaant@gmail.com;

c.blandizzi@gmail.com; vincenzo.calderone@unipi.it

RI Blandizzi, Corrado/AAB-8736-2019; Pellegrini, Carolina/ABE-3462-2021;

Calderone, Vincenzo/L-9288-2015; antonioli, luca/K-1695-2018

OI Pellegrini, Carolina/0000-0003-2644-5430; Calderone,

Vincenzo/0000-0002-1441-5421; BLANDIZZI, CORRADO/0000-0001-9927-5386;

FORNAI, MATTEO/0000-0002-0812-3959; antonioli, luca/0000-0003-1241-617X

FU University of Pisa [PRA_2018_31]

FX The present work has been supported by the PRA_2018_31 granted by the

University of Pisa.

CR Aachoui Y, 2013, CURR OPIN MICROBIOL, V16, P319, DOI 10.1016/j.mib.2013.04.004

Alcocer-Gómez E, 2014, BRAIN BEHAV IMMUN, V36, P111, DOI 10.1016/j.bbi.2013.10.017

Antonopoulos C, 2015, J BIOL CHEM, V290, P20167, DOI 10.1074/jbc.M115.652321

Bauernfeind FG, 2009, J IMMUNOL, V183, P787, DOI 10.4049/jimmunol.0901363

Bellezza I, 2018, MOL NEUROBIOL, V55, P2350, DOI 10.1007/s12035-017-0502-x

Broderick L, 2015, ANNU REV PATHOL-MECH, V10, P395, DOI 10.1146/annurev-pathol-012414-040431

Cao YH, 2015, SCI TRANSL MED, V7, DOI 10.1126/scitranslmed.neurodegenerative disease8038

Cavalcanti NG, 2016, IMMUNOL INVEST, V45, P383, DOI 10.3109/08820139.2016.1153651

Chen MK, 2015, J IMMUNOL, V195, P4962, DOI 10.4049/jimmunol.1500865

Choi HK, 2005, ANN INTERN MED, V143, P499, DOI 10.7326/0003-4819-143-7-200510040-00009

Choulaki C, 2015, ARTHRITIS RES THER, V17, DOI 10.1186/s13075-015-0775-2

Chung H, 2016, CELL DEATH DIFFER, V23, P1331, DOI 10.1038/cdd.2016.14

Coccia M, 2012, J EXP MED, V209, P1595, DOI 10.1084/jem.20111453

Cocco M, 2017, J MED CHEM, V60, P3656, DOI 10.1021/acs.jmedchem.6b01624

Codolo G, 2013, PLOS ONE, V8, DOI 10.1371/journal.pone.0055375

Csiszar A, 2011, ANN NY ACAD SCI, V1215, P117, DOI 10.1111/j.1749-6632.2010.05848.x

Dayer JM, 2003, RHEUMATOLOGY, V42, P3, DOI 10.1093/rheumatology/keg326

Dhanasekar C, 2016, EUR J PHARMACOL, V786, P116, DOI 10.1016/j.ejphar.2016.06.005

Ding T, 2018, PHYTOMEDICINE, V41, P45, DOI 10.1016/j.phymed.2018.01.026

Donath MY, 2010, TRENDS ENDOCRIN MET, V21, P261, DOI 10.1016/j.tem.2009.12.010

Doss HM, 2016, LIFE SCI, V148, P201, DOI 10.1016/j.lfs.2016.02.004

Duncan JA, 2007, P NATL ACAD SCI USA, V104, P8041, DOI 10.1073/pnas.0611496104

Eo H, 2017, J AGR FOOD CHEM, V65, P3811, DOI 10.1021/acs.jafc.7b00357

Esser N, 2014, DIABETES RES CLIN PR, V105, P141, DOI 10.1016/j.diabres.2014.04.006

Fan Z, 2017, J NEUROINFLAMM, V14, DOI 10.1186/s12974-017-1036-x

Feng J, 2018, CNS NEUROSCI THER, V24, P1207, DOI 10.1111/cns.12983

Gao WQ, 2019, METAB BRAIN DIS, V34, P319, DOI 10.1007/s11011-018-0342-z

Goossens GH, 2012, MOL IMMUNOL, V50, P142, DOI 10.1016/j.molimm.2012.01.005

Gordon R, 2018, SCI TRANSL MED, V10, DOI 10.1126/scitranslmed.aah4066

Gringhuis SI, 2012, NAT IMMUNOL, V13, P246, DOI 10.1038/ni.2222

Guo C, 2018, CLIN EXP IMMUNOL, V194, P231, DOI 10.1111/cei.13167

Guo WJ, 2015, INT IMMUNOPHARMACOL, V24, P232, DOI 10.1016/j.intimp.2014.12.009

Gurung P, 2014, J IMMUNOL, V192, P1835, DOI 10.4049/jimmunol.1302839

Hara H, 2013, NAT IMMUNOL, V14, P1247, DOI 10.1038/ni.2749

He XX, 2016, SCI REP-UK, V6, DOI 10.1038/srep28370

Henao-Mejia J, 2014, ANNU REV PHYSIOL, V76, P57, DOI 10.1146/annurev-physiol-021113-170324

Heneka MT, 2018, NAT REV NEUROSCI, V19, P610, DOI 10.1038/s41583-018-0055-7

Heneka MT, 2013, NATURE, V493, P674, DOI 10.1038/nature11729

Honda H, 2014, J LEUKOCYTE BIOL, V96, P1087, DOI 10.1189/jlb.3A0114-005RR

Hsu SC, 2014, INT J BIOCHEM CELL B, V53, P361, DOI 10.1016/j.biocel.2014.06.002

Italiani P, 2014, J NEUROINFLAMM, V11, DOI 10.1186/1742-2094-11-94

Jahan S, 2017, CURR MED CHEM, V24, P1645, DOI 10.2174/0929867324666170227121619

Jenko B, 2016, J MED BIOCHEM, V35, P319, DOI 10.1515/jomb-2016-0008

Jhang JJ, 2016, MOL NUTR FOOD RES, V60, P2297, DOI 10.1002/mnfr.201600106

Jiang DL, 2018, CANCER LETT, V419, P8, DOI 10.1016/j.canlet.2018.01.034

Jiang P, 2017, J NEUROINFLAMM, V14, DOI 10.1186/s12974-017-1013-4

Kaufmann FN, 2017, BRAIN BEHAV IMMUN, V64, P367, DOI 10.1016/j.bbi.2017.03.002

Keane RW, 2018, FRONT NEUROL, V9, DOI 10.3389/fneur.2018.00135

Kim HK, 2016, J PSYCHIATR RES, V72, P43, DOI 10.1016/j.jpsychires.2015.10.015

Kingsbury SR, 2011, J INFLAMM RES, V4, P39, DOI 10.2147/JIR.S11330

Latz E, 2013, NAT REV IMMUNOL, V13, P397, DOI 10.1038/nri3452

Lazaridis LD, 2017, DIGEST DIS SCI, V62, P2348, DOI 10.1007/s10620-017-4609-8

Lee HM, 2013, DIABETES, V62, P194, DOI 10.2337/db12-0420

Li AY, 2016, MOL CELL ENDOCRINOL, V434, P36, DOI 10.1016/j.mce.2016.06.008

Li RP, 2016, BEHAV BRAIN RES, V296, P318, DOI 10.1016/j.bbr.2015.09.031

Lissner D, 2015, INFLAMM BOWEL DIS, V21, P1297, DOI 10.1097/MIB.0000000000000384

Liu HJ, 2017, J NEUROINFLAMM, V14, DOI 10.1186/s12974-017-0849-y

Liu L, 2017, J CROHNS COLITIS, V11, P737, DOI 10.1093/ecco-jcc/jjw219

Liu TY, 2019, PSYCHOPHARMACOLOGY, V236, P1385, DOI [10.1007/s00213-018-5148-5, 10.1109/ieee-iws.2019.8804075]

Liu XX, 2017, INT IMMUNOPHARMACOL, V48, P30, DOI 10.1016/j.intimp.2017.04.019

Liu X, 2015, BRAIN BEHAV IMMUN, V46, P121, DOI 10.1016/j.bbi.2015.01.011

de Oliveira GAL, 2019, CHEM-BIOL INTERACT, V297, P25, DOI 10.1016/j.cbi.2018.10.020

Lu B, 2013, PROTEIN CELL, V4, P163, DOI 10.1007/s13238-012-2118-2

Mangan MSJ, 2018, NAT REV DRUG DISCOV, V17, P588, DOI [10.1038/nrd.2018.97, 10.1038/nrd.2018.149]

Márquez-Flores YK, 2016, J NUTR BIOCHEM, V30, P143, DOI 10.1016/j.jnutbio.2015.12.002

Martin BN, 2016, NAT IMMUNOL, V17, P583, DOI 10.1038/ni.3389

Martinon F, 2006, NATURE, V440, P237, DOI 10.1038/nature04516

Martinon F, 2010, IMMUNOL REV, V233, P218, DOI 10.1111/j.0105-2896.2009.00860.x

Mathews RJ, 2014, ANN RHEUM DIS, V73, P1202, DOI 10.1136/annrheumdis-2013-203276

McGillicuddy FC, 2011, DIABETES, V60, P1688, DOI 10.2337/db10-1278

McGonagle D, 2007, ANN RHEUM DIS, V66, P1683, DOI 10.1136/ard.2007.073759

Ming X, 2002, J NEUROL SCI, V197, P9, DOI 10.1016/S0022-510X(02)00030-8

Misawa T, 2015, INT IMMUNOL, V27, P425, DOI 10.1093/intimm/dxv018

Mitroulis I, 2013, SEMIN IMMUNOPATHOL, V35, P501, DOI 10.1007/s00281-013-0361-0

Moossavi M, 2018, MOL CANCER, V17, DOI 10.1186/s12943-018-0900-3

Mylona EE, 2012, ARTHRITIS RES THER, V14, DOI 10.1186/ar3898

Neurath MF, 2014, NAT REV IMMUNOL, V14, P329, DOI 10.1038/nri3661

Nomura J, 2015, J IMMUNOL, V195, P5718, DOI 10.4049/jimmunol.1402512

Normand S, 2011, P NATL ACAD SCI USA, V108, P9601, DOI 10.1073/pnas.1100981108

Ota T, 2014, FRONT ENDOCRINOL, V5, DOI 10.3389/fendo.2014.00204

Paramel GV, 2015, MEDIAT INFLAMM, V2015, DOI 10.1155/2015/846782

Pellegrini C, 2018, FRONT PHARMACOL, V9, DOI 10.3389/fphar.2018.01405

Pellegrini C, 2017, FRONT IMMUNOL, V8, DOI 10.3389/fimmu.2017.00036

Peng YH, 2016, MOL NUTR FOOD RES, V60, P2331, DOI 10.1002/mnfr.201600332

Perera AP, 2018, SCI REP-UK, V8, DOI 10.1038/s41598-018-26775-w

Perera AP, 2017, CURR PHARM DESIGN, V23, P2321, DOI 10.2174/1381612823666170201162414

Qi Y, 2019, METAB BRAIN DIS, V34, P257, DOI 10.1007/s11011-018-0348-6

Qin XY, 2016, JAMA NEUROL, V73, P1316, DOI 10.1001/jamaneurol.2016.2742

Rheinheimer J, 2017, METABOLISM, V74, P1, DOI 10.1016/j.metabol.2017.06.002

Rosengren S, 2005, ANN RHEUM DIS, V64, P708, DOI 10.1136/ard.2004.025577

Ruscitti P, 2015, CLIN EXP IMMUNOL, V182, P35, DOI 10.1111/cei.12667

Saresella M, 2016, MOL NEURODEGENER, V11, DOI 10.1186/s13024-016-0088-1

Shen HH, 2018, AUTOIMMUN REV, V17, P694, DOI 10.1016/j.autrev.2018.01.020

Shim DW, 2016, J MED FOOD, V19, P310, DOI 10.1089/jmf.2015.3556

So A, 2007, ARTHRITIS RES THER, V9, DOI 10.1186/ar2143

So A, 2010, ARTHRITIS RHEUM-US, V62, P3064, DOI 10.1002/art.27600

Song LM, 2017, FRONT CELL NEUROSCI, V11, DOI 10.3389/fncel.2017.00063

Staurengo-Ferrari L, 2018, FRONT PHARMACOL, V9, DOI 10.3389/fphar.2018.01123

Stienstra R, 2011, P NATL ACAD SCI USA, V108, P15324, DOI 10.1073/pnas.1100255108

Stienstra R, 2010, CELL METAB, V12, P593, DOI 10.1016/j.cmet.2010.11.011

Strowig T, 2012, NATURE, V481, P278, DOI 10.1038/nature10759

Sun Y, 2015, BIOCHEM PHARMACOL, V94, P142, DOI 10.1016/j.bcp.2015.02.002

Sutterwala FS, 2014, ANN NY ACAD SCI, V1319, P82, DOI 10.1111/nyas.12458

Tan FCC, 2014, BIOGERONTOLOGY, V15, P643, DOI 10.1007/s10522-014-9532-1

Terkeltaub R, 2009, ANN RHEUM DIS, V68, P1613, DOI 10.1136/ard.2009.108936

Tha KK, 2000, BRAIN RES, V885, P25, DOI 10.1016/S0006-8993(00)02883-3

Vandanmagsar B, 2011, NAT MED, V17, P179, DOI 10.1038/nm.2279

Venegas C, 2017, NATURE, V552, P355, DOI 10.1038/nature25158

Wang JC, 2018, BIOMED PHARMACOTHER, V106, P1250, DOI 10.1016/j.biopha.2018.07.063

Wang W, 2016, P NATL ACAD SCI USA, V113, P9587, DOI 10.1073/pnas.1610099113

Wang W, 2013, BRIT J PHARMACOL, V169, P1352, DOI 10.1111/bph.12226

Wang X, 2017, MOLECULES, V22, DOI 10.3390/molecules22081315

Wei XQ, 2001, J IMMUNOL, V166, P517, DOI 10.4049/jimmunol.166.1.517

Wen HT, 2011, NAT IMMUNOL, V12, P408, DOI 10.1038/ni.2022

Wu DC, 2018, MEDIAT INFLAMM, V2018, DOI 10.1155/2018/3048532

Wu XF, 2014, TOXICOL APPL PHARM, V281, P146, DOI 10.1016/j.taap.2014.10.002

Yamamoto S, 2017, J NEUROINFLAMM, V14, DOI 10.1186/s12974-017-0923-5

Yang G, 2018, RHEUMATOLOGY, V57, P727, DOI 10.1093/rheumatology/kex499

Yang SJ, 2014, METABOLISM, V63, P693, DOI 10.1016/j.metabol.2014.02.003

Yang Y, 2018, INT IMMUNOPHARMACOL, V54, P153, DOI 10.1016/j.intimp.2017.11.013

Yi YS, 2018, KOREAN J PHYSIOL PHA, V22, P1, DOI 10.4196/kjpp.2018.22.1.1

Yousuf S, 2009, BRAIN RES, V1250, P242, DOI 10.1016/j.brainres.2008.10.068

Zaki MH, 2010, IMMUNITY, V32, P379, DOI 10.1016/j.immuni.2010.03.003

Zendedel A, 2016, MOL NEUROBIOL, V53, P3063, DOI 10.1007/s12035-015-9203-5

Zhang H, 2015, CLIN IMMUNOL, V157, P175, DOI 10.1016/j.clim.2015.02.001

Zhang L, 2018, BRAIN RES BULL, V142, P352, DOI 10.1016/j.brainresbull.2018.08.021

Zhang Y, 2015, INT J NEUROPSYCHOPH, V18, DOI 10.1093/ijnp/pyv006

Zhou RB, 2011, NATURE, V469, P221, DOI 10.1038/nature09663

Zhou RB, 2010, NAT IMMUNOL, V11, P136, DOI 10.1038/ni.1831

Zhou Y, 2016, MOL NEURODEGENER, V11, DOI 10.1186/s13024-016-0094-3

NR 130

TC 67

Z9 73

U1 1

U2 19

PU MDPI

PI BASEL

PA ST ALBAN-ANLAGE 66, CH-4052 BASEL, SWITZERLAND

SN 1661-6596

EI 1422-0067

J9 INT J MOL SCI

JI Int. J. Mol. Sci.

PD JUN 2

PY 2019

VL 20

IS 12

AR 2876

DI 10.3390/ijms20122876

PG 31

WC Biochemistry & Molecular Biology; Chemistry, Multidisciplinary

WE Science Citation Index Expanded (SCI-EXPANDED)

SC Biochemistry & Molecular Biology; Chemistry

GA IG4ED

UT WOS:000473756000026

PM 31200447

OA Green Submitted, Green Published, gold

DA 2024-08-12

ER

PT J

AU Lénárt, N

Brough, D

Dénes, A

AF Lenart, Nikolett

Brough, David

Denes, Adam

TI Inflammasomes link vascular disease with neuroinflammation and brain

disorders

SO JOURNAL OF CEREBRAL BLOOD FLOW AND METABOLISM

LA English

DT Review

DE Inflammation; brain disease; inflammasome; systemic; neurodegeneration

ID NONSTEROIDAL ANTIINFLAMMATORY DRUGS; INTERLEUKIN-1 RECEPTOR ANTAGONIST;

THIOREDOXIN-INTERACTING PROTEIN; INDUCED PULMONARY-HYPERTENSION;

TOLL-LIKE-RECEPTORS; NLRP3 INFLAMMASOME; alzheimer's disease; NALP3

INFLAMMASOME; RISK-FACTOR; CELL-DEATH

AB The role of inflammation in neurological disorders is increasingly recognised. Inflammatory processes are associated with the aetiology and clinical progression of migraine, psychiatric conditions, epilepsy, cerebrovascular diseases, dementia and neurodegeneration, such as seen in Alzheimer's or Parkinson's disease. Both central and systemic inflammatory actions have been linked with the development of brain diseases, suggesting that complex neuro-immune interactions could contribute to pathological changes in the brain across multiple temporal and spatial scales. However, the mechanisms through which inflammation impacts on neurological disease are improperly defined. To develop effective therapeutic approaches, it is imperative to understand how detrimental inflammatory processes could be blocked selectively, or controlled for prolonged periods, without compromising essential immune defence mechanisms. Increasing evidence indicates that common risk factors for brain disorders, such as atherosclerosis, diabetes, hypertension, obesity or infection involve the activation of NLRP3, NLRP1, NLRC4 or AIM2 inflammasomes, which are also associated with various neurological diseases. This review focuses on the mechanisms whereby inflammasomes, which integrate diverse inflammatory signals in response to pathogen-driven stimuli, tissue injury or metabolic alterations in multiple cell types and different organs of the body, could functionally link vascular- and neurological diseases and hence represent a promising therapeutic target.

C1 [Lenart, Nikolett; Denes, Adam] Inst Expt Med, Lab Neuroimmunol, Szigony U 43, H-1083 Budapest, Hungary.

[Brough, David] Univ Manchester, Fac Biol Med & Hlth, Manchester, Lancs, United Kingdom.

C3 Hungarian Academy of Sciences; Hungarian Research Network; HUN-REN

Institute of Experimental Medicine; University of Manchester

RP Dénes, A (corresponding author), Inst Expt Med, Lab Neuroimmunol, Szigony U 43, H-1083 Budapest, Hungary.

EM denes.adam@koki.mta.hu

OI Brough, David/0000-0002-2250-2381

FU OTKA [K109743]; Hungarian Brain Research Program [KTIA_13_NAP-A-I/2];

Hungarian Academy of Sciences; Janos Bolyai Research Scholarship

FX The author(s) disclosed receipt of the following financial support for

the research, authorship, and/or publication of this article: OTKA

K109743 (AD), the Hungarian Brain Research Program KTIA_13_NAP-A-I/2

(AD), and the Janos Bolyai Research Scholarship and the 'Lendulet'

program of the Hungarian Academy of Sciences (AD).

CR Abbate A, 2015, AM J CARDIOL, V115, P288, DOI 10.1016/j.amjcard.2014.11.003

Abderrazak A, 2015, CIRCULATION, V131, P1061, DOI 10.1161/CIRCULATIONAHA.114.013730

Abulafia DP, 2009, J CEREBR BLOOD F MET, V29, P534, DOI 10.1038/jcbfm.2008.143

Adamczak S, 2012, J NEUROSURG, V117, P1119, DOI 10.3171/2012.9.JNS12815

Adamczak SE, 2014, J CEREBR BLOOD F MET, V34, P621, DOI 10.1038/jcbfm.2013.236

Ahmad R, 2006, AIDS, V20, P1907, DOI 10.1097/01.aids.0000244217.46445.45

Alboni S, 2010, J NEUROINFLAMM, V7, DOI 10.1186/1742-2094-7-9

Alcocer-Gómez E, 2014, CNS NEUROSCI THER, V20, P294, DOI 10.1111/cns.12230

Alfonso-Loeches S, 2014, FRONT CELL NEUROSCI, V8, DOI 10.3389/fncel.2014.00216

Allen C, 2012, J IMMUNOL, V189, P381, DOI 10.4049/jimmunol.1200409

Arman A, 2010, EUR CYTOKINE NETW, V21, P116, DOI 10.1684/ecn.2010.0186

Bakele M, 2014, J BIOL CHEM, V289, P5320, DOI 10.1074/jbc.M113.505636

Baldwin AG, 2016, J MED CHEM, V59, P1691, DOI 10.1021/acs.jmedchem.5b01091

Bando S, 2015, ATHEROSCLEROSIS, V242, P407, DOI 10.1016/j.atherosclerosis.2015.07.043

Bargiotas P, 2011, P NATL ACAD SCI USA, V108, P20772, DOI 10.1073/pnas.1018262108

Baroja-Mazo A, 2014, NAT IMMUNOL, V15, P738, DOI 10.1038/ni.2919

Bauernfeind F, 2011, J IMMUNOL, V187, P613, DOI 10.4049/jimmunol.1100613

Benakis C, 2016, NAT MED, V22, P516, DOI 10.1038/nm.4068

Bergsbaken T, 2009, NAT REV MICROBIOL, V7, P99, DOI 10.1038/nrmicro2070

Bleda S, 2014, INT J CARDIOL, V172, pE282, DOI 10.1016/j.ijcard.2013.12.201

BlumDegen D, 1995, NEUROSCI LETT, V202, P17, DOI 10.1016/0304-3940(95)12192-7

Bonora M, 2015, ONCOGENE, V34, P1608, DOI [10.1038/onc.2014.462, 10.1038/onc.2014.96]

Brough D, 2015, EXP PHYSIOL, V100, P1488, DOI 10.1113/EP085135

Brough D, 2015, IUBMB LIFE, V67, P323, DOI 10.1002/iub.1377

Burm SM, 2015, J NEUROSCI, V35, P678, DOI 10.1523/JNEUROSCI.2510-14.2015

Cai H, 2012, CURR ALZHEIMER RES, V9, P5, DOI 10.2174/156720512799015064

Cai X, 2014, CELL, V156, P1207, DOI 10.1016/j.cell.2014.01.063

Caso JR, 2009, AM J PHYSIOL-REG I, V296, pR979, DOI 10.1152/ajpregu.90825.2008

Cero FT, 2015, AM J PHYSIOL-LUNG C, V309, pL378, DOI 10.1152/ajplung.00342.2014

Chen KWW, 2014, CELL REP, V8, P569, DOI 10.1016/j.celrep.2014.06.028

Chen Z, 2015, CIRCULATION, V131, P805, DOI 10.1161/CIRCULATIONAHA.114.013675

Cisneros-Mejorado A, 2015, J CEREBR BLOOD F MET, V35, P843, DOI 10.1038/jcbfm.2014.262

Clarson LE, 2015, ANN RHEUM DIS, V74, P642, DOI 10.1136/annrheumdis-2014-205252

Codolo G, 2013, PLOS ONE, V8, DOI 10.1371/journal.pone.0055375

Coll RC, 2015, NAT MED, V21, P248, DOI 10.1038/nm.3806

Curtiss LK, 2009, J LIPID RES, V50, pS340, DOI 10.1194/jlr.R800056-JLR200

Dalekos GN, 1997, J LAB CLIN MED, V129, P300, DOI 10.1016/S0022-2143(97)90178-5

Denes A, 2010, BRAIN BEHAV IMMUN, V24, P708, DOI 10.1016/j.bbi.2009.09.010

Denes A, 2015, P NATL ACAD SCI USA, V112, P4050, DOI 10.1073/pnas.1419090112

Dénes A, 2014, ANN NEUROL, V75, P670, DOI 10.1002/ana.24146

Denes A, 2012, J AM HEART ASSOC, V1, DOI 10.1161/JAHA.112.002006

Denes A, 2011, CEREBROVASC DIS, V32, P517, DOI 10.1159/000332205

Dinarello CA, 2006, AM J CLIN NUTR, V83, p447S

Dinarello CA, 2011, BLOOD, V117, P3720, DOI 10.1182/blood-2010-07-273417

Dixit VD, 2013, DIABETES, V62, P22, DOI 10.2337/db12-1115

Drake C, 2011, BRAIN BEHAV IMMUN, V25, P1113, DOI 10.1016/j.bbi.2011.02.008

Duewell P, 2010, NATURE, V464, P1357, DOI 10.1038/nature08938

Dumas A, 2014, PLOS PATHOG, V10, DOI 10.1371/journal.ppat.1004150

Emsley HCA, 2005, J NEUROL NEUROSUR PS, V76, P1366, DOI 10.1136/jnnp.2004.054882

Fann DYW, 2013, CELL DEATH DIS, V4, DOI 10.1038/cddis.2013.326

Feng L, 2015, J NEUROINFLAMM, V12, DOI 10.1186/s12974-015-0409-2

Fernandes-Alnemri T, 2009, NATURE, V458, P509, DOI 10.1038/nature07710

Forlenza OV, 2009, DEMENT GERIATR COGN, V28, P507, DOI 10.1159/000255051

Fowler BJ, 2014, SCIENCE, V346, P1000, DOI 10.1126/science.1261754

Frank MG, 2016, BRAIN BEHAV IMMUN, V55, P215, DOI 10.1016/j.bbi.2015.10.009

Franklin BS, 2014, NAT IMMUNOL, V15, P727, DOI 10.1038/ni.2913

Freeman D, 2013, PLOS ONE, V8, DOI 10.1371/journal.pone.0062143

Geldhoff M, 2013, BMC INFECT DIS, V13, DOI 10.1186/1471-2334-13-358

Ghosh S, 2013, J NEUROSCI, V33, P5053, DOI 10.1523/JNEUROSCI.4361-12.2013

Giles JA, 2015, EUR J IMMUNOL, V45, P525, DOI 10.1002/eji.201444748

Gracie JA, 2003, J LEUKOCYTE BIOL, V73, P213, DOI 10.1189/jlb.0602313

Gris D, 2010, J IMMUNOL, V185, P974, DOI 10.4049/jimmunol.0904145

Guo HT, 2015, NAT MED, V21, P677, DOI 10.1038/nm.3893

Gustin A, 2015, PLOS ONE, V10, DOI 10.1371/journal.pone.0130624

Gustot A, 2015, BIOCHEM J, V471, P323, DOI 10.1042/BJ20150617

Hafner-Bratkovic I, 2012, CELL MOL LIFE SCI, V69, P4215, DOI 10.1007/s00018-012-1140-0

Halle A, 2008, NAT IMMUNOL, V9, P857, DOI 10.1038/ni.1636

Hanamsagar R, 2014, J NEUROCHEM, V129, P704, DOI 10.1111/jnc.12669

He WT, 2015, CELL RES, V25, P1285, DOI 10.1038/cr.2015.139

Helmy A, 2014, J CEREBR BLOOD F MET, V34, P845, DOI 10.1038/jcbfm.2014.23

Heneka MT, 2015, LANCET NEUROL, V14, P388, DOI 10.1016/S1474-4422(15)70016-5

Heneka MT, 2013, NATURE, V493, P674, DOI 10.1038/nature11729

Hoegen T, 2011, J IMMUNOL, V187, P5440, DOI 10.4049/jimmunol.1100790

Hölscher C, 2011, BIOCHEM SOC T, V39, P891, DOI 10.1042/BST0390891

Honda H, 2014, J LEUKOCYTE BIOL, V96, P1087, DOI 10.1189/jlb.3A0114-005RR

Horng T, 2014, TRENDS IMMUNOL, V35, P253, DOI 10.1016/j.it.2014.02.007

Hornung V, 2009, NATURE, V458, P514, DOI 10.1038/nature07725

Hottz ED, 2015, MEDIAT INFLAMM, V2015, DOI 10.1155/2015/435783

Hottz ED, 2013, BLOOD, V122, P3405, DOI 10.1182/blood-2013-05-504449

Houlden A, 2016, BRAIN BEHAV IMMUN, V57, P10, DOI 10.1016/j.bbi.2016.04.003

Inoue M, 2012, P NATL ACAD SCI USA, V109, P10480, DOI 10.1073/pnas.1201836109

Ito M, 2015, NAT COMMUN, V6, DOI 10.1038/ncomms8360

Jamilloux Y, 2013, GLIA, V61, P539, DOI 10.1002/glia.22454

Jha S, 2010, J NEUROSCI, V30, P15811, DOI 10.1523/JNEUROSCI.4088-10.2010

Johann S, 2015, GLIA, V63, P2260, DOI 10.1002/glia.22891

Kahlenberg JM, 2011, J IMMUNOL, V187, P6143, DOI 10.4049/jimmunol.1101284

Karmakar M, 2015, J IMMUNOL, V194, P1763, DOI 10.4049/jimmunol.1401624

Kaushal V, 2015, CELL DEATH DIFFER, V22, P1676, DOI 10.1038/cdd.2015.16

Kayagaki N, 2015, NATURE, V526, P666, DOI 10.1038/nature15541

Khakpour S, 2015, INNATE IMMUN-LONDON, V21, P827, DOI 10.1177/1753425915606525

Kleinschnitz C, 2007, CIRCULATION, V115, P2323, DOI 10.1161/CIRCULATIONAHA.107.691279

Klen J, 2015, J DIABETES RES, V2015, DOI 10.1155/2015/616747

Kofoed EM, 2011, NATURE, V477, P592, DOI 10.1038/nature10394

Kolaczkowska E, 2013, NAT REV IMMUNOL, V13, P159, DOI 10.1038/nri3399

Kolly L, 2009, J IMMUNOL, V183, P4003, DOI 10.4049/jimmunol.0802173

Krishnan SM, 2016, BRIT J PHARMACOL, V173, P752, DOI 10.1111/bph.13230

Kunnas T, 2015, IMMUN AGEING, V12, DOI 10.1186/s12979-015-0047-7

L'homme L, 2013, J LIPID RES, V54, P2998, DOI 10.1194/jlr.M037861

Lamkanfi M, 2014, CELL, V157, P1013, DOI 10.1016/j.cell.2014.04.007

Lamkanfi M, 2012, ANNU REV CELL DEV BI, V28, P137, DOI 10.1146/annurev-cellbio-101011-155745

Lau LT, 2001, J NEUROTRAUM, V18, P351, DOI 10.1089/08977150151071035

Lee HM, 2013, DIABETES, V62, P194, DOI 10.2337/db12-0420

Li JR, 2016, MOL NEUROBIOL, V53, P2668, DOI 10.1007/s12035-015-9318-8

Liao KC, 2013, INFECT IMMUN, V81, P570, DOI 10.1128/IAI.01003-12

Lin YH, 2009, CEREBROVASC DIS, V28, P391, DOI 10.1159/000235626

Lindemann S, 2001, J CELL BIOL, V154, P485, DOI 10.1083/jcb.200105058

Liu HD, 2013, NEUROCHEM RES, V38, P2072, DOI 10.1007/s11064-013-1115-z

Liu L, 2014, NEUROBIOL AGING, V35, P309, DOI 10.1016/j.neurobiolaging.2013.08.016

Liu PH, 2015, BIOCHEM BIOPH RES CO, V468, P319, DOI 10.1016/j.bbrc.2015.10.105

Lleo A, 2007, CELL MOL LIFE SCI, V64, P1403, DOI 10.1007/s00018-007-6516-1

Lopez-Castejon G, 2011, CYTOKINE GROWTH F R, V22, P189, DOI 10.1016/j.cytogfr.2011.10.001

Lu A, 2014, CELL, V156, P1193, DOI 10.1016/j.cell.2014.02.008

Lu M, 2014, NEUROBIOL AGING, V35, P421, DOI 10.1016/j.neurobiolaging.2013.08.015

Lu YC, 2008, CYTOKINE, V42, P145, DOI 10.1016/j.cyto.2008.01.006

Ma QY, 2014, ANN NEUROL, V75, P209, DOI 10.1002/ana.24070

Maheshwari P, 2015, J ALZHEIMERS DIS, V43, P957, DOI 10.3233/JAD-140621

Maltez VI, 2016, J IMMUNOL, V196, P956, DOI 10.4049/jimmunol.1502060

Martin-Rodriguez S, 2015, EUR J CLIN INVEST, V45, P160, DOI 10.1111/eci.12392

Martín-Sánchez F, 2016, CELL DEATH DIFFER, V23, P1219, DOI 10.1038/cdd.2015.176

Martinon F, 2006, NATURE, V440, P237, DOI 10.1038/nature04516

Masters SL, 2010, NAT IMMUNOL, V11, P897, DOI 10.1038/ni.1935

Mathews RJ, 2014, ANN RHEUM DIS, V73, P1202, DOI 10.1136/annrheumdis-2013-203276

Mawhinney LJ, 2011, BMC NEUROSCI, V12, DOI 10.1186/1471-2202-12-123

Maysami S, 2016, J CEREBR BLOOD F MET, V36, P596, DOI 10.1177/0271678X15606714

McGeer PL, 2002, NEUROSCI LETT, V326, P67, DOI 10.1016/S0304-3940(02)00300-2

Meissner F, 2010, P NATL ACAD SCI USA, V107, P13046, DOI 10.1073/pnas.1002396107

Meng XF, 2014, MOL NEUROBIOL, V49, P673, DOI 10.1007/s12035-013-8551-2

Migita K, 2014, PLOS ONE, V9, DOI 10.1371/journal.pone.0096703

Miller EC, 2016, CURR NEUROL NEUROSCI, V16, DOI 10.1007/s11910-015-0602-9

Minghetti L, 2005, CURR OPIN NEUROL, V18, P315, DOI 10.1097/01.wco.0000169752.54191.97

Minkiewicz J, 2013, GLIA, V61, P1113, DOI 10.1002/glia.22499

Miranda-Hernandez S, 2013, AM J CLIN EXP IMMUNO, V2, P75

Mohamed IN, 2014, DIABETOLOGIA, V57, P413, DOI 10.1007/s00125-013-3101-z

Morrell CN, 2014, BLOOD, V123, P2759, DOI 10.1182/blood-2013-11-462432

Muñoz-Planillo R, 2013, IMMUNITY, V38, P1142, DOI 10.1016/j.immuni.2013.05.016

Murphy AJ, 2016, CELL METAB, V23, P155, DOI 10.1016/j.cmet.2015.09.024

Murray KN, 2015, FRONT CELL NEUROSCI, V9, DOI 10.3389/fncel.2015.00018

Murray KN, 2013, MOL CELL NEUROSCI, V53, P14, DOI 10.1016/j.mcn.2012.09.004

Nagyoszi P, 2015, J NEUROCHEM, V135, P551, DOI 10.1111/jnc.13197

Najjar S, 2013, J NEUROINFLAMM, V10, DOI 10.1186/1742-2094-10-43

Nakahira K, 2011, NAT IMMUNOL, V12, P222, DOI 10.1038/ni.1980

Netea MG, 2016, CELL METAB, V23, P6, DOI 10.1016/j.cmet.2015.12.014

Newton K, 2012, CSH PERSPECT BIOL, V4, DOI 10.1101/cshperspect.a006049

Nguyen-Oghalai TU, 2008, ARTHRIT RHEUM-ARTHR, V59, P984, DOI 10.1002/art.23816

Niemi K, 2011, J IMMUNOL, V186, P6119, DOI 10.4049/jimmunol.1002843

Ojala J, 2009, NEUROBIOL AGING, V30, P198, DOI 10.1016/j.neurobiolaging.2007.06.006

Omi T, 2006, EUR J HUM GENET, V14, P1295, DOI 10.1038/sj.ejhg.5201698

Pandey S, 2006, IMMUNOL CELL BIOL, V84, P333, DOI 10.1111/j.1440-1711.2006.01444.x

Peng K, 2015, INT J MOL MED, V35, P1179, DOI 10.3892/ijmm.2015.2129

Perry VH, 2007, NAT REV IMMUNOL, V7, P161, DOI 10.1038/nri2015

Petrilli V, 2007, CELL DEATH DIFFER, V14, P1583, DOI 10.1038/sj.cdd.4402195

Pontillo A, 2012, ALZ DIS ASSOC DIS, V26, P277, DOI 10.1097/WAD.0b013e318231a8ac

Qi J, 2015, CARDIOVASC TOXICOL, DOI [10.1007/s12012-015-9344-9, DOI 10.1007/s12012-015-9344-9.]

Qi J, 2016, CARDIOVASC TOXICOL, V16, P298, DOI 10.1007/s12012-015-9338-7

Rainone V., 2016, INT J OBES LOND

Rajamäki K, 2013, J BIOL CHEM, V288, P13410, DOI 10.1074/jbc.M112.426254

Ramaswamy V, 2013, J NEUROINFLAMM, V10, DOI 10.1186/1742-2094-10-152

Rapsinski GJ, 2015, INFECT IMMUN, V83, P693, DOI 10.1128/IAI.02370-14

Ravaglia G, 2006, EXP GERONTOL, V41, P85, DOI 10.1016/j.exger.2005.10.005

Roberts RL, 2011, ATHEROSCLEROSIS, V218, P123, DOI 10.1016/j.atherosclerosis.2011.04.043

Rock KL, 2008, ANNU REV PATHOL-MECH, V3, P99, DOI 10.1146/annurev.pathmechdis.3.121806.151456

Rodrigues RJ, 2015, FRONT NEUROSCI-SWITZ, V9, DOI 10.3389/fnins.2015.00148

Ruscitti P, 2015, CLIN EXP IMMUNOL, V182, P35, DOI 10.1111/cei.12667

Salminen A, 2009, PROG NEUROBIOL, V87, P181, DOI 10.1016/j.pneurobio.2009.01.001

Saresella M, 2016, MOL NEURODEGENER, V11, DOI 10.1186/s13024-016-0088-1

Savage CD, 2012, FRONT IMMUNOL, V3, DOI 10.3389/fimmu.2012.00288

Schiess M, 2003, ARCH NEUROL-CHICAGO, V60, P1043, DOI 10.1001/archneur.60.8.1043

Schuh E, 2015, NEUROL-NEUROIMMUNOL, V2, DOI 10.1212/NXI.0000000000000109

Seminog OO, 2013, RHEUMATOLOGY, V52, P2251, DOI 10.1093/rheumatology/ket293

Serhan CN, 2005, NAT IMMUNOL, V6, P1191, DOI 10.1038/ni1276

Shaftel SS, 2007, J NEUROSCI, V27, P9301, DOI 10.1523/JNEUROSCI.1418-07.2007

Shi FS, 2012, J NEUROINFLAMM, V9, DOI 10.1186/1742-2094-9-73

Shi JJ, 2015, NATURE, V526, P660, DOI 10.1038/nature15514

Shimada K, 2012, IMMUNITY, V36, P401, DOI 10.1016/j.immuni.2012.01.009

Silverman WR, 2009, J BIOL CHEM, V284, P18143, DOI 10.1074/jbc.M109.004804

Simpson JL, 2014, EUR RESPIR J, V43, P1067, DOI 10.1183/09031936.00105013

Smith CJ, 2013, J NEUROIMMUNE PHARM, V8, P867, DOI 10.1007/s11481-013-9469-1

So A, 2007, ARTHRITIS RES THER, V9, DOI 10.1186/ar2143

Tan CC, 2015, J NEUROINFLAMM, V12, DOI 10.1186/s12974-014-0233-0

Tan MS, 2014, CELL DEATH DIS, V5, DOI 10.1038/cddis.2014.348

Tang B, 2015, INT J CARDIOL, V180, P134, DOI 10.1016/j.ijcard.2014.11.161

Terkeltaub R, 2009, ANN RHEUM DIS, V68, P1613, DOI 10.1136/ard.2009.108936

Thornton P, 2010, BLOOD, V115, P3632, DOI 10.1182/blood-2009-11-252643

Umegaki H, 2012, ADV EXP MED BIOL, V724, P258, DOI 10.1007/978-1-4614-0653-2_19

Vaccari JPDR, 2008, J NEUROSCI, V28, P3404, DOI 10.1523/JNEUROSCI.0157-08.2008

Vaccari JPD, 2016, J NEUROCHEM, V136, P39, DOI 10.1111/jnc.13036

Vaccari JPD, 2009, J CEREBR BLOOD F MET, V29, P1251, DOI 10.1038/jcbfm.2009.46

Vagelatos NT, 2013, EPIDEMIOL REV, V35, P152, DOI 10.1093/epirev/mxs012

Vandanmagsar B, 2011, NAT MED, V17, P179, DOI 10.1038/nm.2279

Varghese GP, 2015, MOL MED REP, V11, P4579, DOI 10.3892/mmr.2015.3236

Vezzani A, 2011, NAT REV NEUROL, V7, P31, DOI 10.1038/nrneurol.2010.178

Villegas LR, 2013, ANTIOXID REDOX SIGN, V18, P1753, DOI 10.1089/ars.2012.4799

Waeber C, 2005, NEUROLOGY, V64, pS9, DOI 10.1212/WNL.64.10_suppl_2.S9

Wang JG, 2011, BLOOD, V118, P2366, DOI 10.1182/blood-2011-01-330878

Warnatsch A, 2015, SCIENCE, V349, P316, DOI 10.1126/science.neurodegenerative disease8064

Weber MD, 2015, J NEUROSCI, V35, P316, DOI 10.1523/JNEUROSCI.3561-14.2015

Wen HT, 2013, IMMUNITY, V39, P432, DOI 10.1016/j.immuni.2013.08.037

Wen HT, 2011, NAT IMMUNOL, V12, P408, DOI 10.1038/ni.2022

Wes PD, 2014, PLOS ONE, V9, DOI 10.1371/journal.pone.0106050

Wiseman SJ, 2016, STROKE, V47, P943, DOI 10.1161/STROKEAHA.115.012052

Wong ML, 2016, MOL PSYCHIATR, V21, P797, DOI 10.1038/mp.2016.46

Xia M, 2014, AM J PATHOL, V184, P1617, DOI 10.1016/j.ajpath.2014.01.032

Xiang M, 2011, J IMMUNOL, V187, P4809, DOI 10.4049/jimmunol.1102093

Yaidikar L, 2015, MOL CELL BIOCHEM, V402, P141, DOI 10.1007/s11010-014-2321-y

Yaidikar L, 2014, J STROKE CEREBROVASC, V23, P2869, DOI 10.1016/j.jstrokecerebrovasdis.2014.07.020

Yamazaki H, 2012, RHEUMATOL INT, V32, P1625, DOI 10.1007/s00296-011-1825-y

Yan YQ, 2013, IMMUNITY, V38, P1154, DOI 10.1016/j.immuni.2013.05.015

Yang F, 2014, J CEREBR BLOOD F MET, V34, P660, DOI 10.1038/jcbfm.2013.242

Yin Z, 2014, MOL CELL ENDOCRINOL, V394, P80, DOI 10.1016/j.mce.2014.06.018

Youm YH, 2013, CELL METAB, V18, P519, DOI 10.1016/j.cmet.2013.09.010

Ystgaard MB, 2015, NEONATOLOGY, V108, P211, DOI 10.1159/000437247

Yu M, 2006, SHOCK, V26, P174, DOI 10.1097/01.shk.0000225404.51320.82

Yuan BQ, 2015, J NEUROIMMUNOL, V287, P71, DOI 10.1016/j.jneuroim.2015.08.002

Zeis T, 2015, BRAIN BEHAV IMMUN, V48, P313, DOI 10.1016/j.bbi.2015.04.013

Zhang DD, 2015, J STROKE CEREBROVASC, V24, P972, DOI 10.1016/j.jstrokecerebrovasdis.2014.12.019

Zhang P, 2016, MOVEMENT DISORD, V31, P366, DOI 10.1002/mds.26488

Zhang Y, 2015, ANTIOXID REDOX SIGN, V22, P1084, DOI 10.1089/ars.2014.5978

Zheng F, 2013, HEART LUNG CIRC, V22, P746, DOI 10.1016/j.hlc.2013.01.012

Zhou RB, 2010, NAT IMMUNOL, V11, P136, DOI 10.1038/ni.1831

Zhu ZS, 2016, CELL MOL NEUROBIOL, V36, P981, DOI 10.1007/s10571-015-0288-1

Zou J, 2012, FRONT NEUROSCI-SWITZ, V6, DOI 10.3389/fnins.2012.00077

NR 221

TC 123

Z9 140

U1 2

U2 43

PU SAGE PUBLICATIONS INC

PI THOUSAND OAKS

PA 2455 TELLER RD, THOUSAND OAKS, CA 91320 USA

SN 0271-678X

EI 1559-7016

J9 J CEREBR BLOOD F MET

JI J. Cereb. Blood Flow Metab.

PD OCT

PY 2016

VL 36

IS 10

BP 1668

EP 1685

DI 10.1177/0271678X16662043

PG 18

WC Endocrinology & Metabolism; Hematology; Neurosciences

WE Science Citation Index Expanded (SCI-EXPANDED)

SC Endocrinology & Metabolism; Hematology; Neurosciences & Neurology

GA DY8AC

UT WOS:000385349400002

PM 27486046

OA Green Submitted, Green Accepted, Green Published, Bronze

DA 2024-08-12

ER

PT J

AU Alam, Q

Alam, MZ

Mushtaq, G

Damanhouri, GA

Rasool, M

Kamal, MA

Haque, A

AF Alam, Qamre

Alam, Mohammad Zubair

Mushtaq, Gohar

Damanhouri, Ghazi A.

Rasool, Mahmood

Kamal, Mohammad Amjad

Haque, Absarul

TI Inflammatory Process in Alzheimer's and Parkinson's Diseases: Central

Role of Cytokines

SO CURRENT PHARMACEUTICAL DESIGN

LA English

DT Article

DE Alzheimer's disease; Parkinson's disease; inflammation; cytokines;

interleukin-1; interleukin-6; tumor necrosis factor-alpha; transforming

growth factor-beta; microglia

ID TUMOR-NECROSIS-FACTOR; GROWTH-FACTOR-BETA; CENTRAL-NERVOUS-SYSTEM;

TRANSFORMING GROWTH-FACTOR-BETA-1 GENE; AMYLOID PRECURSOR PROTEIN;

NITRIC-OXIDE; FACTOR-ALPHA; TNF-ALPHA; neurodegenerative disease;

MESENCEPHALIC CULTURES

AB Alzheimer's disease (AD) and Parkinson's disease (PD) are the two most widespread neurological disorders (NDs) characterized by degeneration of cognitive and motor functions due to malfunction and loss of neurons in the central nervous system (CNS). Numerous evidences have established the role of neuroinflammation in the AD and PD pathology. The inflammatory components such as microglia, astrocytes, complement system and cytokines are linked to neuroinflammation in the CNS. More specifically, cytokines have been found to play a central role in the neuroinflammation of AD and PD. A number of studies have demonstrated abnormally elevated levels of inflammatory cytokines such as interleukin-1 beta (IL-1 beta) and tumor necrosis factor (TNF) in AD and PD patients. Activated microglial cells have been shown to be involved in the secretion of pro-inflammatory cytokines such as IL-1, IL-6, TNF-alpha and transforming growth factor-beta, thereby contributing towards the progress of NDs. In addition, studies on AD pathogenesis have demonstrated that microglia produce beta-amyloid protein (A beta), which by itself is pro-inflammatory and causes activation of several inflammatory components. Similarly, chronic inflammation caused by microglial cells is the fundamental process involved in the destruction of neurons associated with dopamine (DA)-production in the brain of PD patients. Hence, there is a need to explore the key inflammatory components in AD and PD pathogenesis in order to fully understand the root cause and establish a substantial link between these two disorders. Such knowledge will help in better management and treatment of AD and PD.

C1 [Alam, Qamre; Alam, Mohammad Zubair; Damanhouri, Ghazi A.; Kamal, Mohammad Amjad; Haque, Absarul] King Abdulaziz Univ, King Fahd Med Res Ctr, POB 80216, Jeddah 21589, Saudi Arabia.

[Mushtaq, Gohar] King Abdulaziz Univ, Coll Sci, Dept Biochem, Jeddah 21589, Saudi Arabia.

[Rasool, Mahmood] King Abdulaziz Univ, Ctr Excellence Genom Med Res, POB 80216, Jeddah 21589, Saudi Arabia.

C3 King Abdulaziz University; King Abdulaziz University; King Abdulaziz

University

RP Kamal, MA; Haque, A (corresponding author), King Abdulaziz Univ, King Fahd Med Res Ctr, POB 80216, Jeddah 21589, Saudi Arabia.

EM prof.makamal@lycos.com; absar99@gmail.com

RI Alam, Mohammad/AAL-1327-2021; Kamal, Mohammad Amjad/J-2918-2014; HAQUE,

ABSARUL/H-9610-2012; Alam, Qamre/H-9614-2012; Damanhouri,

Ghazi/Q-7901-2019; Mushtaq, Gohar/GLU-3263-2022; Alam, Mohammad

Zubair/H-9480-2012; ALAM, Dr QAMRE/B-6563-2019; Mushtaq,

Gohar/GPP-1327-2022; Rasool, Mahmood/H-9475-2012

OI Alam, Mohammad/0000-0003-2843-169X; Kamal, Mohammad

Amjad/0000-0003-0088-0565; Alam, Mohammad Zubair/0000-0003-2843-169X;

Mushtaq, Gohar/0000-0003-4880-4035; Rasool, Mahmood/0000-0003-2997-5891;

Haque, Absarul/0000-0002-1103-9694; Alam, Dr. Qamre/0000-0001-8980-2437

FU Deanship of Scientific Research (DSR), King Abdulaziz University

[424-141-1434]

FX The authors would like to thank Deanship of Scientific Research (DSR),

King Abdulaziz University for providing grant, bearing number:

424-141-1434 for the establishment of state of the art research

facilities at KFMRC.

CR Ahmed F, 2014, CNS NEUROL DISORD-DR, V13, P312, DOI 10.2174/18715273113126660133

Akiyama H, 2000, NEUROBIOL AGING, V21, P383, DOI 10.1016/S0197-4580(00)00124-X

Alam Q, 2014, CNS NEUROL DISORD-DR, V13, P478, DOI 10.2174/18715273113126660159

Araki E, 2001, STROKE, V32, P2370, DOI 10.1161/hs1001.096057

Awad MR, 1998, TRANSPLANTATION, V66, P1014, DOI 10.1097/00007890-199810270-00009

Banati RB, 1998, MOVEMENT DISORD, V13, P221, DOI 10.1002/mds.870130205

Barger SW, 1997, NATURE, V388, P878, DOI 10.1038/42257

Bazzoni F, 1996, NEW ENGL J MED, V334, P1717, DOI 10.1056/NEJM199606273342607

Beattie EC, 2002, SCIENCE, V295, P2282, DOI 10.1126/science.1067859

Bentham P, 2004, LANCET, V363, P2105

Black RA, 1997, NATURE, V385, P729, DOI 10.1038/385729a0

BOKA G, 1994, NEUROSCI LETT, V172, P151, DOI 10.1016/0304-3940(94)90684-X

Booij J, 2001, SYNAPSE, V39, P101, DOI 10.1002/1098-2396(200102)39:2<101::AID-SYN1>3.0.CO;2-1

Bronstein DM, 1995, BRAIN RES, V704, P112, DOI 10.1016/0006-8993(95)01189-7

Brown GC, 2003, MOL NEUROBIOL, V27, P325, DOI 10.1385/MN:27:3:325

Butterfield DA, 2001, TRENDS MOL MED, V7, P548, DOI 10.1016/S1471-4914(01)02173-6

BUXBAUM JD, 1992, P NATL ACAD SCI USA, V89, P10075, DOI 10.1073/pnas.89.21.10075

Cacquevel M, 2004, CURR DRUG TARGETS, V5, P529, DOI 10.2174/1389450043345308

Campbell IL, 1998, BRAIN RES REV, V26, P327, DOI 10.1016/S0165-0173(97)00038-6

Caraci F, 2012, CELL TISSUE RES, V347, P291, DOI 10.1007/s00441-011-1230-6

CARVEY PM, 1993, EXP NEUROL, V120, P149, DOI 10.1006/exnr.1993.1049

CHAO CC, 1994, CLIN DIAGN LAB IMMUN, V1, P109, DOI 10.1128/CDLI.1.1.109-110.1994

Chen HL, 2003, ARCH NEUROL-CHICAGO, V60, P1059, DOI 10.1001/archneur.60.8.1059

Chesneau V, 2000, BIOCHEM J, V351, P509, DOI 10.1042/0264-6021:3510509

Czlonkowska A., 2002, Medical Science Monitor, V8, P165

Dehmer T, 2000, J NEUROCHEM, V74, P2213, DOI 10.1046/j.1471-4159.2000.0742213.x

Dinarello CA, 2002, CLIN EXP RHEUMATOL, V20, pS1

Dinarello CA, 1996, BLOOD, V87, P2095, DOI 10.1182/blood.V87.6.2095.bloodjournal8762095

Dobbs RJ, 1999, ACTA NEUROL SCAND, V100, P34, DOI 10.1111/j.1600-0404.1999.tb00721.x

Domonkos A, 2001, EUR CYTOKINE NETW, V12, P411

Ferri CP, 2005, LANCET, V366, P2112, DOI 10.1016/S0140-6736(05)67889-0

FEUERSTEIN GZ, 1994, CEREBROVAS BRAIN MET, V6, P341

Finch CE, 2007, CURR ALZHEIMER RES, V4, P185, DOI 10.2174/156720507780362254

Fita IG, 2011, ROM J MORPHOL EMBRYO, V52, P975

FLANDERS KC, 1995, NEUROLOGY, V45, P1561, DOI 10.1212/WNL.45.8.1561

FORLONI G, 1992, MOL BRAIN RES, V16, P128, DOI 10.1016/0169-328X(92)90202-M

FREI K, 1989, EUR J IMMUNOL, V19, P689, DOI 10.1002/eji.1830190418

Fujiwara Nagatoshi, 2005, Current Drug Targets - Inflammation and Allergy, V4, P281, DOI 10.2174/1568010054022024

Gadient RA, 1997, PROG NEUROBIOL, V52, P379, DOI 10.1016/S0301-0082(97)00021-X

Gao HM, 2008, TRENDS IMMUNOL, V29, P357, DOI 10.1016/j.it.2008.05.002

Gao HM, 2012, TRENDS PHARMACOL SCI, V33, P295, DOI 10.1016/j.tips.2012.03.008

GOLDGABER D, 1989, P NATL ACAD SCI USA, V86, P7606, DOI 10.1073/pnas.86.19.7606

GOODWIN JL, 1995, BRAIN RES, V692, P207, DOI 10.1016/0006-8993(95)00646-8

Gordon S, 2003, NAT REV IMMUNOL, V3, P23, DOI 10.1038/nri978

Grainger DJ, 1999, HUM MOL GENET, V8, P93, DOI 10.1093/hmg/8.1.93

Griffin WST, 2002, J LEUKOCYTE BIOL, V72, P233

GRIFFIN WST, 1989, P NATL ACAD SCI USA, V86, P7611

Gruol DL, 1997, MOL NEUROBIOL, V15, P307, DOI 10.1007/BF02740665

Hamaguchi T, 2005, J NEUROL NEUROSUR PS, V76, P696, DOI 10.1136/jnnp.2003.034454

Hardy J, 2002, SCIENCE, V297, P353, DOI 10.1126/science.1072994

Heneka MT, 2007, J NEUROIMMUNOL, V184, P69, DOI 10.1016/j.jneuroim.2006.11.017

HETIER E, 1988, J NEUROSCI RES, V21, P391, DOI 10.1002/jnr.490210230

Hirsch EC, 2009, LANCET NEUROL, V8, P382, DOI 10.1016/S1474-4422(09)70062-6

Hwang DY, 2003, MOL BRAIN RES, V114, P123, DOI 10.1016/S0169-328X(03)00162-1

Idriss HT, 2000, MICROSC RES TECHNIQ, V50, P184, DOI 10.1002/1097-0029(20000801)50:3<184::AID-JEMT2>3.0.CO;2-H

Imamura K, 2003, ACTA NEUROPATHOL, V106, P518, DOI 10.1007/s00401-003-0766-2

Itzhak Y, 1999, SYNAPSE, V34, P305, DOI 10.1002/(SICI)1098-2396(19991215)34:4<305::AID-SYN6>3.0.CO;2-#

Izumi Y, 2005, J NEUROSCI RES, V82, P126, DOI 10.1002/jnr.20595

Jenner P, 2003, ANN NEUROL, V53, pS26, DOI 10.1002/ana.10483

Jenner P, 1996, NEUROLOGY, V47, pS161, DOI 10.1212/WNL.47.6_Suppl_3.161S

Kawajiri S, 2011, TRENDS PHARMACOL SCI, V32, P573, DOI 10.1016/j.tips.2011.06.001

Khan W, 2012, CNS NEUROL DISORD-DR, V11, P456, DOI 10.2174/187152712800792929

Krüger R, 2000, J NEURAL TRANSM, V107, P553, DOI 10.1007/s007020070078

Le WD, 1999, PARKINSONISM RELAT D, V5, P19

LEE SC, 1993, J IMMUNOL, V150, P2659

Li MO, 2006, ANNU REV IMMUNOL, V24, P99, DOI 10.1146/annurev.immunol.24.021605.090737

Liberatore GT, 1999, NAT MED, V5, P1403

Lindberg C, 2005, NEUROTOX RES, V8, P267, DOI 10.1007/BF03033980

Liu B, 2003, J PHARMACOL EXP THER, V304, P1, DOI 10.1124/jpet.102.035048

Locksley RM, 2001, CELL, V104, P487, DOI 10.1016/S0092-8674(01)00237-9

Long-Smith CM, 2009, PROG NEUROBIOL, V89, P277, DOI 10.1016/j.pneurobio.2009.08.001

März P, 1998, P NATL ACAD SCI USA, V95, P3251, DOI 10.1073/pnas.95.6.3251

Masliah E, 2001, NEUROCHEM INT, V39, P393, DOI 10.1016/S0197-0186(01)00046-8

MCGEER PL, 1988, NEUROLOGY, V38, P1285, DOI 10.1212/WNL.38.8.1285

MOGI M, 1994, NEUROSCI LETT, V180, P147, DOI 10.1016/0304-3940(94)90508-8

MOGI M, 1995, NEUROSCI LETT, V193, P129, DOI 10.1016/0304-3940(95)11686-Q

Murphy PG, 1997, NEUROSCIENCE, V23, P890

Nagatsu T, 2005, CURR PHARM DESIGN, V11, P999, DOI 10.2174/1381612053381620

Nawashiro H, 1997, BRAIN RES, V778, P265, DOI 10.1016/S0006-8993(97)00981-5

Nazem A, 2008, J ALZHEIMERS DIS, V13, P199

Nedjadi T, 2014, CNS NEUROL DISORD-DR, V13, P203, DOI 10.2174/18715273113126660140

Nishimura M, 2001, NEUROSCI LETT, V311, P1, DOI 10.1016/S0304-3940(01)02111-5

Nyoman G, 2007, FOLIA MED INDONES, V43, P54

OHARA J, 1987, NATURE, V325, P537, DOI 10.1038/325537a0

Olsen JH, 2005, BRIT J CANCER, V92, P201, DOI 10.1038/sj.bjc.6602279

Owens T, 2005, BRAIN RES REV, V48, P178, DOI 10.1016/j.brainresrev.2004.12.007

Pickering M, 2005, EXP PHYSIOL, V90, P663, DOI 10.1113/expphysiol.2005.030734

Puentes F, 2014, J NEUROL NEUROSUR PS, V85, P274, DOI [10.1136/jnnp-2013-307454, 10.1136/jnnp-2013-305494]

Qin L, 2002, J NEUROCHEM, V293, P617

Qiu WQ, 1998, J BIOL CHEM, V273, P32730, DOI 10.1074/jbc.273.49.32730

Rodney R, 2008, J NEUROSCI, V29, P1846

RODRIGUEZ M, 1994, J IMMUNOL, V153, P3811

Ruiz P.J., 2011, NEUROLOGIST, V17, P18

SAITO T, 1991, J IMMUNOL, V147, P168

SATOH T, 1988, MOL CELL BIOL, V8, P3546, DOI 10.1128/MCB.8.8.3546

Sawada M, 2006, J NEURAL TRANSM-SUPP, P373

Sawada M, 2000, J NEURAL TRANSM-SUPP, V70, P373

SEBIRE G, 1993, J IMMUNOL, V150, P1517

Sharma V, 2012, KLIN PSIKOFARMAKOL B, V22, P86, DOI 10.5455/bcp.20120112064639

SHENG JG, 1995, NEUROPATH APPL NEURO, V21, P290, DOI 10.1111/j.1365-2990.1995.tb01063.x

Sheng JG, 1998, NEUROPATH APPL NEURO, V24, P278

Shohami E, 1999, CYTOKINE GROWTH F R, V10, P119, DOI 10.1016/S1359-6101(99)00008-8

Sloane PD, 2002, ANNU REV PUBL HEALTH, V23, P213, DOI 10.1146/annurev.publhealth.23.100901.140525

Stacy MA, 2010, PROG NEURO-PSYCHOPH, V34, P57, DOI 10.1016/j.pnpbp.2009.09.023

Stefanacci RG, 2011, AM J MANAG CARE S13, V17, P356

Streit WJ, 1999, PROG NEUROBIOL, V57, P563, DOI 10.1016/S0301-0082(98)00069-0

Syrris P, 1998, CLIN SCI, V95, P659, DOI 10.1042/cs0950659

Tabrez S, 2012, CNS NEUROL DISORD-DR, V11, P395, DOI 10.2174/187152712800792785

Taipale J, 1998, ADV CANCER RES, V75, P87, DOI 10.1016/S0065-230X(08)60740-X

Tanner CM, 1996, NEUROL CLIN, V14, P317, DOI 10.1016/S0733-8619(05)70259-0

ten Dijke P, 2004, TRENDS BIOCHEM SCI, V29, P265, DOI 10.1016/j.tibs.2004.03.008

Vallieres L, 1997, J NEUROCHEM, V69, P1668, DOI 10.1046/j.1471-4159.1997.69041668.x

VANDERWAL EA, 1993, NEUROREPORT, V4, P69, DOI 10.1097/00001756-199301000-00018

Vitkovic L, 2000, J NEUROCHEM, V74, P457, DOI 10.1046/j.1471-4159.2000.740457.x

Wahner AD, 2007, ARCH NEUROL-CHICAGO, V64, P836, DOI 10.1001/archneur.64.6.836

Wang J, 2003, J BIOL CHEM, V278, P21751, DOI 10.1074/jbc.M212662200

Wyss-Coray Tony, 2006, Current Alzheimer Research, V3, P191, DOI 10.2174/156720506777632916

YU SJ, 1994, EXP NEUROL, V126, P15, DOI 10.1006/exnr.1994.1038

ZHANG ZX, 1993, NEUROEPIDEMIOLOGY, V12, P195, DOI 10.1159/000110318

NR 119

TC 160

Z9 188

U1 0

U2 68

PU BENTHAM SCIENCE PUBL LTD

PI SHARJAH

PA EXECUTIVE STE Y-2, PO BOX 7917, SAIF ZONE, 1200 BR SHARJAH, U ARAB

EMIRATES

SN 1381-6128

EI 1873-4286

J9 CURR PHARM DESIGN

JI Curr. Pharm. Design

PY 2016

VL 22

IS 5

BP 541

EP 548

DI 10.2174/1381612822666151125000300

PG 8

WC Pharmacology & Pharmacy

WE Science Citation Index Expanded (SCI-EXPANDED)

SC Pharmacology & Pharmacy

GA DG4VM

UT WOS:000372071200005

PM 26601965

DA 2024-08-12

ER

PT J

AU Yang, Y

Gong, ZY

Wang, ZY

Lu, Y

AF Yang, Yang

Gong, Zhongying

Wang, Zhiyun

Lu, Yi

TI Activation of Sirt1/PGC1α pathway attenuates neuroinflammation injury in

Parkinson's disease

SO JOURNAL OF RECEPTORS AND SIGNAL TRANSDUCTION

LA English

DT Article

DE Parkinson's disease; Sirt1/PGC1 alpha pathway; viability; apoptosis

ID OXIDATIVE STRESS

AB Parkinson's disease is a brain disorder that is featured by shaking palsy, which affect the motor system. The pathogenesis of Parkinson's disease has been ascribed to neurodegenerative disorder, neural oxidative stress, neuroinflammation, and neurotransmitter disorder. In the present study, we explored the influence of Sirt1/PGC1 alpha pathway in regulating BV-2 cells viability under TNF alpha treatment. Our results demonstrated that the activity of Sirt1/PGC1 alpha pathway was significantly downregulated in response to TNF alpha treatment. Reactivation of Sirt1/PGC1 alpha pathway through supplementation of SRT1720 significantly elevated the viability of BV-2 cells under an in vitro neuroinflammation model. Therefore, our results report a novel signaling pathway responsible for the survival of neuron under neuroinflammation. Re-activation of Sirt1/PGC1 alpha pathway may be a potential therapeutic approach for the treatment of Parkinson's disease through enhancing neuronal viability.

C1 [Yang, Yang; Gong, Zhongying; Wang, Zhiyun; Lu, Yi] Tianjin First Cent Hosp, 24 Fukang Rd, Tianjin 300192, China.

C3 Tianjin Medical University

RP Wang, ZY (corresponding author), Tianjin First Cent Hosp, 24 Fukang Rd, Tianjin 300192, China.

EM yangyang160329@163.com

CR Akbari P, 2019, ANGIOGENESIS, V22, P473, DOI 10.1007/s10456-019-09687-9

Battelli MG, 2019, REDOX BIOL, V21, DOI 10.1016/j.redox.2018.101070

Belarbi K, 2020, MOL NEURODEGENER, V15, DOI 10.1186/s13024-020-00408-1

Bittencourt A, 2022, NUTR NEUROSCI, V25, P1026, DOI 10.1080/1028415X.2020.1831261

CAO J, 2020, ANGIOGENESIS 0204, V23, DOI DOI 10.1007/S10456-020-09707-Z

Dong HJ, 2019, ANGIOGENESIS, V22, P251, DOI 10.1007/s10456-018-9654-1

Farber G, 2019, ANGIOGENESIS, V22, P237, DOI 10.1007/s10456-018-9653-2

Fuhrmann DC, 2019, REDOX BIOL, V20, P204, DOI 10.1016/j.redox.2018.10.007

Galjart B, 2019, ANGIOGENESIS, V22, P355, DOI 10.1007/s10456-019-09661-5

Gentile F, 2020, INT J MOL SCI, V21, DOI 10.3390/ijms21207471

Lang XSJ, 2020, BRAIN RES BULL, V165, P30, DOI 10.1016/j.brainresbull.2020.09.015

Li X, 2020, PHARM RES, V27

Li Y, 2020, OXID MED CELL LONGEV, V2020, DOI 10.1155/2020/4751349

Lin JY, 2020, AGING-US, V12, P6852, DOI 10.18632/aging.103046

Liu T, 2019, REDOX BIOL, V20, P195, DOI 10.1016/j.redox.2018.10.011

Mo J, 2019, REDOX BIOL, V20, P75, DOI 10.1016/j.redox.2018.09.022

Oliveira MD, 2019, REDOX BIOL, V20, P182, DOI 10.1016/j.redox.2018.10.006

Schmidt HM, 2019, REDOX BIOL, V21, DOI 10.1016/j.redox.2018.101072

Shi LN, 2020, FRONT PEDIATR, V8, DOI 10.3389/fped.2020.00455

Silva J, 2020, ALCOHOL

Sivalingam K, 2021, MOL NEUROBIOL, V58, P490, DOI 10.1007/s12035-020-02131-w

Subramani J, 2020, AGING-US, V12, P19809, DOI 10.18632/aging.104071

Varone E, 2019, REDOX BIOL, V24, DOI 10.1016/j.redox.2019.101176

Vellimana AK, 2020, EXP NEUROL, V334, DOI 10.1016/j.expneurol.2020.113484

Wang B, 2020, BRAIN RES, V1748, DOI 10.1016/j.brainres.2020.147108

Wang J, 2020, PHARMACOL RES, V156, DOI 10.1016/j.phrs.2020.104771

Wang J, 2020, THERANOSTICS, V10, P384, DOI 10.7150/thno.40098

Wang Z, 2020, NEUROBIOL AGING, V91, DOI 10.1016/j.neurobiolaging.2020.02.018

Wu LH, 2020, AGING-US, V12, P17990, DOI 10.18632/aging.103542

NR 29

TC 0

Z9 0

U1 0

U2 18

PU TAYLOR & FRANCIS LTD

PI ABINGDON

PA 2-4 PARK SQUARE, MILTON PARK, ABINGDON OR14 4RN, OXON, United Kingdom

SN 1079-9893

EI 1532-4281

J9 J RECEPT SIG TRANSD

JI J. Recept. Signal Transduct.

PD JAN 2

PY 2022

VL 42

IS 1

BP 67

EP 70

DI 10.1080/10799893.2020.1843494

EA NOV 2020

PG 4

WC Biochemistry & Molecular Biology; Cell Biology

WE Science Citation Index Expanded (SCI-EXPANDED)

SC Biochemistry & Molecular Biology; Cell Biology

GA YX0ML

UT WOS:000588181500001

PM 33172325

OA Bronze

DA 2024-08-12

ER

PT J

AU Belarbi, K

Cuvelier, E

Bonte, MA

Desplanque, M

Gressier, B

Devos, D

Chartier-Harlin, MC

AF Belarbi, Karim

Cuvelier, Elodie

Bonte, Marie-Amandine

Desplanque, Mazarine

Gressier, Bernard

Devos, David

Chartier-Harlin, Marie-Christine

TI Glycosphingolipids and neuroinflammation in Parkinson's disease

SO MOLECULAR NEURODEGENERATION

LA English

DT Review

DE Gangliosides; Gaucher Disease; Glucocerebrosides; Glucosylceramides;

Lipids; Microglia; neurodegenerative disease; parkinson's disease;

Sphingolipids; Synucleinopathies

ID MACROPHAGE-INFLAMMATORY PROTEIN-1-ALPHA; GROWTH-FACTOR RECEPTOR;

AMYOTROPHIC-LATERAL-SCLEROSIS; NERVOUS-SYSTEM INFLAMMATION; GENOME-WIDE

ASSOCIATION; ACID BETA-GLUCOSIDASE; GAUCHER-DISEASE; GLUCOCEREBROSIDASE

ACTIVITY; CEREBROSPINAL-FLUID; LYSOSOMAL STORAGE

AB Parkinson's disease is a progressive neurodegenerative disease characterized by the loss of dopaminergic neurons of the nigrostriatal pathway and the formation of neuronal inclusions known as Lewy bodies. Chronic neuroinflammation, another hallmark of the disease, is thought to play an important role in the neurodegenerative process. Glycosphingolipids are a well-defined subclass of lipids that regulate crucial aspects of the brain function and recently emerged as potent regulators of the inflammatory process. Deregulation in glycosphingolipid metabolism has been reported in Parkinson's disease. However, the interrelationship between glycosphingolipids and neuroinflammation in Parkinson's disease is not well known. This review provides a thorough overview of the links between glycosphingolipid metabolism and immune-mediated mechanisms involved in neuroinflammation in Parkinson's disease. After a brief presentation of the metabolism and function of glycosphingolipids in the brain, it summarizes the evidences supporting that glycosphingolipids (i.e. glucosylceramides or specific gangliosides) are deregulated in Parkinson's disease. Then, the implications of these deregulations for neuroinflammation, based on data from human inherited lysosomal glycosphingolipid storage disorders and gene-engineered animal studies are outlined. Finally, the key molecular mechanisms by which glycosphingolipids could control neuroinflammation in Parkinson's disease are highlighted. These include inflammasome activation and secretion of pro-inflammatory cytokines, altered calcium homeostasis, changes in the blood-brain barrier permeability, recruitment of peripheral immune cells or production of autoantibodies.

C1 [Belarbi, Karim; Cuvelier, Elodie; Bonte, Marie-Amandine; Desplanque, Mazarine; Gressier, Bernard; Devos, David; Chartier-Harlin, Marie-Christine] Univ Lille, Lille Neurosci & Cognit, CHU Lille, INSERM, 1 Pl Verdun, F-59006 Lille, France.

[Belarbi, Karim; Cuvelier, Elodie; Desplanque, Mazarine; Gressier, Bernard] Univ Lille, Dept Pharmacol, Fac Pharm, Lille, France.

[Devos, David] LiCEND, Dept Pharmacol Med, I SITE ULNE, Lille, France.

C3 Universite de Lille; CHU Lille; Institut National de la Sante et de la

Recherche Medicale (Inserm); Universite de Lille

RP Belarbi, K; Chartier-Harlin, MC (corresponding author), Univ Lille, Lille Neurosci & Cognit, CHU Lille, INSERM, 1 Pl Verdun, F-59006 Lille, France.; Belarbi, K (corresponding author), Univ Lille, Dept Pharmacol, Fac Pharm, Lille, France.

EM karim.belarbi@inserm.fr; marie-christine.chartier-harlin@inserm.fr

RI belarbi, karim/G-4467-2013; belarbi, karim/T-9461-2018; chartier-harlin,

marie-christine/L-4527-2018; Devos, David/E-4059-2013

OI belarbi, karim/0000-0002-0027-1172; chartier-harlin,

marie-christine/0000-0001-6416-6526; Cuvelier,

Elodie/0000-0002-0799-8526; Devos, David/0000-0002-2417-799X

FU Region Hauts-de France, University of Lille; Centre National de la

Recherche Scientifique (projet exploratoire premier soutien PEPS);

Inserm, CHU of Lille; (PHRC Convergence); Dementia in Neurological and

Mental Diseases (DN2M); Fondation Vaincre Alzheimer; Agence Regionale de

Sante Hauts-de-France; Doctoral School "Biology and Health" of Lille

[446]

FX This work was supported by Region Hauts-de France, University of Lille

and Centre National de la Recherche Scientifique (projet exploratoire

premier soutien PEPS), Inserm, CHU of Lille (the Bonus H, PHRC

Convergence), Dementia in Neurological and Mental Diseases (DN2M), the

Lille FHU-VasCog project, Fondation Vaincre Alzheimer and ANR MetDePaDi.

EC received a research fellowship from Agence Regionale de Sante

Hauts-de-France. MAB received a doctoral scholarship from the Doctoral

School "Biology and Health" of Lille (446).

CR Aflaki E, 2016, AGING CELL, V15, P77, DOI 10.1111/acel.12409

Alcalay RN, 2018, NEUROBIOL DIS, V112, P85, DOI 10.1016/j.nbd.2018.01.012

Alcalay RN, 2015, BRAIN, V138, P2648, DOI 10.1093/brain/awv179

Alcalay RN, 2014, JAMA NEUROL, V71, P752, DOI 10.1001/jamaneurol.2014.313

ARGOV Z, 1984, ANN NEUROL, V16, P14, DOI 10.1002/ana.410160105

Arotcarena ML, 2019, JCI INSIGHT, V4, DOI 10.1172/jci.insight.129719

Atashrazm F, 2018, SCI REP-UK, V8, DOI 10.1038/s41598-018-33921-x

Aureli M, 2015, BBA-MOL CELL BIOL L, V1851, P1006, DOI 10.1016/j.bbalip.2015.02.001

Bakos E, 2017, J IMMUNOL, V198, P4659, DOI 10.4049/jimmunol.1601458

Balducci C, 2007, MOVEMENT DISORD, V22, P1481, DOI 10.1002/mds.21399

Balestrino R, 2018, NEUROSCIENTIST, V24, P540, DOI 10.1177/1073858417748875

Banker BQ, 1962, CEREBRAL SPHINGOLIPI, P73

Barak V, 1999, EUR CYTOKINE NETW, V10, P205

Baris HN, 2014, PEDIATR ENDOCR REV P, V12, P72

Beavan MS, 2013, ANN MED, V45, P511, DOI 10.3109/07853890.2013.849003

Belarbi K, 2012, J NEUROINFLAMM, V9, DOI 10.1186/1742-2094-9-23

Béraud D, 2013, J NEUROIMMUNE PHARM, V8, P94, DOI 10.1007/s11481-012-9401-0

Berridge MJ, 2003, NAT REV MOL CELL BIO, V4, P517, DOI 10.1038/nrm1155

Bin Hong Y, 2006, J KOREAN MED SCI, V21, P733, DOI 10.3346/jkms.2006.21.4.733

Blauwendraat C, 2019, MOVEMENT DISORD, V34, P866, DOI 10.1002/mds.27659

Bossy-Wetzel E, 2004, NAT MED, V10, pS2, DOI 10.1038/nm1067

Bras J, 2014, HUM MOL GENET, V23, P6139, DOI 10.1093/hmg/ddu334

Brennan PJ, 2011, NAT IMMUNOL, V12, P1202, DOI 10.1038/ni.2143

Brochard V, 2009, J CLIN INVEST, V119, P182, DOI 10.1172/JCI36470

Buechner S, 2006, MOVEMENT DISORD, V21, P103, DOI 10.1002/mds.20675

Burke DG, 2013, J INHERIT METAB DIS, V36, P869, DOI 10.1007/s10545-012-9561-3

Campeau PM, 2009, BLOOD, V114, P3181, DOI 10.1182/blood-2009-02-205708

Chan RB, 2017, PLOS ONE, V12, DOI 10.1371/journal.pone.0172348

Chang D, 2017, NAT GENET, V49, P1511, DOI 10.1038/ng.3955

Chavez JA, 2014, J BIOL CHEM, V289, P723, DOI 10.1074/jbc.M113.522847

Chérin P, 2010, J INHERIT METAB DIS, V33, P331, DOI 10.1007/s10545-010-9095-5

Chester MA, 1997, PURE APPL CHEM, V69, P2475, DOI 10.1351/pac199769122475

Chiasserini D, 2015, MOL NEURODEGENER, V10, DOI 10.1186/s13024-015-0010-2

Chou A, 2018, INT J MOL SCI, V19, DOI 10.3390/ijms19061616

Codolo G, 2013, PLOS ONE, V8, DOI 10.1371/journal.pone.0055375

CONRADI NG, 1988, ACTA NEUROPATHOL, V75, P385, DOI 10.1007/BF00687792

Daneman R, 2017, NEUROBIOL DIS, V107, P1, DOI 10.1016/j.nbd.2017.05.008

Daneman R, 2015, CSH PERSPECT BIOL, V7, DOI 10.1101/cshperspect.a020412

De Francesco PN, 2013, MOL GENET METAB, V109, P93, DOI 10.1016/j.ymgme.2013.02.003

de la Mata M, 2017, ORPHANET J RARE DIS, V12, DOI 10.1186/s13023-017-0574-8

Delgado-Alvarado M, 2017, MOVEMENT DISORD, V32, P1066, DOI 10.1002/mds.27001

Djajadikerta A, 2019, J MOL BIOL

Do CB, 2011, PLOS GENET, V7, DOI 10.1371/journal.pgen.1002141

Donato R, 2001, INT J BIOCHEM CELL B, V33, P637, DOI 10.1016/S1357-2725(01)00046-2

Ekdahl CT, 2003, P NATL ACAD SCI USA, V100, P13632, DOI 10.1073/pnas.2234031100

Enquist IB, 2007, P NATL ACAD SCI USA, V104, P17483, DOI 10.1073/pnas.0708086104

Farfel-Becker T, 2011, HUM MOL GENET, V20, P1375, DOI 10.1093/hmg/ddr019

Furukawa Koichi, 2014, Adv Neurobiol, V9, P307, DOI 10.1007/978-1-4939-1154-7_14

Garcia-Ruiz C, 2015, APOPTOSIS, V20, P607, DOI 10.1007/s10495-015-1092-6

Gegg ME, 2015, MOVEMENT DISORD, V30, P1085, DOI 10.1002/mds.26278

Gegg ME, 2012, ANN NEUROL, V72, P455, DOI 10.1002/ana.23614

Gerhard A, 2006, NEUROBIOL DIS, V21, P404, DOI 10.1016/j.nbd.2005.08.002

Giacomello M, 2013, PRION, V7, P76, DOI 10.4161/pri.23581

Giacoppo S, 2017, NEUROREPORT, V28, P996, DOI 10.1097/WNR.0000000000000871

Goedert M, 2017, J PARKINSON DIS, V7, pS53, DOI 10.3233/JPD-179005

Grozdanov V, 2014, ACTA NEUROPATHOL, V128, P651, DOI 10.1007/s00401-014-1345-4

Gustot A, 2015, BIOCHEM J, V471, P323, DOI 10.1042/BJ20150617

Hallett PJ, 2018, NEUROBIOL AGING, V67, P189, DOI 10.1016/j.neurobiolaging.2018.02.028

Hamza TH, 2010, NAT GENET, V42, P781, DOI 10.1038/ng.642

Haughey NJ, 2010, NEUROMOL MED, V12, P301, DOI 10.1007/s12017-010-8135-5

Hayase T, 2010, BRAIN DEV-JPN, V32, P244, DOI 10.1016/j.braindev.2009.01.007

Hayashi Y, 2007, J BIOL CHEM, V282, P30889, DOI 10.1074/jbc.M700832200

HO MW, 1971, P NATL ACAD SCI USA, V68, P2810, DOI 10.1073/pnas.68.11.2810

Hollenbach JA, 2019, P NATL ACAD SCI USA, V116, P7419, DOI 10.1073/pnas.1821778116

Hruska KS, 2008, HUM MUTAT, V29, P567, DOI 10.1002/humu.20676

INZELBERG R, 1994, MOVEMENT DISORD, V9, P375, DOI 10.1002/mds.870090325

Jeyakumar M, 2003, BRAIN, V126, P974, DOI 10.1093/brain/awg089

Kabayama K, 2007, P NATL ACAD SCI USA, V104, P13678, DOI 10.1073/pnas.0703650104

Kain L, 2014, IMMUNITY, V41, P543, DOI 10.1016/j.immuni.2014.08.017

Kasahara K, 2002, NEUROCHEM RES, V27, P823, DOI 10.1023/A:1020265225916

Kawashima N, 2009, J BIOL CHEM, V284, P6147, DOI 10.1074/jbc.M808171200

Kawashita E, 2011, PLOS ONE, V6, DOI 10.1371/journal.pone.0016269

Kawashita E, 2009, J NEUROCHEM, V109, P1215, DOI 10.1111/j.1471-4159.2009.06041.x

KAYE EM, 1986, ANN NEUROL, V20, P223, DOI 10.1002/ana.410200208

Kittaka D, 2008, GLYCOBIOLOGY, V18, P509, DOI 10.1093/glycob/cwn032

Klenk E, 1942, H-S Z PHYSIOL CHEM, V273, P76, DOI 10.1515/bchm2.1942.273.1-2.76

Korkotian E, 1999, J BIOL CHEM, V274, P21673, DOI 10.1074/jbc.274.31.21673

Kortekaas R, 2005, ANN NEUROL, V57, P176, DOI 10.1002/ana.20369

Kyrkanides S, 2008, J NEUROIMMUNOL, V203, P50, DOI 10.1016/j.jneuroim.2008.06.024

Lee JK, 2011, KOREAN J PHYSIOL PHA, V15, P251, DOI 10.4196/kjpp.2011.15.5.251

Lehnardt S, 2003, P NATL ACAD SCI USA, V100, P8514, DOI 10.1073/pnas.1432609100

Lin G, 2019, TRENDS ENDOCRIN MET, V30, P106, DOI 10.1016/j.tem.2018.11.003

Lin G, 2018, CELL METAB, V28, P605, DOI 10.1016/j.cmet.2018.05.019

Lloyd-Evans E, 2003, J BIOL CHEM, V278, P23594, DOI 10.1074/jbc.M300212200

Löhle M, 2015, NEUROLOGY, V84, P1454, DOI 10.1212/WNL.0000000000001450

Marcos AL, 2019, BBA-BIOMEMBRANES, V1861, DOI 10.1016/j.bbamem.2019.05.015

Martinez P, 2007, J INHERIT METAB DIS, V30, P365, DOI 10.1007/s10545-007-0513-2

Massaro G, 2018, NAT MED, V24, P1317, DOI 10.1038/s41591-018-0106-7

Matern H, 1997, J BIOL CHEM, V272, P11261

Mauhin W, 2015, JIMD REP, V22, P1, DOI 10.1007/8904_2014_371

Michel PP, 2016, NEURON, V90, P675, DOI 10.1016/j.neuron.2016.03.038

Mielke MM, 2013, PLOS ONE, V8, DOI 10.1371/journal.pone.0073094

Miklossy J, 2006, EXP NEUROL, V197, P275, DOI 10.1016/j.expneurol.2005.10.034

Mizutani K, 2003, INTERNAL MED, V42, P277, DOI 10.2169/internalmedicine.42.277

Moors TE, 2019, MOL NEUROBIOL, V56, P1344, DOI 10.1007/s12035-018-1090-0

Morikawa M, 2016, J NEUROIMMUNOL, V301, P35, DOI 10.1016/j.jneuroim.2016.10.010

Mullin S, 2020, JAMA NEUROL, V77, P427, DOI 10.1001/jamaneurol.2019.4611

Murphy KE, 2014, BRAIN, V137, P834, DOI 10.1093/brain/awt367

MUTOH T, 1995, P NATL ACAD SCI USA, V92, P5087, DOI 10.1073/pnas.92.11.5087

Nakayama H, 2008, J LEUKOCYTE BIOL, V83, P728, DOI 10.1189/jlb.0707478

Nalls MA, 2014, NAT GENET, V46, P989, DOI 10.1038/ng.3043

Nordström V, 2013, PLOS BIOL, V11, DOI 10.1371/journal.pbio.1001506

NORMAN RM, 1956, J PATHOL BACTERIOL, V72, P121, DOI 10.1002/path.1700720116

NORTON WT, 1966, J NEUROCHEM, V13, P213, DOI 10.1111/j.1471-4159.1966.tb06794.x

OBRIEN JS, 1965, J LIPID RES, V6, P537

Ogawa Y, 2018, NEUROBIOL DIS, V118, P142, DOI 10.1016/j.nbd.2018.07.014

Olsen ASB, 2017, OPEN BIOL, V7, DOI 10.1098/rsob.170069

Orr CF, 2005, BRAIN, V128, P2665, DOI 10.1093/brain/awh625

Pannu R, 2004, J NEUROSCI, V24, P5942, DOI 10.1523/JNEUROSCI.1271-04.2004

Papagiannakis N, 2015, MOVEMENT DISORD, V30, P1830, DOI 10.1002/mds.26433

Parnetti L, 2017, MOVEMENT DISORD, V32, P1423, DOI 10.1002/mds.27136

Parnetti L, 2014, MOVEMENT DISORD, V29, P1019, DOI 10.1002/mds.25772

Pchelina S, 2017, NEUROSCI LETT, V636, P70, DOI 10.1016/j.neulet.2016.10.039

Pelled D, 2005, NEUROBIOL DIS, V18, P83, DOI 10.1016/j.nbd.2004.09.004

Pienaar IS, 2015, NEUROBIOL DIS, V74, P392, DOI 10.1016/j.nbd.2014.12.006

Prinetti A, 1999, J BIOL CHEM, V274, P20916, DOI 10.1074/jbc.274.30.20916

Ransohoff RM, 2010, NATURE, V468, P253, DOI 10.1038/nature09615

Reale M, 2009, BRAIN BEHAV IMMUN, V23, P55, DOI 10.1016/j.bbi.2008.07.003

Rigante D, 2017, IMMUNOL LETT, V188, P79, DOI 10.1016/j.imlet.2017.07.004

Robak LA, 2017, BRAIN, V140, P3191, DOI 10.1093/brain/awx285

Rocha EM, 2015, ANN CLIN TRANSL NEUR, V2, P433, DOI 10.1002/acn3.177

Russo D, 2018, EMBO J, V37, DOI 10.15252/embj.201797674

Sabatino JJ, 2019, NAT REV NEUROSCI, V20, P728, DOI 10.1038/s41583-019-0233-2

Sandhoff R, 2018, FEBS LETT, V592, P3835, DOI 10.1002/1873-3468.13114

SANGO K, 1995, NAT GENET, V11, P170, DOI 10.1038/ng1095-170

SCHENGRUND CL, 1990, BRAIN RES BULL, V24, P131, DOI 10.1016/0361-9230(90)90297-D

Schneider JS, 2018, PLOS ONE, V13, DOI 10.1371/journal.pone.0199189

Schultz ML, 2011, TRENDS NEUROSCI, V34, P401, DOI 10.1016/j.tins.2011.05.006

SEYFRIED TN, 2018, ASN NEURO, V10, DOI DOI 10.1177/1759091418781889

Shahmoradian SH, 2019, NAT NEUROSCI, V22, P1099, DOI 10.1038/s41593-019-0423-2

Shahrizaila N, 2014, NEUROLOGY, V83, P118, DOI 10.1212/WNL.0000000000000577

Shen RN, 2019, FRONT NEUROL, V10, DOI 10.3389/fneur.2019.00035

SHOENFELD Y, 1995, J CLIN IMMUNOL, V15, P363, DOI 10.1007/BF01541326

Shu Liming, 2012, J Glycomics Lipidomics, VSuppl 2, P1

Sidransky E, 2009, NEW ENGL J MED, V361, P1651, DOI 10.1056/NEJMoa0901281

Sidransky E, 2012, DISCOV MED, V14, P273

Stirnemann J, 2017, INT J MOL SCI, V18, DOI 10.3390/ijms18020441

Stojkovska I, 2018, CELL TISSUE RES, V373, P51, DOI 10.1007/s00441-017-2704-y

Sun Q, 2017, MOL MED, V23, P188, DOI 10.2119/molmed.2017.00077

Supnet C, 2010, CELL CALCIUM, V47, P183, DOI 10.1016/j.ceca.2009.12.014

Suzuki KGN, 2012, BIOTECHNOL J, V7, P753, DOI 10.1002/biot.201100360

Suzuki K, 2007, ACTA NEUROPATHOL, V114, P481, DOI 10.1007/s00401-007-0264-z

SVENNERHOLM L, 1994, J NEUROCHEM, V63, P1802

SVENNERHOLM L, 1973, BRAIN RES, V55, P413, DOI 10.1016/0006-8993(73)90306-5

SVENNERHOLM L, 1963, J NEUROCHEM, V10, P613, DOI 10.1111/j.1471-4159.1963.tb08933.x

SVENNERHOLM L, 1989, BIOCHIM BIOPHYS ACTA, V1005, P109, DOI 10.1016/0005-2760(89)90175-6

SVENNERHOLM L, 1956, Acta Soc Med Ups, V61, P74

Tagami S, 2002, J BIOL CHEM, V277, P3085, DOI 10.1074/jbc.M103705200

Tayebi N, 2003, MOL GENET METAB, V79, P104, DOI 10.1016/S1096-7192(03)00071-4

Tettamanti G, 2003, BIOCHIMIE, V85, P423, DOI 10.1016/S0300-9084(03)00047-6

Tsuji D, 2005, J NEUROCHEM, V92, P1497, DOI 10.1111/j.1471-4159.2005.02986.x

Utz JRJ, 2015, MOL GENET METAB, V114, P274, DOI 10.1016/j.ymgme.2014.11.015

van Dijk KD, 2013, MOVEMENT DISORD, V28, P747, DOI 10.1002/mds.25495

VANWEELY S, 1993, BIOCHIM BIOPHYS ACTA, V1181, P55, DOI 10.1016/0925-4439(93)90090-N

Vitner EB, 2012, BRAIN, V135, P1724, DOI 10.1093/brain/aws095

Voet S, 2019, EMBO MOL MED, V11, DOI 10.15252/emmm.201810248

Wada R, 2000, P NATL ACAD SCI USA, V97, P10954, DOI 10.1073/pnas.97.20.10954

WIEGANDT H, 1995, BEHAV BRAIN RES, V66, P85, DOI 10.1016/0166-4328(94)00129-4

Wise AH, 2018, MOL GENET METAB REP, V14, P27, DOI 10.1016/j.ymgmr.2017.10.013

Woeste MA, 2017, FRONT MOL NEUROSCI, V10, DOI 10.3389/fnmol.2017.00386

Wong KD, 2004, MOL GENET METAB, V82, P192, DOI 10.1016/j.ymgme.2004.04.011

Wu YP, 2004, P NATL ACAD SCI USA, V101, P8425, DOI 10.1073/pnas.0400625101

Yamaguchi A, 2004, J CLIN INVEST, V113, P200, DOI 10.1172/JCI200419639

Yamazaki T, 2008, CLIN NEUROL NEUROSUR, V110, P722, DOI 10.1016/j.clineuro.2008.03.010

Yogasundaram H, 2018, J AM HEART ASSOC, V7, DOI 10.1161/JAHA.118.009098

Yoon SJ, 2006, P NATL ACAD SCI USA, V103, P18987, DOI 10.1073/pnas.0609281103

Zahran AM, 2017, ARCH IMMUNOL THER EX, V65, P263, DOI 10.1007/s00005-016-0421-y

Zhang HJ, 2011, NAT CELL BIOL, V13, P1189, DOI 10.1038/ncb2328

Zhang K, 2013, PLOS ONE, V8, DOI 10.1371/journal.pone.0072744

Zhao F, 2016, NEUROSCIENCE, V320, P239, DOI 10.1016/j.neuroscience.2016.02.007

ZHOU QG, 1994, J BIOL CHEM, V269, P1959

Zunke F, 2016, P NATL ACAD SCI USA, V113, P3791, DOI 10.1073/pnas.1514005113

NR 172

TC 73

Z9 77

U1 0

U2 24

PU BMC

PI LONDON

PA CAMPUS, 4 CRINAN ST, LONDON N1 9XW, United Kingdom

EI 1750-1326

J9 MOL NEURODEGENER

JI Mol. Neurodegener.

PD OCT 17

PY 2020

VL 15

IS 1

AR 59

DI 10.1186/s13024-020-00408-1

PG 16

WC Neurosciences

WE Science Citation Index Expanded (SCI-EXPANDED)

SC Neurosciences & Neurology

GA OB4MW

UT WOS:000578446300001

PM 33069254

OA gold, Green Published

DA 2024-08-12

ER

PT J

AU Martínez-Coria, H

Arrieta-Cruz, I

Gutiérrez-Juárez, R

López-Valdés, HE

AF Martinez-Coria, Hilda

Arrieta-Cruz, Isabel

Gutierrez-Juarez, Roger

Lopez-Valdes, Hector Eduardo

TI Anti-Inflammatory Effects of Flavonoids in Common Neurological Disorders

Associated with Aging

SO INTERNATIONAL JOURNAL OF MOLECULAR SCIENCES

LA English

DT Review

DE flavonoids; ischemic stroke; neurodegenerative disease; Alzheimer's

disease; Parkinson's disease; neuroinflammation

ID OXYGEN-GLUCOSE DEPRIVATION; CEREBRAL-ISCHEMIA-REPERFUSION; NF-KAPPA-B;

EPIGALLOCATECHIN GALLATE EXTENDS; PLASMINOGEN ACTIVATOR TREATMENT;

PROTECTS DOPAMINERGIC-NEURONS; INDUCED COGNITIVE IMPAIRMENT;

ALPHA-SYNUCLEIN AGGREGATION; COCOA FLAVANOL CONSUMPTION; TRANSGENIC

MOUSE MODEL

AB Aging reduces homeostasis and contributes to increasing the risk of brain diseases and death. Some of the principal characteristics are chronic and low-grade inflammation, a general increase in the secretion of proinflammatory cytokines, and inflammatory markers. Aging-related diseases include focal ischemic stroke and neurodegenerative disease such as Alzheimer's disease (AD) and Parkinson's disease (PD). Flavonoids are the most common class of polyphenols and are abundantly found in plant-based foods and beverages. A small group of individual flavonoid molecules (e.g., quercetin, epigallocatechin-3-gallate, and myricetin) has been used to explore the anti-inflammatory effect in vitro studies and in animal models of focal ischemic stroke and AD and PD, and the results show that these molecules reduce the activated neuroglia and several proinflammatory cytokines, and also, inactivate inflammation and inflammasome-related transcription factors. However, the evidence from human studies has been limited. In this review article, we highlight the evidence that individual natural molecules can modulate neuroinflammation in diverse studies from in vitro to animal models to clinical studies of focal ischemic stroke and AD and PD, and we discuss future areas of research that can help researchers to develop new therapeutic agents.

C1 [Martinez-Coria, Hilda; Lopez-Valdes, Hector Eduardo] Univ Nacl Autonoma Mexico, Fac Med, Dept Physiol, Mexico City 04510, Mexico.

[Arrieta-Cruz, Isabel] Minist Hlth, Dept Basic Res, Natl Inst Geriatr, Mexico City 10200, Mexico.

[Gutierrez-Juarez, Roger] Univ Nacl Autonoma Mexico, Fac Higher Studies Zaragoza, Sch Med, Dept Biomed Sci, Mexico City 09230, Mexico.

C3 Universidad Nacional Autonoma de Mexico; Universidad Nacional Autonoma

de Mexico

RP López-Valdés, HE (corresponding author), Univ Nacl Autonoma Mexico, Fac Med, Dept Physiol, Mexico City 04510, Mexico.

EM helopezv@comunidad.unam.mx

RI Gutierrez-Juarez, Roger/IST-9343-2023

CR Abd El Mohsen MM, 2002, FREE RADICAL BIO MED, V33, P1693, DOI 10.1016/S0891-5849(02)01137-1

Adams KL, 2018, NAT NEUROSCI, V21, P9, DOI 10.1038/s41593-017-0033-9

Akhlaghi M., 2017, International journal of Nutrition sciences, V2, P180, DOI DOI 10.30906/0869-2092-2011-74-6-33-40

Allan SM, 2005, NAT REV IMMUNOL, V5, P629, DOI 10.1038/nri1664

Altharawi A, 2022, MOLECULES, V27, DOI 10.3390/molecules27217159

Ansari MA, 2009, J NUTR BIOCHEM, V20, P269, DOI 10.1016/j.jnutbio.2008.03.002

Anusha C, 2017, CHEM-BIOL INTERACT, V269, P67, DOI 10.1016/j.cbi.2017.03.016

Ay M, 2017, J NEUROCHEM, V141, P766, DOI 10.1111/jnc.14033

Bai X, 2014, NEUROCHEM RES, V39, P1405, DOI 10.1007/s11064-014-1326-y

Baluchnejadmojarad T, 2017, EUR J PHARMACOL, V801, P72, DOI 10.1016/j.ejphar.2017.03.002

Baluchnejadmojarad T, 2010, BASIC CLIN NEUROSCI, V2, P20

Banati RB, 1998, MOVEMENT DISORD, V13, P221, DOI 10.1002/mds.870130205

Bieschke J, 2010, P NATL ACAD SCI USA, V107, P7710, DOI 10.1073/pnas.0910723107

Blandini F, 2012, FEBS J, V279, P1156, DOI 10.1111/j.1742-4658.2012.08491.x

Blinkouskaya Y, 2021, MECH AGEING DEV, V200, DOI 10.1016/j.mad.2021.111575

Bloem BR, 2021, LANCET, V397, P2284, DOI 10.1016/S0140-6736(21)00218-X

BOKA G, 1994, NEUROSCI LETT, V172, P151, DOI 10.1016/0304-3940(94)90684-X

Bologna M, 2022, J NEUROL SCI, V433, DOI 10.1016/j.jns.2021.120012

Breijyeh Z, 2020, MOLECULES, V25, DOI 10.3390/molecules25245789

Brickman AM, 2014, NAT NEUROSCI, V17, P1798, DOI 10.1038/nn.3850

Brochard V, 2009, J CLIN INVEST, V119, P182, DOI 10.1172/JCI36470

Brooks AJ, 2018, Principles of Endocrinology and Hormone Action. Endocrinology, P157

Burda JE, 2014, NEURON, V81, P229, DOI 10.1016/j.neuron.2013.12.034

Cagnin A, 2001, LANCET, V358, P461, DOI 10.1016/S0140-6736(01)05625-2

Cai YY, 2020, EUR J PHARMACOL, V883, DOI 10.1016/j.ejphar.2020.173360

Caruana M, 2012, BBA-BIOMEMBRANES, V1818, P2502, DOI 10.1016/j.bbamem.2012.05.019

Chen L, 2022, CRIT REV FOOD SCI, V62, P7730, DOI 10.1080/10408398.2021.1917508

Chen XW, 2020, MOL MED REP, V22, P5135, DOI 10.3892/mmr.2020.11637

Cheng YX, 2008, NEUROSCI LETT, V441, P16, DOI 10.1016/j.neulet.2008.05.116

Chinta SJ, 2013, NEUROTOX RES, V23, P145, DOI 10.1007/s12640-012-9328-5

Cho N, 2012, FOOD CHEM TOXICOL, V50, P1940, DOI 10.1016/j.fct.2012.03.052

Choi YT, 2001, LIFE SCI, V70, P603, DOI 10.1016/S0024-3205(01)01438-2

Coe S, 2022, CLIN NUTR ESPEN, V48, P68, DOI 10.1016/j.clnesp.2022.01.023

Costa R, 2021, ANTIOXIDANTS-BASEL, V10, DOI 10.3390/antiox10091376

Cui YJ, 2010, BIOL PHARM BULL, V33, P1814, DOI 10.1248/bpb.33.1814

Currais A, 2014, AGING CELL, V13, P379, DOI 10.1111/acel.12185

Dani M, 2018, BRAIN, V141, P2740, DOI 10.1093/brain/awy188

Darbandi N., 2016, Biologija, V62, P157

Datla KP, 2001, NEUROREPORT, V12, P3871, DOI 10.1097/00001756-200112040-00053

Davies DS, 2017, BRAIN PATHOL, V27, P795, DOI 10.1111/bpa.12456

de Oliveira J, 2021, CELLS-BASEL, V10, DOI 10.3390/cells10102581

Denes A, 2007, J CEREBR BLOOD F MET, V27, P1941, DOI 10.1038/sj.jcbfm.9600495

Desideri G, 2012, HYPERTENSION, V60, P794, DOI 10.1161/HYPERTENSIONAHA.112.193060

Dias MC, 2021, MOLECULES, V26, DOI 10.3390/molecules26175377

Dickson DW, 2018, PARKINSONISM RELAT D, V46, pS30, DOI 10.1016/j.parkreldis.2017.07.033

Ding S, 2014, NEURAL REGEN RES, V9, P2048, DOI 10.4103/1673-5374.147929

Dugger BN, 2017, CSH PERSPECT BIOL, V9, DOI 10.1101/cshperspect.a028035

Edison P, 2008, NEUROBIOL DIS, V32, P412, DOI 10.1016/j.nbd.2008.08.001

Ehrnhoefer DE, 2008, NAT STRUCT MOL BIOL, V15, P558, DOI 10.1038/nsmb.1437

El Mohsen MA, 2006, BRIT J NUTR, V95, P51, DOI 10.1079/BJN20051596

El-Zayat S.R., 2019, Bull Nat Res Centre, V43, P1, DOI [10.1186/s42269-019-0227-2, DOI 10.1186/S42269-019-0227-2]

Fan Z, 2017, BRAIN, V140, P792, DOI 10.1093/brain/aww349

FERREIRA VL., 2018, Autoantibodies and cytokines, V13

Fulop T, 2023, CLIN REV ALLERG IMMU, V64, P109, DOI 10.1007/s12016-021-08899-6

Gaugler J, 2019, ALZHEIMERS DEMENT, V15, P321, DOI 10.1016/j.jalz.2019.01.010

Gelderblom M, 2015, AGEING RES REV, V24, P77, DOI 10.1016/j.arr.2015.07.004

Gelderblom M, 2012, J CEREBR BLOOD F MET, V32, P835, DOI 10.1038/jcbfm.2011.189

Gerhard A, 2004, MOVEMENT DISORD, V19, P1221, DOI 10.1002/mds.20162

Golde TE, 2022, MOL NEURODEGENER, V17, DOI 10.1186/s13024-022-00523-1

Griffin WST, 2006, J NEUROINFLAMM, V3, DOI 10.1186/1742-2094-3-5

Gronberg NV, 2013, J NEUROINFLAMM, V10, DOI 10.1186/1742-2094-10-115

Guo SR, 2022, FRONT AGING NEUROSCI, V14, DOI 10.3389/fnagi.2022.815347

Habib CN, 2022, EUR J PHARMACOL, V914, DOI 10.1016/j.ejphar.2021.174573

Haleagrahara N, 2011, NEUROSCI LETT, V500, P139, DOI 10.1016/j.neulet.2011.06.021

Hendawy OM, 2021, ALTERN THER HEALTH M, V27, P162

Henstridge CM, 2019, NAT REV NEUROSCI, V20, P94, DOI 10.1038/s41583-018-0113-1

Hirohata M, 2007, BIOCHEMISTRY-US, V46, P1888, DOI 10.1021/bi061540x

Hollman PCH, 2004, PHARM BIOL, V42, P74, DOI 10.3109/13880200490893492

Holloway PM, 2016, STROKE, V47, P561, DOI 10.1161/STROKEAHA.115.011932

Holt EM, 2009, J AM DIET ASSOC, V109, P414, DOI 10.1016/j.jada.2008.11.036

Hou JC, 2012, INT IMMUNOPHARMACOL, V14, P749, DOI 10.1016/j.intimp.2012.10.013

Hu Q, 2016, BBA-MOL BASIS DIS, V1862, P1883, DOI 10.1016/j.bbadis.2016.07.008

Hu XM, 2012, STROKE, V43, P3063, DOI 10.1161/STROKEAHA.112.659656

Huang D, 2021, PHARMACOL RES PERSPE, V9, DOI 10.1002/prp2.752

Huang DS, 2017, EVID-BASED COMPL ALT, V2017, DOI 10.1155/2017/3545169

Hung KC, 2016, J ETHNOPHARMACOL, V194, P522, DOI 10.1016/j.jep.2016.10.040

Ishola IO, 2019, BRAIN RES, V1712, P188, DOI 10.1016/j.brainres.2019.02.017

Javed H, 2015, J NEUROL SCI, V348, P51, DOI 10.1016/j.jns.2014.10.044

Jeong KH, 2015, J MED FOOD, V18, P409, DOI 10.1089/jmf.2014.3241

Jiménez-Aliaga K, 2011, LIFE SCI, V89, P939, DOI 10.1016/j.lfs.2011.09.023

Johnson ME, 2015, NEUROTOXICOLOGY, V46, P101, DOI 10.1016/j.neuro.2014.12.002

Khamchai S, 2020, SCI REP-UK, V10, DOI 10.1038/s41598-020-70214-8

Khan MM, 2012, NEUROTOX RES, V22, P1, DOI 10.1007/s12640-011-9295-2

Kim HD, 2016, J MED FOOD, V19, P374, DOI 10.1089/jmf.2015.3581

Kim HD, 2016, J NUTR BIOCHEM, V28, P140, DOI 10.1016/j.jnutbio.2015.10.013

Kirkwood TBL, 2005, CELL, V120, P437, DOI 10.1016/j.cell.2005.01.027

Klegeris A, 2008, NEUROBIOL AGING, V29, P739, DOI 10.1016/j.neurobiolaging.2006.11.013

Kumar A, 2016, BRAIN RES BULL, V122, P35, DOI 10.1016/j.brainresbull.2016.02.016

Kuriakose D, 2020, INT J MOL SCI, V21, DOI 10.3390/ijms21207609

Kwon HS, 2020, TRANSL NEURODEGENER, V9, DOI 10.1186/s40035-020-00221-2

Lambertsen KL, 2004, J NEUROPATH EXP NEUR, V63, P942, DOI 10.1093/jnen/63.9.942

Le K, 2020, INFLAMM RES, V69, P1201, DOI 10.1007/s00011-020-01402-5

Lee E, 2014, J NEUROSCI RES, V92, P130, DOI 10.1002/jnr.23307

Lee HG, 2022, NAT REV DRUG DISCOV, V21, P339, DOI 10.1038/s41573-022-00390-x

Lee JW, 2009, J NUTR, V139, P1987, DOI 10.3945/jn.109.109785

Lee J, 2022, FRONT AGING NEUROSCI, V14, DOI 10.3389/fnagi.2022.931536

Lee KM, 2016, J NEUROSCI RES, V94, P865, DOI 10.1002/jnr.23764

Leem E, 2014, J NUTR BIOCHEM, V25, P801, DOI 10.1016/j.jnutbio.2014.03.006

Levites Y, 2001, J NEUROCHEM, V78, P1073, DOI 10.1046/j.1471-4159.2001.00490.x

Li CY, 2015, BEHAV BRAIN RES, V281, P32, DOI 10.1016/j.bbr.2014.12.012

Li DY, 2021, SIGNAL TRANSDUCT TAR, V6, DOI 10.1038/s41392-021-00687-0

Li HM, 2021, NEUROCHEM RES, V46, P1844, DOI 10.1007/s11064-021-03305-1

Li HY, 2010, EUR J PHARMACOL, V649, P92, DOI 10.1016/j.ejphar.2010.09.023

Li L, 2022, BMC COMPLEMENT MED, V22, DOI 10.1186/s12906-022-03652-8

Li Q, 2021, NEUROTOXICOLOGY, V87, P62, DOI 10.1016/j.neuro.2021.08.018

Li QL, 2019, INT IMMUNOPHARMACOL, V66, P309, DOI 10.1016/j.intimp.2018.11.044

Li S, 2011, BIOL PHARM BULL, V34, P1291, DOI 10.1248/bpb.34.1291

Li TF, 2019, NEUROCHEM INT, V129, DOI 10.1016/j.neuint.2019.104496

Li WH, 2019, BRAIN RES, V1722, DOI 10.1016/j.brainres.2019.146361

Li Y, 2020, INT IMMUNOPHARMACOL, V84, DOI 10.1016/j.intimp.2020.106571

Liang J, 2014, NEUROCHEM RES, V39, P1403, DOI 10.1007/s11064-014-1358-3

Liddelow SA, 2017, NATURE, V541, P481, DOI 10.1038/nature21029

Liu C, 2021, ADV SCI, V8, DOI 10.1002/advs.202004433

Liu Y, 2022, HUM EXP TOXICOL, V41, DOI 10.1177/09603271221125928

López-Otín C, 2013, CELL, V153, P1194, DOI 10.1016/j.cell.2013.05.039

López-Valdés HE, 2016, REV INVEST CLIN, V68, P40

Luo HX, 2017, AFR J TRADIT COMPLEM, V14, P165, DOI [10.21010/ajtcam.v14i1.18, 10.21010/ajtcam.v14i2.36]

Lv CF, 2012, EVID-BASED COMPL ALT, V2012, DOI 10.1155/2012/928643

Lyden S, 2022, NEUROL CLIN, V40, P17, DOI 10.1016/j.ncl.2021.08.002

MacNee W, 2014, EUR RESPIR J, V44, P1332, DOI 10.1183/09031936.00134014

Malpetti M, 2020, BRAIN, V143, P1588, DOI 10.1093/brain/awaa088

Manach C, 2005, AM J CLIN NUTR, V81, p230S, DOI 10.1093/ajcn/81.1.230S

Martínez-Coria H, 2021, NEURAL REGEN RES, V16, P433, DOI 10.4103/1673-5374.293129

Martínez-Cué C, 2020, FRONT CELL NEUROSCI, V14, DOI 10.3389/fncel.2020.00016

Mastroiacovo D, 2015, AM J CLIN NUTR, V101, P538, DOI 10.3945/ajcn.114.092189

Menendez C, 2011, MOL NUTR FOOD RES, V55, P1780, DOI 10.1002/mnfr.201100378

Mishra A, 2021, METAB BRAIN DIS, V36, P1591, DOI 10.1007/s11011-021-00806-4

Mo ZT, 2021, PHARM BIOL, V59, P1473, DOI 10.1080/13880209.2021.1991959

Mu X, 2011, PHARMACOL BIOCHEM BE, V98, P286, DOI 10.1016/j.pbb.2011.01.011

Mullane Kevin, 2019, Curr Protoc Pharmacol, V84, pe57, DOI 10.1002/cpph.57

Nakajima A, 2015, BEHAV BRAIN RES, V289, P69, DOI 10.1016/j.bbr.2015.04.028

Nakajima A, 2013, BEHAV BRAIN RES, V250, P351, DOI 10.1016/j.bbr.2013.05.025

Neumann J, 2006, FASEB J, V20, P714, DOI 10.1096/fj.05-4882fje

Nicastro N, 2020, NEUROBIOL AGING, V94, P236, DOI 10.1016/j.neurobiolaging.2020.06.010

Onozuka H, 2008, J PHARMACOL EXP THER, V326, P739, DOI 10.1124/jpet.108.140293

Panche AN, 2016, J NUTR SCI, V5, DOI 10.1017/jns.2016.41

Park HY, 2012, INT J MOL MED, V30, P204, DOI [10.3892/ijmm.2011.979, 10.3892/ijmm.2012.979]

Pascual M, 2021, IUBMB LIFE, V73, P900, DOI 10.1002/iub.2510

Paula PC, 2019, MOLECULES, V24, DOI 10.3390/molecules24122287

Pingale T, 2020, PHARMACOL BIOCHEM BE, V199, DOI 10.1016/j.pbb.2020.173060

Polis B., 2019, ALZHEIMERS DIS, P1, DOI [DOI 10.15586/ALZHEIMERSDISEASE.2019.CH1, 10.15586/alzheimersdisease.2019.ch1]

Pons V, 2022, PHARMACOL REV, V74, P1, DOI 10.1124/pharmrev.121.000400

Qian YS, 2012, NEUROCHEM INT, V60, P759, DOI 10.1016/j.neuint.2012.03.011

Qin C, 2022, SIGNAL TRANSDUCT TAR, V7, DOI 10.1038/s41392-022-01064-1

Qin LN, 2013, NEUROSCI LETT, V534, P285, DOI 10.1016/j.neulet.2012.12.023

Ramalingayya GV, 2016, PHARMACOGN MAG, V12, pS63, DOI 10.4103/0973-1296.176104

Ramzan I, 2015, PHYTOTHERAPIES: EFFICACY, SAFETY, AND REGULATION, P1

Ran YY, 2021, BRAIN RES, V1770, DOI 10.1016/j.brainres.2021.147626

Raza SS, 2013, NEUROSCIENCE, V230, P157, DOI 10.1016/j.neuroscience.2012.10.041

Raza SS, 2011, BRAIN RES, V1420, P93, DOI 10.1016/j.brainres.2011.08.047

Rea IM, 2018, FRONT IMMUNOL, V9, DOI 10.3389/fimmu.2018.00586

Rezai-Zadeh K, 2005, J NEUROSCI, V25, P8807, DOI 10.1523/JNEUROSCI.1521-05.2005

Rezai-Zadeh K, 2008, BRAIN RES, V1214, P177, DOI 10.1016/j.brainres.2008.02.107

Rezai-Zadeh K, 2009, J CELL MOL MED, V13, P574, DOI 10.1111/j.1582-4934.2008.00344.x

Rezai-Zadeh K, 2008, J NEUROINFLAMM, V5, DOI 10.1186/1742-2094-5-41

Ritzel RM, 2015, J NEUROINFLAMM, V12, DOI 10.1186/s12974-015-0329-1

Rodrigues LP, 2021, CYTOKINE GROWTH F R, V59, P9, DOI 10.1016/j.cytogfr.2021.01.006

Saad MA, 2015, PHARMACOL REP, V67, P115, DOI 10.1016/j.pharep.2014.08.014

Sapkota A, 2017, PLOS ONE, V12, DOI 10.1371/journal.pone.0171479

Sawmiller D, 2016, J NEUROIMMUNOL, V299, P98, DOI 10.1016/j.jneuroim.2016.08.018

Scalbert A, 2000, J NUTR, V130, p2073S, DOI 10.1093/jn/130.8.2073S

Schuitemaker A, 2013, NEUROBIOL AGING, V34, P128, DOI 10.1016/j.neurobiolaging.2012.04.021

Seals DR, 2014, PHYSIOLOGY, V29, P250, DOI 10.1152/physiol.00059.2013

Shabab T, 2017, INT J NEUROSCI, V127, P624, DOI 10.1080/00207454.2016.1212854

Shi M, 2022, ENVIRON TOXICOL, V37, P1529, DOI 10.1002/tox.23504

Sikora E, 2021, FRONT AGING NEUROSCI, V13, DOI 10.3389/fnagi.2021.646924

Simon DK, 2020, CLIN GERIATR MED, V36, P1, DOI 10.1016/j.cger.2019.08.002

Sims NR, 2017, NEUROCHEM INT, V107, P88, DOI 10.1016/j.neuint.2016.12.016

Sofroniew MV, 2010, ACTA NEUROPATHOL, V119, P7, DOI 10.1007/s00401-009-0619-8

Sonawane SK, 2021, CELL COMMUN SIGNAL, V19, DOI 10.1186/s12964-021-00704-3

Sonawane SK, 2019, ARCH BIOCHEM BIOPHYS, V675, DOI 10.1016/j.abb.2019.108119

Song N, 2016, BBA-MOL BASIS DIS, V1862, P1786, DOI 10.1016/j.bbadis.2016.05.016

Steptoe A, 2007, ATHEROSCLEROSIS, V193, P277, DOI 10.1016/j.atherosclerosis.2006.08.054

Subedi L, 2017, NUTRIENTS, V9, DOI 10.3390/nu9030207

Takahashi R, 2015, J NEUROCHEM, V134, P943, DOI 10.1111/jnc.13180

Talavéra S, 2005, J AGR FOOD CHEM, V53, P3902, DOI 10.1021/jf050145v

Tamilselvam K., 2013, Int. J. Nutr. Pharmacol. Neurol. Dis, P294, DOI DOI 10.4103/2231-0738.114875

Tang Y, 2016, MOL NEUROBIOL, V53, P1181, DOI 10.1007/s12035-014-9070-5

Tasca CI, 2015, METHODS MOL BIOL, V1254, P197, DOI 10.1007/978-1-4939-2152-2_15

Teismann P, 2003, P NATL ACAD SCI USA, V100, P5473, DOI 10.1073/pnas.0837397100

Torres-Querol C, 2021, SCI REP-UK, V11, DOI 10.1038/s41598-021-95344-5

Tseng HC, 2020, NEUROTOX RES, V37, P669, DOI 10.1007/s12640-019-00143-6

Vandenbark AA, 2021, J NEUROINFLAMM, V18, DOI 10.1186/s12974-021-02355-0

Vawter MP, 1996, EXP NEUROL, V142, P313, DOI 10.1006/exnr.1996.0200

Vitek MP, 2020, ALZH DEMENT-TRCI, V6, DOI 10.1002/trc2.12114

Wang CP, 2017, MOL NEUROBIOL, V54, P2126, DOI 10.1007/s12035-016-9806-5

Wang CP, 2013, NEUROCHEM INT, V63, P741, DOI 10.1016/j.neuint.2013.09.018

Wang HM, 2016, MOL MED REP, V13, P4215, DOI 10.3892/mmr.2016.5052

Wang L, 2020, MOL ASPECTS MED, V76, DOI 10.1016/j.mam.2020.100889

Wang LM, 2019, CLIN APPL THROMB-HEM, V25, DOI 10.1177/1076029619871359

Wang R, 2022, NEUROTOX RES, V40, P384, DOI 10.1007/s12640-022-00472-z

Wang WB, 2015, J NEUROL SCI, V348, P121, DOI 10.1016/j.jns.2014.11.018

Wang XH, 2017, CLIN NEUROPHARMACOL, V40, P24, DOI 10.1097/WNF.0000000000000197

Wen KM, 2021, CURR MED CHEM, V28, P1042, DOI 10.2174/0929867327666200713184138

Widlansky ME, 2005, FREE RADICAL BIO MED, V38, P499, DOI 10.1016/j.freeradbiomed.2004.11.013

Williamson G, 2018, COMPR REV FOOD SCI F, V17, P1054, DOI 10.1111/1541-4337.12351

Wu H, 2021, ANTIOXIDANTS-BASEL, V10, DOI 10.3390/antiox10040529

Wu LH, 2016, MOL NEUROBIOL, V53, P1080, DOI 10.1007/s12035-014-9042-9

Xu Y, 2016, NEUROCHEM RES, V41, P2788, DOI 10.1007/s11064-016-1995-9

Yabuki Y, 2014, NEUROSCIENCE, V259, P126, DOI 10.1016/j.neuroscience.2013.11.051

Yao L, 2021, HUM EXP TOXICOL, V40, P1320, DOI 10.1177/0960327121993213

Yasuda N, 2014, BRAIN RES, V1559, P46, DOI 10.1016/j.brainres.2014.02.007

Ye-Hao Zhang, 2020, Zhongguo Zhong Yao Za Zhi, V45, P4686, DOI 10.19540/j.cnki.cjcmm.20200511.401

You YP, 2016, J STROKE CEREBROVASC, V25, P990, DOI 10.1016/j.jstrokecerebrovasdis.2016.01.014

Yousufuddin M, 2019, AGING-US, V11, P2542, DOI 10.18632/aging.101931

Yousufuddin M, 2017, J STROKE CEREBROVASC, V26, P1239, DOI 10.1016/j.jstrokecerebrovasdis.2017.01.015

Yu L, 2013, PLOS ONE, V8, DOI 10.1371/journal.pone.0055839

Zang X, 2022, FRONT AGING NEUROSCI, V14, DOI 10.3389/fnagi.2022.872134

Zhang FJ, 2015, NEUROCHEM RES, V40, P1691, DOI 10.1007/s11064-015-1647-5

Zhang P, 2013, BRAIN RES BULL, V97, P8, DOI 10.1016/j.brainresbull.2013.05.005

Zhang S, 2019, J PHARMACOL SCI, V140, P62, DOI 10.1016/j.jphs.2019.04.009

Zhang ZT, 2010, ACTA PHARMACOL SIN, V31, P900, DOI 10.1038/aps.2010.77

Zhao YL, 2020, INFLAMMATION, V43, P1476, DOI 10.1007/s10753-020-01225-w

Zheng WX, 2021, INFLAMMATION, V44, P2091, DOI 10.1007/s10753-021-01486-z

Zhou KC, 2019, J PHARMACOL SCI, V139, P15, DOI 10.1016/j.jphs.2018.10.012

NR 215

TC 12

Z9 12

U1 1

U2 8

PU MDPI

PI BASEL

PA ST ALBAN-ANLAGE 66, CH-4052 BASEL, SWITZERLAND

EI 1422-0067

J9 INT J MOL SCI

JI Int. J. Mol. Sci.

PD MAR

PY 2023

VL 24

IS 5

AR 4297

DI 10.3390/ijms24054297

PG 28

WC Biochemistry & Molecular Biology; Chemistry, Multidisciplinary

WE Science Citation Index Expanded (SCI-EXPANDED)

SC Biochemistry & Molecular Biology; Chemistry

GA 9T9YM

UT WOS:000947378600001

PM 36901731

OA Green Published, gold

DA 2024-08-12

ER

PT J

AU Chen, MM

Lai, XN

Wang, XF

Ying, J

Zhang, LL

Zhou, B

Liu, X

Zhang, J

Wei, G

Hua, FZ

AF Chen, Miaomiao

Lai, Xingning

Wang, Xifeng

Ying, Jun

Zhang, Lieliang

Zhou, Bin

Liu, Xing

Zhang, Jing

Wei, Gen

Hua, Fuzhou

TI Long Non-coding RNAs and Circular RNAs: Insights Into Microglia and

Astrocyte Mediated Neurological Diseases

SO FRONTIERS IN MOLECULAR NEUROSCIENCE

LA English

DT Review

DE lncRNA; circRNA; microglia; astrocyte; neurological diseases

ID TRAUMATIC BRAIN-INJURY; SPINAL-CORD-INJURY; CEREBRAL-ISCHEMIA;

INFLAMMATORY RESPONSE; REPERFUSION INJURY; ACTIVATION; STROKE;

NEUROINFLAMMATION; POLARIZATION; MODULATION

AB Microglia and astrocytes maintain tissue homeostasis in the nervous system. Both microglia and astrocytes have pro-inflammatory phenotype and anti-inflammatory phenotype. Activated microglia and activated astrocytes can contribute to several neurological diseases. Long non-coding RNAs (lncRNAs) and circular RNAs (circRNAs), two groups of non-coding RNAs (ncRNAs), can function as competing endogenous RNAs (ceRNAs) to impair the microRNA (miRNA) inhibition on targeted messenger RNAs (mRNAs). LncRNAs and circRNAs are involved in various neurological disorders. In this review, we summarized that lncRNAs and circRNAs participate in microglia dysfunction, astrocyte dysfunction, neuron damage, and inflammation. Thereby, lncRNAs and circRNAs can positively or negatively regulate neurological diseases, including spinal cord injury (SCI), traumatic brain injury (TBI), ischemia-reperfusion injury (IRI), stroke, neuropathic pain, epilepsy, Parkinson's disease (PD), multiple sclerosis (MS), and Alzheimer's disease (AD). Besides, we also found a lncRNA/circRNA-miRNA-mRNA regulatory network in microglia and astrocyte mediated neurological diseases. Through this review, we hope to cast light on the regulatory mechanisms of lncRNAs and circRNAs in microglia and astrocyte mediated neurological diseases and provide new insights for neurological disease treatment.

C1 [Chen, Miaomiao; Lai, Xingning; Ying, Jun; Zhang, Lieliang; Zhou, Bin; Liu, Xing; Zhang, Jing; Wei, Gen; Hua, Fuzhou] Nanchang Univ, Dept Anesthesiol, Affiliated Hosp 2, Nanchang, Jiangxi, China.

[Chen, Miaomiao; Ying, Jun; Zhang, Lieliang; Zhou, Bin; Liu, Xing; Zhang, Jing; Wei, Gen; Hua, Fuzhou] Key Lab Anesthesiol Jiangxi Prov, Nanchang, Jiangxi, China.

[Chen, Miaomiao] Nanchang Univ, Clin Med Coll 1, Nanchang, Jiangxi, China.

[Lai, Xingning] Sichuan Univ, West China Hosp, Dept Anesthesiol, Chengdu, China.

[Wang, Xifeng] Nanchang Univ, Dept Anesthesiol, Affiliated Hosp 1, Nanchang, Jiangxi, China.

C3 Nanchang University; Nanchang University; Sichuan University; Nanchang

University

RP Hua, FZ (corresponding author), Nanchang Univ, Dept Anesthesiol, Affiliated Hosp 2, Nanchang, Jiangxi, China.; Hua, FZ (corresponding author), Key Lab Anesthesiol Jiangxi Prov, Nanchang, Jiangxi, China.

EM huafuzhou@126.com

RI Hua, Fuzhou/ABB-5380-2021; lu, kai/KBB-4008-2024

OI Hua, Fuzhou/0000-0002-6718-6490

FU National Natural Science Foundation of China [81760261, 82060219];

Provincial Science Foundation of Jiangxi [20192BCB23024,

20202BABL206016]; Youth Team Project of the Second Affiliated Hospital

of Nanchang University [2019YNTD12003]

FX This research was supported by grants from the National Natural Science

Foundation of China (81760261 and 82060219), Provincial Science

Foundation of Jiangxi (20192BCB23024 and 20202BABL206016), and Youth

Team Project of the Second Affiliated Hospital of Nanchang University

(2019YNTD12003).

CR Abbott NJ, 2006, NAT REV NEUROSCI, V7, P41, DOI 10.1038/nrn1824

Alam A, 2020, J NEUROINFLAMM, V17, DOI 10.1186/s12974-020-02005-x

Anjum A, 2020, INT J MOL SCI, V21, DOI 10.3390/ijms21207533

Araki T, 2021, EUR J NEUROSCI, V54, P5880, DOI 10.1111/ejn.14969

Baecher-Allan C, 2018, NEURON, V97, P742, DOI 10.1016/j.neuron.2018.01.021

Bai J, 2015, INT J STROKE, V10, P143, DOI 10.1111/ijs.12434

Bohnsack JP, 2019, TRANSL PSYCHIAT, V9, DOI 10.1038/s41398-019-0367-z

Boison D, 2012, GLIA, V60, P1234, DOI 10.1002/glia.22285

Braga EA, 2020, INT J MOL SCI, V21, DOI 10.3390/ijms21228855

Cai L, 2020, J BIOL REG HOMEOS AG, V34, P815, DOI 10.23812/20-113-A-25

Cai LJ, 2020, MOL BRAIN, V13, DOI 10.1186/s13041-020-00656-8

Cao BQ, 2018, NEUROSCIENCE, V388, P118, DOI 10.1016/j.neuroscience.2018.07.019

Cao H, 2021, AGING-US, V13, P11455, DOI 10.18632/aging.202837

Chen DL, 2019, AGING-US, V11, P12002, DOI 10.18632/aging.102529

Chen JN, 2022, NEURAL REGEN RES, V17, P144, DOI 10.4103/1673-5374.314114

Chen J, 2021, MOL THER, V29, P2873, DOI 10.1016/j.ymthe.2021.04.025

Chen LL, 2015, RNA BIOL, V12, P381, DOI 10.1080/15476286.2015.1020271

Chen M, 2020, NEUROCHEM RES, V45, P1566, DOI 10.1007/s11064-020-03021-2

Chen WH, 2020, MOL THER-NUCL ACIDS, V22, P657, DOI 10.1016/j.omtn.2020.09.027

Chen YQ, 2021, INT IMMUNOPHARMACOL, V92, DOI 10.1016/j.intimp.2020.107339

Cheng DL, 2015, TUMOR BIOL, V36, P479, DOI 10.1007/s13277-015-3093-z

Cheng SQ, 2021, J MOL NEUROSCI, V71, P169, DOI 10.1007/s12031-020-01623-7

Choi J., 2021, SULFASALAZINE

Cui SY, 2021, J ORTHOP SURG RES, V16, DOI 10.1186/s13018-020-02041-6

Dai WY, 2019, INT IMMUNOPHARMACOL, V66, P215, DOI 10.1016/j.intimp.2018.11.029

Deng FM, 2020, 3 BIOTECH, V10, DOI 10.1007/s13205-020-02190-9

Deng YM, 2019, STROKE, V50, P1850, DOI 10.1161/STROKEAHA.118.023376

Devinsky O, 2018, NAT REV DIS PRIMERS, V4, DOI 10.1038/nrdp.2018.24

Duan CF, 2018, BIOCHEM PHARMACOL, V155, P110, DOI 10.1016/j.bcp.2018.06.028

Duren RCD, 2019, NEUROPATH APPL NEURO, V45, P538, DOI 10.1111/nan.12541

Dykes IM, 2017, GENOM PROTEOM BIOINF, V15, P177, DOI 10.1016/j.gpb.2016.12.005

Feng HX, 2020, AM J TRANSL RES, V12, P7968

Fisher RS, 2014, EPILEPSIA, V55, P475, DOI 10.1111/epi.12550

Fu YH, 2021, CANCER CELL INT, V21, DOI 10.1186/s12935-021-01956-2

Gage FH, 2013, NEURON, V80, P588, DOI 10.1016/j.neuron.2013.10.037

Gao S, 2021, LIFE SCI, V266, DOI 10.1016/j.lfs.2020.118863

Gao XY, 2020, J CELL MOL MED, V24, P12980, DOI 10.1111/jcmm.15894

Gao ZW, 2013, MOL NEUROBIOL, V48, P690, DOI 10.1007/s12035-013-8460-4

Gasco V, 2021, FRONT ENDOCRINOL, V12, DOI 10.3389/fendo.2021.634415

Giacobbo BL, 2019, MOL NEUROBIOL, V56, P3295, DOI 10.1007/s12035-018-1283-6

Greenhalgh AD, 2020, NAT REV NEUROSCI, V21, P139, DOI 10.1038/s41583-020-0263-9

Gu XH, 2020, IUBMB LIFE, V72, P2194, DOI 10.1002/iub.2353

Han B, 2018, AUTOPHAGY, V14, P1164, DOI 10.1080/15548627.2018.1458173

Han CL, 2020, CELL PROLIFERAT, V53, DOI 10.1111/cpr.12856

Han CL, 2018, J NEUROINFLAMM, V15, DOI 10.1186/s12974-018-1139-z

Han TS, 2020, CANCERS, V12, DOI 10.3390/cancers12092622

Hanisch UK, 2007, NAT NEUROSCI, V10, P1387, DOI 10.1038/nn1997

He B, 2021, AGING-US, V13, P8127, DOI 10.18632/aging.202618

Hu GK, 2018, MOL THER-NUCL ACIDS, V13, P450, DOI 10.1016/j.omtn.2018.09.019

Hu XM, 2015, NAT REV NEUROL, V11, P56, DOI 10.1038/nrneurol.2014.207

Huang DZ, 2022, J LEUKOCYTE BIOL, V111, P759, DOI 10.1002/JLB.1A0421-190R

Huang RR, 2020, BIOL PSYCHIAT, V88, P392, DOI 10.1016/j.biopsych.2020.02.018

Huang RR, 2017, AUTOPHAGY, V13, P1722, DOI 10.1080/15548627.2017.1356975

Huang X, 2017, PROG NEURO-PSYCHOPH, V73, P73, DOI 10.1016/j.pnpbp.2016.06.004

Huang X, 2021, NEURAL PLAST, V2021, DOI 10.1155/2021/5554824

Jamjoom AAB, 2021, BRAIN, V144, P18, DOI 10.1093/brain/awaa321

Jankovic J, 2020, J NEUROL NEUROSUR PS, V91, P795, DOI 10.1136/jnnp-2019-322338

Jensen TS, 2014, LANCET NEUROL, V13, P924, DOI 10.1016/S1474-4422(14)70102-4

Jha MK, 2019, NEUROSCIENTIST, V25, P227, DOI 10.1177/1073858418783959

Jiang ZS, 2018, INT J BIOL MACROMOL, V120, P66, DOI 10.1016/j.ijbiomac.2018.08.002

Kalia LV, 2015, LANCET, V386, P896, DOI 10.1016/S0140-6736(14)61393-3

Kalpachidou Theodora, 2020, Neuronal Signal, V4, pNS20190099, DOI 10.1042/NS20190099

Karthikeyan A, 2016, CURR MED CHEM, V23, P3528, DOI 10.2174/0929867323666160814001040

Kauppinen A, 2013, CELL SIGNAL, V25, P1939, DOI 10.1016/j.cellsig.2013.06.007

Kierdorf K, 2017, J CLIN INVEST, V127, P3201, DOI [10.1172/jci90602, 10.1172/JCI90602]

Kumar A, 2017, J NEUROINFLAMM, V14, DOI 10.1186/s12974-017-0819-4

Kwon HS, 2020, TRANSL NEURODEGENER, V9, DOI 10.1186/s40035-020-00221-2

Leng FD, 2021, NAT REV NEUROL, V17, P157, DOI 10.1038/s41582-020-00435-y

Lévesque M, 2013, NEUROSCI BIOBEHAV R, V37, P2887, DOI 10.1016/j.neubiorev.2013.10.011

Li LB, 2021, BIOFACTORS, V47, P819, DOI 10.1002/biof.1765

Li PZ, 2020, CYTOKINE, V131, DOI 10.1016/j.cyto.2020.155106

Li TH, 2020, J APPL PHYSIOL, V129, P1460, DOI 10.1152/japplphysiol.00433.2020

Li XT, 2020, BRAIN RES, V1747, DOI 10.1016/j.brainres.2020.147039

Liao K, 2020, J NEUROIMMUNE PHARM, V15, P390, DOI 10.1007/s11481-019-09864-z

Liddelow SA, 2017, IMMUNITY, V46, P957, DOI 10.1016/j.immuni.2017.06.006

Liu DL, 2021, MOL NEUROBIOL, DOI 10.1007/s12035-021-02405-x

Liu LR, 2020, FRONT IMMUNOL, V11, DOI 10.3389/fimmu.2020.01024

Liu N, 2021, BRAIN RES BULL, V171, P91, DOI 10.1016/j.brainresbull.2021.03.004

Liu XM, 2021, J NEUROINFLAMM, V18, DOI 10.1186/s12974-021-02156-5

Liu ZF, 2014, PLOS ONE, V9, DOI 10.1371/journal.pone.0093388

Löscher W, 1999, PROG NEUROBIOL, V58, P31, DOI 10.1016/S0301-0082(98)00075-6

Ma M, 2020, CELL RES, V30, P105, DOI 10.1038/s41422-020-0273-1

Malhi GS, 2018, LANCET, V392, P2299, DOI 10.1016/S0140-6736(18)31948-2

Meng J, 2021, INT IMMUNOPHARMACOL, V90, DOI 10.1016/j.intimp.2020.107141

Meng SJ, 2017, MOL CANCER, V16, DOI 10.1186/s12943-017-0663-2

Moreno-García L, 2020, INT J MOL SCI, V21, DOI 10.3390/ijms21249582

Nelson VL, 2018, GENE DEV, V32, P1035, DOI 10.1101/gad.312355.118

Ni XR, 2020, SCI REP-UK, V10, DOI 10.1038/s41598-020-71411-1

Nutma E, 2020, CELLS-BASEL, V9, DOI 10.3390/cells9030600

Pan J, 2007, NEURORADIOLOGY, V49, P93, DOI 10.1007/s00234-006-0183-z

Payne JL, 2019, FRONT NEUROENDOCRIN, V52, P165, DOI 10.1016/j.yfrne.2018.12.001

Pekny M, 2005, GLIA, V50, P427, DOI 10.1002/glia.20207

Peterson AR, 2020, FRONT NEUROL, V11, DOI 10.3389/fneur.2020.01006

Pietrowski MJ, 2021, FRONT NEUROL, V12, DOI 10.3389/fneur.2021.654850

Qi X, 2017, NEUROSCIENCE, V348, P98, DOI 10.1016/j.neuroscience.2017.02.002

Qian XY, 2019, TRENDS BIOCHEM SCI, V44, P33, DOI 10.1016/j.tibs.2018.09.012

Ren ZH, 2020, STEM CELLS DEV, V29, P1467, DOI 10.1089/scd.2020.0133

Rösing N, 2020, FRONT CELL NEUROSCI, V14, DOI 10.3389/fncel.2020.573950

Salim U, 2022, WIRES RNA, V13, DOI 10.1002/wrna.1680

Salter MW, 2017, NAT MED, V23, P1018, DOI 10.1038/nm.4397

Santello M, 2019, NAT NEUROSCI, V22, P154, DOI 10.1038/s41593-018-0325-8

Scheffer IE, 2017, EPILEPSIA, V58, P512, DOI 10.1111/epi.13709

Scheltens P, 2016, LANCET, V388, P505, DOI 10.1016/S0140-6736(15)01124-1

Shao L, 2021, FASEB J, V35, DOI 10.1096/fj.202001737RR

Shao MH, 2020, INFLAMMATION, V43, P1536, DOI 10.1007/s10753-020-01230-z

Shi ZJ, 2018, J CELL MOL MED, V22, P2055, DOI 10.1111/jcmm.13515

Sommer CJ, 2017, ACTA NEUROPATHOL, V133, P245, DOI 10.1007/s00401-017-1667-0

Sun DY, 2017, EMBO REP, V18, P1801, DOI 10.15252/embr.201643668

Na HT, 2021, INT J NEUROSCI, V132, P77, DOI 10.1080/00207454.2020.1835901

Teng F, 2021, ADV SCI, V8, DOI 10.1002/advs.202003505

Tian JN, 2021, J BIOCHEM MOL TOXIC, V35, DOI 10.1002/jbt.22724

Tripathi S, 2021, MOL NEUROBIOL, V58, P2916, DOI 10.1007/s12035-020-02268-8

Vainchtein ID, 2020, TRENDS NEUROSCI, V43, P144, DOI 10.1016/j.tins.2020.01.003

Wan Y, 2020, CELL CYCLE, V19, P419, DOI 10.1080/15384101.2020.1711578

Wang GT, 2020, EBIOMEDICINE, V52, DOI 10.1016/j.ebiom.2020.102660

Wang HW, 2020, J BIOMED SCI, V27, DOI 10.1186/s12929-020-00635-0

Wang HX, 2020, J CELL BIOCHEM, V121, P4261, DOI 10.1002/jcb.29634

Wang H, 2020, FRONT NEUROSCI-SWITZ, V14, DOI 10.3389/fnins.2020.590570

Wang J, 2017, STROKE, V48, P2211, DOI 10.1161/STROKEAHA.117.017387

Wang LQ, 2018, SCI REP-UK, V8, DOI 10.1038/s41598-018-26421-5

Wolf SA, 2017, ANNU REV PHYSIOL, V79, P619, DOI 10.1146/annurev-physiol-022516-034406

Wu PF, 2010, ACTA PHARMACOL SIN, V31, P1523, DOI 10.1038/aps.2010.186

Xiang WN, 2021, NEUROCHEM INT, V143, DOI 10.1016/j.neuint.2020.104929

Xiao SN, 2021, J CELL MOL MED, V25, P1371, DOI 10.1111/jcmm.16220

Xu KY, 2020, NAT COMMUN, V11, DOI 10.1038/s41467-020-17435-7

Xu QX, 2021, J NEUROINFLAMM, V18, DOI 10.1186/s12974-021-02137-8

Xu S, 2020, CELL DEATH DIS, V11, DOI 10.1038/s41419-020-02824-z

Xu W, 2020, INT IMMUNOPHARMACOL, V85, DOI 10.1016/j.intimp.2020.106614

Yang B, 2021, STEM CELL RES THER, V12, DOI 10.1186/s13287-021-02187-y

Yang J, 2018, ADV EXP MED BIOL, V1087, P309, DOI 10.1007/978-981-13-1426-1_25

Ye YY, 2018, CELL DEATH DIS, V9, DOI 10.1038/s41419-018-0821-5

Yi JP, 2019, J CELL BIOCHEM, V120, P18053, DOI 10.1002/jcb.29108

Yu Q, 2020, NEUROCHEM RES, V45, P783, DOI 10.1007/s11064-019-02952-9

Zhang BP, 2020, J TRANSL MED, V18, DOI 10.1186/s12967-020-02278-z

Zhang H, 2019, SCI REP-UK, V9, DOI 10.1038/s41598-019-48759-0

Zhang M, 2021, EXP THER MED, V22, DOI 10.3892/etm.2021.10356

Zhang N, 2020, LIFE SCI, V252, DOI 10.1016/j.lfs.2020.117637

Zhang S, 2020, DRUG DES DEV THER, V14, P3683, DOI 10.2147/DDDT.S245445

Zhang X, 2019, J NEUROINFLAMM, V16, DOI 10.1186/s12974-019-1464-x

Zhang YM, 2019, J CELL BIOCHEM, V120, P17584, DOI 10.1002/jcb.29025

Zhang Y, 2019, MICROBIOME, V7, DOI 10.1186/s40168-019-0733-3

Zhang Y, 2020, MOL PSYCHIATR, V25, P1175, DOI 10.1038/s41380-018-0285-0

Zhang ZY, 2021, EXP CELL RES, V399, DOI 10.1016/j.yexcr.2020.112435

Zhao Q, 2020, NEUROSCI LETT, V715, DOI 10.1016/j.neulet.2019.134649

Zhao T, 2019, J CELL PHYSIOL, V234, P16054, DOI 10.1002/jcp.28264

Zhao Y, 2020, FRONT NEUROSCI-SWITZ, V14, DOI 10.3389/fnins.2020.00330

Zheng WX, 2021, INFLAMMATION, V44, P2091, DOI 10.1007/s10753-021-01486-z

Zhong JJ, 2017, BRAIN BEHAV IMMUN, V65, P183, DOI 10.1016/j.bbi.2017.05.001

Zhong JJ, 2017, NEUROSCIENCE, V343, P434, DOI 10.1016/j.neuroscience.2016.05.033

Zhou HJ, 2018, AM J PHYSIOL-CELL PH, V315, pC52, DOI 10.1152/ajpcell.00278.2017

Zhu HT, 2020, J CHEM NEUROANAT, V105, DOI 10.1016/j.jchemneu.2020.101749

Zhu JL, 2020, BIOSCIENCE REP, V40, DOI 10.1042/BSR20200672

Zhuang XY, 2011, MOL THER, V19, P1769, DOI 10.1038/mt.2011.164

NR 153

TC 56

Z9 57

U1 1

U2 51

PU FRONTIERS MEDIA SA

PI LAUSANNE

PA AVENUE DU TRIBUNAL FEDERAL 34, LAUSANNE, CH-1015, SWITZERLAND

SN 1662-5099

J9 FRONT MOL NEUROSCI

JI Front. Molec. Neurosci.

PD OCT 5

PY 2021

VL 14

AR 745066

DI 10.3389/fnmol.2021.745066

PG 18

WC Neurosciences

WE Science Citation Index Expanded (SCI-EXPANDED)

SC Neurosciences & Neurology

GA WI5UZ

UT WOS:000708426400001

PM 34675776

OA gold, Green Published

DA 2024-08-12

ER

PT J

AU Pellegrini, C

Antonioli, L

Calderone, V

Colucci, R

Fornai, M

Blandizzi, C

AF Pellegrini, Carolina

Antonioli, Luca

Calderone, Vincenzo

Colucci, Rocchina

Fornai, Matteo

Blandizzi, Corrado

TI Microbiota-gut-brain axis in health and disease: Is NLRP3 inflammasome

at the crossroads of microbiota-gut-brain communications?

SO PROGRESS IN NEUROBIOLOGY

LA English

DT Review

DE Gut microbiota; NLRP3 inflammasome; Enteric inflammation; Gut-brain

axis; Neurological disorders; Psychiatric diseases; Animal models

ID CHAIN FATTY-ACIDS; parkinson's disease; SODIUM-BUTYRATE;

alzheimer's disease; MULTIPLE-SCLEROSIS; IMMUNE-SYSTEM; ACTIVATION;

CASPASE-8; MICE; RECEPTORS

AB Growing evidence highlights the relevance of microbiota-gut-brain axis in the maintenance of brain homeostasis as well as in the pathophysiology of major neurological and psychiatric disorders, including Parkinson's disease (PD), Alzheimer's disease (AD), multiple sclerosis (MS), autism spectrum disorder (ASD) and major depressive disorder (MDD). In particular, changes in gut microbiota can promote enteric and peripheral neurogenic/inflammatory responses, which, in turn, could contribute to neuroinflammation and neurodegeneration in the central nervous system (CNS). Of note, the nucleotide-binding oligomerization domain leucine rich repeat and pyrin domain-containing protein 3 (NLRP3) inflammasome acts as a key player in both coordinating the host physiology and shaping the peripheral and central immune/inflammatory responses in CNS diseases. In this context, there is pioneering evidence supporting the existence of a microbiota-gut-inflammasome-brain axis, in which enteric bacteria modulate, via NLRP3 signaling, inflammatory pathways that, in turn, contribute to influence brain homeostasis.

The present review provides an overview of current knowledge on the role of microbiota-gut-inflammasome-brain axis in the major CNS diseases, including PD, AD, MS, ASD and MDD. In particular, though no direct and causal correlation among altered gut microbiota, NLRP3 activation and brain pathology has been demonstrated and in-depth studies are needed in this setting, our purpose was to pave the way to a novel and pioneering perspective on the pathophysiology of CNS disorders. Our intent was also to highlight and discuss whether alterations of microbiota-gut-inflammasome-brain axis support a holistic view of the pathophysiology of CNS diseases, even though each disorder displays a different clinical picture.

C1 [Pellegrini, Carolina; Calderone, Vincenzo] Univ Pisa, Dept Pharm, Pisa, Italy.

[Antonioli, Luca; Fornai, Matteo; Blandizzi, Corrado] Univ Pisa, Dept Clin & Expt Med, Pisa, Italy.

[Colucci, Rocchina] Univ Padua, Dept Pharmaceut & Pharmacol Sci, Padua, Italy.

C3 University of Pisa; University of Pisa; University of Padua

RP Pellegrini, C (corresponding author), Via Bonanno Pisano 6, I-56126 Pisa, Italy.

EM carolina.pellegrini87@gmail.com

RI Pellegrini, Carolina/ABE-3462-2021; Calderone, Vincenzo/L-9288-2015;

antonioli, luca/K-1695-2018

OI Pellegrini, Carolina/0000-0003-2644-5430; Calderone,

Vincenzo/0000-0002-1441-5421; antonioli, luca/0000-0003-1241-617X;

COLUCCI, ROCCHINA LUCIA/0000-0002-2593-5457; FORNAI,

MATTEO/0000-0002-0812-3959; BLANDIZZI, CORRADO/0000-0001-9927-5386

CR Aachoui Y, 2013, CURR OPIN MICROBIOL, V16, P319, DOI 10.1016/j.mib.2013.04.004

Adams JB, 2011, BMC GASTROENTEROL, V11, DOI 10.1186/1471-230X-11-22

Alcocer-Gómez E, 2014, BRAIN BEHAV IMMUN, V36, P111, DOI 10.1016/j.bbi.2013.10.017

Allen AP, 2016, TRANSL PSYCHIAT, V6, DOI 10.1038/tp.2016.191

Antonopoulos C, 2015, J BIOL CHEM, V290, P20167, DOI 10.1074/jbc.M115.652321

Atarashi K, 2011, SCIENCE, V331, P337, DOI 10.1126/science.1198469

Ayres JS, 2013, CELL HOST MICROBE, V14, P491, DOI 10.1016/j.chom.2013.10.013

Bastianetto S, 2008, MINI-REV MED CHEM, V8, P429, DOI 10.2174/138955708784223512

Bauernfeind FG, 2009, J IMMUNOL, V183, P787, DOI 10.4049/jimmunol.0901363

Bedarf JR, 2017, GENOME MED, V9, DOI 10.1186/s13073-017-0428-y

Beilharz JE, 2018, MOL PSYCHIATR, V23, P351, DOI 10.1038/mp.2017.38

Beilharz JE, 2016, BRAIN BEHAV IMMUN, V57, P304, DOI 10.1016/j.bbi.2016.07.151

Belizário JE, 2018, MEDIAT INFLAMM, V2018, DOI 10.1155/2018/2037838

Bellezza I, 2018, MOL NEUROBIOL, V55, P2350, DOI 10.1007/s12035-017-0502-x

Bischoff SC, 2014, BMC GASTROENTEROL, V14, DOI 10.1186/s12876-014-0189-7

Brinkman BM, 2011, CELL DEATH DIS, V2, DOI 10.1038/cddis.2011.101

Camell C, 2015, SEMIN IMMUNOL, V27, P334, DOI 10.1016/j.smim.2015.10.004

Canfora EE, 2017, GASTROENTEROLOGY, V153, P87, DOI 10.1053/j.gastro.2017.03.051

Cao YH, 2015, SCI TRANSL MED, V7, DOI 10.1126/scitranslmed.neurodegenerative disease8038

Cattaneo A, 2017, NEUROBIOL AGING, V49, P60, DOI 10.1016/j.neurobiolaging.2016.08.019

Chen GY, 2017, FREE RADICAL BIO MED, V105, P35, DOI 10.1016/j.freeradbiomed.2016.11.011

Chen MK, 2015, J IMMUNOL, V195, P4962, DOI 10.4049/jimmunol.1500865

Cheung SG, 2019, FRONT PSYCHIATRY, V10, DOI 10.3389/fpsyt.2019.00034

Chiang HL, 2019, J MOV DISORD, V12, P67, DOI 10.14802/jmd.18067

Chung H, 2016, CELL DEATH DIFFER, V23, P1331, DOI 10.1038/cdd.2016.14

Chung HC, 2010, CURR OPIN IMMUNOL, V22, P455, DOI 10.1016/j.coi.2010.06.008

Claudia C, 2019, DIGEST LIVER DIS, V51, P1366, DOI 10.1016/j.dld.2019.06.006

Cryan JF, 2012, NAT REV NEUROSCI, V13, P701, DOI 10.1038/nrn3346

Daniel H, 2014, ISME J, V8, P295, DOI 10.1038/ismej.2013.155

David LA, 2014, NATURE, V505, P559, DOI 10.1038/nature12820

De Felice A, 2015, TOXICS, V3, P89, DOI 10.3390/toxics3010089

Devos D, 2013, NEUROBIOL DIS, V50, P42, DOI 10.1016/j.nbd.2012.09.007

Duncan JA, 2007, P NATL ACAD SCI USA, V104, P8041, DOI 10.1073/pnas.0611496104

Dzamko N, 2015, NEUROSCIENCE, V302, P89, DOI 10.1016/j.neuroscience.2014.10.028

Erny D, 2015, NAT NEUROSCI, V18, P965, DOI 10.1038/nn.4030

Fang X, 2016, FRONT MICROBIOL, V7, DOI 10.3389/fmicb.2016.01479

Feng YY, 2019, PLOS ONE, V14, DOI 10.1371/journal.pone.0215088

Foster JA, 2017, NEUROBIOL STRESS, V7, P124, DOI 10.1016/j.ynstr.2017.03.001

Fung TC, 2017, NAT NEUROSCI, V20, P145, DOI 10.1038/nn.4476

Gagliani N, 2014, INT IMMUNOL, V26, P495, DOI 10.1093/intimm/dxu066

Geuking MB, 2011, IMMUNITY, V34, P794, DOI 10.1016/j.immuni.2011.03.021

Gordon R, 2018, SCI TRANSL MED, V10, DOI 10.1126/scitranslmed.aah4066

Gringhuis SI, 2012, NAT IMMUNOL, V13, P246, DOI 10.1038/ni.2222

Grosicki GJ, 2018, CALCIFIED TISSUE INT, V102, P433, DOI 10.1007/s00223-017-0345-5

Guo Y, 2019, FRONT BEHAV NEUROSCI, V13, DOI 10.3389/fnbeh.2019.00126

Guo Y, 2018, FRONT PHARMACOL, V9, DOI 10.3389/fphar.2018.01126

Gurung P, 2014, J IMMUNOL, V192, P1835, DOI 10.4049/jimmunol.1302839

Henderson VW, 2012, NEUROLOGY, V78, P1841, DOI 10.1212/WNL.0b013e318258f822

Heneka MT, 2018, NAT REV NEUROSCI, V19, P610, DOI 10.1038/s41583-018-0055-7

Heneka MT, 2013, NATURE, V493, P674, DOI 10.1038/nature11729

Hirota SA, 2011, INFLAMM BOWEL DIS, V17, P1359, DOI 10.1002/ibd.21478

Ho L, 2018, EXPERT REV NEUROTHER, V18, P83, DOI 10.1080/14737175.2018.1400909

Huang RR, 2017, AUTOPHAGY, V13, P1722, DOI 10.1080/15548627.2017.1356975

Inserra A, 2018, BIOESSAYS, V40, DOI 10.1002/bies.201800027

Iqbal K, 2010, ALZHEIMERS DEMENT, V6, P420, DOI 10.1016/j.jalz.2010.04.006

Jiang HY, 2015, BRAIN BEHAV IMMUN, V48, P186, DOI 10.1016/j.bbi.2015.03.016

Joseph J, 2017, FRONT NEUROSCI-SWITZ, V11, DOI 10.3389/fnins.2017.00155

Kaufmann FN, 2017, BRAIN BEHAV IMMUN, V64, P367, DOI 10.1016/j.bbi.2017.03.002

Keane RW, 2018, FRONT NEUROL, V9, DOI 10.3389/fneur.2018.00135

Kelly JR, 2016, J PSYCHIATR RES, V82, P109, DOI 10.1016/j.jpsychires.2016.07.019

Keshavarzian A, 2015, MOVEMENT DISORD, V30, P1351, DOI 10.1002/mds.26307

Kim HK, 2016, J PSYCHIATR RES, V72, P43, DOI 10.1016/j.jpsychires.2015.10.015

Koh A, 2016, CELL, V165, P1332, DOI 10.1016/j.cell.2016.05.041

Latz E, 2013, NAT REV IMMUNOL, V13, P397, DOI 10.1038/nri3452

Li HX, 2016, BRAIN RES, V1642, P70, DOI 10.1016/j.brainres.2016.03.031

Lin CH, 2019, J NEUROINFLAMM, V16, DOI 10.1186/s12974-019-1528-y

Liu FT, 2019, TRANSL PSYCHIAT, V9, DOI 10.1038/s41398-019-0389-6

Liu JM, 2017, J NEUROL SCI, V381, P176, DOI 10.1016/j.jns.2017.08.3235

Lotrich FE, 2014, INT J GERIATR PSYCH, V29, P635, DOI 10.1002/gps.4048

Lowe PP, 2018, J NEUROINFLAMM, V15, DOI 10.1186/s12974-018-1328-9

Lu B, 2013, PROTEIN CELL, V4, P163, DOI 10.1007/s13238-012-2118-2

Luczynski P, 2016, EUR J NEUROSCI, V44, P2654, DOI 10.1111/ejn.13291

Luczynski P, 2016, INT J NEUROPSYCHOPH, V19, DOI 10.1093/ijnp/pyw020

Ma QQ, 2019, J NEUROINFLAMM, V16, DOI 10.1186/s12974-019-1434-3

MacFabe Derrick F., 2015, Microbial Ecology in Health and Disease, V26, P28177, DOI 10.3402/mehd.v26.28177

MacFabe Derrick F., 2012, Microbial Ecology in Health and Disease, V23, P19260, DOI 10.3402/mehd.v23i0.19260

Macia L, 2015, NAT COMMUN, V6, DOI 10.1038/ncomms7734

Mangan MSJ, 2018, NAT REV DRUG DISCOV, V17, P588, DOI [10.1038/nrd.2018.97, 10.1038/nrd.2018.149]

Meguid NA, 2008, CLIN BIOCHEM, V41, P1044, DOI 10.1016/j.clinbiochem.2008.05.013

Rodríguez JM, 2015, MICROB ECOL HEALTH D, V26, DOI 10.3402/mehd.v26.26050

Ming X, 2002, J NEUROL SCI, V197, P9, DOI 10.1016/S0022-510X(02)00030-8

Mowry EM, 2018, NEUROL CLIN, V36, P185, DOI 10.1016/j.ncl.2017.08.008

Obata Y, 2016, GASTROENTEROLOGY, V151, P836, DOI 10.1053/j.gastro.2016.07.044

Peirce JM, 2019, J NEUROSCI RES, V97, P1223, DOI 10.1002/jnr.24476

PELLEGRINI C, 2019, INT J MOL SCI, V20

Pellegrini C, 2018, ACTA NEUROPATHOL, V136, P345, DOI 10.1007/s00401-018-1856-5

Pellegrini C, 2017, FRONT IMMUNOL, V8, DOI 10.3389/fimmu.2017.00036

Perez-Pardo P, 2019, GUT, V68, P829, DOI 10.1136/gutjnl-2018-316844

Piccarducci R, 2019, OXID MED CELL LONGEV, V2019, DOI 10.1155/2019/5030475

Pinto A, 2018, ANTIOXIDANTS-BASEL, V7, DOI 10.3390/antiox7050063

Pistollato F, 2016, NUTR REV, V74, P624, DOI 10.1093/nutrit/nuw023

Qin XY, 2016, JAMA NEUROL, V73, P1316, DOI 10.1001/jamaneurol.2016.2742

Rakoff-Nahoum S, 2004, CELL, V118, P229, DOI 10.1016/j.cell.2004.07.002

Richardson AJ, 2006, INT REV PSYCHIATR, V18, P155, DOI 10.1080/09540260600583031

Rigacci S, 2015, EXPERT REV NEUROTHER, V15, P41, DOI 10.1586/14737175.2015.986101

Rogers GB, 2016, MOL PSYCHIATR, V21, P738, DOI 10.1038/mp.2016.50

Rooks MG, 2016, NAT REV IMMUNOL, V16, P341, DOI 10.1038/nri.2016.42

Rothhammer V, 2016, NAT MED, V22, P586, DOI 10.1038/nm.4106

Sampson TR, 2016, CELL, V167, P1469, DOI 10.1016/j.cell.2016.11.018

Sandhu KV, 2017, TRANSL RES, V179, P223, DOI 10.1016/j.trsl.2016.10.002

Saresella M, 2016, MOL NEURODEGENER, V11, DOI 10.1186/s13024-016-0088-1

Savignac HM, 2015, BEHAV BRAIN RES, V287, P59, DOI 10.1016/j.bbr.2015.02.044

Savignac HM, 2016, BRAIN BEHAV IMMUN, V52, P120, DOI 10.1016/j.bbi.2015.10.007

Scheperjans F, 2015, MOVEMENT DISORD, V30, P350, DOI 10.1002/mds.26069

Seregin SS, 2017, CELL REP, V19, P733, DOI 10.1016/j.celrep.2017.03.080

Sharma Renu, 2010, J Biomed Biotechnol, V2010, P305879, DOI 10.1155/2010/305879

Sharma S, 2015, BEHAV BRAIN RES, V291, P306, DOI 10.1016/j.bbr.2015.05.052

Shen HP, 2020, PROG NEURO-PSYCHOPH, V100, DOI 10.1016/j.pnpbp.2020.109884

Shi N, 2017, MILITARY MED RES, V4, DOI 10.1186/s40779-017-0122-9

Singh RK, 2017, J TRANSL MED, V15, DOI 10.1186/s12967-017-1175-y

Skonieczna-Zydecka K, 2018, NUTRIENTS, V10, DOI 10.3390/nu10121939

Sommer F, 2013, NAT REV MICROBIOL, V11, P227, DOI 10.1038/nrmicro2974

Stefani M, 2014, BIOFACTORS, V40, P482, DOI 10.1002/biof.1171

Sun J, 2016, NEUROSCI LETT, V618, P159, DOI 10.1016/j.neulet.2016.03.003

Sun JC, 2014, PLOS ONE, V9, DOI 10.1371/journal.pone.0099299

Sun MF, 2018, BRAIN BEHAV IMMUN, V70, P48, DOI 10.1016/j.bbi.2018.02.005

Tang C, 2015, CELL HOST MICROBE, V18, P183, DOI 10.1016/j.chom.2015.07.003

Tengeler AC, 2018, NUTR REV, V76, P603, DOI 10.1093/nutrit/nuy016

Tha KK, 2000, BRAIN RES, V885, P25, DOI 10.1016/S0006-8993(00)02883-3

Thongaram T, 2017, J AGR FOOD CHEM, V65, P4184, DOI 10.1021/acs.jafc.7b00851

Unger MM, 2016, PARKINSONISM RELAT D, V32, P66, DOI 10.1016/j.parkreldis.2016.08.019

van de Wouw M, 2018, J PHYSIOL-LONDON, V596, P4923, DOI 10.1113/JP276431

Venegas C, 2017, NATURE, V552, P355, DOI 10.1038/nature25158

Vesikansa A, 2018, J CENT NERV SYST DIS, V10, DOI 10.1177/1179573518787469

Vogt NM, 2017, SCI REP-UK, V7, DOI 10.1038/s41598-017-13601-y

Vuong HE, 2017, BIOL PSYCHIAT, V81, P411, DOI 10.1016/j.biopsych.2016.08.024

Wang JF, 2019, MOL MED REP, V19, P3, DOI 10.3892/mmr.2018.9679

Whitmer RA, 2005, BMJ-BRIT MED J, V330, P1360, DOI 10.1136/bmj.38446.466238.E0

Wikoff WR, 2009, P NATL ACAD SCI USA, V106, P3698, DOI 10.1073/pnas.0812874106

Winer DA, 2016, CELL METAB, V23, P413, DOI 10.1016/j.cmet.2016.01.003

Wong ML, 2016, MOL PSYCHIATR, V21, P797, DOI 10.1038/mp.2016.46

Wu HJ, 2012, GUT MICROBES, V3, P4, DOI 10.4161/gmic.19320

Yao XM, 2017, NAT COMMUN, V8, DOI 10.1038/s41467-017-01917-2

Zhang Y, 2019, INT J COMP METH-SING, V16, DOI 10.1142/S0219876218400029

Zhou Y, 2016, MOL NEURODEGENER, V11, DOI 10.1186/s13024-016-0094-3

Zhou ZL, 2019, NEUROTHERAPEUTICS, V16, P741, DOI 10.1007/s13311-019-00719-2

NR 136

TC 103

Z9 109

U1 5

U2 75

PU PERGAMON-ELSEVIER SCIENCE LTD

PI OXFORD

PA THE BOULEVARD, LANGFORD LANE, KIDLINGTON, OXFORD OX5 1GB, United Kingdom

SN 0301-0082

EI 1873-5118

J9 PROG NEUROBIOL

JI Prog. Neurobiol.

PD AUG

PY 2020

VL 191

AR 101806

DI 10.1016/j.pneurobio.2020.101806

PG 14

WC Neurosciences

WE Science Citation Index Expanded (SCI-EXPANDED)

SC Neurosciences & Neurology

GA MA2SC

UT WOS:000541766600003

PM 32473843

DA 2024-08-12

ER

PT J

AU Barata-Antunes, S

Cristóvao, AC

Pires, J

Rocha, SM

Bernardino, L

AF Barata-Antunes, S.

Cristovao, A. C.

Pires, J.

Rocha, S. M.

Bernardino, L.

TI Dual role of histamine on microglia-induced neurodegeneration

SO BIOCHIMICA ET BIOPHYSICA ACTA-MOLECULAR BASIS OF DISEASE

LA English

DT Review

DE Histamine; Microglia; Lipopolysaccharide; Neuroinflammation;

Neurodegeneration; Parkinson's disease

ID INFLAMMATORY RESPONSE; NEURONAL DEATH; H-3 RECEPTOR; BRAIN; ACTIVATION;

LPS; NEUROPROTECTION; TRANSDUCTION; STIMULATION; EXPRESSION

AB Several hypotheses have been raised about the dual role of histamine in neurological disorders, and evidences have shown its crucial involvement in the modulation of microglia-mediated neuroinflammation. Previously, we reported that the administration of histamine induces a deleterious effect by promoting a pro-inflammatory phenotype on microglia that in turn compromises dopaminergic neuronal survival. Contrary, under lipopolysaccharide challenge, histamine inhibits the injurious effect of microglia-mediated inflammation, protecting dopaminergic neurons, suggesting that the modulation of microglial activity is dependent on the environmental context. Thus, histamine and/or histamine receptor agonists may serve to develop new therapeutic approaches to overcome neurodegenerative disorders. (C) 2017 Elsevier B.V. All rights reserved.

C1 [Barata-Antunes, S.; Cristovao, A. C.; Pires, J.; Rocha, S. M.; Bernardino, L.] Univ Beira Interior, Fac Hlth Sci, Hlth Sci Res Ctr, Covilha, Portugal.

C3 Universidade da Beira Interior

RP Bernardino, L (corresponding author), Univ Beira Interior, Hlth Sci Res Ctr, Brain Repair Grp, Av Infante D Henrique, P-6200506 Covilha, Portugal.

EM libernardino@fcsaude.ubi.pt

RI Cristovao, Ana Clara/HJH-6476-2023; Cristovao, Ana Clara/AAB-7742-2020;

Rocha, Sandra/E-3127-2015

OI Cristovao, Ana Clara/0000-0001-5806-4478; Bernardino,

Liliana/0000-0003-3395-5973; Antunes, Sandra/0000-0003-1858-4318; Pires,

Joel/0000-0003-3782-185X; Rocha, Sandra/0000-0002-4686-2410

FU FCT Portugal; FEDER; COMPETE [PTDC/SAU-NEU/104415/2008]; L'Oreal-UNESCO

Portugal for Women in Science; FEDER (QREN), through Programa Mais

Centro [CENTRO-07-ST24-FEDER-002008, COMPETE: PEst-C/SAU/UI0709/2011];

FEDER funds through the POCI - COMPETE - Operational Programme

Competitiveness and Internationalization in Axis I - Strengthening

research, technological development and innovation [007491]; National

Funds by FCT [UID/Multi/00709]; FCT [SFRH/BPD/69643/2010]; Fundação para

a Ciência e a Tecnologia [SFRH/BPD/69643/2010] Funding Source: FCT

FX This work was supported by FCT Portugal, FEDER and COMPETE,

PTDC/SAU-NEU/104415/2008, L'Oreal-UNESCO Portugal for Women in Science

and co-funded by FEDER (QREN), through Programa Mais Centro under

project CENTRO-07-ST24-FEDER-002008 and COMPETE: PEst-C/SAU/UI0709/2011.

This work was also supported by FEDER funds through the POCI - COMPETE

2020 - Operational Programme Competitiveness and Internationalization in

Axis I - Strengthening research, technological development and

innovation (Project No. 007491) and National Funds by FCT (Project

UID/Multi/00709). Ana Clara Cristovao is supported by FCT

(SFRH/BPD/69643/2010).

CR Alcañiz L, 2013, FASEB J, V27, P2902, DOI 10.1096/fj.12-223867

Anichtchik OV, 2000, EXP NEUROL, V163, P20, DOI 10.1006/exnr.2000.7362

Bell MT, 2013, CIRCULATION, V128, pS152, DOI 10.1161/CIRCULATIONAHA.112.000024

Bell-Temin H, 2015, MOL CELL PROTEOMICS, V14, P3173, DOI 10.1074/mcp.M115.053926

Benarroch EE, 2010, NEUROLOGY, V75, P1472, DOI 10.1212/WNL.0b013e3181f884b1

Brown RE, 2001, PROG NEUROBIOL, V63, P637, DOI 10.1016/S0301-0082(00)00039-3

Cherry JD, 2014, J NEUROINFLAMM, V11, DOI 10.1186/1742-2094-11-98

Chhor V, 2013, BRAIN BEHAV IMMUN, V32, P70, DOI 10.1016/j.bbi.2013.02.005

Cristóvao AC, 2009, ANTIOXID REDOX SIGN, V11, P2105, DOI [10.1089/ars.2009.2459, 10.1089/ARS.2009.2459]

Cunningham C, 2005, J NEUROSCI, V25, P9275, DOI 10.1523/JNEUROSCI.2614-05.2005

Cunningham C, 2013, GLIA, V61, P71, DOI 10.1002/glia.22350

de Esch IJP, 2005, TRENDS PHARMACOL SCI, V26, P462, DOI 10.1016/j.tips.2005.07.002

Dong HQ, 2014, MOL NEUROBIOL, V49, P1487, DOI 10.1007/s12035-014-8697-6

Esteves M, 2015, FRONT AGING NEUROSCI, V7, DOI 10.3389/fnagi.2015.00020

Ferreira R, 2012, J NEUROINFLAMM, V9, DOI 10.1186/1742-2094-9-90

Frei R, 2013, J ALLERGY CLIN IMMUN, V132, P194, DOI 10.1016/j.jaci.2013.01.013

Haas H, 2003, NAT REV NEUROSCI, V4, P121, DOI 10.1038/nrn1034

Haas HL, 2008, PHYSIOL REV, V88, P1183, DOI 10.1152/physrev.00043.2007

Hanisch UK, 2002, GLIA, V40, P140, DOI 10.1002/glia.10161

Hiraga N, 2007, EUR J PHARMACOL, V557, P236, DOI 10.1016/j.ejphar.2006.11.020

Hu WW, 2012, ACS CHEM NEUROSCI, V3, P238, DOI 10.1021/cn200126p

Iida T, 2015, GLIA, V63, P1213, DOI 10.1002/glia.22812

Jadidi-Niaragh F, 2010, NEUROPHARMACOLOGY, V59, P180, DOI 10.1016/j.neuropharm.2010.05.005

Katoh Y, 2001, NEUROSCI LETT, V305, P181, DOI 10.1016/S0304-3940(01)01835-3

Liu CQ, 2008, PHARMACOL BIOCHEM BE, V90, P325, DOI 10.1016/j.pbb.2008.03.010

Liu CQ, 2007, NEUROPHARMACOLOGY, V53, P832, DOI 10.1016/j.neuropharm.2007.08.014

Liu M, 2011, PARKINSONS DIS, V2011, P1

Lu YC, 2008, CYTOKINE, V42, P145, DOI 10.1016/j.cyto.2008.01.006

Marsh BJ, 2009, NEUROSCIENCE, V158, P1007, DOI 10.1016/j.neuroscience.2008.07.067

Marsh B, 2009, J NEUROSCI, V29, P9839, DOI 10.1523/JNEUROSCI.2496-09.2009

Noh H, 2014, NEUROCHEM INT, V69, P35, DOI 10.1016/j.neuint.2014.02.008

Osterud B, 2014, THROMB RES, V133, P477, DOI 10.1016/j.thromres.2013.12.022

Ousman SS, 2012, NAT NEUROSCI, V15, P1096, DOI 10.1038/nn.3161

Pannell M, 2014, GLIA, V62, P667, DOI 10.1002/glia.22633

Panula P, 2013, NAT REV NEUROSCI, V14, P472, DOI 10.1038/nrn3526

Passani MB, 2011, TRENDS PHARMACOL SCI, V32, P242, DOI 10.1016/j.tips.2011.01.003

Polazzi E, 2010, PROG NEUROBIOL, V92, P293, DOI 10.1016/j.pneurobio.2010.06.009

Qin YZ, 2016, METHODS MOL BIOL, V1465, P1, DOI 10.1007/978-1-4939-4011-0_1

Rocha SM, 2016, J NEUROINFLAMM, V13, DOI 10.1186/s12974-016-0600-0

Rocha SM, 2014, FRONT CELL NEUROSCI, V8, DOI 10.3389/fncel.2014.00120

Rosenzweig HL, 2004, STROKE, V35, P2576, DOI 10.1161/01.STR.0000143450.04438.ae

Schneider E. H., 2015, NEUROPHARMACOLOGY, P116

Seifert S, 2011, CELL CALCIUM, V49, P365, DOI 10.1016/j.ceca.2011.03.005

Valero J., 2014, FRONT NEUROSCI, V8, P1

Vartanian KB, 2011, J NEUROINFLAMM, V8, DOI 10.1186/1742-2094-8-140

Vizuete ML, 2000, J NEUROCHEM, V75, P540, DOI 10.1046/j.1471-4159.2000.0750540.x

Yao LL, 2013, J NEUROINFLAMM, V10, DOI 10.1186/1742-2094-10-23

Yokoyama M, 2004, GASTROENTEROLOGY, V127, P892, DOI 10.1053/j.gastro.2004.06.020

Zhu J, 2014, CELL PHYSIOL BIOCHEM, V34, P768, DOI 10.1159/000363041

Zimmermann C, 2001, BRAIN RES, V895, P59, DOI 10.1016/S0006-8993(01)02028-5

NR 50

TC 40

Z9 44

U1 0

U2 5

PU ELSEVIER SCIENCE BV

PI AMSTERDAM

PA PO BOX 211, 1000 AE AMSTERDAM, NETHERLANDS

SN 0925-4439

EI 1879-260X

J9 BBA-MOL BASIS DIS

JI Biochim. Biophys. Acta-Mol. Basis Dis.

PD MAR

PY 2017

VL 1863

IS 3

BP 764

EP 769

DI 10.1016/j.bbadis.2016.12.016

PG 6

WC Biochemistry & Molecular Biology; Biophysics; Cell Biology

WE Science Citation Index Expanded (SCI-EXPANDED)

SC Biochemistry & Molecular Biology; Biophysics; Cell Biology

GA EK8RC

UT WOS:000394190300013

PM 28057587

OA Bronze

DA 2024-08-12

ER

PT J

AU Bellini, G

Benvenuti, L

Ippolito, C

Frosini, D

Segnani, C

Rettura, F

Pancetti, A

Bertani, L

D'Antongiovanni, V

Palermo, G

Del Prete, E

Antonioli, L

Nardini, V

Morganti, R

Pellegrini, C

Bernardini, N

Ceravolo, R

Fornai, M

Bellini, M

AF Bellini, Gabriele

Benvenuti, Laura

Ippolito, Chiara

Frosini, Daniela

Segnani, Cristina

Rettura, Francesco

Pancetti, Andrea

Bertani, Lorenzo

D'Antongiovanni, Vanessa

Palermo, Giovanni

Del Prete, Eleonora

Antonioli, Luca

Nardini, Vincenzo

Morganti, Riccardo

Pellegrini, Carolina

Bernardini, Nunzia

Ceravolo, Roberto

Fornai, Matteo

Bellini, Massimo

TI Intestinal histomorphological and molecular alterations in patients with

Parkinson's disease

SO EUROPEAN JOURNAL OF NEUROLOGY

LA English

DT Article

DE brain-gut axis; enteric inflammation; gut microbiota; intestinal mucosal

barrier; morphologial remodelling; Parkinson's disease

ID GASTROINTESTINAL DYSFUNCTION; INFLAMMATION; NEUROINFLAMMATION; BARRIER;

CELLS; MODEL

AB Background and purpose Changes in gut microbiota composition, enteric inflammation, impairments of the intestinal epithelial barrier and neuroplastic changes in the enteric nervous system have been reported in Parkinson's disease (PD) patients and could contribute to the onset of both neurological and gastrointestinal symptoms. However, their mutual interplay has rarely been investigated. This study evaluated, in an integrated manner, changes in faecal microbiota composition, morphofunctional alterations of colonic mucosal barrier and changes of inflammatory markers in blood and stools of PD patients. Methods Nineteen PD patients and nineteen asymptomatic subjects were enrolled. Blood lipopolysaccharide binding protein (LBP, marker of altered intestinal permeability) and interleukin-1 beta (IL-1 beta) levels, as well as stool IL-1 beta and tumour necrosis factor (TNF) levels, were evaluated. Gut microbiota analysis was performed. Epithelial mucins, collagen fibres, claudin-1 and S100-positive glial cells as markers of an impairment of the intestinal barrier, mucosal remodelling and enteric glial activation were evaluated on colonic mucosal specimens collected during colonoscopy. Results Faecal microbiota analysis revealed a significant difference in the alpha-diversity in PD patients compared to controls, while no differences were found in the beta-diversity. Compared to controls, PD patients showed significant chenags in plasma LBP levels, as well as faecal TNF and IL-1 beta levels. The histological analysis showed a decrease in epithelial neutral mucins and claudin-1 expression and an increased expression of acidic mucins, collagen fibres and S100-positive glial cells. Conclusions Parkinson's disease patients are characterized by enteric inflammation and increased intestinal epithelial barrier permeability, as well as colonic mucosal barrier remodelling, associated with changes in gut microbiota composition.

C1 [Bellini, Gabriele; Palermo, Giovanni; Ceravolo, Roberto] Univ Pisa, Dept Clin & Expt Med, Neurol Unit, Pisa, Italy.

[Benvenuti, Laura; D'Antongiovanni, Vanessa; Antonioli, Luca; Fornai, Matteo] Univ Pisa, Dept Clin & Expt Med, Unit Pharmacol & Pharmacovigilance, Pisa, Italy.

[Ippolito, Chiara; Segnani, Cristina; Pellegrini, Carolina; Bernardini, Nunzia] Univ Pisa, Dept Clin & Expt Med, Unit Histol & Embryol, Via Roma 55, I-56126 Pisa, Italy.

[Frosini, Daniela; Del Prete, Eleonora] AOUP, Neurol Unit, Dept Med Specialties, Pisa, Italy.

[Rettura, Francesco; Pancetti, Andrea; Bertani, Lorenzo; Bellini, Massimo] Univ Pisa, Dept Translat Res & New Technol Med & Surg, Gastrointestinal Unit, Pisa, Italy.

[Bertani, Lorenzo] Pontedera Hosp, Dept Surg & Gastroenterol, Tuscany North West ASL, Pisa, Italy.

[Nardini, Vincenzo] Azienda Osped Univ Pisana, Anat Patol 2, Pisa, Italy.

[Morganti, Riccardo] Univ Hosp, Sect Stat, Pisa, Italy.

[Ceravolo, Roberto] Univ Pisa, Ctr Neurodegenerat Dis, Unit Neurol,Dept Clin & Expt Med, Parkinsons Dis & Movement Disorders, Pisa, Italy.

C3 University of Pisa; University of Pisa; University of Pisa; University

of Pisa; Azienda Ospedaliero Universitaria Pisana; University of Pisa;

University of Pisa; Azienda Ospedaliero Universitaria Pisana; University

of Pisa; Azienda Ospedaliero Universitaria Pisana; University of Pisa

RP Pellegrini, C (corresponding author), Univ Pisa, Dept Clin & Expt Med, Unit Histol & Embryol, Via Roma 55, I-56126 Pisa, Italy.

EM carolina.pellegrini@unipi.it

RI Bertani, Lorenzo/AAF-7576-2019

OI Bertani, Lorenzo/0000-0001-8653-1790; FORNAI,

MATTEO/0000-0002-0812-3959; Rettura, Francesco/0000-0003-3455-1794;

D'ANTONGIOVANNI, Vanessa/0000-0002-3303-0674; Bellini,

Gabriele/0000-0002-0648-6836; palermo, giovanni/0000-0002-9193-3150

CR Breen DP, 2019, MOVEMENT DISORD, V34, P307, DOI 10.1002/mds.27556

Brun P, 2017, PLOS ONE, V12, DOI 10.1371/journal.pone.0181863

Caenepeel C, 2020, ALIMENT PHARM THER, V52, P1453, DOI 10.1111/apt.16096

Clairembault T, 2015, ACTA NEUROPATHOL COM, V3, DOI 10.1186/s40478-015-0196-0

Clairembault T, 2014, J NEUROCHEM, V130, P805, DOI 10.1111/jnc.12742

Corbillé AG, 2016, NEUROGASTROENT MOTIL, V28, P966, DOI 10.1111/nmo.12797

de Goffau MC, 2014, DIABETOLOGIA, V57, P1569, DOI 10.1007/s00125-014-3274-0

Devos D, 2013, NEUROBIOL DIS, V50, P42, DOI 10.1016/j.nbd.2012.09.007

Dorofeyev AE, 2013, GASTROENT RES PRACT, V2013, DOI 10.1155/2013/431231

Elfil M, 2020, MOVEMENT DISORD, V35, P921, DOI 10.1002/mds.28004

Fasano A, 2015, LANCET NEUROL, V14, P625, DOI 10.1016/S1474-4422(15)00007-1

FOLSTEIN MF, 1975, J PSYCHIAT RES, V12, P189, DOI 10.1016/0022-3956(75)90026-6

Furusawa Y, 2013, NATURE, V504, P446, DOI 10.1038/nature12721

Ghaleb AM, 2011, DEV BIOL, V349, P310, DOI 10.1016/j.ydbio.2010.11.001

Goetz CG, 2007, MOVEMENT DISORD, V22, P41, DOI 10.1002/mds.21198

Gulbransen BD, 2012, NAT REV GASTRO HEPAT, V9, P625, DOI 10.1038/nrgastro.2012.138

HAMILTON M, 1960, J NEUROL NEUROSUR PS, V23, P56, DOI 10.1136/jnnp.23.1.56

Hwang GS, 2015, INT J COLORECTAL DIS, V30, P1051, DOI 10.1007/s00384-015-2256-0

Ippolito C, 2015, J CELL MOL MED, V19, P485, DOI 10.1111/jcmm.12428

Jang JH, 2020, BRAIN BEHAV IMMUN, V89, P641, DOI 10.1016/j.bbi.2020.08.015

Jangi S, 2016, NAT COMMUN, V7, DOI 10.1038/ncomms12015

Kalia LV, 2015, LANCET, V386, P896, DOI 10.1016/S0140-6736(14)61393-3

Keshavarzian A, 2015, MOVEMENT DISORD, V30, P1351, DOI 10.1002/mds.26307

Lecomte M, 2016, MOL NUTR FOOD RES, V60, P609, DOI 10.1002/mnfr.201500703

Lettesjö H, 2006, SCAND J GASTROENTERO, V41, P54, DOI 10.1080/00365520510023909

Lin CH, 2019, J NEUROINFLAMM, V16, DOI 10.1186/s12974-019-1528-y

Lluch J, 2015, PLOS ONE, V10, DOI 10.1371/journal.pone.0142334

Longman RJ, 2006, J HISTOCHEM CYTOCHEM, V54, P1335, DOI 10.1369/jhc.5A6904.2006

Lu Z, 2013, TISSUE BARRIERS, V1, DOI 10.4161/tisb.24978

Lubomski M, 2020, J NEUROL, V267, P2507, DOI 10.1007/s00415-019-09320-1

Martin-Gallausiaux C, 2021, P NUTR SOC, V80, P37, DOI 10.1017/S0029665120006916

Mrabet S, 2016, J CLIN GASTROENTEROL, V50, pE85, DOI 10.1097/MCG.0000000000000606

Neunlist M, 2014, GASTROENTEROLOGY, V147, P1230, DOI 10.1053/j.gastro.2014.09.040

Pellegrini C, 2022, NPJ PARKINSONS DIS, V8, DOI 10.1038/s41531-021-00263-x

Pellegrini C, 2016, NEUROGASTROENT MOTIL, V28, P1781, DOI 10.1111/nmo.12933

Pellegrini C, 2020, NEUROBIOL DIS, V139, DOI 10.1016/j.nbd.2020.104821

Perez-Pardo P, 2019, GUT, V68, P829, DOI 10.1136/gutjnl-2018-316844

Periasamy S, 2013, JPEN-PARENTER ENTER, V37, P674, DOI 10.1177/0148607112468768

PETO V, 1995, QUAL LIFE RES, V4, P241, DOI 10.1007/BF02260863

Rescigno M, 2011, TRENDS IMMUNOL, V32, P256, DOI 10.1016/j.it.2011.04.003

Rieder F, 2007, GUT, V56, P130, DOI 10.1136/gut.2006.090456

Sampson TR, 2016, CELL, V167, P1469, DOI 10.1016/j.cell.2016.11.018

Sarkar SR, 2019, J NEUROIMMUNOL, V328, P98, DOI 10.1016/j.jneuroim.2019.01.004

Scher JU, 2013, ELIFE, V2, DOI 10.7554/eLife.01202

Segnani C, 2015, PLOS ONE, V10, DOI 10.1371/journal.pone.0144630

Si X, 2021, J AGR FOOD CHEM, V69, P3658, DOI 10.1021/acs.jafc.0c07637

Unger MM, 2016, PARKINSONISM RELAT D, V32, P66, DOI 10.1016/j.parkreldis.2016.08.019

Visser M, 2004, MOVEMENT DISORD, V19, P1306, DOI 10.1002/mds.20153

Wang W, 2014, J CLIN MICROBIOL, V52, P398, DOI 10.1128/JCM.01500-13

Weis S, 2019, NPJ PARKINSONS DIS, V5, DOI 10.1038/s41531-019-0100-x

NR 50

TC 8

Z9 8

U1 4

U2 6

PU WILEY

PI HOBOKEN

PA 111 RIVER ST, HOBOKEN 07030-5774, NJ USA

SN 1351-5101

EI 1468-1331

J9 EUR J NEUROL

JI Eur. J. Neurol.

PD NOV

PY 2023

VL 30

IS 11

SI SI

BP 3440

EP 3450

DI 10.1111/ene.15607

EA NOV 2022

PG 11

WC Clinical Neurology; Neurosciences

WE Science Citation Index Expanded (SCI-EXPANDED)

SC Neurosciences & Neurology

GA EY7S3

UT WOS:000879341000001

PM 36263629

DA 2024-08-12

ER

PT J

AU Vallée, A

Lecarpentier, Y

Guillevin, R

Vallée, JN

AF Vallee, Alexandre

Lecarpentier, Yves

Guillevin, Remy

Vallee, Jean-Noel

TI Circadian rhythms, Neuroinflammation and Oxidative Stress in the Story

of Parkinson's Disease

SO CELLS

LA English

DT Review

DE circadian rhythms; Parkinson's disease; oxidative stress;

neuroinflammation

ID REV-ERB-ALPHA; alzheimer's disease; CLOCK GENE; INNATE IMMUNITY;

MITOCHONDRIAL DYSFUNCTION; MELATONIN PREVENTS; SUBSTANTIA-NIGRA; HOST

RESPONSE; SLEEP; RAT

AB Parkinson's disease (PD) is one of the main neurodegenerative disease characterized by a progressive degeneration of neurons constituted by dopamine in the substantia nigra pars compacta. The etiologies of PD remain unclear. Aging is the main risk factor for PD. Aging could dysregulate molecular pathways controlling cell homeostatic mechanisms. PD cells are the sites of several metabolic abnormalities including neuroinflammation and oxidative stress. Metabolic structures are driven by circadian rhythms. Biologic rhythms are complex systems interacting with the environment and controlling several physiological pathways. Recent findings have shown that the dysregulation of the circadian rhythms is correlated with PD and its metabolic dysregulations. This review is focused on the key role of circadian rhythms and their impact on neuroinflammation and oxidative stress in Parkinson's disease.

C1 [Vallee, Alexandre; Guillevin, Remy] Univ Poitiers, CHU Poitiers, CNRS, UMR 7348,DACTIM MIS,LMA, F-86021 Poitiers, France.

[Lecarpentier, Yves] GHEF, Ctr Rech Clin, F-77100 Meaux, France.

[Vallee, Jean-Noel] UPJV, CHU Amiens Picardie, F-80000 Amiens, France.

[Vallee, Jean-Noel] Univ Poitiers, CNRS, UMR 7348, LMA, F-86021 Poitiers, France.

C3 CHU Poitiers; Universite de Poitiers; Centre National de la Recherche

Scientifique (CNRS); CNRS - National Institute for Mathematical Sciences

(INSMI); Universite de Picardie Jules Verne (UPJV); CHU Amiens;

Universite de Poitiers; Centre National de la Recherche Scientifique

(CNRS); CNRS - National Institute for Mathematical Sciences (INSMI)

RP Vallée, A (corresponding author), Univ Poitiers, CHU Poitiers, CNRS, UMR 7348,DACTIM MIS,LMA, F-86021 Poitiers, France.

EM alexandre.g.vallee@gmail.com; yves.c.lecarpentier@gmail.com;

remy.guillevin@chu-poitiers.fr; valleejn@gmail.com

RI Vallée, Alexandre/AEA-9496-2022

OI Vallee, Alexandre/0000-0001-9158-4467

CR Abbott RD, 2005, NEUROLOGY, V65, P1442, DOI 10.1212/01.wnl.0000183056.89590.0d

Abe M, 2002, J NEUROSCI, V22, P350, DOI 10.1523/JNEUROSCI.22-01-00350.2002

AMES BN, 1993, P NATL ACAD SCI USA, V90, P7915, DOI 10.1073/pnas.90.17.7915

Anea CB, 2009, CIRCULATION, V119, P1510, DOI 10.1161/CIRCULATIONAHA.108.827477

Anglade P, 1997, HISTOL HISTOPATHOL, V12, P25

Arjona A, 2006, J INTERF CYTOK RES, V26, P645, DOI 10.1089/jir.2006.26.645

Atger F, 2017, FRONT ENDOCRINOL, V8, DOI 10.3389/fendo.2017.00042

Bass J, 2010, SCIENCE, V330, P1349, DOI 10.1126/science.1195027

Beaver LM, 2012, PLOS ONE, V7, DOI 10.1371/journal.pone.0050454

Bell-Pedersen D, 2005, NAT REV GENET, V6, P544, DOI 10.1038/nrg1633

Bellet MM, 2013, P NATL ACAD SCI USA, V110, P9897, DOI 10.1073/pnas.1120636110

Benilova I, 2012, NAT NEUROSCI, V15, P349, DOI 10.1038/nn.3028

Blesa J, 2015, FRONT NEUROANAT, V9, DOI 10.3389/fnana.2015.00091

Bloch G, 2013, P ROY SOC B-BIOL SCI, V280, DOI 10.1098/rspb.2013.0035

Bonuccelli U, 2000, CLIN NEUROPHARMACOL, V23, P28, DOI 10.1097/00002826-200001000-00006

Braak H, 2004, CELL TISSUE RES, V318, P121, DOI 10.1007/s00441-004-0956-9

Breen DP, 2014, JAMA NEUROL, V71, P589, DOI 10.1001/jamaneurol.2014.65

Cai Y, 2010, EUR J NEUROL, V17, P550, DOI 10.1111/j.1468-1331.2009.02848.x

Calvo JR, 2013, J PINEAL RES, V55, P103, DOI 10.1111/jpi.12075

Cardinali DP, 2013, HORM BEHAV, V63, P322, DOI 10.1016/j.yhbeh.2012.02.020

Carneiro BTS, 2012, FRONT BEHAV NEUROSCI, V6, DOI 10.3389/fnbeh.2012.00083

CASSONE VM, 1990, TRENDS NEUROSCI, V13, P457, DOI 10.1016/0166-2236(90)90099-V

Chen LH, 2014, PPAR RES, V2014, DOI 10.1155/2014/653017

Chen ST, 2002, J PINEAL RES, V32, P262, DOI 10.1034/j.1600-079X.2002.01871.x

Choi SH, 2009, TRENDS PHARMACOL SCI, V30, P174, DOI 10.1016/j.tips.2009.01.002

Chuang JI, 2016, J PINEAL RES, V61, P230, DOI 10.1111/jpi.12343

COYLE JT, 1993, SCIENCE, V262, P689, DOI 10.1126/science.7901908

Crowley SJ, 2013, PSYCHOPHARMACOLOGY, V225, P825, DOI 10.1007/s00213-012-2869-8

Csernus V, 2003, NEUROENDOCRINOL LETT, V24, P404

Curtis AM, 2015, P NATL ACAD SCI USA, V112, P7231, DOI 10.1073/pnas.1501327112

CZEISLER CA, 1992, LANCET, V340, P933, DOI 10.1016/0140-6736(92)92817-Y

Devos D, 2003, J NEURAL TRANSM, V110, P997, DOI 10.1007/s00702-003-0016-8

Dias V, 2013, J PARKINSON DIS, V3, P461, DOI 10.3233/JPD-130230

Dibner C, 2010, ANNU REV PHYSIOL, V72, P517, DOI 10.1146/annurev-physiol-021909-135821

Doi M, 2006, CELL, V125, P497, DOI 10.1016/j.cell.2006.03.033

Dubocovich ML, 2007, SLEEP MED, V8, pS34, DOI 10.1016/j.sleep.2007.10.007

Duffy JF, 2002, AM J PHYSIOL-ENDOC M, V282, pE297, DOI 10.1152/ajpendo.00268.2001

Dunlap JC, 1999, CELL, V96, P271, DOI 10.1016/S0092-8674(00)80566-8

Edgar RS, 2012, NATURE, V485, P459, DOI 10.1038/nature11088

Evans JA, 2013, PROG MOL BIOL TRANSL, V119, P283, DOI 10.1016/B978-0-12-396971-2.00010-5

Franco-Iborra S, 2016, NEUROSCIENTIST, V22, P266, DOI 10.1177/1073858415574600

Ortiz GG, 2013, METAB BRAIN DIS, V28, P705, DOI 10.1007/s11011-013-9416-0

Galano A, 2013, J PINEAL RES, V54, P245, DOI 10.1111/jpi.12010

Gao JJ, 2011, AM J EPIDEMIOL, V173, P1032, DOI 10.1093/aje/kwq478

Gardet A, 2010, J IMMUNOL, V185, P5577, DOI 10.4049/jimmunol.1000548

Gekakis N, 1998, SCIENCE, V280, P1564, DOI 10.1126/science.280.5369.1564

Gibbs JE, 2012, P NATL ACAD SCI USA, V109, P582, DOI 10.1073/pnas.1106750109

Giordano S, 2014, REDOX BIOL, V2, P82, DOI 10.1016/j.redox.2013.12.013

Glass CK, 2010, CELL, V140, P918, DOI 10.1016/j.cell.2010.02.016

Goldbeter A, 2017, CHAOS, V27, DOI 10.1063/1.4990783

Gravotta L, 2011, J MOL NEUROSCI, V45, P162, DOI 10.1007/s12031-011-9520-8

Grinberg LT, 2010, J NEUROL SCI, V289, P81, DOI 10.1016/j.jns.2009.08.021

Guerreiro PS, 2016, MOL NEUROBIOL, V53, P3124, DOI 10.1007/s12035-015-9209-z

Gupta A, 2008, ANN NEUROL, V64, pS3, DOI 10.1002/ana.21573

Guven Celal, 2016, Med Sci Monit, V22, P438

Hashiramoto A, 2010, J IMMUNOL, V184, P1560, DOI 10.4049/jimmunol.0903284

Hassanzadeh K, 2019, J CELL PHYSIOL, V234, P23, DOI 10.1002/jcp.26865

Hastings MH, 2019, BIOLOGY-BASEL, V8, DOI 10.3390/biology8010013

Hofman MA, 2000, CHRONOBIOL INT, V17, P245, DOI 10.1081/CBI-100101047

Hogenesch JB, 1998, P NATL ACAD SCI USA, V95, P5474, DOI 10.1073/pnas.95.10.5474

Hood S, 2010, J NEUROSCI, V30, P14046, DOI 10.1523/JNEUROSCI.2128-10.2010

Imbesi M, 2009, NEUROSCIENCE, V158, P537, DOI 10.1016/j.neuroscience.2008.10.044

Ishido M, 2007, J PINEAL RES, V42, P125, DOI 10.1111/j.1600-079X.2006.00390.x

Islam MT, 2017, NEUROL RES, V39, P73, DOI 10.1080/01616412.2016.1251711

Jenner P, 2003, ANN NEUROL, V53, pS26, DOI 10.1002/ana.10483

Johnson CH, 2003, CHRONOBIOL INT, V20, P741, DOI 10.1081/CBI-120024211

Kato H, 2015, J PINEAL RES, V59, P267, DOI 10.1111/jpi.12259

Keller M, 2009, P NATL ACAD SCI USA, V106, P21407, DOI 10.1073/pnas.0906361106

Kim GH, 2015, EXP NEUROBIOL, V24, P325, DOI 10.5607/en.2015.24.4.325

Ko CH, 2006, HUM MOL GENET, V15, pR271, DOI 10.1093/hmg/ddl207

Kondratova AA, 2012, NAT REV NEUROSCI, V13, P325, DOI 10.1038/nrn3208

Krishnan N, 2008, BIOCHEM BIOPH RES CO, V374, P299, DOI 10.1016/j.bbrc.2008.07.011

Krishnan N, 2009, AGING-US, V1, P937, DOI 10.18632/aging.100103

Lauretti E, 2017, MOL PSYCHIATR, V22, P280, DOI 10.1038/mp.2016.47

Lecarpentier Y, 2017, ENTROPY-SWITZ, V19, DOI 10.3390/e19100558

Lee J, 2011, ISLETS, V3, P381, DOI 10.4161/isl.3.6.18157

Lim KL, 2007, BMC BIOCHEM, V8, DOI 10.1186/1471-2091-8-S1-S13

López A, 2017, PLOS ONE, V12, DOI 10.1371/journal.pone.0183090

Lopez de Maturana Rakel, 2016, J Neuroinflammation, V13, P295

Luque-Contreras D, 2014, OXID MED CELL LONGEV, V2014, DOI 10.1155/2014/497802

Mackey SR, 2011, ADV GENET, V74, P13, DOI 10.1016/B978-0-12-387690-4.00002-7

Maguire-Zeiss KA, 2010, J NEURAL TRANSM, V117, P1019, DOI 10.1007/s00702-010-0431-6

Marpegan L, 2011, J NEUROSCI, V31, P8342, DOI 10.1523/JNEUROSCI.6537-10.2011

Mattam U, 2015, BIOGERONTOLOGY, V16, P109, DOI 10.1007/s10522-014-9541-0

Mauriz JL, 2013, J PINEAL RES, V54, P1, DOI 10.1111/j.1600-079X.2012.01014.x

Mayo JC, 1998, J PINEAL RES, V24, P179, DOI 10.1111/j.1600-079X.1998.tb00531.x

Mayo JC, 1999, BRAIN RES, V818, P221, DOI 10.1016/S0006-8993(98)01262-1

MCGEER PL, 1988, NEUROLOGY, V38, P1285, DOI 10.1212/WNL.38.8.1285

MIZOBUCHI M, 1993, BRAIN RES, V612, P319, DOI 10.1016/0006-8993(93)91678-L

Mogi M, 2000, J NEURAL TRANSM, V107, P335, DOI 10.1007/s007020050028

Mohawk JA, 2012, ANNU REV NEUROSCI, V35, P445, DOI 10.1146/annurev-neuro-060909-153128

Moussaud S, 2014, MOL NEURODEGENER, V9, DOI 10.1186/1750-1326-9-43

Musiek ES, 2015, FRONT PHARMACOL, V6, DOI 10.3389/fphar.2015.00029

Musiek ES, 2013, J CLIN INVEST, V123, P5389, DOI 10.1172/JCI70317

Narasimamurthy R, 2012, P NATL ACAD SCI USA, V109, P12662, DOI 10.1073/pnas.1209965109

Nguyen KD, 2013, SCIENCE, V341, P1483, DOI 10.1126/science.1240636

O'Neill JS, 2011, NATURE, V469, P498, DOI 10.1038/nature09702

Olanow CW, 2006, LANCET NEUROL, V5, P1013, DOI 10.1016/S1474-4422(06)70602-0

Ozsoy O, 2015, FREE RADICAL RES, V49, P1004, DOI 10.3109/10715762.2015.1027198

Parekh PK, 2015, ALCOHOL, V49, P341, DOI 10.1016/j.alcohol.2014.09.034

Paul R, 2018, LIFE SCI, V192, P238, DOI 10.1016/j.lfs.2017.11.016

Pevet P, 2011, J PHYSIOL-PARIS, V105, P170, DOI 10.1016/j.jphysparis.2011.07.001

Piccini P, 1991, Riv Neurol, V61, P219

Preitner N, 2002, CELL, V110, P251, DOI 10.1016/S0092-8674(02)00825-5

PRIGOGIN.I, 1974, ANN NY ACAD SCI, V231, P99, DOI 10.1111/j.1749-6632.1974.tb20557.x

PRIGOGINE I, 1986, CELL BIOPHYS, V9, P217, DOI 10.1007/BF02797383

Puspita L, 2017, MOL BRAIN, V10, DOI 10.1186/s13041-017-0340-9

Rahimmi A, 2015, BRAIN RES BULL, V113, P34, DOI 10.1016/j.brainresbull.2015.02.007

Ramesh G, 2013, MEDIAT INFLAMM, V2013, DOI 10.1155/2013/480739

Reppert SM, 2002, NATURE, V418, P935, DOI 10.1038/nature00965

Rocha NP, 2015, BIOMED RES INT, V2015, DOI 10.1155/2015/628192

Rosales-Corral SA, 2012, J PINEAL RES, V52, P167, DOI 10.1111/j.1600-079X.2011.00937.x

Russo I, 2015, J NEUROINFLAMM, V12, DOI 10.1186/s12974-015-0449-7

Russo I, 2014, J NEUROINFLAMM, V11, DOI 10.1186/1742-2094-11-52

Saravanan KS, 2007, J PINEAL RES, V42, P247, DOI 10.1111/j.1600-079X.2006.00412.x

Sato S, 2014, J IMMUNOL, V192, P407, DOI 10.4049/jimmunol.1301982

Schapira AHV, 2008, LANCET NEUROL, V7, P97, DOI 10.1016/S1474-4422(07)70327-7

Schibler U, 2002, CELL, V111, P919, DOI 10.1016/S0092-8674(02)01225-4

Shih RH, 2015, FRONT MOL NEUROSCI, V8, DOI 10.3389/fnmol.2015.00077

Shimoji M, 2009, NEUROTOX RES, V16, P318, DOI 10.1007/s12640-009-9076-3

Silver AC, 2012, IMMUNITY, V36, P251, DOI 10.1016/j.immuni.2011.12.017

Singhal NK, 2011, J PINEAL RES, V50, P97, DOI 10.1111/j.1600-079X.2010.00819.x

Sochocka M, 2013, CNS NEUROL DISORD-DR, V12, P870, DOI 10.2174/18715273113129990072

Spengler ML, 2012, P NATL ACAD SCI USA, V109, pE2457, DOI 10.1073/pnas.1206274109

Stapleton CM, 2005, AM J PHYSIOL-LUNG C, V289, pL144, DOI 10.1152/ajplung.00348.2004

STRUCK LK, 1990, NEUROLOGY, V40, P467, DOI 10.1212/WNL.40.3_Part_1.467

Surace MJ, 2012, CELL MOL LIFE SCI, V69, P2409, DOI 10.1007/s00018-012-1015-4

Tufekci KU, 2012, ADV PROTEIN CHEM STR, V88, P69, DOI 10.1016/B978-0-12-398314-5.00004-0

Vallée A, 2019, CURR ISSUES MOL BIOL, V31, P21, DOI 10.21775/cimb.031.021

Vallée A, 2018, NEUROMOL MED, V20, P174, DOI 10.1007/s12017-018-8486-x

Videnovic A, 2016, MOVEMENT DISORD, V31, P260, DOI 10.1002/mds.26509

Videnovic A, 2015, SLEEP MED CLIN, V10, P469, DOI 10.1016/j.jsmc.2015.08.004

Videnovic A, 2013, EXP NEUROL, V243, P45, DOI 10.1016/j.expneurol.2012.08.018

Wanagat J, 2010, MECH AGEING DEV, V131, P527, DOI 10.1016/j.mad.2010.06.002

Wang X, 2011, J NEUROSCI, V31, P14496, DOI 10.1523/JNEUROSCI.3059-11.2011

WEAVER DR, 1993, J CLIN ENDOCR METAB, V76, P295, DOI 10.1210/jc.76.2.295

Weishaupt JH, 2006, J PINEAL RES, V41, P313, DOI 10.1111/j.1600-079X.2006.00377.x

Wu YH, 2005, J PINEAL RES, V38, P145, DOI 10.1111/j.1600-079X.2004.00196.x

Yacoubian TA, 2009, BBA-MOL BASIS DIS, V1792, P676, DOI 10.1016/j.bbadis.2008.09.009

Yin F, 2014, ANTIOXID REDOX SIGN, V20, P353, DOI 10.1089/ars.2012.4774

Yu XF, 2013, SCIENCE, V342, P727, DOI 10.1126/science.1243884

Yujnovsky I, 2006, P NATL ACAD SCI USA, V103, P6386, DOI 10.1073/pnas.0510691103

Zhang HM, 2014, J PINEAL RES, V57, P131, DOI 10.1111/jpi.12162

NR 143

TC 33

Z9 36

U1 4

U2 37

PU MDPI

PI BASEL

PA ST ALBAN-ANLAGE 66, CH-4052 BASEL, SWITZERLAND

EI 2073-4409

J9 CELLS-BASEL

JI Cells

PD FEB

PY 2020

VL 9

IS 2

AR 314

DI 10.3390/cells9020314

PG 15

WC Cell Biology

WE Science Citation Index Expanded (SCI-EXPANDED)

SC Cell Biology

GA KX5UC

UT WOS:000521944900055

PM 32012898

OA Green Published, gold

DA 2024-08-12

ER

PT J

AU Talebi, M

Talebi, M

Farkhondeh, T

Kopustinskiene, DM

Simal-Gandara, J

Bernatoniene, J

Samarghandian, S

AF Talebi, Marjan

Talebi, Mohsen

Farkhondeh, Tahereh

Kopustinskiene, Dalia M.

Simal-Gandara, Jesus

Bernatoniene, Jurga

Samarghandian, Saeed

TI An updated review on the versatile role of chrysin in neurological

diseases: Chemistry, pharmacology, and drug delivery approaches

SO BIOMEDICINE & PHARMACOTHERAPY

LA English

DT Article

DE Chrysin; Neuroprotective; Signaling pathways; Neurological diseases;

Neurodegenerative disease; Brain-targeted drug delivery

ID BLOOD-BRAIN-BARRIER; CENTRAL-NERVOUS-SYSTEM; OXIDATIVE-STRESS; FLAVONOID

CHRYSIN; IN-VIVO; DIETARY FLAVONOIDS; CEREBRAL ISCHEMIA/REPERFUSION;

SCUTELLARIA-BAICALENSIS; LIQUID-CHROMATOGRAPHY; LIPID NANOPARTICLES

AB Neurological diseases are responsible for a large number of morbidities and mortalities in the world. Flavonoids are phytochemicals that possess various health-promoting impacts. Chrysin, a natural flavonoid isolated from diverse fruits, vegetables, and even mushrooms, has several pharmacological activities comprising antioxidant, anti-inflammatory, antiapoptotic, anticancer, and neuroprotective effects. The current study was designed to review the relationship between chrysin administration and neurological complications by discussing the feasible mechanism and signaling pathways. Herein, we mentioned the sources, pharmacological properties, chemistry, and drug delivery systems associated with chrysin pharmacotherapy. The role of chrysin was discussed in depression, anxiety, neuroinflammation, Alzheimer's disease, Parkinson's disease, Huntington's disease, epilepsy, cerebral ischemia, spinal cord injury, neuropathy, Multiple Sclerosis, and Guillain-Barre ' Syndrome. The findings indicate that chrysin has protective effects against neurological conditions by modulating oxidative stress, inflammation, and apoptosis in animal models. However, more studies should be done to clear the neuroprotective effects of chrysin.

C1 [Talebi, Marjan] Shahid Beheshti Univ Med Sci, Sch Pharm, Dept Pharmacognosy & Pharmaceut Biotechnol, Tehran, Iran.

[Talebi, Mohsen] Univ Texas Arlington, Dept Chem & Biochem, Arlington, TX 76019 USA.

[Talebi, Mohsen] Viatris Pharmaceut Inc, 3300 Res Plaza, San Antonio, TX 78235 USA.

[Farkhondeh, Tahereh] Birjand Univ Med Sci, Cardiovasc Dis Res Ctr, Birjand, Iran.

[Farkhondeh, Tahereh] Birjand Univ Med Sci, Fac Pharm, Birjand, Iran.

[Kopustinskiene, Dalia M.; Bernatoniene, Jurga] Lithuanian Univ Hlth Sci, Med Acad, Fac Pharm, Inst Pharmaceut Technol, Sukileliu Pr 13, LT-50161 Kaunas, Lithuania.

[Simal-Gandara, Jesus] Univ Vigo, Fac Sci, Dept Analyt & Food Chem, Nutr & Bromatol Grp, Ourense Campus, E-32004 Orense, Spain.

[Samarghandian, Saeed] Neyshabur Univ Med Sci, Noncommunicable Dis Res Ctr, Neyshabur, Iran.

C3 Shahid Beheshti University Medical Sciences; University of Texas System;

University of Texas Arlington; Lithuanian University of Health Sciences;

Universidade de Vigo

RP Samarghandian, S (corresponding author), Neyshabur Univ Med Sci, Noncommunicable Dis Res Ctr, Neyshabur, Iran.

EM samarghandians1@nums.ac.ir

RI Simal-Gandara, Jesus/A-9533-2009; Farkhondeh, Tahereh/Y-2083-2018;

Talebi, Marjan/AAS-8240-2020

OI Simal-Gandara, Jesus/0000-0001-9215-9737; Talebi,

Marjan/0000-0001-5513-3204

CR Abd El Hady F., 2015, Int J Pharm Sci Rev Res, V35, P53

Abd El-Hady F. K., 2013, INT J PHARM SCI REV, V21, P338

Ahlawat J, 2020, ACS OMEGA, V5, P12583, DOI 10.1021/acsomega.0c01592

Ahmed-Belkacem A, 2005, CANCER RES, V65, P4852, DOI 10.1158/0008-5472.CAN-04-1817

Al-Hatamleh MAI, 2020, BIOMOLECULES, V10, DOI 10.3390/biom10060923

Alavijeh Mohammad S, 2005, NeuroRx, V2, P554, DOI 10.1602/neurorx.2.4.554

Alfa HH, 2019, PHYTOCHEM REV, V18, P989, DOI 10.1007/s11101-019-09632-0

Ali M, 2017, FRONT PHARMACOL, V8, DOI 10.3389/fphar.2017.00327

Alkahtane AA, 2021, INT J MED SCI, V18, P199, DOI 10.7150/ijms.51382

Anand KV, 2012, GERIATR GERONTOL INT, V12, P741, DOI 10.1111/j.1447-0594.2012.00843.x

Anari E, 2016, ARTIF CELL NANOMED B, V44, P1410, DOI 10.3109/21691401.2015.1029633

Angelopoulou E, 2020, NEUROCHEM INT, V132, DOI 10.1016/j.neuint.2019.104612

[Anonymous], 2015, INT J PHARMACOGNOSY

Atanasov AG, 2021, NAT REV DRUG DISCOV, V20, P200, DOI 10.1038/s41573-020-00114-z

Bagchi S, 2019, DRUG DES DEV THER, V13, P3591, DOI 10.2147/DDDT.S218708

Bajgai SP, 2011, PHYTOCHEMISTRY, V72, P2062, DOI 10.1016/j.phytochem.2011.07.002

Basu A, 2016, J CARDIOVASC PHARM, V68, P89, DOI 10.1097/FJC.0000000000000380

Beaumont Denise M, 2008, AANA J, V76, P113

Begum S, 2020, NAT PROD J, V10, P384, DOI 10.2174/2210315509666190626124539

Bernardo-Castro S, 2020, FRONT NEUROL, V11, DOI 10.3389/fneur.2020.594672

Biglar M, 2014, IRAN J PHARM RES, V13, P195

Borges Filho C, 2016, EUR J PHARMACOL, V791, P284, DOI 10.1016/j.ejphar.2016.09.005

Bortolotto VC, 2020, PHYSIOL BEHAV, V222, DOI 10.1016/j.physbeh.2020.112892

Bortolotto VC, 2018, EUR J PHARMACOL, V822, P78, DOI 10.1016/j.ejphar.2018.01.017

Catarino MD, 2015, CURR TOP MED CHEM, V15, P105, DOI 10.2174/1568026615666141209144506

Caterino M, 2018, NEUROPHARMACOLOGY, V135, P126, DOI 10.1016/j.neuropharm.2018.03.009

Cho I, 2020, FOOD SCI NUTR, V8, P6633, DOI 10.1002/fsn3.1956

Ciccone L, 2020, J ENZYM INHIB MED CH, V35, P1145, DOI 10.1080/14756366.2020.1760262

Cueto-Escobedo J, 2020, BEHAV BRAIN RES, V386, DOI 10.1016/j.bbr.2020.112590

Dao TT, 2004, BIOORG MED CHEM LETT, V14, P1165, DOI 10.1016/j.bmcl.2003.12.087

Darendelioglu E, 2020, NEUROCHEM RES, V45, P1064, DOI 10.1007/s11064-020-02982-8

Del Fabbro L, 2019, J NEUROIMMUNOL, V335, DOI 10.1016/j.jneuroim.2019.577007

Del Fabbro L, 2019, NEUROSCI LETT, V706, P158, DOI 10.1016/j.neulet.2019.05.036

Dhawan Kamaldeep, 2002, Journal of Medicinal Food, V5, P43

Dong XW, 2018, THERANOSTICS, V8, P1481, DOI 10.7150/thno.21254

Dragicevic N, 2011, J ALZHEIMERS DIS, V26, P507, DOI 10.3233/JAD-2011-101629

Durak MA, 2016, TURK J MED SCI, V46, P1926, DOI 10.3906/sag-1508-119

During A, 2013, BIOCHEM PHARMACOL, V86, P1739, DOI 10.1016/j.bcp.2013.10.003

Ebrahimi A, 2012, AGEING RES REV, V11, P329, DOI 10.1016/j.arr.2012.01.006

El-Hady FK Abd., 2015, Int J Pharm Sci Rev Res, V34, P32

Engelbrecht Idalet, 2019, Central Nervous System Agents in Medicinal Chemistry, V19, P133, DOI 10.2174/1871524919666190619090852

Farkhondeh T, 2020, MOLECULES, V25, DOI 10.3390/molecules25215218

Farkhondeh Tahereh, 2020, Cardiovascular & Hematological Disorders - Drug Targets, V20, P47, DOI 10.2174/1871529X19666190618144440

Farkhondeh T, 2019, J CELL PHYSIOL, V234, P17144, DOI 10.1002/jcp.28488

Farkhondeh Tahereh, 2019, Cardiovascular & Hematological Agents in Medicinal Chemistry, V17, P17, DOI 10.2174/1871525717666190114145137

Farkhondeh T, 2018, DOSE-RESPONSE, V16, DOI 10.1177/1559325818761455

Figueira I, 2017, CURR NEUROPHARMACOL, V15, P562, DOI 10.2174/1570159X14666161026151545

Filho CB, 2015, NEUROSCIENCE, V289, P367, DOI 10.1016/j.neuroscience.2014.12.048

Almeida AF, 2018, CURR PHARM DESIGN, V24, P2188, DOI 10.2174/1381612824666180516103609

Fonseca SF, 2015, NEW J CHEM, V39, P3043, DOI 10.1039/c4nj02329c

Rodríguez-Landa JF, 2021, BEHAV BRAIN RES, V397, DOI 10.1016/j.bbr.2020.112952

Rodríguez-Landa JF, 2019, BIOMED PHARMACOTHER, V109, P2387, DOI 10.1016/j.biopha.2018.11.111

Franova S, 2020, J PHARM PHARMACOL

Galijatovic A, 1999, XENOBIOTICA, V29, P1241, DOI 10.1080/004982599237912

Gambelunghe Cristiana, 2003, Journal of Medicinal Food, V6, P387, DOI 10.1089/109662003772519967

Gao X, 2012, NEUROLOGY, V78, P1138, DOI 10.1212/WNL.0b013e31824f7fc4

Garcia-Alloza M, 2006, J NEUROPATH EXP NEUR, V65, P1082, DOI 10.1097/01.jnen.0000240468.12543.af

Gardner I, 2005, CHEM RES TOXICOL, V18, P1384, DOI 10.1021/tx050095o

Gautam R, 2009, MED RES REV, V29, P767, DOI 10.1002/med.20156

Ge SF, 2015, J AGR FOOD CHEM, V63, P2902, DOI 10.1021/jf5056979

Gharari Z, 2020, J APPL RES MED AROMA, V16, DOI 10.1016/j.jarmap.2019.100232

Giacomeli R, 2020, BEHAV BRAIN RES, V390, DOI 10.1016/j.bbr.2020.112696

Giuliano C, 2021, NEURAL REGEN RES, V16, P234, DOI 10.4103/1673-5374.290879

Goes ATR, 2018, CHEM-BIOL INTERACT, V279, P111, DOI 10.1016/j.cbi.2017.10.019

Gresa-Arribas N, 2010, J NEUROCHEM, V115, P526, DOI 10.1111/j.1471-4159.2010.06952.x

Guo BJ, 2016, J AGR FOOD CHEM, V64, P5324, DOI 10.1021/acs.jafc.6b01707

Ha SK, 2010, NEUROSCI LETT, V485, P143, DOI 10.1016/j.neulet.2010.08.064

Hadjmohammadi MR, 2010, J SEP SCI, V33, P3144, DOI 10.1002/jssc.201000326

Haider M, 2022, INT J NEUROSCI, V132, P450, DOI 10.1080/00207454.2020.1821677

Harris GK, 2006, J NUTR, V136, P1517, DOI 10.1093/jn/136.6.1517

Haumann R, 2020, CNS DRUGS, V34, P1121, DOI 10.1007/s40263-020-00766-w

He XL, 2012, EUR J PHARMACOL, V680, P41, DOI 10.1016/j.ejphar.2012.01.025

He YT, 2019, INT IMMUNOPHARMACOL, V68, P171, DOI 10.1016/j.intimp.2018.12.059

Hritcu L, 2017, OXID MED CELL LONGEV, V2017, DOI 10.1155/2017/5762172

Infante-Garcia C, 2019, INT J MOL SCI, V20, DOI 10.3390/ijms20102533

Islam MN, 2011, METABOLOMICS, V7, P446, DOI 10.1007/s11306-010-0269-9

Izuta H, 2008, J AGR FOOD CHEM, V56, P8944, DOI 10.1021/jf8014206

Jaganathan SK, 2009, J BIOMED BIOTECHNOL, DOI 10.1155/2009/830616

Jahani R, 2019, IRAN J PHARM RES, V18, P1456, DOI 10.22037/ijpr.2019.15579.13194

Jana K, 2008, J ENDOCRINOL, V197, P315, DOI 10.1677/JOE-07-0282

Germán-Ponciano LJ, 2020, ACTA PHARMACEUT, V70, P387, DOI 10.2478/acph-2020-0022

Jiang Y, 2014, INT J MOL SCI, V15, P12270, DOI 10.3390/ijms150712270

Jorge A, 2019, WORLD NEUROSURG, V132, P138, DOI 10.1016/j.wneu.2019.08.108

Jung J, 2016, J NANOMATER, V2016, DOI 10.1155/2016/2894089

Kalogeropoulos N, 2013, FOOD CHEM TOXICOL, V55, P378, DOI 10.1016/j.fct.2013.01.010

Kamat S, 2020, SCI REP-UK, V10, DOI 10.1038/s41598-020-72497-3

Kandhare AD, 2014, J NAT MED-TOKYO, V68, P586, DOI 10.1007/s11418-014-0840-1

Kao YC, 1998, ENVIRON HEALTH PERSP, V106, P85, DOI 10.1289/ehp.9810685

Kasala ER, 2016, CANCER GENE THER, V23, P43, DOI 10.1038/cgt.2015.65

Kasala ER, 2015, TOXICOL LETT, V233, P214, DOI 10.1016/j.toxlet.2015.01.008

Khan H, 2021, SEMIN CANCER BIOL, V69, P200, DOI 10.1016/j.semcancer.2019.07.023

Khoo BY, 2010, INT J MOL SCI, V11, P2188, DOI 10.3390/ijms11052188

Ko YH, 2020, ARCH PHARM RES, V43, P1128, DOI 10.1007/s12272-020-01292-5

Kopustinskiene DM, 2020, NUTRIENTS, V12, DOI 10.3390/nu12020457

Krishnamoorthy A., 2020, INT J RES PHARM SCI, V11, P4388

Krishnamoorthy A, 2019, NEUROSCI LETT, V709, DOI 10.1016/j.neulet.2019.134382

Kumar Hitesh, 2017, Pharmaceutical Nanotechnology, V5, P203, DOI 10.2174/2211738505666170515113936

Kuo SM, 1997, CRIT REV ONCOGENESIS, V8, P47, DOI 10.1615/CritRevOncog.v8.i1.30

Kurek-Górecka A, 2020, MOLECULES, V25, DOI 10.3390/molecules25030556

Larit F, 2018, PHYTOMEDICINE, V40, P27, DOI 10.1016/j.phymed.2017.12.032

Lee BK, 2017, INT J MOL SCI, V18, DOI 10.3390/ijms18071424

Lee JA, 2014, VET IMMUNOL IMMUNOP, V161, P193, DOI 10.1016/j.vetimm.2014.08.010

Li J, 2012, J PHARMACEUT BIOMED, V63, P120, DOI 10.1016/j.jpba.2012.01.027

Li K, 2018, ARAB J CHEM, V11, P159, DOI 10.1016/j.arabjc.2017.08.002

Li R, 2014, NEUROL SCI, V35, P1527, DOI 10.1007/s10072-014-1784-7

Li TF, 2019, NEUROCHEM INT, V129, DOI 10.1016/j.neuint.2019.104496

Li Y, 2019, MINI-REV MED CHEM, V19, P555, DOI 10.2174/1389557518666180424094821

Li ZP, 2019, BRAIN BEHAV IMMUN, V79, P228, DOI 10.1016/j.bbi.2019.02.005

Lin SR, 2017, INT J MOL SCI, V18, DOI 10.3390/ijms18071412

LIU YL, 1992, J NAT PROD, V55, P357, DOI 10.1021/np50081a012

Lotito SB, 2006, FREE RADICAL BIO MED, V41, P1727, DOI 10.1016/j.freeradbiomed.2006.04.033

Ma SW, 2019, LAT AM J PHARM, V38, P1618

Maan G, 2020, CURR TOP MED CHEM, V20, P1169, DOI 10.2174/1568026620666200416085330

Maher P, 2019, INT J MOL SCI, V20, DOI 10.3390/ijms20123056

Mamadalieva NZ, 2011, J PHARM PHARMACOL, V63, P1346, DOI 10.1111/j.2042-7158.2011.01336.x

Mangas-Sanjuan V, 2010, THER DELIV, V1, P535, DOI 10.4155/TDE.10.37

Mani R, 2018, PHYTOCHEMISTRY, V145, P187, DOI 10.1016/j.phytochem.2017.09.016

Mani R, 2017, BIOMED PHARMACOTHER, V88, P762, DOI 10.1016/j.biopha.2017.01.081

Martins IL, 2015, J MED CHEM, V58, P4250, DOI 10.1021/acs.jmedchem.5b00230

Maruhashi R, 2019, SCI REP-UK, V9, DOI 10.1038/s41598-019-50276-z

Mayer S, 2020, MOLECULES, V25, DOI 10.3390/molecules25040888

Meckes M, 1998, PHYTOMEDICINE, V5, P459, DOI 10.1016/S0944-7113(98)80042-1

MEDINA JH, 1990, BIOCHEM PHARMACOL, V40, P2227, DOI 10.1016/0006-2952(90)90716-X

Meng XD, 2017, CELL MOL IMMUNOL, V14, P702, DOI 10.1038/cmi.2015.107

Menon S, 2019, J AYURVEDA INTEGR ME, V10, P159, DOI 10.1016/j.jaim.2017.06.007

Miao R, 2019, SCI REP-UK, V9, DOI 10.1038/s41598-019-44773-4

Mikitsh JL, 2014, PERSPECT MED CHEM, V6, P11, DOI 10.4137/PMC.S13384

Moghadam ER, 2020, BIOMOLECULES, V10, DOI 10.3390/biom10101374

Mohammadinejad Sina, 2015, Asian Pac J Cancer Prev, V16, P3753

Mohos V, 2020, DRUG METAB DISPOS, V48, P1064, DOI 10.1124/dmd.120.000085

Mohos V, 2018, INT J MOL SCI, V19, DOI 10.3390/ijms19124073

Monteiro AFM, 2018, OXID MED CELL LONGEV, V2018, DOI 10.1155/2018/7912765

Moosavi F, 2016, DRUG DES DEV THER, V10, P23, DOI 10.2147/DDDT.S96936

Nabavi SF, 2015, NEUROCHEM INT, V90, P224, DOI 10.1016/j.neuint.2015.09.006

Nabavi SM, 2020, BIOTECHNOL ADV, V38, DOI 10.1016/j.biotechadv.2018.11.005

Navarro-Hortal MD, 2020, FOOD CHEM TOXICOL, V146, DOI 10.1016/j.fct.2020.111820

Naz S, 2019, LIFE SCI, V235, DOI 10.1016/j.lfs.2019.116797

Nday CM, 2019, HELL J NUCL MED, V22, P42

Nicolazzo JA, 2006, J PHARM PHARMACOL, V58, P281, DOI 10.1211/jpp.58.3.0001

Nigro A, 2018, PHARMACEUTICS, V10, DOI 10.3390/pharmaceutics10040250

Paladini AC, 1999, J PHARM PHARMACOL, V51, P519, DOI 10.1211/0022357991772790

Pandey PK, 2016, TISSUE BARRIERS, V4, DOI 10.1080/21688370.2015.1129476

Pandit R, 2020, ADV DRUG DELIVER REV, V165-166, P1, DOI [10.1016/j.addr.2019.11.009, 10.1080/2374068X.2020.1810946]

Pardridge WM, 2007, PHARM RES, V24, P1729, DOI 10.1007/s11095-007-9387-0

Pardridge WM, 2012, J CEREBR BLOOD F MET, V32, P1959, DOI 10.1038/jcbfm.2012.126

Paroha S, 2020, ENVIRON CHEM LETT, V18, P1767, DOI 10.1007/s10311-020-01038-1

Pasini F, 2013, FOOD CHEM, V141, P2802, DOI 10.1016/j.foodchem.2013.05.102

Peng SM, 2009, BIOORG MED CHEM LETT, V19, P1264, DOI 10.1016/j.bmcl.2008.12.116

Percário S, 2020, OXID MED CELL LONGEV, V2020, DOI 10.1155/2020/2360872

Perez Carlos A, 2008, Curr Bioact Compd, V4, P150, DOI 10.2174/157340708786305952

Pingili RB, 2019, CHEM-BIOL INTERACT, V308, P51, DOI 10.1016/j.cbi.2019.05.010

Pourbagher-Shahri AM, 2021, BIOMED PHARMACOTHER, V136, DOI 10.1016/j.biopha.2020.111214

Prajit R, 2020, NUTRIENTS, V12, DOI 10.3390/nu12041100

Radbeh Z, 2020, BIOMED PHARMACOTHER, V125, DOI 10.1016/j.biopha.2020.109906

Rahman HS, 2020, INT J NANOMED, V15, P2439, DOI 10.2147/IJN.S227805

Rahman MM, 2014, EVID-BASED COMPL ALT, V2014, DOI 10.1155/2014/958721

Ramos S, 2007, J NUTR BIOCHEM, V18, P427, DOI 10.1016/j.jnutbio.2006.11.004

Rashno M, 2020, PSYCHOPHARMACOLOGY, V237, P1607, DOI 10.1007/s00213-020-05482-3

Rashno M, 2019, LIFE SCI, V228, P285, DOI 10.1016/j.lfs.2019.05.007

Ríos JL, 2016, PLANTA MED, V82, P942, DOI 10.1055/s-0042-107081

Rosado-Ramos R, 2018, CURR PHARM DESIGN, V24, P2076, DOI 10.2174/1381612824666180629112148

Roshanravan B, 2023, ARCH PHYSIOL BIOCHEM, V129, P393, DOI 10.1080/13813455.2020.1828482

Ruan QW, 2018, PHARMACOL RES, V128, P345, DOI 10.1016/j.phrs.2017.08.010

Saarinen N, 2001, J STEROID BIOCHEM, V78, P231, DOI 10.1016/S0960-0760(01)00098-X

Sabzichi M, 2017, PROCESS BIOCHEM, V60, P84, DOI 10.1016/j.procbio.2017.05.024

Samarghandian S, 2003, NEUROSCIENCE, V116, P519, DOI 10.1016/S0306-4522(02)00712-1

Samarghandian S, 2017, DOSE-RESPONSE, V15, DOI 10.1177/1559325817711782

Samarghandian S, 2016, PHYTOTHER RES, V30, P1345, DOI 10.1002/ptr.5638

Samarghandian S, 2016, CAN J PHYSIOL PHARM, V94, P388, DOI 10.1139/cjpp-2014-0412

Samarghandian S, 2014, ANTI-CANCER AGENT ME, V14, P901, DOI 10.2174/1871520614666140209144042

Samarghandian S, 2011, CLINICS, V66, P1073, DOI 10.1590/S1807-59322011000600026

Sanadgol N, 2017, CURR DRUG TARGETS, V18, P1499, DOI 10.2174/1389450118666161108124414

Sarkaki A, 2019, LIFE SCI, V226, P202, DOI 10.1016/j.lfs.2019.04.027

Sela H, 2015, J NANOBIOTECHNOL, V13, DOI 10.1186/s12951-015-0133-1

Sharma P, 2019, NUTR NEUROSCI, V22, P569, DOI 10.1080/1028415X.2017.1418786

Shin JS, 1999, BIOORG MED CHEM LETT, V9, P869, DOI 10.1016/S0960-894X(99)00092-X

Shooshtari MK, 2020, METAB BRAIN DIS, V35, P401, DOI 10.1007/s11011-019-00527-9

Siddiqui Amir, 2018, Journal of Biologically Active Products from Nature, V8, P208, DOI 10.1080/22311866.2018.1498750

Sobocanec S, 2006, J AGR FOOD CHEM, V54, P8018, DOI 10.1021/jf0612023

Souza LC, 2015, PHARMACOL BIOCHEM BE, V134, P22, DOI 10.1016/j.pbb.2015.04.010

Spilsbury A, 2012, BIOCHEM BIOPH RES CO, V418, P578, DOI 10.1016/j.bbrc.2012.01.081

Sucher NJ, 2015, EPILEPSY BEHAV, V52, P308, DOI 10.1016/j.yebeh.2015.05.012

Sulaiman GM, 2011, FOOD CHEM TOXICOL, V49, P2415, DOI 10.1016/j.fct.2011.06.060

Tabatabaei SN, 2015, J CONTROL RELEASE, V206, P49, DOI 10.1016/j.jconrel.2015.02.027

Talebi M, 2019, 12 INT C LAB CLIN SC, P931

Talebi M., 2020, Specification of Drug Substances and Products, P459

Talebi M., 2020, CURR DRUG DISCOV TEC

Talebi M, 2021, INT PHARM ACTA

Talebi M, 2021, EXPERT REV NEUROTHER, V21, P625, DOI 10.1080/14737175.2021.1923479

Talebi M, 2021, CANCER CELL INT, V21, DOI 10.1186/s12935-021-01906-y

Talebi M, 2021, PHYTOTHER RES, V35, P3078, DOI 10.1002/ptr.7033

Talebi M, 2021, INT J BIOL MACROMOL, V172, P93, DOI 10.1016/j.ijbiomac.2021.01.042

Talebi M, 2021, BIOMED PHARMACOTHER, V133, DOI 10.1016/j.biopha.2020.111088

Talebi M, 2021, PHYTOTHER RES, V35, P1739, DOI 10.1002/ptr.6905

Talebi M, 2021, BIOINTERFACE RES APP, V11, P7468, DOI 10.33263/BRIAC111.74687492

Talebi M, 2020, BIOMED PHARMACOTHER, V130, DOI 10.1016/j.biopha.2020.110590

Tavakkoli A, 2019, PHYTOTHER RES, V33, P2256, DOI 10.1002/ptr.6427

Thangarajan S, 2016, BIOMED PHARMACOTHER, V84, P514, DOI 10.1016/j.biopha.2016.09.070

Tobin PJ, 2006, CANCER CHEMOTH PHARM, V57, P309, DOI 10.1007/s00280-005-0053-0

Toropova AP, 2018, CURR NEUROPHARMACOL, V16, P769, DOI 10.2174/1570159X15666171016163951

Troncoso-Escudero P, 2018, FRONT NEUROL, V9, DOI 10.3389/fneur.2018.00860

Tsuji PA, 2008, CHEM-BIOL INTERACT, V171, P37, DOI 10.1016/j.cbi.2007.08.007

Vedagiri A, 2016, NEUROPEPTIDES, V58, P111, DOI 10.1016/j.npep.2016.03.002

Walle T, 2004, FREE RADICAL BIO MED, V36, P829, DOI 10.1016/j.freeradbiomed.2004.01.002

Walle T, 2001, BRIT J CLIN PHARMACO, V51, P143, DOI 10.1111/j.1365-2125.2001.01317.x

Wang MJ, 2016, CHIN MED-UK, V11, DOI 10.1186/s13020-016-0074-0

Wang QQ, 2014, BIOORGAN MED CHEM, V22, P1515, DOI 10.1016/j.bmc.2014.01.038

Wang X, 2018, CURR PHARM DESIGN, V24, P2583, DOI 10.2174/1381612824666180803113106

Wang Y, 2019, SCI REP-UK, V9, DOI 10.1038/s41598-019-42272-0

Williams CA, 1997, PHYTOCHEMISTRY, V46, P1349, DOI 10.1016/S0031-9422(97)00514-1

WOLFMAN C, 1994, PHARMACOL BIOCHEM BE, V47, P1, DOI 10.1016/0091-3057(94)90103-1

Xiao J, 2014, NEUROSCIENCE, V262, P156, DOI 10.1016/j.neuroscience.2014.01.004

Xing HJ, 2017, CURR PHARM DESIGN, V23, P6705, DOI 10.2174/1381612824666171128105853

Xu SL, 2013, BIRTH DEFECTS RES C, V99, P292, DOI 10.1002/bdrc.21054

Yan RY, 2011, FITOTERAPIA, V82, P841, DOI 10.1016/j.fitote.2011.04.006

Yao W, 2021, DRUG CHEM TOXICOL, V44, P601, DOI 10.1080/01480545.2019.1687510

Yao Y, 2014, INT J MOL SCI, V15, P20913, DOI 10.3390/ijms151120913

Yazdani E, 2019, BIOINTERFACE RES APP, V9, P4517, DOI 10.33263/BRIAC96.517521

Zabaleta ME, 2020, FOOD RES INT, V137, DOI 10.1016/j.foodres.2020.109623

Zeng ZY, 2021, CHEMISTRYSELECT, V6, P415, DOI 10.1002/slct.202004358

Zhang J, 2015, INT J POLYM SCI, V2015, DOI 10.1155/2015/529235

Zhang P, 2017, REV NEUROSCIENCE, V28, P87, DOI 10.1515/revneuro-2016-0053

Zhang T, 2004, BIOORGAN MED CHEM, V12, P6097, DOI 10.1016/j.bmc.2004.09.013

Zhang Y, 2021, J BIOCHEM MOL TOXIC, V35, DOI 10.1002/jbt.22634

Zhang ZJ, 2012, CELL MOL NEUROBIOL, V32, P27, DOI 10.1007/s10571-011-9731-0

Zhang ZJ, 2015, FREE RADICAL BIO MED, V84, P331, DOI 10.1016/j.freeradbiomed.2015.02.030

Zhao J, 2020, ACS NANO, V14, P1045, DOI 10.1021/acsnano.9b08595

Zhao TT, 2019, J PHARM PHARMACOL, V71, P1353, DOI 10.1111/jphp.13129

Zheng H, 2014, EUR J PHARM BIOPHARM, V87, P454, DOI 10.1016/j.ejpb.2014.03.011

Zhu HL, 2014, EPILEPSIA, V55, P3, DOI 10.1111/epi.12463

Zhu L, 2016, PHARM BIOL, V54, P2692, DOI 10.1080/13880209.2016.1179334

Zou X.Q, 2012, NITRIC OXIDE DONATIN

Zou XQ, 2010, BIOORGAN MED CHEM, V18, P3020, DOI 10.1016/j.bmc.2010.03.056

NR 233

TC 29

Z9 31

U1 1

U2 14

PU ELSEVIER FRANCE-EDITIONS SCIENTIFIQUES MEDICALES ELSEVIER

PI ISSY-LES-MOULINEAUX

PA 65 RUE CAMILLE DESMOULINS, CS50083, 92442 ISSY-LES-MOULINEAUX, FRANCE

SN 0753-3322

EI 1950-6007

J9 BIOMED PHARMACOTHER

JI Biomed. Pharmacother.

PD SEP

PY 2021

VL 141

AR 111906

DI 10.1016/j.biopha.2021.111906

EA JUL 2021

PG 13

WC Medicine, Research & Experimental; Pharmacology & Pharmacy

WE Science Citation Index Expanded (SCI-EXPANDED)

SC Research & Experimental Medicine; Pharmacology & Pharmacy

GA UO4ZO

UT WOS:000694704500006

PM 34328092

OA gold

DA 2024-08-12

ER

PT J

AU Su, Q

Ng, WL

Goh, SY

Gulam, MY

Wang, LF

Tan, EK

Ahn, M

Chao, YX

AF Su, Qi

Ng, Wei Lun

Goh, Suh Yee

Gulam, Muhammad Yaaseen

Wang, Lin-Fa

Tan, Eng-King

Ahn, Matae

Chao, Yin-Xia

TI Targeting the inflammasome in Parkinson's disease

SO FRONTIERS IN AGING NEUROSCIENCE

LA English

DT Article

DE Parkinson's disease; neuroinflammation; inflammasome; NLRP3; inhibitor

ID NLRP3 INFLAMMASOME; MOUSE MODEL; ALPHA; INHIBITOR; MPTP;

NEUROINFLAMMATION; MUTATIONS; DOPAMINE; NEURODEGENERATION;

SYNUCLEINOPATHY

AB Parkinson's disease (PD) is one of the most common neurodegenerative disease in which neuroinflammation plays pivotal roles. An important mechanism of neuroinflammation is the NLRP3 inflammasome activation that has been implicated in PD pathogenesis. In this perspective, we will discuss the relationship of some key PD-associated proteins including alpha-synuclein and Parkin and their contribution to inflammasome activation. We will also review promising inhibitors of NLRP3 inflammasome pathway that have potential as novel PD therapeutics. Finally, we will provide a summary of current and potential in vitro and in vivo models that are available for therapeutic discovery and development.

C1 [Su, Qi; Ng, Wei Lun; Wang, Lin-Fa; Ahn, Matae] Duke NUS Med Sch, Programme Emerging Infect Dis, Singapore, Singapore.

[Goh, Suh Yee; Gulam, Muhammad Yaaseen; Tan, Eng-King; Chao, Yin-Xia] Singapore Gen Hosp, Dept Neurol, Singapore, Singapore.

[Goh, Suh Yee; Gulam, Muhammad Yaaseen; Tan, Eng-King; Chao, Yin-Xia] Natl Neurosci Inst, Dept Res, Singapore, Singapore.

[Tan, Eng-King; Chao, Yin-Xia] Duke NUS Med Sch, Neurosci & Behav Disorders Program, Singapore, Singapore.

C3 National University of Singapore; Singapore General Hospital; National

Neuroscience Institute (NNI); National University of Singapore

RP Ahn, M (corresponding author), Duke NUS Med Sch, Programme Emerging Infect Dis, Singapore, Singapore.; Chao, YX (corresponding author), Singapore Gen Hosp, Dept Neurol, Singapore, Singapore.; Chao, YX (corresponding author), Natl Neurosci Inst, Dept Res, Singapore, Singapore.; Chao, YX (corresponding author), Duke NUS Med Sch, Neurosci & Behav Disorders Program, Singapore, Singapore.

EM ahn.matae11@u.duke.nus.edu; chao.yinxia@singhealth.com.sg

RI wang, lin/GSE-3040-2022

OI Ng, Wei Lun/0000-0003-1533-9325

FU National Medical Research Council [MOH-OFIRG19may-0011,

MOH-OFIRG19nov-0050, MOH-OFLCG-002]; Ministry of Education

[MOE2019-T2-2-130]; Michael J. Fox Foundation [MJFF-006971]

FX This work was funded by grants from National Medical Research Council

(MOH-OFIRG19may-0011 and MOH-OFIRG19nov-0050, STAR award, CSA award, and

Parkinson's disease Large Collaborative Grant MOH-OFLCG-002), Ministry

of Education (MOE2019-T2-2-130), and Michael J. Fox Foundation

(MJFF-006971).

CR Anderson FL, 2021, NPJ PARKINSONS DIS, V7, DOI 10.1038/s41531-020-00147-6

Armstrong MJ, 2020, JAMA-J AM MED ASSOC, V323, P548, DOI 10.1001/jama.2019.22360

Badanjak K, 2021, FRONT CELL DEV BIOL, V9, DOI 10.3389/fcell.2021.740758

BALLARD PA, 1985, NEUROLOGY, V35, P949, DOI 10.1212/WNL.35.7.949

Bassil F, 2016, P NATL ACAD SCI USA, V113, P9593, DOI 10.1073/pnas.1609291113

Bonifati V, 2003, SCIENCE, V299, P256, DOI 10.1126/science.1077209

Brodacki B, 2008, NEUROSCI LETT, V441, P158, DOI 10.1016/j.neulet.2008.06.040

Chen J, 2021, NEURAL REGEN RES, V16, P1836, DOI 10.4103/1673-5374.306096

Chen XH, 2021, J INT MED RES, V49, DOI 10.1177/0300060521992981

Chen Y, 2021, CELL MOL IMMUNOL, V18, P1425, DOI 10.1038/s41423-021-00683-y

Chia SJ, 2020, INT J MOL SCI, V21, DOI 10.3390/ijms21072464

Chlebanowska P, 2020, INT J MOL SCI, V21, DOI 10.3390/ijms21030694

Ferrari CC, 2006, NEUROBIOL DIS, V24, P183, DOI 10.1016/j.nbd.2006.06.013

Codolo G, 2013, PLOS ONE, V8, DOI 10.1371/journal.pone.0055375

Coll RC, 2019, NAT CHEM BIOL, V15, P556, DOI 10.1038/s41589-019-0277-7

Coll RC, 2015, NAT MED, V21, P248, DOI 10.1038/nm.3806

Cristóvao AC, 2020, EUR J NEUROSCI, V52, P3242, DOI 10.1111/ejn.14683

Denes A, 2015, P NATL ACAD SCI USA, V112, P4050, DOI 10.1073/pnas.1419090112

Deng H, 2018, AGEING RES REV, V42, P72, DOI 10.1016/j.arr.2017.12.007

Devine MJ, 2011, NAT COMMUN, V2, DOI 10.1038/ncomms1453

Dick MS, 2016, NAT COMMUN, V7, DOI 10.1038/ncomms11929

Domico LM, 2006, NEUROTOXICOLOGY, V27, P816, DOI 10.1016/j.neuro.2006.07.009

Dovero S, 2016, SYNAPSE, V70, P49, DOI 10.1002/syn.21875

Fahn S, 2004, NEW ENGL J MED, V351, P2498

Fan Z, 2020, J NEUROINFLAMM, V17, DOI 10.1186/s12974-019-1670-6

Fan Z, 2017, J NEUROINFLAMM, V14, DOI 10.1186/s12974-017-1036-x

Ferrari CC, 2011, PARKINSONS DIS-US, V2011, DOI 10.4061/2011/436813

Flores J, 2020, NAT COMMUN, V11, DOI 10.1038/s41467-020-18405-9

Freundt EC, 2012, ANN NEUROL, V72, P517, DOI 10.1002/ana.23747

Galet B, 2020, FRONT NEUROL, V11, DOI 10.3389/fneur.2020.01005

Gaven F, 2014, JOVE-J VIS EXP, DOI 10.3791/51751

Gong Z, 2018, J NEUROINFLAMM, V15, DOI 10.1186/s12974-018-1282-6

Gordon R, 2018, SCI TRANSL MED, V10, DOI 10.1126/scitranslmed.aah4066

Gorell JM, 1998, NEUROLOGY, V50, P1346, DOI 10.1212/WNL.50.5.1346

Gorell JM, 1997, NEUROLOGY, V48, P650, DOI 10.1212/WNL.48.3.650

Groiss S J, 2009, Ther Adv Neurol Disord, V2, P20, DOI 10.1177/1756285609339382

Guo CQ, 2017, ACS CHEM NEUROSCI, V8, P2194, DOI 10.1021/acschemneuro.7b00124

Hammond TR, 2019, IMMUNITY, V50, P253, DOI 10.1016/j.immuni.2018.11.004

Han CY, 2019, PHYTOTHER RES, V33, P2288, DOI 10.1002/ptr.6388

Han XJ, 2019, AUTOPHAGY, V15, P1860, DOI 10.1080/15548627.2019.1596481

Harms AS, 2013, J NEUROSCI, V33, P9592, DOI 10.1523/JNEUROSCI.5610-12.2013

He HB, 2018, NAT COMMUN, V9, DOI 10.1038/s41467-018-04947-6

Howe K, 2016, OPEN BIOL, V6, DOI 10.1098/rsob.160009

Huang SX, 2021, J NEUROIMMUNOL, V354, DOI 10.1016/j.jneuroim.2021.577543

Innos J, 2021, CHEM RES TOXICOL, V34, P1223, DOI 10.1021/acs.chemrestox.0c00522

Jiang H, 2017, J EXP MED, V214, P3219, DOI 10.1084/jem.20171419

Jiang W, 2017, J NEUROINFLAMM, V14, DOI 10.1186/s12974-017-0980-9

Jo J, 2016, CELL STEM CELL, V19, P248, DOI 10.1016/j.stem.2016.07.005

Juliana C, 2010, J BIOL CHEM, V285, P9792, DOI 10.1074/jbc.M109.082305

Kalia SK, 2013, CURR OPIN NEUROL, V26, P374, DOI 10.1097/WCO.0b013e3283632d08

Kelly R, 2020, MOLECULES, V25, DOI 10.3390/molecules25030453

Keshavarzian A, 2015, MOVEMENT DISORD, V30, P1351, DOI 10.1002/mds.26307

Kin K, 2019, INT J MOL SCI, V20, DOI 10.3390/ijms20215402

Kitada T, 1998, NATURE, V392, P605, DOI 10.1038/33416

Koprich JB, 2017, NAT REV NEUROSCI, V18, P515, DOI 10.1038/nrn.2017.75

Kouli A., 2018, Exon Publications, P3, DOI [DOI 10.15586/CODONPUBLICATIONS.PARKINSONSDISEASE.2018.CH1, 10.17305/bjbms.2020.5181, 10.15586/codonpublications.parkinsonsdisease.2018.ch1]

Lee E, 2019, CELL DEATH DIFFER, V26, P213, DOI 10.1038/s41418-018-0124-5

Li DS, 2018, HUM CELL, V31, P106, DOI 10.1007/s13577-017-0187-5

Lin CH, 2017, J NEUROL NEUROSUR PS, V88, P818, DOI 10.1136/jnnp-2016-314857

Lonnemann N, 2020, P NATL ACAD SCI USA, V117, P32145, DOI 10.1073/pnas.2009680117

Lotharius J, 2002, NAT REV NEUROSCI, V3, P932, DOI 10.1038/nrn983

Luczynski P, 2016, INT J NEUROPSYCHOPH, V19, DOI 10.1093/ijnp/pyw020

Malagelada C, 2008, PARKINSON'S DISEASE: MOLECULAR AND THERAPEUTIC INSIGHTS FROM MODEL SYSTEMS, P375, DOI 10.1016/B978-0-12-374028-1.00029-4

Mangan MSJ, 2018, NAT REV DRUG DISCOV, V17, P588, DOI [10.1038/nrd.2018.97, 10.1038/nrd.2018.149]

Marchetti C, 2018, P NATL ACAD SCI USA, V115, pE1530, DOI 10.1073/pnas.1716095115

Martínez-Morales PL, 2012, STEM CELLS INT, V2012, DOI 10.1155/2012/980941

Mccoy MK, 2006, J NEUROSCI, V26, P9365, DOI 10.1523/JNEUROSCI.1504-06.2006

MCGEER PL, 1988, NEUROLOGY, V38, P1285, DOI 10.1212/WNL.38.8.1285

McLean PJ, 2001, NEUROSCIENCE, V104, P901, DOI 10.1016/S0306-4522(01)00113-0

Mertens J, 2018, ANNU REV GENET, V52, P271, DOI 10.1146/annurev-genet-120417-031534

Mo YS, 2018, EVID-BASED COMPL ALT, V2018, DOI 10.1155/2018/3571604

Mouton-Liger F, 2018, GLIA, V66, P1736, DOI 10.1002/glia.23337

Murray KN, 2015, FRONT CELL NEUROSCI, V9, DOI 10.3389/fncel.2015.00018

Ormel PR, 2018, NAT COMMUN, V9, DOI 10.1038/s41467-018-06684-2

Ou ZJ, 2021, FRONT PUBLIC HEALTH, V9, DOI 10.3389/fpubh.2021.776847

Panicker N, 2022, NEURON, V110, P2422, DOI 10.1016/j.neuron.2022.05.009

Pellegrini C, 2020, PROG NEUROBIOL, V191, DOI 10.1016/j.pneurobio.2020.101806

Pike AF, 2021, GLIA, V69, P1413, DOI 10.1002/glia.23970

Proell M, 2008, PLOS ONE, V3, DOI 10.1371/journal.pone.0002119

Roca V, 2011, PARKINSONS DIS-US, V2011, DOI 10.4061/2011/931572

Rothhammer V, 2018, NATURE, V557, P724, DOI 10.1038/s41586-018-0119-x

Rui WJ, 2020, INT J NEUROPSYCHOPH, V23, P762, DOI 10.1093/ijnp/pyaa060

Rutsch A, 2020, FRONT IMMUNOL, V11, DOI 10.3389/fimmu.2020.604179

Sánchez-Fernández A, 2019, FRONT IMMUNOL, V10, DOI 10.3389/fimmu.2019.02578

SAUER H, 1994, NEUROSCIENCE, V59, P401, DOI 10.1016/0306-4522(94)90605-X

Sawada M, 2006, J NEURAL TRANSM-SUPP, P373

Scheperjans F, 2015, MOVEMENT DISORD, V30, P350, DOI 10.1002/mds.26069

Schrag A, 2015, LANCET NEUROL, V14, P57, DOI 10.1016/S1474-4422(14)70287-X

Sherer TB, 2002, J NEUROSCI, V22, P7006

Shi N, 2017, MILITARY MED RES, V4, DOI 10.1186/s40779-017-0122-9

Skibinski Gaia, 2011, Int J High Throughput Screen, V2011, P15

Strässler ET, 2018, FRONT CARDIOVASC MED, V5, DOI 10.3389/fcvm.2018.00004

Sun R, 2020, J NEUROINFLAMM, V17, DOI 10.1186/s12974-020-01988-x

Tabrizi SJ, 2000, HUM MOL GENET, V9, P2683, DOI 10.1093/hmg/9.18.2683

Tan EK, 2020, NAT REV NEUROL, V16, P303, DOI 10.1038/s41582-020-0344-4

Tan JSY, 2020, INT J MOL SCI, V21, DOI 10.3390/ijms21239302

Trudler D, 2021, P NATL ACAD SCI USA, V118, DOI 10.1073/pnas.2025847118

Valente EM, 2004, SCIENCE, V304, P1158, DOI 10.1126/science.1096284

von Herrmann KM, 2018, NPJ PARKINSON DIS, V4, DOI 10.1038/s41531-018-0061-5

Wang QQ, 2015, TRANSL NEURODEGENER, V4, DOI 10.1186/s40035-015-0042-0

Wang W, 2016, P NATL ACAD SCI USA, V113, P9587, DOI 10.1073/pnas.1610099113

Williams-Gray C.H., 2016, MEDICINE, V44, P542, DOI [DOI 10.1016/J.MPMED.2016.06.001, 10.1016/j.mpmed.2016.06.001]

Yadav SK, 2021, J CLIN MED, V10, DOI 10.3390/jcm10040857

Zhang LP, 2019, J PARKINSON DIS, V9, P157, DOI 10.3233/JPD-181503

Zhang XM, 2014, ACTA PHARMACOL SIN, V35, P945, DOI 10.1038/aps.2014.36

Zhang X, 2020, AGING-US, V12, P9405, DOI 10.18632/aging.103215

Zhang Y, 2019, MICROBIOME, V7, DOI 10.1186/s40168-019-0733-3

Zhao XJ, 2022, J NEUROTRAUM, V39, P530, DOI 10.1089/neu.2021.0466

Zheng DP, 2020, CELL DISCOV, V6, DOI 10.1038/s41421-020-0167-x

Zhou Y, 2016, MOL NEURODEGENER, V11, DOI 10.1186/s13024-016-0094-3

NR 110

TC 8

Z9 8

U1 1

U2 17

PU FRONTIERS MEDIA SA

PI LAUSANNE

PA AVENUE DU TRIBUNAL FEDERAL 34, LAUSANNE, CH-1015, SWITZERLAND

SN 1663-4365

J9 FRONT AGING NEUROSCI

JI Front. Aging Neurosci.

PD OCT 12

PY 2022

VL 14

AR 957705

DI 10.3389/fnagi.2022.957705

PG 11

WC Geriatrics & Gerontology; Neurosciences

WE Science Citation Index Expanded (SCI-EXPANDED)

SC Geriatrics & Gerontology; Neurosciences & Neurology

GA 5U2HY

UT WOS:000876375300001

PM 36313019

OA Green Published, gold

DA 2024-08-12

ER

PT J

AU Zehravi, M

Kabir, J

Akter, R

Malik, S

Ashraf, GM

Tagde, P

Ramproshad, S

Mondal, B

Rahman, MH

Mohan, AG

Cavalu, S

AF Zehravi, Mehrukh

Kabir, Janisa

Akter, Rokeya

Malik, Sumira

Ashraf, Ghulam Md.

Tagde, Priti

Ramproshad, Sarker

Mondal, Banani

Rahman, Md. Habibur

Mohan, Aurel George

Cavalu, Simona

TI A Prospective Viewpoint on Neurological Diseases and Their Biomarkers

SO MOLECULES

LA English

DT Review

DE neurological disorder; neuroinflammation; proteinopathies; biomarkers

ID BLOOD-BRAIN-BARRIER; C-REACTIVE PROTEIN; MULTIPLE-SCLEROSIS;

alzheimer's disease; T-CELLS; NEUROMYELITIS-OPTICA; DIAGNOSTIC-CRITERIA;

INFLAMMATION; NECROPTOSIS; AUTOANTIBODIES

AB neurodegenerative disease (NDDs) are disorders that affect both the central and peripheral nervous systems. To name a few causes, NDDs can be caused by ischemia, oxidative and endoplasmic reticulum (ER) cell stress, inflammation, abnormal protein deposition in neural tissue, autoimmune-mediated neuron loss, and viral or prion infections. These conditions include Alzheimer's disease (AD), Lewy body dementia (LBD), and Parkinson's disease (PD). The formation of beta-sheet-rich aggregates of intra- or extracellular proteins in the CNS hallmarks all neurodegenerative proteinopathies. In systemic lupus erythematosus (SLE), numerous organs, including the central nervous system (CNS), are affected. However, the inflammatory process is linked to several neurodegenerative pathways that are linked to depression because of NDDs. Pro-inflammatory signals activated by aging may increase vulnerability to neuropsychiatric disorders. Viruses may increase macrophages and CCR5+ T cells within the CNS during dementia formation and progression. Unlike medical symptoms, which are just signs of a patient's health as expressed and perceived, biomarkers are reproducible and quantitative. Therefore, this current review will highlight and summarize the neurological disorders and their biomarkers.

C1 [Zehravi, Mehrukh] Prince Sattam Bin Abdul Aziz Univ, Dept Clin Pharm, Girls Sect, Alkharj 11942, Saudi Arabia.

[Kabir, Janisa] China Pharmaceut Univ, Key Lab Modern Chinese Med, Nanjing 210009, China.

[Akter, Rokeya; Rahman, Md. Habibur] Yonsei Univ, Wonju Coll Med, Dept Global Med Sci, Wonju 26426, Gangwon Do, South Korea.

[Malik, Sumira] Amity Univ Jharkhand, Amity Inst Biotechnol, Ranchi, Bihar, India.

[Ashraf, Ghulam Md.] King Abdulaziz Univ, Pre Clin Res Unit, King Fahd Med Res Ctr, Jeddah 21589, Saudi Arabia.

[Ashraf, Ghulam Md.] King Abdulaziz Univ, Fac Appl Med Sci, Dept Med Lab Sci, Jeddah 21589, Saudi Arabia.

[Tagde, Priti] Amity Univ, Amity Inst Pharm, Noida 201301, India.

[Ramproshad, Sarker; Mondal, Banani] Ranada Prasad Shaha Univ, Dept Pharm, Narayanganj 1400, Bangladesh.

[Mohan, Aurel George; Cavalu, Simona] Univ Oradea, Fac Med & Pharm, P Ta 1 Decembrie 10, Oradea 410087, Romania.

C3 Prince Sattam Bin Abdulaziz University; China Pharmaceutical University;

Yonsei University; King Abdulaziz University; King Abdulaziz University;

Amity University Noida; University of Oradea

RP Zehravi, M (corresponding author), Prince Sattam Bin Abdul Aziz Univ, Dept Clin Pharm, Girls Sect, Alkharj 11942, Saudi Arabia.; Rahman, MH (corresponding author), Yonsei Univ, Wonju Coll Med, Dept Global Med Sci, Wonju 26426, Gangwon Do, South Korea.; Cavalu, S (corresponding author), Univ Oradea, Fac Med & Pharm, P Ta 1 Decembrie 10, Oradea 410087, Romania.

EM mahrukh.zehravi@hotmail.com; janisa_kabir@yahoo.com;

rokeyahabib94@gmail.com; smalik@rnc.amity.edu; ashraf.gm@gmail.com;

tagde_priti@rediffmail.com; ramproshad131135@gmail.com;

banani091110@gmail.com; pharmacisthabib@gmail.com; mohanaurel@yahoo.com;

simona.cavalu@gmail.com

RI Zehravi, Mehrukh/CAI-9639-2022; Ashraf, Ghulam Md/H-9485-2012; CAVALU,

SIMONA Daniela/AAV-5882-2021; Kabir, Janisa/GXG-9687-2022

OI Zehravi, Mehrukh/0000-0003-3156-2826; Ashraf, Ghulam

Md/0000-0002-9820-2078; CAVALU, SIMONA Daniela/0000-0001-7810-8925;

Kabir, Janisa/0000-0003-0942-9877; Malik, Sumira/0000-0001-5077-1493;

Ramproshad, Sarker/0000-0001-9277-2451

FU University of Oradea

FX The authors acknowledge the financial support provided by University of

Oradea for APC.

CR Abramov AY, 2017, BIOCHEM SOC T, V45, P1025, DOI 10.1042/BST20170024

Adeola HA, 2021, BIOMED PHARMACOTHER, V141, DOI 10.1016/j.biopha.2021.111809

Akter R, 2021, CNS NEUROL DISORD-DR, V20, P430, DOI 10.2174/1871527320666210218084444

Akter R, 2021, CURR PHARM DESIGN, V27, P345, DOI 10.2174/1381612826666201102102810

Al-Obaidi MMJ, 2018, CELL MOL NEUROBIOL, V38, P1349, DOI 10.1007/s10571-018-0609-2

Allison DJ, 2014, J NEUROINFLAMM, V11, DOI 10.1186/s12974-014-0151-1

[Anonymous], 2010, YEARB CRIT CARE MED, DOI DOI 10.1016/S0734-3299(10)79402-8

Arendt T, 1998, J NEURAL TRANSM, V105, P949, DOI 10.1007/s007020050104

Arrambide G, 2019, MULT SCLER J, V25, P1690, DOI 10.1177/1352458519846103

Arya A, 2021, BIOMOLECULES, V11, DOI 10.3390/biom11030350

Bachurin SO, 2018, PHARMACOL RES, V129, P216, DOI 10.1016/j.phrs.2017.11.021

Bae EJ, 2012, J NEUROSCI, V32, P13454, DOI 10.1523/JNEUROSCI.1292-12.2012

Bajgai J, 2021, CURR PHARM DESIGN, V27, P737, DOI 10.2174/1381612826666200925124235

Balin BJ, 2008, J ALZHEIMERS DIS, V13, P371

Bauer PO, 2010, NAT BIOTECHNOL, V28, P256, DOI 10.1038/nbt.1608

Baune BT, 2012, PSYCHONEUROENDOCRINO, V37, P1521, DOI 10.1016/j.psyneuen.2012.02.006

Bayer TA, 2015, EUR NEUROPSYCHOPHARM, V25, P713, DOI 10.1016/j.euroneuro.2013.03.007

Becker J., 2017, NEUROL INT OPEN, V01, pE256, DOI [10.1055/s-0043-115429, DOI 10.1055/S-0043-115429]

Bhattacharya T, 2022, MATERIALS, V15, DOI 10.3390/ma15030804

Bhattacharya T, 2021, EXP GERONTOL, V150, DOI 10.1016/j.exger.2021.111352

Boiko DI, 2022, ENVIRON SCI POLLUT R, V29, P28062, DOI 10.1007/s11356-021-18384-4

Bonnan M, 2015, MULT SCLER INT, V2015, DOI 10.1155/2015/296184

Bravo-Zehnder M, 2015, ARTHRITIS RHEUMATOL, V67, P204, DOI 10.1002/art.38900

Brew BJ, 2009, J NEUROIMMUNE PHARM, V4, P163, DOI 10.1007/s11481-008-9143-1

Bruunsgaard H, 2001, CURR OPIN HEMATOL, V8, P131, DOI 10.1097/00062752-200105000-00001

Bullmore ET, 2012, NAT REV NEUROSCI, V13, P336, DOI 10.1038/nrn3214

Bullmore ET, 2009, NAT REV NEUROSCI, V10, P186, DOI 10.1038/nrn2575

Capuron L, 2011, PSYCHOL MED, V41, P1517, DOI 10.1017/S0033291710001984

Capuron L, 2008, BIOL PSYCHIAT, V64, P896, DOI 10.1016/j.biopsych.2008.05.019

Cavalu S, 2007, J OPTOELECTRON ADV M, V9, P3297

Cavalu S, 2020, MATERIALS, V13, DOI 10.3390/ma13235391

Chang JR, 2011, J BIOL CHEM, V286, P41125, DOI 10.1074/jbc.M111.268466

Chen GF, 2017, ACTA PHARMACOL SIN, V38, P1205, DOI 10.1038/aps.2017.28

Chen LN, 2011, PLOS ONE, V6, DOI 10.1371/journal.pone.0025994

Chhor V, 2013, BRAIN BEHAV IMMUN, V32, P70, DOI 10.1016/j.bbi.2013.02.005

Chiti F, 2017, ANNU REV BIOCHEM, V86, P27, DOI 10.1146/annurev-biochem-061516-045115

Chitnis T, 2017, J CLIN INVEST, V127, P3577, DOI 10.1172/JCI90609

Chiu IM, 2013, NATURE, V501, P52, DOI 10.1038/nature12479

Cohen AD, 2019, MOL CELL NEUROSCI, V97, P3, DOI 10.1016/j.mcn.2018.12.004

Compston A, 2008, LANCET, V372, P1502, DOI 10.1016/S0140-6736(08)61620-7

Dalle S, 2017, FRONT PHYSIOL, V8, DOI 10.3389/fphys.2017.01045

De Rosa V, 2015, NAT IMMUNOL, V16, P1174, DOI 10.1038/ni.3269

Deleidi Michela, 2012, Int J Alzheimers Dis, V2012, P391438, DOI 10.1155/2012/391438

DeTure MA, 2019, MOL NEURODEGENER, V14, DOI 10.1186/s13024-019-0333-5

Deutschman CS, 2014, IMMUNITY, V40, P464, DOI 10.1016/j.immuni.2014.04.001

Di Napoli M, 2014, STROKE, V45, P59, DOI 10.1161/STROKEAHA.113.001721

Di Napoli M, 2012, NEUROLOGY, V79, P690, DOI 10.1212/WNL.0b013e318264e3be

Dinan TG, 2015, CURR OPIN CLIN NUTR, V18, P552, DOI 10.1097/MCO.0000000000000221

Dobson R, 2019, EUR J NEUROL, V26, P27, DOI 10.1111/ene.13819

Dubois B, 2014, LANCET NEUROL, V13, P614, DOI 10.1016/S1474-4422(14)70090-0

Dugger BN, 2017, CSH PERSPECT BIOL, V9, DOI 10.1101/cshperspect.a028035

El Aidy S, 2014, FRONT MICROBIOL, V5, DOI 10.3389/fmicb.2014.00146

Fahn S, 2018, PARKINSONISM RELAT D, V46, pS1, DOI 10.1016/j.parkreldis.2017.07.020

Fattakhov N, 2021, VIRUSES-BASEL, V13, DOI 10.3390/v13091883

Faust TW, 2010, P NATL ACAD SCI USA, V107, P18569, DOI 10.1073/pnas.1006980107

Fletcher JM, 2010, CLIN EXP IMMUNOL, V162, P1, DOI 10.1111/j.1365-2249.2010.04143.x

Franceschi C, 2018, NAT REV ENDOCRINOL, V14, P576, DOI 10.1038/s41574-018-0059-4

Fünfschilling U, 2012, NATURE, V485, P517, DOI 10.1038/nature11007

Ghavami S, 2014, PROG NEUROBIOL, V112, P24, DOI 10.1016/j.pneurobio.2013.10.004

Giridharan VV, 2019, FRONT AGING NEUROSCI, V11, DOI 10.3389/fnagi.2019.00122

Golde TE, 2016, NAT REV NEUROL, V12, P433, DOI 10.1038/nrneurol.2016.105

Golde TE, 2009, ALZHEIMERS RES THER, V1, DOI 10.1186/alzrt5

Goossens GH, 2011, CIRCULATION, V124, P67, DOI 10.1161/CIRCULATIONAHA.111.027813

Grygiel-Gorniak Bogna, 2018, Reumatologia (Warsaw), V56, P243, DOI 10.5114/reum.2018.77976

Hampel H, 2018, NAT REV NEUROL, V14, P639, DOI 10.1038/s41582-018-0079-7

Hansson O, 2021, NAT MED, V27, P954, DOI 10.1038/s41591-021-01382-x

Holmes C, 2009, NEUROLOGY, V73, P768, DOI 10.1212/WNL.0b013e3181b6bb95

Howcroft TK, 2013, AGING-US, V5, P84, DOI 10.18632/aging.100531

Huang Y.-Y.Y., 2018, ACS CHEM NEUROSCI, V13

Irwin DJ, 2016, PARKINSONISM RELAT D, V22, pS29, DOI 10.1016/j.parkreldis.2015.09.020

Ito Y, 2016, SCIENCE, V353, P603, DOI 10.1126/science.aaf6803

Jack CR, 2018, ALZHEIMERS DEMENT, V14, P535, DOI 10.1016/j.jalz.2018.02.018

Jack CR, 2013, NEURON, V80, P1347, DOI 10.1016/j.neuron.2013.12.003

Jarius S, 2018, J NEUROINFLAMM, V15, DOI 10.1186/s12974-018-1144-2

Jellinger KA, 2009, J NEURAL TRANSM, V116, P1111, DOI 10.1007/s00702-009-0240-y

Jeltsch-David H, 2014, NAT REV NEUROL, V10, P579, DOI 10.1038/nrneurol.2014.148

Ji H, 2014, MUCOSAL IMMUNOL, V7, P335, DOI 10.1038/mi.2013.52

Johnson KA, 2013, ALZHEIMERS DEMENT, V9, pE1, DOI [10.1016/j.jalz.2012.10.007, 10.1016/j.jalz.2013.01.002, 10.1016/j.jalz.2013.06.001]

Jung YJ, 2019, FRONT CELL DEV BIOL, V7, DOI 10.3389/fcell.2019.00313

Karthika C, 2021, BIOMED PHARMACOTHER, V140, DOI 10.1016/j.biopha.2021.111704

Klunk WE, 2015, ALZHEIMERS DEMENT, V11, P1, DOI 10.1016/j.jalz.2014.07.003

Knopman DS, 2021, NAT REV DIS PRIMERS, V7, DOI 10.1038/s41572-021-00269-y

Kooij G, 2014, ACTA NEUROPATHOL, V127, P699, DOI 10.1007/s00401-014-1244-8

Kovacs GG, 2017, J NEUROPATH EXP NEUR, V76, P270, DOI 10.1093/jnen/nlx007

Kuhlmann T, 2017, ACTA NEUROPATHOL, V133, P13, DOI 10.1007/s00401-016-1653-y

Lee YJ, 2012, NATURE, V487, P443, DOI 10.1038/nature11314

Leonard BE, 2018, ACTA NEUROPSYCHIATR, V30, P1, DOI 10.1017/neu.2016.69

LeVine SM, 2016, BMC NEUROL, V16, DOI 10.1186/s12883-016-0564-9

Ling H, 2016, BRAIN, V139, P3237, DOI 10.1093/brain/aww256

Long JM, 2019, CELL, V179, P312, DOI 10.1016/j.cell.2019.09.001

Lucchinetti CF, 2014, BRAIN PATHOL, V24, P83, DOI 10.1111/bpa.12099

Maggi P, 2018, ANN NEUROL, V83, P283, DOI 10.1002/ana.25146

Maia LF, 2013, SCI TRANSL MED, V5, DOI 10.1126/scitranslmed.3006446

Mak A, 2012, SEMIN ARTHRITIS RHEU, V41, P830, DOI 10.1016/j.semarthrit.2011.11.002

Mauffrey P, 2019, NATURE, V569, P672, DOI 10.1038/s41586-019-1219-y

McCreary M, 2018, Mult Scler J Exp Transl Clin, V4, p2055217318815925, DOI 10.1177/2055217318815925

McCrimmon RJ, 2012, LANCET, V379, P2291, DOI 10.1016/S0140-6736(12)60360-2

Miere F, 2020, PHARMACOPHORE, V11, P1

Mina-Osorio P, 2012, MOL MED, V18, P618, DOI 10.2119/molmed.2012.00027

Mitsdoerffer M, 2013, ANN NY ACAD SCI, V1283, P57, DOI 10.1111/nyas.12118

Najjar S, 2013, J NEUROINFLAMM, V10, DOI 10.1186/1742-2094-10-142

Narayan R, 2018, MULT SCLER RELAT DIS, V25, P66, DOI 10.1016/j.msard.2018.07.025

Ofengeim D, 2015, CELL REP, V10, P1836, DOI 10.1016/j.celrep.2015.02.051

Ouchi N, 2011, NAT REV IMMUNOL, V11, P85, DOI 10.1038/nri2921

Palmqvist S, 2019, EMBO MOL MED, V11, DOI 10.15252/emmm.201911170

Papakostas GI, 2013, MOL PSYCHIATR, V18, P332, DOI 10.1038/mp.2011.166

Peng C, 2020, NAT REV NEUROL, V16, P199, DOI 10.1038/s41582-020-0333-7

Pérez-Grijalba V, 2013, J ALZHEIMERS DIS, V36, P211, DOI 10.3233/JAD-121744

Peschl P, 2017, FRONT IMMUNOL, V8, DOI 10.3389/fimmu.2017.00529

Petzold A, 2018, LANCET NEUROL, V17, P496, DOI 10.1016/S1474-4422(18)30159-5

Portelius E, 2010, ALZHEIMERS RES THER, V2, DOI 10.1186/alzrt30

Pryce G, 2018, MULT SCLER RELAT DIS, V25, P131, DOI 10.1016/j.msard.2018.07.030

P┬u┬rez V., 2012, ALZHEIMERS DEMENT, V8, P240, DOI [10.1016/j.jalz.2012.05.638, DOI 10.1016/J.JALZ.2012.05.638]

Rahman MH, 2021, MOLECULES, V26, DOI 10.3390/molecules26175327

Rahman MH, 2021, PROCESSES, V9, DOI 10.3390/pr9020308

Rahman MH, 2020, FRONT PHARMACOL, V11, DOI 10.3389/fphar.2020.619024

Rahman MH, 2021, CNS NEUROL DISORD-DR, V20, P694, DOI 10.2174/1871527319666200722153611

Rasmussen MK, 2018, LANCET NEUROL, V17, P1016, DOI 10.1016/S1474-4422(18)30318-1

Re DB, 2014, NEURON, V81, P1001, DOI 10.1016/j.neuron.2014.01.011

Rosas-Ballina M, 2015, BRAIN BEHAV IMMUN, V44, P19, DOI 10.1016/j.bbi.2014.07.010

Satapathy SK, 2011, MOL MED, V17, P599, DOI 10.2119/molmed.2011.00083

Sato C, 2018, NEURON, V98, P861, DOI 10.1016/j.neuron.2018.04.035

Schiffrin EL, 2014, J HYPERTENS, V32, P228, DOI 10.1097/HJH.0000000000000042

Schneider A, 2020, CNS NEUROL DISORD-DR, V19, P195, DOI 10.2174/1871527319666200525011110

Schnell G, 2011, PLOS PATHOG, V7, DOI 10.1371/journal.ppat.1002286

Selkoe DJ, 2016, EMBO MOL MED, V8, P595, DOI 10.15252/emmm.201606210

Sharifi-Rad M, 2020, J CLIN MED, V9, DOI 10.3390/jcm9041061

Shimizu M, 2011, ATHEROSCLEROSIS, V219, P316, DOI 10.1016/j.atherosclerosis.2011.06.030

Sierksma A, 2020, SCIENCE, V370, P61, DOI 10.1126/science.abb8575

Singleton A, 2016, NEURON, V90, P1154, DOI 10.1016/j.neuron.2016.05.040

Skillbäck T, 2015, BRAIN, V138, P2716, DOI 10.1093/brain/awv181

Skovronsky DM, 2006, ANNU REV PATHOL-MECH, V1, P151, DOI 10.1146/annurev.pathol.1.110304.100113

Sochocka M, 2017, CURR NEUROPHARMACOL, V15, P996, DOI 10.2174/1570159X15666170313122937

Strimbu K, 2010, CURR OPIN HIV AIDS, V5, P463, DOI 10.1097/COH.0b013e32833ed177

Tagde P, 2021, ENVIRON SCI POLLUT R, V28, P52810, DOI 10.1007/s11356-021-16223-0

Tagde P, 2021, BIOMED PHARMACOTHER, V141, DOI 10.1016/j.biopha.2021.111928

Thompson AJ, 2018, LANCET NEUROL, V17, P162, DOI 10.1016/S1474-4422(17)30470-2

Tian L, 2012, J NEUROINFLAMM, V9, DOI 10.1186/1742-2094-9-155

Toga AW, 2012, NEUROSURGERY, V71, P1, DOI 10.1227/NEU.0b013e318258e9ff

Tohidpour A, 2017, FRONT CELL INFECT MI, V7, DOI 10.3389/fcimb.2017.00276

Tousoulis D, 2011, CURR PHARM DESIGN, V17, P4089, DOI 10.2174/138161211798764843

Trojano M, 2017, NAT REV NEUROL, V13, P105, DOI 10.1038/nrneurol.2016.188

Tyler PerrachioneK., 2008, Journal of Consumer Behaviour J. Consumer Behav, V7, P303, DOI [10.1002/cb.253, DOI 10.1002/CB.253]

Ugalde CL, 2016, J NEUROCHEM, V139, P162, DOI 10.1111/jnc.13772

United Nations Environment Programme International Labour Organization World Health Organization International Program on Chemical Safety, 2014, BIOM RISK ASS VAL VA, P1

Uversky VN, 2009, FRONT BIOSCI-LANDMRK, V14, P5188, DOI 10.2741/3594

Verkman AS, 2012, ANNU REV MED, V63, P303, DOI 10.1146/annurev-med-043010-193843

Vives-Bauza C, 2010, AUTOPHAGY, V6, P315, DOI 10.4161/auto.6.2.11199

Walia V, 2022, MOL NEUROBIOL, V59, P657, DOI 10.1007/s12035-021-02617-1

Walker L, 2015, ACTA NEUROPATHOL, V129, P729, DOI 10.1007/s00401-015-1406-3

Weiss N, 2009, BBA-BIOMEMBRANES, V1788, P842, DOI 10.1016/j.bbamem.2008.10.022

Weksler BB, 2005, FASEB J, V19, P1872, DOI 10.1096/fj.04-3458fje

Wolkowitz OM, 2010, DEPRESS ANXIETY, V27, P327, DOI 10.1002/da.20686

Wu SC, 2017, NAT COMMUN, V8, DOI 10.1038/s41467-017-00040-6

Yang Y, 2001, J NEUROSCI, V21, P2661, DOI 10.1523/JNEUROSCI.21-08-02661.2001

Ziemssen T, 2016, BMC NEUROL, V16, DOI 10.1186/s12883-016-0639-7

Zunszain Patricia A, 2013, Curr Top Behav Neurosci, V14, P135, DOI 10.1007/7854_2012_211

NR 157

TC 10

Z9 11

U1 0

U2 12

PU MDPI

PI BASEL

PA ST ALBAN-ANLAGE 66, CH-4052 BASEL, SWITZERLAND

EI 1420-3049

J9 MOLECULES

JI Molecules

PD JUN

PY 2022

VL 27

IS 11

AR 3516

DI 10.3390/molecules27113516

PG 17

WC Biochemistry & Molecular Biology; Chemistry, Multidisciplinary

WE Science Citation Index Expanded (SCI-EXPANDED)

SC Biochemistry & Molecular Biology; Chemistry

GA 1Z6XB

UT WOS:000808963600001

PM 35684455

OA Green Published, gold

DA 2024-08-12

ER

PT J

AU Kalampokini, S

Becker, A

Fassbender, K

Lyros, E

Unger, MM

AF Kalampokini, Stefania

Becker, Anouck

Fassbender, Klaus

Lyros, Epameinondas

Unger, Marcus M.

TI Nonpharmacological Modulation of Chronic Inflammation in Parkinson's

Disease: Role of Diet Interventions

SO PARKINSONS DISEASE

LA English

DT Review

ID INTESTINAL BACTERIAL OVERGROWTH; PROTECTS DOPAMINERGIC-NEURONS;

GUT-BRAIN AXIS; ALPHA-SYNUCLEIN; MOUSE MODEL; MEDITERRANEAN DIET;

MICROGLIAL ACTIVATION; FATTY-ACIDS; RAT MODEL; COGNITIVE DECLINE

AB Neuroinflammation is increasingly recognized as an important pathophysiological feature of neurodegenerative disease such as Parkinson's disease (PD). Recent evidence suggests that neuroinflammation in PD might originate in the intestine and the bidirectional communication between the central and enteric nervous system, the so-called "gut-brain axis," has received growing attention due to its contribution to the pathogenesis of neurological disorders. Diet targets mediators of inflammation with various mechanisms and combined with dopaminergic treatment can exert various beneficial effects in PD. Food-based therapies may favorably modulate gut microbiota composition and enhance the intestinal epithelial integrity or decrease the proinflammatory response by direct effects on immune cells. Diets rich in pre- and probiotics, polyunsaturated fatty acids, phenols including flavonoids, and vitamins, such as the Mediterranean diet or a plant-based diet, may attenuate chronic inflammation and positively influence PD symptoms and even progression of the disease. Dietary strategies should be encouraged in the context of a healthy lifestyle with physical activity, which also has neuroimmune-modifying properties. Thus, diet adaptation appears to be an effective additive, nonpharmacological therapeutic strategy that can attenuate the chronic inflammation implicated in PD, potentially slow down degeneration, and thereby modify the course of the disease. PD patients should be highly encouraged to adopt corresponding lifestyle modifications, in order to improve not only PD symptoms, but also general quality of life. Future research should focus on planning larger clinical trials with dietary interventions in PD in order to obtain hard evidence for the hypothesized beneficial effects.

C1 [Kalampokini, Stefania; Becker, Anouck; Fassbender, Klaus; Lyros, Epameinondas; Unger, Marcus M.] Univ Hosp Saarland, Dept Neurol, Kirrberger Str, D-66421 Homburg, Germany.

C3 Universitatsklinikum des Saarlandes

RP Kalampokini, S (corresponding author), Univ Hosp Saarland, Dept Neurol, Kirrberger Str, D-66421 Homburg, Germany.

EM stkalampok@yahoo.gr; Anouck.Becker@uks.eu; klaus.fassbender@uks.eu;

lyrosep@upatras.gr; marcus.unger@me.com

RI Kalampokini, Stefania/KIL-5351-2024; Unger, Marcus M./ABA-9295-2020;

Stefanadis, Christodoulos/ABH-2232-2020

OI Unger, Marcus M./0000-0003-0805-6968; Stefanadis,

Christodoulos/0000-0001-5974-6454; Becker, Anouck/0000-0003-2465-2276;

Fassbender, Klaus/0000-0003-3596-868X

CR Abbott RD, 2003, J NEUROL, V250, P30, DOI 10.1007/s00415-003-1306-7

Al-Asmakh M, 2015, TISSUE BARRIERS, V3, DOI 10.1080/21688370.2015.1039691

Alcalay RN, 2012, MOVEMENT DISORD, V27, P771, DOI 10.1002/mds.24918

Alfa MJ, 2018, CLIN NUTR, V37, P797, DOI 10.1016/j.clnu.2017.03.025

Allen AP, 2016, TRANSL PSYCHIAT, V6, DOI 10.1038/tp.2016.191

Allen NE, 2015, J PARKINSON DIS, V5, P229, DOI 10.3233/JPD-140508

Ananthakrishnan AN, 2014, GUT, V63, P776, DOI 10.1136/gutjnl-2013-305304

Ang ET, 2004, NEUROSCIENCE, V129, P337, DOI 10.1016/j.neuroscience.2004.06.087

Appenzeller S, 2012, MOVEMENT DISORD, V27, P1189, DOI 10.1002/mds.25059

Arpaia N, 2013, NATURE, V504, P451, DOI 10.1038/nature12726

Bae JR, 2015, BMB REP, V48, P243, DOI 10.5483/BMBRep.2015.48.5.032

Banati RB, 1998, MOVEMENT DISORD, V13, P221, DOI 10.1002/mds.870130205

Barichella M, 2016, NEUROLOGY, V87, P1274, DOI 10.1212/WNL.0000000000003127

Baroni L, 2011, NUTR NEUROSCI, V14, P1, DOI 10.1179/174313211X12966635733231

Barrientos RM, 2011, J NEUROSCI, V31, P11578, DOI 10.1523/JNEUROSCI.2266-11.2011

Bialecka M, 2007, NEUROSCI RES, V57, P473, DOI 10.1016/j.neures.2006.11.012

Blaut Michael, 2002, European Journal of Nutrition, V41, P11

Bloomer RJ, 2008, MED SCI SPORT EXER, V40, P1385, DOI 10.1249/MSS.0b013e31816f1550

Böttner M, 2012, NEUROBIOL DIS, V48, P474, DOI 10.1016/j.nbd.2012.07.018

Braak H, 2006, NEUROSCI LETT, V396, P67, DOI 10.1016/j.neulet.2005.11.012

Braak H, 2003, J NEURAL TRANSM, V110, P517, DOI 10.1007/s00702-002-0808-2

Braak H, 2003, NEUROBIOL AGING, V24, P197, DOI 10.1016/S0197-4580(02)00065-9

Brochard V, 2009, J CLIN INVEST, V119, P182, DOI 10.1172/JCI36470

Brodacki B, 2008, NEUROSCI LETT, V441, P158, DOI 10.1016/j.neulet.2008.06.040

Brothers HM, 2010, NEUROSCI LETT, V480, P97, DOI 10.1016/j.neulet.2010.06.013

Bureau G, 2008, J NEUROSCI RES, V86, P403, DOI 10.1002/jnr.21503

Caracciolo B, 2014, MECH AGEING DEV, V136, P59, DOI 10.1016/j.mad.2013.11.011

Casas R, 2014, PLOS ONE, V9, DOI 10.1371/journal.pone.0100084

Cassani E, 2011, Minerva Gastroenterol Dietol, V57, P117

Chao JF, 2012, NUTR REV, V70, P373, DOI 10.1111/j.1753-4887.2012.00484.x

Chapelet G, 2019, PARKINSONISM RELAT D, V59, P26, DOI 10.1016/j.parkreldis.2018.11.014

Chaudhuri KR, 2006, LANCET NEUROL, V5, P235, DOI 10.1016/S1474-4422(06)70373-8

Chen WC, 2014, INDIAN J MED RES, V140, P582

Chrysohoou C, 2004, J AM COLL CARDIOL, V44, P152, DOI 10.1016/j.jacc.2004.03.039

Corbillé AG, 2017, NEUROSCI LETT, V641, P81, DOI 10.1016/j.neulet.2017.01.050

Costa J, 2010, J ALZHEIMERS DIS, V20, pS221, DOI 10.3233/JAD-2010-091525

Cunningham C, 2013, GLIA, V61, P71, DOI 10.1002/glia.22350

De Filippis Francesca, 2016, Gut, V65, P1812, DOI 10.1136/gutjnl-2015-309957

de Lau LML, 2005, NEUROLOGY, V64, P2040, DOI 10.1212/01.WNL.0000166038.67153.9F

Del Chierico F, 2014, INT J MOL SCI, V15, P11678, DOI 10.3390/ijms150711678

Deleu D, 2006, NEUROLOGY, V67, P897, DOI 10.1212/01.wnl.0000233916.57415.9d

Devore EE, 2012, ANN NEUROL, V72, P135, DOI 10.1002/ana.23594

Devos D, 2013, NEUROBIOL DIS, V50, P42, DOI 10.1016/j.nbd.2012.09.007

Dilger RN, 2008, J LEUKOCYTE BIOL, V84, P932, DOI 10.1189/jlb.0208108

Dobbs RJ, 2012, GUT PATHOG, V4, DOI 10.1186/1757-4749-4-12

Edison P, 2013, NEUROPSYCHOPHARMACOL, V38, P938, DOI 10.1038/npp.2012.255

Ehninger D, 2011, CELL TISSUE RES, V345, P69, DOI 10.1007/s00441-011-1200-z

Erro R, 2018, J NEUROL, V265, P12, DOI 10.1007/s00415-017-8639-0

Etminan M, 2005, LANCET NEUROL, V4, P362, DOI 10.1016/S1474-4422(05)70097-1

Fan Z, 2015, ALZHEIMERS DEMENT, V11, P608, DOI 10.1016/j.jalz.2014.06.016

Fasano A, 2015, LANCET NEUROL, V14, P625, DOI 10.1016/S1474-4422(15)00007-1

Fasano A, 2013, MOVEMENT DISORD, V28, P1241, DOI 10.1002/mds.25522

Fisher BE, 2008, ARCH PHYS MED REHAB, V89, P1221, DOI 10.1016/j.apmr.2008.01.013

Forsyth CB, 2011, PLOS ONE, V6, DOI 10.1371/journal.pone.0028032

Franceschi C, 2014, J GERONTOL A-BIOL, V69, pS4, DOI 10.1093/gerona/glu057

Frei R, 2015, CURR OPIN GASTROEN, V31, P153, DOI 10.1097/MOG.0000000000000151

Gao X, 2012, NEUROLOGY, V78, P1138, DOI 10.1212/WNL.0b013e31824f7fc4

Gao X, 2007, AM J CLIN NUTR, V86, P1486, DOI 10.1093/ajcn/86.5.1486

Gao XA, 2011, NEUROLOGY, V76, P863, DOI 10.1212/WNL.0b013e31820f2d79

Garcion E, 2002, TRENDS ENDOCRIN MET, V13, P100, DOI 10.1016/S1043-2760(01)00547-1

Garrido-Gil P, 2018, MOL NEUROBIOL, V55, P7297, DOI 10.1007/s12035-018-0937-8

Gerhard A, 2006, NEUROBIOL DIS, V21, P404, DOI 10.1016/j.nbd.2005.08.002

Gleeson M, 2006, EXERC IMMUNOL REV, V12, P34

Gleeson M, 2011, NAT REV IMMUNOL, V11, P607, DOI 10.1038/nri3041

Gold A, 2013, MOVEMENT DISORD, V28, P237, DOI 10.1002/mds.25298

Guo SH, 2007, BIOL PSYCHIAT, V62, P1353, DOI 10.1016/j.biopsych.2007.04.020

Gyorkos AM, 2014, NEUROSCIENCE, V257, P111, DOI 10.1016/j.neuroscience.2013.10.068

Habil N, 2014, BENEF MICROBES, V5, P483, DOI 10.3920/BM2013.0061

Hang LT, 2016, NEUROMOL MED, V18, P306, DOI 10.1007/s12017-016-8398-6

Havenaar R, 2011, BENEF MICROBES, V2, P103, DOI 10.3920/BM2011.0003

Hawkes CH, 2010, PARKINSONISM RELAT D, V16, P79, DOI 10.1016/j.parkreldis.2009.08.007

Herrera AJ, 2000, NEUROBIOL DIS, V7, P429, DOI 10.1006/nbdi.2000.0289

Hilton D, 2014, ACTA NEUROPATHOL, V127, P235, DOI 10.1007/s00401-013-1214-6

Hirsch EC, 2009, LANCET NEUROL, V8, P382, DOI 10.1016/S1474-4422(09)70062-6

Houser MC, 2018, MOVEMENT DISORD, V33, P793, DOI 10.1002/mds.27326

Houser MC, 2017, NPJ PARKINSONS DIS, V3, DOI 10.1038/s41531-016-0002-0

Hui KY, 2018, SCI TRANSL MED, V10, DOI 10.1126/scitranslmed.aai7795

Hunot S, 1999, J NEUROSCI, V19, P3440

Imamura K, 2003, ACTA NEUROPATHOL, V106, P518, DOI 10.1007/s00401-003-0766-2

Jackson PA, 2016, J PHYSIOL-LONDON, V594, P4485, DOI 10.1113/JP271270

Jang S, 2010, BRAIN RES, V1313, P25, DOI 10.1016/j.brainres.2009.12.012

Jang Y, 2017, BRAIN RES, V1655, P186, DOI 10.1016/j.brainres.2016.10.029

Jaquet M, 2009, INT J FOOD MICROBIOL, V130, P117, DOI 10.1016/j.ijfoodmicro.2009.01.011

Jeurink PV, 2013, AM J CLIN NUTR, V98, p572S, DOI 10.3945/ajcn.112.038596

Kelly JR, 2017, BRAIN BEHAV IMMUN, V61, P50, DOI 10.1016/j.bbi.2016.11.018

Keshavarzian A, 2015, MOVEMENT DISORD, V30, P1351, DOI 10.1002/mds.26307

Khadrawy Yasser A., 2017, Journal of Dietary Supplements, V14, P553, DOI 10.1080/19390211.2016.1275916

Khan MM, 2010, BRAIN RES, V1328, P139, DOI 10.1016/j.brainres.2010.02.031

Khasnavis S, 2014, J NEUROIMMUNE PHARM, V9, P569, DOI 10.1007/s11481-014-9552-2

Klingelhoefer L, 2015, NAT REV NEUROL, V11, P625, DOI 10.1038/nrneurol.2015.197

Kohman RA, 2012, BRAIN BEHAV IMMUN, V26, P803, DOI 10.1016/j.bbi.2011.10.006

Kortekaas R, 2005, ANN NEUROL, V57, P176, DOI 10.1002/ana.20369

Kovács Z, 2014, ADV BIOCHEM ENG BIOT, V143, P257, DOI 10.1007/10_2013_237

Krüger R, 2000, J NEURAL TRANSM, V107, P553, DOI 10.1007/s007020070078

Kurkowska-Jastrzebska I, 1999, EXP NEUROL, V156, P50, DOI 10.1006/exnr.1998.6993

Lau Francis C, 2007, Subcell Biochem, V42, P299

Lees AJ, 2009, LANCET, V373, P2055, DOI 10.1016/S0140-6736(09)60492-X

Liberatore GT, 1999, NAT MED, V5, P1403

Lu KT, 2008, J AGR FOOD CHEM, V56, P6910, DOI 10.1021/jf8007212

Maraki MI, 2019, MOVEMENT DISORD, V34, P48, DOI 10.1002/mds.27489

Marion-Letellier R, 2013, INFLAMM BOWEL DIS, V19, P205, DOI [10.1097/MIB.0b013e3182810122, 10.1002/ibd.23001]

Mazereeuw G, 2012, NEUROBIOL AGING, V33, DOI 10.1016/j.neurobiolaging.2011.12.014

McFarlin BK, 2006, J GERONTOL A-BIOL, V61, P388, DOI 10.1093/gerona/61.4.388

McGeer PL, 2003, ANN NEUROL, V54, P599, DOI 10.1002/ana.10728

McGeer PL, 2002, NEUROSCI LETT, V326, P67, DOI 10.1016/S0304-3940(02)00300-2

Melancon MO, 2014, APPL PHYSIOL NUTR ME, V39, P1250, DOI 10.1139/apnm-2014-0092

Meng XY, 2009, BIOCHEMISTRY-US, V48, P8206, DOI 10.1021/bi900506b

Mercer LD, 2005, BIOCHEM PHARMACOL, V69, P339, DOI 10.1016/j.bcp.2004.09.018

Miller RL, 2009, NEUROCHEM RES, V34, P55, DOI 10.1007/s11064-008-9656-2

Mischley LK, 2017, OXID MED CELL LONGEV, V2017, DOI 10.1155/2017/6405278

Monteiro RS, 2015, MED HYPOTHESES, V85, P537, DOI 10.1016/j.mehy.2015.07.011

Musa NH, 2017, J DAIRY RES, V84, P488, DOI [10.1017/S0022029917000620, 10.1017/s0022029917000620]

Nagatsu T, 2000, J NEURAL TRANSM-SUPP, P143

Nakayama T, 2013, FEMS MICROBIOL LETT, V343, P161, DOI 10.1111/1574-6968.12142

Nalls MA, 2011, LANCET, V377, P641, DOI 10.1016/S0140-6736(10)62345-8

Niu XL, 2016, J NEURAL TRANSM, V123, P1381, DOI 10.1007/s00702-016-1612-8

Obeso JA, 2010, NAT MED, V16, P653, DOI 10.1038/nm.2165

Olszak T, 2012, SCIENCE, V336, P489, DOI 10.1126/science.1219328

Panza F, 2015, J NUTR HEALTH AGING, V19, P313, DOI 10.1007/s12603-014-0563-8

Parashar A, 2017, PARKINSONISM RELAT D, V38, P1, DOI 10.1016/j.parkreldis.2017.02.002

Pedersen BK, 2009, J APPL PHYSIOL, V107, P1006, DOI 10.1152/japplphysiol.00734.2009

Perez-Pardo P, 2017, EUR J PHARMACOL, V817, P86, DOI 10.1016/j.ejphar.2017.05.042

Perez-Pardo P, 2018, NUTR NEUROSCI, V21, P391, DOI 10.1080/1028415X.2017.1294555

Petersson SD, 2016, ADV NUTR, V7, P889, DOI 10.3945/an.116.012138

Poly TN, 2019, EUR J CLIN PHARMACOL, V75, P99, DOI 10.1007/s00228-018-2561-y

Postuma RB, 2017, NEUROLOGY, V89, P310, DOI 10.1212/WNL.0000000000004163

Postuma RB, 2012, NEUROLOGY, V79, P651, DOI 10.1212/WNL.0b013e318263570d

Qi H, 2014, GERIATR GERONTOL INT, V14, P430, DOI 10.1111/ggi.12123

Qin LY, 2007, GLIA, V55, P453, DOI 10.1002/glia.20467

Rabassa M, 2015, J AM GERIATR SOC, V63, P938, DOI 10.1111/jgs.13379

Reuter I, 2000, MOVEMENT DISORD, V15, P862, DOI 10.1002/1531-8257(200009)15:5<862::AID-MDS1015>3.0.CO;2-S

Ricciarelli R, 2007, MOL ASPECTS MED, V28, P591, DOI 10.1016/j.mam.2007.01.004

Rimmelzwaan LM, 2016, J PARKINSON DIS, V6, P29, DOI 10.3233/JPD-150615

Sartor RB, 2005, CURR OPIN GASTROEN, V21, P44

Schwiertz A, 2018, PARKINSONISM RELAT D, V50, P104, DOI 10.1016/j.parkreldis.2018.02.022

Shannon KM, 2012, MOVEMENT DISORD, V27, P709, DOI 10.1002/mds.23838

Sharma N, 2017, INFLAMMOPHARMACOLOGY, V25, P351, DOI 10.1007/s10787-017-0346-z

Shen L, 2015, NUTRIENTS, V7, P7197, DOI 10.3390/nu7095333

Shores DR, 2011, INFLAMM BOWEL DIS, V17, P2192, DOI 10.1002/ibd.21560

Singh N, 2014, IMMUNITY, V40, P128, DOI 10.1016/j.immuni.2013.12.007

Sonsalla PK, 2012, EXP NEUROL, V234, P482, DOI 10.1016/j.expneurol.2012.01.022

Spagnuolo C, 2018, EUR J MED CHEM, V153, P105, DOI 10.1016/j.ejmech.2017.09.001

Spencer JPE, 2012, MOL ASPECTS MED, V33, P83, DOI 10.1016/j.mam.2011.10.016

Spielman LJ, 2016, BRAIN RES BULL, V125, P19, DOI 10.1016/j.brainresbull.2016.03.012

Stocker P, 2003, BBA-GEN SUBJECTS, V1621, P1, DOI 10.1016/S0304-4165(03)00008-4

Stolzenberg E, 2017, J INNATE IMMUN, V9, P456, DOI 10.1159/000477990

Sureda A, 2018, NUTRIENTS, V10, DOI 10.3390/nu10010062

Tan AH, 2014, PARKINSONISM RELAT D, V20, P535, DOI 10.1016/j.parkreldis.2014.02.019

Tan LC, 2008, AM J EPIDEMIOL, V167, P553, DOI 10.1093/aje/kwm338

Tansey MG, 2010, NEUROBIOL DIS, V37, P510, DOI 10.1016/j.nbd.2009.11.004

Tarozzi A, 2013, OXID MED CELL LONGEV, V2013, DOI 10.1155/2013/415078

Theodore S, 2008, J NEUROPATH EXP NEUR, V67, P1149, DOI 10.1097/NEN.0b013e31818e5e99

Thomas J, 2015, BIOMED RES INT, V2015, DOI 10.1155/2015/172801

Timmerman KL, 2008, J LEUKOCYTE BIOL, V84, P1271, DOI 10.1189/jlb.0408244

Trichopoulou A, 2003, NEW ENGL J MED, V348, P2599, DOI 10.1056/NEJMoa025039

Tuon T, 2012, NEUROSCIENCE, V227, P305, DOI 10.1016/j.neuroscience.2012.09.063

Uhrbrand A, 2015, J NEUROL SCI, V353, P9, DOI 10.1016/j.jns.2015.04.004

Ullegaddi R, 2004, CLIN SCI, V107, P477, DOI 10.1042/CS20040134

Unger MM, 2016, PARKINSONISM RELAT D, V32, P66, DOI 10.1016/j.parkreldis.2016.08.019

Uysal N, 2015, BIOTECH HISTOCHEM, V90, P55, DOI 10.3109/10520295.2014.946968

Varankovich NV, 2015, FRONT MICROBIOL, V6, DOI 10.3389/fmicb.2015.00685

Vauzour D, 2017, AGEING RES REV, V35, P222, DOI 10.1016/j.arr.2016.09.010

Venema WTCU, 2017, J PATHOL, V241, P146, DOI 10.1002/path.4817

Wang J, 2012, SCAND J MED SCI SPOR, V22, P643, DOI 10.1111/j.1600-0838.2010.01288.x

Wang JY, 2001, BRAIN RES, V904, P67, DOI 10.1016/S0006-8993(01)02450-7

Wang LH, 2014, J BIOL CHEM, V289, P20234, DOI 10.1074/jbc.M114.553800

Weatherill AR, 2005, J IMMUNOL, V174, P5390, DOI 10.4049/jimmunol.174.9.5390

Williams CM, 2008, FREE RADICAL BIO MED, V45, P295, DOI 10.1016/j.freeradbiomed.2008.04.008

Williams-Gray CH, 2016, MOVEMENT DISORD, V31, P995, DOI 10.1002/mds.26563

Wondrak Georg T, 2012, Subcell Biochem, V56, P291, DOI 10.1007/978-94-007-2199-9_15

Wu DC, 2002, J NEUROSCI, V22, P1763, DOI 10.1523/JNEUROSCI.22-05-01763.2002

Wu PL, 2017, PLOS ONE, V12, DOI 10.1371/journal.pone.0181515

Xu Q, 2010, NEUROLOGY, V75, P341, DOI 10.1212/WNL.0b013e3181ea1597

Xu Q, 2017, J NUTR, V147, P1926, DOI 10.3945/jn.117.255034

Zaharoni H, 2011, J NUTR HEALTH AGING, V15, P215, DOI 10.1007/s12603-010-0323-3

Zhang F, 2010, MOL PHARMACOL, V78, P466, DOI 10.1124/mol.110.064535

Zhang F, 2010, EUR J PHARMACOL, V636, P1, DOI 10.1016/j.ejphar.2010.03.043

Zhang W, 2005, FASEB J, V19, P533, DOI 10.1096/fj.04-2751com

Zhou TT, 2018, MOL MED REP, V17, P4883, DOI 10.3892/mmr.2018.8470

Zoladz JA, 2014, J PHYSIOL PHARMACOL, V65, P441

Zou YM, 2015, NEUROPSYCH DIS TREAT, V11, P695, DOI 10.2147/NDT.S79707

NR 181

TC 20

Z9 21

U1 0

U2 17

PU HINDAWI LTD

PI LONDON

PA ADAM HOUSE, 3RD FLR, 1 FITZROY SQ, LONDON, W1T 5HF, United Kingdom

SN 2090-8083

EI 2042-0080

J9 PARKINSONS DIS-US

JI Parkinsons Dis.

PD AUG 25

PY 2019

VL 2019

AR 7535472

DI 10.1155/2019/7535472

PG 12

WC Clinical Neurology

WE Science Citation Index Expanded (SCI-EXPANDED)

SC Neurosciences & Neurology

GA IX7KE

UT WOS:000485861400001

PM 31534664

OA Green Submitted, Green Published, gold

DA 2024-08-12

ER

PT J

AU Mo, JL

Hu, J

Cheng, XL

AF Mo, Jialu

Hu, Jiao

Cheng, Xianglin

TI The role of high mobility group box 1 in neuroinflammatory related

diseases

SO BIOMEDICINE & PHARMACOTHERAPY

LA English

DT Review

DE Neuroinflammation; High mobility group box 1; Toll-like receptor 4;

Receptor for advanced glycation endproducts

ID MONOCLONAL-ANTIBODY; EXPRESSION LEVEL; PROTEIN HMGB1; TNF-ALPHA;

EPILEPSY; MODEL; AUTOPHAGY; DAMAGE

AB High mobility group box 1 (HMGB1) is a ubiquitous and highly conserved non-histone DNA-binding protein with different biological functions according to its subcellular localization. It is widely believed that HMGB1, which is released into the extracellular space, plays a key role in the inflammatory response. In recent years, numerous studies have shown that the development of various neurological diseases such as epilepsy, Alzheimer's disease (AD), Parkinson's disease (PD), amyotrophic lateral sclerosis (ALS), multiple sclerosis (MS), cerebrovascular disease and traumatic brain injury (TBI) are inextricably linked to inflammation. We will review the mechanisms of HMGB1 and its receptors in nervous system inflammation to provide a basis for further development of new HMGB1-based therapies.

C1 [Mo, Jialu; Hu, Jiao; Cheng, Xianglin] Yangtze Univ, Affiliated Hosp 1, Jingzhou 434000, Hubei, China.

C3 Yangtze University

RP Cheng, XL (corresponding author), Yangtze Univ, Affiliated Hosp 1, Jingzhou 434000, Hubei, China.

EM chengxianglin@yangtzeu.edu.cn

CR Abbott NJ, 2006, NAT REV NEUROSCI, V7, P41, DOI 10.1038/nrn1824

Abuelezz SA, 2021, LIFE SCI, V273, DOI 10.1016/j.lfs.2021.119310

Alabed S, 2021, INT J MOL SCI, V22, DOI 10.3390/ijms22094781

Ana R, 2019, INT J PHARMACEUT, V554, P352, DOI 10.1016/j.ijpharm.2018.11.028

Balestrini S, 2021, EPILEPTIC DISORD, V23, P1, DOI 10.1684/epd.2021.1255

Brambilla L, 2018, FRONT NEUROSCI-SWITZ, V12, DOI 10.3389/fnins.2018.00622

Bucova M, 2020, NEUROL SCI, V41, P599, DOI 10.1007/s10072-019-04136-3

Casula M, 2011, NEUROSCIENCE, V179, P233, DOI 10.1016/j.neuroscience.2011.02.001

Chen RC, 2022, EXP MOL MED, V54, P91, DOI 10.1038/s12276-022-00736-w

Chen RC, 2020, HELIYON, V6, DOI 10.1016/j.heliyon.2020.e05672

Chen XR, 2018, J NEUROINFLAMM, V15, DOI [10.1186/s12974-018-1151-3, 10.1186/s12974-017-0917-3]

Chen Y, 2018, EUR REV MED PHARMACO, V22, P223, DOI [10.26355/eurrev_201801_14120, 10.26355/eurrev_201801_14121]

Chu YJ, 2021, J NEUROIMMUNOL, V352, DOI 10.1016/j.jneuroim.2021.577480

Dewan MC, 2019, J NEUROSURG, V130, P1080, DOI 10.3171/2017.10.JNS17352

Diaz-Arrastia R, 2014, J NEUROTRAUM, V31, P135, DOI 10.1089/neu.2013.3019

DiSabato DJ, 2016, J NEUROCHEM, V139, P136, DOI 10.1111/jnc.13607

Evran S, 2020, BRAIN RES BULL, V154, P68, DOI 10.1016/j.brainresbull.2019.10.013

Festoff BW, 2016, J NEUROINFLAMM, V13, DOI 10.1186/s12974-016-0670-z

Fu L, 2017, SCI REP-UK, V7, DOI 10.1038/s41598-017-01325-y

Fujita K, 2016, SCI REP-UK, V6, DOI 10.1038/srep31895

Gaikwad S, 2021, CELL REP, V36, DOI 10.1016/j.celrep.2021.109419

Gan P, 2020, FRONT PHARMACOL, V11, DOI 10.3389/fphar.2020.00776

Gao JM, 2022, ACTA PHARMACOL SIN, V43, P2482, DOI 10.1038/s41401-022-00888-5

Gao TL, 2018, BIOCHEM BIOPH RES CO, V497, P430, DOI 10.1016/j.bbrc.2018.02.102

George MG, 2017, JAMA NEUROL, V74, P695, DOI 10.1001/jamaneurol.2017.0020

GOODWIN GH, 1973, EUR J BIOCHEM, V40, P215, DOI 10.1111/j.1432-1033.1973.tb03188.x

GOODWIN GH, 1973, EUR J BIOCHEM, V38, P14, DOI 10.1111/j.1432-1033.1973.tb03026.x

Greene C, 2022, NAT COMMUN, V13, DOI 10.1038/s41467-022-29657-y

Gu JJ, 2016, MOL MED REP, V14, P5217, DOI 10.3892/mmr.2016.5891

Han YW, 2021, EUR J PHARMACOL, V893, DOI 10.1016/j.ejphar.2020.173811

Hei Y, 2018, NEUROSCIENCE, V383, P150, DOI 10.1016/j.neuroscience.2018.05.010

Hiramoto S, 2020, CELLS-BASEL, V9, DOI 10.3390/cells9081748

Hoftberger R, 2018, HAND CLINIC, V145, P263, DOI 10.1016/B978-0-12-802395-2.00019-5

Huang JS, 2015, EUR REV MED PHARMACO, V19, P2928

Huang QJ, 2020, CELL MOL BIOL, V66, P39, DOI 10.14715/cmb/2020.66.3.6

Hwang CS, 2013, NEUROBIOL DIS, V58, P13, DOI 10.1016/j.nbd.2013.04.013

Izrael M, 2020, FRONT NEUROSCI-SWITZ, V14, DOI 10.3389/fnins.2020.00824

Jayaraj RL, 2019, J NEUROINFLAMM, V16, DOI 10.1186/s12974-019-1516-2

Jin LL, 2023, BIOACT MATER, V19, P38, DOI 10.1016/j.bioactmat.2022.03.040

Kamasak T, 2020, EPILEPTIC DISORD, V22, P183, DOI 10.1684/epd.2020.1155

Kamel H, 2012, ARCH NEUROL-CHICAGO, V69, P576, DOI 10.1001/archneurol.2011.3590

Kaneko Y, 2017, CELL PHYSIOL BIOCHEM, V41, P947, DOI 10.1159/000460513

Kaya MA, 2021, EPILEPSY RES, V169, DOI 10.1016/j.eplepsyres.2020.106516

Kinney Jefferson W, 2018, Alzheimers Dement (N Y), V4, P575, DOI 10.1016/j.trci.2018.06.014

Lassmann H, 2018, CSH PERSPECT MED, V8, DOI 10.1101/cshperspect.a028936

Lee JY, 2015, J NEUROINFLAMM, V12, DOI 10.1186/s12974-015-0310-z

Lee JD, 2019, J NEUROINFLAMM, V16, DOI 10.1186/s12974-019-1435-2

Lei C, 2015, NEUROSCIENCE, V295, P39, DOI 10.1016/j.neuroscience.2015.03.032

Lei CY, 2022, BRAIN RES, V1792, DOI 10.1016/j.brainres.2022.148003

Li D, 2015, NEUROSCI LETT, V609, P109, DOI 10.1016/j.neulet.2015.10.035

Li EC, 2022, EPILEPSIA, V63, P2173, DOI 10.1111/epi.17315

Lin F, 2018, J CELL PHYSIOL, V233, P6683, DOI 10.1002/jcp.26468

Lindersson EK, 2004, NEUROREPORT, V15, P2735

Löscher W, 2020, INT J MOL SCI, V21, DOI 10.3390/ijms21020591

Lv RX, 2019, LIFE SCI, V223, P158, DOI 10.1016/j.lfs.2019.03.030

McCauley ME, 2019, ACTA NEUROPATHOL, V137, P715, DOI 10.1007/s00401-018-1933-9

Nakano T, 2022, NEUROREPORT, V33, P297, DOI 10.1097/WNR.0000000000001780

Nan K, 2019, INT IMMUNOPHARMACOL, V67, P294, DOI 10.1016/j.intimp.2018.12.027

Nass RD, 2020, EPILEPSY RES, V162, DOI 10.1016/j.eplepsyres.2020.106301

Neri S, 2022, EPILEPTIC DISORD, V24, P249, DOI 10.1684/epd.2021.1406

Nishibori M, 2020, CELLS-BASEL, V9, DOI 10.3390/cells9122650

Novellino F, 2020, INT J MOL SCI, V21, DOI 10.3390/ijms21031115

Nowicka N, 2022, INT J MOL SCI, V23, DOI 10.3390/ijms23042184

Ossenkoppele R, 2022, LANCET NEUROL, V21, P726, DOI 10.1016/S1474-4422(22)00168-5

Pajares M, 2020, CELLS-BASEL, V9, DOI 10.3390/cells9071687

Paudel YN, 2020, CELLS-BASEL, V9, DOI 10.3390/cells9020383

Paudel YN, 2019, LIFE SCI, V238, DOI 10.1016/j.lfs.2019.116924

Paudel YN, 2018, FRONT NEUROSCI-SWITZ, V12, DOI 10.3389/fnins.2018.00628

Pellegrini L, 2019, PHARMACOL THERAPEUT, V196, P160, DOI 10.1016/j.pharmthera.2018.12.005

Ransohoff RM, 2016, SCIENCE, V353, P777, DOI 10.1126/science.aag2590

Rapoport BL, 2020, CELLS-BASEL, V9, DOI 10.3390/cells9071664

Ren QY, 2022, J ETHNOPHARMACOL, V292, DOI 10.1016/j.jep.2022.115234

Robinson AP, 2013, J AUTOIMMUN, V43, P32, DOI 10.1016/j.jaut.2013.02.005

Santoro M, 2016, NEUROBIOL DIS, V91, P59, DOI 10.1016/j.nbd.2016.02.018

Sasaki T, 2016, EXP NEUROL, V275, P220, DOI 10.1016/j.expneurol.2015.11.003

Scaffidi P, 2002, NATURE, V418, P191, DOI 10.1038/nature00858

Sharafkhah M, 2022, NEUROL INDIA, V70, P238, DOI 10.4103/0028-3886.338707

Shi YB, 2018, MOL MED REP, V17, P5125, DOI 10.3892/mmr.2018.8485

Song JX, 2014, AUTOPHAGY, V10, P144, DOI 10.4161/auto.26751

Sun RJ, 2020, J VIROL, V94, DOI 10.1128/JVI.00238-20

Sun Y, 2018, FRONT IMMUNOL, V9, DOI 10.3389/fimmu.2018.01518

Tan SW, 2021, J NEUROINFLAMM, V18, DOI 10.1186/s12974-021-02274-0

Tanaka H, 2021, COMMUN BIOL, V4, DOI 10.1038/s42003-021-02671-4

Terrone G, 2020, NEUROPHARMACOLOGY, V167, DOI 10.1016/j.neuropharm.2019.107742

Terrone G, 2017, CURR PHARM DESIGN, V23, P5569, DOI 10.2174/1381612823666170926113754

Thakur S, 2023, INFLAMMATION, V46, P1, DOI 10.1007/s10753-022-01721-1

Tian Y, 2020, CLIN SCI, V134, P2075, DOI 10.1042/CS20200553

Tsukagawa T, 2017, J STROKE CEREBROVASC, V26, P2404, DOI 10.1016/j.jstrokecerebrovasdis.2017.05.033

Uddin MS, 2021, MOL NEUROBIOL, V58, P281, DOI 10.1007/s12035-020-02116-9

Umahara T, 2018, HISTOL HISTOPATHOL, V33, P215, DOI 10.14670/HH-11-914

Uzawa A, 2013, CLIN EXP IMMUNOL, V172, P37, DOI 10.1111/cei.12036

Voong CK, 2021, BIOMOLECULES, V11, DOI 10.3390/biom11101451

Wakabayashi A, 2018, CELL DEATH DIS, V9, DOI 10.1038/s41419-018-0665-z

Walker LE, 2022, EPILEPSIA, V63, pE1, DOI 10.1111/epi.17116

Walker LE, 2019, J CLIN INVEST, V129, P2166, DOI 10.1172/JCI129285

Wang S, 2021, CURR DRUG TARGETS, V22, P171, DOI 10.2174/1389450121999200729150443

Williams GP, 2021, BRAIN, V144, P2047, DOI 10.1093/brain/awab103

Xie WJ, 2019, BIOMOLECULES, V9, DOI 10.3390/biom9100512

Yang H, 2015, MOL MED, V21, pS6, DOI 10.2119/molmed.2015.00087

Yang LJ, 2018, CELL PHYSIOL BIOCHEM, V46, P2532, DOI 10.1159/000489659

Yang Y, 2022, NATURE, V610, P791, DOI 10.1038/s41586-022-05319-3

Yang Y, 2018, BRAIN BEHAV, V8, DOI 10.1002/brb3.948

Ying Cui, 2020, Acta Histochem, V122, P151485, DOI 10.1016/j.acthis.2019.151485

Zhang HL, 2018, EUR REV MED PHARMACO, V22, P2372, DOI 10.26355/eurrev_201804_14829

Zhang XS, 2016, INT J MOL SCI, V17, DOI 10.3390/ijms17081331

Zhao JL, 2020, NEUROTHERAPEUTICS, V17, P710, DOI 10.1007/s13311-019-00815-3

Zhao JL, 2017, BRAIN BEHAV IMMUN, V64, P308, DOI 10.1016/j.bbi.2017.02.002

Zhao M, 2021, J EXP CLIN CANC RES, V40, DOI 10.1186/s13046-021-01897-8

Zhen C, 2019, MULT SCLER RELAT DIS, V31, P87, DOI 10.1016/j.msard.2019.03.030

Zhong HH, 2020, J IMMUNOL, V205, P767, DOI 10.4049/jimmunol.1900860

Zhou R, 2021, FRONT IMMUNOL, V12, DOI 10.3389/fimmu.2021.739130

Zhu M, 2018, J CHILD NEUROL, V33, P909, DOI 10.1177/0883073818801654

NR 112

TC 8

Z9 9

U1 1

U2 8

PU ELSEVIER FRANCE-EDITIONS SCIENTIFIQUES MEDICALES ELSEVIER

PI ISSY-LES-MOULINEAUX

PA 65 RUE CAMILLE DESMOULINS, CS50083, 92442 ISSY-LES-MOULINEAUX, FRANCE

SN 0753-3322

EI 1950-6007

J9 BIOMED PHARMACOTHER

JI Biomed. Pharmacother.

PD MAY

PY 2023

VL 161

AR 114541

DI 10.1016/j.biopha.2023.114541

EA MAR 2023

PG 11

WC Medicine, Research & Experimental; Pharmacology & Pharmacy

WE Science Citation Index Expanded (SCI-EXPANDED)

SC Research & Experimental Medicine; Pharmacology & Pharmacy

GA M5DK0

UT WOS:001030415800001

PM 36963363

OA gold

DA 2024-08-12

ER

PT J

AU Vitner, EB

Farfel-Becker, T

Ferreira, NS

Leshkowitz, D

Sharma, P

Lang, KS

Futerman, AH

AF Vitner, Einat B.

Farfel-Becker, Tamar

Ferreira, Natalia Santos

Leshkowitz, Dena

Sharma, Piyush

Lang, Karl S.

Futerman, Anthony H.

TI Induction of the type I interferon response in neurological forms of

Gaucher disease

SO JOURNAL OF NEUROINFLAMMATION

LA English

DT Article

ID INNATE IMMUNE-RESPONSES; CENTRAL-NERVOUS-SYSTEM; LYSOSOMAL STORAGE

DISEASES; PROGRAMMED NECROSIS; MOUSE MODEL; NEURONOPATHIC FORMS;

parkinson's disease; SANDHOFF-DISEASE; CELL-DEATH; ACTIVATION

AB Background: Neuroinflammation is a key phenomenon in the pathogenesis of many neurodegenerative disease. Understanding the mechanisms by which brain inflammation is engaged and delineating the key players in the immune response and their contribution to brain pathology is of great importance for the identification of novel therapeutic targets for these devastating diseases. Gaucher disease, the most common lysosomal storage disease, is caused by mutations in the GBA1 gene and is a significant risk factor for Parkinson's disease; in some forms of Gaucher disease, neuroinflammation is observed.

Methods: An unbiased gene profile analysis was performed on a severely affected brain area of a neurological form of a Gaucher disease mouse at a pre-symptomatic stage; the mouse used for this study, the Gba(flox/flox); nestin-Cre mouse, was engineered such that GBA1 deficiency is restricted to cells of neuronal lineage, i.e., neurons and macroglia.

Results: The 10 most up-regulated genes in the ventral posteromedial/posterolateral region of the thalamus were inflammatory genes, with the gene expression signature significantly enriched in interferon signaling genes. Interferon beta levels were elevated in neurons, and interferon-stimulated genes were elevated mainly in microglia. Interferon signaling pathways were elevated to a small extent in the brain of another lysosomal storage disease mouse model, Krabbe disease, but not in Niemann-Pick C or Sandhoff mouse brain. Ablation of the type I interferon receptor attenuated neuroinflammation but had no effect on GD mouse viability.

Conclusions: Our results imply that the type I interferon response is involved in the development of nGD pathology, and possibly in other lysosomal storage diseases in which simple glycosphingolipids accumulate, and support the notion that interferon signaling pathways play a vital role in the sterile inflammation that often occurs during chronic neurodegenerative disease in which neuroinflammation is present.

C1 [Vitner, Einat B.; Farfel-Becker, Tamar; Ferreira, Natalia Santos; Futerman, Anthony H.] Weizmann Inst Sci, Dept Biomol Sci, IL-76100 Rehovot, Israel.

[Leshkowitz, Dena] Weizmann Inst Sci, Biol Serv Dept, Bioinformat Unit, IL-76100 Rehovot, Israel.

[Sharma, Piyush; Lang, Karl S.] Univ Duisburg Essen, Fac Med, Inst Immunol, Essen, Germany.

[Lang, Karl S.] Univ Dusseldorf, Dept Gastroenterol Hepatol & Infect Dis, Dusseldorf, Germany.

[Vitner, Einat B.] Israel Inst Biol Res, Dept Infect Dis, IL-74100 Ness Ziona, Israel.

[Farfel-Becker, Tamar] NINDS, Synapt Funct Sect, Porter Neurosci Res Ctr, NIH, Bldg 36,Rm 4D04, Bethesda, MD 20892 USA.

C3 Weizmann Institute of Science; Weizmann Institute of Science; University

of Duisburg Essen; Heinrich Heine University Dusseldorf; National

Institutes of Health (NIH) - USA; NIH National Institute of Neurological

Disorders & Stroke (NINDS)

RP Futerman, AH (corresponding author), Weizmann Inst Sci, Dept Biomol Sci, IL-76100 Rehovot, Israel.

EM tony.futerman@weizmann.ac.il

RI Leshkowitz, Dena/HPG-3677-2023; Lang, Karl S/R-2505-2016; Sharma,

Piyush/K-8927-2019

OI Sharma, Piyush/0000-0003-1185-9534; Bleiberg Vitner,

Einat/0000-0001-8578-8551; Lang, Karl/0000-0001-9783-7605

FU Children's Gaucher Research Fund; Minerva Foundation

FX This work was generously supported by the Children's Gaucher Research

Fund and by the Minerva Foundation.

CR Alam MS, 2012, PLOS ONE, V7, DOI 10.1371/journal.pone.0048273

Amor S, 2014, IMMUNOLOGY, V142, P151, DOI 10.1111/imm.12233

Baruch K, 2014, SCIENCE, V346, P89, DOI 10.1126/science.1252945

Bauer S, 2008, BIOINFORMATICS, V24, P1650, DOI 10.1093/bioinformatics/btn250

Beutler E., 2001, METABOLIC MOL BASES, V3, P3635

Borden EC, 2007, NAT REV DRUG DISCOV, V6, P975, DOI 10.1038/nrd2422

Broz P, 2013, NAT REV IMMUNOL, V13, P551, DOI 10.1038/nri3479

Canton J, 2013, NAT REV IMMUNOL, V13, P621, DOI 10.1038/nri3515

Cho H, 2013, NAT MED, V19, P458, DOI 10.1038/nm.3108

Cho Y, 2009, CELL, V137, P1112, DOI 10.1016/j.cell.2009.05.037

Cohen KW, 2015, J IMMUNOL, V194, P2769, DOI 10.4049/jimmunol.1400952

CONRADI NG, 1984, ACTA NEUROPATHOL, V65, P99, DOI 10.1007/BF00690463

Crow YJ, 2015, NAT REV IMMUNOL, V15, P429, DOI 10.1038/nri3850

Dalet A, 2015, FEBS LETT, V589, P1539, DOI 10.1016/j.febslet.2015.05.006

Detje CN, 2009, J IMMUNOL, V182, P2297, DOI 10.4049/jimmunol.0800596

Diamond MS, 2013, NAT REV IMMUNOL, V13, P46, DOI 10.1038/nri3344

Ejlerskov P, 2015, CELL, V163, P324, DOI 10.1016/j.cell.2015.08.069

Enquist IB, 2007, P NATL ACAD SCI USA, V104, P17483, DOI 10.1073/pnas.0708086104

Farfel-Becker T, 2014, HUM MOL GENET, V23, P843, DOI 10.1093/hmg/ddt468

Farfel-Becker T, 2011, DIS MODEL MECH, V4, P746, DOI 10.1242/dmm.008185

Farfel-Becker T, 2011, HUM MOL GENET, V20, P1375, DOI 10.1093/hmg/ddr019

Farina C, 2007, TRENDS IMMUNOL, V28, P138, DOI 10.1016/j.it.2007.01.005

González-Navajas JM, 2012, NAT REV IMMUNOL, V12, P125, DOI 10.1038/nri3133

Huang DW, 2007, NUCLEIC ACIDS RES, V35, pW169, DOI 10.1093/nar/gkm415

Hulsen T, 2008, BMC GENOMICS, V9, DOI 10.1186/1471-2164-9-488

Irizarry RA, 2003, BIOSTATISTICS, V4, P249, DOI 10.1093/biostatistics/4.2.249

Jeyakumar M, 2004, ANN NEUROL, V56, P642, DOI 10.1002/ana.20242

Jeyakumar M, 2003, BRAIN, V126, P974, DOI 10.1093/brain/awg089

Jouan-Lanhouet S, 2014, SEMIN CELL DEV BIOL, V35, P2, DOI 10.1016/j.semcdb.2014.08.010

Kamphuis E, 2006, BLOOD, V108, P3253, DOI 10.1182/blood-2006-06-027599

KANFER JN, 1975, BIOCHEM BIOPH RES CO, V67, P85, DOI 10.1016/0006-291X(75)90286-7

Khorooshi R, 2010, J IMMUNOL, V185, P1258, DOI 10.4049/jimmunol.0901753

Köberlin MS, 2015, CELL, V162, P170, DOI 10.1016/j.cell.2015.05.051

LEBON P, 1988, J NEUROL SCI, V84, P201, DOI 10.1016/0022-510X(88)90125-6

Lee-Kirsch MA, 2015, SEMIN IMMUNOPATHOL, V37, P349, DOI 10.1007/s00281-015-0500-x

Liu SY, 2011, CURR OPIN IMMUNOL, V23, P57, DOI 10.1016/j.coi.2010.11.003

MULLER U, 1994, SCIENCE, V264, P1918, DOI 10.1126/science.8009221

NILSSON O, 1982, J NEUROCHEM, V39, P709, DOI 10.1111/j.1471-4159.1982.tb07950.x

Ning S, 2011, GENES IMMUN, V12, P399, DOI 10.1038/gene.2011.21

Okun E, 2009, BRAIN RES REV, V59, P278, DOI 10.1016/j.brainresrev.2008.09.001

Osorio F, 2011, IMMUNITY, V34, P651, DOI 10.1016/j.immuni.2011.05.001

Paun A, 2007, BIOCHIMIE, V89, P744, DOI 10.1016/j.biochi.2007.01.014

Pelled D, 2005, NEUROBIOL DIS, V18, P83, DOI 10.1016/j.nbd.2004.09.004

PENTCHEV PG, 1984, J BIOL CHEM, V259, P5784

Petry H, 2006, J INTERF CYTOK RES, V26, P699, DOI 10.1089/jir.2006.26.699

Robinson N, 2012, NAT IMMUNOL, V13, P954, DOI 10.1038/ni.2397

Rönnblom L, 2011, UPSALA J MED SCI, V116, P227, DOI 10.3109/03009734.2011.624649

Sadler AJ, 2008, NAT REV IMMUNOL, V8, P559, DOI 10.1038/nri2314

SANGO K, 1995, NAT GENET, V11, P170, DOI 10.1038/ng1095-170

Schneider WM, 2014, ANNU REV IMMUNOL, V32, P513, DOI 10.1146/annurev-immunol-032713-120231

Sidransky E, 2009, NEW ENGL J MED, V361, P1651, DOI 10.1056/NEJMoa0901281

Smith D, 2009, NEUROBIOL DIS, V36, P242, DOI 10.1016/j.nbd.2009.07.010

Sridharan H, 2014, TRENDS MICROBIOL, V22, P199, DOI 10.1016/j.tim.2014.01.005

Sun LM, 2012, CELL, V148, P213, DOI 10.1016/j.cell.2011.11.031

SUZUKI K, 1995, MICROSC RES TECHNIQ, V32, P204, DOI 10.1002/jemt.1070320304

Suzuki M, 2007, J NEUROSCI, V27, P1879, DOI 10.1523/JNEUROSCI.5282-06.2007

Taylor JM, 2014, NEUROBIOL AGING, V35, P1012, DOI 10.1016/j.neurobiolaging.2013.10.089

Thapa RJ, 2013, P NATL ACAD SCI USA, V110, pE3109, DOI 10.1073/pnas.1301218110

Upton JW, 2012, CELL HOST MICROBE, V11, P290, DOI [10.1016/j.chom.2012.01.016, 10.1016/j.chom.2019.09.004]

Vitner EB, 2015, BIOL CHEM, V396, P659, DOI 10.1515/hsz-2014-0301

Vitner EB, 2014, NAT MED, V20, P204, DOI 10.1038/nm.3449

Vitner EB, 2012, BRAIN, V135, P1724, DOI 10.1093/brain/aws095

Vitner EB, 2010, HUM MOL GENET, V19, P3583, DOI 10.1093/hmg/ddq273

Vitner EB, 2010, J BIOL CHEM, V285, P20423, DOI 10.1074/jbc.R110.134452

Wada R, 2000, P NATL ACAD SCI USA, V97, P10954, DOI 10.1073/pnas.97.20.10954

Wang RG, 2011, GLIA, V59, P946, DOI 10.1002/glia.21167

Weng D, 2014, P NATL ACAD SCI USA, V111, P7391, DOI 10.1073/pnas.1403477111

Wong KD, 2004, MOL GENET METAB, V82, P192, DOI 10.1016/j.ymgme.2004.04.011

Yona S, 2013, IMMUNITY, V38, P79, DOI 10.1016/j.immuni.2012.12.001

Zigdon H, 2015, PLOS ONE, V10, DOI 10.1371/journal.pone.0120194

NR 70

TC 51

Z9 56

U1 0

U2 8

PU BIOMED CENTRAL LTD

PI LONDON

PA 236 GRAYS INN RD, FLOOR 6, LONDON WC1X 8HL, United Kingdom

SN 1742-2094

J9 J NEUROINFLAMM

JI J. Neuroinflamm.

PD MAY 12

PY 2016

VL 13

AR 104

DI 10.1186/s12974-016-0570-2

PG 15

WC Immunology; Neurosciences

WE Science Citation Index Expanded (SCI-EXPANDED)

SC Immunology; Neurosciences & Neurology

GA DL8OL

UT WOS:000375901200001

PM 27175482

OA Green Published, gold

DA 2024-08-12

ER

PT J

AU Bartels, AL

Leenders, KL

AF Bartels, Anna L.

Leenders, Klaus L.

TI Cyclooxygenase and Neuroinflammation in Parkinson's Disease

Neurodegeneration

SO CURRENT NEUROPHARMACOLOGY

LA English

DT Article

DE COX-2; Parkinson; neuroinflammation; microglia; neurodegeneration;

neuroprotection

ID NONSTEROIDAL ANTIINFLAMMATORY DRUGS; PERIPHERAL

BENZODIAZEPINE-RECEPTORS; BLOOD-BRAIN-BARRIER; MICROGLIAL ACTIVATION;

P-GLYCOPROTEIN; DOPAMINERGIC NEURODEGENERATION; SUBSTANTIA-NIGRA;

GASTROINTESTINAL TOXICITY; INDUCIBLE CYCLOOXYGENASE;

RHEUMATOID-ARTHRITIS

AB Cyclooxygenase (COX) expression in the brain is associated with pro-inflammatory activities, which are instrumental in neurodegenerative processes such as Parkinson's disease (PD). It is discussed that drugs with the capacity to rescue dopaminergic neurons from microglia toxicity and neuroinflammatory processes may result in an amelioration of parkinsonian symptoms by delaying the onset or slowing progression. This article reviews the involvement of COX in neuroinflammation, specifically focussing at the role of selective COX-2 inhibition in neuroinflammation and neurodegeneration in Parkinson's disease.

C1 [Bartels, Anna L.; Leenders, Klaus L.] Univ Groningen, Univ Med Ctr Groningen, Dept Neurol, NL-9700 RB Groningen, Netherlands.

C3 University of Groningen

RP Bartels, AL (corresponding author), Univ Groningen, Univ Med Ctr Groningen, Dept Neurol, Hanzepl 1, NL-9700 RB Groningen, Netherlands.

EM a.l.bartels@neuro.umcg.nl

CR Aid S, 2008, J NEUROINFLAMM, V5, DOI 10.1186/1742-2094-5-17

Bartels AL, 2008, J NEURAL TRANSM, V115, P1001, DOI 10.1007/s00702-008-0030-y

Bartels AL, 2010, PARKINSONISM RELAT D, V16, P57, DOI 10.1016/j.parkreldis.2009.05.005

Bauer B, 2008, MOL PHARMACOL, V73, P1444, DOI 10.1124/mol.107.041210

Beach TG, 2007, ACTA NEUROPATHOL, V114, P419, DOI 10.1007/s00401-007-0250-5

Bezzi P, 2001, NAT NEUROSCI, V4, P702, DOI 10.1038/89490

Bombardier C, 2000, NEW ENGL J MED, V343, P1520, DOI 10.1056/NEJM200011233432103

Boutin H, 2007, GLIA, V55, P1459, DOI 10.1002/glia.20562

Boutin H, 2007, J NUCL MED, V48, P573, DOI 10.2967/jnumed.106.036764

Bower JH, 2006, NEUROLOGY, V67, P494, DOI 10.1212/01.wnl.0000227906.99570.cc

Braak H, 2000, J NEUROL, V247, P3

BREDER CD, 1995, J COMP NEUROL, V355, P296, DOI 10.1002/cne.903550208

Carrasco E, 2005, J NEUROSCI RES, V81, P121, DOI 10.1002/jnr.20541

Chae SW, 2008, NEUROSCI LETT, V436, P205, DOI 10.1016/j.neulet.2008.03.031

Chandrasekharan NV, 2002, P NATL ACAD SCI USA, V99, P13926, DOI 10.1073/pnas.162468699

Chen HL, 2005, ANN NEUROL, V58, P963, DOI 10.1002/ana.20682

Chen HL, 2003, ARCH NEUROL-CHICAGO, V60, P1059, DOI 10.1001/archneur.60.8.1059

Choi SH, 2009, TRENDS PHARMACOL SCI, V30, P174, DOI 10.1016/j.tips.2009.01.002

Colville-Nash PR, 2001, BIODRUGS, V15, P1, DOI 10.2165/00063030-200115010-00001

De Simone R, 2004, MOL NEUROBIOL, V29, P197, DOI 10.1385/MN:29:2:197

De Vries EFJ, 2008, NUCL MED BIOL, V35, P459, DOI 10.1016/j.nucmedbio.2008.01.005

Dehmer T, 2004, J NEUROCHEM, V88, P494, DOI 10.1046/j.1471-4159.2003.02210.x

Doorduin J, 2009, MOL IMAGING BIOL, V11, P386, DOI 10.1007/s11307-009-0211-6

Etminan M, 2008, J CLIN NEUROSCI, V15, P576, DOI 10.1016/j.jocn.2007.02.095

Etminan M, 2006, CURR DRUG SAF, V1, P223, DOI 10.2174/157488606777934404

Feng ZH, 2002, NEUROSCI LETT, V329, P354, DOI 10.1016/S0304-3940(02)00704-8

Gerhard A, 2006, NEUROBIOL DIS, V21, P404, DOI 10.1016/j.nbd.2005.08.002

Hancock DB, 2007, ARCH NEUROL-CHICAGO, V64, P576, DOI 10.1001/archneur.64.4.576

Hernán MA, 2006, NEUROLOGY, V66, P1097, DOI 10.1212/01.wnl.0000204446.82823.28

Hunter RL, 2007, J NEUROCHEM, V100, P1375, DOI 10.1111/j.1471-4159.2006.04327.x

Imaizumi M, 2008, NEUROIMAGE, V39, P1289, DOI 10.1016/j.neuroimage.2007.09.063

Jenner P, 1996, NEUROLOGY, V47, pS161, DOI 10.1212/WNL.47.6_Suppl_3.161S

Jones SC, 2005, ANN PHARMACOTHER, V39, P1249, DOI 10.1345/aph.1E654

Kaufmann WE, 1996, P NATL ACAD SCI USA, V93, P2317, DOI 10.1073/pnas.93.6.2317

Knott C, 2000, MOL CELL NEUROSCI, V16, P724, DOI 10.1006/mcne.2000.0914

Kunz A, 2007, J CEREBR BLOOD F MET, V27, P545, DOI 10.1038/sj.jcbfm.9600369

McCullough L, 2004, J NEUROSCI, V24, P257, DOI 10.1523/JNEUROSCI.4485-03.2004

MCGEER PL, 1988, NEUROLOGY, V38, P1285, DOI 10.1212/WNL.38.8.1285

Minghetti L, 2004, J NEUROPATH EXP NEUR, V63, P901, DOI 10.1093/jnen/63.9.901

Minghetti L., 2005, BRAIN RES BRAIN RES, V48, P251

Mirza B, 2000, NEUROSCIENCE, V95, P425

Niwa K, 2000, J NEUROSCI, V20, P763, DOI 10.1523/JNEUROSCI.20-02-00763.2000

Ouchi Y, 2005, ANN NEUROL, V57, P168, DOI 10.1002/ana.20338

Rohn TT, 2001, NEUROREPORT, V12, P839, DOI 10.1097/00001756-200103260-00043

Sánchez-Pernaute R, 2004, J NEUROINFLAMM, V1, DOI 10.1186/1742-2094-1-6

Shaftel SS, 2003, MOL BRAIN RES, V119, P213, DOI 10.1016/j.molbrainres.2003.09.006

Shaw KN, 2003, EUR J NEUROSCI, V17, P2438, DOI 10.1046/j.1460-9568.2003.02643.x

Silverstein FE, 2000, JAMA-J AM MED ASSOC, V284, P1247, DOI 10.1001/jama.284.10.1247

Smith WL, 2000, ANNU REV BIOCHEM, V69, P145, DOI 10.1146/annurev.biochem.69.1.145

Sriram K, 2006, J NEUROCHEM, V96, P706, DOI 10.1111/j.1471-4159.2005.03566.x

Sugama S, 2003, BRAIN RES, V964, P288, DOI 10.1016/S0006-8993(02)04085-4

Teather LA, 2002, LEARN MEMORY, V9, P41, DOI 10.1101/lm.43602

Teismann P, 2003, P NATL ACAD SCI USA, V100, P5473, DOI 10.1073/pnas.0837397100

Teismann P, 2003, MOVEMENT DISORD, V18, P121, DOI 10.1002/mds.10332

Ton TG, 2006, MOVEMENT DISORD, V21, P964, DOI 10.1002/mds.20856

Venneti S, 2008, NEUROBIOL DIS, V29, P232, DOI 10.1016/j.nbd.2007.08.016

Vijitruth R, 2006, J NEUROINFLAMM, V3, DOI 10.1186/1742-2094-3-6

Vogelgesang S, 2001, ACTA NEUROPATHOL, V102, P545

Wahner AD, 2007, NEUROLOGY, V69, P1836, DOI 10.1212/01.wnl.0000279519.99344.ad

Wang TG, 2005, FASEB J, V19, P1134, DOI 10.1096/fj.04-2457fje

Willoughby DA, 2000, NAT MED, V6, P137, DOI 10.1038/72224

YAMAGATA K, 1993, NEURON, V11, P371, DOI 10.1016/0896-6273(93)90192-T

Yasojima K, 1999, BRAIN RES, V830, P226, DOI 10.1016/S0006-8993(99)01389-X

NR 63

TC 81

Z9 86

U1 0

U2 9

PU BENTHAM SCIENCE PUBL LTD

PI SHARJAH

PA EXECUTIVE STE Y-2, PO BOX 7917, SAIF ZONE, 1200 BR SHARJAH, U ARAB

EMIRATES

SN 1570-159X

EI 1875-6190

J9 CURR NEUROPHARMACOL

JI Curr. Neuropharmacol.

PD MAR

PY 2010

VL 8

IS 1

BP 62

EP 68

DI 10.2174/157015910790909485

PG 7

WC Neurosciences; Pharmacology & Pharmacy

WE Science Citation Index Expanded (SCI-EXPANDED)

SC Neurosciences & Neurology; Pharmacology & Pharmacy

GA 568JB

UT WOS:000275517500007

PM 20808546

OA Green Published, Green Submitted

DA 2024-08-12

ER

PT J

AU Kim, ME

Park, PR

Na, JY

Jung, I

Cho, JH

Lee, JS

AF Kim, Mi Eun

Park, Pu Reum

Na, Ju Yong

Jung, Inae

Cho, Jun Hwi

Lee, Jun Sik

TI Anti-neuroinflammatory effects of galangin in LPS-stimulated BV-2

microglia through regulation of IL-1 production and the NF-B signaling

pathways

SO MOLECULAR AND CELLULAR BIOCHEMISTRY

LA English

DT Article

DE Galangin; Neuroinflammation; BV-2 microglial cells; IL-1 production

ID KAPPA-B; MEDIATED NEUROINFLAMMATION; MAPK; INFLAMMATION; INHIBITION;

INDUCTION; CELLS; INOS

AB Neuroinflammation resulting from microglial activation is involved in the pathogenesis of neurodegenerative disease, including Parkinson's diseases. Microglial activation plays an important role in neuroinflammation and contributes to several neurological disorders. Hence, inhibition of both microglial activation and the generation of pro-inflammatory cytokines may lead to an effective treatment for neurodegenerative disease. In the present study, the anti-neuroinflammatory effects of galangin were investigated in lipopolysaccharide (LPS)-stimulated BV-2 microglial cells. Galangin significantly decreased the generation of nitric oxide, interleukin-1, and inducible nitric oxide synthase in LPS-stimulated BV-2 microglial cells. In addition, galangin inhibited the phosphorylation of p38 mitogen-activated protein kinase (MAPK) and c-Jun N-terminal kinase 1/2. Furthermore, it was observed that activation of both IB- and nuclear factor kappa B (NF-B) was significantly increased following LPS stimulation, and this effect was suppressed by galangin treatment. In conclusion, galangin displayed an anti-neuroinflammatory activity in LPS-stimulated BV-2 microglial cells. Galangin inhibited LPS-induced neuroinflammation via the MAPK and NF-B signaling pathways and might act as a natural therapeutic agent for the treatment of various neuroinflammatory conditions.

C1 [Kim, Mi Eun; Park, Pu Reum; Na, Ju Yong; Jung, Inae; Cho, Jun Hwi; Lee, Jun Sik] Chosun Univ, Coll Nat Sci, Dept Life Sci, BK21 Plus Res Team Bioact Control Technol, Gwangju 61452, South Korea.

C3 Chosun University

RP Lee, JS (corresponding author), Chosun Univ, Coll Nat Sci, Dept Life Sci, BK21 Plus Res Team Bioact Control Technol, Gwangju 61452, South Korea.

EM junsiklee@chosun.ac.kr

FU Chosun University [K206212009]

FX This study was supported by research fund from Chosun University 2015

(K206212009).

CR Aloud AA, 2017, REDOX REP, V22, P290, DOI 10.1080/13510002.2016.1273437

Barlow BK, 2007, REPROD TOXICOL, V23, P457, DOI 10.1016/j.reprotox.2007.01.007

Chen WW, 2016, MOL MED REP, V13, P3391, DOI 10.3892/mmr.2016.4948

Chen YF, 2016, NEUROMOL MED, V18, P322, DOI 10.1007/s12017-016-8395-9

Chien ST, 2015, CANCER CELL INT, V15, DOI 10.1186/s12935-015-0168-2

Culbert AA, 2006, J BIOL CHEM, V281, P23658, DOI 10.1074/jbc.M513646200

Dai JN, 2011, PLOS ONE, V6, DOI [10.1371/journal.pone.0028903, 10.1371/journal.pone.0021891]

Fan H, 2015, ANTIOXID REDOX SIGN, V22, P832, DOI 10.1089/ars.2014.6022

Guo C, 2016, PHYTOMEDICINE, V23, P1629, DOI 10.1016/j.phymed.2016.10.007

Hawkes C, 2003, MOVEMENT DISORD, V18, P364, DOI 10.1002/mds.10379

Hong H, 2016, INT NEUROUROL J, V20, pS2, DOI 10.5213/inj.1632604.302

Khan MS, 2016, NEUROCHEM INT, V100, P1, DOI 10.1016/j.neuint.2016.08.005

Kim BW, 2013, PLOS ONE, V8, DOI 10.1371/journal.pone.0055792

Lai JL, 2017, INFLAMMATION, V40, P1, DOI 10.1007/s10753-016-0447-7

Lee YY, 2016, J NEUROIMMUNE PHARM, V11, P294, DOI 10.1007/s11481-016-9657-x

Lopes PC, 2016, INFLAMMOPHARMACOLOGY, V24, P291, DOI 10.1007/s10787-016-0283-2

Park EJ, 2013, FOOD CHEM TOXICOL, V55, P386, DOI 10.1016/j.fct.2012.12.027

Park J, 2015, NEUROSCI LETT, V584, P191, DOI 10.1016/j.neulet.2014.10.016

Park SY, 2016, FOOD CHEM TOXICOL, V97, P159, DOI 10.1016/j.fct.2016.09.010

Parsa R, 2016, GLIA, V64, P1925, DOI 10.1002/glia.23033

Raposo C, 2013, MEDIAT INFLAMM, V2013, DOI 10.1155/2013/321460

Ren KW, 2016, BIOMED PHARMACOTHER, V84, P1748, DOI 10.1016/j.biopha.2016.10.111

Schain M, 2017, CURR NEUROL NEUROSCI, V17, DOI 10.1007/s11910-017-0733-2

Schmidt AF, 2016, J NEUROINFLAMM, V13, DOI 10.1186/s12974-016-0706-4

Tiwari PC, 2017, DIALOGUES CLIN NEURO, V19, P71

Walter L, 2009, SEMIN IMMUNOPATHOL, V31, P513, DOI 10.1007/s00281-009-0180-5

Wang W, 2017, SCI REP-UK, V7, DOI 10.1038/srep44252

Wang XH, 2013, ENVIRON TOXICOL PHAR, V36, P288, DOI 10.1016/j.etap.2013.04.004

Zablocka Agnieszka, 2006, Postepy Hig Med Dosw (Online), V60, P209

Zhou MA, 2013, BRAIN RES, V1506, P105, DOI 10.1016/j.brainres.2013.02.010

NR 30

TC 37

Z9 42

U1 4

U2 26

PU SPRINGER

PI DORDRECHT

PA VAN GODEWIJCKSTRAAT 30, 3311 GZ DORDRECHT, NETHERLANDS

SN 0300-8177

EI 1573-4919

J9 MOL CELL BIOCHEM

JI Mol. Cell. Biochem.

PD JAN

PY 2019

VL 451

IS 1-2

BP 145

EP 153

DI 10.1007/s11010-018-3401-1

PG 9

WC Cell Biology

WE Science Citation Index Expanded (SCI-EXPANDED)

SC Cell Biology

GA HJ2BR

UT WOS:000456971000014

PM 29995265

DA 2024-08-12

ER

PT J

AU Malinovskaya, NA

Salmina, AB

Prokopenko, SV

Morgun, AV

Kuvacheva, NV

Panina, YA

Shilina, EV

AF Malinovskaya, N. A.

Salmina, A. B.

Prokopenko, S. V.

Morgun, A. V.

Kuvacheva, N. V.

Panina, Yu. A.

Shilina, E. V.

TI The coexpression of CD157/CD11b/CD18 in an experimental model of

Parkinson's disease

SO NEUROCHEMICAL JOURNAL

LA English

DT Article

DE MAC-1; CD157; microglia; neuroinflammation; neurodegeneration; rotenone

model; Parkinson's disease

ID CD157; NEURODEGENERATION; GENE; CD38

AB Neurodegeneration is progressive neuronal death. Research on neuroinflammation mechanisms is necessary for understanding of the pathogenesis of acute and chronical neurodegeneration. The purpose of this research was to evaluate the coexpression of neuroinflammation molecules (CD157, CD11b/CD18) in the midbrain of animals under physiological conditions and in an experimental model of Parkinson's disease. Immunohistochemical evaluation of the coexpression of CD157 and MAC-1 was performed in rat midbrain sections. A significant increase in the number of cells that coexpress CD157/MAC-1 (CD11b/CD18) was found in the midbrain during Parkinson's disease. The possible roles of the neuroinflammatory molecules CD157 and MAC-1 in the pathogenesis of Parkinson's disease may include activation of microglial cells and performance of the neuroinflammatory response.

C1 [Malinovskaya, N. A.; Salmina, A. B.; Prokopenko, S. V.; Morgun, A. V.; Kuvacheva, N. V.; Panina, Yu. A.; Shilina, E. V.] Voyno Yasenetsky Krasnoyarsk State Med Univ, Minist Hlth, Krasnoyarsk, Russia.

C3 Krasnoyarsk State Medical University; Ministry of Health of the Russian

Federation

RP Malinovskaya, NA (corresponding author), Ul Partizana Zheleznyaka 1, Krasnoyarsk 660022, Russia.

EM reg.kgmu@gmail.com

RI Salmina, Alla B./L-7977-2013; Malinovskaya, Nataliya/AAN-4918-2020;

Prokopenko, Semen V./I-1651-2017; Panina, Yulia/neurodegenerative disease-8439-2020; Morgun,

Andrey V/AAN-2770-2020

OI Malinovskaya, Nataliya/0000-0002-0033-3804; Panina,

Yulia/0000-0002-8675-3489; Morgun, Andrey V/0000-0002-9644-5500

CR Funaro A, 2009, FRONT BIOSCI-LANDMRK, V14, P929, DOI 10.2741/3287

Gao HM, 2011, J NEUROSCI, V31, P1081, DOI 10.1523/JNEUROSCI.3732-10.2011

Gavrikova V.V., 2011, SIBIRSKOE MED OBOZRE, P59

Katunina EA, 2009, ZH NEVROL PSIKHIATR, V109, P76

Klein J, 2000, J NEURAL TRANSM, V107, P1027, DOI 10.1007/s007020070051

Lapchak PA, 2012, EXPERT REV MED DEVIC, V9, P71, DOI 10.1586/ERD.11.64

Lopatina O, 2014, FRONT BEHAV NEUROSCI, V8, DOI 10.3389/fnbeh.2014.00133

Ortolan E, 2002, CELL BIOCHEM FUNCT, V20, P309, DOI 10.1002/cbf.978

Quarona V, 2013, CYTOM PART B-CLIN CY, V84B, P207, DOI 10.1002/cyto.b.21092

Roebuck KA, 1999, J LEUKOCYTE BIOL, V66, P876, DOI 10.1002/jlb.66.6.876

Sutherland DR, 2014, CYTOM PART B-CLIN CY, V86, P44, DOI 10.1002/cyto.b.21111

Whitton PS, 2007, BRIT J PHARMACOL, V150, P963, DOI 10.1038/sj.bjp.0707167

NR 12

TC 2

Z9 2

U1 0

U2 10

PU MAIK NAUKA/INTERPERIODICA/SPRINGER

PI NEW YORK

PA 233 SPRING ST, NEW YORK, NY 10013-1578 USA

SN 1819-7124

EI 1819-7132

J9 NEUROCHEM J+

JI Neurochem. J.

PD OCT

PY 2015

VL 9

IS 4

BP 279

EP 283

DI 10.1134/S181971241504011X

PG 5

WC Neurosciences

WE Science Citation Index Expanded (SCI-EXPANDED)

SC Neurosciences & Neurology

GA CX7HV

UT WOS:000365873700008

DA 2024-08-12

ER

PT J

AU Yadav, H

Jaldhi

Bhardwaj, R

Anamika

Bakshi, A

Gupta, S

Maurya, SK

AF Yadav, Himanshi

Jaldhi

Bhardwaj, Rati

Anamika

Bakshi, Amrita

Gupta, Suchi

Maurya, Shashank Kumar

TI Unveiling the role of gut-brain axis in regulating neurodegenerative

diseases: A comprehensive review

SO LIFE SCIENCES

LA English

DT Review

DE Brain; Gut; Microbiome; Neurodegeneration; Neuroinflammation

ID CHAIN FATTY-ACIDS; INFLAMMATION-INDUCED DEPRESSION;

TRIMETHYLAMINE-N-OXIDE; CENTRAL-NERVOUS-SYSTEM; GERM-FREE-MICE;

BILE-ACIDS; NEUROTROPHIC FACTOR; parkinson's disease; AMINO-ACIDS;

INDOLEAMINE 2,3-DIOXYGENASE

AB Emerging evidence have shown the importance of gut microbiota in regulating brain functions. The diverse molecular mechanisms involved in cross-talk between gut and brain provide insight into importance of this communication in maintenance of brain homeostasis. It has also been observed that disturbed gut microbiota contributes to neurological diseases such as Alzheimer's disease, Parkinson's disease, multiple sclerosis, amyotrophic lateral sclerosis and aging. Recently, gut microbiome-derived exosomes have also been reported to play an essential role in the development and progression of neurodegenerative disease and could thereby act as a therapeutic target. Further, pharmacological interventions including antibiotics, prebiotics and probiotics can influence gut microbiome-mediated management of neurological diseases. However, extensive research is warranted to better comprehend this interconnection in maintenance of brain homeostasis and its implication in neurological diseases. Thus, the present review is aimed to provide a detailed understanding of gut-brain axis followed by possibilities to target the gut microbiome for improving neurological health.

C1 [Yadav, Himanshi; Jaldhi; Maurya, Shashank Kumar] Univ Delhi, Fac Sci, Dept Zool, Biochem & Mol Biol Lab, Delhi 110007, India.

[Bhardwaj, Rati] Delhi Tech Univ, Dept Biotechnol, Delhi, India.

[Anamika; Bakshi, Amrita] Univ Delhi, Ramjas Coll, Dept Zool, Delhi, India.

[Gupta, Suchi] All India Inst Med Sci, Tech Cell Innovat Pvt Ltd, Ctr Med Innovat & Entrepreneurship CMIE, New Delhi, India.

C3 University of Delhi; Delhi Technological University; University of

Delhi; All India Institute of Medical Sciences (AIIMS) New Delhi

RP Maurya, SK (corresponding author), Univ Delhi, Fac Sci, Dept Zool, Biochem & Mol Biol Lab, Delhi 110007, India.

EM smaurya1@zoology.du.ac.in

OI MAURYA, SHASHANK KUMAR/0000-0002-4681-2691; ,

Anamika/0000-0002-0013-7547

FU Institution of Eminence, University of Delhi, University of Delhi, Delhi

[IOE/2021/12/FRP]; Department of Health and Research, Delhi [R.12014/54/

2022-HR]; Department of Biotechnology, Delhi Technical University, Delhi

FX SKM and AB acknowledge financial support from the Institution of

Eminence, University of Delhi (IOE/2021/12/FRP), University of Delhi,

Delhi and Department of Health and Research (File No.R.12014/54/

2022-HR), Delhi, respectively. RB acknowledges guidance and support from

the Department of Biotechnology, Delhi Technical University, Delhi. AB

and Anamika acknowledge Ramjas College, University of Delhi, Delhi for

its continuous support and motivation. Figures were created using

Biorender.com.

CR Abbott NJ, 2000, CELL MOL NEUROBIOL, V20, P131, DOI 10.1023/A:1007074420772

Abot A, 2021, GUT, V70, P1078, DOI 10.1136/gutjnl-2019-320230

Agus A, 2018, CELL HOST MICROBE, V23, P716, DOI 10.1016/j.chom.2018.05.003

Ait-Belgnaoui A, 2014, NEUROGASTROENT MOTIL, V26, P510, DOI 10.1111/nmo.12295

Al-Lahham SH, 2010, BBA-MOL CELL BIOL L, V1801, P1175, DOI 10.1016/j.bbalip.2010.07.007

Al-Sarraf H, 2002, DEV BRAIN RES, V139, P121, DOI 10.1016/S0165-3806(02)00537-0

ALTSCHULER SM, 1993, GASTROENTEROLOGY, V104, P502, DOI 10.1016/0016-5085(93)90419-D

Amenta F, 2008, CURR MED CHEM, V15, P488, DOI 10.2174/092986708783503203

Arentsen T, 2017, MOL PSYCHIATR, V22, P257, DOI 10.1038/mp.2016.182

Arentsen T, 2018, BRAIN BEHAV IMMUN, V67, P345, DOI 10.1016/j.bbi.2017.09.014

Arpaia N, 2013, NATURE, V504, P451, DOI 10.1038/nature12726

Atzori Marco, 2016, Front Synaptic Neurosci, V8, P25, DOI 10.3389/fnsyn.2016.00025

BACHMANN C, 1979, CLIN CHIM ACTA, V92, P153, DOI 10.1016/0009-8981(79)90109-8

Badawy AAB, 2017, NEUROPHARMACOLOGY, V112, P248, DOI 10.1016/j.neuropharm.2015.11.015

Bairamian D, 2022, MOL NEURODEGENER, V17, DOI 10.1186/s13024-022-00522-2

Bak LK, 2006, J NEUROCHEM, V98, P641, DOI 10.1111/j.1471-4159.2006.03913.x

Barrett E, 2012, J APPL MICROBIOL, V113, P411, DOI 10.1111/j.1365-2672.2012.05344.x

Barroso A, 2021, CELL MOL IMMUNOL, V18, P259, DOI 10.1038/s41423-020-00585-5

Bennett BJ, 2013, CELL METAB, V17, P49, DOI 10.1016/j.cmet.2012.12.011

Bercik P, 2011, GASTROENTEROLOGY, V141, P599, DOI 10.1053/j.gastro.2011.04.052

Berer K, 2017, P NATL ACAD SCI USA, V114, P10719, DOI 10.1073/pnas.1711233114

Bereswill S, 2010, PLOS ONE, V5, DOI 10.1371/journal.pone.0015099

Berger M, 2009, ANNU REV MED, V60, P355, DOI 10.1146/annurev.med.60.042307.110802

BERGMAN EN, 1990, PHYSIOL REV, V70, P567, DOI 10.1152/physrev.1990.70.2.567

Bettcher BM, 2021, NAT REV NEUROL, V17, P689, DOI 10.1038/s41582-021-00549-x

Bischoff SC, 2014, BMC GASTROENTEROL, V14, DOI 10.1186/s12876-014-0189-7

Bjurström H, 2008, J NEUROIMMUNOL, V205, P44, DOI 10.1016/j.jneuroim.2008.08.017

Blacher E, 2019, NATURE, V572, P474, DOI 10.1038/s41586-019-1443-5

Boddy SL, 2021, BMC MED, V19, DOI 10.1186/s12916-020-01885-3

Bogunovic M, 2007, AM J PHYSIOL-GASTR L, V292, pG1770, DOI 10.1152/ajpgi.00249.2006

Bosco N, 2021, GENES IMMUN, V22, P289, DOI 10.1038/s41435-021-00126-8

Bouskra D, 2008, NATURE, V456, P507, DOI 10.1038/nature07450

Braniste V, 2014, SCI TRANSL MED, V6, DOI 10.1126/scitranslmed.3009759

Bravo JA, 2011, P NATL ACAD SCI USA, V108, P16050, DOI 10.1073/pnas.1102999108

Brosnan JT, 2006, J NUTR, V136, p1636S, DOI 10.1093/jn/136.6.1636S

Brown AJ, 2003, J BIOL CHEM, V278, P11312, DOI 10.1074/jbc.M211609200

Brun P, 2015, MOL CELL NEUROSCI, V68, P24, DOI 10.1016/j.mcn.2015.03.018

Budhwani M, 2018, FRONT ONCOL, V8, DOI 10.3389/fonc.2018.00322

Burbidge K, 2022, AUTOPHAGY, V18, P1020, DOI 10.1080/15548627.2021.1967615

Calabresi P, 2007, TRENDS NEUROSCI, V30, P211, DOI 10.1016/j.tins.2007.03.001

Carabotti M, 2015, ANN GASTROENTEROL, V28, P203

Caspani G, 2022, INT J MOL SCI, V23, DOI 10.3390/ijms23063259

Caspani G, 2019, CURR OPIN PHARMACOL, V48, P99, DOI 10.1016/j.coph.2019.08.001

Cervantes-Barragan L, 2017, SCIENCE, V357, P806, DOI 10.1126/science.aah5825

Chaplin A, 2018, NUTRIENTS, V10, DOI 10.3390/nu10111651

Chelakkot C, 2018, EXP MOL MED, V50, DOI 10.1038/emm.2017.282

Chen GF, 2017, ACTA PHARMACOL SIN, V38, P1205, DOI 10.1038/aps.2017.28

Chen J, 2016, SCI REP-UK, V6, DOI 10.1038/srep28484

Chen K, 2019, CELL HOST MICROBE, V25, P537, DOI 10.1016/j.chom.2019.02.003

Chen QY, 2021, FRONT CELL DEV BIOL, V9, DOI 10.3389/fcell.2021.718803

Chen XD, 2021, MOL NEUROBIOL, V58, P4828, DOI 10.1007/s12035-021-02444-4

Chen-Roetling Jing, 2014, BMC Res Notes, V7, P561, DOI 10.1186/1756-0500-7-561

Cherbut C, 1998, AM J PHYSIOL-GASTR L, V275, pG1415, DOI 10.1152/ajpgi.1998.275.6.G1415

Chevalier AC, 2017, J NEUROCHEM, V141, P721, DOI 10.1111/jnc.14032

Chiang JYL, 2013, COMPR PHYSIOL, V3, P1191, DOI 10.1002/cphy.c120023

Chiang JYL, 2009, J LIPID RES, V50, P1955, DOI 10.1194/jlr.R900010-JLR200

Chung JY, 2020, FRONT AGING NEUROSCI, V12, DOI 10.3389/fnagi.2020.588044

dos Santos JCC, 2023, AGEING RES REV, V84, DOI 10.1016/j.arr.2022.101812

Conway J, 2021, AGEING RES REV, V68, DOI 10.1016/j.arr.2021.101323

Cook SI, 1998, ALIMENT PHARM THER, V12, P499, DOI 10.1046/j.1365-2036.1998.00337.x

Costantini L, 2017, INT J MOL SCI, V18, DOI 10.3390/ijms18122645

Cryan JF, 2005, TRENDS PHARMACOL SCI, V26, P36, DOI 10.1016/j.tips.2004.11.004

Cryan JF, 2015, NAT REV GASTRO HEPAT, V12, P495, DOI 10.1038/nrgastro.2015.127

Cryan JF, 2012, NAT REV NEUROSCI, V13, P701, DOI 10.1038/nrn3346

Cuesta CM, 2021, INT J MOL SCI, V22, DOI 10.3390/ijms22084235

Cui J, 2014, HUM VACC IMMUNOTHER, V10, P3270, DOI 10.4161/21645515.2014.979640

da Silva FL, 2013, NEUROCHEM INT, V62, P103, DOI 10.1016/j.neuint.2012.10.001

Dai ZL, 2010, AMINO ACIDS, V39, P1201, DOI 10.1007/s00726-010-0556-9

Dantzer R, 2008, NAT REV NEUROSCI, V9, P46, DOI 10.1038/nrn2297

Dantzer R, 2017, CURR TOP BEHAV NEURO, V31, P117, DOI 10.1007/7854_2016_6

De Palma G, 2015, NAT COMMUN, V6, DOI 10.1038/ncomms8735

De Preter V, 2011, ALIMENT PHARM THER, V34, P526, DOI 10.1111/j.1365-2036.2011.04757.x

De Vadder F, 2014, CELL, V156, P84, DOI 10.1016/j.cell.2013.12.016

de Vos WM, 2022, GUT, V71, P1020, DOI 10.1136/gutjnl-2021-326789

Dehhaghi M, 2019, INT J TRYPTOPHAN RES, V12, DOI 10.1177/1178646919852996

Del Rio D, 2017, NUTRIENTS, V9, DOI 10.3390/nu9101053

den Besten G, 2013, J LIPID RES, V54, P2325, DOI 10.1194/jlr.R036012

Dendrou CA, 2015, NAT REV IMMUNOL, V15, P545, DOI 10.1038/nri3871

Deng HY, 2020, AGING-US, V12, P4010, DOI 10.18632/aging.102810

Diamond B, 2011, BIOESSAYS, V33, P588, DOI 10.1002/bies.201100042

Díaz-Garrido N, 2021, J EXTRACELL VESICLES, V10, DOI 10.1002/jev2.12161

Díez-Sainz E, 2022, J PHYSIOL BIOCHEM, V78, P485, DOI 10.1007/s13105-021-00837-6

Dinan TG, 2017, J PHYSIOL-LONDON, V595, P489, DOI 10.1113/JP273106

Donlon E, 2021, MOV DISORD CLIN PRAC, V8, P1129, DOI 10.1002/mdc3.13321

Dumas ME, 2017, CELL REP, V20, P136, DOI 10.1016/j.celrep.2017.06.039

Duque GA, 2014, FRONT IMMUNOL, V5, P1, DOI 10.3389/fimmu.2014.00491

Dürk T, 2005, INT IMMUNOL, V17, P599, DOI 10.1093/intimm/dxh242

Egerod KL, 2018, MOL METAB, V12, P62, DOI 10.1016/j.molmet.2018.03.016

Eisenhofer G, 1997, J CLIN ENDOCR METAB, V82, P3864, DOI 10.1210/jc.82.11.3864

Erny D, 2015, NAT NEUROSCI, V18, P965, DOI 10.1038/nn.4030

Feng WH, 2017, BBA-GEN SUBJECTS, V1861, P1801, DOI 10.1016/j.bbagen.2017.03.017

Fernstrom JD, 2005, J NUTR, V135, p1539S, DOI 10.1093/jn/135.6.1539S

Finglas PM, 2003, P NUTR SOC, V62, P591, DOI 10.1079/PNS2003271

Foong JPP, 2020, AM J PHYSIOL-GASTR L, V319, pG541, DOI 10.1152/ajpgi.00288.2020

Forsythe P, 2014, ADV EXP MED BIOL, V817, P115, DOI 10.1007/978-1-4939-0897-4_5

Foster JA, 2017, NEUROBIOL STRESS, V7, P124, DOI 10.1016/j.ynstr.2017.03.001

Fransen F, 2017, FRONT IMMUNOL, V8, DOI 10.3389/fimmu.2017.01385

Frost G, 2014, NAT COMMUN, V5, DOI 10.1038/ncomms4611

Fung TC, 2017, NAT NEUROSCI, V20, P145, DOI 10.1038/nn.4476

Furness JB, 2012, NAT REV GASTRO HEPAT, V9, P286, DOI 10.1038/nrgastro.2012.32

Furusawa Y, 2013, NATURE, V504, P446, DOI 10.1038/nature12721

Ortiz GG, 2014, ARCH MED RES, V45, P687, DOI 10.1016/j.arcmed.2014.11.013

Galland L, 2014, J MED FOOD, V17, P1261, DOI 10.1089/jmf.2014.7000

Gao K, 2019, J NEUROCHEM, V149, P641, DOI 10.1111/jnc.14709

Gao K, 2018, J NEUROCHEM, V146, P219, DOI 10.1111/jnc.14333

Gareau MG, 2011, GUT, V60, P307, DOI 10.1136/gut.2009.202515

Gautier EL, 2012, NAT IMMUNOL, V13, P1118, DOI 10.1038/ni.2419

Gerhardt S, 2018, NUTRIENTS, V10, DOI 10.3390/nu10060708

Gershon MD, 2013, CURR OPIN ENDOCRINOL, V20, P14, DOI 10.1097/MED.0b013e32835bc703

Giau VV, 2018, NUTRIENTS, V10, DOI 10.3390/nu10111765

Gibson GR, 2017, NAT REV GASTRO HEPAT, V14, P491, DOI 10.1038/nrgastro.2017.75

Giles EM, 2017, INFLAMM BOWEL DIS, V23, P524, DOI 10.1097/MIB.0000000000001078

Gillam EMJ, 2000, BIOCHEMISTRY-US, V39, P13817, DOI 10.1021/bi001229u

Glebov K, 2015, GLIA, V63, P626, DOI 10.1002/glia.22772

Goehler LE, 2005, BRAIN BEHAV IMMUN, V19, P334, DOI 10.1016/j.bbi.2004.09.002

Gorski JA, 2003, J NEUROSCI, V23, P6856

LeBlanc JG, 2013, CURR OPIN BIOTECH, V24, P160, DOI 10.1016/j.copbio.2012.08.005

Haas-Neill S, 2020, INT J MOL SCI, V21, DOI 10.3390/ijms21238899

Haberland M, 2009, NAT REV GENET, V10, P32, DOI 10.1038/nrg2485

Haghighat N, 2021, NUTR NEUROSCI, V24, P490, DOI 10.1080/1028415X.2019.1646975

Haghikia A, 2015, IMMUNITY, V43, P817, DOI 10.1016/j.immuni.2015.09.007

Harrison IF, 2013, PHARMACOL THERAPEUT, V140, P34, DOI 10.1016/j.pharmthera.2013.05.010

Hayden MS, 2011, CELL RES, V21, P223, DOI 10.1038/cr.2011.13

Heijtza RD, 2011, P NATL ACAD SCI USA, V108, P3047, DOI 10.1073/pnas.1010529108

Hemarajata P, 2013, THER ADV GASTROENTER, V6, P39, DOI 10.1177/1756283X12459294

Ho L, 2018, EXPERT REV NEUROTHER, V18, P83, DOI 10.1080/14737175.2018.1400909

Hoban AE, 2016, NEUROSCIENCE, V339, P463, DOI 10.1016/j.neuroscience.2016.10.003

Hofmann AF, 1999, ARCH INTERN MED, V159, P2647, DOI 10.1001/archinte.159.22.2647

Hooper LV, 2004, TRENDS MICROBIOL, V12, P129, DOI 10.1016/j.tim.2004.01.001

Horiuchi Y, 2003, LIFE SCI, V72, P1745, DOI 10.1016/S0024-3205(02)02478-5

Hou KJ, 2022, SIGNAL TRANSDUCT TAR, V7, DOI 10.1038/s41392-022-00974-4

Houtman TA, 2022, SCI REP-UK, V12, DOI 10.1038/s41598-022-07176-6

HOVERSTAD T, 1986, J NUTR, V116, P1772, DOI 10.1093/jn/116.9.1772

Hu J, 2016, FRONT MICROBIOL, V7, DOI 10.3389/fmicb.2016.01727

Huang EJ, 2003, ANNU REV BIOCHEM, V72, P609, DOI 10.1146/annurev.biochem.72.121801.161629

Humann J, 2016, CELL HOST MICROBE, V19, P388, DOI [10.1016/j.chom.2016.05.017, 10.1016/j.chom.2016.02.009]

Hyland NP, 2016, DEV BIOL, V417, P182, DOI 10.1016/j.ydbio.2016.06.027

Inazu M, 2019, NUTRIENTS, V11, DOI 10.3390/nu11102265

Jaglin M, 2018, FRONT NEUROSCI-SWITZ, V12, DOI 10.3389/fnins.2018.00216

Jameson KG, 2020, MOL CELL, V78, P577, DOI 10.1016/j.molcel.2020.03.006

Jang HM, 2018, MUCOSAL IMMUNOL, V11, P1386, DOI 10.1038/s41385-018-0042-3

Jang SE, 2018, MUCOSAL IMMUNOL, V11, P369, DOI 10.1038/mi.2017.49

Jangi S, 2016, NAT COMMUN, V7, DOI 10.1038/ncomms12015

Jenkins TA, 2016, NUTRIENTS, V8, DOI 10.3390/nu8010056

Jeon SW, 2017, J NEUROIMMUNOL, V313, P92, DOI 10.1016/j.jneuroim.2017.10.016

Olguín HJ, 2016, OXID MED CELL LONGEV, V2016, DOI 10.1155/2016/9730467

Kaczmarczyk R, 2018, J NEUROCHEM, V146, P76, DOI 10.1111/jnc.14284

Kaelberer MM, 2020, ANNU REV NEUROSCI, V43, P337, DOI 10.1146/annurev-neuro-091619-022657

Kaelberer MM, 2018, SCIENCE, V361, P1219, DOI 10.1126/science.aat5236

Kang DW, 2017, MICROBIOME, V5, DOI 10.1186/s40168-016-0225-7

Kelly JR, 2017, FRONT NEUROSCI-SWITZ, V11, DOI 10.3389/fnins.2017.00490

Kelly JR, 2016, J PSYCHIATR RES, V82, P109, DOI 10.1016/j.jpsychires.2016.07.019

Keszthelyi D, 2009, NEUROGASTROENT MOTIL, V21, P1239, DOI 10.1111/j.1365-2982.2009.01370.x

Kim YK, 2021, J CLIN MED, V10, DOI 10.3390/jcm10173819

Kimura I, 2011, P NATL ACAD SCI USA, V108, P8030, DOI 10.1073/pnas.1016088108

Kinney Jefferson W, 2018, Alzheimers Dement (N Y), V4, P575, DOI 10.1016/j.trci.2018.06.014

Knauf C, 2020, NEUROENDOCRINOLOGY, V110, P139, DOI 10.1159/000500602

Koeth RA, 2013, NAT MED, V19, P576, DOI 10.1038/nm.3145

Konsman JP, 2000, EUR J NEUROSCI, V12, P4434, DOI 10.1046/j.0953-816X.2000.01319.x

Konsoula Z, 2012, J PHARMACOL TOX MET, V66, P215, DOI 10.1016/j.vascn.2012.08.001

Koussoulas K, 2018, FRONT PHYSIOL, V9, DOI 10.3389/fphys.2018.00097

Koutzoumis DN, 2020, EXP NEUROL, V325, DOI 10.1016/j.expneurol.2019.113159

Krantis A, 2000, NEWS PHYSIOL SCI, V15, P284

Kratsman N, 2016, NEUROPHARMACOLOGY, V102, P136, DOI 10.1016/j.neuropharm.2015.11.003

Krnjevic K, 1997, CAN J PHYSIOL PHARM, V75, P439, DOI 10.1139/cjpp-75-5-439

LANGOHR HD, 1981, J NEUROL, V225, P95, DOI 10.1007/BF00313323

Lassmann H, 2018, CSH PERSPECT MED, V8, DOI 10.1101/cshperspect.a028936

Laumet G, 2017, BRAIN BEHAV IMMUN, V66, P94, DOI 10.1016/j.bbi.2017.07.008

Laye Sophie, 1995, American Journal of Physiology, V268, pR1327

Leblhuber F, 2021, NUTRIENTS, V13, DOI 10.3390/nu13020361

Lee KE, 2020, MICROBIOME, V8, DOI 10.1186/s40168-020-00881-2

Lee SE, 2019, ARCH PHARM RES, V42, P1031, DOI 10.1007/s12272-019-01196-z

Li M, 2008, P NATL ACAD SCI USA, V105, P2117, DOI 10.1073/pnas.0712038105

Li W, 2017, SCI CHINA LIFE SCI, V60, P1223, DOI 10.1007/s11427-016-9001-4

Liu JM, 2015, BIOMED RES INT, V2015, DOI 10.1155/2015/412946

Liu SH, 2020, NEUROPSYCH DIS TREAT, V16, P859, DOI 10.2147/NDT.S243551

López-Figueroa AL, 2004, BIOL PSYCHIAT, V55, P225, DOI 10.1016/j.biopsych.2003.09.017

Lovenberg W, 1968, Adv Pharmacol, V6, P21

Lowe PP, 2020, ALCOHOL CLIN EXP RES, V44, P567, DOI 10.1111/acer.14272

Lu B, 2013, NAT REV NEUROSCI, V14, P401, DOI 10.1038/nrn3505

Luczynski P, 2016, INT J NEUROPSYCHOPH, V19, DOI 10.1093/ijnp/pyw020

Luheshi GN, 2000, AUTON NEUROSCI-BASIC, V85, P127, DOI 10.1016/S1566-0702(00)00231-9

Lukhele S, 2019, SEMIN IMMUNOL, V43, DOI 10.1016/j.smim.2019.05.001

Lupachyk S, 2013, DIABETES, V62, P944, DOI 10.2337/db12-0716

Luqman A, 2018, CELL REP, V22, P535, DOI 10.1016/j.celrep.2017.12.058

Ma QQ, 2019, J NEUROINFLAMM, V16, DOI 10.1186/s12974-019-1434-3

MacFabe Derrick F., 2015, Microbial Ecology in Health and Disease, V26, P28177, DOI 10.3402/mehd.v26.28177

Macia L, 2015, NAT COMMUN, V6, DOI 10.1038/ncomms7734

Mahgoub M, 2014, LEARN MEMORY, V21, P564, DOI 10.1101/lm.036012.114

Maier TV, 2017, MBIO, V8, DOI [10.1128/mBio.01343-17, 10.1128/mbio.01343-17]

Mandic AD, 2019, SCI REP-UK, V9, DOI 10.1038/s41598-018-38018-z

Masanta WO, 2013, CLIN DEV IMMUNOL, DOI 10.1155/2013/526860

Matcovitch-Natan O, 2016, SCIENCE, V353, DOI 10.1126/science.aad8670

Matsumoto M, 2017, PLOS ONE, V12, DOI 10.1371/journal.pone.0169207

Mayer EA, 2015, J CLIN INVEST, V125, P926, DOI 10.1172/JCI76304

Mayer EA, 2014, GASTROENTEROLOGY, V146, P1500, DOI 10.1053/j.gastro.2014.02.037

McFall-Ngai M, 2013, P NATL ACAD SCI USA, V110, P3229, DOI 10.1073/pnas.1218525110

Mead RJ, 2011, PLOS ONE, V6, DOI 10.1371/journal.pone.0023244

Medvedev A, 2022, FRONT BEHAV NEUROSCI, V16, DOI 10.3389/fnbeh.2022.922274

Mehrabadi Shima, 2020, Iranian Biomedical Journal, V24, P220, DOI 10.29252/ibj.24.4.220

Meneses G, 2016, J INFLAMM-LOND, V13, DOI 10.1186/s12950-016-0140-5

Mestre L, 2019, FRONT IMMUNOL, V10, DOI 10.3389/fimmu.2019.01374

Miller BR, 2015, CURR OPIN NEUROBIOL, V30, P51, DOI 10.1016/j.conb.2014.08.012

Möhle L, 2016, CELL REP, V15, P1945, DOI 10.1016/j.celrep.2016.04.074

Möhler H, 2012, NEUROPHARMACOLOGY, V62, P42, DOI 10.1016/j.neuropharm.2011.08.040

Moreau M, 2005, J INFECT DIS, V192, P537, DOI 10.1086/431603

Moresco EMY, 2011, CURR BIOL, V21, pR488, DOI 10.1016/j.cub.2011.05.039

Morowitz MJ, 2011, SURG CLIN N AM, V91, P771, DOI 10.1016/j.suc.2011.05.001

Mucida D, 2007, SCIENCE, V317, P256, DOI 10.1126/science.1145697

Müller N, 2007, J NEURAL TRANSM-SUPP, P269

Mukherjee T, 2019, ARCH BIOCHEM BIOPHYS, V670, P69, DOI 10.1016/j.abb.2018.12.022

Nagahara AH, 2009, NAT MED, V15, P331, DOI 10.1038/nm.1912

Natale G, 2019, CELLS-BASEL, V8, DOI 10.3390/cells8101124

Neis EPJG, 2015, NUTRIENTS, V7, P2930, DOI 10.3390/nu7042930

Nelson PT, 2009, J NEUROPATH EXP NEUR, V68, P1, DOI 10.1097/NEN.0b013e3181919a48

Neufeld KAM, 2015, NEUROGASTROENT MOTIL, V27, P627, DOI 10.1111/nmo.12534

Neufeld KM, 2011, NEUROGASTROENT MOTIL, V23, DOI 10.1111/j.1365-2982.2010.01620.x

Neufeld Karen-Anne M, 2011, Commun Integr Biol, V4, P492, DOI 10.4161/cib.4.4.15702

Nicholson JK, 2012, SCIENCE, V336, P1262, DOI 10.1126/science.1223813

Nogal A, 2021, GUT MICROBES, V13, DOI 10.1080/19490976.2021.1897212

Nohr MK, 2013, ENDOCRINOLOGY, V154, P3552, DOI 10.1210/en.2013-1142

Novarino G, 2012, SCIENCE, V338, P394, DOI 10.1126/science.1224631

O'Donnell J, 2012, NEUROCHEM RES, V37, P2496, DOI 10.1007/s11064-012-0818-x

O'Mahony SM, 2015, BEHAV BRAIN RES, V277, P32, DOI 10.1016/j.bbr.2014.07.027

O'Riordan KJ, 2022, MOL CELL ENDOCRINOL, V546, DOI 10.1016/j.mce.2022.111572

Ogbonnaya ES, 2015, BIOL PSYCHIAT, V78, pE7, DOI 10.1016/j.biopsych.2014.12.023

Ostaff MJ, 2013, EMBO MOL MED, V5, P1465, DOI 10.1002/emmm.201201773

Owen RT, 2013, DRUG TODAY, V49, P791, DOI 10.1358/dot.2013.49.12.2086138

Pajares M, 2020, CELLS-BASEL, V9, DOI 10.3390/cells9071687

Park H, 2013, NAT REV NEUROSCI, V14, P7, DOI 10.1038/nrn3379

Parker A, 2020, GUT MICROBES, V11, P135, DOI 10.1080/19490976.2019.1638722

Patnala R, 2017, MOL NEUROBIOL, V54, P6391, DOI 10.1007/s12035-016-0149-z

Pedroso JAB, 2015, NUTRIENTS, V7, P3914, DOI 10.3390/nu7053914

Philpott DJ, 2014, NAT REV IMMUNOL, V14, P9, DOI 10.1038/nri3565

Picciotto MR, 2012, NEURON, V76, P116, DOI 10.1016/j.neuron.2012.08.036

Pirolli NH, 2021, ADV BIOL-GER, V5, DOI 10.1002/adbi.202000540

Pittenger Christopher, 2013, Dialogues Clin Neurosci, V15, P455

PRECHTL JC, 1990, ANAT EMBRYOL, V181, P101

Priyadarshini M, 2015, MOL ENDOCRINOL, V29, P1055, DOI 10.1210/me.2015-1007

Ramos B, 2006, NEUROBIOL AGING, V27, P1658, DOI 10.1016/j.neurobiolaging.2005.09.022

Rao M, 2016, NAT REV GASTRO HEPAT, V13, DOI 10.1038/nrgastro.2016.107

Raskov H, 2016, GUT MICROBES, V7, P365, DOI 10.1080/19490976.2016.1218585

Rath S, 2017, MICROBIOME, V5, DOI 10.1186/s40168-017-0271-9

Rea IM, 2018, FRONT IMMUNOL, V9, DOI 10.3389/fimmu.2018.00586

Reddy Doodipala Samba, 2018, Curr Protoc Pharmacol, V81, pe41, DOI 10.1002/cpph.41

Rehman A, 2001, CYTOKINE, V13, P8, DOI 10.1006/cyto.2000.0800

Reigstad CS, 2015, FASEB J, V29, P1395, DOI 10.1096/fj.14-259598

Ressler KJ, 2007, NAT NEUROSCI, V10, P1116, DOI 10.1038/nn1944

Riedel WJ, 1999, PSYCHOPHARMACOLOGY, V141, P362, DOI 10.1007/s002130050845

Roberfroid M, 2010, BRIT J NUTR, V104, pS1, DOI 10.1017/S0007114510003363

Rolls A, 2007, NAT CELL BIOL, V9, P1081, DOI 10.1038/ncb1629

Romano KA, 2015, MBIO, V6, DOI 10.1128/mBio.02481-14

Ross JA, 2021, FRONT PSYCHIATRY, V11, DOI 10.3389/fpsyt.2020.601519

Rothhammer V, 2018, NATURE, V557, P724, DOI 10.1038/s41586-018-0119-x

Rothhammer V, 2016, NAT MED, V22, P586, DOI 10.1038/nm.4106

Round JL, 2009, NAT REV IMMUNOL, V9, P313, DOI 10.1038/nri2515

Rowland I, 2018, EUR J NUTR, V57, P1, DOI 10.1007/s00394-017-1445-8

Royet J, 2007, NAT REV MICROBIOL, V5, P264, DOI 10.1038/nrmicro1620

Ryu KY, 2019, J NEUROINFLAMM, V16, DOI 10.1186/s12974-019-1561-x

Salazar N, 2017, GUT MICROBES, V8, P82, DOI 10.1080/19490976.2016.1256525

Sampson TR, 2016, CELL, V167, P1469, DOI 10.1016/j.cell.2016.11.018

Sanz Y, 2014, ADV EXP MED BIOL, V817, P291, DOI 10.1007/978-1-4939-0897-4_14

Sato Y, 2021, NATURE, V599, P458, DOI 10.1038/s41586-021-03832-5

Savignac HM, 2013, NEUROCHEM INT, V63, P756, DOI 10.1016/j.neuint.2013.10.006

Scheperjans F, 2015, MOVEMENT DISORD, V30, P350, DOI 10.1002/mds.26069

Schepici G, 2019, CELL TRANSPLANT, V28, P1507, DOI 10.1177/0963689719873890

Schönfeld P, 2016, J LIPID RES, V57, P943, DOI 10.1194/jlr.R067629

Schousboe A, 2007, PROG BRAIN RES, V160, P9, DOI 10.1016/S0079-6123(06)60002-2

Schreiner TG, 2022, BIOMOLECULES, V12, DOI 10.3390/biom12040538

Schretter CE, 2018, NATURE, V563, P402, DOI 10.1038/s41586-018-0634-9

Schwarcz R, 2012, NAT REV NEUROSCI, V13, P465, DOI 10.1038/nrn3257

Segain JP, 2000, GUT, V47, P397, DOI 10.1136/gut.47.3.397

Sharon G, 2016, CELL, V167, P915, DOI 10.1016/j.cell.2016.10.027

Shi Y, 2018, FRONT MICROBIOL, V9, DOI 10.3389/fmicb.2018.01948

Silva YP, 2020, FRONT ENDOCRINOL, V11, DOI 10.3389/fendo.2020.00025

Skonieczna-Zydecka K, 2020, J CLIN MED, V9, DOI 10.3390/jcm9040999

Smith PA, 2015, NATURE, V526, P312, DOI 10.1038/526312a

Soret R, 2010, GASTROENTEROLOGY, V138, P1772, DOI 10.1053/j.gastro.2010.01.053

Soutif-Veillon A, 2016, MATURITAS, V93, P131, DOI 10.1016/j.maturitas.2016.02.004

Sperringer JE, 2017, NEUROCHEM RES, V42, P1697, DOI 10.1007/s11064-017-2261-5

Staley C, 2017, APPL MICROBIOL BIOT, V101, P47, DOI 10.1007/s00253-016-8006-6

Strandwitz P, 2019, NAT MICROBIOL, V4, P396, DOI 10.1038/s41564-018-0307-3

Sudo N, 2004, J PHYSIOL-LONDON, V558, P263, DOI 10.1113/jphysiol.2004.063388

Suganya K, 2020, INT J MOL SCI, V21, DOI 10.3390/ijms21207551

Sun J, 2019, EUR J NEUROL, V26, P1355, DOI 10.1111/ene.13986

Sun J, 2019, TRANSL PSYCHIAT, V9, DOI 10.1038/s41398-019-0525-3

Sun J, 2019, J AGR FOOD CHEM, V67, P3006, DOI 10.1021/acs.jafc.8b07313

Sun J, 2016, NEUROSCI LETT, V613, P30, DOI 10.1016/j.neulet.2015.12.047

Sun MF, 2018, BRAIN BEHAV IMMUN, V70, P48, DOI 10.1016/j.bbi.2018.02.005

Sun MF, 2018, AGEING RES REV, V45, P53, DOI 10.1016/j.arr.2018.04.004

Talbott EO, 2016, HAND CLINIC, V138, P225, DOI 10.1016/B978-0-12-802973-2.00013-6

Tan J, 2014, ADV IMMUNOL, V121, P91, DOI 10.1016/B978-0-12-800100-4.00003-9

Tan XQ, 2021, BEHAV BRAIN RES, V398, DOI 10.1016/j.bbr.2020.112968

Tazoe H, 2009, BIOMED RES-TOKYO, V30, P149, DOI 10.2220/biomedres.30.149

Ticho Alexander L, 2019, Liver Res, V3, P31, DOI 10.1016/j.livres.2019.01.001

Ticinesi A, 2018, CLIN INTERV AGING, V13, P1497, DOI 10.2147/CIA.S139163

Tolhurst G, 2012, DIABETES, V61, P364, DOI 10.2337/db11-1019

Tomova A, 2015, PHYSIOL BEHAV, V138, P179, DOI 10.1016/j.physbeh.2014.10.033

Tosoni G, 2019, CURR OPIN PHARMACOL, V48, P107, DOI 10.1016/j.coph.2019.08.003

Truax AD, 2018, CELL HOST MICROBE, V24, P364, DOI 10.1016/j.chom.2018.08.009

Tsavkelova E A, 2000, Dokl Biochem, V372, P115

Tseng HC, 1998, BIOCHEM BIOPH RES CO, V250, P726, DOI 10.1006/bbrc.1998.9382

Tysnes OB, 2017, J NEURAL TRANSM, V124, P901, DOI 10.1007/s00702-017-1686-y

Unger MM, 2016, PARKINSONISM RELAT D, V32, P66, DOI 10.1016/j.parkreldis.2016.08.019

Vaghef-Mehrabany E, 2020, CLIN NUTR, V39, P1395, DOI 10.1016/j.clnu.2019.06.004

Valladares R, 2013, FASEB J, V27, P1711, DOI 10.1096/fj.12-223339

Vallim TQD, 2013, CELL METAB, V17, P657, DOI 10.1016/j.cmet.2013.03.013

Vaughan CJ, 2000, AM J PHYSIOL-REG I, V279, pR599, DOI 10.1152/ajpregu.2000.279.2.R599

Vijay N, 2014, CURR PHARM DESIGN, V20, P1487, DOI 10.2174/13816128113199990462

Vogt NM, 2017, SCI REP-UK, V7, DOI 10.1038/s41598-017-13601-y

Waclawiková B, 2018, PHARMACEUTICALS-BASE, V11, DOI 10.3390/ph11030063

Wahlström A, 2016, CELL METAB, V24, P41, DOI 10.1016/j.cmet.2016.05.005

Waldecker M, 2008, J NUTR BIOCHEM, V19, P587, DOI 10.1016/j.jnutbio.2007.08.002

Wan Yu-Jui Yvonne, 2018, Liver Res, V2, P180, DOI 10.1016/j.livres.2018.09.008

Wang XY, 2019, CELL RES, V29, P787, DOI 10.1038/s41422-019-0216-x

Wang Y, 2014, BRAIN BEHAV IMMUN, V38, P1, DOI 10.1016/j.bbi.2013.12.015

Wehrwein EA, 2016, COMPR PHYSIOL, V6, P1239, DOI 10.1002/cphy.c150037

Williams BB, 2014, CELL HOST MICROBE, V16, P495, DOI 10.1016/j.chom.2014.09.001

Williams EK, 2016, CELL, V166, P209, DOI 10.1016/j.cell.2016.05.011

Winston JA, 2020, GUT MICROBES, V11, P158, DOI 10.1080/19490976.2019.1674124

Wong ML, 2016, MOL PSYCHIATR, V21, P797, DOI 10.1038/mp.2016.46

Wu C, 2015, METAB BRAIN DIS, V30, P367, DOI 10.1007/s11011-014-9560-1

Wu SP, 2015, PHYSIOL REP, V3, DOI 10.14814/phy2.12356

Wu YC, 2013, NEUROSCIENCE, V239, P67, DOI 10.1016/j.neuroscience.2012.10.024

Xie J, 2020, BIOMED PHARMACOTHER, V127, DOI 10.1016/j.biopha.2020.110147

Xu MD, 2021, MOL NUTR FOOD RES, V65, DOI 10.1002/mnfr.202000660

Xu RH, 2015, IMMUNITY, V43, P1148, DOI 10.1016/j.immuni.2015.11.015

Yamawaki Y, 2018, BRAIN RES, V1680, P13, DOI 10.1016/j.brainres.2017.12.004

Yamout BI, 2018, SEMIN NEUROL, V38, P212, DOI 10.1055/s-0038-1649502

Yang XD, 2018, BMC ANESTHESIOL, V18, DOI 10.1186/s12871-018-0642-1

Yang XQ, 2020, ACTA PHARM SIN B, V10, P475, DOI 10.1016/j.apsb.2019.07.001

Yano JM, 2015, CELL, V161, P264, DOI 10.1016/j.cell.2015.02.047

Yoo BB, 2017, IMMUNITY, V46, P910, DOI 10.1016/j.immuni.2017.05.011

Yu X, 2016, IMMUNITY, V45, P1093, DOI 10.1016/j.immuni.2016.10.001

Yulug B, 2018, PSYCHIAT CLIN NEUROS, V72, P152, DOI 10.1111/pcn.12637

ZAFRA F, 1992, J NEUROSCI, V12, P4793

Zeng QQ, 2020, SCI REP-UK, V10, DOI 10.1038/s41598-020-69845-8

Zeraati M, 2019, NEUROPHARMACOLOGY, V157, DOI 10.1016/j.neuropharm.2019.107685

Zhang BB, 2022, FRONT IMMUNOL, V13, DOI 10.3389/fimmu.2022.893617

Zhang X, 2020, BIOL PHARM BULL, V43, P348, DOI 10.1248/bpb.b19-00862

Zhang ZH, 2019, FRONT CELL DEV BIOL, V7, DOI 10.3389/fcell.2019.00256

Zhang ZB, 2022, J NUTR, V152, P140, DOI 10.1093/jn/nxab328

Zheng DP, 2020, CELL DISCOV, V6, DOI 10.1038/s41421-020-0167-x

Zhu F, 2020, MOL PSYCHIATR, V25, P2905, DOI 10.1038/s41380-019-0475-4

Zhuang ZQ, 2018, J ALZHEIMERS DIS, V63, P1337, DOI 10.3233/JAD-180176

NR 345

TC 4

Z9 4

U1 3

U2 19

PU PERGAMON-ELSEVIER SCIENCE LTD

PI OXFORD

PA THE BOULEVARD, LANGFORD LANE, KIDLINGTON, OXFORD OX5 1GB, United Kingdom

SN 0024-3205

EI 1879-0631

J9 LIFE SCI

JI Life Sci.

PD OCT 1

PY 2023

VL 330

AR 122022

DI 10.1016/j.lfs.2023.122022

EA AUG 2023

PG 21

WC Medicine, Research & Experimental; Pharmacology & Pharmacy

WE Science Citation Index Expanded (SCI-EXPANDED)

SC Research & Experimental Medicine; Pharmacology & Pharmacy

GA S1MN8

UT WOS:001068883300001

PM 37579835

DA 2024-08-12

ER

PT J

AU Shao, LF

Liu, XJ

Zhu, SX

Liu, C

Gao, YL

Xu, XD

AF Shao, Lifei

Liu, Xiaojuan

Zhu, Shunxing

Liu, Chun

Gao, Yilu

Xu, Xide

TI The Role of Smurf1 in Neuronal Necroptosis after

Lipopolysaccharide-Induced Neuroinflammation

SO CELLULAR AND MOLECULAR NEUROBIOLOGY

LA English

DT Article

DE Smad ubiquitination regulatory factor 1; Lipopolysaccharide;

Neuroinflammation; Necroptosis; Receptor-interacting protein 1;

Receptor-interacting protein 3

ID SPINAL-CORD-INJURY; UP-REGULATION; MULTIPLE-SCLEROSIS; SUBARACHNOID

HEMORRHAGE; CELL-DEATH; INHIBITION; NEUROPROTECTION; DEGRADATION;

ASTROCYTES; MECHANISMS

AB The role of inflammation in neurological disorders such as Alzheimer's disease and Parkinson's disease is gradually recognized and leads to an urgent challenge. Smad ubiquitination regulatory factor 1 (Smurf1), one member of the HECT family, is up-regulated by proinflammatory cytokines and associated with apoptosis in acute spinal cord injury. However, the function of Smurf1 through promoting neuronal necroptosis is still limited in the central nervous system (CNS). Hence, we developed a neuroinflammatory model in adult rats following lipopolysaccharide (LPS) lateral ventral injection to elaborate whether Smurf1 is involved in necroptosis in CNS injury. The up-regulation of Smurf1 detected in the rat brain cortex was similar to the necroptotic marker RIP1 expression in a time-dependent manner after LPS-induced neuroinflammation. Meanwhile, Smurf1 knockdown with siRNA inhibited neuronal necroptosis following LPS-stimulated rat pheochromocytomal PC12 cells. Thus, it was indicated that LPS-induced necroptosis could be promoted by Smurf1. In short, these studies suggest that Smurf1 might promote neuronal necroptosis after LPS-induced neuroinflammation, which might act as a novel and potential molecular target for the treatment of neuroinflammation associated diseases.

C1 [Shao, Lifei; Gao, Yilu; Xu, Xide] Nantong Univ, Dept Neurosurg, Affiliated Hosp, Nantong 226001, Jiangsu, China.

[Liu, Xiaojuan] Nantong Univ, Dept Pathogen Biol, Coll Med, Nantong 226001, Jiangsu, China.

[Zhu, Shunxing; Liu, Chun] Nantong Univ, Ctr Expt Anim, Nantong 226001, Jiangsu, China.

C3 Nantong University; Nantong University; Nantong University

RP Gao, YL (corresponding author), Nantong Univ, Dept Neurosurg, Affiliated Hosp, Nantong 226001, Jiangsu, China.

EM ntdxgyl@163.com

FU National Natural Science Foundation of China [81371335, 81401013,

81401365]; Nantong Science and Technology Project [MS12015056]; Priority

Academic Program Development of Jiangsu Higher Education Institutions

(PAPD)

FX This work was partly supported by the National Natural Science

Foundation of China (Nos. 81371335, 81401013, 81401365), Nantong Science

and Technology Project (MS12015056), and a Project funded by the

Priority Academic Program Development of Jiangsu Higher Education

Institutions (PAPD).

CR Cabal-Hierro L, 2012, CELL SIGNAL, V24, P1297, DOI 10.1016/j.cellsig.2012.02.006

Chen XB, 2016, J ADV MECH DES SYST, V10, DOI 10.1299/jamdsm.2016jamdsm0064

Chiurchiù V, 2016, PHARMACOL RES, V113, P313, DOI 10.1016/j.phrs.2016.09.003

Cui Y, 2011, EMBO J, V30, P2675, DOI 10.1038/emboj.2011.155

De Santi L, 2011, CURR MED CHEM, V18, P1775, DOI 10.2174/092986711795496881

Degterev A, 2008, NAT CHEM BIOL, V4, P313, DOI 10.1038/nchembio.83

Dhib-Jalbut S, 2015, CYTOKINE, V76, P583, DOI 10.1016/j.cyto.2015.06.004

Fakharnia F, 2017, J MOL NEUROSCI, V61, P52, DOI 10.1007/s12031-016-0843-3

Fan H, 2015, NEUROSCIENCE, V311, P362, DOI 10.1016/j.neuroscience.2015.10.049

Fan H, 2016, MOL NEURODEGENER, V11, DOI 10.1186/s13024-016-0081-8

He MQ, 2016, NEUROCHEM RES, V41, P3063, DOI 10.1007/s11064-016-2027-5

Hirsch EC, 2009, LANCET NEUROL, V8, P382, DOI 10.1016/S1474-4422(09)70062-6

Koinuma D, 2003, EMBO J, V22, P6458, DOI 10.1093/emboj/cdg632

Lee MG, 2016, CANCER RES, V76, P1847, DOI 10.1158/0008-5472.CAN-15-1752

Lee YS, 2011, NAT COMMUN, V2, DOI 10.1038/ncomms1469

Li DB, 2013, J MOL HISTOL, V44, P381, DOI 10.1007/s10735-013-9499-2

Li MC, 2016, SCI REP-UK, V6, DOI 10.1038/srep35055

Liu T, 2016, SCI REP-UK, V6, DOI 10.1038/srep24547

Morales I, 2014, FRONT CELL NEUROSCI, V8, DOI 10.3389/fncel.2014.00112

Nagley P, 2010, BBA-MOL BASIS DIS, V1802, P167, DOI 10.1016/j.bbadis.2009.09.004

Nikseresht S, 2015, J MOL NEUROSCI, V57, P219, DOI 10.1007/s12031-015-0609-3

Pannu R, 2006, NEUROCHEM INT, V49, P170, DOI 10.1016/j.neuint.2006.04.010

Parajuli B, 2015, GLIA, V63, P2274, DOI 10.1002/glia.22892

Politi K, 2016, CURR BIOL, V26, pR932, DOI 10.1016/j.cub.2016.08.052

Qian GH, 2016, BIOCHEM BIOPH RES CO, V474, P491, DOI 10.1016/j.bbrc.2016.04.135

Rothman AMK, 2016, J CLIN INVEST, V126, P2495, DOI 10.1172/JCI83361

Sasso V, 2016, J NEUROINFLAMM, V13, DOI 10.1186/s12974-016-0616-5

Vandenabeele P, 2010, NAT REV MOL CELL BIO, V11, P700, DOI 10.1038/nrm2970

Wen SH, 2017, J CELL MOL MED, V21, P432, DOI 10.1111/jcmm.12987

Wong SY, 2016, J NEUROINFLAMM, V13, DOI 10.1186/s12974-016-0498-6

Wu CS, 2016, NEUROCHEM RES, V41, P3103, DOI 10.1007/s11064-016-2033-7

Xie Y, 2017, BRAIN BEHAV IMMUN, V60, P346, DOI 10.1016/j.bbi.2016.11.004

Xu Y, 2016, SCI REP-UK, V6, DOI 10.1038/srep29362

Zhang J, 2016, CELL MOL LIFE SCI, V73, P2153, DOI 10.1007/s00018-016-2190-5

Zhang T, 2016, NAT MED, V22, P175, DOI 10.1038/nm.4017

Zhang Y. D., 2016, SHOCK VIB, V2016, P1, DOI DOI 10.1155/2016/1317232

Zhou YB, 2016, PHARMACOL RES, V111, P885, DOI 10.1016/j.phrs.2016.08.007

Zhu YJ, 2016, PLOS ONE, V11, DOI 10.1371/journal.pone.0156729

NR 38

TC 28

Z9 30

U1 1

U2 29

PU SPRINGER/PLENUM PUBLISHERS

PI NEW YORK

PA 233 SPRING ST, NEW YORK, NY 10013 USA

SN 0272-4340

EI 1573-6830

J9 CELL MOL NEUROBIOL

JI Cell. Mol. Neurobiol.

PD MAY

PY 2018

VL 38

IS 4

BP 809

EP 816

DI 10.1007/s10571-017-0553-6

PG 8

WC Cell Biology; Neurosciences

WE Science Citation Index Expanded (SCI-EXPANDED)

SC Cell Biology; Neurosciences & Neurology

GA GB5MM

UT WOS:000429108800005

PM 28940129

DA 2024-08-12

ER

PT J

AU Castillo-Rangel, C

Marin, G

Hernández-Contreras, KA

Vichi-Ramírez, MM

Zarate-Calderon, C

Torres-Pineda, O

Diaz-Chiguer, DL

González, DD

Apo, EG

Teco-Cortes, JA

Santos-Paez, FD

Coello-Torres, MD

Baldoncini, M

Soto, GR

Aranda-Abreu, GE

García, LI

AF Castillo-Rangel, Carlos

Marin, Gerardo

Hernandez-Contreras, Karla Aketzalli

Vichi-Ramirez, Micheel Merari

Zarate-Calderon, Cristofer

Torres-Pineda, Osvaldo

Diaz-Chiguer, Dylan L.

Gonzalez, David de la Mora

Apo, Erick Gomez

Teco-Cortes, Javier Alejandro

Santos-Paez, Flor de Maria

Coello-Torres, Maria de los Angeles

Baldoncini, Matias

Soto, Gervith Reyes

Aranda-Abreu, Gonzalo Emiliano

Garcia, Luis I.

TI Neuroinflammation in Parkinson's Disease: From Gene to Clinic: A

Systematic Review

SO INTERNATIONAL JOURNAL OF MOLECULAR SCIENCES

LA English

DT Review

DE Parkinson's disease; gene expression; molecular pathways; cellular

alterations; neuroanatomical alterations; neurobehavioral; clinical

manifestations

ID alzheimer's disease; MODEL; DIAGNOSIS

AB Parkinson's disease is a neurodegenerative disease whose progression and clinical characteristics have a close bidirectional and multilevel relationship with the process of neuroinflammation. In this context, it is necessary to understand the mechanisms involved in this neuroinflammation-PD link. This systematic search was, hereby, conducted with a focus on the four levels where alterations associated with neuroinflammation in PD have been described (genetic, cellular, histopathological and clinical-behavioral) by consulting the PubMed, Google Scholar, Scielo and Redalyc search engines, including clinical studies, review articles, book chapters and case studies. Initially, 585,772 articles were included, and, after applying the inclusion and exclusion criteria, 84 articles were obtained that contained information about the multilevel association of neuroinflammation with alterations in gene, molecular, cellular, tissue and neuroanatomical expression as well as clinical-behavioral manifestations in PD.

C1 [Castillo-Rangel, Carlos; Diaz-Chiguer, Dylan L.] Hosp Reg 1 Octubre, Inst Social Secur Serv State Workers ISSSTE, Neurosurg Dept, Mexico City 07300, Mexico.

[Marin, Gerardo] Cleveland Clin, Neural Dynam & Modulat Lab, Cleveland Hts, OH 44195 USA.

[Hernandez-Contreras, Karla Aketzalli; Vichi-Ramirez, Micheel Merari; Zarate-Calderon, Cristofer; Torres-Pineda, Osvaldo; Gonzalez, David de la Mora; Santos-Paez, Flor de Maria; Aranda-Abreu, Gonzalo Emiliano; Garcia, Luis I.] Univ Veracruzana, Brain Res Inst, Xalapa 91192, Mexico.

[Apo, Erick Gomez; Teco-Cortes, Javier Alejandro] Hosp Gen Mexico City, Pathol Dept, Dr Eduardo Liceaga, Mexico City 06720, Mexico.

[Coello-Torres, Maria de los Angeles] Inst Nacl Ciencias Med & Nutr Salvador Zubiran, Res Dept, Mexico City 14080, Mexico.

[Baldoncini, Matias] Univ Buenos Aires, Chair Gross Anat 2, Lab Microsurg Neuroanat, RA-C1052neurodegenerative disease Buenos Aires, Argentina.

[Soto, Gervith Reyes] Natl Canc Inst INCAN, Mexico City 14080, Mexico.

C3 Cleveland Clinic Foundation; Universidad Veracruzana; Hospital General

de Mexico; Instituto Nacional de Ciencias Medicas y Nutricion Salvador

Zubiran - Mexico; University of Buenos Aires; Instituto Nacional de

Cancerologia (INCAN)

RP Marin, G (corresponding author), Cleveland Clin, Neural Dynam & Modulat Lab, Cleveland Hts, OH 44195 USA.

EM MARINMG@ccf.org

OI Baldoncini, Matias/0000-0001-9323-8306; Marin Marquez,

Gerardo/0000-0003-4782-5504; Zarate Calderon, Cristofer de

Jesus/0000-0002-6039-3745; Torres-Pineda, Osvaldo/0000-0002-8937-4433;

Vichi-Ramirez, Micheel Merari/0000-0003-0434-4030; Coello Torres,

Angeles/0000-0001-5204-6332; Aranda-Abreu, Gonzalo/0000-0001-8519-5473;

De la Mora Gonzalez, David/0009-0006-6764-1162; Garcia, Luis

I/0000-0001-6967-1919

CR Alarcon-Gil J, 2022, CELLS-BASEL, V11, DOI 10.3390/cells11152297

Albarracín AL, 2022, DATA BRIEF, V40, DOI 10.1016/j.dib.2021.107712

Andrejack J, 2020, J PARKINSON DIS, V10, pS5, DOI 10.3233/JPD-202107

Arena G, 2022, CURR NEUROL NEUROSCI, V22, P427, DOI 10.1007/s11910-022-01207-5

Aryal B, 2019, BMB REP, V52, P250, DOI 10.5483/BMBRep.2019.52.4.204

Atri A, 2019, MED CLIN N AM, V103, P263, DOI 10.1016/j.mcna.2018.10.009

Badanjak K, 2021, INT J MOL SCI, V22, DOI 10.3390/ijms22094676

Bandres-Ciga S, 2020, NEUROBIOL DIS, V137, DOI 10.1016/j.nbd.2020.104782

Barazzuol L, 2020, INT J MOL SCI, V21, DOI 10.3390/ijms21051772

Barnhill LM, 2020, BIOMEDICINES, V8, DOI 10.3390/biomedicines8070197

Benvenuti L, 2020, INT J MOL SCI, V21, DOI 10.3390/ijms21239199

Bidesi NSR, 2021, J NEUROCHEM, V159, P660, DOI 10.1111/jnc.15516

Blauwendraat C, 2020, LANCET NEUROL, V19, P170, DOI 10.1016/S1474-4422(19)30287-X

Bloem BR, 2021, LANCET, V397, P2284, DOI 10.1016/S0140-6736(21)00218-X

Borsche M, 2021, J PARKINSON DIS, V11, P45, DOI 10.3233/JPD-201981

Brundin P, 2017, J NEUROSCI, V37, P9808, DOI 10.1523/JNEUROSCI.1788-16.2017

Caputi V, 2018, INT J MOL SCI, V19, DOI 10.3390/ijms19061689

Carballo-Carbajal I, 2019, NAT COMMUN, V10, DOI 10.1038/s41467-019-08858-y

Celaya L.V., 2019, REV ENEUROBIOL, V10, P1503

Cerri S, 2019, J PARKINSON DIS, V9, P501, DOI 10.3233/JPD-191683

Chen RB, 2022, CLIN NEUROPHYS PRACT, V7, P201, DOI 10.1016/j.cnp.2022.06.002

Chen Y, 2020, EXPERT OPIN THER TAR, V24, P287, DOI 10.1080/14728222.2020.1733970

Chu HY, 2020, ACTA PHARMACOL SIN, V41, P447, DOI 10.1038/s41401-020-0371-0

Çinar E, 2022, BALK MED J, V39, P318, DOI 10.4274/balkanmedj.galenos.2022.2022-7-100

Coskuner-Weber O, 2018, INT J MOL SCI, V19, DOI 10.3390/ijms19020336

Dallé E, 2018, MOL BRAIN, V11, DOI 10.1186/s13041-018-0356-9

Darweesh SKL, 2018, J PARKINSON DIS, V8, P495, DOI 10.3233/JPD-181374

Ferreira LPD, 2022, CLINICS, V77, DOI 10.1016/j.clinsp.2022.100121

Di Martino R, 2021, INT J MOL SCI, V22, DOI 10.3390/ijms22158177

Dickson DW, 2018, PARKINSONISM RELAT D, V46, pS30, DOI 10.1016/j.parkreldis.2017.07.033

Do J, 2019, MOL NEURODEGENER, V14, DOI 10.1186/s13024-019-0336-2

Du XY, 2020, INT J MOL SCI, V21, DOI 10.3390/ijms21228645

Ellis TD, 2021, SEMIN NEUROL, V41, P189, DOI 10.1055/s-0041-1725133

Oyarzún JE, 2019, BBA-MOL BASIS DIS, V1865, P1076, DOI 10.1016/j.bbadis.2019.03.009

Fais M, 2021, INT J MOL SCI, V22, DOI 10.3390/ijms22147630

Fecchio C, 2018, MOLECULES, V23, DOI 10.3390/molecules23071531

Fernandez-Irigoyen J, 2021, BIOMEDICINES, V9, DOI 10.3390/biomedicines9050491

Flores-Cuadrado A, 2021, NPJ PARKINSONS DIS, V7, DOI 10.1038/s41531-020-00154-7

Fontoura JL, 2017, PARKINSONS DIS-US, V2017, DOI 10.1155/2017/9124160

García-Sanz P, 2018, AUTOPHAGY, V14, P717, DOI 10.1080/15548627.2018.1427396

Gelders G, 2018, J IMMUNOL RES, V2018, DOI 10.1155/2018/4784268

Gómez-Benito M, 2020, FRONT PHARMACOL, V11, DOI 10.3389/fphar.2020.00356

Guatteo E, 2022, INT J MOL SCI, V23, DOI 10.3390/ijms23094508

Gundersen V, 2021, FRONT NEUROSCI-SWITZ, V14, DOI 10.3389/fnins.2020.580311

Hallett PJ, 2019, J NEUROINFLAMM, V16, DOI 10.1186/s12974-019-1532-2

Hirsch EC, 2021, MOVEMENT DISORD, V36, P16, DOI 10.1002/mds.28075

Horne KL, 2021, MOV DISORD CLIN PRAC, V8, P390, DOI 10.1002/mdc3.13151

Ingram TL, 2020, AGING-US, V12, P17713, DOI 10.18632/aging.103937

Jin XH, 2020, INT J ENV RES PUB HE, V17, DOI 10.3390/ijerph17010031

Jozwiak N, 2017, SLEEP, V40, DOI 10.1093/sleep/zsx101

Junqueira SC, 2019, BBA-MOL BASIS DIS, V1865, P2001, DOI 10.1016/j.bbadis.2018.10.025

Karimi-Moghadam A, 2018, CELL MOL NEUROBIOL, V38, P1153, DOI 10.1007/s10571-018-0587-4

Kempuraj D, 2018, J NEUROIMMUNE PHARM, V13, P100, DOI 10.1007/s11481-017-9766-1

Khan MA, 2017, B MENNINGER CLIN, V81, P53, DOI 10.1521/bumc.2017.81.1.53

Kip E, 2022, AGEING RES REV, V78, DOI 10.1016/j.arr.2022.101618

Kouli A, 2020, ACTA NEUROPATHOL COM, V8, DOI 10.1186/s40478-020-01083-5

Lamonaca G, 2020, CELLS-BASEL, V9, DOI 10.3390/cells9051115

Larsen SB, 2018, CELL TISSUE RES, V373, P21, DOI 10.1007/s00441-017-2768-8

Lesage S, 2020, FRONT NEUROL, V11, DOI 10.3389/fneur.2020.00682

Li HY, 2019, AUTOPHAGY, V15, P113, DOI 10.1080/15548627.2018.1509818

Lin KJ, 2019, INT J MOL SCI, V20, DOI 10.3390/ijms20215312

Liu WL, 2021, FRONT PSYCHOL, V12, DOI 10.3389/fpsyg.2021.612030

Lo Buono V, 2021, ACTA NEUROL BELG, V121, P837, DOI 10.1007/s13760-021-01684-4

Lozovaya N, 2018, CELL STRESS, V2, P147, DOI 10.15698/cst2018.06.142

Ma L, 2018, BBA-MOL BASIS DIS, V1864, P3714, DOI 10.1016/j.bbadis.2018.10.003

Mamula D, 2022, NPJ PARKINSONS DIS, V8, DOI 10.1038/s41531-022-00438-0

Marin G, 2022, Neurol Perspect, V2, P151

Marin G., 2022, REPOS I U VERACRUZAN, P1, DOI [10.13140/RG.2.2.22075.49449, DOI 10.13140/RG.2.2.22075.49449]

Marín G, 2022, REV MEX NEUROCIENC, V23, P195, DOI [10.24875/RMN.22000015, 10.24875/rmn.22000015]

Marogianni C, 2020, INT J MOL SCI, V21, DOI 10.3390/ijms21228421

Mazumder S, 2022, FRONT NEUROSCI-SWITZ, V16, DOI 10.3389/fnins.2022.948523

Michelet E., 2022, F1000Res, V2022, P1176, DOI [10.12688/f1000research.124160.1, DOI 10.12688/F1000RESEARCH.124160.1]

Miller S, 2019, NEUROSCI LETT, V705, P7, DOI 10.1016/j.neulet.2019.04.029

Moonen AJH, 2021, MOVEMENT DISORD, V36, P2539, DOI 10.1002/mds.28533

Myasnikov A, 2021, CELL, V184, P3519, DOI 10.1016/j.cell.2021.05.004

Nagatsu T, 2022, INT J MOL SCI, V23, DOI 10.3390/ijms23084176

Natale G, 2021, LIFE-BASEL, V11, DOI 10.3390/life11080732

Page MJ, 2021, BMJ-BRIT MED J, V372, DOI [10.1016/j.ijsu.2021.105906, 10.1136/bmj.n71, 10.1136/bmj.n160]

Pajares M, 2020, CELLS-BASEL, V9, DOI 10.3390/cells9071687

Pajarillo E, 2019, BBA-MOL BASIS DIS, V1865, P1992, DOI 10.1016/j.bbadis.2018.11.017

Pan MK, 2018, TREMOR OTHER HYPERK, V8, DOI 10.7916/D89S37MV

Park JS, 2018, CURR NEUROL NEUROSCI, V18, DOI 10.1007/s11910-018-0829-3

Parkinson J, 2002, J NEUROPSYCH CLIN N, V14, P223, DOI 10.1176/appi.neuropsych.14.2.223

Picca A, 2021, BIOMOLECULES, V11, DOI 10.3390/biom11101508

Pisa D, 2020, INT J BIOL SCI, V16, P1135, DOI 10.7150/ijbs.42257

Pons-Espinal M, 2021, CELL MOL LIFE SCI, V78, P2081, DOI 10.1007/s00018-020-03700-x

Ponsi G, 2021, NPJ PARKINSONS DIS, V7, DOI 10.1038/s41531-021-00167-w

Portz P, 2021, CELLS-BASEL, V10, DOI 10.3390/cells10040885

Prange S, 2022, DRUG AGING, V39, P417, DOI 10.1007/s40266-022-00942-1

Prasuhn J, 2021, MOL MED, V27, DOI 10.1186/s10020-021-00281-8

Prediger RD, 2019, HAND CLINIC, V164, P431, DOI 10.1016/B978-0-444-63855-7.00024-1

Reichmann H, 2017, NEUROSCI BULL, V33, P526, DOI 10.1007/s12264-017-0159-5

Rektorova I, 2019, PARKINSONISM RELAT D, V59, P65, DOI 10.1016/j.parkreldis.2019.02.042

Rey F, 2021, ANTIOXIDANTS-BASEL, V10, DOI 10.3390/antiox10010121

Riboldi GM, 2019, CELLS-BASEL, V8, DOI 10.3390/cells8040364

Rostami J, 2020, J NEUROINFLAMM, V17, DOI 10.1186/s12974-020-01776-7

Schapira AHV, 2017, NAT REV NEUROSCI, V18, P435, DOI 10.1038/nrn.2017.62

Selvaraj S, 2019, GENES DIS, V6, P120, DOI 10.1016/j.gendis.2019.01.004

Senkevich KA, 2021, ACTA NATURAE, V13, P70, DOI 10.32607/actanaturae.11031

Shalash A, 2020, MOVEMENT DISORD, V35, P1097, DOI 10.1002/mds.28134

Simon DK, 2020, CLIN GERIATR MED, V36, P1, DOI 10.1016/j.cger.2019.08.002

Sommer A, 2018, CELL STEM CELL, V23, P123, DOI 10.1016/j.stem.2018.06.015

Stoker T. B., 2018, PARKINSONS DIS PATHO

Tan SH, 2019, BIOMED PHARMACOTHER, V111, P765, DOI 10.1016/j.biopha.2018.12.101

Tansey MG, 2022, NAT REV IMMUNOL, V22, P657, DOI 10.1038/s41577-022-00684-6

Tolosa E, 2021, LANCET NEUROL, V20, P385, DOI 10.1016/S1474-4422(21)00030-2

Trist BG, 2019, AGING CELL, V18, DOI 10.1111/acel.13031

van den Berge N, 2022, NEUROBIOL DIS, V163, DOI 10.1016/j.nbd.2021.105599

Vasquez-Celaya L., 2022, NEUROLOGIA, DOI [10.1016/j.nrl.2021.12.003, DOI 10.1016/J.NRL.2021.12.003]

Vázquez-Vélez GE, 2021, ANNU REV NEUROSCI, V44, P87, DOI 10.1146/annurev-neuro-100720-034518

Vila M, 2019, MOVEMENT DISORD, V34, P1440, DOI 10.1002/mds.27776

Wang T, 2020, INT J ENV RES PUB HE, V17, DOI 10.3390/ijerph17134878

Wang YM, 2021, FRONT PHARMACOL, V12, DOI 10.3389/fphar.2021.757161

Weng MR, 2018, PARKINSONS DIS-US, V2018, DOI 10.1155/2018/9163040

Williams GP, 2021, BRAIN, V144, P2047, DOI 10.1093/brain/awab103

Wu PL, 2017, PLOS ONE, V12, DOI 10.1371/journal.pone.0181515

Xia ML, 2020, J NEUROINFLAMM, V17, DOI 10.1186/s12974-020-01791-8

Xia NN, 2022, FRONT NEUROSCI-SWITZ, V16, DOI 10.3389/fnins.2022.894620

Yan AJ, 2019, FRONT CELL NEUROSCI, V12, DOI 10.3389/fncel.2018.00529

Zeng XS, 2018, FRONT AGING NEUROSCI, V10, DOI 10.3389/fnagi.2018.00109

Zhang CT, 2022, BIOMED PHARMACOTHER, V155, DOI 10.1016/j.biopha.2022.113768

NR 121

TC 13

Z9 13

U1 1

U2 8

PU MDPI

PI BASEL

PA ST ALBAN-ANLAGE 66, CH-4052 BASEL, SWITZERLAND

SN 1661-6596

EI 1422-0067

J9 INT J MOL SCI

JI Int. J. Mol. Sci.

PD MAR

PY 2023

VL 24

IS 6

AR 5792

DI 10.3390/ijms24065792

PG 29

WC Biochemistry & Molecular Biology; Chemistry, Multidisciplinary

WE Science Citation Index Expanded (SCI-EXPANDED)

SC Biochemistry & Molecular Biology; Chemistry

GA A9KQ7

UT WOS:000958239200001

PM 36982866

OA Green Published, gold

DA 2024-08-12

ER

PT J

AU Wang, YM

Xia, Y

Kou, L

Yin, SJ

Chi, XS

Li, JW

Sun, YD

Wu, JW

Zhou, QL

Zou, WK

Jin, ZJ

Huang, JS

Xiong, N

Wang, T

AF Wang, Yiming

Xia, Yun

Kou, Liang

Yin, Sijia

Chi, Xiaosa

Li, Jingwen

Sun, Yadi

Wu, Jiawei

Zhou, Qiulu

Zou, Wenkai

Jin, Zongjie

Huang, Jinsha

Xiong, Nian

Wang, Tao

TI Astrocyte-to-neuron reprogramming and crosstalk in the treatment of

Parkinson's disease

SO NEUROBIOLOGY OF DISEASE

LA English

DT Review

DE Parkinson's disease (PD); Astrocyte; Reprogramming; Damaged mitochondria

ID PLURIPOTENT STEM-CELLS; KETOGLUTARATE DEHYDROGENASE COMPLEX;

ALPHA-SYNUCLEIN; DOPAMINERGIC-NEURONS; FUNCTIONAL-NEURONS; DIRECT

CONVERSION; RAT MODEL; IN-VITRO; GENERATION; MOUSE

AB Parkinson's disease (PD) is currently the fastest growing disabling neurological disorder worldwide, with motor and non-motor symptoms being its main clinical manifestations. The primary pathological features include a reduction in the number of dopaminergic neurons in the substantia nigra and decrease in dopamine levels in the nigrostriatal pathway. Existing treatments only alleviate clinical symptoms and do not stop disease progression; slowing down the loss of dopaminergic neurons and stimulating their regeneration are emerging therapies. Preclinical studies have demonstrated that transplantation of dopamine cells generated from human embryonic or induced pluripotent stem cells can restore the loss of dopamine. However, the application of cell transplantation is limited owing to ethical controversies and the restricted source of cells. Until recently, the reprogramming of astrocytes to replenish lost dopaminergic neurons has provided a promising alternative therapy for PD. In addition, repair of mitochondrial perturbations, clearance of damaged mitochondria in astrocytes, and control of astrocyte inflammation may be extensively neuroprotective and beneficial against chronic neuroinflammation in PD. Therefore, this review primarily focuses on the progress and remaining issues in astrocyte reprogramming using transcription factors (TFs) and miRNAs, as well as exploring possible new targets for treating PD by repairing astrocytic mitochondria and reducing astrocytic inflammation.

C1 [Wang, Yiming; Xia, Yun; Kou, Liang; Yin, Sijia; Chi, Xiaosa; Li, Jingwen; Sun, Yadi; Wu, Jiawei; Zhou, Qiulu; Zou, Wenkai; Jin, Zongjie; Huang, Jinsha; Xiong, Nian; Wang, Tao] Huazhong Univ Sci & Technol, Union Hosp, Tongji Med Coll, Dept Neurol, Wuhan 430022, China.

C3 Huazhong University of Science & Technology

RP Wang, T (corresponding author), Huazhong Univ Sci & Technol, Union Hosp, Tongji Med Coll, Dept Neurol, Wuhan 430022, China.

EM wangtaowh@hust.edu.cn

RI wu, jiawei/ABG-4857-2020

OI wu, jiawei/0000-0001-7382-9993

FU National Natural Science Foundation of China [81974201, 82171424]

FX This work was supported by grants 82101338 from the National Natural

Science Foundation of China (to YX) , grants 81974201, and 82171424 from

the National Natural Science Foundation of China (to TW) .

CR Ablasser A, 2019, SCIENCE, V363, P1055, DOI 10.1126/science.aat8657

Adler AF, 2019, CELL REP, V28, P3462, DOI 10.1016/j.celrep.2019.08.058

Aflaki E, 2020, NEUROBIOL DIS, V134, DOI 10.1016/j.nbd.2019.104647

Alterman RL, 2021, NEUROSURGERY, V88, pE216, DOI 10.1093/neuros/nyaa487

Alvarez-Erviti L, 2011, NEUROBIOL DIS, V42, P360, DOI 10.1016/j.nbd.2011.01.029

Amamoto R, 2014, SCIENCE, V343, P504, DOI 10.1126/science.1239882

Aoki T, 2010, TISSUE ENG PT A, V16, P2197, DOI [10.1089/ten.tea.2009.0747, 10.1089/ten.TEA.2009.0747]

Arenas E, 2020, NATURE, V582, P489, DOI 10.1038/d41586-020-01817-4

Ariga H, 2013, OXID MED CELL LONGEV, V2013, DOI 10.1155/2013/683920

Armstrong MJ, 2020, JAMA-J AM MED ASSOC, V323, P548, DOI 10.1001/jama.2019.22360

Bahat-Stroomza M, 2009, J MOL NEUROSCI, V39, P199, DOI 10.1007/s12031-008-9166-3

Balestrino R, 2020, EUR J NEUROL, V27, P27, DOI 10.1111/ene.14108

Bandopadhyay R, 2004, BRAIN, V127, P420, DOI 10.1093/brain/awh054

Barker RA, 2019, NAT MED, V25, P1045, DOI 10.1038/s41591-019-0507-2

Barodia SK, 2019, NPJ PARKINSON DIS, V5, DOI 10.1038/s41531-019-0101-9

Björkblom B, 2013, J BIOL CHEM, V288, P22809, DOI 10.1074/jbc.M113.482091

Bloem BR, 2021, LANCET, V397, P2284, DOI 10.1016/S0140-6736(21)00218-X

Bonifati V, 2003, SCIENCE, V299, P256, DOI 10.1126/science.1077209

Booth HDE, 2017, TRENDS NEUROSCI, V40, P358, DOI 10.1016/j.tins.2017.04.001

Boutz PL, 2007, GENE DEV, V21, P1636, DOI 10.1101/gad.1558107

Braak H, 2007, ACTA NEUROPATHOL, V114, P231, DOI 10.1007/s00401-007-0244-3

Braidy N, 2013, TRANSL NEURODEGENER, V2, DOI 10.1186/2047-9158-2-20

Brot S, 2022, CELLS-BASEL, V11, DOI 10.3390/cells11101596

Brown ME, 2010, PLOS ONE, V5, DOI 10.1371/journal.pone.0011373

Brulet R, 2017, STEM CELL REP, V8, P1506, DOI 10.1016/j.stemcr.2017.04.013

Buffo A, 2005, P NATL ACAD SCI USA, V102, P18183, DOI 10.1073/pnas.0506535102

Buffo A, 2008, P NATL ACAD SCI USA, V105, P3581, DOI 10.1073/pnas.0709002105

Caiazzo M, 2011, NATURE, V476, P224, DOI 10.1038/nature10284

Cardoso T, 2018, J COMP NEUROL, V526, P2133, DOI 10.1002/cne.24500

Cavaliere F, 2017, NEUROBIOL DIS, V103, P101, DOI 10.1016/j.nbd.2017.04.011

Cervo PRD, 2017, NAT BIOTECHNOL, V35, P444, DOI 10.1038/nbt.3835

Charron G, 2014, FRONT AGING NEUROSCI, V6, DOI 10.3389/fnagi.2014.00258

Chavarría C, 2018, BIOCHEM J, V475, P3153, DOI 10.1042/BCJ20180297

Chen WZ, 2022, ELIFE, V11, DOI 10.7554/eLife.75636

Chen YC, 2020, MOL THER, V28, P217, DOI 10.1016/j.ymthe.2019.09.003

Cheng LC, 2009, NAT NEUROSCI, V12, P399, DOI 10.1038/nn.2294

Cheng XY, 2020, TRANSL NEURODEGENER, V9, DOI 10.1186/s40035-020-00190-6

Choi I, 2013, GLIA, V61, P800, DOI 10.1002/glia.22475

Connolly BS, 2014, JAMA-J AM MED ASSOC, V311, P1670, DOI 10.1001/jama.2014.3654

Cooper JM, 2014, MOVEMENT DISORD, V29, P1476, DOI 10.1002/mds.25978

Corti S, 2012, EXP CELL RES, V318, P1528, DOI 10.1016/j.yexcr.2012.02.040

Cowan CA, 2005, SCIENCE, V309, P1369, DOI 10.1126/science.1116447

Dar GH, 2022, FRONT AGING NEUROSCI, V14, DOI 10.3389/fnagi.2022.967231

Delenclos M, 2017, FRONT NEUROSCI-SWITZ, V11, DOI 10.3389/fnins.2017.00172

Deleo AM, 2018, J NEUROIMMUNE PHARM, V13, P292, DOI 10.1007/s11481-017-9768-z

DeLong MR, 2015, JAMA NEUROL, V72, P1354, DOI 10.1001/jamaneurol.2015.2397

di Domenico A, 2019, STEM CELL REP, V12, P213, DOI 10.1016/j.stemcr.2018.12.011

Doi D, 2020, NAT COMMUN, V11, DOI 10.1038/s41467-020-17165-w

Ebrahimi-Fakhari D, 2012, ACTA NEUROPATHOL, V124, P153, DOI 10.1007/s00401-012-1004-6

Erustes AG, 2018, J NEUROSCI RES, V96, P160, DOI 10.1002/jnr.24092

Escartin C, 2021, NAT NEUROSCI, V24, P312, DOI 10.1038/s41593-020-00783-4

Escartin C, 2019, GLIA, V67, P2221, DOI 10.1002/glia.23687

Feigin VL, 2019, LANCET NEUROL, V18, P459, DOI [10.1016/S1474-4422(18)30499-X, 10.1016/S1474-4422(19)30034-1, 10.1016/S1474-4422(18)30415-0]

Fellner L, 2013, GLIA, V61, P349, DOI 10.1002/glia.22437

Fellner L, 2011, ACTA NEUROPATHOL, V121, P675, DOI 10.1007/s00401-011-0833-z

Galves M, 2019, TRENDS BIOCHEM SCI, V44, P872, DOI 10.1016/j.tibs.2019.04.007

Gan L, 2012, J NEUROSCI, V32, P17775, DOI 10.1523/JNEUROSCI.3049-12.2012

Gascón S, 2016, CELL STEM CELL, V18, P396, DOI 10.1016/j.stem.2015.12.003

Ge LJ, 2020, FRONT CELL DEV BIOL, V8, DOI 10.3389/fcell.2020.590008

Giehrl-Schwab J, 2022, EMBO MOL MED, V14, DOI 10.15252/emmm.202114797

Giorgetti A, 2009, CELL STEM CELL, V5, P353, DOI 10.1016/j.stem.2009.09.008

Gkikas I, 2018, FRONT IMMUNOL, V9, DOI 10.3389/fimmu.2018.01283

Glass CK, 2010, CELL, V140, P918, DOI 10.1016/j.cell.2010.02.016

Gordon GRJ, 2007, GLIA, V55, P1214, DOI 10.1002/glia.20543

Gosselin RD, 2013, FRONT CELL NEUROSCI, V7, DOI 10.3389/fncel.2013.00251

Grande A, 2013, NAT COMMUN, V4, DOI 10.1038/ncomms3373

Guo ZY, 2014, CELL STEM CELL, V14, P188, DOI 10.1016/j.stem.2013.12.001

Gutierrez-Aranda I, 2010, STEM CELLS, V28, P1568, DOI 10.1002/stem.471

Halassa MM, 2007, TRENDS MOL MED, V13, P54, DOI 10.1016/j.molmed.2006.12.005

Haney MJ, 2015, J CONTROL RELEASE, V207, P18, DOI 10.1016/j.jconrel.2015.03.033

Hao YJ, 2023, NATURE, V618, DOI 10.1038/s41586-023-06067-8

Hastings N, 2022, CELL TRANSPLANT, V31, DOI 10.1177/09636897221105499

Hebron ML, 2013, HUM MOL GENET, V22, P3315, DOI 10.1093/hmg/ddt192

Heiland DH, 2019, NAT COMMUN, V10, DOI 10.1038/s41467-019-10493-6

Heinrich C, 2014, STEM CELL REP, V3, P1000, DOI 10.1016/j.stemcr.2014.10.007

Heo JY, 2020, CURR BIOL, V30, P276, DOI 10.1016/j.cub.2019.11.079

Hoang T, 2023, NATURE, V618, DOI 10.1038/s41586-023-06066-9

Hoang T, 2022, CELL REP, V39, DOI 10.1016/j.celrep.2022.110849

Honbou K, 2003, J BIOL CHEM, V278, P31380, DOI 10.1074/jbc.M305878200

Hong MH, 2020, CANCER CELL, V38, P473, DOI 10.1016/j.ccell.2020.07.005

Hsieh CH, 2019, CELL METAB, V30, P1131, DOI 10.1016/j.cmet.2019.08.023

Irie T, 2023, GENES CELLS, V28, P526, DOI 10.1111/gtc.13033

Jankovic J, 2020, MOVEMENT DISORD, V35, P1312, DOI 10.1002/mds.28187

Jiang PZ, 2010, MOL NEURODEGENER, V5, DOI 10.1186/1750-1326-5-56

Jo S, 2014, NAT MED, V20, P886, DOI 10.1038/nm.3639

Joshi AU, 2019, NAT NEUROSCI, V22, P1635, DOI 10.1038/s41593-019-0486-0

June CH, 2018, NEW ENGL J MED, V379, P64, DOI 10.1056/NEJMra1706169

Kalia LV, 2015, LANCET, V386, P896, DOI 10.1016/S0140-6736(14)61393-3

Kim C, 2013, NAT COMMUN, V4, DOI 10.1038/ncomms2534

Kim JM, 2016, SCI REP-UK, V6, DOI 10.1038/srep28823

Kim RH, 2005, P NATL ACAD SCI USA, V102, P5215, DOI 10.1073/pnas.0501282102

Kordower JH, 2017, ANN NEUROL, V81, P46, DOI 10.1002/ana.24820

Krichevsky AM, 2006, STEM CELLS, V24, P857, DOI 10.1634/stemcells.2005-0441

Kumamaru H, 2018, NAT METHODS, V15, P723, DOI 10.1038/s41592-018-0074-3

Latchman DS, 1997, INT J BIOCHEM CELL B, V29, P1305, DOI 10.1016/S1357-2725(97)00085-X

Lee HJ, 2010, J BIOL CHEM, V285, P9262, DOI 10.1074/jbc.M109.081125

Lee MK, 2002, P NATL ACAD SCI USA, V99, P8968, DOI 10.1073/pnas.132197599

Lee S, 2022, ACTA BIOMATER, V151, P561, DOI 10.1016/j.actbio.2022.07.058

Leggio L, 2022, ADV HEALTHC MATER, V11, DOI 10.1002/adhm.202201203

Li Q, 2014, ELIFE, V3, DOI 10.7554/eLife.01201

Li Y, 2010, CELL REPROGRAM, V12, P237, DOI 10.1089/cell.2009.0103

Liddelow SA, 2017, IMMUNITY, V46, P957, DOI 10.1016/j.immuni.2017.06.006

Liddelow SA, 2017, NATURE, V541, P481, DOI 10.1038/nature21029

Lim WA, 2022, SCIENCE, V378, P848, DOI 10.1126/science.add9665

Liu F, 2021, CELL DEATH DIS, V12, DOI 10.1038/s41419-021-03498-x

Liu H, 2010, HEPATOLOGY, V51, P1810, DOI 10.1002/hep.23626

Liu MH, 2020, NEURAL REGEN RES, V15, P342, DOI 10.4103/1673-5374.265185

Liu Y, 2023, ACTA PHARM SIN B, V13, P662, DOI 10.1016/j.apsb.2022.07.021

Liu Y, 2015, NEUROPHARMACOLOGY, V91, P87, DOI 10.1016/j.neuropharm.2014.11.028

Liu YG, 2015, J NEUROSCI, V35, P9336, DOI 10.1523/JNEUROSCI.3975-14.2015

Llorente IL, 2021, STEM CELL RES, V55, DOI 10.1016/j.scr.2021.102458

Loria F, 2017, ACTA NEUROPATHOL, V134, P789, DOI 10.1007/s00401-017-1746-2

Luan X, 2017, ACTA PHARMACOL SIN, V38, P754, DOI 10.1038/aps.2017.12

Luk KC, 2012, SCIENCE, V338, P949, DOI 10.1126/science.1227157

MacAskill AF, 2009, NEURON, V61, P541, DOI 10.1016/j.neuron.2009.01.030

Maimon R, 2021, NAT NEUROSCI, V24, P1089, DOI 10.1038/s41593-021-00864-y

Maiorano NA, 2009, NEURAL DEV, V4, DOI 10.1186/1749-8104-4-40

Makeyev EV, 2007, MOL CELL, V27, P435, DOI 10.1016/j.molcel.2007.07.015

Mao SS, 2015, PROTEIN CELL, V6, P529, DOI 10.1007/s13238-015-0168-y

Mao XB, 2016, SCIENCE, V353, DOI 10.1126/science.aah3374

Martin-Jiménez CA, 2017, PROG NEUROBIOL, V158, P45, DOI 10.1016/j.pneurobio.2017.08.001

Matsuda T, 2019, NEURON, V101, P472, DOI 10.1016/j.neuron.2018.12.010

Matsuda-Ito K, 2022, SCI REP-UK, V12, DOI 10.1038/s41598-022-22802-z

Mattugini N, 2019, NEURON, V103, P1086, DOI 10.1016/j.neuron.2019.08.009

MIZUNO Y, 1994, ANN NEUROL, V35, P204, DOI 10.1002/ana.410350212

Niu WZ, 2018, STEM CELL REP, V11, P1156, DOI 10.1016/j.stemcr.2018.09.004

Niu WZ, 2013, NAT CELL BIOL, V15, P1164, DOI 10.1038/ncb2843

Oertel W, 2016, J NEUROCHEM, V139, P325, DOI 10.1111/jnc.13750

Ogura T, 2018, DEVELOPMENT, V145, DOI 10.1242/dev.162214

Packer AN, 2008, J NEUROSCI, V28, P14341, DOI 10.1523/JNEUROSCI.2390-08.2008

Pandey S, 2017, ANN INDIAN ACAD NEUR, V20, P190, DOI 10.4103/aian.AIAN_239_17

Park YJ, 2014, DIABETOLOGIA, V57, P765, DOI 10.1007/s00125-013-3152-1

Parmar M, 2020, NAT REV NEUROSCI, V21, P103, DOI 10.1038/s41583-019-0257-7

Piao JH, 2021, CELL STEM CELL, V28, P217, DOI 10.1016/j.stem.2021.01.004

Poewe W, 2017, NAT REV DIS PRIMERS, V3, DOI 10.1038/nrdp.2017.13

Poewe W, 2010, CLIN INTERV AGING, V5, P229, DOI 10.2147/CIA.86456

Powell EM, 1999, GLIA, V26, P73, DOI 10.1002/(SICI)1098-1136(199903)26:1<73::AID-GLIA8>3.0.CO;2-S

Proia P, 2008, INT J MOL MED, V21, P63

Proschel C, 2014, EMBO MOL MED, V6, P504, DOI 10.1002/emmm.201302878

Przedborski S, 2017, NAT REV NEUROSCI, V18, P251, DOI 10.1038/nrn.2017.25

Qian H, 2020, NATURE, V582, P550, DOI 10.1038/s41586-020-2388-4

Rao YX, 2021, NEURON, V109, P4094, DOI 10.1016/j.neuron.2021.11.008

Rekdal VM, 2019, SCIENCE, V364, P1055, DOI 10.1126/science.aau6323

Repici M, 2019, J CLIN MED, V8, DOI 10.3390/jcm8091377

Rohn TT, 2011, NEUROBIOL DIS, V43, P68, DOI 10.1016/j.nbd.2010.11.003

Ruiz S, 2010, PLOS ONE, V5, DOI 10.1371/journal.pone.0015526

Sarkar S, 2007, J BIOL CHEM, V282, P5641, DOI 10.1074/jbc.M609532200

Schousboe Arne, 2013, Front Endocrinol (Lausanne), V4, P102, DOI 10.3389/fendo.2013.00102

Schwartz M, 2014, EMBO J, V33, P7, DOI 10.1002/embj.201386609

Schweitzer JS, 2020, NEW ENGL J MED, V382, P1926, DOI 10.1056/NEJMoa1915872

Shimada IS, 2012, J NEUROSCI, V32, P7926, DOI 10.1523/JNEUROSCI.4303-11.2012

Simard M, 2004, NEUROSCIENCE, V129, P877, DOI 10.1016/j.neuroscience.2004.09.053

Sirko S, 2013, CELL STEM CELL, V12, P426, DOI 10.1016/j.stem.2013.01.019

Sofroniew MV, 2015, NAT REV NEUROSCI, V16, P249, DOI 10.1038/nrn3898

Sofroniew MV, 2010, ACTA NEUROPATHOL, V119, P7, DOI 10.1007/s00401-009-0619-8

Söllvander S, 2016, MOL NEURODEGENER, V11, DOI 10.1186/s13024-016-0098-z

Song B, 2020, J CLIN INVEST, V130, P904, DOI 10.1172/JCI130767

Song JJ, 2018, J CLIN INVEST, V128, P463, DOI 10.1172/JCI93924

Sorrentino ZA, 2019, ACTA NEUROPATHOL, V138, P1, DOI 10.1007/s00401-019-01977-2

Starkov AA, 2004, J NEUROSCI, V24, P7779, DOI 10.1523/JNEUROSCI.1899-04.2004

Stephen TL, 2015, J NEUROSCI, V35, P15996, DOI 10.1523/JNEUROSCI.2068-15.2015

Sun N, 2009, P NATL ACAD SCI USA, V106, P15720, DOI 10.1073/pnas.0908450106

Sun Y, 2021, STEM CELL TRANSL MED, V10, pS31, DOI 10.1002/sctm.20-0522

Takahashi Jun, 2020, Regen Ther, V13, P18, DOI 10.1016/j.reth.2020.06.002

Takahashi K, 2006, CELL, V126, P663, DOI 10.1016/j.cell.2006.07.024

Tamaoki N, 2010, J DENT RES, V89, P773, DOI 10.1177/0022034510366846

Tao YL, 2021, NAT MED, V27, DOI 10.1038/s41591-021-01257-1

Tian LL, 2022, INT J MOL SCI, V23, DOI 10.3390/ijms23020845

Torper O, 2013, P NATL ACAD SCI USA, V110, P7038, DOI 10.1073/pnas.1303829110

Treiber T, 2019, NAT REV MOL CELL BIO, V20, P5, DOI 10.1038/s41580-018-0059-1

van Kessel SP, 2019, NAT COMMUN, V10, DOI 10.1038/s41467-019-08294-y

Verkhratsky A, 2016, EMBO J, V35, P239, DOI 10.15252/embj.201592705

Visvanathan J, 2007, GENE DEV, V21, P744, DOI 10.1101/gad.1519107

Voutilainen MH, 2009, J NEUROSCI, V29, P9651, DOI 10.1523/JNEUROSCI.0833-09.2009

Walsh JG, 2014, NAT REV NEUROSCI, V15, P84, DOI 10.1038/nrn3638

Wang LL, 2021, CELL, V184, P5465, DOI 10.1016/j.cell.2021.09.005

Wang SW, 2011, J NEUROSCI, V31, P7275, DOI 10.1523/JNEUROSCI.6476-10.2011

Wang XN, 2009, CELL, V136, P163, DOI 10.1016/j.cell.2008.11.046

Weber EW, 2020, CELL, V181, P46, DOI 10.1016/j.cell.2020.03.001

West AP, 2015, NATURE, V520, P553, DOI 10.1038/nature14156

Wilmut I, 1997, NATURE, V385, P810, DOI 10.1038/385810a0

Wu Z, 2020, NAT COMMUN, V11, DOI 10.1038/s41467-020-14855-3

Xia Y, 2021, BRAIN, V144, P2024, DOI 10.1093/brain/awab122

Xiang ZQ, 2021, NEURAL REGEN RES, V16, P750, DOI 10.4103/1673-5374.295925

Xie Y, 2022, CELL REP, V39, DOI 10.1016/j.celrep.2022.110960

Xu JH, 2023, NAT BIOMED ENG, V7, P253, DOI 10.1038/s41551-022-00963-6

Xue YC, 2016, NAT NEUROSCI, V19, P807, DOI 10.1038/nn.4297

Xue YC, 2013, CELL, V152, P82, DOI 10.1016/j.cell.2012.11.045

Yang H, 2019, MOL THER, V27, P1467, DOI 10.1016/j.ymthe.2019.05.006

Yoo AS, 2011, NATURE, V476, P228, DOI 10.1038/nature10323

Yoo AS, 2009, NATURE, V460, P642, DOI 10.1038/nature08139

Yu XZ, 2020, NAT REV NEUROSCI, V21, P121, DOI 10.1038/s41583-020-0264-8

Yun SP, 2018, NAT MED, V24, P931, DOI 10.1038/s41591-018-0051-5

Zarei-Kheirabadi M, 2019, J CELL PHYSIOL, V234, P18697, DOI 10.1002/jcp.28510

Zhang L, 2020, FRONT CELL NEUROSCI, V14, DOI 10.3389/fncel.2020.594170

Zhang L, 2015, CELL STEM CELL, V17, P735, DOI 10.1016/j.stem.2015.09.012

Zhang YJ, 2022, P NATL ACAD SCI USA, V119, DOI 10.1073/pnas.2107339119

Zhou HB, 2020, CELL, V181, P590, DOI 10.1016/j.cell.2020.03.024

NR 198

TC 4

Z9 5

U1 3

U2 11

PU ACADEMIC PRESS INC ELSEVIER SCIENCE

PI SAN DIEGO

PA 525 B ST, STE 1900, SAN DIEGO, CA 92101-4495 USA

SN 0969-9961

EI 1095-953X

J9 NEUROBIOL DIS

JI Neurobiol. Dis.

PD AUG

PY 2023

VL 184

AR 106224

DI 10.1016/j.nbd.2023.106224

EA JUL 2023

PG 13

WC Neurosciences

WE Science Citation Index Expanded (SCI-EXPANDED)

SC Neurosciences & Neurology

GA P0VI8

UT WOS:001047899200001

PM 37433411

OA gold

DA 2024-08-12

ER

PT J

AU Xie, LL

Hu, LD

AF Xie, LinLin

Hu, LuDong

TI Research progress in the early diagnosis of Parkinson's disease

SO NEUROLOGICAL SCIENCES

LA English

DT Review

DE Parkinson's disease; Pathophysiology; Non-motor symptom; Neuroimaging

ID SLEEP BEHAVIOR DISORDER; INCREASED FREE-WATER; SUBSTANTIA-NIGRA;

NEUROINFLAMMATION; NEURODEGENERATION; HYPERINTENSITY; ACCURACY; SYSTEM;

TARGET; MODEL

AB Parkinson's disease is the second major neurodegenerative disease with increasing incidence and population in the world year by year. The pathogenesis of Parkinson's disease is still not completely clear, and the identification of Parkinson's disease prodromal manifestations and accurate diagnosis is still facing great challenges. This review summarizes the interaction of environmental toxicants, mitochondrial dysfunction, alpha-synuclein, gut microbiome dysbiosis, neuroinflammation, and other pathophysiological factors in Parkinson's disease. It also discusses the prodromal manifestations of Parkinson's disease, such as olfactory dysfunction, sleep dysfunction and other non-motor symptoms, the current diagnostic challenges, and the application status of emerging neuroimaging technologies such as magnetic resonance imaging (MRI), positron emission tomography (PET), and single-photon emission tomography (SPECT).

C1 [Xie, LinLin] Qingdao Univ, Qingdao 266071, Shandong, China.

[Hu, LuDong] Weifang Med Univ, Weifang 261053, Shandong, China.

C3 Qingdao University; Shandong Second Medical University

RP Xie, LL (corresponding author), Qingdao Univ, Qingdao 266071, Shandong, China.

EM XLL961231@163.com; 386811939@qq.com

RI Xie, linlin/GZG-9107-2022

OI Xie, LinLin/0000-0002-4798-2078

CR Archer DB, 2019, LANCET DIGIT HEALTH, V1, pE222, DOI 10.1016/S2589-7500(19)30105-0

Armstrong MJ, 2020, JAMA-J AM MED ASSOC, V323, P548, DOI 10.1001/jama.2019.22360

Aurora RN, 2010, J CLIN SLEEP MED, V6, P85

Bae YJ, 2016, MOVEMENT DISORD, V31, P684, DOI 10.1002/mds.26584

Beach TG, 2009, ACTA NEUROPATHOL, V117, P613, DOI [10.1007/s00401-010-0664-3, 10.1007/s00401-009-0538-8]

Betarbet R, 2000, NAT NEUROSCI, V3, P1301, DOI 10.1038/81834

Bloem BR, 2021, LANCET, V397, P2284, DOI 10.1016/S0140-6736(21)00218-X

Boertien JM, 2019, J PARKINSON DIS, V9, pS297, DOI 10.3233/JPD-191711

Braak H, 2003, NEUROBIOL AGING, V24, P197, DOI 10.1016/S0197-4580(02)00065-9

Brochard V, 2009, J CLIN INVEST, V119, P182, DOI 10.1172/JCI36470

Burciu RG, 2017, BRAIN, V140, P2183, DOI 10.1093/brain/awx146

Chiu CC, 2019, MOL NEUROBIOL, V56, P3835, DOI 10.1007/s12035-018-1118-5

Darweesh SKL, 2017, BRAIN, V140, P429, DOI 10.1093/brain/aww291

De Marzi R, 2016, ANN NEUROL, V79, P1026, DOI 10.1002/ana.24646

Erny D, 2015, NAT NEUROSCI, V18, P965, DOI 10.1038/nn.4030

Feigin VL, 2019, LANCET NEUROL, V18, P459, DOI [10.1016/S1474-4422(18)30499-X, 10.1016/S1474-4422(19)30034-1, 10.1016/S1474-4422(18)30415-0]

Fereshtehnejad SM, 2019, BRAIN, V142, P2051, DOI 10.1093/brain/awz111

Galbiati A, 2019, SLEEP MED REV, V43, P37, DOI 10.1016/j.smrv.2018.09.008

Hirsch EC, 2009, LANCET NEUROL, V8, P382, DOI 10.1016/S1474-4422(09)70062-6

Jin LR, 2019, FRONT NEUROL, V10, DOI 10.3389/fneur.2019.00100

Kasten M, 2018, MOVEMENT DISORD, V33, P730, DOI 10.1002/mds.27352

Keshavarzian A, 2015, MOVEMENT DISORD, V30, P1351, DOI 10.1002/mds.26307

Knudsen K, 2018, LANCET NEUROL, V17, P618, DOI 10.1016/S1474-4422(18)30162-5

Kordower JH, 2013, BRAIN, V136, P2419, DOI 10.1093/brain/awt192

Lerche S, 2014, J NEUROL, V261, P180, DOI 10.1007/s00415-013-7171-0

Liddelow SA, 2017, NATURE, V541, P481, DOI 10.1038/nature21029

Marques TM, 2019, NEUROLOGY, V92, pE1479, DOI 10.1212/WNL.0000000000007179

Marsili L, 2018, FRONT NEUROL, V9, DOI 10.3389/fneur.2018.00156

Mueller C, 2018, PARKINSONISM RELAT D, V54, P90, DOI 10.1016/j.parkreldis.2018.04.005

Noh Y, 2015, AM J NEURORADIOL, V36, P2010, DOI 10.3174/ajnr.A4412

Ofori E, 2015, NEUROBIOL AGING, V36, P1097, DOI 10.1016/j.neurobiolaging.2014.10.029

Pont-Sunyer C, 2015, MOVEMENT DISORD, V30, P229, DOI 10.1002/mds.26077

Postuma RB, 2018, MOVEMENT DISORD, V33, P1601, DOI 10.1002/mds.27362

Postuma RB, 2015, MOVEMENT DISORD, V30, P1591, DOI 10.1002/mds.26424

Quadalti C, 2021, NPJ PARKINSONS DIS, V7, DOI 10.1038/s41531-021-00232-4

Rajput AH, 2014, NEUROLOGY, V83, P386, DOI 10.1212/WNL.0000000000000653

Rizzo G, 2016, NEUROLOGY, V86, P566, DOI 10.1212/WNL.0000000000002350

Ross G Webster, 2012, Parkinsonism Relat Disord, V18 Suppl 1, pS199, DOI 10.1016/S1353-8020(11)70062-1

Rouaud T, 2021, REV NEUROL-FRANCE, V177, P260, DOI 10.1016/j.neurol.2020.07.016

Saeed U, 2017, TRANSL NEURODEGENER, V6, DOI 10.1186/s40035-017-0076-6

Sampson TR, 2016, CELL, V167, P1469, DOI 10.1016/j.cell.2016.11.018

Sasaki M, 2006, NEUROREPORT, V17, P1215, DOI 10.1097/01.wnr.0000227984.84927.a7

Scherfler C, 2007, MOVEMENT DISORD, V22, P1229, DOI 10.1002/mds.21505

Schröder JB, 2018, FRONT NEUROL, V9, DOI 10.3389/fneur.2018.01081

Simuni T, 2018, MOVEMENT DISORD, V33, P771, DOI 10.1002/mds.27361

Simuni T, 2016, PARKINSONISM RELAT D, V28, P62, DOI 10.1016/j.parkreldis.2016.04.027

SINGER TP, 1990, FEBS LETT, V274, P1, DOI 10.1016/0014-5793(90)81315-F

Strafella AP, 2017, MOVEMENT DISORD, V32, P181, DOI 10.1002/mds.26907

Sun JY, 2020, MOVEMENT DISORD, V35, P478, DOI 10.1002/mds.27929

Thiruchelvam M, 2000, J NEUROSCI, V20, P9207

Travin MI, 2019, J NUCL CARDIOL, V26, P1434, DOI 10.1007/s12350-018-1394-5

Trinh J, 2018, MOVEMENT DISORD, V33, P1857, DOI 10.1002/mds.27527

Vollstedt EJ, 2019, ANN NEUROL, V86, P153, DOI 10.1002/ana.25514

Volpicelli-Daley Laura, 2018, Handb Clin Neurol, V153, P321, DOI 10.1016/B978-0-444-63945-5.00017-9

Walter U, 2018, FRONT NEUROL, V9, DOI 10.3389/fneur.2018.00805

Wang T, 2022, NEUROSCIENTIST, V28, P364, DOI 10.1177/1073858421991066

Wijeyekoon Ruwani S, 2018, Front Neurol, V9, P870, DOI 10.3389/fneur.2018.00870

Zhou LC, 2021, BRAIN, V144, P1488, DOI 10.1093/brain/awab039

Zhou RB, 2011, NATURE, V469, P221, DOI 10.1038/nature09663

NR 59

TC 5

Z9 5

U1 8

U2 56

PU SPRINGER-VERLAG ITALIA SRL

PI MILAN

PA VIA DECEMBRIO, 28, MILAN, 20137, ITALY

SN 1590-1874

EI 1590-3478

J9 NEUROL SCI

JI Neurol. Sci.

PD NOV

PY 2022

VL 43

IS 11

BP 6225

EP 6231

DI 10.1007/s10072-022-06316-0

EA AUG 2022

PG 7

WC Clinical Neurology; Neurosciences

WE Science Citation Index Expanded (SCI-EXPANDED)

SC Neurosciences & Neurology

GA 5U5OA

UT WOS:000838496500002

PM 35948779

DA 2024-08-12

ER

PT J

AU Pacifici, F

Salimei, C

Pastore, D

Malatesta, G

Ricordi, C

Donadel, G

Bellia, A

Rovella, V

Tafani, M

Garaci, E

Tesauro, M

Lauro, D

Di Daniele, N

Della-Morte, D

AF Pacifici, Francesca

Salimei, Chiara

Pastore, Donatella

Malatesta, Gina

Ricordi, Camillo

Donadel, Giulia

Bellia, Alfonso

Rovella, Valentina

Tafani, Marco

Garaci, Enrico

Tesauro, Manfredi

Lauro, Davide

Di Daniele, Nicola

Della-Morte, David

TI The Protective Effect of a Unique Mix of Polyphenols and Micronutrients

against Neurodegeneration Induced by an In Vitro Model of Parkinson's

Disease

SO INTERNATIONAL JOURNAL OF MOLECULAR SCIENCES

LA English

DT Article

DE SIRT1; polyphenols; Parkinson's disease; inflammation; oxidative stress

ID RESVERATROL; NEUROINFLAMMATION; APOPTOSIS; TOXICITY; PROTEIN; CELLS

AB Parkinson's disease (PD) is second-most common disabling neurological disorder worldwide, and unfortunately, there is not yet a definitive way to prevent it. Polyphenols have been widely shown protective efficacy against various PD symptoms. However, data on their effect on physio-pathological mechanisms underlying this disease are still lacking. In the present work, we evaluated the activity of a mixture of polyphenols and micronutrients, named A5(+), in the murine neuroblastoma cell line N1E115 treated with 6-Hydroxydopamine (6-OHDA), an established neurotoxic stimulus used to induce an in vitro PD model. We demonstrate that a pretreatment of these cells with A5(+) causes significant reduction of inflammation, resulting in a decrease in pro-inflammatory cytokines (IFN-gamma, IL-6, TNF-alpha, and CXCL1), a reduction in ROS production and activation of extracellular signal-regulated kinases (ERK)1/2, and a decrease in apoptotic mechanisms with the related increase in cell viability. Intriguingly, A5(+) treatment promoted cellular differentiation into dopaminergic neurons, as evident by the enhancement in the expression of tyrosine hydroxylase, a well-established dopaminergic neuronal marker. Overall, these results demonstrate the synergic and innovative efficacy of A5(+) mixture against PD cellular pathological processes, although further studies are needed to clarify the mechanisms underlying its beneficial effect.

C1 [Pacifici, Francesca; Salimei, Chiara; Bellia, Alfonso; Rovella, Valentina; Tesauro, Manfredi; Lauro, Davide; Di Daniele, Nicola; Della-Morte, David] Univ Roma Tor Vergata, Dept Syst Med, I-00133 Rome, Italy.

[Pastore, Donatella; Garaci, Enrico; Della-Morte, David] San Raffaele Univ, Dept Human Sci & Qual Life Promot, I-00166 Rome, Italy.

[Malatesta, Gina] Univ Roma Tor Vergata, Dept Biomed & Prevent, I-00133 Rome, Italy.

[Ricordi, Camillo] Univ Miami, Cell Transplant Ctr, Miller Sch Med, Diabet Res Inst, Miami, FL 33136 USA.

[Donadel, Giulia; Tesauro, Manfredi; Lauro, Davide; Di Daniele, Nicola] Univ Roma Tor Vergata, Dept Clin Sci & Translat Med, I-00133 Rome, Italy.

[Bellia, Alfonso; Rovella, Valentina] Fdn Policlin Tor Vergata, Dept Med Sci, I-00133 Rome, Italy.

[Tafani, Marco] Sapienza Univ Rome, Dept Expt Med, I-00161 Rome, Italy.

[Della-Morte, David] Univ Miami, Miller Sch Med, Evelyn F McKnight Brain Inst, Dept Neurol, Miami, FL 33136 USA.

[Della-Morte, David] Univ Roma Tor Vergata, Interdisciplinary Ctr Adv Studies Lab Chip & Orga, I-00133 Rome, Italy.

C3 University of Rome Tor Vergata; Vita-Salute San Raffaele University;

University of Rome Tor Vergata; University of Miami; University of Rome

Tor Vergata; University of Rome Tor Vergata; Sapienza University Rome;

University of Miami; University of Rome Tor Vergata

RP Della-Morte, D (corresponding author), Univ Roma Tor Vergata, Dept Syst Med, I-00133 Rome, Italy.; Della-Morte, D (corresponding author), San Raffaele Univ, Dept Human Sci & Qual Life Promot, I-00166 Rome, Italy.; Della-Morte, D (corresponding author), Univ Miami, Miller Sch Med, Evelyn F McKnight Brain Inst, Dept Neurol, Miami, FL 33136 USA.; Della-Morte, D (corresponding author), Univ Roma Tor Vergata, Interdisciplinary Ctr Adv Studies Lab Chip & Orga, I-00133 Rome, Italy.

EM francesca.pacifici@uniroma2.it; chiara.salimei@gmail.com;

d.pastore3@inwind.it; G.malatesta@outlook.it; ricordi@miami.edu;

donadel@uniroma2.it; bellia@med.uniroma2.it; valerovix@yahoo.it;

marco.tafani@uniroma1.it; enrico.garaci@sanraffaele.it;

mtesauro@tiscali.it; d.lauro@med.uniroma2.it; didaniele@med.uniroma2.it;

david.dellamorte@uniroma2.it

RI Donadel, Giulia/M-1900-2019; bellia, alfonso/AIA-9860-2022; Lauro,

Davide/K-4683-2018; Tafani, Marco/C-3275-2017; Pacifici,

Francesca/J-2800-2018

OI Donadel, Giulia/0000-0002-2454-5940; Tafani, Marco/0000-0001-7128-810X;

Lauro, Davide/0000-0002-8597-4415; SALIMEI, CHIARA/0000-0002-1649-0417;

Pacifici, Francesca/0000-0001-9014-7492; Rovella,

Valentina/0000-0002-3311-486X

FU Fondazione Roma Diabetes Mellitus; Regenerative and Reparative

Processes; Improvement of Pancreatic Beta Cell Function: Role of Bone

MarrowMesenchymal Stem Cells, MicroRNAs; M2 Macrophages and Myeloid

Derived Suppressor Cells; Evelyn F. McKnight Brain Institute

FX This research was funded by Fondazione Roma Diabetes Mellitus,

Regenerative and Reparative Processes, and Improvement of Pancreatic

Beta Cell Function: Role of Bone MarrowMesenchymal Stem Cells,

MicroRNAs, M2 Macrophages and Myeloid Derived Suppressor Cells; The

Evelyn F. McKnight Brain Institute.

CR Arbo BD, 2020, FRONT AGING NEUROSCI, V12, DOI 10.3389/fnagi.2020.00103

Bai H, 2020, NEUROCHEM INT, V134, DOI 10.1016/j.neuint.2020.104671

Chen LY, 2013, FOOD CHEM TOXICOL, V52, P28, DOI 10.1016/j.fct.2012.10.037

Chen YP, 2015, MOL NEURODEGENER, V10, DOI 10.1186/1750-1326-10-4

Daubner SC, 2011, ARCH BIOCHEM BIOPHYS, V508, P1, DOI 10.1016/j.abb.2010.12.017

De Angelis M, 2021, BIOMEDICINES, V9, DOI 10.3390/biomedicines9111721

Elyasi L, 2021, J BASIC CLIN PHYSIOL, V32, P11, DOI 10.1515/jbcpp-2019-0270

Emamzadeh FN, 2018, FRONT NEUROSCI-SWITZ, V12, DOI 10.3389/fnins.2018.00612

Erkkinen MG, 2018, CSH PERSPECT BIOL, V10, DOI 10.1101/cshperspect.a033118

Galvan A, 2008, CLIN NEUROPHYSIOL, V119, P1459, DOI 10.1016/j.clinph.2008.03.017

Goes ATR, 2018, CHEM-BIOL INTERACT, V279, P111, DOI 10.1016/j.cbi.2017.10.019

Guicciardi ME, 2009, FASEB J, V23, P1625, DOI 10.1096/fj.08-111005

Hirsch EC, 2009, LANCET NEUROL, V8, P382, DOI 10.1016/S1474-4422(09)70062-6

Huang BX, 2018, FRONT IMMUNOL, V9, DOI 10.3389/fimmu.2018.02527

Huang N, 2019, EXP GERONTOL, V124, DOI 10.1016/j.exger.2019.110653

Jankovic J, 2008, J NEUROL NEUROSUR PS, V79, P368, DOI 10.1136/jnnp.2007.131045

Javaid N, 2021, CURR PHARM DESIGN, V27, P3591, DOI 10.2174/1381612826666201112144006

Jin F, 2008, EUR J PHARMACOL, V600, P78, DOI 10.1016/j.ejphar.2008.10.005

Katsetos CD, 2003, J CHILD NEUROL, V18, P851, DOI 10.1177/088307380301801205

Kujawska M, 2018, NUTRIENTS, V10, DOI 10.3390/nu10050642

Li QS, 2018, J CELL BIOCHEM, V119, P7053, DOI 10.1002/jcb.27023

Mamelak M, 2018, NEUROL THER, V7, P5, DOI 10.1007/s40120-018-0091-2

Mhyre Timothy R, 2012, Subcell Biochem, V65, P389, DOI 10.1007/978-94-007-5416-4_16

Muzerengi S, 2007, PARKINSONISM RELAT D, V13, pS450, DOI 10.1016/S1353-8020(08)70048-8

Nagatsu T, 2002, J NEURAL TRANSM, V109, P731, DOI 10.1007/s007020200061

Nuttall JR, 2012, NEUROTOX RES, V21, P128, DOI 10.1007/s12640-011-9291-6

Oh J, 2005, NEUROCHEM RES, V30, P333, DOI 10.1007/s11064-005-2607-2

Pacifici F, 2020, ANTIOXIDANTS-BASEL, V9, DOI 10.3390/antiox9040329

Palle S, 2018, N-S ARCH PHARMACOL, V391, P445, DOI 10.1007/s00210-018-1474-8

Potdar S, 2018, ARCH TOXICOL, V92, P669, DOI 10.1007/s00204-017-2073-z

Rea S, 2020, FRONT PHARMACOL, V11, DOI 10.3389/fphar.2020.00779

Sairanen T, 2009, ACTA NEUROPATHOL, V118, P541, DOI 10.1007/s00401-009-0559-3

Sarkaki Alireza, 2016, Acta Med Iran, V54, P494

Song Z, 2015, J PHARM PHARMACOL, V67, P720, DOI 10.1111/jphp.12360

Storch A, 2000, J NEURAL TRANSM, V107, P281, DOI 10.1007/s007020050023

Surguchov A, 2021, BIOMOLECULES, V11, DOI 10.3390/biom11050624

Talarek S, 2017, BIOFACTORS, V43, P760, DOI 10.1002/biof.1385

Traore K, 2008, J CELL PHYSIOL, V216, P276, DOI 10.1002/jcp.21403

Troncoso-Escudero P, 2018, FRONT NEUROL, V9, DOI 10.3389/fneur.2018.00860

Vermes I, 2000, J IMMUNOL METHODS, V243, P167, DOI 10.1016/S0022-1759(00)00233-7

Wang P, 2018, BIOFACTORS, V44, P16, DOI 10.1002/biof.1410

Wang YH, 2013, MOLECULES, V18, P14726, DOI 10.3390/molecules181214726

Weng MR, 2018, PARKINSONS DIS-US, V2018, DOI 10.1155/2018/9163040

Xu ZH, 2021, FRONT NEUROSCI-SWITZ, V15, DOI 10.3389/fnins.2021.631501

Yan JQ, 2014, MOL MED REP, V10, P2223, DOI 10.3892/mmr.2014.2563

Zhu Q, 2020, OXID MED CELL LONGEV, V2020, DOI 10.1155/2020/3986348

NR 46

TC 7

Z9 7

U1 1

U2 1

PU MDPI

PI BASEL

PA ST ALBAN-ANLAGE 66, CH-4052 BASEL, SWITZERLAND

EI 1422-0067

J9 INT J MOL SCI

JI Int. J. Mol. Sci.

PD MAR

PY 2022

VL 23

IS 6

AR 3110

DI 10.3390/ijms23063110

PG 14

WC Biochemistry & Molecular Biology; Chemistry, Multidisciplinary

WE Science Citation Index Expanded (SCI-EXPANDED)

SC Biochemistry & Molecular Biology; Chemistry

GA 0C6AW

UT WOS:000775394700001

PM 35328530

OA Green Published, gold

DA 2024-08-12

ER

PT J

AU Jang, JH

Yeom, MJ

Ahn, S

Oh, JY

Ji, S

Kim, TH

Park, HJ

AF Jang, Jae-Hwan

Yeom, Mi-Jung

Ahn, Sora

Oh, Ju-Young

Ji, Suhwan

Kim, Tae-Hong

Park, Hi-Joon

TI Acupuncture inhibits neuroinflammation and gut microbial dysbiosis in a

mouse model of Parkinson's disease

SO BRAIN BEHAVIOR AND IMMUNITY

LA English

DT Article

DE Acupuncture; Parkinson's disease; Gut microbial dysbiosis;

Neuroinflammation; Microbiome-gut-brain axis

ID ELECTROACUPUNCTURE STIMULATION; BRAIN; EXPRESSION; COMPLEMENTARY;

THERAPY; SYSTEM; AXIS

AB Growing evidences show that gut microbiota is associated with the pathogenesis of Parkinson's disease (PD) and the gut-brain axis can be promising target for the development of the therapeutic strategies for PD. Acupuncture has been used to improve brain functions and inflammation in neurological disorders such as PD, and to recover the gastrointestinal dysfunctions in various gastrointestinal disorders. Thus, we investigated whether acupuncture could improve Parkinsonism and gut microbial dysbiosis induced by 1-methyl-4-phenyl-1,2,3,6-tetrahydropyridine. First, we observed that acupuncture treatment at acupoints GB34 and ST36 could improve motor functions and comorbid anxiety in PD mice. Next, we found that acupuncture increased the levels of dopaminergic fibers and neurons in the striatum and the substantia nigra, respectively. Acupuncture also restored the overexpression of microglia and astrocyte as well as conversion of Bax and Bcl-2 expression in both the striatum and the substantia nigra, indicating that inflammatory responses and apoptosis were blocked by acupuncture. Additionally, via 16S rRNA sequence analysis, we observed that the relative abundance of 18 genera were changed in acupuncture-treated mice compared to the PD mice. Of them, Butyricimonas, Holdemania, Frisingicoccus, Gracilibacter, Phocea, and Aestuariispira showed significant correlations with anxiety as well as motor functions. Furthermore, the predicted functional analyses showed that acupuncture restored the physiology functions such as glutathione metabolism, methane metabolism, and PD pathway. In conclusion, we suggest that the effects of acupuncture on the enhanced motor function and the protection of the dopaminergic neurons may be associated with the regulation of the gut microbial dysbiosis and thus the inhibition of the neuroinflammation in the PD mice.

C1 [Jang, Jae-Hwan; Yeom, Mi-Jung; Ahn, Sora; Oh, Ju-Young; Kim, Tae-Hong; Park, Hi-Joon] Kyung Hee Univ, Acupuncture & Meridian Sci Res Ctr, Integrat Parkinsons Dis Res Grp, 26 Kyungheedae Ro, Seoul 02447, South Korea.

[Ji, Suhwan; Park, Hi-Joon] Kyung Hee Univ, Grad Sch Korean Med, Dept Korean Med Sci, 26 Kyungheedae Ro, Seoul 02447, South Korea.

C3 Kyung Hee University; Kyung Hee University

RP Park, HJ (corresponding author), Kyung Hee Univ, Coll Korean Med, Acupuncture & Meridian Sci Res Ctr AMSRC, 26 Kyungheedae Ro, Seoul 02447, South Korea.

EM acufind@khu.ac.kr

RI Oh, Ju-Young/AHB-2445-2022; Park, Hi-Joon/AAG-1196-2021

OI Oh, Ju-Young/0000-0001-9141-0133; Park, Hi-Joon/0000-0002-0538-2664;

Jang, Jae-Hwan/0000-0001-8422-5183

FU National Research Foundation of Korea - Korean government

[NRF-2017R1A2B4009963, 2020R1A4A1018598]; Korea Institute of Oriental

Medicine [K18182]

FX The present research was supported by grants from the National Research

Foundation of Korea funded by the Korean government

(NRF-2017R1A2B4009963 and 2020R1A4A1018598) and from the Korea Institute

of Oriental Medicine (grant K18182).

CR Abraham TS, 2011, J CHEM NEUROANAT, V41, P129, DOI 10.1016/j.jchemneu.2011.01.001

Ahn S, 2019, FRONT AGING NEUROSCI, V11, DOI 10.3389/fnagi.2019.00230

AVTANDILOV GG, 1991, ARK PATOL, V53, P41

Bonaz B, 2018, FRONT NEUROSCI-SWITZ, V12, DOI 10.3389/fnins.2018.00049

Borovikova LV, 2000, NATURE, V405, P458, DOI 10.1038/35013070

Cai RL, 2018, J INTEGR MED-JIM, V16, P26, DOI 10.1016/j.joim.2017.12.002

Chae Y, 2009, MOVEMENT DISORD, V24, P1794, DOI 10.1002/mds.22673

Chey WD, 2011, GUT LIVER, V5, P253, DOI 10.5009/gnl.2011.5.3.253

Choi VM, 2016, NAT MED, V22, P563, DOI 10.1038/nm.4077

Chon TY, 2013, MAYO CLIN PROC, V88, P1141, DOI 10.1016/j.mayocp.2013.06.009

Chung YCE, 2019, J PSYCHIATR RES, V111, P74, DOI 10.1016/j.jpsychires.2019.01.016

DELAHOOKE DM, 1995, INFECT IMMUN, V63, P840, DOI 10.1128/IAI.63.3.840-846.1995

Dickson DW, 2012, CSH PERSPECT MED, V2, DOI 10.1101/cshperspect.a009258

Santos JG, 2008, PHYSIOL BEHAV, V93, P155, DOI 10.1016/j.physbeh.2007.08.014

Du MH, 2013, WORLD J GASTROENTERO, V19, P5988, DOI 10.3748/wjg.v19.i36.5988

EDWARDS LL, 1991, MOVEMENT DISORD, V6, P151, DOI 10.1002/mds.870060211

Erny D, 2015, NAT NEUROSCI, V18, P965, DOI 10.1038/nn.4030

Felice VD, 2016, PARKINSONISM RELAT D, V27, P1, DOI 10.1016/j.parkreldis.2016.03.012

Baizabal-Carvallo JF, 2020, NEUROSCIENCE, V432, P160, DOI 10.1016/j.neuroscience.2020.02.030

Gao HM, 2011, ENVIRON HEALTH PERSP, V119, P807, DOI 10.1289/ehp.1003013

Ghaisas S, 2016, PHARMACOL THERAPEUT, V158, P52, DOI 10.1016/j.pharmthera.2015.11.012

Goldman N, 2010, NAT NEUROSCI, V13, P883, DOI 10.1038/nn.2562

Gorecki AM, 2019, FRONT NEUROSCI-SWITZ, V13, DOI 10.3389/fnins.2019.00839

Hartmann Andreas, 2004, Dialogues Clin Neurosci, V6, P281

Hu S, 2013, EVID-BASED COMPL ALT, V2013, DOI 10.1155/2013/592127

Huang M, 2018, SCI REP-UK, V8, DOI 10.1038/s41598-018-24654-y

Huang Y, 2010, INT J NEUROSCI, V120, P150, DOI 10.3109/00207450903316527

Hur J., 1991, NAMSANDANG, P1

Jang JH, 2019, FRONT NEUROSCI-SWITZ, V13, DOI 10.3389/fnins.2019.00995

Jankovic J, 2008, J NEUROL NEUROSUR PS, V79, P368, DOI 10.1136/jnnp.2007.131045

Jeon S, 2008, PROTEOMICS, V8, P4822, DOI 10.1002/pmic.200700955

Keshavarzian A, 2015, MOVEMENT DISORD, V30, P1351, DOI 10.1002/mds.26307

Kim S, 2019, NEURON, V103, P627, DOI 10.1016/j.neuron.2019.05.035

Kim SN, 2011, PLOS ONE, V6, DOI 10.1371/journal.pone.0027566

Kim SN, 2011, INT J NEUROSCI, V121, P562, DOI 10.3109/00207454.2011.591515

Kung YY, 2006, AM J CHINESE MED, V34, P47, DOI 10.1142/S0192415X0600362X

Lai F, 2018, NEUROCHEM RES, V43, P1986, DOI 10.1007/s11064-018-2620-x

Langille MGI, 2013, NAT BIOTECHNOL, V31, P814, DOI 10.1038/nbt.2676

Li XM, 2017, FREE RADICAL BIO MED, V108, P32, DOI 10.1016/j.freeradbiomed.2017.03.011

Liu M, 2011, PARKINSONS DIS-US, V2011, DOI 10.4061/2011/327089

Mitchell RW, 2011, J NEUROCHEM, V117, P735, DOI 10.1111/j.1471-4159.2011.07245.x

Mulak A, 2015, WORLD J GASTROENTERO, V21, P10609, DOI 10.3748/wjg.v21.i37.10609

Na BJ, 2009, NEUROSCI LETT, V464, P1, DOI 10.1016/j.neulet.2009.08.009

Park HJ, 2003, EXP NEUROL, V180, P92, DOI 10.1016/S0014-4886(02)00031-6

Park JY, 2018, J INFLAMM RES, V11, P227, DOI 10.2147/JIR.S141198

Park JY, 2017, MOL NEUROBIOL, V54, P7706, DOI 10.1007/s12035-016-0258-8

Park JY, 2015, BIOCHEM BIOPH RES CO, V460, P772, DOI 10.1016/j.bbrc.2015.03.105

Park JY, 2014, J PAIN, V15, P535, DOI 10.1016/j.jpain.2014.01.498

Qian YW, 2018, BRAIN BEHAV IMMUN, V70, P194, DOI 10.1016/j.bbi.2018.02.016

Rho SW, 2008, MOL CELLS, V25, P178

Sampson TR, 2016, CELL, V167, P1469, DOI 10.1016/j.cell.2016.11.018

Scheperjans F, 2015, MOVEMENT DISORD, V30, P350, DOI 10.1002/mds.26069

Sedelis M, 2001, BEHAV BRAIN RES, V125, P109, DOI 10.1016/S0166-4328(01)00309-6

SHIRAI J, 1988, ANN RECH VET, V19, P267

Song YF, 2020, AM J CHINESE MED, V48, P77, DOI 10.1142/S0192415X20500044

Sun MF, 2018, BRAIN BEHAV IMMUN, V70, P48, DOI 10.1016/j.bbi.2018.02.005

Torres-Rosas R, 2014, NAT MED, V20, P291, DOI 10.1038/nm.3479

Triantafyllou K, 2014, J NEUROGASTROENTEROL, V20, P31, DOI 10.5056/jnm.2014.20.1.31

Tysnes OB, 2017, J NEURAL TRANSM, V124, P901, DOI 10.1007/s00702-017-1686-y

Villarán RF, 2010, J NEUROCHEM, V114, P1687, DOI 10.1111/j.1471-4159.2010.06879.x

Wang HY, 2019, EVID-BASED COMPL ALT, V2019, DOI 10.1155/2019/4958294

Wang H, 2005, WORLD J GASTROENTERO, V11, P5517, DOI 10.3748/wjg.v11.i35.5517

Wang H, 2015, EVID-BASED COMPL ALT, V2015, DOI 10.1155/2015/787393

Yeo S, 2014, BMC COMPLEM ALTERN M, V14, DOI 10.1186/1472-6882-14-336

Zhou H, 2013, SHOCK, V40, P144, DOI 10.1097/SHK.0b013e318299e9c0

NR 65

TC 119

Z9 130

U1 16

U2 86

PU ACADEMIC PRESS INC ELSEVIER SCIENCE

PI SAN DIEGO

PA 525 B ST, STE 1900, SAN DIEGO, CA 92101-4495 USA

SN 0889-1591

EI 1090-2139

J9 BRAIN BEHAV IMMUN

JI Brain Behav. Immun.

PD OCT

PY 2020

VL 89

BP 641

EP 655

DI 10.1016/j.bbi.2020.08.015

PG 15

WC Immunology; Neurosciences; Psychiatry

WE Science Citation Index Expanded (SCI-EXPANDED)

SC Immunology; Neurosciences & Neurology; Psychiatry

GA OH9GJ

UT WOS:000582897600068

PM 32827699

OA hybrid

DA 2024-08-12

ER

PT J

AU Fleury, V

Zekeridou, A

Lazarevic, V

Gaia, N

Giannopoulou, C

Genton, L

Cancela, J

Girard, M

Goldstein, R

Bally, JF

Mombelli, A

Schrenzel, J

Burkhard, PR

AF Fleury, Vanessa

Zekeridou, Alkisti

Lazarevic, Vladimir

Gaia, Nadia

Giannopoulou, Catherine

Genton, Laurence

Cancela, Jose

Girard, Myriam

Goldstein, Rachel

Bally, Julien F.

Mombelli, Andrea

Schrenzel, Jacques

Burkhard, Pierre R.

TI Oral Dysbiosis and Inflammation in Parkinson's Disease

SO JOURNAL OF PARKINSONS DISEASE

LA English

DT Article

DE Oral microbiome; inflammation; biomarker; non-motor symptoms; cytokine;

Parkinson's disease; microbiota

ID GLAND NEEDLE-BIOPSY; ALPHA-SYNUCLEIN; GUT MICROBIOTA;

STREPTOCOCCUS-MUTANS; IMMUNE-RESPONSES; PATHOLOGY; CARIES; SCALE;

NEUROINFLAMMATION; QUESTIONNAIRE

AB Background: Oral microbiota has largely escaped attention in Parkinson's disease (PD), despite its pivotal role in maintaining oral and systemic health.

Objective: The aim of our study was to examine the composition of the oral microbiota and the degree of oral inflammation in PD.

Methods: Twenty PD patients were compared to 20 healthy controls. Neurological, periodontal and dental examinations were performed as well as dental scaling and gingival crevicular fluid sampling for cytokines measurement (interleukine (IL)-1 beta, IL-6, IL-1 receptor antagonist (RA), interferon-lambda and tumor necrosis factor (TNF)-alpha). Two months later, oral microbiota was sampled from saliva and subgingival dental plaque. A 16S rRNA gene amplicon sequencing was used to assess bacterial communities.

Results: PD patients were in the early and mid-stage phases of their disease (Hoehn & Yahr 2-2.5). Dental and periodontal parameters did not differ between groups. The levels of IL-1 beta and IL-1RA were significantly increased in patients compared to controls with a trend for an increased level of TNF-alpha in patients. Both saliva and subgingival dental plaque microbiota differed between patients and controls. Streptococcus mutans, Kingella oralis, Actinomyces AFQC s, Veillonella AFUJ s, Scardovia, Lactobacillaceae, Negativicutes and Firmicutes were more abundant in patients, whereas Treponema KE332528 s, Lachnospiraceae AM420052 s, and phylum SR1 were less abundant.

Conclusion: Our findings show that the oral microbiome is altered in early and mid-stage PD. Although PD patients had good dental and periodontal status, local inflammation was already present in the oral cavity. The relationship between oral dysbiosis, inflammation and the pathogenesis of PD requires further study.

C1 [Fleury, Vanessa; Schrenzel, Jacques; Burkhard, Pierre R.] Univ Geneva, Fac Med, CMU, Geneva, Switzerland.

[Fleury, Vanessa; Goldstein, Rachel; Bally, Julien F.; Burkhard, Pierre R.] Geneva Univ Hosp, Div Neurol, Rue Gabrielle Perret Gentil 4, CH-1211 Geneva 14, Switzerland.

[Zekeridou, Alkisti; Giannopoulou, Catherine; Cancela, Jose; Mombelli, Andrea] Univ Geneva, Univ Clin Dent Med, Div Periodontol, Geneva, Switzerland.

[Lazarevic, Vladimir; Gaia, Nadia; Girard, Myriam; Schrenzel, Jacques] Geneva Univ Hosp, Serv Infect Dis, Genom Res Lab, Geneva, Switzerland.

[Genton, Laurence] Geneva Univ Hosp, Clin Nutr, Geneva, Switzerland.

[Genton, Laurence] Univ Geneva, Geneva, Switzerland.

C3 University of Geneva; University of Geneva; University of Geneva;

University of Geneva; University of Geneva

RP Fleury, V (corresponding author), Geneva Univ Hosp, Div Neurol, Rue Gabrielle Perret Gentil 4, CH-1211 Geneva 14, Switzerland.

EM Vanessa.FleuryNissen@hcuge.ch

RI Fleury, Vanessa/AAC-4584-2020; Schrenzel, Jacques/P-8525-2017;

Lazarevic, Vladimir/H-4755-2019; Genton, Laurence/HKE-7419-2023

OI Fleury, Vanessa/0000-0001-5744-6829; Lazarevic,

Vladimir/0000-0002-1782-4423; Lazarevic, Vladimir/0000-0002-6839-2536;

Bally, Julien/0000-0002-0970-8844

FU Association Swiss Parkinson

FX Wewould like to thank Lotfi Hatjas for his help during the recruitment

phase of the study. We thank Dr Michael Nissen for his assistance for

proofreading. This studywas supported by a grant from the Association

Swiss Parkinson.

CR Adler CH, 2016, MOVEMENT DISORD, V31, P1114, DOI 10.1002/mds.26605

Adler CH, 2016, MOVEMENT DISORD, V31, P250, DOI 10.1002/mds.26476

Adler CH, 2014, NEUROLOGY, V82, P858, DOI 10.1212/WNL.0000000000000204

Adler CJ, 2013, NAT GENET, V45, P450, DOI 10.1038/ng.2536

Aho VTE, 2019, EBIOMEDICINE, V44, P691, DOI 10.1016/j.ebiom.2019.05.064

Anderson MJ, 2003, ECOLOGY, V84, P511, DOI 10.1890/0012-9658(2003)084[0511:CAOPCA]2.0.CO;2

Arend WP, 2000, ARTHRITIS RES, V2, P245, DOI 10.1186/ar94

Arimatsu K, 2014, SCI REP-UK, V4, DOI 10.1038/srep04828

Astafurov K, 2014, PLOS ONE, V9, DOI 10.1371/journal.pone.0104416

Lourenço TGB, 2018, J ORAL MICROBIOL, V10, DOI 10.1080/20002297.2018.1487741

Beach TG, 2010, ACTA NEUROPATHOL, V119, P689, DOI 10.1007/s00401-010-0664-3

Becker T, 2007, J DENT EDUC, V71, P677

Bedarf JR, 2017, GENOME MED, V9, DOI 10.1186/s13073-017-0428-y

Belstrom D, 2014, J ORAL MICROBIOL, V6, DOI 10.3402/jom.v6.23609

BRAY J. ROGER, 1957, ECOL MONOGR, V27, P325, DOI 10.2307/1942268

Breen DP, 2019, MOVEMENT DISORD, V34, P307, DOI 10.1002/mds.27556

Cersósimo MG, 2009, AUTON NEUROSCI-BASIC, V150, P150, DOI 10.1016/j.autneu.2009.04.004

Chaudhuri KR, 2006, MOVEMENT DISORD, V21, P916, DOI 10.1002/mds.20844

Chen C, 1996, ORAL MICROBIOL IMMUN, V11, P425, DOI 10.1111/j.1399-302X.1996.tb00206.x

Chen SG, 2016, SCI REP-UK, V6, DOI 10.1038/srep34477

Daniele SG, 2015, SCI SIGNAL, V8, DOI 10.1126/scisignal.2005965

De Filippis F, 2014, PLOS ONE, V9, DOI 10.1371/journal.pone.0112373

Devos D, 2013, NEUROBIOL DIS, V50, P42, DOI 10.1016/j.nbd.2012.09.007

Dunning CJR, 2012, PROG NEUROBIOL, V97, P205, DOI 10.1016/j.pneurobio.2011.11.003

Edgar RC., 2016, BIORXIV

Edgar RC, 2013, NAT METHODS, V10, P996, DOI [10.1038/NMETH.2604, 10.1038/nmeth.2604]

Elfil M, 2020, MOVEMENT DISORD, V35, P921, DOI 10.1002/mds.28004

Everard A, 2014, ISME J, V8, P2116, DOI 10.1038/ismej.2014.45

Fasano A, 2015, LANCET NEUROL, V14, P625, DOI 10.1016/S1474-4422(15)00007-1

Forsyth CB, 2011, PLOS ONE, V6, DOI 10.1371/journal.pone.0028032

Friedland RP, 2017, PLOS PATHOG, V13, DOI 10.1371/journal.ppat.1006654

Gill SR, 2006, SCIENCE, V312, P1355, DOI 10.1126/science.1124234

Goetz CG, 2004, MOVEMENT DISORD, V19, P1020, DOI 10.1002/mds.20213

Goetz CG, 2008, MOVEMENT DISORD, V23, P2129, DOI 10.1002/mds.22340

Griffen AL, 2012, ISME J, V6, P1176, DOI 10.1038/ismej.2011.191

Hanaoka A, 2009, J CLIN NEUROSCI, V16, P1279, DOI 10.1016/j.jocn.2008.12.027

Hasegawa S, 2015, PLOS ONE, V10, DOI 10.1371/journal.pone.0142164

Hawkes CH, 2007, NEUROPATH APPL NEURO, V33, P599, DOI 10.1111/j.1365-2990.2007.00874.x

Hill-Burns EM, 2017, MOVEMENT DISORD, V32, P739, DOI 10.1002/mds.26942

Hilton D, 2014, ACTA NEUROPATHOL, V127, P235, DOI 10.1007/s00401-013-1214-6

Holmqvist S, 2014, ACTA NEUROPATHOL, V128, P805, DOI 10.1007/s00401-014-1343-6

Hopfner F, 2017, BRAIN RES, V1667, P41, DOI 10.1016/j.brainres.2017.04.019

HUGHES AJ, 1992, J NEUROL NEUROSUR PS, V55, P181, DOI 10.1136/jnnp.55.3.181

Kato I, 2017, J AM COLL NUTR, V36, P88, DOI 10.1080/07315724.2016.1185386

Kennedy M A, 1994, Spec Care Dentist, V14, P164, DOI 10.1111/j.1754-4505.1994.tb01125.x

Keshavarzian A, 2015, MOVEMENT DISORD, V30, P1351, DOI 10.1002/mds.26307

Kim R, 2018, J CLIN NEUROSCI, V58, P30, DOI 10.1016/j.jocn.2018.10.079

Kumar PS, 2013, ANAEROBE, V24, P90, DOI 10.1016/j.anaerobe.2013.09.010

Laine M, 1999, ARCH ORAL BIOL, V44, P93, DOI 10.1016/S0003-9969(98)00097-1

Lamont RJ, 2018, NAT REV MICROBIOL, V16, P745, DOI 10.1038/s41579-018-0089-x

Lewis SJ, 1997, SCAND J GASTROENTERO, V32, P920, DOI 10.3109/00365529709011203

Lin CH, 2019, J NEUROINFLAMM, V16, DOI 10.1186/s12974-019-1528-y

Liu JM, 2011, MICROBIOL-SGM, V157, P2433, DOI 10.1099/mic.0.048314-0

Lloret SP, 2007, MOVEMENT DISORD, V22, P107, DOI 10.1002/mds.21152

LOESCHE WJ, 1986, MICROBIOL REV, V50, P353, DOI 10.1128/MMBR.50.4.353-380.1986

Love MI, 2014, GENOME BIOL, V15, DOI 10.1186/s13059-014-0550-8

Manne S, 2020, MOVEMENT DISORD, V35, P268, DOI 10.1002/mds.27907

Manor Y, 2007, MOVEMENT DISORD, V22, P1917, DOI 10.1002/mds.21625

Mertsalmi TH, 2020, MOVEMENT DISORD, V35, P431, DOI 10.1002/mds.27924

Mihaila D, 2019, PLOS ONE, V14, DOI 10.1371/journal.pone.0218252

Minato T, 2017, PLOS ONE, V12, DOI 10.1371/journal.pone.0187307

MORHART RE, 1976, DENT CLIN N AM, V20, P473

Mu LC, 2015, DYSPHAGIA, V30, P404, DOI 10.1007/s00455-015-9612-7

Müller T, 2011, SPEC CARE DENT, V31, P178, DOI 10.1111/j.1754-4505.2011.00205.x

Murtaza N., 2019, NUTRIENTS, V11

Nagatsu T, 2000, J NEURAL TRANSM-SUPP, P143

Nasreddine ZS, 2005, J AM GERIATR SOC, V53, P695, DOI 10.1111/j.1532-5415.2005.53221.x

Newbrun E, 1996, J PERIODONTOL, V67, P555, DOI 10.1902/jop.1996.67.6.555

Noyce AJ, 2012, MOVEMENT DISORD, V27, P457, DOI 10.1002/mds.24006

Oli MW, 2012, MICROBIOL-SGM, V158, P2903, DOI 10.1099/mic.0.060855-0

Olsen I, 2019, J ORAL MICROBIOL, V11, DOI 10.1080/20002297.2019.1586422

Pan-Montojo F, 2012, SCI REP-UK, V2, DOI 10.1038/srep00898

Peelaerts W, 2015, NATURE, V522, P340, DOI 10.1038/nature14547

Pereira PAB, 2017, PARKINSONISM RELAT D, V38, P61, DOI 10.1016/j.parkreldis.2017.02.026

Perez-Pardo P, 2018, BENEF MICROBES, V9, P799, DOI [10.3920/bm2017.0202, 10.3920/BM2017.0202]

Petrov VA, 2017, B EXP BIOL MED+, V162, P734, DOI 10.1007/s10517-017-3700-7

Poewe W, 2017, NAT REV DIS PRIMERS, V3, DOI 10.1038/nrdp.2017.13

Godoy MCP, 2008, BRAIN, V131, P1880, DOI 10.1093/brain/awn101

Qin N, 2014, NATURE, V513, P59, DOI 10.1038/nature13568

Qin XY, 2016, JAMA NEUROL, V73, P1316, DOI 10.1001/jamaneurol.2016.2742

Sampson TR, 2016, CELL, V167, P1469, DOI 10.1016/j.cell.2016.11.018

Sawada M, 2006, J NEURAL TRANSM-SUPP, P373

Scheperjans F, 2015, MOVEMENT DISORD, V30, P350, DOI 10.1002/mds.26069

Schloss PD, 2009, APPL ENVIRON MICROB, V75, P7537, DOI 10.1128/AEM.01541-09

Schonhoff AM, 2020, PROG BRAIN RES, V252, P169, DOI 10.1016/bs.pbr.2019.10.006

Schulze-Schweifing K, 2014, FRONT CELL INFECT MI, V4, DOI 10.3389/fcimb.2014.00164

Schwiertz A, 2018, PARKINSONISM RELAT D, V50, P104, DOI 10.1016/j.parkreldis.2018.02.022

Seedorf H, 2014, CELL, V159, P253, DOI 10.1016/j.cell.2014.09.008

SILNESS J, 1964, Acta Odontol Scand, V22, P121, DOI 10.3109/00016356408993968

Singh SS, 2020, NEUROTOX RES, V37, P491, DOI 10.1007/s12640-019-00147-2

Spillantini MG, 1997, NATURE, V388, P839, DOI 10.1038/42166

Stokholm MG, 2016, ANN NEUROL, V79, P940, DOI 10.1002/ana.24648

Tanner ACR, 2015, J ORAL BIOSCI, V57, P18, DOI 10.1016/j.job.2014.08.001

Tükel Ç, 2010, CELL MICROBIOL, V12, P1495, DOI 10.1111/j.1462-5822.2010.01485.x

Unger MM, 2016, PARKINSONISM RELAT D, V32, P66, DOI 10.1016/j.parkreldis.2016.08.019

Ursell LK, 2012, J ALLERGY CLIN IMMUN, V129, P1204, DOI 10.1016/j.jaci.2012.03.010

van Stiphout MAE, 2018, PARKINSONS DIS-US, V2018, DOI 10.1155/2018/9315285

Vilas D, 2016, LANCET NEUROL, V15, P708, DOI 10.1016/S1474-4422(16)00080-6

Wade WG, 2013, PHARMACOL RES, V69, P137, DOI 10.1016/j.phrs.2012.11.006

Zekeridou A, 2017, CLIN EXP DENT RES, V3, P62, DOI 10.1002/cre2.61

Zekeridou A, 2019, CLIN EXP DENT RES, V5, P128, DOI 10.1002/cre2.162

Zhang X, 2015, NAT MED, V21, P895, DOI 10.1038/nm.3914

ZIGMOND AS, 1983, ACTA PSYCHIAT SCAND, V67, P361, DOI 10.1111/j.1600-0447.1983.tb09716.x

Zlotnik Y, 2015, PARKINSONS DIS-US, V2015, DOI 10.1155/2015/379482

NR 104

TC 42

Z9 45

U1 6

U2 18

PU IOS PRESS

PI AMSTERDAM

PA NIEUWE HEMWEG 6B, 1013 BG AMSTERDAM, NETHERLANDS

SN 1877-7171

EI 1877-718X

J9 J PARKINSON DIS

JI J. Parkinsons Dis.

PY 2021

VL 11

IS 2

BP 619

EP 631

DI 10.3233/JPD-202459

PG 13

WC Neurosciences

WE Science Citation Index Expanded (SCI-EXPANDED)

SC Neurosciences & Neurology

GA RN7SS

UT WOS:000640553700021

PM 33646178

OA Green Published

DA 2024-08-12

ER

PT J

AU Surendranathan, A

Rowe, JB

O'Brien, JT

AF Surendranathan, Ajenthan

Rowe, James B.

O'Brien, John T.

TI Neuroinflammation in Lewy body dementia

SO PARKINSONISM & RELATED DISORDERS

LA English

DT Article

DE Dementia; Lewy body; Neuroinflammation; Positron emission tomography;

Microglia

ID ALPHA-SYNUCLEIN; parkinson's disease; MICROGLIAL ACTIVATION;

CEREBROSPINAL-FLUID; DOPAMINERGIC NEURODEGENERATION; ALZHEIMER-DISEASE;

SUBSTANTIA-NIGRA; FREE-RADICALS; MOUSE MODEL; TNF-ALPHA

AB Neuroinflammation is increasingly recognized as a key factor in the pathogenesis of neurodegenerative conditions. However, it remains unclear whether it has a protective or damaging role. Studies of Alzheimer's disease and Parkinson's disease have provided much of the evidence for inflammatory pathology in neurodegeneration. Here we review the evidence for inflammation in dementia with Lewy bodies and Parkinson's disease dementia.

Neuroinflammation has been confirmed in vivo using PET imaging, with microglial activation seen in Parkinson's disease dementia and recently in dementia with Lewy bodies. In Parkinson's disease and Parkinson's disease dementia, microglial activation suggests a chronic inflammatory process, although there is also evidence of its association with cognitive ability and neuronal function.

Alpha-synuclein in various conformations has also been linked to activation of microglia, with a broad range of components of the innate and adaptive immune systems associated with this interaction.

Evidence of neuroinflammation in Lewy body dementia is further supported by pathological and biomarker studies. Genetic and epidemiological studies support a role for inflammation in Parkinson's disease, but have yet to provide the same for Lewy body dementia.

This review highlights the need to identify whether the nature and extent of microglial activation in Lewy body dementia can be linked to structural change, progression of domain specific cognitive symptoms and peripheral inflammation as a marker of central microglial pathology. Answers to these questions will enable the evaluation of immunotherapies as potential therapeutic options for prevention or treatment of dementia with Lewy bodies and Parkinson's disease dementia. (C) 2015 Elsevier Ltd. All rights reserved.

C1 [Surendranathan, Ajenthan; O'Brien, John T.] Univ Cambridge, Dept Psychiat, Cambridge CB2 0QQ, United Kingdom.

[Rowe, James B.] Univ Cambridge, Dept Clin Neurosci, Cambridge CB2 0QQ, United Kingdom.

C3 University of Cambridge; University of Cambridge

RP Surendranathan, A (corresponding author), Dept Psychiat, Box 189,Cambridge Biomedical Campus, Cambridge CB2 0SP, United Kingdom.

EM as2489@medschl.cam.ac.uk

RI Tick, Beata/ABA-1728-2020; Rowe, James/C-3661-2013

OI Rowe, James/0000-0001-7216-8679; O'Brien, John/0000-0002-0837-5080

FU NIHR; ARUK; Alzheimer's Society; MRC; Medical Research Council; Wellcome

Trust; McDonnell foundation; Evelyn Trust; PSP Association; Alzheimer's

Research UK; AZMedimmune; MRC [MC_U105597119] Funding Source: UKRI;

Wellcome Trust [103838/Z/14/Z] Funding Source: Wellcome Trust

FX John O'Brien has acted as a consultant for GE Healthcare, Lilly and

TauRx and received grant funding from NIHR, ARUK, the Alzheimer's

Society and MRC.; James Rowe holds research grants funded by the Medical

Research Council, The Wellcome Trust, The McDonnell foundation, the

Evelyn Trust, the PSP Association, Alzheimer's Research UK, and

AZMedimmune.

CR Aarsland D, 2005, MOVEMENT DISORD, V20, P1255, DOI 10.1002/mds.20527

Ajami B, 2011, NAT NEUROSCI, V14, P1142, DOI 10.1038/nn.2887

Bartels T, 2011, NATURE, V477, P107, DOI 10.1038/nature10324

Bas J, 2001, J NEUROIMMUNOL, V113, P146, DOI 10.1016/S0165-5728(00)00422-7

BlumDegen D, 1995, NEUROSCI LETT, V202, P17, DOI 10.1016/0304-3940(95)12192-7

Boza-Serrano A, 2014, ACTA NEUROPATHOL COM, V2, DOI 10.1186/s40478-014-0156-0

Braak H, 2003, NEUROBIOL AGING, V24, P197, DOI 10.1016/S0197-4580(02)00065-9

Brochard V, 2009, J CLIN INVEST, V119, P182, DOI 10.1172/JCI36470

Cagnin A, 2001, LANCET, V358, P461, DOI 10.1016/S0140-6736(01)05625-2

Cao SW, 2012, J NEUROINFLAMM, V9, DOI 10.1186/1742-2094-9-259

Chen H, 2008, AM J EPIDEMIOL, V167, P90, DOI 10.1093/aje/kwm260

Daher JPL, 2014, P NATL ACAD SCI USA, V111, P9289, DOI 10.1073/pnas.1403215111

Daniele SG, 2015, SCI SIGNAL, V8, DOI 10.1126/scisignal.2005965

Dobbs RJ, 1999, ACTA NEUROL SCAND, V100, P34, DOI 10.1111/j.1600-0404.1999.tb00721.x

Edison P, 2013, NEUROPSYCHOPHARMACOL, V38, P938, DOI 10.1038/npp.2012.255

Edison P, 2008, NEUROBIOL DIS, V32, P412, DOI 10.1016/j.nbd.2008.08.001

Ernst A, 2007, J NEUROIMMUNOL, V189, P169, DOI 10.1016/j.jneuroim.2007.07.009

Fan Z., 2014, ALZHEIMERS DEMENT, P1

Fauvet B, 2012, J BIOL CHEM, V287, P15345, DOI 10.1074/jbc.M111.318949

Fellner L, 2013, GLIA, V61, P349, DOI 10.1002/glia.22437

Gagne JJ, 2010, NEUROLOGY, V74, P995, DOI 10.1212/WNL.0b013e3181d5a4a3

Gao HM, 2003, FASEB J, V17, DOI 10.1096/fj.03-0109fje

Gao HM, 2008, TRENDS IMMUNOL, V29, P357, DOI 10.1016/j.it.2008.05.002

Gerhard A, 2006, NEUROBIOL DIS, V21, P404, DOI 10.1016/j.nbd.2005.08.002

Gerhard A, 2003, NEUROLOGY, V61, P686, DOI 10.1212/01.WNL.0000078192.95645.E6

Ginhoux F, 2013, FRONT CELL NEUROSCI, V7, DOI 10.3389/fncel.2013.00045

Hamza TH, 2010, NAT GENET, V42, P781, DOI 10.1038/ng.642

Harms AS, 2013, J NEUROSCI, V33, P9592, DOI 10.1523/JNEUROSCI.5610-12.2013

Hely MA, 2008, MOVEMENT DISORD, V23, P837, DOI 10.1002/mds.21956

Hu Y, 2015, NEUROLOGY, V84, P888, DOI 10.1212/WNL.0000000000001308

Iannaccone S, 2013, PARKINSONISM RELAT D, V19, P47, DOI 10.1016/j.parkreldis.2012.07.002

Imamura K, 2003, ACTA NEUROPATHOL, V106, P518, DOI 10.1007/s00401-003-0766-2

Jin JJ, 2007, J NEUROINFLAMM, V4, DOI 10.1186/1742-2094-4-2

Jones SAV, 2014, PSYCHOL MED, V44, P673, DOI 10.1017/S0033291713000494

Kannarkat GT, 2015, NPJ PARKINSON DIS, V1, DOI 10.1038/npjparkd.2015.2

Kim C, 2014, EXP MOL MED, V46, DOI 10.1038/emm.2014.6

Kim C, 2013, NAT COMMUN, V4, DOI 10.1038/ncomms2534

Kim WS, 2014, ALZHEIMERS RES THER, V6, DOI 10.1186/s13195-014-0073-2

Lashuel HA, 2013, NAT REV NEUROSCI, V14, P38, DOI 10.1038/nrn3406

Lastres-Becker I, 2012, HUM MOL GENET, V21, P3173, DOI 10.1093/hmg/dds143

Latta C.H., 2014, NEUROSCIENCE

Lee YM, 2010, ARCH PHARM RES, V33, P231, DOI 10.1007/s12272-010-0207-4

LENNOX G, 1989, J NEUROL NEUROSUR PS, V52, P1236, DOI 10.1136/jnnp.52.11.1236

Loeffler DA, 2006, J NEUROINFLAMM, V3, DOI 10.1186/1742-2094-3-29

Mackenzie IRA, 2000, NEUROLOGY, V55, P132, DOI 10.1212/WNL.55.1.132

McGeer PL, 2013, ACTA NEUROPATHOL, V126, P479, DOI 10.1007/s00401-013-1177-7

McGeer PL, 2002, NEUROSCI LETT, V326, P67, DOI 10.1016/S0304-3940(02)00300-2

MCGEER PL, 1988, NEUROLOGY, V38, P1285, DOI 10.1212/WNL.38.8.1285

Meeus B, 2012, ARCH NEUROL-CHICAGO, V69, P1113, DOI 10.1001/archneurol.2011.3678

Miyajima M, 2013, PLOS ONE, V8, DOI 10.1371/journal.pone.0074453

MOGI M, 1994, NEUROSCI LETT, V180, P147, DOI 10.1016/0304-3940(94)90508-8

MOGI M, 1994, NEUROSCI LETT, V165, P208, DOI 10.1016/0304-3940(94)90746-3

Mollenhauer B, 2009, MOVEMENT DISORD, V24, P1411, DOI 10.1002/mds.22510

Morales I, 2014, FRONT CELL NEUROSCI, V8, DOI 10.3389/fncel.2014.00112

Muller T, 1998, ACTA NEUROL SCAND, V98, P142

Nalls MA, 2011, LANCET, V377, P641, DOI 10.1016/S0140-6736(10)62345-8

Nalls MA, 2014, NAT GENET, V46, P989, DOI 10.1038/ng.3043

Nash KR, 2015, MOL THER, V23, P17, DOI 10.1038/mt.2014.175

Nayak D, 2014, ANNU REV IMMUNOL, V32, P367, DOI 10.1146/annurev-immunol-032713-120240

Nimmerjahn A, 2005, SCIENCE, V308, P1314, DOI 10.1126/science.1110647

Orr CF, 2005, BRAIN, V128, P2665, DOI 10.1093/brain/awh625

Ouchi Y, 2005, ANN NEUROL, V57, P168, DOI 10.1002/ana.20338

Parnetti L, 2013, NAT REV NEUROL, V9, P131, DOI 10.1038/nrneurol.2013.10

PERRY RH, 1989, LANCET, V1, P165

Perry VH, 2014, NAT REV NEUROL, V10, P217, DOI 10.1038/nrneurol.2014.38

Prinz M, 2014, NAT REV NEUROSCI, V15, P300, DOI 10.1038/nrn3722

Rayaprolu S, 2013, MOL NEURODEGENER, V8, DOI 10.1186/1750-1326-8-19

Rentzos M, 2007, ACTA NEUROL SCAND, V116, P374, DOI 10.1111/j.1600-0404.2007.00894.x

Samii A, 2009, DRUG AGING, V26, P769, DOI 10.2165/11316780-000000000-00000

Schade S, 2014, ALZHEIMERS RES THER, V6, DOI 10.1186/s13195-014-0072-3

Sharma N, 2015, NEUROCHEM INT, V87, P92, DOI 10.1016/j.neuint.2015.06.004

Shepherd CE, 2000, ARCH NEUROL-CHICAGO, V57, P817, DOI 10.1001/archneur.57.6.817

Shi M, 2011, ANN NEUROL, V69, P570, DOI 10.1002/ana.22311

Song I. U., 2013, INTERN MED RELATED D, V52, P1787

Spillantini MG, 1997, NATURE, V388, P839, DOI 10.1038/42166

Tanaka S, 2013, J NEUROINFLAMM, V10, DOI 10.1186/1742-2094-10-143

Tansey MG, 2010, NEUROBIOL DIS, V37, P510, DOI 10.1016/j.nbd.2009.11.004

Theodore S, 2008, J NEUROPATH EXP NEUR, V67, P1149, DOI 10.1097/NEN.0b013e31818e5e99

Togo T, 2001, J NEUROL SCI, V184, P71, DOI 10.1016/S0022-510X(00)00498-6

Wahner AD, 2007, ARCH NEUROL-CHICAGO, V64, P836, DOI 10.1001/archneur.64.6.836

Wang MX, 2015, EXP NEUROL, V271, P112, DOI 10.1016/j.expneurol.2015.05.008

Wang SJ, 2015, P NATL ACAD SCI USA, V112, pE1926, DOI 10.1073/pnas.1417883112

Wang W, 2011, P NATL ACAD SCI USA, V108, P17797, DOI 10.1073/pnas.1113260108

Wissemann WT, 2013, AM J HUM GENET, V93, P984, DOI 10.1016/j.ajhg.2013.10.009

Yu SY, 2014, BMC NEUROL, V14, DOI 10.1186/1471-2377-14-113

Zhang W, 2005, FASEB J, V19, P533, DOI 10.1096/fj.04-2751com

Zhang W, 2007, GLIA, V55, P1178, DOI 10.1002/glia.20532

NR 87

TC 64

Z9 70

U1 0

U2 24

PU ELSEVIER SCI LTD

PI OXFORD

PA THE BOULEVARD, LANGFORD LANE, KIDLINGTON, OXFORD OX5 1GB, OXON, United Kingdom

SN 1353-8020

EI 1873-5126

J9 PARKINSONISM RELAT D

JI Parkinsonism Relat. Disord.

PD DEC

PY 2015

VL 21

IS 12

BP 1398

EP 1406

DI 10.1016/j.parkreldis.2015.10.009

PG 9

WC Clinical Neurology

WE Science Citation Index Expanded (SCI-EXPANDED)

SC Neurosciences & Neurology

GA CZ0AW

UT WOS:000366768500002

PM 26493111

OA Green Accepted, Bronze

DA 2024-08-12

ER

PT J

AU Bartels, AL

Leenders, KL

AF Bartels, Anna L.

Leenders, Klaus L.

TI Neuroinflammation in the pathophysiology of Parkinson's disease::

Evidence from animal models to human in vivo studies with

[<SUP>11</SUP>C]-PKI1195 PET

SO MOVEMENT DISORDERS

LA English

DT Review

DE neuroinflammation; Parkinson's disease; microglia; PK11195PET; COX-2

inhibition; neuroprotection

ID NONSTEROIDAL ANTIINFLAMMATORY DRUGS; MICROGLIAL ACTIVATION;

alzheimer's disease; OXIDATIVE STRESS; SUBSTANTIA-NIGRA; BINDING-SITES;

NEURODEGENERATION; DEGENERATION; RISK; INFLAMMATION

AB Increasing evidence suggests that neuroinflammation is an active process in Parkinson's disease (PD) that contributes to ongoing neurodegeneration. PD brains and experimental PD models show elevated cytokine levels and upregulation of inflammatory-associated factors as cyclo-oxygenase-2 and inducible nitric oxide oxidase. Antiinflammatory treatment reduced neuronal degeneration in experimental models. In this review, we summarize the place of neuroinflammation in the pathophysiology of PD. In vivo PET studies are discussed. These methods provide a means to monitor in vivo potential clinical relevance of antiinflammatory treatment strategies in PD. (C) 2007 Movement Disorder Society.

C1 Univ Groningen, UMCG, Dept Neurol, NL-9700 RB Groningen, Netherlands.

C3 University of Groningen

RP Bartels, AL (corresponding author), Univ Groningen, UMCG, Dept Neurol, Hanzepl 1, NL-9700 RB Groningen, Netherlands.

EM a.l.bartels@neuro.umcg.nl

CR Banati RB, 2002, GLIA, V40, P206, DOI 10.1002/glia.10144

Banati RB, 1997, J NEUROCYTOL, V26, P77, DOI 10.1023/A:1018567510105

Batchelor PE, 1999, J NEUROSCI, V19, P1708

Cagnin A, 2000, NEUROLOGY, V54, pA476

Cagnin A, 2002, EUR NEUROPSYCHOPHARM, V12, P581, DOI 10.1016/S0924-977X(02)00107-4

Carrasco E, 2005, J NEUROSCI RES, V81, P121, DOI 10.1002/jnr.20541

Chen HL, 2005, ANN NEUROL, V58, P963, DOI 10.1002/ana.20682

Chen HL, 2003, ARCH NEUROL-CHICAGO, V60, P1059, DOI 10.1001/archneur.60.8.1059

Cicchetti F, 2002, EUR J NEUROSCI, V15, P991, DOI 10.1046/j.1460-9568.2002.01938.x

Gao HM, 2002, J NEUROSCI, V22, P782, DOI 10.1523/JNEUROSCI.22-03-00782.2002

Gerhard A, 2006, MOVEMENT DISORD, V21, P89, DOI 10.1002/mds.20668

Gerhard A, 2004, MOVEMENT DISORD, V19, P1221, DOI 10.1002/mds.20162

Gerhard A, 2001, NEUROLOGY, V56, pA270

Gerhard A, 2006, NEUROBIOL DIS, V21, P404, DOI 10.1016/j.nbd.2005.08.002

Gerhard A, 2003, NEUROLOGY, V61, P686, DOI 10.1212/01.WNL.0000078192.95645.E6

Hald A, 2005, EXP NEUROL, V193, P279, DOI 10.1016/j.expneurol.2005.01.013

Hunot S, 2003, ANN NEUROL, V53, pS49, DOI 10.1002/ana.10481

Hunot S, 1996, NEUROSCIENCE, V72, P355, DOI 10.1016/0306-4522(95)00578-1

Imamura K, 2003, ACTA NEUROPATHOL, V106, P518, DOI 10.1007/s00401-003-0766-2

KORRELL M, 2005, PARKINSONS DIS, P39

Kreutzberg GW, 1996, TRENDS NEUROSCI, V19, P312, DOI 10.1016/0166-2236(96)10049-7

Langston JW, 1999, ANN NEUROL, V46, P598, DOI 10.1002/1531-8249(199910)46:4<598::AID-ANA7>3.0.CO;2-F

McGeer PL, 2007, NEUROBIOL AGING, V28, P639, DOI 10.1016/j.neurobiolaging.2006.03.013

McGeer PL, 2004, PARKINSONISM RELAT D, V10, pS3, DOI 10.1016/j.parkreldis.2004.01.005

McGeer PL, 2004, NEUROBIOL AGING, V25, pS18

McGeer PL, 1998, ALZ DIS ASSOC DIS, V12, pS1

MCGEER PL, 1988, NEUROLOGY, V38, P1285, DOI 10.1212/WNL.38.8.1285

Muller T, 1998, ACTA NEUROL SCAND, V98, P142

Ouchi Y, 2005, ANN NEUROL, V57, P168, DOI 10.1002/ana.20338

Przedborski S, 2003, J NEUROCHEM, V85, P24

Sánchez-Pernaute R, 2004, J NEUROINFLAMM, V1, DOI 10.1186/1742-2094-1-6

SHAH F, 1994, NUCL MED BIOL, V21, P573, DOI 10.1016/0969-8051(94)90022-1

Sherer TB, 2003, NEUROSCI LETT, V341, P87, DOI 10.1016/S0304-3940(03)00172-1

Teismann P, 2003, P NATL ACAD SCI USA, V100, P5473, DOI 10.1073/pnas.0837397100

Teismann P, 2003, MOVEMENT DISORD, V18, P121, DOI 10.1002/mds.10332

Ton TG, 2006, MOVEMENT DISORD, V21, P964, DOI 10.1002/mds.20856

Wang TG, 2005, FASEB J, V19, P1134, DOI 10.1096/fj.04-2457fje

Wu DC, 2003, P NATL ACAD SCI USA, V100, P6145, DOI 10.1073/pnas.0937239100

Zhang W, 2005, FASEB J, V19, P533, DOI 10.1096/fj.04-2751com

Zhou Y, 2005, MOL CELL PROTEOMICS, V4, P1471, DOI 10.1074/mcp.M500114-MCP200

NR 40

TC 96

Z9 104

U1 0

U2 8

PU WILEY-LISS

PI HOBOKEN

PA DIV JOHN WILEY & SONS INC, 111 RIVER ST, HOBOKEN, NJ 07030 USA

SN 0885-3185

J9 MOVEMENT DISORD

JI Mov. Disord.

PD OCT 15

PY 2007

VL 22

IS 13

BP 1852

EP 1856

DI 10.1002/mds.21552

PG 5

WC Clinical Neurology

WE Science Citation Index Expanded (SCI-EXPANDED)

SC Neurosciences & Neurology

GA 228NR

UT WOS:000250737100002

PM 17592621

DA 2024-08-12

ER

PT J

AU Li, LX

Luo, P

Wu, SC

Wang, YW

AF Li, Linxi

Luo, Ping

Wu, Shaochang

Wang, Yanwen

TI Deciphering the neuroprotective effect of ascorbic acid mediated

synthesis of iron oxide nanoparticles against Parkinson's disease: an in

vitro and in vivo approach

SO MACROMOLECULAR RESEARCH

LA English

DT Article

DE Ascorbic acid; Iron oxide nanoparticles; Neuroprotection; Parkinson's

disease

ID INDUCTION; EXPOSURE; PATHWAY; MODEL

AB One of the most prevalent neurological movement diseases affecting the geriatric population globally is Parkinson's disease (PD). Recent studies have highlighted the potency of biomolecules in the generation of nanomaterials and also over their impact on neuroprotection. The objective of this research was to investigate the potential of iron oxide nanoparticles produced using ascorbic acid (AA-IONPs) against PD. Numerous analytical methods including UV-Vis analysis, Fourier-Transform Infrared Spectroscopy (FTIR), dynamic light scattering (DLS), and electron microscopy (SEM, TEM), were used to analyze the produced AA-IONPs. Nitric oxide, prostaglandin E2, and inflammatory cytokines analyses such as IL-6 and IL-1 were employed to assess the neuroprotective effect of synthesized AA-IONPs on inflammatory agent lipopolysaccharides driven murine microglial BV2 cells. And also Parkinson-induced C57BL/6 mice were given the nanoparticle treatment to confirm the in vivo effects of the produced nanoparticles. Our characterization findings had demonstrated that AA-IONPs have a significant role in acting as an ideal nano drug and may have the ability to reduce inflammation in in vitro murine microglial BV2. The outcomes of in vivo tests conclusively show that AA-IONPs had reduced neuroinflammation and enhanced motor coordination in Parkinson's disease-induced rats.

C1 [Li, Linxi] Nanchang Univ, Sch Basic Med Sci, Nanchang 330006, Jiangxi, China.

[Luo, Ping] First Peoples Hosp Jiangxia Dist, Dept Pharm, Wuhan 430200, Hubei, China.

[Wu, Shaochang] Second Peoples Hosp LiShui, Dept Geriatr, Lishui 323000, Zhejiang, China.

[Wang, Yanwen] Wuhan Brain Hosp, Gen Hosp Yangtze River Shipping, Dept Neurol, Wuhan 430015, Hubei, China.

C3 Nanchang University

RP Wang, YW (corresponding author), Wuhan Brain Hosp, Gen Hosp Yangtze River Shipping, Dept Neurol, Wuhan 430015, Hubei, China.

EM wangyanwen140718@sina.com

CR Abbas G, 2021, MATER TODAY COMMUN, V26, DOI 10.1016/j.mtcomm.2021.102175

Ang A, 2018, BIOCHEM SOC T, V46, P1147, DOI 10.1042/BST20180169

Asefi Y, 2022, BIOINTERFACE RES APP, V12, P3215, DOI 10.33263/BRIAC123.32153224

Ballaz S, 2013, J NEUROSCI RES, V91, P1609, DOI 10.1002/jnr.23276

Balu P, 2020, J MATER SCI-MATER EL, V31, P10669, DOI 10.1007/s10854-020-03616-z

Böttger F, 2021, J EXP CLIN CANC RES, V40, DOI 10.1186/s13046-021-02134-y

Candelario-Jalil E, 2006, J NEUROIMMUNOL, V174, P39, DOI 10.1016/j.jneuroim.2006.01.003

Choi HW, 2018, INT J MOL MED, V41, P1103, DOI 10.3892/ijmm.2017.3309

Chong TL, 2019, FRONT CELL DEV BIOL, V7, DOI 10.3389/fcell.2019.00128

De Nuccio F, 2021, BIOLOGY-BASEL, V10, DOI 10.3390/biology10111155

Elamawi RM, 2018, EGYPT J BIOL PEST CO, V28, DOI 10.1186/s41938-018-0028-1

Fletcher SC, 2020, BIOCHEM SOC T, V48, P1843, DOI 10.1042/BST20190333

Haghighat M, 2022, BIOPROC BIOSYST ENG, V45, P97, DOI 10.1007/s00449-021-02643-2

Huang YN, 2014, PLOS ONE, V9, DOI 10.1371/journal.pone.0097276

Izadiyan Z, 2020, ARAB J CHEM, V13, P2011, DOI 10.1016/j.arabjc.2018.02.019

Jack CR, 2011, ALZHEIMERS DEMENT, V7, P257, DOI 10.1016/j.jalz.2011.03.004

Janhom P, 2015, J TOXICOL-US, V2015, DOI 10.1155/2015/919058

Kavithaa K, 2016, RSC ADV, V6, P64531, DOI 10.1039/c6ra11658b

Leybo D, 2020, NANOMATERIALS-BASEL, V10, DOI 10.3390/nano10010037

Li BD, 2017, CNS NEUROSCI THER, V23, P827, DOI 10.1111/cns.12727

Li Q, 2022, BMC COMPLEMENT MED, V22, DOI 10.1186/s12906-022-03780-1

Lotia M, 2016, EXPERT OPIN PHARMACO, V17, P895, DOI 10.1517/14656566.2016.1149163

Ngo B, 2019, NAT REV CANCER, V19, P271, DOI 10.1038/s41568-019-0135-7

Niraimathee VA, 2016, INT J ENVIRON SUSTAI, V15, P227, DOI 10.1504/IJESD.2016.077370

Oueslati A, 2016, J PARKINSON DIS, V6, P39, DOI 10.3233/JPD-160779

Park SY, 2019, INT J NANOMED, V14, P2861, DOI 10.2147/IJN.S195218

Ponraj T, 2017, INT J BIOL MACROMOL, V95, P1235, DOI 10.1016/j.ijbiomac.2016.11.018

Sandhya J, 2020, MATER RES EXPRESS, V7, DOI 10.1088/2053-1591/ab6642

Sood A, 2016, J EXP NANOSCI, V11, P370, DOI 10.1080/17458080.2015.1066514

Tartaj P, 2003, J PHYS D APPL PHYS, V36, pR182, DOI 10.1088/0022-3727/36/13/202

Umarao P, 2016, J NANOSCI NANOTECHNO, V16, P261, DOI 10.1166/jnn.2016.11103

Umer A, 2014, MATERIA-BRAZIL, V19, P197, DOI 10.1590/S1517-70762014000300002

Vidal-Martinez G, 2019, NEUROSCIENCE, V411, P1, DOI 10.1016/j.neuroscience.2019.05.029

Xue JW, 2019, J PHOTOCH PHOTOBIO B, V200, DOI 10.1016/j.jphotobiol.2019.111635

Yallapu MM, 2011, BIOMATERIALS, V32, P1890, DOI 10.1016/j.biomaterials.2010.11.028

Zhang N, 2021, SCI REP-UK, V11, DOI 10.1038/s41598-021-93861-x

NR 36

TC 1

Z9 1

U1 2

U2 11

PU POLYMER SOC KOREA

PI SEOUL

PA ROOM 601, HATCHON BUILDING, 831 YEOKSAM-DONG, KANGNAM-KU, SEOUL 135-792,

SOUTH KOREA

SN 1598-5032

EI 2092-7673

J9 MACROMOL RES

JI Macromol. Res.

PD OCT

PY 2023

VL 31

IS 10

BP 949

EP 960

DI 10.1007/s13233-023-00186-x

EA AUG 2023

PG 12

WC Polymer Science

WE Science Citation Index Expanded (SCI-EXPANDED)

SC Polymer Science

GA U2MX8

UT WOS:001052815700002

DA 2024-08-12

ER

PT J

AU Wang, J

Ding, X

Wu, XY

Liu, J

Zhou, R

Wei, PX

Zhang, QP

Zhang, CY

Zen, K

Li, L

AF Wang, Jin

Ding, Xin

Wu, Xiangyu

Liu, Jing

Zhou, Rui

Wei, Pingxuan

Zhang, Qipeng

Zhang, Chenyu

Zen, Ke

Li, Liang

TI SIRPα deficiency accelerates the pathologic process in models of

parkinson's disease

SO GLIA

LA English

DT Article

DE aging; microglia; neuroinflammation; Parkinson's disease; SIRP alpha

ID MICROGLIA; ACTIVATION; CD47; INFLAMMATION; ASTROCYTES; MODULATION;

EXPRESSION; RESPONSES; SYNAPSES; SURVIVAL

AB Microglia-mediated neuroinflammation is a crucial pathophysiological contributor to several aging-related neurodegenerative disorders, including Parkinson's disease (PD). During the process of aging or stress, microglia undergoes several transcriptional and morphological changes that contribute to aberrant immunological responses, which is known as priming. Key molecules involved in the process, however, are not clearly defined. In the present study, we have demonstrated that level of microglial signal regulatory protein alpha (SIRP alpha) decreased during aging or inflammatory challenge. Functional studies suggested that downregulation of SIRP alpha released the brake of inflammatory response in microglia, revealing an inhibitory effect of SIRP alpha in microglial activation. Furthermore, we assessed the impact of SIRP alpha downregulation in PD pathogenesis using both cell culture and animal models. Our results showed that SIRP alpha deficiency resulted in abnormal inflammatory response and phagocytic activity of microglia, which in turn, further accelerated degeneration of dopaminergic neurons in 1-Methyl-4-phenyl-1,2,3,6-tetrahydropyridine or lipopolysaccharides mice models. These results collectively demonstrate that dysregulation of SIRP alpha signaling in microglia during aging plays a critical role in the pathogenesis of age-related neurological disorders such as PD.

C1 [Wang, Jin; Ding, Xin; Wu, Xiangyu; Liu, Jing; Zhou, Rui; Wei, Pingxuan; Zhang, Qipeng; Zhang, Chenyu; Zen, Ke; Li, Liang] Nanjing Univ, Sch Life Sci, NAILS, State Key Lab Pharmaceut Biotechnol, 163 Xianlin Ave, Nanjing 210023, Jiangsu, China.

[Zhang, Qipeng; Zen, Ke; Li, Liang] Nanjing Univ, Inst Brain Sci, Nanjing, Jiangsu, China.

C3 Nanjing University; Nanjing University

RP Zen, K; Li, L (corresponding author), Nanjing Univ, Sch Life Sci, NAILS, State Key Lab Pharmaceut Biotechnol, 163 Xianlin Ave, Nanjing 210023, Jiangsu, China.

EM kzen@nju.edu.cn; liangli@nju.edu.cn

OI li, liang/0000-0003-0978-2881

FU National Natural Science Foundation of China [31471019, 31471053]

FX National Natural Science Foundation of China, Grant/Award Numbers:

31471019, 31471053

CR Askew K, 2017, CELL REP, V18, P391, DOI 10.1016/j.celrep.2016.12.041

Barcia C, 2012, SCI REP-UK, V2, DOI 10.1038/srep00809

Barclay AN, 2014, ANNU REV IMMUNOL, V32, P25, DOI 10.1146/annurev-immunol-032713-120142

Bialas AR, 2013, NAT NEUROSCI, V16, P1773, DOI 10.1038/nn.3560

Bian Z, 2016, P NATL ACAD SCI USA, V113, pE5434, DOI 10.1073/pnas.1521069113

Blaylock Russell L, 2017, Surg Neurol Int, V8, P65, DOI 10.4103/sni.sni_441_16

Bohlen CJ, 2017, NEURON, V94, P759, DOI 10.1016/j.neuron.2017.04.043

Chatterjee P, 2017, BMC GENOMICS, V18, DOI 10.1186/s12864-017-4098-3

Cornejo F, 2016, ADV EXP MED BIOL, V949, P205, DOI 10.1007/978-3-319-40764-7_10

Dihan Z., 2013, J ALLERGY CLIN IMMUN, V132

Fan XL, 2009, NEUROIMMUNOMODULAT, V16, P162, DOI 10.1159/000204229

Fonken LK, 2016, J NEUROSCI, V36, P7946, DOI 10.1523/JNEUROSCI.1161-16.2016

Gaven F, 2014, JOVE-J VIS EXP, DOI 10.3791/51751

Gitik M, 2014, FRONT CELL NEUROSCI, V8, DOI 10.3389/fncel.2014.00104

Gitik M, 2011, J NEUROINFLAMM, V8, DOI 10.1186/1742-2094-8-24

GUPTA M, 1986, NEUROSCI LETT, V70, P326, DOI 10.1016/0304-3940(86)90573-2

Han MH, 2012, J EXP MED, V209, P1325, DOI 10.1084/jem.20101974

Hong S, 2016, SCIENCE, V352, P712, DOI 10.1126/science.aad8373

Huang DP, 2018, NEUROSCIENCE, V369, P303, DOI 10.1016/j.neuroscience.2017.11.041

Joshi N, 2018, J NEUROSCI RES, V96, P379, DOI 10.1002/jnr.24185

Jyothi HJ, 2015, NEUROBIOL AGING, V36, P3321, DOI 10.1016/j.neurobiolaging.2015.08.024

Koh E, 2017, BIOMATERIALS, V121, P121, DOI 10.1016/j.biomaterials.2017.01.004

Kohutnicka M, 1998, IMMUNOPHARMACOLOGY, V39, P167, DOI 10.1016/S0162-3109(98)00022-8

Labzin LI, 2018, ANNU REV MED, V69, P437, DOI 10.1146/annurev-med-050715-104343

Lehrman EK, 2018, NEURON, V100, P120, DOI 10.1016/j.neuron.2018.09.017

Liu J, 2015, SCI REP-UK, V5, DOI 10.1038/srep15720

Liu W, 2011, LIFE SCI, V89, P141, DOI 10.1016/j.lfs.2011.05.011

Liu Y, 2002, J BIOL CHEM, V277, P10028, DOI 10.1074/jbc.M109720200

Londino JD, 2015, J BIOL CHEM, V290, P31113, DOI 10.1074/jbc.M115.682914

Madeira JM, 2014, J NEUROIMMUNOL, V276, P71, DOI 10.1016/j.jneuroim.2014.08.615

Matt SM, 2016, NEUROBIOL AGING, V47, P1, DOI 10.1016/j.neurobiolaging.2016.07.006

Moehle MS, 2015, NEUROSCIENCE, V302, P59, DOI 10.1016/j.neuroscience.2014.11.018

Molteni M, 2017, J NEUROIMMUNOL, V313, P109, DOI 10.1016/j.jneuroim.2017.11.002

Muñoz-Manchado AB, 2016, J NEUROCHEM, V136, P373, DOI 10.1111/jnc.13409

Myers SA, 2011, NEUROBIOL DIS, V42, P21, DOI 10.1016/j.nbd.2010.12.010

Norden DM, 2015, NEUROPHARMACOLOGY, V96, P29, DOI 10.1016/j.neuropharm.2014.10.028

Norden DM, 2014, GLIA, V62, P881, DOI 10.1002/glia.22647

Norris GT, 2018, J EXP MED, V215, P1789, DOI 10.1084/jem.20172244

Panicker N, 2015, J NEUROSCI, V35, P10058, DOI 10.1523/JNEUROSCI.0302-15.2015

Perry VH, 2014, NAT REV NEUROL, V10, P217, DOI 10.1038/nrneurol.2014.38

Phillipson OT, 2017, AGEING RES REV, V40, P149, DOI 10.1016/j.arr.2017.09.006

Polazzi E, 2002, REV NEUROSCIENCE, V13, P221

Presumey J, 2017, ADV IMMUNOL, V135, P53, DOI 10.1016/bs.ai.2017.06.004

Raj DDA, 2015, AGING CELL, V14, P1003, DOI 10.1111/acel.12370

Schafer DP, 2012, NEURON, V74, P691, DOI 10.1016/j.neuron.2012.03.026

Schipke CG, 2001, FASEB J, V15, P255, DOI 10.1096/fj.01-0514fje

Sellgren CM, 2017, MOL PSYCHIATR, V22, P170, DOI 10.1038/mp.2016.220

Sepe S, 2016, CELL REP, V15, P1866, DOI 10.1016/j.celrep.2016.04.071

Song WM, 2018, NAT IMMUNOL, V19, P1048, DOI 10.1038/s41590-018-0212-1

Soreq L, 2017, CELL REP, V18, P557, DOI 10.1016/j.celrep.2016.12.011

Sugama S, 2003, BRAIN RES, V964, P288, DOI 10.1016/S0006-8993(02)04085-4

Tang Y, 2014, CELL DEATH DIFFER, V21, P369, DOI 10.1038/cdd.2013.159

Toth AB, 2013, NAT NEUROSCI, V16, P1417, DOI 10.1038/nn.3516

von Bernhardi R, 2015, FRONT AGING NEUROSCI, V7, DOI 10.3389/fnagi.2015.00124

Walker DG, 2017, FRONT AGING NEUROSCI, V9, DOI 10.3389/fnagi.2017.00244

Wang L, 2012, J NEUROCHEM, V122, P834, DOI 10.1111/j.1471-4159.2012.07818.x

Wood H, 2017, NAT REV NEUROL, V13, DOI 10.1038/nrneurol.2017.62

Zen K, 2013, NAT COMMUN, V4, DOI 10.1038/ncomms3436

Zhang HY, 2015, BRAIN RES, V1623, P74, DOI 10.1016/j.brainres.2015.03.012

Zhang JF, 2014, NEURON, V82, P195, DOI 10.1016/j.neuron.2014.01.043

Zhang QS, 2017, ACTA PHARMACOL SIN, V38, P1317, DOI 10.1038/aps.2017.49

Zhou X, 2014, NEURAL REGEN RES, V9, P1787, DOI 10.4103/1673-5374.143423

NR 62

TC 10

Z9 10

U1 2

U2 22

PU WILEY

PI HOBOKEN

PA 111 RIVER ST, HOBOKEN 07030-5774, NJ USA

SN 0894-1491

EI 1098-1136

J9 GLIA

JI Glia

PD DEC

PY 2019

VL 67

IS 12

BP 2343

EP 2359

DI 10.1002/glia.23689

EA JUL 2019

PG 17

WC Neurosciences

WE Science Citation Index Expanded (SCI-EXPANDED)

SC Neurosciences & Neurology

GA JF4CZ

UT WOS:000477174200001

PM 31322787

DA 2024-08-12

ER

PT J

AU Zhao, YJ

Qi, YZ

Li, QR

Quan, HH

Liu, D

Zhou, H

AF Zhao, Yujia

Qi, Yuze

Li, Qingru

Quan, Huihui

Liu, Dan

Zhou, Hui

TI Connexin43 inhibition attenuated dopaminergic neuronal loss in the

lipopolysaccharide-induced mice model of Parkinson's disease

SO NEUROSCIENCE LETTERS

LA English

DT Article

DE Connexin43; Astrocytes; Parkinson 's disease; Lipopolysaccharide;

Inflammation; Microglia

ID GAP-JUNCTION CHANNELS; CX43 HEMICHANNELS; NEUROINFLAMMATION; ASTROCYTES;

ACTIVATION

AB Studies using in vitro Parkinson's disease (PD) models have found that lipopolysaccharide (LPS) induced reduction of connexin 43 (Cx43) gap junction communication and elevation of hemichannel function, which could cause neurotoxicity directly and indirectly via excessive ATP and glutamate release. However, in vivo study about Cx43 expression and function, as well as the efficacy of Cx43 inhibition for neuronal survival in PD is lacking. This study aimed to unravel the role of Cx43 in PD and understand the underlying mechanisms using an in vivo PD model. Male C57BL/6 mice received intranigral injection of LPS (5 mu g) and 43Gap27 (4 mu g), a Cx43 inhibitor, simultaneously. Results showed that following LPS treatment, total Cx43 expression decreased by about 60%, but the relative level of phosphorylated Cx43 increased to about double that controls (all p < 0.05). The administration of 43Gap27 significantly attenuated the loss of dopaminergic neurons and restored dopamine and its metabolites levels. Moreover, 43Gap27 treatment inhibited intense microgliosis and astrogliosis in nigrostriatal system induced by LPS and also ameliorated elevated levels of inflammatory mediators. Interestingly, Cx43 inhibition also increased nerve growth factors. In conclusion, Cx43 inhibition was able to prevent LPS-mediated dopaminergic neuronal death, possibly via neuroinflammation reaction reduction and neurotrophic factors elevation. Therefore, Cx43 may be a promising therapeutic target for degenerative neurological disorders such as PD.

C1 [Zhao, Yujia; Qi, Yuze; Li, Qingru; Quan, Huihui; Zhou, Hui] Peking Univ, Sch Publ Hlth, Dept Occupat & Environm Hlth Sci, Beijing, China.

[Zhao, Yujia] Univ Utrecht, Fac Vet Med, Inst Risk Assessment Sci, Utrecht, Netherlands.

[Liu, Dan] German Ctr Neurodegenerat Dis, Populat Hlth Sci, Bonn, Germany.

C3 Peking University; Utrecht University; Helmholtz Association; German

Center for neurodegenerative disease (DZNE)

RP Zhou, H (corresponding author), Peking Univ, Sch Publ Hlth, Dept Occupat & Environm Hlth Sci, Beijing, China.; Liu, D (corresponding author), German Ctr Neurodegenerat Dis, Populat Hlth Sci, Bonn, Germany.

EM hardhui@gmail.com

FU National Natural Science Foundation of China [21577004]; Beijing

Municipal Natural Science Foundation [7162104]

FX This study was supported by National Natural Science Foundation of China

[grant number 21577004] and Beijing Municipal Natural Science Foundation

[grant number 7162104] .

CR Abudara V, 2015, GLIA, V63, P795, DOI 10.1002/glia.22785

Arroyo DS, 2011, INT IMMUNOPHARMACOL, V11, P1415, DOI 10.1016/j.intimp.2011.05.006

Booth HDE, 2017, TRENDS NEUROSCI, V40, P358, DOI 10.1016/j.tins.2017.04.001

Evans WH, 2006, BIOCHEM J, V397, P1, DOI 10.1042/BJ20060175

Faustmann PM, 2003, GLIA, V42, P101, DOI 10.1002/glia.10141

Froger N, 2009, J NEUROCHEM, V111, P1383, DOI 10.1111/j.1471-4159.2009.06407.x

Gajardo-Gómez R, 2017, GLIA, V65, P122, DOI 10.1002/glia.23080

Gao HM, 2003, J NEUROSCI, V23, P1228, DOI 10.1523/JNEUROSCI.23-04-01228.2003

Giaume C, 2013, FRONT PHARMACOL, V4, DOI 10.3389/fphar.2013.00088

Hoban DB, 2013, BRAIN BEHAV IMMUN, V27, P91, DOI 10.1016/j.bbi.2012.10.001

[黄焕焕 Huang Huanhuan], 2018, [毒理学杂志, Journal of Toxicology], V32, P186

Karpuk N, 2011, J NEUROSCI, V31, P414, DOI 10.1523/JNEUROSCI.5247-10.2011

Laird DW, 2006, BIOCHEM J, V394, P527, DOI 10.1042/BJ20051922

Lewerenz J, 2015, FRONT NEUROSCI-SWITZ, V9, DOI 10.3389/fnins.2015.00469

Liao CK, 2013, PLOS ONE, V8, DOI 10.1371/journal.pone.0079350

Liao CK, 2010, INT J BIOCHEM CELL B, V42, P762, DOI 10.1016/j.biocel.2010.01.016

Liu D, 2020, J NEUROINFLAMM, V17, DOI 10.1186/s12974-020-01925-y

Même W, 2006, FASEB J, V20, P494, DOI 10.1096/fj.05-4297fje

Orellana JA, 2013, NEUROPHARMACOLOGY, V75, P567, DOI 10.1016/j.neuropharm.2013.02.020

Orellana JA, 2011, J NEUROSCI, V31, P4962, DOI 10.1523/JNEUROSCI.6417-10.2011

Orellana JA, 2011, J NEUROCHEM, V118, P826, DOI 10.1111/j.1471-4159.2011.07210.x

Palatinus JA, 2016, J DIABETES RES, V2016, DOI 10.1155/2016/8789617

Qin LY, 2007, GLIA, V55, P453, DOI 10.1002/glia.20467

Retamal MA, 2007, J NEUROSCI, V27, P13781, DOI 10.1523/JNEUROSCI.2042-07.2007

Rodrigues RJ, 2015, FRONT NEUROSCI-SWITZ, V9, DOI 10.3389/fnins.2015.00148

Theis M, 2005, TRENDS NEUROSCI, V28, P188, DOI 10.1016/j.tins.2005.02.006

Volonté C, 2003, CNS NEUROL DISORD-DR, V2, P403, DOI 10.2174/1568007033482643

NR 27

TC 9

Z9 9

U1 0

U2 11

PU ELSEVIER IRELAND LTD

PI CLARE

PA ELSEVIER HOUSE, BROOKVALE PLAZA, EAST PARK SHANNON, CO, CLARE, 00000,

IRELAND

SN 0304-3940

EI 1872-7972

J9 NEUROSCI LETT

JI Neurosci. Lett.

PD FEB 6

PY 2022

VL 771

AR 136471

DI 10.1016/j.neulet.2022.136471

EA JAN 2022

PG 8

WC Neurosciences

WE Science Citation Index Expanded (SCI-EXPANDED)

SC Neurosciences & Neurology

GA YP4KH

UT WOS:000748592100005

PM 35065246

DA 2024-08-12

ER

PT J

AU Kang, S

Noh, Y

Oh, SJ

Yoon, HJ

Im, S

Kwon, HT

Pak, YK

AF Kang, Sora

Noh, Youngjin

Oh, Seung Jun

Yoon, Hye Ji

Im, Suyeol

Kwon, Hung Taeck

Pak, Youngmi Kim

TI Neuroprotective Effects of Aldehyde-Reducing Composition in an

LPS-Induced Neuroinflammation Model of Parkinson's Disease

SO MOLECULES

LA English

DT Article

DE ALDH; oxidative stress; neuroinflammation; Parkinson's disease

ID OXIDATIVE STRESS; ANIMAL-MODELS; DEHYDROGENASE; INHIBITION; MICROGLIA;

ACTIVATION; MECHANISMS; DEATH

AB Parkinson's disease (PD) is a complex neurodegenerative disease in which neuroinflammation and oxidative stress interact to contribute to pathogenesis. This study investigates the in vivo neuroprotective effects of a patented yeast extract lysate in a lipopolysaccharide (LPS)-induced neuroinflammation model. The yeast extract lysate, named aldehyde-reducing composition (ARC), exhibited potent antioxidant and anti-aldehyde activities in vitro. Oral administration of ARC at 10 or 20 units/kg/day for 3 days prior to intraperitoneal injection of LPS (10 mg/kg) effectively preserved dopaminergic neurons in the substantia nigra (SN) and striatum by preventing LPS-induced cell death. ARC also normalized the activation of microglia and astrocytes in the SN, providing further evidence for its neuroprotective properties. In the liver, ARC downregulated the LPS-induced increase in inflammatory cytokines and reversed the LPS-induced decrease in antioxidant-related genes. These findings indicate that ARC exerts potent antioxidant, anti-aldehyde, and anti-inflammatory effects in vivo, suggesting its potential as a disease-modifying agent for the prevention and treatment of neuroinflammation-related diseases, including Parkinson's disease.

C1 [Kang, Sora; Yoon, Hye Ji; Pak, Youngmi Kim] Kyung Hee Univ, Grad Sch, Dept Neurosci, Seoul 02447, South Korea.

[Kang, Sora; Noh, Youngjin; Kwon, Hung Taeck] Picoentech Co Ltd, Seongnam Si 13201, Gyeong Gi Do, South Korea.

[Oh, Seung Jun; Im, Suyeol; Pak, Youngmi Kim] Kyung Hee Univ, Grad Sch, Dept Biomed Sci, Seoul 02447, South Korea.

[Pak, Youngmi Kim] Kyung Hee Univ, Coll Med, Dept Physiol, Seoul 02447, South Korea.

C3 Kyung Hee University; Kyung Hee University; Kyung Hee University

RP Pak, YK (corresponding author), Kyung Hee Univ, Grad Sch, Dept Neurosci, Seoul 02447, South Korea.; Pak, YK (corresponding author), Kyung Hee Univ, Grad Sch, Dept Biomed Sci, Seoul 02447, South Korea.; Pak, YK (corresponding author), Kyung Hee Univ, Coll Med, Dept Physiol, Seoul 02447, South Korea.

EM ksr8947@khu.ac.kr; yjnoh0614@picoentech.com; ohsungjun124@khu.ac.kr;

yhj2271@khu.ac.kr; suryeol@khu.ac.kr; thomas@picoentech.com;

ykpak@khu.ac.kr

OI Pak, Youngmi/0000-0001-7424-3484

FU National Research Foundation of Korea (NRF)

FX No Statement Available

CR Alam F, 2021, CURR RES PHYSIOL, V4, P119, DOI 10.1016/j.crphys.2021.03.002

Ayala A, 2014, OXID MED CELL LONGEV, V2014, DOI 10.1155/2014/360438

Bachiller S, 2018, FRONT CELL NEUROSCI, V12, DOI 10.3389/fncel.2018.00488

Barrera G, 2018, ANTIOXIDANTS-BASEL, V7, DOI 10.3390/antiox7080102

Braak H, 2003, NEUROBIOL AGING, V24, P197, DOI 10.1016/S0197-4580(02)00065-9

Burke WJ, 2003, BRAIN RES, V989, P205, DOI 10.1016/S0006-8993(03)03354-7

Cagle Brianna S., 2019, Current Opinion in Toxicology, V13, P16, DOI 10.1016/j.cotox.2018.12.002

Castro JP, 2017, FREE RADICAL BIO MED, V111, P309, DOI 10.1016/j.freeradbiomed.2016.10.497

Chen CH, 2014, PHYSIOL REV, V94, P1, DOI 10.1152/physrev.00017.2013

Crabb DW, 2004, P NUTR SOC, V63, P49, DOI 10.1079/PNS2003327

Dauer W, 2003, NEURON, V39, P889, DOI 10.1016/S0896-6273(03)00568-3

Fitzmaurice AG, 2013, P NATL ACAD SCI USA, V110, P636, DOI 10.1073/pnas.1220399110

Frakes AE, 2014, NEURON, V81, P1009, DOI 10.1016/j.neuron.2014.01.013

Goldstein DS, 2013, J NEUROCHEM, V126, P591, DOI 10.1111/jnc.12345

Grigoryan R, 2021, FRONT MED-LAUSANNE, V8, DOI 10.3389/fmed.2021.664666

Grünblatt E, 2016, J NEURAL TRANSM, V123, P83, DOI 10.1007/s00702-014-1320-1

Guo JM, 2013, CELL RES, V23, P915, DOI 10.1038/cr.2013.69

He JY, 2020, OXID MED CELL LONGEV, V2020, DOI 10.1155/2020/6137521

Heo JY, 2020, CURR BIOL, V30, P276, DOI 10.1016/j.cub.2019.11.079

Hirsch EC, 2003, ANN NY ACAD SCI, V991, P214

Hirsch EC, 2009, LANCET NEUROL, V8, P382, DOI 10.1016/S1474-4422(09)70062-6

HJELLE JJ, 1982, TOXICOL LETT, V14, P35, DOI 10.1016/0378-4274(82)90007-8

Huh SH, 2011, J IMMUNOL, V187, P960, DOI 10.4049/jimmunol.1100009

Im S, 2022, ANTIOXIDANTS-BASEL, V11, DOI 10.3390/antiox11112109

Imamura K, 2003, ACTA NEUROPATHOL, V106, P518, DOI 10.1007/s00401-003-0766-2

Joe EH, 2018, EXP NEUROBIOL, V27, P77, DOI 10.5607/en.2018.27.2.77

Juan CA, 2021, INT J MOL SCI, V22, DOI 10.3390/ijms22094642

Kam TI, 2020, NEUROBIOL DIS, V144, DOI 10.1016/j.nbd.2020.105028

Kang S, 2022, INT J MOL SCI, V23, DOI 10.3390/ijms232314871

Kang S, 2022, BIOMED PHARMACOTHER, V145, DOI 10.1016/j.biopha.2021.112389

Kang S, 2020, ARCH PHARM RES, V43, P553, DOI 10.1007/s12272-020-01238-x

Kedare SB, 2011, J FOOD SCI TECH MYS, V48, P412, DOI 10.1007/s13197-011-0251-1

Kwon H.T., 2023, U.S. Patent, Patent No. [6, 11,618,889 B2, 611618889]

Kwon HS, 2020, TRANSL NEURODEGENER, V9, DOI 10.1186/s40035-020-00221-2

Liu YM, 2020, FRONT AGING NEUROSCI, V11, DOI 10.3389/fnagi.2019.00372

Masato A, 2023, NPJ PARKINSONS DIS, V9, DOI 10.1038/s41531-023-00485-1

Masato A, 2019, MOL NEURODEGENER, V14, DOI 10.1186/s13024-019-0332-6

Moore DJ, 2005, ANNU REV NEUROSCI, V28, P57, DOI 10.1146/annurev.neuro.28.061604.135718

Moretti M, 2017, CNS NEUROSCI THER, V23, P921, DOI 10.1111/cns.12767

Pan G, 2016, J DIABETES RES, V2016, DOI 10.1155/2016/1390861

Przedborski S, 2005, PARKINSONISM RELAT D, V11, pS3, DOI 10.1016/j.parkreldis.2004.10.012

Redza-Dutordoir M, 2016, BBA-MOL CELL RES, V1863, P2977, DOI 10.1016/j.bbamcr.2016.09.012

Salim S, 2017, J PHARMACOL EXP THER, V360, P201, DOI 10.1124/jpet.116.237503

Shen YH, 2020, BIOMED PHARMACOTHER, V132, DOI 10.1016/j.biopha.2020.110928

Simpson DSA, 2020, ANTIOXIDANTS-BASEL, V9, DOI 10.3390/antiox9080743

Sin H.J., 2023, J. Environ. Health Sci, V49, P99, DOI [10.5668/JEHS.2023.49.2.99, DOI 10.5668/JEHS.2023.49.2.99]

Singh CK, 2018, ANTIOXID REDOX SIGN, V28, P643, DOI 10.1089/ars.2017.7290

Sukharev S, 2019, ENVIRON CHEM LETT, V17, P1405, DOI 10.1007/s10311-019-00875-z

Tansey MG, 2010, NEUROBIOL DIS, V37, P510, DOI 10.1016/j.nbd.2009.11.004

Turner MD, 2014, BBA-MOL CELL RES, V1843, P2563, DOI 10.1016/j.bbamcr.2014.05.014

Uversky VN, 2004, CELL TISSUE RES, V318, P225, DOI 10.1007/s00441-004-0937-z

Wang TG, 2004, J NEUROCHEM, V88, P939, DOI 10.1046/j.1471-4159.2003.02242.x

Wang YM, 2021, FRONT PHARMACOL, V12, DOI 10.3389/fphar.2021.757161

NR 53

TC 0

Z9 0

U1 5

U2 7

PU MDPI

PI BASEL

PA ST ALBAN-ANLAGE 66, CH-4052 BASEL, SWITZERLAND

EI 1420-3049

J9 MOLECULES

JI Molecules

PD DEC

PY 2023

VL 28

IS 24

AR 7988

DI 10.3390/molecules28247988

PG 15

WC Biochemistry & Molecular Biology; Chemistry, Multidisciplinary

WE Science Citation Index Expanded (SCI-EXPANDED)

SC Biochemistry & Molecular Biology; Chemistry

GA DF2X6

UT WOS:001130560900001

PM 38138478

OA Green Published, gold

DA 2024-08-12

ER

PT J

AU Zhu, ZM

Huang, PL

Sun, RF

Li, XL

Li, WS

Gong, WJ

AF Zhu, Ziman

Huang, Peiling

Sun, Ruifeng

Li, Xiaoling

Li, Wenshan

Gong, Weijun

TI A Novel Long-Noncoding RNA LncZFAS1 Prevents MPP<SUP>+</SUP>-Induced

Neuroinflammation Through MIB1 Activation

SO MOLECULAR NEUROBIOLOGY

LA English

DT Article

DE LncZFAS; Parkinson's disease; Pyroptosis; MIB1; TXNIP; NLRP3

ID parkinson's disease; MICROGLIAL ACTIVATION; INFLAMMASOMES MECHANISM;

NLRP3 INFLAMMASOME; SUBSTANTIA-NIGRA; NEURODEGENERATION; MITOCHONDRIAL;

CELLS; EXPRESSION; MICRORNAS

AB Parkinson's disease remains one of the leading neurodegenerative disease in developed countries. Despite well-defined symptomology and pathology, the complexity of Parkinson's disease prevents a full understanding of its etiological mechanism. Mechanistically, alpha-synuclein misfolding and aggregation appear to be central for disease progression, but mitochondrial dysfunction, dysfunctional protein clearance and ubiquitin/proteasome systems, and neuroinflammation have also been associated with Parkinson's disease. Particularly, neuroinflammation, which was initially thought to be a side effect of Parkinson's disease pathogenesis, has now been recognized as driver of Parkinson's disease exacerbation. Next-generation sequencing has been used to identify a plethora of long noncoding RNAs (lncRNA) with important transcriptional regulatory functions. Moreover, a myriad of lncRNAs are known to be regulators of inflammatory signaling and neurodegenerative disease, including IL-1 beta secretion and Parkinson's disease. Here, LncZFAS1 was identified as a regulator of inflammasome activation, and pyroptosis in human neuroblast SH-SY5Y cells following MPP+ treatment, a common in vitro Parkinson's disease cell model. Mechanistically, TXNIP ubiquitination through MIB1 E3 ubiquitin ligase regulates NLRP3 inflammasome activation in neuroblasts. In contrast, MPP+ activates the NLPR3 inflammasome through miR590-3p upregulation and direct interference with MIB1-dependent TXNIP ubiquitination. LncZFAS overexpression inhibits this entire pathway through direct interference with miR590-3p, exposing a novel research idea regarding the mechanism of Parkinson's disease.

C1 [Zhu, Ziman; Sun, Ruifeng; Li, Xiaoling; Li, Wenshan] Capital Med Univ, Beijing Rehabil Med Acad, Beijing 100144, China.

[Huang, Peiling; Gong, Weijun] Capital Med Univ, Beijing Rehabil Hosp, Dept Neurol Rehabil, Beijing 100144, China.

C3 Capital Medical University; Capital Medical University

RP Gong, WJ (corresponding author), Capital Med Univ, Beijing Rehabil Hosp, Dept Neurol Rehabil, Beijing 100144, China.

EM gwj197104@ccmu.edu.cn

RI wang, xu/IAN-4886-2023; liu, jiaming/IWE-3196-2023; li,

xiao/HJP-5134-2023; yuan, liping/JPK-7584-2023; li, xiao/HKV-8405-2023;

liang, liang/IAO-8518-2023; li, xiao/GSN-6181-2022; yi,

li/KFR-6141-2024; li, xiaofeng/GXF-9442-2022; Zhang,

Lanyue/JNS-8209-2023; wang, yu/IUQ-6654-2023; Li, Ly/JCD-4746-2023; li,

yang/IQV-3559-2023

FU Natural Science Foundation of China [81972148]; Beijing Municipal

Science and Technology Commission Capital Clinical Feature Applied

Research Project [Z181100001718205]; National Key Research and

Development Program of China [2018YFC0115400]

FX This work was supported by the Natural Science Foundation of China

(81972148), the Beijing Municipal Science and Technology Commission

Capital Clinical Feature Applied Research Project (Z181100001718205),

and the National Key Research and Development Program of China

(2018YFC0115400).

CR Ago T, 2008, CELL, V133, P978, DOI 10.1016/j.cell.2008.04.041

Akula N, 2014, MOL PSYCHIATR, V19, P1179, DOI 10.1038/mp.2013.170

Arnot CJ, 2010, J BIOL CHEM, V285, P19502, DOI 10.1074/jbc.M109.098186

Askarian-Amiri ME, 2011, RNA, V17, P878, DOI 10.1261/rna.2528811

Barry G, 2014, MOL PSYCHIATR, V19, P410, DOI 10.1038/mp.2013.196

Beraud D, 2011, FRONT NEUROSCI-SWITZ, V5, DOI 10.3389/fnins.2011.00080

Broz P, 2016, NAT REV IMMUNOL, V16, P407, DOI 10.1038/nri.2016.58

Chakrabarti S, 2016, AGING DIS, V7, P111, DOI 10.14336/AD.2016.0312

Codolo G, 2013, PLOS ONE, V8, DOI 10.1371/journal.pone.0055375

de Rijk MC, 2000, NEUROLOGY, V54, pS21

Dong D, 2018, CANCER CELL INT, V18, DOI 10.1186/s12935-018-0623-y

Dong D, 2017, ONCOTARGET, V8, P84497, DOI 10.18632/oncotarget.21100

Dorsey ER, 2007, NEUROLOGY, V68, P384, DOI 10.1212/01.wnl.0000247740.47667.03

Edison P, 2013, NEUROPSYCHOPHARMACOL, V38, P938, DOI 10.1038/npp.2012.255

Elkouris M, 2019, FRONT CELL NEUROSCI, V13, DOI 10.3389/fncel.2019.00058

Faghihi MA, 2008, NAT MED, V14, P723, DOI 10.1038/nm1784

Filhoulaud G, 2019, FRONT ENDOCRINOL, V10, DOI 10.3389/fendo.2019.00291

Gao MR, 2020, BRAIN RES BULL, V164, P55, DOI 10.1016/j.brainresbull.2020.08.015

Gerhard A, 2006, NEUROBIOL DIS, V21, P404, DOI 10.1016/j.nbd.2005.08.002

Gordon R, 2018, SCI TRANSL MED, V10, DOI 10.1126/scitranslmed.aah4066

Guo HT, 2015, NAT MED, V21, P677, DOI 10.1038/nm.3893

Gurung P, 2015, TRENDS MOL MED, V21, P193, DOI 10.1016/j.molmed.2014.11.008

Hadjicharalambous MR, 2018, FRONT IMMUNOL, V9, DOI 10.3389/fimmu.2018.02906

He AB, 2019, CELL PROLIFERAT, V52, DOI 10.1111/cpr.12513

He Y, 2001, BRAIN RES, V909, P187, DOI 10.1016/S0006-8993(01)02681-6

Heid ME, 2013, J IMMUNOL, V191, P5230, DOI 10.4049/jimmunol.1301490

Ismael S, 2020, MOL NEUROBIOL, V57, P2495, DOI 10.1007/s12035-020-01893-7

Jagmag SA, 2016, FRONT NEUROSCI-SWITZ, V9, DOI 10.3389/fnins.2015.00503

Javed H, 2020, INT IMMUNOPHARMACOL, V83, DOI 10.1016/j.intimp.2020.106441

Jellinger KA, 2012, MOVEMENT DISORD, V27, P8, DOI 10.1002/mds.23795

Jeon JH, 2005, CANCER RES, V65, P4485, DOI 10.1158/0008-5472.CAN-04-2271

Jo EK, 2016, CELL MOL IMMUNOL, V13, P148, DOI 10.1038/cmi.2015.95

Johnson R, 2010, PHYSIOL GENOMICS, V41, P269, DOI 10.1152/physiolgenomics.00019.2010

Kinney Jefferson W, 2018, Alzheimers Dement (N Y), V4, P575, DOI 10.1016/j.trci.2018.06.014

Korecka JA, 2013, PLOS ONE, V8, DOI 10.1371/journal.pone.0063862

Krishna A, 2014, BMC GENOMICS, V15, DOI 10.1186/1471-2164-15-1154

LANGSTON JW, 1984, ACTA NEUROL SCAND, V70, P49

Lee E, 2019, CELL DEATH DIFFER, V26, P213, DOI 10.1038/s41418-018-0124-5

Li LX, 2019, J ALZHEIMERS DIS, V68, P255, DOI 10.3233/JAD-180814

Lopez-Castejon G, 2020, FEBS J, V287, P11, DOI 10.1111/febs.15118

Makkar R, 2020, INFLAMMATION, V43, P2033, DOI 10.1007/s10753-020-01301-1

Márki S, 2018, PARKINSONS DIS-US, V2018, DOI 10.1155/2018/9351598

Moss DW, 2001, EUR J NEUROSCI, V13, P529, DOI 10.1046/j.1460-9568.2001.01418.x

Nayak D, 2014, ANNU REV IMMUNOL, V32, P367, DOI 10.1146/annurev-immunol-032713-120240

Ng SY, 2013, TRENDS GENET, V29, P461, DOI 10.1016/j.tig.2013.03.002

Ni YH, 2017, CELL MOL NEUROBIOL, V37, P329, DOI 10.1007/s10571-016-0373-0

Nishiyama A, 1999, J BIOL CHEM, V274, P21645, DOI 10.1074/jbc.274.31.21645

Ozaki E, 2015, J INFLAMM RES, V8, P15, DOI 10.2147/JIR.S51250

Poliseno L, 2010, NATURE, V465, P1033, DOI 10.1038/nature09144

Qin Y, 2021, BRAIN BEHAV IMMUN, V91, P324, DOI 10.1016/j.bbi.2020.10.010

Rathinam VAK, 2016, CELL, V165, P792, DOI 10.1016/j.cell.2016.03.046

Rocha NP, 2015, BIOMED RES INT, V2015, DOI 10.1155/2015/628192

Salmena L, 2011, CELL, V146, P353, DOI 10.1016/j.cell.2011.07.014

Samidurai M, 2021, FRONT AGING NEUROSCI, V13, DOI 10.3389/fnagi.2021.661505

Saracchi E, 2014, AGING DIS, V5, P27, DOI 10.14366/AD.2014.050027

Sarkar SN, 2019, AGING DIS, V10, P329, DOI 10.14336/AD.2018.0409

Sarkar S, 2017, NPJ PARKINSON DIS, V3, DOI 10.1038/s41531-017-0032-2

Schmidt F, 2009, PLOS ONE, V4, DOI 10.1371/journal.pone.0006215

Schroder K, 2010, CELL, V140, P821, DOI 10.1016/j.cell.2010.01.040

Shao BZ, 2015, FRONT PHARMACOL, V6, DOI 10.3389/fphar.2015.00262

Son JH, 1999, J NEUROSCI, V19, P10, DOI 10.1523/JNEUROSCI.19-01-00010.1999

Sorbara MT, 2011, CELL RES, V21, P558, DOI 10.1038/cr.2011.20

Soreq L, 2014, PLOS COMPUT BIOL, V10, DOI 10.1371/journal.pcbi.1003517

Vanaja SK, 2015, TRENDS CELL BIOL, V25, P308, DOI 10.1016/j.tcb.2014.12.009

von Herrmann KM, 2018, NPJ PARKINSON DIS, V4, DOI 10.1038/s41531-018-0061-5

Wang HJ, 2017, BBA-MOL BASIS DIS, V1863, P2019, DOI 10.1016/j.bbadis.2016.11.034

Wang S, 2017, ONCOTARGET, V8, P24449, DOI 10.18632/oncotarget.15511

Wang S, 2019, INT IMMUNOPHARMACOL, V67, P458, DOI 10.1016/j.intimp.2018.12.019

Wu DC, 2002, J NEUROSCI, V22, P1763, DOI 10.1523/JNEUROSCI.22-05-01763.2002

Wu T, 2017, J CELL BIOCHEM, V118, P3281, DOI 10.1002/jcb.25979

Xicoy H, 2017, MOL NEURODEGENER, V12, DOI 10.1186/s13024-017-0149-0

Yamaguchi F, 2008, INT J ONCOL, V32, P377

Yao RW, 2019, NAT CELL BIOL, V21, P542, DOI 10.1038/s41556-019-0311-8

Ye Y, 2018, HUM CELL, V31, P14, DOI 10.1007/s13577-017-0179-5

Yoshihara E, 2014, FRONT IMMUNOL, V4, DOI 10.3389/fimmu.2013.00514

Yoshihara E, 2010, NAT COMMUN, V1, DOI 10.1038/ncomms1127

Zhang Q, 2021, CELL MOL NEUROBIOL, V41, P1773, DOI 10.1007/s10571-020-00946-8

Zhang XM, 2014, ACTA PHARMACOL SIN, V35, P945, DOI 10.1038/aps.2014.36

Zhang Y, 2016, SCI REP-UK, V6, DOI 10.1038/srep22384

Zhou RB, 2010, NAT IMMUNOL, V11, P136, DOI 10.1038/ni.1831

NR 80

TC 11

Z9 11

U1 1

U2 13

PU SPRINGER

PI NEW YORK

PA ONE NEW YORK PLAZA, SUITE 4600, NEW YORK, NY, UNITED STATES

SN 0893-7648

EI 1559-1182

J9 MOL NEUROBIOL

JI Mol. Neurobiol.

PD FEB

PY 2022

VL 59

IS 2

BP 778

EP 799

DI 10.1007/s12035-021-02619-z

EA NOV 2021

PG 22

WC Neurosciences

WE Science Citation Index Expanded (SCI-EXPANDED)

SC Neurosciences & Neurology

GA ZD0FK

UT WOS:000718083500001

PM 34775541

OA Green Published, Green Submitted, hybrid

DA 2024-08-12

ER

PT J

AU Chen, HY

Wu, DH

Ding, XT

Ying, WH

AF Chen, Heyu

Wu, Danhong

Ding, Xianting

Ying, Weihai

TI SIRT2 is required for lipopolysaccharide-induced activation of BV2

microglia

SO NEUROREPORT

LA English

DT Article

DE cytokines; inflammation; microglia; sirtuin 2

ID POLY(ADP-RIBOSE) POLYMERASE; parkinson's disease; REGULATOR; CELLS;

NEUROINFLAMMATION; NEUROPROTECTION; DIFFERENTIATION; INFLAMMATION;

ISCHEMIA; RECOVERY

AB It has been reported that inhibition of sirtuin 2 (SIRT2), a sirtuin family protein, can decrease cellular and tissue injuries in models of Parkinson's disease (PD) and Huntington's disease (HD); however, the mechanisms underlying these observations have remained unclear. Because inflammation plays key pathological roles in multiple major neurological disorders including PD and HD, in our current study we tested our hypothesis that SIRT2 plays an important role in microglial activation. We found that treatment of BV2 microglia with lipopolysaccharides led to significant increases in NO and inducible nitric oxide synthase mRNA levels, as well as increases in the levels of tumor necrosis factor-a and interleukin 6 mRNA, which indicated microglial activation. These increases were significantly decreased in the cells with SIRT2 silencing-produced decreases in the SIRT2 level. These observations suggest that SIRT2 is required for lipopolysaccharide-induced microglial activation. The findings also suggest that SIRT2 may be a therapeutic target for inhibiting the inflammatory responses in neurological disorders such as PD and HD. Copyright (C) 2015 Wolters Kluwer Health, Inc. All rights reserved.

C1 [Chen, Heyu; Ding, Xianting; Ying, Weihai] Shanghai Jiao Tong Univ, Sch Biomed Engn, Med X Res Inst, Shanghai 200030, China.

[Ying, Weihai] Shanghai Jiao Tong Univ, Ruijin Hosp, Dept Neurol, Shanghai 200030, China.

[Wu, Danhong] Shanghai Jiao Tong Univ, Peoples Hosp 3, Dept Neurol, Shanghai 200030, China.

C3 Shanghai Jiao Tong University; Shanghai Jiao Tong University; Shanghai

Jiao Tong University

RP Ying, WH (corresponding author), Shanghai Jiao Tong Univ, Sch Biomed Engn, Med X Res Inst, 1954 Huashan Rd, Shanghai 200030, China.

EM weihaiy@sjtu.edu.cn

OI Ding, Xianting/0000-0002-1549-3499

FU Chinese National Science Foundation [81171098, 81271305]; National

Science and Technology Major Project Grant for 'Major New Drugs

Innovation and Development' [2014ZX09507008]; Shanghai Jiao Tong

University Grants for Interdisciplinary Research on Medicine and

Engineering; Science and Technology Committee, Baoshan District of

Shanghai [11-E-3]

FX This study was supported by Chinese National Science Foundation Grants

#81171098 and #81271305 (to W. Y.), a National Science and Technology

Major Project Grant for 'Major New Drugs Innovation and Development'

#2014ZX09507008 (to X. D.), Shanghai Jiao Tong University Grants for

Interdisciplinary Research on Medicine and Engineering (to W. Y.), and a

Research Fund (#11-E-3) of the Science and Technology Committee, Baoshan

District of Shanghai (to D. W.). The authors acknowledge Jiaxiang Shao,

Hui Nie, and Yexin Li for their technical assistance.

CR Bi W, 2011, BRAIN RES, V1395, P12, DOI 10.1016/j.brainres.2011.04.019

BLASI E, 1990, J NEUROIMMUNOL, V27, P229, DOI 10.1016/0165-5728(90)90073-V

Chen X, 2013, J NEUROSCI, V33, P9202, DOI 10.1523/JNEUROSCI.5336-12.2013

Chopra V, 2012, CELL REP, V2, P1492, DOI 10.1016/j.celrep.2012.11.001

Donmez G, 2013, EMBO MOL MED, V5, P344, DOI 10.1002/emmm.201302451

Endres M, 1997, J CEREBR BLOOD F MET, V17, P1143, DOI 10.1097/00004647-199711000-00002

Frank-Cannon TC, 2009, MOL NEURODEGENER, V4, DOI 10.1186/1750-1326-4-47

Goto S, 2002, STROKE, V33, P1101, DOI 10.1161/01.STR.0000014203.65693.1E

Guarente L, 2000, GENE DEV, V14, P1021

Harting K, 2010, EUR J CELL BIOL, V89, P262, DOI 10.1016/j.ejcb.2009.11.006

He X, 2012, BIOCHEM BIOPH RES CO, V417, P468, DOI 10.1016/j.bbrc.2011.11.141

Hirsch EC, 2009, LANCET NEUROL, V8, P382, DOI 10.1016/S1474-4422(09)70062-6

Lee JK, 2011, J NEUROSCI, V31, P11879, DOI 10.1523/JNEUROSCI.1002-11.2011

Li WB, 2007, J NEUROSCI, V27, P2606, DOI 10.1523/JNEUROSCI.4181-06.2007

Li YZ, 2011, GENES CELLS, V16, P34, DOI 10.1111/j.1365-2443.2010.01460.x

Liu L, 2014, FRONT AGING NEUROSCI, V6, DOI 10.3389/fnagi.2014.00184

Luthi-Carter R, 2010, P NATL ACAD SCI USA, V107, P7927, DOI 10.1073/pnas.1002924107

Nagayama T, 2000, J NEUROCHEM, V74, P1636, DOI 10.1046/j.1471-4159.2000.0741636.x

Outeiro TF, 2007, SCIENCE, V317, P516, DOI 10.1126/science.1143780

Pais TF, 2013, EMBO J, V32, P2603, DOI 10.1038/emboj.2013.200

Pandithage R, 2008, J CELL BIOL, V180, P915, DOI 10.1083/jcb.200707126

Tang BL, 2008, CELL MOTIL CYTOSKEL, V65, P179, DOI 10.1002/cm.20253

Wang F, 2007, AGING CELL, V6, P505, DOI 10.1111/j.1474-9726.2007.00304.x

Yang Y, 2010, J NEUROCHEM, V112, P134, DOI 10.1111/j.1471-4159.2009.06433.x

NR 24

TC 34

Z9 36

U1 0

U2 15

PU LIPPINCOTT WILLIAMS & WILKINS

PI PHILADELPHIA

PA TWO COMMERCE SQ, 2001 MARKET ST, PHILADELPHIA, PA 19103 USA

SN 0959-4965

EI 1473-558X

J9 NEUROREPORT

JI Neuroreport

PD JAN 21

PY 2015

VL 26

IS 2

BP 88

EP 93

DI 10.1097/WNR.0000000000000305

PG 6

WC Neurosciences

WE Science Citation Index Expanded (SCI-EXPANDED)

SC Neurosciences & Neurology

GA AX9UG

UT WOS:000347246900007

PM 25536118

DA 2024-08-12

ER

PT J

AU Lahooti, B

Chhibber, T

Bagchi, S

Varahachalam, SP

Jayant, RD

AF Lahooti, Behnaz

Chhibber, Tanya

Bagchi, Sounak

Varahachalam, Sree Pooja

Jayant, Rahul D.

TI Therapeutic role of inflammasome inhibitors in neurodegenerative

disorders

SO BRAIN BEHAVIOR AND IMMUNITY

LA English

DT Review

DE Neuroinflammation; neurodegenerative disease; NLRP3 inflammasomes;

Inflammasome inhibitors; NeuroHIV; Blood-brain barrier (BBB);

Nanotechnology

ID WEST-NILE-VIRUS; HIPPOCAMPAL NEURON MORPHOLOGY; BLOOD MONONUCLEAR-CELLS;

CENTRAL-NERVOUS-SYSTEM; NLRP3 INFLAMMASOME; parkinson's disease;

alzheimer's disease; MOUSE MODEL; INTERLEUKIN-1-BETA PRODUCTION; MICRORNA

THERAPEUTICS

AB Neuroinflammation, characterized by the activation of glial cells, is a hallmark in several neurological and neurodegenerative disorders. Inadequate inflammation cannot eliminate the infection of pathogens, while excessive or hyper-reactive inflammation can cause chronic or systemic inflammatory diseases affecting the central nervous system (CNS). In response to a brain injury or pathogen invasion, the pathogen recognition receptors (PRRs) expressed on glial cells are activated via binding to cellular damage-associated molecular patterns (DAMPs) or pathogen-associated molecular patterns (PAMPs). This subsequently leads to the activation of NOD (nucleotide-binding oligomerization domain)-like receptor proteins (NLRs). In neurodegenerative disease such as HIV-1-associated neurocognitive disorders (HAND), Alzheimer's disease (AD), Parkinson's disease (PD), and multiple sclerosis (MS), chronic inflammation is a critical contributing factor for disease manifestation including pathogenesis. Emerging evidence points to the involvement of "inflammasomes", especially the nucleotide-binding oligomerization domain, leucine-rich repeat, and pyrin domain-containing (NLRP) complex in the development of these diseases. The activated NLRP3 results in the proteolytic activation of caspase-1 that facilitates the cleavage of pro-IL-1 beta and the secretion of IL-1 beta and IL-18 proinflammatory cytokines. Accordingly, these and other seminal findings have led to the development of NLRP-targeting small-molecule therapeutics as possible treatment options for neurodegenerative disorders. In this review, we will discuss the new advances and evidence-based literature concerning the role of inflammasomes in neurodegenerative disease, its role in the neurological repercussions of CNS chronic infection, and the examples of preclinical or clinically tested NLRP inhibitors as potential strategies for the treatment of chronic neurological diseases.

C1 [Lahooti, Behnaz; Chhibber, Tanya; Bagchi, Sounak; Varahachalam, Sree Pooja; Jayant, Rahul D.] Texas Tech Univ, Hlth Sci Ctr TTUHSC, Jerry H Hodge Sch Pharm, Dept Pharmaceut Sci, Amarillo, TX 79106 USA.

C3 Texas Tech University System; Texas Tech University Health Science

Center; Texas Tech University Health Sciences Center Amarillo

RP Jayant, RD (corresponding author), Texas Tech Univ, Hlth Sci Ctr TTUHSC, Jerry H Hodge Sch Pharm, Dept Pharmaceut Sci, Amarillo, TX 79106 USA.

EM rahuldev.jayant@gmail.com

RI Bagchi, Sounak/AAS-9533-2021; Jayant, Rahul/K-7934-2019

OI Bagchi, Sounak/0000-0002-7209-3213;

FU TTUHSC, Department of Pharmaceutical Sciences start-up funds

FX This work was supported by TTUHSC, Department of Pharmaceutical Sciences

start-up funds for RDJ.

CR Abulafia DP, 2009, J CEREBR BLOOD F MET, V29, P534, DOI 10.1038/jcbfm.2008.143

Adamczak S, 2012, J NEUROSURG, V117, P1119, DOI 10.3171/2012.9.JNS12815

Adamczak SE, 2014, J CEREBR BLOOD F MET, V34, P621, DOI 10.1038/jcbfm.2013.236

Ahmad R, 2002, J VIROL, V76, P12448, DOI 10.1128/JVI.76.24.12448-12456.2002

Ahn H, 2014, IMMUNOBIOLOGY, V219, P315, DOI 10.1016/j.imbio.2013.11.003

Alboni S, 2010, J NEUROINFLAMM, V7, DOI 10.1186/1742-2094-7-9

Albornoz EA, 2018, EXPERIENTIA SUPPL, V108, P41, DOI 10.1007/978-3-319-89390-7_3

Alter G, 2007, J IMMUNOL, V178, P7658, DOI 10.4049/jimmunol.178.12.7658

Altfeld M, 2015, NAT IMMUNOL, V16, P554, DOI 10.1038/ni.3157

Atluri VSR, 2016, SCI REP, V6, P1, DOI [10.1038/s41598-016-0001-8, DOI 10.1038/S41598-016-0001-8]

Baecher-Allan C, 2018, NEURON, V97, P742, DOI 10.1016/j.neuron.2018.01.021

Bagchi S, 2019, DRUG DES DEV THER, V13, P3591, DOI 10.2147/DDDT.S218708

Bai FW, 2010, J INFECT DIS, V202, P1804, DOI 10.1086/657416

Balasubramaniam Muthukumar, 2019, J Life Sci (Westlake Village), V1, P4

Bauernfeind FG, 2009, J IMMUNOL, V183, P787, DOI 10.4049/jimmunol.0901363

Beatman E, 2012, VIROLOGY, V433, P262, DOI 10.1016/j.virol.2012.08.016

Bekkering S, 2018, CELL, V172, P135, DOI 10.1016/j.cell.2017.11.025

Bilgrami Mohammed, 2014, Handb Clin Neurol, V121, P1321, DOI 10.1016/B978-0-7020-4088-7.00090-0

Brück W, 2005, J NEUROL, V252, P10, DOI 10.1007/s00415-005-2011-5

Centers for Disease Control and Prevention (CDC), 2012, MMWR Morb Mortal Wkly Rep, V60, P1729

Cheeran MCJ, 2005, J NEUROVIROL, V11, P512, DOI 10.1080/13550280500384982

Chen JQ, 2018, NATURE, V564, P71, DOI 10.1038/s41586-018-0761-3

Chen LN, 2011, PLOS ONE, V6, DOI 10.1371/journal.pone.0025994

Chhibber T, 2020, DRUG DISCOV TODAY, V25, P456, DOI 10.1016/j.drudis.2019.11.010

Chivero ET, 2017, J NEUROSCI, V37, P3599, DOI 10.1523/JNEUROSCI.3045-16.2017

Cho MH, 2014, AUTOPHAGY, V10, P1761, DOI 10.4161/auto.29647

Christ A, 2018, CELL, V172, P162, DOI 10.1016/j.cell.2017.12.013

Ciraci C, 2016, PLOS ONE, V11, DOI 10.1371/journal.pone.0151252

Codolo G, 2013, PLOS ONE, V8, DOI 10.1371/journal.pone.0055375

Coll RC, 2015, NAT MED, V21, P248, DOI 10.1038/nm.3806

Colomer-Lluch M, 2018, FRONT IMMUNOL, V9, DOI 10.3389/fimmu.2018.02876

Leal VNC, 2020, MOL IMMUNOL, V118, P9, DOI 10.1016/j.molimm.2019.12.001

Daniels BP, 2014, MBIO, V5, DOI 10.1128/mBio.01476-14

Daniels MJD, 2016, NAT COMMUN, V7, DOI 10.1038/ncomms12504

Davis BK, 2011, ANNU REV IMMUNOL, V29, P707, DOI 10.1146/annurev-immunol-031210-101405

Dempsey C, 2017, BRAIN BEHAV IMMUN, V61, P306, DOI 10.1016/j.bbi.2016.12.014

Dendrou CA, 2015, NAT REV IMMUNOL, V15, P545, DOI 10.1038/nri3871

Doitsh G, 2016, CELL HOST MICROBE, V19, P280, DOI 10.1016/j.chom.2016.02.012

Dunning CJR, 2013, PRION, V7, P92, DOI 10.4161/pri.23806

Ekstrand JJ, 2012, SEMIN PEDIATR NEUROL, V19, P96, DOI 10.1016/j.spen.2012.02.004

EPSTEIN LG, 1993, ANN NEUROL, V33, P429, DOI 10.1002/ana.410330502

Fann DYW, 2013, CELL DEATH DIS, V4, DOI 10.1038/cddis.2013.326

Feng L, 2015, J NEUROINFLAMM, V12, DOI 10.1186/s12974-015-0409-2

Fernandes-Alnemri T, 2013, J IMMUNOL, V191, P3995, DOI 10.4049/jimmunol.1301681

Freeman LC, 2016, J NEUROCHEM, V136, P29, DOI 10.1111/jnc.13217

Frigerio CS, 2016, ANNU REV NEUROSCI, V39, P57, DOI 10.1146/annurev-neuro-070815-014015

Frohman EM, 2006, NEW ENGL J MED, V354, P942, DOI 10.1056/NEJMra052130

Garcia-Tapia D, 2006, VET IMMUNOL IMMUNOP, V110, P229, DOI 10.1016/j.vetimm.2005.10.003

Getts DR, 2008, J EXP MED, V205, P2319, DOI 10.1084/jem.20080421

Glass CK, 2010, CELL, V140, P918, DOI 10.1016/j.cell.2010.02.016

Gold M, 2015, SEMIN IMMUNOPATHOL, V37, P607, DOI 10.1007/s00281-015-0518-0

Griciuc A, 2013, NEURON, V78, P631, DOI 10.1016/j.neuron.2013.04.014

Gris D, 2010, J IMMUNOL, V185, P974, DOI 10.4049/jimmunol.0904145

Guo HT, 2015, NAT MED, V21, P677, DOI 10.1038/nm.3893

Gustin A, 2015, PLOS ONE, V10, DOI 10.1371/journal.pone.0130624

Halle A, 2008, NAT IMMUNOL, V9, P857, DOI 10.1038/ni.1636

He HB, 2018, NAT COMMUN, V9, DOI 10.1038/s41467-018-04947-6

He XL, 2020, CELL MOL IMMUNOL, V17, P283, DOI 10.1038/s41423-019-0260-y

He Y, 2014, J BIOL CHEM, V289, P1142, DOI 10.1074/jbc.M113.515080

Heaton NS, 2010, CELL HOST MICROBE, V8, P422, DOI 10.1016/j.chom.2010.10.006

Heneka MT, 2018, NAT REV NEUROSCI, V19, P610, DOI 10.1038/s41583-018-0055-7

Heneka MT, 2017, BRAIN PATHOL, V27, P220, DOI 10.1111/bpa.12483

Heneka MT, 2013, NATURE, V493, P674, DOI 10.1038/nature11729

Hernandez JC, 2015, PLOS ONE, V10, DOI 10.1371/journal.pone.0119844

Herskovitz J, 2019, J NEUROIMMUNE PHARM, V14, P52, DOI 10.1007/s11481-018-9785-6

Hettiarachchi S.D., 2019, J CONTROL RELEASE

Hewett Sandra J, 2012, Eur J Neurodegener Dis, V1, P195

Hirsch EC, 2009, LANCET NEUROL, V8, P382, DOI 10.1016/S1474-4422(09)70062-6

Hook VYH, 2008, J BIOL CHEM, V283, P7745, DOI 10.1074/jbc.M708362200

Hosseini S, 2018, J NEUROSCI, V38, P3060, DOI 10.1523/JNEUROSCI.1740-17.2018

Huang WX, 2004, MULT SCLER J, V10, P482, DOI 10.1191/1352458504ms1071oa

Hunsperger E, 2005, J NEUROVIROL, V11, P11, DOI 10.1080/13550280590900454

Hunsperger EA, 2006, J NEUROVIROL, V12, P129, DOI 10.1080/13550280600758341

Iannello A, 2010, J INFECT DIS, V201, P608, DOI 10.1086/650314

Inoue M, 2013, AUTOIMMUN DIS, V2013, DOI 10.1155/2013/859145

Inoue M, 2012, SCI SIGNAL, V5, DOI 10.1126/scisignal.2002767

Irrera N, 2017, FRONT PHARMACOL, V8, DOI 10.3389/fphar.2017.00459

Jackson AL, 2010, DISCOV MED, V9, P311

Jang H, 2012, J NEUROSCI, V32, P1545, DOI 10.1523/JNEUROSCI.5123-11.2012

Jayant RD, 2018, SCI REP-UK, V8, DOI 10.1038/s41598-018-31285-w

Jayant RD, 2017, J NEUROVIROL, V23, P603, DOI 10.1007/s13365-017-0538-8

Jayant RD, 2015, INT J NANOMED, V10, P1077, DOI 10.2147/IJN.S76517

Jha S, 2010, J NEUROSCI, V30, P15811, DOI 10.1523/JNEUROSCI.4088-10.2010

Jiang W, 2017, J NEUROINFLAMM, V14, DOI 10.1186/s12974-017-0980-9

Jiao JH, 2020, FRONT MOL BIOSCI, V7, DOI 10.3389/fmolb.2020.00037

Johann S, 2015, GLIA, V63, P2260, DOI 10.1002/glia.22891

Juliana C, 2012, J BIOL CHEM, V287, P36617, DOI 10.1074/jbc.M112.407130

Juliana C, 2010, J BIOL CHEM, V285, P9792, DOI 10.1074/jbc.M109.082305

Jurgens HA, 2012, J NEUROSCI, V32, P3958, DOI 10.1523/JNEUROSCI.6389-11.2012

Katuri A, 2019, EXP MOL PATHOL, V108, P64, DOI 10.1016/j.yexmp.2019.03.008

Kaushal V, 2015, CELL DEATH DIFFER, V22, P1676, DOI 10.1038/cdd.2015.16

Kaushik A., 2018, FUTURE MED

Kaushik A, 2018, DRUG DISCOV TODAY, V23, P1007, DOI 10.1016/j.drudis.2017.11.010

Kaushik A, 2014, EXPERT OPIN DRUG DEL, V11, P1635, DOI 10.1517/17425247.2014.933803

Kaushik DK, 2012, PLOS ONE, V7, DOI 10.1371/journal.pone.0032270

Kelleher P, 2018, NAT IMMUNOL, V19, P433, DOI 10.1038/s41590-018-0089-z

Kelley N, 2019, INT J MOL SCI, V20, DOI 10.3390/ijms20133328

Kerr N, 2018, PLOS ONE, V13, DOI 10.1371/journal.pone.0210128

Kigerl KA, 2014, EXP NEUROL, V258, P5, DOI 10.1016/j.expneurol.2014.01.001

Kuwar R, 2019, J NEUROINFLAMM, V16, DOI 10.1186/s12974-019-1471-y

Lamkanfi M, 2009, J CELL BIOL, V187, P61, DOI 10.1083/jcb.200903124

Lawana V, 2017, J NEUROIMMUNE PHARM, V12, P624, DOI 10.1007/s11481-017-9746-5

Li DS, 2018, HUM CELL, V31, P106, DOI 10.1007/s13577-017-0187-5

Li JK, 2012, MICROBES INFECT, V14, P159, DOI 10.1016/j.micinf.2011.09.001

Liang YR, 2015, BRAIN RES, V1622, P43, DOI 10.1016/j.brainres.2015.06.008

Lim SM, 2017, FRONT MICROBIOL, V8, DOI 10.3389/fmicb.2017.01556

LINDESAY J, 1989, SOC PSYCH PSYCH EPID, V24, P249, DOI 10.1007/BF01788966

Liu HD, 2013, NEUROCHEM RES, V38, P2072, DOI 10.1007/s11064-013-1115-z

Liu L, 2014, AGEING RES REV, V15, P6, DOI 10.1016/j.arr.2013.12.007

Liu L, 2014, NEUROBIOL AGING, V35, P309, DOI 10.1016/j.neurobiolaging.2013.08.016

Lu B, 2012, NATURE, V488, P670, DOI 10.1038/nature11290

Ma CC, 2014, J CLIN NEUROSCI, V21, P1388, DOI 10.1016/j.jocn.2013.11.032

Mamik MK, 2017, BRAIN, V140, P2273, DOI 10.1093/brain/awx133

Mao ZJ, 2017, NEUROCHEM RES, V42, P1104, DOI 10.1007/s11064-017-2185-0

Marchetti C, 2018, P NATL ACAD SCI USA, V115, pE1530, DOI 10.1073/pnas.1716095115

Martinon F, 2002, MOL CELL, V10, P417, DOI 10.1016/S1097-2765(02)00599-3

Maturana CJ, 2017, DEV NEUROBIOL, V77, P625, DOI 10.1002/dneu.22409

McKenzie BA, 2018, P NATL ACAD SCI USA, V115, pE6065, DOI 10.1073/pnas.1722041115

Mega JL, 2015, LANCET, V385, P2264, DOI 10.1016/S0140-6736(14)61730-X

Mhyre Timothy R, 2012, Subcell Biochem, V65, P389, DOI 10.1007/978-94-007-5416-4_16

Minkiewicz J, 2013, GLIA, V61, P1113, DOI 10.1002/glia.22499

Miñones-Moyano E, 2011, HUM MOL GENET, V20, P3067, DOI 10.1093/hmg/ddr210

Mogensen TH, 2009, CLIN MICROBIOL REV, V22, P240, DOI 10.1128/CMR.00046-08

MONATH TP, 1983, LAB INVEST, V48, P399

Mouton-Liger F, 2018, GLIA, V66, P1736, DOI 10.1002/glia.23337

Murray K, 2006, EPIDEMIOL INFECT, V134, P1325, DOI 10.1017/S0950268806006339

Nagyoszi P, 2015, J NEUROCHEM, V135, P551, DOI 10.1111/jnc.13197

Nair M, 2016, ADV DRUG DELIVER REV, V103, P202, DOI 10.1016/j.addr.2016.02.008

Netea MG, 2009, BLOOD, V113, P2324, DOI 10.1182/blood-2008-03-146720

Nowarski R, 2015, CELL, V163, P1444, DOI 10.1016/j.cell.2015.10.072

O'Brien WT, 2020, J NEUROINFLAMM, V17, DOI 10.1186/s12974-020-01778-5

Obeso JA, 2010, NAT MED, V16, P653, DOI 10.1038/nm.2165

Ojeda DS, 2018, FRONT IMMUNOL, V9, DOI 10.3389/fimmu.2018.02633

Paoletti A, 2019, CELL REP, V28, P3381, DOI 10.1016/j.celrep.2019.02.095

Parquet MD, 2001, FEBS LETT, V500, P17, DOI 10.1016/S0014-5793(01)02573-X

Periyasamy P, 2019, BRAIN BEHAV IMMUN, V80, P227, DOI 10.1016/j.bbi.2019.03.011

Przedborski S, 2017, NAT REV NEUROSCI, V18, P251, DOI 10.1038/nrn.2017.25

Quick ED, 2014, J VIROL, V88, P13005, DOI 10.1128/JVI.01994-14

Riazi K, 2015, J NEUROSCI, V35, P4942, DOI 10.1523/JNEUROSCI.4485-14.2015

Ridker PM, 2017, NEW ENGL J MED, V377, P1119, DOI 10.1056/NEJMoa1707914

Rossini Giada, 2013, World J Virol, V2, P49, DOI 10.5501/wjv.v2.i2.49

Rupaimoole R, 2017, NAT REV DRUG DISCOV, V16, P203, DOI 10.1038/nrd.2016.246

Saadane A, 2007, AM J RESP CELL MOL, V36, P728, DOI 10.1165/rcmb.2006-0323OC

Samuel MA, 2007, P NATL ACAD SCI USA, V104, P17140, DOI 10.1073/pnas.0705837104

Sánchez-Fernández A, 2019, FRONT IMMUNOL, V10, DOI 10.3389/fimmu.2019.02578

Santos LE, 2016, BRAIN BEHAV IMMUN, V55, P151, DOI 10.1016/j.bbi.2015.11.011

Saresella M, 2016, MOL NEURODEGENER, V11, DOI 10.1186/s13024-016-0088-1

Savage CD, 2012, FRONT IMMUNOL, V3, DOI 10.3389/fimmu.2012.00288

Shah S, 2014, J CHILD NEUROL, V29, pNP49, DOI 10.1177/0883073813499610

Shiraishi Y, 2021, J ORTHOP SCI, V26, P487, DOI 10.1016/j.jos.2020.04.006

Shrestha B, 2003, J VIROL, V77, P13203, DOI 10.1128/JVI.77.24.13203-13213.2003

Simonson B, 2015, MINI-REV MED CHEM, V15, P467, DOI 10.2174/1389557515666150324123208

Song JJ, 2015, PLOS ONE, V10, DOI 10.1371/journal.pone.0121011

Song LM, 2017, FRONT CELL NEUROSCI, V11, DOI 10.3389/fncel.2017.00063

Sun BB, 2013, SMALL, V9, P1595, DOI 10.1002/smll.201201962

Sun ZZ, 2020, OXID MED CELL LONGEV, V2020, DOI 10.1155/2020/7879629

Tan MS, 2014, CELL DEATH DIS, V5, DOI 10.1038/cddis.2014.348

Tarawneh Rawan, 2012, Cold Spring Harb Perspect Med, V2, pa006148, DOI 10.1101/cshperspect.a006148

Thakkar R, 2016, OXID MED CELL LONGEV, V2016, DOI 10.1155/2016/8309031

Thomson CA, 2014, J NEUROINFLAMM, V11, DOI 10.1186/1742-2094-11-73

Tomonaga K, 2004, TRENDS MOL MED, V10, P71, DOI 10.1016/j.molmed.2003.12.001

Tsai SJ, 2017, CYTOKINE GROWTH F R, V37, P89, DOI 10.1016/j.cytogfr.2017.06.001

Vandergaast R, 2012, PLOS ONE, V7, DOI 10.1371/journal.pone.0045800

Verma S, 2009, VIROLOGY, V385, P425, DOI 10.1016/j.virol.2008.11.047

Voet S, 2019, EMBO MOL MED, V11, DOI 10.15252/emmm.201810248

Walsh JG, 2014, RETROVIROLOGY, V11, DOI 10.1186/1742-4690-11-35

Walsh JG, 2014, NAT REV NEUROSCI, V15, P84, DOI 10.1038/nrn3638

Wang PH, 2010, NAT IMMUNOL, V11, P912, DOI 10.1038/ni.1933

Wang S, 2014, PLOS ONE, V9, DOI 10.1371/journal.pone.0089848

Wang SB, 2015, J GASTROEN HEPATOL, V30, P504, DOI 10.1111/jgh.12710

Wang S, 2019, INT IMMUNOPHARMACOL, V67, P458, DOI 10.1016/j.intimp.2018.12.019

Wang T, 2004, NAT MED, V10, P1366, DOI 10.1038/nm1140

Wang W, 2016, P NATL ACAD SCI USA, V113, P9587, DOI 10.1073/pnas.1610099113

Yan YQ, 2015, CELL, V160, P62, DOI 10.1016/j.cell.2014.11.047

Yang SJ, 2018, CELL MOL NEUROBIOL, V38, P595, DOI 10.1007/s10571-017-0526-9

Yang Y, 2019, CELL DEATH DIS, V10, DOI 10.1038/s41419-019-1413-8

Yin J, 2018, MOL NEUROBIOL, V55, P1977, DOI 10.1007/s12035-017-0467-9

Yong VW, 2010, NEUROSCIENTIST, V16, P408, DOI 10.1177/1073858410371379

Youm YH, 2015, NAT MED, V21, P263, DOI 10.1038/nm.3804

Yuan Y, 2019, CURR PHARM DESIGN, V25, P2375, DOI 10.2174/1381612825666190722114248

Zahid A, 2019, FRONT IMMUNOL, V10, DOI 10.3389/fimmu.2019.02538

Zendedel A, 2016, MOL NEUROBIOL, V53, P3063, DOI 10.1007/s12035-015-9203-5

Zeng R, 2019, J CLIN NEUROSCI, V65, P125, DOI 10.1016/j.jocn.2019.04.004

Zhang ZY, 2013, J CELL MOL MED, V17, P1566, DOI 10.1111/jcmm.12124

Zhao G, 2017, ONCOTARGET, V8, P68153, DOI 10.18632/oncotarget.19249

Zhou Y, 2016, MOL NEURODEGENER, V11, DOI 10.1186/s13024-016-0094-3

Zhou YQ, 2019, NANOSCALE, V11, P22387, DOI 10.1039/c9nr08194a

NR 187

TC 29

Z9 33

U1 1

U2 35

PU ACADEMIC PRESS INC ELSEVIER SCIENCE

PI SAN DIEGO

PA 525 B ST, STE 1900, SAN DIEGO, CA 92101-4495 USA

SN 0889-1591

EI 1090-2139

J9 BRAIN BEHAV IMMUN

JI Brain Behav. Immun.

PD JAN

PY 2021

VL 91

BP 771

EP 783

DI 10.1016/j.bbi.2020.11.004

PG 13

WC Immunology; Neurosciences; Psychiatry

WE Science Citation Index Expanded (SCI-EXPANDED)

SC Immunology; Neurosciences & Neurology; Psychiatry

GA PK3VT

UT WOS:000602377700008

PM 33157255

DA 2024-08-12

ER

PT J

AU Earls, RH

Lee, JK

AF Earls, Rachael H.

Lee, Jae-Kyung

TI The role of natural killer cells in Parkinson's disease

SO EXPERIMENTAL AND MOLECULAR MEDICINE

LA English

DT Review

ID AGGREGATED ALPHA-SYNUCLEIN; HUMAN NK CELLS; EXPERIMENTAL AUTOIMMUNE

ENCEPHALOMYELITIS; T-CELLS; INTERFERON-GAMMA; SELF-TOLERANCE; ACTIVATES

MICROGLIA; LYMPHOCYTE SUBSETS; ADAPTIVE IMMUNITY; MEDIATED LYSIS

AB Numerous lines of evidence indicate an association between sustained inflammation and Parkinson's disease, but whether increased inflammation is a cause or consequence of Parkinson's disease remains highly contested. Extensive efforts have been made to characterize microglial function in Parkinson's disease, but the role of peripheral immune cells is less understood. Natural killer cells are innate effector lymphocytes that primarily target and kill malignant cells. Recent scientific discoveries have unveiled numerous novel functions of natural killer cells, such as resolving inflammation, forming immunological memory, and modulating antigen-presenting cell function. Furthermore, natural killer cells are capable of homing to the central nervous system in neurological disorders that exhibit exacerbated inflammation and inhibit hyperactivated microglia. Recently, a study demonstrated that natural killer cells scavenge alpha-synuclein aggregates, the primary component of Lewy bodies, and systemic depletion of natural killer cells results in exacerbated neuropathology in a mouse model of alpha-synucleinopathy, making them a highly relevant cell type in Parkinson's disease. However, the exact role of natural killer cells in Parkinson's disease remains elusive. In this review, we introduce the systemic inflammatory process seen in Parkinson's disease, with a particular focus on the direct and indirect modulatory capacity of natural killer cells in the context of Parkinson's disease.

Parkinson's disease: How natural killer cells can help Understanding the roles that inflammation and immune cells called natural killer (NK) cells play in Parkinson's disease (PD) may help in finding new treatments. In PD, a neurodegenerative disease, the protein alpha-synuclein is misshapen and accumulates in brain cells, causing inflammation. NK cells, which mostly target cancer cells, have recently been shown to resolve inflammation. Jae-Kyung Lee and Rachael H. Earls of the University of Georgia College of Veterinary Medicine, Athens, USA, reviewed the role of NK cells in PD. They report that NK cells can degrade alpha-synuclein aggregates. Further, NK cells are recruited to areas of inflammation where they then decrease the alpha-synuclein burden and reduce inflammation. Although further research is needed to understand how age and PD affect NK cell number and functions, these results may illuminate pathways to better treatments for PD.

C1 [Earls, Rachael H.; Lee, Jae-Kyung] Univ Georgia, Coll Vet Med, Dept Physiol & Pharmacol, Athens, GA 30602 USA.

C3 University System of Georgia; University of Georgia

RP Lee, JK (corresponding author), Univ Georgia, Coll Vet Med, Dept Physiol & Pharmacol, Athens, GA 30602 USA.

EM jamlee@uga.edu

OI LEE, JAE-KYUNG/0000-0003-0104-8623

FU Department of Physiology and Pharmacology at the University of Georgia;

Michael J. Fox Foundation Target Advancement Grant

FX This work was supported by startup funds from the Department of

Physiology and Pharmacology at the University of Georgia and the Michael

J. Fox Foundation Target Advancement Grant.

CR Abel AM, 2018, FRONT IMMUNOL, V9, DOI 10.3389/fimmu.2018.01869

Anfossi N, 2006, IMMUNITY, V25, P331, DOI 10.1016/j.immuni.2006.06.013

Angelova DM, 2018, BIOMOLECULES, V8, DOI 10.3390/biom8030067

Antonangeli F, 2019, J LEUKOCYTE BIOL, V105, P1275, DOI 10.1002/JLB.MR0718-299R

Ashkar AA, 2009, INFECT IMMUN, V77, P214, DOI 10.1128/IAI.01066-08

Backström E, 2000, J IMMUNOL, V165, P4895, DOI 10.4049/jimmunol.165.9.4895

Béraud D, 2013, J NEUROIMMUNE PHARM, V8, P94, DOI 10.1007/s11481-012-9401-0

BOKOR M, 1993, J NEUROL SCI, V115, P47, DOI 10.1016/0022-510X(93)90065-7

Bowen KE, 2018, PLOS ONE, V13, DOI 10.1371/journal.pone.0193008

Braak H, 2006, NEUROSCI LETT, V396, P67, DOI 10.1016/j.neulet.2005.11.012

Braak H, 2003, J NEURAL TRANSM, V110, P517, DOI 10.1007/s00702-002-0808-2

Bregenholt S, 2001, J IMMUNOL, V166, P1871, DOI 10.4049/jimmunol.166.3.1871

BURKHARDT JK, 1990, J CELL BIOL, V111, P2327, DOI 10.1083/jcb.111.6.2327

Burré J, 2015, J PARKINSON DIS, V5, P699, DOI 10.3233/JPD-150642

Burre J, 2010, SCIENCE, V329, P1663, DOI 10.1126/science.1195227

Carapito R, 2015, IMMUNOL REV, V267, P88, DOI 10.1111/imr.12328

Hernández-Romero MC, 2012, NEUROTOXICOLOGY, V33, P347, DOI 10.1016/j.neuro.2012.01.018

Cebrián C, 2014, NAT COMMUN, V5, DOI 10.1038/ncomms4633

Cen L, 2017, NEUROSCI BULL, V33, P493, DOI 10.1007/s12264-017-0163-9

Cerboni C, 2007, BLOOD, V110, P606, DOI 10.1182/blood-2006-10-052720

Cerwenka A, 2001, NAT REV IMMUNOL, V1, P41, DOI 10.1038/35095564

Cheent K, 2009, IMMUNOLOGY, V126, P449, DOI 10.1111/j.1365-2567.2009.03045.x

Clairembault T, 2015, ACTA NEUROPATHOL COM, V3, DOI 10.1186/s40478-015-0196-0

Coppé JP, 2010, ANNU REV PATHOL-MECH, V5, P99, DOI 10.1146/annurev-pathol-121808-102144

Crouse J, 2015, TRENDS IMMUNOL, V36, P49, DOI 10.1016/j.it.2014.11.001

Cunningham C, 2005, J NEUROSCI, V25, P9275, DOI 10.1523/JNEUROSCI.2614-05.2005

Davies AJ, 2019, CELL, V176, P716, DOI 10.1016/j.cell.2018.12.022

Devos D, 2013, NEUROBIOL DIS, V50, P42, DOI 10.1016/j.nbd.2012.09.007

Di Lorenzo G, 1999, MECH AGEING DEV, V108, P25, DOI 10.1016/S0047-6374(98)00156-0

Di Santo JP, 2006, ANNU REV IMMUNOL, V24, P257, DOI 10.1146/annurev.immunol.24.021605.090700

Dumitriu A, 2016, BMC MED GENOMICS, V9, DOI 10.1186/s12920-016-0164-y

Earls RH, 2020, P NATL ACAD SCI USA, V117, P1762, DOI 10.1073/pnas.1909110117

Earls RH, 2019, J NEUROINFLAMM, V16, DOI 10.1186/s12974-019-1636-8

Edelblum KL, 2009, CURR OPIN PHARMACOL, V9, P715, DOI 10.1016/j.coph.2009.06.022

El-Agnaf OMA, 2006, FASEB J, V20, P419, DOI 10.1096/fj.03-1449com

Fellner L, 2013, GLIA, V61, P349, DOI 10.1002/glia.22437

Ferreira SA, 2018, FRONT CELL NEUROSCI, V12, DOI 10.3389/fncel.2018.00247

Forsyth CB, 2011, PLOS ONE, V6, DOI 10.1371/journal.pone.0028032

Foulds PG, 2013, SCI REP-UK, V3, DOI 10.1038/srep02540

Gao HM, 2008, TRENDS IMMUNOL, V29, P357, DOI 10.1016/j.it.2008.05.002

Garofalo S, 2020, NAT COMMUN, V11, DOI 10.1038/s41467-020-15644-8

Gasser S, 2006, IMMUNOL REV, V214, P130, DOI 10.1111/j.1600-065X.2006.00460.x

Gruden MA, 2011, J NEUROIMMUNOL, V233, P221, DOI 10.1016/j.jneuroim.2010.12.001

Guillerey C, 2016, NAT IMMUNOL, V17, P1025, DOI 10.1038/ni.3518

Hall LJ, 2013, INFECT IMMUN, V81, P460, DOI 10.1128/IAI.00953-12

Halonen SK, 2006, J NEUROIMMUNOL, V175, P19, DOI 10.1016/j.jneuroim.2006.02.015

Hamza TH, 2010, NAT GENET, V42, P781, DOI 10.1038/ng.642

Han S, 2014, J IMMUNOL, V192, P2551, DOI 10.4049/jimmunol.1302884

Hao JW, 2010, J EXP MED, V207, P1907, DOI 10.1084/jem.20092749

Hazeldine J, 2013, AGEING RES REV, V12, P1069, DOI 10.1016/j.arr.2013.04.003

Hazeldine J, 2012, AGING CELL, V11, P751, DOI 10.1111/j.1474-9726.2012.00839.x

Hazenberg MD, 2014, BLOOD, V124, P700, DOI 10.1182/blood-2013-11-427781

Houser MC, 2017, NPJ PARKINSONS DIS, V3, DOI 10.1038/s41531-016-0002-0

Huang D, 2006, FASEB J, V20, P896, DOI 10.1096/fj.05-5465com

Jiang S, 2017, NEUROL SCI, V38, P1373, DOI 10.1007/s10072-017-2988-4

Jiang W, 2017, P NATL ACAD SCI USA, V114, pE6202, DOI 10.1073/pnas.1705491114

KARRE K, 1986, NATURE, V319, P675, DOI 10.1038/319675a0

KIM C, 2013, NAT COMMUN, V4

Kim WS, 2014, ALZHEIMERS RES THER, V6, DOI 10.1186/s13195-014-0073-2

Krishnaraj R, 1996, IMMUNOL LETT, V50, P59, DOI 10.1016/0165-2478(96)02519-9

Kritsilis M, 2018, INT J MOL SCI, V19, DOI 10.3390/ijms19102937

Kustrimovic N, 2018, J NEUROINFLAMM, V15, DOI 10.1186/s12974-018-1248-8

Lanier LL, 1997, IMMUNOL REV, V155, P145, DOI 10.1111/j.1600-065X.1997.tb00947.x

Lau A, 2020, NAT NEUROSCI, V23, P21, DOI 10.1038/s41593-019-0541-x

Lee HJ, 2008, BIOCHEM BIOPH RES CO, V372, P423, DOI 10.1016/j.bbrc.2008.05.045

Lee MK, 2002, P NATL ACAD SCI USA, V99, P8968, DOI 10.1073/pnas.132197599

Lees AJ, 2009, LANCET, V373, P2055, DOI 10.1016/S0140-6736(09)60492-X

Lettau M, 2007, IMMUNOL LETT, V108, P10, DOI 10.1016/j.imlet.2006.10.001

Li QY, 2019, NEURON, V101, P207, DOI 10.1016/j.neuron.2018.12.006

Liu Q, 2016, NAT NEUROSCI, V19, P243, DOI 10.1038/nn.4211

Loria F, 2017, ACTA NEUROPATHOL, V134, P789, DOI 10.1007/s00401-017-1746-2

Lutz CT, 2011, J IMMUNOL, V186, P4590, DOI 10.4049/jimmunol.1002732

Lutz CT, 2005, MECH AGEING DEV, V126, P722, DOI 10.1016/j.mad.2005.01.004

Malek N, 2014, ACTA NEUROL SCAND, V130, P59, DOI 10.1111/ane.12247

Marcenaro E, 2017, FRONT IMMUNOL, V8, DOI 10.3389/fimmu.2017.01363

Marras C, 2018, NPJ PARKINSONS DIS, V4, DOI 10.1038/s41531-018-0058-0

Masilamani M, 2009, TRAFFIC, V10, P1735, DOI 10.1111/j.1600-0854.2009.00973.x

Masliah E, 2000, SCIENCE, V287, P1265, DOI 10.1126/science.287.5456.1265

Massey A. R., 2020, BIORXIV, DOI 10.1101/2020.04.25.061762

Mihara T, 2008, PARKINSONISM RELAT D, V14, P46, DOI 10.1016/j.parkreldis.2007.05.013

Mikulak J, 2017, FRONT IMMUNOL, V8, DOI 10.3389/fimmu.2017.00314

Moretta A, 2001, ANNU REV IMMUNOL, V19, P197, DOI 10.1146/annurev.immunol.19.1.197

Morse RHA, 2001, J NEUROIMMUNOL, V116, P107, DOI 10.1016/S0165-5728(01)00289-2

Mount MP, 2007, J NEUROSCI, V27, P3328, DOI 10.1523/JNEUROSCI.5321-06.2007

Naper C, 2011, J INNATE IMMUN, V3, P289, DOI 10.1159/000324143

Nedvetzki S, 2007, BLOOD, V109, P3776, DOI 10.1182/blood-2006-10-052977

Nikzad R, 2019, SCI IMMUNOL, V4, DOI 10.1126/sciimmunol.aat8116

Niwa F, 2012, GERIATR GERONTOL INT, V12, P102, DOI 10.1111/j.1447-0594.2011.00740.x

OTTUM PA, 2015, FRONT IMMUNOL, V6

Paolini R, 2015, CYTOKINE GROWTH F R, V26, P113, DOI 10.1016/j.cytogfr.2014.11.003

Phillips RJ, 2013, ANAT REC, V296, P654, DOI 10.1002/ar.22675

Poewe W, 2017, NAT REV DIS PRIMERS, V3, DOI 10.1038/nrdp.2017.13

Poggi A, 2019, FRONT IMMUNOL, V10, DOI 10.3389/fimmu.2019.00961

Poli A, 2009, IMMUNOLOGY, V126, P458, DOI 10.1111/j.1365-2567.2008.03027.x

Rabinovich BA, 2003, J IMMUNOL, V170, P3572, DOI 10.4049/jimmunol.170.7.3572

Ramesh G., 2013, Mediators of inflammation, V2013

Rannikko EH, 2015, BMC NEUROSCI, V16, DOI 10.1186/s12868-015-0192-0

Raulet DH, 2006, SEMIN IMMUNOL, V18, P145, DOI 10.1016/j.smim.2006.03.003

Raulet DH, 2006, NAT REV IMMUNOL, V6, P520, DOI 10.1038/nri1863

Ravetch JV, 2000, SCIENCE, V290, P84, DOI 10.1126/science.290.5489.84

Reish HEA, 2015, J PARKINSON DIS, V5, P1, DOI 10.3233/JPD-140491

Richfield EK, 2002, EXP NEUROL, V175, P35, DOI 10.1006/exnr.2002.7882

Romee R, 2014, SCIENTIFICA, V2014, DOI 10.1155/2014/205796

Rostami J, 2017, J NEUROSCI, V37, P11835, DOI 10.1523/JNEUROSCI.0983-17.2017

Salat-Foix D, 2012, CAN J NEUROL SCI, V39, P185, DOI 10.1017/S0317167100013202

Schmidt RL, 2011, PLOS PATHOG, V7, DOI 10.1371/journal.ppat.1002368

Schoenborn JR, 2007, ADV IMMUNOL, V96, P41, DOI 10.1016/S0065-2776(07)96002-2

Shalash A, 2017, FRONT NEUROL, V8, DOI 10.3389/fneur.2017.00720

Shi FD, 2011, NAT REV IMMUNOL, V11, P658, DOI 10.1038/nri3065

Small CL, 2008, J IMMUNOL, V180, P5558, DOI 10.4049/jimmunol.180.8.5558

Sotolongo J, 2011, J EXP MED, V208, P2705, DOI 10.1084/jem.20110547

Su XM, 2008, NEUROBIOL AGING, V29, P1690, DOI 10.1016/j.neurobiolaging.2007.04.006

Su XM, 2009, NEUROTOX RES, V16, P238, DOI 10.1007/s12640-009-9053-x

Sulzer D, 2007, TRENDS NEUROSCI, V30, P244, DOI 10.1016/j.tins.2007.03.009

Sulzer D, 2017, NATURE, V546, P656, DOI 10.1038/nature22815

Thorén FB, 2012, J IMMUNOL, V188, P1668, DOI 10.4049/jimmunol.1102002

Topham NJ, 2009, IMMUNOLOGY, V128, P7, DOI 10.1111/j.1365-2567.2009.03123.x

Tremblay-McLean A, 2019, BMC IMMUNOL, V20, DOI 10.1186/s12865-018-0272-x

Van Hove H, 2019, NAT NEUROSCI, V22, P1021, DOI 10.1038/s41593-019-0393-4

Vivier E, 2008, NAT IMMUNOL, V9, P503, DOI 10.1038/ni1582

VOORTHUIS JAC, 1990, CLIN EXP IMMUNOL, V81, P183

Waggoner SN, 2012, FRONT IMMUNOL, V3, DOI 10.3389/fimmu.2012.00377

Wakabayashi K, 2000, ACTA NEUROPATHOL, V99, P14, DOI 10.1007/PL00007400

White AJ, 2018, FRONT NEUROL, V9, DOI 10.3389/fneur.2018.00946

Williams-Gray CH, 2016, MOVEMENT DISORD, V31, P995, DOI 10.1002/mds.26563

Xu W, 2005, J NEUROIMMUNOL, V163, P24, DOI 10.1016/j.jneuroim.2005.02.011

Zamora Anthony E, 2015, Curr Protoc Immunol, V110, DOI 10.1002/0471142735.im1437s110

Zella MAS, 2019, CELLS-BASEL, V8, DOI 10.3390/cells8020105

Zhang BN, 1997, J EXP MED, V186, P1677, DOI 10.1084/jem.186.10.1677

Zhang QS, 2017, TOXICOL LETT, V265, P30, DOI 10.1016/j.toxlet.2016.11.002

Zhang W, 2005, FASEB J, V19, P533, DOI 10.1096/fj.04-2751com

Zhang W, 2007, GLIA, V55, P1178, DOI 10.1002/glia.20532

NR 132

TC 38

Z9 41

U1 0

U2 10

PU SPRINGERNATURE

PI LONDON

PA CAMPUS, 4 CRINAN ST, LONDON, N1 9XW, United Kingdom

SN 1226-3613

EI 2092-6413

J9 EXP MOL MED

JI Exp. Mol. Med.

PD SEP

PY 2020

VL 52

IS 9

BP 1517

EP 1525

DI 10.1038/s12276-020-00505-7

EA SEP 2020

PG 9

WC Biochemistry & Molecular Biology; Medicine, Research & Experimental

WE Science Citation Index Expanded (SCI-EXPANDED)

SC Biochemistry & Molecular Biology; Research & Experimental Medicine

GA OB9LX

UT WOS:000572585700001

PM 32973221

OA gold, Green Published

DA 2024-08-12

ER

PT J

AU Di Stadio, A

De Luca, P

Koohi, N

Kaski, D

Ralli, M

Giesemann, A

Hartung, HP

Altieri, M

Messineo, D

Warnecke, A

Frohman, T

Frohman, EM

AF Di Stadio, Arianna

De Luca, Pietro

Koohi, Nehzat

Kaski, Diego

Ralli, Massimo

Giesemann, Anja

Hartung, Hans-Peter

Altieri, Marta

Messineo, Daniela

Warnecke, Athanasia

Frohman, Teresa

Frohman, Elliot M.

TI Neuroinflammatory disorders of the brain and inner ear: a systematic

review of auditory function in patients with migraine, multiple

sclerosis, and neurodegeneration to support the idea of an innovative

'window of discovery'

SO FRONTIERS IN NEUROLOGY

LA English

DT Review

DE inner ear; neuroinflammation; CSF; Parkinson's disease; multiple

sclerosis; headache

ID HEARING-LOSS; COMMUNICATION; PERILYMPH; MARKERS

AB Background: Hearing can be impaired in many neurological conditions and can even represent a forme fruste of specific disorders. Auditory function can be measured by either subjective or objective tests. Objective tests are more useful in identifying which auditory pathway (superior or inferior) is most affected by disease. The inner ear's perilymphatic fluid communicates with the cerebrospinal fluid (CSF) via the cochlear aqueduct representing a window from which pathological changes in the contents of the CSF due to brain inflammation could, therefore, spread to and cause inflammation in the inner ear, damaging inner hair cells and leading to hearing impairment identifiable on tests of auditory function.

Methods: A systematic review of the literature was performed, searching for papers with case-control studies that analyzed the hearing and migraine function in patients with neuro-inflammatory, neurodegenerative disorders. With data extracted from these papers, the risk of patients with neurological distortion product otoacoustic emission (DPOAE) was then calculated.

Results: Patients with neurological disorders (headache, Parkinson's disease, and multiple sclerosis) had a higher risk of having peripheral auditory deficits when compared to healthy individuals.

Conclusion: Existing data lend credence to the hypothesis that inflammatory mediators transmitted via fluid exchange across this communication window, thereby represents a key pathobiological mechanism capable of culminating in hearing disturbances associated with neuroimmunological and neuroinflammatory disorders of the nervous system.

C1 [Di Stadio, Arianna] Univ Catania, GF Ingrassia Dept, Catania, Italy.

[Di Stadio, Arianna] IRCCS Santa Lucia, I-00179 Rome, Italy.

[De Luca, Pietro] San Giovanni Addolorata Hosp, Head & Neck Dept, Rome, Italy.

[Koohi, Nehzat; Kaski, Diego] UCL Queen Sq Inst Neurol, London, United Kingdom.

[Ralli, Massimo] Univ Sapienza, Dept Sense Organs, Rome, Italy.

[Giesemann, Anja] Hannover Med Sch, Dept Intervent Neuroradiol, Hannover, Germany.

[Hartung, Hans-Peter] Klin Neurol UKD Heinrich Heine Univ Dusseldorf, Dusseldorf, Germany.

[Altieri, Marta] Univ Sapienza, Dept Neurol, Rome, Italy.

[Messineo, Daniela] Univ Sapienza, Dept Radiol & Pathol, Rome, Italy.

[Warnecke, Athanasia] Hannover Med Sch, Dept Otolaryngol Head & Neck Surg, Hannover, Germany.

[Frohman, Teresa; Frohman, Elliot M.] Stanford Univ, Lab Neuroimmunol, Sch Med, Palo Alto, CA 94305 USA.

C3 University of Catania; IRCCS Santa Lucia; Azienda Ospedaliera San

Giovanni Addolorata; University of London; University College London;

Sapienza University Rome; Hannover Medical School; Sapienza University

Rome; Sapienza University Rome; Hannover Medical School; Stanford

University

RP Di Stadio, A (corresponding author), Univ Catania, GF Ingrassia Dept, Catania, Italy.; Di Stadio, A (corresponding author), IRCCS Santa Lucia, I-00179 Rome, Italy.

EM ariannadistadio@hotmail.com

RI Warnecke, Athanasia/HHC-6174-2022; Ralli, Massimo/E-1780-2018; MESSINEO,

DANIELA/AAP-8097-2020

OI Warnecke, Athanasia/0000-0001-9652-1035; MESSINEO,

DANIELA/0000-0003-3059-4439; Koohi, Nehzat/0000-0003-1545-8527

FU Santa Lucia Research Hospital of Rome

FX This study was supported by current research funds of Santa Lucia

Research Hospital of Rome.

CR Albanese M, 2021, J CLIN MED, V10, DOI 10.3390/jcm10215007

Albrecht DS, 2019, NEUROLOGY, V92, pE2038, DOI 10.1212/WNL.0000000000007371

Atula S, 2016, Mult Scler J Exp Transl Clin, V2, p2055217316652155, DOI 10.1177/2055217316652155

Bakhos D, 2017, EUR ANN OTORHINOLARY, V134, P325, DOI 10.1016/j.anorl.2017.02.011

Bjelobaba I, 2017, CURR PHARM DESIGN, V23, P693, DOI 10.2174/1381612822666161214153108

Brotini S, 2017, CNS NEUROL DISORD-DR, V16, P705, DOI 10.2174/1871527316666170321124949

Cameron L., 2014, J OTOL, V9, P169, DOI [10.1016/j.joto.2015.03.001, DOI 10.1016/J.JOTO.2015.03.001]

Cruz RA, 2022, J NEUROL, V269, P2792, DOI 10.1007/s00415-021-10915-w

Di Mauro R, 2019, MULT SCLER RELAT DIS, V33, P55, DOI 10.1016/j.msard.2019.05.020

Di Stadio A, 2018, EUR REV MED PHARMACO, V22, P4611, DOI 10.26355/eurrev_201807_15520

Di Stadio A, 2021, NEUROL SCI, V42, P579, DOI 10.1007/s10072-020-04948-8

Di Stadio A, 2020, MULT SCLER RELAT DIS, V45, DOI 10.1016/j.msard.2020.102431

Di Stadio A, 2020, NEUROL SCI, V41, P1891, DOI 10.1007/s10072-020-04295-8

Di Stadio A, 2019, MITOCHONDRION, V46, P334, DOI 10.1016/j.mito.2018.09.003

Di Stadio A, 2018, MULT SCLER J, V24, P1264, DOI 10.1177/1352458517750010

Doherty JK, 2004, OTOL NEUROTOL, V25, P457, DOI 10.1097/00129492-200407000-00010

Frohman EM, 2008, NAT CLIN PRACT NEURO, V4, P664, DOI 10.1038/ncpneuro0950

Frye MD, 2019, J ACOUST SOC AM, V146, P4020, DOI 10.1121/1.5132545

Gopen Q, 1997, HEARING RES, V107, P9, DOI 10.1016/S0378-5955(97)00017-8

Grigoriadis N, 2006, CLIN NEUROL NEUROSUR, V108, P241, DOI 10.1016/j.clineuro.2005.11.006

Grotemeyer A, 2022, FRONT IMMUNOL, V13, DOI 10.3389/fimmu.2022.878771

Guo SR, 2022, FRONT AGING NEUROSCI, V14, DOI 10.3389/fnagi.2022.815347

Gür E, 2022, BMJ OPEN, V12, DOI 10.1136/bmjopen-2021-060540

Hirsch Etienne C, 2012, Parkinsonism Relat Disord, V18 Suppl 1, pS210, DOI 10.1016/S1353-8020(11)70065-7

Karikó K, 2004, J CEREBR BLOOD F MET, V24, P1288, DOI 10.1097/01.WCB.0000145666.68576.71

Kim R, 2018, J CLIN NEUROSCI, V58, P30, DOI 10.1016/j.jocn.2018.10.079

Kothur K, 2016, CYTOKINE, V77, P227, DOI 10.1016/j.cyto.2015.10.001

Leng FD, 2021, NAT REV NEUROL, V17, P157, DOI 10.1038/s41582-020-00435-y

Liu Y, 2020, NEUROSCI LETT, V717, DOI 10.1016/j.neulet.2019.134705

Lo Sasso B, 2019, MEDICINA-LITHUANIA, V55, DOI 10.3390/medicina55060245

Lopes Marcia da Silva, 2018, Rev. CEFAC, V20, P573

MacMahon H, 2022, IRISH J MED SCI, V191, P391, DOI 10.1007/s11845-021-02508-3

Magnusen AF, 2021, FRONT NEUROL, V12, DOI 10.3389/fneur.2021.636139

Mokbel K, 2014, J INT ADV OTOL, V10, P150, DOI 10.5152/iao.2014.46

Musiek FE, 2017, J AM ACAD AUDIOL, V28, P655, DOI 10.3766/jneurodegenerative disease.16061

Naganawa S, 2008, RADIAT MED, V26, P597, DOI 10.1007/s11604-008-0286-z

National Heart Lung and Blood Institute, 2014, Study quality assessment tools

Ng HS, 2022, NEUROL-NEUROIMMUNOL, V9, DOI 10.1212/NXI.0000000000200005

O'Malley JT, 2016, OTOL NEUROTOL, V37, P99, DOI 10.1097/MAO.0000000000000879

Osman S, 2022, DIAGN INTERV IMAG, V103, P225, DOI 10.1016/j.diii.2021.10.003

Pisani V, 2015, PARKINSONISM RELAT D, V21, P987, DOI 10.1016/j.parkreldis.2015.06.007

Ralli M, 2019, AM J OTOLARYNG, V40, P1, DOI 10.1016/j.amjoto.2018.09.012

Saberi Alia, 2012, Acta Med Iran, V50, P679

Salt AN, 2018, HEARING RES, V362, P25, DOI 10.1016/j.heares.2017.12.010

Salt AN, 2012, JARO-J ASSOC RES OTO, V13, P771, DOI 10.1007/s10162-012-0347-y

Salvi R, 2018, HEARING BALANC COMMU, V16, P74, DOI 10.1080/21695717.2018.1442282

Shen H, 2015, J HEADACHE PAIN, V16, DOI 10.1186/s10194-015-0557-x

Sisto R, 2020, BRAIN COMMUN, V2, DOI 10.1093/braincomms/fcaa144

Tansey MG, 2022, NAT REV IMMUNOL, V22, P657, DOI 10.1038/s41577-022-00684-6

Warchol ME, 2019, CSH PERSPECT MED, V9, DOI 10.1101/cshperspect.a033555

Warnecke A, 2019, FRONT NEUROL, V10, DOI 10.3389/fneur.2019.00665

Xue JF, 2020, AUDIOL NEURO-OTOL, V25, P209, DOI 10.1159/000506147

Yu WT, 2021, FRONT NEUROSCI-SWITZ, V15, DOI 10.3389/fnins.2021.774585

Zhang XW, 2020, BIOCHEM PHARMACOL, V177, DOI 10.1016/j.bcp.2020.113932

NR 54

TC 1

Z9 1

U1 1

U2 1

PU FRONTIERS MEDIA SA

PI LAUSANNE

PA AVENUE DU TRIBUNAL FEDERAL 34, LAUSANNE, CH-1015, SWITZERLAND

SN 1664-2295

J9 FRONT NEUROL

JI Front. Neurol.

PD AUG 10

PY 2023

VL 14

AR 1204132

DI 10.3389/fneur.2023.1204132

PG 11

WC Clinical Neurology; Neurosciences

WE Science Citation Index Expanded (SCI-EXPANDED)

SC Neurosciences & Neurology

GA Q6NT5

UT WOS:001058679100001

PM 37662038

OA Green Published, gold

DA 2024-08-12

ER

PT J

AU Ferini-Strambi, L

Salsone, M

AF Ferini-Strambi, Luigi

Salsone, Maria

TI COVID-19 and neurological disorders: are neurodegenerative or

neuroimmunological diseases more vulnerable?

SO JOURNAL OF NEUROLOGY

LA English

DT Review

DE COVID-19 infection; Parkinson's disease; Alzheimer's disease; Multiple

sclerosis

ID MULTIPLE-SCLEROSIS; parkinson's disease; CORONAVIRUS; INFLAMMATION;

SYMPTOMS; MICE

AB Neurological disorders and coronavirus 2019 (COVID-19) pandemic are two conditions with a recent well-documented association. Intriguing evidences showed that COVID-19 infection can modify clinical spectrum of manifested neurological disorders but also it plays a crucial role in the development of future diseases as long-tem consequences. In this viewpoint review, we aimed to assess the vulnerability to SARS-CoV-2 infection and development of COVID-19 among neurological disorders. With this in mind, we tested the hypothesis that age rather than neuropathology itself could be decisive in neurodegenerative disease such as Parkinson's disease, whereas neuropathology rather than age may be critical in neuroimmunological diseases such as Multiple Sclerosis. Highlighting the role of potential susceptibility or protection factors from this disastrous infection, we also stratify the risk for future neurodegeneration.

C1 [Ferini-Strambi, Luigi] Univ Vita Salute San Raffaele, Milan, Italy.

[Ferini-Strambi, Luigi; Salsone, Maria] Ist Sci San Raffaele, Div Neurosci, Sleep Disorders Ctr, Milan, Italy.

[Salsone, Maria] CNR, Inst Mol Bioimaging & Physiol, Catanzaro, Italy.

C3 Vita-Salute San Raffaele University; Vita-Salute San Raffaele

University; IRCCS Ospedale San Raffaele; Consiglio Nazionale delle

Ricerche (CNR); Istituto di Bioimmagini e Fisiologia Molecolare

(IBFM-CNR)

RP Ferini-Strambi, L (corresponding author), Univ Vita Salute San Raffaele, Milan, Italy.; Ferini-Strambi, L (corresponding author), Ist Sci San Raffaele, Div Neurosci, Sleep Disorders Ctr, Milan, Italy.

EM ferinistrambi.luigi@hsr.it

RI Salsone, Maria/K-1045-2018

OI Salsone, Maria/0000-0001-5375-3733

CR Akhoundi FH, 2020, MULT SCLER RELAT DIS, V41, DOI 10.1016/j.msard.2020.102164

Antonini A, 2020, MOVEMENT DISORD, V35, P905, DOI 10.1002/mds.28104

Austin SA, 2011, NEUROCHEM RES, V36, P994, DOI 10.1007/s11064-011-0439-9

Baker D, 2020, MULT SCLER RELAT DIS, V43, DOI 10.1016/j.msard.2020.102174

Bauer K, 2014, BMC GERIATR, V14, DOI 10.1186/1471-2318-14-10

Berger JR, 2020, NEUROL-NEUROIMMUNOL, V7, DOI 10.1212/NXI.0000000000000761

Bianchetti A, 2020, J NUTR HEALTH AGING, V24, P560, DOI 10.1007/s12603-020-1389-1

Bonavita S, 2020, NEUROL SCI, V41, P1007, DOI 10.1007/s10072-020-04391-9

Boutoleau-Bretonnière C, 2020, J ALZHEIMERS DIS, V76, P41, DOI 10.3233/JAD-200604

Braak H, 2003, NEUROBIOL AGING, V24, P197, DOI 10.1016/S0197-4580(02)00065-9

Brown EE, 2020, AM J GERIAT PSYCHIAT, V28, P712, DOI 10.1016/j.jagp.2020.04.010

Butler MJ, 2020, BRAIN BEHAV IMMUN, V87, P53, DOI 10.1016/j.bbi.2020.04.040

Chen XG, 2020, GLOB HEALTH RES POL, V5, DOI [10.1186/s41256-020-00137-4, 10.1186/s41256-020-00142-7]

Cilia R, 2020, MOVEMENT DISORD, V35, P1287, DOI 10.1002/mds.28170

Corman VM, 2018, ADV VIRUS RES, V100, P163, DOI 10.1016/bs.aivir.2018.01.001

Covino M, 2020, GERIATR GERONTOL INT, V20, P704, DOI 10.1111/ggi.13960

Cubo E, 2020, J PARKINSON DIS, V10, P911, DOI 10.3233/JPD-202108

Cuffaro L, 2020, NEUROL SCI, V41, P1977, DOI 10.1007/s10072-020-04512-4

Doobay MF, 2007, AM J PHYSIOL-REG I, V292, pR373, DOI 10.1152/ajpregu.00292.2006

Fan ML, 2020, NEUROL-NEUROIMMUNOL, V7, DOI 10.1212/NXI.0000000000000787

Fasano A, 2020, MOV DISORD CLIN PRAC, V7, P361, DOI 10.1002/mdc3.12965

FAZZINI E, 1992, MOVEMENT DISORD, V7, P153, DOI 10.1002/mds.870070210

Fox NC, 2013, LANCET, V382, P1968, DOI 10.1016/S0140-6736(13)62426-5

Frischer JM, 2009, BRAIN, V132, P1175, DOI 10.1093/brain/awp070

Goetz CG, 2020, MOVEMENT DISORD, V35, P911, DOI 10.1002/mds.28094

Gomez-Pinedo U, 2020, J MED VIROL, V92, P2354, DOI 10.1002/jmv.26046

Hartung HP, 2020, NAT REV NEUROL, V16, P347, DOI 10.1038/s41582-020-0368-9

Hatcher-Martin JM, 2020, NEUROLOGY, V94, P30, DOI 10.1212/WNL.0000000000008708

Helmich RC, 2020, J PARKINSON DIS, V10, P351, DOI 10.3233/JPD-202038

Helmich RC, 2009, J NEUROSCI, V29, P6105, DOI 10.1523/JNEUROSCI.0704-09.2009

Isaia G, 2020, AM J GERIAT PSYCHIAT, V28, P790, DOI [10.1016/j.archger.2004.04.022, 10.1016/j.jagp.2020.04.018]

Jo T, 2018, PARKINSONISM RELAT D, V54, P25, DOI 10.1016/j.parkreldis.2018.03.028

Lanctot Krista L, 2017, Alzheimers Dement (N Y), V3, P440, DOI 10.1016/j.trci.2017.07.001

Lara B, 2020, EUR J NEUROL, V27, P1744, DOI 10.1111/ene.14339

Leocani L, 2020, EUR J NEUROL, V27, pE50, DOI 10.1111/ene.14320

Leung C, 2020, MECH AGEING DEV, V188, DOI 10.1016/j.mad.2020.111255

Li K, 2016, J INFECT DIS, V213, P712, DOI 10.1093/infdis/jiv499

Li YC, 2020, J MED VIROL, V92, P552, DOI 10.1002/jmv.25728

Lippi A, 2020, MOVEMENT DISORD, V35, P716, DOI 10.1002/mds.28084

Liu K, 2020, J INFECTION, V80, pE14, DOI 10.1016/j.jinf.2020.03.005

Liu XD, 2012, J BIOL CHEM, V287, P19687, DOI 10.1074/jbc.M112.350934

Louapre C, 2020, JAMA NEUROL, V77, P1079, DOI 10.1001/jamaneurol.2020.2581

Luna G, 2020, JAMA NEUROL, V77, P184, DOI 10.1001/jamaneurol.2019.3365

Lyketsos CG, 2001, INT J GERIATR PSYCH, V16, P1043, DOI 10.1002/gps.448

Maghzi AH, 2020, J NEUROL, V267, P2790, DOI 10.1007/s00415-020-09944-8

Mansoor S, 2020, EGYPT J NEUROL PSYCH, V56, DOI 10.1186/s41983-020-00177-0

Marcantonio ER, 2017, NEW ENGL J MED, V377, P1456, DOI 10.1056/NEJMcp1605501

Marreiros R, 2020, P NATL ACAD SCI USA, V117, P6741, DOI 10.1073/pnas.1906466117

Matías-Guiu J, 2020, NEUROLOGIA, V35, P170, DOI 10.1016/j.nrl.2020.03.001

Naughton SX, 2020, J ALZHEIMERS DIS, V76, P21, DOI 10.3233/JAD-200537

Onder G, 2020, JAMA-J AM MED ASSOC, V323, P1775, DOI 10.1001/jama.2020.4683

Papa SM, 2020, MOV DISORD CLIN PRAC, V7, P357, DOI 10.1002/mdc3.12953

Persson R, 2020, MULT SCLER RELAT DIS, V41, DOI 10.1016/j.msard.2020.101982

Prasad S, 2020, MOVEMENT DISORD, V35, P912, DOI 10.1002/mds.28088

Racke MK, 2020, J NEUROIMMUNOL, V345, DOI 10.1016/j.jneuroim.2020.577283

Robbins TW, 2014, MOVEMENT DISORD, V29, P597, DOI 10.1002/mds.25853

Rodriguez-Perez AI, 2019, BRAIN BEHAV IIMMUN, VS0889-1591, P31128

Salari M, 2020, MOVEMENT DISORD, V35, P1095, DOI 10.1002/mds.28116

Schenkman M, 2018, JAMA NEUROL, V75, P219, DOI 10.1001/jamaneurol.2017.3517

Shalash A, 2020, MOVEMENT DISORD, V35, P1097, DOI 10.1002/mds.28134

Shalhoub S, 2020, LANCET, V395, P1670, DOI 10.1016/S0140-6736(20)31101-6

Sormani MP, 2020, LANCET NEUROL, V19, P481, DOI 10.1016/S1474-4422(20)30147-2

Srivastav AK, 2020, PARKINSONISM RELAT D, V75, P128, DOI 10.1016/j.parkreldis.2020.05.021

Stoessl AJ, 2020, MOVEMENT DISORD, V35, P709, DOI 10.1002/mds.28069

Torres J, 2007, PROTEIN SCI, V16, P2065, DOI 10.1110/ps.062730007

Ulasli M, 2010, CELL MICROBIOL, V12, P844, DOI 10.1111/j.1462-5822.2010.01437.x

van der Kolk NM, 2019, LANCET NEUROL, V18, P998, DOI 10.1016/S1474-4422(19)30285-6

Wahmane SA, 2020, DISORD, DOI [10.1002/mds.28185, DOI 10.1002/MDS.28185]

Wang HL, 2020, LANCET, V395, P1190, DOI 10.1016/S0140-6736(20)30755-8

Wang L, 2020, J INFECTION, V80, P639, DOI 10.1016/j.jinf.2020.03.019

Wijnands JMA, 2018, J NEUROL NEUROSUR PS, V89, P1050, DOI 10.1136/jnnp-2017-317493

Willis MD, 2020, J NEUROL, V267, P1567, DOI 10.1007/s00415-020-09822-3

Wu F, 2020, NATURE, V579, P265, DOI 10.1038/s41586-020-2008-3

NR 73

TC 100

Z9 105

U1 0

U2 15

PU SPRINGER HEIDELBERG

PI HEIDELBERG

PA TIERGARTENSTRASSE 17, D-69121 HEIDELBERG, GERMANY

SN 0340-5354

EI 1432-1459

J9 J NEUROL

JI J. Neurol.

PD FEB

PY 2021

VL 268

IS 2

BP 409

EP 419

DI 10.1007/s00415-020-10070-8

EA JUL 2020

PG 11

WC Clinical Neurology

WE Science Citation Index Expanded (SCI-EXPANDED)

SC Neurosciences & Neurology

GA QG4EL

UT WOS:000551033000003

PM 32696341

OA hybrid, Green Published

DA 2024-08-12

ER

PT J

AU Mani, S

Sevanan, M

Krishnamoorthy, A

Sekar, S

AF Mani, Sugumar

Sevanan, Murugan

Krishnamoorthy, Alagudurai

Sekar, Sathiya

TI A systematic review of molecular approaches that link mitochondrial

dysfunction and neuroinflammation in Parkinson's disease

SO NEUROLOGICAL SCIENCES

LA English

DT Review

DE Parkinson's disease; Mitochondrial dysfunction; Neuroinflammation;

Mitochondrial proteins

ID OXIDATIVE STRESS; ALPHA-SYNUCLEIN; LIPID-PEROXIDATION; SUBSTANTIA-NIGRA;

4-HYDROXY-2-NONENAL-MODIFIED PROTEINS; DOPAMINERGIC-NEURONS; LEWY

BODIES; PINK1; NEURODEGENERATION; FUSION

AB Parkinson's disease (PD) is a chronic and progressive neurodegenerative disorder that affects 1% of the population worldwide. Etiology of PD is likely to be multi-factorial such as protein misfolding, mitochondrial dysfunction, oxidative stress, and neuroinflammation that contributes to the pathology of Parkinson's disease (PD), numerous studies have shown that mitochondrial dysfunction may play a key role in the dopaminergic neuronal loss. In multiple ways, the two most important are the activation of neuroinflammation and mitochondrial dysfunction, while mitochondrial dysfunction could cause neuroinflammation and vice versa. Thus, the mitochondrial proteins are the highly promising target for the development of PD. However, the limited amount of dopaminergic neurons prevented the detailed investigation of Parkinson's disease with regard to mitochondrial dysfunction. Both genetic and environmental factors are also associated with mitochondrial dysfunction and PD pathogenesis. The induction of PD by neurotoxins that inhibit mitochondrial complex I provide direct evidence linking mitochondrial dysfunction to PD. A decrease of mitochondrial complex I activity is observed in PD brain and in neurotoxin- or genetic factor-induced in vitro and in vivo models. Moreover, PINK1, Parkin, DJ-1 and LRRK2 mitochondrial PD gene products have important roles in mitophagy, a cellular process that clear damaged mitochondria. This review paper would discuss the evidence for the mitochondrial dysfunction and neuroinflammation in PD.

C1 [Mani, Sugumar; Krishnamoorthy, Alagudurai] Bharathiar Univ, Res & Dev Ctr, Coimbatore 641046, Tamil Nadu, India.

[Sevanan, Murugan] Karunya Inst Technol & Sci, Dept Biotechnol, Coimbatore 641114, Tamil Nadu, India.

[Sekar, Sathiya] Dr MGR Educ Res Inst, Dept Biotechnol, Chennai, Tamil Nadu, India.

C3 Bharathiar University; Karunya Institute of Technology & Sciences

RP Sevanan, M (corresponding author), Karunya Inst Technol & Sci, Dept Biotechnol, Coimbatore 641114, Tamil Nadu, India.

EM micromurugans@gmail.com

OI Sevanan, Murugan/0000-0002-2432-1883

CR Abrams AJ, 2011, BIOCHEMISTRY-US, V50, P3405, DOI 10.1021/bi200266v

Al-kuraishy HM, 2020, BASIC CLIN NEUROSCI, V11, P423, DOI 10.32598/bcn.11.4.1097.1

Al-Kuraishy HM, 2020, BRAIN CIRC, V6, P1, DOI 10.4103/bc.bc_46_19

Angeles DC, 2011, HUM MUTAT, V32, P1390, DOI 10.1002/humu.21582

Archer SL, 2013, NEW ENGL J MED, V369, P2236, DOI 10.1056/NEJMra1215233

Beal MF, 2002, FREE RADICAL BIO MED, V32, P797, DOI 10.1016/S0891-5849(02)00780-3

Beal MF, 2005, ANN NEUROL, V58, P495, DOI 10.1002/ana.20624

Bilbo SD, 2012, FRONT NEUROENDOCRIN, V33, P267, DOI 10.1016/j.yfrne.2012.08.006

Bjarnadóttir K, 2016, SCI REP-UK, V6, DOI 10.1038/srep34594

Björklund A, 2010, PROG BRAIN RES, V184, P113, DOI 10.1016/S0079-6123(10)84006-3

Blandini Fabio, 2008, Parkinsonism Relat Disord, V14 Suppl 2, pS124, DOI 10.1016/j.parkreldis.2008.04.015

Burté F, 2011, J PROTEOME RES, V10, P1974, DOI 10.1021/pr101211k

Castellani RJ, 2002, NEUROSCI LETT, V319, P25, DOI 10.1016/S0304-3940(01)02514-9

Castillo-Quan JI, 2011, DIS MODEL MECH, V4, P427, DOI 10.1242/dmm.008227

Cerveny KL, 2007, TRENDS CELL BIOL, V17, P563, DOI 10.1016/j.tcb.2007.08.006

Chao YX, 2009, J NEUROIMMUNOL, V216, P39, DOI 10.1016/j.jneuroim.2009.09.003

Chen HC, 2009, HUM MOL GENET, V18, pR169, DOI 10.1093/hmg/ddp326

Costa, 2014, VULNERABILITY COGNIT

Culbertson CT, 2014, ANAL CHEM, V86, P95, DOI 10.1021/ac403688g

Cunningham C, 2013, GLIA, V61, P71, DOI 10.1002/glia.22350

Dagda RK, 2009, J BIOL CHEM, V284, P13843, DOI 10.1074/jbc.M808515200

Deng HS, 2008, P NATL ACAD SCI USA, V105, P14503, DOI 10.1073/pnas.0803998105

Dexter DT, 2013, FREE RADICAL BIO MED, V62, P132, DOI 10.1016/j.freeradbiomed.2013.01.018

Dickinson DA, 2002, BIOCHEM PHARMACOL, V64, P1019, DOI 10.1016/S0006-2952(02)01172-3

ESTERBAUER H, 1991, FREE RADICAL BIO MED, V11, P81, DOI 10.1016/0891-5849(91)90192-6

Exner N, 2012, EMBO J, V31, P3038, DOI 10.1038/emboj.2012.170

Fujikake N, 2018, FRONT NEUROSCI-SWITZ, V12, DOI 10.3389/fnins.2018.00255

Gautier CA, 2008, P NATL ACAD SCI USA, V105, P11364, DOI 10.1073/pnas.0802076105

Geisler S, 2010, AUTOPHAGY, V6, P871, DOI 10.4161/auto.6.7.13286

Gu XL, 2010, MOL BRAIN, V3, DOI 10.1186/1756-6606-3-12

Hartley DP, 1997, CHEM RES TOXICOL, V10, P895, DOI 10.1021/tx960181b

Heeman B, 2011, J CELL SCI, V124, P1115, DOI 10.1242/jcs.078303

Henchcliffe C, 2008, NAT CLIN PRACT NEURO, V4, P600, DOI 10.1038/ncpneuro0924

Hong Z, 2010, BRAIN, V133, P713, DOI 10.1093/brain/awq008

Hsieh MH, 2012, BEHAV BRAIN RES, V229, P41, DOI 10.1016/j.bbr.2011.12.035

Hu QS, 2016, TRANSL NEURODEGENER, V5, DOI 10.1186/s40035-016-0060-6

Hu XM, 2008, J IMMUNOL, V181, P7194, DOI 10.4049/jimmunol.181.10.7194

Hyun DH, 2002, J NEUROCHEM, V83, P360, DOI 10.1046/j.1471-4159.2002.01125.x

Jomova K, 2010, MOL CELL BIOCHEM, V345, P91, DOI 10.1007/s11010-010-0563-x

Kang SS, 2009, CURR TOP MICROBIOL, V334, P227, DOI 10.1007/978-3-540-93864-4_10

Kawajiri S, 2011, TRENDS PHARMACOL SCI, V32, P573, DOI 10.1016/j.tips.2011.06.001

Kazlauskaite A, 2015, FEBS J, V282, P215, DOI 10.1111/febs.13127

Keeney PM, 2006, J NEUROSCI, V26, P5256, DOI 10.1523/JNEUROSCI.0984-06.2006

Kikuchi A, 2002, NEUROBIOL DIS, V9, P244, DOI 10.1006/nbdi.2002.0466

Kim Y, 2008, BIOCHEM BIOPH RES CO, V377, P975, DOI 10.1016/j.bbrc.2008.10.104

Kitada T, 1998, NATURE, V392, P605, DOI 10.1038/33416

Lin MT, 2006, NATURE, V443, P787, DOI 10.1038/nature05292

Lin X, 2012, J NEUROSCI, V32, P9248, DOI 10.1523/JNEUROSCI.1731-12.2012

Liu DX, 2011, NEUROPHARMACOLOGY, V61, P592, DOI 10.1016/j.neuropharm.2011.04.033

Lu M, 2016, INT J NEUROPSYCHOPH, V19, DOI 10.1093/ijnp/pyw047

Luk KC, 2012, SCIENCE, V338, P949, DOI 10.1126/science.1227157

Ma HY, 2018, PHARMACOGN MAG, V14, pS237, DOI 10.4103/pm.pm_418_17

Martens S, 2008, NAT REV MOL CELL BIO, V9, P543, DOI 10.1038/nrm2417

Masoud ST, 2015, NEUROBIOL DIS, V74, P66, DOI 10.1016/j.nbd.2014.10.016

Moehle MS, 2012, J NEUROSCI, V32, P1602, DOI 10.1523/JNEUROSCI.5601-11.2012

Mogi M, 2000, J NEURAL TRANSM, V107, P335, DOI 10.1007/s007020050028

Morris G, 2015, BMC MED, V13, DOI 10.1186/s12916-015-0310-y

Mosley RL, 2006, CLIN NEUROSCI RES, V6, P261, DOI 10.1016/j.cnr.2006.09.006

Nguyen TN, 2016, TRENDS CELL BIOL, V26, P733, DOI 10.1016/j.tcb.2016.05.008

Norris EH, 2007, AM J PATHOL, V170, P658, DOI 10.2353/ajpath.2007.060359

Paisán-Ruiz C, 2013, J PARKINSON DIS, V3, P85, DOI 10.3233/JPD-130192

Patel AS, 2015, PLOS ONE, V10, DOI 10.1371/journal.pone.0121246

Pickrell AM, 2015, NEURON, V85, P257, DOI 10.1016/j.neuron.2014.12.007

Prorok T, 2019, NEUROCHEM RES, V44, P751, DOI 10.1007/s11064-018-02705-0

Raivich G, 1999, ACTA NEUROCHIR SUPPL, V73, P21

Raza H, 2008, TOXICOL APPL PHARM, V226, P161, DOI 10.1016/j.taap.2007.09.002

Roberts LJ, 2005, BRAIN PATHOL, V15, P143

Ryan BJ, 2015, TRENDS BIOCHEM SCI, V40, P200, DOI 10.1016/j.tibs.2015.02.003

Sanders LH, 2013, FREE RADICAL BIO MED, V62, P111, DOI 10.1016/j.freeradbiomed.2013.01.003

Schulz-Schaeffer WJ, 2010, ACTA NEUROPATHOL, V120, P131, DOI 10.1007/s00401-010-0711-0

Serviddio G, 2008, GUT, V57, P957, DOI 10.1136/gut.2007.147496

Shadrina MI, 2010, INT REV CEL MOL BIO, V281, P229, DOI 10.1016/S1937-6448(10)81006-8

Sharp WW, 2014, FASEB J, V28, P316, DOI 10.1096/fj.12-226225

Somayajulu-Nitu M, 2009, BMC NEUROSCI, V10, DOI 10.1186/1471-2202-10-88

Song XJ, 2019, FRONT PHYSIOL, V9, DOI 10.3389/fphys.2018.01957

Su B, 2010, BBA-MOL BASIS DIS, V1802, P135, DOI 10.1016/j.bbadis.2009.09.013

Sun N, 2016, MOL CELL, V61, P654, DOI 10.1016/j.molcel.2016.01.028

TOYOKUNI S, 1994, P NATL ACAD SCI USA, V91, P2616, DOI 10.1073/pnas.91.7.2616

Trempe JF, 2013, FRONT NEUROL, V4, DOI 10.3389/fneur.2013.00038

UNGERSTEDT U, 1968, EUR J PHARMACOL, V5, P107, DOI 10.1016/0014-2999(68)90164-7

Uttara B, 2009, CURR NEUROPHARMACOL, V7, P65, DOI 10.2174/157015909787602823

Uversky VN, 2008, CURR PROTEIN PEPT SC, V9, P507, DOI 10.2174/138920308785915218

Uversky VN, 2004, CELL TISSUE RES, V318, P225, DOI 10.1007/s00441-004-0937-z

Vakifahmetoglu-Norberg H, 2017, BIOCHEM BIOPH RES CO, V482, P426, DOI 10.1016/j.bbrc.2016.11.088

Valko M, 2007, INT J BIOCHEM CELL B, V39, P44, DOI 10.1016/j.biocel.2006.07.001

van der Merwe C, 2015, EUR J NEUROSCI, V41, P1113, DOI 10.1111/ejn.12872

Vandiver MS, 2013, NAT COMMUN, V4, DOI 10.1038/ncomms2623

Vekrellis K, 2011, LANCET NEUROL, V10, P1015, DOI 10.1016/S1474-4422(11)70213-7

Wareski P, 2009, J BIOL CHEM, V284, P21379, DOI 10.1074/jbc.M109.018911

Whitton PS, 2007, BRIT J PHARMACOL, V150, P963, DOI 10.1038/sj.bjp.0707167

Winklhofer KF, 2010, BBA-MOL BASIS DIS, V1802, P29, DOI 10.1016/j.bbadis.2009.08.013

Yamagami K, 2000, ANTIOXID REDOX SIGN, V2, P127, DOI 10.1089/ars.2000.2.1-127

Yang YF, 2006, P NATL ACAD SCI USA, V103, P10793, DOI 10.1073/pnas.0602493103

Yin ZY, 2016, MICROB CELL, V3, P457, DOI 10.15698/mic2016.12.546

Zhang J, 1999, AM J PATHOL, V154, P1423, DOI 10.1016/S0002-9440(10)65396-5

Zhang S, 2018, NEUROBIOL AGING, V61, P52, DOI 10.1016/j.neurobiolaging.2017.09.016

Zhou WB, 2011, J BIOL CHEM, V286, P14941, DOI 10.1074/jbc.M110.211029

NR 97

TC 24

Z9 26

U1 2

U2 20

PU SPRINGER-VERLAG ITALIA SRL

PI MILAN

PA VIA DECEMBRIO, 28, MILAN, 20137, ITALY

SN 1590-1874

EI 1590-3478

J9 NEUROL SCI

JI Neurol. Sci.

PD NOV

PY 2021

VL 42

IS 11

BP 4459

EP 4469

DI 10.1007/s10072-021-05551-1

EA SEP 2021

PG 11

WC Clinical Neurology; Neurosciences

WE Science Citation Index Expanded (SCI-EXPANDED)

SC Neurosciences & Neurology

GA WH4LQ

UT WOS:000692448700004

PM 34480241

DA 2024-08-12

ER

PT J

AU Mehta, N

Luthra, NS

Corcos, DM

Fantuzzi, G

AF Mehta, Niyati

Luthra, Nijee S. S.

Corcos, Daniel M. M.

Fantuzzi, Giamila

TI C-reactive protein as the biomarker of choice to monitor the effects of

exercise on inflammation in Parkinson's disease

SO FRONTIERS IN IMMUNOLOGY

LA English

DT Article

DE C-reactive protein; Parkinson's disease; inflammation; exercise;

biomarker

ID EXPRESSION; INTENSITY; CYTOKINES; RISK; BETA; CRP

AB Parkinson's disease (PD), a heterogeneous disease with no disease-modifying treatments available, is the fastest growing neurological disease worldwide. Currently, physical exercise is the most promising treatment to slow disease progression, with evidence suggesting it is neuroprotective in animal models. The onset, progression, and symptom severity of PD are associated with low grade, chronic inflammation which can be quantified by measuring inflammatory biomarkers. In this perspective, we argue that C-reactive protein (CRP) should be used as the primary biomarker for monitoring inflammation and therefore disease progression and severity, particularly in studies examining the impact of an intervention on the signs and symptoms of PD. CRP is the most studied biomarker of inflammation, and it can be detected using relatively well-standardized assays with a wide range of detection, allowing for comparability across studies while generating robust data. An additional advantage of CRP is its ability to detect inflammation irrespective of its origin and specific pathways, an advantageous characteristic when the cause of inflammation remains unknown, such as PD and other chronic, heterogeneous diseases.

C1 [Mehta, Niyati; Corcos, Daniel M. M.] Northwestern Univ, Dept Phys Therapy & Human Movement Sci, Chicago, IL 60208 USA.

[Luthra, Nijee S. S.] Univ Calif San Francisco, Movement Disorder & Neuromodulat Ctr, San Francisco, CA USA.

[Fantuzzi, Giamila] Univ Illinois, Coll Appl Hlth Sci, Dept Kinesiol & Nutr, Chicago, IL USA.

C3 Northwestern University; University of California System; University of

California San Francisco; University of Illinois System; University of

Illinois Chicago; University of Illinois Chicago Hospital

RP Mehta, N (corresponding author), Northwestern Univ, Dept Phys Therapy & Human Movement Sci, Chicago, IL 60208 USA.

EM niyati.mehta@northwestern.edu

RI Fantuzzi, Giamila/A-1758-2009

FU [K23NS123506]; [U01NS113851]

FX Funding Author NL - Recipient of grant funding through K23NS123506.

Author DC - Recipient of grant funding through U01NS113851.

CR Agrawal A, 2003, IMMUNOLOGY, V108, P539, DOI 10.1046/j.1365-2567.2003.01608.x

Andican G, 2012, ACTA NEUROL BELG, V112, P155, DOI 10.1007/s13760-012-0015-3

Babcock MC, 2022, J CLIN ENDOCR METAB, V107, pE500, DOI 10.1210/clinem/dgab715

Banait T, 2022, CUREUS J MED SCIENCE, V14, DOI 10.7759/cureus.30225

Bartus RT, 2004, J PHARMACOL EXP THER, V310, P828, DOI 10.1124/jpet.103.064121

Bautmans I, 2021, EXP GERONTOL, V146, DOI 10.1016/j.exger.2021.111236

Bennett DA, 1996, NEW ENGL J MED, V334, P71, DOI 10.1056/NEJM199601113340202

Black S, 2004, J BIOL CHEM, V279, P48487, DOI 10.1074/jbc.R400025200

Chen HL, 2003, ARCH NEUROL-CHICAGO, V60, P1059, DOI 10.1001/archneur.60.8.1059

Collier TJ, 2017, MOVEMENT DISORD, V32, P983, DOI 10.1002/mds.27037

Connolly BS, 2014, JAMA-J AM MED ASSOC, V311, P1670, DOI 10.1001/jama.2014.3654

Corporation OHIR, 2019, EX TRAIN PAT PERS PE

Cullinane PW, 2023, MOVEMENT DISORD, V38, P162, DOI 10.1002/mds.29298

Daniele TMD, 2020, BEHAV BRAIN RES, V383, DOI 10.1016/j.bbr.2020.112488

Du Clos TW, 2000, ANN MED, V32, P274, DOI 10.3109/07853890009011772

Eklund CM, 2009, ADV CLIN CHEM, V48, P111, DOI 10.1016/S0065-2423(09)48005-3

Gamborg M, 2022, ACTA NEUROL SCAND, V145, P504, DOI 10.1111/ane.13579

GANAPATHI MK, 1991, J IMMUNOL, V147, P1261

Gollan R, 2022, J PARKINSON DIS, V12, P1783, DOI 10.3233/JPD-223252

Group TPS, 2021, STUD PARK DIS EX

Hirsch Etienne C, 2012, Parkinsonism Relat Disord, V18 Suppl 1, pS210, DOI 10.1016/S1353-8020(11)70065-7

Khalafi M, 2021, EXP GERONTOL, V150, DOI 10.1016/j.exger.2021.111398

Kohut ML, 2006, BRAIN BEHAV IMMUN, V20, P201, DOI 10.1016/j.bbi.2005.12.002

Lindqvist D, 2013, BRAIN BEHAV IMMUN, V33, P183, DOI 10.1016/j.bbi.2013.07.007

Luan YY, 2018, FRONT IMMUNOL, V9, DOI 10.3389/fimmu.2018.01302

Marnell L, 2005, CLIN IMMUNOL, V117, P104, DOI 10.1016/j.clim.2005.08.004

Medical Center P, 2022, EXERCISE ADV PARKINS

Michigan A, 2011, MOL DIAGN THER, V15, P265, DOI 10.2165/11593400-000000000-00000

Moghaddam HS, 2018, NEUROMOL MED, V20, P376, DOI 10.1007/s12017-018-8499-5

Nalls MA, 2014, NAT GENET, V46, P989, DOI 10.1038/ng.3043

Patterson CG, 2022, TRIALS, V23, DOI 10.1186/s13063-022-06703-0

Qiu XH, 2019, FRONT NEUROL, V10, DOI 10.3389/fneur.2019.00384

RAMADORI G, 1985, J EXP MED, V162, P930, DOI 10.1084/jem.162.3.930

Rose GL, 2021, J SCI MED SPORT, V24, P345, DOI 10.1016/j.jsams.2020.10.004

Sawada H, 2015, PLOS ONE, V10, DOI 10.1371/journal.pone.0134118

Schenkman M, 2018, JAMA NEUROL, V75, P219, DOI 10.1001/jamaneurol.2017.3517

Song IU, 2011, NEUROL SCI, V32, P31, DOI 10.1007/s10072-010-0335-0

Suffredini AF, 1999, J CLIN IMMUNOL, V19, P203, DOI 10.1023/A:1020563913045

Szalai AJ, 2000, J IMMUNOL, V165, P1030, DOI 10.4049/jimmunol.165.2.1030

Tysnes OB, 2017, J NEURAL TRANSM, V124, P901, DOI 10.1007/s00702-017-1686-y

University IM, 2022, COMP EFF GREEN EX PR

Volanakis JE, 2001, MOL IMMUNOL, V38, P189, DOI 10.1016/S0161-5890(01)00042-6

NR 42

TC 3

Z9 3

U1 2

U2 2

PU FRONTIERS MEDIA SA

PI LAUSANNE

PA AVENUE DU TRIBUNAL FEDERAL 34, LAUSANNE, CH-1015, SWITZERLAND

SN 1664-3224

J9 FRONT IMMUNOL

JI Front. Immunol.

PD MAY 12

PY 2023

VL 14

AR 1178448

DI 10.3389/fimmu.2023.1178448

PG 5

WC Immunology

WE Science Citation Index Expanded (SCI-EXPANDED)

SC Immunology

GA H1EH2

UT WOS:000993455100001

PM 37251392

OA Green Published, gold

DA 2024-08-12

ER

PT J

AU Wu, JY

Xu, XQ

Zheng, L

Mo, JF

Jin, XH

Bao, Y

AF Wu, Jiayuan

Xu, Xinqin

Zheng, Li

Mo, Juanfen

Jin, Xiuhui

Bao, Yi

TI Nilotinib inhibits microglia-mediated neuroinflammation to protect

against dopaminergic neuronal death in Parkinson's disease models

SO INTERNATIONAL IMMUNOPHARMACOLOGY

LA English

DT Article

DE Nilotinib; Microglia; Neuroinflammation; NF-kappa B; Parkinson's disease

ID ALPHA-SYNUCLEIN; DEGRADATION; ACTIVATION; DEMENTIA; SYSTEM

AB Microglia-mediated neuroinflammation is tightly correlated with the etiology and progression of neurodegenerative disorders, including Parkinson's disease (PD). Nilotinib, a c-Abl inhibitor used for chronic myeloid leukemia, has been proven effective in relieving PD progression. However, whether nilotinib could affect neuroinflammation is largely unknown. In this current study, we investigated the role of nilotinib in microgliamediated neuro-inflammatory response in Parkinson's disease. Lipopolysaccharide (LPS)-induced neuroinflammation in BV2 microglial cells and mouse brains were used as models for Parkinson's disease. Our results demonstrated that nilotinib significantly suppressed LPS-induced neuroinflammation by reducing the production of pro-inflammatory factors including iNOS, COX-2, IL-1 beta, IL-6 and TNF-alpha in BV2 cells. Moreover, pretreatment of nilotinib attenuated the neurotoxicity of LPS-treated microglial conditioned medium to MES23.5 dopaminergic (DA) neurons. Mechanismly, nilotinib inhibited NF-kappa B signaling pathway and suppressed the nuclear translocation of p65 upon LPS stimulation. In LPS-injected mouse brains, nilotinib administration markedly suppressed the activation of microglia and down-regulated COX-2 as well as IL-1 beta expression. Most importantly, nilotinib effectively protected against microglial activation-mediated mouse DA neuronal loss. Taken together, our study suggests that nilotinib exerts anti-neuroinflammatory effect and protects DA neurons from activated microglia-induced inflammatory damage through suppressing NF-kappa B signaling pathway, indicating its potential application in further clinical trials.

C1 [Wu, Jiayuan; Zheng, Li; Mo, Juanfen; Bao, Yi] Jiaxing Univ, Affiliated Hosp 2, Key Lab, Jiaxing 314000, Zhejiang, China.

[Xu, Xinqin] Jiaxing Univ, Affiliated Hosp 2, Rehabil Med Ctr, Jiaxing 314000, Zhejiang, China.

[Jin, Xiuhui] Univ Toronto, Dept Immunol Human Biol, Toronto M4Y 0B9, ON, Canada.

C3 Jiaxing University; Jiaxing University; University of Toronto

RP Bao, Y (corresponding author), Jiaxing Univ, Affiliated Hosp 2, Key Lab, Jiaxing 314000, Zhejiang, China.

EM ybao2011@gmail.com

FU Health Bureau of Zhejiang Province [2020KY952]; Science and Technology

Bureau of Jiaxing [2018AY32007, 2018AD32008]

FX This work was supported by Health Bureau of Zhejiang Province (grant no.

2020KY952) , and Science and Technology Bureau of Jiaxing (grant no.

2018AY32007 and 2018AD32008) .

CR Ball N, 2019, FRONT NEUROL, V10, DOI 10.3389/fneur.2019.00218

Chen ZH, 2012, J NEUROSCI, V32, P11706, DOI 10.1523/JNEUROSCI.0730-12.2012

Colonna M, 2017, ANNU REV IMMUNOL, V35, P441, DOI 10.1146/annurev-immunol-051116-052358

DeRemer DL, 2008, CLIN THER, V30, P1956, DOI 10.1016/j.clinthera.2008.11.014

Dickson DW, 2018, PARKINSONISM RELAT D, V46, pS30, DOI 10.1016/j.parkreldis.2017.07.033

Gerhard A, 2006, NEUROBIOL DIS, V21, P404, DOI 10.1016/j.nbd.2005.08.002

Glass CK, 2010, CELL, V140, P918, DOI 10.1016/j.cell.2010.02.016

Gu C, 2018, FRONT CELL NEUROSCI, V12, DOI 10.3389/fncel.2018.00400

Hebron ML, 2013, HUM MOL GENET, V22, P3315, DOI 10.1093/hmg/ddt192

Hirsch Etienne C, 2012, Parkinsonism Relat Disord, V18 Suppl 1, pS210, DOI 10.1016/S1353-8020(11)70065-7

Hong Z, 2010, BRAIN, V133, P713, DOI 10.1093/brain/awq008

Hou X, 2020, J MOL BIOL, V432, P2651, DOI 10.1016/j.jmb.2020.01.037

Imam SZ, 2011, J NEUROSCI, V31, P157, DOI 10.1523/JNEUROSCI.1833-10.2011

Karuppagounder SS, 2014, SCI REP-UK, V4, DOI 10.1038/srep04874

Kasuga K, 2010, J NEUROL NEUROSUR PS, V81, P608, DOI 10.1136/jnnp.2009.197483

LeWitt PA, 2015, MOVEMENT DISORD, V30, P64, DOI 10.1002/mds.26082

Mahul-Mellier AL, 2014, HUM MOL GENET, V23, P2858, DOI 10.1093/hmg/ddt674

Malkus KA, 2009, MOL NEURODEGENER, V4, DOI 10.1186/1750-1326-4-24

MCGEER PL, 1988, NEUROLOGY, V38, P1285, DOI 10.1212/WNL.38.8.1285

Nagatsu T, 2005, CURR PHARM DESIGN, V11, P999, DOI 10.2174/1381612053381620

Napetschnig J, 2013, ANNU REV BIOPHYS, V42, P443, DOI 10.1146/annurev-biophys-083012-130338

Oeckinghaus A, 2009, CSH PERSPECT BIOL, V1, DOI 10.1101/cshperspect.a000034

Olanow CW, 2006, MOVEMENT DISORD, V21, P1806, DOI 10.1002/mds.21013

Pagan F, 2016, J PARKINSON DIS, V6, P503, DOI 10.3233/JPD-160867

Pagan FL, 2021, MOVEMENT DISORD, V36, P740, DOI 10.1002/mds.28389

Pagan FL, 2020, JAMA NEUROL, V77, P309, DOI 10.1001/jamaneurol.2019.4200

Pagan FL, 2019, PHARMACOL RES PERSPE, V7, DOI 10.1002/prp2.470

Pickrell AM, 2015, NEURON, V85, P257, DOI 10.1016/j.neuron.2014.12.007

Rahimifard M, 2017, AGEING RES REV, V36, P11, DOI 10.1016/j.arr.2017.02.004

Saitgareeva AR, 2020, NEUROL SCI, V41, P3609, DOI 10.1007/s10072-020-04468-5

Smith JA, 2012, BRAIN RES BULL, V87, P10, DOI 10.1016/j.brainresbull.2011.10.004

Subhramanyam CS, 2019, SEMIN CELL DEV BIOL, V94, P112, DOI 10.1016/j.semcdb.2019.05.004

Turner RS, 2020, ANN NEUROL, V88, P183, DOI 10.1002/ana.25775

Wang QQ, 2015, TRANSL NEURODEGENER, V4, DOI 10.1186/s40035-015-0042-0

NR 34

TC 21

Z9 21

U1 0

U2 14

PU ELSEVIER

PI AMSTERDAM

PA RADARWEG 29, 1043 NX AMSTERDAM, NETHERLANDS

SN 1567-5769

EI 1878-1705

J9 INT IMMUNOPHARMACOL

JI Int. Immunopharmacol.

PD OCT

PY 2021

VL 99

AR 108025

DI 10.1016/j.intimp.2021.108025

EA AUG 2021

PG 10

WC Immunology; Pharmacology & Pharmacy

WE Science Citation Index Expanded (SCI-EXPANDED)

SC Immunology; Pharmacology & Pharmacy

GA UM0FK

UT WOS:000693016400001

PM 34364303

DA 2024-08-12

ER

PT J

AU Huang, QH

Yang, P

Liu, Y

Ding, JH

Lu, M

Hu, G

AF Huang, Qianhui

Yang, Pei

Liu, Yang

Ding, Jianhua

Lu, Ming

Hu, Gang

TI The interplay between α-Synuclein and NLRP3 inflammasome in Parkinson's

disease

SO BIOMEDICINE & PHARMACOTHERAPY

LA English

DT Review

DE Parkinson's disease; alpha-Synuclein; NLRP3; Pathophysiology;

Neuroinflammation

ID MITOCHONDRIAL COMPLEX I; RECEPTOR 2; NEURODEGENERATION; ACTIVATION;

EPIDEMIOLOGY; AGGREGATION; INHIBITION; IMPAIRMENT; OXIDATION; FIBRILS

AB alpha-Synuclein is a member of a protein of synucleins, which is a presynaptic neuron protein. It is usually highly expressed in the brain and participates in the formation and transmission of nerve synapses. It has been reported that abnormal aggregation of alpha-Syn can induce the activation of NLRP3 inflammasome in microglia, increase the production of IL-113, and aggravate neuroinflammation. Therefore, it is recognized as one of the important factors leading to neuroinflammation in Parkinson's disease. In this paper, we aimed to explore the influence of post translational modification of alpha-Syn on its pathological aggregation and summarize various pathways that activate NLRP3 triggered by alpha-Syn and targeted therapeutic strategies, which provided new insights for further exploring the origin and targeted therapy of Parkinson's disease.

C1 [Huang, Qianhui; Yang, Pei; Liu, Yang; Hu, Gang] Nanjing Univ Chinese Med, Sch Med & Holist Integrat Med, Nanjing 210023, China.

[Ding, Jianhua; Lu, Ming; Hu, Gang] Nanjing Med Univ, Dept Pharmacol, Nanjing 211166, Jiangsu, China.

[Lu, Ming] Nanjing Med Univ, Dept Pharmacol, Jiangsu Key Lab Neurodegenerat, 101 Longmian Ave, Nanjing 211166, Jiangsu, China.

[Hu, Gang] Nanjing Univ Chinese Med, Sch Med & Holist Integrat Med, 138 Xian Lin Ave, Nanjing 211166, Jiangsu, China.

C3 Nanjing University of Chinese Medicine; Nanjing Medical University;

Nanjing Medical University; Nanjing University of Chinese Medicine

RP Lu, M (corresponding author), Nanjing Med Univ, Dept Pharmacol, Jiangsu Key Lab Neurodegenerat, 101 Longmian Ave, Nanjing 211166, Jiangsu, China.; Hu, G (corresponding author), Nanjing Univ Chinese Med, Sch Med & Holist Integrat Med, 138 Xian Lin Ave, Nanjing 211166, Jiangsu, China.

EM lum@njmu.edu.cn; neuropha@njmu.edu.cn

RI Lu, Ming/JDM-5513-2023

FU National Natural Science Foundation of China [82003725, 81991523];

Natural Science Foundation of Nanjing University of Chinese Medicine

[NZY82003725]

FX This work was funded by the National Natural Science Foundation of China

(Grants Nos. 82003725 and 81991523) and the Natural Science Foundation

of Nanjing University of Chinese Medicine (No. NZY82003725) .

CR Barrett PJ, 2015, BRAIN RES, V1628, P247, DOI 10.1016/j.brainres.2015.06.002

Bender A, 2013, PLOS ONE, V8, DOI 10.1371/journal.pone.0062277

Block ML, 2007, NAT REV NEUROSCI, V8, P57, DOI 10.1038/nrn2038

Boyer DR, 2020, P NATL ACAD SCI USA, V117, P3592, DOI 10.1073/pnas.1917914117

Buneeva O, 2022, INT J MOL SCI, V23, DOI 10.3390/ijms23073705

Burbulla LF, 2017, SCIENCE, V357, P1255, DOI 10.1126/science.aam9080

Burmann BM, 2020, NATURE, V577, P127, DOI 10.1038/s41586-019-1808-9

Burré J, 2018, CSH PERSPECT MED, V8, DOI 10.1101/cshperspect.a024091

Cai BS, 2022, AUTOPHAGY, V18, P990, DOI 10.1080/15548627.2021.1965426

Campolo M, 2019, BRAIN BEHAV IMMUN, V76, P236, DOI 10.1016/j.bbi.2018.12.003

Champy P, 2004, J NEUROCHEM, V88, P63, DOI 10.1046/j.1471-4159.2003.02138.x

Chen JL, 2021, J NEUROINFLAMM, V18, DOI 10.1186/s12974-021-02349-y

Cheng JB, 2020, AUTOPHAGY, V16, P2193, DOI 10.1080/15548627.2020.1719723

Choi I, 2020, AUTOPHAGY, V16, P1718, DOI 10.1080/15548627.2020.1774149

Choi I, 2020, NAT COMMUN, V11, DOI 10.1038/s41467-020-15119-w

Chu YP, 2009, NEUROBIOL DIS, V35, P385, DOI 10.1016/j.nbd.2009.05.023

Clayton DF, 1998, TRENDS NEUROSCI, V21, P249, DOI 10.1016/S0166-2236(97)01213-7

Coll RC, 2022, TRENDS PHARMACOL SCI, V43, P653, DOI 10.1016/j.tips.2022.04.003

Coll RC, 2015, NAT MED, V21, P248, DOI 10.1038/nm.3806

Cuervo AM, 2004, SCIENCE, V305, P1292, DOI 10.1126/science.1101738

de Lau LML, 2006, LANCET NEUROL, V5, P525, DOI 10.1016/S1474-4422(06)70471-9

Devi L, 2008, J BIOL CHEM, V283, P9089, DOI 10.1074/jbc.M710012200

Di Maio R, 2016, SCI TRANSL MED, V8, DOI 10.1126/scitranslmed.aaf3634

Di QQ, 2023, AUTOPHAGY, V19, P873, DOI 10.1080/15548627.2022.2107314

Dzamko N, 2017, ACTA NEUROPATHOL, V133, P303, DOI 10.1007/s00401-016-1648-8

Elliott EI, 2015, IMMUNOL REV, V265, P35, DOI 10.1111/imr.12286

Ellis CE, 2005, MOL CELL BIOL, V25, P10190, DOI 10.1128/MCB.25.22.10190-10201.2005

Fan Z, 2016, MOL NEUROBIOL, V53, P7057, DOI 10.1007/s12035-015-9620-5

Fox SH, 2018, MOVEMENT DISORD, V33, P1248, DOI 10.1002/mds.27372

Fu JN, 2023, ANNU REV IMMUNOL, V41, P301, DOI 10.1146/annurev-immunol-081022-021207

Ganjam GK, 2019, CELL DEATH DIS, V10, DOI 10.1038/s41419-019-2091-2

Gordon R, 2018, SCI TRANSL MED, V10, DOI 10.1126/scitranslmed.aah4066

Gustot A, 2015, BIOCHEM J, V471, P323, DOI 10.1042/BJ20150617

Han XJ, 2019, AUTOPHAGY, V15, P1860, DOI 10.1080/15548627.2019.1596481

He WT, 2015, CELL RES, V25, P1285, DOI 10.1038/cr.2015.139

Hodara R, 2004, J BIOL CHEM, V279, P47746, DOI 10.1074/jbc.M408906200

Hou LY, 2018, REDOX BIOL, V14, P600, DOI 10.1016/j.redox.2017.11.010

Huang Y, 2021, CELL MOL IMMUNOL, V18, P2114, DOI 10.1038/s41423-021-00740-6

Hunter SF, 2016, CNS DRUGS, V30, P135, DOI 10.1007/s40263-015-0297-0

Inzelberg R, 2002, AM J MED GENET, V111, P459, DOI 10.1002/ajmg.10586

Kalia LV, 2015, LANCET, V386, P896, DOI 10.1016/S0140-6736(14)61393-3

Kim C, 2015, CELL REP, V13, P771, DOI 10.1016/j.celrep.2015.09.044

Kim C, 2013, NAT COMMUN, V4, DOI 10.1038/ncomms2534

Kouli A, 2019, BRAIN BEHAV IMMUN, V81, P41, DOI 10.1016/j.bbi.2019.06.042

Lee E, 2019, CELL DEATH DIFFER, V26, P213, DOI 10.1038/s41418-018-0124-5

Lee KW, 2011, J NEUROSCI, V31, P6963, DOI 10.1523/JNEUROSCI.6513-10.2011

Levine PM, 2019, P NATL ACAD SCI USA, V116, P1511, DOI 10.1073/pnas.1808845116

Levine PM, 2017, BIOORGAN MED CHEM, V25, P4977, DOI 10.1016/j.bmc.2017.04.038

Li DS, 2018, HUM CELL, V31, P106, DOI 10.1007/s13577-017-0187-5

MANN VM, 1994, ANN NEUROL, V36, P876, DOI 10.1002/ana.410360612

Marotta NP, 2015, NAT CHEM, V7, P913, DOI [10.1038/nchem.2361, 10.1038/NCHEM.2361]

Mehra S, 2019, BBA-PROTEINS PROTEOM, V1867, P890, DOI 10.1016/j.bbapap.2019.03.001

Mizushima N, 2011, CELL, V147, P728, DOI 10.1016/j.cell.2011.10.026

NICKLAS WJ, 1985, LIFE SCI, V36, P2503, DOI 10.1016/0024-3205(85)90146-8

Nordström E, 2021, NEUROBIOL DIS, V161, DOI 10.1016/j.nbd.2021.105543

Ou Z, 2021, MOL NEUROBIOL, V58, P1303, DOI 10.1007/s12035-020-02198-5

Park JH, 2020, MOL NEURODEGENER, V15, DOI 10.1186/s13024-019-0349-x

Pike AF, 2021, GLIA, V69, P1413, DOI 10.1002/glia.23970

Polissidis A, 2020, BIOMOLECULES, V10, DOI 10.3390/biom10060912

Samii A, 2004, LANCET, V363, P1783, DOI 10.1016/S0140-6736(04)16305-8

SCHAPIRA AHV, 1989, LANCET, V1, P1269, DOI 10.1016/S0140-6736(89)92366-0

Schofield DJ, 2019, NEUROBIOL DIS, V132, DOI 10.1016/j.nbd.2019.104582

Sevcsik E, 2011, J AM CHEM SOC, V133, P7152, DOI 10.1021/ja2009554

Shao BZ, 2015, FRONT PHARMACOL, V6, DOI 10.3389/fphar.2015.00262

Shen Y, 2022, BRAIN SCI, V12, DOI 10.3390/brainsci12081057

Shimada K, 2012, IMMUNITY, V36, P401, DOI 10.1016/j.immuni.2012.01.009

Sun YP, 2021, NAT COMMUN, V12, DOI 10.1038/s41467-021-26433-2

Sun YP, 2020, CELL RES, V30, P360, DOI 10.1038/s41422-020-0299-4

Tanaka Y, 2001, HUM MOL GENET, V10, P919, DOI 10.1093/hmg/10.9.919

Tao YQ, 2022, NAT COMMUN, V13, DOI 10.1038/s41467-022-31790-7

Tapias V, 2017, CELL MOL LIFE SCI, V74, P2851, DOI 10.1007/s00018-017-2541-x

Tolosa E, 2021, LANCET NEUROL, V20, P385, DOI 10.1016/S1474-4422(21)00030-2

Trove T., TrialTroveID-360928, The Multiple Proof of Concept Studies of Phase II Inzomelid in Patients with Parkinson's Disease

Tysnes OB, 2017, J NEURAL TRANSM, V124, P901, DOI 10.1007/s00702-017-1686-y

Wang S, 2019, INT IMMUNOPHARMACOL, V67, P458, DOI 10.1016/j.intimp.2018.12.019

Winner B, 2011, P NATL ACAD SCI USA, V108, P4194, DOI 10.1073/pnas.1100976108

Wu J, 2012, NEUROSCIENCE, V207, P288, DOI 10.1016/j.neuroscience.2012.01.028

Xie WL, 2012, J NEUROCHEM, V122, P404, DOI 10.1111/j.1471-4159.2012.07769.x

Yao S, 2019, J NEUROIMMUNE PHARM, V14, P478, DOI 10.1007/s11481-019-09843-4

Ying ZM, 2022, ANN TRANSL MED, DOI 10.21037/atm-22-5159

Yuan JX, 2022, ADV MATER, V34, DOI 10.1002/adma.202108435

Zhang Y, 2019, PHARMACOL RES, V150, DOI 10.1016/j.phrs.2019.104538

Zhao K, 2020, P NATL ACAD SCI USA, V117, P20305, DOI 10.1073/pnas.1922741117

Zhong ZY, 2018, NATURE, V560, P198, DOI 10.1038/s41586-018-0372-z

Zhou RB, 2011, NATURE, V469, P221, DOI 10.1038/nature09663

Zhou Y, 2016, MOL NEURODEGENER, V11, DOI 10.1186/s13024-016-0094-3

NR 86

TC 5

Z9 5

U1 17

U2 22

PU ELSEVIER FRANCE-EDITIONS SCIENTIFIQUES MEDICALES ELSEVIER

PI ISSY-LES-MOULINEAUX

PA 65 RUE CAMILLE DESMOULINS, CS50083, 92442 ISSY-LES-MOULINEAUX, FRANCE

SN 0753-3322

EI 1950-6007

J9 BIOMED PHARMACOTHER

JI Biomed. Pharmacother.

PD DEC

PY 2023

VL 168

AR 115735

DI 10.1016/j.biopha.2023.115735

EA OCT 2023

PG 8

WC Medicine, Research & Experimental; Pharmacology & Pharmacy

WE Science Citation Index Expanded (SCI-EXPANDED)

SC Research & Experimental Medicine; Pharmacology & Pharmacy

GA X2WT4

UT WOS:001097116500001

PM 37852103

OA gold

DA 2024-08-12

ER

EF

FN Clarivate Analytics Web of Science

VR 1.0

PT J

AU Chen, ZC

Chen, SD

Liu, J

AF Chen, Zhichun

Chen, Shengdi

Liu, Jun

TI The role of T cells in the pathogenesis of Parkinson's disease

SO PROGRESS IN NEUROBIOLOGY

LA English

DT Review

DE Parkinson's disease; T cell; Biomarker; Therapy; Neuroinflammation

ID EXPERIMENTAL AUTOIMMUNE ENCEPHALOMYELITIS; CENTRAL-NERVOUS-SYSTEM;

BLOOD-BRAIN-BARRIER; TUMOR-NECROSIS-FACTOR; MPTP MOUSE MODEL; PROTECTS

DOPAMINERGIC-NEURONS; CEREBROSPINAL FLUID BARRIER; GERMINAL-CENTER

FORMATION; ADAPTIVE IMMUNE-RESPONSE; ANTIGEN-PRESENTING CELLS

AB Recent evidence has shown that neuroinflammation plays a key role in the pathogenesis of Parkinson's disease (PD). However, different components of the brain's immune system may exert diverse effects on neuroinflammatory events in PD. The adaptive immune response, especially the T cell response, can trigger type 1 pro inflammatory activities and suppress type 2 anti-inflammatory activities, eventually resulting in deregulated neuroinflammation and subsequent dopaminergic neurodegeneration. Additionally, studies have increasingly shown that therapies targeting T cells can alleviate neurodegeneration and motor behavior impairment in animal models of PD. Therefore, we conclude that abnormal T cell-mediated immunity is a fundamental pathological process that may be a promising translational therapeutic target for Parkinson's disease.

C1 [Liu, Jun] Shanghai Jiao Tong Univ, Dept Neurol, Ruijin Hosp, Sch Med, Shanghai 200025, China.

Shanghai Jiao Tong Univ, Inst Neurol, Ruijin Hosp, Sch Med, Shanghai 200025, China.

C3 Shanghai Jiao Tong University; Shanghai Jiao Tong University

RP Liu, J (corresponding author), Shanghai Jiao Tong Univ, Dept Neurol, Ruijin Hosp, Sch Med, Shanghai 200025, China.

EM jly0520@hotmail.com

RI Chen, Zhichun/IQU-9542-2023; Huang, Jingyi/KCY-2239-2024

OI Chen, Zhichun/0000-0002-1250-0999; Liu, Jun/0000-0002-0843-5127

CR Abt MC, 2012, IMMUNITY, V37, P158, DOI 10.1016/j.immuni.2012.04.011

Adeeb N, 2013, CHILD NERV SYST, V29, P1803, DOI 10.1007/s00381-013-2044-5

Adeeb N, 2013, CHILD NERV SYST, V29, P17, DOI 10.1007/s00381-012-1910-x

Adeeb N, 2012, CHILD NERV SYST, V28, P827, DOI 10.1007/s00381-012-1744-6

Afkarian M, 2002, NAT IMMUNOL, V3, P549, DOI 10.1038/ni794

Aguiar AS, 2013, NEUROTOX RES, V24, P280, DOI 10.1007/s12640-013-9389-0

Ahmed I, 2012, MOVEMENT DISORD, V27, P1104, DOI 10.1002/mds.25035

Alberio T, 2014, J PROTEOME RES, V13, P3554, DOI 10.1021/pr401142p

Alberio T, 2012, SCI REP-UK, V2, DOI 10.1038/srep00953

Almolda B, 2011, FRONT BIOSCI-LANDMRK, V16, P1157, DOI 10.2741/3781

Almolda B, 2010, J NEUROIMMUNOL, V223, P39, DOI 10.1016/j.jneuroim.2010.03.021

Aloisi F, 1998, J IMMUNOL, V160, P4671

Aluvihare VR, 2004, NAT IMMUNOL, V5, P266, DOI 10.1038/ni1037

Alvarez-Sánchez N, 2015, BRAIN BEHAV IMMUN, V50, P101, DOI 10.1016/j.bbi.2015.06.021

Anandasabapathy N, 2011, J EXP MED, V208, P1695, DOI 10.1084/jem.20102657

Ascherio A, 2016, LANCET NEUROL, V15, P1255, DOI 10.1016/S1474-4422(16)30230-7

Aspelund A, 2015, J EXP MED, V212, P991, DOI 10.1084/jem.20142290

Baba Y, 2005, PARKINSONISM RELAT D, V11, P493, DOI 10.1016/j.parkreldis.2005.07.005

Bai FQ, 2012, CANCER IMMUNOL IMMUN, V61, P523, DOI 10.1007/s00262-011-1109-0

Bailey SL, 2007, NAT IMMUNOL, V8, P172, DOI 10.1038/ni1430

Bandres-Ciga S, 2017, MOVEMENT DISORD, V32, P1327, DOI 10.1002/mds.27116

Barbeau B, 1998, J VIROL, V72, P7125, DOI 10.1128/JVI.72.9.7125-7136.1998

Barcia C, 2012, CELL DEATH DIS, V3, DOI 10.1038/cddis.2012.123

Barnett R, 2016, LANCET, V387, P217, DOI 10.1016/S0140-6736(16)00049-0

Bartholomäus I, 2009, NATURE, V462, P94, DOI 10.1038/nature08478

Bartok B, 2011, EXP CELL RES, V317, P1312, DOI 10.1016/j.yexcr.2011.04.002

Baruch K, 2015, NAT COMMUN, V6, DOI 10.1038/ncomms8967

Baruch K, 2013, P NATL ACAD SCI USA, V110, P2264, DOI 10.1073/pnas.1211270110

Bas J, 2001, J NEUROIMMUNOL, V113, P146, DOI 10.1016/S0165-5728(00)00422-7

Basler M, 2014, EMBO MOL MED, V6, P226, DOI 10.1002/emmm.201303543

Battistini L, 2003, BLOOD, V101, P4775, DOI 10.1182/blood-2002-10-3309

Becher B, 2003, J CLIN INVEST, V112, P1186, DOI 10.1172/JCI200319079

Beers DR, 2011, BRAIN BEHAV IMMUN, V25, P1025, DOI 10.1016/j.bbi.2010.12.008

Beers DR, 2011, BRAIN, V134, P1293, DOI 10.1093/brain/awr074

Benner EJ, 2004, P NATL ACAD SCI USA, V101, P9435, DOI 10.1073/pnas.0400569101

Benner EJ, 2008, PLOS ONE, V3, DOI 10.1371/journal.pone.0001376

Berg JV, 2013, J EXP MED, V210, P2803, DOI 10.1084/jem.20130678

Bernard-Valnet R, 2016, P NATL ACAD SCI USA, V113, P10956, DOI 10.1073/pnas.1603325113

BOKA G, 1994, NEUROSCI LETT, V172, P151, DOI 10.1016/0304-3940(94)90684-X

Bollig N, 2012, P NATL ACAD SCI USA, V109, P8664, DOI 10.1073/pnas.1205834109

Bongioanni P, 1997, J NEUROL SCI, V149, P41, DOI 10.1016/S0022-510X(97)05382-3

Bongioanni P, 1997, J NEURAL TRANSM, V104, P199, DOI 10.1007/BF01273181

Boska MD, 2005, J NEUROSCI, V25, P1691, DOI 10.1523/JNEUROSCI.4364-04.2005

Brinker T, 2014, FLUIDS BARRIERS CNS, V11, DOI 10.1186/2045-8118-11-10

Brizic I, 2018, EUR J IMMUNOL, V48, P950, DOI 10.1002/eji.201847526

Brochard V, 2009, J CLIN INVEST, V119, P182, DOI 10.1172/JCI36470

Browne TC, 2013, J IMMUNOL, V190, P2241, DOI 10.4049/jimmunol.1200947

Bynoe MS, 2003, IMMUNITY, V19, P317, DOI 10.1016/S1074-7613(03)00239-5

Caggiu E, 2017, J NEUROIMMUNOL, V310, P26, DOI 10.1016/j.jneuroim.2017.06.004

CANTOR H, 1975, EUR J IMMUNOL, V5, P330, DOI 10.1002/eji.1830050508

Cao CH, 2009, NEUROBIOL DIS, V34, P63, DOI 10.1016/j.nbd.2008.12.015

Carare RO, 2014, BRAIN BEHAV IMMUN, V36, P9, DOI 10.1016/j.bbi.2013.10.012

Cassan C, 2007, J NEUROCHEM, V100, P883, DOI 10.1111/j.1471-4159.2006.04270.x

Cebrián C, 2014, NAT COMMUN, V5, DOI 10.1038/ncomms4633

Chandra G, 2017, J IMMUNOL, V198, P4312, DOI 10.4049/jimmunol.1700149

Chandra G, 2016, J BIOL CHEM, V291, P15267, DOI 10.1074/jbc.M116.714824

Chen HL, 2005, ANN NEUROL, V58, P963, DOI 10.1002/ana.20682

Chen HL, 2003, ARCH NEUROL-CHICAGO, V60, P1059, DOI 10.1001/archneur.60.8.1059

Chen SY, 2017, NEUROSCI LETT, V648, P21, DOI 10.1016/j.neulet.2017.03.045

Chen WJ, 2003, J EXP MED, V198, P1875, DOI 10.1084/jem.20030152

Chen YF, 2017, PLOS ONE, V12, DOI 10.1371/journal.pone.0189380

Chi Y, 2011, AGING CELL, V10, P368, DOI 10.1111/j.1474-9726.2011.00677.x

CHIBA S, 1995, J NEUROL SCI, V132, P170, DOI 10.1016/0022-510X(95)00136-P

Choi GB, 2016, SCIENCE, V351, P933, DOI 10.1126/science.aad0314

Chuang YH, 2017, ANN NEUROL, V82, P655, DOI 10.1002/ana.25065

Chung ES, 2015, J IMMUNOL, V195, P4853, DOI 10.4049/jimmunol.1500386

Chung ES, 2012, BRAIN BEHAV IMMUN, V26, P1322, DOI 10.1016/j.bbi.2012.08.013

Ciesielska A, 2003, ACTA NEUROBIOL EXP, V63, P117, DOI 10.55782/ane-2003-1461

Clemente-Casares X, 2016, NATURE, V530, P434, DOI 10.1038/nature16962

Codarri L, 2011, NAT IMMUNOL, V12, P560, DOI 10.1038/ni.2027

Cohn L, 1997, J EXP MED, V186, P1737, DOI 10.1084/jem.186.10.1737

Constantinescu CS, 2005, J NEUROCHEM, V95, P331, DOI 10.1111/j.1471-4159.2005.03368.x

Contreras F, 2016, J IMMUNOL, V196, P4143, DOI 10.4049/jimmunol.1502420

Couturier N, 2011, BRAIN, V134, P693, DOI 10.1093/brain/awr010

Crawford A, 2011, PLOS PATHOG, V7, DOI 10.1371/journal.ppat.1002098

Cruz-Orengo L, 2011, J EXP MED, V208, P327, DOI 10.1084/jem.20102010

Csóka B, 2008, FASEB J, V22, P3491, DOI 10.1096/fj.08-107458

Cua DJ, 2003, NATURE, V421, P744, DOI 10.1038/nature01355

Danielski LG, 2018, MOL NEUROBIOL, V55, P1045, DOI 10.1007/s12035-016-0356-7

Dansokho C, 2016, BRAIN, V139, P1237, DOI 10.1093/brain/awv408

Darlington PJ, 2013, ANN NEUROL, V73, P341, DOI 10.1002/ana.23784

Das J, 2001, NAT IMMUNOL, V2, P45, DOI 10.1038/83158

de Graaf MT, 2011, CYTOM PART B-CLIN CY, V80B, P43, DOI 10.1002/cyto.b.20542

Delgoffe GM, 2011, NAT IMMUNOL, V12, P295, DOI 10.1038/ni.2005

Derecki NC, 2010, J EXP MED, V207, P1067, DOI 10.1084/jem.20091419

Dhaeze T, 2015, J IMMUNOL, V195, P832, DOI 10.4049/jimmunol.1500759

Doerck S, 2010, PLOS ONE, V5, DOI 10.1371/journal.pone.0015478

Doi H, 2014, MOVEMENT DISORD, V29, P562, DOI 10.1002/mds.25777

Dufek M, 2015, PARKINSONS DIS-US, V2015, DOI 10.1155/2015/898192

Dunford PJ, 2006, J IMMUNOL, V176, P7062, DOI 10.4049/jimmunol.176.11.7062

Enders U, 1998, BRAIN, V121, P1257, DOI 10.1093/brain/121.7.1257

Engelhardt B, 1998, J CLIN INVEST, V102, P2096, DOI 10.1172/JCI4271

Engelhardt B, 2008, CURR PHARM DESIGN, V14, P1555, DOI 10.2174/138161208784705432

Engelhardt B, 2017, NAT IMMUNOL, V18, P123, DOI 10.1038/ni.3666

Engelhardt B, 2012, TRENDS IMMUNOL, V33, P579, DOI 10.1016/j.it.2012.07.004

Fabriek BO, 2005, GLIA, V51, P297, DOI 10.1002/glia.20208

Ferreira R, 2012, J NEUROINFLAMM, V9, DOI 10.1186/1742-2094-9-90

Filiano AJ, 2017, NAT REV NEUROSCI, V18, P375, DOI 10.1038/nrn.2017.39

FISZER U, 1994, ACTA NEUROL SCAND, V90, P160

FISZER U, 1994, J NEUROL SCI, V121, P39, DOI 10.1016/0022-510X(94)90154-6

Fitzgerald DC, 2014, J NEUROINFLAMM, V11, DOI 10.1186/1742-2094-11-130

Flynn KM, 2013, AM J PATHOL, V182, P1322, DOI 10.1016/j.ajpath.2013.01.003

Fontenot JD, 2003, NAT IMMUNOL, V4, P330, DOI 10.1038/ni904

Frank-Cannon TC, 2008, J NEUROSCI, V28, P10825, DOI 10.1523/JNEUROSCI.3001-08.2008

Frischer JM, 2014, MULT SCLER J, V20, P1171, DOI 10.1177/1352458514521516

Fujisaki J, 2011, NATURE, V474, P216, DOI 10.1038/nature10160

Gagliano SA, 2016, ANN CLIN TRANSL NEUR, V3, P924, DOI 10.1002/acn3.369

Gagne JJ, 2010, NEUROLOGY, V74, P995, DOI 10.1212/WNL.0b013e3181d5a4a3

Garris CS, 2013, NAT IMMUNOL, V14, P1166, DOI 10.1038/ni.2730

Ge RM, 2017, J NEUROINFLAMM, V14, DOI 10.1186/s12974-017-0909-3

GELFAND MC, 1975, J IMMUNOL, V115, P1

Geri G, 2011, J ALLERGY CLIN IMMUN, V128, P655, DOI 10.1016/j.jaci.2011.05.029

Gerwien H, 2016, SCI TRANSL MED, V8, DOI 10.1126/scitranslmed.aaf8020

Ghersi-Egea JF, 2018, ACTA NEUROPATHOL, V135, P337, DOI 10.1007/s00401-018-1807-1

Ghorbani S, 2017, FRONT IMMUNOL, V8, DOI 10.3389/fimmu.2017.00758

Ghoreschi K, 2010, NATURE, V467, P967, DOI 10.1038/nature09447

Ghosh A, 2010, ANN NEUROSCI, V17, P191, DOI 10.5214/ans.0972.7531.1017410

González H, 2013, J IMMUNOL, V190, P5048, DOI 10.4049/jimmunol.1203121

Goverman J, 2009, NAT REV IMMUNOL, V9, P393, DOI 10.1038/nri2550

Grant JL, 2012, SCI TRANSL MED, V4, DOI 10.1126/scitranslmed.3004145

Gu LJ, 2012, STROKE, V43, P1941, DOI 10.1161/STROKEAHA.112.656611

Guo F, 2008, J IMMUNOL, V181, P2285, DOI 10.4049/jimmunol.181.4.2285

Guo YW, 2014, J NEUROIMMUNOL, V276, P64, DOI 10.1016/j.jneuroim.2014.08.616

Gutzmer R, 2009, J ALLERGY CLIN IMMUN, V123, P619, DOI 10.1016/j.jaci.2008.12.1110

Haghikia A, 2015, IMMUNITY, V43, P817, DOI 10.1016/j.immuni.2015.09.007

Harms AS, 2013, J NEUROSCI, V33, P9592, DOI 10.1523/JNEUROSCI.5610-12.2013

Hatzi K, 2015, J EXP MED, V212, P539, DOI 10.1084/jem.20141380

He R, 2016, NATURE, V537, P412, DOI 10.1038/nature19317

Heesters BA, 2016, TRENDS IMMUNOL, V37, P844, DOI 10.1016/j.it.2016.10.003

Hermingsson A. J., 2011, J NEUROINFLAMM, V8, P36

Herz J, 2017, IMMUNITY, V46, P943, DOI 10.1016/j.immuni.2017.06.007

Hill DA, 2012, NAT MED, V18, P538, DOI 10.1038/nm.2657

Hirsch EC, 2003, ANN NY ACAD SCI, V991, P214

Hoehlig K, 2012, EUR J IMMUNOL, V42, P1164, DOI 10.1002/eji.201142242

Hollmann C, 2016, J IMMUNOL, V197, P3130, DOI 10.4049/jimmunol.1600691

Horvath S, 2015, AGING-US, V7, P1130, DOI 10.18632/aging.100859

Hou JH, 2016, TISSUE BARRIERS, V4, DOI 10.1080/21688370.2016.1142492

Hsu P, 2015, J IMMUNOL, V195, P3665, DOI 10.4049/jimmunol.1402898

Hu XF, 2015, DRUG DES DEV THER, V9, P4847, DOI 10.2147/DDDT.S89792

Huang W, 2015, NATURE, V528, P517, DOI 10.1038/nature16193

Huang Y, 2017, CELL PHYSIOL BIOCHEM, V41, P1240, DOI 10.1159/000464388

Huber AK, 2014, NEUROLOGY, V83, P1500, DOI 10.1212/WNL.0000000000000908

Huber M, 2008, P NATL ACAD SCI USA, V105, P20846, DOI 10.1073/pnas.0809077106

Huizinga R, 2012, GLIA, V60, P422, DOI 10.1002/glia.22276

Hunot S, 1999, J NEUROSCI, V19, P3440

Hunter Z, 2014, ACS NANO, V8, P2148, DOI 10.1021/nn405033r

Ichiyama K, 2016, IMMUNITY, V44, P1284, DOI 10.1016/j.immuni.2016.05.015

Ifergan I, 2011, ANN NEUROL, V70, P751, DOI 10.1002/ana.22519

Iliff Jeffrey J, 2012, Sci Transl Med, V4, p147ra111, DOI 10.1126/scitranslmed.3003748

Iliff JJ, 2013, J CLIN INVEST, V123, P1299, DOI 10.1172/JCI67677

Iñiguez MA, 1999, J IMMUNOL, V163, P111

Ivanov II, 2006, CELL, V126, P1121, DOI 10.1016/j.cell.2006.07.035

Janssens K, 2015, BRAIN BEHAV IMMUN, V45, P180, DOI 10.1016/j.bbi.2014.11.010

Johnston J, 2012, DRUGS, V72, P1195, DOI 10.2165/11634010-000000000-00000

Johnston TH, 2010, MOVEMENT DISORD, V25, P1379, DOI 10.1002/mds.23069

Kalia LV, 2015, LANCET, V386, P896, DOI 10.1016/S0140-6736(14)61393-3

Kallies A, 2017, TRENDS IMMUNOL, V38, P287, DOI 10.1016/j.it.2017.02.003

Kang JE, 2009, SCIENCE, V326, P1005, DOI 10.1126/science.1180962

Kang SM, 2008, MOL CELLS, V25, P64

Kannan AK, 2015, J NEUROSCI, V35, P221, DOI 10.1523/JNEUROSCI.1957-14.2015

Kannarkat GT, 2015, NPJ PARKINSON DIS, V1, DOI 10.1038/npjparkd.2015.2

Kannarkat GT, 2013, J PARKINSON DIS, V3, P493, DOI 10.3233/JPD-130250

Khattri R, 2003, NAT IMMUNOL, V4, P337, DOI 10.1038/ni909

Kim Hyun-Jin, 2010, Immune Netw, V10, P92, DOI 10.4110/in.2010.10.3.92

Kim S, 2017, NATURE, V549, P528, DOI 10.1038/nature23910

KIMURA K, 2018, NAT COMMUN, V9

Kipnis J, 2016, SCIENCE, V353, P766, DOI 10.1126/science.aag2638

Kissick HT, 2014, P NATL ACAD SCI USA, V111, P9887, DOI 10.1073/pnas.1402468111

Kitoh A, 2009, IMMUNITY, V31, P609, DOI 10.1016/j.immuni.2009.09.003

Kivisäkk P, 2003, P NATL ACAD SCI USA, V100, P8389, DOI 10.1073/pnas.1433000100

Kivisäkk P, 2009, ANN NEUROL, V65, P457, DOI 10.1002/ana.21379

Kleiter I, 2010, BRAIN, V133, P1067, DOI 10.1093/brain/awq039

Klunker S, 2009, J EXP MED, V206, P2701, DOI 10.1084/jem.20090596

Knowland D, 2014, NEURON, V82, P603, DOI 10.1016/j.neuron.2014.03.003

Kolbinger F, 2016, CURR DRUG TARGETS, V17, P1882, DOI 10.2174/1389450117666160307144027

Kong WM, 2016, BRAIN BEHAV IMMUN, V53, P59, DOI 10.1016/j.bbi.2015.11.002

Koprich JB, 2008, J NEUROINFLAMM, V5, DOI 10.1186/1742-2094-5-8

Korn T, 2017, NAT REV IMMUNOL, V17, P179, DOI 10.1038/nri.2016.144

Korn T, 2009, ANNU REV IMMUNOL, V27, P485, DOI 10.1146/annurev.immunol.021908.132710

Kosloski LM, 2013, J NEUROIMMUNOL, V265, P1, DOI 10.1016/j.jneuroim.2013.10.009

Krishnamoorthy G, 2009, NAT MED, V15, P626, DOI 10.1038/nm.1975

Krishnamoorthy V, 2017, IMMUNITY, V47, P481, DOI 10.1016/j.immuni.2017.09.001

Kuo PC, 2016, BRAIN BEHAV IMMUN, V57, P173, DOI 10.1016/j.bbi.2016.03.015

Kurkowska-Jastrzebska I, 1999, ACTA NEUROBIOL EXP, V59, P1, DOI 10.55782/ane-1999-1289

Kurup SP, 2017, NAT MED, V23, P1220, DOI 10.1038/nm.4395

Kusam S, 2003, J IMMUNOL, V170, P2435, DOI 10.4049/jimmunol.170.5.2435

Kustrimovic N, 2016, SCI REP-UK, V6, DOI 10.1038/srep33738

Lacan G, 2013, J NEUROSCI RES, V91, P1292, DOI 10.1002/jnr.23253

Lahl K, 2007, J EXP MED, V204, P57, DOI 10.1084/jem.20061852

Landrith TA, 2017, FRONT IMMUNOL, V8, DOI 10.3389/fimmu.2017.00335

Lanteri MC, 2009, J CLIN INVEST, V119, P3266, DOI 10.1172/JCI39387

Lanz TV, 2013, P NATL ACAD SCI USA, V110, P14735, DOI 10.1073/pnas.1302569110

Laurence A, 2007, IMMUNITY, V26, P371, DOI 10.1016/j.immuni.2007.02.009

Laurie C, 2007, J NEUROIMMUNOL, V183, P60, DOI 10.1016/j.jneuroim.2006.11.009

Lauterbach H, 2006, J EXP MED, V203, P1963, DOI 10.1084/jem.20060039

Lee MJ, 2016, MOL NEURODEGENER, V11, DOI 10.1186/s13024-016-0116-1

Lee PW, 2015, BRAIN BEHAV IMMUN, V46, P44, DOI 10.1016/j.bbi.2014.12.007

Lee Y, 2012, NAT IMMUNOL, V13, P991, DOI 10.1038/ni.2416

Lengfeld JE, 2017, P NATL ACAD SCI USA, V114, pE1168, DOI 10.1073/pnas.1609905114

Levine AG, 2014, NAT IMMUNOL, V15, P1070, DOI 10.1038/ni.3004

Li B, 2013, EXP NEUROL, V250, P239, DOI 10.1016/j.expneurol.2013.10.002

Li PY, 2013, ANN NEUROL, V74, P458, DOI 10.1002/ana.23815

Li YH, 2015, EXP MOL PATHOL, V99, P220, DOI 10.1016/j.yexmp.2015.06.016

Li YF, 2017, MULT SCLER RELAT DIS, V18, P20, DOI 10.1016/j.msard.2017.09.003

Liberman AC, 2012, PLOS ONE, V7, DOI 10.1371/journal.pone.0035155

Liesz A, 2009, NAT MED, V15, P192, DOI 10.1038/nm.1927

Lim S, 2015, NAT COMMUN, V6, DOI 10.1038/ncomms9244

Litteljohn Darcy, 2012, Methods Mol Biol, V934, P121, DOI 10.1007/978-1-62703-071-7_7

Liu SQ, 2014, J BIOL CHEM, V289, P12446, DOI 10.1074/jbc.M114.550723

Liu YD, 2014, J IMMUNOL, V192, P59, DOI 10.4049/jimmunol.1301513

Liu Z, 2017, MOL NEUROBIOL, V54, P7762, DOI 10.1007/s12035-016-0249-9

Louveau A, 2017, J CLIN INVEST, V127, P3210, DOI 10.1172/JCI90603

Louveau A, 2015, NATURE, V523, P337, DOI 10.1038/nature14432

Lovett-Racke AE, 2004, IMMUNITY, V21, P719, DOI 10.1016/j.immuni.2004.09.010

Lucca LE, 2016, EUR J IMMUNOL, V46, P2247, DOI 10.1002/eji.201646416

Luo Y, 2015, BRAIN RES, V1597, P86, DOI 10.1016/j.brainres.2014.12.005

Lutz SE, 2017, CELL REP, V21, P2104, DOI 10.1016/j.celrep.2017.10.094

Ma K, 2016, J NEUROCHEM, V139, P1151, DOI 10.1111/jnc.13871

Ma QL, 2017, NAT COMMUN, V8, DOI 10.1038/s41467-017-01484-6

Mackay LK, 2015, J IMMUNOL, V194, P2059, DOI 10.4049/jimmunol.1402256

Main BS, 2016, GLIA, V64, P1590, DOI 10.1002/glia.23028

Mao LL, 2017, BRAIN, V140, P1914, DOI 10.1093/brain/awx111

Martin HL, 2016, GLIA, V64, P386, DOI 10.1002/glia.22935

Martinelli R, 2015, JOVE-J VIS EXP, DOI 10.3791/53288

Masini D, 2017, TRANSL PSYCHIAT, V7, DOI 10.1038/tp.2017.58

Masliah E, 2005, NEURON, V46, P857, DOI 10.1016/j.neuron.2005.05.010

Masliah E, 2011, PLOS ONE, V6, DOI 10.1371/journal.pone.0019338

Massa MG, 2017, AM J PATHOL, V187, P1613, DOI 10.1016/j.ajpath.2017.03.006

Matheoud D, 2016, CELL, V166, P314, DOI 10.1016/j.cell.2016.05.039

Matsumoto J, 2017, CURR PHARM DESIGN, V23, P6206, DOI 10.2174/1381612823666170913164738

Matyszak MK, 1999, EUR J IMMUNOL, V29, P3063, DOI 10.1002/(SICI)1521-4141(199910)29:10<3063::AID-IMMU3063>3.0.CO;2-G

Mazo IB, 2005, IMMUNITY, V22, P259, DOI 10.1016/j.immuni.2005.01.008

McCandless EE, 2008, P NATL ACAD SCI USA, V105, P11270, DOI 10.1073/pnas.0800898105

McCandless EE, 2006, J IMMUNOL, V177, P8053, DOI 10.4049/jimmunol.177.11.8053

McMenamin PG, 1999, J COMP NEUROL, V405, P553, DOI 10.1002/(SICI)1096-9861(19990322)405:4<553::AID-CNE8>3.0.CO;2-6

McMenamin PG, 2003, CELL TISSUE RES, V313, P259, DOI 10.1007/s00441-003-0779-0

McWilliams IL, 2015, J NEUROINFLAMM, V12, DOI 10.1186/s12974-015-0351-3

Mele DA, 2013, J EXP MED, V210, P2181, DOI 10.1084/jem.20130376

Mero IL, 2010, EUR J HUM GENET, V18, P502, DOI 10.1038/ejhg.2009.195

Miles B, 2015, NAT COMMUN, V6, DOI 10.1038/ncomms9608

Miranda AM, 2018, NAT COMMUN, V9, DOI 10.1038/s41467-017-02533-w

Mohammed J, 2016, NAT IMMUNOL, V17, P414, DOI 10.1038/ni.3396

Montoya MM, 2017, J IMMUNOL, V199, P559, DOI 10.4049/jimmunol.1700170

Mosley RL, 2012, CSH PERSPECT MED, V2, DOI 10.1101/cshperspect.a009381

Mues M, 2013, NAT MED, V19, P778, DOI 10.1038/nm.3180

Murdock BJ, 2014, J IMMUNOL, V193, P4107, DOI 10.4049/jimmunol.1400650

Mykicki N, 2016, SCI TRANSL MED, V8, DOI 10.1126/scitranslmed.aaf8732

Nagashima H, 2016, J IMMUNOL, V196, P4082, DOI 10.4049/jimmunol.1501610

Nagashima H, 2014, NAT IMMUNOL, V15, P449, DOI 10.1038/ni.2863

Nagatsu T, 2000, ADV RES NEURODEGENER, V8, P277

Nance JP, 2015, P NATL ACAD SCI USA, V112, P13324, DOI 10.1073/pnas.1507312112

Nizri E, 2009, J IMMUNOL, V183, P6681, DOI 10.4049/jimmunol.0902212

Oakley MS, 2013, J IMMUNOL, V191, P4699, DOI 10.4049/jimmunol.1300396

Oberländer U, 2011, BMC NEUROSCI, V12, DOI 10.1186/1471-2202-12-116

Obermeier B, 2016, HAND CLINIC, V133, P39, DOI 10.1016/B978-0-444-63432-0.00003-7

Obermeier B, 2013, NAT MED, V19, P1584, DOI 10.1038/nm.3407

Obeso JA, 2017, MOVEMENT DISORD, V32, P1, DOI 10.1002/mds.26904

Odoardi F, 2012, NATURE, V488, P675, DOI 10.1038/nature11337

Oertel Wolfgang H, 2017, F1000Res, V6, P260, DOI 10.12688/f1000research.10100.1

Olson JK, 2004, J IMMUNOL, V173, P3916, DOI 10.4049/jimmunol.173.6.3916

Olson KE, 2015, J NEUROSCI, V35, P16463, DOI 10.1523/JNEUROSCI.2131-15.2015

Orsini H, 2014, BRAIN BEHAV IMMUN, V37, P177, DOI 10.1016/j.bbi.2013.12.012

Paladugu M, 2013, CANCER IMMUNOL IMMUN, V62, P75, DOI 10.1007/s00262-012-1312-7

Park L, 2017, CIRC RES, V121, P258, DOI 10.1161/CIRCRESAHA.117.311054

Passani MB, 2012, FRONT SYST NEUROSCI, V6, DOI 10.3389/fnsys.2012.00032

Payne NL, 2013, BRAIN BEHAV IMMUN, V30, P103, DOI 10.1016/j.bbi.2013.01.079

Pearce EL, 2003, SCIENCE, V302, P1041, DOI 10.1126/science.1090148

Pedreño M, 2014, BRAIN BEHAV IMMUN, V37, P152, DOI 10.1016/j.bbi.2013.11.021

Perry VH, 2012, CSH PERSPECT MED, V2, DOI 10.1101/cshperspect.a009373

Pesic M, 2013, J CLIN INVEST, V123, P1192, DOI 10.1172/JCI67233

Peter I, 2018, JAMA NEUROL, V75, P939, DOI 10.1001/jamaneurol.2018.0605

Peters A, 2011, CURR OPIN IMMUNOL, V23, P702, DOI 10.1016/j.coi.2011.08.007

Piccio L, 2005, J IMMUNOL, V174, P5805, DOI 10.4049/jimmunol.174.9.5805

Pikor NB, 2015, IMMUNITY, V43, P1160, DOI 10.1016/j.immuni.2015.11.010

Pinheiro MAL, 2016, ELIFE, V5, DOI 10.7554/eLife.13149

Plog BA, 2018, ANNU REV PATHOL-MECH, V13, P379, DOI 10.1146/annurev-pathol-051217-111018

Plog BA, 2015, J NEUROSCI, V35, P518, DOI 10.1523/JNEUROSCI.3742-14.2015

Pöllinger B, 2009, J EXP MED, V206, P1303, DOI 10.1084/jem.20090299

Pranzatelli MR, 2018, CLIN EXP IMMUNOL, V193, P103, DOI 10.1111/cei.13122

Prineas JW, 2012, ANN NEUROL, V72, P18, DOI 10.1002/ana.23634

Prinz M, 2017, NAT IMMUNOL, V18, P385, DOI 10.1038/ni.3703

Qin HW, 2016, J NEUROSCI, V36, P5144, DOI 10.1523/JNEUROSCI.4658-15.2016

Qin HW, 2012, P NATL ACAD SCI USA, V109, P5004, DOI 10.1073/pnas.1117218109

Qin XY, 2016, JAMA NEUROL, V73, P1316, DOI 10.1001/jamaneurol.2016.2742

Quintana FJ, 2017, J CLIN INVEST, V127, P2897, DOI 10.1172/JCI95769

Ramadan A, 2016, BRAIN, V139, P1433, DOI 10.1093/brain/aww032

Ransohoff RM, 2016, SCIENCE, V353, P777, DOI 10.1126/science.aag2590

Raper D, 2016, TRENDS NEUROSCI, V39, P581, DOI 10.1016/j.tins.2016.07.001

Reale M, 2009, BRAIN BEHAV IMMUN, V23, P55, DOI 10.1016/j.bbi.2008.07.003

Rentzos M, 2007, ACTA NEUROL SCAND, V116, P374, DOI 10.1111/j.1600-0404.2007.00894.x

Restorick SM, 2017, BRAIN BEHAV IMMUN, V64, P71, DOI 10.1016/j.bbi.2017.03.008

Revu S, 2018, CELL REP, V22, P2642, DOI 10.1016/j.celrep.2018.02.044

Reynolds AD, 2007, J LEUKOCYTE BIOL, V82, P1083, DOI 10.1189/jlb.0507296

Reynolds AD, 2010, J IMMUNOL, V184, P2261, DOI 10.4049/jimmunol.0901852

Reynolds AD, 2009, J IMMUNOL, V182, P4137, DOI 10.4049/jimmunol.0803982

Rocha NP, 2018, MOL NEUROBIOL, V55, P1488, DOI 10.1007/s12035-017-0404-y

Rocha SM, 2016, J NEUROINFLAMM, V13, DOI 10.1186/s12974-016-0600-0

Rocha SM, 2014, FRONT CELL NEUROSCI, V8, DOI 10.3389/fncel.2014.00120

Rockenstein E, 2018, J NEUROSCI, V38, P1000, DOI 10.1523/JNEUROSCI.1170-17.2017

Rosenkranz D, 2007, J NEUROIMMUNOL, V188, P117, DOI 10.1016/j.jneuroim.2007.05.011

Sa QL, 2014, INFECT IMMUN, V82, P2826, DOI 10.1128/IAI.01494-13

Saligrama N, 2013, J IMMUNOL, V191, P614, DOI 10.4049/jimmunol.1203137

Samii A, 2009, DRUG AGING, V26, P769, DOI 10.2165/11316780-000000000-00000

Sampson TR, 2016, CELL, V167, P1469, DOI 10.1016/j.cell.2016.11.018

Sanchez-Guajardo V, 2013, J NEUROPATH EXP NEUR, V72, P624, DOI 10.1097/NEN.0b013e31829768d2

Sandquist Ivy, 2018, F1000Res, V7, P205, DOI 10.12688/f1000research.13020.1

Santarlasci V, 2012, IMMUNITY, V36, P201, DOI 10.1016/j.immuni.2011.12.013

Saresella M, 2011, BRAIN BEHAV IMMUN, V25, P539, DOI 10.1016/j.bbi.2010.12.004

Saunders JAH, 2012, J NEUROIMMUNE PHARM, V7, P927, DOI 10.1007/s11481-012-9402-z

Schain AJ, 2018, ANN NEUROL, V83, P508, DOI 10.1002/ana.25169

Schapira AHV, 2014, LANCET, V384, P545, DOI 10.1016/S0140-6736(14)61010-2

Scheperjans F, 2015, MOVEMENT DISORD, V30, P350, DOI 10.1002/mds.26069

Schiering C, 2014, NATURE, V513, P564, DOI 10.1038/nature13577

Schläger C, 2016, NATURE, V530, P349, DOI 10.1038/nature16939

Schmidt AM, 2015, J IMMUNOL, V194, P4362, DOI 10.4049/jimmunol.1402384

Schmitz J, 2005, IMMUNITY, V23, P479, DOI 10.1016/j.immuni.2005.09.015

Schneider-Hohendorf T, 2010, EUR J IMMUNOL, V40, P3581, DOI 10.1002/eji.201040558

Schober SL, 1999, J IMMUNOL, V163, P3662

Schraml BU, 2009, NATURE, V460, P405, DOI 10.1038/nature08114

Schwartz M, 2014, J AUTOIMMUN, V54, P8, DOI 10.1016/j.jaut.2014.08.002

Serafini B, 2000, AM J PATHOL, V157, P1991, DOI 10.1016/S0002-9440(10)64838-9

Severin ME, 2016, BRAIN, V139, P1747, DOI 10.1093/brain/aww084

Sheean RK, 2018, JAMA NEUROL, V75, P681, DOI 10.1001/jamaneurol.2018.0035

Shi M, 2014, ACTA NEUROPATHOL, V128, P639, DOI 10.1007/s00401-014-1314-y

Shin H, 2009, IMMUNITY, V31, P309, DOI 10.1016/j.immuni.2009.06.019

Shin HM, 2013, IMMUNITY, V39, P661, DOI 10.1016/j.immuni.2013.08.032

Siffrin V, 2010, IMMUNITY, V33, P424, DOI 10.1016/j.immuni.2010.08.018

Simon DW, 2017, NAT REV NEUROL, V13, P572, DOI 10.1038/nrneurol.2017.116

Singh R, 2017, J IMMUNOL, V198, P4394, DOI 10.4049/jimmunol.1602010

Skon CN, 2013, NAT IMMUNOL, V14, P1285, DOI 10.1038/ni.2745

Skundric DS, 2006, J NEUROINFLAMM, V3, DOI 10.1186/1742-2094-3-13

Sloka S, 2011, J NEUROINFLAMM, V8, DOI 10.1186/1742-2094-8-56

Slyepchenko A, 2016, NEUROSCI BIOBEHAV R, V64, P83, DOI 10.1016/j.neubiorev.2016.02.002

Sonar SA, 2018, J LEUKOCYTE BIOL, V103, P839, DOI 10.1002/JLB.1RU1117-428R

Sosa RA, 2013, J IMMUNOL, V191, P5848, DOI 10.4049/jimmunol.1300771

Souza-Moreira L, 2013, J IMMUNOL, V191, P2144, DOI 10.4049/jimmunol.1300384

Souza-Moreira L, 2013, BRAIN BEHAV IMMUN, V30, P54, DOI 10.1016/j.bbi.2013.01.080

Srinivasan BS, 2009, HUM MUTAT, V30, P228, DOI 10.1002/humu.20840

Staines DR, 2007, MED HYPOTHESES, V69, P1208, DOI 10.1016/j.mehy.2007.04.004

Steinbach K, 2016, J EXP MED, V213, P1571, DOI 10.1084/jem.20151916

Steiner O, 2010, J IMMUNOL, V185, P4846, DOI 10.4049/jimmunol.0903732

Stienne C, 2016, IMMUNITY, V45, P774, DOI 10.1016/j.immuni.2016.09.010

Stopschinski BE, 2017, LANCET NEUROL, V16, P323, DOI 10.1016/S1474-4422(17)30037-6

STROBER S, 1989, J IMMUNOL, V143, P1118

Subramaniam SR, 2017, FRONT AGING NEUROSCI, V9, DOI 10.3389/fnagi.2017.00176

Sulzer D, 2017, NATURE, V546, P656, DOI 10.1038/nature22815

Sun BL, 2018, PROG NEUROBIOL, V163, P118, DOI 10.1016/j.pneurobio.2017.08.007

Sun CC, 2012, PLOS ONE, V7, DOI 10.1371/journal.pone.0048594

Swanson A, 2018, BEHAV BRAIN RES, V347, P49, DOI 10.1016/j.bbr.2018.02.015

Talebi F, 2017, J NEUROINFLAMM, V14, DOI 10.1186/s12974-017-0832-7

Teuscher C, 2007, P NATL ACAD SCI USA, V104, P10146, DOI 10.1073/pnas.0702291104

Theodore S, 2008, J NEUROPATH EXP NEUR, V67, P1149, DOI 10.1097/NEN.0b013e31818e5e99

Tischner D, 2006, BRAIN, V129, P2635, DOI 10.1093/brain/awl213

TRENTINI A, 2016, DIS MARKERS, V2016, DOI DOI 10.1155/2016/3672353

Tumani H, 2018, HAND CLINIC, V146, P21, DOI 10.1016/B978-0-12-804279-3.00002-2

Ueno M, 2016, BRAIN TUMOR PATHOL, V33, P89, DOI 10.1007/s10014-016-0255-7

Utzschneider DT, 2016, IMMUNITY, V45, P415, DOI 10.1016/j.immuni.2016.07.021

Vajkoczy P, 2001, J CLIN INVEST, V108, P557

Van der Perren A, 2015, NEUROBIOL AGING, V36, P1559, DOI 10.1016/j.neurobiolaging.2015.01.014

van Nierop GP, 2017, ACTA NEUROPATHOL, V134, P383, DOI 10.1007/s00401-017-1744-4

Viceconte N, 2015, J NEUROINFLAMM, V12, DOI 10.1186/s12974-014-0228-x

Vogelaar CF, 2018, SCI TRANSL MED, V10, DOI 10.1126/scitranslmed.aao2304

Vojdani A, 2011, EVID-BASED COMPL ALT, V2011, DOI 10.1093/ecam/nep064

Volta M, 2015, LANCET NEUROL, V14, P1054, DOI 10.1016/S1474-4422(15)00186-6

Walker MR, 2003, J CLIN INVEST, V112, P1437, DOI 10.1172/JCI200319441

Wallen W C, 1979, Trans Am Neurol Assoc, V104, P111

WALLEN WC, 1981, NEUROLOGY, V31, P668, DOI 10.1212/WNL.31.6.668

Wang HP, 2014, NAT IMMUNOL, V15, P393, DOI 10.1038/ni.2846

Wang LM, 2018, BRAIN BEHAV IMMUN, V68, P44, DOI 10.1016/j.bbi.2017.09.015

Wang Y, 2014, IMMUNITY, V40, P355, DOI 10.1016/j.immuni.2014.01.002

Wasser B, 2016, J NEUROIMMUNE PHARM, V11, P721, DOI 10.1007/s11481-016-9696-3

Wei G, 2011, IMMUNITY, V35, P299, DOI 10.1016/j.immuni.2011.08.007

Weller RO, 2018, ACTA NEUROPATHOL, V135, P363, DOI 10.1007/s00401-018-1809-z

Weng QJ, 2018, CELL DEATH DIS, V9, DOI 10.1038/s41419-018-0290-x

Wheeler Christopher J, 2014, J Neurol Neurophysiol, V5

Whish S, 2015, FRONT NEUROSCI-SWITZ, V9, DOI 10.3389/fnins.2015.00016

Wierzba-Bobrowicz T, 2000, FOLIA NEUROPATHOL, V38, P111

Wilms H, 2003, FASEB J, V17, P500, DOI 10.1096/fj.02-0314fje

Wilson EH, 2010, J CLIN INVEST, V120, P1368, DOI 10.1172/JCI41911

Wissemann WT, 2013, AM J HUM GENET, V93, P984, DOI 10.1016/j.ajhg.2013.10.009

Witoelar A, 2017, JAMA NEUROL, V74, P780, DOI 10.1001/jamaneurol.2017.0469

Woodberry T, 2005, J IMMUNOL, V175, P4355, DOI 10.4049/jimmunol.175.7.4355

Woulfe JM, 2014, PARKINSONISM RELAT D, V20, P685, DOI 10.1016/j.parkreldis.2014.02.031

Wu C, 2009, NAT MED, V15, P519, DOI 10.1038/nm.1957

Xie JH, 2003, J IMMUNOL, V170, P3662, DOI 10.4049/jimmunol.170.7.3662

Xin A, 2016, NAT IMMUNOL, V17, P422, DOI 10.1038/ni.3410

Xu LF, 2017, IMMUNITY, V47, P538, DOI 10.1016/j.immuni.2017.08.011

Xue Y, 2014, MOL MED REP, V9, P899, DOI 10.3892/mmr.2014.1893

Yadav SK, 2017, P NATL ACAD SCI USA, V114, pE9318, DOI 10.1073/pnas.1615715114

Yang LK, 2017, MED SCI MONITOR, V23, P2972, DOI 10.12659/MSM.904240

Yang XXO, 2008, IMMUNITY, V28, P29, DOI 10.1016/j.immuni.2007.11.016

Yao SQ, 2012, J NEUROCHEM, V123, P100, DOI 10.1111/j.1471-4159.2012.07807.x

Yao SY, 2013, IMMUNITY, V39, P833, DOI 10.1016/j.immuni.2013.10.007

Ye BH, 1997, NAT GENET, V16, P161, DOI 10.1038/ng0697-161

Yen JH, 2013, BRAIN BEHAV IMMUN, V29, P70, DOI 10.1016/j.bbi.2012.12.004

Yoshida Y, 2014, IMMUNITY, V40, P187, DOI 10.1016/j.immuni.2013.11.022

Zecca L, 2008, ACTA NEUROPATHOL, V116, P47, DOI 10.1007/s00401-008-0361-7

Zhang J, 2014, J NEUROIMMUNOL, V266, P56, DOI 10.1016/j.jneuroim.2013.09.019

Zhang N, 2013, IMMUNITY, V39, P687, DOI 10.1016/j.immuni.2013.08.019

Zhang Y, 2018, EXP THER MED, V15, P1855, DOI 10.3892/etm.2017.5638

Zhang ZR, 2009, J IMMUNOL, V183, P3081, DOI 10.4049/jimmunol.0901088

Zheng WP, 1997, CELL, V89, P587, DOI 10.1016/S0092-8674(00)80240-8

Zhong C, 2017, TRENDS IMMUNOL, V38, P229, DOI 10.1016/j.it.2017.02.006

Zhou K, 2017, J CEREBR BLOOD F MET, V37, P967, DOI 10.1177/0271678X16648712

Zhu HB, 2017, PLOS ONE, V12, DOI 10.1371/journal.pone.0183484

Zhu JF, 2010, CELL RES, V20, P4, DOI 10.1038/cr.2009.138

NR 406

TC 47

Z9 50

U1 0

U2 23

PU PERGAMON-ELSEVIER SCIENCE LTD

PI OXFORD

PA THE BOULEVARD, LANGFORD LANE, KIDLINGTON, OXFORD OX5 1GB, United Kingdom

SN 0301-0082

EI 1873-5118

J9 PROG NEUROBIOL

JI Prog. Neurobiol.

PD OCT

PY 2018

VL 169

BP 1

EP 23

DI 10.1016/j.pneurobio.2018.08.002

PG 23

WC Neurosciences

WE Science Citation Index Expanded (SCI-EXPANDED)

SC Neurosciences & Neurology

GA GU5GC

UT WOS:000445313000001

PM 30114440

DA 2024-08-12

ER

PT J

AU Kim, JH

Lee, HW

Hwang, J

Kim, J

Lee, MJ

Han, HS

Lee, WH

Suk, K

AF Kim, Jong-Heon

Lee, Ho-Won

Hwang, Jaegyu

Kim, Jaehong

Lee, Min-Jeong

Han, Hyung-Soo

Lee, Won-Ha

Suk, Kyoungho

TI Microglia-inhibiting activity of Parkinson's disease drug amantadine

SO NEUROBIOLOGY OF AGING

LA English

DT Article

DE Amantadine; Microglia; Neuroinflammation; Parkinson's disease

ID FORCED SWIMMING TEST; RAT-BRAIN; 1-ADAMANTANAMINE AMANTADINE;

INFLAMMATORY ACTIVATION; ANTIVIRAL ACTIVITY; NEUROINFLAMMATION;

INFLUENZA; MEMANTINE; NEURONS; NEURODEGENERATION

AB Amantadine is currently used as an antiviral and an antiparkinsonian drug. Although the drug is known to bind a viral proton channel protein, the mechanism of action in Parkinson's disease (PD) remains to be determined. This study investigated whether the drug has an inhibitory effect on microglial activation and neuroinflammation, which have been implicated in the progression of neurodegenerative processes. Using cultured microglial cells, it was demonstrated that the drug inhibited inflammatory activation of microglia and a signaling pathway that governs the microglial activation. The drug reduced the expression and production of proinflammatory mediators in bacterial lipopolysaccharide-stimulated microglia cells. The microglia-inhibiting activity of amantadine was also demonstrated in a microglia/neuron coculture and animal models of neuroinflammation and Parkinson's disease. Collectively, our results suggest that amantadine may act on microglia in the central nervous system to inhibit their inflammatory activation, thereby attenuating neuroinflammation. These results provide a molecular basis of the glia-targeted mechanism of action for amantadine. (C) 2012 Elsevier Inc. All rights reserved.

C1 [Kim, Jong-Heon; Hwang, Jaegyu; Kim, Jaehong; Suk, Kyoungho] Kyungpook Natl Univ, Dept Pharmacol, Sch Med, Taegu 700422, South Korea.

[Kim, Jong-Heon; Lee, Ho-Won; Hwang, Jaegyu; Kim, Jaehong; Lee, Min-Jeong; Han, Hyung-Soo; Suk, Kyoungho] Kyungpook Natl Univ, CMRI, Sch Med, Brain Sci & Engn Inst, Taegu 700422, South Korea.

[Lee, Ho-Won] Kyungpook Natl Univ, Dept Neurol, Sch Med, Taegu 700422, South Korea.

[Lee, Min-Jeong; Han, Hyung-Soo] Kyungpook Natl Univ, Dept Physiol, Sch Med, Taegu 700422, South Korea.

[Lee, Won-Ha] Kyungpook Natl Univ, Dept Genet Engn, Sch Life Sci & Biotechnol, Taegu 700422, South Korea.

C3 Kyungpook National University (KNU); Kyungpook National University

(KNU); Kyungpook National University (KNU); Kyungpook National

University (KNU); Kyungpook National University (KNU)

RP Suk, K (corresponding author), Kyungpook Natl Univ, Dept Pharmacol, Sch Med, 101 Dong In, Taegu 700422, South Korea.

EM ksuk@knu.ac.kr

FU Ministry of Health and Welfare, Republic of Korea [A100176]

FX This study was supported by a grant of the Korea Healthcare Technology

R&D Project, Ministry of Health and Welfare, Republic of Korea

(A100176).

CR Abdel-Salam OME, 2008, CNS NEUROL DISORD-DR, V7, P321, DOI 10.2174/187152708786441867

Arif M, 2009, NEUROSCIENCE, V164, P1199, DOI 10.1016/j.neuroscience.2009.08.063

Beigel J, 2008, ANTIVIR RES, V78, P91, DOI 10.1016/j.antiviral.2008.01.003

Betakova T, 2007, CURR PHARM DESIGN, V13, P3231, DOI 10.2174/138161207782341295

Blanpied TA, 2005, J NEUROSCI, V25, P3312, DOI 10.1523/JNEUROSCI.4262-04.2005

Block ML, 2007, NAT REV NEUROSCI, V8, P57, DOI 10.1038/nrn2038

BOCCHINI V, 1992, J NEUROSCI RES, V31, P616, DOI 10.1002/jnr.490310405

Cady SD, 2010, NATURE, V463, P689, DOI 10.1038/nature08722

Cheepsunthorn P, 2001, GLIA, V35, P53, DOI 10.1002/glia.1070

Chen JJ, 2007, PHARMACOTHERAPY, V27, p161S, DOI 10.1592/phco.27.12part2.161S

Cunningham C, 2005, J NEUROSCI, V25, P9275, DOI 10.1523/JNEUROSCI.2614-05.2005

DAVIES WL, 1964, SCIENCE, V144, P862, DOI 10.1126/science.144.3620.862

de Roin S, 1990, J Neurosci Nurs, V22, P322

Diaz NL, 2009, EXPERT REV NEUROTHER, V9, P1781, DOI [10.1586/ern.09.117, 10.1586/ERN.09.117]

Gao HM, 2003, TRENDS PHARMACOL SCI, V24, P395, DOI 10.1016/S0165-6147(03)00176-7

GODWINAUSTEN RB, 1970, LANCET, V2, P383

GRUNERT RR, 1965, VIROLOGY, V26, P262, DOI 10.1016/0042-6822(65)90273-4

Hwang J, 2008, NEUROPHARMACOLOGY, V55, P826, DOI 10.1016/j.neuropharm.2008.06.045

Hwang J, 2010, NEUROPHARMACOLOGY, V58, P1122, DOI 10.1016/j.neuropharm.2010.02.003

Imai Y, 1996, BIOCHEM BIOPH RES CO, V224, P855, DOI 10.1006/bbrc.1996.1112

Kim WG, 2000, J NEUROSCI, V20, P6309, DOI 10.1523/JNEUROSCI.20-16-06309.2000

Kim YS, 2006, EXP MOL MED, V38, P333, DOI 10.1038/emm.2006.40

Kubera M, 2006, INT J NEUROPSYCHOPH, V9, P297, DOI 10.1017/S1461145705005663

Kunz A, 2008, J NEUROSCI, V28, P1649, DOI 10.1523/JNEUROSCI.5205-07.2008

LAWSON LJ, 1990, NEUROSCIENCE, V39, P151, DOI 10.1016/0306-4522(90)90229-W

Lee H, 2003, FASEB J, V17, P1943, DOI 10.1096/fj.03-0057fje

Lee S, 2009, J NEUROSCI, V29, P234, DOI 10.1523/JNEUROSCI.5273-08.2009

Lee Sung Joong, 2002, Current Drug Targets - Inflammation and Allergy, V1, P181

Lehnardt S, 2003, P NATL ACAD SCI USA, V100, P8514, DOI 10.1073/pnas.1432609100

Liu B, 2005, MOL CELL BIOL, V25, P1113, DOI 10.1128/MCB.25.3.1113-1123.2005

Liu M, 2011, PARKINSONS DIS-US, V2011, DOI 10.4061/2011/327089

Long-Smith CM, 2009, PROG NEUROBIOL, V89, P277, DOI 10.1016/j.pneurobio.2009.08.001

MCCARTHY KD, 1980, J CELL BIOL, V85, P890, DOI 10.1083/jcb.85.3.890

MCCLELLAND RA, 1990, CANCER RES, V50, P3545

McGeer EG, 2007, CNS DRUGS, V21, P789, DOI 10.2165/00023210-200721100-00001

McGeer PL, 2008, MOVEMENT DISORD, V23, P474, DOI 10.1002/mds.21751

McLean PJ, 2001, NEUROSCIENCE, V104, P901, DOI 10.1016/S0306-4522(01)00113-0

MCMILLIAN M, 1995, BIOCHEM BIOPH RES CO, V215, P572, DOI 10.1006/bbrc.1995.2503

Morgan MJ, 2011, CELL RES, V21, P103, DOI 10.1038/cr.2010.178

Ock J, 2010, BRIT J PHARMACOL, V159, P1646, DOI 10.1111/j.1476-5381.2010.00659.x

Ohsawa K, 2000, J CELL SCI, V113, P3073

Ossola B, 2011, NEUROPHARMACOLOGY, V61, P574, DOI 10.1016/j.neuropharm.2011.04.030

Pierri M, 2005, NEUROPHARMACOLOGY, V48, P517, DOI 10.1016/j.neuropharm.2004.11.009

Qin LY, 2007, GLIA, V55, P453, DOI 10.1002/glia.20467

Roman A, 2009, PHARMACOL REP, V61, P1069, DOI 10.1016/S1734-1140(09)70169-0

Rosi S, 2006, NEUROSCIENCE, V142, P1303, DOI 10.1016/j.neuroscience.2006.08.017

Rosi S, 2009, BRAIN, V132, P2464, DOI 10.1093/brain/awp148

Saniova B, 2004, J NEURAL TRANSM, V111, P511, DOI 10.1007/s00702-004-0112-4

Saura J, 2003, GLIA, V44, P183, DOI 10.1002/glia.10274

SCHREIBER E, 1989, NUCLEIC ACIDS RES, V17, P6419, DOI 10.1093/nar/17.15.6419

SCHUBERT D, 1974, NATURE, V249, P224, DOI 10.1038/249224a0

SCHWAB RS, 1969, J AMER MED ASSOC, V208, P1168, DOI 10.1001/jama.208.7.1168

Takeda K, 2009, J MOL NEUROSCI, V39, P380, DOI 10.1007/s12031-009-9224-5

Tampi Rajesh R, 2007, Neuropsychiatr Dis Treat, V3, P245, DOI 10.2147/nedt.2007.3.2.245

Thomas SJ, 2009, CLIN INTERV AGING, V4, P367

TOIDE K, 1990, PHARM RES-DORDR, V7, P670, DOI 10.1023/A:1015890800131

Tufekci KU, 2011, PARKINSONS DIS-US, V2011, DOI 10.4061/2011/487450

Vallabhapurapu S, 2009, ANNU REV IMMUNOL, V27, P693, DOI 10.1146/annurev.immunol.021908.132641

Waak J, 2009, FASEB J, V23, P2478, DOI 10.1096/fj.08-125153

Whitton PS, 2007, BRIT J PHARMACOL, V150, P963, DOI 10.1038/sj.bjp.0707167

Wilms H, 2007, CURR PHARM DESIGN, V13, P1925

Wu HM, 2009, NEUROPSYCHOPHARMACOL, V34, P2344, DOI 10.1038/npp.2009.64

YAHR MD, 1972, NEW ENGL J MED, V287, P20, DOI 10.1056/NEJM197207062870105

Zheng LT, 2008, J NEUROCHEM, V107, P1225, DOI 10.1111/j.1471-4159.2008.05675.x

NR 64

TC 40

Z9 43

U1 2

U2 33

PU ELSEVIER SCIENCE INC

PI NEW YORK

PA 360 PARK AVE SOUTH, NEW YORK, NY 10010-1710 USA

SN 0197-4580

J9 NEUROBIOL AGING

JI Neurobiol. Aging

PD SEP

PY 2012

VL 33

IS 9

BP 2145

EP 2159

DI 10.1016/j.neurobiolaging.2011.08.011

PG 15

WC Geriatrics & Gerontology; Neurosciences

WE Science Citation Index Expanded (SCI-EXPANDED)

SC Geriatrics & Gerontology; Neurosciences & Neurology

GA 981TW

UT WOS:000306994600027

PM 22035588

DA 2024-08-12

ER

PT J

AU de Souza, FRO

Ribeiro, FM

Lima, PMD

AF Oliveira de Souza, Filipe Resende

Ribeiro, Fabiola Mara

d'Almeida Lima, Patricia Maria

TI Implications of VIP and PACAP in Parkinson's Disease: What do we Know So

Far?

SO CURRENT MEDICINAL CHEMISTRY

LA English

DT Review

DE Vasoactive intestinal peptide (VIP); pituitary adenylate-cyclase

activating polypeptide (PACAP); Parkinson's disease; neuroinflammation;

oxidative stress; neuropeptides; neurodegenerative disease

ID VASOACTIVE-INTESTINAL-PEPTIDE; CYCLASE-ACTIVATING POLYPEPTIDE; SH-SY5Y

CELLS IMPLICATION; ADENYLATE-CYCLASE; TNF-ALPHA; T(H)17 CELLS; MOUSE

MODEL; RECEPTOR; BRAIN; MICROGLIA

AB Background: Parkinson's disease is one of the most common neurodegenerative disorders and although its aetiology is not yet fully understood, neuroinflammation has been identified as a key factor in the progression of the disease. Vasoactive intestinal peptide and pituitary adenylate-cyclase activating polypeptide are two neuropeptides that exhibit anti-inflammatory and neuroprotective properties, modulating the production of cytokines and chemokines and the behaviour of immune cells. However, the role of chemokines and cytokines modulated by the endogenous receptors of the peptides varies according to the stage of the disease.

Methods: We present an overview of the relationship between some cytokines and chemokines with vasoactive intestinal peptide, pituitary adenylate cyclase activating polypeptide and their endogenous receptors in the context of Parkinson's disease neuroinflammation and oxidative stress, as well as the modulation of microglial cells by the peptides in this context.

Results: The two peptides exhibit neuroprotective and anti-inflammatory properties in models of Parkinson's disease, as they ameliorate cognitive functions, decrease the level of neuroinflammation and promote dopaminergic neuronal survival. The peptides have been tested in a variety of in vivo and in vitro models of Parkinson's disease, demonstrating the potential for therapeutic application.

Conclusion: More studies are needed to establish the clinical use of vasoactive intestinal peptide and pituitary adenylate cyclase activating polypeptide as safe candidates for treating Parkinson's disease, as the use of the peptides in different stages of the disease could produce different results concerning effectiveness.

C1 [Oliveira de Souza, Filipe Resende; d'Almeida Lima, Patricia Maria] Univ Fed Sao Joao del Rei, Dept Nat Sci, Lab Immunol & Microbiol, Praca Dom Helvecio 74, BR-36301160 Sao Joao Del Rei, MG, Brazil.

[Ribeiro, Fabiola Mara] Univ Fed Minas Gerais, Dept Biochem & Immunol, Lab Neurobiochem, Ave Antonio Carlos 6627, BR-31270901 Belo Horizonte, MG, Brazil.

C3 Universidade Federal de Sao Joao del-Rei; Universidade Federal de Minas

Gerais

RP Lima, PMD (corresponding author), Univ Fed Sao Joao del Rei, Dept Nat Sci, Lab Immunol & Microbiol, Praca Dom Helvecio 74, BR-36301160 Sao Joao Del Rei, MG, Brazil.; Ribeiro, FM (corresponding author), Univ Fed Minas Gerais, Dept Biochem & Immunol, Lab Neurobiochem, Ave Antonio Carlos 6627, BR-31270901 Belo Horizonte, MG, Brazil.

EM patlima@ufsj.edu.br; fmribeiro2013@gmail.com

RI Lima, Patrícia Maria/JBJ-2997-2023; Ribeiro, Fabiola/E-8831-2013

OI Ribeiro, Fabiola/0000-0001-7042-9433; , Filipe/0000-0003-2118-6169

FU Fundacao de Amparo a Pesquisa do Estado de Minas Gerais (FAPEMIG)

[5.270/15, 13553]

FX Filipe Resende Oliveira de Souza recieved financial support from

Fundacao de Amparo a Pesquisa do Estado de Minas Gerais (FAPEMIG), Grant

number 5.270/15, identifier number 13553.

CR Armstrong BD, 2008, NEUROSCIENCE, V151, P63, DOI 10.1016/j.neuroscience.2007.09.084

Beringer A, 2016, TRENDS MOL MED, V22, P230, DOI 10.1016/j.molmed.2016.01.001

Bisaglia M, 2007, J BIOL CHEM, V282, P15597, DOI 10.1074/jbc.M610893200

Brifault C, 2015, STROKE, V46, P520, DOI 10.1161/STROKEAHA.114.006864

Brown D, 2014, NEUROTOX RES, V26, P230, DOI 10.1007/s12640-014-9468-x

Brown D, 2013, J MOL NEUROSCI, V50, P600, DOI 10.1007/s12031-013-0015-7

Brundin P, 2017, J NEUROSCI, V37, P9808, DOI 10.1523/JNEUROSCI.1788-16.2017

Bruning JM, 2019, CELL CHEM BIOL, V26, P674, DOI 10.1016/j.chembiol.2019.02.002

Caggiu E, 2019, FRONT NEUROL, V10, DOI 10.3389/fneur.2019.00122

Campos-Acuña J, 2019, FRONT IMMUNOL, V10, DOI 10.3389/fimmu.2019.00239

Castaño A, 2002, J NEUROCHEM, V81, P150, DOI 10.1046/j.1471-4159.2002.00799.x

Chen L, 2015, INT J ONCOL, V47, P1361, DOI 10.3892/ijo.2015.3126

Chen S, 2019, FRONT NEUROL, V10, DOI 10.3389/fneur.2019.01231

COUVINEAU A, 1994, BIOCHEM BIOPH RES CO, V200, P769, DOI 10.1006/bbrc.1994.1517

Couvineau A, 2012, BRIT J PHARMACOL, V166, P42, DOI 10.1111/j.1476-5381.2011.01676.x

Cui X, 2013, PHARMAZIE, V68, P69, DOI 10.1691/ph.2013.1148

de Molliens MP, 2019, BBA-GEN SUBJECTS, V1863, DOI 10.1016/j.bbagen.2019.07.007

de Vera IMS, 2016, ACS CHEM BIOL, V11, P1795, DOI 10.1021/acschembio.6b00037

Delgado M, 1999, J IMMUNOL, V162, P1707

Delgado M, 2003, FASEB J, V17, P1922, DOI 10.1096/fj.02-1029fje

Delgado M, 2003, FASEB J, V17, P944, DOI 10.1096/fj.02-0799fje

Delgado M, 2003, J BIOL CHEM, V278, P27620, DOI 10.1074/jbc.M303199200

Delgado M, 2003, BIOCHEM BIOPH RES CO, V301, P825, DOI 10.1016/S0006-291X(03)00059-7

Delgado M, 2003, J LEUKOCYTE BIOL, V73, P155, DOI 10.1189/jlb.0702372

Delgado M, 2004, PHARMACOL REV, V56, P249, DOI 10.1124/pr.56.2.7

Delgado M, 2001, J BIOL CHEM, V276, P369, DOI 10.1074/jbc.M006923200

Delgado M, 2013, AMINO ACIDS, V45, P25, DOI 10.1007/s00726-011-1184-8

DESBUQUOIS B, 1973, BIOCHEM BIOPH RES CO, V53, P1187, DOI 10.1016/0006-291X(73)90590-1

Dickson L, 2006, NEUROPHARMACOLOGY, V51, P1086, DOI 10.1016/j.neuropharm.2006.07.017

Dickson L, 2009, PHARMACOL THERAPEUT, V121, P294, DOI 10.1016/j.pharmthera.2008.11.006

Dogrukol-Ak D, 2003, PEPTIDES, V24, P437, DOI 10.1016/S0196-9781(03)00059-7

DOMSCHKE S, 1978, GUT, V19, P1049, DOI 10.1136/gut.19.11.1049

Dufes C, 2003, INT J PHARMACEUT, V255, P87, DOI 10.1016/S0378-5173(03)00039-5

Dugger BN, 2017, CSH PERSPECT BIOL, V9, DOI 10.1101/cshperspect.a028035

Dutta D, 2019, NEUROBIOL DIS, V132, DOI 10.1016/j.nbd.2019.104575

Fernandes A, 2014, CELL MOL LIFE SCI, V71, P3969, DOI 10.1007/s00018-014-1670-8

Ganea D, 2015, ACTA PHYSIOL, V213, P442, DOI 10.1111/apha.12427

Gelders G, 2018, J IMMUNOL RES, V2018, DOI 10.1155/2018/4784268

Giancola F, 2017, NEUROGASTROENT MOTIL, V29, DOI 10.1111/nmo.12995

Gonzalez-Rey E, 2008, BRAIN BEHAV IMMUN, V22, P35, DOI 10.1016/j.bbi.2007.07.004

Ha SJ, 1999, J IMMUNOL, V163, P2902

Hammond SL, 2018, J PHARMACOL EXP THER, V365, P636, DOI 10.1124/jpet.117.246389

Hamnett R, 2019, NAT COMMUN, V10, DOI 10.1038/s41467-019-08427-3

Harmar AJ, 2004, ENDOCRINOLOGY, V145, P1203, DOI 10.1210/en.2003-1058

Hedya SA, 2018, MOL NEUROBIOL, V55, P7579, DOI 10.1007/s12035-018-0923-1

Henle F, 2006, J BIOL CHEM, V281, P24955, DOI 10.1074/jbc.M604114200

Henning RJ, 2001, CARDIOVASC RES, V49, P27, DOI 10.1016/S0008-6363(00)00229-7

Hirabayashi T, 2018, J HEADACHE PAIN, V19, DOI 10.1186/s10194-018-0855-1

JEGOU S, 1988, PEPTIDES, V9, P787, DOI 10.1016/0196-9781(88)90123-4

Joers V, 2017, PROG NEUROBIOL, V155, P57, DOI 10.1016/j.pneurobio.2016.04.006

Kuhn DM, 1999, J NEUROCHEM, V73, P1309, DOI 10.1046/j.1471-4159.1999.0731309.x

LARSSON LI, 1976, P NATL ACAD SCI USA, V73, P3197, DOI 10.1073/pnas.73.9.3197

Lee EH, 2014, BMB REP, V47, P369, DOI 10.5483/BMBRep.2014.47.7.086

Liang YZ, 2018, J BIOMED SCI, V25, DOI 10.1186/s12929-018-0410-z

Liu JQ, 2019, BRAIN RES BULL, V152, P11, DOI 10.1016/j.brainresbull.2019.05.020

Liu Z, 2019, BRAIN BEHAV IMMUN, V81, P630, DOI 10.1016/j.bbi.2019.07.026

Liu Z, 2017, MOL NEUROBIOL, V54, P7762, DOI 10.1007/s12035-016-0249-9

Luo Y, 2007, EXP NEUROL, V203, P221, DOI 10.1016/j.expneurol.2006.08.005

LUTZ EM, 1993, FEBS LETT, V334, P3, DOI 10.1016/0014-5793(93)81668-P

Maasz G, 2017, DIS MODEL MECH, V10, P127, DOI 10.1242/dmm.027185

MacKenzie CJ, 2001, ENDOCRINOLOGY, V142, P1209, DOI 10.1210/en.142.3.1209

Miossec P, 2012, NAT REV DRUG DISCOV, V11, P763, DOI 10.1038/nrd3794

MIYATA A, 1989, BIOCHEM BIOPH RES CO, V164, P567, DOI 10.1016/0006-291X(89)91757-9

Mocellin S, 2004, GENES IMMUN, V5, P621, DOI 10.1038/sj.gene.6364135

MOGI M, 1994, NEUROSCI LETT, V180, P147, DOI 10.1016/0304-3940(94)90508-8

Mosser DM, 2008, IMMUNOL REV, V226, P205, DOI 10.1111/j.1600-065X.2008.00706.x

Nasrolahi A, 2019, REV NEUROSCIENCE, V30, P709, DOI 10.1515/revneuro-2018-0105

Nonaka N, 2012, PEPTIDES, V36, P168, DOI 10.1016/j.peptides.2012.05.021

Offen D, 2000, BRAIN RES, V854, P257, DOI 10.1016/S0006-8993(99)02375-6

Ohtsuka M, 2008, BIOCHEM BIOPH RES CO, V369, P1144, DOI 10.1016/j.bbrc.2008.03.028

Olson KE, 2016, NEUROTHERAPEUTICS, V13, P635, DOI 10.1007/s13311-016-0449-z

Olson KE, 2015, J NEUROSCI, V35, P16463, DOI 10.1523/JNEUROSCI.2131-15.2015

Panicker N, 2019, J EXP MED, V216, P1411, DOI 10.1084/jem.20182191

Patel MM, 2017, CNS DRUGS, V31, P109, DOI 10.1007/s40263-016-0405-9

Paul KC, 2018, MECH AGEING DEV, V173, P1, DOI 10.1016/j.mad.2018.04.004

Popichak KA, 2018, MOL PHARMACOL, V94, P1174, DOI 10.1124/mol.118.112631

Qian Y, 2020, CNS NEUROSCI THER, V26, P55, DOI 10.1111/cns.13149

Ransohoff RM, 2015, NEUROTHERAPEUTICS, V12, P896, DOI 10.1007/s13311-015-0385-3

Reglödi D, 2004, BEHAV BRAIN RES, V151, P303, DOI 10.1016/j.bbr.2003.09.007

Reglodi D, 2012, NEUROPEPTIDES, V46, P61, DOI 10.1016/j.npep.2011.05.001

Rentzos M, 2009, ACTA NEUROL SCAND, V119, P332, DOI 10.1111/j.1600-0404.2008.01103.x

Rousselet E, 2002, EXP NEUROL, V177, P183, DOI 10.1006/exnr.2002.7960

Sanchez-Guajardo V, 2013, ASN NEURO, V5, P113, DOI 10.1042/AN20120066

Schropp V, 2019, J NEUROINFLAMM, V16, DOI 10.1186/s12974-019-1500-x

Shivers KY, 2014, BBA-MOL BASIS DIS, V1842, P1707, DOI 10.1016/j.bbadis.2014.06.020

Shrivastava R, 2019, LIFE SCI, V224, P222, DOI 10.1016/j.lfs.2019.03.062

Starr CG, 2018, PEPTIDES, V104, P35, DOI 10.1016/j.peptides.2018.04.006

Stephenson J, 2018, IMMUNOLOGY, V154, P204, DOI 10.1111/imm.12922

Subhramanyam CS, 2019, SEMIN CELL DEV BIOL, V94, P112, DOI 10.1016/j.semcdb.2019.05.004

Sulzer D, 2017, NATURE, V546, P656, DOI 10.1038/nature22815

Tan YV, 2015, BRAIN BEHAV IMMUN, V44, P167, DOI 10.1016/j.bbi.2014.09.020

Temerozo JR, 2018, FRONT IMMUNOL, V9, DOI 10.3389/fimmu.2018.01336

Tulisiak CT, 2019, PROG MOL BIOL TRANSL, V168, P299, DOI 10.1016/bs.pmbts.2019.06.002

USDIN TB, 1994, ENDOCRINOLOGY, V135, P2662, DOI 10.1210/en.135.6.2662

Vaudry D, 2000, PHARMACOL REV, V52, P269

Vertongen P, 1998, ANN NY ACAD SCI, V865, P412, DOI 10.1111/j.1749-6632.1998.tb11206.x

Wang G, 2008, NEUROPEPTIDES, V42, P267, DOI 10.1016/j.npep.2008.03.002

Wei XB, 2016, MOL NEUROBIOL, V53, P5876, DOI 10.1007/s12035-015-9477-7

Xu CL, 2017, MOLECULES, V22, DOI 10.3390/molecules22111963

Yadav M, 2008, J IMMUNOL, V180, P2772, DOI 10.4049/jimmunol.180.5.2772

Yang HM, 2017, FRONT AGING NEUROSCI, V9, DOI 10.3389/fnagi.2017.00193

Yelkenli IH, 2016, J MOL NEUROSCI, V59, P280, DOI 10.1007/s12031-016-0757-0

Yilmaz R, 2018, FRONT NEUROL, V9, DOI 10.3389/fneur.2018.01123

Zetterstrom RH, 1996, MOL BRAIN RES, V41, P111, DOI 10.1016/0169-328X(96)00074-5

Zhao Y, 2017, BLOOD, V130, P1995, DOI 10.1182/blood-2017-04-778779

NR 105

TC 8

Z9 9

U1 5

U2 20

PU BENTHAM SCIENCE PUBL LTD

PI SHARJAH

PA EXECUTIVE STE Y-2, PO BOX 7917, SAIF ZONE, 1200 BR SHARJAH, U ARAB

EMIRATES

SN 0929-8673

EI 1875-533X

J9 CURR MED CHEM

JI Curr. Med. Chem.

PY 2021

VL 28

IS 9

BP 1703

EP 1715

DI 10.2174/0929867327666200320162436

PG 13

WC Biochemistry & Molecular Biology; Chemistry, Medicinal; Pharmacology &

Pharmacy

WE Science Citation Index Expanded (SCI-EXPANDED)

SC Biochemistry & Molecular Biology; Pharmacology & Pharmacy

GA RP2RH

UT WOS:000641580700003

PM 32196442

DA 2024-08-12

ER

PT J

AU Leonoudakis, D

Rane, A

Angeli, S

Lithgow, GJ

Andersen, JK

Chinta, SJ

AF Leonoudakis, Dmitri

Rane, Anand

Angeli, Suzanne

Lithgow, Gordon J.

Andersen, Julie K.

Chinta, Shankar J.

TI Anti-Inflammatory and Neuroprotective Role of Natural Product Securinine

in Activated Glial Cells: Implications for Parkinson's Disease

SO MEDIATORS OF INFLAMMATION

LA English

DT Article

ID MICROGLIAL ACTIVATION; NEUROTOXICITY; INFLAMMATION; INHIBITORS;

APOPTOSIS; PROTEINS; CANCER; HERBS

AB Glial activation and subsequent release of neurotoxic proinflammatory factors are believed to play an important role in the pathogenesis of several neurological disorders including Parkinson's disease (PD). Inhibition of glial activation and inflammatory processes may represent a therapeutic target to alleviate neurodegeneration. Securinine, a major natural alkaloid product from the root of the plant Securinega suffruticosa, has been reported to have potent biological activity and is used in the treatment of neurological conditions such as amyotrophic lateral sclerosis, poliomyelitis, and multiple sclerosis. In this study, we explored the underlying mechanisms of neuroprotection elicited by securinine, particularly its anti-inflammatory effects in glial cells. Our results demonstrate that securinine significantly and dose-dependently suppressed the nitric oxide production in microglia and astrocytic cultures. In addition, securinine inhibited the activation of the inflammatory mediator NF-kappa B, as well asmitogen-activated protein kinases in lipopolysaccharide-(LPS-) stimulated BV2 cells. Additionally, securinine also inhibited interferon-gamma-(IFN-gamma-) induced nitric oxide levels and iNOS mRNA expression. Furthermore, conditioned media (CM) from securinine pretreated BV2 cells significantly reduced mesencephalic dopaminergic neurotoxicity compared withCMfromLPS stimulated microglia. These findings suggest that securininemay be a potential candidate for the treatment of neurodegenerative disease related to neuroinflammation.

C1 [Leonoudakis, Dmitri; Rane, Anand; Angeli, Suzanne; Lithgow, Gordon J.; Andersen, Julie K.; Chinta, Shankar J.] Buck Inst Res Aging, 8001 Redwood Blvd, Novato, CA 94945 USA.

[Chinta, Shankar J.] Touro Univ, Calif Coll Pharm, 1310 Club Dr, Vallejo, CA 94592 USA.

C3 Buck Institute for Research on Aging; Touro University California

RP Chinta, SJ (corresponding author), Buck Inst Res Aging, 8001 Redwood Blvd, Novato, CA 94945 USA.; Chinta, SJ (corresponding author), Touro Univ, Calif Coll Pharm, 1310 Club Dr, Vallejo, CA 94592 USA.

EM schinta@buckinstitute.org

OI Angeli, Suzanne/0000-0002-6229-9648; Andersen, Julie/0000-0003-1324-4875

FU National Institutes of Health Grants [PO1 AG025901, R01AG029631-08]

FX This work was supported by National Institutes of Health Grants PO1

AG025901 to Julie K. Andersen and R01AG029631-08 to Gordon J. Lithgow.

The authors thank Subramanian Rajagopalan for assistance with primary

culture experiments.

CR Akira S, 2001, NAT IMMUNOL, V2, P675, DOI 10.1038/90609

Auriel Eitan, 2014, Handb Clin Neurol, V119, P577, DOI 10.1016/B978-0-7020-4086-3.00038-2

BEUTLER JA, 1985, BRAIN RES, V330, P135, DOI 10.1016/0006-8993(85)90014-9

Blanchette J, 2003, IMMUNOLOGY, V108, P513, DOI 10.1046/j.1365-2567.2003.01620.x

Block ML, 2007, NAT REV NEUROSCI, V8, P57, DOI 10.1038/nrn2038

BURAVTSEVA G R, 1958, Farmakol Toksikol, V21, P7

Chen Liang-Wei, 2007, CNS & Neurological Disorders-Drug Targets, V6, P273

Chinta SJ, 2012, PARKINSONS DIS-US, V2012, DOI 10.1155/2012/364684

Chinta SJ, 2013, NEUROTOX RES, V23, P145, DOI 10.1007/s12640-012-9328-5

Chirkin Eqor, 2015, Alkaloids Chem Biol, V74, P1

Choi DK, 2011, MOLECULES, V16, P1021, DOI 10.3390/molecules16021021

Copperman R, 1973, Pa Med, V76, P36

Das M, 2012, J NEUROINFLAMM, V9, DOI 10.1186/1742-2094-9-236

Dheen ST, 2007, CURR MED CHEM, V14, P1189, DOI 10.2174/092986707780597961

Glass CK, 2010, CELL, V140, P918, DOI 10.1016/j.cell.2010.02.016

Jäger AK, 2011, MOLECULES, V16, P1471, DOI 10.3390/molecules16021471

Kleinert H, 2004, EUR J PHARMACOL, V500, P255, DOI 10.1016/j.ejphar.2004.07.030

Kulkarni AP, 2005, ANN NY ACAD SCI, V1056, P413, DOI 10.1196/annals.1352.020

Li CQ, 2002, P NATL ACAD SCI USA, V99, P10364, DOI 10.1073/pnas.162356399

Li HF, 2014, REJUV RES, V17, P201, DOI 10.1089/rej.2013.1513

Liu DX, 2011, NEUROPHARMACOLOGY, V61, P592, DOI 10.1016/j.neuropharm.2011.04.033

Lubick K, 2007, J LEUKOCYTE BIOL, V82, P1062, DOI 10.1189/jlb.0407255

Lull ME, 2010, NEUROTHERAPEUTICS, V7, P354, DOI 10.1016/j.nurt.2010.05.014

Minghetti L, 2005, CURR OPIN NEUROL, V18, P315, DOI 10.1097/01.wco.0000169752.54191.97

Perry VH, 2014, NAT REV NEUROL, V10, P217, DOI 10.1038/nrneurol.2014.38

Prinz M, 2017, NAT NEUROSCI, V20, P136, DOI 10.1038/nn.4475

Rajagopalan S, 2016, J NEUROSCI, V36, P1086, DOI 10.1523/JNEUROSCI.3117-15.2016

Rana S, 2010, FASEB J, V24, P2126, DOI 10.1096/fj.09-148999

Ransohoff RM, 2009, ANNU REV IMMUNOL, V27, P119, DOI 10.1146/annurev.immunol.021908.132528

Shimazawa M, 2005, EVID-BASED COMPL ALT, V2, P201, DOI 10.1093/ecam/neh078

Shipman M, 2012, PLOS ONE, V7, DOI 10.1371/journal.pone.0041278

Tamashiro TT, 2012, JOVE-J VIS EXP, DOI 10.3791/3814

Viatour P, 2005, TRENDS BIOCHEM SCI, V30, P43, DOI 10.1016/j.tibs.2004.11.009

Wang S, 2011, NEUROSCIENCE, V185, P150, DOI 10.1016/j.neuroscience.2011.04.005

Xu L, 2004, NEUROL RES, V26, P792, DOI 10.1179/016164104225014148

Yuste JE, 2015, FRONT CELL NEUROSCI, V9, DOI 10.3389/fncel.2015.00322

NR 36

TC 45

Z9 47

U1 0

U2 14

PU HINDAWI LTD

PI LONDON

PA ADAM HOUSE, 3RD FLR, 1 FITZROY SQ, LONDON, W1T 5HF, United Kingdom

SN 0962-9351

EI 1466-1861

J9 MEDIAT INFLAMM

JI Mediat. Inflamm.

PY 2017

VL 2017

AR 8302636

DI 10.1155/2017/8302636

PG 11

WC Cell Biology; Immunology

WE Science Citation Index Expanded (SCI-EXPANDED)

SC Cell Biology; Immunology

GA ER1CS

UT WOS:000398527500001

PM 28473732

OA Green Published, gold

DA 2024-08-12

ER

PT J

AU Houser, MC

Caudle, WM

Chang, JJ

Kannarkat, GT

Yang, Y

Kelly, SD

Oliver, D

Joers, V

Shannon, KM

Keshavarzian, A

Tansey, MG

AF Houser, Madelyn C.

Caudle, W. Michael

Chang, Jianjun

Kannarkat, George T.

Yang, Yuan

Kelly, Sean D.

Oliver, Danielle

Joers, Valerie

Shannon, Kathleen M.

Keshavarzian, Ali

Tansey, Malu Gamez

TI Experimental colitis promotes sustained, sex-dependent,

T-cell-associated neuroinflammation and parkinsonian neuropathology

SO ACTA NEUROPATHOLOGICA COMMUNICATIONS

LA English

DT Article

DE Parkinson's disease; Intestine; Gut; Inflammation; Immune; Colitis;

Neurodegeneration; MPTP; DSS

ID VESICULAR MONOAMINE TRANSPORTER; MOUSE MODEL; TYROSINE-HYDROXYLASE;

COLONIC INFLAMMATION; INTERFERON-GAMMA; ALPHA-SYNUCLEIN; MURINE MODEL;

KAPPA-B; DISEASE; PROTEIN

AB Background: The etiology of sporadic Parkinson's disease (PD) remains uncertain, but genetic, epidemiological, and physiological overlap between PD and inflammatory bowel disease suggests that gut inflammation could promote dysfunction of dopamine-producing neurons in the brain. Mechanisms behind this pathological gut-brain effect and their interactions with sex and with environmental factors are not well understood but may represent targets for therapeutic intervention.

Methods: We sought to identify active inflammatory mechanisms which could potentially contribute to neuroinflammation and neurological disease in colon biopsies and peripheral blood immune cells from PD patients. Then, in mouse models, we assessed whether dextran sodium sulfate-mediated colitis could exert lingering effects on dopaminergic pathways in the brain and whether colitis increased vulnerability to a subsequent exposure to the dopaminergic neurotoxicant 1-methyl-4-phenyl-1,2,3,6-tetrahydropyridine (MPTP). We assessed the involvement of inflammatory mechanisms identified in the PD patients in colitis-related neurological dysfunction in male and female mice, utilizing mice lacking the Regulator of G-Protein Signaling 10 (RGS10)-an inhibitor of nuclear factor kappa B (NF kappa B)-to model enhanced NF kappa B activity, and mice in which CD8(+) T-cells were depleted.

Results: High levels of inflammatory markers including CD8B and NF kappa B p65 were found in colon biopsies from PD patients, and reduced levels of RGS10 were found in immune cells in the blood. Male mice that experienced colitis exhibited sustained reductions in tyrosine hydroxylase but not in dopamine as well as sustained CD8(+) T-cell infiltration and elevated Ifng expression in the brain. CD8(+) T-cell depletion prevented colitis-associated reductions in dopaminergic markers in males. In both sexes, colitis potentiated the effects of MPTP. RGS10 deficiency increased baseline intestinal inflammation, colitis severity, and neuropathology.

Conclusions: This study identifies peripheral inflammatory mechanisms in PD patients and explores their potential to impact central dopaminergic pathways in mice. Our findings implicate a sex-specific interaction between gastrointestinal inflammation and neurologic vulnerability that could contribute to PD pathogenesis, and they establish the importance of CD8(+) T-cells in this process in male mice.

C1 [Houser, Madelyn C.; Chang, Jianjun; Kannarkat, George T.; Yang, Yuan; Kelly, Sean D.; Oliver, Danielle; Joers, Valerie; Tansey, Malu Gamez] Emory Univ, Sch Med, Dept Physiol, Atlanta, GA 30322 USA.

[Houser, Madelyn C.] Emory Univ, Nell Hodgson Woodruff Sch Nursing, Atlanta, GA 30322 USA.

[Caudle, W. Michael; Kelly, Sean D.] Emory Univ, Rollins Sch Publ Hlth, Dept Environm Hlth, Atlanta, GA 30322 USA.

[Kannarkat, George T.] Johns Hopkins Med, Dept Neurol, Baltimore, MD USA.

[Shannon, Kathleen M.] Univ Wisconsin, Dept Neurol, Sch Med & Publ Hlth, Madison, WI 53706 USA.

[Keshavarzian, Ali] Rush Med Coll, Dept Internal Med, Chicago, IL 60612 USA.

[Joers, Valerie; Tansey, Malu Gamez] Univ Florida, Ctr Translat Res Neurodegenerat Dis, Dept Neurosci & Neurol, Coll Med, Gainesville, FL 32610 USA.

C3 Emory University; Emory University; Emory University; Rollins School

Public Health; Johns Hopkins University; Johns Hopkins Medicine;

University of Wisconsin System; University of Wisconsin Madison; Rush

University; State University System of Florida; University of Florida

RP Tansey, MG (corresponding author), Emory Univ, Sch Med, Dept Physiol, Atlanta, GA 30322 USA.; Tansey, MG (corresponding author), Univ Florida, Ctr Translat Res Neurodegenerat Dis, Dept Neurosci & Neurol, Coll Med, Gainesville, FL 32610 USA.

EM mgtansey@UFL.edu

RI Kelly, Sean/W-6808-2019; Houser, Madelyn/AAK-8387-2021; Kannarkat,

George/ABB-6064-2021

OI Houser, Madelyn/0000-0002-4049-9876; Shannon,

Kathleen/0000-0001-5171-6779; Kannarkat, George/0000-0003-4428-1820

FU National Center for Georgia Clinical & Translational Science Alliance of

the National Institutes of Health [UL1TR002378]; NIH [5R01NS092122,

1RF1AG057247]; National Science Foundation [DGE-1444932]

FX Funding was provided by the National Center for Georgia Clinical &

Translational Science Alliance of the National Institutes of Health

under Award Number UL1TR002378 and by NIH grants 5R01NS092122 (MGT) and

1RF1AG057247 (MGT). This material is also based on work supported by the

National Science Foundation under Grant No. DGE-1444932 (MCH)"The

content is solely the responsibility of the authors and does not

necessarily reflect the official views of the National Institutes of

Health or the National Science Foundation. The funding agencies had no

role in the design of the study, the collection, analysis, and

interpretation of data, or in the writing of the manuscript.

CR Alqinyah M, 2018, MOL PHARMACOL, V94, P1103, DOI 10.1124/mol.118.111674

Alqinyah M, 2017, MOL PHARMACOL, V91, P197, DOI 10.1124/mol.116.106963

Barnum CJ, 2008, NEUROSCIENCE, V156, P30, DOI 10.1016/j.neuroscience.2008.07.016

Barnum CJ, 2014, J PARKINSON DIS, V4, P349, DOI 10.3233/JPD-140410

Becker A, 2019, PARKINSONISM RELAT D, V60, P43, DOI 10.1016/j.parkreldis.2018.09.032

Breiman L, 2001, MACH LEARN, V45, P5, DOI 10.1023/A:1010933404324

Brochard V, 2009, J CLIN INVEST, V119, P182, DOI 10.1172/JCI36470

Camacho-Soto A, 2018, PARKINSONISM RELAT D, V50, P23, DOI 10.1016/j.parkreldis.2018.02.008

Christensen Rune Haubo Bojesen, 2023, CRAN

Cook DA, 2017, NPJ PARKINSONS DIS, V3, DOI 10.1038/s41531-017-0010-8

Dai SX, 2017, CLIN RES HEPATOL GAS, V41, P693, DOI 10.1016/j.clinre.2017.03.009

Darwich L, 2009, IMMUNOLOGY, V126, P386, DOI 10.1111/j.1365-2567.2008.02905.x

Rodrigues MED, 2017, BRAIN BEHAV IMMUN, V59, P158, DOI 10.1016/j.bbi.2016.08.021

de Waal GM, 2018, SCI REP-UK, V8, DOI 10.1038/s41598-018-35009-y

Devos D, 2013, NEUROBIOL DIS, V50, P42, DOI 10.1016/j.nbd.2012.09.007

Ehirchiou D, 2007, J EXP MED, V204, P1519, DOI 10.1084/jem.20062292

Eichele DD, 2017, WORLD J GASTROENTERO, V23, P6016, DOI 10.3748/wjg.v23.i33.6016

Ellrichmann G, 2012, J NEUROINFLAMM, V9, DOI 10.1186/1742-2094-9-15

Forsyth CB, 2011, PLOS ONE, V6, DOI 10.1371/journal.pone.0028032

Fox J., 2011, MULTIVARIATE LINEAR

Freyaldenhoven TE, 1996, BRAIN RES, V735, P232, DOI 10.1016/0006-8993(96)00598-7

Gadotti VM, 2019, ISCIENCE, V16, P12, DOI 10.1016/j.isci.2019.05.012

Gao Y, 2018, B EXP BIOL MED+, V165, P503, DOI 10.1007/s10517-018-4204-9

Garrido-Gil P, 2018, MOL NEUROBIOL, V55, P7297, DOI 10.1007/s12035-018-0937-8

Gibbons DL, 2011, J IMMUNOL, V187, P2067, DOI 10.4049/jimmunol.1100833

Guo YM, 2015, MEDIAT INFLAMM, V2015, DOI 10.1155/2015/843089

Hamza TH, 2010, NAT GENET, V42, P781, DOI 10.1038/ng.642

Harada K, 1996, J NEUROCHEM, V67, P629

Henry Lionel, 2022, CRAN

Houser MC, 2018, MOVEMENT DISORD, V33, P793, DOI 10.1002/mds.27326

Houser MC, 2017, NPJ PARKINSONS DIS, V3, DOI 10.1038/s41531-016-0002-0

Hui KY, 2018, SCI TRANSL MED, V10, DOI 10.1126/scitranslmed.aai7795

ICHINOSE H, 1994, J NEURAL TRANSM-PARK, V8, P149, DOI 10.1007/BF02250926

Ishibashi K, 2014, J NEUROL SCI, V339, P207, DOI 10.1016/j.jns.2014.01.015

Joling M, 2019, NEUROIMAGE-CLIN, V22, DOI 10.1016/j.nicl.2019.101755

Joniec I, 2009, BRAIN RES, V1261, P7, DOI 10.1016/j.brainres.2008.12.081

Kannarkat GT, 2015, NPJ PARKINSON DIS, V1, DOI 10.1038/npjparkd.2015.2

Kappeler A, 2000, HISTOL HISTOPATHOL, V15, P167, DOI 10.14670/HH-15.167

Keshavarzian A, 2015, MOVEMENT DISORD, V30, P1351, DOI 10.1002/mds.26307

Kido J, 2003, J PERIODONTAL RES, V38, P557, DOI 10.1034/j.1600-0765.2003.00691.x

Kishimoto Y, 2019, NEUROMOL MED, V21, P239, DOI 10.1007/s12017-019-08539-5

Kitajima S, 1999, J VET MED SCI, V61, P67, DOI 10.1292/jvms.61.67

Kyrkanides S, 2008, J NEUROIMMUNOL, V203, P50, DOI 10.1016/j.jneuroim.2008.06.024

Labadorf A, 2018, FRONT MOL NEUROSCI, V10, DOI 10.3389/fnmol.2017.00430

Lee JK, 2008, J NEUROSCI, V28, P8517, DOI 10.1523/JNEUROSCI.1806-08.2008

Lee JK, 2015, PROG MOL BIOL TRANSL, V133, P153, DOI 10.1016/bs.pmbts.2015.01.005

Lee JK, 2013, PLOS ONE, V8, DOI 10.1371/journal.pone.0081785

Lee JK, 2012, J NEUROCHEM, V122, P333, DOI 10.1111/j.1471-4159.2012.07780.x

Lee JK, 2011, J NEUROSCI, V31, P11879, DOI 10.1523/JNEUROSCI.1002-11.2011

Liddle RA, 2018, BRAIN RES, V1693, P201, DOI 10.1016/j.brainres.2018.01.010

Lin JC, 2016, INFLAMM BOWEL DIS, V22, P1049, DOI 10.1097/MIB.0000000000000735

Liu T, 2017, SIGNAL TRANSDUCT TAR, V2, DOI 10.1038/sigtrans.2017.23

Lohr KM, 2014, P NATL ACAD SCI USA, V111, P9977, DOI 10.1073/pnas.1402134111

Marras C, 2018, NPJ PARKINSONS DIS, V4, DOI 10.1038/s41531-018-0058-0

Mccoy MK, 2011, ADV EXP MED BIOL, V691, P539, DOI 10.1007/978-1-4419-6612-4_56

Miller GW, 1999, EXP NEUROL, V156, P138, DOI 10.1006/exnr.1998.7008

Mingazov ER, 2018, MOL NEUROBIOL, V55, P2991, DOI 10.1007/s12035-017-0559-6

MOGI M, 1988, J NEURAL TRANSM, V72, P77, DOI 10.1007/BF01244634

Morais LH, 2018, BEHAV PHARMACOL, V29, P199, DOI 10.1097/FBP.0000000000000389

Mulak A, 2019, FRONT NEUROSCI-SWITZ, V13, DOI 10.3389/fnins.2019.00992

Nakashima A, 2013, J NEURAL TRANSM, V120, P49, DOI 10.1007/s00702-012-0828-5

Nakashima A, 2011, BIOCHEM BIOPH RES CO, V407, P343, DOI 10.1016/j.bbrc.2011.03.020

Orbán C, 2017, IMMUNOL LETT, V185, P48, DOI 10.1016/j.imlet.2017.03.009

Paschalis EI, 2018, P NATL ACAD SCI USA, V115, pE11359, DOI 10.1073/pnas.1807123115

Perez-Pardo P, 2018, BENEF MICROBES, V9, P799, DOI [10.3920/bm2017.0202, 10.3920/BM2017.0202]

Perez-Pardo P, 2019, GUT, V68, P829, DOI 10.1136/gutjnl-2018-316844

Perez-Pardo P, 2018, FRONT AGING NEUROSCI, V10, DOI 10.3389/fnagi.2018.00237

Perez-Pardo P, 2018, NUTR NEUROSCI, V21, P391, DOI 10.1080/1028415X.2017.1294555

Perga S, 2017, NEUROTOX RES, V32, P1, DOI 10.1007/s12640-017-9724-y

Peter I, 2018, JAMA NEUROL, V75, P939, DOI 10.1001/jamaneurol.2018.0605

Pournelle G. H., 1953, Journal of Mammalogy, V34, P133, DOI 10.1890/0012-9658(2002)083[1421:SDEOLC]2.0.CO;2

Przedborski S, 2003, ANN NY ACAD SCI, V991, P189

Qin XY, 2016, JAMA NEUROL, V73, P1316, DOI 10.1001/jamaneurol.2016.2742

Rabe H, 2019, CLIN EXP IMMUNOL, V197, P111, DOI 10.1111/cei.13294

RStudio Team, 2019, RStudio, DOI DOI 10.1007/978-81-322-2340-5

Schmid MC, 2018, NAT COMMUN, V9, DOI 10.1038/s41467-018-07387-4

Schwiertz A, 2018, PARKINSONISM RELAT D, V50, P104, DOI 10.1016/j.parkreldis.2018.02.022

Shannon KM, 2012, MOVEMENT DISORD, V27, P709, DOI 10.1002/mds.23838

Song CH, 2012, NEUROBIOL DIS, V48, P66, DOI 10.1016/j.nbd.2012.05.009

Stolzenberg E, 2017, J INNATE IMMUN, V9, P456, DOI 10.1159/000477990

Sulzer D, 2017, NATURE, V546, P656, DOI 10.1038/nature22815

Thäle C, 2005, IMMUNOBIOLOGY, V210, P673, DOI 10.1016/j.imbio.2005.07.003

VanderBorght T, 1995, EUR J PHARMACOL, V294, P577

Villarán RF, 2010, J NEUROCHEM, V114, P1687, DOI 10.1111/j.1471-4159.2010.06879.x

Villumsen M, 2019, GUT, V68, P18, DOI 10.1136/gutjnl-2017-315666

Weimers P, 2019, INFLAMM BOWEL DIS, V25, P111, DOI 10.1093/ibd/izy190

Williams G, 2011, USE R, P3, DOI 10.1007/978-1-4419-9890-3

Williams-Gray CH, 2018, J NEUROINFLAMM, V15, DOI 10.1186/s12974-018-1206-5

Witoelar A, 2017, JAMA NEUROL, V74, P780, DOI 10.1001/jamaneurol.2017.0469

Yang XD, 2018, FRONT AGING NEUROSCI, V9, DOI 10.3389/fnagi.2017.00441

Zhu F, 2019, DIGEST LIVER DIS, V51, P38, DOI 10.1016/j.dld.2018.09.017

NR 91

TC 28

Z9 28

U1 0

U2 4

PU BMC

PI LONDON

PA CAMPUS, 4 CRINAN ST, LONDON N1 9XW, United Kingdom

SN 2051-5960

J9 ACTA NEUROPATHOL COM

JI Acta Neuropathol. Commun.

PD AUG 19

PY 2021

VL 9

IS 1

AR 139

DI 10.1186/s40478-021-01240-4

PG 23

WC Neurosciences

WE Science Citation Index Expanded (SCI-EXPANDED)

SC Neurosciences & Neurology

GA UC8LE

UT WOS:000686772700001

PM 34412704

OA gold, Green Published

DA 2024-08-12

ER

PT J

AU Li, DC

Ren, TZ

Li, H

Liao, G

Zhang, X

AF Li, Dongcheng

Ren, Tengzhu

Li, Hao

Liao, Geng

Zhang, Xiong

TI <i>Porphyromonas gingivalis</i>: A key role in Parkinson's disease with

cognitive impairment?

SO FRONTIERS IN NEUROLOGY

LA English

DT Review

DE Parkinson's disease; cognitive impairment; Porphyromonas gingivalis;

neuroinflammation; alpha-Synuclein; amyloid-beta; gut microbiota

ID LEWY BODY PATHOLOGY; ALPHA-SYNUCLEIN; PERIODONTAL PATHOGENS;

alzheimer's disease; VIRULENCE FACTORS; RISK-FACTOR; BIOMARKERS;

DEMENTIA; DEFICITS; MARKERS

AB Cognitive impairment (CI) is a common complication of Parkinson's disease (PD). The major features of Parkinson's disease with cognitive impairment (PD-CI) include convergence of alpha-Synuclein (alpha-Syn) and Alzheimer's disease (AD)-like pathologies, neuroinflammation, and dysbiosis of gut microbiota. Porphyromonas gingivalis (P. gingivalis) is an important pathogen in periodontitis. Recent research has suggested a rote of P. gingivalis and its virulence factor in the pathogenesis of PD and AD, in particular concerning neuroinflammation and deposition of alpha-Synuclein (alpha-Syn) and amyloid-beta (A beta). Furthermore, in animal models, oral P. gingivalis could cause neurodegeneration through regulating the gut-brain axis, suggesting an oral-gut-brain axis might exist. In this article, we discussed the pathological characteristics of PD-CI and the rote of P. gingivalis in them.

C1 [Li, Dongcheng; Li, Hao; Liao, Geng; Zhang, Xiong] Southern Med Univ, Affiliated Maoming Peoples Hosp, Dept Neurol, Maoming, China.

[Ren, Tengzhu] Guangdong Second Prov Gen Hosp, Dept Neurol, Guangzhou, China.

C3 Southern Medical University - China

RP Zhang, X (corresponding author), Southern Med Univ, Affiliated Maoming Peoples Hosp, Dept Neurol, Maoming, China.

EM xiong715@126.com

FU Special Fund Project for Science and Technology Innovation Strategy of

Guangdong Province; High-level Hospital Construction Research Project of

Maoming People's Hospital; Doctoral Research Foundation of Maoming

People's Hospital

FX This study was supported by the Special Fund Project for Science and

Technology Innovation Strategy of Guangdong Province and the High-level

Hospital Construction Research Project of Maoming People's Hospital. XZ

was supported by the Doctoral Research Foundation of Maoming People's

Hospital.

CR Adams B, 2019, FRONT AGING NEUROSCI, V11, DOI 10.3389/fnagi.2019.00210

Arimatsu K, 2014, SCI REP-UK, V4, DOI 10.1038/srep04828

Banks WA, 2015, J NEUROINFLAMM, V12, DOI 10.1186/s12974-015-0434-1

Beach TG, 2009, ACTA NEUROPATHOL, V117, P613, DOI [10.1007/s00401-010-0664-3, 10.1007/s00401-009-0538-8]

Berggreen E, 2013, J DENT RES, V92, P1074, DOI 10.1177/0022034513504589

Bhattacharyya D, 2019, ACS CHEM NEUROSCI, V10, P2229, DOI 10.1021/acschemneuro.8b00733

Bhattarai Y, 2018, NEUROGASTROENT MOTIL, V30, DOI 10.1111/nmo.13366

Boutaga K, 2007, J PERIODONTOL, V78, P79, DOI 10.1902/jop.2007.060078

Braak H, 2005, NEUROLOGY, V64, P1404, DOI 10.1212/01.WNL.0000158422.41380.82

Braak H, 2003, NEUROBIOL AGING, V24, P197, DOI 10.1016/S0197-4580(02)00065-9

Braak H, 2007, ACTA NEUROPATHOL, V113, P421, DOI 10.1007/s00401-007-0193-x

Chen CK, 2018, INT J ENV RES PUB HE, V15, DOI 10.3390/ijerph15081587

Chen CK, 2017, PEERJ, V5, DOI 10.7717/peerj.3647

Chen CK, 2017, ALZHEIMERS RES THER, V9, DOI 10.1186/s13195-017-0282-6

Chi L, 2021, FRONT CELL INFECT MI, V11, DOI 10.3389/fcimb.2021.755925

Chopra A, 2020, J ORAL MICROBIOL, V12, DOI 10.1080/20002297.2020.1801090

Compta Y, 2011, BRAIN, V134, P1493, DOI 10.1093/brain/awr031

Dioguardi M, 2020, J CLIN MED, V9, DOI 10.3390/jcm9020495

Dodiya HB, 2021, J EXP MED, V219, DOI 10.1084/jem.20200895

Dominy SS, 2019, SCI ADV, V5, DOI 10.1126/sciadv.aau3333

Du XY, 2020, INT J MOL SCI, V21, DOI 10.3390/ijms21228645

Fellner L, 2013, GLIA, V61, P349, DOI 10.1002/glia.22437

Fellner L, 2013, MOL NEUROBIOL, V47, P575, DOI 10.1007/s12035-012-8340-3

Feng YK, 2020, J NEUROINFLAMM, V17, DOI 10.1186/s12974-020-02027-5

Fereshtehnejad SM, 2017, BRAIN, V140, P1959, DOI 10.1093/brain/awx118

Forner L, 2006, J CLIN PERIODONTOL, V33, P401, DOI 10.1111/j.1600-051X.2006.00924.x

Forsyth CB, 2011, PLOS ONE, V6, DOI 10.1371/journal.pone.0028032

Gaig C, 2006, ARCH NEUROL-CHICAGO, V63, P377, DOI 10.1001/archneur.63.3.377

Guo YH, 2010, PERIODONTOL 2000, V54, P15, DOI 10.1111/j.1600-0757.2010.00377.x

Hanaoka A, 2009, J CLIN NEUROSCI, V16, P1279, DOI 10.1016/j.jocn.2008.12.027

Harding AJ, 2001, ACTA NEUROPATHOL, V102, P355

Hoogland J, 2017, MOVEMENT DISORD, V32, P1056, DOI 10.1002/mds.27002

Ide M, 2016, PLOS ONE, V11, DOI 10.1371/journal.pone.0151081

Irwin DJ, 2013, NAT REV NEUROSCI, V14, P626, DOI 10.1038/nrn3549

Irwin DJ, 2012, ANN NEUROL, V72, P587, DOI 10.1002/ana.23659

Jeong E, 2021, SCI REP-UK, V11, DOI 10.1038/s41598-021-96147-4

Jia L, 2019, FRONT CELL INFECT MI, V9, DOI 10.3389/fcimb.2019.00262

Jin YJ, 2022, ACTA NEUROPATHOL, V143, P641, DOI 10.1007/s00401-022-02421-8

Kamer AR, 2008, ALZHEIMERS DEMENT, V4, P242, DOI 10.1016/j.jalz.2007.08.004

Kashiwagi Y, 2021, SCI REP-UK, V11, DOI 10.1038/s41598-021-97868-2

Kaur T, 2016, GERODONTOLOGY, V33, P434, DOI 10.1111/ger.12188

Kim S, 2019, NEURON, V103, P627, DOI 10.1016/j.neuron.2019.05.035

Kimura A, 2018, J ALZHEIMERS DIS, V61, P581, DOI 10.3233/JAD-170519

Kordower JH, 2008, NAT MED, V14, P504, DOI 10.1038/nm1747

Kövari E, 2003, ACTA NEUROPATHOL, V106, P83, DOI 10.1007/s00401-003-0705-2

La Vitola P, 2021, NEUROPATH APPL NEURO, V47, P43, DOI 10.1111/nan.12644

Lee SJ, 2010, NAT REV NEUROL, V6, P702, DOI 10.1038/nrneurol.2010.145

Li JY, 2008, NAT MED, V14, P501, DOI 10.1038/nm1746

Lindqvist D, 2013, BRAIN BEHAV IMMUN, V33, P183, DOI 10.1016/j.bbi.2013.07.007

Liu CQ, 2015, PARKINSONISM RELAT D, V21, P271, DOI 10.1016/j.parkreldis.2014.12.027

Luk KC, 2012, SCIENCE, V338, P949, DOI 10.1126/science.1227157

Luk KC, 2012, J EXP MED, V209, P975, DOI 10.1084/jem.20112457

Mattila PM, 2000, ACTA NEUROPATHOL, V100, P285, DOI 10.1007/s004019900168

Mei F, 2020, PATHOGENS, V9, DOI 10.3390/pathogens9110944

Nakajima M, 2015, PLOS ONE, V10, DOI 10.1371/journal.pone.0134234

Navarro V, 2018, FRONT AGING NEUROSCI, V10, DOI 10.3389/fnagi.2018.00140

Nie R, 2019, J ALZHEIMERS DIS, V72, P479, DOI 10.3233/JAD-190298

Noble JM, 2009, J NEUROL NEUROSUR PS, V80, P1206, DOI 10.1136/jnnp.2009.174029

Olsen I, 2020, EUR J CLIN MICROBIOL, V39, P2013, DOI 10.1007/s10096-020-03944-2

Olsen I, 2017, J ORAL MICROBIOL, V9, DOI 10.1080/20002297.2017.1340085

Pan LN, 2022, BRAIN, V145, P3454, DOI 10.1093/brain/awac171

Pan-Montojo F, 2012, SCI REP-UK, V2, DOI 10.1038/srep00898

Parnetti L, 2014, FRONT AGING NEUROSCI, V6, DOI 10.3389/fnagi.2014.00053

Pedersen KF, 2017, NEUROLOGY, V88, P767, DOI 10.1212/WNL.0000000000003634

Peng XY, 2021, FRONT CELL INFECT MI, V11, DOI 10.3389/fcimb.2021.768108

Poole S, 2015, J ALZHEIMERS DIS, V43, P67, DOI 10.3233/JAD-140315

Poole S, 2013, J ALZHEIMERS DIS, V36, P665, DOI 10.3233/JAD-121918

Pretorius E, 2018, FRONT AGING NEUROSCI, V10, DOI 10.3389/fnagi.2018.00257

Ren TZ, 2020, FRONT NEUROL, V11, DOI 10.3389/fneur.2020.00137

Sampson TR, 2016, CELL, V167, P1469, DOI 10.1016/j.cell.2016.11.018

Sato K, 2017, SCI REP-UK, V7, DOI 10.1038/s41598-017-07196-7

Sato N, 2022, GERODONTOLOGY, V39, P139, DOI 10.1111/ger.12545

Saygun I, 2011, J PERIODONTAL RES, V46, P235, DOI 10.1111/j.1600-0765.2010.01335.x

Schrag A, 2017, LANCET NEUROL, V16, P66, DOI 10.1016/S1474-4422(16)30328-3

Sharon G, 2016, CELL, V167, P915, DOI 10.1016/j.cell.2016.10.027

Sorrentino ZA, 2020, FEBS LETT, V594, P1271, DOI 10.1002/1873-3468.13728

Stein PS, 2012, ALZHEIMERS DEMENT, V8, P196, DOI 10.1016/j.jalz.2011.04.006

Volpicelli-Daley LA, 2011, NEURON, V72, P57, DOI 10.1016/j.neuron.2011.08.033

vonTroilLinden B, 1995, J DENT RES, V74, P1789, DOI 10.1177/00220345950740111201

Wang T, 2022, FRONT MED-LAUSANNE, V9, DOI 10.3389/fmed.2022.822190

Wijeyekoon RS, 2020, MOVEMENT DISORD, V35, P1062, DOI 10.1002/mds.28015

Williams-Gray CH, 2016, MOVEMENT DISORD, V31, P995, DOI 10.1002/mds.26563

Wu Z, 2017, BRAIN BEHAV IMMUN, V65, P350, DOI 10.1016/j.bbi.2017.06.002

Zeng F, 2021, J NEUROCHEM, V158, P724, DOI 10.1111/jnc.15096

Zhang J, 2018, J NEUROINFLAMM, V15, DOI 10.1186/s12974-017-1052-x

Zhang X, 2021, J LEUKOCYTE BIOL, V110, P511, DOI 10.1002/JLB.3MA0521-742RRR

NR 86

TC 9

Z9 10

U1 9

U2 23

PU FRONTIERS MEDIA SA

PI LAUSANNE

PA AVENUE DU TRIBUNAL FEDERAL 34, LAUSANNE, CH-1015, SWITZERLAND

SN 1664-2295

J9 FRONT NEUROL

JI Front. Neurol.

PD JUL 26

PY 2022

VL 13

AR 945523

DI 10.3389/fneur.2022.945523

PG 8

WC Clinical Neurology; Neurosciences

WE Science Citation Index Expanded (SCI-EXPANDED)

SC Neurosciences & Neurology

GA 3Q7HA

UT WOS:000838396300001

PM 35959396

OA Green Published, gold

DA 2024-08-12

ER

PT J

AU Siddiqui, T

Bhatt, LK

AF Siddiqui, Talha

Bhatt, Lokesh Kumar

TI Targeting Sigma-1 Receptor: A Promising Strategy in the Treatment of

Parkinson's Disease

SO NEUROCHEMICAL RESEARCH

LA English

DT Review

DE Parkinson's disease; Sigma-1 receptor; S1R agonists; Neuroprotection

ID ANTIINFLAMMATORY CYTOKINES; MOUSE MODEL; LIGAND; MICROGLIA;

NEURODEGENERATION; ACTIVATION; PATHWAY; STRESS; REGION; DAMAGE

AB Parkinson's disease is a neurodegenerative disease affecting mainly the elderly population. It is characterized by the loss of dopaminergic neurons of the substantia nigra pars compacta region. Parkinson's disease patients exhibit motor symptoms like tremors, rigidity, bradykinesia/hypokinesia, and non-motor symptoms like depression, cognitive decline, delusion, and pain. Major pathophysiological factors which contribute to neuron loss include excess/misfolded alpha-synuclein aggregates, microglial cell-mediated neuroinflammation, excitotoxicity, oxidative stress, and defective mitochondrial function. Sigma-1 receptors are molecular chaperones located at mitochondria-associated ER membrane. Their activation (by endogenous ligands or agonists) has shown neuroprotective and neurorestorative effects in various diseases. This review discusses the roles of activated Sig-1 receptors in modulating various pathophysiological features of Parkinson's disease like alpha-synuclein aggregates, neuroinflammation, excitotoxicity, and oxidative stress.

C1 [Siddiqui, Talha; Bhatt, Lokesh Kumar] SVKMs Dr Bhanuben Nanavati Coll Pharm, Dept Pharmacol, Mumbai 400056, India.

RP Bhatt, LK (corresponding author), SVKMs Dr Bhanuben Nanavati Coll Pharm, Dept Pharmacol, Mumbai 400056, India.

EM bhatt.lokesh@gmail.com

RI Bhatt, Lokesh Kumar/O-3277-2015

OI Bhatt, Lokesh Kumar/0000-0002-4302-9300

CR Aborode AT, 2022, OXID MED CELL LONGEV, V2022, DOI 10.1155/2022/7934442

Adams CJ, 2019, FRONT MOL BIOSCI, V6, DOI 10.3389/fmolb.2019.00011

Ajmo CT, 2006, CURR NEUROVASC RES, V3, P89, DOI 10.2174/156720206776875849

Allahtavakoli M, 2011, BRAIN RES BULL, V85, P219, DOI 10.1016/j.brainresbull.2011.03.019

Araújo B, 2022, CELLS-BASEL, V11, DOI 10.3390/cells11182908

Bourrié B, 2002, EUR J PHARMACOL, V456, P123, DOI 10.1016/S0014-2999(02)02646-8

Burré J, 2018, CSH PERSPECT MED, V8, DOI 10.1101/cshperspect.a024091

Cuevas J, 2011, J PHARMACOL EXP THER, V339, P161, DOI 10.1124/jpet.111.182816

de Lau LML, 2006, LANCET NEUROL, V5, P525, DOI 10.1016/S1474-4422(06)70471-9

DECOSTER MA, 1995, BRAIN RES, V671, P45, DOI 10.1016/0006-8993(94)01294-R

Dias V, 2013, J PARKINSON DIS, V3, P461, DOI 10.3233/JPD-130230

Francardo V, 2019, NEUROTHERAPEUTICS, V16, P465, DOI 10.1007/s13311-018-00699-9

Francardo V, 2014, BRAIN, V137, P1998, DOI 10.1093/brain/awu107

Gaja-Capdevila N, 2021, FRONT PHARMACOL, V12, DOI 10.3389/fphar.2021.780588

Gardner BM, 2013, CSH PERSPECT BIOL, V5, DOI 10.1101/cshperspect.a013169

Gelders G, 2018, J IMMUNOL RES, V2018, DOI 10.1155/2018/4784268

Geva M, 2021, SCI REP-UK, V11, DOI 10.1038/s41598-021-01077-w

Geva M, 2016, HUM MOL GENET, V25, P3975, DOI 10.1093/hmg/ddw238

Goguadze N, 2019, NEUROTOX RES, V35, P1, DOI 10.1007/s12640-017-9838-2

Guo YJ, 2020, EXP THER MED, V19, P1997, DOI 10.3892/etm.2020.8454

Hall AA, 2009, GLIA, V57, P744, DOI 10.1002/glia.20802

Hayashi T, 2019, FRONT NEUROSCI-SWITZ, V13, DOI 10.3389/fnins.2019.00733

Hayashi T, 2011, EXPERT OPIN THER TAR, V15, P557, DOI 10.1517/14728222.2011.560837

Hickman S, 2018, NAT NEUROSCI, V21, P1359, DOI 10.1038/s41593-018-0242-x

Iovino L, 2020, J PHARMACOL SCI, V144, P151, DOI 10.1016/j.jphs.2020.07.011

Jia J, 2018, FRONT CELL NEUROSCI, V12, DOI 10.3389/fncel.2018.00314

Jiang TF, 2016, PROG NEUROBIOL, V147, P1, DOI 10.1016/j.pneurobio.2016.07.005

Kadnikov IA, 2020, INT J MOL SCI, V21, DOI 10.3390/ijms21207620

Keller M, 2008, EUR J NEUROSCI, V27, P874, DOI 10.1111/j.1460-9568.2008.06062.x

Nguyen L, 2015, J PHARMACOL SCI, V127, P17, DOI 10.1016/j.jphs.2014.12.005

Lu CW, 2012, J PHARMACOL EXP THER, V341, P532, DOI 10.1124/jpet.111.191189

MARTIN WR, 1976, J PHARMACOL EXP THER, V197, P517

Mori T, 2013, PLOS ONE, V8, DOI 10.1371/journal.pone.0076941

Nguyen L, 2017, ADV EXP MED BIOL, V964, P133, DOI 10.1007/978-3-319-50174-1_10

Nguyen L, 2014, EUR J PHARMACOL, V743, P42, DOI 10.1016/j.ejphar.2014.09.022

Nwabufo CK, 2022, J NEUROL, V269, P5762, DOI 10.1007/s00415-022-11267-9

Olanow CW, 1999, ANNU REV NEUROSCI, V22, P123, DOI 10.1002/mds.23732

Ossa F, 2017, ADV EXP MED BIOL, V964, P15, DOI 10.1007/978-3-319-50174-1_3

Pajares M, 2020, CELLS-BASEL, V9, DOI 10.3390/cells9071687

Penke B, 2018, CURR NEUROPHARMACOL, V16, P97, DOI 10.2174/1570159X15666170529104323

Pisanu A, 2014, NEUROBIOL DIS, V71, P280, DOI 10.1016/j.nbd.2014.08.011

Plastira I, 2016, J NEUROINFLAMM, V13, DOI 10.1186/s12974-016-0701-9

Prasad PD, 1998, J NEUROCHEM, V70, P443

Puchalowicz K, 2014, INT J MOL SCI, V15, P23672, DOI 10.3390/ijms151223672

Rousseaux CG, 2016, J RECEPT SIG TRANSD, V36, P327, DOI 10.3109/10799893.2015.1015737

Ruoho AE, 2012, CURR PHARM DESIGN, V18, P920

Ruscher K, 2015, J PHARMACOL SCI, V127, P30, DOI 10.1016/j.jphs.2014.11.011

Ryskamp DA, 2019, FRONT NEUROSCI-SWITZ, V13, DOI 10.3389/fnins.2019.00862

Sánchez-Blazquez P, 2020, MOL BRAIN, V13, DOI 10.1186/s13041-020-00676-4

Sanders LH, 2013, FREE RADICAL BIO MED, V62, P111, DOI 10.1016/j.freeradbiomed.2013.01.003

Sano R, 2013, BBA-MOL CELL RES, V1833, P3460, DOI 10.1016/j.bbamcr.2013.06.028

Schröder M, 2005, ANNU REV BIOCHEM, V74, P739, DOI 10.1146/annurev.biochem.73.011303.074134

Sharma N, 2021, J BIOL CHEM, V297, DOI 10.1016/j.jbc.2021.101299

Shenkman M, 2021, J NEUROCHEM, V158, P467, DOI 10.1111/jnc.15366

Shimazu S, 2000, EUR J PHARMACOL, V388, P139, DOI 10.1016/S0014-2999(99)00852-3

Song QX, 2021, NEUROTOXICOLOGY, V87, P188, DOI 10.1016/j.neuro.2021.10.003

Tang Y, 2016, MOL NEUROBIOL, V53, P1181, DOI 10.1007/s12035-014-9070-5

Tansey MG, 2022, NAT REV IMMUNOL, V22, P657, DOI 10.1038/s41577-022-00684-6

Tomkins JE, 2021, NEUROBIOL DIS, V155, DOI 10.1016/j.nbd.2021.105395

Tsai SY, 2012, SYNAPSE, V66, P42, DOI 10.1002/syn.20984

Tu HY, 2021, AGING CELL, V20, DOI 10.1111/acel.13522

Tuerxun T, 2010, NEUROSCI LETT, V469, P303, DOI 10.1016/j.neulet.2009.12.013

Urfer R, 2014, STROKE, V45, P3304, DOI 10.1161/STROKEAHA.114.005835

Vomhof-DeKrey EE, 2012, J NUTR BIOCHEM, V23, P1201, DOI 10.1016/j.jnutbio.2012.03.005

Voronin MV, 2019, SCI REP-UK, V9, DOI 10.1038/s41598-019-53413-w

WALKER JM, 1990, PHARMACOL REV, V42, P355

Wang J, 2019, FREE RADICAL BIO MED, V134, P604, DOI 10.1016/j.freeradbiomed.2019.02.001

Wang J, 2020, FRONT NEUROSCI-SWITZ, V14, DOI 10.3389/fnins.2020.585584

Wang MM, 2021, NEUROPHARMACOLOGY, V196, DOI 10.1016/j.neuropharm.2020.108360

Weng TY, 2017, J BIOMED SCI, V24, DOI 10.1186/s12929-017-0380-6

Whittemore ER, 1997, J PHARMACOL EXP THER, V282, P326

Wu Z, 2015, J NEUROCHEM, V134, P904, DOI 10.1111/jnc.13182

Zhang YL, 2020, ACS CHEM NEUROSCI, V11, P406, DOI 10.1021/acschemneuro.9b00609

Zhou ZX, 2021, ANIM CELLS SYST, V25, P347, DOI 10.1080/19768354.2021.2020901

NR 74

TC 7

Z9 7

U1 1

U2 5

PU SPRINGER/PLENUM PUBLISHERS

PI NEW YORK

PA 233 SPRING ST, NEW YORK, NY 10013 USA

SN 0364-3190

EI 1573-6903

J9 NEUROCHEM RES

JI Neurochem. Res.

PD OCT

PY 2023

VL 48

IS 10

BP 2925

EP 2935

DI 10.1007/s11064-023-03960-6

EA MAY 2023

PG 11

WC Biochemistry & Molecular Biology; Neurosciences

WE Science Citation Index Expanded (SCI-EXPANDED)

SC Biochemistry & Molecular Biology; Neurosciences & Neurology

GA Q8BD4

UT WOS:000999258800001

PM 37259012

OA Bronze, Green Published

DA 2024-08-12

ER

PT J

AU Zorina, SA

Jurja, S

Mehedinti, M

Stoica, AM

Chita, DS

Floris, SA

Axelerad, A

AF Zorina, Stuparu Alina

Jurja, Sanda

Mehedinti, Mihaela

Stoica, Ana-Maria

Chita, Dana Simona

Floris, Stuparu Alexandru

Axelerad, Any

TI Infectious Microorganisms Seen as Etiologic Agents in Parkinson's

Disease

SO LIFE-BASEL

LA English

DT Review

DE Parkinson's disease; virus; bacteria; pathogens; neuroinflammation

ID CENTRAL-NERVOUS-SYSTEM; C VIRUS-INFECTION; HELICOBACTER-PYLORI;

POSTENCEPHALITIC PARKINSONISM; NOCARDIA-ASTEROIDES; RISK-FACTOR;

ASSOCIATION; NEUROINFLAMMATION; ALZHEIMERS; ANTIBODIES

AB Infections represent a possible risk factor for parkinsonism and Parkinson's disease (PD) based on information from epidemiology and fundamental science. The risk is unclear for the majority of agents. Moreover, the latency between infection and PD seems to be very varied and often lengthy. In this review, the evidence supporting the potential involvement of infectious microorganisms in the development of Parkinson's disease is examined. Consequently, it is crucial to determine the cause and give additional treatment accordingly. Infection is an intriguing suggestion regarding the cause of Parkinson's disease. These findings demonstrate that persistent infection with viral and bacterial microorganisms might be a cause of Parkinson's disease. As an initiating factor, infection may generate a spectrum of gut microbiota dysbiosis, engagement of glial tissues, neuroinflammation, and alpha-synuclein accumulation, all of which may trigger and worsen the onset in Parkinson's disease also contribute to its progression. Still uncertain is the primary etiology of PD with infection. The possible pathophysiology of PD infection remains a matter of debate. Furthermore, additional study is required to determine if PD patients develop the disease due to infectious microorganisms or solely since they are more sensitive to infectious causes.

C1 [Zorina, Stuparu Alina; Axelerad, Any] St Andrew County Clin Emergency Hosp Constanta, Dept Neurol, Constanta 900591, Romania.

[Zorina, Stuparu Alina; Axelerad, Any] Ovidius Univ, Gen Med Fac, Dept Neurol, Constanta 900470, Romania.

[Jurja, Sanda; Stoica, Ana-Maria] St Andrew County Clin Emergency Hosp Constanta, Dept Ophthalmol, Constanta 900591, Romania.

[Jurja, Sanda; Stoica, Ana-Maria] Ovidius Univ, Gen Med Fac, Dept Ophthalmol, Constanta 900470, Romania.

[Mehedinti, Mihaela] Univ Med & Pharm Dunarea De Jos, Dept Morphol & Funct Sci, Galati 800017, Romania.

[Chita, Dana Simona] Vasile Goldis Western Univ Arad, Fac Gen Med & Pharm, Dept Neurol, Arad 310045, Romania.

[Floris, Stuparu Alexandru] St Andrew Cty Clin Emergency Hosp Constanta, Dept Orthopedy & Traumatol, Constanta 900591, Romania.

C3 Ovidius University; Ovidius University; Dunarea De Jos University

Galati; Vasile Goldis Western University of Arad

RP Jurja, S (corresponding author), St Andrew County Clin Emergency Hosp Constanta, Dept Ophthalmol, Constanta 900591, Romania.; Jurja, S (corresponding author), Ovidius Univ, Gen Med Fac, Dept Ophthalmol, Constanta 900470, Romania.

EM jurjasanda@yahoo.com

RI Stroe Stuparu, Zorina Alina/AAC-9177-2021; AXELERAD, ANY

DOCU/F-5825-2012

OI Stroe Stuparu, Zorina Alina/0000-0001-7584-5188;

CR Acioglu C, 2022, BRAIN BEHAV IMMUN, V102, P163, DOI 10.1016/j.bbi.2022.02.016

Al-Nusaif M, 2022, INT J MOL SCI, V23, DOI 10.3390/ijms232416184

Alam Q, 2016, CURR PHARM DESIGN, V22, P541, DOI 10.2174/1381612822666151125000300

Albornoz EA, 2023, MOL PSYCHIATR, V28, P2878, DOI 10.1038/s41380-022-01831-0

Arsenijevic VSA, 2014, BMC DERMATOL, V14, DOI 10.1186/1471-5945-14-5

Bopeththa B V K M, 2017, BMC Res Notes, V10, P655, DOI 10.1186/s13104-017-2954-5

Braak H, 2006, NEUROSCI LETT, V396, P67, DOI 10.1016/j.neulet.2005.11.012

Braak H, 2003, J NEURAL TRANSM, V110, P517, DOI 10.1007/s00702-002-0808-2

Breckenridge CB, 2016, PLOS ONE, V11, DOI 10.1371/journal.pone.0151841

Bu XL, 2015, PARKINSONISM RELAT D, V21, P877, DOI 10.1016/j.parkreldis.2015.05.015

Caggiu E, 2019, FRONT NEUROL, V10, DOI 10.3389/fneur.2019.00122

Chen SL, 2022, NEUROCHEM RES, V47, P3213, DOI 10.1007/s11064-022-03645-6

Choi JG, 2018, SCI REP-UK, V8, DOI 10.1038/s41598-018-19646-x

Crespo J, 2020, J VIRAL HEPATITIS, V27, P360, DOI 10.1111/jvh.13238

De Chirico F, 2022, FRONT CELL NEUROSCI, V16, DOI 10.3389/fncel.2022.1038721

De Miranda BR, 2022, J PARKINSON DIS, V12, P45, DOI 10.3233/JPD-212922

DiSabato DJ, 2016, J NEUROCHEM, V139, P136, DOI 10.1111/jnc.13607

Dobbs RJ, 2000, ALIMENT PHARM THER, V14, P1199

Dourmashkin RR, 2012, BMC INFECT DIS, V12, DOI 10.1186/1471-2334-12-136

EBMEIER KP, 1989, J NEUROL NEUROSUR PS, V52, P911, DOI 10.1136/jnnp.52.7.911

Espay AJ, 2011, NEUROLOGY, V76, P1529, DOI 10.1212/WNL.0b013e318217e7dd

FAZZINI E, 1992, MOVEMENT DISORD, V7, P153, DOI 10.1002/mds.870070210

Fiszer U, 2004, ACTA NEUROL SCAND, V110, P113, DOI 10.1111/j.1600-0404.2004.00285.x

Fletcher NF, 2012, GASTROENTEROLOGY, V142, P634, DOI 10.1053/j.gastro.2011.11.028

Forsyth CB, 2011, PLOS ONE, V6, DOI 10.1371/journal.pone.0028032

Goiran T, 2022, CELLS-BASEL, V11, DOI 10.3390/cells11132097

Goshi N, 2020, J NEUROINFLAMM, V17, DOI 10.1186/s12974-020-01819-z

Harris MA, 2012, MOVEMENT DISORD, V27, P1111, DOI 10.1002/mds.25077

Hawkes CH, 2007, NEUROPATH APPL NEURO, V33, P599, DOI 10.1111/j.1365-2990.2007.00874.x

He S, 2015, NEUROL SCI, V36, P653, DOI 10.1007/s10072-015-2064-x

Hemling N, 2003, ANN NEUROL, V54, P267, DOI 10.1002/ana.10662

HIRAI Y, 1995, J BACTERIOL, V177, P5327, DOI 10.1128/jb.177.18.5327-5333.1995

Hristina VD, 2010, INT J NEUROSCI, V120, P361, DOI 10.3109/00207451003668374

HUBBLE JP, 1995, J CLIN MICROBIOL, V33, P2768, DOI 10.1128/JCM.33.10.2768-2769.1995

Iseki T, 2023, BIOMOLECULES, V13, DOI 10.3390/biom13010178

Jang Y, 2023, MOL CELL PROTEOMICS, V22, DOI 10.1016/j.mcpro.2022.100452

Jeon YM, 2020, FRONT CELL DEV BIOL, V8, DOI 10.3389/fcell.2020.548283

Jin XM, 2016, J BIOCHEM, V159, P491, DOI 10.1093/jb/mvw009

Johnson BH, 2015, BMC INFECT DIS, V15, DOI 10.1186/s12879-015-1262-8

Johnson ME, 2019, TRENDS NEUROSCI, V42, P4, DOI 10.1016/j.tins.2018.09.007

Kannarkat GT, 2013, J PARKINSON DIS, V3, P493, DOI 10.3233/JPD-130250

Kim JM, 2016, MOVEMENT DISORD, V31, P1584, DOI 10.1002/mds.26755

KOHBATA S, 1991, INFECT IMMUN, V59, P181, DOI 10.1128/IAI.59.1.181-191.1991

KOHBATA S, 1993, ADV NEUROL, V60, P355

Kohbata S, 2009, MICROBES INFECT, V11, P744, DOI 10.1016/j.micinf.2009.04.013

Kwon HS, 2020, TRANSL NEURODEGENER, V9, DOI 10.1186/s40035-020-00221-2

Lai SW, 2017, MEDICINE, V96, DOI [10.1097/MD.0000000000006075, 10.1097/md.0000000000006075]

Laurence M, 2019, FRONT NEUROL, V10, DOI 10.3389/fneur.2019.00758

Leta V, 2022, J NEURAL TRANSM, V129, P1119, DOI 10.1007/s00702-022-02536-y

Li KL, 2022, NEURAL REGEN RES, V17, P1898, DOI 10.4103/1673-5374.335143

Li L, 2021, BRAIN BEHAV IMMUN, V91, P740, DOI 10.1016/j.bbi.2020.10.007

Li YY, 2022, J NEUROINFLAMM, V19, DOI 10.1186/s12974-022-02481-3

Lin WY, 2019, JAMA NEUROL, V76, P1019, DOI 10.1001/jamaneurol.2019.1368

Linnerbauer M, 2020, NEURON, V108, P608, DOI 10.1016/j.neuron.2020.08.012

Liu TW, 2022, INT J MOL SCI, V23, DOI 10.3390/ijms23084148

Liu ZR, 2022, J NEUROINFLAMM, V19, DOI 10.1186/s12974-022-02413-1

Lu L, 2005, MICROB PATHOGENESIS, V39, P205, DOI 10.1016/j.micpath.2005.08.001

McKee DH, 2005, MOVEMENT DISORD, V20, P1661, DOI 10.1002/mds.20641

Meng L, 2019, J NEUROVIROL, V25, P221, DOI 10.1007/s13365-018-0707-4

Miller SJ, 2022, FRONT NEUROSCI-SWITZ, V16, DOI 10.3389/fnins.2022.824191

Mitkovski M, 2012, FRONT PHYSIOL, V3, DOI 10.3389/fphys.2012.00372

More SV, 2013, MEDIAT INFLAMM, V2013, DOI 10.1155/2013/952375

Morini R, 2021, FRONT IMMUNOL, V12, DOI 10.3389/fimmu.2021.640937

Mridula KR, 2017, J CLIN NEUROL, V13, P181, DOI 10.3988/jcn.2017.13.2.181

Mullin S, 2021, MOVEMENT DISORD, V36, P774, DOI 10.1002/mds.28375

Muzio L, 2021, FRONT NEUROSCI-SWITZ, V15, DOI 10.3389/fnins.2021.742065

Navarro-López EM, 2021, COGN NEURODYNAMICS, V15, P693, DOI 10.1007/s11571-020-09653-y

Olude MA, 2022, FRONT IMMUNOL, V13, DOI 10.3389/fimmu.2022.795089

Prunell G, 2022, BIOMOLECULES, V12, DOI 10.3390/biom12121745

Rabaneda-Lombarte N, 2019, FRONT MOL NEUROSCI, V11, DOI 10.3389/fnmol.2018.00479

Radkowski M, 2002, J VIROL, V76, P600, DOI 10.1128/JVI.76.2.600-608.2002

Rees K, 2011, COCHRANE DB SYST REV, DOI 10.1002/14651858.CD008453.pub2

SASCO AJ, 1985, AM J EPIDEMIOL, V122, P1017, DOI 10.1093/oxfordjournals.aje.a114183

Shamsdin Seyedeh Azra, 2022, Middle East J Dig Dis, V14, P96, DOI 10.34172/mejdd.2022.261

Shen XL, 2017, HELICOBACTER, V22, DOI 10.1111/hel.12398

Silva DF, 2022, ANTIOXIDANTS-BASEL, V11, DOI 10.3390/antiox11112141

SINGER C, 1989, J NEUROL NEUROSUR PS, V52, P931, DOI 10.1136/jnnp.52.7.931

Spiteri AG, 2022, ACTA NEUROPATHOL, V143, P179, DOI 10.1007/s00401-021-02384-2

Su Q, 2022, FRONT AGING NEUROSCI, V14, DOI 10.3389/fnagi.2022.957705

Suwarnalata G, 2016, PLOS ONE, V11, DOI 10.1371/journal.pone.0153725

Tanner CM, 2009, ARCH NEUROL-CHICAGO, V66, P1106, DOI 10.1001/archneurol.2009.195

Tansey MG, 2022, NAT REV IMMUNOL, V22, P657, DOI 10.1038/s41577-022-00684-6

Turkel Y, 2015, NIGER J CLIN PRACT, V18, P612, DOI 10.4103/1119-3077.154215

Villarán RF, 2010, J NEUROCHEM, V114, P1687, DOI 10.1111/j.1471-4159.2010.06879.x

Vlajinac H, 2013, INT J NEUROSCI, V123, P329, DOI 10.3109/00207454.2012.760560

Wang H, 2020, BRAIN BEHAV, V10, DOI 10.1002/brb3.1549

Wang Q, 2022, NEUROL-NEUROIMMUNOL, V9, DOI 10.1212/NXI.0000000000001152

Wang X, 2022, FRONT PSYCHIATRY, V13, DOI 10.3389/fpsyt.2022.991085

Wijarnpreecha K, 2018, EUR J GASTROEN HEPAT, V30, P9, DOI 10.1097/MEG.0000000000000991

Woodburn SC, 2021, J NEUROINFLAMM, V18, DOI 10.1186/s12974-021-02309-6

Xu JH, 2022, FRONT IMMUNOL, V13, DOI 10.3389/fimmu.2022.1063928

Yankam BM, 2019, PAN AFR MED J, V33, DOI 10.11604/pamj.2019.33.310.16894

Yemula N, 2021, J PARKINSON DIS, V11, P1491, DOI 10.3233/JPD-212707

Yi SL, 2022, INT J MOL SCI, V23, DOI 10.3390/ijms232314753

Zhang HT, 2022, FRONT AGING NEUROSCI, V14, DOI 10.3389/fnagi.2022.975248

Zheng ZJ, 2022, OXID MED CELL LONGEV, V2022, DOI 10.1155/2022/7965433

NR 96

TC 2

Z9 2

U1 3

U2 7

PU MDPI

PI BASEL

PA ST ALBAN-ANLAGE 66, CH-4052 BASEL, SWITZERLAND

EI 2075-1729

J9 LIFE-BASEL

JI Life-Basel

PD MAR

PY 2023

VL 13

IS 3

AR 805

DI 10.3390/life13030805

PG 13

WC Biology; Microbiology

WE Science Citation Index Expanded (SCI-EXPANDED)

SC Life Sciences & Biomedicine - Other Topics; Microbiology

GA A8TD3

UT WOS:000957778700001

PM 36983960

OA Green Published, gold

DA 2024-08-12

ER

PT J

AU Graeber, MB

AF Graeber, Manuel B.

TI Neuroinflammation: No Rose by Any Other Name

SO BRAIN PATHOLOGY

LA English

DT Article

DE Alzheimer's disease; inflammation; microglia; multiple sclerosis;

Parkinson's disease; schizophrenia; transcriptome

ID MICROGLIA; DISEASE; CNS

C1 [Graeber, Manuel B.] Univ Sydney, Brain & Mind Res Inst, Fac Med, Sydney, NSW 2050, Australia.

[Graeber, Manuel B.] Univ Sydney, Fac Hlth Sci, Sydney, NSW 2050, Australia.

C3 University of Sydney; University of Sydney

RP Graeber, MB (corresponding author), Univ Sydney, Brain & Mind Res Inst, 94 Mallett St, Sydney, NSW 2050, Australia.

EM manuel@graeber.net

RI Graeber, Manuel B/H-3224-2011

FU Cure Brain Cancer Foundation, Australia

FX Funding from the Cure Brain Cancer Foundation, Australia, to MBG is

gratefully acknowledged.

CR Alzheimer A., 1907, Allgemeine Zeitschrift fur Psychiatrie und Psychisch-Gerichtliche Medizin, P146

Block ML, 2009, TRENDS NEUROSCI, V32, P506, DOI 10.1016/j.tins.2009.05.009

desKloet AD, 2014, PHYSIOL BEHAV, DOI [10.1016/jphysbeh201401016, DOI 10.1016/JPHYSBEH201401016]

Estes M, 2014, BRAIN PATHO IN PRESS, V24

Filiou MD, 2014, NEUROGENETICS, V15, P201, DOI 10.1007/s10048-014-0409-x

Graeber MB, 1990, BRAIN PATHOL, V1, P2, DOI 10.1111/j.1750-3639.1990.tb00630.x

Graeber MB, 2010, SCIENCE, V330, P783, DOI 10.1126/science.1190929

Graeber MB, 1998, NEUROGENETICS, V1, P223, DOI 10.1007/s100480050033

Kleinewietfeld M, 2014, IMMUNOL REV, V259, P231, DOI 10.1111/imr.12169

Krstic D, 2012, J NEUROINFLAMM, V9, DOI 10.1186/1742-2094-9-151

Liu G-J, 2014, BRAIN PATHO IN PRESS, V24

Orwell G, 2013, Politics and the english language

Salter MW, 2014, CELL, V158, P15, DOI 10.1016/j.cell.2014.06.008

Searle JR, 1992, REDISCOVERY MIND, P126

Svahn AJ, 2014, BRAIN PATHO IN PRESS, V24

Wisor JP, 2011, SLEEP, V34, P261, DOI 10.1093/sleep/34.3.261

Zahr NM, 2014, BRAIN PATHO IN PRESS, V24

NR 17

TC 25

Z9 25

U1 0

U2 4

PU WILEY-BLACKWELL

PI HOBOKEN

PA 111 RIVER ST, HOBOKEN 07030-5774, NJ USA

SN 1015-6305

EI 1750-3639

J9 BRAIN PATHOL

JI Brain Pathol.

PD NOV

PY 2014

VL 24

IS 6

BP 620

EP 622

DI 10.1111/bpa.12192

PG 3

WC Clinical Neurology; Neurosciences; Pathology

WE Science Citation Index Expanded (SCI-EXPANDED)

SC Neurosciences & Neurology; Pathology

GA AS6AI

UT WOS:000344347000008

PM 25345892

OA Green Published

DA 2024-08-12

ER

PT J

AU Gill, EL

Raman, S

Yost, RA

Garrett, TJ

Vedam-Mai, V

AF Gill, Emily L.

Raman, Shreya

Yost, Richard A.

Garrett, Timothy J.

Vedam-Mai, Vinata

TI L-Carnitine Inhibits Lipopolysaccharide-Induced Nitric Oxide Production

of SIM-A9 Microglia Cells

SO ACS CHEMICAL NEUROSCIENCE

LA English

DT Article

DE Parkinson's disease; microglia; lipopolysaccharide; L-carnitine;

gliosis; neuroinflammation

ID DEEP-BRAIN-STIMULATION; OXIDATIVE STRESS; PARKINSONS; ACTIVATION;

ANTIOXIDANT; DISEASE; NRF2

AB Microglia are the resident immune effector cells of the central nervous system. They account for approximately 10-15% of all cells found in the brain and spinal cord, acting as macrophages, sensing and engaging in phagocytosis to eliminate toxic proteins. Microglia are dynamic and can change their morphology in response to cues from their milieu. Parkinson's disease is a neurodegenerative disease, associated with reactive gliosis, neuroinflammation, and oxidative stress. It is thought that Parkinson's disease is caused by the accumulation of abnormally folded alpha-synuclein protein, accompanied by persistent neuroinflammation, oxidative stress, and subsequent neuronal injury/death. There is evidence in the literature for mitochondrial dysfunction in Parkinson's disease as well as fatty acid beta-oxidation, involving Lecarnitine. Here we investigate L-carnitine in the context of microglial activation, suggesting a potential new strategy of supplementation for PD patients. Preliminary results from our studies suggest that the treatment of activated microglia with the endogenous antioxidant L-carnitine can reverse the effects of detrimental neuroinflammation in vitro.

C1 [Gill, Emily L.; Yost, Richard A.] Univ Florida, Dept Chem, Gainesville, FL 32611 USA.

[Raman, Shreya; Vedam-Mai, Vinata] Univ Florida, Dept Neurosurg, Gainesville, FL 32610 USA.

[Yost, Richard A.; Garrett, Timothy J.] Univ Florida, Dept Pathol Immunol & Lab Med, Gainesville, FL 32610 USA.

C3 State University System of Florida; University of Florida; State

University System of Florida; University of Florida; State University

System of Florida; University of Florida

RP Vedam-Mai, V (corresponding author), Univ Florida, Dept Neurosurg, Gainesville, FL 32610 USA.

EM vinved@neurosurgery.ufl.edu

RI Garrett, Timothy/T-9833-2019

OI Garrett, Timothy/0000-0003-1623-009X; Yost, Richard/0000-0002-1293-5669;

Gill, Emily/0000-0002-6562-3001; Raman, Shreya/0000-0002-4521-7276;

Vedam-Mai, Vinata/0000-0003-0148-3297

FU Department of Neurosurgery; Southeast Center for Integrated Metabolomics

(SECIM) at the University of Florida (NIH) [U24 DK097209]

FX The authors would like to thank the Department of Neurosurgery and the

Southeast Center for Integrated Metabolomics (SECIM) at the University

of Florida (NIH Grant #U24 DK097209) for funding this study.

CR Bendor JT, 2013, NEURON, V79, P1044, DOI 10.1016/j.neuron.2013.09.004

Burté F, 2017, MOVEMENT DISORD, V32, P927, DOI 10.1002/mds.26992

Famularo G, 2004, ANN NY ACAD SCI, V1033, P132, DOI 10.1196/annals.1320.012

Giustarini D, 2008, METHOD ENZYMOL, V440, P361, DOI 10.1016/S0076-6879(07)00823-3

Gründemann D, 2005, P NATL ACAD SCI USA, V102, P5256, DOI 10.1073/pnas.0408624102

Gülçin I, 2006, LIFE SCI, V78, P803, DOI 10.1016/j.lfs.2005.05.103

Jang JH, 2004, FREE RADICAL BIO MED, V36, P288, DOI 10.1016/j.freeradbiomed.2003.11.005

Jenner P, 2003, ANN NEUROL, V53, pS26, DOI 10.1002/ana.10483

Koc A, 2011, CELL BIOCHEM FUNCT, V29, P679, DOI 10.1002/cbf.1807

Le WD, 2016, FRONT MOL NEUROSCI, V9, DOI 10.3389/fnmol.2016.00089

Lees AJ, 2009, LANCET, V373, P2055, DOI 10.1016/S0140-6736(09)60492-X

Li JL, 2016, CAN J PHYSIOL PHARM, V94, P517, DOI 10.1139/cjpp-2015-0305

Li YK, 2001, J NEUROSCI RES, V66, P163, DOI 10.1002/jnr.1208

Long-Smith CM, 2009, PROG NEUROBIOL, V89, P277, DOI 10.1016/j.pneurobio.2009.08.001

Lull ME, 2010, NEUROTHERAPEUTICS, V7, P354, DOI 10.1016/j.nurt.2010.05.014

Ma Q, 2013, ANNU REV PHARMACOL, V53, P401, DOI 10.1146/annurev-pharmtox-011112-140320

MCGEER PL, 1988, NEUROLOGY, V38, P1285, DOI 10.1212/WNL.38.8.1285

Moon HE, 2015, EXP NEUROBIOL, V24, P103, DOI 10.5607/en.2015.24.2.103

Nagamoto-Combs K, 2014, J NEUROSCI METH, V233, P187, DOI 10.1016/j.jneumeth.2014.05.021

Petersen MS, 2016, NEUROLOGY, V86

Pienaar IS, 2015, NEUROBIOL DIS, V74, P392, DOI 10.1016/j.nbd.2014.12.006

Rani PJA, 2002, J GERONTOL A-BIOL, V57, pB134, DOI 10.1093/gerona/57.4.B134

Rojo AI, 2010, GLIA, V58, P588, DOI 10.1002/glia.20947

Saiki S, 2017, SCI REP-UK, V7, DOI 10.1038/s41598-017-06767-y

Sarkar S., 2017, NEUROTOXICOLOGY, V59, P231

Serban V, 2003, ADV EXP MED BIOL, V526, P357

Subramaniam SR, 2017, FRONT AGING NEUROSCI, V9, DOI 10.3389/fnagi.2017.00176

Sun GY, 2015, PLOS ONE, V10, DOI 10.1371/journal.pone.0141509

Vedam-Mai V, 2016, NEUROMODULATION, V19, P451, DOI 10.1111/ner.12406

Wainwright MS, 2003, PEDIATR RES, V54, P688, DOI 10.1203/01.PDR.0000085036.07561.9C

NR 30

TC 22

Z9 26

U1 0

U2 5

PU AMER CHEMICAL SOC

PI WASHINGTON

PA 1155 16TH ST, NW, WASHINGTON, DC 20036 USA

SN 1948-7193

J9 ACS CHEM NEUROSCI

JI ACS Chem. Neurosci.

PD MAY

PY 2018

VL 9

IS 5

BP 901

EP 905

DI 10.1021/acschemneuro.7b00468

PG 9

WC Biochemistry & Molecular Biology; Chemistry, Medicinal; Neurosciences

WE Science Citation Index Expanded (SCI-EXPANDED)

SC Biochemistry & Molecular Biology; Pharmacology & Pharmacy; Neurosciences

& Neurology

GA GG5RC

UT WOS:000432752400005

PM 29370524

DA 2024-08-12

ER

PT J

AU Mutti, V

Carini, G

Filippini, A

Castrezzati, S

Giugno, L

Gennarelli, M

Russo, I

AF Mutti, Veronica

Carini, Giulia

Filippini, Alice

Castrezzati, Stefania

Giugno, Lorena

Gennarelli, Massimo

Russo, Isabella

TI LRRK2 Kinase Inhibition Attenuates Neuroinflammation and Cytotoxicity in

Animal Models of Alzheimer's and Parkinson's Disease-Related

Neuroinflammation

SO CELLS

LA English

DT Article

DE LRRK2; inhibitor; neuroinflammation; Parkinson's disease; Alzheimer's

disease

ID NECROSIS-FACTOR-ALPHA; AMYLOID PRECURSOR PROTEIN; REACTIVE MICROGLIA;

INNATE IMMUNITY; BRAIN; EXPRESSION; RISK; CELL

AB Chronic neuroinflammation plays a crucial role in the progression of several neurodegenerative disease (NDDs), including Parkinson's disease (PD) and Alzheimer's disease (AD). Intriguingly, in the last decade, leucine-rich repeat kinase-2 (LRRK2), a gene mutated in familial and sporadic PD, was revealed as a key mediator of neuroinflammation. Therefore, the anti-inflammatory properties of LRRK2 inhibitors have started to be considered as a disease-modifying treatment for PD; however, to date, there is little evidence on the beneficial effects of targeting LRRK2-related neuroinflammation in preclinical models. In this study, we further validated LRRK2 kinase modulation as a pharmacological intervention in preclinical models of AD- and PD-related neuroinflammation. Specifically, we reported that LRRK2 kinase inhibition with MLi2 and PF-06447475 (PF) molecules attenuated neuroinflammation, gliosis and cytotoxicity in mice with intracerebral injection of A & beta;(1-42) fibrils or & alpha;-syn preformed fibrils (pffs). Moreover, for the first time in vivo, we showed that LRRK2 kinase activity participates in AD-related neuroinflammation and therefore might contribute to AD pathogenesis. Overall, our findings added evidence on the anti-inflammatory effects of LRRK2 kinase inhibition in preclinical models and indicate that targeting LRRK2 activity could be a disease-modifying treatment for NDDs with an inflammatory component.

C1 [Mutti, Veronica; Carini, Giulia; Filippini, Alice; Gennarelli, Massimo; Russo, Isabella] IRCCS Ist Ctr San Giovanni Dio Fatebenefratelli, I-25125 Brescia, Italy.

[Carini, Giulia; Filippini, Alice; Gennarelli, Massimo; Russo, Isabella] Univ Brescia, Dept Mol & Translat Med, Biol & Genet Unit, I-25123 Brescia, Italy.

[Castrezzati, Stefania; Giugno, Lorena] Univ Brescia, Dept Biomed Sci & Biotechnol, Human Anat Unit, I-25123 Brescia, Italy.

C3 IRCCS Fatebenefratelli; University of Brescia; University of Brescia

RP Russo, I (corresponding author), IRCCS Ist Ctr San Giovanni Dio Fatebenefratelli, I-25125 Brescia, Italy.; Russo, I (corresponding author), Univ Brescia, Dept Mol & Translat Med, Biol & Genet Unit, I-25123 Brescia, Italy.

EM vmutti@fatebenefratelli.eu; isabella.russo@unibs.it

RI Carini, Giulia/neurodegenerative disease-8800-2022

OI Carini, Giulia/0000-0001-8096-9417; MUTTI, VERONICA/0000-0001-7969-8744;

Filippini, Alice/0000-0001-6271-148X

CR Ahmad S, 2021, INT J MOL SCI, V22, DOI 10.3390/ijms22179583

Almer G, 2001, ANN NEUROL, V49, P176, DOI 10.1002/1531-8249(20010201)49:2<176::AID-ANA37>3.3.CO;2-O

Bae EJ, 2020, BBA-MOL BASIS DIS, V1866, DOI 10.1016/j.bbadis.2019.165632

Bae EJ, 2018, NAT COMMUN, V9, DOI 10.1038/s41467-018-05958-z

Bieri G, 2019, ACTA NEUROPATHOL, V137, P961, DOI 10.1007/s00401-019-01995-0

Brendecke SM, 2015, SEMIN IMMUNOPATHOL, V37, P591, DOI 10.1007/s00281-015-0520-6

Chen JY, 2018, BIOCHEM BIOPH RES CO, V498, P171, DOI 10.1016/j.bbrc.2018.02.007

Daher JPL, 2015, J BIOL CHEM, V290, P19433, DOI 10.1074/jbc.M115.660001

Daher JPL, 2014, P NATL ACAD SCI USA, V111, P9289, DOI 10.1073/pnas.1403215111

Dzamko N, 2010, BIOCHEM J, V430, P405, DOI 10.1042/BJ20100784

Filippini A, 2023, BIOMOLECULES, V13, DOI 10.3390/biom13020307

Filippini A, 2021, BIOCHEM SOC T, V49, P1375, DOI 10.1042/BST20201092

Filippini A, 2021, GLIA, V69, P681, DOI 10.1002/glia.23920

Filippone A, 2022, ANTIOXIDANTS-BASEL, V11, DOI 10.3390/antiox11091634

Gao HM, 2008, TRENDS IMMUNOL, V29, P357, DOI 10.1016/j.it.2008.05.002

Griffin WST, 2006, J NEUROINFLAMM, V3, DOI 10.1186/1742-2094-3-5

Griffiths M, 2007, INT REV NEUROBIOL, V82, P29, DOI 10.1016/S0074-7742(07)82002-2

Hirsch EC, 2009, LANCET NEUROL, V8, P382, DOI 10.1016/S1474-4422(09)70062-6

Ho DH, 2017, BIOCHEM BIOPH RES CO, V482, P1088, DOI 10.1016/j.bbrc.2016.11.163

Ising C, 2018, CELL DEATH DIS, V9, DOI 10.1038/s41419-017-0153-x

Kwon HS, 2020, TRANSL NEURODEGENER, V9, DOI 10.1186/s40035-020-00221-2

Labzin LI, 2018, ANNU REV MED, V69, P437, DOI 10.1146/annurev-med-050715-104343

Lee MS, 2003, J CELL BIOL, V163, P83, DOI 10.1083/jcb.200301115

Liao YF, 2004, J BIOL CHEM, V279, P49523, DOI 10.1074/jbc.M402034200

Lin X, 2009, NEURON, V64, P807, DOI 10.1016/j.neuron.2009.11.006

Liy PM, 2021, EXP BIOL MED, V246, P2399, DOI 10.1177/1535370221997052

Maekawa T, 2016, BMC NEUROSCI, V17, DOI 10.1186/s12868-016-0315-2

Marín I, 2006, MOL BIOL EVOL, V23, P2423, DOI 10.1093/molbev/msl114

Marker DF, 2012, J NEUROINFLAMM, V9, DOI 10.1186/1742-2094-9-261

MCGEER PL, 1987, NEUROSCI LETT, V79, P195, DOI 10.1016/0304-3940(87)90696-3

MCGEER PL, 1988, NEUROLOGY, V38, P1285, DOI 10.1212/WNL.38.8.1285

McGregor MM, 2019, NEURON, V101, P1042, DOI 10.1016/j.neuron.2019.03.004

Moehle MS, 2012, J NEUROSCI, V32, P1602, DOI 10.1523/JNEUROSCI.5601-11.2012

MOGI M, 1994, NEUROSCI LETT, V165, P208, DOI 10.1016/0304-3940(94)90746-3

Munoz L, 2015, EUR J MED CHEM, V95, P29, DOI 10.1016/j.ejmech.2015.03.003

Nogawa S, 1997, J NEUROSCI, V17, P2746

Novello S, 2022, NEUROBIOL DIS, V162, DOI 10.1016/j.nbd.2021.105579

Justo AFO, 2022, J CELL COMMUN SIGNAL, V16, P155, DOI 10.1007/s12079-021-00663-x

Padmanabhan S, 2020, J PARKINSON DIS, V10, P623, DOI 10.3233/JPD-191786

Paisán-Ruiz C, 2013, J PARKINSON DIS, V3, P85, DOI 10.3233/JPD-130192

Perez-Nievas BG, 2018, FRONT AGING NEUROSCI, V10, DOI 10.3389/fnagi.2018.00114

Puccini JM, 2015, J NEUROSCI, V35, P5271, DOI 10.1523/JNEUROSCI.0650-14.2015

Rao YL, 2022, 3 BIOTECH, V12, DOI 10.1007/s13205-022-03123-4

Russo I, 2022, NEUROBIOL DIS, V169, DOI 10.1016/j.nbd.2022.105724

Russo I, 2019, NEUROBIOL DIS, V129, P67, DOI 10.1016/j.nbd.2019.05.012

Russo I, 2018, J NEUROINFLAMM, V15, DOI 10.1186/s12974-018-1337-8

Russo I, 2015, J NEUROINFLAMM, V12, DOI 10.1186/s12974-015-0449-7

Russo I, 2014, J NEUROINFLAMM, V11, DOI 10.1186/1742-2094-11-52

Russo I, 2011, J NEUROCHEM, V116, P947, DOI 10.1111/j.1471-4159.2010.07168.x

Sastre M, 2003, J NEUROSCI, V23, P9796

Satake W, 2009, NAT GENET, V41, P1303, DOI 10.1038/ng.485

Schapansky J, 2014, HUM MOL GENET, V23, P4201, DOI 10.1093/hmg/ddu138

Sonninen TM, 2020, SCI REP-UK, V10, DOI 10.1038/s41598-020-71329-8

Tweedie D, 2012, J NEUROINFLAMM, V9, DOI 10.1186/1742-2094-9-106

Van der Perren A, 2021, NEUROTHERAPEUTICS, V18, P949, DOI 10.1007/s13311-021-01007-8

Wang WY, 2015, ANN TRANSL MED, V3, DOI 10.3978/j.issn.2305-5839.2015.03.49

Zheng C, 2016, TRANSL NEURODEGENER, V5, DOI 10.1186/s40035-016-0054-4

Zimprich A, 2004, NEURON, V44, P601, DOI 10.1016/j.neuron.2004.11.005

NR 58

TC 8

Z9 8

U1 1

U2 5

PU MDPI

PI BASEL

PA ST ALBAN-ANLAGE 66, CH-4052 BASEL, SWITZERLAND

EI 2073-4409

J9 CELLS-BASEL

JI Cells

PD JUL

PY 2023

VL 12

IS 13

AR 1799

DI 10.3390/cells12131799

PG 16

WC Cell Biology

WE Science Citation Index Expanded (SCI-EXPANDED)

SC Cell Biology

GA M2GQ0

UT WOS:001028421300001

PM 37443833

OA Green Published, gold

DA 2024-08-12

ER

PT J

AU Liu, L

Zhou, TT

Li, T

Liang, ZH

Luo, XG

AF Liu, Lin

Zhou, Tingting

Li, Tao

Liang, Zhanhua

Luo, Xiaoguang

TI LncRNA DLX6-AS1 promotes microglial inflammatory response in Parkinson?s

disease by regulating the miR-223-3p/NRP1 axis

SO BEHAVIOURAL BRAIN RESEARCH

LA English

DT Article

DE LncRNA DLX6-AS1; parkinson's disease; Microglia; NLRP3; MiR-223-3p;

NRP1; Neurological function; Inflammatory reaction

ID LYMPH-NODES; EXPRESSION; NEUROINFLAMMATION; MICRORNAS; APOPTOSIS;

PATHWAY; CELLS; MODEL

AB Parkinson's disease (PD) is a prevailing neurodegenerative disorder. This study discussed the mechanism of lncRNA distal-less homeobox 6 antisense 1 (DLX6-AS1) on inflammatory responses in PD. With healthy male C57BL/6 mice (8-10 weeks) and BV2 microglia as study subjects, we established PD models in vivo/in vitro by injection of 1-methyl-4-phenyl-2, 3, 6-tetrahydropyridine (MPTP) for 4 weeks and treatment of lipopolysaccharide (LPS) for 24 h, respectively. DLX6-AS1 expression in PD mice and BV2 microglia was examined using reverse transcription quantitative-polymerase chain reaction and then down-regulated via stereotaxic catheter injection or cell transfection to evaluate its effect on neurological function. Meanwhile, the cell number of TH+ /Caspase3 + /IBA1 + in substantia nigra, cell viability, and apoptosis rate of BV2 microglia, inflammatory levels, and NLR family pyrin domain containing 3 (NLRP3) inflammasome were determined using immunohistochemistry, MTT assay, flow cytometry, ELIZA assay, and Western blot. The binding relationship between miR223-3p and DLX6-AS1/Neuropilin-1 (NRP1) was verified by dual-luciferase assay and RNA immunoprecipitation assay. After down-regulation of DLX6-AS1, we down-regulated/overexpressed miR-223-3p/NRP1 levels in BV2 microglia. DLX6-AS1 was overexpressed in PD mice. Silencing DLX6-AS1 improved neurological function and alleviated microglial inflammation in PD mice. Specifically, the latency of mice falling from the rotating rod was longer, and the latency of climbing rod test was shorter; TH+ cells increased, while Caspase3 + /IBA1 + cells decreased; the levels of inflammatory were lowered. Silencing DLX6-AS1 inhibited LPS-induced inflammation of BV2 microglia. DLX6-AS1 acted as the ceRNA of miR-223-3p to promote NRP1. Down-regulation of miR-223-3p or overexpression of NRP1 partially annulled the effect of silencing DLX6-AS1 on BV2 microglial inflammation. Overall, DLX6-AS1 promotes the microglial inflammatory response in PD through the ceRNA mechanism of miR223-3p/NRP1.

C1 [Liu, Lin; Zhou, Tingting; Li, Tao; Liang, Zhanhua] Dalian Med Univ, Dept Neurol, Affiliated Hosp 1, 222 Zhongshan Rd, Dalian 116011, Liaoning, China.

[Luo, Xiaoguang] Shenzhen Peoples Hosp, Dept Neurol, Shenzhen 518020, Guangdong, China.

C3 Dalian Medical University; Jinan University

RP Liang, ZH (corresponding author), Dalian Med Univ, Dept Neurol, Affiliated Hosp 1, 222 Zhongshan Rd, Dalian 116011, Liaoning, China.

EM liangzhanhua@dmu.edu.cn

CR Bartel DP, 2009, CELL, V136, P215, DOI 10.1016/j.cell.2009.01.002

Battaglia A, 2008, IMMUNOLOGY, V123, P129, DOI 10.1111/j.1365-2567.2007.02737.x

Battaglia A, 2010, INT J RADIAT ONCOL, V76, P1546, DOI 10.1016/j.ijrobp.2009.10.014

Bauernfeind F, 2012, J IMMUNOL, V189, P4175, DOI 10.4049/jimmunol.1201516

Bosseboeuf E, 2020, CELLS-BASEL, V9, DOI 10.3390/cells9092055

Boxberger N, 2019, J IMMUNOL, V202, P2177, DOI 10.4049/jimmunol.1801416

Cao H, 2021, AGING-US, V13, P11455, DOI 10.18632/aging.202837

Chaudhary B, 2014, CANCER IMMUNOL IMMUN, V63, P81, DOI 10.1007/s00262-013-1500-0

Chen LJ, 2019, PATHOL RES PRACT, V215, P12, DOI 10.1016/j.prp.2018.11.012

Codolo G, 2013, PLOS ONE, V8, DOI 10.1371/journal.pone.0055375

Douyère M, 2021, FRONT ONCOL, V11, DOI 10.3389/fonc.2021.665634

Du C, 2020, CELL TRANSPLANT, V29, DOI 10.1177/0963689720929983

Fan Y, 2020, BRAIN RES BULL, V157, P119, DOI 10.1016/j.brainresbull.2020.02.003

Garai K, 2021, FRONT PHYSIOL, V12, DOI 10.3389/fphys.2021.658218

Goh SY, 2019, INT J MOL SCI, V20, DOI 10.3390/ijms20225649

Hu XM, 2020, AGING-US, V12, P23096, DOI 10.18632/aging.104070

Huang Y, 2022, MOL IMMUNOL, V143, DOI 10.1016/j.molimm.2022.01.002

Jia PS, 2020, IUBMB LIFE, V72, P2627, DOI 10.1002/iub.2392

Juzwik CA, 2019, PROG NEUROBIOL, V182, DOI 10.1016/j.pneurobio.2019.101664

Kakhaki RD, 2020, CLIN NEUROL NEUROSUR, V192, DOI 10.1016/j.clineuro.2020.105833

Kong WQ, 2021, CANCER BIOTHER RADIO, V36, P753, DOI 10.1089/cbr.2020.3589

Leggio L, 2017, INT J MOL SCI, V18, DOI 10.3390/ijms18122698

Li JN, 2021, FRONT CELL DEV BIOL, V9, DOI 10.3389/fcell.2021.671244

Li Q, 2021, NEUROTOXICOLOGY, V87, P62, DOI 10.1016/j.neuro.2021.08.018

Lim KH, 2021, FRONT GENET, V12, DOI 10.3389/fgene.2021.741175

Liu RH, 2020, PLACENTA, V93, P34, DOI 10.1016/j.placenta.2020.02.001

Lu Y, 2020, J CELL BIOCHEM, V121, P4838, DOI 10.1002/jcb.29711

Lv QK, 2020, PARKINSONS DIS-US, V2020, DOI 10.1155/2020/5374307

Mahmoudian-Sani MR, 2018, AUDIOL NEURO-OTOL, V23, P208, DOI 10.1159/000493557

Martín-Fernández F, 2022, ELIFE, V11, DOI [10.7554/eLife.69776, 10.7554/eLife.69776.sa0, 10.7554/eLife.69776.sa1, 10.7554/eLife.69776.sa2]

Mayi BS, 2021, PLOS PATHOG, V17, DOI 10.1371/journal.ppat.1009153

Mone Pasquale, 2021, Res Sq, DOI 10.21203/rs.3.rs-192099/v1

Moore AM, 2020, NEUROBIOL AGING, V87, P18, DOI 10.1016/j.neurobiolaging.2019.10.021

Morquette B, 2019, BRAIN, V142, P2979, DOI 10.1093/brain/awz245

Oe S, 2019, FRONT BIOSCI-LANDMRK, V24, P1203, DOI 10.2741/4776

Qi L, 2016, J NEUROL SCI, V370, P21, DOI 10.1016/j.jns.2016.08.061

Rezaei O, 2021, EUR J PHARMACOL, V896, DOI 10.1016/j.ejphar.2021.173914

Shen M, 2022, EXP CELL RES, V412, DOI 10.1016/j.yexcr.2022.113019

Shen WB, 2022, bioRxiv, DOI [10.1101/2022.01.31.478476, 10.1101/2022.01.31.478476, DOI 10.1101/2022.01.31.478476]

Sheng YY, 2019, J CELL BIOCHEM, V120, P10737, DOI 10.1002/jcb.28365

Singh SS, 2020, NEUROTOX RES, V37, P491, DOI 10.1007/s12640-019-00147-2

Tan JX, 2020, J BIOENERG BIOMEMBR, V52, P367, DOI 10.1007/s10863-020-09845-5

Tay Y, 2014, NATURE, V505, P344, DOI 10.1038/nature12986

Tian J, 2021, CLIN EXP IMMUNOL, V204, P396, DOI 10.1111/cei.13587

Tysnes OB, 2017, J NEURAL TRANSM, V124, P901, DOI 10.1007/s00702-017-1686-y

Wang LP, 2021, J EXP CLIN CANC RES, V40, DOI 10.1186/s13046-021-01973-z

Wang X, 2021, INT J RHEUM DIS, V24, P599, DOI 10.1111/1756-185X.14089

Wu SH, 2022, INFLAMMATION, V45, P1162, DOI 10.1007/s10753-021-01610-z

Xia B, 2020, THERANOSTICS, V10, P8974, DOI 10.7150/thno.44912

Xiao X, 2021, NEUROPSYCH DIS TREAT, V17, P1153, DOI 10.2147/NDT.S286778

Xu W, 2020, INT IMMUNOPHARMACOL, V85, DOI 10.1016/j.intimp.2020.106614

Xu WJ, 2020, RESP RES, V21, DOI 10.1186/s12931-020-01374-4

Xue HG, 2016, MED SCI MONITOR, V22, P4779, DOI 10.12659/MSM.896225

Zalpoor H, 2022, CELL MOL BIOL LETT, V27, DOI 10.1186/s11658-022-00311-1

Zhang L, 2017, BIOMED PHARMACOTHER, V96, P884, DOI 10.1016/j.biopha.2017.10.056

Zhang Y, 2020, NEUROCHEM RES, V45, P1700, DOI 10.1007/s11064-020-03035-w

Zhou SF, 2021, BRAIN RES, V1750, DOI 10.1016/j.brainres.2020.147156

NR 57

TC 8

Z9 8

U1 0

U2 16

PU ELSEVIER

PI AMSTERDAM

PA RADARWEG 29, 1043 NX AMSTERDAM, NETHERLANDS

SN 0166-4328

EI 1872-7549

J9 BEHAV BRAIN RES

JI Behav. Brain Res.

PD AUG 5

PY 2022

VL 431

AR 113923

DI 10.1016/j.bbr.2022.113923

EA JUN 2022

PG 13

WC Behavioral Sciences; Neurosciences

WE Science Citation Index Expanded (SCI-EXPANDED)

SC Behavioral Sciences; Neurosciences & Neurology

GA 2F6HO

UT WOS:000813008700001

PM 35550840

DA 2024-08-12

ER

PT J

AU Grimmig, B

Morganti, J

Nash, K

Bickford, PC

AF Grimmig, Bethany

Morganti, Josh

Nash, Kevin

Bickford, Paula C.

TI Immunomodulators as Therapeutic Agents in Mitigating the Progression of

Parkinson's Disease

SO BRAIN SCIENCES

LA English

DT Review

DE Parkinson's disease; neuroinflammation; microglia; fractalkine;

astaxanthin

ID MITOCHONDRIAL OXIDANT STRESS; ALPHA-SYNUCLEIN; MICROGLIAL ACTIVATION;

OXIDATIVE STRESS; ASTAXANTHIN PROTECTS; DOPAMINERGIC-NEURONS; MOUSE

MODEL; DOWN-REGULATION; CELL-DEATH; FRACTALKINE

AB Parkinson's disease (PD) is a common neurodegenerative disorder that primarily afflicts the elderly. It is characterized by motor dysfunction due to extensive neuron loss in the substantia nigra pars compacta. There are multiple biological processes that are negatively impacted during the pathogenesis of PD, and are implicated in the cell death in this region. Neuroinflammation is evidently involved in PD pathology and mitigating the inflammatory cascade has been a therapeutic strategy. Age is the number one risk factor for PD and thus needs to be considered in the context of disease pathology. Here, we discuss the role of neuroinflammation within the context of aging as it applies to the development of PD, and the potential for two representative compounds, fractalkine and astaxanthin, to attenuate the pathophysiology that modulates neurodegeneration that occurs in Parkinson's disease.

C1 [Grimmig, Bethany; Bickford, Paula C.] Univ S Florida, Morsani Coll Med, Ctr Excellence Aging & Brain Repair, Dept Neurosurg & Brain Repair, Tampa, FL 33612 USA.

[Morganti, Josh] Univ Kentucky, Dept Anat & Neurobiol, Sanders Brown Ctr Aging, Lexington, KY 40508 USA.

[Nash, Kevin] Univ S Florida, Morsani Coll Med, Dept Mol Pharmacol & Physiol, Byrd Alzheimers Inst, Tampa, FL 33613 USA.

[Bickford, Paula C.] James A Haley VA Hosp, 13000 Bruce B Downs Blvd, Tampa, FL 33612 USA.

C3 State University System of Florida; University of South Florida;

University of Kentucky; State University System of Florida; University

of South Florida

RP Bickford, PC (corresponding author), Univ S Florida, Morsani Coll Med, Ctr Excellence Aging & Brain Repair, Dept Neurosurg & Brain Repair, Tampa, FL 33612 USA.; Bickford, PC (corresponding author), James A Haley VA Hosp, 13000 Bruce B Downs Blvd, Tampa, FL 33612 USA.

EM bgrimmig@health.usf.edu; josh.morganti@uky.edu; Knash@health.usf.edu;

pbickfor@health.usf.edu

RI Nash, Kevin/B-4567-2009; Morganti, Josh/AAL-9727-2020; Bickford,

Paula/J-5970-2012

OI Morganti, Josh/0000-0002-4183-0049; Bickford, Paula/0000-0001-9657-7725

FU NIA [AG04418, AG044919]; VA [IO1BX000231]; Michael J Fox foundation;

federal government

FX This work was supported by NIA grants AG04418 (PCB), AG044919 (PCB); VA

IO1BX000231 (PCB), and the Michael J Fox foundation (KN/PCB). This work

was supported by the federal government. The contents of this manuscript

do not represent the views of the Department of Veterans Affairs or the

United States Government.

CR Augusti PR, 2012, J CLIN BIOCHEM NUTR, V51, P42, DOI 10.3164/jcbn.11-74

Bachstetter AD, 2011, NEUROBIOL AGING, V32, P2030, DOI 10.1016/j.neurobiolaging.2009.11.022

Bharath S, 2002, BIOCHEM PHARMACOL, V64, P1037, DOI 10.1016/S0006-2952(02)01174-7

Braak H, 2004, CELL TISSUE RES, V318, P121, DOI 10.1007/s00441-004-0956-9

Brunk UT, 2002, EUR J BIOCHEM, V269, P1996, DOI 10.1046/j.1432-1033.2002.02869.x

Cao SW, 2012, J NEUROINFLAMM, V9, DOI 10.1186/1742-2094-9-259

Cao SW, 2010, MOL NEURODEGENER, V5, DOI 10.1186/1750-1326-5-42

Cardona AE, 2006, NAT NEUROSCI, V9, P917, DOI 10.1038/nn1715

Cho SH, 2015, J NEUROSCI, V35, P807, DOI 10.1523/JNEUROSCI.2939-14.2015

Choi SK, 2008, J MICROBIOL BIOTECHN, V18, P1990, DOI 10.4014/jmb.0800.489

Codolo G, 2013, PLOS ONE, V8, DOI 10.1371/journal.pone.0055375

Collier TJ, 2011, NAT REV NEUROSCI, V12, P359, DOI 10.1038/nrn3039

Danzer KM, 2007, J NEUROSCI, V27, P9220, DOI 10.1523/JNEUROSCI.2617-07.2007

Dias V, 2013, J PARKINSON DIS, V3, P461, DOI 10.3233/JPD-130230

Dryanovski DI, 2013, J NEUROSCI, V33, P10154, DOI 10.1523/JNEUROSCI.5311-12.2013

Du YS, 2001, P NATL ACAD SCI USA, V98, P14669, DOI 10.1073/pnas.251341998

Gerhard A, 2006, MOVEMENT DISORD, V21, P89, DOI 10.1002/mds.20668

Goldman SM, 2006, ANN NEUROL, V60, P65, DOI 10.1002/ana.20882

Hickman SE, 2013, NAT NEUROSCI, V16, P1896, DOI 10.1038/nn.3554

HIRSCH E, 1988, NATURE, V334, P345, DOI 10.1038/334345a0

Hurtig HI, 2000, NEUROLOGY, V54, P1916, DOI 10.1212/WNL.54.10.1916

Jones BA, 2010, MOL INTERV, V10, P263, DOI 10.1124/mi.10.5.3

Jurgens HA, 2012, EXP NEUROL, V233, P40, DOI 10.1016/j.expneurol.2010.11.014

Kidd P, 2011, ALTERN MED REV, V16, P355

Kim YH, 2010, INT IMMUNOPHARMACOL, V10, P1560, DOI 10.1016/j.intimp.2010.09.007

Knott C, 2000, MOL CELL NEUROSCI, V16, P724, DOI 10.1006/mcne.2000.0914

Kowal SL, 2013, MOVEMENT DISORD, V28, P311, DOI 10.1002/mds.25292

Lashuel HA, 2002, J MOL BIOL, V322, P1089, DOI 10.1016/S0022-2836(02)00735-0

LAWSON LJ, 1990, NEUROSCIENCE, V39, P151, DOI 10.1016/0306-4522(90)90229-W

Lee DH, 2011, FOOD CHEM TOXICOL, V49, P271, DOI 10.1016/j.fct.2010.10.029

Lee DC, 2013, NEUROBIOL AGING, V34, P1610, DOI 10.1016/j.neurobiolaging.2012.12.014

Lee SM, 2004, J NEUROCHEM, V91, P568, DOI 10.1111/j.1471-4159.2004.02780.x

Lee S, 2014, J NEUROSCI, V34, P12538, DOI 10.1523/JNEUROSCI.0853-14.2014

Li ZR, 2013, MOL VIS, V19, P1656

Liu XB, 2009, BRAIN RES, V1254, P18, DOI 10.1016/j.brainres.2008.11.076

Luth ES, 2014, J BIOL CHEM, V289, P21490, DOI 10.1074/jbc.M113.545749

MCGEER PL, 1988, ANN NEUROL, V24, P574, DOI 10.1002/ana.410240415

Minogue AM, 2014, NEUROBIOL AGING, V35, P1442, DOI 10.1016/j.neurobiolaging.2013.12.026

Morganti JM, 2012, J NEUROSCI, V32, P14592, DOI 10.1523/JNEUROSCI.0539-12.2012

Nash KR, 2015, MOL THER, V23, P17, DOI 10.1038/mt.2014.175

Nash KR, 2013, NEUROBIOL AGING, V34, P1540, DOI 10.1016/j.neurobiolaging.2012.12.011

Norden DM, 2013, NEUROPATH APPL NEURO, V39, P19, DOI 10.1111/j.1365-2990.2012.01306.x

Norden DM, 2015, NEUROPHARMACOLOGY, V96, P29, DOI 10.1016/j.neuropharm.2014.10.028

Otton R, 2010, CHEM-BIOL INTERACT, V186, P306, DOI 10.1016/j.cbi.2010.05.011

Pabon MM, 2012, PLOS ONE, V7, DOI 10.1371/journal.pone.0045256

Pabon MM, 2011, J NEUROINFLAMM, V8, DOI 10.1186/1742-2094-8-9

Paolicelli RC, 2011, NEURON GLIA BIOL, V7, P77, DOI 10.1017/S1740925X12000105

Park J., 2012, J ANIM SCI, V91

Polinski NK, 2015, NEUROBIOL AGING, V36, P1110, DOI 10.1016/j.neurobiolaging.2014.07.047

Qin YW, 2015, INFLAMMATION, V38, P1609, DOI 10.1007/s10753-015-0136-y

Rees K, 2011, COCHRANE DB SYST REV, DOI 10.1002/14651858.CD008454.pub2

Reeve A, 2014, AGEING RES REV, V14, P19, DOI 10.1016/j.arr.2014.01.004

Reynolds AD, 2008, J NEUROCHEM, V104, P1504, DOI 10.1111/j.1471-4159.2007.05087.x

Sanchez-Padilla J, 2014, NAT NEUROSCI, V17, P832, DOI 10.1038/nn.3717

Schafer DP, 2012, NEURON, V74, P691, DOI 10.1016/j.neuron.2012.03.026

Sharma N, 2015, NEUROCHEM INT, V87, P92, DOI 10.1016/j.neuint.2015.06.004

Simuni T, 2013, MOVEMENT DISORD, V28, P1823, DOI 10.1002/mds.25639

Stefanis L, 2012, CSH PERSPECT MED, V2, DOI 10.1101/cshperspect.a009399

Stefanovic AND, 2014, FEBS J, V281, P2838, DOI 10.1111/febs.12824

Surmeier DJ, 2011, NEUROSCIENCE, V198, P221, DOI 10.1016/j.neuroscience.2011.08.045

Surmeier DJ, 2010, PROG BRAIN RES, V183, P59, DOI 10.1016/S0079-6123(10)83004-3

Surmeier DJ, 2010, CELL CALCIUM, V47, P175, DOI 10.1016/j.ceca.2009.12.003

Tanaka M, 2004, J BIOL CHEM, V279, P4625, DOI 10.1074/jbc.M310994200

Thome AD, 2015, PLOS ONE, V10, DOI 10.1371/journal.pone.0140566

Venkateshappa C, 2012, NEUROCHEM RES, V37, P358, DOI 10.1007/s11064-011-0619-7

Volpicelli-Daley LA, 2014, MOL BIOL CELL, V25, P4010, DOI 10.1091/mbc.E14-02-0741

Wang HQ, 2010, BRAIN RES, V1360, P159, DOI 10.1016/j.brainres.2010.08.100

Wolf AM, 2010, J NUTR BIOCHEM, V21, P381, DOI 10.1016/j.jnutbio.2009.01.011

Wynne AM, 2010, BRAIN BEHAV IMMUN, V24, P1190, DOI 10.1016/j.bbi.2010.05.011

Ye QY, 2012, BMC NEUROSCI, V13, DOI 10.1186/1471-2202-13-156

Zhang W, 2005, FASEB J, V19, P533, DOI 10.1096/fj.04-2751com

NR 71

TC 16

Z9 19

U1 0

U2 8

PU MDPI

PI BASEL

PA ST ALBAN-ANLAGE 66, CH-4052 BASEL, SWITZERLAND

EI 2076-3425

J9 BRAIN SCI

JI Brain Sci.

PD DEC

PY 2016

VL 6

IS 4

AR 41

DI 10.3390/brainsci6040041

PG 12

WC Neurosciences

WE Science Citation Index Expanded (SCI-EXPANDED)

SC Neurosciences & Neurology

GA EH3VJ

UT WOS:000391699800001

PM 27669315

OA Green Published, Green Submitted, gold

DA 2024-08-12

ER

PT J

AU Meng, F

Guo, ZG

Hu, YL

Mai, WH

Zhang, ZJ

Zhang, B

Ge, QQ

Lou, HF

Guo, F

Chen, JF

Duan, SM

Gao, ZH

AF Meng, Fan

Guo, Zhige

Hu, Yaling

Mai, Weihao

Zhang, Zhenjie

Zhang, Bin

Ge, Qianqian

Lou, Huifang

Guo, Fang

Chen, Jiangfan

Duan, Shumin

Gao, Zhihua

TI CD73-derived adenosine controls inflammation and neurodegeneration by

modulating dopamine signalling

SO BRAIN

LA English

DT Article

DE CD73; adenosine A2AR; microglia; neuroinflammation; Parkinson's disease

ID A(2A) RECEPTOR REGULATION; parkinson's disease; MICROGLIAL RESPONSE;

EXTRACELLULAR ATP; TISSUE-DAMAGE; A2A RECEPTOR; BRAIN-INJURY; MOUSE

MODEL; NEUROINFLAMMATION; NEUROPROTECTION

AB Ectonucleotidase-mediated ATP catabolism provides a powerful mechanism to control the levels of extracellular adenosine. While increased adenosine A(2A) receptor (A(2A)R) signaling has been well-documented in both Parkinson's disease models and patients, the source of this enhanced adenosine signalling remains unclear. Here, we show that the ecto-5'-nucleotidase (CD73)-mediated adenosine formation provides an important input to activate A(2A)R, and upregulated CD73 and A(2A)R in the 1-methyl-4-phenyl-1,2,3,6-tetrahydropyridine (MPTP)-induced Parkinson's disease models coordinatively contribute to the elevated adenosine signalling. Importantly, we demonstrate that CD73-derived adenosine-A(2A)R signalling modulates microglial immunoresponses and morphological dynamics. CD73 inactivation significantly attenuated lipopolysaccharide-induced pro-inflammatory responses in microglia, but enhanced microglia process extension, movement and morphological transformation in the laser injury and acute MPTP-induced Parkinson's disease models. Limiting CD73-derived adenosine substantially suppressed microglia-mediated neuroinflammation and improved the viability of dopaminergic neurons and motor behaviours in Parkinson's disease models. Moreover, CD73 inactivation suppressed A(2A)R induction and A(2A)R-mediated pro-inflammatory responses, whereas replenishment of adenosine analogues restored these effects, suggesting that CD73 produces a self-regulating feed-forward adenosine formation to activate A(2A)R and promote neuroinflammation. We further provide the first evidence that A(2A) enhanced inflammation by antagonizing dopamine-mediated anti-inflammation, suggesting that the homeostatic balance between adenosine and dopamine signalling is key to microglia immunoresponses. Our study thus reveals a novel role for CD73-mediated nucleotide metabolism in regulating neuroinflammation and provides the proof-of-principle that targeting nucleotide metabolic pathways to limit adenosine production and neuroinflammation in Parkinson's disease might be a promising therapeutic strategy.

C1 [Meng, Fan; Guo, Zhige; Hu, Yaling; Mai, Weihao; Zhang, Zhenjie; Zhang, Bin; Ge, Qianqian; Lou, Huifang; Guo, Fang; Chen, Jiangfan; Duan, Shumin; Gao, Zhihua] Zhejiang Univ, Sch Med, Dept Neurobiol, Hangzhou, China.

[Meng, Fan; Guo, Zhige; Hu, Yaling; Mai, Weihao; Zhang, Zhenjie; Zhang, Bin; Ge, Qianqian; Lou, Huifang; Guo, Fang; Chen, Jiangfan; Duan, Shumin; Gao, Zhihua] Zhejiang Univ, Sch Med, Affiliated Hosp 2, NHC & CAMS Key Lab Med Neurobiol, Hangzhou, China.

[Chen, Jiangfan] Sch Optometry & Ophthalmol, Mol Neuropharmacol Lab, Wenzhou, Zhejiang, China.

[Chen, Jiangfan] Sch Optometry & Ophthalmol, State Key Lab Optometry Ophthalmol & Vis Sci, Wenzhou, Zhejiang, China.

C3 Zhejiang University; Zhejiang University

RP Duan, SM; Gao, ZH (corresponding author), Zhejiang Univ, Sch Med, Dept Neurobiol, Hangzhou, China.; Duan, SM; Gao, ZH (corresponding author), Zhejiang Univ, Sch Med, Affiliated Hosp 2, NHC & CAMS Key Lab Med Neurobiol, Hangzhou, China.

EM duanshumin@zju.edu.cn; zhihuagao@zju.edu.cn

RI Zhang, Bin/C-9887-2013; Hu, Yaling/GXG-3705-2022

OI Zhang, Bin/0000-0002-2597-0578; Chen, Jiang-Fan/0000-0002-0446-3956;

Gao, Zhihua/0000-0001-8603-0777; Guo, Zhige/0000-0002-7769-8326; Meng,

Fan/0000-0001-6558-0905

FU National Key Research and Development Program of China [2016YFA0501000,

2017YFA0104200]; National Natural Science Foundation of China [31471308,

31490590, 31671057, 81521062]; Fundamental Research Funds for the

Central Universities [2018FZA7004]; Chinese Ministry of Education

Project 111 Program [B13026]

FX This work was supported by the National Key Research and Development

Program of China (2016YFA0501000; 2017YFA0104200), the National Natural

Science Foundation of China (31471308; 31490590; 31671057; 81521062),

the Fundamental Research Funds for the Central Universities

(2018FZA7004) and the Chinese Ministry of Education Project 111 Program

(B13026).

CR Adolfsson O, 2012, J NEUROSCI, V32, P9677, DOI 10.1523/JNEUROSCI.4742-11.2012

Ahmad S, 2013, J NEUROIMMUNOL, V264, P54, DOI 10.1016/j.jneuroim.2013.09.015

Augusto E, 2013, J NEUROSCI, V33, P11390, DOI 10.1523/JNEUROSCI.5817-12.2013

Azdad K, 2009, NEUROPSYCHOPHARMACOL, V34, P972, DOI 10.1038/npp.2008.144

Birbeck JA, 2013, ANAL CHEM, V85, P7398, DOI 10.1021/ac4013144

Boia R, 2017, CELL DEATH DIS, V8, DOI 10.1038/cddis.2017.451

Boison D, 2011, CURR TOP MED CHEM, V11, P1068, DOI 10.2174/156802611795347609

Braun N, 1998, J NEUROSCI, V18, P4891

Braun N, 1997, BRAIN RES, V766, P213, DOI 10.1016/S0006-8993(97)00559-3

Caetano L, 2017, MOL PSYCHIATR, V22, P1035, DOI 10.1038/mp.2016.173

Calon F, 2004, BRAIN, V127, P1075, DOI 10.1093/brain/awh128

Chen JF, 2001, J NEUROSCI, V21, part. no., DOI 10.1523/JNEUROSCI.21-10-j0001.2001

Chen JF, 2007, PROG NEUROBIOL, V83, P310, DOI 10.1016/j.pneurobio.2007.09.002

Colella M, 2018, FRONT NEUROL, V9, DOI 10.3389/fneur.2018.00605

Cunha RA, 2016, J NEUROCHEM, V139, P1019, DOI 10.1111/jnc.13724

Dai SS, 2010, J NEUROSCI, V30, P5802, DOI 10.1523/JNEUROSCI.0268-10.2010

Davalos D, 2005, NAT NEUROSCI, V8, P752, DOI 10.1038/nn1472

Di Virgilio F, 2009, TRENDS NEUROSCI, V32, P79, DOI 10.1016/j.tins.2008.11.003

Double Kay L, 2012, Parkinsonism Relat Disord, V18 Suppl 1, pS52, DOI 10.1016/S1353-8020(11)70018-9

Ena SL, 2013, J NEUROSCI, V33, P8794, DOI 10.1523/JNEUROSCI.2989-12.2013

Eyo UB, 2015, J NEUROSCI, V35, P2417, DOI 10.1523/JNEUROSCI.3279-14.2015

Färber K, 2006, PFLUG ARCH EUR J PHY, V452, P615, DOI 10.1007/s00424-006-0064-7

Ferré S, 2007, TRENDS NEUROSCI, V30, P440, DOI 10.1016/j.tins.2007.07.001

Frakes AE, 2014, NEURON, V81, P1009, DOI 10.1016/j.neuron.2014.01.013

Fredholm BB, 2007, PROG NEUROBIOL, V83, P263, DOI 10.1016/j.pneurobio.2007.07.005

Furman D, 2017, NAT MED, V23, P174, DOI 10.1038/nm.4267

Fuxe K, 2005, J MOL NEUROSCI, V26, P209, DOI 10.1385/JMN:26:2-3:209

George J, 2015, GLIA, V63, P1636, DOI 10.1002/glia.22833

Gomes C, 2013, J NEUROINFLAMM, V10, DOI 10.1186/1742-2094-10-16

Gyoneva S, 2014, GLIA, V62, P1345, DOI 10.1002/glia.22686

Gyoneva S, 2014, NEUROBIOL DIS, V67, P191, DOI 10.1016/j.nbd.2014.03.004

Haskó G, 2005, TRENDS PHARMACOL SCI, V26, P511, DOI 10.1016/j.tips.2005.08.004

Hauser RA, 2014, LANCET NEUROL, V13, P767, DOI 10.1016/S1474-4422(14)70148-6

Hauser RA, 2011, LANCET NEUROL, V10, P221, DOI 10.1016/S1474-4422(11)70012-6

Hong S, 2016, SCIENCE, V352, P712, DOI 10.1126/science.aad8373

Hu QD, 2016, EXP NEUROL, V283, P213, DOI 10.1016/j.expneurol.2016.05.040

Huck JHJ, 2015, J CEREBR BLOOD F MET, V35, P1804, DOI 10.1038/jcbfm.2015.128

Ikeda K, 2002, J NEUROCHEM, V80, P262, DOI 10.1046/j.0022-3042.2001.00694.x

Imura Y, 2013, GLIA, V61, P1320, DOI 10.1002/glia.22517

Ingwersen J, 2016, J NEUROINFLAMM, V13, DOI 10.1186/s12974-016-0512-z

Jackson-Lewis V, 2007, NAT PROTOC, V2, P141, DOI 10.1038/nprot.2006.342

Jakowec MW, 2004, COMPARATIVE MED, V54, P497

JAMES S, 1993, J NEUROCHEM, V60, P219, DOI 10.1111/j.1471-4159.1993.tb05841.x

Joe EH, 2018, EXP NEUROBIOL, V27, P77, DOI 10.5607/en.2018.27.2.77

Joers V, 2017, PROG NEUROBIOL, V155, P57, DOI 10.1016/j.pneurobio.2016.04.006

Joshi N, 2018, J NEUROSCI RES, V96, P379, DOI 10.1002/jnr.24185

Jucaite A, 2015, BRAIN, V138, P2687, DOI 10.1093/brain/awv184

Kaur K, 2017, J NEUROL SCI, V381, P308, DOI 10.1016/j.jns.2017.08.3251

Kim DS, 2008, FRONT BIOSCI-LANDMRK, V13, P2311, DOI 10.2741/2845

Kim YS, 2006, EXP MOL MED, V38, P333, DOI 10.1038/emm.2006.40

Koizumi S, 2013, GLIA, V61, P47, DOI 10.1002/glia.22358

Kondo T, 2015, CLIN NEUROPHARMACOL, V38, P41, DOI 10.1097/WNF.0000000000000073

Koshimori Y, 2015, PLOS ONE, V10, DOI 10.1371/journal.pone.0138721

Kulesskaya N, 2013, PLOS ONE, V8, DOI 10.1371/journal.pone.0066896

Laurent C, 2016, MOL PSYCHIATR, V21, P149, DOI 10.1038/mp.2015.115

Li QY, 2018, NAT REV IMMUNOL, V18, P225, DOI 10.1038/nri.2017.125

Livak KJ, 2001, METHODS, V25, P402, DOI 10.1006/meth.2001.1262

Lopes LV, 2011, CURR TOP MED CHEM, V11, P1087

Luongo L, 2014, GLIA, V62, P122, DOI 10.1002/glia.22592

Madeira JM, 2014, J NEUROIMMUNOL, V276, P71, DOI 10.1016/j.jneuroim.2014.08.615

Madeira MH, 2017, MEDIAT INFLAMM, V2017, DOI 10.1155/2017/4761081

Matyash M, 2017, PLOS ONE, V12, DOI 10.1371/journal.pone.0175012

MEGHJI P, 1989, J NEUROCHEM, V53, P1852, DOI 10.1111/j.1471-4159.1989.tb09252.x

Melani A, 2012, EXP NEUROL, V233, P193, DOI 10.1016/j.expneurol.2011.09.036

Merighi S, 2015, GLIA, V63, P1933, DOI 10.1002/glia.22861

Mills JH, 2012, J IMMUNOL, V188, P5713, DOI 10.4049/jimmunol.1200545

Mohamed RA, 2016, NEUROSCIENCE, V314, P145, DOI 10.1016/j.neuroscience.2015.11.059

Morissette M, 2006, SYNAPSE, V60, P239, DOI 10.1002/syn.20295

Newell EA, 2015, BRAIN RES, V1594, P27, DOI 10.1016/j.brainres.2014.10.059

Ohnishi A, 2016, NUCL MED BIOL, V43, P438, DOI 10.1016/j.nucmedbio.2016.04.005

Orr AG, 2009, NAT NEUROSCI, V12, P872, DOI 10.1038/nn.2341

Ouyang XS, 2013, NAT COMMUN, V4, DOI 10.1038/ncomms3909

Pani AK, 2014, PLOS ONE, V9, DOI 10.1371/journal.pone.0092422

Parkinson FE, 2005, NEUROL RES, V27, P153, DOI 10.1179/016164105X21878

Pedata F, 2014, MEDIAT INFLAMM, V2014, DOI 10.1155/2014/805198

Ramlackhansingh AF, 2011, NEUROLOGY, V76, P1811, DOI 10.1212/WNL.0b013e31821ccce4

Rebola N, 2011, J NEUROCHEM, V117, P100, DOI 10.1111/j.1471-4159.2011.07178.x

Rodrigues RJ, 2015, FRONT NEUROSCI-SWITZ, V9, DOI 10.3389/fnins.2015.00148

Salter MW, 2017, NAT MED, V23, P1018, DOI 10.1038/nm.4397

Santiago AR, 2014, MEDIAT INFLAMM, V2014, DOI 10.1155/2014/465694

Schiffmann SN, 2007, PROG NEUROBIOL, V83, P277, DOI 10.1016/j.pneurobio.2007.05.001

SCHOEN SW, 1992, GLIA, V6, P314, DOI 10.1002/glia.440060410

Sitkovsky MV, 2004, ANNU REV IMMUNOL, V22, P657, DOI 10.1146/annurev.immunol.22.012703.104731

Svenningsson P, 1999, PROG NEUROBIOL, V59, P355, DOI 10.1016/S0301-0082(99)00011-8

Torres-Rosas R, 2014, NAT MED, V20, P291, DOI 10.1038/nm.3479

Tristao FSM, 2014, NEUROTOX RES, V25, P24, DOI 10.1007/s12640-013-9401-8

van der Putten C, 2009, J IMMUNOL, V182, P7603, DOI 10.4049/jimmunol.0803383

Villar-Menéndez I, 2014, NEUROBIOL DIS, V69, P206, DOI 10.1016/j.nbd.2014.05.030

Wall MJ, 2013, J PHYSIOL-LONDON, V591, P3853, DOI 10.1113/jphysiol.2013.253450

Wang YM, 2015, CELL, V160, P1061, DOI 10.1016/j.cell.2015.01.049

Wei CJ, 2011, LEARN MEMORY, V18, P459, DOI 10.1101/lm.2136011

Wei CJ, 2011, BBA-BIOMEMBRANES, V1808, P1358, DOI 10.1016/j.bbamem.2010.12.018

Wills AMA, 2013, MOVEMENT DISORD, V28, P380, DOI 10.1002/mds.25319

Wu HJ, 2014, NAT PROTOC, V9, P491, DOI 10.1038/nprot.2014.015

Xu K, 2002, NEUROSCI LETT, V322, P13, DOI 10.1016/S0304-3940(02)00069-1

Yao SQ, 2012, J NEUROCHEM, V123, P100, DOI 10.1111/j.1471-4159.2012.07807.x

Yu LQ, 2008, ANN NEUROL, V63, P338, DOI 10.1002/ana.21313

Yun SP, 2018, NAT MED, V24, P931, DOI 10.1038/s41591-018-0051-5

Zhang B, 2018, CEREB CORTEX, V28, P3457, DOI 10.1093/cercor/bhx212

Zimmermann Herbert, 2006, Novartis Found Symp, V276, P113

Zimmermann H, 2012, PURINERG SIGNAL, V8, P437, DOI 10.1007/s11302-012-9309-4

Zylka MJ, 2008, NEURON, V60, P111, DOI 10.1016/j.neuron.2008.08.024

NR 102

TC 64

Z9 70

U1 1

U2 58

PU OXFORD UNIV PRESS

PI OXFORD

PA GREAT CLARENDON ST, OXFORD OX2 6DP, United Kingdom

SN 0006-8950

EI 1460-2156

J9 BRAIN

JI Brain

PD MAR

PY 2019

VL 142

BP 700

EP 718

DI 10.1093/brain/awy351

PN 3

PG 19

WC Clinical Neurology; Neurosciences

WE Science Citation Index Expanded (SCI-EXPANDED)

SC Neurosciences & Neurology

GA KK4PB

UT WOS:000512724900024

PM 30689733

OA Bronze

DA 2024-08-12

ER

PT J

AU Tsagkaris, C

Bilal, M

Aktar, I

Aboufandi, Y

Tas, A

Aborode, AT

Suvvari, TK

Ahmad, S

Shkodina, A

Phadke, R

Emhamed, MS

Baig, AA

Alexiou, A

Ashraf, GM

Kamal, MA

AF Tsagkaris, Christos

Bilal, Muhammad

Aktar, Irem

Aboufandi, Youssef

Tas, Ahmet

Aborode, Abdullahi Tunde

Suvvari, Tarun Kumar

Ahmad, Shoaib

Shkodina, Anastasiia

Phadke, Rachana

Emhamed, Marwa S.

Baig, Atif Amin

Alexiou, Athanasios

Ashraf, Ghulam Md.

Kamal, Mohammad Amjad

TI Cytokine Storm and Neuropathological Alterations in Patients with

Neurological Manifestations of COVID-19

SO CURRENT ALZHEIMER RESEARCH

LA English

DT Review

DE Cytokine storm; SARS-Coronavirus-2 infection; neuropathological;

neuroinflammation; neutrophil extracellular traps; ARDS

ID CEREBROSPINAL-FLUID; parkinson's disease; CORONAVIRUS; SARS-COV-2;

INFLAMMATION; INFECTION; PROTEIN; ACE2; RECOGNITION; EXPRESSION

AB The COVID-19 pandemic is caused by the severe acute respiratory syndrome coronavirus (SARS-CoV-2), a respiratory pathogen with neuroinvasive potential. Neurological COVID-19 manifestations include loss of smell and taste, headache, dizziness, stroke, and potentially fatal encephalitis. Several studies found elevated proinflammatory cytokines, such as TNF-alpha, IFN-gamma, IL-6 IL-8, IL-10 IL-16, IL-17A, and IL-18 in severely and critically ill COVID-19 patients may persist even after apparent recovery from infection. Biomarker studies on CSF and plasma and serum from COVID-19 patients have also shown a high level of IL-6, intrathecal IgG, neurofilament light chain (NFL), glial fibrillary acidic protein (GFAP), and tau protein. Emerging evidence on the matter has established the concept of COVID-19-associated neuroinflammation, in the context of COVID-19-associated cytokine storm. While the short-term implications of this condition are extensively documented, its long-term implications are yet to be understood. The association of the aforementioned cytokines with the pathogenesis of neurodegenerative disease, including Alzheimer's disease, Parkinson's disease, Huntington's disease, and amyotrophic lateral sclerosis, may increase COVID-19 patients' risk of developing neurodegenerative disease. Analysis of proinflammatory cytokines and CSF biomarkers in patients with COVID-19 can contribute to the early detection of the disease's exacerbation, monitoring the neurological implications of the disease and devising risk scales, and identifying treatment targets.

C1 [Tsagkaris, Christos] Univ Crete, Fac Med, Iraklion, Greece.

[Bilal, Muhammad] Liaquat Univ Med & Hlth Sci, Coll Pharm, Jamshoro, Pakistan.

[Aktar, Irem; Tas, Ahmet] Istanbul Univ, Istanbul Fac Med, Istanbul, Turkey.

[Aboufandi, Youssef] Med Univ Sofia, Fac Med, Sofia, Bulgaria.

[Aborode, Abdullahi Tunde] Mississipi State Univ, Dept Chem, Starkville, MS USA.

[Suvvari, Tarun Kumar] Dr NTR Univ Hlth Sci, Fac Med, Vijayawada, India.

[Ahmad, Shoaib] Punjab Med Coll, Dept Med Sci, Faisalabad, Pakistan.

[Ahmad, Shoaib] Faisalabad Med Univ, Faisalabad, Pakistan.

[Shkodina, Anastasiia] Poltava State Med Univ, Dept Neurol Dis Neurosurg & Med Genet, Poltava, Ukraine.

[Phadke, Rachana] Indira Gandhi Govt Med Coll, Sch Med, Nagpur, Maharashtra, India.

[Emhamed, Marwa S.] Univ Tripoli, Fac Med, Tripoli, Libya.

[Baig, Atif Amin] Sultan Zainal Abidin Univ, Fac Med, Kuala Terengganu, Malaysia.

[Alexiou, Athanasios] Novel Global Community Educ Fdn, Dept Sci & Engn, Hebersham, NSW 2770, Australia.

[Alexiou, Athanasios] AFNP Med Austria, A-1010 Vienna, Austria.

[Ashraf, Ghulam Md.] Univ Sharjah, Coll Hlth Sci, Dept Med Lab Sci, Sharjah 27272, U Arab Emirates.

[Kamal, Mohammad Amjad] Sichuan Univ, West China Hosp, Frontiers Sci Ctr DiseaseRelated Mol Network, West China Sch Nursing,Inst Syst Genet, Chengdu 610041, Sichuan, China.

[Kamal, Mohammad Amjad] King Abdulaziz Univ, King Fahd Med Res Ctr, Jeddah 21589, Saudi Arabia.

[Kamal, Mohammad Amjad] Novel Global Community Educ Fdn, 7 Peterlee Pl, Hebersham, NSW, Australia.

C3 University of Crete; Liaquat University of Medical & Health Sciences;

Istanbul University; Medical University Sofia; Mississippi State

University; Punjab Medical College; Poltava State Medical University;

University of Tripoli; Universiti Sultan Zainal Abidin; University of

Sharjah; Sichuan University; King Abdulaziz University

RP Tsagkaris, C (corresponding author), Univ Crete, Fac Med, Iraklion, Greece.

EM chriss20x@gmail.com

RI Baig, Atif/AAY-3558-2021; Shkodina, Anastasiia/GXU-0271-2022; Ahmad,

Shoaib/AHD-5244-2022; Tsagkaris, Christos/Y-5951-2019; Bilal,

Muhammad/AAH-6461-2020; Suvvari, Tarun Kumar/AAN-5135-2021; Emhemed,

Marwa SaedAli/AAI-4521-2021; Kamal, Mohammad Amjad/J-2918-2014;

Abdullahi, Aborode/AAL-6793-2021; Ashraf, Ghulam Md/H-9485-2012;

Alexiou, Athanasios/AAT-8491-2021

OI Shkodina, Anastasiia/0000-0002-7198-5498; Ahmad,

Shoaib/0000-0002-7241-7724; Tsagkaris, Christos/0000-0002-4250-574X;

Bilal, Muhammad/0000-0001-5388-3183; Suvvari, Tarun

Kumar/0000-0003-0063-0339; Emhemed, Marwa SaedAli/0000-0003-2522-3254;

Kamal, Mohammad Amjad/0000-0003-0088-0565; Abdullahi,

Aborode/0000-0003-2516-5920; Ashraf, Ghulam Md/0000-0002-9820-2078;

Alexiou, Athanasios/0000-0002-2206-7236; Tas, Ahmet/0000-0002-1944-2576

CR Aarli JA, 2003, CURR MED CHEM, V10, P1931, DOI 10.2174/0929867033456918

Akira S, 2006, CELL, V124, P783, DOI 10.1016/j.cell.2006.02.015

Alexopoulou L, 2001, NATURE, V413, P732, DOI 10.1038/35099560

Allsopp K, 2019, PSYCHIAT RES, V279, P15, DOI 10.1016/j.psychres.2019.07.005

Amor S, 2010, IMMUNOLOGY, V129, P154, DOI 10.1111/j.1365-2567.2009.03225.x

Auyeung TW, 2005, J INFECTION, V51, P98, DOI 10.1016/j.jinf.2004.09.008

Bai T., 2020, SSRN ELECT J, DOI [10.2139/ssrn.3546118, DOI 10.2139/SSRN.3546118]

Baig AM, 2020, ACS CHEM NEUROSCI, V11, P1204, DOI 10.1021/acschemneuro.0c00181

Baig AM, 2020, ACS CHEM NEUROSCI, V11, P995, DOI 10.1021/acschemneuro.0c00122

Bansal M, 2020, DIAB MET SYND CLIN R, V14, P247, DOI 10.1016/j.dsx.2020.03.013

Belvis R, 2020, HEADACHE, V60, P1422, DOI 10.1111/head.13841

Bernstein S, 2020, NEED NEUROLOGICAL DI

Bialuk I, 2018, NEUROBIOL LEARN MEM, V155, P21, DOI 10.1016/j.nlm.2018.06.006

Bianchetti A, 2020, J NUTR HEALTH AGING, V24, P560, DOI 10.1007/s12603-020-1389-1

Boiko DI, 2022, ENVIRON SCI POLLUT R, V29, P28062, DOI 10.1007/s11356-021-18384-4

Brundin P, 2020, TRENDS NEUROSCI, V43, P931, DOI 10.1016/j.tins.2020.10.009

Buzhdygan TP, 2020, NEUROBIOL DIS, V146, DOI [10.1016/j.nbd.2020.105131, 10.1101/2020.06.15.150912]

Cao LY, 2021, PHARMACOL RES, V163, DOI 10.1016/j.phrs.2020.105297

Caterino M, 2021, SCI REP-UK, V11, DOI 10.1038/s41598-021-82426-7

Chen G, 2020, J CLIN INVEST, V130, P2620, DOI 10.1172/JCI137244

Chen MF, 2020, EUR RESPIR J, V56, DOI 10.1183/13993003.01948-2020

Chen NS, 2020, LANCET, V395, P507, DOI 10.1016/S0140-6736(20)30211-7

Chen RR, 2021, FRONT NEUROL, V11, DOI 10.3389/fneur.2020.573095

Chen XH, 2020, CLIN INFECT DIS, V71, P1937, DOI 10.1093/cid/ciaa449

Ferrari CC, 2006, NEUROBIOL DIS, V24, P183, DOI 10.1016/j.nbd.2006.06.013

Cohen ME, 2020, LANCET NEUROL, V19, P804, DOI 10.1016/S1474-4422(20)30305-7

Cojocaru Inimioara Mihaela, 2011, Rom J Intern Med, V49, P55

Crouse JJ, 2016, REV NEUROSCIENCE, V27, P549, DOI 10.1515/revneuro-2016-0002

Crunfli F, PREPRINT, DOI [10.21203/rs.3.rs-104944/v1, DOI 10.21203/RS.3.RS-104944/V1]

Daou BJ, 2020, NEUROSURGERY, V87, pE234, DOI 10.1093/neuros/nyaa186

Davatzikos C, 2019, NEUROIMAGE, V197, P652, DOI 10.1016/j.neuroimage.2018.10.003

DeKosky ST, 2021, J NEUROTRAUM, V38, P1, DOI 10.1089/neu.2020.7332

Diao B, 2020, FRONT IMMUNOL, V11, DOI 10.3389/fimmu.2020.00827

Dijkman R, 2013, J VIROL, V87, P6081, DOI 10.1128/JVI.03368-12

Divani AA, 2020, J STROKE CEREBROVASC, V29, DOI 10.1016/j.jstrokecerebrovasdis.2020.104941

Doobay MF, 2007, AM J PHYSIOL-REG I, V292, pR373, DOI 10.1152/ajpregu.00292.2006

Earhart AP, 2020, NEW MICROB NEW INFEC, V35, DOI 10.1016/j.nmni.2020.100689

Edén A, 2021, NEUROLOGY, V96, pE294, DOI 10.1212/WNL.0000000000010977

Espinoza JA, 2013, P NATL ACAD SCI USA, V110, P9112, DOI 10.1073/pnas.1217508110

Esposito G, 2020, BRAIN BEHAV IMMUN, V87, P93, DOI 10.1016/j.bbi.2020.04.060

Faber I, 2020, MOVEMENT DISORD, V35, P1721, DOI 10.1002/mds.28277

Feng YM, 2012, PLOS ONE, V7, DOI 10.1371/journal.pone.0048910

Fierce Biotech, 2021, GSK PUSHING MIDPHASE

Filatov A, 2020, CUREUS J MED SCIENCE, V12, DOI 10.7759/cureus.7352

Fisicaro F, 2021, INT J ENV RES PUB HE, V18, DOI 10.3390/ijerph18041415

Franchini M, 2020, DIAGNOSIS, V7, P357, DOI 10.1515/dx-2020-0078

Frontera JA, 2022, ALZHEIMERS DEMENT, V18, P899, DOI 10.1002/alz.12556

Galimberti D, 2008, J NEUROL, V255, P539, DOI 10.1007/s00415-008-0737-6

Gattinoni L, 2020, AM J RESP CRIT CARE, V201, P1299, DOI 10.1164/rccm.202003-0817LE

Glushakova Olena Y, 2016, Brain Circ, V2, P28, DOI 10.4103/2394-8108.178546

Goldberg MF, 2020, AM J NEURORADIOL, V41, P1170, DOI 10.3174/ajnr.A6588

Hall S, 2018, SCI REP-UK, V8, DOI 10.1038/s41598-018-31517-z

Hamming I, 2004, J PATHOL, V203, P631, DOI 10.1002/path.1570

Hasegawa S, 2011, CYTOKINE, V54, P167, DOI 10.1016/j.cyto.2011.01.006

Helms J, 2020, INTENS CARE MED, V46, P1089, DOI 10.1007/s00134-020-06062-x

Helms J, 2020, NEW ENGL J MED, V382, P2268, DOI 10.1056/NEJMc2008597

Hermine O, 2022, EUR RESPIR J, V60, DOI 10.1183/13993003.02523-2021

Hess DC, 2020, TRANSL STROKE RES, V11, P322, DOI 10.1007/s12975-020-00818-9

Hoffmann M, 2020, CELL, V181, P271, DOI 10.1016/j.cell.2020.02.052

Hoffmann M, 2020, MOL CELL, V78, P779, DOI 10.1016/j.molcel.2020.04.022

Hojyo S, 2020, INFLAMM REGEN, V40, DOI 10.1186/s41232-020-00146-3

Horby P, 2021, NEW ENGL J MED, V384, P693, DOI [10.1056/NEJMoa2021436, 10.1056/NEJMoa2022926]

Hosking MP, 2010, CRIT REV IMMUNOL, V30, P119, DOI 10.1615/CritRevImmunol.v30.i2.20

Huang CL, 2020, LANCET, V395, P497, DOI [10.1016/S0140-6736(20)30183-5, 10.1016/S0140-6736(20)30211-7]

Huang YH, 2020, BRAIN BEHAV IMMUN, V87, P149, DOI 10.1016/j.bbi.2020.05.012

Iba T, 2020, CRIT CARE, V24, DOI 10.1186/s13054-020-03077-0

Ichiyama T, 2003, PEDIATR INT, V45, P734, DOI 10.1111/j.1442-200X.2003.01822.x

Jakhmola Shweta, 2020, SN Compr Clin Med, V2, P2137, DOI 10.1007/s42399-020-00522-7

Jarrahi A, 2020, J NEUROINFLAMM, V17, DOI 10.1186/s12974-020-01957-4

Jasti M, 2021, J NEUROL, V268, P2007, DOI 10.1007/s00415-020-09950-w

Kaidashev I, 2021, HELIYON, V7, DOI 10.1016/j.heliyon.2021.e07863

Kaplan E, 2020, FIERCE HEALTHCARE

Khatoon F, 2020, J NEUROVIROL, V26, P619, DOI 10.1007/s13365-020-00895-4

Kimura En, 2008, J Med Case Rep, V2, P220, DOI 10.1186/1752-1947-2-220

Klein RS, 2019, ANNU REV IMMUNOL, V37, P73, DOI 10.1146/annurev-immunol-042718-041417

Koenigsknecht-Talboo J, 2005, J NEUROSCI, V25, P8240, DOI 10.1523/JNEUROSCI.1808-05.2005

Levi M, 2010, CRIT CARE MED, V38, pS26, DOI 10.1097/CCM.0b013e3181c98d21

Li ZQ, 2020, FRONT MED-PRC, V14, P533, DOI 10.1007/s11684-020-0786-5

Lippi G, 2020, CLIN CHIM ACTA, V506, P145, DOI 10.1016/j.cca.2020.03.022

Liu YW, 2020, J INFECTION, V81, pE6, DOI 10.1016/j.jinf.2020.04.002

Lochhead JJ, 2012, ADV DRUG DELIVER REV, V64, P614, DOI 10.1016/j.addr.2011.11.002

Mahajan RK, 2020, J ANAESTH CLIN PHARM, V36, P435, DOI 10.4103/joacp.JOACP_359_20

Mahammedi A, 2020, RADIOLOGY, V297, pE270, DOI 10.1148/radiol.2020201933

Mao L, 2020, JAMA NEUROL, V77, P683, DOI 10.1001/jamaneurol.2020.1127

Mehta P, 2020, LANCET, V395, P1033, DOI 10.1016/S0140-6736(20)30628-0

Meinhardt J, 2021, NAT NEUROSCI, V24, P168, DOI 10.1038/s41593-020-00758-5

Méndez-Guerrero A, 2020, NEUROLOGY, V95, pE2109, DOI 10.1212/WNL.0000000000010282

Miesbach W, 2020, CLIN APPL THROMB-HEM, V26, DOI 10.1177/1076029620938149

Moghimi M., 2021, ACTA MED IRAN, V59, P747, DOI [10.21203/rs.3.rs-33179/v1, DOI 10.21203/RS.3.RS-33179/V1]

Moore AH, 2009, NEUROSCIENCE, V164, P1484, DOI 10.1016/j.neuroscience.2009.08.073

Mori I, 2005, J NEUROVIROL, V11, P129, DOI 10.1080/13550280590922793

Moriguchi T, 2020, INT J INFECT DIS, V94, P55, DOI 10.1016/j.ijid.2020.03.062

Mrak RE, 2000, J NEUROPATH EXP NEUR, V59, P471, DOI 10.1093/jnen/59.6.471

Mukandala G, 2016, BRAIN SCI, V6, DOI 10.3390/brainsci6010006

Murphy O, 2018, NEUROLOGY, V90

Nagafuchi M, 2006, INTERNAL MED, V45, P1209, DOI 10.2169/internalmedicine.45.1769

Nagant C, 2020, INT J INFECT DIS, V101, P342, DOI 10.1016/j.ijid.2020.10.003

Nagatsu T, 2005, CURR PHARM DESIGN, V11, P999, DOI 10.2174/1381612053381620

Najjar S, 2020, J NEUROINFLAMM, V17, DOI 10.1186/s12974-020-01896-0

Nara A, 2015, AM J FOREN MED PATH, V36, P3, DOI 10.1097/PAF.0000000000000129

Ngoi SM, 2008, J IMMUNOL, V181, P7670, DOI 10.4049/jimmunol.181.11.7670

Novi G, 2020, MULT SCLER RELAT DIS, V42, DOI 10.1016/j.msard.2020.102120

O'Brien RJ, 2011, ANNU REV NEUROSCI, V34, P185, DOI 10.1146/annurev-neuro-061010-113613

Paidi RK, 2021, J NEUROIMMUNE PHARM, V16, P59, DOI 10.1007/s11481-020-09979-8

Paniz-Mondolfi A, 2020, J MED VIROL, V92, P699, DOI 10.1002/jmv.25915

Parsons T, 2020, J NEUROL, V267, P2799, DOI 10.1007/s00415-020-09951-9

Poillon G, 2021, J NEURORADIOLOGY, V48, P121, DOI 10.1016/j.neurad.2020.05.003

Prieto GA, 2019, ACS CHEM NEUROSCI, V10, P1197, DOI 10.1021/acschemneuro.8b00511

Ragab D, 2020, FRONT IMMUNOL, V11, DOI 10.3389/fimmu.2020.01446

Romano M, 2020, CELLS-BASEL, V9, DOI 10.3390/cells9051267

Romero-Sánchez CM, 2020, NEUROLOGY, V95, pE1060, DOI 10.1212/WNL.0000000000009937

Ruan QR, 2020, INTENS CARE MED, V46, P846, DOI 10.1007/s00134-020-05991-x

Savchenko L, 2018, INT J CHRONIC OBSTR, V13, P2569, DOI 10.2147/COPD.S164833

Scalzo P, 2010, NEUROSCI LETT, V468, P56, DOI 10.1016/j.neulet.2009.10.062

Shah VK, 2020, FRONT IMMUNOL, V11, DOI 10.3389/fimmu.2020.01949

Sharif-Askari NS, 2020, MOL THER METH CLIN D, V18, P1, DOI 10.1016/j.omtm.2020.05.013

Shkodina AD, 2022, NEUROSCI LETT, V781, DOI 10.1016/j.neulet.2022.136675

Sivapalasingam S, 2022, CLIN INFECT DIS, V75, pE380, DOI 10.1093/cid/ciac153

Song ER, 2021, J EXP MED, V218, DOI 10.1084/jem.20202135

Sonja A., 2020, ANN ONCOL, V2020, P19

Sulzer D, 2020, NPJ PARKINSONS DIS, V6, DOI 10.1038/s41531-020-00123-0

Tancheva L, 2020, BRAIN SCI, V10, DOI 10.3390/brainsci10110852

Tang N, 2020, J THROMB HAEMOST, V18, P1094, DOI 10.1111/jth.14817

Tang YJ, 2020, FRONT IMMUNOL, V11, DOI 10.3389/fimmu.2020.01708

Tarawneh R, 2012, CSH PERSPECT MED, V2, DOI 10.1101/cshperspect.a006148

Tarianyk K., 2021, Georgian Med News, V320, P100

Tjalkens Ronald B, 2017, Adv Neurobiol, V18, P159, DOI 10.1007/978-3-319-60189-2_8

Tsutsumi H, 2011, ADV OTO-RHINO-LARYNG, V72, P153, DOI 10.1159/000324777

Vaibhav K, 2020, SCI ADV, V6, DOI 10.1126/sciadv.aax8847

Virhammar J, 2021, EUR J NEUROL, V28, P3324, DOI 10.1111/ene.14703

Volpicelli-Daley LA, 2014, MOL BIOL CELL, V25, P4010, DOI 10.1091/mbc.E14-02-0741

Wang WY, 2015, ANN TRANSL MED, V3, DOI 10.3978/j.issn.2305-5839.2015.03.49

Whitelaw S, 2020, LANCET DIGIT HEALTH, V2, pE435, DOI 10.1016/S2589-7500(20)30142-4

Xia HJ, 2008, J NEUROCHEM, V107, P1482, DOI 10.1111/j.1471-4159.2008.05723.x

Xiao L, 2013, AM J PHYSIOL-CELL PH, V304, pC1073, DOI 10.1152/ajpcell.00364.2012

Xu JX, 2022, CELL MOL NEUROBIOL, V42, P305, DOI 10.1007/s10571-020-00915-1

Xu Z, 2020, LANCET RESP MED, V8, P420, DOI 10.1016/S2213-2600(20)30076-X

Yan RH, 2020, SCIENCE, V367, P1444, DOI 10.1126/science.abb2762

Yang Y, 2020, J ALLERGY CLIN IMMUN, V146, P119, DOI 10.1016/j.jaci.2020.04.027

ZANDER R, 1990, SCAND J CLIN LAB INV, V50, P187, DOI 10.3109/00365519009087509

Zhai XF, 2020, J VIROL, V94, DOI 10.1128/JVI.00831-20

Zhang JC, 2020, BRAIN BEHAV IMMUN, V87, P59, DOI 10.1016/j.bbi.2020.04.046

Zhang J, 2020, J TRANSL MED, V18, DOI 10.1186/s12967-020-02571-x

Zhang JM, 2007, INT ANESTHESIOL CLIN, V45, P27, DOI 10.1097/AIA.0b013e318034194e

Zhang Luoping, 2020, Adv Biomark Sci Technol, V2, P1, DOI 10.1016/j.abst.2020.08.001

Zhao JC, 2012, J VIROL, V86, P11416, DOI 10.1128/JVI.01410-12

Zhou L, 2013, VIROL J, V10, DOI 10.1186/1743-422X-10-172

Zhuang YP, 2021, WORLD J GASTROENTERO, V27, P4248, DOI 10.3748/wjg.v27.i26.4248

NR 148

TC 14

Z9 14

U1 3

U2 9

PU BENTHAM SCIENCE PUBL LTD

PI SHARJAH

PA EXECUTIVE STE Y-2, PO BOX 7917, SAIF ZONE, 1200 BR SHARJAH, U ARAB

EMIRATES

SN 1567-2050

EI 1875-5828

J9 CURR ALZHEIMER RES

JI Curr. Alzheimer Res.

PY 2022

VL 19

IS 9

BP 641

EP 657

DI 10.2174/1567205019666220908084559

PG 17

WC Clinical Neurology; Neurosciences

WE Science Citation Index Expanded (SCI-EXPANDED)

SC Neurosciences & Neurology

GA 8H7VG

UT WOS:000921237700002

PM 36089786

DA 2024-08-12

ER

PT J

AU Olsen, LK

Cairns, AG

Ådén, J

Moriarty, N

Cabre, S

Alamilla, VR

Almqvist, F

Dowd, E

McKernan, DP

AF Olsen, Laura K.

Cairns, Andrew G.

Aden, Jorgen

Moriarty, Niamh

Cabre, Silvia

Alamilla, Veronica R.

Almqvist, Fredrik

Dowd, Eilis

McKernan, Declan P.

TI Viral mimetic priming enhances α-synuclein-induced degeneration:

Implications for Parkinson's disease

SO BRAIN BEHAVIOR AND IMMUNITY

LA English

DT Article

DE Parkinson's disease; Viral infection; Neuroinflammation;

alpha-Synuclein; Neurodegeneration

ID CENTRAL-NERVOUS-SYSTEM; TOLL-LIKE RECEPTOR-3; NF-KAPPA-B;

HERPES-SIMPLEX; RAT MODELS; MOVEMENT-DISORDERS; VIRUS-ANTIBODIES;

ACTIVATION; AUTOPHAGY; INFECTION

AB Evidence is accumulating to suggest that viral infections and consequent viral-mediated neuroinflammation may contribute to the etiology of idiopathic Parkinson's disease. Moreover, viruses have been shown to influence asynuclein oligomerization as well as the autophagic clearance of abnormal intra-cellular proteins aggregations, both of which are key neuropathological events in Parkinson's disease pathogenesis. To further investigate the interaction between viral-mediated neuroinflammation and alpha-synuclein aggregation in the context of Parkinson's disease, this study sought to determine the impact of viral neuroinflammatory priming on alpha-synuclein aggregate-induced neuroinflammation and neurotoxicity in the rat nigrostriatal pathway. To do so, male Sprague-Dawley rats were intra-nigrally injected with a synthetic mimetic of viral dsRNA (poly I:C) followed two weeks later by a peptidomimetic small molecule which accelerates alpha-synuclein fibril formation (FN075). The impact of the viral priming on alpha-synuclein aggregation-induced neuroinflammation, neurodegeneration and motor dysfunction was assessed. We found that prior administration of the viral mimetic poly I:C significantly exacerbated or precipitated the alpha-synuclein aggregate induced neuropathological and behavioral effects. Specifically, sequential exposure to the two challenges caused a significant increase in nigral microgliosis (p < 0.001) and astrocytosis (p < 0.01); precipitated a significant degeneration of the nigrostriatal cell bodies (p < 0.05); and precipitated a significant impairment in forelimb kinesis (p < 0.01) and sensorimotor integration (p < 0.01). The enhanced sensitivity of the nigrostriatal neurons to pathological alpha-synuclein aggregation after viral neuroinflammatory priming further suggests that viral infections may contribute to the etiology and pathogenesis of Parkinson's disease.

C1 [Olsen, Laura K.; Moriarty, Niamh; Cabre, Silvia; Alamilla, Veronica R.; Dowd, Eilis; McKernan, Declan P.] Natl Univ Ireland, Sch Med, Pharmacol & Therapeut, Galway, Ireland.

[Cairns, Andrew G.; Aden, Jorgen; Almqvist, Fredrik] Umea Univ, Dept Chem, Umea, Sweden.

C3 Ollscoil na Gaillimhe-University of Galway; Umea University

RP McKernan, DP (corresponding author), Natl Univ Ireland, Sch Med, Pharmacol & Therapeut, Galway, Ireland.

EM declan.mckernan@nuigalway.ie

RI McKernan, Declan P/AEK-0857-2022; Olsen, Laura/KLY-8927-2024

OI McKernan, Declan P/0000-0002-9691-1671; Cabre,

Silvia/0000-0002-6896-7301; Olsen, Laura/0000-0002-3577-9769; Almqvist,

Fredrik/0000-0002-0504-4446; Moriarty, Niamh/0000-0001-5321-465X

FU College of Medicine, Nursing and Health Science at National University

of Ireland, Galway; Swedish Research Council; Knut and Alice Wallenberg

Foundation; Goran Gustafsson Foundation; Swedish Foundation for

Strategic Research

FX This work was funded by a Postgraduate Scholarship to Laura Olsen from

the College of Medicine, Nursing and Health Science at National

University of Ireland, Galway. The authors also acknowledge funding from

the Swedish Research Council, the Knut and Alice Wallenberg Foundation,

the Goran Gustafsson Foundation and the Swedish Foundation for Strategic

Research.

CR Alexopoulou L, 2001, NATURE, V413, P732, DOI 10.1038/35099560

Barkholt P, 2012, NEUROSCIENCE, V208, P85, DOI 10.1016/j.neuroscience.2012.02.004

BENMOHAMED L, 2016, J IMMUNOL S1, V196

Bobyn J, 2012, J NEUROINFLAMM, V9, DOI 10.1186/1742-2094-9-86

Bu XL, 2015, EUR J NEUROL, V22, P1519, DOI 10.1111/ene.12477

Cameron JS, 2007, J NEUROSCI, V27, P13033, DOI 10.1523/JNEUROSCI.4290-06.2007

Carpentier PA, 2005, GLIA, V49, P360, DOI 10.1002/glia.20117

Cegelski L, 2009, NAT CHEM BIOL, V5, P913, DOI 10.1038/nchembio.242

Chermenina M, 2015, NPJ PARKINSON DIS, V1, DOI 10.1038/npjparkd.2015.24

Clayton KB, 2007, NEUROSCI LETT, V427, P132, DOI 10.1016/j.neulet.2007.09.025

Concannon RM, 2016, EXP NEUROL, V283, P204, DOI 10.1016/j.expneurol.2016.06.014

Daniele SG, 2015, SCI SIGNAL, V8, DOI 10.1126/scisignal.2005965

de Jong MD, 2005, NEW ENGL J MED, V352, P686, DOI 10.1056/NEJMoa044307

de Mattos JP, 2002, ARQ NEURO-PSIQUIAT, V60, P525, DOI 10.1590/S0004-282X2002000400002

Deleidi M, 2012, SCI TRANSL MED, V4, DOI 10.1126/scitranslmed.3003492

Deleidi M, 2010, J NEUROSCI, V30, P16091, DOI 10.1523/JNEUROSCI.2400-10.2010

Drouin-Ouellet J, 2015, INT J NEUROPSYCHOPH, V18, DOI 10.1093/ijnp/pyu103

Ejlerskov P, 2015, CELL, V163, P324, DOI 10.1016/j.cell.2015.08.069

Field R, 2010, BRAIN BEHAV IMMUN, V24, P996, DOI 10.1016/j.bbi.2010.04.004

Friedman LG, 2012, J NEUROSCI, V32, P7585, DOI 10.1523/JNEUROSCI.5809-11.2012

Gao HM, 2011, PROG NEUROBIOL, V94, P1, DOI 10.1016/j.pneurobio.2011.03.005

Halford WP, 1996, J IMMUNOL, V157, P3542

Harris MA, 2012, MOVEMENT DISORD, V27, P1111, DOI 10.1002/mds.25077

Hoban DB, 2013, BRAIN BEHAV IMMUN, V27, P91, DOI 10.1016/j.bbi.2012.10.001

Horvath I, 2012, J AM CHEM SOC, V134, P3439, DOI 10.1021/ja209829m

Itzhaki RF, 2016, J ALZHEIMERS DIS, V51, P979, DOI 10.3233/JAD-160152

Jack CS, 2005, J IMMUNOL, V175, P4320, DOI 10.4049/jimmunol.175.7.4320

Jang HM, 2009, P NATL ACAD SCI USA, V106, P14063, DOI 10.1073/pnas.0900096106

Jeong YH, 2017, PHARMACOL RES, V119, P431, DOI 10.1016/j.phrs.2017.02.027

Kalia LV, 2015, LANCET, V386, P896, DOI 10.1016/S0140-6736(14)61393-3

Kavouras JH, 2007, J NEUROVIROL, V13, P416, DOI 10.1080/13550280701460573

Kim C, 2013, NAT COMMUN, V4, DOI 10.1038/ncomms2534

Kirik D, 2002, J NEUROSCI, V22, P2780, DOI 10.1523/JNEUROSCI.22-07-02780.2002

Komatsu M, 2006, NATURE, V441, P880, DOI 10.1038/nature04723

Liddelow SA, 2017, NATURE, V541, P481, DOI 10.1038/nature21029

Lin RT, 2004, J VIROL, V78, P1675, DOI 10.1128/JVI.78.4.1675-1684.2004

Lövheim H, 2015, ALZHEIMERS DEMENT, V11, P587, DOI 10.1016/j.jalz.2014.07.157

Lussignol M, 2013, J VIROL, V87, P859, DOI 10.1128/JVI.01158-12

MARTTILA RJ, 1982, J NEUROL SCI, V54, P227, DOI 10.1016/0022-510X(82)90184-8

MARTTILA RJ, 1981, ARCH NEUROL-CHICAGO, V38, P19, DOI 10.1001/archneur.1981.00510010045007

McCabe K, 2017, J NEUROIMMUNOL, V310, P103, DOI 10.1016/j.jneuroim.2017.07.007

Menendez CM, 2016, J IMMUNOL, V197, P1262, DOI 10.4049/jimmunol.1600207

Moriarty N, 2017, SCI REP-UK, V7, DOI 10.1038/s41598-017-15970-w

Mulvey M, 1999, J VIROL, V73, P3375, DOI 10.1128/JVI.73.4.3375-3385.1999

O'Neill LAJ, 2013, NAT REV IMMUNOL, V13, P453, DOI 10.1038/nri3446

Olsen Laura K, 2018, Neuronal Signal, V2, pNS20170166, DOI 10.1042/NS20170166

Olsson J, 2016, DIS MODEL MECH, V9, P1349, DOI 10.1242/dmm.026674

OLSSON M, 1995, J NEUROSCI, V15, P3863

Orvedahl A, 2007, CELL HOST MICROBE, V1, P23, DOI 10.1016/j.chom.2006.12.001

Parkinson J, 2002, J NEUROPSYCH CLIN N, V14, P223, DOI 10.1176/appi.neuropsych.14.2.223

Parkinson J., 1817, ESSAY SHAKING PALSY

Pedersen MN, 2015, SCI REP-UK, V5, DOI 10.1038/srep15692

Pokrzywa M, 2017, PLOS ONE, V12, DOI 10.1371/journal.pone.0184117

Poppers J, 2000, J VIROL, V74, P11215, DOI 10.1128/JVI.74.23.11215-11221.2000

POSER CM, 1969, ACTA NEUROL SCAND, V45, P199

PRANZATELLI MR, 1994, PEDIATR NEUROL, V10, P131, DOI 10.1016/0887-8994(94)90045-0

Raman SNT, 2016, FRONT MICROBIOL, V7, DOI 10.3389/fmicb.2016.00654

Reed C, 2012, PLOS ONE, V7, DOI 10.1371/journal.pone.0048187

Sadasivan S, 2017, NPJ PARKINSONS DIS, V3, DOI 10.1038/s41531-017-0019-z

Schallert T, 2000, NEUROPHARMACOLOGY, V39, P777, DOI 10.1016/S0028-3908(00)00005-8

Schapira AH, 2011, MOVEMENT DISORD, V26, P1049, DOI 10.1002/mds.23732

Smith JS, 2002, J INFECT DIS, V186, pS3, DOI 10.1086/343739

Suzumura A, 2006, ANN NY ACAD SCI, V1088, P219, DOI 10.1196/annals.1366.012

Talloczy Z, 2006, AUTOPHAGY, V2, P24, DOI 10.4161/auto.2176

Theil D, 2003, AM J PATHOL, V163, P2179, DOI 10.1016/S0002-9440(10)63575-4

Torres-Odio S, 2017, J NEUROINFLAMM, V14, DOI 10.1186/s12974-017-0928-0

Tse W, 2004, PARKINSONISM RELAT D, V10, P323, DOI 10.1016/j.parkreldis.2004.03.001

Valyi-Nagy T, 2000, VIROLOGY, V278, P309, DOI 10.1006/viro.2000.0678

Vlajinac H, 2013, INT J NEUROSCI, V123, P329, DOI 10.3109/00207454.2012.760560

Watson MB, 2012, EXP NEUROL, V237, P318, DOI 10.1016/j.expneurol.2012.06.025

Yordy B, 2012, CELL HOST MICROBE, V12, P334, DOI 10.1016/j.chom.2012.07.013

Zhao Y, 2006, GLIA, V54, P775, DOI 10.1002/glia.20418

NR 72

TC 14

Z9 17

U1 0

U2 5

PU ACADEMIC PRESS INC ELSEVIER SCIENCE

PI SAN DIEGO

PA 525 B ST, STE 1900, SAN DIEGO, CA 92101-4495 USA

SN 0889-1591

EI 1090-2139

J9 BRAIN BEHAV IMMUN

JI Brain Behav. Immun.

PD AUG

PY 2019

VL 80

BP 525

EP 535

DI 10.1016/j.bbi.2019.04.036

PG 11

WC Immunology; Neurosciences; Psychiatry

WE Science Citation Index Expanded (SCI-EXPANDED)

SC Immunology; Neurosciences & Neurology; Psychiatry

GA IM6LX

UT WOS:000478105500051

PM 31029796

DA 2024-08-12

ER

PT J

AU Ham, HJ

Yeo, IJ

Jeon, SH

Lim, JH

Yoo, SS

Son, DJ

Jang, SS

Lee, H

Shin, SJ

Han, SB

Yun, JS

Hong, JT

AF Ham, Hyeon Joo

Yeo, In Jun

Jeon, Seong Hee

Lim, Jun Hyung

Yoo, Sung Sik

Son, Dong Ju

Jang, Sung-Su

Lee, Haksup

Shin, Seung-Jin

Han, Sang Bae

Yun, Jae Suk

Hong, Jin Tae

TI Botulinum Toxin A Ameliorates Neuroinflammation in the MPTP and

6-OHDA-Induced Parkinson's Disease Models

SO BIOMOLECULES & THERAPEUTICS

LA English

DT Article

DE Parkinson's disease; Botulinum toxin A; Anti-neuroinflammation

ID NEUROTOXIN; NEURODEGENERATION; SYMPTOMS; NEURONS

AB Recently, increasing evidence suggests that neuroinflammation may be a critical factor in the development of Parkinson's disease (PD) in addition to the ratio of acetylcholine/dopamine because dopaminergic neurons are particularly vulnerable to inflammatory attack. In this study, we investigated whether botulinum neurotoxin A (BoNT-A) was effective for the treatment of PD through its anti-neuroinflammatory effects and the modulation of acetylcholine and dopamine release. We found that BoNT-A ameliorated MPTP and 6-OHDA-induced PD progression, reduced acetylcholine release, levels of IL-1 beta, IL-6 and TNF-alpha as well as GFAP expression, but enhanced dopamine release and tyrosine hydroxylase expression. These results indicated that BoNT-A had beneficial effects on MPTP or 6-OHDA-induced PD-like behavior impairments via its anti-neuroinflammation properties, recovering dopamine, and reducing acetylcholine release.

C1 [Ham, Hyeon Joo; Yeo, In Jun; Jeon, Seong Hee; Lim, Jun Hyung; Yoo, Sung Sik; Son, Dong Ju; Han, Sang Bae; Yun, Jae Suk; Hong, Jin Tae] Chungbuk Natl Univ, Coll Pharm, Cheongju 28644, South Korea.

[Ham, Hyeon Joo; Yeo, In Jun; Jeon, Seong Hee; Lim, Jun Hyung; Yoo, Sung Sik; Son, Dong Ju; Han, Sang Bae; Yun, Jae Suk; Hong, Jin Tae] Chungbuk Natl Univ, Med Res Ctr, Cheongju 28644, South Korea.

[Jang, Sung-Su; Lee, Haksup; Shin, Seung-Jin] ATGC Co, Seoul 06372, South Korea.

C3 Chungbuk National University; Chungbuk National University

RP Hong, JT (corresponding author), Chungbuk Natl Univ, Coll Pharm, Cheongju 28644, South Korea.; Hong, JT (corresponding author), Chungbuk Natl Univ, Med Res Ctr, Cheongju 28644, South Korea.

EM jinthong@chungbuk.ac.kr

FU National Research Foundation of Korea [NRF] - Korea government (MSIP)

[2017R1A5A2015541]

FX This work is financially supported by the National Research Foundation

of Korea [NRF] Grant funded by the Korea government (MSIP) (No. MRC,

2017R1A5A2015541) .

CR Antipova V, 2013, J NEUROSCI RES, V91, P838, DOI 10.1002/jnr.23210

Boll MC, 2011, CURR NEUROPHARMACOL, V9, P350, DOI 10.2174/157015911795596577

Bonte MA, 2021, INT IMMUNOPHARMACOL, V95, DOI 10.1016/j.intimp.2021.107526

Butler CA, 2021, J NEUROCHEM, V158, P621, DOI 10.1111/jnc.15327

Chaudhuri KR, 2006, LANCET NEUROL, V5, P235, DOI 10.1016/S1474-4422(06)70373-8

Choi DY, 2013, NEUROBIOL DIS, V49, P159, DOI 10.1016/j.nbd.2012.08.001

Coffield JA, 2009, J PHARMACOL EXP THER, V330, P352, DOI 10.1124/jpet.108.147744

Connolly BS, 2014, JAMA-J AM MED ASSOC, V311, P1670, DOI 10.1001/jama.2014.3654

Day M, 2006, NAT NEUROSCI, V9, P251, DOI 10.1038/nn1632

Ding J, 2006, NAT NEUROSCI, V9, P832, DOI 10.1038/nn1700

Hawlitschka A, 2013, CURR PHARM BIOTECHNO, V14, P124

Hwang CJ, 2017, REDOX BIOL, V11, P456, DOI 10.1016/j.redox.2016.12.008

Jankovic J, 2004, J NEUROL NEUROSUR PS, V75, P951, DOI 10.1136/jnnp.2003.034702

Knuepfer S, 2014, THER ADV UROL, V6, P34, DOI 10.1177/1756287213510962

Liu M, 2011, PARKINSONS DIS-US, V2011, DOI 10.4061/2011/327089

Muñoz-Lora VRM, 2020, TOXICON, V187, P116, DOI 10.1016/j.toxicon.2020.08.027

MARSDEN CD, 1982, NEUROLOGY, V32, P514, DOI 10.1212/WNL.32.5.514

MOGI M, 1994, NEUROSCI LETT, V180, P147, DOI 10.1016/0304-3940(94)90508-8

Oldenburg IA, 2011, CURR OPIN NEUROBIOL, V21, P425, DOI 10.1016/j.conb.2011.04.004

Paxinos G, 1998, RAT BRAIN IN STEREOTAXIC COORDINATES, FOURTH ED., pix

Piotrowska A, 2017, FRONT CELL INFECT MI, V7, DOI 10.3389/fcimb.2017.00141

Godoy MCP, 2008, BRAIN, V131, P1880, DOI 10.1093/brain/awn101

Pringsheim T, 2014, MOVEMENT DISORD, V29, P1583, DOI 10.1002/mds.25945

Rojewska E, 2018, TOXINS, V10, DOI 10.3390/toxins10040145

Rossetto O, 2013, TOXICON, V67, P31, DOI 10.1016/j.toxicon.2013.01.017

Sahinkanat T, 2009, UROLOGY, V73, P405, DOI 10.1016/j.urology.2008.07.051

Stubblefield MD, 2008, ARCH PHYS MED REHAB, V89, P417, DOI 10.1016/j.apmr.2007.11.022

Wang L, 2017, TOXICON, V126, P70, DOI 10.1016/j.toxicon.2016.11.009

Wree A, 2011, NEUROBIOL DIS, V41, P291, DOI 10.1016/j.nbd.2010.09.017

Zhang LY, 2021, NEURAL REGEN RES, V16, P1660, DOI 10.4103/1673-5374.303045

Zhang ZJ, 2021, J PARKINSON DIS, V11, P529, DOI 10.3233/JPD-202318

Zhao YF, 2021, FRONT MOL NEUROSCI, V14, DOI 10.3389/fnmol.2021.641570

NR 32

TC 8

Z9 8

U1 1

U2 15

PU KOREAN SOC APPLIED PHARMACOLOGY

PI SEOUL

PA RM 805, KOREAN FEDERATION SCIENCE & TECHNOLOGY B/D, 635-4 YEOKSAM-DONG,

KANGNAM-GU, SEOUL, 135-703, SOUTH KOREA

SN 1976-9148

EI 2005-4483

J9 BIOMOL THER

JI Biomol. Ther.

PD JAN

PY 2022

VL 30

IS 1

BP 90

EP 97

DI 10.4062/biomolther.2021.077

EA JUN 2021

PG 8

WC Biochemistry & Molecular Biology; Pharmacology & Pharmacy

WE Science Citation Index Expanded (SCI-EXPANDED)

SC Biochemistry & Molecular Biology; Pharmacology & Pharmacy

GA YJ5YT

UT WOS:000701533500001

PM 34078752

OA Green Published, hybrid

DA 2024-08-12

ER

PT J

AU Vivekanantham, S

Shah, S

Dewji, R

Dewji, A

Khatri, C

Ologunde, R

AF Vivekanantham, Sayinthen

Shah, Savan

Dewji, Rizwan

Dewji, Abbas

Khatri, Chetan

Ologunde, Rele

TI Neuroinflammation in Parkinson's disease: role in neurodegeneration and

tissue repair

SO INTERNATIONAL JOURNAL OF NEUROSCIENCE

LA English

DT Review

DE Parkinson's disease; neuroinflammation; inflammation; dopamine;

microglia; nigrostriatal; alpha-synuclein; ageing

ID NONSTEROIDAL ANTIINFLAMMATORY DRUGS; REGULATORY T-CELLS;

ALPHA-SYNUCLEIN; DOPAMINERGIC NEURODEGENERATION; MICROGLIAL ACTIVATION;

OXIDATIVE STRESS; NEUROTROPHIC FACTOR; SUBSTANTIA-NIGRA; LRRK2 MUTATION;

MOUSE MODEL

AB Neuroinflammation in Parkinson's disease [PD] is a process that occurs alongside the loss of dopaminergic neurons, and is associated with alterations to many cell types, most notably microglia. This review examines the key evidence contributing to our understanding of the role of inflammation-mediated degeneration of the dopaminergic (DA) nigrostriatal pathway in PD. It will consider the potential role inflammation plays in tissue repair within the brain, inflammation linked gene products that are associated with sporadic Parkinsonian phenotypes (alpha-synuclein, Parkin and Nurr 1), and developing anti-inflammatory drug treatments in PD. With growing evidence supporting the key role of neuroinflammation in PD pathogenesis, new molecular targets are being found that could potentially prevent or delay nigrostriatal DA neuron loss. Hence, this creates the opportunity for disease modifying treatment, to currently what is an incurable disease.

C1 [Vivekanantham, Sayinthen; Shah, Savan; Dewji, Rizwan; Dewji, Abbas; Khatri, Chetan; Ologunde, Rele] Univ London Imperial Coll Sci Technol & Med, Imperial Coll Sch Med, London SW7 2AZ, United Kingdom.

C3 Imperial College London

RP Vivekanantham, S (corresponding author), Univ London Imperial Coll Sci Technol & Med, Imperial Coll Sch Med, Exhibition Rd, London SW7 2AZ, United Kingdom.

EM sayinthen.vivekanantham09@imperial.ac.uk

RI Ghosh, Dhruva N/AAK-1231-2020

OI Ghosh, Dhruva N/0000-0003-2976-0241; Ologunde, Rele/0000-0002-1203-8327;

Khatri, Chetan/0000-0002-3961-1314

FU Royal College of Surgeons of United Kingdom

FX S. Vivekanantham is supported by the Royal College of Surgeons of

United Kingdom.

CR Austin SA, 2006, J NEUROSCI, V26, P10558, DOI 10.1523/JNEUROSCI.1799-06.2006

Baba Y, 2005, PARKINSONISM RELAT D, V11, P493, DOI 10.1016/j.parkreldis.2005.07.005

Batchelor PE, 2002, MOL CELL NEUROSCI, V21, P436, DOI 10.1006/mcne.2002.1185

Becker C, 2011, EUR J NEUROL, V18, P1336, DOI 10.1111/j.1468-1331.2011.03399.x

Botta-Orfila T, 2014, J NEUROSCI RES, V92, P1071, DOI 10.1002/jnr.23377

Brochard V, 2009, J CLIN INVEST, V119, P182, DOI 10.1172/JCI36470

Carvey PM, 2005, EUR J NEUROSCI, V22, P1158, DOI 10.1111/j.1460-9568.2005.04281.x

Castaño A, 1998, J NEUROCHEM, V70, P1584

Chen HL, 2005, ANN NEUROL, V58, P963, DOI 10.1002/ana.20682

Chen HL, 2003, ARCH NEUROL-CHICAGO, V60, P1059, DOI 10.1001/archneur.60.8.1059

Corti O, 2011, PHYSIOL REV, V91, P1161, DOI 10.1152/physrev.00022.2010

De Pablos RM, 2004, FASEB J, V18, P407, DOI 10.1096/fj.04-2153fje

Driver JA, 2011, BMJ-BRIT MED J, V342, DOI 10.1136/bmj.d198

Faucheux BA, 2003, J NEUROCHEM, V86, P1142, DOI 10.1046/j.1471-4159.2003.01923.x

Floyd RA, 1999, P SOC EXP BIOL MED, V222, P236, DOI 10.1046/j.1525-1373.1999.d01-140.x

Gaig C, 2006, ARCH NEUROL-CHICAGO, V63, P377, DOI 10.1001/archneur.63.3.377

Gao HM, 2008, J NEUROSCI, V28, P7687, DOI 10.1523/JNEUROSCI.0143-07.2008

Gao HM, 2011, ENVIRON HEALTH PERSP, V119, P807, DOI 10.1289/ehp.1003013

Gerhard A, 2006, NEUROBIOL DIS, V21, P404, DOI 10.1016/j.nbd.2005.08.002

Ghosh A, 2007, P NATL ACAD SCI USA, V104, P18754, DOI 10.1073/pnas.0704908104

Gill SS, 2003, NAT MED, V9, P589, DOI 10.1038/nm850

Gregori S, 2001, J IMMUNOL, V167, P1945, DOI 10.4049/jimmunol.167.4.1945

Griffin WST, 2006, J NEUROINFLAMM, V3, DOI 10.1186/1742-2094-3-5

Hakimi M, 2011, J NEURAL TRANSM, V118, P795, DOI 10.1007/s00702-011-0653-2

Hamza TH, 2010, NAT GENET, V42, P781, DOI 10.1038/ng.642

Hernán MA, 2006, NEUROLOGY, V66, P1097, DOI 10.1212/01.wnl.0000204446.82823.28

Hisanaga K, 2001, ARCH NEUROL-CHICAGO, V58, P1580, DOI 10.1001/archneur.58.10.1580

Iravani MM, 2012, NEUROSCI LETT, V510, P138, DOI 10.1016/j.neulet.2012.01.022

Jenner P, 1996, NEUROLOGY, V47, pS161, DOI 10.1212/WNL.47.6_Suppl_3.161S

Johnston LC, 2008, MOL THER, V16, P1392, DOI 10.1038/mt.2008.113

Klegeris A, 2008, NEUROBIOL AGING, V29, P739, DOI 10.1016/j.neurobiolaging.2006.11.013

Kortekaas R, 2005, ANN NEUROL, V57, P176, DOI 10.1002/ana.20369

Lang AE, 2006, ANN NEUROL, V59, P459, DOI 10.1002/ana.20737

Le WD, 2003, NAT GENET, V33, P85, DOI 10.1038/ng1066

Lee JK, 2009, J NEUROIMMUNE PHARM, V4, P419, DOI 10.1007/s11481-009-9176-0

Liberatore GT, 1999, NAT MED, V5, P1403

Lindqvist D, 2012, PLOS ONE, V7, DOI 10.1371/journal.pone.0047387

Liu ZH, 2011, NAT IMMUNOL, V12, P1063, DOI 10.1038/ni.2113

Mccoy MK, 2006, J NEUROSCI, V26, P9365, DOI 10.1523/JNEUROSCI.1504-06.2006

McGeer E G, 1988, Alzheimer Dis Assoc Disord, V2, P331, DOI 10.1097/00002093-198802040-00001

McGeer PL, 2008, MOVEMENT DISORD, V23, P474, DOI 10.1002/mds.21751

McGeer PL, 2004, PARKINSONISM RELAT D, V10, pS3, DOI 10.1016/j.parkreldis.2004.01.005

MCGEER PL, 1988, ANN NEUROL, V24, P574, DOI 10.1002/ana.410240415

Mizuno Y, 2001, ADV NEUROL, V86, P13

Monticelli S, 2002, EUR J IMMUNOL, V32, P2971, DOI 10.1002/1521-4141(2002010)32:10<2971::AID-IMMU2971>3.0.CO;2-G

Moore DJ, 2005, ANNU REV NEUROSCI, V28, P57, DOI 10.1146/annurev.neuro.28.061604.135718

Mosley RL, 2012, CSH PERSPECT MED, V2, DOI 10.1101/cshperspect.a009381

Nagatsu T, 2006, CELL MOL NEUROBIOL, V26, P781, DOI 10.1007/s10571-006-9061-9

Nakamura Y, 2002, BIOL PHARM BULL, V25, P945, DOI 10.1248/bpb.25.945

Nalls MA, 2011, LANCET, V377, P641, DOI 10.1016/S0140-6736(10)62345-8

Nichols WC, 2005, LANCET, V365, P410, DOI 10.1016/S0140-6736(05)17828-3

Olanow CW, 2007, MOVEMENT DISORD, V22, pS335, DOI 10.1002/mds.21675

Ossola B, 2011, NEUROPHARMACOLOGY, V61, P574, DOI 10.1016/j.neuropharm.2011.04.030

Purisai MG, 2007, NEUROBIOL DIS, V25, P392, DOI 10.1016/j.nbd.2006.10.008

Puschmann A, 2011, PARKINSONISM RELAT D, V17, P376, DOI 10.1016/j.parkreldis.2011.03.008

Qin LY, 2007, GLIA, V55, P453, DOI 10.1002/glia.20467

Rees K, 2011, COCHRANE DB SYST REV, DOI 10.1002/14651858.CD008454.pub2

Reinhardt P, 2013, CELL STEM CELL, V12, P354, DOI 10.1016/j.stem.2013.01.008

Reynolds AD, 2007, J LEUKOCYTE BIOL, V82, P1083, DOI 10.1189/jlb.0507296

Reynolds AD, 2010, J IMMUNOL, V184, P2261, DOI 10.4049/jimmunol.0901852

Reynolds AD, 2009, J IMMUNOL, V182, P4137, DOI 10.4049/jimmunol.0803982

Rezai-Zadeh K, 2009, J NEUROIMMUNE PHARM, V4, P462, DOI 10.1007/s11481-009-9166-2

Saijo K, 2009, CELL, V137, P47, DOI 10.1016/j.cell.2009.01.038

Saiki M, 2010, J NEUROL NEUROSUR PS, V81, P890, DOI 10.1136/jnnp.2008.162883

Samii A, 2009, DRUG AGING, V26, P769, DOI 10.2165/11316780-000000000-00000

Sánchez-Pernaute R, 2004, J NEUROINFLAMM, V1, DOI 10.1186/1742-2094-1-6

Schwartz M, 2006, TRENDS NEUROSCI, V29, P68, DOI 10.1016/j.tins.2005.12.005

Shamoto-Nagai M, 2006, J NEURAL TRANSM, V113, P633, DOI 10.1007/s00702-005-0410-5

Shimura H, 2000, NAT GENET, V25, P302, DOI 10.1038/77060

Shoham S, 2000, CELL MOL BIOL, V46, P743

Simón-Sánchez J, 2011, EUR J HUM GENET, V19, P655, DOI 10.1038/ejhg.2010.254

Stone DK, 2009, ANTIOXID REDOX SIGN, V11, P2151, DOI [10.1089/ars.2009.2460, 10.1089/ARS.2009.2460]

Su XM, 2008, NEUROBIOL AGING, V29, P1690, DOI 10.1016/j.neurobiolaging.2007.04.006

Tanaka S, 2013, J NEUROINFLAMM, V10, DOI 10.1186/1742-2094-10-143

Tanner CM, 2003, ADV NEUROL, V91, P133

Tansey MG, 2007, EXP NEUROL, V208, P1, DOI 10.1016/j.expneurol.2007.07.004

Tasaki Y, 2012, NEUROSCI LETT, V521, P15, DOI 10.1016/j.neulet.2012.05.045

Teismann P, 2004, CELL TISSUE RES, V318, P149, DOI 10.1007/s00441-004-0944-0

Teismann P, 2003, ANN NY ACAD SCI, V991, P272

Thakur P, 2013, NEUROSCIENCE, V231, P420, DOI 10.1016/j.neuroscience.2012.11.006

Tran TA, 2011, PLOS ONE, V6, DOI 10.1371/journal.pone.0023660

Witte ME, 2010, MITOCHONDRION, V10, P411, DOI 10.1016/j.mito.2010.05.014

Wu DC, 2003, P NATL ACAD SCI USA, V100, P6145, DOI 10.1073/pnas.0937239100

Zecca L, 2004, P NATL ACAD SCI USA, V101, P9843, DOI 10.1073/pnas.0403495101

Zecca L, 2008, ACTA NEUROPATHOL, V116, P47, DOI 10.1007/s00401-008-0361-7

NR 85

TC 130

Z9 140

U1 3

U2 44

PU TAYLOR & FRANCIS LTD

PI ABINGDON

PA 2-4 PARK SQUARE, MILTON PARK, ABINGDON OR14 4RN, OXON, United Kingdom

SN 0020-7454

EI 1563-5279

J9 INT J NEUROSCI

JI Int. J. Neurosci.

PY 2015

VL 125

IS 10

BP 717

EP 725

DI 10.3109/00207454.2014.982795

PG 9

WC Neurosciences

WE Science Citation Index Expanded (SCI-EXPANDED)

SC Neurosciences & Neurology

GA CQ9BT

UT WOS:000360906600001

PM 25364880

DA 2024-08-12

ER

PT J

AU La Vitola, P

Balducci, C

Baroni, M

Artioli, L

Santamaria, G

Castiglioni, M

Cerovic, M

Colombo, L

Caldinelli, L

Pollegioni, L

Forloni, G

AF La Vitola, P.

Balducci, C.

Baroni, M.

Artioli, L.

Santamaria, G.

Castiglioni, M.

Cerovic, M.

Colombo, L.

Caldinelli, L.

Pollegioni, L.

Forloni, G.

TI Peripheral inflammation exacerbates α-synuclein toxicity and

neuropathology in Parkinson's models

SO NEUROPATHOLOGY AND APPLIED NEUROBIOLOGY

LA English

DT Article

DE Parkinson's disease; oligomeropathies; alpha-synuclein oligomers;

nonmotor deficits; neuroinflammation

ID LONG-TERM POTENTIATION; GLIAL-CELL ACTIVATION; SYNAPTIC PLASTICITY;

CEREBROSPINAL-FLUID; IMPAIR MEMORY; OLIGOMERS; DISEASE;

NEUROINFLAMMATION; BRAIN; INTERLEUKIN-6

AB Aims: Parkinson's disease and related disorders are devastating neurodegenerative pathologies. Since alpha-synuclein was identified as a main component of Lewy bodies and neurites, efforts have been made to clarify the pathogenic mechanisms of alpha-synuclein's detrimental effects. alpha-synuclein oligomers are the most harmful species and may recruit and activate glial cells. Inflammation is emerging as a bridge between genetic susceptibility and environmental factors co-fostering Parkinson's disease. However, direct evidence linking inflammation to the harmful activities of alpha-synuclein oligomers or to the Parkinson's disease behavioural phenotype is lacking. Methods: To clarify whether neuroinflammation influences Parkinson's disease pathogenesis, we developed: (i) a 'double-hit' approach in C57BL/6 naive mice where peripherally administered lipopolysaccharides were followed by intracerebroventricular injection of an inactive oligomer dose; (ii) a transgenic 'double-hit' model where lipopolysaccharides were given to A53T alpha-synuclein transgenic Parkinson's disease mice. Results: Lipopolysaccharides induced a long-lasting neuroinflammatory response which facilitated the detrimental cognitive activities of oligomers. LPS-activated microglia and astrocytes responded differently to the oligomers with microglia activating further and acquiring a pro-inflammatory M1 phenotype, while astrocytes atrophied. In the transgenic 'double-hit' A53T mouse model, lipopolysaccharides aggravated cognitive deficits and increased microgliosis. Again, astrocytes responded differently to the double challenge. These findings indicate that peripherally induced neuroinflammation potentiates the alpha-synuclein oligomer's actions and aggravates cognitive deficits in A53T mice. Conclusions: The fine management of both peripheral and central inflammation may offer a promising therapeutic approach to prevent or slow down some behavioural aspects in alpha-synucleinopathies.

C1 [La Vitola, P.; Balducci, C.; Baroni, M.; Artioli, L.; Santamaria, G.; Castiglioni, M.; Cerovic, M.; Forloni, G.] Ist Ric Farmacol Mario Negri IRCCS, Dept Neurosci, Milan, Italy.

[Colombo, L.] Ist Ric Farmacol Mario Negri IRCCS, Dept Mol Biochem & Pharmacol, Milan, Italy.

[Caldinelli, L.; Pollegioni, L.] Univ Insubria, Dept Biotechnol & Life Sci, Varese, Italy.

C3 Istituto di Ricerche Farmacologiche Mario Negri IRCCS; Istituto di

Ricerche Farmacologiche Mario Negri IRCCS; University of Insubria

RP Forloni, G (corresponding author), Mario Negri Inst Pharmacol Res IRCCS, Dept Neurosci, Via Mario Negri 2, I-20156 Milan, Italy.

EM gianluigi.forloni@marionegri.it

RI Forloni, Gianluigi/AAB-2115-2020; Santamaria, Giulia/HKO-3470-2023;

Baroni, Marco Giorgio/AIA-6973-2022; colombo, laura/neurodegenerative disease-6716-2020; La

Vitola, Pietro/AHE-9241-2022; Balducci, Claudia/neurodegenerative disease-8465-2020

OI Forloni, Gianluigi/0000-0001-5374-3914; colombo,

laura/0000-0002-3243-0270; Balducci, Claudia/0000-0002-3870-835X;

Cerovic, Milica/0000-0002-8417-0503; Artioli, Luisa/0000-0001-8763-0715;

Santamaria, Giulia/0000-0002-2952-9325; CALDINELLI,

LAURA/0000-0002-0691-0203; La Vitola, Pietro/0000-0001-7415-0739

CR Ascherio A, 2016, LANCET NEUROL, V15, P1255, DOI 10.1016/S1474-4422(16)30230-7

Bacci A, 1999, PHILOS T R SOC B, V354, P403, DOI 10.1098/rstb.1999.0393

Balducci C, 2017, BRAIN BEHAV IMMUN, V60, P188, DOI 10.1016/j.bbi.2016.10.012

Balducci C, 2010, P NATL ACAD SCI USA, V107, P2295, DOI 10.1073/pnas.0911829107

Bengoa-Vergniory N, 2017, ACTA NEUROPATHOL, V134, P819, DOI 10.1007/s00401-017-1755-1

Biosa A, 2018, MOL NEUROBIOL, V55, P8754, DOI 10.1007/s12035-018-1025-9

Blank T, 2013, GLIA, V61, P62, DOI 10.1002/glia.22372

BlumDegen D, 1995, NEUROSCI LETT, V202, P17, DOI 10.1016/0304-3940(95)12192-7

Caggiu E, 2019, FRONT NEUROL, V10, DOI 10.3389/fneur.2019.00122

Caldinelli L, 2013, BMC BIOTECHNOL, V13, DOI 10.1186/1472-6750-13-32

Chao YX, 2014, BIOMED RES INT, V2014, DOI 10.1155/2014/308654

Ferrari CC, 2006, NEUROBIOL DIS, V24, P183, DOI 10.1016/j.nbd.2006.06.013

Clough Z, 2013, ALZ DIS ASSOC DIS, V29, P97

Diógenes MJ, 2012, J NEUROSCI, V32, P11750, DOI 10.1523/JNEUROSCI.0234-12.2012

Donzis EJ, 2014, NEUROBIOL LEARN MEM, V115, P68, DOI 10.1016/j.nlm.2014.08.008

Durante V, 2019, BRAIN, V142, P1365, DOI 10.1093/brain/awz065

Dzamko N, 2015, NEUROSCIENCE, V302, P89, DOI 10.1016/j.neuroscience.2014.10.028

Fellner L, 2013, MOL NEUROBIOL, V47, P575, DOI 10.1007/s12035-012-8340-3

Fellner L, 2011, ACTA NEUROPATHOL, V121, P675, DOI 10.1007/s00401-011-0833-z

Forloni G, 2016, MOVEMENT DISORD, V31, P771, DOI 10.1002/mds.26624

Gao HM, 2011, ENVIRON HEALTH PERSP, V119, P807, DOI 10.1289/ehp.1003013

Graham DR, 2010, J NEUROSCI RES, V88, P1777, DOI 10.1002/jnr.22331

Halliday GM, 2011, MOVEMENT DISORD, V26, P6, DOI 10.1002/mds.23455

Hertz L, 2016, FRONT INTEGR NEUROSC, V10, DOI 10.3389/fnint.2016.00031

Hirsch EC, 2009, LANCET NEUROL, V8, P382, DOI 10.1016/S1474-4422(09)70062-6

Jander S, 2000, J NEUROIMMUNOL, V109, P181, DOI 10.1016/S0165-5728(00)00317-9

Kalia LV, 2015, LANCET, V386, P896, DOI 10.1016/S0140-6736(14)61393-3

Kim C, 2016, EXP NEUROBIOL, V25, P113, DOI 10.5607/en.2016.25.3.113

Kim C, 2013, NAT COMMUN, V4, DOI 10.1038/ncomms2534

Kuo YM, 1996, J BIOL CHEM, V271, P4077

La Vitola P, 2019, BRAIN, V142, P249, DOI 10.1093/brain/awy318

La Vitola P, 2018, BRAIN BEHAV IMMUN, V69, P591, DOI 10.1016/j.bbi.2018.02.012

Li AJ, 1997, BRAIN RES, V748, P30, DOI 10.1016/S0006-8993(96)01283-8

Lim S, 2016, BRAIN PATHOL, V26, P404, DOI 10.1111/bpa.12371

Ma JH, 2014, MOL BRAIN, V7, DOI 10.1186/1756-6606-7-33

Marques O, 2012, CELL DEATH DIS, V3, DOI 10.1038/cddis.2012.94

Martin ZS, 2012, J NEUROCHEM, V120, P440, DOI 10.1111/j.1471-4159.2011.07576.x

McKenzie Jordan A, 2017, Curr Aging Sci, V10, P158, DOI 10.2174/1874609810666170315113244

MOGI M, 1994, NEUROSCI LETT, V165, P208, DOI 10.1016/0304-3940(94)90746-3

Morris GP, 2013, NEUROBIOL LEARN MEM, V105, P40, DOI 10.1016/j.nlm.2013.07.002

Mucke L, 2012, CSH PERSPECT MED, V2, DOI 10.1101/cshperspect.a006338

Muller T, 1998, ACTA NEUROL SCAND, V98, P142

Nenov MN, 2019, INT J MOL SCI, V20, DOI 10.3390/ijms20133375

Ono K, 2017, NEUROCHEM RES, V42, P3362, DOI 10.1007/s11064-017-2382-x

Outeiro TF, 2019, MOL NEURODEGENER, V14, DOI 10.1186/s13024-019-0306-8

Paleologou KE, 2009, BRAIN, V132, P1093, DOI 10.1093/brain/awn349

Paumier KL, 2013, PLOS ONE, V8, DOI 10.1371/journal.pone.0070274

Pischiutta F, 2018, EXP NEUROL, V300, P167, DOI 10.1016/j.expneurol.2017.11.003

Qin LY, 2007, GLIA, V55, P453, DOI 10.1002/glia.20467

Rai S, 2013, J NEUROIMMUNOL, V254, P1, DOI 10.1016/j.jneuroim.2012.08.008

Richwine AF, 2009, BRAIN BEHAV IMMUN, V23, P794, DOI 10.1016/j.bbi.2009.02.020

Rosi S, 2004, J NEUROINFLAMM, V1, DOI 10.1186/1742-2094-1-12

Scott DA, 2010, J NEUROSCI, V30, P8083, DOI 10.1523/JNEUROSCI.1091-10.2010

Singh B, 2019, ACTA NEUROPATHOL, V138, P551, DOI 10.1007/s00401-019-02032-w

Sorrentino ZA, 2019, ACTA NEUROPATHOL, V138, P1, DOI 10.1007/s00401-019-01977-2

Subramaniam SR, 2017, FRONT AGING NEUROSCI, V9, DOI 10.3389/fnagi.2017.00176

Surendranathan A, 2015, PARKINSONISM RELAT D, V21, P1398, DOI 10.1016/j.parkreldis.2015.10.009

Tancredi V, 2000, J NEUROCHEM, V75, P634, DOI 10.1046/j.1471-4159.2000.0750634.x

Tong JC, 2015, NEUROBIOL DIS, V82, P243, DOI 10.1016/j.nbd.2015.06.010

Wang JT, 2019, MOL NEUROBIOL, V56, P7664, DOI 10.1007/s12035-019-1567-5

Winner B, 2011, P NATL ACAD SCI USA, V108, P4194, DOI 10.1073/pnas.1100976108

Xu YJ, 2018, BIOMED RES INT, V2018, DOI 10.1155/2018/4606752

Yu SY, 2014, BMC NEUROL, V14, DOI 10.1186/1471-2377-14-113

NR 63

TC 55

Z9 55

U1 3

U2 20

PU WILEY

PI HOBOKEN

PA 111 RIVER ST, HOBOKEN 07030-5774, NJ USA

SN 0305-1846

EI 1365-2990

J9 NEUROPATH APPL NEURO

JI Neuropathol. Appl. Neurobiol.

PD FEB

PY 2021

VL 47

IS 1

BP 43

EP 60

DI 10.1111/nan.12644

EA AUG 2020

PG 18

WC Clinical Neurology; Neurosciences; Pathology

WE Science Citation Index Expanded (SCI-EXPANDED)

SC Neurosciences & Neurology; Pathology

GA PZ1VY

UT WOS:000556080600001

PM 32696999

DA 2024-08-12

ER

PT J

AU Lee, Y

Lee, S

Chang, SC

Lee, J

AF Lee, Yujeong

Lee, Seulah

Chang, Seung-Cheol

Lee, Jaewon

TI Significant roles of neuroinflammation in Parkinson's disease:

therapeutic targets for PD prevention

SO ARCHIVES OF PHARMACAL RESEARCH

LA English

DT Review

DE Parkinson's disease; Neuroinflammation; Glia; A1 astrocyte; A2

astrocyte; Microglia

ID NF-KAPPA-B; PROTECTS DOPAMINERGIC-NEURONS; TUMOR-NECROSIS-FACTOR;

ALPHA-SYNUCLEIN; MOUSE MODEL; MICROGLIAL ACTIVATION; MEDIATED

NEUROPROTECTION; INFLAMMATORY RESPONSES; MACROPHAGE ACTIVATION;

ASTROGLIAL ACTIVATION

AB Glial cells outnumber neurons in the brain and play important roles in the neuroinflammation that accompanies brain damage in neurodegenerative disease. In Parkinson's disease (PD), dopaminergic neuronal loss is accompanied by inflammatory changes in microglia, astrocytes, innate immune cells, and infiltrating peripheral immune cells. Neuroinflammation is probably a fundamental immune response to protect neurons from harm and compensate for neuronal damage, but at the same time, its neurotoxic effects exacerbate neuron damage. Furthermore, neuroinflammatory response is regulated by immune cells, such as microglia, astrocytes, and peripheral immune cells, and by cytokines and chemokines. Accordingly, it is crucial that we understand how such immune cells in the brain regulate neuroinflammatory responses in PD pathology. This review describes the roles played by glia-mediated neuroinflammation in PD, both good and bad, and the therapeutic strategies used to treat PD.

C1 [Lee, Yujeong; Lee, Seulah; Lee, Jaewon] Pusan Natl Univ, Dept Pharm, Coll Pharm, Busan 46241, South Korea.

[Chang, Seung-Cheol] Pusan Natl Univ, Inst BioPhysio Sensor Technol, Busan 46241, South Korea.

C3 Pusan National University; Pusan National University

RP Lee, J (corresponding author), Pusan Natl Univ, Dept Pharm, Coll Pharm, Busan 46241, South Korea.

EM neuron@pusan.ac.kr

RI chang, seung-cheol/K-8778-2017; Lee, Jaewon/IQR-8432-2023; Lee,

Jaewon/N-9064-2013

OI Lee, Jaewon/0000-0003-3203-2130; Chang, Seung-Cheol/0000-0002-2333-9424

FU National Research Foundation of Korea (NRF) [2018R1D1A1B07043493]

FX This work was supported by the National Research Foundation of Korea

(NRF) (Grant no. 2018R1D1A1B07043493).

CR Agarwal S, 2017, BIOCHEM BIOPH RES CO, V483, P1166, DOI 10.1016/j.bbrc.2016.08.043

Agostinho P, 2010, CURR PHARM DESIGN, V16, P2766, DOI 10.2174/138161210793176572

Aïd S, 2011, BIOCHIMIE, V93, P46, DOI 10.1016/j.biochi.2010.09.009

Aloisi F, 2001, GLIA, V36, P165, DOI 10.1002/glia.1106

Anderson MA, 2016, NATURE, V532, P195, DOI 10.1038/nature17623

Appel SH, 2010, TRENDS IMMUNOL, V31, P7, DOI 10.1016/j.it.2009.09.003

Attwell D, 2010, NATURE, V468, P232, DOI 10.1038/nature09613

Barcia C, 2011, CELL DEATH DIS, V2, DOI 10.1038/cddis.2011.17

Bayraktar OA, 2015, CSH PERSPECT BIOL, V7, DOI 10.1101/cshperspect.a020362

Becerra-Calixto A, 2017, FRONT MOL NEUROSCI, V10, DOI 10.3389/fnmol.2017.00088

Benarroch EE, 2013, NEUROLOGY, V81, P1079, DOI 10.1212/WNL.0b013e3182a4a577

Bernardinelli Y, 2014, CURR BIOL, V24, P1679, DOI 10.1016/j.cub.2014.06.025

Boato F, 2011, J NEUROINFLAMM, V8, DOI 10.1186/1742-2094-8-183

BOKA G, 1994, NEUROSCI LETT, V172, P151, DOI 10.1016/0304-3940(94)90684-X

Bortolanza M, 2015, NEUROBIOL DIS, V73, P377, DOI 10.1016/j.nbd.2014.10.017

Breidert T, 2002, J NEUROCHEM, V82, P615, DOI 10.1046/j.1471-4159.2002.00990.x

BRENNEMAN DE, 1992, J NEUROCHEM, V58, P454, DOI 10.1111/j.1471-4159.1992.tb09743.x

Brochard V, 2009, J CLIN INVEST, V119, P182, DOI 10.1172/JCI36470

Calabrese V, 2018, FREE RADICAL BIO MED, V115, P80, DOI 10.1016/j.freeradbiomed.2017.10.379

Chen PC, 2009, P NATL ACAD SCI USA, V106, P2933, DOI 10.1073/pnas.0813361106

Chen ZC, 2018, PROG NEUROBIOL, V169, P1, DOI 10.1016/j.pneurobio.2018.08.002

Cheray M, 2018, FRONT CELL NEUROSCI, V12, DOI 10.3389/fncel.2018.00243

Cherry JD, 2014, J NEUROINFLAMM, V11, DOI 10.1186/1742-2094-11-98

Chhor V, 2013, BRAIN BEHAV IMMUN, V32, P70, DOI 10.1016/j.bbi.2013.02.005

Choi SY, 2012, J NEUROSCI RES, V90, P1842, DOI 10.1002/jnr.23061

Collins LM, 2012, NEUROPHARMACOLOGY, V62, P2154, DOI 10.1016/j.neuropharm.2012.01.028

Curran CS, 2012, J NEUROINFLAMM, V9, DOI 10.1186/1742-2094-9-11

Dehmer T, 2004, J NEUROCHEM, V88, P494, DOI 10.1046/j.1471-4159.2003.02210.x

Doorn KJ, 2014, ACTA NEUROPATHOL COM, V2, DOI 10.1186/s40478-014-0090-1

Eikelenboom P, 2010, NEURODEGENER DIS, V7, P38, DOI 10.1159/000283480

'Episcopo Francesca L, 2013, Curr Aging Sci, V6, P45

Fawcett JW, 1999, BRAIN RES BULL, V49, P377, DOI 10.1016/S0361-9230(99)00072-6

Franco R, 2015, PROG NEUROBIOL, V131, P65, DOI 10.1016/j.pneurobio.2015.05.003

Gan L, 2012, J NEUROSCI, V32, P17775, DOI 10.1523/JNEUROSCI.3049-12.2012

Gelders G, 2018, J IMMUNOL RES, V2018, DOI 10.1155/2018/4784268

Glass CK, 2010, CELL, V140, P918, DOI 10.1016/j.cell.2010.02.016

Glastras SJ, 2016, SCI REP-UK, V6, DOI 10.1038/srep23525

Gorshkov K, 2018, DRUG DISCOV TODAY, V23, P673, DOI 10.1016/j.drudis.2018.01.011

Graeber MB, 2010, SCIENCE, V330, P783, DOI 10.1126/science.1190929

Gu XL, 2010, MOL BRAIN, V3, DOI 10.1186/1756-6606-3-12

Harms AS, 2011, MOL THER, V19, P46, DOI 10.1038/mt.2010.217

Harry GJ, 2008, EXPERT OPIN DRUG MET, V4, P1265, DOI 10.1517/17425255.4.10.1265

Hashida K, 2012, PLOS ONE, V7, DOI 10.1371/journal.pone.0047950

Hirsch EC, 2009, LANCET NEUROL, V8, P382, DOI 10.1016/S1474-4422(09)70062-6

Hoenen C, 2016, PLOS ONE, V11, DOI 10.1371/journal.pone.0162717

Hühner L, 2017, FRONT MOL NEUROSCI, V10, DOI 10.3389/fnmol.2017.00062

Iannaccone S, 2013, PARKINSONISM RELAT D, V19, P47, DOI 10.1016/j.parkreldis.2012.07.002

Imamura K, 2003, ACTA NEUROPATHOL, V106, P518, DOI 10.1007/s00401-003-0766-2

Jakel RJ, 2007, BRAIN RES, V1144, P192, DOI 10.1016/j.brainres.2007.01.131

Janda E, 2018, FRONT MOL NEUROSCI, V11, DOI 10.3389/fnmol.2018.00144

Joers V, 2017, PROG NEUROBIOL, V155, P57, DOI 10.1016/j.pneurobio.2016.04.006

Kim C, 2013, NAT COMMUN, V4, DOI 10.1038/ncomms2534

Kim JH, 2013, NEUROBIOL DIS, V60, P1, DOI 10.1016/j.nbd.2013.08.007

Kim ME, 2016, ARCH PHARM RES, V39, P1160, DOI 10.1007/s12272-016-0802-0

Klegeris A, 2008, NEUROBIOL AGING, V29, P739, DOI 10.1016/j.neurobiolaging.2006.11.013

Koprich JB, 2008, J NEUROINFLAMM, V5, DOI 10.1186/1742-2094-5-8

Kreft M, 2012, ASN NEURO, V4, DOI 10.1042/AN20120007

Kreutzberg GW, 1996, TRENDS NEUROSCI, V19, P312, DOI 10.1016/0166-2236(96)10049-7

Lan X, 2017, NAT REV NEUROL, V13, P420, DOI 10.1038/nrneurol.2017.69

Lastres-Becker I, 2012, HUM MOL GENET, V21, P3173, DOI 10.1093/hmg/dds143

Ledesma MD, 2002, J NEUROCHEM, V83, P1431, DOI 10.1046/j.1471-4159.2002.01253.x

Lee E, 2014, J NEUROSCI RES, V92, P130, DOI 10.1002/jnr.23307

Lee HJ, 2010, J BIOL CHEM, V285, P9262, DOI 10.1074/jbc.M109.081125

Lee KW, 2015, NEUROBIOL AGING, V36, P519, DOI 10.1016/j.neurobiolaging.2014.07.034

Lee KM, 2016, J NEUROSCI RES, V94, P865, DOI 10.1002/jnr.23764

Lee Y, 2019, BRAIN RES, V1704, P47, DOI 10.1016/j.brainres.2018.09.036

Lee Y, 2015, BRAIN RES, V1627, P233, DOI 10.1016/j.brainres.2015.09.029

Li R, 2004, J NEUROSCI RES, V78, P723, DOI 10.1002/jnr.20315

Liddelow SA, 2017, IMMUNITY, V46, P957, DOI 10.1016/j.immuni.2017.06.006

Liddelow SA, 2017, NATURE, V541, P481, DOI 10.1038/nature21029

Litteljohn D, 2009, NEUROSCIENCE, V164, P1894, DOI 10.1016/j.neuroscience.2009.09.025

Loane DJ, 2010, NEUROTHERAPEUTICS, V7, P366, DOI 10.1016/j.nurt.2010.07.002

Lucas SM, 2006, BRIT J PHARMACOL, V147, pS232, DOI 10.1038/sj.bjp.0706400

MacCormac LP, 2004, EUR J NEUROSCI, V20, P2038, DOI 10.1111/j.1460-9568.2004.03659.x

Mantovani A, 2004, TRENDS IMMUNOL, V25, P677, DOI 10.1016/j.it.2004.09.015

Martinez FO, 2008, FRONT BIOSCI-LANDMRK, V13, P453, DOI 10.2741/2692

McCoy MK, 2008, MOL THER, V16, P1572, DOI 10.1038/mt.2008.146

McGuire SO, 2001, EXP NEUROL, V169, P219, DOI 10.1006/exnr.2001.7688

Mirza B, 2000, NEUROSCIENCE, V95, P425

MOGI M, 1994, NEUROSCI LETT, V180, P147, DOI 10.1016/0304-3940(94)90508-8

Mullett SJ, 2009, NEUROBIOL DIS, V33, P28, DOI 10.1016/j.nbd.2008.09.013

Nakagawa Y, 2015, PHARMACOL THERAPEUT, V154, P21, DOI 10.1016/j.pharmthera.2015.06.010

Neal M, 2018, BBA-MOL BASIS DIS, V1864, P432, DOI 10.1016/j.bbadis.2017.11.004

Niranjan R, 2018, NEUROCHEM INT, V120, P13, DOI 10.1016/j.neuint.2018.07.003

Niranjan R, 2014, MOL NEUROBIOL, V49, P28, DOI 10.1007/s12035-013-8483-x

Ouchi Y, 2005, ANN NEUROL, V57, P168, DOI 10.1002/ana.20338

Pekny M, 2014, NEUROSCI LETT, V565, P30, DOI 10.1016/j.neulet.2013.12.071

Pisanu A, 2014, NEUROBIOL DIS, V71, P280, DOI 10.1016/j.nbd.2014.08.011

Prabhakaran K, 2011, TOXICOL MECH METHOD, V21, P435, DOI 10.3109/15376516.2011.560210

Ransohoff RM, 2016, SCIENCE, V353, P777, DOI 10.1126/science.aag2590

Rappold PM, 2010, NEUROTHERAPEUTICS, V7, P413, DOI 10.1016/j.nurt.2010.07.001

Reynolds AD, 2007, J LEUKOCYTE BIOL, V82, P1083, DOI 10.1189/jlb.0507296

Rojo AI, 2014, ANTIOXID REDOX SIGN, V21, P1766, DOI 10.1089/ars.2013.5745

Saijo K, 2009, CELL, V137, P47, DOI 10.1016/j.cell.2009.01.038

Sawada M, 2006, ROLE CYTOKINES INFLA, P273

Schnegg CI, 2012, FREE RADICAL BIO MED, V52, P1734, DOI 10.1016/j.freeradbiomed.2012.02.032

Simon C, 2011, GLIA, V59, P869, DOI 10.1002/glia.21156

Sofroniew MV, 2015, NAT REV NEUROSCI, V16, P249, DOI 10.1038/nrn3898

Streit WJ, 2004, J NEUROINFLAMM, V1, DOI 10.1186/1742-2094-1-14

Suh HS, 2013, J NEUROINFLAMM, V10, DOI 10.1186/1742-2094-10-37

Surmeier DJ, 2012, CSH PERSPECT MED, V2, DOI 10.1101/cshperspect.a009290

Wang QQ, 2015, TRANSL NEURODEGENER, V4, DOI 10.1186/s40035-015-0042-0

Wang WY, 2015, ANN TRANSL MED, V3, DOI 10.3978/j.issn.2305-5839.2015.03.49

Wang YL, 2017, CELL PHYSIOL BIOCHEM, V43, P2226, DOI 10.1159/000484302

Wilms H, 2009, INT J IMMUNOPATH PH, V22, P897, DOI 10.1177/039463200902200405

Xu XT, 2019, J CELL PHYSIOL, V234, P904, DOI 10.1002/jcp.26918

Yadav S, 2012, NEUROCHEM RES, V37, P875, DOI 10.1007/s11064-011-0682-0

Yang JS, 2017, INFLAMMOPHARMACOLOGY, V25, P471, DOI 10.1007/s10787-017-0348-x

Yang TT, 2013, BRAIN STRUCT FUNCT, V218, P1051, DOI 10.1007/s00429-012-0446-x

Yun SP, 2018, NAT MED, V24, P931, DOI 10.1038/s41591-018-0051-5

Zamanian JL, 2012, J NEUROSCI, V32, P6391, DOI 10.1523/JNEUROSCI.6221-11.2012

Zhang QS, 2017, ACTA PHARMACOL SIN, V38, P1317, DOI 10.1038/aps.2017.49

NR 112

TC 113

Z9 122

U1 0

U2 28

PU PHARMACEUTICAL SOC KOREA

PI SEOUL

PA 1489-3 SUHCHO-DONG, SUHCHO-KU, SEOUL 137-071, SOUTH KOREA

SN 0253-6269

EI 1976-3786

J9 ARCH PHARM RES

JI Arch. Pharm. Res.

PD MAY

PY 2019

VL 42

IS 5

SI SI

BP 416

EP 425

DI 10.1007/s12272-019-01133-0

PG 10

WC Chemistry, Medicinal; Pharmacology & Pharmacy

WE Science Citation Index Expanded (SCI-EXPANDED)

SC Pharmacology & Pharmacy

GA HX4RB

UT WOS:000467386600004

PM 30830660

DA 2024-08-12

ER

PT J

AU Padhi, P

Worth, C

Zenitsky, G

Jin, HJ

Sambamurti, K

Anantharam, V

Kanthasamy, A

Kanthasamy, AG

AF Padhi, Piyush

Worth, Carter

Zenitsky, Gary

Jin, Huajun

Sambamurti, Kumar

Anantharam, Vellareddy

Kanthasamy, Arthi

Kanthasamy, Anumantha G.

TI Mechanistic Insights Into Gut Microbiome Dysbiosis-Mediated Neuroimmune

Dysregulation and Protein Misfolding and Clearance in the Pathogenesis

of Chronic Neurodegenerative Disorders

SO FRONTIERS IN NEUROSCIENCE

LA English

DT Review

DE neurodegeneration; microbiome; neuroinflammation; protein aggregation;

gut microbiota; gut metabolome; gut inflammation; Alzheimer's and

Parkinson's diseases

ID INFLAMMATORY-BOWEL-DISEASE; CHAIN FATTY-ACIDS; parkinson's disease;

ALPHA-SYNUCLEIN; VITAMIN-B12 DEFICIENCY; NONMOTOR SYMPTOMS; LEWY BODIES;

APPENDIX; BARRIER; CELLS

AB The human gut microbiota is a complex, dynamic, and highly diverse community of microorganisms. Beginning as early as in utero fetal development and continuing through birth to late-stage adulthood, the crosstalk between the gut microbiome and brain is essential for modulating various metabolic, neurodevelopmental, and immune-related pathways. Conversely, microbial dysbiosis - defined as alterations in richness and relative abundances - of the gut is implicated in the pathogenesis of several chronic neurological and neurodegenerative disorders. Evidence from large-population cohort studies suggests that individuals with neurodegenerative conditions have an altered gut microbial composition as well as microbial and serum metabolomic profiles distinct from those in the healthy population. Dysbiosis is also linked to psychiatric and gastrointestinal complications - comorbidities often associated with the prodromal phase of Parkinson's disease (PD) and Alzheimer's disease (AD). Studies have identified potential mediators that link gut dysbiosis and neurological disorders. Recent findings have also elucidated the potential mechanisms of disease pathology in the enteric nervous system prior to the onset of neurodegeneration. This review highlights the functional pathways and mechanisms, particularly gut microbe-induced chronic inflammation, protein misfolding, propagation of disease-specific pathology, defective protein clearance, and autoimmune dysregulation, linking gut microbial dysbiosis and neurodegeneration. In addition, we also discuss how pathogenic transformation of microbial composition leads to increased endotoxin production and fewer beneficial metabolites, both of which could trigger immune cell activation and enteric neuronal dysfunction. These can further disrupt intestinal barrier permeability, aggravate the systemic pro-inflammatory state, impair blood-brain barrier permeability and recruit immune mediators leading to neuroinflammation and neurodegeneration. Continued biomedical advances in understanding the microbiota-gut-brain axis will extend the frontier of neurodegenerative disorders and enable the utilization of novel diagnostic and therapeutic strategies to mitigate the pathological burden of these diseases.

C1 [Padhi, Piyush; Worth, Carter; Kanthasamy, Anumantha G.] Iowa State Univ, Dept Biomed Sci, Parkinsons Disorder Res Lab, Ames, IA 50011 USA.

[Padhi, Piyush; Zenitsky, Gary; Jin, Huajun; Anantharam, Vellareddy; Kanthasamy, Arthi; Kanthasamy, Anumantha G.] Univ Georgia, Dept Physiol & Pharmacol, Ctr Brain Sci & Neurodegenerat Dis, Athens, GA 30602 USA.

[Sambamurti, Kumar] Med Univ South Carolina, Dept Neurosci, Charleston, SC 29425 USA.

C3 Iowa State University; University System of Georgia; University of

Georgia; Medical University of South Carolina

RP Kanthasamy, AG (corresponding author), Iowa State Univ, Dept Biomed Sci, Parkinsons Disorder Res Lab, Ames, IA 50011 USA.; Kanthasamy, AG (corresponding author), Univ Georgia, Dept Physiol & Pharmacol, Ctr Brain Sci & Neurodegenerat Dis, Athens, GA 30602 USA.

EM anumantha.kanthasamy@uga.edu

RI Sambamurti, Kumar/A-1620-2012

OI Padhi, Piyush/0000-0003-0866-5720; Kanthasamy, Arthi/0000-0003-4654-9907

FU NIH [R61NS112441, R01 NS100090, R01 ES026892, R21 AG062378, R01

EB026533]; National Institute of Biomedical Imaging and Bioengineering

[R01EB026533] Funding Source: NIH RePORTER

FX This work was funded by NIH R61NS112441, R01 NS100090,R01 ES026892, R21

AG062378, and R01 EB026533

CR Aho VTE, 2021, MOL NEURODEGENER, V16, DOI 10.1186/s13024-021-00427-6

ALBERT MJ, 1980, NATURE, V283, P781, DOI 10.1038/283781a0

Alkasir R, 2017, PROTEIN CELL, V8, P90, DOI 10.1007/s13238-016-0338-6

Amorim Neto D.P., 2021, AKKERMANSIA MUCINIPH, DOI [10.1101/2021.02.12.430931, DOI 10.1101/2021.02.12.430931]

An GY, 2007, J EXP MED, V204, P1417, DOI 10.1084/jem.20061929

Arumugam M, 2011, NATURE, V473, P174, DOI 10.1038/nature09944

Atashrazm F, 2018, SCI REP-UK, V8, DOI 10.1038/s41598-018-33921-x

Bäckhed F, 2005, SCIENCE, V307, P1915, DOI 10.1126/science.1104816

Baert F, 2021, NPJ PARKINSONS DIS, V7, DOI 10.1038/s41531-021-00215-5

Basurto-Islas G, 2018, J ALZHEIMERS DIS, V63, P821, DOI 10.3233/JAD-170715

Baxter NT, 2019, MBIO, V10, DOI [10.1128/mBio.02566-18, 10.1128/mbio.02566-18]

Bhattacharyya D, 2019, ACS CHEM NEUROSCI, V10, P2229, DOI 10.1021/acschemneuro.8b00733

Bhattarai Y, 2021, GUT MICROBES, V13, DOI 10.1080/19490976.2020.1866974

Bieri G, 2019, ACTA NEUROPATHOL, V137, P961, DOI 10.1007/s00401-019-01995-0

Boleij A, 2021, COMMUN BIOL, V4, DOI 10.1038/s42003-021-02014-3

Bollinger RR, 2007, J THEOR BIOL, V249, P826, DOI 10.1016/j.jtbi.2007.08.032

Bosco N, 2021, GENES IMMUN, V22, P289, DOI 10.1038/s41435-021-00126-8

Braak H, 2006, NEUROSCI LETT, V396, P67, DOI 10.1016/j.neulet.2005.11.012

Braniste V, 2014, SCI TRANSL MED, V6, DOI 10.1126/scitranslmed.3009759

BRITT RP, 1970, BRIT J HAEMATOL, V18, P637, DOI 10.1111/j.1365-2141.1970.tb01588.x

BRITT RP, 1976, LANCET, V2, P799

BRITT RP, 1971, Q J MED, V40, P499

Brown GC, 2019, J NEUROINFLAMM, V16, DOI 10.1186/s12974-019-1564-7

Burgaletto C, 2020, J NEUROINFLAMM, V17, DOI 10.1186/s12974-020-01968-1

Cabrera C, 2021, CURR ALZHEIMER RES, V18, P993, DOI 10.2174/1567205018666211215152411

Cannon T, 2020, GUT MICROBES, V12, P1, DOI 10.1080/19490976.2020.1830694

Cao HL, 2017, SCI REP-UK, V7, DOI 10.1038/s41598-017-10835-8

Cao YY, 2021, GASTROENTEROLOGY, V161, P1552, DOI 10.1053/j.gastro.2021.08.003

Cattaneo A, 2017, NEUROBIOL AGING, V49, P60, DOI 10.1016/j.neurobiolaging.2016.08.019

Chalazonitis A, 2018, BRAIN RES, V1693, P207, DOI 10.1016/j.brainres.2018.01.011

Challis C, 2020, NAT NEUROSCI, V23, P327, DOI 10.1038/s41593-020-0589-7

Chaudhuri KR, 2006, LANCET NEUROL, V5, P235, DOI 10.1016/S1474-4422(06)70373-8

Chen C, 2020, SCI ADV, V6, DOI 10.1126/sciadv.aba0466

Chen YH, 2015, MOL MED REP, V12, P6105, DOI 10.3892/mmr.2015.4136

Chen ZC, 2018, PROG NEUROBIOL, V169, P1, DOI 10.1016/j.pneurobio.2018.08.002

Chopra N, 2021, MOL PSYCHIATR, V26, P5636, DOI 10.1038/s41380-019-0610-2

Claesson MJ, 2009, PLOS ONE, V4, DOI 10.1371/journal.pone.0006669

Collins J, 2014, NEUROGASTROENT MOTIL, V26, P98, DOI 10.1111/nmo.12236

Costea PI, 2018, NAT MICROBIOL, V3, DOI 10.1038/s41564-017-0072-8

De Giorgio R, 2004, GASTROENTEROLOGY, V126, P1872, DOI 10.1053/j.gastro.2004.02.024

De Schepper S, 2018, CELL, V175, P400, DOI 10.1016/j.cell.2018.07.048

De Vedder F, 2018, P NATL ACAD SCI USA, V115, P6458, DOI 10.1073/pnas.1720017115

Delungahawatta T, 2017, FRONT NEUROSCI-SWITZ, V11, DOI 10.3389/fnins.2017.00588

Desai MS, 2016, CELL, V167, P1339, DOI 10.1016/j.cell.2016.10.043

Dinan TG, 2017, J PHYSIOL-LONDON, V595, P489, DOI 10.1113/JP273106

Dodiya HB, 2020, NEUROBIOL DIS, V135, DOI 10.1016/j.nbd.2018.12.012

Erny D, 2015, NAT NEUROSCI, V18, P965, DOI 10.1038/nn.4030

Erturk-Hasdemir D, 2019, P NATL ACAD SCI USA, V116, P26157, DOI 10.1073/pnas.1915978116

Fang P, 2020, CELL HOST MICROBE, V28, P201, DOI 10.1016/j.chom.2020.06.008

Farkas SA, 2005, EUR SURG RES, V37, P115, DOI 10.1159/000084543

Fasano A, 2015, LANCET NEUROL, V14, P625, DOI 10.1016/S1474-4422(15)00007-1

Fellner L, 2011, ACTA NEUROPATHOL, V121, P675, DOI 10.1007/s00401-011-0833-z

Flügel A, 2001, IMMUNITY, V14, P547, DOI 10.1016/S1074-7613(01)00143-1

Franceschi C, 2018, NAT REV ENDOCRINOL, V14, P576, DOI 10.1038/s41574-018-0059-4

Gabanyi I, 2016, CELL, V164, P378, DOI 10.1016/j.cell.2015.12.023

Galiano-Landeira J, 2020, BRAIN, V143, P3717, DOI 10.1093/brain/awaa269

Ge P, 2020, MOL NEURODEGENER, V15, DOI 10.1186/s13024-020-00367-7

Geirnaert A, 2014, ANAEROBE, V30, P70, DOI 10.1016/j.anaerobe.2014.08.010

Gershon M., 2019, The second brain: a groundbreaking new understanding of nervous disorders of the stomach and intestine

Geuking MB, 2020, MUCOSAL IMMUNOL, V13, P855, DOI 10.1038/s41385-020-00335-w

Gill SR, 2006, SCIENCE, V312, P1355, DOI 10.1126/science.1124234

Gordevicius J, 2021, NAT COMMUN, V12, DOI 10.1038/s41467-021-25474-x

Goya ME, 2020, CELL REP, V30, P367, DOI 10.1016/j.celrep.2019.12.078

Gray MT, 2014, MOVEMENT DISORD, V29, P991, DOI 10.1002/mds.25779

Grün D, 2020, PARKINSONISM RELAT D, V70, P20, DOI 10.1016/j.parkreldis.2019.11.020

Guinane CM, 2013, MBIO, V4, DOI 10.1128/mBio.00366-12

Gury-BenAri M, 2016, CELL, V166, P1231, DOI 10.1016/j.cell.2016.07.043

Hammond TR, 2019, IMMUNITY, V50, P955, DOI 10.1016/j.immuni.2019.03.016

Harach T, 2017, SCI REP-UK, V7, DOI 10.1038/srep46856

He XF, 2021, J NEUROINFLAMM, V18, DOI 10.1186/s12974-021-02199-8

Hill-Burns EM, 2017, MOVEMENT DISORD, V32, P739, DOI 10.1002/mds.26942

Hilton D, 2014, ACTA NEUROPATHOL, V127, P235, DOI 10.1007/s00401-013-1214-6

Ho L, 2019, J NUTR BIOCHEM, V64, P170, DOI 10.1016/j.jnutbio.2018.10.019

Houser MC, 2021, ACTA NEUROPATHOL COM, V9, DOI 10.1186/s40478-021-01240-4

Huang TT, 2021, NEUROREPORT, V32, P244, DOI 10.1097/WNR.0000000000001585

Hung LY, 2020, AM J PHYSIOL-GASTR L, V318, pG1042, DOI 10.1152/ajpgi.00088.2020

Ihekweazu FD, 2019, GUT MICROBES, V10, P504, DOI 10.1080/19490976.2018.1560753

Im GY, 2011, CLIN GASTROENTEROL H, V9, P1072, DOI 10.1016/j.cgh.2011.06.006

Inlender T, 2021, SCI REP-UK, V11, DOI 10.1038/s41598-021-92384-9

Ivanova MI, 2021, BIOPHYS CHEM, V269, DOI 10.1016/j.bpc.2020.106507

Jansma J, 2021, MICROBIOME, V9, DOI 10.1186/s40168-020-00955-1

Jarret A, 2020, CELL, V180, P50, DOI 10.1016/j.cell.2019.12.016

Kabouridis PS, 2015, NEURON, V85, P289, DOI 10.1016/j.neuron.2014.12.037

Kanthasamy A, 2019, PHARMACOL THERAPEUT, V197, P61, DOI 10.1016/j.pharmthera.2019.01.001

Killinger BA, 2018, SCI TRANSL MED, V10, DOI 10.1126/scitranslmed.aar5280

Kim GH, 2022, J CROHNS COLITIS, V16, P436, DOI 10.1093/ecco-jcc/jjab162

Kim S, 2019, NEURON, V103, P627, DOI 10.1016/j.neuron.2019.05.035

Kimono D, 2019, FRONT PHYSIOL, V10, DOI 10.3389/fphys.2019.01229

Koch TR, 2005, MIL MED, V170, P696, DOI 10.7205/MILMED.170.8.696

Kooij IA, 2016, CLIN EXP IMMUNOL, V186, P1, DOI 10.1111/cei.12821

Kowalski K, 2019, J NEUROGASTROENTEROL, V25, P48, DOI 10.5056/jnm18087

Kuan WL, 2016, EXP NEUROL, V285, P72, DOI 10.1016/j.expneurol.2016.09.003

KUPSKY WJ, 1987, NEUROLOGY, V37, P1253, DOI 10.1212/WNL.37.7.1253

Lahiri DK, 2009, MOL PSYCHIATR, V14, P992, DOI 10.1038/mp.2009.82

Laval L, 2015, GUT MICROBES, V6, P1, DOI 10.4161/19490976.2014.990784

Lawton M, 2018, J NEUROL NEUROSUR PS, V89, P1279, DOI 10.1136/jnnp-2018-318337

Lee HS, 2021, GUT, V70, P408, DOI 10.1136/gutjnl-2020-322429

Lee JW, 2008, J NEUROINFLAMM, V5, DOI 10.1186/1742-2094-5-37

Lee JK, 2011, J NEUROSCI, V31, P11879, DOI 10.1523/JNEUROSCI.1002-11.2011

Lee J, 2021, J MICROBIOL BIOTECHN, V31, P705, DOI 10.4014/jmb.2103.03029

Lei ZN, 2019, CELL DEATH DIS, V10, DOI 10.1038/s41419-019-1364-0

Li BY, 2019, ALZHEIMERS DEMENT, V15, P1357, DOI 10.1016/j.jalz.2019.07.002

Li MH, 2020, MOL MED, V26, DOI 10.1186/s10020-020-00234-7

Ling ZX, 2021, FRONT CELL DEV BIOL, V8, DOI 10.3389/fcell.2020.634069

Lippai R, 2021, SCI REP-UK, V11, DOI 10.1038/s41598-021-93671-1

Liu BJ, 2021, NPJ PARKINSONS DIS, V7, DOI 10.1038/s41531-020-00145-8

Liu P, 2019, BRAIN BEHAV IMMUN, V80, P633, DOI 10.1016/j.bbi.2019.05.008

Lopez-Siles M, 2016, INFLAMM BOWEL DIS, V22, P28, DOI 10.1097/MIB.0000000000000590

Lukiw W.J., 2021, J. Alzheimers Dis. Park, V11, P525, DOI DOI 10.4172/2161-0460.1000525

Lukiw WJ, 2016, FRONT MICROBIOL, V7, DOI 10.3389/fmicb.2016.01544

Ma QQ, 2019, J NEUROINFLAMM, V16, DOI 10.1186/s12974-019-1434-3

Maloney B, 2016, LANCET NEUROL, V15, P760, DOI 10.1016/S1474-4422(16)00065-X

Marizzoni M, 2020, J ALZHEIMERS DIS, V78, P683, DOI 10.3233/JAD-200306

Martin R, 2016, PLOS ONE, V11, DOI 10.1371/journal.pone.0158498

Martínez I, 2018, ELIFE, V7, DOI 10.7554/eLife.36521

Masahata K, 2014, NAT COMMUN, V5, DOI 10.1038/ncomms4704

MATHAN VI, 1974, J CLIN INVEST, V54, P598, DOI 10.1172/JCI107797

Matheis F, 2020, CELL, V180, P64, DOI 10.1016/j.cell.2019.12.002

Matheoud D, 2019, NATURE, V571, P565, DOI 10.1038/s41586-019-1405-y

Matsumoto M, 2008, BIOSCI BIOTECH BIOCH, V72, P572, DOI 10.1271/bbb.70474

Mazzulli JR, 2011, CELL, V146, P37, DOI 10.1016/j.cell.2011.06.001

McManus RM, 2017, ALZHEIMERS RES THER, V9, DOI 10.1186/s13195-017-0241-2

McMenamin CA, 2018, NEUROSCIENCE, V393, P369, DOI 10.1016/j.neuroscience.2018.09.033

Metidji A, 2018, IMMUNITY, V49, P353, DOI 10.1016/j.immuni.2018.07.010

Miao ZH, 2021, BMC MICROBIOL, V21, DOI 10.1186/s12866-021-02263-6

Minato T, 2017, PLOS ONE, V12, DOI 10.1371/journal.pone.0187307

Minter MR, 2016, SCI REP-UK, V6, DOI 10.1038/srep30028

Mitchell CM, 2020, CELL REP MED, V1, DOI 10.1016/j.xcrm.2020.100156

Mizoguchi A, 1996, J EXP MED, V184, P707, DOI 10.1084/jem.184.2.707

Möhle L, 2016, CELL REP, V15, P1945, DOI 10.1016/j.celrep.2016.04.074

Monaco A, 2020, FRONT MOL NEUROSCI, V13, DOI 10.3389/fnmol.2020.00037

Morrison DJ, 2016, GUT MICROBES, V7, P189, DOI 10.1080/19490976.2015.1134082

Muller PA, 2020, SCIENCE, V370, P314, DOI 10.1126/science.abd6176

Muller PA, 2020, NATURE, V583, P441, DOI 10.1038/s41586-020-2474-7

Nara PL, 2021, J ALZHEIMERS DIS, V82, P1417, DOI 10.3233/JAD-210448

Natale G, 2021, LIFE-BASEL, V11, DOI 10.3390/life11080732

Nishiwaki H, 2020, MOVEMENT DISORD, V35, P1626, DOI 10.1002/mds.28119

Obata Y, 2020, NATURE, V578, P284, DOI 10.1038/s41586-020-1975-8

Venegas DP, 2019, FRONT IMMUNOL, V10, DOI 10.3389/fimmu.2019.00277

Park S, 2019, J CLIN MED, V8, DOI 10.3390/jcm8081191

Peladic NJ, 2021, NUTRIENTS, V13, DOI 10.3390/nu13092919

Pellegrini C, 2018, ACTA NEUROPATHOL, V136, P345, DOI 10.1007/s00401-018-1856-5

Peniche AG, 2018, ANAEROBE, V54, P83, DOI 10.1016/j.anaerobe.2018.07.011

Peter I, 2018, JAMA NEUROL, V75, P939, DOI 10.1001/jamaneurol.2018.0605

Peterson J, 2009, GENOME RES, V19, P2317, DOI 10.1101/gr.096651.109

Pickard JM, 2017, IMMUNOL REV, V279, P70, DOI 10.1111/imr.12567

Ríos JP, 2019, BMJ OPEN, V9, DOI 10.1136/bmjopen-2018-025574

Plotegher N, 2019, FRONT NEUROSCI-SWITZ, V13, DOI 10.3389/fnins.2019.00330

Powell N, 2017, NAT REV GASTRO HEPAT, V14, P143, DOI 10.1038/nrgastro.2016.191

Qian Yiwei, 2020, Brain, V143, P2474, DOI 10.1093/brain/awaa201

Qin LY, 2007, GLIA, V55, P453, DOI 10.1002/glia.20467

Quinn PMJ, 2020, ACTA NEUROPATHOL COM, V8, DOI 10.1186/s40478-020-01062-w

Ramakrishna C, 2019, NAT COMMUN, V10, DOI 10.1038/s41467-019-09884-6

Rao M, 2016, NAT REV GASTRO HEPAT, V13, DOI 10.1038/nrgastro.2016.107

ROBERTS PD, 1973, BMJ-BRIT MED J, V3, P67, DOI 10.1136/bmj.3.5871.67

Romano S, 2021, NPJ PARKINSONS DIS, V7, DOI 10.1038/s41531-021-00156-z

Rosario D, 2021, CELL REP, V34, DOI 10.1016/j.celrep.2021.108807

Rossi E, 2018, CRIT REV MICROBIOL, V44, P1, DOI 10.1080/1040841X.2017.1303660

Rothhammer V, 2019, NAT REV IMMUNOL, V19, P184, DOI 10.1038/s41577-019-0125-8

Rothhammer V, 2018, NATURE, V557, P724, DOI 10.1038/s41586-018-0119-x

Rothhammer V, 2016, NAT MED, V22, P586, DOI 10.1038/nm.4106

Sahami S, 2016, AM J GASTROENTEROL, V111, P163, DOI 10.1038/ajg.2015.301

Saji N, 2020, SCI REP-UK, V10, DOI 10.1038/s41598-020-65196-6

Sampson TR, 2020, ELIFE, V9, DOI 10.7554/eLife.53111

Sampson TR, 2016, CELL, V167, P1469, DOI 10.1016/j.cell.2016.11.018

Sánchez-Ferro A, 2015, MOVEMENT DISORD, V30, P517, DOI 10.1002/mds.25988

Sanmarco LM, 2021, NATURE, V590, P473, DOI 10.1038/s41586-020-03116-4

Schäfer KH, 1999, J INTERF CYTOK RES, V19, P527, DOI 10.1089/107999099313974

Scheld M, 2016, J NEUROSCI, V36, P1410, DOI 10.1523/JNEUROSCI.2456-15.2016

Scheperjans F, 2015, MOVEMENT DISORD, V30, P350, DOI 10.1002/mds.26069

Sender R, 2016, PLOS BIOL, V14, DOI 10.1371/journal.pbio.1002533

Seppi K, 2011, MOVEMENT DISORD, V26, pS42, DOI 10.1002/mds.23884

Shannon KM, 2012, MOVEMENT DISORD, V27, P709, DOI 10.1002/mds.23838

Sittipo P, 2018, FRONT MICROBIOL, V9, DOI 10.3389/fmicb.2018.01588

Soto M, 2018, MOL PSYCHIATR, V23, P2287, DOI 10.1038/s41380-018-0086-5

STEWART JS, 1970, LANCET, V2, P542

Stinson LF, 2019, FRONT MICROBIOL, V10, DOI 10.3389/fmicb.2019.01124

Suez J, 2019, NAT MED, V25, P716, DOI 10.1038/s41591-019-0439-x

Sun MF, 2018, BRAIN BEHAV IMMUN, V70, P48, DOI 10.1016/j.bbi.2018.02.005

Takagawa T, 2018, SCI TRANSL MED, V10, DOI 10.1126/scitranslmed.aan8162

Tan AH, 2021, JGH OPEN, V5, P414, DOI 10.1002/jgh3.12450

Tarallo S, 2022, GUT, V71, P1302, DOI 10.1136/gutjnl-2021-325168

Tierney BT, 2019, CELL HOST MICROBE, V26, P283, DOI 10.1016/j.chom.2019.07.008

Tükel Ç, 2009, CELL HOST MICROBE, V6, P45, DOI 10.1016/j.chom.2009.05.020

Vacca M, 2020, MICROORGANISMS, V8, DOI 10.3390/microorganisms8040573

Van Den Berge N, 2019, ACTA NEUROPATHOL, V138, P535, DOI 10.1007/s00401-019-02040-w

van IJzendoorn SCD, 2019, J PARKINSON DIS, V9, pS323, DOI 10.3233/JPD-191707

Vascellari S, 2021, BIOMOLECULES, V11, DOI 10.3390/biom11020144

Vogt NM, 2017, SCI REP-UK, V7, DOI 10.1038/s41598-017-13601-y

WAKABAYASHI K, 1988, ACTA NEUROPATHOL, V76, P217, DOI 10.1007/BF00687767

Wallen ZD, 2020, NPJ PARKINSONS DIS, V6, DOI 10.1038/s41531-020-0112-6

Wang CY, 2021, P NATL ACAD SCI USA, V118, DOI 10.1073/pnas.2106504118

Wang HL, 2018, P NATL ACAD SCI USA, V115, pE12427, DOI 10.1073/pnas.1815915115

Wang RX, 2020, P NATL ACAD SCI USA, V117, P11648, DOI 10.1073/pnas.1917597117

Warner BB, 2019, PEDIATR RES, V85, P216, DOI 10.1038/s41390-018-0191-9

Weersma RK, 2020, GUT, V69, P1510, DOI 10.1136/gutjnl-2019-320204

Wei GZ, 2021, P NATL ACAD SCI USA, V118, DOI 10.1073/pnas.2021091118

Wekerle H, 2017, TRENDS IMMUNOL, V38, P483, DOI 10.1016/j.it.2017.03.008

Wu SC, 2017, NAT COMMUN, V8, DOI 10.1038/s41467-017-00040-6

Xie WJ, 2020, NEUROTOXICOLOGY, V76, P200, DOI 10.1016/j.neuro.2019.11.009

Xu CM, 2019, BMC MICROBIOL, V19, DOI 10.1186/s12866-019-1616-2

Yan X, 2020, FRONT NEUROSCI-SWITZ, V14, DOI 10.3389/fnins.2020.530219

Yan YP, 2021, NPJ BIOFILMS MICROBI, V7, DOI 10.1038/s41522-021-00242-3

Yang R, 2019, PHYSIOL GENOMICS, V51, P368, DOI 10.1152/physiolgenomics.00026.2019

Zhang B, 2021, GUT, V70, P85, DOI 10.1136/gutjnl-2020-320789

Zhang J, 2020, J BIOL CHEM, V295, P4237, DOI 10.1074/jbc.RA119.010143

Zhao YH, 2018, FRONT NEUROL, V9, DOI 10.3389/fneur.2018.00145

Zhao YH, 2017, FRONT CELL INFECT MI, V7, DOI 10.3389/fcimb.2017.00318

Zheng DP, 2020, CELL RES, V30, P492, DOI 10.1038/s41422-020-0332-7

Zheng QY, 2016, FRONT AGING NEUROSCI, V8, DOI 10.3389/fnagi.2016.00303

Zheng WX, 2020, BRAIN BEHAV IMMUN, V87, P890, DOI 10.1016/j.bbi.2020.01.009

NR 211

TC 12

Z9 12

U1 3

U2 22

PU FRONTIERS MEDIA SA

PI LAUSANNE

PA AVENUE DU TRIBUNAL FEDERAL 34, LAUSANNE, CH-1015, SWITZERLAND

EI 1662-453X

J9 FRONT NEUROSCI-SWITZ

JI Front. Neurosci.

PD FEB 25

PY 2022

VL 16

AR 836605

DI 10.3389/fnins.2022.836605

PG 19

WC Neurosciences

WE Science Citation Index Expanded (SCI-EXPANDED)

SC Neurosciences & Neurology

GA ZV8MQ

UT WOS:000770778300001

PM 35281490

OA gold, Green Published

DA 2024-08-12

ER

PT J

AU Pizarro-Galleguillos, BM

Kunert, L

Brüggemann, N

Prasuhn, J

AF Pizarro-Galleguillos, Benjamin Matis

Kunert, Liesa

Brueggemann, Norbert

Prasuhn, Jannik

TI Neuroinflammation and Mitochondrial Dysfunction in Parkinson's Disease:

Connecting Neuroimaging with Pathophysiology

SO ANTIOXIDANTS

LA English

DT Review

DE Parkinson's disease; neuroinflammation; mitochondrial dysfunction;

magnetic resonance imaging (MRI); magnetic resonance spectroscopy

imaging (MRSI); positron emission tomography (PET) imaging; TSPO;

mitochondria; neuroimaging

ID BRAIN-BARRIER DISRUPTION; FREE-WATER; SUBSTANTIA-NIGRA; RAT MODEL;

IMAGING NEUROINFLAMMATION; DIFFUSION KURTOSIS; OXIDATIVE STRESS; PET;

MRI; ACCUMULATION

AB There is a pressing need for disease-modifying therapies in patients suffering from neurodegenerative disease, including Parkinson's disease (PD). However, these disorders face unique challenges in clinical trial designs to assess the neuroprotective properties of potential drug candidates. One of these challenges relates to the often unknown individual disease mechanisms that would, however, be relevant for targeted treatment strategies. Neuroinflammation and mitochondrial dysfunction are two proposed pathophysiological hallmarks and are considered to be highly interconnected in PD. Innovative neuroimaging methods can potentially help to gain deeper insights into one's predominant disease mechanisms, can facilitate patient stratification in clinical trials, and could potentially map treatment responses. This review aims to highlight the role of neuroinflammation and mitochondrial dysfunction in patients with PD (PwPD). We will specifically introduce different neuroimaging modalities, their respective technical hurdles and challenges, and their implementation into clinical practice. We will gather preliminary evidence for their potential use in PD research and discuss opportunities for future clinical trials.

C1 [Pizarro-Galleguillos, Benjamin Matis] Univ Chile, Fac Med, Santiago 8380453, Chile.

[Pizarro-Galleguillos, Benjamin Matis; Kunert, Liesa; Brueggemann, Norbert; Prasuhn, Jannik] Univ Med Ctr Schleswig Holstein, Dept Neurol, Campus Lubeck, D-23562 Lubeck, Germany.

[Pizarro-Galleguillos, Benjamin Matis; Kunert, Liesa; Prasuhn, Jannik] Univ Lubeck, Inst Neurogenet, D-23562 Lubeck, Germany.

[Pizarro-Galleguillos, Benjamin Matis; Kunert, Liesa; Brueggemann, Norbert; Prasuhn, Jannik] Univ Lubeck, Ctr Brain Behav & Metab, D-23562 Lubeck, Germany.

[Prasuhn, Jannik] Johns Hopkins Univ, Russell H Morgan Dept Radiol & Radiol Sci, Sch Med, Baltimore, MD 21205 USA.

[Prasuhn, Jannik] Kennedy Krieger Inst, FM Kirby Res Ctr Funct Brain Imaging, Baltimore, MD 21287 USA.

C3 Universidad de Chile; University of Kiel; Schleswig Holstein University

Hospital; University of Lubeck; University of Lubeck; Johns Hopkins

University; Kennedy Krieger Institute

RP Brüggemann, N (corresponding author), Univ Med Ctr Schleswig Holstein, Dept Neurol, Campus Lubeck, D-23562 Lubeck, Germany.; Brüggemann, N (corresponding author), Univ Lubeck, Ctr Brain Behav & Metab, D-23562 Lubeck, Germany.

EM benjaminpizarro@ug.uchile.cl; liesa.kunert@neuro.uni-luebeck.de;

norbert.brueggemann@uni-luebeck.de; jannik.prasuhn@neuro.uni-luebeck.de

OI Prasuhn, Jannik/0000-0003-4184-9409; Bruggemann,

Norbert/0000-0001-5969-6899

FU Global Parkinson's Genetics Program (GP2); Aligning Science Across

Parkinson's (ASAP); Deutsche Forschungsgemeinschaft [BR4328.2-1

[FOR2488], GRK1957, DFG-GEPRIS 413535489]; Michael J. Fox Foundation; EU

Joint Programme-Neurodegenerative Disease Research (JPND); Parkinson's

Foundation; Thiemann Stiftung

FX B.M.P.-G. was supported by the Global Parkinson's Genetics Program

(GP2). GP2 is funded by the Aligning Science Across Parkinson's (ASAP)

initiative and implemented by The Michael J. Fox Foundation for

Parkinson's Research. For a complete list of GP2 members see

https://gp2.org/. He also receeived the Chilean National ANID Ph.D.

scholarship (2020). N.B. received funding from the Deutsche

Forschungsgemeinschaft (BR4328.2-1 [FOR2488], GRK1957), the Michael J.

Fox Foundation, and the EU Joint Programme-Neurodegenerative Disease

Research (JPND). J.P. received funding from the Parkinson's Foundation,

the Deutsche Parkinsongesellschaft, the Thiemann Stiftung, and the

Deutsche Forschungsgemeinschaft via the Clinician Scientist School

Luebeck (DFG-GEPRIS 413535489).

CR Ajalin RM, 2022, MOVEMENT DISORD, V37, P1673, DOI 10.1002/mds.29117

Al-Bachari S, 2020, FRONT PHYSIOL, V11, DOI 10.3389/fphys.2020.593026

Alam MM, 2017, NUCL MED MOLEC IMAG, V51, P283, DOI 10.1007/s13139-017-0475-8

Andica C, 2020, J NEUROSCI RES, V98, P936, DOI 10.1002/jnr.24584

Andica C, 2019, CELLS-BASEL, V8, DOI 10.3390/cells8080839

Andica C, 2018, PARKINSONISM RELAT D, V51, P55, DOI 10.1016/j.parkreldis.2018.02.046

Arab A, 2019, NEUROTOX RES, V36, P724, DOI 10.1007/s12640-019-00068-0

Arpin DJ, 2022, MOVEMENT DISORD, V37, P325, DOI 10.1002/mds.28838

Arribarat G, 2020, FRONT NEUROL, V11, DOI 10.3389/fneur.2020.00791

Arribarat G, 2020, CURR OPIN NEUROL, V33, P222, DOI 10.1097/WCO.0000000000000796

Bange M, 2022, J PARKINSON DIS, V12, P1575, DOI 10.3233/JPD-223225

Bartels AL, 2008, PARKINSONISM RELAT D, V14, P505, DOI 10.1016/j.parkreldis.2007.11.007

Beamer E, 2016, NEUROPHARMACOLOGY, V104, P94, DOI 10.1016/j.neuropharm.2015.09.019

BENSON P F, 1961, Cereb Palsy Bull, V3, P510

Berkowitz BA, 2018, J MAGN RESON, V291, P94, DOI 10.1016/j.jmr.2018.01.013

Bloem BR, 2021, LANCET, V397, P2284, DOI 10.1016/S0140-6736(21)00218-X

Borsche M, 2020, BRAIN, V143, P3041, DOI 10.1093/brain/awaa246

Bradl M, 2009, SEMIN IMMUNOPATHOL, V31, P455, DOI 10.1007/s00281-009-0182-3

Brakedal B, 2022, CELL METAB, V34, P396, DOI 10.1016/j.cmet.2022.02.001

Burciu RG, 2017, BRAIN, V140, P2183, DOI 10.1093/brain/awx146

Burciu RG, 2016, HUM BRAIN MAPP, V37, P2894, DOI 10.1002/hbm.23213

Cassano T, 2017, FRONT NEUROSCI-SWITZ, V11, DOI 10.3389/fnins.2017.00030

Casteels C, 2010, BRAIN RES, V1316, P153, DOI 10.1016/j.brainres.2009.12.026

Ceccarini J, 2019, EUR J NUCL MED MOL I, V46, P2348, DOI 10.1007/s00259-019-04445-x

Chen C, 2022, WIRES NANOMED NANOBI, V14, DOI 10.1002/wnan.1740

Choi IY, 2021, NMR BIOMED, V34, DOI 10.1002/nbm.4411

Chung JW, 2017, NEUROIMAGE-CLIN, V14, P417, DOI 10.1016/j.nicl.2017.02.012

Clifford B, 2020, IEEE T BIO-MED ENG, V67, P2745, DOI 10.1109/TBME.2020.2969892

Coles LD, 2018, J CLIN PHARMACOL, V58, P158, DOI 10.1002/jcph.1008

Corot C, 2006, ADV DRUG DELIVER REV, V58, P1471, DOI 10.1016/j.addr.2006.09.013

Cova I, 2018, J NEURAL TRANSM, V125, P1417, DOI 10.1007/s00702-018-1910-4

Ding XB, 2021, NAT MED, V27, P411, DOI 10.1038/s41591-020-01198-1

Elschot EP, 2021, INVEST RADIOL, V56, P10, DOI 10.1097/RLI.0000000000000723

Esposito G, 2008, J NUCL MED, V49, P1414, DOI 10.2967/jnumed.107.049619

Ezura M, 2021, FRONT AGING NEUROSCI, V13, DOI 10.3389/fnagi.2021.761010

Farsad M, 2020, CURR RADIOPHARM, V13, P195, DOI [10.2174/1874471013666191223153755, 10.2174/1874471013669191223153755]

Febo M, 2020, BRAIN STRUCT FUNCT, V225, P427, DOI 10.1007/s00429-019-02017-1

Filippi M, 2011, RADIOLOGY, V259, P659, DOI 10.1148/radiol.11101362

Fujita K, 2021, J NEUROL, V268, P2246, DOI 10.1007/s00415-021-10411-1

Gao XA, 2011, NEUROLOGY, V76, P863, DOI 10.1212/WNL.0b013e31820f2d79

Gao ZY, 2016, SMALL, V12, P556, DOI 10.1002/smll.201502309

Gianferrara T, 2022, CURR MED CHEM, V29, P4631, DOI 10.2174/0929867329666220216113517

Gómez-Gálvez Y, 2016, PROG NEURO-PSYCHOPH, V64, P200, DOI 10.1016/j.pnpbp.2015.03.017

Gordon Y, 2014, CARDIOVASC DIAGN THE, V4, P147, DOI 10.3978/j.issn.2223-3652.2014.03.01

Guttuso T, 2018, AM J NEURORADIOL, V39, P479, DOI 10.3174/ajnr.A5545

Harada R, 2022, FRONT NEUROSCI-SWITZ, V16, DOI 10.3389/fnins.2022.807435

HAWKINS CP, 1990, BRAIN, V113, P365, DOI 10.1093/brain/113.2.365

Heye AK, 2014, NEUROIMAGE-CLIN, V6, P262, DOI 10.1016/j.nicl.2014.09.002

Ikawa M, 2020, ANTIOXIDANTS-BASEL, V9, DOI 10.3390/antiox9090861

Ikawa M, 2011, NUCL MED BIOL, V38, P945, DOI 10.1016/j.nucmedbio.2011.02.016

Ishibashi K, 2018, NEUROPHARMACOLOGY, V143, P106, DOI 10.1016/j.neuropharm.2018.09.036

Jain P, 2020, J NUCL MED, V61, P1107, DOI 10.2967/jnumed.119.229443

Javadov S, 2021, J MOL MED, V99, P57, DOI 10.1007/s00109-020-02004-8

Jensen JH, 2010, NMR BIOMED, V23, P698, DOI 10.1002/nbm.1518

Jin JN, 2023, J NEUROL, V270, P2451, DOI 10.1007/s00415-022-11556-3

Jucaite A, 2015, BRAIN, V138, P2687, DOI 10.1093/brain/awv184

Kamagata K, 2021, INT J MOL SCI, V22, DOI 10.3390/ijms22105216

Kamagata K, 2016, EUR RADIOL, V26, P2567, DOI 10.1007/s00330-015-4066-8

Kamagata K, 2016, EXPERT REV NEUROTHER, V16, P241, DOI 10.1586/14737175.2016.1142876

Kamiya K, 2020, J NEUROSCI METH, V346, DOI 10.1016/j.jneumeth.2020.108908

Karayel O, 2022, CELL REP MED, V3, DOI 10.1016/j.xcrm.2022.100661

Khairnar A, 2016, J NEUROCHEM, V136, P1259, DOI 10.1111/jnc.13500

Kim SY, 2017, SCHIZOPHRENIA BULL, V43, P197, DOI 10.1093/schbul/sbw129

Konícková D, 2022, BIOMEDICINES, V10, DOI 10.3390/biomedicines10071760

Kreisl WC, 2020, LANCET NEUROL, V19, P940, DOI 10.1016/S1474-4422(20)30346-X

Kwon EH, 2022, BIOMOLECULES, V12, DOI 10.3390/biom12020329

Li JD, 2019, CURR TOP MED CHEM, V19, P1445, DOI 10.2174/1568026619666190708163407

Li JW, 2022, BRAIN SCI, V12, DOI 10.3390/brainsci12070851

Liu H, 2019, J NUCL MED, V60

Liu TW, 2022, INT J MOL SCI, V23, DOI 10.3390/ijms23084148

Liu Y, 2022, EUR J NUCL MED MOL I, V49, P1275, DOI 10.1007/s00259-021-05640-5

Loehrer PA, 2022, BRAIN COMMUN, V4, DOI 10.1093/braincomms/fcac137

Masdeu JC, 2022, J NUCL MED, V63, p45S, DOI 10.2967/jnumed.121.263200

Meldolesi J, 2021, BIOMEDICINES, V9, DOI 10.3390/biomedicines9030252

Mishina M, 2007, SYNAPSE, V61, P778, DOI 10.1002/syn.20423

Mishina M, 2017, SYNAPSE, V71, DOI 10.1002/syn.21979

Mishina M, 2011, PLOS ONE, V6, DOI 10.1371/journal.pone.0017338

Mitchell T, 2019, HUM BRAIN MAPP, V40, P5094, DOI 10.1002/hbm.24760

Miyazaki I, 2020, CELLS-BASEL, V9, DOI 10.3390/cells9122623

Nag S, 2009, ACTA NEUROPATHOL, V118, P197, DOI 10.1007/s00401-009-0541-0

Narayanaswami V, 2018, MOL IMAGING, V17, DOI 10.1177/1536012118792317

Oestreich LKL, 2022, BIOL PSYCHIAT-COGN N, V7, P638, DOI 10.1016/j.bpsc.2022.01.003

Ofori E, 2017, MOVEMENT DISORD, V32, P1457, DOI 10.1002/mds.27100

Ofori E, 2015, BRAIN, V138, P2322, DOI 10.1093/brain/awv136

Ofori E, 2015, NEUROBIOL AGING, V36, P1097, DOI 10.1016/j.neurobiolaging.2014.10.029

Ogawa T, 2021, PARKINSONISM RELAT D, V90, P8, DOI 10.1016/j.parkreldis.2021.07.021

Olmedo-Díaz S, 2017, NEUROSCIENCE, V362, P141, DOI 10.1016/j.neuroscience.2017.08.023

Pagano G, 2016, CLIN MED, V16, P371, DOI 10.7861/clinmedicine.16-4-371

Pasternak O, 2009, MAGN RESON MED, V62, P717, DOI 10.1002/mrm.22055

Picca A, 2020, ANTIOXIDANTS-BASEL, V9, DOI 10.3390/antiox9080647

Pizarro-Galleguillos BM, 2022, INT J MOL SCI, V23, DOI 10.3390/ijms232213678

Prabhakaran J, 2021, MOLECULES, V26, DOI 10.3390/molecules26113208

Praet J, 2018, ALZHEIMERS RES THER, V10, DOI 10.1186/s13195-017-0329-8

Prasuhn J, 2022, PARKINSONISM RELAT D, V99, P91, DOI 10.1016/j.parkreldis.2022.05.008

Prasuhn J, 2022, NEUROLOGY, V99, pE2683, DOI 10.1212/WNL.0000000000201288

Prasuhn J, 2022, INT J MOL SCI, V23, DOI 10.3390/ijms23137263

Prasuhn J, 2022, MOL MED, V28, DOI 10.1186/s10020-021-00426-9

Prasuhn J, 2021, GENES-BASEL, V12, DOI 10.3390/genes12111840

Prasuhn J, 2021, MOL MED, V27, DOI 10.1186/s10020-021-00281-8

Prasuhn J, 2021, FRONT NEUROL, V11, DOI 10.3389/fneur.2020.592104

Prasuhn J, 2020, NEUROL RES PRACT, V2, DOI 10.1186/s42466-020-00092-y

Prasuhn J, 2019, NEUROL RES PRACT, V1, DOI 10.1186/s42466-019-0033-1

Quinn PMJ, 2020, ACTA NEUROPATHOL COM, V8, DOI 10.1186/s40478-020-01062-w

Ramlackhansingh AF, 2011, NEUROLOGY, V76, P1811, DOI 10.1212/WNL.0b013e31821ccce4

Rango M, 2020, MOVEMENT DISORD, V35, P504, DOI 10.1002/mds.27946

Rehnmark A, 2012, J EXP NEUROSCI, V6, P21, DOI 10.4137/JEN.S10424

Roussakis AA, 2018, INT REV NEUROBIOL, V141, P347, DOI 10.1016/bs.irn.2018.08.009

Sacco S, 2020, AM J NEURORADIOL, V41, P2219, DOI 10.3174/ajnr.A6862

Saeed U, 2017, TRANSL NEURODEGENER, V6, DOI 10.1186/s40035-017-0076-6

Saito S, 2019, RADIOL PHYS TECHNOL, V12, P46, DOI 10.1007/s12194-018-0490-1

Santoro A, 2016, EUR J PHARM SCI, V88, P124, DOI 10.1016/j.ejps.2016.04.006

Schönecker S, 2019, FRONT AGING NEUROSCI, V11, DOI 10.3389/fnagi.2019.00249

Schröder S, 2020, MOLECULES, V25, DOI 10.3390/molecules25071633

Schulz JB, 2000, EUR J BIOCHEM, V267, P4904, DOI 10.1046/j.1432-1327.2000.01595.x

Shukla AK, 2006, J MED PHYS, V31, P13, DOI 10.4103/0971-6203.25665

Sliter DA, 2018, NATURE, V561, P258, DOI 10.1038/s41586-018-0448-9

Sone D, 2020, ALZH DEMENT-DADM, V12, DOI 10.1002/dad2.12125

Szczepankiewicz F, 2016, NEUROIMAGE, V142, P522, DOI 10.1016/j.neuroimage.2016.07.038

Tan YY, 2022, J PARKINSON DIS, V12, P477, DOI 10.3233/JPD-212976

Tansey MG, 2022, NAT REV IMMUNOL, V22, P657, DOI 10.1038/s41577-022-00684-6

Terpstra M, 2006, MAGN RESON MED, V55, P979, DOI 10.1002/mrm.20854

Tong JC, 2017, BRAIN, V140, P2460, DOI 10.1093/brain/awx172

Tourdias T, 2013, NEUROTHERAPEUTICS, V10, P111, DOI 10.1007/s13311-012-0155-4

Virel A, 2015, EUR J NEUROSCI, V42, P2761, DOI 10.1111/ejn.13044

Vuorimaa A, 2017, CONTRAST MEDIA MOL I, DOI 10.1155/2017/6975841

Waggan I, 2023, J NEUROL, V270, P300, DOI 10.1007/s00415-022-11342-1

Waubant E, 2006, DIS MARKERS, V22, P235, DOI 10.1155/2006/709869

Weiduschat N, 2015, J NEUROIMAGING, V25, P105, DOI 10.1111/jon.12074

Weinstein JS, 2010, J CEREBR BLOOD F MET, V30, P15, DOI 10.1038/jcbfm.2009.192

Werry EL, 2019, INT J MOL SCI, V20, DOI 10.3390/ijms20133161

Wilson H, 2020, MOVEMENT DISORD, V35, P1416, DOI 10.1002/mds.28064

Wilson H, 2019, BRAIN, V142, P3116, DOI 10.1093/brain/awz260

Witte ME, 2010, MITOCHONDRION, V10, P411, DOI 10.1016/j.mito.2010.05.014

Xu ZY, 2018, INT REV NEUROBIOL, V142, P3, DOI 10.1016/bs.irn.2018.09.001

Yang J, 2019, PARKINSONISM RELAT D, V62, P10, DOI 10.1016/j.parkreldis.2019.01.007

Yi SY, 2019, FRONT NEUROSCI-SWITZ, V13, DOI 10.3389/fnins.2019.00081

Zeng LY, 2018, CURR MED CHEM, V25, P2970, DOI 10.2174/0929867324666170314124616

Zhang LL, 2021, ACTA PHARM SIN B, V11, P373, DOI 10.1016/j.apsb.2020.08.006

Zhang PF, 2022, J NEUROL, V269, P2304, DOI 10.1007/s00415-021-10877-z

Zhuo JC, 2012, NEUROIMAGE, V59, P467, DOI 10.1016/j.neuroimage.2011.07.050

NR 140

TC 4

Z9 4

U1 2

U2 8

PU MDPI

PI BASEL

PA ST ALBAN-ANLAGE 66, CH-4052 BASEL, SWITZERLAND

EI 2076-3921

J9 ANTIOXIDANTS-BASEL

JI Antioxidants

PD JUL

PY 2023

VL 12

IS 7

AR 1411

DI 10.3390/antiox12071411

PG 19

WC Biochemistry & Molecular Biology; Chemistry, Medicinal; Food Science &

Technology

WE Science Citation Index Expanded (SCI-EXPANDED)

SC Biochemistry & Molecular Biology; Pharmacology & Pharmacy; Food Science

& Technology

GA N6DZ9

UT WOS:001037908800001

PM 37507950

OA gold, Green Published

DA 2024-08-12

ER

PT J

AU Marchetti, B

Tirolo, C

L'Episcopo, F

Caniglia, S

Testa, N

Smith, JA

Pluchino, S

Serapide, MF

AF Marchetti, Bianca

Tirolo, Cataldo

L'Episcopo, Francesca

Caniglia, Salvatore

Testa, Nunzio

Smith, Jayden A.

Pluchino, Stefano

Serapide, Maria F.

TI Parkinson's disease, aging and adult neurogenesis: Wnt/β-catenin

signalling as the key to unlock the mystery of endogenous brain repair

SO AGING CELL

LA English

DT Review

DE adult neurogenesis; aging; neuroinflammation; Parkinson's disease;

plasticity; self-repair; Wnt; beta-catenin signalling

ID NEURAL STEM-CELLS; TRANSCRIPTION FACTOR NRF2; CENTRAL-NERVOUS-SYSTEM;

MPTP MOUSE MODEL; SUBVENTRICULAR ZONE; HIPPOCAMPAL NEUROGENESIS;

PROGENITOR CELLS; BETA-CATENIN; DOPAMINERGIC-NEURONS; OXIDATIVE STRESS

AB A common hallmark of age-dependent neurodegenerative disease is an impairment of adult neurogenesis. Wingless-type mouse mammary tumor virus integration site (Wnt)/beta-catenin (W beta C) signalling is a vital pathway for dopaminergic (DAergic) neurogenesis and an essential signalling system during embryonic development and aging, the most critical risk factor for Parkinson's disease (PD). To date, there is no known cause or cure for PD. Here we focus on the potential to reawaken the impaired neurogenic niches to rejuvenate and repair the aged PD brain. Specifically, we highlight W beta C-signalling in the plasticity of the subventricular zone (SVZ), the largest germinal region in the mature brain innervated by nigrostriatal DAergic terminals, and the mesencephalic aqueduct-periventricular region (Aq-PVR) Wnt-sensitive niche, which is in proximity to the SNpc and harbors neural stem progenitor cells (NSCs) with DAergic potential. The hallmark of the W beta C pathway is the cytosolic accumulation of beta-catenin, which enters the nucleus and associates with T cell factor/lymphoid enhancer binding factor (TCF/LEF) transcription factors, leading to the transcription of Wnt target genes. Here, we underscore the dynamic interplay between DAergic innervation and astroglial-derived factors regulating W beta C-dependent transcription of key genes orchestrating NSC proliferation, survival, migration and differentiation. Aging, inflammation and oxidative stress synergize with neurotoxin exposure in "turning off" the W beta C neurogenic switch via down-regulation of the nuclear factor erythroid-2-related factor 2/Wnt-regulated signalosome, a key player in the maintenance of antioxidant self-defense mechanisms and NSC homeostasis. Harnessing W beta C-signalling in the aged PD brain can thus restore neurogenesis, rejuvenate the microenvironment, and promote neurorescue and regeneration.

C1 [Marchetti, Bianca; Serapide, Maria F.] Univ Catania, Med Sch, Dept Biomed & Biotechnol Sci BIOMETEC, Pharmacol Sect, Via S Sofia 65, I-95125 Catania, Italy.

[Marchetti, Bianca; Serapide, Maria F.] Univ Catania, Med Sch, Dept Biomed & Biotechnol Sci BIOMETEC, Physiol Sect, Catania, Italy.

[Marchetti, Bianca; Tirolo, Cataldo; L'Episcopo, Francesca; Caniglia, Salvatore; Testa, Nunzio] IRCCS, Neuropharmacol Sect, OASI Res Inst, Via Conte Ruggero 73, I-94018 Troina, EN, Italy.

[Smith, Jayden A.; Pluchino, Stefano] Univ Cambridge, Dept Clin Neurosci, Cambridge, United Kingdom.

[Smith, Jayden A.; Pluchino, Stefano] Univ Cambridge, NIHR Biomed Res Ctr, Cambridge, United Kingdom.

C3 University of Catania; University of Catania; University of Cambridge;

University of Cambridge

RP Marchetti, B (corresponding author), Univ Catania, Med Sch, Dept Biomed & Biotechnol Sci BIOMETEC, Pharmacol Sect, Via S Sofia 65, I-95125 Catania, Italy.; Marchetti, B (corresponding author), IRCCS, Neuropharmacol Sect, OASI Res Inst, Via Conte Ruggero 73, I-94018 Troina, EN, Italy.

EM biancamarchetti@libero.it

RI Caniglia, Salvatore/D-1001-2017; Tirolo, Cataldo/C-9929-2017; Marchetti,

Bianca Maria/K-4623-2016; L'Episcopo, Francesca/K-4602-2016; Nunzio,

Testa/D-8023-2017

OI Caniglia, Salvatore/0000-0002-9416-8458; Tirolo,

Cataldo/0000-0003-1747-8134; Marchetti, Bianca

Maria/0000-0002-9287-8448; L'Episcopo, Francesca/0000-0003-3292-9677;

Nunzio, Testa/0000-0002-4024-2325; SERAPIDE, MARIA

FRANCESCA/0000-0001-6332-4772; Pluchino, Stefano/0000-0002-6267-9472;

Smith, Jayden/0000-0003-2307-8452

FU Italian Ministry of Health (Ricerca Corrente-2011-2019); Italian

Ministry of Education, University and Research (MIUR),

PRIN/CHANCE-Project-2015-2019; OASI Research Institute-IRCCS, Troina,

Italy; OASI (IRCCS),Troina (EN) Italy; BIOMETEC, at the University of

Catania, Medical School, Catania, Italy

FX This research program has received support from different funding

agencies, in particular the Italian Ministry of Health (Ricerca

Corrente-2011-2019) the Italian Ministry of Education, University and

Research (MIUR), PRIN/CHANCE-Project-2015-2019; the OASI Research

Institute-IRCCS, Troina, Italy OASI (IRCCS),Troina (EN) Italy and

BIOMETEC, at the University of Catania, Medical School, Catania, Italy.

CR Abdalkader M, 2018, FRONT NEUROSCI-SWITZ, V12, DOI 10.3389/fnins.2018.00466

Adachi K, 2007, STEM CELLS, V25, P2827, DOI 10.1634/stemcells.2007-0177

Agnihotri SK, 2019, BEHAV BRAIN RES, V363, P161, DOI 10.1016/j.bbr.2019.02.006

Ahlenius H, 2009, J NEUROSCI, V29, P4408, DOI 10.1523/JNEUROSCI.6003-08.2009

Alvarez-Buylla A, 2004, NEURON, V41, P683, DOI 10.1016/S0896-6273(04)00111-4

Alvarez-Buylla A, 2001, NAT REV NEUROSCI, V2, P287, DOI 10.1038/35067582

Anacker C, 2017, NAT REV NEUROSCI, V18, P335, DOI 10.1038/nrn.2017.45

Anbrosone A, 2016, ACS NANO, V10, P4828, DOI 10.1021/acsnano.5b07817

Anderegg Angela, 2015, Neurogenesis (Austin), V2, pe998101, DOI 10.1080/23262133.2014.998101

Anderegg A, 2013, PLOS GENET, V9, DOI 10.1371/journal.pgen.1003973

Apple DM, 2017, BIOCHEM PHARMACOL, V141, P77, DOI 10.1016/j.bcp.2017.06.116

Arenas E, 2014, J MOL CELL BIOL, V6, P42, DOI 10.1093/jmcb/mju001

Arrázola MS, 2015, FRONT CELL NEUROSCI, V9, DOI 10.3389/fncel.2015.00166

Ashmawy AM, 2017, ARAB J GASTROENTEROL, V18, P144, DOI 10.1016/j.ajg.2017.09.001

Awad O, 2017, STEM CELL REP, V9, P1853, DOI 10.1016/j.stemcr.2017.10.029

Azim K, 2014, GLIA, V62, P778, DOI 10.1002/glia.22641

Baker SA, 2004, EUR J NEUROSCI, V20, P575, DOI 10.1111/j.1460-9568.2004.03486.x

Banerjee A, 2019, ENDOCR METAB IMMUNE, V19, P233, DOI 10.2174/1871530319666190118103907

Bänziger C, 2006, CELL, V125, P509, DOI 10.1016/j.cell.2006.02.049

Barkers RA, 2018, NATURE, V557, P329, DOI 10.1038/s41586-018-0087-1

Barkho BZ, 2006, STEM CELLS DEV, V15, P407, DOI 10.1089/scd.2006.15.407

Bayod S, 2015, NEUROBIOL AGING, V36, P720, DOI 10.1016/j.neurobiolaging.2014.09.017

Beckervordersandforth Ruth, 2017, Brain Plast, V3, P73, DOI 10.3233/BPL-170044

Beckervordersandforth R, 2017, NEURON, V93, P560, DOI 10.1016/j.neuron.2016.12.017

Bédard A, 2002, NEUROSCI LETT, V328, P213, DOI 10.1016/S0304-3940(02)00530-X

Bergmann O, 2015, CSH PERSPECT BIOL, V7, DOI 10.1101/cshperspect.a018994

Berwick DC, 2017, MOL NEURODEGENER, V12, DOI 10.1186/s13024-017-0153-4

Berwick DC, 2014, J MOL CELL BIOL, V6, P3, DOI 10.1093/jmcb/mjt037

Beurel E, 2015, PHARMACOL THERAPEUT, V148, P114, DOI 10.1016/j.pharmthera.2014.11.016

Bezard E, 1998, PROG NEUROBIOL, V55, P93, DOI 10.1016/S0301-0082(98)00006-9

Bhat R, 2003, J BIOL CHEM, V278, P45937, DOI 10.1074/jbc.M306268200

Bitar MS, 2011, AM J PHYSIOL-ENDOC M, V301, pE1119, DOI 10.1152/ajpendo.00047.2011

Blauwendraat C, 2019, MOVEMENT DISORD, V34, P866, DOI 10.1002/mds.27659

Bodine PVN, 2009, BONE, V44, P1063, DOI 10.1016/j.bone.2009.02.013

Boger HA, 2010, PROG NEUROBIOL, V90, P217, DOI 10.1016/j.pneurobio.2009.10.013

Bonaguidi MA, 2011, CELL, V145, P1142, DOI 10.1016/j.cell.2011.05.024

Bond AM, 2015, CELL STEM CELL, V17, P385, DOI 10.1016/j.stem.2015.09.003

Bonilla S, 2008, GLIA, V56, P809, DOI 10.1002/glia.20654

Borta A, 2007, J NEUROCHEM, V100, P587, DOI 10.1111/j.1471-4159.2006.04241.x

Briona LK, 2015, DEV BIOL, V403, P15, DOI 10.1016/j.ydbio.2015.03.025

Brodski C, 2019, J DEV BIOL, V7, DOI 10.3390/jdb7010003

Butovsky O, 2006, MOL CELL NEUROSCI, V31, P149, DOI 10.1016/j.mcn.2005.10.006

Cannon JR, 2013, NEUROBIOL DIS, V57, P38, DOI 10.1016/j.nbd.2012.06.025

Carmon KS, 2011, P NATL ACAD SCI USA, V108, P11452, DOI 10.1073/pnas.1106083108

Castelo-Branco G, 2004, J CELL SCI, V117, P5731, DOI 10.1242/jcs.01505

Castelo-Branco GA, 2006, MOL CELL NEUROSCI, V31, P251, DOI 10.1016/j.mcn.2005.09.014

Chandel NS, 2016, NAT CELL BIOL, V18, P823, DOI 10.1038/ncb3385

Chen DZ, 2019, BIOORG CHEM, V84, P285, DOI 10.1016/j.bioorg.2018.11.020

Chen PC, 2009, P NATL ACAD SCI USA, V106, P2933, DOI 10.1073/pnas.0813361106

Chen XM, 2018, FREE RADICAL BIO MED, V117, P158, DOI 10.1016/j.freeradbiomed.2018.02.011

Chinta SJ, 2013, J INTERN MED, V273, P429, DOI 10.1111/joim.12029

Chinta SJ, 2018, CELL REP, V22, P930, DOI 10.1016/j.celrep.2017.12.092

Chiu WH, 2015, NEUROPHARMACOLOGY, V95, P367, DOI 10.1016/j.neuropharm.2015.03.020

Chmielarz P, 2017, CELL DEATH DIS, V8, DOI 10.1038/cddis.2017.214

Cho CH, 2019, AGING CELL, V18, DOI 10.1111/acel.12899

Chong ZZ, 2010, OXID MED CELL LONGEV, V3, P153, DOI 10.4161/oxim.3.2.11758

Ciani L, 2005, NAT REV NEUROSCI, V6, P351, DOI 10.1038/nrn1665

Clément-Lacroix P, 2005, P NATL ACAD SCI USA, V102, P17406, DOI 10.1073/pnas.0505259102

Coghlan MP, 2000, CHEM BIOL, V7, P793, DOI 10.1016/S1074-5521(00)00025-9

Collier TJ, 2007, NEUROBIOL DIS, V26, P56, DOI 10.1016/j.nbd.2006.11.013

Cooper RJ, 2019, NEUROTOXICOLOGY, V71, P102, DOI 10.1016/j.neuro.2018.12.010

Corenblum MJ, 2016, AGING CELL, V15, P725, DOI 10.1111/acel.12482

Cruciat CM, 2013, CSH PERSPECT BIOL, V5, DOI 10.1101/cshperspect.a015081

Das NR, 2014, CURR NEUROVASC RES, V11, P114, DOI 10.2174/1567202611666140318114037

Dayer AG, 2005, J CELL BIOL, V168, P415, DOI 10.1083/jcb.200407053

Daynac M, 2016, SCI REP-UK, V6, DOI 10.1038/srep21505

de la Fuente-Fernández R, 2011, ANN NEUROL, V69, P803, DOI 10.1002/ana.22284

de Lau W, 2014, GENE DEV, V28, P305, DOI 10.1101/gad.235473.113

Desplats P, 2012, J BIOL CHEM, V287, P31691, DOI 10.1074/jbc.M112.354522

Ding S, 2003, P NATL ACAD SCI USA, V100, P7632, DOI 10.1073/pnas.0732087100

Doetsch F, 1999, CELL, V97, P703, DOI 10.1016/S0092-8674(00)80783-7

Doetsch F, 1997, J NEUROSCI, V17, P5046

Donega V, 2019, ACTA NEUROPATHOL COM, V7, DOI 10.1186/s40478-019-0736-0

Dorsey ER, 2018, JAMA NEUROL, V75, P9, DOI 10.1001/jamaneurol.2017.3299

Driehuis E, 2017, BRIT J PHARMACOL, V174, P4547, DOI 10.1111/bph.13758

Dzamko N, 2015, NEUROSCIENCE, V302, P89, DOI 10.1016/j.neuroscience.2014.10.028

Ehninger D, 2011, CELL TISSUE RES, V345, P69, DOI 10.1007/s00441-011-1200-z

Ekdahl CT, 2009, NEUROSCIENCE, V158, P1021, DOI 10.1016/j.neuroscience.2008.06.052

Encinas JM, 2011, CELL STEM CELL, V8, P566, DOI 10.1016/j.stem.2011.03.010

Enwere E, 2004, J NEUROSCI, V24, P8354, DOI 10.1523/JNEUROSCI.2751-04.2004

Eriksson PS, 1998, NAT MED, V4, P1313, DOI 10.1038/3305

Ermine CM, 2018, HIPPOCAMPUS, V28, P327, DOI 10.1002/hipo.22835

Ernst A, 2014, CELL, V156, P1072, DOI 10.1016/j.cell.2014.01.044

Esfandiari F, 2012, STEM CELLS DEV, V21, P3233, DOI 10.1089/scd.2011.0678

Farzanehfar P, 2018, NEUROSCI RES, V134, P1, DOI 10.1016/j.neures.2018.01.002

Freundlieb N, 2006, J NEUROSCI, V26, P2321, DOI 10.1523/JNEUROSCI.4859-05.2006

Fuentealba LC, 2012, CELL STEM CELL, V10, P698, DOI 10.1016/j.stem.2012.05.012

Gage FH, 2000, SCIENCE, V287, P1433, DOI 10.1126/science.287.5457.1433

Galceran J, 2000, DEVELOPMENT, V127, P469

Gammons MV, 2016, MOL CELL, V64, P92, DOI 10.1016/j.molcel.2016.08.026

Gao HM, 2011, ENVIRON HEALTH PERSP, V119, P807, DOI 10.1289/ehp.1003013

Garcia AL, 2018, NEURAL REGEN RES, V13, P43, DOI 10.4103/1673-5374.224359

García-Velázquez L, 2017, AGEING RES REV, V37, P135, DOI 10.1016/j.arr.2017.06.001

Giguère N, 2018, FRONT NEUROL, V9, DOI 10.3389/fneur.2018.00455

Glasl L, 2012, EXP NEUROL, V235, P214, DOI 10.1016/j.expneurol.2012.01.002

Guttuso T, 2019, J NEUROL SCI, V402, P121, DOI 10.1016/j.jns.2019.05.016

Hain EG, 2018, J NEUROINFLAMM, V15, DOI 10.1186/s12974-018-1179-4

Hao HX, 2012, NATURE, V485, P195, DOI 10.1038/nature11019

Hayes JD, 2015, BIOCHEM SOC T, V43, P611, DOI 10.1042/BST20150011

He P, 2009, J NEUROSCI, V29, P6545, DOI 10.1523/JNEUROSCI.0421-09.2009

He XJ, 2006, J NEUROPATH EXP NEUR, V65, P873, DOI 10.1097/01.jnen.0000235115.29440.ce

He XJ, 2008, NEUROTOXICOLOGY, V29, P1141, DOI 10.1016/j.neuro.2008.05.009

Hebsgaard JB, 2009, GLIA, V57, P1648, DOI 10.1002/glia.20877

Hermann A, 2006, STEM CELLS, V24, P949, DOI 10.1634/stemcells.2005-0192

Hermann A, 2008, STEM CELLS, V26, P2749, DOI 10.1634/stemcells.2008-0567

Hermann A, 2009, STEM CELLS, V27, P928, DOI 10.1002/stem.21

Herr P, 2012, DEV BIOL, V361, P392, DOI 10.1016/j.ydbio.2011.11.003

Herrera-Arozamena C, 2016, MOLECULES, V21, DOI 10.3390/molecules21091165

Hindle JV, 2010, AGE AGEING, V39, P156, DOI 10.1093/ageing/afp223

Hirota Y, 2016, NEUROCHEM RES, V41, P222, DOI 10.1007/s11064-015-1766-z

Hirsch EC, 2009, LANCET NEUROL, V8, P382, DOI 10.1016/S1474-4422(09)70062-6

Ho HYH, 2012, P NATL ACAD SCI USA, V109, P4044, DOI 10.1073/pnas.1200421109

Höglinger GU, 2014, J COMP NEUROL, V522, P2336, DOI 10.1002/cne.23537

Hofmann JW, 2014, AGE, V36, P1033, DOI 10.1007/s11357-014-9618-3

Höglinger GU, 2004, NAT NEUROSCI, V7, P726, DOI 10.1038/nn1265

HORNYKIEWICZ O, 1993, ADV NEUROL, V60, P140

Hsieh J, 2012, GENE DEV, V26, P1010, DOI 10.1101/gad.187336.112

Huang HL, 2019, CAN J PHYSIOL PHARM, V97, P15, DOI 10.1139/cjpp-2018-0226

Huang P, 2019, PHARMACOL THERAPEUT, V196, P79, DOI 10.1016/j.pharmthera.2018.11.008

Inestrosa NC, 2010, NAT REV NEUROSCI, V11, P77, DOI 10.1038/nrn2755

Inta D, 2015, TRENDS NEUROSCI, V38, P517, DOI 10.1016/j.tins.2015.07.005

Jakubs K, 2008, J NEUROSCI, V28, P12477, DOI 10.1523/JNEUROSCI.3240-08.2008

Janda CY, 2017, NATURE, V545, P234, DOI 10.1038/nature22306

Janda CY, 2012, SCIENCE, V337, P59, DOI 10.1126/science.1222879

Jang MH, 2013, CELL STEM CELL, V12, P215, DOI 10.1016/j.stem.2012.11.021

Jang Y, 2018, LIFE SCI, V209, P455, DOI 10.1016/j.lfs.2018.08.045

Jang Y, 2018, NEUROSCIENCE, V379, P292, DOI 10.1016/j.neuroscience.2018.03.015

Jankovic J, 2019, MOVEMENT DISORD, V34, P41, DOI 10.1002/mds.27534

Jessberger S, 2009, LEARN MEMORY, V16, P147, DOI 10.1101/lm.1172609

Jho EH, 2002, MOL CELL BIOL, V22, P1172, DOI 10.1128/MCB.22.4.1172-1183.2002

Jiao J, 2008, STEM CELLS, V26, P1221, DOI 10.1634/stemcells.2007-0513

Jin N, 2017, EXP NEUROL, V297, P36, DOI 10.1016/j.expneurol.2017.07.006

Johnson DA, 2015, FREE RADICAL BIO MED, V88, P253, DOI 10.1016/j.freeradbiomed.2015.07.147

Joksimovic M, 2014, J MOL CELL BIOL, V6, P27, DOI 10.1093/jmcb/mjt043

Jope RS, 2017, TRENDS BIOCHEM SCI, V42, P180, DOI 10.1016/j.tibs.2016.10.009

Kahn M, 2014, NAT REV DRUG DISCOV, V13, P513, DOI 10.1038/nrd4233

Kalamakis G, 2019, CELL, V176, P1407, DOI 10.1016/j.cell.2019.01.040

Kalani MYS, 2008, P NATL ACAD SCI USA, V105, P16970, DOI 10.1073/pnas.0808616105

Kang I, 2018, FEBS LETT, V592, P793, DOI 10.1002/1873-3468.12989

Kase Y, 2019, STEM CELL REP, V12, P1313, DOI 10.1016/j.stemcr.2019.04.010

Katsimpardi L, 2018, CURR OPIN NEUROBIOL, V53, P131, DOI 10.1016/j.conb.2018.07.006

Kaur P, 2017, SCI REP-UK, V7, DOI 10.1038/s41598-017-16879-0

Kempermann G, 1998, J NEUROSCI, V18, P3206

Kempermann G, 1997, NATURE, V386, P493, DOI 10.1038/386493a0

Kempermann G, 2018, CELL STEM CELL, V23, P25, DOI 10.1016/j.stem.2018.04.004

Khan MM, 2015, J STEROID BIOCHEM, V146, P38, DOI 10.1016/j.jsbmb.2014.05.001

Kim CH, 2015, P NATL ACAD SCI USA, V112, P8756, DOI 10.1073/pnas.1509742112

Kim JI, 2016, STEM CELL RES, V17, P534, DOI 10.1016/j.scr.2016.09.027

Kirkeby A, 2017, PROG BRAIN RES, V230, P165, DOI 10.1016/bs.pbr.2016.11.011

Kirkeby A, 2012, CELL REPORTS, V1, P703, DOI 10.1016/j.celrep.2012.04.009

Klaissle P, 2012, BMC NEUROSCI, V13, DOI 10.1186/1471-2202-13-132

Klein C, 2016, BEHAV BRAIN RES, V307, P227, DOI 10.1016/j.bbr.2016.02.040

Kodali M, 2018, BRAIN BEHAV IMMUN, V69, P499, DOI 10.1016/j.bbi.2018.01.009

Kodali M, 2016, J NEUROSCI, V36, P8112, DOI 10.1523/JNEUROSCI.0766-16.2016

Kohl Z, 2016, NEUROBIOL DIS, V85, P206, DOI 10.1016/j.nbd.2015.10.021

Kojima T, 2010, STEM CELLS, V28, P545, DOI 10.1002/stem.306

Kriks S, 2011, NATURE, V480, P547, DOI 10.1038/nature10648

L'Episcopo F, 2016, CELL DEATH DIS, V7, DOI 10.1038/cddis.2016.104

L'Episcopo F, 2011, NEUROBIOL DIS, V41, P508, DOI 10.1016/j.nbd.2010.10.023

L'Episcopo F, 2018, STEM CELLS, V36, P1179, DOI 10.1002/stem.2827

L'Episcopo F, 2018, FRONT AGING NEUROSCI, V10, DOI 10.3389/fnagi.2018.00012

L'Episcopo F, 2014, STEM CELLS, V32, P2147, DOI 10.1002/stem.1708

L'Episcopo F, 2014, J MOL CELL BIOL, V6, P13, DOI 10.1093/jmcb/mjt053

L'Episcopo F, 2013, J NEUROSCI, V33, P1462, DOI 10.1523/JNEUROSCI.3206-12.2013

L'Episcopo F, 2012, J NEUROSCI, V32, P2062, DOI 10.1523/JNEUROSCI.5259-11.2012

L'Episcopo F, 2011, MOL NEURODEGENER, V6, DOI 10.1186/1750-1326-6-49

L'Episcopo F, 2011, REJUV RES, V14, P411, DOI 10.1089/rej.2010.1134

L'Episcopo F, 2010, J NEUROINFLAMM, V7, DOI 10.1186/1742-2094-7-83

L'Episcopo F, 2010, CNS NEUROL DISORD-DR, V9, P349, DOI 10.2174/187152710791292639

Langston JW, 2017, J PARKINSON DIS, V7, pS11, DOI 10.3233/JPD-179006

Lao CL, 2013, GLIA, V61, P475, DOI 10.1002/glia.22449

Lastres-Becker I, 2012, HUM MOL GENET, V21, P3173, DOI 10.1093/hmg/dds143

Le Grand JN, 2015, CELL MOL LIFE SCI, V72, P773, DOI 10.1007/s00018-014-1774-1

Lee JA, 2018, NEUROCHEM INT, V112, P96, DOI 10.1016/j.neuint.2017.11.006

Lee SMK, 2000, DEVELOPMENT, V127, P457

Leonard BW, 2009, J COMP NEUROL, V515, P269, DOI 10.1002/cne.22040

Lesemann A, 2012, BRAIN RES, V1457, P51, DOI 10.1016/j.brainres.2012.03.046

Li XR, 2018, BIOMATERIALS, V183, P114, DOI 10.1016/j.biomaterials.2018.08.037

Li XT, 2017, CNS NEUROL DISORD-DR, V16, P828, DOI 10.2174/1871527316666170518170422

Licht-Murava A, 2016, SCI SIGNAL, V9, DOI 10.1126/scisignal.aah7102

Lie DC, 2002, J NEUROSCI, V22, P6639

Lie DC, 2005, NATURE, V437, P1370, DOI 10.1038/nature04108

Lim DA, 2016, CSH PERSPECT BIOL, V8, DOI 10.1101/cshperspect.a018820

Lim J, 2018, ARCH PHARM RES, V41, P943, DOI 10.1007/s12272-018-1063-x

Liu D, 2019, BIOMED PHARMACOTHER, V117, DOI 10.1016/j.biopha.2019.108990

Lledo PM, 2016, CSH PERSPECT BIOL, V8, DOI 10.1101/cshperspect.a018945

Llorens-Bobadilla E, 2015, CELL STEM CELL, V17, P329, DOI 10.1016/j.stem.2015.07.002

Logan CY, 2004, ANNU REV CELL DEV BI, V20, P781, DOI 10.1146/annurev.cellbio.20.010403.113126

Luo J, 2006, AGING CELL, V5, P139, DOI 10.1111/j.1474-9726.2006.00197.x

Luzzati F, 2011, PLOS ONE, V6, DOI 10.1371/journal.pone.0025088

Madhavan L, 2008, STEM CELLS, V26, P254, DOI 10.1634/stemcells.2007-0221

Madhavan L, 2012, EUR J NEUROSCI, V35, P883, DOI 10.1111/j.1460-9568.2012.08019.x

Madhavan L, 2009, J COMP NEUROL, V515, P102, DOI 10.1002/cne.22033

Mahmood S, 2016, J RECEPT SIG TRANSD, V36, P310, DOI 10.3109/10799893.2015.1080273

Maiese K, 2008, PHARMACOL THERAPEUT, V118, P58, DOI 10.1016/j.pharmthera.2008.01.004

Maiese K, 2015, NEURAL REGEN RES, V10, P518, DOI 10.4103/1673-5374.155427

Marchetti B, 2005, ANN NY ACAD SCI, V1057, P296, DOI 10.1196/annals.1356.023

Marchetti B, 2005, BRAIN RES REV, V48, P302, DOI 10.1016/j.brainresrev.2004.12.030

Marchetti B, 2005, TRENDS PHARMACOL SCI, V26, P517, DOI 10.1016/j.tips.2005.08.007

Marchetti B, 2005, BRAIN RES REV, V48, P129, DOI 10.1016/j.brainresrev.2004.12.002

Marchetti B, 2011, ENCY ENV HLTH, V5, P690, DOI DOI 10.1016/B978-0-444-52272-6.00079-9

Marchetti B, 2018, INT J MOL SCI, V19, DOI 10.3390/ijms19123743

Marchetti B, 2013, EUR J NEUROSCI, V37, P1550, DOI 10.1111/ejn.12166

Marchetti B, 2013, TRENDS MOL MED, V19, P144, DOI 10.1016/j.molmed.2012.12.001

Maretto S, 2003, P NATL ACAD SCI USA, V100, P3299, DOI 10.1073/pnas.0434590100

Arzate DM, 2019, NEUROSCIENCE, V408, P308, DOI 10.1016/j.neuroscience.2019.03.042

Marinaro C, 2012, CEREB CORTEX, V22, P2415, DOI 10.1093/cercor/bhr320

Orellana AMM, 2015, AGING-US, V7, P1094, DOI 10.18632/aging.100853

Mastrodonato A, 2018, SCI REP-UK, V8, DOI 10.1038/s41598-017-18676-1

McGeer PL, 2008, MOVEMENT DISORD, V23, P474, DOI 10.1002/mds.21751

Ming GL, 2005, ANNU REV NEUROSCI, V28, P223, DOI 10.1146/annurev.neuro.28.051804.101459

Miranda CJ, 2012, AGING CELL, V11, P542, DOI 10.1111/j.1474-9726.2012.00816.x

Mirzadeh Z, 2008, CELL STEM CELL, V3, P265, DOI 10.1016/j.stem.2008.07.004

Mishra A, 2019, NEUROCHEM INT, V129, DOI 10.1016/j.neuint.2019.104463

Mishra V, 2015, SCI REP-UK, V5, DOI 10.1038/srep17807

Mohana S, 2018, BMC CANCER, V18, DOI 10.1186/s12885-018-5103-1

Momcilovic O, 2014, STEM CELLS DEV, V23, P406, DOI 10.1089/scd.2013.0412

Monje ML, 2003, SCIENCE, V302, P1760, DOI 10.1126/science.1088417

Moore WJ, 2009, J MED CHEM, V52, P105, DOI 10.1021/jm801144h

Morrow CS, 2019, CELL STEM CELL, V24, P353, DOI 10.1016/j.stem.2019.02.011

Moya N, 2014, STEM CELL REP, V3, P1015, DOI 10.1016/j.stemcr.2014.10.004

Narcisi R, 2016, TISSUE ENG PT A, V22, P1264, DOI 10.1089/ten.tea.2016.0081

Nato G, 2015, DEVELOPMENT, V142, P840, DOI 10.1242/dev.116657

Neves J, 2017, CELL STEM CELL, V20, P161, DOI 10.1016/j.stem.2017.01.008

Nguyen M, 2019, TRENDS NEUROSCI, V42, P140, DOI 10.1016/j.tins.2018.11.001

Niclis JC, 2017, STEM CELL TRANSL MED, V6, P937, DOI 10.5966/sctm.2016-0073

Niehrs C, 2012, NAT REV MOL CELL BIO, V13, P767, DOI 10.1038/nrm3470

Nierode GJ, 2019, BIOTECHNOL BIOENG, V116, P168, DOI 10.1002/bit.26839

NUSSE R, 1982, CELL, V31, P99, DOI 10.1016/0092-8674(82)90409-3

Nusse R, 2017, CELL, V169, P985, DOI 10.1016/j.cell.2017.05.016

O'Keeffe GC, 2009, CELL CYCLE, V8, P2888, DOI 10.4161/cc.8.18.9512

O'Keeffe GC, 2009, P NATL ACAD SCI USA, V106, P8754, DOI 10.1073/pnas.0803955106

Obernier K, 2018, CELL STEM CELL, V22, P221, DOI 10.1016/j.stem.2018.01.003

Obeso JA, 2017, MOVEMENT DISORD, V32, P1, DOI 10.1002/mds.26904

Okamoto M, 2011, FASEB J, V25, P3570, DOI 10.1096/fj.11-184697

Olanow CW, 2019, MOVEMENT DISORD, V34, P812, DOI 10.1002/mds.27690

Olanow CW, 2013, ANN NEUROL, V74, P337, DOI 10.1002/ana.24011

Ortiz-Matamoros A, 2013, CURR NEUROPHARMACOL, V11, P465, DOI 10.2174/1570159X11311050001

Osakada F, 2007, J NEUROSCI, V27, P4210, DOI 10.1523/JNEUROSCI.4193-06.2007

Ourednik V, 2009, STEM CELLS, V27, P2846, DOI 10.1002/stem.227

Palomer E, 2019, FRONT CELL NEUROSCI, V13, DOI 10.3389/fncel.2019.00227

Palomera-Avalos V, 2017, MOL NEUROBIOL, V54, P1661, DOI 10.1007/s12035-016-9770-0

Parish CL, 2008, J CLIN INVEST, V118, P149, DOI 10.1172/JCI32273

Parish CL, 2014, J MOL CELL BIOL, V6, P54, DOI 10.1093/jmcb/mjt045

Peng J, 2011, AGING CELL, V10, P255, DOI 10.1111/j.1474-9726.2010.00656.x

Piccin D, 2011, STEM CELLS, V29, P528, DOI 10.1002/stem.589

Pluchino S, 2008, BRAIN, V131, P2564, DOI 10.1093/brain/awn198

Pons-Espinal M, 2017, STEM CELL REP, V8, P1046, DOI 10.1016/j.stemcr.2017.02.012

Prakash N, 2006, J PHYSIOL-LONDON, V575, P403, DOI 10.1113/jphysiol.2006.113464

Qiu CW, 2018, EXP NEUROL, V309, P44, DOI 10.1016/j.expneurol.2018.07.011

Qu QH, 2010, NAT CELL BIOL, V12, P31, DOI 10.1038/ncb2001

Radad K, 2017, CNS NEUROL DISORD-DR, V16, P740, DOI 10.2174/1871527316666170623094728

Raslan AA, 2019, INT J BIOCHEM CELL B, V106, P26, DOI 10.1016/j.biocel.2018.11.005

Rasmussen ML, 2018, GENES-BASEL, V9, DOI 10.3390/genes9020109

Rawal N, 2009, BIOCHEM BIOPH RES CO, V388, P473, DOI 10.1016/j.bbrc.2009.07.014

Ray S, 2018, CELL TRANSPLANT, V27, P589, DOI 10.1177/0963689718774030

Razak S, 2018, BMC CANCER, V18, DOI 10.1186/s12885-018-4959-4

Redmond DE, 2007, P NATL ACAD SCI USA, V104, P12175, DOI 10.1073/pnas.0704091104

Reichmann H, 2016, CURR OPIN NEUROL, V29, P467, DOI 10.1097/WCO.0000000000000348

Riabov V, 2017, ACTA BIOMATER, V53, P389, DOI 10.1016/j.actbio.2017.01.071

Richetin K, 2017, NEUROBIOL DIS, V102, P113, DOI 10.1016/j.nbd.2017.03.002

Ring DB, 2003, DIABETES, V52, P588, DOI 10.2337/diabetes.52.3.588

Rolando C, 2016, CELL STEM CELL, V19, P653, DOI 10.1016/j.stem.2016.07.003

Ryoo IG, 2018, TOXICOL APPL PHARM, V359, P24, DOI 10.1016/j.taap.2018.09.014

Salinas PC, 2012, CSH PERSPECT BIOL, V4, DOI 10.1101/cshperspect.a008003

Salinas PC, 2005, BIOCHEM SOC T, V33, P1295, DOI 10.1042/BST0331295

Salinas PC, 2005, J NEUROBIOL, V64, P435, DOI 10.1002/neu.20159

Salvi R, 2016, FRONT NEUROSCI-SWITZ, V10, DOI 10.3389/fnins.2016.00077

Sato N, 2004, NAT MED, V10, P55, DOI 10.1038/nm979

Schapira AHV, 2017, NAT REV NEUROSCI, V18, P435, DOI 10.1038/nrn.2017.62

Schapira AHV, 2014, LANCET, V384, P545, DOI 10.1016/S0140-6736(14)61010-2

Schulte G, 2005, J NEUROCHEM, V92, P1550, DOI 10.1111/j.1471-4159.2004.03022.x

Seib DRM, 2015, GERONTOLOGY, V61, P327, DOI 10.1159/000368575

Seib DRM, 2013, CELL STEM CELL, V12, P204, DOI 10.1016/j.stem.2012.11.010

Shetty AK, 2018, AGING DIS, V9, P1165, DOI 10.14336/AD.2018.1026

Shetty AK, 2016, STEM CELL TRANSL MED, V5, P1204, DOI 10.5966/sctm.2015-0270

Shibui Y, 2009, NEUROTOXICOLOGY, V30, P1036, DOI 10.1016/j.neuro.2009.07.003

Silva-Vargas V, 2016, CELL STEM CELL, V19, P643, DOI 10.1016/j.stem.2016.06.013

Singh S, 2018, FREE RADICAL BIO MED, V129, P73, DOI 10.1016/j.freeradbiomed.2018.08.033

Singh S, 2018, MOL NEUROBIOL, V55, P6500, DOI 10.1007/s12035-017-0860-4

Singh S, 2016, MOL NEUROBIOL, V53, P4286, DOI 10.1007/s12035-015-9361-5

Sirerol-Piquer M, 2011, HIPPOCAMPUS, V21, P910, DOI 10.1002/hipo.20805

Smith GA, 2015, PLOS ONE, V10, DOI 10.1371/journal.pone.0121072

Song JL, 2015, CELL SIGNAL, V27, P1380, DOI 10.1016/j.cellsig.2015.03.018

Song SH, 2002, NATURE, V417, P39, DOI 10.1038/417039a

Song W, 2006, J CELL PHYSIOL, V206, P655, DOI 10.1002/jcp.20509

Sousa-Victor P, 2018, FRONT PHYSIOL, V9, DOI 10.3389/fphys.2018.01629

Spalding KL, 2013, CELL, V153, P1219, DOI 10.1016/j.cell.2013.05.002

Srivastava NS, 2019, PHYTOMEDICINE, V52, P117, DOI 10.1016/j.phymed.2018.09.224

Sung YH, 2015, J PHYS THER SCI, V27, P3203, DOI 10.1589/jpts.27.3203

Surh YJ, 2009, ARCH PHARM RES, V32, P1163, DOI 10.1007/s12272-009-1807-8

Surmeier DJ, 2018, FEBS J, V285, P3657, DOI 10.1111/febs.14607

Suzuki K, 2010, PLOS ONE, V5, DOI 10.1371/journal.pone.0009260

Takei Y, 2019, HUM CELL, V32, P88, DOI 10.1007/s13577-019-00241-9

Tang MZ, 2010, J NEUROSCI, V30, P9280, DOI 10.1523/JNEUROSCI.0860-10.2010

Tang MZ, 2009, DEVELOPMENT, V136, P2027, DOI 10.1242/dev.034330

Tapia-Rojas C, 2018, NEURAL REGEN RES, V13, P1705, DOI 10.4103/1673-5374.238606

Tatar C, 2013, GLIA, V61, P192, DOI 10.1002/glia.22426

Tauc H, 2019, CELL STEM CELL, V24, P499, DOI 10.1016/j.stem.2019.03.008

Tebay LE, 2015, FREE RADICAL BIO MED, V88, P108, DOI 10.1016/j.freeradbiomed.2015.06.021

Tepavcevic V, 2011, J CLIN INVEST, V121, P4722, DOI 10.1172/JCI59145

Thored P, 2009, GLIA, V57, P835, DOI 10.1002/glia.20810

Titova N, 2017, INT REV NEUROBIOL, V132, P33, DOI 10.1016/bs.irn.2017.02.016

Tiwari SK, 2019, MOL NEUROBIOL, V56, P6660, DOI 10.1007/s12035-019-01685-8

Tiwari SK, 2015, MOL NEUROBIOL, V52, P1735, DOI 10.1007/s12035-014-8940-1

Tiwari SK, 2014, ACS NANO, V8, P76, DOI 10.1021/nn405077y

Toledo C, 2019, AGING-US, V11, P5924, DOI 10.18632/aging.102150

Toledo EM, 2017, BRIT J PHARMACOL, V174, P4716, DOI 10.1111/bph.13871

Tom S, 2019, MOL NEUROBIOL, V56, P7851, DOI 10.1007/s12035-019-1636-9

Torres VI, 2019, PHARMACOL THERAPEUT, V198, P34, DOI 10.1016/j.pharmthera.2019.02.009

van den Berge SA, 2013, EUR J PHARMACOL, V719, P117, DOI 10.1016/j.ejphar.2013.04.058

van den Berge SA, 2011, BRAIN, V134, P3249, DOI 10.1093/brain/awr256

van den Berge SA, 2010, AGING CELL, V9, P313, DOI 10.1111/j.1474-9726.2010.00556.x

Van Kampen JM, 2006, J NEUROSCI, V26, P7272, DOI 10.1523/JNEUROSCI.0837-06.2006

Van Kampen JM, 2004, EUR J NEUROSCI, V19, P2377, DOI 10.1111/j.0953-816X.2004.03342.x

van Praag H, 2005, J NEUROSCI, V25, P8680, DOI 10.1523/JNEUROSCI.1731-05.2005

Varela-Nallar L, 2015, NEURAL PLAST, V2015, DOI 10.1155/2015/935403

Varela-Nallar L, 2013, FRONT CELL NEUROSCI, V7, DOI 10.3389/fncel.2013.00100

Veena J, 2011, J Nat Sci Biol Med, V2, P26, DOI 10.4103/0976-9668.82312

Villeda SA, 2011, NATURE, V477, P90, DOI 10.1038/nature10357

Vukovic J, 2012, J NEUROSCI, V32, P6435, DOI 10.1523/JNEUROSCI.5925-11.2012

Wallenquist U, 2012, RESTOR NEUROL NEUROS, V30, P9, DOI 10.3233/RNN-2011-0606

Walter J, 2019, STEM CELL REP, V12, P878, DOI 10.1016/j.stemcr.2019.03.004

Wang KZQ, 2014, MITOCHONDRION, V17, P132, DOI 10.1016/j.mito.2014.04.008

Wang LL, 2017, INT J DEV NEUROSCI, V57, P77, DOI 10.1016/j.ijdevneu.2017.01.002

Wang SS, 2012, BRAIN RES, V1464, P61, DOI 10.1016/j.brainres.2012.04.039

Wang S, 2013, NAT CHEM BIOL, V9, P579, DOI [10.1038/NCHEMBIO.1309, 10.1038/nchembio.1309]

Wang WY, 2007, NEUROPHARMACOLOGY, V52, P1678, DOI 10.1016/j.neuropharm.2007.03.017

Wang YL, 2017, CELL PHYSIOL BIOCHEM, V43, P2226, DOI 10.1159/000484302

Warrier S, 2016, INT J BIOCHEM CELL B, V75, P104, DOI 10.1016/j.biocel.2016.04.002

Wen YA, 2019, CELL DEATH DIFFER, V26, P1955, DOI 10.1038/s41418-018-0265-6

Wenker SD, 2020, GENE THER, V27, P6, DOI 10.1038/s41434-019-0077-4

Wexler EM, 2009, STEM CELLS, V27, P1130, DOI 10.1002/stem.36

Winner B, 2015, CSH PERSPECT BIOL, V7, DOI 10.1101/cshperspect.a021287

Winner B, 2012, J NEUROSCI, V32, P16906, DOI 10.1523/JNEUROSCI.2723-12.2012

Winner B, 2011, EUR J NEUROSCI, V33, P1139, DOI 10.1111/j.1460-9568.2011.07613.x

Winner B, 2009, EXP NEUROL, V219, P543, DOI 10.1016/j.expneurol.2009.07.013

Winner Beate, 2009, Ther Adv Neurol Disord, V2, P51, DOI 10.1177/1756285608100324

Worlitzer MMA, 2012, STEM CELL RES THER, V3, DOI 10.1186/scrt124

Wurst W, 2014, J MOL CELL BIOL, V6, P34, DOI 10.1093/jmcb/mjt046

Xia Tian-Guang, 2019, Zhongguo Ying Yong Sheng Li Xue Za Zhi, V35, P256, DOI 10.12047/j.cjap.5731.2019.054

Xie MQ, 2017, NEUROSCIENCE, V352, P64, DOI 10.1016/j.neuroscience.2017.03.045

Yamashita T, 2006, J NEUROSCI, V26, P6627, DOI 10.1523/JNEUROSCI.0149-06.2006

Yang F, 2014, NAT COMMUN, V5, DOI 10.1038/ncomms6627

Yang JW, 2016, GROWTH FACTORS, V34, P19, DOI 10.3109/08977194.2016.1157791

Zeng QH, 2019, FRONT AGING NEUROSCI, V11, DOI 10.3389/fnagi.2019.00062

Zhang BQ, 2016, EVID-BASED COMPL ALT, V2016, DOI 10.1155/2016/9753189

Zhang H, 2018, MECH DEVELOP, V152, P38, DOI 10.1016/j.mod.2018.07.001

Zhang JZ, 2015, NEUROBIOL DIS, V82, P32, DOI 10.1016/j.nbd.2015.05.015

Zhang LQ, 2018, CELL TRANSPLANT, V27, P471, DOI 10.1177/0963689717752945

Zhang LS, 2016, J GENET GENOMICS, V43, P587, DOI 10.1016/j.jgg.2016.05.002

Zhang WM, 2018, MOL MED REP, V17, P330, DOI 10.3892/mmr.2017.7856

Zhang WP, 2018, J BIOL CHEM, V293, P15641, DOI 10.1074/jbc.RA118.002840

Zhang YW, 2016, ACS NANO, V10, P3881, DOI 10.1021/acsnano.6b02284

Zhao ML, 2018, J ALZHEIMERS DIS, V63, P303, DOI 10.3233/JAD-180002

Zhou W, 2018, J GINSENG RES, V42, P199, DOI 10.1016/j.jgr.2017.03.004

Zhu YH, 2014, J CLIN INVEST, V124, P3263, DOI 10.1172/JCI73015

NR 357

TC 100

Z9 104

U1 2

U2 23

PU WILEY

PI HOBOKEN

PA 111 RIVER ST, HOBOKEN 07030-5774, NJ USA

SN 1474-9718

EI 1474-9726

J9 AGING CELL

JI Aging Cell

PD MAR

PY 2020

VL 19

IS 3

AR e13101

DI 10.1111/acel.13101

EA FEB 2020

PG 41

WC Cell Biology; Geriatrics & Gerontology

WE Science Citation Index Expanded (SCI-EXPANDED)

SC Cell Biology; Geriatrics & Gerontology

GA KU3LS

UT WOS:000512876100001

PM 32050297

OA gold, Green Published

DA 2024-08-12

ER

PT J

AU Yang, HY

Wang, L

Zang, CX

Wang, Y

Shang, JM

Zhang, ZH

Liu, H

Bao, XQ

Wang, XL

Zhang, D

AF Yang, Hanyu

Wang, Lu

Zang, Caixia

Wang, Yue

Shang, Junmei

Zhang, Zihong

Liu, Hui

Bao, Xiuqi

Wang, Xiaoliang

Zhang, Dan

TI Src Inhibition Attenuates Neuroinflammation and Protects Dopaminergic

Neurons in Parkinson's Disease Models

SO FRONTIERS IN NEUROSCIENCE

LA English

DT Article

DE Src; microglia; neuroinflammation; Parkinson's disease; neuroprotection

ID MICROGLIAL ACTIVATION; FAMILY KINASES; C-SRC; CELL; NEURODEGENERATION;

MITOCHONDRIAL; INFLAMMATION; MECHANISM; BRAIN; ALPHA

AB Chronic neuroinflammation is of great importance in the pathogenesis of Parkinson's disease (PD). During the process of neuroinflammation, overactivated microglia release many proinflammatory factors, which eventually induce neurodegeneration. Inhibition of excessive microglial activation is regarded as a promising strategy for PD treatment. Src is a non-receptor tyrosine kinase that is closely related to tumors. Recently, some reports indicated that Src is a central mediator in multiple signaling pathways including neuroinflammation. The aim of our study was to demonstrate the role of Src in microglial regulation and neuroinflammation. The lipopolysaccharide (LPS)-stimulated BV2 microglia model and the 1-methyl-4-phenyl-1,2,3,6-tetrahydropyridine (MPTP)-induced PD model were applied in this study. The results showed that inhibition of Src could significantly relieve microgliosis and decrease levels of inflammatory factors. Besides, inhibition of Src function reduced the loss of dopaminergic neurons and improved the motor behavior of the MPTP-treated mice. Thus, this study not only verified the critical role of Src tyrosine kinase in neuroinflammation but also further proved that interfering neuroinflammation is beneficial for PD treatment. More importantly, this study shed a light on the hypothesis that Src tyrosine kinase might be a potential therapeutic target for PD and other neuroinflammation-related diseases.

C1 [Yang, Hanyu; Wang, Lu; Zang, Caixia; Wang, Yue; Shang, Junmei; Zhang, Zihong; Liu, Hui; Bao, Xiuqi; Wang, Xiaoliang; Zhang, Dan] Chinese Acad Med Sci & Peking Union Med Coll, State Key Lab Bioact Substrate & Funct Nat Med, Inst Mat Med, Dept Pharmacol, Beijing, China.

C3 Chinese Academy of Medical Sciences - Peking Union Medical College;

Peking Union Medical College

RP Zhang, D (corresponding author), Chinese Acad Med Sci & Peking Union Med Coll, State Key Lab Bioact Substrate & Funct Nat Med, Inst Mat Med, Dept Pharmacol, Beijing, China.

EM danzhang@imm.ac.cn

OI Wang, Xiaoliang/0009-0007-7036-1089

FU National Science Foundation of China [81630097, 81773718]; CAMS

Innovation Fund for Medical Sciences [2016-I2M-3-011]; National Major

Scientific and Technological Special Project for "Significant New Drugs

Development" during the Thirteenth Five-year Plan Period

[2018ZX09711001-003-005, 2018ZX09711001-008-005,

2018ZX09711001-003-020]; CAMS Fundamental Research Funds for the Central

Universities [2018RC350002]

FX This work was supported by grants from the National Science Foundation

of China (81630097 and 81773718), CAMS Innovation Fund for Medical

Sciences (No. 2016-I2M-3-011), National Major Scientific and

Technological Special Project for "Significant New Drugs Development"

during the Thirteenth Five-year Plan Period (2018ZX09711001-003-005,

2018ZX09711001-008-005, and 2018ZX09711001-003-020), and CAMS

Fundamental Research Funds for the Central Universities (2018RC350002).

CR Amor S, 2014, IMMUNOLOGY, V142, P151, DOI 10.1111/imm.12233

Calvello R, 2017, J NEUROIMMUNE PHARM, V12, P327, DOI 10.1007/s11481-016-9720-7

Chen HL, 2005, ANN NEUROL, V58, P963, DOI 10.1002/ana.20682

CLEETER MWJ, 1992, J NEUROCHEM, V58, P786, DOI 10.1111/j.1471-4159.1992.tb09789.x

Dhawan G, 2012, NEUROBIOL AGING, V33, P2247, DOI 10.1016/j.neurobiolaging.2011.10.027

Dhawan G, 2012, J NEUROINFLAMM, V9, DOI 10.1186/1742-2094-9-117

Dheen ST, 2007, CURR MED CHEM, V14, P1189, DOI 10.2174/092986707780597961

Fellner A, 2017, INT J MOL SCI, V18, DOI 10.3390/ijms18081666

Gao XA, 2011, NEUROLOGY, V76, P863, DOI 10.1212/WNL.0b013e31820f2d79

González-Scarano F, 1999, ANNU REV NEUROSCI, V22, P219, DOI 10.1146/annurev.neuro.22.1.219

Gu RN, 2017, BRAIN BEHAV IMMUN, V60, P206, DOI 10.1016/j.bbi.2016.10.018

Hanke JH, 1996, J BIOL CHEM, V271, P695, DOI 10.1074/jbc.271.2.695

Harms AS, 2011, MOL THER, V19, P46, DOI 10.1038/mt.2010.217

He Y, 2001, BRAIN RES, V909, P187, DOI 10.1016/S0006-8993(01)02681-6

Hou LY, 2018, REDOX BIOL, V14, P250, DOI 10.1016/j.redox.2017.09.017

Iliopoulos D, 2009, CELL, V139, P693, DOI 10.1016/j.cell.2009.10.014

Iselin-Chaves IA, 2009, ANAESTHESIA, V64, P1359, DOI 10.1111/j.1365-2044.2009.06068.x

Ishizawar R, 2004, CANCER CELL, V6, P209, DOI 10.1016/j.ccr.2004.09.001

Joers V, 2017, PROG NEUROBIOL, V155, P57, DOI 10.1016/j.pneurobio.2016.04.006

Kirkley KS, 2017, J NEUROINFLAMM, V14, DOI 10.1186/s12974-017-0871-0

Kurkowska-Jastrzebska I, 2004, INT IMMUNOPHARMACOL, V4, P1307, DOI 10.1016/j.intimp.2004.05.006

Li XF, 2017, HEPATOLOGY, V66, P1934, DOI 10.1002/hep.29372

Liu ST, 2014, FRONT PHYSIOL, V4, DOI 10.3389/fphys.2013.00416

Lowell CA, 2004, MOL IMMUNOL, V41, P631, DOI 10.1016/j.molimm.2004.04.010

Manocha GD, 2015, PLOS ONE, V10, DOI 10.1371/journal.pone.0132604

Giráldez-Pérez RM, 2014, ACTA NEUROPATHOL COM, V2, DOI 10.1186/s40478-014-0176-9

Miller RM, 2007, EXP NEUROL, V204, P421, DOI 10.1016/j.expneurol.2006.12.005

MOGI M, 1994, NEUROSCI LETT, V180, P147, DOI 10.1016/0304-3940(94)90508-8

Moss DW, 2001, EUR J NEUROSCI, V13, P529, DOI 10.1046/j.1460-9568.2001.01418.x

Nakagawa Y, 2015, PHARMACOL THERAPEUT, V154, P21, DOI 10.1016/j.pharmthera.2015.06.010

Okada M, 2012, INT J BIOL SCI, V8, P1385, DOI 10.7150/ijbs.5141

Roskoski R, 2015, PHARMACOL RES, V94, P9, DOI 10.1016/j.phrs.2015.01.003

Sen Banibrata, 2011, J Signal Transduct, V2011, P865819, DOI 10.1155/2011/865819

Sherer TB, 2003, J NEUROSCI, V23, P10756, DOI 10.1523/jneurosci.23-34-10756.2003

Shin EJ, 2018, FOOD CHEM TOXICOL, V121, P254, DOI 10.1016/j.fct.2018.09.005

Smeyne RJ, 2016, PLOS ONE, V11, DOI 10.1371/journal.pone.0164094

Socodato R, 2015, GLIA, V63, P497, DOI 10.1002/glia.22767

STEHELIN D, 1976, NATURE, V260, P170, DOI 10.1038/260170a0

Subedi L, 2017, BMC NEUROSCI, V18, DOI 10.1186/s12868-017-0399-3

Sveinbjornsdottir S, 2016, J NEUROCHEM, V139, P318, DOI 10.1111/jnc.13691

Thomas SM, 1997, ANNU REV CELL DEV BI, V13, P513, DOI 10.1146/annurev.cellbio.13.1.513

Wang YD, 2016, J MED CHEM, V59, P9062, DOI 10.1021/acs.jmedchem.6b00976

Yan AJ, 2017, MOL MED REP, V16, P2491, DOI 10.3892/mmr.2017.6884

Yeh CH, 2014, ENVIRON TOXICOL, V29, P1162, DOI 10.1002/tox.21847

Zhou P, 2019, BRAIN BEHAV IMMUN, V76, P61, DOI 10.1016/j.bbi.2018.11.006

NR 45

TC 26

Z9 27

U1 1

U2 10

PU FRONTIERS MEDIA SA

PI LAUSANNE

PA AVENUE DU TRIBUNAL FEDERAL 34, LAUSANNE, CH-1015, SWITZERLAND

EI 1662-453X

J9 FRONT NEUROSCI-SWITZ

JI Front. Neurosci.

PD FEB 18

PY 2020

VL 14

AR 45

DI 10.3389/fnins.2020.00045

PG 14

WC Neurosciences

WE Science Citation Index Expanded (SCI-EXPANDED)

SC Neurosciences & Neurology

GA KT3SQ

UT WOS:000518937200001

PM 32132891

OA Green Published, gold

DA 2024-08-12

ER

PT J

AU Ghasemi, F

Bagheri, H

Barreto, GE

Read, MI

Sahebkar, A

AF Ghasemi, Faezeh

Bagheri, Hossein

Barreto, George E.

Read, Morgayn I.

Sahebkar, Amirhossein

TI Effects of Curcumin on Microglial Cells

SO NEUROTOXICITY RESEARCH

LA English

DT Review

DE Curcumin; Microglia; neuroinflammation; Neuroprotection;

neurodegenerative disease

ID CENTRAL-NERVOUS-SYSTEM; FATTY LIVER-DISEASE; I CLINICAL-TRIAL;

NF-KAPPA-B; OXIDATIVE DAMAGE; INFLAMMATORY RESPONSES; PIPERINE

COMBINATION; SIGNALING PATHWAY; DOPAMINERGIC-NEURONS; METABOLIC SYNDROME

AB Microglia are innate immune system cells which reside in the central nervous system (CNS). Resting microglia regulate the homeostasis of the CNS via phagocytic activity to clear pathogens and cell debris. Sometimes, however, to protect neurons and fight invading pathogens, resting microglia transform to an activated-form, producing inflammatory mediators, such as cytokines, chemokines, iNOS/NO and cyclooxygenase-2 (COX-2). Excessive inflammation, however, leads to damaged neurons and neurodegenerative disease (NDs), such as Parkinson's disease (PD), Alzheimer's disease (AD), Huntington's disease (HD), multiple sclerosis (MS) and amyotrophic lateral sclerosis (ALS). Curcumin is a phytochemical isolated from Curcuma longa. It is widely used in Asia and has many therapeutic properties, including antioxidant, anti-viral, anti-bacterial, anti-mutagenic, anti-amyloidogenic and anti-inflammatory, especially with respect to neuroinflammation and neurological disorders (NDs). Curcumin is a pleiotropic molecule that inhibits microglia transformation, inflammatory mediators and subsequent NDs. In this mini-review, we discuss the effects of curcumin on microglia and explore the underlying mechanisms.

C1 [Ghasemi, Faezeh] High Inst Res & Educ Transfus Med, Blood Transfus Res Ctr, Tehran, Iran.

[Bagheri, Hossein] Arak Univ Med Sci, Dept Biotechnol, Fac Med, Arak, Iran.

[Barreto, George E.] Pontificia Univ Javeriana, Fac Ciencias, Dept Nutr & Bioquim, Bogota, DC, Colombia.

[Barreto, George E.] Univ Autonoma Chile, Inst Ciencias Biomed, Santiago, Chile.

[Read, Morgayn I.] Univ Otago, Sch Med Sci, Dept Pharmacol, Dunedin, New Zealand.

[Sahebkar, Amirhossein] Mashhad Univ Med Sci, Neurogen Inflammat Res Ctr, Mashhad, Razavi Khorasan, Iran.

[Sahebkar, Amirhossein] Mashhad Univ Med Sci, Pharmaceut Technol Inst, Biotechnol Res Ctr, Mashhad, Razavi Khorasan, Iran.

[Sahebkar, Amirhossein] Mashhad Univ Med Sci, Sch Pharm, Mashhad, Razavi Khorasan, Iran.

[Sahebkar, Amirhossein] Mashhad Univ Med Sci, Sch Med, Dept Med Biotechnol, POB 91779-48564, Mashhad, Razavi Khorasan, Iran.

C3 Pontificia Universidad Javeriana; Universidad Autonoma de Chile;

University of Otago; Mashhad University Medical Science; Mashhad

University Medical Science; Mashhad University Medical Science; Mashhad

University Medical Science

RP Sahebkar, A (corresponding author), Mashhad Univ Med Sci, Neurogen Inflammat Res Ctr, Mashhad, Razavi Khorasan, Iran.; Sahebkar, A (corresponding author), Mashhad Univ Med Sci, Pharmaceut Technol Inst, Biotechnol Res Ctr, Mashhad, Razavi Khorasan, Iran.; Sahebkar, A (corresponding author), Mashhad Univ Med Sci, Sch Pharm, Mashhad, Razavi Khorasan, Iran.; Sahebkar, A (corresponding author), Mashhad Univ Med Sci, Sch Med, Dept Med Biotechnol, POB 91779-48564, Mashhad, Razavi Khorasan, Iran.

EM sahebkara@mums.ac.ir

RI Barreto, George E./AAD-7420-2020; Sahebkar, Amirhossein/B-5124-2018;

Bagheri, Hossein/JWP-3070-2024

OI Barreto, George E./0000-0002-6644-1971; Bagheri,

Hossein/0000-0002-5802-908X

CR Abdollahi E, 2018, J CELL PHYSIOL, V233, P830, DOI 10.1002/jcp.25778

Agrawal DK, 2010, MED RES REV, V30, P818, DOI 10.1002/med.20188

Akaishi T, 2018, EUR J PHARMACOL, V819, P190, DOI 10.1016/j.ejphar.2017.12.008

Alexiou A, 2019, MOL NEUROBIOL, V56, P4530, DOI 10.1007/s12035-018-1397-x

Alliot F, 1999, DEV BRAIN RES, V117, P145, DOI 10.1016/S0165-3806(99)00113-3

Ameruoso A, 2017, FRONT IMMUNOL, V8, DOI 10.3389/fimmu.2017.01411

Bavarsad K, 2019, MOL NEUROBIOL, V56, P1391, DOI 10.1007/s12035-018-1169-7

Bennett ML, 2016, P NATL ACAD SCI USA, V113, pE1738, DOI 10.1073/pnas.1525528113

Bhattacharjee S, 2016, PLOS ONE, V11, DOI 10.1371/journal.pone.0150211

Bianconi V, 2018, CURR CARDIOL REP, V20, DOI 10.1007/s11886-018-0994-7

Cabezas R, 2019, MOL NEUROBIOL, V56, P2339, DOI 10.1007/s12035-018-1203-9

Cabezas R, 2012, NEUROSCI RES, V74, P80, DOI 10.1016/j.neures.2012.07.008

Canales-Aguirre AA, 2012, NUTR NEUROSCI, V15, P62, DOI 10.1179/1476830511Y.0000000034

Chen GL, 2018, CELL MOL NEUROBIOL, V38, P1465, DOI 10.1007/s10571-018-0616-3

Chen HY, 2018, RESTOR NEUROL NEUROS, V36, P583, DOI 10.3233/RNN-180834

Cheng AL, 2001, ANTICANCER RES, V21, P2895

Chiu SS, 2011, ANTICANCER RES, V31, P907

Choi DK, 2011, MOLECULES, V16, P1021, DOI 10.3390/molecules16021021

Cianciulli A, 2016, INT IMMUNOPHARMACOL, V36, P282, DOI 10.1016/j.intimp.2016.05.007

Cicero AFG, 2017, ARCH MED SCI, V13, P965, DOI [10.5114/aoms.2017.69326, 10.1093/nutrit/nux047]

Colonna M, 2017, ANNU REV IMMUNOL, V35, P441, DOI 10.1146/annurev-immunol-051116-052358

Deguine Jacques, 2014, F1000Prime Rep, V6, P97, DOI 10.12703/P6-97

Ding FJ, 2016, EXP THER MED, V12, P823, DOI 10.3892/etm.2016.3413

Dong WW, 2018, TOXICOL APPL PHARM, V346, P28, DOI 10.1016/j.taap.2018.03.020

Eun CS, 2017, BMC COMPLEM ALTERN M, V17, DOI 10.1186/s12906-017-1880-3

Ganjali S, 2017, PHARMACOL RES, V119, P208, DOI 10.1016/j.phrs.2017.02.008

Ghandadi M, 2017, CURR PHARM DESIGN, V23, P921, DOI 10.2174/1381612822666161006151605

Ghosh S, 2015, FOOD CHEM TOXICOL, V83, P111, DOI 10.1016/j.fct.2015.05.022

Ginhoux F, 2010, SCIENCE, V330, P841, DOI 10.1126/science.1194637

Guo LY, 2013, PLOS ONE, V8, DOI 10.1371/journal.pone.0070565

Gupta SC, 2013, BIOFACTORS, V39, P2, DOI 10.1002/biof.1079

Hamzehzadeh L, 2018, J CELL PHYSIOL, V233, P6530, DOI 10.1002/jcp.26620

Hanisch UK, 2013, FRONT CELL NEUROSCI, V7, DOI 10.3389/fncel.2013.00065

He GL, 2016, PLOS ONE, V11, DOI 10.1371/journal.pone.0147721

He GL, 2014, J NEUROINFLAMM, V11, DOI 10.1186/1742-2094-11-49

He LF, 2010, BRAIN RES, V1339, P60, DOI 10.1016/j.brainres.2010.04.014

Hesari A, 2018, J CELL BIOCHEM, V119, P7898, DOI 10.1002/jcb.26829

Lanussa OH, 2016, CNS NEUROL DISORD-DR, V15, P242, DOI 10.2174/1871527315666160202122032

Hidalgo-Lanussa O, 2018, MOL NEUROBIOL, V55, P5462, DOI 10.1007/s12035-017-0777-y

Hillmer EJ, 2016, CYTOKINE GROWTH F R, V31, P1, DOI 10.1016/j.cytogfr.2016.05.001

Hoppe JB, 2013, NEUROBIOL LEARN MEM, V106, P134, DOI 10.1016/j.nlm.2013.08.001

Hosseini S, 2018, REP BIOCHEM MOL BIOL, V6, P137

Hu SX, 2015, EXPERT REV NEUROTHER, V15, P629, DOI 10.1586/14737175.2015.1044981

Huang YB, 2018, NAT NEUROSCI, V21, P530, DOI 10.1038/s41593-018-0090-8

Iglesias J, 2017, MOL NEUROBIOL, V54, P2518, DOI 10.1007/s12035-016-9833-2

Imbimbo BP, 2010, FRONT AGING NEUROSCI, V2, DOI 10.3389/fnagi.2010.00019

Iranshahi M, 2009, EUR J CANCER PREV, V18, P412, DOI 10.1097/CEJ.0b013e32832c389e

Jack CS, 2005, J IMMUNOL, V175, P4320, DOI 10.4049/jimmunol.175.7.4320

Jacob A, 2007, PPAR RES, V2007, DOI 10.1155/2007/89369

Karimian MS, 2017, CURR PHARM DESIGN, V23, P2462, DOI 10.2174/1381612823666170222122822

Karimian MS, 2017, CYTOKINE GROWTH F R, V33, P55, DOI 10.1016/j.cytogfr.2016.10.001

Karlstetter M, 2011, J NEUROINFLAMM, V8, DOI 10.1186/1742-2094-8-125

Kaur H, 2015, NEUROCHEM INT, V89, P40, DOI 10.1016/j.neuint.2015.07.009

Keihanian F, 2018, J CELL PHYSIOL, V233, P4497, DOI 10.1002/jcp.26249

Kettenmann H, 2011, PHYSIOL REV, V91, P461, DOI 10.1152/physrev.00011.2010

Kodali M, 2018, BRAIN BEHAV IMMUN, V69, P499, DOI 10.1016/j.bbi.2018.01.009

Lao Christopher D, 2006, BMC Complement Altern Med, V6, P10, DOI 10.1186/1472-6882-6-10

Lee KH, 2012, INT J MOL SCI, V13, P2985, DOI 10.3390/ijms13032985

Lelli D, 2017, PHARMACOL RES, V115, P133, DOI 10.1016/j.phrs.2016.11.017

Liang G, 2008, BIOORG MED CHEM LETT, V18, P1525, DOI 10.1016/j.bmcl.2007.12.068

Liu WX, 2016, NEUROCHEM INT, V100, P164, DOI 10.1016/j.neuint.2016.09.014

Liu ZJ, 2017, FRONT AGING NEUROSCI, V9, DOI 10.3389/fnagi.2017.00233

Liu ZJ, 2016, FRONT PHARMACOL, V7, DOI 10.3389/fphar.2016.00261

Lund H, 2018, NAT IMMUNOL, V19, P435, DOI 10.1038/s41590-018-0091-5

Maiti P, 2018, BMC NEUROSCI, V19, DOI 10.1186/s12868-018-0406-3

Mantovani A, 2005, IMMUNITY, V23, P344, DOI 10.1016/j.immuni.2005.10.001

Martinez F.O., 2014, F1000PRIME REP, V6, P12703, DOI DOI 10.12703/P6-13

Mazzolani F, 2013, CLIN OPHTHALMOL, V7, P939, DOI 10.2147/OPTH.S45820

Miobedzka J, 1910, GESELLSCHAFT, V43, P2163

Mirzaei H, 2016, INT J CANCER, V139, P1683, DOI 10.1002/ijc.30224

Mokhtari-Zaer A, 2019, J CELL PHYSIOL, V234, P214, DOI 10.1002/jcp.26848

Momtazi AA, 2016, REV PHYSIOL BIOCH P, V171, P1, DOI 10.1007/112_2016_3

Momtazi AA, 2016, CURR PHARM DESIGN, V22, P4386, DOI 10.2174/1381612822666160527113501

Momtazi AA, 2016, MOL DIAGN THER, V20, P335, DOI 10.1007/s40291-016-0202-7

Morales I, 2014, FRONT CELL NEUROSCI, V8, DOI 10.3389/fncel.2014.00112

Mukherjee S, 2018, J EXP CLIN CANC RES, V37, DOI 10.1186/s13046-018-0792-5

Mukherjee S, 2016, INT J CANCER, V139, P2838, DOI 10.1002/ijc.30398

Naeimi R, 2018, NEUROSCI LETT, V674, P1, DOI 10.1016/j.neulet.2018.03.018

Napoli I, 2009, NEUROSCIENCE, V158, P1030, DOI 10.1016/j.neuroscience.2008.06.046

Niskanen J, 2016, NANOMEDICINE-UK, V11, P447, DOI 10.2217/nnm.15.214

Panahi Y, 2018, DRUG RES, V68, P403, DOI 10.1055/s-0044-101752

Panahi Y, 2017, DRUG RES, V67, P244, DOI 10.1055/s-0043-100019

Panahi Y, 2017, INFLAMMOPHARMACOLOGY, V25, P25, DOI 10.1007/s10787-016-0301-4

Panahi Y, 2016, J CARDIOVASC PHARM, V68, P223, DOI 10.1097/FJC.0000000000000406

Panahi Y, 2015, CLIN NUTR, V34, P1101, DOI 10.1016/j.clnu.2014.12.019

Panahi Yunes, 2016, Journal of Dietary Supplements, V13, P93, DOI 10.3109/19390211.2014.952865

Panahi Y, 2014, COMPLEMENT THER MED, V22, P851, DOI 10.1016/j.ctim.2014.07.006

Parada E, 2015, MOL NUTR FOOD RES, V59, P1690, DOI 10.1002/mnfr.201500279

Parsamanesh N, 2018, PHARMACOL RES, V136, P181, DOI 10.1016/j.phrs.2018.09.012

Ransohoff RM, 2016, NAT NEUROSCI, V19, P987, DOI 10.1038/nn.4338

Ransohoff RM, 2010, NATURE, V468, P253, DOI 10.1038/nature09615

Réu P, 2017, CELL REP, V20, P779, DOI 10.1016/j.celrep.2017.07.004

Riazi K, 2015, J NEUROSCI, V35, P4942, DOI 10.1523/JNEUROSCI.4485-14.2015

Rossi F, 2018, NAT NEUROSCI, V21, P455, DOI 10.1038/s41593-018-0123-3

Saeidinia A, 2018, PHARMACOL RES, V131, P112, DOI 10.1016/j.phrs.2018.03.009

Sahebkar A, 2016, PHARMACOL RES, V107, P234, DOI 10.1016/j.phrs.2016.03.026

Sahebkar A, 2015, J FUNCT FOODS, V18, P898, DOI 10.1016/j.jff.2015.01.005

Sahebkar A, 2010, FERTIL STERIL, V94, pE75, DOI 10.1016/j.fertnstert.2010.07.1071

Sales SS, 2018, ANN ONCOL, V29

Sawikr Y, 2017, ADV PROTEIN CHEM STR, V108, P33, DOI 10.1016/bs.apcsb.2017.02.001

Shakeri A, 2019, CURR VASC PHARMACOL, V17, P262, DOI 10.2174/1570161116666180209113014

Shakeri A, 2016, J NEUROSCI RES, V94, P111, DOI 10.1002/jnr.23696

Sharma N, 2017, INFLAMMOPHARMACOLOGY, V25, P351, DOI 10.1007/s10787-017-0346-z

Sharma RA, 2004, CLIN CANCER RES, V10, P6847, DOI 10.1158/1078-0432.CCR-04-0744

Shi XL, 2015, NEUROSCI LETT, V594, P105, DOI 10.1016/j.neulet.2015.03.045

Sorrenti V, 2018, FRONT PHARMACOL, V9, DOI 10.3389/fphar.2018.00183

Sousa C, 2017, FRONT IMMUNOL, V8, DOI 10.3389/fimmu.2017.00198

Subramaniam A, 2013, BBA-REV CANCER, V1835, P46, DOI 10.1016/j.bbcan.2012.10.002

Tegenge MA, 2014, EXP NEUROL, V253, P102, DOI 10.1016/j.expneurol.2013.12.016

Teymouri M, 2017, BIOFACTORS, V43, P331, DOI 10.1002/biof.1344

Tocharus J, 2012, J NAT MED-TOKYO, V66, P400, DOI 10.1007/s11418-011-0599-6

Tripanichkul W, 2013, EUR REV MED PHARMACO, V17, P1360

Tripanichkul W, 2012, INT J NEUROSCI, V122, P263, DOI 10.3109/00207454.2011.648760

Tsai YM, 2011, INT J PHARMACEUT, V416, P331, DOI 10.1016/j.ijpharm.2011.06.030

Vainchtein ID, 2014, GLIA, V62, P1724, DOI 10.1002/glia.22711

Valverde Y, 2016, HAEMATOLOGICA, V101, pE44, DOI 10.3324/haematol.2015.137489

Venigalla M, 2016, NEUROCHEM INT, V95, P63, DOI 10.1016/j.neuint.2015.10.011

Wang HM, 2010, J ALZHEIMERS DIS, V20, P1189, DOI 10.3233/JAD-2010-091336

Wang WY, 2015, ANN TRANSL MED, V3, DOI 10.3978/j.issn.2305-5839.2015.03.49

Wang YH, 2017, CELL PHYSIOL BIOCHEM, V44, P479, DOI 10.1159/000485085

Xu MX, 2016, BRAIN BEHAV IMMUN, V58, P69, DOI 10.1016/j.bbi.2016.01.001

Yang SF, 2008, NEUROCHEM RES, V33, P2044, DOI 10.1007/s11064-008-9675-z

Yu Y, 2018, FRONT PHARM, V9

Yuan J, 2017, J SURG RES, V207, P85, DOI 10.1016/j.jss.2016.08.090

Yue YK, 2014, J OCUL PHARMACOL TH, V30, P657, DOI 10.1089/jop.2014.0022

Zabihi NA, 2017, CURR PHARM DESIGN, V23, P969, DOI 10.2174/1381612822666161010115235

Zhang LJ, 2010, INT IMMUNOPHARMACOL, V10, P331, DOI 10.1016/j.intimp.2009.12.004

Zhang ZY, 2017, MOL NEUROBIOL, V54, P1, DOI 10.1007/s12035-015-9635-y

Zhou JJ, 2017, INFLAMM RES, V66, P177, DOI 10.1007/s00011-016-1004-4

Zhu HT, 2014, J NEUROINFLAMM, V11, DOI 10.1186/1742-2094-11-59

Zhuang XY, 2011, MOL THER, V19, P1769, DOI 10.1038/mt.2011.164

Zusso M, 2017, BRIT J PHARMACOL, V174, P1090, DOI 10.1111/bph.13746

NR 132

TC 63

Z9 66

U1 2

U2 33

PU SPRINGER

PI NEW YORK

PA 233 SPRING ST, NEW YORK, NY 10013 USA

SN 1029-8428

EI 1476-3524

J9 NEUROTOX RES

JI Neurotox. Res.

PD JUL

PY 2019

VL 36

IS 1

BP 12

EP 26

DI 10.1007/s12640-019-00030-0

PG 15

WC Neurosciences

WE Science Citation Index Expanded (SCI-EXPANDED)

SC Neurosciences & Neurology

GA ID4OI

UT WOS:000471655800002

PM 30949950

DA 2024-08-12

ER

PT J

AU Açar, Y

Agagünduz, D

De Cicco, P

Capasso, R

AF Acar, Yasemin

Agagunduz, Duygu

De Cicco, Paola

Capasso, Raffaele

TI Flavonoids: Their putative neurologic roles, epigenetic changes, and gut

microbiota alterations in Parkinson's disease

SO BIOMEDICINE & PHARMACOTHERAPY

LA English

DT Article

DE Flavonoids; neurodegenerative disease; Parkinson's disease; Polyphenols

ID ALPHA-SYNUCLEIN; EPIGALLOCATECHIN GALLATE; DIETARY FLAVONOIDS; SH-SY5Y

CELLS; NEUROTOXICITY; ANTHOCYANIN; SYMPTOMS; MODEL; RISK; FAT

AB Parkinson's Disease (PD), a neurodegenerative disorder, is characterized by the degeneration of progressive dopaminergic (DA) neurons in the substantia nigra region of the human midbrain. Although just what causes PD remains a mystery, it is known that oxidative stress (OS) as well as mitochondrial dysfunction, neuroinflammation, and insufficient neurotrophic support play a role in the disease's pathophysiology. Phytochemicals are a diverse small molecule group derived from plants that can be classified into numerous classes on the basis of their biological activities and chemical structure. Of these groups of phytochemicals, the most abundant, which has well-established anti-Parkinson's effects, are polyphenols. Flavonoids, including naringin and naringenin, genistein, kaempferol, anthocyanins, epigallocatechin-3-gallate, and baicalein are plant-based biologically active polyphenols, which have been shown to exhibit therapeutic potential when used as treatment for a variety of pathological illnesses, such as neurodegenerative disease (NDs) and PD. Recently, it was reported that flavonoids have beneficial effects on PD, such as the protection of DA neurons, improvement of motor and cognitive abilities, regulation of signaling pathways, and modulation of OS and neuro-inflammation. In addition, by changing the composition of bacteria in gut microbiota, flavonoids reduce pathogenic strains and promote the growth of beneficial strains. In this context, the current paper will provide a literature review on the neurological roles that flavonoids play, as one of the most abundant phytochemical families, in PD.

C1 [Acar, Yasemin; Agagunduz, Duygu] Gazi Univ, Dept Nutr & Dietet, Ankara, Turkey.

[De Cicco, Paola] Univ Naples Federico II, Dept Pharm, I-80131 Naples, Italy.

[Capasso, Raffaele] Univ Naples Federico II, Dept Agr Sci, I-80055 Portici, Naples, Italy.

C3 Gazi University; University of Naples Federico II; University of Naples

Federico II

RP Açar, Y (corresponding author), Gazi Univ, Dept Nutr & Dietet, Ankara, Turkey.; Capasso, R (corresponding author), Univ Naples Federico II, Dept Agr Sci, I-80055 Portici, Naples, Italy.

EM dytyaseminacar@gmail.com; rafcapas@unina.it

RI Capasso, Raffaele/AFK-6983-2022

OI Capasso, Raffaele/0000-0002-3335-1822

FU Italian Ministry of University and Research - European Union -

NextGenerationEU; Italian Ministry of University and Research

[PE00000003, CUP D93C22000890001]

FX The paper was funded under the National Recovery and Resilience Plan

(NRRP) , Mission 4 Component 2 Investment 1.3-Call for tender No. 341 of

15 March 2022 of Italian Ministry of University and Research funded by

the European Union - NextGenerationEU; Award Number: Project code

PE00000003, Concession Decree No. 1550 of 11 October 2022 adopted by the

Italian Ministry of University and Research, CUP D93C22000890001,

Project title "ON Foods-Research and innovation network on food and

nutrition Sustainability, Safety and Security - Working ON Foods".

CR Abdul-Latif R, 2021, J INTEGR MED-JIM, V19, P300, DOI 10.1016/j.joim.2021.03.007

Ahmad MH, 2021, NEUROPHARMACOLOGY, V201, DOI 10.1016/j.neuropharm.2021.108831

Angelopoulou E, 2022, BRAIN SCI, V12, DOI 10.3390/brainsci12020175

Arora I, 2020, GENES-BASEL, V11, DOI 10.3390/genes11091094

Aryal S, 2020, MOLECULES, V25, DOI 10.3390/molecules25194382

Balakrishnan R, 2021, OXID MED CELL LONGEV, V2021, DOI 10.1155/2021/6680935

Bangar SP, 2023, CRIT REV FOOD SCI, V63, P9580, DOI 10.1080/10408398.2022.2067121

Chagraoui A, 2020, INT J MOL SCI, V21, DOI 10.3390/ijms21010294

Chai XY, 2020, NEUROGASTROENT MOTIL, V32, DOI 10.1111/nmo.13893

Chang KH, 2020, ANTIOXIDANTS-BASEL, V9, DOI 10.3390/antiox9070597

Chaudhuri JR, 2021, TURK J NEUROL, V27, P52, DOI 10.4274/tnd.2021.52993

Cheatham CL, 2022, FRONT NEUROSCI-SWITZ, V16, DOI 10.3389/fnins.2022.833202

Chen C, 2019, FRONT IMMUNOL, V10, DOI 10.3389/fimmu.2019.00936

Chen L, 2017, ARCH GERONTOL GERIAT, V73, P29, DOI 10.1016/j.archger.2017.07.004

Chen M, 2015, NEUROSCIENCE, V286, P383, DOI 10.1016/j.neuroscience.2014.12.003

Chen M, 2022, FRONT NEUROL, V12, DOI 10.3389/fneur.2021.646817

Chen X, 2022, CRIT REV FOOD SCI, V62, P5658, DOI 10.1080/10408398.2021.1888693

Cheng CY, 2021, INT J MOL SCI, V22, DOI 10.3390/ijms22063037

Chu CQ, 2021, TRENDS FOOD SCI TECH, V116, P90, DOI 10.1016/j.tifs.2021.07.004

Coe S, 2022, CLIN NUTR ESPEN, V48, P68, DOI 10.1016/j.clnesp.2022.01.023

Cosme P, 2020, ANTIOXIDANTS-BASEL, V9, DOI 10.3390/antiox9121263

Creighton SD, 2020, INT J MOL SCI, V21, DOI 10.3390/ijms21186918

Dashputre NL, 2023, EUR J MED CHEM REP, V8, DOI 10.1016/j.ejmcr.2023.100105

Day JO, 2021, GENES-BASEL, V12, DOI 10.3390/genes12071006

De Araújo FM, 2023, NEUROTOX RES, V41, P224, DOI 10.1007/s12640-022-00616-1

Dias MC, 2021, MOLECULES, V26, DOI 10.3390/molecules26175377

Du ZR, 2021, J STEROID BIOCHEM, V214, DOI 10.1016/j.jsbmb.2021.105989

Elmazoglu Z, 2020, DRUG CHEM TOXICOL, V43, P96, DOI 10.1080/01480545.2018.1504961

Emran TB, 2023, LIFE-BASEL, V13, DOI 10.3390/life13010099

Fatima Ambreen, 2017, Front Biosci (Elite Ed), V9, P44

Filaferro M, 2022, ANTIOXIDANTS-BASEL, V11, DOI 10.3390/antiox11020211

Forni C, 2019, BIOMED RES INT-UK, V2019, DOI 10.1155/2019/8748253

Gaba B, 2019, BIOMED RES INT-UK, V2019, DOI 10.1155/2019/2382563

Gao X, 2012, NEUROLOGY, V78, P1138, DOI 10.1212/WNL.0b013e31824f7fc4

Garabadu D, 2020, NEUROMOL MED, V22, P314, DOI 10.1007/s12017-019-08590-2

Ghaffafi F, 2018, BASIC CLIN NEUROSCI, V9, P317, DOI 10.32598/bcn.9.5.317

Goel R, 2020, BRAZ J PHARM SCI, V56, DOI 10.1590/s2175-97902019000318388

Goh SY, 2019, INT J MOL SCI, V20, DOI 10.3390/ijms20225649

Gómez-Benito M, 2020, FRONT PHARMACOL, V11, DOI 10.3389/fphar.2020.00356

Gonçalves PB, 2021, BIOMOLECULES, V11, DOI 10.3390/biom11050767

Goyal A, 2022, J FOOD BIOCHEM, V46, DOI 10.1111/jfbc.14415

Guo TT, 2019, FOOD SCI BIOTECHNOL, V28, P1617

Hajizadeh Moghaddam A, 2023, AVICENNA J PHYTOMEDI, V13, P290, DOI [10.22038/AJP.2022.21532, 10.22038/ajp.2022.21532]

Han XJ, 2021, REDOX BIOL, V41, DOI 10.1016/j.redox.2021.101911

He L, 2022, NEUROCHEM RES, V47, P1491, DOI 10.1007/s11064-022-03560-w

Isah T, 2019, BIOL RES, V52

Jacquet AD, 2021, FOOD FUNCT, V12, P11987, DOI [10.1039/d1fo00007a, 10.1039/D1FO00007A]

Javed H., 2019, Synucleins-Biochem. Role Dis., DOI [10.5772/intechopen.83589, DOI 10.5772/INTECHOPEN.83589]

Jennings A, 2020, AM J CLIN NUTR, V111, P340, DOI 10.1093/ajcn/nqz299

Jin S, 2023, BIOMED PHARMACOTHER, V165, DOI 10.1016/j.biopha.2023.115215

Juan CA, 2021, INT J MOL SCI, V22, DOI 10.3390/ijms22094642

Jung UJ, 2018, J MED FOOD, V21, P421, DOI 10.1089/jmf.2017.4078

Kim KO, 2019, CLIN ENDOSC, V52, P137, DOI 10.5946/ce.2019.009

Kwon DK, 2022, CELLS-BASEL, V11, DOI 10.3390/cells11233736

Lamptey RNL, 2022, INT J MOL SCI, V23, DOI 10.3390/ijms23031851

Lard M., 2022, Effects of curcumin, quercetin, and their combinationson neurodegeneration and motor impairment in rotenone-induced Parkinson's disease in rats, DOI [10.21203/rs.3.rs-2228676/v1, DOI 10.21203/RS.3.RS-2228676/V1]

Lei H, 2020, MOLECULES, V25, DOI 10.3390/molecules25215206

Levin J, 2019, LANCET NEUROL, V18, P724, DOI 10.1016/S1474-4422(19)30141-3

Li J, 2020, TROP J PHARM RES, V19, P1197, DOI 10.4314/tjpr.v19i6.12

Li JW, 2019, PHYTOMEDICINE, V60, DOI 10.1016/j.phymed.2019.152954

Li W, 2021, FRONT CELL DEV BIOL, V9, DOI 10.3389/fcell.2021.612476

Lin ZH, 2022, OXID MED CELL LONGEV, V2022, DOI 10.1155/2022/7769355

Ling Z., 2022, Chen Gut microbiome: the cornerstone of life and health, V2022, DOI [DOI 10.1155/2022/9894812, 10.1155/2022/9894812]

Liu YM, 2020, FRONT AGING NEUROSCI, V11, DOI 10.3389/fnagi.2019.00372

Lorenzen N, 2014, J BIOL CHEM, V289, P21299, DOI 10.1074/jbc.M114.554667

Madiha S, 2023, PLANT FOOD HUM NUTR, V78, P654, DOI 10.1007/s11130-023-01103-4

Malar DS, 2020, MOLECULES, V25, DOI 10.3390/molecules25173926

Marques C, 2018, SCI REP-UK, V8, DOI 10.1038/s41598-018-29744-5

Mijatovic E., 2023, Med. Flum. Med. Flum, V59, P280, DOI [10.21860/medflum2023_306353, DOI 10.21860/MEDFLUM2023_306353]

Mittal P, 2023, PHARMACEUTICALS-BASE, V16, DOI 10.3390/ph16070908

Miyazaki I, 2020, NEUROSCI-BASEL, V1, DOI 10.3390/neurosci1010001

Muhammad T, 2019, NUTRIENTS, V11, DOI 10.3390/nu11030648

Murray LK, 2019, NUTR RES REV, V32, P218, DOI 10.1017/S0954422419000106

Musial C, 2020, INT J MOL SCI, V21, DOI 10.3390/ijms21051744

Naoi M, 2022, J NEURAL TRANSM, V129, P737, DOI 10.1007/s00702-021-02427-8

Naoi M, 2019, INT J MOL SCI, V20, DOI 10.3390/ijms20102451

Naoi M, 2019, FUTUR NEUROL, V14, DOI 10.2217/fnl-2018-0028

Nies YH, 2021, FRONT NEUROSCI-SWITZ, V15, DOI 10.3389/fnins.2021.660379

Nishiyama A, 2021, TRENDS GENET, V37, P1012, DOI 10.1016/j.tig.2021.05.002

Özduran G, 2022, NEUROL RES, V44, P511, DOI 10.1080/01616412.2021.2024715

Pan XH, 2020, J FUNCT FOODS, V74, DOI 10.1016/j.jff.2020.104140

Pang SYY, 2019, TRANSL NEURODEGENER, V8, DOI 10.1186/s40035-019-0165-9

Park J, 2022, SIGNAL TRANSDUCT TAR, V7, DOI 10.1038/s41392-022-01078-9

Perez-Pardo P, 2019, GUT, V68, P829, DOI 10.1136/gutjnl-2018-316844

Pokimica B, 2018, NUTRIENTS, V10, DOI 10.3390/nu10070807

Prakash B, 2020, FUNCTIONAL AND PRESERVATIVE PROPERTIES OF PHYTOCHEMICALS, P1, DOI 10.1016/B978-0-12-818593-3.00001-4

Radhakrishnan DM, 2018, NEUROL INDIA, V66, pS26, DOI 10.4103/0028-3886.226451

Rahul, 2020, SCI REP-UK, V10, DOI 10.1038/s41598-020-70236-2

Rasheed M, 2021, INT J MOL SCI, V22, DOI 10.3390/ijms22094956

Renani PG, 2019, J CELL PHYSIOL, V234, P19307, DOI 10.1002/jcp.28622

Sahebnasagh A, 2022, IMMUN AGEING, V19, DOI 10.1186/s12979-022-00292-x

Salehi B, 2019, INT J MOL SCI, V20, DOI 10.3390/ijms20061305

Sergi CM, 2022, CLIN EXP PHARMACOL P, V49, P1029, DOI 10.1111/1440-1681.13691

Shen YH, 2020, BIOMED PHARMACOTHER, V132, DOI 10.1016/j.biopha.2020.110928

Siddique YH, 2018, BRAZ J PHARM SCI, V54, DOI 10.1590/s2175-97902018000317760

dos Santos JS, 2021, FRONT PHARMACOL, V11, DOI 10.3389/fphar.2020.565700

Singh A, 2019, MOLECULES, V24, DOI 10.3390/molecules24081583

Song QX, 2021, NEUROTOXICOLOGY, V87, P188, DOI 10.1016/j.neuro.2021.10.003

Strathearn KE, 2014, BRAIN RES, V1555, P60, DOI 10.1016/j.brainres.2014.01.047

Sugumar M, 2019, INT J NEUROSCI, V129, P534, DOI 10.1080/00207454.2018.1545772

Surguchov A, 2021, BIOMOLECULES, V11, DOI 10.3390/biom11050624

Tambe Mitali A, 2023, bioRxiv, DOI 10.1101/2023.04.26.538474

Tsalenchuk M, 2023, NPJ PARKINSONS DIS, V9, DOI 10.1038/s41531-023-00568-z

Tseng HC, 2020, NEUROTOX RES, V37, P669, DOI 10.1007/s12640-019-00143-6

Tungmunnithum Duangjai, 2018, Medicines (Basel), V5, DOI 10.3390/medicines5030093

Ullah A, 2020, MOLECULES, V25, DOI 10.3390/molecules25225243

Ushiroda C, 2019, J CLIN BIOCHEM NUTR, V65, P34, DOI 10.3164/jcbn.18-116

Venturelli S., 2017, Handbook of Nutrition, Diet, and Epigenetics, P1

Vongsavath T., 2023, Appl. Microbiol, P993, DOI [10.3390/applmicrobiol3030067, DOI 10.3390/APPLMICROBIOL3030067]

Wall-Medrano A., 2020, Functional Foods in Cancer Prevention and Therapy, DOI 10.1016/b978-0-12-816151-7.00011-9

Wang W, 2022, FRONT AGING NEUROSCI, V14, DOI 10.3389/fnagi.2022.877078

Wang WW, 2021, AGING-US, V13, P11738, DOI 10.18632/aging.202868

Wang XR, 2022, J FUNCT FOODS, V92, DOI 10.1016/j.jff.2022.105060

Wang Y, 2022, ANTIOXIDANTS-BASEL, V11, DOI 10.3390/antiox11112287

Wang Y, 2020, FRONT AGING NEUROSCI, V12, DOI 10.3389/fnagi.2020.00277

Wang ZY, 2023, PHYTOMEDICINE, V116, DOI 10.1016/j.phymed.2023.154866

Wang ZY, 2020, INT J MOL SCI, V21, DOI 10.3390/ijms21041515

Westfall S, 2019, FRONT NEUROSCI-SWITZ, V13, DOI 10.3389/fnins.2019.01196

Westfall S, 2018, ARTIF CELL NANOMED B, V46, P441, DOI 10.1080/21691401.2018.1458731

Williamson G, 2018, COMPR REV FOOD SCI F, V17, P1054, DOI 10.1111/1541-4337.12351

Witucki L, 2022, NUTRIENTS, V14, DOI 10.3390/nu14102014

World Health Organization Switzerland, 2022, Epidemiology of Parkinson's disease

Wu HC, 2018, NEURAL REGEN RES, V13, P1375, DOI 10.4103/1673-5374.235250

Wu Q, 2022, J BIOCHEM MOL TOXIC, V36, DOI 10.1002/jbt.22949

Xu Q, 2017, J NUTR, V147, P1926, DOI 10.3945/jn.117.255034

Yang DM, 2019, FRONT NEUROL, V10, DOI 10.3389/fneur.2019.01155

Yang YL, 2019, INT J MOL SCI, V20, DOI 10.3390/ijms20030491

Yao LP, 2019, FASEB J, V33, P8648, DOI 10.1096/fj.201900363R

Yuan H, 2020, AGING CELL, V19, DOI 10.1111/acel.13199

Zaim M, 2021, CYTOTECHNOLOGY, V73, P827, DOI 10.1007/s10616-021-00500-4

Zeng XS, 2018, ASN NEURO, V10, DOI 10.1177/1759091418777438

Zhai H, 2019, J INTEGR NEUROSCI, V18, P423, DOI 10.31083/j.jin.2019.04.192

Zhang JL, 2019, EUR J PHARMACOL, V858, DOI 10.1016/j.ejphar.2019.172500

Zhang LJ, 2019, J NUTR BIOCHEM, V69, P19, DOI 10.1016/j.jnutbio.2019.03.009

Zhang XY, 2022, NEUROLOGY, V98, pE1064, DOI 10.1212/WNL.0000000000013275

Zhang X, 2017, SCI REP-UK, V7, DOI 10.1038/s41598-017-07442-y

Zhao J, 2017, RSC ADV, V7, P32508, DOI 10.1039/c7ra03752j

Zhao XY, 2021, BIOMED PHARMACOTHER, V140, DOI 10.1016/j.biopha.2021.111556

Zheng ZV, 2019, J CLIN NEUROSCI, V64, P242, DOI 10.1016/j.jocn.2019.02.005

Zhu ML, 2022, FRONT IMMUNOL, V13, DOI 10.3389/fimmu.2022.937555

Zhu Q, 2019, PHYTOMEDICINE, V55, P302, DOI 10.1016/j.phymed.2018.09.215

NR 141

TC 25

Z9 25

U1 12

U2 18

PU ELSEVIER FRANCE-EDITIONS SCIENTIFIQUES MEDICALES ELSEVIER

PI ISSY-LES-MOULINEAUX

PA 65 RUE CAMILLE DESMOULINS, CS50083, 92442 ISSY-LES-MOULINEAUX, FRANCE

SN 0753-3322

EI 1950-6007

J9 BIOMED PHARMACOTHER

JI Biomed. Pharmacother.

PD DEC

PY 2023

VL 168

AR 115788

DI 10.1016/j.biopha.2023.115788

EA OCT 2023

PG 13

WC Medicine, Research & Experimental; Pharmacology & Pharmacy

WE Science Citation Index Expanded (SCI-EXPANDED)

SC Research & Experimental Medicine; Pharmacology & Pharmacy

GA Y1FO1

UT WOS:001102797000001

PM 37913731

OA gold

DA 2024-08-12

ER

PT J

AU Chen, SY

Liu, YQ

Niu, YY

Xu, YH

Zhou, QW

Xu, XJ

Wang, J

Yu, M

AF Chen, Siyuan

Liu, Yueqin

Niu, Yuanyuan

Xu, Yuhao

Zhou, Qianwen

Xu, Xiujian

Wang, Jia

Yu, Ming

TI Increased abundance of myeloid-derived suppressor cells and Th17 cells

in peripheral blood of newly-diagnosed Parkinson's disease patients

SO NEUROSCIENCE LETTERS

LA English

DT Article

DE Parkinson's disease; T-helper 17 cells; Myeloid-derived suppressor

cells; Neuroinflammation

ID EXPERIMENTAL AUTOIMMUNE ENCEPHALOMYELITIS; CD4(+) T-CELLS;

BRAIN-BARRIER; NEUROINFLAMMATION; PROMOTE; NEURODEGENERATION;

DIFFERENTIATION; CANCER; TUMOR; CNS

AB Parkinson's disease (PD) is a neurodegenerative disease that is often associated with corresponding neuroinflammation. In the present study, flow cytometry was used to detect T-helper 17 (Th17) cells and myeloid-derived suppressor cells (MDSCs) in 18 patients newly diagnosed with PD as well as 18 normal controls. Results showed that Th17 cells and MDSCs were significantly higher in peripheral blood of PD patients compared to controls (P < 0.001). Furthermore, there was no correlation between Th17 cells and MDSCs in peripheral blood of PD patients. Our findings suggest that Th17 cells and MDSCs may be important factors related to the occurrence and progression of PD, as well as the development of PD-related neuroinflammation. (C) 2017 Elsevier B.V. All rights reserved.

C1 [Chen, Siyuan; Niu, Yuanyuan; Xu, Yuhao; Zhou, Qianwen; Xu, Xiujian; Yu, Ming] Jiangsu Univ, Dept Neurol, Affiliated Hosp, Zhenjiang 212001, Jiangsu, China.

[Liu, Yueqin] Jiangsu Univ, Cent Lab, Affiliated Hosp 4, Zhenjiang 212001, Jiangsu, China.

[Wang, Jia] Jiangsu Univ, Dept Immunol, 301 Xuefu Rd, Zhenjiang 212013, Jiangsu, China.

C3 Jiangsu University; Jiangsu University; Jiangsu University

RP Yu, M (corresponding author), Jiangsu Univ, Dept Neurol, Affiliated Hosp, Zhenjiang 212001, Jiangsu, China.; Wang, J (corresponding author), Jiangsu Univ, Dept Immunol, 301 Xuefu Rd, Zhenjiang 212013, Jiangsu, China.

EM wangjia5698@sina.com; yuming7251@l63.com

RI 周, 倩/GXH-6115-2022; Zhou, Qian/JMB-8448-2023

OI Wang, Jia/0000-0002-9544-9366

FU Six Talent Peaks Project of Jiangsu Province [WSN038]; 333 Project of

Jiangsu Province [BRA2014173]; Social Development Project of Zhenjiang

[SH2015024, SH2015047]

FX The present study was supported by Six Talent Peaks Project of Jiangsu

Province (WSN038), 333 Project of Jiangsu Province (BRA2014173), Social

Development Project of Zhenjiang (SH2015024, SH2015047). We thank the

patients and their families for their participation in this project.

CR Abrams SI, 2016, J INTERF CYTOK RES, V36, P442, DOI 10.1089/jir.2015.0174

Aktas O, 2007, ARCH NEUROL-CHICAGO, V64, P185, DOI 10.1001/archneur.64.2.185

Bahbouhi B, 2009, J LEUKOCYTE BIOL, V86, P1049, DOI 10.1189/jlb.1008666

Barnes PJ, 2016, J ALLERGY CLIN IMMUN, V138, P16, DOI 10.1016/j.jaci.2016.05.011

Basdeo SA, 2016, SPRINGERPLUS, V5, DOI 10.1186/s40064-016-2510-0

Brochard V, 2009, J CLIN INVEST, V119, P182, DOI 10.1172/JCI36470

Chatterjee S, 2013, IMMUNOBIOLOGY, V218, P718, DOI 10.1016/j.imbio.2012.08.271

Chen YH, 2015, INT J CLIN EXP PATHO, V8, P2495

Dong ZH, 2016, NEUROSCI LETT, V619, P92, DOI 10.1016/j.neulet.2016.01.008

Driver JA, 2014, BIOGERONTOLOGY, V15, P547, DOI 10.1007/s10522-014-9523-2

Fan K, 2015, J NEUROINFLAMM, V12, DOI 10.1186/s12974-015-0268-x

Gleason MK, 2014, BLOOD, V123, P3016, DOI 10.1182/blood-2013-10-533398

Heusinkveld HJ, 2016, NEUROTOXICOLOGY, V56, P94, DOI 10.1016/j.neuro.2016.07.007

Hoechst B, 2011, BLOOD, V117, P6532, DOI 10.1182/blood-2010-11-317321

Holmgaard RB, 2015, CELL REP, V13, P412, DOI 10.1016/j.celrep.2015.08.077

Jha MK, 2013, EXPERT REV PROTEOMIC, V10, P43, DOI [10.1586/EPR.12.70, 10.1586/epr.12.70]

Ji JJ, 2016, CLIN SCI, V130, P1453, DOI 10.1042/CS20160311

Joniec-Maciejak I, 2014, PHARMACOL REP, V66, P660, DOI 10.1016/j.pharep.2014.03.008

Kannan AK, 2015, J NEUROSCI, V35, P221, DOI 10.1523/JNEUROSCI.1957-14.2015

Kebir H, 2007, NAT MED, V13, P1173, DOI 10.1038/nm1651

Kim JH, 2016, J NEUROINFLAMM, V13, DOI 10.1186/s12974-016-0656-x

Kim SU, 2013, NEUROPATHOLOGY, V33, P491, DOI 10.1111/neup.12020

Landoni VI, 2016, CLIN SCI, V130, P259, DOI 10.1042/CS20150653

Levite M, 2016, ACTA PHYSIOL, V216, P42, DOI 10.1111/apha.12476

Lim S, 2016, BRAIN PATHOL, V26, P404, DOI 10.1111/bpa.12371

Limagne E, 2016, CANCER RES, V76, P5241, DOI 10.1158/0008-5472.CAN-15-3164

Murphy AC, 2010, BRAIN BEHAV IMMUN, V24, P641, DOI 10.1016/j.bbi.2010.01.014

Nuñez S, 2013, IMMUNOLOGY, V139, P61, DOI 10.1111/imm.12055

Obermeier B, 2016, HAND CLINIC, V133, P39, DOI 10.1016/B978-0-444-63432-0.00003-7

Ostanin DV, 2013, INFLAMM BOWEL DIS, V19, P2468, DOI 10.1097/MIB.0b013e3182902b11

Qiu F, 2016, INT IMMUNOPHARMACOL, V38, P31, DOI 10.1016/j.intimp.2016.05.004

Saraiva C, 2016, J CONTROL RELEASE, V235, P34, DOI 10.1016/j.jconrel.2016.05.044

Sasaki T, 2016, EXP NEUROL, V275, P220, DOI 10.1016/j.expneurol.2015.11.003

Smith AR, 2014, J LEUKOCYTE BIOL, V96, P361, DOI 10.1189/jlb.3CE0414-205R

Verschoor CP, 2013, J LEUKOCYTE BIOL, V93, P633, DOI 10.1189/jlb.0912461

Vivekanantham S, 2015, INT J NEUROSCI, V125, P717, DOI 10.3109/00207454.2014.982795

Yi HF, 2012, J IMMUNOL, V189, P4295, DOI 10.4049/jimmunol.1200086

Zhang H, 2015, CLIN IMMUNOL, V157, P175, DOI 10.1016/j.clim.2015.02.001

NR 38

TC 50

Z9 51

U1 0

U2 11

PU ELSEVIER IRELAND LTD

PI CLARE

PA ELSEVIER HOUSE, BROOKVALE PLAZA, EAST PARK SHANNON, CO, CLARE, 00000,

IRELAND

SN 0304-3940

EI 1872-7972

J9 NEUROSCI LETT

JI Neurosci. Lett.

PD MAY 1

PY 2017

VL 648

BP 21

EP 25

DI 10.1016/j.neulet.2017.03.045

PG 5

WC Neurosciences

WE Science Citation Index Expanded (SCI-EXPANDED)

SC Neurosciences & Neurology

GA EV3RO

UT WOS:000401677000004

PM 28359932

DA 2024-08-12

ER

PT J

AU Hirsch, EC

Standaert, DG

AF Hirsch, Etienne C.

Standaert, David G.

TI Ten Unsolved Questions About Neuroinflammation in Parkinson's Disease

SO MOVEMENT DISORDERS

LA English

DT Review

DE lymphocytes; microglial cells; neurodegeneration; alpha-synuclein

ID VENTRICULAR CEREBROSPINAL-FLUID; MICROGLIAL ACTIVATION;

SUBSTANTIA-NIGRA; MOUSE MODEL; REACTIVE MICROGLIA; CELL-DEATH; RISK;

NEURODEGENERATION; ALZHEIMERS; PATHWAY

AB Parkinson's disease is a progressive and debilitating disorder that has so far eluded attempts to develop disease-modifying treatment. Both epidemiological and genetic studies support a role of neuroinflammation in the pathophysiology of Parkinson's disease. Postmortem studies and experimental analyses suggest the involvement of both innate and adaptive immunity in the degenerative process. There is also some circumstantial evidence for effects of immune therapies on the disease. In the present article, we review 10 unanswered questions related to neuroinflammatory processes in Parkinson's disease with the goal of stimulating research in the field and accelerating the clinical development of neuroprotective therapies based on anti-inflammatory strategies. (c) 2020 International Parkinson and Movement Disorder Society

C1 [Hirsch, Etienne C.] Sorbonne Univ, Fac Med, INSERM,ICM, U1127,CNRS,UMR 7225,Inst Cerveau & Moelle Epinier, Paris, France.

[Standaert, David G.] Univ Alabama Birmingham, Dept Neurol, 1719 6th Ave S,CIRC 516, Birmingham, AL 35294 USA.

C3 Centre National de la Recherche Scientifique (CNRS); CNRS - National

Institute for Biology (INSB); Sorbonne Universite; Institut National de

la Sante et de la Recherche Medicale (Inserm); University of Alabama

System; University of Alabama Birmingham

RP Standaert, DG (corresponding author), Univ Alabama Birmingham, Dept Neurol, 1719 6th Ave S,CIRC 516, Birmingham, AL 35294 USA.

EM dstandaert@uab.edu

OI Hirsch, Etienne/0000-0003-4823-276X; Standaert,

David/0000-0003-2921-8348

CR Barcia C, 2005, J NEURAL TRANSM, V112, P1237, DOI 10.1007/s00702-004-0256-2

Bartels AL, 2010, PARKINSONISM RELAT D, V16, P57, DOI 10.1016/j.parkreldis.2009.05.005

Benner EJ, 2008, PLOS ONE, V3, DOI 10.1371/journal.pone.0001376

Bliederhaeuser C, 2016, ACTA NEUROPATHOL, V131, P379, DOI 10.1007/s00401-015-1504-2

BlumDegen D, 1995, NEUROSCI LETT, V202, P17, DOI 10.1016/0304-3940(95)12192-7

Braak H, 2003, NEUROBIOL AGING, V24, P197, DOI 10.1016/S0197-4580(02)00065-9

Breidert T, 2002, J NEUROCHEM, V82, P615, DOI 10.1046/j.1471-4159.2002.00990.x

Brochard V, 2009, J CLIN INVEST, V119, P182, DOI 10.1172/JCI36470

Calabrese V, 2018, FREE RADICAL BIO MED, V115, P80, DOI 10.1016/j.freeradbiomed.2017.10.379

Carvey PM, 2003, FRONT BIOSCI-LANDMRK, V8, pS826, DOI 10.2741/1158

Chahine LM, 2013, JAMA NEUROL, V70, P852, DOI 10.1001/jamaneurol.2013.1274

Faucheux BA, 1999, LANCET, V353, P981, DOI 10.1016/S0140-6736(99)00641-8

Gagliano SA, 2016, ANN CLIN TRANSL NEUR, V3, P924, DOI 10.1002/acn3.369

Gagne JJ, 2010, NEUROLOGY, V74, P995, DOI 10.1212/WNL.0b013e3181d5a4a3

Gao XA, 2011, NEUROLOGY, V76, P863, DOI 10.1212/WNL.0b013e31820f2d79

Gardai SJ, 2013, PLOS ONE, V8, DOI 10.1371/journal.pone.0071634

Gerhard A, 2006, NEUROBIOL DIS, V21, P404, DOI 10.1016/j.nbd.2005.08.002

Ghadery C, 2017, J NEUROINFLAMM, V14, DOI 10.1186/s12974-016-0778-1

Gomez-Nicola D, 2015, NEUROSCIENTIST, V21, P169, DOI 10.1177/1073858414530512

Grozdanov V, 2014, ACTA NEUROPATHOL, V128, P651, DOI 10.1007/s00401-014-1345-4

Hamza TH, 2010, NAT GENET, V42, P781, DOI 10.1038/ng.642

Harms AS, 2018, EXP NEUROL, V300, P179, DOI 10.1016/j.expneurol.2017.11.010

Harms AS, 2013, J NEUROSCI, V33, P9592, DOI 10.1523/JNEUROSCI.5610-12.2013

Hawkes CH, 2010, PARKINSONISM RELAT D, V16, P79, DOI 10.1016/j.parkreldis.2009.08.007

HIRSCH EC, 1992, ANN NEUROL, V32, pS88, DOI 10.1002/ana.410320715

Hirsch EC, 2009, LANCET NEUROL, V8, P382, DOI 10.1016/S1474-4422(09)70062-6

Holmans P, 2013, HUM MOL GENET, V22, P1039, DOI 10.1093/hmg/dds492

Horti AG, 2019, P NATL ACAD SCI USA, V116, P1686, DOI 10.1073/pnas.1812155116

Janelidze S, 2015, NEUROLOGY, V85, P1834, DOI 10.1212/WNL.0000000000002151

Johnson ME, 2019, TRENDS NEUROSCI, V42, P4, DOI 10.1016/j.tins.2018.09.007

Kannarkat GT, 2015, NPJ PARKINSON DIS, V1, DOI 10.1038/npjparkd.2015.2

Kierdorf K, 2019, NAT REV NEUROSCI, V20, P547, DOI 10.1038/s41583-019-0201-x

Kim R, 2019, MOVEMENT DISORD, V34, P1739, DOI 10.1002/mds.27841

King E, 2019, INT J GERIATR PSYCH, V34, P1244, DOI 10.1002/gps.5124

Kortekaas R, 2005, ANN NEUROL, V57, P176, DOI 10.1002/ana.20369

Langston JW, 1999, ANN NEUROL, V46, P598, DOI 10.1002/1531-8249(199910)46:4<598::AID-ANA7>3.0.CO;2-F

Lindestam Arlehamn CS, 2020, NATURE COMM

Lindqvist D, 2013, BRAIN BEHAV IMMUN, V33, P183, DOI 10.1016/j.bbi.2013.07.007

Ling ZD, 2004, EXP NEUROL, V190, P373, DOI 10.1016/j.expneurol.2004.08.006

McGeer PL, 2003, ANN NEUROL, V54, P599, DOI 10.1002/ana.10728

MCGEER PL, 1988, NEUROLOGY, V38, P1285, DOI 10.1212/WNL.38.8.1285

Michel PP, 2016, NEURON, V90, P675, DOI 10.1016/j.neuron.2016.03.038

Mogi M, 1996, NEUROSCI LETT, V211, P13, DOI 10.1016/0304-3940(96)12706-3

MOGI M, 1995, NEUROSCI LETT, V193, P129, DOI 10.1016/0304-3940(95)11686-Q

Mondello S, 2018, NEUROLOGY, V91, pE1710, DOI 10.1212/WNL.0000000000006439

Nagatsu T, 2000, ADV RES NEURODEGENER, V8, P277

Nalls MA, 2011, LANCET, V377, P641, DOI 10.1016/S0140-6736(10)62345-8

Noelker C, 2014, J NEUROINFLAMM, V11, DOI 10.1186/1742-2094-11-86

Noelker C, 2013, SCI REP-UK, V3, DOI 10.1038/srep01393

Obeso JA, 2010, NAT MED, V16, P653, DOI 10.1038/nm.2165

Ohlin KE, 2012, NEUROIMAGE, V61, P228, DOI 10.1016/j.neuroimage.2012.02.066

Ouchi Y, 2005, ANN NEUROL, V57, P168, DOI 10.1002/ana.20338

Parillaud VR, 2017, J NEUROINFLAMM, V14, DOI 10.1186/s12974-017-0830-9

Parkinson J., 1817, ESSAY SHAKING PALSY

Pereira PAB, 2017, PARKINSONISM RELAT D, V38, P61, DOI 10.1016/j.parkreldis.2017.02.026

Peter I, 2018, JAMA NEUROL, V75, P939, DOI 10.1001/jamaneurol.2018.0605

Prinz M, 2011, NAT NEUROSCI, V14, P1227, DOI 10.1038/nn.2923

Qin XY, 2016, JAMA NEUROL, V73, P1316, DOI 10.1001/jamaneurol.2016.2742

Raj T, 2014, SCIENCE, V344, P519, DOI 10.1126/science.1249547

Rasmussen MK, 2018, LANCET NEUROL, V17, P1016, DOI 10.1016/S1474-4422(18)30318-1

Raval AP, 2019, NEUROCHEM INT, V127, P29, DOI 10.1016/j.neuint.2018.11.018

Ravina B, 2006, NEUROLOGY, V66, P664

Ren L, 2018, MEDICINE, V97, DOI 10.1097/MD.0000000000012172

Reynolds AD, 2008, J NEUROCHEM, V104, P1504, DOI 10.1111/j.1471-4159.2007.05087.x

Reynolds AD, 2009, J IMMUNOL, V182, P4137, DOI 10.4049/jimmunol.0803982

Ros-Bernal F, 2011, P NATL ACAD SCI USA, V108, P6632, DOI 10.1073/pnas.1017820108

Samii A, 2009, DRUG AGING, V26, P769, DOI 10.2165/11316780-000000000-00000

Sampson TR, 2016, CELL, V167, P1469, DOI 10.1016/j.cell.2016.11.018

Saunders JAH, 2012, J NEUROIMMUNE PHARM, V7, P927, DOI 10.1007/s11481-012-9402-z

Schonhoff AM, 2020, PROG BRAIN RES, V252, P169, DOI 10.1016/bs.pbr.2019.10.006

Seok J, 2013, P NATL ACAD SCI USA, V110, P3507, DOI 10.1073/pnas.1222878110

Sommer A, 2018, CELL STEM CELL, V23, P123, DOI 10.1016/j.stem.2018.06.015

Stokholm MG, 2017, LANCET NEUROL, V16, P789, DOI 10.1016/S1474-4422(17)30173-4

Su XM, 2008, NEUROBIOL AGING, V29, P1690, DOI 10.1016/j.neurobiolaging.2007.04.006

Sulzer D, 2017, NATURE, V546, P656, DOI 10.1038/nature22815

Terada T, 2016, ANN NUCL MED, V30, P579, DOI 10.1007/s12149-016-1099-2

Tome CML, 2013, MOL NEUROBIOL, V47, P561, DOI 10.1007/s12035-012-8267-8

Valdinocci D, 2017, INT J MOL SCI, V18, DOI 10.3390/ijms18020469

Volpicelli-Daley Laura, 2018, Handb Clin Neurol, V153, P321, DOI 10.1016/B978-0-444-63945-5.00017-9

Williams-Gray CH, 2018, J NEUROINFLAMM, V15, DOI 10.1186/s12974-018-1206-5

Williams-Gray CH, 2016, MOVEMENT DISORD, V31, P995, DOI 10.1002/mds.26563

Wissemann WT, 2013, AM J HUM GENET, V93, P984, DOI 10.1016/j.ajhg.2013.10.009

Wu DC, 2002, J NEUROSCI, V22, P1763, DOI 10.1523/JNEUROSCI.22-05-01763.2002

Zecca L, 2008, ACTA NEUROPATHOL, V116, P47, DOI 10.1007/s00401-008-0361-7

Zhang MM, 2017, NEUROSCIENCE, V340, P398, DOI 10.1016/j.neuroscience.2016.11.004

Zhu YG, 2007, NEUROCHEM INT, V50, P671, DOI 10.1016/j.neuint.2006.12.013

NR 86

TC 126

Z9 131

U1 6

U2 33

PU WILEY

PI HOBOKEN

PA 111 RIVER ST, HOBOKEN 07030-5774, NJ USA

SN 0885-3185

EI 1531-8257

J9 MOVEMENT DISORD

JI Mov. Disord.

PD JAN

PY 2021

VL 36

IS 1

BP 16

EP 24

DI 10.1002/mds.28075

EA MAY 2020

PG 9

WC Clinical Neurology

WE Science Citation Index Expanded (SCI-EXPANDED)

SC Neurosciences & Neurology

GA PX3BR

UT WOS:000529675100001

PM 32357266

OA Green Submitted

DA 2024-08-12

ER

PT J

AU Russo, I

Bubacco, L

Greggio, E

AF Russo, Isabella

Bubacco, Luigi

Greggio, Elisa

TI LRRK2 and neuroinflammation: partners in crime in Parkinson's disease?

SO JOURNAL OF NEUROINFLAMMATION

LA English

DT Review

DE LRRK2; Neuroinflammation; Microglia; Neurodegeneration; Parkinson's

disease; Dopaminergic neurons

ID MIDBRAIN DOPAMINERGIC-NEURONS; SUBSTANTIA-NIGRA; HIPPOCAMPAL

NEUROGENESIS; SUSCEPTIBILITY LOCI; ACTIN CYTOSKELETON; GENE

POLYMORPHISMS; ADHESION DYNAMICS; MICROGLIAL CELLS; KINASE-ACTIVITY;

GTP-BINDING

AB It is now well established that chronic inflammation is a prominent feature of several neurodegenerative disorders including Parkinson's disease (PD). Growing evidence indicates that neuroinflammation can contribute greatly to dopaminergic neuron degeneration and progression of the disease. Recent literature highlights that leucine-rich repeat kinase 2 (LRRK2), a kinase mutated in both autosomal-dominantly inherited and sporadic PD cases, modulates inflammation in response to different pathological stimuli. In this review, we outline the state of the art of LRRK2 functions in microglia cells and in neuroinflammation. Furthermore, we discuss the potential role of LRRK2 in cytoskeleton remodeling and vesicle trafficking in microglia cells under physiological and pathological conditions. We also hypothesize that LRRK2 mutations might sensitize microglia cells toward a pro-inflammatory state, which in turn results in exacerbated inflammation with consequent neurodegeneration.

C1 [Russo, Isabella; Bubacco, Luigi; Greggio, Elisa] Univ Padua, Dept Biol, I-35121 Padua, Italy.

C3 University of Padua

RP Russo, I (corresponding author), Univ Padua, Dept Biol, Via Ugo Bassi 58-B, I-35121 Padua, Italy.

EM isabella.russo@unipd.it; elisa.greggio@unipd.it

RI Russo, Isabella/AAB-4882-2020; Bubacco, Luigi/B-5602-2012; Greggio,

Elisa/H-6119-2013

OI Russo, Isabella/0000-0002-6151-2451; Bubacco, Luigi/0000-0001-7927-9208;

FU Michael J. Fox Foundation for Parkinson's Disease; Italian Ministry of

University and Research (Rientro dei Cervelli, lncentivazione alla

mobilita di studiosi stranieri e italiani residenti all'estero); CARIPLO

Foundation [2011-0540]; Telethon - Italy [GGP12237]

FX This work was supported by the Michael J. Fox Foundation for Parkinson's

Disease, the Italian Ministry of University and Research (Rientro dei

Cervelli, lncentivazione alla mobilita di studiosi stranieri e italiani

residenti all'estero to EG), and the CARIPLO Foundation (grant number

2011-0540). The financial support of Telethon - Italy (grant number

GGP12237) is gratefully acknowledged.

CR Abd-El-Basset EM, 2011, ANAT REC, V294, P16, DOI 10.1002/ar.21303

Ahmed I, 2012, MOVEMENT DISORD, V27, P1104, DOI 10.1002/mds.25035

Alegre-Abarrategui J, 2009, HUM MOL GENET, V18, P4022, DOI 10.1093/hmg/ddp346

Aloisi F, 2001, GLIA, V36, P165, DOI 10.1002/glia.1106

Anand VS, 2009, FEBS J, V276, P466, DOI 10.1111/j.1742-4658.2008.06789.x

Barcia C, 2012, SCI REP-UK, V2, DOI 10.1038/srep00809

Barrett JC, 2008, NAT GENET, V40, P955, DOI 10.1038/ng.175

Belluzzi E, 2012, BIOCHEM SOC T, V40, P1111, DOI 10.1042/BST20120124

Berwick DC, 2011, TRENDS CELL BIOL, V21, P257, DOI 10.1016/j.tcb.2011.01.001

Bialecka M, 2008, PARKINSONISM RELAT D, V14, P636, DOI 10.1016/j.parkreldis.2008.02.001

Biskup S, 2006, ANN NEUROL, V60, P557, DOI 10.1002/ana.21019

Bosgraaf L, 2003, BBA-MOL CELL RES, V1643, P5, DOI 10.1016/j.bbamcr.2003.08.008

Bucci Cecilia, 2010, Commun Integr Biol, V3, P401, DOI 10.4161/cib.3.5.12341

Caesar M, 2013, NEUROBIOL DIS, V54, P280, DOI 10.1016/j.nbd.2012.12.019

Castellano F, 2001, SEMIN IMMUNOL, V13, P347, DOI 10.1006/smim.2001.0331

Choi SH, 2008, FASEB J, V22, P1491, DOI 10.1096/fj.07-9411com

Chung CY, 2005, HUM MOL GENET, V14, P1709, DOI 10.1093/hmg/ddi178

Cookson MR, 2010, NAT REV NEUROSCI, V11, P791, DOI 10.1038/nrn2935

Cross AK, 1999, J NEUROSCI RES, V55, P17, DOI 10.1002/(SICI)1097-4547(19990101)55:1<17::AID-JNR3>3.0.CO;2-J

Daniëls V, 2011, J NEUROCHEM, V116, P304, DOI 10.1111/j.1471-4159.2010.07105.x

Dodson MW, 2012, HUM MOL GENET, V21, P1350, DOI 10.1093/hmg/ddr573

Dzamko N, 2012, PLOS ONE, V7, DOI 10.1371/journal.pone.0039132

Faff L, 2000, BRAIN RES, V853, P22, DOI 10.1016/S0006-8993(99)02221-0

Funayama M, 2005, ANN NEUROL, V57, P918, DOI 10.1002/ana.20484

Gandhi PN, 2008, J NEUROSCI RES, V86, P1711, DOI 10.1002/jnr.21622

Gao HM, 2008, TRENDS IMMUNOL, V29, P357, DOI 10.1016/j.it.2008.05.002

Gardel ML, 2010, ANNU REV CELL DEV BI, V26, P315, DOI 10.1146/annurev.cellbio.011209.122036

Garden GA, 2006, J NEUROIMMUNE PHARM, V1, P127, DOI 10.1007/s11481-006-9015-5

Ghosh A, 2007, P NATL ACAD SCI USA, V104, P18754, DOI 10.1073/pnas.0704908104

Giesert F, 2013, PLOS ONE, V8, DOI 10.1371/journal.pone.0063778

Gilks WP, 2005, LANCET, V365, P415, DOI 10.1016/S0140-6736(05)17830-1

Gillardon F, 2012, NEUROSCIENCE, V208, P41, DOI 10.1016/j.neuroscience.2012.02.001

Gillardon F, 2009, J NEUROCHEM, V110, P1514, DOI 10.1111/j.1471-4159.2009.06235.x

Gloeckner CJ, 2006, HUM MOL GENET, V15, P223, DOI 10.1093/hmg/ddi439

Goldwurm S, 2005, J MED GENET, V42, DOI 10.1136/jmg.2005.035568

Gómez-Suaga P, 2012, PARKINSONS DIS-US, V2012, DOI 10.1155/2012/324521

Gómez-Suaga P, 2012, HUM MOL GENET, V21, P511, DOI 10.1093/hmg/ddr481

Greggio E, 2009, ASN NEURO, V1, DOI 10.1042/AN20090007

Hakimi M, 2011, J NEURAL TRANSM, V118, P795, DOI 10.1007/s00702-011-0653-2

Hamza TH, 2010, NAT GENET, V42, P781, DOI 10.1038/ng.642

Han BS, 2008, NEUROSCI LETT, V442, P190, DOI 10.1016/j.neulet.2008.06.086

Haugarvoll K, 2008, NEUROLOGY, V70, P1456, DOI 10.1212/01.wnl.0000304044.22253.03

Hirsch EC, 2009, LANCET NEUROL, V8, P382, DOI 10.1016/S1474-4422(09)70062-6

Ho CCY, 2009, J NEUROSCI, V29, P1011, DOI 10.1523/JNEUROSCI.5175-08.2009

HOLMES KC, 1990, NATURE, V347, P44, DOI 10.1038/347044a0

Hurley SD, 2003, EXP NEUROL, V184, P659, DOI 10.1016/S0014-4886(03)00273-5

Imamura K, 2003, ACTA NEUROPATHOL, V106, P518, DOI 10.1007/s00401-003-0766-2

Ito G, 2007, BIOCHEMISTRY-US, V46, P1380, DOI 10.1021/bi061960m

Jaleel M, 2007, BIOCHEM J, V405, P307, DOI 10.1042/BJ20070209

Jenner P, 1998, ANN NEUROL, V44, pS72, DOI 10.1002/ana.410440712

Kawakami F, 2012, PLOS ONE, V7, DOI 10.1371/journal.pone.0030834

Kett LR, 2012, HUM MOL GENET, V21, P890, DOI 10.1093/hmg/ddr526

Kim B, 2012, PLOS ONE, V7, DOI 10.1371/journal.pone.0034693

Kumari U, 2009, FEBS J, V276, P6455, DOI 10.1111/j.1742-4658.2009.07344.x

Langston JW, 1999, ANN NEUROL, V46, P598, DOI 10.1002/1531-8249(199910)46:4<598::AID-ANA7>3.0.CO;2-F

Law BMH, 2014, J BIOL CHEM, V289, P895, DOI 10.1074/jbc.M113.507913

Lee M, 2013, CURR PROTEIN PEPT SC, V14, P21, DOI 10.2174/1389203711314010005

Li YP, 2009, NAT NEUROSCI, V12, P826, DOI 10.1038/nn.2349

Li YC, 2009, BBA-MOL BASIS DIS, V1792, P1194, DOI 10.1016/j.bbadis.2009.09.010

Lin X, 2009, NEURON, V64, P807, DOI 10.1016/j.neuron.2009.11.006

Liu ZH, 2011, NAT IMMUNOL, V12, P1063, DOI 10.1038/ni.2113

de Maturana RL, 2014, NEUROBIOL AGING, V35, P1116, DOI 10.1016/j.neurobiolaging.2013.11.018

Lucin KM, 2009, NEURON, V64, P110, DOI 10.1016/j.neuron.2009.08.039

Luzón-Toro B, 2007, HUM MOL GENET, V16, P2031, DOI 10.1093/hmg/ddm151

MacLeod D, 2006, NEURON, V52, P587, DOI 10.1016/j.neuron.2006.10.008

MacLeod DA, 2013, NEURON, V77, P425, DOI 10.1016/j.neuron.2012.11.033

Manzoni C, 2013, BBA-MOL CELL RES, V1833, P2900, DOI 10.1016/j.bbamcr.2013.07.020

Marín I, 2006, MOL BIOL EVOL, V23, P2423, DOI 10.1093/molbev/msl114

Marker DF, 2012, J NEUROINFLAMM, V9, DOI 10.1186/1742-2094-9-261

Matta S, 2012, NEURON, V75, P1008, DOI 10.1016/j.neuron.2012.08.022

MCGEER PL, 1988, NEUROLOGY, V38, P1285, DOI 10.1212/WNL.38.8.1285

Meixner A, 2011, MOL CELL PROTEOMICS, V10, DOI 10.1074/mcp.M110.001172

Migheli R, 2013, PLOS ONE, V8, DOI 10.1371/journal.pone.0077198

Miklossy J, 2006, J NEUROPATH EXP NEUR, V65, P953, DOI 10.1097/01.jnen.0000235121.98052.54

Moehle MS, 2012, J NEUROSCI, V32, P1602, DOI 10.1523/JNEUROSCI.5601-11.2012

MOGI M, 1994, NEUROSCI LETT, V165, P208, DOI 10.1016/0304-3940(94)90746-3

Mogi M, 2000, J NEURAL TRANSM, V107, P335, DOI 10.1007/s007020050028

Neumann H, 2009, BRAIN, V132, P288, DOI 10.1093/brain/awn109

Nimmerjahn A, 2005, SCIENCE, V308, P1314, DOI 10.1126/science.1110647

Ohsawa K, 2011, GLIA, V59, P1793, DOI 10.1002/glia.21238

Orenstein SJ, 2013, NAT NEUROSCI, V16, P394, DOI 10.1038/nn.3350

Ouchi Y, 2009, PARKINSONISM RELAT D, V15, pS200, DOI 10.1016/S1353-8020(09)70814-4

Paisán-Ruíz C, 2004, NEURON, V44, P595, DOI 10.1016/j.neuron.2004.10.023

Papkovskaia TD, 2012, HUM MOL GENET, V21, P4201, DOI 10.1093/hmg/dds244

Parisiadou Loukia, 2010, Commun Integr Biol, V3, P396, DOI 10.4161/cib.3.5.12286

Parisiadou L, 2009, J NEUROSCI, V29, P13971, DOI 10.1523/JNEUROSCI.3799-09.2009

Piccoli G, 2011, J NEUROSCI, V31, P2225, DOI 10.1523/JNEUROSCI.3730-10.2011

Plowey ED, 2008, J NEUROCHEM, V105, P1048, DOI 10.1111/j.1471-4159.2008.05217.x

Pradhan S, 2013, EXP NEUROL, V241, P148, DOI 10.1016/j.expneurol.2012.12.008

Ravikumar B, 2008, J CELL SCI, V121, P1649, DOI 10.1242/jcs.025726

Russo Isabella, 2012, Am J Neurodegener Dis, V1, P217

Russo I, 2012, J NEUROCHEM, V122, P1181, DOI 10.1111/j.1471-4159.2012.07846.x

Russo I, 2011, CELL CYCLE, V10, P2568, DOI 10.4161/cc.10.15.15946

Russo I, 2011, J NEUROCHEM, V116, P947, DOI 10.1111/j.1471-4159.2010.07168.x

Sepulveda B, 2013, PLOS ONE, V8, DOI 10.1371/journal.pone.0061986

Sheng ZJ, 2012, SCI TRANSL MED, V4, DOI 10.1126/scitranslmed.3004485

Shin N, 2008, EXP CELL RES, V314, P2055, DOI 10.1016/j.yexcr.2008.02.015

Simón-Sánchez J, 2009, NAT GENET, V41, P1308, DOI 10.1038/ng.487

Small JV, 2002, TRENDS CELL BIOL, V12, P112, DOI 10.1016/S0962-8924(01)02237-1

Sriram K, 2006, J NEUROCHEM, V96, P706, DOI 10.1111/j.1471-4159.2005.03566.x

Stanley AC, 2010, PHYSIOLOGY, V25, P218, DOI 10.1152/physiol.00017.2010

Stow JL, 2006, NAT REV IMMUNOL, V6, P919, DOI 10.1038/nri1980

Stow JL, 2009, IMMUNOBIOLOGY, V214, P601, DOI 10.1016/j.imbio.2008.11.005

Stuart LM, 2005, IMMUNITY, V22, P539, DOI 10.1016/j.immuni.2005.05.002

Stuart LM, 2007, J BIOL CHEM, V282, P27392, DOI 10.1074/jbc.M702887200

Swanson JA, 1999, J CELL SCI, V112, P307

Swanson JA, 2008, NAT REV MOL CELL BIO, V9, P639, DOI 10.1038/nrm2447

Tansey MG, 2010, NEUROBIOL DIS, V37, P510, DOI 10.1016/j.nbd.2009.11.004

Taymans JM, 2011, PLOS ONE, V6, DOI 10.1371/journal.pone.0023207

Thévenet J, 2011, PLOS ONE, V6, DOI 10.1371/journal.pone.0021519

Umeno J, 2011, INFLAMM BOWEL DIS, V17, P2407, DOI 10.1002/ibd.21651

Wahner AD, 2007, ARCH NEUROL-CHICAGO, V64, P836, DOI 10.1001/archneur.64.6.836

Wang XL, 2012, HUM MOL GENET, V21, P1931, DOI 10.1093/hmg/dds003

Wehrle-Haller B, 2003, INT J BIOCHEM CELL B, V35, P39, DOI 10.1016/S1357-2725(02)00071-7

Welch MD, 2002, ANNU REV CELL DEV BI, V18, P247, DOI 10.1146/annurev.cellbio.18.040202.112133

West AB, 2005, P NATL ACAD SCI USA, V102, P16842, DOI 10.1073/pnas.0507360102

West AB, 2007, HUM MOL GENET, V16, P223, DOI 10.1093/hmg/ddl471

Whitton PS, 2007, BRIT J PHARMACOL, V150, P963, DOI 10.1038/sj.bjp.0707167

Xiong YL, 2012, BIOCHEM SOC T, V40, P1074, DOI 10.1042/BST20120093

Xiong YL, 2010, PLOS GENET, V6, DOI 10.1371/journal.pgen.1000902

Yun HJ, 2013, EXP MOL MED, V45, DOI 10.1038/emm.2013.68

Zhang FR, 2009, NEW ENGL J MED, V361, P2609, DOI 10.1056/NEJMoa0903753

Zimprich A, 2004, NEURON, V44, P601, DOI 10.1016/j.neuron.2004.11.005

NR 123

TC 129

Z9 141

U1 4

U2 25

PU BMC

PI LONDON

PA CAMPUS, 4 CRINAN ST, LONDON N1 9XW, United Kingdom

SN 1742-2094

J9 J NEUROINFLAMM

JI J. Neuroinflamm.

PD MAR 21

PY 2014

VL 11

AR 52

DI 10.1186/1742-2094-11-52

PG 9

WC Immunology; Neurosciences

WE Science Citation Index Expanded (SCI-EXPANDED)

SC Immunology; Neurosciences & Neurology

GA AF8DM

UT WOS:000334945600002

PM 24655756

OA gold, Green Published

DA 2024-08-12

ER

PT J

AU Araújo, B

Caridade-Silva, R

Soares-Guedes, C

Martins-Macedo, J

Gomes, ED

Monteiro, S

Teixeira, FG

AF Araujo, Bruna

Caridade-Silva, Rita

Soares-Guedes, Carla

Martins-Macedo, Joana

Gomes, Eduardo D.

Monteiro, Susana

Teixeira, Fabio G.

TI Neuroinflammation and Parkinson's Disease-From Neurodegeneration to

Therapeutic Opportunities

SO CELLS

LA English

DT Review

DE Parkinson's disease; neuroinflammation; inflammatory cells; acute and

chronic responses; gut-brain axis

ID BRAIN-BARRIER PERMEABILITY; REGULATORY T-CELLS; VENTRICULAR

CEREBROSPINAL-FLUID; SYNUCLEIN ACTIVATES MICROGLIA; AGGREGATED

ALPHA-SYNUCLEIN; GROWTH-FACTOR-ALPHA; MOUSE MODEL; GUT MICROBIOTA; HUMAN

ASTROCYTES; OLFACTORY-BULB

AB Parkinson's disease (PD) is the second most prevalent neurodegenerative disorder worldwide. Clinically, it is characterized by a progressive degeneration of dopaminergic neurons (DAn), resulting in severe motor complications. Preclinical and clinical studies have indicated that neuroinflammation can play a role in PD pathophysiology, being associated with its onset and progression. Nevertheless, several key points concerning the neuroinflammatory process in PD remain to be answered. Bearing this in mind, in the present review, we cover the impact of neuroinflammation on PD by exploring the role of inflammatory cells (i.e., microglia and astrocytes) and the interconnections between the brain and the peripheral system. Furthermore, we discuss both the innate and adaptive immune responses regarding PD pathology and explore the gut-brain axis communication and its influence on the progression of the disease.

C1 [Araujo, Bruna; Caridade-Silva, Rita; Monteiro, Susana; Teixeira, Fabio G.] Univ Minho, Sch Med, Life & Hlth Sci Res Inst ICVS, P-4710057 Braga, Portugal.

[Araujo, Bruna; Caridade-Silva, Rita; Monteiro, Susana; Teixeira, Fabio G.] ICVS 3Bs PT Govt Associate Lab, P-4805017 Braga, Portugal.

[Araujo, Bruna; Caridade-Silva, Rita; Soares-Guedes, Carla; Martins-Macedo, Joana; Gomes, Eduardo D.; Teixeira, Fabio G.] Porto Polytech Inst, Med & Ind Biotechnol Lab LABMI, Porto Res Technol & Innovat Ctr PORTIC, P-4200375 Porto, Portugal.

[Araujo, Bruna; Caridade-Silva, Rita; Soares-Guedes, Carla; Martins-Macedo, Joana; Gomes, Eduardo D.; Teixeira, Fabio G.] Univ Porto, I3S Inst Invest & Inovacao Saude, P-4200135 Porto, Portugal.

C3 Universidade do Minho; Instituto Politecnico do Porto; Universidade do

Porto; i3S - Instituto de Investigacao e Inovacao em Saude, Universidade

do Porto

RP Teixeira, FG (corresponding author), Univ Minho, Sch Med, Life & Hlth Sci Res Inst ICVS, P-4710057 Braga, Portugal.; Teixeira, FG (corresponding author), ICVS 3Bs PT Govt Associate Lab, P-4805017 Braga, Portugal.; Teixeira, FG (corresponding author), Porto Polytech Inst, Med & Ind Biotechnol Lab LABMI, Porto Res Technol & Innovat Ctr PORTIC, P-4200375 Porto, Portugal.; Teixeira, FG (corresponding author), Univ Porto, I3S Inst Invest & Inovacao Saude, P-4200135 Porto, Portugal.

EM fteixeira@i3s.up.pt

RI Gomes, Eduardo/HPC-9065-2023; Gomes, Eduardo D./ABC-9041-2020; Teixeira,

Fabio Miguel da Cruz/JHV-1347-2023; Monteiro, Susana/G-6965-2015;

Martins-Macedo, Joana/JNT-6145-2023; Guedes, Carla Soares/D-3704-2017;

Monteiro, Susana/AAC-7090-2020; Teixeira, Fabio/F-8204-2012

OI Teixeira, Fabio Miguel da Cruz/0000-0003-0497-995X; Monteiro,

Susana/0000-0002-3037-9635; Martins-Macedo, Joana/0000-0003-0442-927X;

Guedes, Carla Soares/0000-0002-3277-7821; Gomes,

Eduardo/0000-0003-3179-1741; Teixeira, Fabio/0000-0003-1461-9077;

Araujo, Bruna/0000-0002-7365-0077; Caridade Silva,

Rita/0000-0003-4819-6359

FU Premios Santa Casa Neurociencias-Prize Mantero Belard for

neurodegenerative disease Research [MB-28-2019]; Portuguese Foundation

for Science and Technology (FCT) [CEECIND/01902/2017,

2021.00643.CEECIND]; European Regional Development Fund (FEDER);

Competitiveness Internationalization Operational Programme (POCI);

national funds through the Foundation for Science and Technology (FCT)

[UIDB/50026/2020, UIDP/50026/2020, POCI-01-0145-FEDER-029751,

EXPL/MED-PAT/0931/2021, NORTE-01-0145-FEDER-000023]; Northern Portugal

Regional Operational Programme (NORTE 2020); Portugal 2020 Partnership

Agreement, through the European Regional Development Fund (FEDER)

FX The present work was supported by Premios Santa Casa Neurociencias-Prize

Mantero Belard for neurodegenerative disease Research (MB-28-2019), the

Portuguese Foundation for Science and Technology (FCT) to S.M.

(CEECIND/01902/2017) and F.G.T. (2021.00643.CEECIND), the European

Regional Development Fund (FEDER), the Competitiveness

Internationalization Operational Programme (POCI), national funds

through the Foundation for Science and Technology (FCT), under the scope

of projects UIDB/50026/2020, UIDP/50026/2020, POCI-01-0145-FEDER-029751,

and EXPL/MED-PAT/0931/2021, project NORTE-01-0145-FEDER-000023,

supported by the Northern Portugal Regional Operational Programme (NORTE

2020), and under the Portugal 2020 Partnership Agreement, through the

European Regional Development Fund (FEDER).

CR Abbott NJ, 2002, J ANAT, V200, P629, DOI 10.1046/j.1469-7580.2002.00064.x

Aguzzi A, 2003, BRIT MED BULL, V66, P141, DOI 10.1093/bmb/66.1.141

Ahlskog JE, 2011, NEUROLOGY, V77, P288, DOI 10.1212/WNL.0b013e318225ab66

Akira S, 2006, CELL, V124, P783, DOI 10.1016/j.cell.2006.02.015

Al-Bachari S, 2017, J CEREBR BLOOD F MET, V37, P3409, DOI 10.1177/0271678X16688919

Ambrosi G, 2017, NEUROTOX RES, V32, P71, DOI 10.1007/s12640-017-9712-2

Argaw AT, 2006, J IMMUNOL, V177, P5574, DOI 10.4049/jimmunol.177.8.5574

Argaw AT, 2012, J CLIN INVEST, V122, P2454, DOI 10.1172/JCI60842

Argaw AT, 2009, P NATL ACAD SCI USA, V106, P1977, DOI 10.1073/pnas.0808698106

Armentero MT, 2006, NEUROBIOL DIS, V24, P492, DOI 10.1016/j.nbd.2006.08.016

Baba Y, 2005, PARKINSONISM RELAT D, V11, P493, DOI 10.1016/j.parkreldis.2005.07.005

Bae EJ, 2012, J NEUROSCI, V32, P13454, DOI 10.1523/JNEUROSCI.1292-12.2012

Barcia C, 2005, J NEURAL TRANSM, V112, P1237, DOI 10.1007/s00702-004-0256-2

Bas J, 2001, J NEUROIMMUNOL, V113, P146, DOI 10.1016/S0165-5728(00)00422-7

Beach TG, 2009, ACTA NEUROPATHOL, V117, P169, DOI 10.1007/s00401-008-0450-7

Belloli S, 2020, FRONT AGING NEUROSCI, V12, DOI 10.3389/fnagi.2020.00152

Benner EJ, 2008, PLOS ONE, V3, DOI 10.1371/journal.pone.0001376

Bercik P, 2011, GASTROENTEROLOGY, V141, P599, DOI 10.1053/j.gastro.2011.04.052

Bergström AL, 2016, MOVEMENT DISORD, V31, P203, DOI 10.1002/mds.26481

Bialecka M, 2007, NEUROSCI RES, V57, P473, DOI 10.1016/j.neures.2006.11.012

Björklund A, 2007, TRENDS NEUROSCI, V30, P194, DOI 10.1016/j.tins.2007.03.006

Blandini F, 2003, ANN NY ACAD SCI, V1010, P675, DOI 10.1196/annals.1299.123

Blauwendraat C, 2020, LANCET NEUROL, V19, P170, DOI 10.1016/S1474-4422(19)30287-X

Bliederhaeuser C, 2016, ACTA NEUROPATHOL, V131, P379, DOI 10.1007/s00401-015-1504-2

Block ML, 2007, NAT REV NEUROSCI, V8, P57, DOI 10.1038/nrn2038

Böttner M, 2012, NEUROBIOL DIS, V48, P474, DOI 10.1016/j.nbd.2012.07.018

Booth HDE, 2017, TRENDS NEUROSCI, V40, P358, DOI 10.1016/j.tins.2017.04.001

Borah A, 2010, BRAIN RES, V1344, P192, DOI 10.1016/j.brainres.2010.05.010

Bousquet M, 2008, FASEB J, V22, P1213, DOI 10.1096/fj.07-9677com

Bousquet M, 2011, AGEING RES REV, V10, P453, DOI 10.1016/j.arr.2011.03.001

Braak H, 2006, NEUROSCI LETT, V396, P67, DOI 10.1016/j.neulet.2005.11.012

Braak H, 2003, J NEURAL TRANSM, V110, P517, DOI 10.1007/s00702-002-0808-2

Braak H, 2007, ACTA NEUROPATHOL, V114, P231, DOI 10.1007/s00401-007-0244-3

Bradaric BD, 2012, J NEURAL TRANSM, V119, P59, DOI 10.1007/s00702-011-0684-8

Brochard V, 2009, J CLIN INVEST, V119, P182, DOI 10.1172/JCI36470

Brunialti E, 2021, J NEUROINFLAMM, V18, DOI 10.1186/s12974-021-02272-2

Butler CA, 2021, J NEUROCHEM, V158, P621, DOI 10.1111/jnc.15327

Caggiu E, 2019, FRONT NEUROL, V10, DOI 10.3389/fneur.2019.00122

Cankaya Seyda, 2019, Drugs Context, V8, P212553, DOI 10.7573/dic.212553

Carvey PM, 2005, EUR J NEUROSCI, V22, P1158, DOI 10.1111/j.1460-9568.2005.04281.x

Castaño A, 1998, J NEUROCHEM, V70, P1584

Cebrián C, 2014, NAT COMMUN, V5, DOI 10.1038/ncomms4633

Chapelet G, 2019, PARKINSONISM RELAT D, V59, P26, DOI 10.1016/j.parkreldis.2018.11.014

Chen DF, 2013, NEUROSCI LETT, V538, P49, DOI 10.1016/j.neulet.2013.01.044

Chen HL, 2015, TRANSL NEURODEGENER, V4, DOI 10.1186/2047-9158-4-1

Chen XS, 2008, J NEUROCHEM, V107, P1147, DOI 10.1111/j.1471-4159.2008.05697.x

Chen ZH, 2016, J NEUROCHEM, V136, P10, DOI 10.1111/jnc.13062

Chen ZM, 2013, NEUROL SCI, V34, P1559, DOI 10.1007/s10072-012-1284-6

Chhor V, 2013, BRAIN BEHAV IMMUN, V32, P70, DOI 10.1016/j.bbi.2013.02.005

Chinta SJ, 2018, CELL REP, V22, P930, DOI 10.1016/j.celrep.2017.12.092

Choi JG, 2018, SCI REP-UK, V8, DOI 10.1038/s41598-018-19646-x

Chui R, 2010, J NEUROINFLAMM, V7, DOI 10.1186/1742-2094-7-1

Codolo G, 2013, PLOS ONE, V8, DOI 10.1371/journal.pone.0055375

Coll RC, 2019, NAT CHEM BIOL, V15, P556, DOI 10.1038/s41589-019-0277-7

Couch Y, 2011, J NEUROINFLAMM, V8, DOI 10.1186/1742-2094-8-166

Croisier E, 2005, J NEUROINFLAMM, V2, DOI 10.1186/1742-2094-2-14

Crotti A, 2016, IMMUNITY, V44, P505, DOI 10.1016/j.immuni.2016.02.013

D'Mello C, 2009, J NEUROSCI, V29, P2089, DOI 10.1523/JNEUROSCI.3567-08.2009

Davalos D, 2005, NAT NEUROSCI, V8, P752, DOI 10.1038/nn1472

De Biase LM, 2017, NEURON, V95, P341, DOI 10.1016/j.neuron.2017.06.020

Devos D, 2013, NEUROBIOL DIS, V50, P42, DOI 10.1016/j.nbd.2012.09.007

Didier N, 2003, J NEUROCHEM, V86, P246, DOI 10.1046/j.1471-4159.2003.01829.x

Diniz LP, 2020, NEUROCHEM INT, V138, DOI 10.1016/j.neuint.2020.104758

Dobbs RJ, 1999, ACTA NEUROL SCAND, V100, P34, DOI 10.1111/j.1600-0404.1999.tb00721.x

Dohgu S, 2019, MICROVASC RES, V124, P61, DOI 10.1016/j.mvr.2019.03.005

Doty RL, 2012, NEUROBIOL DIS, V46, P527, DOI 10.1016/j.nbd.2011.10.026

Doursout MF, 2013, J INTERF CYTOK RES, V33, P376, DOI 10.1089/jir.2012.0088

Drobny A, 2021, FRONT MED-LAUSANNE, V8, DOI 10.3389/fmed.2021.655123

El-Agnaf OMA, 2003, FASEB J, V17, P1945, DOI 10.1096/fj.03-0098fje

Erny D, 2015, NAT NEUROSCI, V18, P965, DOI 10.1038/nn.4030

Fan Z, 2020, J NEUROINFLAMM, V17, DOI 10.1186/s12974-019-1670-6

Ferreira SA, 2018, FRONT CELL NEUROSCI, V12, DOI 10.3389/fncel.2018.00247

FISZER U, 1994, ACTA NEUROL SCAND, V90, P160

Flores-Cuadrado A, 2021, NPJ PARKINSONS DIS, V7, DOI 10.1038/s41531-020-00154-7

Fu QZ, 2017, IUBMB LIFE, V69, P887, DOI 10.1002/iub.1685

Fuzzati-Armentero MT, 2019, FRONT NEUROL, V10, DOI 10.3389/fneur.2019.00232

Games D, 2014, J NEUROSCI, V34, P9441, DOI 10.1523/JNEUROSCI.5314-13.2014

Gao HM, 2011, ENVIRON HEALTH PERSP, V119, P807, DOI 10.1289/ehp.1003013

Gao X, 2012, NEUROLOGY, V78, P1138, DOI 10.1212/WNL.0b013e31824f7fc4

Gardai SJ, 2013, PLOS ONE, V8, DOI 10.1371/journal.pone.0071634

Garrido-Gil P, 2018, MOL NEUROBIOL, V55, P7297, DOI 10.1007/s12035-018-0937-8

Gelders G, 2018, J IMMUNOL RES, V2018, DOI 10.1155/2018/4784268

Gendelman HE, 2017, NPJ PARKINSONS DIS, V3, DOI 10.1038/s41531-017-0013-5

George S, 2019, MOL NEURODEGENER, V14, DOI 10.1186/s13024-019-0335-3

Gerhard A, 2006, NEUROBIOL DIS, V21, P404, DOI 10.1016/j.nbd.2005.08.002

Glezer I, 2007, NEUROSCIENCE, V147, P867, DOI 10.1016/j.neuroscience.2007.02.055

Gold A, 2013, MOVEMENT DISORD, V28, P237, DOI 10.1002/mds.25298

Gordon R, 2018, SCI TRANSL MED, V10, DOI 10.1126/scitranslmed.aah4066

Gosselin D, 2017, SCIENCE, V356, DOI 10.1126/science.aal3222

Gray MT, 2015, J CEREBR BLOOD F MET, V35, P747, DOI 10.1038/jcbfm.2015.32

Grozdanov V, 2014, ACTA NEUROPATHOL, V128, P651, DOI 10.1007/s00401-014-1345-4

Gu XL, 2010, MOL BRAIN, V3, DOI 10.1186/1756-6606-3-12

Gustot A, 2015, BIOCHEM J, V471, P323, DOI 10.1042/BJ20150617

Hammond TR, 2019, IMMUNITY, V50, P253, DOI 10.1016/j.immuni.2018.11.004

Hanisch UK, 2002, GLIA, V40, P140, DOI 10.1002/glia.10161

Harms AS, 2021, ACTA NEUROPATHOL, V141, P527, DOI 10.1007/s00401-021-02268-5

Harms AS, 2018, EXP NEUROL, V300, P179, DOI 10.1016/j.expneurol.2017.11.010

Harms AS, 2013, J NEUROSCI, V33, P9592, DOI 10.1523/JNEUROSCI.5610-12.2013

He MF, 2016, CELL PHYSIOL BIOCHEM, V38, P859, DOI 10.1159/000443040

Heijtza RD, 2011, P NATL ACAD SCI USA, V108, P3047, DOI 10.1073/pnas.1010529108

Heintz-Buschart A, 2018, MOVEMENT DISORD, V33, P88, DOI 10.1002/mds.27105

Heneka MT, 2018, NAT REV NEUROSCI, V19, P610, DOI 10.1038/s41583-018-0055-7

Henry CJ, 2008, J NEUROINFLAMM, V5, DOI 10.1186/1742-2094-5-15

Hijaz BA, 2020, MOL NEURODEGENER, V15, DOI 10.1186/s13024-020-00368-6

Holmqvist S, 2014, ACTA NEUROPATHOL, V128, P805, DOI 10.1007/s00401-014-1343-6

Houser MC, 2017, NPJ PARKINSONS DIS, V3, DOI 10.1038/s41531-016-0002-0

Huang SX, 2021, J NEUROIMMUNOL, V354, DOI 10.1016/j.jneuroim.2021.577543

Huarte OU, 2021, FRONT IMMUNOL, V12, DOI 10.3389/fimmu.2021.639613

Hui KY, 2018, SCI TRANSL MED, V10, DOI 10.1126/scitranslmed.aai7795

Iglesias J, 2017, MOL NEUROBIOL, V54, P2518, DOI 10.1007/s12035-016-9833-2

Imamura K, 2003, ACTA NEUROPATHOL, V106, P518, DOI 10.1007/s00401-003-0766-2

Jackson A, 2019, FRONT NEUROL, V10, DOI 10.3389/fneur.2019.01245

Janda E, 2018, FRONT MOL NEUROSCI, V11, DOI 10.3389/fnmol.2018.00144

Jankovic J, 2020, J NEUROL NEUROSUR PS, V91, P795, DOI 10.1136/jnnp-2019-322338

Jin HY, 2021, FRONT PSYCHIATRY, V12, DOI 10.3389/fpsyt.2021.748958

Joshi AU, 2019, NAT NEUROSCI, V22, P1635, DOI 10.1038/s41593-019-0486-0

Joshi N, 2018, J NEUROSCI RES, V96, P379, DOI 10.1002/jnr.24185

Kam TI, 2020, NEUROBIOL DIS, V144, DOI 10.1016/j.nbd.2020.105028

Kata D, 2017, BRAIN RES BULL, V132, P61, DOI 10.1016/j.brainresbull.2017.05.009

Keatinge M, 2015, HUM MOL GENET, V24, P6640, DOI 10.1093/hmg/ddv369

Keshavarzian A, 2015, MOVEMENT DISORD, V30, P1351, DOI 10.1002/mds.26307

Killinger BA, 2018, SCI TRANSL MED, V10, DOI 10.1126/scitranslmed.aar5280

Kim S, 2019, NEURON, V103, P627, DOI 10.1016/j.neuron.2019.05.035

Klegeris A, 2006, FASEB J, V20, P2000, DOI 10.1096/fj.06-6183com

Kölliker-Frers R, 2021, MEDIAT INFLAMM, V2021, DOI 10.1155/2021/9999146

Koprich JB, 2008, J NEUROINFLAMM, V5, DOI 10.1186/1742-2094-5-8

Kortekaas R, 2005, ANN NEUROL, V57, P176, DOI 10.1002/ana.20369

Kuan WL, 2016, EXP NEUROL, V285, P72, DOI 10.1016/j.expneurol.2016.09.003

Kurkowska-Jastrzebska I, 2004, INT IMMUNOPHARMACOL, V4, P1307, DOI 10.1016/j.intimp.2004.05.006

Kustrimovic N, 2018, J NEUROINFLAMM, V15, DOI 10.1186/s12974-018-1248-8

Le WD, 2016, FRONT MOL NEUROSCI, V9, DOI 10.3389/fnmol.2016.00089

Lee HJ, 2008, BIOCHEM BIOPH RES CO, V372, P423, DOI 10.1016/j.bbrc.2008.05.045

Lee HJ, 2013, EXP MOL MED, V45, DOI 10.1038/emm.2013.45

Lee HJ, 2010, OXID MED CELL LONGEV, V3, P283, DOI 10.4161/oxim.3.4.12809

Lee HJ, 2010, J BIOL CHEM, V285, P9262, DOI 10.1074/jbc.M109.081125

Lee HS, 2021, GUT, V70, P408, DOI 10.1136/gutjnl-2020-322429

Lee S, 2015, CRIT REV IMMUNOL, V35, P77, DOI 10.1615/CritRevImmunol.2015012127

Li QY, 2019, NEURON, V101, P207, DOI 10.1016/j.neuron.2018.12.006

Li YY, 2022, NEUROCHEM RES, V47, P3627, DOI 10.1007/s11064-022-03586-0

Liddelow SA, 2017, NATURE, V541, P481, DOI 10.1038/nature21029

Lin CH, 2019, J NEUROINFLAMM, V16, DOI 10.1186/s12974-019-1528-y

Lindström V, 2014, NEUROBIOL DIS, V69, P134, DOI 10.1016/j.nbd.2014.05.009

Liu BJ, 2017, NEUROLOGY, V88, P1996, DOI 10.1212/WNL.0000000000003961

Lynch NJ, 2004, MOL IMMUNOL, V40, P709, DOI 10.1016/j.molimm.2003.08.009

Machida T, 2017, PLOS ONE, V12, DOI 10.1371/journal.pone.0177447

Copas ANM, 2021, FRONT NEUROL, V12, DOI 10.3389/fneur.2021.666737

Mahmoud S, 2019, CELLS-BASEL, V8, DOI 10.3390/cells8020184

Mandler M, 2014, ACTA NEUROPATHOL, V127, P861, DOI 10.1007/s00401-014-1256-4

Mark KS, 1999, LIFE SCI, V64, P1941, DOI 10.1016/S0024-3205(99)00139-3

Masliah E, 2005, NEURON, V46, P857, DOI 10.1016/j.neuron.2005.05.010

Masliah E, 2011, PLOS ONE, V6, DOI 10.1371/journal.pone.0019338

Matheoud D, 2016, CELL, V166, P314, DOI 10.1016/j.cell.2016.05.039

Mejias NH, 2018, J INFLAMM-LOND, V15, DOI 10.1186/s12950-018-0198-3

Mena MA, 2002, MOL NEUROBIOL, V25, P245, DOI 10.1385/MN:25:3:245

Menozzi E, 2021, ANN MED, V53, P611, DOI 10.1080/07853890.2021.1890330

Migliore L, 2002, NEUROLOGY, V58, P1809, DOI 10.1212/WNL.58.12.1809

Miki Y, 2018, NEUROBIOL AGING, V63, P33, DOI 10.1016/j.neurobiolaging.2017.11.006

Miller RM, 2007, EXP NEUROL, V204, P421, DOI 10.1016/j.expneurol.2006.12.005

Mogi M, 1996, NEUROSCI LETT, V211, P13, DOI 10.1016/0304-3940(96)12706-3

MOGI M, 1994, NEUROSCI LETT, V180, P147, DOI 10.1016/0304-3940(94)90508-8

MOGI M, 1994, NEUROSCI LETT, V165, P208, DOI 10.1016/0304-3940(94)90746-3

Mogi M, 2000, J NEURAL TRANSM, V107, P335, DOI 10.1007/s007020050028

MOGI M, 1995, NEUROSCI LETT, V193, P129, DOI 10.1016/0304-3940(95)11686-Q

Mogi M, 1996, J NEURAL TRANSM, V103, P1077, DOI 10.1007/BF01291792

Mollenhauer B, 2011, LANCET NEUROL, V10, P230, DOI 10.1016/S1474-4422(11)70014-X

Monteiro RS, 2015, MED HYPOTHESES, V85, P537, DOI 10.1016/j.mehy.2015.07.011

Mullin S, 2021, MOVEMENT DISORD, V36, P774, DOI 10.1002/mds.28375

Mullin S, 2020, JAMA NEUROL, V77, P427, DOI 10.1001/jamaneurol.2019.4611

Murray DK, 2014, TRANSL NEURODEGENER, V3, DOI 10.1186/2047-9158-3-5

Nakajima K, 2007, NEUROCHEM INT, V50, P807, DOI 10.1016/j.neuint.2007.02.006

Nimmo JT, 2022, ACTA NEUROPATHOL, V143, P55, DOI 10.1007/s00401-021-02381-5

Niranjan R, 2018, NEUROCHEM INT, V120, P13, DOI 10.1016/j.neuint.2018.07.003

Niwa F, 2012, GERIATR GERONTOL INT, V12, P102, DOI 10.1111/j.1447-0594.2011.00740.x

O'Donovan SM, 2020, NEUROGASTROENT MOTIL, V32, DOI 10.1111/nmo.13726

Ouchi Y, 2005, ANN NEUROL, V57, P168, DOI 10.1002/ana.20338

Pajares M, 2020, CELLS-BASEL, V9, DOI 10.3390/cells9071687

Pan-Montojo F, 2010, PLOS ONE, V5, DOI 10.1371/journal.pone.0008762

Parillaud VR, 2017, J NEUROINFLAMM, V14, DOI 10.1186/s12974-017-0830-9

Parrella E, 2019, TRANSL NEURODEGENER, V8, DOI 10.1186/s40035-019-0154-z

Ramos JMP, 2019, FRONT IMMUNOL, V10, DOI 10.3389/fimmu.2019.00080

Perez-Pardo P, 2019, GUT, V68, P829, DOI 10.1136/gutjnl-2018-316844

Perez-Pardo P, 2017, EUR J PHARMACOL, V817, P86, DOI 10.1016/j.ejphar.2017.05.042

Petrov VA, 2017, B EXP BIOL MED+, V162, P734, DOI 10.1007/s10517-017-3700-7

Phillips RJ, 2008, NEUROSCIENCE, V153, P733, DOI 10.1016/j.neuroscience.2008.02.074

Prinz M, 2010, J NEUROIMMUNOL, V224, P80, DOI 10.1016/j.jneuroim.2010.05.015

Pull SL, 2005, P NATL ACAD SCI USA, V102, P99, DOI 10.1073/pnas.0405979102

Qiao C, 2016, CNS NEUROSCI THER, V22, P451, DOI 10.1111/cns.12514

Qureshi YH, 2022, CELL REP, V38, DOI 10.1016/j.celrep.2021.110262

Racette BA, 2018, ANN CLIN TRANSL NEUR, V5, P870, DOI 10.1002/acn3.580

Rani L, 2021, NEUROSCI RES, V168, P100, DOI 10.1016/j.neures.2021.01.001

Ransohoff RM, 2016, NAT NEUROSCI, V19, P987, DOI 10.1038/nn.4338

Rees K, 2011, COCHRANE DB SYST REV, DOI 10.1002/14651858.CD008454.pub2

Reynolds AD, 2007, J LEUKOCYTE BIOL, V82, P1083, DOI 10.1189/jlb.0507296

Reynolds AD, 2010, J IMMUNOL, V184, P2261, DOI 10.4049/jimmunol.0901852

Rite I, 2007, J NEUROCHEM, V101, P1567, DOI 10.1111/j.1471-4159.2007.04567.x

Rodriguez-Pallares J, 2007, J NEUROCHEM, V103, P145, DOI 10.1111/j.1471-4159.2007.04699.x

Rooks MG, 2016, NAT REV IMMUNOL, V16, P341, DOI 10.1038/nri.2016.42

Rosenkranz D, 2007, J NEUROIMMUNOL, V188, P117, DOI 10.1016/j.jneuroim.2007.05.011

Ross GW, 2000, JAMA-J AM MED ASSOC, V283, P2674, DOI 10.1001/jama.283.20.2674

Rostami J, 2020, J NEUROINFLAMM, V17, DOI 10.1186/s12974-020-01776-7

Rostami J, 2017, J NEUROSCI, V37, P11835, DOI 10.1523/JNEUROSCI.0983-17.2017

Saijo K, 2009, CELL, V137, P47, DOI 10.1016/j.cell.2009.01.038

Salminen A, 2011, EUR J NEUROSCI, V34, P3, DOI 10.1111/j.1460-9568.2011.07738.x

Salter MW, 2017, NAT MED, V23, P1018, DOI 10.1038/nm.4397

Sampson TR, 2016, CELL, V167, P1469, DOI 10.1016/j.cell.2016.11.018

Sarkar S, 2020, FRONT IMMUNOL, V11, DOI 10.3389/fimmu.2020.00033

Sarkar S, 2017, NPJ PARKINSON DIS, V3, DOI 10.1038/s41531-017-0032-2

Satoh J, 2016, NEUROPATHOLOGY, V36, P39, DOI 10.1111/neup.12235

Saunders JAH, 2012, J NEUROIMMUNE PHARM, V7, P927, DOI 10.1007/s11481-012-9402-z

Savitt D, 2019, DRUGS, V79, P797, DOI 10.1007/s40265-019-01104-1

Sawada M, 1999, J NEUROCHEM, V72, P1466, DOI 10.1046/j.1471-4159.1999.721466.x

SAWADA M, 1995, INT J DEV NEUROSCI, V13, P253, DOI 10.1016/0736-5748(94)00076-F

Scalzo P, 2009, J NEUROIMMUNOL, V216, P122, DOI 10.1016/j.jneuroim.2009.08.001

Scheiblich H, 2021, CELL, V184, P5089, DOI 10.1016/j.cell.2021.09.007

Schenk DB, 2017, MOVEMENT DISORD, V32, P211, DOI 10.1002/mds.26878

Schneeberger A, 2016, MOVEMENT DISORD, V31, P214, DOI 10.1002/mds.26377

Schofield DJ, 2019, NEUROBIOL DIS, V132, DOI 10.1016/j.nbd.2019.104582

Schonhoff AM, 2020, PROG BRAIN RES, V252, P169, DOI 10.1016/bs.pbr.2019.10.006

Schroder K, 2010, CELL, V140, P821, DOI 10.1016/j.cell.2010.01.040

Schröder JB, 2018, FRONT NEUROL, V9, DOI 10.3389/fneur.2018.01081

Selkrig J, 2014, GUT MICROBES, V5, P369, DOI 10.4161/gmic.28681

Seppi K, 2019, MOVEMENT DISORD, V34, P180, DOI 10.1002/mds.27602

Serbina NV, 2006, NAT IMMUNOL, V7, P311, DOI 10.1038/ni1309

Shahaduzzaman M, 2015, PLOS ONE, V10, DOI 10.1371/journal.pone.0116841

Shannon KM, 2012, MOVEMENT DISORD, V27, P709, DOI 10.1002/mds.23838

Sherer TB, 2003, NEUROSCI LETT, V341, P87, DOI 10.1016/S0304-3940(03)00172-1

Shih RH, 2015, FRONT MOL NEUROSCI, V8, DOI 10.3389/fnmol.2015.00077

Shimoji M, 2009, NEUROTOX RES, V16, P318, DOI 10.1007/s12640-009-9076-3

Siddiqui MF, 2002, PARKINSONISM RELAT D, V8, P277, DOI 10.1016/S1353-8020(01)00052-9

Simuni T, 2021, JAMA NEUROL, V78, P312, DOI 10.1001/jamaneurol.2020.4725

Singh A, 2021, INFLAMMOPHARMACOLOGY, V29, P5, DOI 10.1007/s10787-020-00764-w

Smajic S, 2022, BRAIN, V145, P964, DOI 10.1093/brain/awab446

Sommer A, 2018, CELL STEM CELL, V23, P123, DOI 10.1016/j.stem.2018.06.015

Sonninen TM, 2020, SCI REP-UK, V10, DOI 10.1038/s41598-020-71329-8

Sousa C, 2018, EMBO REP, V19, DOI 10.15252/embr.201846171

Stefanis L, 2019, J NEUROCHEM, V150, P577, DOI 10.1111/jnc.14704

Stevens CH, 2012, J NEUROIMMUNOL, V252, P95, DOI 10.1016/j.jneuroim.2012.07.015

Stokholm MG, 2017, LANCET NEUROL, V16, P789, DOI 10.1016/S1474-4422(17)30173-4

Stoll G, 2002, ADV EXP MED BIOL, V513, P87

Strowig T, 2012, NATURE, V481, P278, DOI 10.1038/nature10759

Su XM, 2008, NEUROBIOL AGING, V29, P1690, DOI 10.1016/j.neurobiolaging.2007.04.006

Su XM, 2009, NEUROTOX RES, V16, P238, DOI 10.1007/s12640-009-9053-x

Subramaniam SR, 2017, FRONT AGING NEUROSCI, V9, DOI 10.3389/fnagi.2017.00176

Sulzer D, 2017, NATURE, V546, P656, DOI 10.1038/nature22815

Surmeier DJ, 2017, NAT REV NEUROSCI, V18, P101, DOI 10.1038/nrn.2016.178

Svensson E, 2015, ANN NEUROL, V78, P522, DOI 10.1002/ana.24448

Takeda K, 2005, INT IMMUNOL, V17, P1, DOI 10.1093/intimm/dxh186

Takemoto M, 2021, J ALZHEIMERS DIS, V80, P331, DOI 10.3233/JAD-201495

Tan EK, 2020, NAT REV NEUROL, V16, P303, DOI 10.1038/s41582-020-0344-4

Tan YL, 2020, MOL PSYCHIATR, V25, P351, DOI 10.1038/s41380-019-0609-8

Tang FL, 2020, CELL DEATH DIFFER, V27, P2099, DOI 10.1038/s41418-019-0487-2

Tang Y, 2016, MOL NEUROBIOL, V53, P1181, DOI 10.1007/s12035-014-9070-5

Teismann P, 2003, P NATL ACAD SCI USA, V100, P5473, DOI 10.1073/pnas.0837397100

Theodore S, 2008, J NEUROPATH EXP NEUR, V67, P1149, DOI 10.1097/NEN.0b013e31818e5e99

Tran HT, 2014, CELL REP, V7, P2054, DOI 10.1016/j.celrep.2014.05.033

Troncoso-Escudero P, 2018, FRONT NEUROL, V9, DOI 10.3389/fneur.2018.00860

Tsao N, 2001, J MED MICROBIOL, V50, P812, DOI 10.1099/0022-1317-50-9-812

Tu HY, 2021, AGING CELL, V20, DOI 10.1111/acel.13522

Tysnes OB, 2015, ANN NEUROL, V78, P1011, DOI 10.1002/ana.24531

Ueki A, 2004, J NEUROL, V251, P18, DOI 10.1007/s00415-004-1706-3

Uemura N, 2018, MOL NEURODEGENER, V13, DOI 10.1186/s13024-018-0257-5

Ugen KE, 2015, HUM VACC IMMUNOTHER, V11, P922, DOI 10.1080/21645515.2015.1012033

Vasile F, 2017, BRAIN STRUCT FUNCT, V222, P2017, DOI 10.1007/s00429-017-1383-5

Vawter MP, 1996, EXP NEUROL, V142, P313, DOI 10.1006/exnr.1996.0200

Volc D, 2020, LANCET NEUROL, V19, P591, DOI 10.1016/S1474-4422(20)30136-8

Wallen ZD, 2020, NPJ PARKINSONS DIS, V6, DOI 10.1038/s41531-020-0112-6

Walsh JG, 2014, NAT REV NEUROSCI, V15, P84, DOI 10.1038/nrn3638

Wang L, 2010, BIOMED ENVIRON SCI, V23, P327, DOI 10.1016/S0895-3988(10)60072-4

Wang SJ, 2015, P NATL ACAD SCI USA, V112, pE1926, DOI 10.1073/pnas.1417883112

Weihofen A, 2019, NEUROBIOL DIS, V124, P276, DOI 10.1016/j.nbd.2018.10.016

Wheeler C.J., 2014, Journal of neurology and neurophysiology, V5, P209, DOI [DOI 10.4172/2155-9562.1000209, 10.4172/2155-9562.1000209, 10.4172/2155-9562, DOI 10.4172/2155-9562]

Wijeyekoon RS, 2018, FRONT NEUROL, V9, DOI 10.3389/fneur.2018.00870

Wilkinson BL, 2012, NEUROBIOL AGING, V33, DOI 10.1016/j.neurobiolaging.2010.06.014

Witoelar A, 2017, JAMA NEUROL, V74, P780, DOI 10.1001/jamaneurol.2017.0469

Wu FJ, 2015, J NEUROINFLAMM, V12, DOI 10.1186/s12974-015-0316-6

Yang XP, 2022, BIOCHEM BIOPH RES CO, V594, P161, DOI 10.1016/j.bbrc.2022.01.047

Yao LP, 2021, FRONT CELL DEV BIOL, V9, DOI 10.3389/fcell.2021.655819

Yasuda T, 2007, EXP NEUROL, V206, P308, DOI 10.1016/j.expneurol.2007.05.012

Zamanian JL, 2012, J NEUROSCI, V32, P6391, DOI 10.1523/JNEUROSCI.6221-11.2012

Zaminelli T, 2014, NEUROTOX RES, V26, P351, DOI 10.1007/s12640-014-9467-y

Zapiec B, 2017, BRAIN, V140, P2722, DOI 10.1093/brain/awx208

Zella SMA, 2019, NEUROL THER, V8, P29, DOI 10.1007/s40120-018-0122-z

Zhang W, 2005, FASEB J, V19, P533, DOI 10.1096/fj.04-2751com

Zhang W, 2018, MOL NEUROBIOL, V55, P619, DOI 10.1007/s12035-016-0308-2

Zhou Y, 2016, MOL NEURODEGENER, V11, DOI 10.1186/s13024-016-0094-3

Zhu JL, 2018, CELL DEATH DIFFER, V25, P2037, DOI 10.1038/s41418-018-0127-2

Zhu ML, 2022, FRONT IMMUNOL, V13, DOI 10.3389/fimmu.2022.937555

Zoladz JA, 2014, J PHYSIOL PHARMACOL, V65, P441

NR 288

TC 31

Z9 33

U1 7

U2 24

PU MDPI

PI BASEL

PA ST ALBAN-ANLAGE 66, CH-4052 BASEL, SWITZERLAND

EI 2073-4409

J9 CELLS-BASEL

JI Cells

PD SEP

PY 2022

VL 11

IS 18

AR 2908

DI 10.3390/cells11182908

PG 40

WC Cell Biology

WE Science Citation Index Expanded (SCI-EXPANDED)

SC Cell Biology

GA 4T5LI

UT WOS:000858158100001

PM 36139483

OA Green Published, gold

DA 2024-08-12

ER

PT J

AU Czlonkowska, A

Kurkowska-Jastrzebska, I

AF Czlonkowska, Anna

Kurkowska-Jastrzebska, Iwona

TI Inflammation and gliosis in neurological diseases - clinical

implications

SO JOURNAL OF NEUROIMMUNOLOGY

LA English

DT Article

DE Neurodegeneration; Multiple sclerosis; Parkinson's disease; Alzheimer's

disease; Stroke

ID NONSTEROIDAL ANTIINFLAMMATORY DRUGS; APPEARING WHITE-MATTER;

AMYLOID-BETA PEPTIDE; parkinson's disease; MULTIPLE-SCLEROSIS; MOUSE

MODEL; MICROGLIAL ACTIVATION; T-CELLS; DOPAMINERGIC-NEURONS;

ALZHEIMER-DISEASE

AB The inflammatory reaction accompany all acute processes in the central nervous system (CNS), (as stroke or traumatic brain injury) and chronic neurodegenerative processes (as Parkinson's or Alzheimer's disease), and through the stage of cleaning of damage tissue, contribute to recovery and regeneration and eventually to restoration of the function. However many studies showed that inflammation in the CNS may be harmful because of an excessive vulnerability of the nervous tissue or impaired regulation. Manipulation of the inflammation is now one of the approaches in the treatment of the various diseases of the CNS. (C) 2010 Elsevier B.V. All rights reserved.

C1 [Czlonkowska, Anna; Kurkowska-Jastrzebska, Iwona] Inst Psychiat & Neurol, Dept Neurol 2, Warsaw, Poland.

[Czlonkowska, Anna] Med Univ Warsaw, Dept Clin & Expt Pharmacol, Warsaw, Poland.

C3 Institute of Psychiatry & Neurology; Medical University of Warsaw

RP Czlonkowska, A (corresponding author), Sobieskiego 9, PL-02957 Warsaw, Poland.

EM czlonkow@ipin.edu.pl

OI Kurkowska-Jastrzebska, Iwona/0000-0001-6553-9080; Czlonkowska,

Anna/0000-0002-1956-1866

CR Aharoni R, 2010, J NEUROIMMUNOL, V225, P100, DOI 10.1016/j.jneuroim.2010.04.022

Aisen PS, 2008, CURR ALZHEIMER RES, V5, P73, DOI 10.2174/156720508783884602

Akiyama H, 2000, NEUROBIOL AGING, V21, P383, DOI 10.1016/S0197-4580(00)00124-X

Aktas O, 2007, ARCH NEUROL-CHICAGO, V64, P185, DOI 10.1001/archneur.64.2.185

Aktas O, 2010, NAT REV NEUROL, V6, P373, DOI 10.1038/nrneurol.2010.76

Asahi M, 2000, J CEREBR BLOOD F MET, V20, P1681, DOI 10.1097/00004647-200012000-00007

Barnett MH, 2004, ANN NEUROL, V55, P458, DOI 10.1002/ana.20016

Barouch R, 2002, FASEB J, V16, P1304, DOI 10.1096/fj.01-0467fje

Bartels AL, 2007, MOVEMENT DISORD, V22, P1852, DOI 10.1002/mds.21552

Benner EJ, 2004, P NATL ACAD SCI USA, V101, P9435, DOI 10.1073/pnas.0400569101

Bjartmar C, 2001, NEUROLOGY, V57, P1248, DOI 10.1212/WNL.57.7.1248

Bjartmar C, 2001, CURR OPIN NEUROL, V14, P271, DOI 10.1097/00019052-200106000-00003

BlumDegen D, 1995, NEUROSCI LETT, V202, P17, DOI 10.1016/0304-3940(95)12192-7

Butovsky O, 2005, MOL CELL NEUROSCI, V29, P381, DOI 10.1016/j.mcn.2005.03.005

Carvey PM, 2001, EXP NEUROL, V171, P98, DOI 10.1006/exnr.2001.7735

Castellani ML, 2007, INT J IMMUNOPATH PH, V20, P447, DOI 10.1177/039463200702000303

Chen HL, 2005, ANN NEUROL, V58, P963, DOI 10.1002/ana.20682

Chen HL, 2003, ARCH NEUROL-CHICAGO, V60, P1059, DOI 10.1001/archneur.60.8.1059

Ciesielska A, 2003, ACTA NEUROBIOL EXP, V63, P117, DOI 10.55782/ane-2003-1461

Compston A, 1996, Mult Scler, V1, P388

Danilov AI, 2006, EUR J NEUROSCI, V23, P394, DOI 10.1111/j.1460-9568.2005.04563.x

Dheen ST, 2007, CURR MED CHEM, V14, P1189, DOI 10.2174/092986707780597961

Dodel R, 2002, ANN NEUROL, V52, P253, DOI 10.1002/ana.10253

Emsley HCA, 2005, J NEUROL NEUROSUR PS, V76, P1366, DOI 10.1136/jnnp.2004.054882

Evangelou N, 2000, ANN NEUROL, V47, P391, DOI 10.1002/1531-8249(200003)47:3<391::AID-ANA20>3.0.CO;2-J

Flügel A, 2001, IMMUNITY, V14, P547, DOI 10.1016/S1074-7613(01)00143-1

Fontaine V, 2002, J NEUROSCI, V22, DOI 10.1523/JNEUROSCI.22-07-j0001.2002

Gerhard A, 2006, NEUROBIOL DIS, V21, P404, DOI 10.1016/j.nbd.2005.08.002

Gilman S, 2005, NEUROLOGY, V64, P1553, DOI 10.1212/01.WNL.0000159740.16984.3C

Gimsa U, 2000, BRAIN PATHOL, V10, P365

Graeber MB, 2010, ACTA NEUROPATHOL, V119, P89, DOI 10.1007/s00401-009-0622-0

Gromadzka G, 2007, J NEUROL NEUROSUR PS, V78, P183, DOI 10.1136/jnnp.2006.093104

HAMA T, 1991, NEUROSCIENCE, V40, P445, DOI 10.1016/0306-4522(91)90132-8

Hendrix S, 2007, J NEUROIMMUNOL, V184, P100, DOI 10.1016/j.jneuroim.2006.11.019

Hernán MA, 2006, NEUROLOGY, V66, P1097, DOI 10.1212/01.wnl.0000204446.82823.28

Imamura K, 2003, ACTA NEUROPATHOL, V106, P518, DOI 10.1007/s00401-003-0766-2

Johansson S, 2002, J COMP NEUROL, V443, P237, DOI 10.1002/cne.10119

Joniec I, 2009, BRAIN RES, V1261, P7, DOI 10.1016/j.brainres.2008.12.081

Kokjohn TA, 2009, CNS NEUROL DISORD-DR, V8, P88, DOI 10.2174/187152709787847315

Krady JK, 2005, DIABETES, V54, P1559, DOI 10.2337/diabetes.54.5.1559

Kurkowska-Jastrzebska I, 2007, PARKINSONISM RELAT D, V13, pS141, DOI 10.1016/S1353-8020(08)70772-7

Kurkowska-Jastrzebska I, 2006, ANN NEUROL, V59, P988, DOI 10.1002/ana.20860

Kurkowska-Jastrzebska I, 1999, EXP NEUROL, V156, P50, DOI 10.1006/exnr.1998.6993

Kurkowska-Jastrzebska I, 2005, NEUROSCIENCE, V131, P247, DOI 10.1016/j.neuroscience.2004.10.027

Kurkowska-Jastrzebska I, 2004, INT IMMUNOPHARMACOL, V4, P1307, DOI 10.1016/j.intimp.2004.05.006

Kurkowska-Jastrzebska I, 2002, INT IMMUNOPHARMACOL, V2, P1213, DOI 10.1016/S1567-5769(02)00078-4

Kurkowska-Jastrzebska I, 2009, INT IMMUNOPHARMACOL, V9, P781, DOI 10.1016/j.intimp.2009.03.003

Kurkowska-Jastrzebska I, 2007, NEUROREPORT, V18, P425, DOI 10.1097/WNR.0b013e3280586777

Kurkowska-Jastrzebska I, 2008, J NEUROIMMUNOL, V203, P233

Langston JW, 1999, ANN NEUROL, V46, P598, DOI 10.1002/1531-8249(199910)46:4<598::AID-ANA7>3.0.CO;2-F

Lassmann H, 2001, TRENDS MOL MED, V7, P115, DOI 10.1016/S1471-4914(00)01909-2

Lassmann H, 2007, BRAIN PATHOL, V17, P210, DOI 10.1111/j.1750-3639.2007.00064.x

Lisak RP, 2007, J NEUROINFLAMM, V4, DOI 10.1186/1742-2094-4-30

Lisak RP, 2006, MULT SCLER J, V12, P149, DOI 10.1191/135248506ms1251oa

Lucchinetti C, 2000, ANN NEUROL, V47, P707, DOI 10.1002/1531-8249(200006)47:6<707::AID-ANA3>3.0.CO;2-Q

Lyketsos CG, 2007, NEUROLOGY, V68, P1800

McCombe PA, 2008, INT J STROKE, V3, P254, DOI 10.1111/j.1747-4949.2008.00222.x

McGeer PL, 2007, NEUROBIOL AGING, V28, P639, DOI 10.1016/j.neurobiolaging.2006.03.013

MCGEER PL, 1988, NEUROLOGY, V38, P1285, DOI 10.1212/WNL.38.8.1285

Moalem G, 2000, J AUTOIMMUN, V15, P331, DOI 10.1006/jaut.2000.0441

MOGI M, 1995, J NEURAL TRANSM-PARK, V9, P87, DOI 10.1007/BF02252965

Naismith RT, 2010, NEUROLOGY, V74, P1860, DOI 10.1212/WNL.0b013e3181e24373

Nau R, 2002, TRENDS NEUROSCI, V25, P38, DOI 10.1016/S0166-2236(00)02024-5

Nicoll JAR, 2003, NAT MED, V9, P448, DOI 10.1038/nm840

Noto ATW, 2006, THROMB HAEMOSTASIS, V96, P53, DOI 10.1160/TH05-11-0772

Orgogozo JM, 2003, NEUROLOGY, V61, P46, DOI 10.1212/01.WNL.0000073623.84147.A8

Peterson JW, 2001, ANN NEUROL, V50, P389, DOI 10.1002/ana.1123

Przedborski S, 2010, MOVEMENT DISORD, V25, pS55, DOI 10.1002/mds.22638

Przybylkowski A, 2004, BRAIN RES, V1019, P144, DOI 10.1016/j.brainres.2004.05.095

Purisai MG, 2007, NEUROBIOL DIS, V25, P392, DOI 10.1016/j.nbd.2006.10.008

Reines SA, 2004, NEUROLOGY, V62, P66, DOI 10.1212/WNL.62.1.66

Relkin NR, 2009, NEUROBIOL AGING, V30, P1728, DOI 10.1016/j.neurobiolaging.2007.12.021

Samii A, 2009, DRUG AGING, V26, P769, DOI 10.2165/11316780-000000000-00000

Sawada M, 2008, NEURODEGENER DIS, V5, P254, DOI 10.1159/000113717

Schiefer J, 1999, J NEUROCYTOL, V28, P439, DOI 10.1023/A:1007048903862

Schwartz M, 2006, TRENDS NEUROSCI, V29, P68, DOI 10.1016/j.tins.2005.12.005

Schwartz M, 2005, J NEUROL SCI, V233, P163, DOI 10.1016/j.jns.2005.03.014

Schwartz M, 2003, J CEREBR BLOOD F MET, V23, P385, DOI 10.1097/01.WCB.0000061881.75234.5E

Schwartz M, 2006, J NEUROIMMUNE PHARM, V1, P4, DOI 10.1007/s11481-005-9010-2

Schwartz M, 2008, TRENDS PHARMACOL SCI, V29, P287, DOI 10.1016/j.tips.2008.03.006

Serrano-Pozo A, 2010, BRAIN, V133, P1312, DOI 10.1093/brain/awq056

Streit WJ, 2009, ACTA NEUROPATHOL, V118, P475, DOI 10.1007/s00401-009-0556-6

STRIJBOS PJLM, 1995, J NEUROSCI, V15, P3468

Teismann P, 2003, P NATL ACAD SCI USA, V100, P5473, DOI 10.1073/pnas.0837397100

Tsutsui S, 2009, ANN NEUROL, V66, P711, DOI 10.1002/ana.21845

Um JY, 2003, MOL BRAIN RES, V115, P50, DOI 10.1016/S0169-328X(03)00179-7

Van der Walt A, 2010, PHARMACOL THERAPEUT, V126, P82, DOI 10.1016/j.pharmthera.2010.01.006

Villoslada P, 2008, CLIN IMMUNOL, V128, P294, DOI 10.1016/j.clim.2008.04.003

Wang XJ, 2007, NEUROCHEM INT, V50, P39, DOI 10.1016/j.neuint.2006.07.014

Wilcock DM, 2004, J NEUROSCI, V24, P6144, DOI 10.1523/JNEUROSCI.1090-04.2004

Willenborg DO, 1999, J NEUROIMMUNOL, V100, P21, DOI 10.1016/S0165-5728(99)00212-X

Worrall BB, 2007, STROKE, V38, P1189, DOI 10.1161/01.STR.0000260099.42744.b0

Zhang SC, 2003, GLIA, V41, P191, DOI 10.1002/glia.10172

NR 93

TC 68

Z9 72

U1 1

U2 5

PU ELSEVIER

PI AMSTERDAM

PA RADARWEG 29, 1043 NX AMSTERDAM, NETHERLANDS

SN 0165-5728

EI 1872-8421

J9 J NEUROIMMUNOL

JI J. Neuroimmunol.

PD FEB

PY 2011

VL 231

IS 1-2

SI SI

BP 78

EP 85

DI 10.1016/j.jneuroim.2010.09.020

PG 8

WC Immunology; Neurosciences

WE Science Citation Index Expanded (SCI-EXPANDED)

SC Immunology; Neurosciences & Neurology

GA 744YT

UT WOS:000289131800012

PM 20943275

DA 2024-08-12

ER

PT J

AU Wu, LM

Walas, S

Leung, W

Sykes, DB

Wu, J

Lo, EH

Lok, J

AF Wu, Limin

Walas, Samantha

Leung, Wendy

Sykes, David B.

Wu, Jiang

Lo, Eng H.

Lok, Josephine

TI Neuregulin1-β Decreases IL-1β-Induced Neutrophil Adhesion to Human Brain

Microvascular Endothelial Cells

SO TRANSLATIONAL STROKE RESEARCH

LA English

DT Article

DE Neuroinflammation; IL-1 beta; NRG1-beta; Neutrophil; VCAM-1; E-selectin

ID RECEPTOR SUBUNIT; ERBB RECEPTORS; INJURY; SCHIZOPHRENIA; INFLAMMATION;

PROMOTES; LEADS; INTERLEUKIN-1-BETA; MICRODIALYSIS; ANGIOGENESIS

AB Neuroinflammation contributes to the pathophysiology of diverse diseases including stroke, traumatic brain injury, Alzheimer's disease, Parkinson's disease, and multiple sclerosis, resulting in neurodegeneration and loss of neurological function. The response of the microvascular endothelium often contributes to neuroinflammation. One such response is the upregulation of endothelial adhesion molecules which facilitate neutrophil adhesion to the endothelium and their migration from blood to tissue. Neuregulin-1 (NRG1) is an endogenous growth factor which has been reported to have anti-inflammatory effects in experimental stroke models. We hypothesized that NRG1 would decrease the endothelial response to inflammation and result in a decrease in neutrophil adhesion to endothelial cells. We tested this hypothesis in an in vitro model of cytokine-induced endothelial injury, in which human brain microvascular endothelial cells (BMECs) were treated with IL-1 beta, along with co-incubation with vehicle or NRG1-beta. Outcome measures included protein levels of endothelial ICAM-1, VCAM-1, and E-selectin, as well as the number of neutrophils that adhere to the endothelial monolayer. Our data show that NRG1-beta decreased the levels of VCAM-1, E-selectin, and neutrophil adhesion to brain microvascular endothelial cells activated by IL1-beta. These findings open new possibilities for investigating NRG1 in neuroprotective strategies in brain injury.

C1 [Wu, Limin; Walas, Samantha; Leung, Wendy; Lo, Eng H.; Lok, Josephine] Harvard Univ, Sch Med, Neuroprotect Res Lab, Massachusetts Gen Hosp, Charlestown, MA 02129 USA.

[Wu, Limin; Wu, Jiang] Jilin Univ, Hosp 1, Dept Neurol, Changchun 130021, Jilin, China.

[Lok, Josephine] Harvard Univ, Sch Med, Dept Pediat, Div Pediat Crit Care,Massachusetts Gen Hosp, Boston, MA 02115 USA.

[Sykes, David B.] Harvard Univ, Sch Med, Massachusetts Gen Hosp, Dept Med,Div Hematol Oncol, Boston, MA 02115 USA.

[Sykes, David B.] Broad Inst Massachusetts Inst Technol & Harvard U, Cambridge, MA USA.

C3 Harvard University; Massachusetts General Hospital; Jilin University;

Harvard University; Harvard Medical School; Massachusetts General

Hospital; Harvard University; Massachusetts General Hospital; Harvard

Medical School; Harvard University; Massachusetts Institute of

Technology (MIT); Broad Institute

RP Lok, J (corresponding author), Harvard Univ, Sch Med, Neuroprotect Res Lab, Massachusetts Gen Hosp, Charlestown, MA 02129 USA.

EM jlok1@mgh.harvard.edu

FU China Scholarship Council [201206170107]; [R37NS037074-13];

[R01NS076694-02]; [P01NS055104-05]; [K08N5057339-04]

FX This work is supported by the following grants:; R37NS037074-13 (EHL);

R01NS076694-02(EHL); P01NS055104-05 (EHL); K08N5057339-04 (JL); China

Scholarship Council (#201206170107; LW)

CR Acosta SA, 2013, PLOS ONE, V8, DOI 10.1371/journal.pone.0053376

Asp L, 2009, BRAIN BEHAV IMMUN, V23, P733, DOI 10.1016/j.bbi.2009.04.004

BEVILACQUA MP, 1987, SEMIN THROMB HEMOST, V13, P425, DOI 10.1055/s-2007-1003519

Boato F, 2013, J NEUROINFLAMM, V10, DOI 10.1186/1742-2094-10-6

Borlongan CV, 2011, PROG NEUROBIOL, V95, P213, DOI 10.1016/j.pneurobio.2011.08.005

Brooks TA, 2005, AM J PHYSIOL-HEART C, V289, pH738, DOI 10.1152/ajpheart.01288.2004

Brothers Holly M, 2013, J Alzheimers Dis Parkinsonism, V3, P110

Calvo M, 2010, J NEUROSCI, V30, P5437, DOI 10.1523/JNEUROSCI.5169-09.2010

Dimayuga FO, 2003, J NEUROIMMUNOL, V136, P67, DOI 10.1016/S0165-5728(03)00003-1

Falls DL, 2003, EXP CELL RES, V284, P14, DOI 10.1016/S0014-4827(02)00102-7

Finigan JH, 2013, EUR RESPIR J, V41, P396, DOI 10.1183/09031936.00004912

Finnie JW, 2013, INFLAMMOPHARMACOLOGY, V21, P309, DOI 10.1007/s10787-012-0164-2

Fisahn A, 2009, CEREB CORTEX, V19, P612, DOI 10.1093/cercor/bhn107

Ghosh S, 2013, J NEUROSCI, V33, P5053, DOI 10.1523/JNEUROSCI.4361-12.2013

Hedhli N, 2012, CARDIOVASC RES, V93, P516, DOI 10.1093/cvr/cvr352

Hutchinson PJ, 2007, J NEUROTRAUM, V24, P1545, DOI 10.1089/neu.2007.0295

Iivanainen E, 2007, EXP CELL RES, V313, P2896, DOI 10.1016/j.yexcr.2007.03.042

Kalinowski A, 2010, FASEB J, V24, P2567, DOI 10.1096/fj.08-129072

Kanakry CG, 2007, PLOS ONE, V2, DOI 10.1371/journal.pone.0001369

Kwon OB, 2005, J NEUROSCI, V25, P9378, DOI 10.1523/JNEUROSCI.2100-05.2005

Lok J, 2007, J NEUROTRAUM, V24, P1817, DOI 10.1089/neu.2007.0372

Lok J, 2012, TRANSL STROKE RES, V3, pS119, DOI 10.1007/s12975-012-0157-x

Lyman M, 2014, NEUROSCI RES, V79, P1, DOI 10.1016/j.neures.2013.10.004

Maki T, 2013, CNS NEUROL DISORD-DR, V12, P302, DOI 10.2174/1871527311312030004

Makinodan M, 2012, SCIENCE, V337, P1357, DOI 10.1126/science.1220845

Makó V, 2010, CYTOM PART A, V77A, P962, DOI 10.1002/cyto.a.20952

Marballi K, 2010, J MOL MED, V88, P1133, DOI 10.1007/s00109-010-0653-y

Mellergård P, 2011, NEUROSURGERY, V68, P12, DOI 10.1227/NEU.0b013e3181ef2a40

Mencel M, 2013, PLOS ONE, V8, DOI 10.1371/journal.pone.0070338

Norton N, 2006, AM J MED GENET B, V141B, P96, DOI 10.1002/ajmg.b.30236

Ozaki M, 1997, NATURE, V390, P691, DOI 10.1038/37795

Ridder DA, 2011, J EXP MED, V208, P2615, DOI 10.1084/jem.20110398

Rieff HI, 1999, J NEUROSCI, V19, P10757, DOI 10.1523/JNEUROSCI.19-24-10757.1999

Rigor RR, 2012, AM J PHYSIOL-CELL PH, V302, pC1513, DOI 10.1152/ajpcell.00371.2011

Rio C, 1997, NEURON, V19, P39, DOI 10.1016/S0896-6273(00)80346-3

Russell KS, 1999, AM J PHYSIOL-HEART C, V277, pH2205, DOI 10.1152/ajpheart.1999.277.6.H2205

Safa RN, 2011, AM J PHYSIOL-HEART C, V300, pH1311, DOI 10.1152/ajpheart.01104.2009

Schaff U, 2008, J LEUKOCYTE BIOL, V83, P99, DOI 10.1189/jlb.0507304

Tan GH, 2012, NAT NEUROSCI, V15, P258, DOI 10.1038/nn.3005

Ting AK, 2011, J NEUROSCI, V31, P15, DOI 10.1523/JNEUROSCI.2538-10.2011

Wen Y, 2008, J NEUROSCI, V28, P2624, DOI 10.1523/JNEUROSCI.5245-07.2008

Winter CD, 2002, J NEUROSCI METH, V119, P45, DOI 10.1016/S0165-0270(02)00153-X

WOODROOFE MN, 1991, J NEUROIMMUNOL, V33, P227, DOI 10.1016/0165-5728(91)90110-S

Xu ZF, 2004, BIOCHEM BIOPH RES CO, V322, P440, DOI 10.1016/j.bbrc.2004.07.149

NR 44

TC 50

Z9 60

U1 0

U2 19

PU SPRINGER

PI NEW YORK

PA 233 SPRING ST, NEW YORK, NY 10013 USA

SN 1868-4483

EI 1868-601X

J9 TRANSL STROKE RES

JI Transl. Stroke Res.

PD APR

PY 2015

VL 6

IS 2

BP 116

EP 124

DI 10.1007/s12975-014-0347-9

PG 9

WC Clinical Neurology; Neurosciences

WE Science Citation Index Expanded (SCI-EXPANDED)

SC Neurosciences & Neurology

GA CD8AH

UT WOS:000351317000004

PM 24863743

OA Green Accepted

DA 2024-08-12

ER

PT J

AU Lee, HJ

Kim, C

Lee, SJ

AF Lee, He-Jin

Kim, Changyoun

Lee, Seung-Jae

TI Alpha-synuclein stimulation of astrocytes Potential role for

neuroinflammation and neuroprotection

SO OXIDATIVE MEDICINE AND CELLULAR LONGEVITY

LA English

DT Article

DE Parkinson's disease; neuroinflammation; alpha-synuclein; amyloid;

neurodegeneration

ID DISEASE; TRANSMISSION; EXOCYTOSIS; EXPRESSION; CLEARANCE; NEURON

AB Selective loss of neurons, abnormal protein deposition and neuroinflammation are the common pathological features of neurodegenerative disease, and these features are closely related to one another. In Parkinson's disease, abnormal aggregation and deposition of alpha-synuclein is known as a critical event in pathogenesis of the disease, as well as in other related neurodegenerative disorders, such as dementia with Lewy bodies and multiple system atrophy. Increasing evidence suggests that alpha-synuclein aggregates can activate glial cells to induce neuroinflammation. However, how an inflammatory microenvironment is established and maintained by this protein remains unknown. Findings from our recent study suggest that neuronal alpha-synuclein can be directly transferred to astrocytes through sequential exocytosis and endocytosis and induce inflammatory responses from astrocytes. Here we discuss potential roles of astrocytes in a cascade of events leading to alpha-synuclein-induced neuroinflammation.

C1 [Lee, He-Jin] Konkuk Univ, Dept Anat, Sch Med, Seoul, South Korea.

[Kim, Changyoun; Lee, Seung-Jae] Konkuk Univ, Dept Biomed Sci, Seoul, South Korea.

[Lee, He-Jin; Kim, Changyoun; Lee, Seung-Jae] Konkuk Univ, IBST, Seoul, South Korea.

C3 Konkuk University; Konkuk University Medical Center; Konkuk University;

Konkuk University

RP Lee, HJ (corresponding author), Konkuk Univ, Dept Anat, Sch Med, Seoul, South Korea.

EM hjlee@kku.ac.kr; sjlee@knokuk.ac.kr

RI Kim, Changyoun/AAZ-5901-2021

OI Kim, Changyoun/0000-0001-9936-8195; Lee, Seung-Jae/0000-0002-5155-5335

FU Konkuk University

FX This work was supported by the faculty research fund of Konkuk

University in 2007.

CR Bertollini C, 2006, NEUROPHARMACOLOGY, V51, P816, DOI 10.1016/j.neuropharm.2006.05.027

Braak H, 2003, NEUROBIOL AGING, V24, P197, DOI 10.1016/S0197-4580(02)00065-9

Desplats P, 2009, P NATL ACAD SCI USA, V106, P13010, DOI 10.1073/pnas.0903691106

Fahn Stanley, 2004, NeuroRx, V1, P139, DOI 10.1602/neurorx.1.1.139

Farina C, 2007, TRENDS IMMUNOL, V28, P138, DOI 10.1016/j.it.2007.01.005

Farrer MJ, 2006, NAT REV GENET, V7, P306, DOI 10.1038/nrg1831

Hirsch EC, 2005, PARKINSONISM RELAT D, V11, pS9, DOI 10.1016/j.parkreldis.2004.10.013

Jang A, 2010, J NEUROCHEM, V113, P1263, DOI 10.1111/j.1471-4159.2010.06695.x

Kim HC, 2002, MOL BRAIN RES, V108, P60, DOI 10.1016/S0169-328X(02)00514-4

Lee HJ, 2008, INT J BIOCHEM CELL B, V40, P1835, DOI 10.1016/j.biocel.2008.01.017

Lee HJ, 2008, BIOCHEM BIOPH RES CO, V372, P423, DOI 10.1016/j.bbrc.2008.05.045

Lee HJ, 2010, J BIOL CHEM, V285, P9262, DOI 10.1074/jbc.M109.081125

Lee HJ, 2005, J NEUROSCI, V25, P6016, DOI 10.1523/JNEUROSCI.0692-05.2005

Li KR, 2009, J MOL NEUROSCI, V37, P288, DOI 10.1007/s12031-008-9146-7

Mogi M, 1996, NEUROSCI LETT, V211, P13, DOI 10.1016/0304-3940(96)12706-3

Opal SM, 2000, CHEST, V117, P1162, DOI 10.1378/chest.117.4.1162

Reale M, 2009, MINI-REV MED CHEM, V9, P1229, DOI 10.2174/138955709789055199

Satake W, 2009, NAT GENET, V41, P1303, DOI 10.1038/ng.485

Savarin-Vuaillat C, 2007, NEUROTHERAPEUTICS, V4, P590, DOI 10.1016/j.nurt.2007.07.004

Simón-Sánchez J, 2009, NAT GENET, V41, P1308, DOI 10.1038/ng.487

Sofroniew MV, 2010, ACTA NEUROPATHOL, V119, P7, DOI 10.1007/s00401-009-0619-8

Song JH, 2006, J NEUROSCI, V26, P3299, DOI 10.1523/JNEUROSCI.5572-05.2006

Spillantini MG, 1998, P NATL ACAD SCI USA, V95, P6469, DOI 10.1073/pnas.95.11.6469

Yanagida T, 2009, OXID MED CELL LONGEV, V2, P36, DOI 10.4161/oxim.2.1.7985

NR 24

TC 127

Z9 141

U1 1

U2 11

PU HINDAWI LTD

PI LONDON

PA ADAM HOUSE, 3RD FLR, 1 FITZROY SQ, LONDON, W1T 5HF, United Kingdom

SN 1942-0900

EI 1942-0994

J9 OXID MED CELL LONGEV

JI Oxidative Med. Cell. Longev.

PD JUL-AUG

PY 2010

VL 3

IS 4

BP 283

EP 287

DI 10.4161/oxim.3.4.12809

PG 5

WC Cell Biology

WE Science Citation Index Expanded (SCI-EXPANDED)

SC Cell Biology

GA 665EQ

UT WOS:000283018400008

PM 20972375

OA hybrid, Green Published

DA 2024-08-12

ER

PT J

AU Sheng, S

Zhao, S

Zhang, F

AF Sheng, Shuo

Zhao, Shuo

Zhang, Feng

TI Insights into the roles of bacterial infection and antibiotics in

Parkinson's disease

SO FRONTIERS IN CELLULAR AND INFECTION MICROBIOLOGY

LA English

DT Review

DE Parkinson's disease; bacterial infection; neuroinflammation;

antibiotics; anti-PD

ID HELICOBACTER-PYLORI INFECTION; MOTOR DEFICITS; CEFTRIAXONE; RIFAMPICIN;

MODEL; DOXYCYCLINE; ACTIVATION

AB Parkinson's disease (PD) is one of the most common neurodegenerative disorders, which is accompanied with the classical motor symptoms and a range of non-motor symptoms. Bacterial infection affects the neuroinflammation associated with the pathology of PD and various antibiotics have also been confirmed to play an important role not only in bacterial infection, but also in the PD progression. This mini-review summarized the role of common bacterial infection in PD and introduced several antibiotics that had anti-PD effects.

C1 [Sheng, Shuo; Zhang, Feng] Zunyi Med Univ, Key Lab Basic Pharmacol, Minist Educ, Zunyi, China.

[Sheng, Shuo; Zhang, Feng] Zunyi Med Univ, Joint Int Res Lab Ethnomed, Minist Educ, Zunyi, China.

[Sheng, Shuo; Zhang, Feng] Zunyi Med Univ, Key Lab Basic Pharmacol Guizhou Prov, Zunyi, China.

[Zhao, Shuo] Zunyi Med Univ, Sch Basic Med, Electron Microscopy Room, Zunyi, China.

[Zhang, Feng] Zunyi Med Univ, Lab Anim Ctr, Zunyi, China.

C3 Zunyi Medical University; Zunyi Medical University; Zunyi Medical

University; Zunyi Medical University; Zunyi Medical University

RP Zhang, F (corresponding author), Zunyi Med Univ, Key Lab Basic Pharmacol, Minist Educ, Zunyi, China.; Zhang, F (corresponding author), Zunyi Med Univ, Joint Int Res Lab Ethnomed, Minist Educ, Zunyi, China.; Zhang, F (corresponding author), Zunyi Med Univ, Key Lab Basic Pharmacol Guizhou Prov, Zunyi, China.; Zhang, F (corresponding author), Zunyi Med Univ, Lab Anim Ctr, Zunyi, China.

EM zhangfengzmc@163.com

FU special grant of academic new seedling cultivation and innovation

exploration from Guizhou Science and Technology Department [Qian Ke He

Ping Tai Ren Cai [2018]5772-036, Qian Ke Ping Tai Ren Cai [2020]-012];

Science and Technology Foundation of Guizhou Province [ZK[2021]-014];

Science and Technology Project of Zunyi city [372, 286]; Natural Science

Foundation of Guizhou Province [604]

FX The work was supported by special grant of academic new seedling

cultivation and innovation exploration from Guizhou Science and

Technology Department (Qian Ke He Ping Tai Ren Cai [2018]5772-036 and

Qian Ke Ping Tai Ren Cai [2020]-012), Science and Technology Foundation

of Guizhou Province (No. ZK[2021]-014), Science and Technology Project

of Zunyi city (Zun Shi Ke He HZ Zi [2021] No. 286), Natural Science

Foundation of Guizhou Province (Qian Ke He Ji Chu - ZK [2022] No. 604),

and Science and Technology Project of Zunyi city (Zun Shi Ke He HZ Zi

[2022] No. 372).

CR Actis GC, 2019, MINERVA GASTROENTERO, V65, P164, DOI 10.23736/S1121-421X.19.02554-6

Adams B, 2019, FRONT AGING NEUROSCI, V11, DOI 10.3389/fnagi.2019.00210

Alby K, 2016, MICROBIOL SPECTR, V4, DOI 10.1128/microbiolspec.DMIH2-0005-2015

Arru G, 2016, J NEUROIMMUNOL, V293, P86, DOI 10.1016/j.jneuroim.2016.02.016

Balestrino R, 2020, EUR J NEUROL, V27, P27

Bi W, 2013, NEUROL SCI, V34, P137, DOI 10.1007/s10072-012-1156-0

Bisht R, 2014, NEUROTOXICOLOGY, V44, P71, DOI 10.1016/j.neuro.2014.05.009

Blaecher C, 2013, ALIMENT PHARM THER, V38, P1347, DOI 10.1111/apt.12520

Bortolanza M, 2018, J NEURAL TRANSM, V125, P1403, DOI 10.1007/s00702-018-1913-1

Cathcart JM, 2015, FRONT BIOSCI-LANDMRK, V20, P1164, DOI 10.2741/4365

Chen CK, 2017, PEERJ, V5, DOI 10.7717/peerj.3647

Cho DC, 2011, J KOREAN NEUROSURG S, V49, P83, DOI 10.3340/jkns.2011.49.2.83

Chotibut T, 2017, MOVEMENT DISORD, V32, P1547, DOI 10.1002/mds.27077

Cookson MR, 2017, ADV NEUROBIOL, V14, P227, DOI 10.1007/978-3-319-49969-7_12

Corsello SM, 2017, NAT MED, V23, P405, DOI 10.1038/nm.4306

De Chiara G, 2012, MOL NEUROBIOL, V46, P614, DOI 10.1007/s12035-012-8320-7

Deusenbery C, 2021, ACS INFECT DIS, V7, P695, DOI 10.1021/acsinfecdis.0c00890

Dobbs SM, 2016, J NEUROVIROL, V22, P22, DOI 10.1007/s13365-015-0357-8

Faustman DL, 2020, J INTERN MED, V288, P641, DOI 10.1111/joim.13050

Gooch CL, 2017, ANN NEUROL, V81, P479, DOI 10.1002/ana.24897

Haditsch U, 2020, J ALZHEIMERS DIS, V75, P1361, DOI 10.3233/JAD-200393

Hajishengallis G, 2012, NAT REV MICROBIOL, V10, P717, DOI 10.1038/nrmicro2873

Hirsch EC, 2021, MOVEMENT DISORD, V36, P16, DOI 10.1002/mds.28075

Ho SC, 2014, BEHAV BRAIN RES, V268, P177, DOI 10.1016/j.bbr.2014.04.022

Hocevar K, 2018, BIOL CHEM, V399, P1353, DOI 10.1515/hsz-2018-0215

Homayoun H, 2018, ANN INTERN MED, V169, pITC33, DOI 10.7326/AITC201809040

Huang HK, 2018, PARKINSONISM RELAT D, V47, P26, DOI 10.1016/j.parkreldis.2017.11.331

Imamura T, 2001, J BIOL CHEM, V276, P18984, DOI 10.1074/jbc.M006760200

Kadowaki Tomoko, 2021, Methods Mol Biol, V2210, P97, DOI 10.1007/978-1-0716-0939-2_10

Kang X, 2020, EUR J NEUROL, V27, P2134, DOI 10.1111/ene.14400

Kaur Baninder, 2017, Pathophysiology, V24, P71, DOI 10.1016/j.pathophys.2017.02.001

Kim CY, 2017, SEMIN NEUROL, V37, P135, DOI 10.1055/s-0037-1601567

Kim MS, 2020, GUT, V69, P283, DOI 10.1136/gutjnl-2018-317431

Lam HS, 2011, CURR OPIN PEDIATR, V23, P156, DOI 10.1097/MOP.0b013e3283431f2a

Lee HM, 2016, SCHIZOPHR RES TREAT, V2016, DOI 10.1155/2016/6378137

Leffler DA, 2015, NEW ENGL J MED, V373, P287, DOI 10.1056/NEJMc1506004

Liang YR, 2017, NEUROTOXICOLOGY, V63, P137, DOI 10.1016/j.neuro.2017.09.015

Mcgee DJ, 2018, J PARKINSON DIS, V8, P367, DOI 10.3233/JPD-181327

Miklossy J, 2011, EXPERT REV MOL MED, V13, DOI 10.1017/S1462399411002006

Mo WY, 2020, J MOL HISTOL, V51, P147, DOI 10.1007/s10735-020-09865-w

Molloy DW, 2013, INT J GERIATR PSYCH, V28, P463, DOI 10.1002/gps.3846

Moon A, 2012, J INFLAMM-LOND, V9, DOI 10.1186/1476-9255-9-31

Morais LH, 2021, NAT REV MICROBIOL, V19, P241, DOI 10.1038/s41579-020-00460-0

Morris G, 2018, MOL NEUROBIOL, V55, P5767, DOI 10.1007/s12035-017-0793-y

Nerius M, 2020, GUT, V69, P1154, DOI 10.1136/gutjnl-2019-318822

Noto JM, 2017, PLOS PATHOG, V13, DOI 10.1371/journal.ppat.1006573

Nyholm D, 2021, J PARKINSON DIS, V11, P61, DOI 10.3233/JPD-202298

Patrick KL, 2019, FRONT CELL INFECT MI, V9, DOI 10.3389/fcimb.2019.00138

Poewe W, 2017, NAT REV DIS PRIMERS, V3, DOI 10.1038/nrdp.2017.13

Rashed R, 2022, FRONT MED-LAUSANNE, V9, DOI 10.3389/fmed.2022.887044

Reglodi D, 2015, SPRINGERPLUS, V4, DOI 10.1186/2193-1801-4-S1-L56

Romero-Miguel D, 2021, EUR J NEUROL, V28, P1056, DOI 10.1111/ene.14642

Roulis E, 2013, TRENDS MICROBIOL, V21, P120, DOI 10.1016/j.tim.2012.10.009

Ruzza C, 2014, BRIT J PHARMACOL, V171, P4125, DOI 10.1111/bph.12727

Ruzza P, 2014, ACS CHEM NEUROSCI, V5, P30, DOI 10.1021/cn400149k

Sampson TR, 2016, CELL, V167, P1469, DOI 10.1016/j.cell.2016.11.018

Santa-Cecília FV, 2016, NEUROTOX RES, V29, P447, DOI 10.1007/s12640-015-9592-2

Shen S, 2022, J PARKINSON DIS, V12, P27, DOI 10.3233/JPD-212929

Smits WK, 2016, NAT REV DIS PRIMERS, V2, DOI [10.1128/microbiolspec.EI10-0007-2015, 10.1038/nrdp.2016.20]

Socias SB, 2018, PROG NEUROBIOL, V162, P17, DOI 10.1016/j.pneurobio.2017.12.002

Stoilova T, 2013, J MED CHEM, V56, P5987, DOI 10.1021/jm400161p

Sultan S, 2013, FRONT NEUROSCI-SWITZ, V7, DOI 10.3389/fnins.2013.00131

Tambasco N, 2018, CURR NEUROPHARMACOL, V16, P1239, DOI 10.2174/1570159X15666170510143821

Wang Z, 2018, BMC SYST BIOL, V12, DOI 10.1186/s12918-017-0524-z

Weindel CG, 2020, ELIFE, V9, DOI 10.7554/eLife.51071

Yulug B, 2014, BRAIN RES BULL, V107, P37, DOI 10.1016/j.brainresbull.2014.05.007

NR 66

TC 3

Z9 3

U1 0

U2 3

PU FRONTIERS MEDIA SA

PI LAUSANNE

PA AVENUE DU TRIBUNAL FEDERAL 34, LAUSANNE, CH-1015, SWITZERLAND

SN 2235-2988

J9 FRONT CELL INFECT MI

JI Front. Cell. Infect. Microbiol.

PD JUL 28

PY 2022

VL 12

AR 939085

DI 10.3389/fcimb.2022.939085

PG 6

WC Immunology; Microbiology

WE Science Citation Index Expanded (SCI-EXPANDED)

SC Immunology; Microbiology

GA 3R6BU

UT WOS:000838996500001

PM 35967873

OA gold, Green Published

DA 2024-08-12

ER

PT J

AU Tambe, SM

Mali, S

Amin, PD

Oliveira, M

AF Tambe, Srushti M.

Mali, Suraj

Amin, Purnima D.

Oliveira, Mozaniel

TI Neuroprotective potential of cannabidiol: Molecular mechanisms and

clinical implications

SO JOURNAL OF INTEGRATIVE MEDICINE-JIM

LA English

DT Review

DE Cannabis sativa; Cannabidiol; Natural products; Bioactive compounds;

Anti-neurodegenerative disease

ID WNT/BETA-CATENIN PATHWAY; OXIDATIVE STRESS; UP-REGULATION; PPAR-GAMMA;

RECEPTOR; EPILEPSY; TARGETS; CELLS; NEUROINFLAMMATION; INFLAMMATION

AB Cannabidiol (CBD), a nonpsychotropic phytocannabinoid that was once largely disregarded, is currently the subject of significant medicinal study. CBD is found in Cannabis sativa, and has a myriad of neuropharmacological impacts on the central nervous system, including the capacity to reduce neuroinflammation, protein misfolding and oxidative stress. On the other hand, it is well established that CBD generates its biological effects without exerting a large amount of intrinsic activity upon cannabinoid receptors. Because of this, CBD does not produce undesirable psychotropic effects that are typical of marijuana derivatives. Nonetheless, CBD displays the exceptional potential to become a supplementary medicine in various neurological diseases. Currently, many clinical trials are being conducted to investigate this possibility. This review focuses on the therapeutic effects of CBD in managing neurological disorders like Alzheimer's disease, Parkinson's disease and epilepsy. Overall, this review aims to build a stronger understanding of CBD and provide guidance for future fundamental scientific and clinical investigations, opening a new therapeutic window for neuroprotection.Please cite this article as: Tambe SM, Mali S, Amin PD, Oliveira M. Neuroprotective potential of Cannabidiol: Molecular mechanisms and clinical implications. J Integr Med. 2023; 21(3): 236-244 & COPY; 2023 Shanghai Yueyang Hospital Affiliated to Shanghai University of Traditional Chinese Medicine. All rights reserved.

C1 [Tambe, Srushti M.; Amin, Purnima D.] Inst Chem Technol, Dept Pharmaceut Sci & Technol, Mumbai 400019, India.

[Mali, Suraj] Birla Inst Technol, Dept Pharmaceut Sci & Technol, Ranchi 835215, India.

[Oliveira, Mozaniel] Museu Paraense Emilio Goeldi, Adolpho Ducke Lab, BR-66077830 Belem, Para, Brazil.

C3 Institute of Chemical Technology - Mumbai; Birla Institute of Technology

Mesra; Museu Paraense Emilio Goeldi

RP Oliveira, M (corresponding author), Museu Paraense Emilio Goeldi, Adolpho Ducke Lab, BR-66077830 Belem, Para, Brazil.

EM mozaniel.oliveira@yahoo.com.br

RI Tambe, Srushti/HDO-6107-2022; de Oliveira, Mozaniel Santana/L-9553-2017;

MALI, Dr. SURAJ N./W-3936-2019

OI Tambe, Srushti/0000-0003-4892-4889; de Oliveira, Mozaniel

Santana/0000-0002-4076-2443; MALI, Dr. SURAJ N./0000-0003-1995-136X

FU CNPq [300983/2022-0]; PCI-MCTIC/MPEG

FX The author Dr. Mozaniel Santana de Oliveira, thanks PCI-MCTIC/MPEG, as

well as CNPq for the scholarship (process number: 300983/2022-0) .

CR Abu-Sawwa Renad, 2020, J Pediatr Pharmacol Ther, V25, P485, DOI 10.5863/1551-6776-25.6.485

Adams R, 1942, Bull N Y Acad Med, V18, P705

Alzheimers Association, 2015, Alzheimers Dement, V11, P332

Arout CA, 2022, BRIT J CLIN PHARMACO, V88, P347, DOI 10.1111/bcp.14973

Atalay S, 2020, ANTIOXIDANTS-BASEL, V9, DOI 10.3390/antiox9010021

Bisogno T, 2001, BRIT J PHARMACOL, V134, P845, DOI 10.1038/sj.bjp.0704327

Blaskovich MAT, 2021, COMMUN BIOL, V4, DOI 10.1038/s42003-020-01530-y

Brown KJ, 2017, BIOCHEM BIOPH RES CO, V493, P451, DOI 10.1016/j.bbrc.2017.09.001

Burstein S, 2015, BIOORGAN MED CHEM, V23, P1377, DOI 10.1016/j.bmc.2015.01.059

Campos AC, 2017, FRONT PHARMACOL, V8, DOI 10.3389/fphar.2017.00269

Campos AC, 2016, PHARMACOL RES, V112, P119, DOI 10.1016/j.phrs.2016.01.033

Cassano T, 2020, FRONT PHARMACOL, V11, DOI 10.3389/fphar.2020.00124

Celorrio M, 2017, NEUROPHARMACOLOGY, V125, P319, DOI 10.1016/j.neuropharm.2017.08.017

Chandorkar N., 2021, Phytomedicine plus, V1, DOI [10.1016/j.phyplu.2021.100089, DOI 10.3389/FMICB.2020.00409]

Chen C., 2021, J. Future Foods, V1, P113, DOI [DOI 10.1016/J.JFUTFO.2022.01.001, 10.1016/j.jfutfo.2022.01.001]

Chen J, 2016, MOL MED REP, V14, P2321, DOI 10.3892/mmr.2016.5513

Costa AM, 2022, PHARMACEUTICALS-BASE, V15, DOI 10.3390/ph15050495

Crippa JAS, 2019, EUR ARCH PSY CLIN N, V269, P121, DOI 10.1007/s00406-019-00982-6

da Costa LS, 2022, ANTIOXIDANTS-BASEL

Dauer W, 2003, NEURON, V39, P889, DOI 10.1016/S0896-6273(03)00568-3

Daynes RA, 2002, NAT REV IMMUNOL, V2, P748, DOI 10.1038/nri912

de Faria SM, 2020, J PSYCHOPHARMACOL, V34, P189, DOI 10.1177/0269881119895536

de la Monte SM, 2006, J ALZHEIMERS DIS, V9, P167

Faria DD, 2022, INT J MOL SCI, V23, DOI 10.3390/ijms23031076

do Nascimento GC, 2020, NEUROPHARMACOLOGY, V163, DOI 10.1016/j.neuropharm.2019.107808

Do Val-da Silva RA, 2017, FRONT PHARMACOL, V8, DOI 10.3389/fphar.2017.00131

Elliott DM, 2018, FRONT IMMUNOL, V9, DOI 10.3389/fimmu.2018.01782

ElSohly MA, 2005, LIFE SCI, V78, P539, DOI 10.1016/j.lfs.2005.09.011

Esposito G, 2007, BRIT J PHARMACOL, V151, P1272, DOI 10.1038/sj.bjp.0707337

Esposito G, 2006, J MOL MED, V84, P253, DOI 10.1007/s00109-005-0025-1

Esposito G, 2011, PLOS ONE, V6, DOI 10.1371/journal.pone.0028668

Farag S, 2017, HANDBOOK OF CANNABIS AND RELATED PATHOLOGIES: BIOLOGY, PHARMACOLOGY, DIAGNOSIS, AND TREATMENT, P3, DOI 10.1016/B978-0-12-800756-3.00001-6

Fisher RS, 2005, EPILEPSIA, V46, P470, DOI 10.1111/j.0013-9580.2005.66104.x

Franco V, 2019, DRUGS, V79, P1435, DOI 10.1007/s40265-019-01171-4

Frandsen J, 2022, ACS CHEM NEUROSCI, V13, P1165, DOI 10.1021/acschemneuro.1c00667

Freeman TP, 2019, BMJ-BRIT MED J, V365, DOI 10.1136/bmj.l1141

Giacoppo S, 2017, FITOTERAPIA, V116, P77, DOI 10.1016/j.fitote.2016.11.010

Giuliano C, 2021, INT J MOL SCI, V22, DOI 10.3390/ijms22168920

Gray RA, 2020, EPILEPTIC DISORD, V22, pS10, DOI 10.1684/epd.2020.1135

Greene NZ, 2018, PSYCHOPHARMACOLOGY, V235, P3289, DOI 10.1007/s00213-018-5036-z

Gugliandolo A, 2020, FITOTERAPIA, V143, DOI 10.1016/j.fitote.2020.104553

Hampson AJ, 1998, P NATL ACAD SCI USA, V95, P8268, DOI 10.1073/pnas.95.14.8268

Hao FJ, 2021, LIFE SCI, V264, DOI 10.1016/j.lfs.2020.118624

Harris RA, 2014, BIOGERONTOLOGY, V15, P559, DOI 10.1007/s10522-014-9534-z

Hughes B, 2019, NEUROCHEM RES, V44, P703, DOI 10.1007/s11064-018-2513-z

Iuvone T, 2004, J NEUROCHEM, V89, P134, DOI 10.1111/j.1471-4159.2003.02327.x

Jadoon KA, 2016, DIABETES CARE, V39, P1777, DOI 10.2337/dc16-0650

Janisset NRLL, 2022, EPILEPSY BEHAV, V129, DOI 10.1016/j.yebeh.2022.108615

Jastrzab A, 2021, OXID MED CELL LONGEV, V2021, DOI 10.1155/2021/6647222

JONES PG, 1977, ACTA CRYSTALLOGR B, V33, P3211, DOI 10.1107/S0567740877010577

Khan AA, 2018, BRIT J PHARMACOL, V175, P2097, DOI 10.1111/bph.14202

Khodadadi H, 2021, J ALZHEIMERS DIS, V80, P973, DOI 10.3233/JAD-210026

Klein BD, 2017, NEUROCHEM RES, V42, P1939, DOI 10.1007/s11064-017-2287-8

Klimaszewska-Wisniewska A, 2017, ACTA HISTOCHEM, V119, P99, DOI 10.1016/j.acthis.2016.11.003

Kozela E, 2010, J BIOL CHEM, V285, P1616, DOI 10.1074/jbc.M109.069294

Laprairie RB, 2015, BRIT J PHARMACOL, V172, P4790, DOI 10.1111/bph.13250

Lattanzi S, 2018, DRUGS, V78, P1791, DOI 10.1007/s40265-018-0992-5

Laun AS, 2017, BIOCHEM BIOPH RES CO, V490, P17, DOI 10.1016/j.bbrc.2017.05.165

Ltd. GR, 2014, STUD ASS EFF CANN LI

Mali SN, 2022, ESSENTIAL OILS APPL, P417, DOI DOI 10.1007/978-3-030-99476-1_18

McPartland JM, 2015, BRIT J PHARMACOL, V172, P737, DOI 10.1111/bph.12944

Mechoulam R, 2002, CHEM PHYS LIPIDS, V121, P35, DOI 10.1016/S0009-3084(02)00144-5

MECHOULAM R, 1963, TETRAHEDRON, V19, P2073, DOI 10.1016/0040-4020(63)85022-X

Melas PA, 2021, INT J MOL SCI, V22, DOI 10.3390/ijms22041863

O'Sullivan SE, 2009, EUR J PHARMACOL, V612, P61, DOI 10.1016/j.ejphar.2009.03.010

Pacifici R, 2017, CLIN CHEM LAB MED, V55, P1555, DOI 10.1515/cclm-2016-1060

Pan H, 2009, J PHARMACOL EXP THER, V328, P708, DOI 10.1124/jpet.108.147181

Park KS, 2004, EXP CELL RES, V297, P424, DOI 10.1016/j.yexcr.2004.03.034

Peres FF, 2018, FRONT PHARMACOL, V9, DOI 10.3389/fphar.2018.00482

Pertwee RG, 1999, CURR MED CHEM, V6, P635

Petrovici AR, 2021, ANTIOXIDANTS-BASEL, V10, DOI 10.3390/antiox10050738

Rajan TS, 2016, FITOTERAPIA, V112, P104, DOI 10.1016/j.fitote.2016.05.008

Rajesh M, 2010, J AM COLL CARDIOL, V56, P2115, DOI 10.1016/j.jacc.2010.07.033

Risner ME, 2006, PHARMACOGENOMICS J, V6, P246, DOI 10.1038/sj.tpj.6500369

Rock EM, 2012, BRIT J PHARMACOL, V165, P2620, DOI 10.1111/j.1476-5381.2011.01621.x

Rout S, 2022, TRENDS FOOD SCI TECH, V129, P421, DOI 10.1016/j.tifs.2022.10.012

Russo EB, 2005, NEUROCHEM RES, V30, P1037, DOI 10.1007/s11064-005-6978-1

Ryberg E, 2007, BRIT J PHARMACOL, V152, P1092, DOI 10.1038/sj.bjp.0707460

Saleem S, 2020, EUR ARCH PSY CLIN N, V270, P135, DOI 10.1007/s00406-019-01023-y

Scuderi C, 2014, PHYTOTHER RES, V28, P1007, DOI 10.1002/ptr.5095

Shannon Scott, 2019, Perm J, V23, P18, DOI 10.7812/TPP/18-041

Showalter VM, 1996, J PHARMACOL EXP THER, V278, P989

Thomas A, 2007, BRIT J PHARMACOL, V150, P613, DOI 10.1038/sj.bjp.0707133

Thomas BF, 1998, J PHARMACOL EXP THER, V285, P285

Thurman DJ, 2011, EPILEPSIA, V52, P2, DOI 10.1111/j.1528-1167.2011.03121.x

TODD AR, 1946, EXPERIENTIA, V2, P55, DOI 10.1007/BF02163886

Vallée A, 2017, ACTA BIOCH BIOPH SIN, V49, P853, DOI 10.1093/abbs/gmx073

Vallée A, 2016, FRONT NEUROSCI-SWITZ, V10, DOI 10.3389/fnins.2016.00459

WATANABE K, 1991, J PHARMACOBIO-DYNAM, V14, P421, DOI 10.1248/bpb1978.14.421

WATZL B, 1991, ADV EXP MED BIOL, V288, P63

Weiss L, 2006, AUTOIMMUNITY, V39, P143, DOI 10.1080/08916930500356674

Willems LM, 2018, EPILEPSY BEHAV, V83, P28, DOI 10.1016/j.yebeh.2018.03.025

Wise J, 2019, BMJ-BRIT MED J, V366, DOI 10.1136/bmj.l5708

Yue XA, 2010, BRAIN RES, V1366, P27, DOI 10.1016/j.brainres.2010.10.032

NR 94

TC 6

Z9 6

U1 4

U2 14

PU ELSEVIER

PI AMSTERDAM

PA RADARWEG 29, 1043 NX AMSTERDAM, NETHERLANDS

SN 2095-4964

J9 J INTEGR MED-JIM

JI J. Integr. Med.-JIM

PD MAY

PY 2023

VL 21

IS 3

BP 236

EP 244

DI 10.1016/j.joim.2023.03.004

EA MAY 2023

PG 9

WC Integrative & Complementary Medicine

WE Science Citation Index Expanded (SCI-EXPANDED)

SC Integrative & Complementary Medicine

GA M3WM1

UT WOS:001029528000001

PM 36973157

DA 2024-08-12

ER

PT J

AU Worlitzer, MMA

Bunk, EC

Hemmer, K

Schwamborn, JC

AF Worlitzer, Maik M. A.

Bunk, Eva C.

Hemmer, Kathrin

Schwamborn, Jens C.

TI Anti-inflammatory treatment induced regenerative oligodendrogenesis in

parkinsonian mice

SO STEM CELL RESEARCH & THERAPY

LA English

DT Article

ID NEURAL STEM-CELLS; ADULT HUMAN OLIGODENDROCYTES; SUBVENTRICULAR ZONE;

SUBSTANTIA-NIGRA; DISEASE BRAINS; INDUCED DEATH; PROLIFERATION;

INFLAMMATION; NEUROGENESIS; DIFFERENTIATION

AB Introduction: The adult mammalian brain retains niches for neural stem cells (NSCs), which can generate glial and neuronal components of the brain tissue. However, it is barely established how chronic neuroinflammation, as it occurs in neurodegenerative disease, such as Alzheimer's and Parkinson's disease, affects adult neurogenesis and, therefore, modulates the brain's potential for self-regeneration.

Methods: Neural stem cell culture techniques, intraventricular tumor necrosis factor (TNF)-alpha infusion and the 6-hydroxydopamine mouse model were used to investigate the influence of neuroinflammation on adult neurogenesis in the Parkinson's disease background. Microscopic methods and behavioral tests were used to analyze samples.

Results: Here, we demonstrate that differences in the chronicity of TNF-alpha application to cultured NSCs result in opposed effects on their proliferation. However, chronic TNF-alpha treatment, mimicking Parkinson's disease associated neuroinflammation, shows detrimental effects on neural progenitor cell activity. Inversely, pharmacological inhibition of neuroinflammation in a 6-hydroxydopamine mouse model led to increased neural progenitor cell proliferation in the subventricular zone and neuroblast migration into the lesioned striatum. Four months after surgery, we measured improved Parkinson's disease-associated behavior, which was correlated with long-term anti-inflammatory treatment. But surprisingly, instead of newly generated striatal neurons, oligodendrogenesis in the striatum of treated mice was enhanced.

Conclusions: We conclude that anti-inflammatory treatment, in a 6-hydroxydopamine mouse model for Parkinson's disease, leads to activation of adult neural stem cells. These adult neural stem cells generate striatal oligodendrocytes. The higher numbers of newborn oligodendrocytes possibly contribute to axonal stability and function in this mouse model of Parkinson's disease and thereby attenuate dysfunctions of basalganglian motorcontrol.

C1 [Worlitzer, Maik M. A.; Bunk, Eva C.; Hemmer, Kathrin; Schwamborn, Jens C.] Univ Munster, ZMBE, Inst Cell Biol, Stem Cell Biol & Regenerat Grp, D-48149 Munster, Germany.

[Worlitzer, Maik M. A.; Schwamborn, Jens C.] Interdisciplinary Ctr Clin Res IZKF Munster, D-48149 Munster, Germany.

C3 University of Munster

RP Schwamborn, JC (corresponding author), Univ Munster, ZMBE, Inst Cell Biol, Stem Cell Biol & Regenerat Grp, Von Esmarch Str 56, D-48149 Munster, Germany.

EM jschwamb@uni-muenster.de

RI Schwamborn, Jens C/F-9395-2011

OI Schwamborn, Jens C/0000-0003-4496-0559

FU German Research Foundation (DFG) [SCHW1392/2-1, SFB629, SPP1356,

SCHW1392/4-1]; Kompetenznetzwerk Stammzellforschung NRW; Fonds der

Chemischen Industrie; German-Israeli Foundation (GIF) for Scientific

Research and Development [G-2226-2034.1/2009]; Schram-Stiftung

[T287/21795/2011]; Else Krner-Fresenius-Stiftung [2011_A94]; EU FP7

network EuroSyStem; University of Munster Medical School [SC120901,

SC411003]; Interdisciplinary Center for Clinical Research (IZKF) Munster

[SchwJ3/001/11]

FX JCS's lab is supported by the German Research Foundation (DFG: Emmy

Noether Program, SCHW1392/2-1; SFB629 and SPP1356, SCHW1392/4-1),

Kompetenznetzwerk Stammzellforschung NRW, Fonds der Chemischen

Industrie, German-Israeli Foundation (GIF) for Scientific Research and

Development (G-2226-2034.1/2009), Schram-Stiftung (T287/21795/2011),

Else Krner-Fresenius-Stiftung (2011_A94) and EU FP7 network EuroSyStem.

Furthermore, this work was supported by the fund Innovative Medical

Research of the University of Munster Medical School (SC120901 and

SC411003) and the Interdisciplinary Center for Clinical Research (IZKF)

Munster (SchwJ3/001/11).

CR Alvarez-Buylla A, 2004, NEURON, V41, P683, DOI 10.1016/S0896-6273(04)00111-4

Arvidsson A, 2002, NAT MED, V8, P963, DOI 10.1038/nm747

Baker SA, 2004, EUR J NEUROSCI, V20, P575, DOI 10.1111/j.1460-9568.2004.03486.x

Bastos GN, 2008, NEUROSCIENCE, V155, P454, DOI 10.1016/j.neuroscience.2008.06.020

BERNHEIMER H, 1973, J NEUROL SCI, V20, P415, DOI 10.1016/0022-510X(73)90175-5

Bilousova TV, 2009, J MED GENET, V46, P94, DOI 10.1136/jmg.2008.061796

Braak H, 2004, NEUROBIOL AGING, V25, P19, DOI 10.1016/j.neurobiolaging.2003.04.001

Cacci E, 2008, GLIA, V56, P412, DOI 10.1002/glia.20616

Campbell BCV, 2001, J NEUROCHEM, V76, P87, DOI 10.1046/j.1471-4159.2001.00021.x

Carpenter AE, 2006, GENOME BIOL, V7, DOI 10.1186/gb-2006-7-10-r100

Cicchetti F, 2002, EUR J NEUROSCI, V15, P991, DOI 10.1046/j.1460-9568.2002.01938.x

Conti L, 2005, PLOS BIOL, V3, P1594, DOI 10.1371/journal.pbio.0030283

Cooper O, 2004, J NEUROSCI, V24, P8924, DOI 10.1523/JNEUROSCI.2344-04.2004

Crosio C, 2006, CELL DEATH DIFFER, V13, P2150, DOI 10.1038/sj.cdd.4401943

da Conceicao F. S., 2010, J VIS EXP

Das S, 2011, PLOS ONE, V6, DOI 10.1371/journal.pone.0017225

Ekdahl CT, 2003, P NATL ACAD SCI USA, V100, P13632, DOI 10.1073/pnas.2234031100

Gandhi PN, 2009, J NEUROSCI RES, V87, P1283, DOI 10.1002/jnr.21949

Gao HM, 2008, TRENDS IMMUNOL, V29, P357, DOI 10.1016/j.it.2008.05.002

Glass CK, 2010, CELL, V140, P918, DOI 10.1016/j.cell.2010.02.016

Griffiths MR, 2009, J NEUROPATH EXP NEUR, V68, P217, DOI 10.1097/NEN.0b013e3181996688

He Y, 2001, BRAIN RES, V909, P187, DOI 10.1016/S0006-8993(01)02681-6

Hillje AL, 2011, STEM CELLS, V29, P1437, DOI 10.1002/stem.687

Hoehn BD, 2005, STROKE, V36, P2718, DOI 10.1161/01.STR.0000190020.30282.cc

Höglinger GU, 2004, NAT NEUROSCI, V7, P726, DOI 10.1038/nn1265

Iosif RE, 2006, J NEUROSCI, V26, P9703, DOI 10.1523/JNEUROSCI.2723-06.2006

JEON BS, 1995, NEURODEGENERATION, V4, P131, DOI 10.1006/neur.1995.0016

Johansson S, 2008, STEM CELLS, V26, P2444, DOI 10.1634/stemcells.2008-0116

Jurewicz A, 2005, BRAIN, V128, P2675, DOI 10.1093/brain/awh627

Jurewicz A, 2003, BRAIN, V126, P1358, DOI 10.1093/brain/awg146

Juurlink BHJ, 1997, NEUROSCI BIOBEHAV R, V21, P151, DOI 10.1016/S0149-7634(96)00005-X

Mathieu P, 2010, J NEUROCHEM, V112, P1368, DOI 10.1111/j.1471-4159.2009.06548.x

MCGEER PL, 1988, NEUROLOGY, V38, P1285, DOI 10.1212/WNL.38.8.1285

Miklossy J, 2006, J NEUROPATH EXP NEUR, V65, P953, DOI 10.1097/01.jnen.0000235121.98052.54

Ming GL, 2011, NEURON, V70, P687, DOI 10.1016/j.neuron.2011.05.001

Nait-Oumesmar B, 2007, P NATL ACAD SCI USA, V104, P4694, DOI 10.1073/pnas.0606835104

Nakaguchi K, 2011, NEUROL RES INT, V2011, DOI 10.1155/2011/898012

Ng SY, 2012, J NEUROTRAUMA

Palm T, 2012, RNA BIOL, V9, P720, DOI 10.4161/rna.19984

Picard-Riera N, 2002, P NATL ACAD SCI USA, V99, P13211, DOI 10.1073/pnas.192314199

Pizza V, 2011, REV RECENT CLIN TRIA, V6, P189, DOI 10.2174/157488711796575577

Russo I, 2011, J NEUROCHEM, V116, P947, DOI 10.1111/j.1471-4159.2010.07168.x

Takagi S, 2007, J NEURAL TRANSM, V114, P1553, DOI 10.1007/s00702-007-0790-9

UNGERSTEDT U, 1970, Brain Research, V24, P485, DOI 10.1016/0006-8993(70)90187-3

van den Berge SA, 2011, BRAIN, V134, P3249, DOI 10.1093/brain/awr256

Wakabayashi K, 2000, ACTA NEUROPATHOL, V99, P14, DOI 10.1007/PL00007400

Widera D, 2006, BMC NEUROSCI, V7, DOI 10.1186/1471-2202-7-64

Winner B, 2008, J NEUROPATH EXP NEUR, V67, P105, DOI 10.1097/nen.0b013e3181630cff

Yong VW, 2004, LANCET NEUROL, V3, P744, DOI 10.1016/S1474-4422(04)00937-8

Zhao CM, 2008, CELL, V132, P645, DOI 10.1016/j.cell.2008.01.033

NR 50

TC 16

Z9 16

U1 1

U2 15

PU BIOMED CENTRAL LTD

PI LONDON

PA 236 GRAYS INN RD, FLOOR 6, LONDON WC1X 8HL, United Kingdom

SN 1757-6512

J9 STEM CELL RES THER

JI Stem Cell Res. Ther.

PD AUG 14

PY 2012

VL 3

AR 33

DI 10.1186/scrt124

PG 12

WC Cell & Tissue Engineering; Cell Biology; Medicine, Research &

Experimental

WE Science Citation Index Expanded (SCI-EXPANDED)

SC Cell Biology; Research & Experimental Medicine

GA 041NW

UT WOS:000311409500001

PM 22892385

OA Green Published, gold

DA 2024-08-12

ER

PT J

AU Deng, I

Bobrovskaya, L

AF Deng, Isaac

Bobrovskaya, Larisa

TI Lipopolysaccharide mouse models for Parkinson's disease research: a

critical appraisal

SO NEURAL REGENERATION RESEARCH

LA English

DT Review

DE C57BL/6 mice; intranasal models; lipopolysaccharide models;

neuroinflammation; Parkinson's disease; stereotaxic models; substantia

nigra; systemic models

ID PROTECTS DOPAMINERGIC-NEURONS; LPS-INDUCED NEUROINFLAMMATION; MICROGLIAL

ACTIVATION; INDUCED NEUROTOXICITY; NADPH OXIDASE; ANIMAL-MODELS;

INFLAMMATION; NEURODEGENERATION; ENDOTOXIN; SUSCEPTIBILITY

AB Parkinson's disease, the most common movement disorder, has a strong neuroinflammatory aspect. This is evident by increased pro-inflammatory cytokines in the serum, and the presence of activated microglial cells, and inflammatory cytokines in the substantia nigra of post-mortem brains as well as cerebrospinal fluid of Parkinson's disease patients. The central and peripheral neuroinflammatory aspects of Parkinson's disease can be investigated in vivo via administration of the inflammagen lipopolysaccharide, a component of the cell wall of gram-negative bacteria. In this mini-review, we will critically evaluate different routes of lipopolysaccharide administration (including intranasal systemic and stereotasic), their relevance to clinical Parkinson's disease as well as the recent findings in lipopolysaccharide mouse models. We will also share our own experiences with systemic and intrastriatal lipopolysaccharide models in C57BL/6 mice and will discuss the usefulness of lipopolysaccharide mouse models for future research in the field.

C1 [Deng, Isaac; Bobrovskaya, Larisa] Univ South Australia, Clin & Hlth Sci, Hlth & Biomed Innovat, Adelaide, SA, Australia.

C3 University of South Australia

RP Bobrovskaya, L (corresponding author), Univ South Australia, Clin & Hlth Sci, Hlth & Biomed Innovat, Adelaide, SA, Australia.

EM Larisa.Bobrovskaya@unisa.edu.au

RI Bobrovskaya, Larisa/D-3573-2011

OI Bobrovskaya, Larisa/0000-0002-5793-6147; Deng, Isaac

(Bul)/0000-0002-8088-3469

CR Adams B, 2019, FRONT AGING NEUROSCI, V11, DOI 10.3389/fnagi.2019.00210

Badshah H, 2016, J NEUROIMMUNE PHARM, V11, P48, DOI 10.1007/s11481-015-9623-z

Beier EE, 2017, NEUROBIOL DIS, V108, P115, DOI 10.1016/j.nbd.2017.08.009

Block ML, 2007, NAT REV NEUROSCI, V8, P57, DOI 10.1038/nrn2038

Bodea LG, 2014, J NEUROSCI, V34, P8546, DOI 10.1523/JNEUROSCI.5002-13.2014

Borghammer P, 2019, J PARKINSON DIS, V9, pS281, DOI 10.3233/JPD-191721

Brudek T, 2019, J PARKINSON DIS, V9, pS331, DOI 10.3233/JPD-191729

Chang CC, 2018, ANN MED, V50, P83, DOI 10.1080/07853890.2017.1412088

Chen CK, 2017, PEERJ, V5, DOI 10.7717/peerj.3647

Dantzer R, 2008, NAT REV NEUROSCI, V9, P46, DOI 10.1038/nrn2297

Deneyer L, 2019, PARKINSONISM RELAT D, V68, P26, DOI 10.1016/j.parkreldis.2019.09.025

Deng I, 2021, INT J MOL SCI, V22, DOI 10.3390/ijms22147380

Deng I, 2021, NEUROTOXICOLOGY, V85, P254, DOI 10.1016/j.neuro.2021.05.015

Deng IS, 2020, BRAIN BEHAV IMMUN-HL, V4, DOI 10.1016/j.bbih.2020.100060

Doty RL, 2012, NAT REV NEUROL, V8, P329, DOI 10.1038/nrneurol.2012.80

Duty S, 2011, BRIT J PHARMACOL, V164, P1357, DOI 10.1111/j.1476-5381.2011.01426.x

Forsyth CB, 2011, PLOS ONE, V6, DOI 10.1371/journal.pone.0028032

Gao HM, 2008, J NEUROSCI, V28, P7687, DOI 10.1523/JNEUROSCI.0143-07.2008

García C, 2018, J NEUROINFLAMM, V15, DOI 10.1186/s12974-018-1060-5

Garrido-Gil P, 2018, MOL NEUROBIOL, V55, P7297, DOI 10.1007/s12035-018-0937-8

Gómez-Gálvez Y, 2016, PROG NEURO-PSYCHOPH, V64, P200, DOI 10.1016/j.pnpbp.2015.03.017

Gorecki AM, 2019, FRONT NEUROSCI-SWITZ, V13, DOI 10.3389/fnins.2019.00839

Halliday GM, 2010, ANN NY ACAD SCI, V1184, P188, DOI 10.1111/j.1749-6632.2009.05118.x

Hasegawa S, 2015, PLOS ONE, V10, DOI 10.1371/journal.pone.0142164

He Q, 2016, EUR J NEUROSCI, V43, P41, DOI 10.1111/ejn.13132

He Q, 2013, PLOS ONE, V8, DOI 10.1371/journal.pone.0078418

Henry J, 2010, PARKINSONISM RELAT D, V16, P566, DOI 10.1016/j.parkreldis.2010.06.012

Hunter R, 2017, NEUROSCI RES, V114, P62, DOI 10.1016/j.neures.2016.09.007

Hunter RL, 2007, J NEUROCHEM, V100, P1375, DOI 10.1111/j.1471-4159.2006.04327.x

Hunter RL, 2009, J NEUROSCI RES, V87, P1913, DOI 10.1002/jnr.22012

Jiang MJ, 2017, MOL MED REP, V16, P6184, DOI 10.3892/mmr.2017.7352

Jiminez-Ferrer I, 2018, PARKINSONS DIS PATHO, DOI DOI 10.15586/CODONPUBLICATIONS.PARKINSONSDISEASE.2018.CH5

Ju UH, 2019, MEDICINE, V98, DOI 10.1097/MD.0000000000014984

Kelly LP, 2014, MOVEMENT DISORD, V29, P999, DOI 10.1002/mds.25736

Khan MS, 2019, MOL NEUROBIOL, V56, P671, DOI 10.1007/s12035-018-1101-1

Kim WG, 2000, J NEUROSCI, V20, P6309, DOI 10.1523/JNEUROSCI.20-16-06309.2000

Konsman JP, 2002, TRENDS NEUROSCI, V25, P154, DOI 10.1016/S0166-2236(00)02088-9

Kordower JH, 2013, BRAIN, V136, P2419, DOI 10.1093/brain/awt192

Li YH, 2015, METAB BRAIN DIS, V30, P1217, DOI 10.1007/s11011-015-9698-5

Liao H, 2021, GLIA, V69, P2845, DOI 10.1002/glia.24073

Liu YX, 2008, NEUROTOXICOLOGY, V29, P864, DOI 10.1016/j.neuro.2008.02.014

Matheoud D, 2019, NATURE, V571, P565, DOI 10.1038/s41586-019-1405-y

Meneses G, 2018, PLOS ONE, V13, DOI 10.1371/journal.pone.0201375

Milde S, 2021, J NEUROINFLAMMATION, V18, P1

Niehaus I, 2003, OCCUP ENVIRON MED, V60, P378, DOI 10.1136/oem.60.5.378

Niu HC, 2020, BRAIN PATHOL, V30, P1102, DOI 10.1111/bpa.12886

Obeso JA, 2010, NAT MED, V16, P653, DOI 10.1038/nm.2165

Pajares M, 2020, CELLS-BASEL, V9, DOI 10.3390/cells9071687

Pardon MC, 2015, ROM J MORPHOL EMBRYO, V56, P903

Park S, 2019, J CLIN MED, V8, DOI 10.3390/jcm8081191

Perry VH, 2010, ACTA NEUROPATHOL, V120, P277, DOI 10.1007/s00401-010-0722-x

Piirsalu M, 2020, FRONT PHARMACOL, V11, DOI 10.3389/fphar.2020.00371

Pulendran B, 2001, J IMMUNOL, V167, P5067, DOI 10.4049/jimmunol.167.9.5067

Qin LY, 2007, GLIA, V55, P453, DOI 10.1002/glia.20467

Qin LY, 2013, GLIA, V61, P855, DOI 10.1002/glia.22479

Qin LY, 2004, J BIOL CHEM, V279, P1415, DOI 10.1074/jbc.M307657200

Rocha-Ferreira E, 2015, NEUROSCIENCE, V311, P292, DOI 10.1016/j.neuroscience.2015.10.035

Saijo K, 2009, CELL, V137, P47, DOI 10.1016/j.cell.2009.01.038

Song GB, 2020, NEUROSCI LETT, V724, DOI 10.1016/j.neulet.2020.134869

Song S, 2019, MOL NEUROBIOL, V56, P2653, DOI 10.1007/s12035-018-1235-1

Tansey MG, 2010, NEUROBIOL DIS, V37, P510, DOI 10.1016/j.nbd.2009.11.004

Zhang FX, 2018, BIOMED PHARMACOTHER, V97, P1011, DOI 10.1016/j.biopha.2017.08.132

Zhang Z, 2012, MOL MED REP, V5, P562, DOI 10.3892/mmr.2011.671

Zhao YF, 2018, AGING DIS, V9, P785, DOI 10.14336/AD.2017.1028

Zheng HF, 2013, PLOS ONE, V8, DOI 10.1371/journal.pone.0070472

NR 65

TC 6

Z9 6

U1 3

U2 18

PU WOLTERS KLUWER MEDKNOW PUBLICATIONS

PI MUMBAI

PA WOLTERS KLUWER INDIA PVT LTD , A-202, 2ND FLR, QUBE, C T S NO 1498A-2

VILLAGE MAROL, ANDHERI EAST, MUMBAI, Maharashtra, INDIA

SN 1673-5374

EI 1876-7958

J9 NEURAL REGEN RES

JI Neural Regen. Res.

PD NOV

PY 2022

VL 17

IS 11

BP 2413

EP 2417

DI 10.4103/1673-5374.331866

PG 5

WC Cell Biology; Neurosciences

WE Science Citation Index Expanded (SCI-EXPANDED)

SC Cell Biology; Neurosciences & Neurology

GA 5F5JL

UT WOS:000866351500033

PM 35535880

OA Green Published, gold

DA 2024-08-12

ER

PT J

AU Maguire-Zeiss, KA

Federoff, HJ

AF Maguire-Zeiss, Kathleen A.

Federoff, Howard J.

TI Future directions for immune modulation in neurodegenerative disorders:

focus on Parkinson's disease

SO JOURNAL OF NEURAL TRANSMISSION

LA English

DT Review

DE Parkinson's disease; Neuroinflammation; Nurr1

ID VASOACTIVE-INTESTINAL-PEPTIDE; ACTIVATING POLYPEPTIDE INHIBIT;

CENTRAL-NERVOUS-SYSTEM; ALPHA-SYNUCLEIN; MICROGLIAL ACTIVATION;

DOPAMINERGIC-NEURONS; IN-VIVO; GENE-THERAPY; RISK-FACTORS; OPEN-LABEL

AB One common feature of neurodegenerative disease is neuroinflammation. In the case of Parkinson's disease (PD), neuroinflammation appears early and persists throughout the disease course. The principal cellular mediator of brain inflammation is the resident microglia which share many features with related hematopoietically derived macrophages. Microglia can become activated by misfolded proteins including the PD relevant example, alpha-synuclein, a presynaptic protein. When activated, microglia release pro-inflammatory diffusible mediators that promote dysfunction and contribute to the death of the PD vulnerable dopaminergic neurons in the midbrain. Recently, the orphan nuclear receptor Nurr1, well known as a critical determinant in dopaminergic neuron maturation, has been ascribed two new properties. First, it promotes the production and release of the neuropeptide vasoactive intestinal peptide that functions both to stimulate dopaminergic neuron survival and inhibit neuroinflammation. Second, Nurr1 suppresses the expression and release of pro-inflammatory cytokines in glial cells. Herein, we discuss these new findings in context of strategies to attenuate neuroinflammation in PD.

C1 [Maguire-Zeiss, Kathleen A.; Federoff, Howard J.] Georgetown Univ, Med Ctr, Dept Neurosci, Washington, DC 20007 USA.

[Federoff, Howard J.] Georgetown Univ, Med Ctr, Dept Neurol, Washington, DC 20007 USA.

C3 Georgetown University; Georgetown University

RP Federoff, HJ (corresponding author), Georgetown Univ, Med Ctr, Dept Neurosci, Washington, DC 20007 USA.

EM hjf8@georgetown.edu

OI Maguire-Zeiss, Kathleen/0000-0002-2868-848X

CR Ahn TB, 2008, NEUROLOGY, V70, P43, DOI 10.1212/01.wnl.0000271080.53272.c7

Alavian KN, 2008, MOVEMENT DISORD, V23, P319, DOI 10.1002/mds.21640

Banati RB, 2002, GLIA, V40, P206, DOI 10.1002/glia.10144

Bartels AL, 2010, PARKINSONISM RELAT D, V16, P57, DOI 10.1016/j.parkreldis.2009.05.005

Bartels AL, 2007, MOVEMENT DISORD, V22, P1852, DOI 10.1002/mds.21552

Bensinger SJ, 2009, CELL, V137, P26, DOI 10.1016/j.cell.2009.03.024

Braak H, 2008, NEUROLOGY, V70, P1916, DOI 10.1212/01.wnl.0000312279.49272.9f

Brenneman DE, 1998, ANN NY ACAD SCI, V865, P207, DOI 10.1111/j.1749-6632.1998.tb11180.x

Brooks DJ, 2007, PARKINSONISM RELAT D, V13, pS268, DOI 10.1016/S1353-8020(08)70015-4

Bucciantini M, 2002, NATURE, V416, P507, DOI 10.1038/416507a

Chesselet MF, 2008, EXP NEUROL, V209, P22, DOI 10.1016/j.expneurol.2007.08.006

Conway KA, 2000, P NATL ACAD SCI USA, V97, P571, DOI 10.1073/pnas.97.2.571

Dejda A, 2005, PHARMACOL REP, V57, P307

Delgado M, 2003, FASEB J, V17, P1922, DOI 10.1096/fj.02-1029fje

Delgado M, 2003, FASEB J, V17, P944, DOI 10.1096/fj.02-0799fje

Delgado M, 2003, J LEUKOCYTE BIOL, V73, P155, DOI 10.1189/jlb.0702372

Delgado M, 2002, GLIA, V39, P148, DOI 10.1002/glia.10098

Dorsey ER, 2007, NEUROLOGY, V68, P384, DOI 10.1212/01.wnl.0000247740.47667.03

Eberling JL, 2008, NEUROLOGY, V70, P1980, DOI 10.1212/01.wnl.0000312381.29287.ff

El-Agnaf OMA, 2001, J MOL BIOL, V310, P157, DOI 10.1006/jmbi.2001.4743

El-Agnaf OMA, 2003, LANCET NEUROL, V2, P461, DOI 10.1016/S1474-4422(03)00481-2

Federoff HJ, 2009, NEUROTOX RES, V16, P261, DOI 10.1007/s12640-009-9056-7

Gerhard A, 2006, NEUROBIOL DIS, V21, P404, DOI 10.1016/j.nbd.2005.08.002

Graeber MB, 2010, ACTA NEUROPATHOL, V119, P89, DOI 10.1007/s00401-009-0622-0

Grinberg LT, 2010, J NEUROL SCI, V289, P81, DOI 10.1016/j.jns.2009.08.021

Imamura K, 2003, ACTA NEUROPATHOL, V106, P518, DOI 10.1007/s00401-003-0766-2

Jankovic J, 2005, PROG NEUROBIOL, V77, P128, DOI 10.1016/j.pneurobio.2005.09.001

Kaplitt MG, 2007, LANCET, V369, P2097, DOI 10.1016/S0140-6736(07)60982-9

Kaufmann H, 2004, NEUROLOGY, V63, P1093, DOI 10.1212/01.WNL.0000138500.73671.DC

Khoo Tien K, 2009, Practitioner, V253, P19

Krogh K, 2008, ACTA NEUROL SCAND, V117, P60, DOI 10.1111/j.1600-0404.2007.00900.x

Krüger R, 1998, NAT GENET, V18, P106, DOI 10.1038/ng0298-106

Lapointe Nicolas, 2004, FASEB J, V18, P717

Le W, 1999, J NEUROCHEM, V73, P2218

Le WD, 2003, NAT GENET, V33, P85, DOI 10.1038/ng1066

Le WD, 2008, J NEUROL SCI, V273, P29, DOI 10.1016/j.jns.2008.06.007

Lee HJ, 2010, J BIOL CHEM, V285, P9262, DOI 10.1074/jbc.M109.081125

Luo Y, 2008, J NEUROSCI, V28, P9277, DOI 10.1523/JNEUROSCI.3021-08.2008

Luo Y, 2007, EXP NEUROL, V203, P221, DOI 10.1016/j.expneurol.2006.08.005

Maguire-Zeiss KA, 2009, J NEUROIMMUNE PHARM, V4, P298, DOI 10.1007/s11481-008-9133-3

Maguire-Zeiss KA, 2008, PHARMACOL RES, V58, P271, DOI 10.1016/j.phrs.2008.09.006

MAGUIREZEISS KA, 2008, MICROGLIAL ACTIVATIO

Marks WJ, 2008, LANCET NEUROL, V7, P400, DOI 10.1016/S1474-4422(08)70065-6

MCGEER PL, 1988, NEUROLOGY, V38, P1285, DOI 10.1212/WNL.38.8.1285

Offen D, 2000, BRAIN RES, V854, P257, DOI 10.1016/S0006-8993(99)02375-6

Ouchi Y, 2005, ANN NEUROL, V57, P168, DOI 10.1002/ana.20338

Ouchi Y, 2009, PARKINSONISM RELAT D, V15, pS200, DOI 10.1016/S1353-8020(09)70814-4

Perlmann T, 2004, CELL TISSUE RES, V318, P45, DOI 10.1007/s00441-004-0974-7

Poewe W, 2007, MOVEMENT DISORD, V22, pS374, DOI 10.1002/mds.21681

Polymeropoulos MH, 1996, SCIENCE, V274, P1197, DOI 10.1126/science.274.5290.1197

Polymeropoulos MH, 2000, ANN NY ACAD SCI, V920, P28

Rezak M, 2007, DM-DIS MON, V53, P259, DOI 10.1016/j.disamonth.2007.02.003

Saijo K, 2009, CELL, V137, P47, DOI 10.1016/j.cell.2009.01.038

Satake W, 2009, NAT GENET, V41, P1303, DOI 10.1038/ng.485

Simón-Sánchez J, 2009, NAT GENET, V41, P1308, DOI 10.1038/ng.487

Singleton A, 2004, BRAIN, V127, P768, DOI 10.1093/brain/awh081

Singleton AB, 2003, SCIENCE, V302, P841, DOI 10.1126/science.1090278

Spillantini MG, 1998, P NATL ACAD SCI USA, V95, P6469, DOI 10.1073/pnas.95.11.6469

Spillantini MG, 1997, NATURE, V388, P839, DOI 10.1038/42166

Su XM, 2008, NEUROBIOL AGING, V29, P1690, DOI 10.1016/j.neurobiolaging.2007.04.006

Su XM, 2009, NEUROTOX RES, V16, P238, DOI 10.1007/s12640-009-9053-x

Thiruchelvam MJ, 2004, EUR J NEUROSCI, V19, P845, DOI 10.1111/j.0953-816X.2004.03139.x

Tolosa E, 2007, PARKINSONISM RELAT D, V13, pS2, DOI 10.1016/j.parkreldis.2007.06.007

Wang ZL, 2003, NATURE, V423, P555, DOI 10.1038/nature01645

Zetterstrom RH, 1997, SCIENCE, V276, P248, DOI 10.1126/science.276.5310.248

Zhang W, 2005, FASEB J, V19, P533, DOI 10.1096/fj.04-2751com

Zheng KN, 2003, ARCH NEUROL-CHICAGO, V60, P722, DOI 10.1001/archneur.60.5.722

NR 67

TC 27

Z9 33

U1 2

U2 6

PU SPRINGER WIEN

PI WIEN

PA SACHSENPLATZ 4-6, PO BOX 89, A-1201 WIEN, AUSTRIA

SN 0300-9564

EI 1435-1463

J9 J NEURAL TRANSM

JI J. Neural Transm.

PD AUG

PY 2010

VL 117

IS 8

SI SI

BP 1019

EP 1025

DI 10.1007/s00702-010-0431-6

PG 7

WC Clinical Neurology; Neurosciences

WE Science Citation Index Expanded (SCI-EXPANDED)

SC Neurosciences & Neurology

GA 636AX

UT WOS:000280701200011

PM 20549523

DA 2024-08-12

ER

PT J

AU Tome, CML

Tyson, T

Rey, NL

Grathwohl, S

Britschgi, M

Brundin, P

AF Tome, Carla M. Lema

Tyson, Trevor

Rey, Nolwen L.

Grathwohl, Stefan

Britschgi, Markus

Brundin, Patrik

TI Inflammation and α-Synuclein's Prion-like Behavior in Parkinson's

Disease-Is There a Link?

SO MOLECULAR NEUROBIOLOGY

LA English

DT Review

DE alpha-Synuclein; Neuroinflammation; Prion-like; Parkinson's disease;

Synucleinopathies; Dual-hit hypothesis; Proteinopathy

ID DORSAL MOTOR NUCLEUS; OBLIGATORY TRIGGER SITE; CENTRAL-NERVOUS-SYSTEM;

TUMOR-NECROSIS-FACTOR; LEWY BODY DISORDERS; INFLUENZA-VIRUS;

DOPAMINERGIC-NEURONS; RAT MODEL; DIFFERENTIAL EXPRESSION; MICROGLIAL

ACTIVATION

AB Parkinson's disease patients exhibit progressive spreading of aggregated alpha-synuclein in the nervous system. This slow process follows a specific pattern in an inflamed tissue environment. Recent research suggests that prion-like mechanisms contribute to the propagation of alpha-synuclein pathology. Little is known about factors that might affect the prion-like behavior of misfolded alpha-synuclein. In this review, we suggest that neuroinflammation plays an important role. We discuss causes of inflammation in the olfactory bulb and gastrointestinal tract and how this may promote the initial misfolding and aggregation of alpha-synuclein, which might set in motion events that lead to Parkinson's disease neuropathology. We propose that neuroinflammation promotes the prion-like behavior of alpha-synuclein and that novel anti-inflammatory therapies targeting this mechanism could slow disease progression.

C1 [Tome, Carla M. Lema; Tyson, Trevor; Rey, Nolwen L.; Brundin, Patrik] Lund Univ, Neuronal Survival Unit, Wallenberg Neurosci Ctr, S-22184 Lund, Sweden.

[Grathwohl, Stefan; Britschgi, Markus] F Hoffmann Roche Ltd, pRED, Pharma Res & Early Dev, DTA CNS, CH-4070 Basel, Switzerland.

[Brundin, Patrik] Van Andel Res Inst, Ctr Neurodegenerat Sci, Grand Rapids, MI 49503 USA.

C3 Lund University; Roche Holding; Van Andel Institute

RP Tome, CML (corresponding author), Lund Univ, Neuronal Survival Unit, Wallenberg Neurosci Ctr, BMC B11, S-22184 Lund, Sweden.

EM Carla.lema_tome@med.lu.se; patrik.brundin@vai.org

OI Rey, Nolwen L./0000-0003-1381-3492; Britschgi,

Markus/0000-0001-6151-4257

FU European Research Council; Swedish Research Council; Human Frontier

Science Program; Parkinson Foundation in Sweden; ERA-Net

NEURON-MIPROTRAN; Roche Postdoctoral Fellowship

FX This work was supported by a European Research Council Advanced Grant,

Swedish Research Council, Human Frontier Science Program, the Parkinson

Foundation in Sweden, and ERA-Net NEURON-MIPROTRAN to Patrik Brundin and

a Roche Postdoctoral Fellowship to Stefan Grathwohl. Patrik Brundin is a

member of BAGADILICO Linnaeus environment and MultiPark strategic

research area at Lund University, both of which are sponsored by the

Swedish Research Council.

CR Abbott RD, 2001, NEUROLOGY, V57, P456, DOI 10.1212/WNL.57.3.456

Ache BW, 2005, NEURON, V48, P417, DOI 10.1016/j.neuron.2005.10.022

Aguzzi A, 2003, BRIT MED BULL, V66, P141, DOI 10.1093/bmb/66.1.141

Alafuzoff I, 2009, ACTA NEUROPATHOL, V117, P635, DOI 10.1007/s00401-009-0523-2

Alvarez-Erviti L, 2011, NEUROSCI RES, V69, P337, DOI 10.1016/j.neures.2010.12.020

Anderson JP, 2006, J BIOL CHEM, V281, P29739, DOI 10.1074/jbc.M600933200

Angot E, 2010, LANCET NEUROL, V9, P1128, DOI 10.1016/S1474-4422(10)70213-1

Arif IA, 2010, TOXICOL IND HEALTH, V26, P121, DOI 10.1177/0748233710362382

Armien AG, 2010, BRAIN PATHOL, V20, P738, DOI 10.1111/j.1750-3639.2009.00354.x

Aronsson F, 2003, VIRAL IMMUNOL, V16, P415, DOI 10.1089/088282403322396208

Attems J, 2008, NEUROPATH APPL NEURO, V34, P466, DOI 10.1111/j.1365-2990.2008.00937.x

BANG FB, 1961, BACTERIOL REV, V25, P228, DOI 10.1128/MMBR.25.3.228-236.1961

Barrett JC, 2008, NAT GENET, V40, P955, DOI 10.1038/ng.175

Bassetti CL, 2011, NEURODEGENER DIS, V8, P95, DOI 10.1159/000316613

Beach TG, 2009, ACTA NEUROPATHOL, V117, P613, DOI [10.1007/s00401-010-0664-3, 10.1007/s00401-009-0538-8]

Beach TG, 2009, ACTA NEUROPATHOL, V117, P169, DOI 10.1007/s00401-008-0450-7

Benner EJ, 2008, PLOS ONE, V3, DOI 10.1371/journal.pone.0001376

Beraud D, 2011, FRONT NEUROSCI-SWITZ, V5, DOI 10.3389/fnins.2011.00080

Bloch A, 2006, NEUROPATH APPL NEURO, V32, P284, DOI 10.1111/j.1365-2990.2006.00727.x

BlumDegen D, 1995, NEUROSCI LETT, V202, P17, DOI 10.1016/0304-3940(95)12192-7

BOKA G, 1994, NEUROSCI LETT, V172, P151, DOI 10.1016/0304-3940(94)90684-X

Bower JH, 2006, NEUROLOGY, V67, P494, DOI 10.1212/01.wnl.0000227906.99570.cc

Braak H, 2006, NEUROSCI LETT, V396, P67, DOI 10.1016/j.neulet.2005.11.012

Braak H, 2003, J NEURAL TRANSM, V110, P517, DOI 10.1007/s00702-002-0808-2

Braak H, 2003, NEUROBIOL AGING, V24, P197, DOI 10.1016/S0197-4580(02)00065-9

Braak H., 2002, Journal of neurology, V249, pIII/1, DOI [DOI 10.1007/S00415-002-1301-4, 10.1007/s00415-002-1301-4]

Brochard V, 2009, J CLIN INVEST, V119, P182, DOI 10.1172/JCI36470

Brooks DJ, 2010, MOVEMENT DISORD, V25, pS83, DOI 10.1002/mds.22720

Brundin P, 2008, NAT REV NEUROSCI, V9, P741, DOI 10.1038/nrn2477

CAGGIANO AO, 1993, NEUROSCIENCE, V52, P717, DOI 10.1016/0306-4522(93)90420-K

Calderón-Garcidueñas L, 2003, TOXICOL PATHOL, V31, P524, DOI 10.1080/01926230390226645

CHEN XH, 1995, GENOMICS, V26, P425, DOI 10.1016/0888-7543(95)80237-G

Cicchetti F, 2009, TRENDS PHARMACOL SCI, V30, P475, DOI 10.1016/j.tips.2009.06.005

CREWS L, 1994, PERSPECT DEV NEUROBI, V2, P151

Curtis MA, 2011, EUR J NEUROSCI, V33, P1170, DOI 10.1111/j.1460-9568.2011.07616.x

da Silveira SA, 2009, HUM MOL GENET, V18, P872, DOI 10.1093/hmg/ddn417

DAMIER P, 1993, NEUROSCIENCE, V52, P1, DOI 10.1016/0306-4522(93)90175-F

Del Tredici K, 2002, J NEUROPATH EXP NEUR, V61, P413, DOI 10.1093/jnen/61.5.413

Del Tredici K, 2010, ACTA NEUROPATHOL, V119, P703, DOI 10.1007/s00401-010-0665-2

Desplats P, 2009, P NATL ACAD SCI USA, V106, P13010, DOI 10.1073/pnas.0903691106

Detje CN, 2009, J IMMUNOL, V182, P2297, DOI 10.4049/jimmunol.0800596

Donmez G, 2012, J NEUROSCI, V32, P124, DOI 10.1523/JNEUROSCI.3442-11.2012

Doty R.L., 2003, HDB OLFACTION GUSTAT

Doty RL, 2007, PARKINSONISM RELAT D, V13, pS225, DOI 10.1016/S1353-8020(08)70006-3

Doyle KM, 2011, J CELL MOL MED, V15, P2025, DOI 10.1111/j.1582-4934.2011.01374.x

Duda JE, 2000, AM J PATHOL, V157, P1439, DOI 10.1016/S0002-9440(10)64781-5

Dunning CJR, 2012, PROG NEUROBIOL, V97, P205, DOI 10.1016/j.pneurobio.2011.11.003

DVORSKA I, 1992, Endocrine Regulations, V26, P77

EDWARDS LL, 1992, NEUROLOGY, V42, P726, DOI 10.1212/WNL.42.4.726

El-Habashi N, 2010, J NEUROVIROL, V16, P242, DOI 10.3109/13550284.2010.489596

Elder A, 2006, ENVIRON HEALTH PERSP, V114, P1172, DOI 10.1289/ehp.9030

Ermisch A., 1992, PROG BRAIN RES, V91, P155

Fahn S, 2003, ANN NY ACAD SCI, V991, P1, DOI 10.1111/j.1749-6632.2003.tb07458.x

Forsyth CB, 2011, PLOS ONE, V6, DOI 10.1371/journal.pone.0028032

Fujiwara H, 2002, NAT CELL BIOL, V4, P160, DOI 10.1038/ncb748

Gallí M, 2011, BIOCHEM PHARMACOL, V81, P569, DOI 10.1016/j.bcp.2010.12.010

Gao HM, 2008, J NEUROSCI, V28, P7687, DOI 10.1523/JNEUROSCI.0143-07.2008

Gao HM, 2011, ENVIRON HEALTH PERSP, V119, P807, DOI 10.1289/ehp.1003013

Garcia GJM, 2009, INHAL TOXICOL, V21, P1165, DOI 10.3109/08958370902882713

Gardet A, 2010, J IMMUNOL, V185, P5577, DOI 10.4049/jimmunol.1000548

Genter MB, 2009, ANN NY ACAD SCI, V1170, P623, DOI 10.1111/j.1749-6632.2009.03877.x

Gerhard A, 2006, NEUROBIOL DIS, V21, P404, DOI 10.1016/j.nbd.2005.08.002

Glass CK, 2010, CELL, V140, P918, DOI 10.1016/j.cell.2010.02.016

Gousset K, 2009, NAT CELL BIOL, V11, P328, DOI 10.1038/ncb1841

Cersósimo MG, 2011, MOVEMENT DISORD, V26, P188, DOI 10.1002/mds.23344

Greene JG, 2011, J PARKINSON DIS, V1, P137, DOI 10.3233/JPD-2011-11033

Gundersen V, 2010, ACTA NEUROL SCAND, V122, P82, DOI 10.1111/j.1600-0404.2010.01382.x

Hansen C, 2011, J CLIN INVEST, V121, P715, DOI 10.1172/JCI43366

Hardy J, 2009, CURR OPIN GENET DEV, V19, P254, DOI 10.1016/j.gde.2009.03.008

Hawkes CH, 2007, NEUROPATH APPL NEURO, V33, P599, DOI 10.1111/j.1365-2990.2007.00874.x

Hawkes CH, 1999, QJM-MON J ASSOC PHYS, V92, P473, DOI 10.1093/qjmed/92.8.473

Hawkes CH, 2010, PARKINSONISM RELAT D, V16, P79, DOI 10.1016/j.parkreldis.2009.08.007

Hawkes CH, 2009, ANN NY ACAD SCI, V1170, P615, DOI 10.1111/j.1749-6632.2009.04365.x

HIRSCH EC, 1991, J NEUROCHEM, V56, P446, DOI 10.1111/j.1471-4159.1991.tb08170.x

Hirsch EC, 2009, LANCET NEUROL, V8, P382, DOI 10.1016/S1474-4422(09)70062-6

Hodgson NR, 2012, BRAIN BEHAV IMMUN, V26, P83, DOI 10.1016/j.bbi.2011.07.238

Imamura K, 2003, ACTA NEUROPATHOL, V106, P518, DOI 10.1007/s00401-003-0766-2

Ishikawa A, 2005, ANN NEUROL, V57, P429, DOI 10.1002/ana.20393

Islam Z, 2007, TOXICOL SCI, V98, P526, DOI 10.1093/toxsci/kfm102

Jang HM, 2009, P NATL ACAD SCI USA, V106, P14063, DOI 10.1073/pnas.0900096106

Jellinger KA, 2010, ANN NEUROL, V67, P550, DOI 10.1002/ana.21638

Kahle PJ, 2009, FREE RADICAL BIO MED, V47, P1354, DOI 10.1016/j.freeradbiomed.2009.08.003

Kalaitzakis ME, 2008, NEUROPATH APPL NEURO, V34, P284, DOI 10.1111/j.1365-2990.2007.00923.x

Kalinke U, 2011, VIRULENCE, V2, P367, DOI 10.4161/viru.2.4.16138

Khandelwal PJ, 2012, MOL CELL NEUROSCI, V49, P44, DOI 10.1016/j.mcn.2011.09.002

Kim CY, 2008, J NEUROCHEM, V107, P303, DOI 10.1111/j.1471-4159.2008.05612.x

Kim YS, 2006, EXP MOL MED, V38, P333, DOI 10.1038/emm.2006.40

Klegeris A, 2008, NEUROBIOL AGING, V29, P739, DOI 10.1016/j.neurobiolaging.2006.11.013

Koob AO, 2010, NEUROSCI LETT, V469, P11, DOI 10.1016/j.neulet.2009.11.034

Kordower JH, 2008, NAT MED, V14, P504, DOI 10.1038/nm1747

Kordower JH, 2011, NEUROBIOL DIS, V43, P552, DOI 10.1016/j.nbd.2011.05.001

Kordower JH, 2008, MOVEMENT DISORD, V23, P2303, DOI 10.1002/mds.22369

Kovács T, 2004, AGEING RES REV, V3, P215, DOI 10.1016/j.arr.2003.10.003

Krüger R, 1998, NAT GENET, V18, P106, DOI 10.1038/ng0298-106

Kuo YM, 2010, HUM MOL GENET, V19, P1633, DOI 10.1093/hmg/ddq038

Kurowska Z, 2011, J PARKINSON DIS, V1, P83, DOI 10.3233/JPD-2011-11004

LAWSON LJ, 1990, NEUROSCIENCE, V39, P151, DOI 10.1016/0306-4522(90)90229-W

Le WD, 2003, NAT GENET, V33, P85, DOI 10.1038/ng1066

Lebouvier T, 2008, GUT, V57, P1741, DOI 10.1136/gut.2008.162503

Lebouvier T, 2010, PLOS ONE, V5, DOI 10.1371/journal.pone.0012728

Lee EJ, 2010, J IMMUNOL, V185, P615, DOI 10.4049/jimmunol.0903480

Lee HJ, 2008, INT J BIOCHEM CELL B, V40, P1835, DOI 10.1016/j.biocel.2008.01.017

Lee HJ, 2008, BIOCHEM BIOPH RES CO, V372, P423, DOI 10.1016/j.bbrc.2008.05.045

Lee HJ, 2010, J BIOL CHEM, V285, P9262, DOI 10.1074/jbc.M109.081125

Lee KW, 2011, J NEUROSCI, V31, P6963, DOI 10.1523/JNEUROSCI.6513-10.2011

Lee SJ, 2010, NAT REV NEUROL, V6, P702, DOI 10.1038/nrneurol.2010.145

Lemer A, 2008, MOVEMENT DISORD, V23, P1076, DOI 10.1002/mds.22066

Leyva-Grado VH, 2009, J NEUROIMMUNOL, V211, P73, DOI 10.1016/j.jneuroim.2009.03.016

Li JY, 2008, NAT MED, V14, P501, DOI 10.1038/nm1746

Lin X, 2009, NEURON, V64, P807, DOI 10.1016/j.neuron.2009.11.006

Linazasoro G, 2007, EUR J NEUROL, V14, P721, DOI 10.1111/j.1468-1331.2007.01853.x

Loseva E, 2009, BRAIN RES REV, V59, P374, DOI 10.1016/j.brainresrev.2008.10.004

Lucin KM, 2009, NEURON, V64, P110, DOI 10.1016/j.neuron.2009.08.039

Luk KC, 2009, P NATL ACAD SCI USA, V106, P20051, DOI 10.1073/pnas.0908005106

Majde JA, 2007, J NEUROVIROL, V13, P399, DOI 10.1080/13550280701427069

Majde JA, 2010, MED HYPOTHESES, V75, P204, DOI 10.1016/j.mehy.2010.02.023

MAROTEAUX L, 1988, J NEUROSCI, V8, P2804

Martyn CN, 1997, BRIT MED BULL, V53, P24

Mata IF, 2010, ARCH NEUROL-CHICAGO, V67, P1350, DOI 10.1001/archneurol.2010.279

MATTOCK C, 1988, J NEUROL NEUROSUR PS, V51, P753, DOI 10.1136/jnnp.51.6.753

McGeer PL, 2008, MOVEMENT DISORD, V23, P474, DOI 10.1002/mds.21751

MCGEER PL, 1988, ACTA NEUROPATHOL, V76, P550, DOI 10.1007/BF00689592

MCGEER PL, 1988, NEUROLOGY, V38, P1285, DOI 10.1212/WNL.38.8.1285

Ming GL, 2011, NEURON, V70, P687, DOI 10.1016/j.neuron.2011.05.001

Minguez-Castellanos A, 2007, NEUROLOGY, V68, P2012, DOI 10.1212/01.wnl.0000264429.59379.d9

Miwa H, 2006, NEUROSCI LETT, V401, P146, DOI 10.1016/j.neulet.2006.03.003

Mori K, 2005, BRAIN RES, V1039, P116, DOI 10.1016/j.brainres.2005.01.078

Morley JF, 2010, BIOMARK MED, V4, P661, DOI [10.2217/bmm.10.95, 10.2217/BMM.10.95]

Mougenot AL, 2012, NEUROBIOL AGING, V33, P2225, DOI 10.1016/j.neurobiolaging.2011.06.022

Myöhänen TT, 2012, BRIT J PHARMACOL, V166, P1097, DOI 10.1111/j.1476-5381.2012.01846.x

Nonaka N, 2008, J PHARMACOL EXP THER, V325, P513, DOI 10.1124/jpet.107.132381

Oberdörster G, 2005, ENVIRON HEALTH PERSP, V113, P823, DOI 10.1289/ehp.7339

Oberdörster G, 2004, INHAL TOXICOL, V16, P437, DOI 10.1080/08958370490439597

Ota A, 2008, ANN NY ACAD SCI, V1148, P127, DOI 10.1196/annals.1410.071

Ouchi Y, 2009, PARKINSONISM RELAT D, V15, pS200, DOI 10.1016/S1353-8020(09)70814-4

Oueslati A, 2012, J NEUROSCI, V32, P1536, DOI 10.1523/JNEUROSCI.3784-11.2012

Paisán-Ruíz C, 2004, NEURON, V44, P595, DOI 10.1016/j.neuron.2004.10.023

Paleologou KE, 2010, J NEUROSCI, V30, P3184, DOI 10.1523/JNEUROSCI.5922-09.2010

Pan-Montojo F, 2010, PLOS ONE, V5, DOI 10.1371/journal.pone.0008762

Parihar MS, 2009, INT J BIOCHEM CELL B, V41, P2015, DOI 10.1016/j.biocel.2009.05.008

Parkkinen L, 2008, ACTA NEUROPATHOL, V115, P399, DOI 10.1007/s00401-008-0346-6

Polymeropoulos MH, 1997, SCIENCE, V276, P2045, DOI 10.1126/science.276.5321.2045

Powers KM, 2009, PARKINSONISM RELAT D, V15, P47, DOI 10.1016/j.parkreldis.2008.03.002

Prediger RDS, 2012, NEUROTOX RES, V21, P90, DOI 10.1007/s12640-011-9281-8

Probst A, 2008, EUR J NEUROL, V15, P1, DOI 10.1111/j.1468-1331.2008.02057.x

Reynolds AD, 2008, J NEUROIMMUNE PHARM, V3, P59, DOI 10.1007/s11481-008-9100-z

Reynolds AD, 2008, J NEUROCHEM, V104, P1504, DOI 10.1111/j.1471-4159.2007.05087.x

Rhodin J., 1999, Journal of Submicroscopic Cytology and Pathology, V31, P305

Rohn TT, 2011, PLOS ONE, V6, DOI 10.1371/journal.pone.0020495

Saijo K, 2009, CELL, V137, P47, DOI 10.1016/j.cell.2009.01.038

Sato H, 2011, J NEUROSCI, V31, P16884, DOI 10.1523/JNEUROSCI.3967-11.2011

Schapira AHV, 2011, PARKINSONS DIS-US, V2011, DOI 10.4061/2011/159160

Schapira AHV, 2010, NAT REV NEUROL, V6, P309, DOI 10.1038/nrneurol.2010.52

SHANKAR V, 1992, J VIROL, V66, P992, DOI 10.1128/JVI.66.2.992-998.1992

Shannon KM, 2012, MOVEMENT DISORD, V27, P709, DOI 10.1002/mds.23838

Shi M, 2011, LANCET NEUROL, V10, P681, DOI 10.1016/S1474-4422(11)70130-2

Simkó M, 2010, PART FIBRE TOXICOL, V7, DOI 10.1186/1743-8977-7-42

Singleton AB, 2003, SCIENCE, V302, P841, DOI 10.1126/science.1090278

Snider BJ, 2005, ARCH NEUROL-CHICAGO, V62, P1821, DOI 10.1001/archneur.62.12.1821

Spillantini MG, 1998, P NATL ACAD SCI USA, V95, P6469, DOI 10.1073/pnas.95.11.6469

Spillantini MG, 1997, NATURE, V388, P839, DOI 10.1038/42166

Spillantini MG, 1998, NEUROSCI LETT, V251, P205, DOI 10.1016/s0304-3940(98)00504-7

Stefanova N, 2011, AM J PATHOL, V179, P954, DOI 10.1016/j.ajpath.2011.04.013

Steiner JA, 2011, CELL DEATH DIFFER, V18, P1425, DOI 10.1038/cdd.2011.53

Steinman L, 2008, J CLIN INVEST, V118, P3557, DOI 10.1172/JCI36532

Sultan B, 2011, LARYNGOSCOPE, V121, P2481, DOI 10.1002/lary.22190

Tansey MG, 2007, EXP NEUROL, V208, P1, DOI 10.1016/j.expneurol.2007.07.004

Tin-Tin-Win-Shwe, 2008, TOXICOL APPL PHARM, V226, P192, DOI 10.1016/j.taap.2007.09.009

Tremblay ME, 2011, J NEUROSCI, V31, P16064, DOI 10.1523/JNEUROSCI.4158-11.2011

Turner JH, 2010, AM J RHINOL ALLERGY, V24, P336, DOI 10.2500/ajra.2010.24.3498

UEDA K, 1993, P NATL ACAD SCI USA, V90, P11282, DOI 10.1073/pnas.90.23.11282

Ueki A, 2004, J NEUROL, V251, P18, DOI 10.1007/s00415-004-1706-3

Vawter MP, 1996, EXP NEUROL, V142, P313, DOI 10.1006/exnr.1996.0200

Venda LL, 2010, TRENDS NEUROSCI, V33, P559, DOI 10.1016/j.tins.2010.09.004

Volpicelli-Daley LA, 2011, NEURON, V72, P57, DOI 10.1016/j.neuron.2011.08.033

Vroon A, 2007, EXP GERONTOL, V42, P762, DOI 10.1016/j.exger.2007.04.010

Wakabayashi K, 1999, J NEUROL SCI, V165, P188, DOI 10.1016/S0022-510X(99)00101-X

Wakabayashi K, 1999, No To Shinkei, V51, P433

Wang T, 2004, NAT MED, V10, P1366, DOI 10.1038/nm1140

Wang Y, 2011, AM J PATHOL, V178, P1509, DOI 10.1016/j.ajpath.2011.01.006

Wang Y, 2011, TOXICOL LETT, V205, P26, DOI 10.1016/j.toxlet.2011.05.001

Watelet JB, 2009, B-ENT, P21

Weber B, 2009, SEMIN IMMUNOPATHOL, V31, P171, DOI 10.1007/s00281-009-0156-5

Winkler F, 2001, J INFECT DIS, V183, P1749, DOI 10.1086/320730

Wu CH, 1997, NEUROSCI RES, V27, P133, DOI 10.1016/S0168-0102(96)01140-6

Wyss-Coray T, 2006, NAT MED, V12, P1005, DOI 10.1038/nml1484

Yamada K, 2007, J DRUG TARGET, V15, P59, DOI 10.1080/10611860601029496

Yamada T, 1996, Parkinsonism Relat Disord, V2, P113, DOI 10.1016/1353-8020(96)00006-5

Zaccai J, 2008, NEUROLOGY, V70, P1042, DOI 10.1212/01.wnl.0000306697.48738.b6

Zarranz JJ, 2004, ANN NEUROL, V55, P164, DOI 10.1002/ana.10795

Zetterstrom RH, 1997, SCIENCE, V276, P248, DOI 10.1126/science.276.5310.248

Zhang J, 2008, AM J CLIN PATHOL, V129, P526, DOI 10.1309/W01Y0B808EMEH12L

Zhang W, 2005, FASEB J, V19, P533, DOI 10.1096/fj.04-2751com

Zimprich A, 2004, NEURON, V44, P601, DOI 10.1016/j.neuron.2004.11.005

NR 194

TC 164

Z9 173

U1 2

U2 26

PU SPRINGER

PI NEW YORK

PA ONE NEW YORK PLAZA, SUITE 4600, NEW YORK, NY, UNITED STATES

SN 0893-7648

EI 1559-1182

J9 MOL NEUROBIOL

JI Mol. Neurobiol.

PD APR

PY 2013

VL 47

IS 2

BP 561

EP 574

DI 10.1007/s12035-012-8267-8

PG 14

WC Neurosciences

WE Science Citation Index Expanded (SCI-EXPANDED)

SC Neurosciences & Neurology

GA 106DC

UT WOS:000316120900011

PM 22544647

OA hybrid, Green Published

DA 2024-08-12

ER

PT J

AU Bassani, TB

Vital, MABF

Rauh, LK

AF Bassani, Taysa Bervian

Vital, Maria A. B. F.

Rauh, Laryssa K.

TI Neuroinflammation in the pathophysiology of Parkinson's disease and

therapeutic evidence of anti-inflammatory drugs

SO ARQUIVOS DE NEURO-PSIQUIATRIA

LA English

DT Review

DE Parkinson's disease; neuroinflammation; anti-inflammatory

ID MOUSE MODEL; SUBSTANTIA-NIGRA; NSAID USE; RISK; MICROGLIA;

NEURODEGENERATION; DEXAMETHASONE; INHIBITION; ACTIVATION; MECHANISMS

AB Parkinson's disease (PD) is the second most common neurodegenerative disease affecting approximately 1.6% of the population over 60 years old. The cardinal motor symptoms are the result of progressive degeneration of substantia nigra pars compacta dopaminergic neurons which are involved in the fine motor control. Currently, there is no cure for this pathology and the cause of the neurodegeneration remains unknown. Several studies suggest the involvement of neuroinflammation in the pathophysiology of PD as well as a protective effect of anti-inflammatory drugs both in animal models and epidemiological studies, although there are controversial reports. In this review, we address evidences of involvement of inflammatory process and possible therapeutic usefulness of anti-inflammatory drugs in PD.

C1 [Bassani, Taysa Bervian; Rauh, Laryssa K.] Pontificia Univ Catolica Parana, Curitiba, PR, Brazil.

[Bassani, Taysa Bervian; Vital, Maria A. B. F.] Univ Fed Parana, Dept Farmacol, BR-80060000 Curitiba, Parana, Brazil.

C3 Pontificia Universidade Catolica do Parana; Universidade Federal do

Parana

RP Bassani, TB (corresponding author), UFPR, Dept Farmacol, Ctr Politecn, Rua Cel Francisco H Santos 100,Caixa Postal 19031, BR-81531980 Curitiba, Parana, Brazil.

EM taysa_bassani@yahoo.com.br

RI Bassani, Taysa B/I-5648-2017

OI Bassani, Taysa B/0000-0002-0771-7552

FU Capes; CNPq

FX Capes, CNPq.

CR Asanuma M, 2007, EXP NEUROL, V206, P172, DOI 10.1016/j.expneurol.2007.05.006

Barbiero JK, 2011, BEHAV BRAIN RES, V216, P186, DOI 10.1016/j.bbr.2010.07.033

Barbosa MT, 2006, MOVEMENT DISORD, V21, P800, DOI 10.1002/mds.20806

Becker C, 2011, EUR J NEUROL, V18, P1336, DOI 10.1111/j.1468-1331.2011.03399.x

Bornebroek M, 2007, NEUROEPIDEMIOLOGY, V28, P193, DOI 10.1159/000108110

Bower JH, 2006, NEUROLOGY, V67, P494, DOI 10.1212/01.wnl.0000227906.99570.cc

Brochard V, 2009, J CLIN INVEST, V119, P182, DOI 10.1172/JCI36470

Castaño A, 2002, J NEUROCHEM, V81, P150, DOI 10.1046/j.1471-4159.2002.00799.x

Chen HL, 2005, ANN NEUROL, V58, P963, DOI 10.1002/ana.20682

Chen HL, 2003, ARCH NEUROL-CHICAGO, V60, P1059, DOI 10.1001/archneur.60.8.1059

Collins LM, 2012, NEUROPHARMACOLOGY, V62, P2154, DOI 10.1016/j.neuropharm.2012.01.028

Dauer W, 2003, NEURON, V39, P889, DOI 10.1016/S0896-6273(03)00568-3

Lima MDS, 2006, BRAIN RES, V1101, P117, DOI 10.1016/j.brainres.2006.05.016

Driver JA, 2011, BMJ-BRIT MED J, V342, DOI 10.1136/bmj.d198

Esposito E, 2007, EXP NEUROL, V205, P295, DOI 10.1016/j.expneurol.2007.02.008

Etminan M, 2008, J CLIN NEUROSCI, V15, P576, DOI 10.1016/j.jocn.2007.02.095

Etminan M, 2006, CURR DRUG SAF, V1, P223, DOI 10.2174/157488606777934404

Gagne JJ, 2010, NEUROLOGY, V74, P995, DOI 10.1212/WNL.0b013e3181d5a4a3

Gao XA, 2011, NEUROLOGY, V76, P863, DOI 10.1212/WNL.0b013e31820f2d79

Ghosh A, 2007, P NATL ACAD SCI USA, V104, P18754, DOI 10.1073/pnas.0704908104

Hald A, 2005, EXP NEUROL, V193, P279, DOI 10.1016/j.expneurol.2005.01.013

Hernán MA, 2006, NEUROLOGY, V66, P1097, DOI 10.1212/01.wnl.0000204446.82823.28

Hirsch EC, 2005, PARKINSONISM RELAT D, V11, pS9, DOI 10.1016/j.parkreldis.2004.10.013

Hirsch EC, 2013, MOVEMENT DISORD, V28, P24, DOI 10.1002/mds.25032

Hirsch Etienne C, 2012, Parkinsonism Relat Disord, V18 Suppl 1, pS210, DOI 10.1016/S1353-8020(11)70065-7

Hirsch EC, 2009, LANCET NEUROL, V8, P382, DOI 10.1016/S1474-4422(09)70062-6

Kurkowska-Jastrzebska I, 2004, INT IMMUNOPHARMACOL, V4, P1307, DOI 10.1016/j.intimp.2004.05.006

Long-Smith CM, 2009, PROG NEUROBIOL, V89, P277, DOI 10.1016/j.pneurobio.2009.08.001

Manthripragada AD, 2011, NEUROEPIDEMIOLOGY, V36, P155, DOI 10.1159/000325653

MCGEER PL, 1988, NEUROLOGY, V38, P1285, DOI 10.1212/WNL.38.8.1285

Mondal S, 2012, J NEUROIMMUNE PHARM, V7, P544, DOI 10.1007/s11481-012-9377-9

Munhoz RP, 2010, CLIN NEUROL NEUROSUR, V112, P431, DOI 10.1016/j.clineuro.2010.03.003

Politis M, 2012, FRONT PHARMACOL, V3, DOI 10.3389/fphar.2012.00096

Godoy MCP, 2008, BRAIN, V131, P1880, DOI 10.1093/brain/awn101

Rees K, 2011, COCHRANE DB SYST REV, DOI 10.1002/14651858.CD008454.pub2

Reichmann H, 2009, PARKINSONISM RELAT D, V15, pS87, DOI 10.1016/S1353-8020(09)70789-8

Reksidler AB, 2007, EUR J PHARMACOL, V560, P163, DOI 10.1016/j.ejphar.2006.12.032

Samii A, 2009, DRUG AGING, V26, P769, DOI 10.2165/11316780-000000000-00000

Sanders LH, 2013, FREE RADICAL BIO MED, V62, P111, DOI 10.1016/j.freeradbiomed.2013.01.003

Singh N, 2007, PROG NEUROBIOL, V81, P29, DOI 10.1016/j.pneurobio.2006.11.009

Swiatkiewicz M, 2013, PHARMACOL REP, V65, P1227

Tansey MG, 2007, EXP NEUROL, V208, P1, DOI 10.1016/j.expneurol.2007.07.004

Tansey MG, 2010, NEUROBIOL DIS, V37, P510, DOI 10.1016/j.nbd.2009.11.004

Ton TG, 2006, MOVEMENT DISORD, V21, P964, DOI 10.1002/mds.20856

Wahner AD, 2007, NEUROLOGY, V69, P1836, DOI 10.1212/01.wnl.0000279519.99344.ad

Wilms H, 2003, FASEB J, V17, P500, DOI 10.1096/fj.02-0314fje

Witte ME, 2010, MITOCHONDRION, V10, P411, DOI 10.1016/j.mito.2010.05.014

Zaminelli T, 2014, NEUROTOX RES, V25

Zhang F, 2010, J PHARMACOL EXP THER, V333, P822, DOI 10.1124/jpet.110.165829

NR 49

TC 66

Z9 75

U1 0

U2 13

PU ASSOC ARQUIVOS NEURO- PSIQUIATRIA

PI SAO PAULO SP

PA PR AMADEU AMARAL 47/33, 01327-010 SAO PAULO SP, BRAZIL

SN 0004-282X

EI 1678-4227

J9 ARQ NEURO-PSIQUIAT

JI Arq. Neuro-Psiquiatr.

PD JUL

PY 2015

VL 73

IS 7

BP 616

EP 623

DI 10.1590/0004-282X20150057

PG 8

WC Neurosciences; Psychiatry

WE Science Citation Index Expanded (SCI-EXPANDED)

SC Neurosciences & Neurology; Psychiatry

GA CN4WX

UT WOS:000358432300012

PM 26200058

OA Green Published, Green Submitted, gold

DA 2024-08-12

ER

PT J

AU Crea, R

Bitler, CM

Bolin, LM

Pontoniere, P

AF Crea, Roberto

Bitler, Catherine M.

Bolin, Laurel M.

Pontoniere, Paolo

TI Anti-inflammatory activity of hydroxytyrosol Inhibition of cytokine

production in a parkinson's model system of neuroinflammation

SO AGRO FOOD INDUSTRY HI-TECH

LA English

DT Article

DE Olive vegetation water; Lewy bodies; Parkinson's disease; inflammation;

Hidrox (R); hydroxytyrosol; DOPET

ID NITRIC-OXIDE; DISEASE; RESVERATROL; APOPTOSIS; DOPAMINE

AB The Mediterranean diet, consisting of high concentrations of natural polyphenols and flavonoids, is associated with a low incidence of cancer, diabetes and cardiovascular disease. Olive phenolics, in particular hydroxytyrosol, demonstrate some of the highest free-radical scavenging and anti-inflammatory activities of known naturally-occurring antioxidants. Here we demonstrate that hydrolyzed olive vegetation water (Liquid Hidrox (R) or Hidrox (R)), a proprietary formulation of olive polyphenols rich in hydroxytyrosol, inhibits the production of pro-inflammatory cytokines in an in vitro model of neuroinflammation. In contrast oleuropein, the principal phenolic in olive leaf, was significantly less effective at inhibiting cytokine production. These results suggest that the anti-inflammatory activity of hydroxytyrosol may impact neuroinflammation in Parkinson's and other neurodegenerative disease.

C1 [Crea, Roberto; Bitler, Catherine M.; Pontoniere, Paolo] CreAgri Inc, Hayward, CA 94545 USA.

[Bolin, Laurel M.] Parkinsons Inst, Sunnyvale, CA 94085 USA.

RP Crea, R (corresponding author), CreAgri Inc, 25565 Whitesell St, Hayward, CA 94545 USA.

CR BECKMAN JS, 1990, P NATL ACAD SCI USA, V87, P1620, DOI 10.1073/pnas.87.4.1620

Bertelli AAE, 2001, THERAPIE, V56, P613

Bitler CM, 2005, J NUTR, V135, P1475, DOI 10.1093/jn/135.6.1475

BOKA G, 1994, NEUROSCI LETT, V172, P151, DOI 10.1016/0304-3940(94)90684-X

Buckley CE, 2008, DIS MODEL MECH, V1, P221, DOI 10.1242/dmm.001248

Cavallaro A, 2003, PHYSIOL RES, V52, P555, DOI 10.33549/physiolres.930364

Choi DK, 2011, MOLECULES, V16, P1021, DOI 10.3390/molecules16021021

Christian MS, 2004, DRUG CHEM TOXICOL, V27, P309, DOI 10.1081/DCT-200039714

Cicerale S, 2010, INT J MOL SCI, V11, P458, DOI 10.3390/ijms11020458

Cicero AFG, 2008, J AM COLL NUTR, V27, P314, DOI 10.1080/07315724.2008.10719705

Fahn S, 2003, ANN NY ACAD SCI, V991, P1, DOI 10.1111/j.1749-6632.2003.tb07458.x

Fernandez-Espejo E, 2004, MOL NEUROBIOL, V29, P15, DOI 10.1385/MN:29:1:15

Ferreira C., 2011, Physiological changes induced by the quaternary ammonium compound benzyldimethyldodecylammonium chloride on Pseudomonas fluorescens, P1

FREI K, 1992, J NEUROIMMUNOL, V40, P189, DOI 10.1016/0165-5728(92)90133-6

Handa O, 2002, REDOX REP, V7, P324, DOI 10.1179/135100002125000901

Hashimoto T, 2004, FREE RADICAL BIO MED, V36, P555, DOI 10.1016/j.freeradbiomed.2003.12.003

Hunot S, 1996, NEUROSCIENCE, V72, P355, DOI 10.1016/0306-4522(95)00578-1

Jenner P, 1998, MOVEMENT DISORD, V13, P24

Joseph JA, 1999, J NEUROSCI, V19, P8114

Knott C, 2000, MOL CELL NEUROSCI, V16, P724, DOI 10.1006/mcne.2000.0914

Levites Y, 2001, J NEUROCHEM, V78, P1073, DOI 10.1046/j.1471-4159.2001.00490.x

Manna SK, 2000, J IMMUNOL, V164, P6509, DOI 10.4049/jimmunol.164.12.6509

McGeer PL, 2004, PARKINSONISM RELAT D, V10, pS3, DOI 10.1016/j.parkreldis.2004.01.005

MCGEER PL, 1988, NEUROLOGY, V38, P1285, DOI 10.1212/WNL.38.8.1285

Muller T, 1998, ACTA NEUROL SCAND, V98, P142

Obeso JA, 2004, NEUROLOGY, V62, pS17, DOI 10.1212/WNL.62.1_suppl_1.S17

Ruano C., 2011, EUR J CLIN NUTR, V2011

Schaffer S, 2010, PHARMACOL RES, V62, P322, DOI 10.1016/j.phrs.2010.06.004

Shults CW, 2002, ARCH NEUROL-CHICAGO, V59, P1541, DOI 10.1001/archneur.59.10.1541

Stupans I, 2002, J AGR FOOD CHEM, V50, P2464, DOI 10.1021/jf0112320

Wang MJ, 2001, J NEUROIMMUNOL, V112, P28, DOI 10.1016/S0165-5728(00)00374-X

Woo SH, 2002, INT J ONCOL, V21, P57

Xu X, 2011, ANTIMICROB AGENTS CH, V55, P1229, DOI 10.1128/AAC.01016-10

Zhu W, 2007, FASEB J, V21, P3835, DOI 10.1096/fj.07-8386com

NR 34

TC 4

Z9 4

U1 0

U2 15

PU TEKNOSCIENZE PUBL

PI MILANO

PA VIALE BRIANZA 22, 20127 MILANO, ITALY

SN 1722-6996

EI 2035-4606

J9 AGRO FOOD IND HI TEC

JI Agro Food Ind. Hi-Tech

PD MAR-APR

PY 2012

VL 23

IS 2

BP 26

EP 29

PG 4

WC Biotechnology & Applied Microbiology; Food Science & Technology

WE Science Citation Index Expanded (SCI-EXPANDED)

SC Biotechnology & Applied Microbiology; Food Science & Technology

GA 953CQ

UT WOS:000304844900006

DA 2024-08-12

ER

PT J

AU Post, Z

Manfready, RA

Keshavarzian, A

AF Post, Zoe

Manfready, Richard A.

Keshavarzian, Ali

TI Overview of the Gut-Brain Axis: From Gut to Brain and Back Again

SO SEMINARS IN NEUROLOGY

LA English

DT Article

DE gut-brain axis; microbiome-gut-brain axis; neuroendocrinology;

neuroinflammation

ID CHAIN FATTY-ACIDS; parkinson's disease; NLRP3 INFLAMMASOME; MOUSE MODEL;

ALPHA-SYNUCLEIN; INTESTINAL PERMEABILITY; SYSTEMIC INFLAMMATION;

MICROBIAL METABOLITES; SLEEP-DEPRIVATION; ANIMAL-MODEL

AB The gut-brain axis refers to a bidirectional communication pathway linking the gastrointestinal system to the central nervous system. The hardware of this multifaceted pathway takes many forms, at once structural (neurons, microglia, intestinal epithelial cell barrier), chemical (neurotransmitters, enteroendocrine hormones, bacterial metabolites), and cellular (immune signaling, inflammatory pathways). The gut-brain axis is exquisitely influenced by our environment, diet, and behaviors. Here, we will describe recent progress in understanding the gut-brain axis in neurological disease, using Parkinson's disease as a guide. We will see that each component of the gut-brain axis is heavily mediated by intestinal microbiota and learn how gut-brain communication can go awry in microbial dysbiosis.

C1 [Post, Zoe; Manfready, Richard A.; Keshavarzian, Ali] Rush Univ, Med Ctr, Dept Internal Med, Div Digest Dis & Nutr, Chicago, IL USA.

[Manfready, Richard A.; Keshavarzian, Ali] Rush Univ, Med Ctr, Rush Ctr Integrated Microbiome & Chronobiol Res, Chicago, IL USA.

[Manfready, Richard A.; Keshavarzian, Ali] Rush Univ, Med Ctr, Dept Physiol & Anat & Cell Biol, Chicago, IL USA.

[Keshavarzian, Ali] 1725 W Harrison St Suite 207, Chicago, IL 60612 USA.

C3 Rush University; Rush University; Rush University

RP Keshavarzian, A (corresponding author), 1725 W Harrison St Suite 207, Chicago, IL 60612 USA.

EM Ali_Keshavarzian@rush.edu

RI Manfready, Richard/JFS-0371-2023; Post, Zoe/KHE-1355-2024

OI Post, Zoe/0000-0002-3397-507X

CR Aaldijk E, 2022, AGEING RES REV, V75, DOI 10.1016/j.arr.2021.101556

Abdel-Gadir A, 2019, NAT MED, V25, P1164, DOI 10.1038/s41591-019-0461-z

Abdel-Haq R, 2019, J EXP MED, V216, P41, DOI 10.1084/jem.20180794

Agirman G, 2021, SCIENCE, V374, P1087, DOI 10.1126/science.abi6087

Alabdali A, 2014, J NEUROINFLAMM, V11, DOI 10.1186/1742-2094-11-4

Alboni S, 2017, MOL PSYCHIATR, V22, P635, DOI 10.1038/mp.2015.191

Alcalay RN, 2012, MOVEMENT DISORD, V27, P771, DOI 10.1002/mds.24918

Asbjornsdottir B, 2020, NUTRIENTS, V12, DOI 10.3390/nu12071982

Athauda D, 2016, PROG NEUROBIOL, V145, P98, DOI 10.1016/j.pneurobio.2016.10.001

Athauda D, 2018, NEUROPHARMACOLOGY, V136, P260, DOI 10.1016/j.neuropharm.2017.09.023

Athauda D, 2017, LANCET, V390, P1664, DOI 10.1016/S0140-6736(17)31585-4

Baloni P, 2020, CELL REP MED, V1, DOI 10.1016/j.xcrm.2020.100138

Benakis C, 2016, NAT MED, V22, P516, DOI 10.1038/nm.4068

BERGINER VM, 1984, NEW ENGL J MED, V311, P1649, DOI 10.1056/NEJM198412273112601

Bertilsson G, 2008, J NEUROSCI RES, V86, P326, DOI 10.1002/jnr.21483

Bishehsari F, 2020, CELL MOL GASTROENTER, V9, P219, DOI 10.1016/j.jcmgh.2019.10.011

Biton M, 2018, CELL, V175, P1307, DOI 10.1016/j.cell.2018.10.008

Blume J, 2011, BRAIN BEHAV IMMUN, V25, P221, DOI 10.1016/j.bbi.2010.10.008

Borghammer P, 2019, J PARKINSON DIS, V9, pS281, DOI 10.3233/JPD-191721

Bougeard C, 2021, FRONT PSYCHIATRY, V12, DOI 10.3389/fpsyt.2021.744709

Braak H, 2003, J NEURAL TRANSM, V110, P517, DOI 10.1007/s00702-002-0808-2

Campbell C, 2020, NATURE, V581, P475, DOI 10.1038/s41586-020-2193-0

Caputi V, 2018, INT J MOL SCI, V19, DOI 10.3390/ijms19061689

Caspani G, 2019, MICROB CELL, V6, P454, DOI 10.15698/mic2019.10.693

Castro-Nallar E, 2015, PEERJ, V3, DOI 10.7717/peerj.1140

Choi J, 2022, FRONT IMMUNOL, V13, DOI 10.3389/fimmu.2022.916066

Christ A, 2018, CELL, V172, P162, DOI 10.1016/j.cell.2017.12.013

Cicchetti F, 2009, TRENDS PHARMACOL SCI, V30, P475, DOI 10.1016/j.tips.2009.06.005

Clarke G, 2012, FRONT PHARMACOL, V3, DOI 10.3389/fphar.2012.00090

Codolo G, 2013, PLOS ONE, V8, DOI 10.1371/journal.pone.0055375

CROSS AJ, 1984, J NEUROCHEM, V43, P1574, DOI 10.1111/j.1471-4159.1984.tb06081.x

Cruz-Pereira JS, 2020, ANNU REV PSYCHOL, V71, P49, DOI 10.1146/annurev-psych-122216-011613

Cryan JF, 2019, PHYSIOL REV, V99, P1877, DOI 10.1152/physrev.00018.2018

Ding XB, 2021, NAT MED, V27, P411, DOI 10.1038/s41591-020-01198-1

Dodiya HB, 2020, NEUROBIOL DIS, V135, DOI 10.1016/j.nbd.2018.12.012

Dogra N, 2022, CELL MOL NEUROBIOL, V42, P315, DOI 10.1007/s10571-021-01066-7

Dutta G, 2008, FUND CLIN PHARMACOL, V22, P453, DOI 10.1111/j.1472-8206.2008.00616.x

Dyall SC, 2015, FRONT AGING NEUROSCI, V7, DOI 10.3389/fnagi.2015.00052

Elliott EI, 2015, IMMUNOL REV, V265, P35, DOI 10.1111/imr.12286

Fairbrass KM, 2021, GUT, DOI 10.1136/gutjnl-2021-325985

Fann DYW, 2014, EXP NEUROL, V257, P114, DOI 10.1016/j.expneurol.2014.04.017

Fasano A, 2015, LANCET NEUROL, V14, P625, DOI 10.1016/S1474-4422(15)00007-1

Fiorentino M, 2016, MOL AUTISM, V7, DOI 10.1186/s13229-016-0110-z

Forsyth CB, 2017, ALCOHOL CLIN EXP RES, V41, P2100, DOI 10.1111/acer.13519

Forsyth CB, 2011, PLOS ONE, V6, DOI 10.1371/journal.pone.0028032

Foster JA, 2021, FRONT NEUROL, V12, DOI 10.3389/fneur.2021.721126

Gao X., 2012, HABITUAL INTAKE DIET

Gareau MG, 2008, CURR MOL MED, V8, P274, DOI 10.2174/156652408784533760

Gilbert JA, 2018, NAT MED, V24, P392, DOI 10.1038/nm.4517

Golofast B, 2020, NEUROSCI BIOBEHAV R, V108, P712, DOI 10.1016/j.neubiorev.2019.12.011

Gordon R, 2018, SCI TRANSL MED, V10, DOI 10.1126/scitranslmed.aah4066

Gruber J, 2017, CELL, V169, P1168, DOI 10.1016/j.cell.2017.05.048

Guo M, 2020, BRAIN, V143, P1476, DOI 10.1093/brain/awaa090

Hanslik KL, 2020, FRONT NEUROL, V11, DOI 10.3389/fneur.2020.570711

Hao WZ, 2020, PSYCHIAT RES, V284, DOI 10.1016/j.psychres.2019.112691

Harmer CJ, 2017, LANCET PSYCHIAT, V4, P409, DOI 10.1016/S2215-0366(17)30015-9

Harrell CR, 2019, CELLS-BASEL, V8, DOI 10.3390/cells8121605

Hartstra AV, 2020, MOL METAB, V42, DOI 10.1016/j.molmet.2020.101076

Hatcher JM, 2008, TRENDS PHARMACOL SCI, V29, P322, DOI 10.1016/j.tips.2008.03.007

Hegazy AN, 2017, GASTROENTEROLOGY, V153, P1320, DOI 10.1053/j.gastro.2017.07.047

Heintz-Buschart A, 2018, MOVEMENT DISORD, V33, P88, DOI 10.1002/mds.27105

Heppner KM, 2015, ENDOCRINOLOGY, V156, P255, DOI 10.1210/en.2014-1675

Hölscher C, 2014, J ENDOCRINOL, V221, pT31, DOI 10.1530/JOE-13-0221

Holmqvist S, 2014, ACTA NEUROPATHOL, V128, P805, DOI 10.1007/s00401-014-1343-6

Hoyles L, 2018, MICROBIOME, V6, DOI 10.1186/s40168-018-0439-y

Hyun MK, 2019, BMC GASTROENTEROL, V19, DOI 10.1186/s12876-019-0945-9

Ito M, 2019, NATURE, V565, P246, DOI 10.1038/s41586-018-0824-5

Jackson A, 2019, FRONT NEUROL, V10, DOI 10.3389/fneur.2019.01245

Jamwal DR, 2020, GASTROENTEROLOGY, V159, P1342, DOI 10.1053/j.gastro.2020.06.049

Janeway CA, 2002, ANNU REV IMMUNOL, V20, P197, DOI 10.1146/annurev.immunol.20.083001.084359

Jena PK, 2018, FASEB J, V32, P2866, DOI 10.1096/fj.201700984RR

Kaczmarek JL, 2017, AM J CLIN NUTR, V106, P1220, DOI 10.3945/ajcn.117.156380

Kaelberer MM, 2018, SCIENCE, V361, P1219, DOI 10.1126/science.aat5236

Kelly JR, 2016, J PSYCHIATR RES, V82, P109, DOI 10.1016/j.jpsychires.2016.07.019

Kelly JR, 2015, FRONT CELL NEUROSCI, V9, DOI 10.3389/fncel.2015.00392

Kelly LP, 2014, MOVEMENT DISORD, V29, P999, DOI 10.1002/mds.25736

Keshavarzian A, 2015, MOVEMENT DISORD, V30, P1351, DOI 10.1002/mds.26307

Kessing LV, 2009, J AFFECT DISORDERS, V117, P24, DOI 10.1016/j.jad.2008.11.020

Knudsen K, 2017, MOVEMENT DISORD, V32, P94, DOI 10.1002/mds.26866

Koch CE, 2017, NEUROBIOL STRESS, V6, P57, DOI 10.1016/j.ynstr.2016.09.001

Latorre R, 2016, NEUROGASTROENT MOTIL, V28, P620, DOI 10.1111/nmo.12754

Lee CH, 2019, FRONT IMMUNOL, V10, DOI 10.3389/fimmu.2019.01696

Li GF, 2021, GUT MICROBES, V13, DOI 10.1080/19490976.2021.1968257

Li YY, 2020, NAT SCI SLEEP, V12, P895, DOI 10.2147/NSS.S271927

Liddle RA, 2018, BRAIN RES, V1693, P201, DOI 10.1016/j.brainres.2018.01.010

Lightman SL, 2020, ENDOCR REV, V41, P470, DOI 10.1210/endrev/bnaa002

Lin CH, 2019, J NEUROINFLAMM, V16, DOI 10.1186/s12974-019-1528-y

Liu RT, 2019, NEUROSCI BIOBEHAV R, V102, P13, DOI 10.1016/j.neubiorev.2019.03.023

Longo VD, 2014, CELL METAB, V19, P181, DOI 10.1016/j.cmet.2013.12.008

Louveau A, 2015, NATURE, V523, P337, DOI 10.1038/nature14432

Lui PP, 2020, IMMUNOLOGY, V161, P4, DOI 10.1111/imm.13208

Maes M, 2008, NEUROENDOCRINOL LETT, V29, P117

Makki K, 2018, CELL HOST MICROBE, V23, P705, DOI 10.1016/j.chom.2018.05.012

Manfready RA, 2022, CURR NEUROL NEUROSCI, V22, P335, DOI 10.1007/s11910-022-01196-5

Manfready RA, 2021, FRONT NEUROSCI-SWITZ, V15, DOI 10.3389/fnins.2021.660942

Manosso LM, 2021, BRAIN RES BULL, V171, P196, DOI 10.1016/j.brainresbull.2021.04.001

Maraki MI, 2019, MOVEMENT DISORD, V34, P48, DOI 10.1002/mds.27489

Margolis KG, 2021, GASTROENTEROLOGY, V160, P1486, DOI 10.1053/j.gastro.2020.10.066

Mason GM, 2021, SLEEP MED REV, V57, DOI 10.1016/j.smrv.2021.101472

Maswood N, 2004, P NATL ACAD SCI USA, V101, P18171, DOI 10.1073/pnas.0405831102

Moon M, 2009, NEUROTOX RES, V15, P332, DOI 10.1007/s12640-009-9037-x

Moravcová S, 2018, PLOS ONE, V13, DOI 10.1371/journal.pone.0199405

Moreno-Indias I, 2015, EUR RESPIR J, V45, P1055, DOI 10.1183/09031936.00184314

Morris MC, 2015, ALZHEIMERS DEMENT, V11, P1007, DOI 10.1016/j.jalz.2014.11.009

Mowat AM, 2014, NAT REV IMMUNOL, V14, P667, DOI 10.1038/nri3738

Neufeld KAM, 2019, SCI REP-UK, V9, DOI 10.1038/s41598-019-50807-8

Noelker C, 2013, SCI REP-UK, V3, DOI 10.1038/srep01393

Noristani HN, 2012, AGING CELL, V11, P810, DOI 10.1111/j.1474-9726.2012.00845.x

Nuamnaichati N, 2022, J CARDIOVASC PHARM, V79, P50, DOI 10.1097/FJC.0000000000001159

Ogawa Y, 2020, SCI REP-UK, V10, DOI 10.1038/s41598-020-76562-9

Pal G, 2021, NPJ PARKINSONS DIS, V7, DOI 10.1038/s41531-021-00254-y

Payne SC, 2019, FRONT NEUROSCI-SWITZ, V13, DOI 10.3389/fnins.2019.00418

Perez-Pardo P, 2019, GUT, V68, P829, DOI 10.1136/gutjnl-2018-316844

Petrov ME, 2019, SLEEP, V42

Pires GN, 2016, SLEEP MED, V23, P109, DOI 10.1016/j.sleep.2016.07.019

Poole DP, 2010, NEUROGASTROENT MOTIL, V22, DOI 10.1111/j.1365-2982.2010.01487.x

Reigstad CS, 2015, FASEB J, V29, P1395, DOI 10.1096/fj.14-259598

Rietdijk CD, 2017, FRONT NEUROL, V8, DOI 10.3389/fneur.2017.00037

Ruben MD, 2018, SCI TRANSL MED, V10, DOI 10.1126/scitranslmed.aat8806

Rutsch A, 2020, FRONT IMMUNOL, V11, DOI 10.3389/fimmu.2020.604179

Salminen A, 2012, CELL MOL LIFE SCI, V69, P2999, DOI 10.1007/s00018-012-0962-0

Sampson TR, 2016, CELL, V167, P1469, DOI 10.1016/j.cell.2016.11.018

Scheiermann C, 2013, NAT REV IMMUNOL, V13, P190, DOI 10.1038/nri3386

Schwartz K, 2013, CURR OPIN MICROBIOL, V16, P93, DOI 10.1016/j.mib.2012.12.001

Sen P, 2021, TRENDS MOL MED, V27, P935, DOI 10.1016/j.molmed.2021.07.004

Shannon KM, 2012, MOVEMENT DISORD, V27, P716, DOI 10.1002/mds.25020

Shannon KM, 2012, MOVEMENT DISORD, V27, P709, DOI 10.1002/mds.23838

Sheline YI, 2020, NEUROLOGY, V95, pE2658, DOI 10.1212/WNL.0000000000010725

Silva YP, 2020, FRONT ENDOCRINOL, V11, DOI 10.3389/fendo.2020.00025

Smith PM, 2013, SCIENCE, V341, P569, DOI 10.1126/science.1241165

Song LM, 2017, FRONT CELL NEUROSCI, V11, DOI 10.3389/fncel.2017.00063

Stilling RM, 2016, NEUROCHEM INT, V99, P110, DOI 10.1016/j.neuint.2016.06.011

Summa KC, 2013, PLOS ONE, V8, DOI 10.1371/journal.pone.0067102

Svensson E, 2015, ANN NEUROL, V78, P522, DOI 10.1002/ana.24448

Swanson GR, 2016, AM J PHYSIOL-GASTR L, V311, pG192, DOI 10.1152/ajpgi.00087.2016

Tanner CM, 2011, ENVIRON HEALTH PERSP, V119, P866, DOI 10.1289/ehp.1002839

Terry N, 2017, HANDB EXP PHARMACOL, V239, P319, DOI 10.1007/164_2016_103

Thompson RS, 2020, SCI REP-UK, V10, DOI 10.1038/s41598-020-60679-y

Tozsér J, 2016, MEDIAT INFLAMM, V2016, DOI 10.1155/2016/5460302

Traxinger BR, 2022, MUCOSAL IMMUNOL, V15, P398, DOI 10.1038/s41385-021-00471-x

Valles-Colomer M, 2019, NAT MICROBIOL, V4, P623, DOI 10.1038/s41564-018-0337-x

van de Wouw M, 2018, J PHYSIOL-LONDON, V596, P4923, DOI 10.1113/JP276431

Videnovic A, 2013, EXP NEUROL, V243, P45, DOI 10.1016/j.expneurol.2012.08.018

Voigt RM, 2016, INT REV NEUROBIOL, V131, P193, DOI 10.1016/bs.irn.2016.07.002

Wieckowska-Gacek A, 2021, AGEING RES REV, V70, DOI 10.1016/j.arr.2021.101397

Yano JM, 2015, CELL, V161, P264, DOI 10.1016/j.cell.2015.02.047

Yanovsky Y, 2012, PLOS ONE, V7, DOI 10.1371/journal.pone.0042512

Youm YH, 2015, NAT MED, V21, P263, DOI 10.1038/nm.3804

Youm YH, 2013, CELL METAB, V18, P519, DOI 10.1016/j.cmet.2013.09.010

Yu LL, 2020, MICROBIOL RES, V233, DOI 10.1016/j.micres.2020.126409

Yuki N, 2004, P NATL ACAD SCI USA, V101, P11404, DOI 10.1073/pnas.0402391101

Zanini MA, 2015, SCHIZOPHR RES, V169, P262, DOI 10.1016/j.schres.2015.08.023

Zhang YJ, 2021, LIFE SCI, V265, DOI 10.1016/j.lfs.2020.118736

Zheng P, 2016, MOL PSYCHIATR, V21, P786, DOI 10.1038/mp.2016.44

Zheng P, 2019, SCI ADV, V5, DOI 10.1126/sciadv.aau8317

Zhu F, 2020, MOL PSYCHIATR, V25, P2905, DOI 10.1038/s41380-019-0475-4

NR 156

TC 1

Z9 1

U1 49

U2 105

PU THIEME MEDICAL PUBL INC

PI NEW YORK

PA 333 SEVENTH AVE, NEW YORK, NY 10001 USA

SN 0271-8235

EI 1098-9021

J9 SEMIN NEUROL

JI Semin. Neurol.

PD AUG

PY 2023

VL 43

IS 04

BP 506

EP 517

DI 10.1055/s-0043-1771464

EA AUG 2023

PG 12

WC Clinical Neurology

WE Science Citation Index Expanded (SCI-EXPANDED)

SC Neurosciences & Neurology

GA R8ZP3

UT WOS:001045991800008

PM 37562457

DA 2024-08-12

ER

PT J

AU Lee, S

Suh, YJ

Lee, Y

Yang, S

Hong, DG

Thirumalai, D

Chang, SC

Chung, KW

Jung, YS

Moon, HR

Chung, HY

Lee, J

AF Lee, Seulah

Suh, Yeon Ji

Lee, Yujeong

Yang, Seonguk

Hong, Dong Geun

Thirumalai, Dinakaran

Chang, Seung-Cheol

Chung, Ki Wung

Jung, Young-Suk

Moon, Hyung Ryong

Chung, Hae Young

Lee, Jaewon

TI Anti-Inflammatory Effects of the Novel Barbiturate Derivative MHY2699 in

an MPTP-Induced Mouse Model of Parkinson's Disease

SO ANTIOXIDANTS

LA English

DT Article

DE Parkinson's disease; neuroinflammation; MHY2699; barbiturate; MPTP

ID ASTROGLIAL ACTIVATION; KAPPA-B; ANTIOXIDANTS; ERK; NEUROINFLAMMATION;

INFLAMMATION

AB Parkinson's disease (PD) is one of the most common neurodegenerative disorders, and is caused by the death of dopamine neurons and neuroinflammation in the striatum and substantia nigra. Furthermore, the inflammatory response in PD is closely related to glial cell activation. This study examined the neuroprotective effects of the barbiturate derivative, MHY2699 [5-(4-hydroxy 3,5-dimethoxybenzyl)-2 thioxodihydropyrimidine-4,6(1H,5H)-dione] in a mouse model of PD. MHY2699 ameliorated MPP?-induced astrocyte activation and ROS production in primary astrocytes and inhibited the MPP?-induced phosphorylation of MAPK and NF-kappa B. The anti-inflammatory effects of MHY2699 in protecting neurons were examined in an MPTP-induced mouse model of PD. MHY2699 inhibited MPTP-induced motor dysfunction and prevented dopaminergic neuronal death, suggesting that it attenuated neuroinflammation. Overall, MHY2699 has potential as a neuroprotective treatment for PD.

C1 [Lee, Seulah; Suh, Yeon Ji; Lee, Yujeong; Yang, Seonguk; Hong, Dong Geun; Chung, Ki Wung; Jung, Young-Suk; Chung, Hae Young; Lee, Jaewon] Pusan Natl Univ, Coll Pharm, Dept Pharm, Busan 46241, South Korea.

[Lee, Seulah; Suh, Yeon Ji; Yang, Seonguk; Hong, Dong Geun; Chung, Ki Wung; Jung, Young-Suk; Moon, Hyung Ryong; Chung, Hae Young; Lee, Jaewon] Pusan Natl Univ, Res Inst Drug Dev, Busan 46241, South Korea.

[Lee, Yujeong] Korea Brain Res Inst, Cognit Sci Res Grp, Daegu 41062, South Korea.

[Thirumalai, Dinakaran; Chang, Seung-Cheol] Pusan Natl Univ, Coll Nanosci & Nanotechnol, Dept Cognomechatron Engn, Busan 46241, South Korea.

[Moon, Hyung Ryong] Pusan Natl Univ, Coll Pharm, Dept Mfg Pharm, Busan 46241, South Korea.

C3 Pusan National University; Pusan National University; Korea Brain

Research Institute (KBRI); Pusan National University; Pusan National

University

RP Lee, J (corresponding author), Pusan Natl Univ, Coll Pharm, Dept Pharm, Busan 46241, South Korea.; Lee, J (corresponding author), Pusan Natl Univ, Res Inst Drug Dev, Busan 46241, South Korea.

EM neuron@pusan.ac.kr

RI chang, seung-cheol/K-8778-2017; Thirumalai, Dinakaran/K-5303-2013; Lee,

Jaewon/IQR-8432-2023; Lee, Jaewon/N-9064-2013

OI Thirumalai, Dinakaran/0000-0002-9005-5990; Chung, Hae

Young/0000-0002-3215-8828; Lee, Seulah/0000-0003-1019-5991; Jung,

Young-Suk/0000-0003-0299-4451; Moon, Hyung Ryong/0000-0003-1119-7112;

Lee, Jaewon/0000-0003-3203-2130; Chang, Seung-Cheol/0000-0002-2333-9424

FU National Research Foundation of Korea (NRF) [NRF-2021R1A2C1010091]

FX FundingThis work was supported by the National Research Foundation of

Korea (NRF) (Grant no. NRF-2021R1A2C1010091).

CR Arulselvan P, 2016, OXID MED CELL LONGEV, V2016, DOI 10.1155/2016/5276130

Bassani TB, 2015, ARQ NEURO-PSIQUIAT, V73, P616, DOI 10.1590/0004-282X20150057

Bauerfeld CP, 2012, J IMMUNOL, V188, P2847, DOI 10.4049/jimmunol.1102157

BORLONGAN CV, 1995, BRAIN RES, V697, P254, DOI 10.1016/0006-8993(95)00901-2

Brochard V, 2009, J CLIN INVEST, V119, P182, DOI 10.1172/JCI36470

Chen WW, 2016, MOL MED REP, V13, P3391, DOI 10.3892/mmr.2016.4948

Choi SY, 2012, J NEUROSCI RES, V90, P1842, DOI 10.1002/jnr.23061

Chung YC, 2017, EXP MOL MED, V49, DOI 10.1038/emm.2016.159

Commercialization Promotion Agency for R&D Outcomes, NEURODEGENERATIVE DI, V33

DAMIER P, 1993, NEUROSCIENCE, V52, P1, DOI 10.1016/0306-4522(93)90175-F

de Pablos RM, 2014, J NEUROINFLAMM, V11, DOI 10.1186/1742-2094-11-34

Dias V, 2013, J PARKINSON DIS, V3, P461, DOI 10.3233/JPD-130230

Filograna R, 2016, CURR NEUROPHARMACOL, V14, P260, DOI 10.2174/1570159X13666151030102718

Gelders G, 2018, J IMMUNOL RES, V2018, DOI 10.1155/2018/4784268

Geronikaki AA, 2006, COMB CHEM HIGH T SCR, V9, P425, DOI 10.2174/138620706777698481

Hague MA, 2018, INT IMMUNOPHARMACOL, V55, P312, DOI 10.1016/j.intimp.2018.01.001

Hunot S, 1997, P NATL ACAD SCI USA, V94, P7531, DOI 10.1073/pnas.94.14.7531

Kritsilis M, 2018, INT J MOL SCI, V19, DOI 10.3390/ijms19102937

Kumar A, 2018, FRONT AGING NEUROSCI, V10, DOI 10.3389/fnagi.2018.00413

Kuter K, 2018, MOL NEUROBIOL, V55, P3049, DOI 10.1007/s12035-017-0529-z

Lee E, 2014, J NEUROSCI RES, V92, P130, DOI 10.1002/jnr.23307

Lee KM, 2016, J NEUROSCI RES, V94, P865, DOI 10.1002/jnr.23764

Lee S, 2021, INT J MOL SCI, V22, DOI 10.3390/ijms22042098

Lee S, 2020, BRAIN RES, V1730, DOI 10.1016/j.brainres.2019.146642

Lee Y, 2019, ARCH PHARM RES, V42, P416, DOI 10.1007/s12272-019-01133-0

Lee Y, 2019, BRAIN RES, V1704, P47, DOI 10.1016/j.brainres.2018.09.036

Lee Y, 2017, BRAIN RES, V1663, P184, DOI 10.1016/j.brainres.2017.03.018

Lee Y, 2015, BRAIN RES, V1627, P233, DOI 10.1016/j.brainres.2015.09.029

Lucas SM, 2006, BRIT J PHARMACOL, V147, pS232, DOI 10.1038/sj.bjp.0706400

McFarthing K, 2020, J PARKINSON DIS, V10, P757, DOI 10.3233/JPD-202128

Mckee CA, 2016, FRONT IMMUNOL, V7, DOI 10.3389/fimmu.2016.00556

Moon KM, 2017, ONCOTARGET, V8, P91662, DOI 10.18632/oncotarget.21714

Muthuraju S, 2020, BIOMED RES INT, V2020, DOI 10.1155/2020/9231452

Pawate S, 2004, J NEUROSCI RES, V77, P540, DOI 10.1002/jnr.20180

SCHULZ DW, 1981, BRAIN RES, V209, P177, DOI 10.1016/0006-8993(81)91179-3

Shahzad S, 2016, MAT SCI ENG C-MATER, V66, P156, DOI 10.1016/j.msec.2016.04.056

Singh S, 2021, HELIYON, V7, DOI 10.1016/j.heliyon.2021.e06216

Sokmen BB, 2013, APPL BIOCHEM BIOTECH, V171, P2030, DOI 10.1007/s12010-013-0486-6

Son TG, 2009, J NEUROSCI RES, V87, P2890, DOI 10.1002/jnr.22122

Stephenson J, 2018, IMMUNOLOGY, V154, P204, DOI 10.1111/imm.12922

Tschoe C, 2020, J STROKE, V22, P29

Wang YH, 2020, FRONT PHARMACOL, V11, DOI 10.3389/fphar.2020.00388

Xu CJ, 2016, INT IMMUNOPHARMACOL, V38, P223, DOI 10.1016/j.intimp.2016.06.004

NR 43

TC 6

Z9 7

U1 1

U2 12

PU MDPI

PI BASEL

PA ST ALBAN-ANLAGE 66, CH-4052 BASEL, SWITZERLAND

EI 2076-3921

J9 ANTIOXIDANTS-BASEL

JI Antioxidants

PD NOV

PY 2021

VL 10

IS 11

AR 1855

DI 10.3390/antiox10111855

PG 15

WC Biochemistry & Molecular Biology; Chemistry, Medicinal; Food Science &

Technology

WE Science Citation Index Expanded (SCI-EXPANDED)

SC Biochemistry & Molecular Biology; Pharmacology & Pharmacy; Food Science

& Technology

GA XL2KY

UT WOS:000727979300001

PM 34829726

OA Green Published, gold

DA 2024-08-12

ER

PT J

AU Mayne, K

White, JA

McMurran, CE

Rivera, FJ

de la Fuente, AG

AF Mayne, Katie

White, Jessica A.

McMurran, Christopher E.

Rivera, Francisco J.

de la Fuente, Alerie G.

TI Aging and Neurodegenerative Disease: Is the Adaptive Immune System a

Friend or Foe?

SO FRONTIERS IN AGING NEUROSCIENCE

LA English

DT Review

DE aging; adaptive immune system; neurodegenerative disease; degeneration;

regeneration

ID SPINAL-CORD-INJURY; REGULATORY T-CELLS; CENTRAL-NERVOUS-SYSTEM; MYELIN

BASIC-PROTEIN; AMYOTROPHIC-LATERAL-SCLEROSIS; AGE-RELATED-CHANGES;

EXPERIMENTAL AUTOIMMUNE ENCEPHALOMYELITIS; EXPERIMENTAL ALLERGIC

ENCEPHALOMYELITIS; PLACEBO-CONTROLLED TRIAL; BLOOD-BRAIN-BARRIER

AB neurodegenerative disease of the central nervous system (CNS) are characterized by progressive neuronal death and neurological dysfunction, leading to increased disability and a loss of cognitive or motor functions. Alzheimer's disease, Parkinson's disease and amyotrophic lateral sclerosis have neurodegeneration as a primary feature. However, in other CNS diseases such as multiple sclerosis, stroke, traumatic brain injury, and spinal cord injury, neurodegeneration follows another insult, such as demyelination or ischaemia. Although there are different primary causes to these diseases, they all share a hallmark of neuroinflammation. Neuroinflammation can occur through the activation of resident immune cells such as microglia, cells of the innate and adaptive peripheral immune system, meningeal inflammation and autoantibodies directed toward components of the CNS. Despite chronic inflammation being pathogenic in these diseases, local inflammation after insult can also promote endogenous regenerative processes in the CNS, which are key to slowing disease progression. The normal aging process in the healthy brain is associated with a decline in physiological function, a steady increase in levels of neuroinflammation, brain shrinkage, and memory deficits. Likewise, aging is also a key contributor to the progression and exacerbation of neurodegenerative disease. As there are associated co-morbidities within an aging population, pinpointing the precise relationship between aging and neurodegenerative disease progression can be a challenge. The CNS has historically been considered an isolated, "immune privileged" site, however, there is mounting evidence that adaptive immune cells are present in the CNS of both healthy individuals and diseased patients. Adaptive immune cells have also been implicated in both the degeneration and regeneration of the CNS. In this review, we will discuss the key role of the adaptive immune system in CNS degeneration and regeneration, with a focus on how aging influences this crosstalk.

C1 [Mayne, Katie; White, Jessica A.; de la Fuente, Alerie G.] Queens Univ Belfast, Sch Med Dent & Biomed Sci, Wellcome Wolfson Inst Expt Med, Belfast, Antrim, North Ireland.

[McMurran, Christopher E.] Addenbrookes Hosp, Dept Med, Cambridge, United Kingdom.

[Rivera, Francisco J.] Univ Austral Chile, Fac Med, Inst Anat Histol & Pathol, Lab Stem Cells & Neuroregenerat, Valdivia, Chile.

[Rivera, Francisco J.] Univ Austral Chile, Ctr Interdisciplinary Studies Nervous Syst CISNe, Valdivia, Chile.

[Rivera, Francisco J.] Paracelsus Med Univ, Inst Mol Regenerat Med, Salzburg, Austria.

[Rivera, Francisco J.] Paracelsus Med Univ, Spinal Cord Injury & Tissue Regenerat Ctr Salzbur, Salzburg, Austria.

C3 Queens University Belfast; University of Cambridge; Cambridge University

Hospitals NHS Foundation Trust; Addenbrooke's Hospital; Universidad

Austral de Chile; Universidad Austral de Chile; Paracelsus Private

Medical University; Paracelsus Private Medical University

RP de la Fuente, AG (corresponding author), Queens Univ Belfast, Sch Med Dent & Biomed Sci, Wellcome Wolfson Inst Expt Med, Belfast, Antrim, North Ireland.

EM a.guzmandelafuente@qub.ac.uk

RI de la Fuente, Alerie Guzman/ABG-4521-2020; Rivera, Francisco

J./D-7398-2015

OI de la Fuente, Alerie Guzman/0000-0001-8366-4969; Rivera, Francisco

J./0000-0001-7895-6318; McMurran, Christopher/0000-0002-8710-0930;

Mayne, Katie/0000-0002-4555-6438

FU Department for the Economy; Agencia Nacional de Investigacion y

Desarrollo (ANID) de Chile FONDECYT Program Regular Grant [1201706];

ECTRIMS Postdoctoral Fellowship; Wellcome Institutional Strategic Fund

(ISSF) Fellowship from QUB

FX This work was supported by the Department for the Economy (to KM and

JAW), Agencia Nacional de Investigacion y Desarrollo (ANID) de Chile

FONDECYT Program Regular Grant No. 1201706 (to FJR), ECTRIMS

Postdoctoral Fellowship (to AdlF), and Wellcome Institutional Strategic

Fund (ISSF) Fellowship from QUB (to AdlF).

CR Acevedo-Arozena A, 2011, DIS MODEL MECH, V4, P686, DOI 10.1242/dmm.007237

Akhtar RS, 2018, J NEUROCHEM, V145, P489, DOI 10.1111/jnc.14330

Alcina A, 2012, PLOS ONE, V7, DOI 10.1371/journal.pone.0029819

Alizadeh A, 2019, FRONT NEUROL, V10, DOI 10.3389/fneur.2019.00282

Alonso A, 2009, EUR J NEUROL, V16, P745, DOI 10.1111/j.1468-1331.2009.02586.x

Amor S, 2010, IMMUNOLOGY, V129, P154, DOI 10.1111/j.1365-2567.2009.03225.x

Ankeny DP, 2006, J NEUROCHEM, V99, P1073, DOI 10.1111/j.1471-4159.2006.04147.x

Ankeny DP, 2009, J CLIN INVEST, V119, P2990, DOI 10.1172/JCI39780

Arlehamn CSL, 2020, NAT COMMUN, V11, DOI 10.1038/s41467-020-15626-w

Baba Y, 2005, PARKINSONISM RELAT D, V11, P493, DOI 10.1016/j.parkreldis.2005.07.005

Bachiller S, 2018, FRONT CELL NEUROSCI, V12, DOI 10.3389/fncel.2018.00488

Baek H, 2016, ONCOTARGET, V7, P69347, DOI 10.18632/oncotarget.12469

Bake S, 2009, MICROVASC RES, V78, P413, DOI 10.1016/j.mvr.2009.06.009

Banerjee R, 2008, PLOS ONE, V3, DOI 10.1371/journal.pone.0002740

Bargh SRC, 2018, IRISH J MED SCI, V187, P1065, DOI 10.1007/s11845-018-1793-2

Barnett MH, 2004, ANN NEUROL, V55, P458, DOI 10.1002/ana.20016

Baruch K, 2016, NAT MED, V22, P135, DOI 10.1038/nm.4022

Baruch K, 2015, NAT COMMUN, V6, DOI 10.1038/ncomms8967

Baruch K, 2014, SCIENCE, V346, P89, DOI 10.1126/science.1252945

Baruch K, 2013, P NATL ACAD SCI USA, V110, P2264, DOI 10.1073/pnas.1211270110

Bas J, 2001, J NEUROIMMUNOL, V113, P146, DOI 10.1016/S0165-5728(00)00422-7

Bature F, 2017, BMJ OPEN, V7, DOI 10.1136/bmjopen-2016-015746

Baxi EG, 2015, J NEUROSCI, V35, P8626, DOI 10.1523/JNEUROSCI.3817-14.2015

Becker KJ, 2005, J CEREBR BLOOD F MET, V25, P1634, DOI 10.1038/sj.jcbfm.9600160

Becker KJ, 1997, P NATL ACAD SCI USA, V94, P10873, DOI 10.1073/pnas.94.20.10873

Becker KJ, 2011, STROKE, V42, P2763, DOI 10.1161/STROKEAHA.111.619593

Beers DR, 2008, P NATL ACAD SCI USA, V105, P15558, DOI 10.1073/pnas.0807419105

Beers DR, 2017, JCI INSIGHT, V2, DOI 10.1172/jci.insight.89530

Beers DR, 2011, BRAIN, V134, P1293, DOI 10.1093/brain/awr074

Benner EJ, 2004, P NATL ACAD SCI USA, V101, P9435, DOI 10.1073/pnas.0400569101

Benner EJ, 2008, PLOS ONE, V3, DOI 10.1371/journal.pone.0001376

Bergold PJ, 2016, EXP NEUROL, V275, P367, DOI 10.1016/j.expneurol.2015.05.024

Bieber AJ, 2003, ANN NEUROL, V53, P680, DOI 10.1002/ana.10578

Blau CW, 2012, NEUROBIOL AGING, V33, DOI 10.1016/j.neurobiolaging.2011.09.035

Brochard V, 2009, J CLIN INVEST, V119, P182, DOI 10.1172/JCI36470

Brown RH, 2017, NEW ENGL J MED, V377, P1602, DOI [10.1056/NEJMra1603471, 10.1016/S0140-6736(17)31287-4, 10.1016/S0140-6736(10)61156-7, 10.1056/NEJMc1710379, 10.1038/nrdp.2017.85]

Browne TC, 2013, J IMMUNOL, V190, P2241, DOI 10.4049/jimmunol.1200947

Brynskikh A, 2008, BRAIN BEHAV IMMUN, V22, P861, DOI 10.1016/j.bbi.2007.12.008

CACABELOS R, 1994, METHOD FIND EXP CLIN, V16, P141

Campbell BCV, 2019, NAT REV DIS PRIMERS, V5, DOI 10.1038/s41572-019-0118-8

Cantuti-Castelvetri L, 2018, SCIENCE, V359, P684, DOI 10.1126/science.aan4183

Cao CH, 2009, NEUROBIOL DIS, V34, P63, DOI 10.1016/j.nbd.2008.12.015

Cao W, 2018, MOL NEURODEGENER, V13, DOI 10.1186/s13024-018-0284-2

Casili G, 2016, NEUROTHERAPEUTICS, V13, P880, DOI 10.1007/s13311-016-0446-2

Chaplin DD, 2010, J ALLERGY CLIN IMMUN, V125, pS3, DOI 10.1016/j.jaci.2009.12.980

Chen HL, 2003, ARCH NEUROL-CHICAGO, V60, P1059, DOI 10.1001/archneur.60.8.1059

Chen XQ, 2019, FRONT NEUROSCI-SWITZ, V13, DOI 10.3389/fnins.2019.00659

Chen XP, 2014, J NEUROL SCI, V347, P90, DOI 10.1016/j.jns.2014.09.025

Chen YH, 2015, MOL MED REP, V12, P6105, DOI 10.3892/mmr.2015.4136

Cheung P, 2018, CELL, V173, P1385, DOI 10.1016/j.cell.2018.03.079

Chiang T, 2011, JOVE-J VIS EXP, DOI 10.3791/2761

Chiu BC, 2013, J IMMUNOL, V191, P5793, DOI 10.4049/jimmunol.1302509

Chiu IM, 2008, P NATL ACAD SCI USA, V105, P17913, DOI 10.1073/pnas.0804610105

Ciccocioppo F, 2019, SCI REP-UK, V9, DOI 10.1038/s41598-019-45433-3

Clarkson BDS, 2014, J EXP MED, V211, P595, DOI 10.1084/jem.20131377

Coder B, 2017, ONCOTARGET, V8, P7116, DOI 10.18632/oncotarget.12572

Coder BD, 2015, J IMMUNOL, V194, P5825, DOI 10.4049/jimmunol.1500082

Coles AJ, 2008, NEW ENGL J MED, V359, P1786, DOI 10.1056/NEJMoa0802670

Confavreux C, 2006, BRAIN, V129, P595, DOI 10.1093/brain/awh714

Constantinescu CS, 2011, BRIT J PHARMACOL, V164, P1079, DOI 10.1111/j.1476-5381.2011.01302.x

Coque E, 2019, P NATL ACAD SCI USA, V116, P2312, DOI 10.1073/pnas.1815961116

Cossburn M, 2012, MULT SCLER J, V18, P45, DOI 10.1177/1352458511417479

Cox AL, 2006, J NEUROIMMUNOL, V174, P180, DOI 10.1016/j.jneuroim.2006.01.007

Da Mesquita S, 2018, NATURE, V560, P185, DOI 10.1038/s41586-018-0368-8

Dambinova SA, 2003, CLIN CHEM, V49, P1752, DOI 10.1373/49.10.1752

Dansokho C, 2016, BRAIN, V139, P1237, DOI 10.1093/brain/awv408

Deczkowska A, 2018, CELL, V173, P1073, DOI 10.1016/j.cell.2018.05.003

DeMaagd George, 2015, P T, V40, P504

Derecki NC, 2010, J EXP MED, V207, P1067, DOI 10.1084/jem.20091419

Desai MK, 2011, GLIA, V59, P627, DOI 10.1002/glia.21131

Deshpande NR, 2018, PLOS ONE, V13, DOI 10.1371/journal.pone.0198354

Dhungana H, 2013, AGING CELL, V12, P842, DOI 10.1111/acel.12106

Dickson, 2017, CSH PERSPECT BIOL, V9, pa028035, DOI DOI 10.1101/CSHPERSPECT.A028035

Djikic J, 2015, J NEUROIMMUNOL, V278, P123, DOI 10.1016/j.jneuroim.2014.12.014

Dombrowski Y, 2017, NAT NEUROSCI, V20, P674, DOI 10.1038/nn.4528

Domingues HS, 2010, PLOS ONE, V5, DOI 10.1371/journal.pone.0015531

Doyle KP, 2015, J NEUROSCI, V35, P2133, DOI 10.1523/JNEUROSCI.4098-14.2015

Dulken B, 2019, NATURE, V571, P205, DOI 10.1038/s41586-019-1362-5

Edri-Brami M, 2015, EXP NEUROL, V267, P95, DOI 10.1016/j.expneurol.2015.02.023

Edri-Brami M, 2012, PLOS ONE, V7, DOI 10.1371/journal.pone.0035772

Elkins J, 2017, LANCET NEUROL, V16, P217, DOI 10.1016/S1474-4422(16)30357-X

Ellwardt E, 2016, TRENDS IMMUNOL, V37, P154, DOI 10.1016/j.it.2015.12.008

ENGELHARDT JI, 1993, ARCH NEUROL-CHICAGO, V50, P30, DOI 10.1001/archneur.1993.00540010026013

Engstad T., 2012, Norsk Epidemiologi, V22, P121

Eriksen JL, 2005, ARCH NEUROL-CHICAGO, V62, P353, DOI 10.1001/archneur.62.3.353

Felger JC, 2010, BRAIN BEHAV IMMUN, V24, P724, DOI 10.1016/j.bbi.2009.11.002

Ferrari R, 2011, CURR ALZHEIMER RES, V8, P273, DOI 10.2174/156720511795563700

FISZER U, 1994, ACTA NEUROL SCAND, V90, P160

Fleming JC, 2006, BRAIN, V129, P3249, DOI 10.1093/brain/awl296

Franklin RJM, 2017, NAT REV NEUROSCI, V18, P753, DOI 10.1038/nrn.2017.136

Frasca D, 2018, EXP GERONTOL, V107, P55, DOI 10.1016/j.exger.2017.07.002

Frasca D, 2017, EXP GERONTOL, V87, P113, DOI 10.1016/j.exger.2016.12.001

Frasca D, 2014, EXP GERONTOL, V54, P116, DOI 10.1016/j.exger.2014.01.004

Frenkel D, 2005, J NEUROL SCI, V233, P125, DOI 10.1016/j.jns.2005.03.022

Gate D, 2020, NATURE, V577, P399, DOI 10.1038/s41586-019-1895-7

Gelderblom M, 2012, BLOOD, V120, P3793, DOI 10.1182/blood-2012-02-412726

Gendelman HE, 2017, NPJ PARKINSONS DIS, V3, DOI 10.1038/s41531-017-0013-5

Gibson KL, 2009, AGING CELL, V8, P18, DOI 10.1111/j.1474-9726.2008.00443.x

GILSON J, 1993, NEUROPATH APPL NEURO, V19, P173, DOI 10.1111/j.1365-2990.1993.tb00424.x

Giovannoni G, 2010, NEW ENGL J MED, V362, P416, DOI 10.1056/NEJMoa0902533

González H, 2013, J IMMUNOL, V190, P5048, DOI 10.4049/jimmunol.1203121

Gonzalez R, 2003, EXP NEUROL, V184, P456, DOI 10.1016/S0014-4886(03)00257-7

Gucluler G, 2017, PLOS ONE, V12, DOI 10.1371/journal.pone.0171003

Hao Y, 2011, BLOOD, V118, P1294, DOI 10.1182/blood-2011-01-330530

HARDY J, 1991, TRENDS PHARMACOL SCI, V12, P383, DOI 10.1016/0165-6147(91)90609-V

Harris NM, 2020, EXP NEUROL, V326, DOI 10.1016/j.expneurol.2020.113202

Hauben E, 2000, J NEUROSCI, V20, P6421, DOI 10.1523/JNEUROSCI.20-17-06421.2000

Hauser SL, 2017, NEW ENGL J MED, V376, P221, DOI 10.1056/NEJMoa1601277

Henkel JS, 2013, EMBO MOL MED, V5, P64, DOI 10.1002/emmm.201201544

Hjelmström P, 1998, J IMMUNOL, V161, P4480

Holmin S, 1999, NEUROREPORT, V10, P1889, DOI 10.1097/00001756-199906230-00017

Holmin S, 1998, NEUROSURGERY, V42, P291, DOI 10.1097/00006123-199802000-00047

HOLMIN S, 1995, ACTA NEUROCHIR, V132, P110, DOI 10.1007/BF01404857

Horvath I, 2017, ACS CHEM NEUROSCI, V8, P1170, DOI 10.1021/acschemneuro.7b00063

Huang DW, 1999, NEURON, V24, P639, DOI 10.1016/S0896-6273(00)81118-6

HUELL M, 1995, ACTA NEUROPATHOL, V89, P544

Hurn PD, 2007, J CEREBR BLOOD F MET, V27, P1798, DOI 10.1038/sj.jcbfm.9600482

Ishii H, 2012, CELL DEATH DIS, V3, DOI 10.1038/cddis.2012.106

Ito M, 2019, NATURE, V565, P246, DOI 10.1038/s41586-018-0824-5

Jäger A, 2009, J IMMUNOL, V183, P7169, DOI 10.4049/jimmunol.0901906

Jantaratnotai N, 2003, NEUROREPORT, V14, P1429, DOI 10.1097/00001756-200308060-00005

Javidi E, 2019, FRONT IMMUNOL, V10, DOI 10.3389/fimmu.2019.00686

Jiao SS, 2016, TRANSL PSYCHIAT, V6, DOI 10.1038/tp.2016.186

Jin M, 2020, SCI REP-UK, V10, DOI 10.1038/s41598-020-62756-8

Jin WN, 2018, STROKE, V49, P1471, DOI 10.1161/STROKEAHA.118.020203

Johnson VE, 2013, BRAIN, V136, P28, DOI 10.1093/brain/aws322

Jones TB, 2002, J NEUROSCI, V22, P2690, DOI 10.1523/JNEUROSCI.22-07-02690.2002

Kalincik T, 2014, MULT SCLER J, V20, P1511, DOI 10.1177/1352458514528762

Kamel H, 2012, ARCH NEUROL-CHICAGO, V69, P576, DOI 10.1001/archneurol.2011.3590

Kametani F, 2018, FRONT NEUROSCI-SWITZ, V12, DOI 10.3389/fnins.2018.00025

Kappos L, 2010, NEW ENGL J MED, V362, P387, DOI 10.1056/NEJMoa0909494

Kempster PA, 2010, BRAIN, V133, P1755, DOI 10.1093/brain/awq059

Keren-Shaul H, 2017, CELL, V169, P1276, DOI 10.1016/j.cell.2017.05.018

Kil K, 1999, J NEUROIMMUNOL, V98, P201, DOI 10.1016/S0165-5728(99)00057-0

Kipnis J, 2003, J NEUROTRAUM, V20, P559, DOI 10.1089/089771503767168483

Kipnis J, 2004, P NATL ACAD SCI USA, V101, P8180, DOI 10.1073/pnas.0402268101

Kivisäkk P, 2003, P NATL ACAD SCI USA, V100, P8389, DOI 10.1073/pnas.1433000100

Kleinschnitz C, 2010, BLOOD, V115, P3835, DOI 10.1182/blood-2009-10-249078

Knight A, 2016, ONCOTARGET, V7, P42873, DOI 10.18632/oncotarget.10146

Kobo H, 2016, MOL GENET METAB, V117, P179, DOI 10.1016/j.ymgme.2015.09.005

Kosloski LM, 2013, J NEUROIMMUNOL, V265, P1, DOI 10.1016/j.jneuroim.2013.10.009

Kotter MR, 2006, J NEUROSCI, V26, P328, DOI 10.1523/JNEUROSCI.2615-05.2006

Lam L, 2016, FASEB J, V30, P3461, DOI 10.1096/fj.201600259RR

Lassmann H, 2018, CSH PERSPECT MED, V8, DOI 10.1101/cshperspect.a028936

Laurent C, 2017, BRAIN, V140, P184, DOI 10.1093/brain/aww270

Lee P, 2012, EXP NEUROL, V234, P50, DOI 10.1016/j.expneurol.2011.12.016

Liesz A, 2011, BRAIN, V134, P704, DOI 10.1093/brain/awr008

Liesz A, 2009, NAT MED, V15, P192, DOI 10.1038/nm.1927

Linehan E, 2014, AGING CELL, V13, P699, DOI 10.1111/acel.12223

Linton PJ, 2004, NAT IMMUNOL, V5, P133, DOI 10.1038/ni1033

Liu Q, 2014, SCI REP-UK, V4, DOI 10.1038/srep07554

Lucchinetti C, 2000, ANN NEUROL, V47, P707, DOI 10.1002/1531-8249(200006)47:6<707::AID-ANA3>3.0.CO;2-Q

LUDOWYK PA, 1993, J NEUROIMMUNOL, V48, P121, DOI 10.1016/0165-5728(93)90184-Z

Luo DD, 2019, PROTEIN CELL, V10, P566, DOI 10.1007/s13238-018-0559-y

Manwani B, 2013, EXP NEUROL, V249, P120, DOI 10.1016/j.expneurol.2013.08.011

Manwani B, 2011, BRAIN BEHAV IMMUN, V25, P1689, DOI 10.1016/j.bbi.2011.06.015

Marchetti B, 2020, AGING CELL, V19, DOI 10.1111/acel.13101

Marsh SE, 2016, P NATL ACAD SCI USA, V113, pE1316, DOI 10.1073/pnas.1525466113

Matejuk A, 2005, J IMMUNOL, V174, P2387, DOI 10.4049/jimmunol.174.4.2387

Mckee AC, 2009, J NEUROPATH EXP NEUR, V68, P709, DOI 10.1097/NEN.0b013e3181a9d503

McQuillan K, 2010, BRAIN BEHAV IMMUN, V24, P598, DOI 10.1016/j.bbi.2010.01.003

Mehanna R, 2014, PARKINSONISM RELAT D, V20, P530, DOI 10.1016/j.parkreldis.2014.02.013

Meisel C, 2005, NAT REV NEUROSCI, V6, P775, DOI 10.1038/nrn1765

Mencl S, 2014, J NEUROIMMUNOL, V274, P125, DOI 10.1016/j.jneuroim.2014.07.010

Meredith GE, 2011, J PARKINSON DIS, V1, P19, DOI 10.3233/JPD-2011-11023

Merlini M, 2018, NEURODEGENER DIS, V18, P49, DOI 10.1159/000486200

Mondal S, 2019, J IMMUNOL, V202, P3412, DOI 10.4049/jimmunol.1800587

Morgan S, 2016, BRIT MED BULL, V119, P87, DOI 10.1093/bmb/ldw026

Moskowitz DM, 2017, SCI IMMUNOL, V2, DOI 10.1126/sciimmunol.aag0192

Mosley RL, 2012, CSH PERSPECT MED, V2, DOI 10.1101/cshperspect.a009381

Mracsko E, 2014, J NEUROSCI, V34, P16784, DOI 10.1523/JNEUROSCI.1867-14.2014

Naor S, 2009, J NEUROL, V256, P1228, DOI 10.1007/s00415-009-5097-3

Narang A, 2017, J NEUROINFLAMM, V14, DOI 10.1186/s12974-017-0894-6

Nardo G, 2018, MOL NEURODEGENER, V13, DOI 10.1186/s13024-018-0271-7

Natrajan MS, 2015, ANN CLIN TRANSL NEUR, V2, P1071, DOI 10.1002/acn3.260

Neumann B, 2019, CELL STEM CELL, V25, P473, DOI 10.1016/j.stem.2019.08.015

Neumann M, 2006, SCIENCE, V314, P130, DOI 10.1126/science.1134108

Nikolich-Zugich J, 2018, NAT IMMUNOL, V19, P10, DOI 10.1038/s41590-017-0006-x

Niwa F, 2012, GERIATR GERONTOL INT, V12, P102, DOI 10.1111/j.1447-0594.2011.00740.x

Nordengen K, 2019, J NEUROINFLAMM, V16, DOI 10.1186/s12974-019-1399-2

Oberstein TJ, 2018, FRONT IMMUNOL, V9, DOI 10.3389/fimmu.2018.01213

Orr CF, 2005, BRAIN, V128, P2665, DOI 10.1093/brain/awh625

ORTIZORTIZ L, 1976, J EXP MED, V144, P604, DOI 10.1084/jem.144.3.604

Pagano G, 2016, NEUROLOGY, V86, P1400, DOI 10.1212/WNL.0000000000002461

Paulson JB, 2008, AM J PATHOL, V173, P762, DOI 10.2353/ajpath.2008.080175

Peferoen LAN, 2016, IMMUNOLOGY, V149, P146, DOI 10.1111/imm.12644

PETTINELLI CB, 1981, J IMMUNOL, V127, P1420

Phukan J, 2012, J NEUROL NEUROSUR PS, V83, P102, DOI 10.1136/jnnp-2011-300188

Pinti M, 2016, EUR J IMMUNOL, V46, P2286, DOI 10.1002/eji.201546178

Polman CH, 2006, NEW ENGL J MED, V354, P899, DOI 10.1056/NEJMoa044397

Pool M, 2012, MOL CELL NEUROSCI, V49, P68, DOI 10.1016/j.mcn.2011.09.004

Popovich PG, 1996, J NEUROSCI RES, V45, P349, DOI 10.1002/(SICI)1097-4547(19960815)45:4<349::AID-JNR4>3.0.CO;2-9

Popovich PG, 1997, J COMP NEUROL, V377, P443, DOI 10.1002/(SICI)1096-9861(19970120)377:3<443::AID-CNE10>3.0.CO;2-S

Potas JR, 2006, J NEUROTRAUM, V23, P660, DOI 10.1089/neu.2006.23.660

Pringsheim T, 2014, MOVEMENT DISORD, V29, P1583, DOI 10.1002/mds.25945

Prüss H, 2012, ARCH NEUROL-CHICAGO, V69, P714, DOI 10.1001/archneurol.2011.3252

Prüss H, 2011, BRAIN PATHOL, V21, P652, DOI 10.1111/j.1750-3639.2011.00488.x

Pulko V, 2016, NAT IMMUNOL, V17, P966, DOI 10.1038/ni.3483

Qin HW, 2016, J NEUROSCI, V36, P5144, DOI 10.1523/JNEUROSCI.4658-15.2016

Radjavi A, 2014, BRAIN BEHAV IMMUN, V35, P58, DOI 10.1016/j.bbi.2013.08.013

Raine CS, 1999, ANN NEUROL, V46, P144, DOI 10.1002/1531-8249(199908)46:2<144::AID-ANA3>3.0.CO;2-K

Ramagopalan SV, 2008, TRENDS NEUROSCI, V31, P645, DOI 10.1016/j.tins.2008.09.001

Ratliff M, 2013, AGING CELL, V12, P303, DOI 10.1111/acel.12055

Reynolds AD, 2007, J LEUKOCYTE BIOL, V82, P1083, DOI 10.1189/jlb.0507296

Reynolds AD, 2010, J IMMUNOL, V184, P2261, DOI 10.4049/jimmunol.0901852

Reynolds AD, 2009, J IMMUNOL, V182, P4137, DOI 10.4049/jimmunol.0803982

Riley RL, 2017, CELL IMMUNOL, V321, P61, DOI 10.1016/j.cellimm.2017.04.008

Ritzel RM, 2018, ACTA NEUROPATHOL, V136, P89, DOI 10.1007/s00401-018-1859-2

Ritzel RM, 2016, J IMMUNOL, V196, P3318, DOI 10.4049/jimmunol.1502021

RODRIGUEZ M, 1993, MAYO CLIN PROC, V68, P627, DOI 10.1016/S0025-6196(12)60597-7

ROGERS J, 1988, NEUROBIOL AGING, V9, P339, DOI 10.1016/S0197-4580(88)80079-4

Rosenkranz D, 2007, J NEUROIMMUNOL, V188, P117, DOI 10.1016/j.jneuroim.2007.05.011

Rothweiler B, 1998, ARCH PHYS MED REHAB, V79, P881, DOI 10.1016/S0003-9993(98)90082-X

Rudd BD, 2011, P NATL ACAD SCI USA, V108, P13694, DOI 10.1073/pnas.1107594108

Rudehill S, 2006, ACTA NEUROCHIR, V148, P199, DOI 10.1007/s00701-005-0673-5

Russo MV, 2016, SCIENCE, V353, P783, DOI 10.1126/science.aaf6260

Sabatino JJ, 2019, NAT REV NEUROSCI, V20, P728, DOI 10.1038/s41583-019-0233-2

Saposnik G, 2008, STROKE, V39, P2310, DOI 10.1161/STROKEAHA.107.511402

Saresella M, 2013, CLIN IMMUNOL, V148, P79, DOI 10.1016/j.clim.2013.04.010

Sargento-Freitas J, 2018, FRONT NEUROL, V9, DOI 10.3389/fneur.2018.00302

Saunders JAH, 2012, J NEUROIMMUNE PHARM, V7, P927, DOI 10.1007/s11481-012-9402-z

Sawcer S, 2005, AM J HUM GENET, V77, P454

Sawcer S, 2011, NATURE, V476, P214, DOI 10.1038/nature10251

Schnell L, 1997, EUR J NEUROSCI, V9, P1000, DOI 10.1111/j.1460-9568.1997.tb01450.x

Schnell L, 1999, EUR J NEUROSCI, V11, P3648, DOI 10.1046/j.1460-9568.1999.00792.x

Schori H, 2001, P NATL ACAD SCI USA, V98, P3398, DOI 10.1073/pnas.041609498

SCHROETER M, 1994, J NEUROIMMUNOL, V55, P195, DOI 10.1016/0165-5728(94)90010-8

Schutt CR, 2018, MOL NEURODEGENER, V13, DOI 10.1186/s13024-018-0255-7

Schwab JM, 2014, EXP NEUROL, V258, P121, DOI 10.1016/j.expneurol.2014.04.023

Seo JE, 2015, J NEUROIMMUNOL, V287, P43, DOI 10.1016/j.jneuroim.2015.08.005

Shah GN, 1997, EXP GERONTOL, V32, P501, DOI 10.1016/S0531-5565(96)00158-1

Shahaduzzaman M, 2013, AGE, V35, P2071, DOI 10.1007/s11357-012-9496-5

Shalash A, 2017, FRONT NEUROL, V8, DOI 10.3389/fneur.2017.00720

Sheean RK, 2018, JAMA NEUROL, V75, P681, DOI 10.1001/jamaneurol.2018.0035

Shi N, 2007, J NEUROIMMUNOL, V182, P232, DOI 10.1016/j.jneuroim.2006.10.001

Shichita T, 2009, NAT MED, V15, P946, DOI 10.1038/nm.1999

Shin BS, 2013, J DERMATOL SCI, V70, P141, DOI 10.1016/j.jdermsci.2013.01.006

Sicotte M, 2003, MOL CELL NEUROSCI, V23, P251, DOI 10.1016/S1044-7431(03)00053-8

Sim FJ, 2002, J NEUROSCI, V22, P2451, DOI 10.1523/JNEUROSCI.22-07-02451.2002

SMITH KJ, 1979, NATURE, V280, P395, DOI 10.1038/280395a0

Smith LK, 2015, NAT MED, V21, P932, DOI 10.1038/nm.3898

Smithey MJ, 2011, EUR J IMMUNOL, V41, P1352, DOI 10.1002/eji.201041141

Smolders J, 2018, NAT COMMUN, V9, DOI 10.1038/s41467-018-07053-9

Smolders J, 2013, ACTA NEUROPATHOL, V126, P525, DOI 10.1007/s00401-013-1155-0

Sommer A, 2019, CELL STEM CELL, V24, P1006, DOI 10.1016/j.stem.2019.04.019

SRIRAM S, 1982, J IMMUNOL, V129, P1649

Sroga JM, 2003, J COMP NEUROL, V462, P223, DOI 10.1002/cne.10736

Stephenson J, 2018, IMMUNOLOGY, V154, P204, DOI 10.1111/imm.12922

Stevens CH, 2012, J NEUROIMMUNOL, V252, P95, DOI 10.1016/j.jneuroim.2012.07.015

Stichel CC, 2007, NEUROBIOL AGING, V28, P1507, DOI 10.1016/j.neurobiolaging.2006.07.022

Stojic-Vukanic Z, 2015, IMMUN AGEING, V12, DOI 10.1186/s12979-015-0044-x

Sulzer D, 2017, NATURE, V546, P656, DOI 10.1038/nature22815

Szewczyk-Krolikowski K, 2014, PARKINSONISM RELAT D, V20, P99, DOI 10.1016/j.parkreldis.2013.09.025

Tabibian-Keissar H, 2016, EUR J IMMUNOL, V46, P480, DOI 10.1002/eji.201545586

Taguchi A, 2004, J CLIN INVEST, V114, P330, DOI 10.1172/jci200420622

Tanzi RE, 2012, CSH PERSPECT MED, V2, DOI 10.1101/cshperspect.a006296

Tobin RP, 2014, ACTA NEUROPATHOL COM, V2, DOI 10.1186/s40478-014-0143-5

Togo T, 2002, J NEUROIMMUNOL, V124, P83, DOI 10.1016/S0165-5728(01)00496-9

TRAUGOTT U, 1983, SCIENCE, V219, P308, DOI 10.1126/science.6217550

TRAUGOTT U, 1988, ANN NEUROL, V24, P243, DOI 10.1002/ana.410240211

Tweedie D, 2012, J NEUROINFLAMM, V9, DOI 10.1186/1742-2094-9-106

Ucar D, 2017, J EXP MED, V214, P3123, DOI 10.1084/jem.20170416

Villeda SA, 2011, NATURE, V477, P90, DOI 10.1038/nature10357

Walsh James T, 2015, J Clin Invest, V125, P2547, DOI 10.1172/JCI82458

WANG WZ, 1992, CLIN EXP IMMUNOL, V88, P157

Weckbach S, 2012, J NEUROTRAUM, V29, P1233, DOI 10.1089/neu.2011.2169

Werner C, 2007, BRIT J ANAESTH, V99, P4, DOI 10.1093/bja/aem131

Westerlind H, 2014, BRAIN, V137, P770, DOI 10.1093/brain/awt356

Wolf SA, 2009, FASEB J, V23, P3121, DOI 10.1096/fj.08-113944

Wolf SA, 2009, J IMMUNOL, V182, P3979, DOI 10.4049/jimmunol.0801218

Wu B, 2012, EXP NEUROL, V237, P274, DOI 10.1016/j.expneurol.2012.07.016

Wyss-Coray T, 2016, NATURE, V539, P180, DOI 10.1038/nature20411

Xu YZ, 2010, BRAIN BEHAV IMMUN, V24, P138, DOI 10.1016/j.bbi.2009.09.006

ZAMVIL S, 1985, NATURE, V317, P355, DOI 10.1038/317355a0

Zhang JX, 2013, PLOS ONE, V8, DOI [10.1371/journal.pone.0076378, 10.1371/journal.pone.0058115]

Zhang Y, 2013, J NEUROSCI, V33, P12970, DOI 10.1523/JNEUROSCI.1974-13.2013

Ziv Y, 2006, NAT NEUROSCI, V9, P268, DOI 10.1038/nn1629

NR 277

TC 83

Z9 91

U1 2

U2 18

PU FRONTIERS MEDIA SA

PI LAUSANNE

PA AVENUE DU TRIBUNAL FEDERAL 34, LAUSANNE, CH-1015, SWITZERLAND

SN 1663-4365

J9 FRONT AGING NEUROSCI

JI Front. Aging Neurosci.

PD SEP 23

PY 2020

VL 12

AR 572090

DI 10.3389/fnagi.2020.572090

PG 24

WC Geriatrics & Gerontology; Neurosciences

WE Science Citation Index Expanded (SCI-EXPANDED)

SC Geriatrics & Gerontology; Neurosciences & Neurology

GA NX1GC

UT WOS:000575464600001

PM 33173502

OA Green Published, gold

DA 2024-08-12

ER

EF

FN Clarivate Analytics Web of Science

VR 1.0

PT J

AU Labandeira, CM

Fraga-Bau, A

Ron, DA

Alvarez-Rodriguez, E

Vicente-Alba, P

Lago-Garma, J

Rodriguez-Perez, AI

AF Labandeira, Carmen M.

Fraga-Bau, Arturo

Ron, David Arias

Alvarez-Rodriguez, Elena

Vicente-Alba, Pablo

Lago-Garma, Javier

Rodriguez-Perez, Ana I.

TI Parkinson's disease and diabetes mellitus: common mechanisms and

treatment repurposing

SO NEURAL REGENERATION RESEARCH

LA English

DT Review

DE antidiabetic; diabetes mellitus; dopamine; exenatide; glucagon-like

peptide-1; insulin; neurodegeneration; neuroinflammation; Parkinson's

disease; repurposing

ID GLUCAGON-LIKE PEPTIDE-1; MPTP MOUSE MODEL; NF-KAPPA-B; DIPEPTIDYL

PEPTIDASE-4 INHIBITORS; L-DOPA; DYSKINETIC MOVEMENTS; RECEPTOR AGONIST;

INSULIN ACTION; LIRAGLUTIDE; EXENDIN-4

AB In the last decade, attention has become greater to the relationship between neurodegeneration and abnormal insulin signaling in the central nervous system, as insulin in the brain is implicated in neuronal survival, plasticity, oxidative stress and neuroinflammation. Diabetes mellitus and Parkinson's disease are both aging-associated diseases that are turning into epidemics worldwide. Diabetes mellitus and insulin resistance not only increase the possibility of developing Parkinson's disease but can also determine the prognosis and progression of Parkinsonian symptoms. Today, there are no available curative or disease modifying treatments for Parkinson's disease, but the role of insulin and antidiabetic medications in neurodegeneration opens a door to treatment repurposing to fight against Parkinson's disease, both in diabetic and nondiabetic Parkinsonian patients. Furthermore, it is essential to comprehend how a frequent and treatable disease such as diabetes can influence the progression of neurodegeneration in a challenging disease such as Parkinson's disease. Here, we review the present evidence on the connection between Parkinson's disease and diabetes and the consequential implications of the existing antidiabetic molecules in the severity and development of Parkinsonism, with a particular focus on glucagon-like peptide-1 receptor agonists.

C1 [Labandeira, Carmen M.; Fraga-Bau, Arturo; Alvarez-Rodriguez, Elena; Vicente-Alba, Pablo] Hosp Alvaro Cunqueiro, Dept Clin Neurol, Univ Hosp Complex, Vigo, Spain.

[Labandeira, Carmen M.; Rodriguez-Perez, Ana I.] Univ Santiago de Compostela, Lab Cellular & Mol Neurobiol Parkinsons Dis, Santiago De Compostela, Spain.

[Labandeira, Carmen M.; Rodriguez-Perez, Ana I.] Univ Santiago de Compostela, Res Ctr Mol Med & Chron Dis CIMUS, IDIS, Santiago De Compostela, Spain.

[Ron, David Arias] Univ Hosp Complex, Dept Clin Oncol, Orense, Spain.

[Lago-Garma, Javier] Univ Hosp Complex, Hosp Meixoeiro, Dept Endocrinol, Vigo, Spain.

[Rodriguez-Perez, Ana I.] Networking Res Ctr Neurodegenerat Dis CiberNed, Madrid, Spain.

C3 Universidade de Santiago de Compostela; Universidade de Santiago de

Compostela; Complexo Hospitalario Universitario de Vigo; CIBERNED

RP Labandeira, CM (corresponding author), Hosp Alvaro Cunqueiro, Dept Clin Neurol, Univ Hosp Complex, Vigo, Spain.; Labandeira, CM (corresponding author), Univ Santiago de Compostela, Lab Cellular & Mol Neurobiol Parkinsons Dis, Santiago De Compostela, Spain.; Labandeira, CM (corresponding author), Univ Santiago de Compostela, Res Ctr Mol Med & Chron Dis CIMUS, IDIS, Santiago De Compostela, Spain.

EM carmen.labandeira@hotmail.com

RI Perez, Ana Isabel Rodriguez/AAM-1198-2021

OI Perez, Ana Isabel Rodriguez/0000-0003-1354-8799; Alvarez Rodriguez,

Elena/0000-0003-2687-7532; Labandeira Guerra, Carmen

M./0000-0002-6274-5871

FU Spanish Ministry of Health [PI17/00828]; Galician Government (XUGA)

[ED431C 2018/10, ED431G/05]; FEDER (Regional European Development Fund);

Spanish Ministry of Health (Ciberned)

FX This work was supported by the Spanish Ministry of Health (PI17/00828

and Ciberned), Galician Government (XUGA, ED431C 2018/10, ED431G/05) and

FEDER (Regional European Development Fund), all to AIRP.

CR Abdelsalam RM, 2015, J NEUROCHEM, V133, P700, DOI 10.1111/jnc.13087

Abuirmeileh A, 2012, J PHARM PHARMACOL, V64, P637, DOI 10.1111/j.2042-7158.2011.01394.x

Athauda D, 2019, JAMA NEUROL, V76, P420, DOI 10.1001/jamaneurol.2018.4304

Athauda D, 2019, EUR J NEUROSCI, V49, P410, DOI 10.1111/ejn.14096

Athauda D, 2018, J PARKINSON DIS, V8, P247, DOI 10.3233/JPD-181329

Athauda D, 2017, LANCET, V390, P1664, DOI 10.1016/S0140-6736(17)31585-4

Aviles-Olmos I, 2014, J PARKINSON DIS, V4, P337, DOI 10.3233/JPD-140364

Aviles-Olmos I, 2013, J CLIN INVEST, V123, P2730, DOI 10.1172/JCI68295

Badawi GA, 2019, NEUROTOX RES, V35, P635, DOI 10.1007/s12640-019-9998-3

Bertilsson G, 2008, J NEUROSCI RES, V86, P326, DOI 10.1002/jnr.21483

Biosa A, 2018, MOL NEUROBIOL, V55, P8754, DOI 10.1007/s12035-018-1025-9

Blázquez E, 2014, FRONT ENDOCRINOL, V5, DOI 10.3389/fendo.2014.00161

Boccia L, 2020, PEPTIDES, V132, DOI 10.1016/j.peptides.2020.170366

Brauer R, 2020, BRAIN, V143, P3067, DOI 10.1093/brain/awaa262

Cereda E, 2012, NEUROLOGY, V78, P1507, DOI 10.1212/WNL.0b013e3182553cc9

Cereda E, 2011, DIABETES CARE, V34, P2614, DOI 10.2337/dc11-1584

Chang YH, 2021, EUR J NEUROL, V28, P430, DOI 10.1111/ene.14542

Chen JK, 2017, J NEUROIMMUNOL, V305, P108, DOI 10.1016/j.jneuroim.2017.02.010

Chen SC, 2018, SCI REP-UK, V8, DOI 10.1038/s41598-018-28449-z

De Pablo-Fernandez E, 2017, ACTA NEUROL SCAND, V136, P732, DOI 10.1111/ane.12793

de Pablo-Fernandez E, 2021, NEUROPATH APPL NEURO, V47, P1080, DOI 10.1111/nan.12728

Deischinger C, 2021, J PARKINSON DIS, V11, P793, DOI 10.3233/JPD-202486

Dorsey ER, 2018, LANCET NEUROL, V17, P939, DOI 10.1016/S1474-4422(18)30295-3

Elbassuoni EA, 2019, NEUROCHEM INT, V131, DOI 10.1016/j.neuint.2019.104583

Farr OM, 2016, DIABETOLOGIA, V59, P954, DOI 10.1007/s00125-016-3874-y

Filchenko I, 2018, DIABETES VASC DIS RE, V15, P567, DOI 10.1177/1479164118788079

Fiory F, 2019, FRONT NEUROSCI-SWITZ, V13, DOI 10.3389/fnins.2019.00868

Hong CT, 2020, CELLS-BASEL, V9, DOI 10.3390/cells9030740

Horvath I, 2016, P NATL ACAD SCI USA, V113, P12473, DOI 10.1073/pnas.1610371113

Huot P, 2015, SYNAPSE, V69, P99, DOI 10.1002/syn.21801

Hussain S, 2020, NEUROL SCI, V41, P3697, DOI 10.1007/s10072-020-04494-3

Iravanpour F, 2021, CNS NEUROSCI THER, V27, P308, DOI 10.1111/cns.13609

Jeong SH, 2021, BRAIN, V144, P1127, DOI 10.1093/brain/awab015

Jhamandas JH, 2011, AM J PATHOL, V178, P140, DOI 10.1016/j.ajpath.2010.11.022

Juárez-Flores DL, 2020, ANTIOXIDANTS-BASEL, V9, DOI 10.3390/antiox9111063

Kabel AM, 2018, PHYSIOL BEHAV, V188, P108, DOI 10.1016/j.physbeh.2018.01.028

Katila N, 2017, NEUROPHARMACOLOGY, V125, P396, DOI 10.1016/j.neuropharm.2017.08.015

Kim DS, 2017, CELL TRANSPLANT, V26, P1560, DOI 10.1177/0963689717721234

Kleinridders A, 2015, P NATL ACAD SCI USA, V112, P3463, DOI 10.1073/pnas.1500877112

Klimek P, 2015, PLOS COMPUT BIOL, V11, DOI 10.1371/journal.pcbi.1004125

Kong YY, 2020, FRONT NEUROSCI-SWITZ, V14, DOI 10.3389/fnins.2020.00682

Labandeira-Garcia JL, 2017, FRONT AGING NEUROSCI, V9, DOI 10.3389/fnagi.2017.00365

Li YW, 2017, EUR J PHARMACOL, V797, P162, DOI 10.1016/j.ejphar.2016.11.050

Li YZ, 2009, P NATL ACAD SCI USA, V106, P1285, DOI 10.1073/pnas.0806720106

Lim YA, 2008, FEBS LETT, V582, P2188, DOI 10.1016/j.febslet.2008.05.006

Liu W, 2015, NEUROSCIENCE, V303, P42, DOI 10.1016/j.neuroscience.2015.06.054

Ma DL, 2019, BRAIN RES, V1714, P126, DOI 10.1016/j.brainres.2019.02.030

Maffei A, 2015, MOL ENDOCRINOL, V29, P542, DOI 10.1210/me.2014-1273

Martinez-Valbuena I, 2018, ACTA NEUROPATHOL, V135, P877, DOI 10.1007/s00401-018-1832-0

Mazucanti CH, 2019, JCI INSIGHT, V4, DOI 10.1172/jci.insight.131682

Mietlicki-Baase EG, 2015, NEUROPSYCHOPHARMACOL, V40, P372, DOI 10.1038/npp.2014.180

Mousa SA, 2019, NEURAL REGEN RES, V14, P745, DOI 10.4103/1673-5374.249217

Mucibabic M, 2020, SCI REP-UK, V10, DOI 10.1038/s41598-020-77409-z

Mulvaney CA, 2020, COCHRANE DB SYST REV, DOI 10.1002/14651858.CD012990.pub2

Muscogiuri G, 2017, TRENDS ENDOCRIN MET, V28, P88, DOI 10.1016/j.tem.2016.10.001

Nassar NN, 2015, NEUROPHARMACOLOGY, V89, P308, DOI 10.1016/j.neuropharm.2014.10.007

Ou RW, 2021, ANN CLIN TRANSL NEUR, V8, P887, DOI 10.1002/acn3.51343

Pagano G, 2018, NEUROLOGY, V90, pE1654, DOI 10.1212/WNL.0000000000005475

Palleria C, 2017, BEHAV BRAIN RES, V321, P157, DOI 10.1016/j.bbr.2017.01.004

Perez-Taboada I, 2020, MOVEMENT DISORD, V35, P1636, DOI 10.1002/mds.28124

Ping F, 2020, BMJ OPEN DIAB RES CA, V8, DOI 10.1136/bmjdrc-2020-001370

Pisanu A, 2014, NEUROBIOL DIS, V71, P280, DOI 10.1016/j.nbd.2014.08.011

Rampersaud N, 2012, NEUROPEPTIDES, V46, P183, DOI 10.1016/j.npep.2012.07.004

Rhee SY, 2020, DIABETES CARE, V43, P2169, DOI 10.2337/dc19-0760

Ryu YK, 2020, NEUROPHARMACOLOGY, V175, DOI 10.1016/j.neuropharm.2020.108173

Sharma SK, 2015, SCI REP-UK, V5, DOI 10.1038/srep12531

Simuni T, 2015, LANCET NEUROL, V14, P795, DOI 10.1016/S1474-4422(15)00144-1

Spielman LJ, 2017, EUR J CELL BIOL, V96, P240, DOI 10.1016/j.ejcb.2017.03.004

Steculorum SM, 2014, ALZHEIMERS DEMENT, V10, pS3, DOI 10.1016/j.jalz.2013.12.008

Sun Y, 2020, FRONT NEUROSCI-SWITZ, V14, DOI 10.3389/fnins.2020.00063

Svenningsson P, 2016, MOVEMENT DISORD, V31, P1422, DOI 10.1002/mds.26734

Vella A, 2002, DIABETOLOGIA, V45, P1410, DOI 10.1007/s00125-002-0924-4

Vicente Miranda H, 2017, BRAIN, V140, P1399, DOI 10.1093/brain/awx056

Wang SY, 2020, INT J ENV RES PUB HE, V17, DOI 10.3390/ijerph17134805

Witoelar A, 2017, JAMA NEUROL, V74, P780, DOI 10.1001/jamaneurol.2017.0469

Yuan ZY, 2017, EUR J PHARMACOL, V812, P82, DOI 10.1016/j.ejphar.2017.06.029

Yue XJ, 2016, MEDICINE, V95, DOI 10.1097/MD.0000000000003549

Yun SP, 2018, NAT MED, V24, P931, DOI 10.1038/s41591-018-0051-5

Zhang LY, 2021, NEURAL REGEN RES, V16, P1660, DOI 10.4103/1673-5374.303045

Zhang LP, 2019, J PARKINSON DIS, V9, P157, DOI 10.3233/JPD-181503

NR 80

TC 28

Z9 31

U1 2

U2 37

PU WOLTERS KLUWER MEDKNOW PUBLICATIONS

PI MUMBAI

PA WOLTERS KLUWER INDIA PVT LTD , A-202, 2ND FLR, QUBE, C T S NO 1498A-2

VILLAGE MAROL, ANDHERI EAST, MUMBAI, Maharashtra, INDIA

SN 1673-5374

EI 1876-7958

J9 NEURAL REGEN RES

JI Neural Regen. Res.

PD AUG

PY 2022

VL 17

IS 8

BP 1652

EP 1658

DI 10.4103/1673-5374.332122

PG 7

WC Cell Biology; Neurosciences

WE Science Citation Index Expanded (SCI-EXPANDED)

SC Cell Biology; Neurosciences & Neurology

GA ZP5EI

UT WOS:000766445300006

PM 35017411

OA gold, Green Published

DA 2024-08-12

ER

PT J

AU Hoenig, MC

Dzialas, V

Drzezga, A

Van Eimeren, T

AF Hoenig, Merle C.

Dzialas, Verena

Drzezga, Alexander

Van Eimeren, Thilo

TI The Concept of Motor Reserve in Parkinson's Disease: New Wine in Old

Bottles?

SO MOVEMENT DISORDERS

LA English

DT Article

ID COGNITIVE RESERVE; NEUROINFLAMMATION; PATHOLOGY; EXERCISE; NETWORKS

C1 [Hoenig, Merle C.; Drzezga, Alexander] Res Ctr Juelich, Mol Org Brain, Inst Neurosci & Med 2, Julich, Germany.

[Hoenig, Merle C.; Dzialas, Verena; Drzezga, Alexander; Van Eimeren, Thilo] Univ Cologne, Fac Med, Dept Nucl Med, Cologne, Germany.

[Hoenig, Merle C.; Dzialas, Verena; Drzezga, Alexander; Van Eimeren, Thilo] Univ Hosp Cologne, Cologne, Germany.

[Dzialas, Verena] Univ Cologne, Fac Math & Nat Sci, Cologne, Germany.

[Drzezga, Alexander] Bonn Cologne, German Ctr Neurodegenerat Dis DZNE, Bonn, Germany.

[Van Eimeren, Thilo] Univ Cologne, Dept Neurol, Fac Med, Cologne, Germany.

[Van Eimeren, Thilo] Univ Cologne, Univ Hosp Cologne, Cologne, Germany.

C3 Helmholtz Association; Research Center Julich; University of Cologne;

University of Cologne; University of Cologne; Helmholtz Association;

German Center for neurodegenerative disease (DZNE); University of

Cologne; University of Cologne

RP Hoenig, MC (corresponding author), Res Ctr Juelich, Mol Org Brain, Inst Neurosci & Med II, Wilhelm Johnen Str, Julich, Germany.

EM m.hoenig@fz-juelich.de

RI van Eimeren, Thilo/C-6049-2014

OI van Eimeren, Thilo/0000-0002-6951-2325

FU Deutsche Forschungsgemeinschaft (German Research Foundation) [431549029,

SFB 1451, 233886668, GRK 1960]; German Research Foundation; Projekt DEAL

FX This work was supported by the Deutsche Forschungsgemeinschaft (German

Research Foundation; Project ID 431549029, SFB 1451 and Project ID

233886668, GRK 1960). We thank the German Research Foundation for their

funding. Open Access funding enabled and organized by Projekt DEAL.

CR Arenaza-Urquijo EM, 2020, ALZHEIMERS RES THER, V12, DOI 10.1186/s13195-020-00609-2

Arenaza-Urquijo EM, 2018, NEUROLOGY, V90, P695, DOI 10.1212/WNL.0000000000005303

Cascone AD, 2021, COMMUN BIOL, V4, DOI 10.1038/s42003-021-02478-3

Chen Y, 2018, SCI REP-UK, V8, DOI 10.1038/s41598-018-22868-8

Chung SJ, 2020, J MOV DISORD, V13, P171, DOI 10.14802/jmd.20029

Chung SJ, 2020, NEUROBIOL AGING, V92, P1, DOI 10.1016/j.neurobiolaging.2020.02.028

Chung SJ, 2020, MOVEMENT DISORD, V35, P577, DOI 10.1002/mds.28012

Chung SJ, 2019, J MOV DISORD, V12, P113, DOI 10.14802/jmd.18064

Coddington LT, 2019, NEURON, V104, P63, DOI 10.1016/j.neuron.2019.08.036

Dumitrescu Logan, 2020, Brain, V143, P2561, DOI 10.1093/brain/awaa209

Eickhoff CR, 2021, BRAIN COMMUN, V3, DOI 10.1093/braincomms/fcab191

Elkurd M, 2021, FRONT NEUROL, V12, DOI 10.3389/fneur.2021.711045

Elman JA, 2022, ALZHEIMERS RES THER, V14, DOI 10.1186/s13195-022-01049-w

Folle AD, 2019, PARKINSONISM RELAT D, V62, P105, DOI 10.1016/j.parkreldis.2019.01.018

Habeck C, 2003, NEUROIMAGE, V20, P1723, DOI 10.1016/j.neuroimage.2003.07.032

Hillary FG, 2017, TRENDS COGN SCI, V21, P385, DOI 10.1016/j.tics.2017.03.003

Hindle JV, 2015, PARKINSONS DIS-US, V2015, DOI 10.1155/2015/943572

Hoenig MC, 2017, NEUROBIOL AGING, V57, P1, DOI 10.1016/j.neurobiolaging.2017.05.004

Jang Y, 2017, BRAIN RES, V1655, P186, DOI 10.1016/j.brainres.2016.10.029

Kim YJ, 2022, PARKINSONISM RELAT D, V102, P108, DOI 10.1016/j.parkreldis.2022.08.005

Lee CS, 2000, ANN NEUROL, V47, P493, DOI 10.1002/1531-8249(200004)47:4<493::AID-ANA13>3.0.CO;2-4

Lucero C, 2015, PARKINSONISM RELAT D, V21, P899, DOI 10.1016/j.parkreldis.2015.05.020

Palermo G, 2019, CELLS-BASEL, V8, DOI 10.3390/cells8080872

Palmer SJ, 2009, EUR J NEUROSCI, V29, P2187, DOI 10.1111/j.1460-9568.2009.06753.x

Petkus A., 2020, INNOV AGING, V4, P501

Real CC, 2019, J CEREBR BLOOD F MET, V39, P989, DOI 10.1177/0271678X17750351

Real Caroline Cristiano, 2021, Dement. neuropsychol., V15, P41, DOI 10.1590/1980-57642021dn15-010004

Real CC, 2017, J MOL NEUROSCI, V63, P36, DOI 10.1007/s12031-017-0955-4

Reed BR, 2010, BRAIN, V133, P2196, DOI 10.1093/brain/awq154

Sacheli MA, 2018, MOVEMENT DISORD, V33, P1945, DOI 10.1002/mds.27498

Shine JM, 2019, BRAIN, V142, P1024, DOI 10.1093/brain/awz034

Stern Y, 2002, J INT NEUROPSYCH SOC, V8, P448, DOI 10.1017/S1355617702813248

Stern Y, 2020, ALZHEIMERS DEMENT, V16, P1305, DOI 10.1016/j.jalz.2018.07.219

Thaler A, 2018, PARKINSONISM RELAT D, V55, P45, DOI 10.1016/j.parkreldis.2018.05.009

Wang LB, 2022, NEUROLOGY, V99, pE977, DOI 10.1212/WNL.0000000000200814

Wood KH, 2021, J PARKINSON DIS, V11, P703, DOI 10.3233/JPD-202215

NR 36

TC 7

Z9 7

U1 0

U2 3

PU WILEY

PI HOBOKEN

PA 111 RIVER ST, HOBOKEN 07030-5774, NJ USA

SN 0885-3185

EI 1531-8257

J9 MOVEMENT DISORD

JI Mov. Disord.

PD JAN

PY 2023

VL 38

IS 1

BP 16

EP 20

DI 10.1002/mds.29266

EA NOV 2022

PG 5

WC Clinical Neurology

WE Science Citation Index Expanded (SCI-EXPANDED)

SC Neurosciences & Neurology

GA L4ZW5

UT WOS:000879614200001

PM 36345092

OA Green Published, hybrid

DA 2024-08-12

ER

PT J

AU Borrageiro, G

Haylett, W

Seedat, S

Kuivaniemi, H

Bardien, S

AF Borrageiro, Genevie

Haylett, William

Seedat, Soraya

Kuivaniemi, Helena

Bardien, Soraya

TI A review of genome-wide transcriptomics studies in Parkinson's disease

SO EUROPEAN JOURNAL OF NEUROSCIENCE

LA English

DT Review

DE blood; brain; differential gene expression; Parkinson's disease; pathway

analysis

ID CELL-RELATED GENES; SUBSTANTIA-NIGRA; DOPAMINE NEURONS; ALPHA-SYNUCLEIN;

CANDIDATE GENES; RNA-SEQ; UBIQUITIN-PROTEASOME; BRAIN TRANSCRIPTOME;

EXPRESSION ANALYSIS; REGULATORY NETWORK

AB Parkinson's disease (PD) is a progressive and incurable neurodegenerative disorder. Although numerous genetic and environmental factors have been linked to the aetiology of PD the underlying pathobiology remains poorly understood, hampering the development of improved therapies. Transcriptomics has the potential to reveal significant insights into disease processes. In this review, we focused on published transcriptomics studies on PD with the aim of summarizing studies and identifying common biological pathways. A total of 96 articles were identified as follows: 12 meta-analyses, 21 re-analyses of existing data and 63 original studies. Of the 63 original studies, 33 were performed on brain tissue, 26 on blood, three on cerebrospinal fluid and one on skin. In the brain studies, altered pathways identified included those involved in dopamine metabolism, mitochondrial function, oxidative stress, protein degradation, neuroinflammation, vesicular transport and synaptic transmission. Studies on blood samples revealed alterations in pathways involved in immune function, inflammation, RNA processing, protein chaperones, mitochondrial function and programmed cell death. Limitations of these studies include small sample sizes (generally <40 cases/40 controls) and the application of widely varying statistical analysis and parameters. Only eight studies used the RNA-Seq technique. This review highlights the need for harmonization of transcriptomic approaches and the statistical analyses, and for the data to be deposited into publicly available databases in a standardized format for meta-analyses. Notably, the concordance of several pathways such as mitochondrial function, protein degradation and inflammation, identified in both blood and brain tissues, suggests that the disease process is systemic and not restricted to neurological tissues.

C1 [Borrageiro, Genevie; Haylett, William; Kuivaniemi, Helena; Bardien, Soraya] Stellenbosch Univ, Fac Med & Hlth Sci, Dept Biomed Sci, Div Mol Biol & Human Genet, POB 241, Cape Town, South Africa.

[Seedat, Soraya; Kuivaniemi, Helena] Stellenbosch Univ, Fac Med & Hlth Sci, Dept Psychiat, Cape Town, South Africa.

C3 Stellenbosch University; Stellenbosch University

RP Bardien, S (corresponding author), Stellenbosch Univ, Fac Med & Hlth Sci, Dept Biomed Sci, Div Mol Biol & Human Genet, POB 241, Cape Town, South Africa.

EM sbardien@sun.ac.za

RI Kuivaniemi, Helena/Q-8178-2019; Bardien, Soraya/ABB-3313-2020; Bardien,

Soraya/AAJ-9003-2020; Siabani, Soraya/U-7739-2017

OI Kuivaniemi, Helena/0000-0001-5753-8766; Bardien,

Soraya/0000-0002-3508-3438; Seedat, Soraya/0000-0002-5118-786X

FU South African Medical Research Council Flagship Grant; Stellenbosch

University Faculty of Medicine and Health Sciences Subcommittee C

Postdoctoral Fellowship; National Research Foundation Grantholder-Linked

Postdoctoral Bursary

FX The South African Medical Research Council Flagship Grant

(RFA-UFSP-01-2013) is acknowledged for funding this work. WH is funded

by a Stellenbosch University Faculty of Medicine and Health Sciences

Subcommittee C Postdoctoral Fellowship and a National Research

Foundation Grantholder-Linked Postdoctoral Bursary.

CR Alieva AK, 2014, BIOMED RES INT, V2014, DOI 10.1155/2014/718732

Bender A, 2006, NAT GENET, V38, P515, DOI 10.1038/ng1769

Betarbet R, 2000, NAT NEUROSCI, V3, P1301, DOI 10.1038/81834

Bossers K, 2009, BRAIN PATHOL, V19, P91, DOI 10.1111/j.1750-3639.2008.00171.x

Botta-Orfila T, 2014, J NEUROSCI RES, V92, P1071, DOI 10.1002/jnr.23377

Botta-Orfila T, 2012, BRAIN RES, V1466, P152, DOI 10.1016/j.brainres.2012.05.036

Botta-Orfila T, 2012, NEUROBIOL DIS, V45, P462, DOI 10.1016/j.nbd.2011.08.033

Braak H, 2003, NEUROBIOL AGING, V24, P197, DOI 10.1016/S0197-4580(02)00065-9

Brazma A, 2001, NAT GENET, V29, P365, DOI 10.1038/ng1201-365

Briggs CE, 2015, BRAIN RES, V1618, P111, DOI 10.1016/j.brainres.2015.05.021

Burgos K, 2014, PLOS ONE, V9, DOI 10.1371/journal.pone.0094839

Calligaris R, 2015, BMC GENOMICS, V16, DOI 10.1186/s12864-015-2058-3

Cantuti-Castelvetri I, 2007, NEUROBIOL DIS, V26, P606, DOI 10.1016/j.nbd.2007.02.009

Capurro A, 2015, FRONT NEUROSCI-SWITZ, V8, DOI 10.3389/fnins.2014.00441

Cardo LF, 2014, J MOL NEUROSCI, V54, P830, DOI 10.1007/s12031-014-0428-y

Cardo LF, 2013, J NEUROL, V260, P1420, DOI 10.1007/s00415-013-6900-8

Chandrasekaran S, 2013, COMPUT STRUCT BIOTEC, V7, DOI 10.5936/csbj.201304004

Chatterjee P, 2017, BIOCHEM BIOPH RES CO, V484, P557, DOI 10.1016/j.bbrc.2017.01.121

Chatterjee P, 2014, PLOS ONE, V9, DOI 10.1371/journal.pone.0093751

Cooper-Knock J, 2012, NAT REV NEUROL, V8, P518, DOI 10.1038/nrneurol.2012.156

Corradini BR, 2014, BIOMED RES INT-UK, V2014, DOI 10.1155/2014/543673

Costa V, 2013, EUR J HUM GENET, V21, P134, DOI 10.1038/ejhg.2012.129

Cruz-Monteagudo M, 2016, BMC MED GENOMICS, V9, DOI 10.1186/s12920-016-0173-x

Dauer W, 2003, NEURON, V39, P889, DOI 10.1016/S0896-6273(03)00568-3

Aguiar PMD, 2010, EINSTEIN-SAO PAULO, V8, P291, DOI [10.1590/S1679-45082010AO1674, 10.1590/s1679-45082010ao1674]

de Lau LML, 2006, LANCET NEUROL, V5, P525, DOI 10.1016/S1474-4422(06)70471-9

Devine MJ, 2011, PLOS ONE, V6, DOI 10.1371/journal.pone.0022489

Diao B, 2011, AFR J BIOTECHNOL, V10, P15656, DOI 10.5897/AJB11.2121

Diao HY, 2013, GENE, V515, P159, DOI 10.1016/j.gene.2012.11.003

Dijkstra AA, 2015, PLOS ONE, V10, DOI 10.1371/journal.pone.0128651

Ding HX, 2016, PARKINSONISM RELAT D, V22, P68, DOI 10.1016/j.parkreldis.2015.11.014

Dong H, 2016, BIOMARKERS, V21, P129, DOI 10.3109/1354750X.2015.1118544

Dong N, 2017, MOL MED REP, V15, P731, DOI 10.3892/mmr.2016.6044

Doxakis E, 2010, J BIOL CHEM, V285, P12726, DOI 10.1074/jbc.M109.086827

Duke DC, 2006, NEUROGENETICS, V7, P139, DOI 10.1007/s10048-006-0033-5

Dumitriu A, 2016, BMC MED GENOMICS, V9, DOI 10.1186/s12920-016-0164-y

Dumitriu A, 2012, PARKINSONS DIS-US, V2012, DOI 10.1155/2012/614212

Durrenberger PF, 2012, PARKINSONS DIS-US, V2012, DOI 10.1155/2012/214714

Dusonchet J, 2014, HUM MOL GENET, V23, P4887, DOI 10.1093/hmg/ddu202

Edwards YJK, 2011, PLOS ONE, V6, DOI 10.1371/journal.pone.0016917

Elstner M, 2011, ACTA NEUROPATHOL, V122, P75, DOI 10.1007/s00401-011-0828-9

Elstner M, 2009, ANN NEUROL, V66, P792, DOI 10.1002/ana.21780

Feng YL, 2017, MOL MED REP, V15, P1252, DOI 10.3892/mmr.2017.6124

Gan-Or Z, 2015, AUTOPHAGY, V11, P1443, DOI 10.1080/15548627.2015.1067364

Garcia-Esparcia P, 2013, J NEUROPATH EXP NEUR, V72, P524, DOI 10.1097/NEN.0b013e318294fd76

Gautier CA, 2014, REV NEUROL-FRANCE, V170, P339, DOI 10.1016/j.neurol.2013.06.003

Glaab E, 2015, NEUROBIOL DIS, V74, P1, DOI 10.1016/j.nbd.2014.11.002

Greene JG, 2012, NEUROBIOL DIS, V45, P76, DOI 10.1016/j.nbd.2010.10.022

Grünblatt E, 2004, J NEURAL TRANSM, V111, P1543, DOI 10.1007/s00702-004-0212-1

Guhathakurta S, 2017, PROG NEUROBIOL, V154, P21, DOI 10.1016/j.pneurobio.2017.04.004

Gui YX, 2015, ONCOTARGET, V6, P37043, DOI 10.18632/oncotarget.6158

Hauser MA, 2005, ARCH NEUROL-CHICAGO, V62, P917, DOI 10.1001/archneur.62.6.917

Hawkes CH, 1999, QJM-MON J ASSOC PHYS, V92, P473, DOI 10.1093/qjmed/92.8.473

Henderson-Smith A, 2016, NEUROL-GENET, V2, DOI 10.1212/NXG.0000000000000075

Hoss AG, 2016, FRONT AGING NEUROSCI, V8, DOI 10.3389/fnagi.2016.00036

Hossein-nezhad A, 2016, J PARKINSON DIS, V6, P109, DOI 10.3233/JPD-150737

Hu M, 2011, J NEUROL, V258, P1513, DOI 10.1007/s00415-011-5972-6

Infante J, 2016, NEUROBIOL AGING, V38, DOI 10.1016/j.neurobiolaging.2015.10.026

Infante J, 2015, NEUROBIOL AGING, V36, P1105, DOI 10.1016/j.neurobiolaging.2014.10.039

Karlsson MK, 2013, J PARKINSON DIS, V3, P19, DOI 10.3233/JPD-120159

Kavanagh T, 2013, J MOL NEUROSCI, V51, P28, DOI 10.1007/s12031-012-9940-0

Kedmi M, 2011, MOL NEURODEGENER, V6, DOI 10.1186/1750-1326-6-66

Khoo SK, 2012, J PARKINSON DIS, V2, P321, DOI 10.3233/JPD-012144

Kim W, 2014, NEUROBIOL AGING, V35, P1712, DOI 10.1016/j.neurobiolaging.2014.01.021

Klein C, 2012, CSH PERSPECT MED, V2, DOI 10.1101/cshperspect.a008888

Kobo H, 2016, MOL GENET METAB, V117, P179, DOI 10.1016/j.ymgme.2015.09.005

Lev N, 2003, PROG NEURO-PSYCHOPH, V27, P245, DOI 10.1016/S0278-5846(03)00019-8

Lewandowski NM, 2010, P NATL ACAD SCI USA, V107, P16970, DOI 10.1073/pnas.1011751107

Li TR, 2016, NEUROL SCI, V37, P1327, DOI 10.1007/s10072-016-2595-9

Liao X.-Y., 2013, INT J GENOMICS, V2013, P1

Lill CM, 2016, MOL CELL PROBE, V30, P386, DOI 10.1016/j.mcp.2016.11.001

Liu S, 2016, NEUROL SCI, V37, P73, DOI 10.1007/s10072-015-2360-5

Liu WC, 2011, P NATL ACAD SCI USA, V108, P12920, DOI 10.1073/pnas.1107332108

Mariani E, 2016, PLOS ONE, V11, DOI 10.1371/journal.pone.0161567

Martins M, 2011, PLOS ONE, V6, DOI 10.1371/journal.pone.0025443

Martins-Branco D, 2012, EXP NEUROL, V238, P89, DOI 10.1016/j.expneurol.2012.08.008

Miller RM, 2006, NEUROBIOL DIS, V21, P305, DOI 10.1016/j.nbd.2005.07.010

Miñones-Moyano E, 2013, RNA BIOL, V10, P1093, DOI 10.4161/rna.24813

Miñones-Moyano E, 2011, HUM MOL GENET, V20, P3067, DOI 10.1093/hmg/ddr210

Moran LB, 2006, NEUROGENETICS, V7, P1, DOI 10.1007/s10048-005-0020-2

Moran LB, 2008, NEUROGENETICS, V9, P1, DOI 10.1007/s10048-007-0116-y

Mutez E, 2014, NEUROBIOL DIS, V63, P165, DOI 10.1016/j.nbd.2013.11.007

Mutez E, 2011, NEUROBIOL AGING, V32, P1839, DOI 10.1016/j.neurobiolaging.2009.10.016

Nair VD, 2016, NEUROSCI LETT, V629, P99, DOI 10.1016/j.neulet.2016.06.061

Narendra D, 2009, AUTOPHAGY, V5, P706, DOI 10.4161/auto.5.5.8505

Naydenov AV, 2010, ACTA NEUROPATHOL, V120, P623, DOI 10.1007/s00401-010-0740-8

Nkiliza A, 2016, NEUROBIOL DIS, V96, P312, DOI 10.1016/j.nbd.2016.09.014

Papapetropoulos S, 2006, GENE EXPRESSION, V13, P205, DOI 10.3727/000000006783991827

Picconi B, 2012, ADV EXP MED BIOL, V970, P553, DOI 10.1007/978-3-7091-0932-8_24

Pinho R, 2016, PLOS ONE, V11, DOI 10.1371/journal.pone.0157852

Planken A, 2017, BMC NEUROL, V17, DOI 10.1186/s12883-016-0784-z

Potashkin JA, 2012, PLOS ONE, V7, DOI 10.1371/journal.pone.0043595

Rakshit H, 2014, PLOS ONE, V9, DOI 10.1371/journal.pone.0103047

Riley BE, 2014, PLOS ONE, V9, DOI 10.1371/journal.pone.0102909

Santiago JA, 2015, P NATL ACAD SCI USA, V112, P2257, DOI 10.1073/pnas.1423573112

SCHAPIRA AHV, 1990, J NEUROCHEM, V54, P823, DOI 10.1111/j.1471-4159.1990.tb02325.x

Scherzer CR, 2007, P NATL ACAD SCI USA, V104, P955, DOI 10.1073/pnas.0610204104

Shehadeh LA, 2010, PLOS ONE, V5, DOI 10.1371/journal.pone.0009104

Sherer TB, 2003, EXP NEUROL, V179, P9, DOI 10.1006/exnr.2002.8072

SIAN J., 1999, MPTP-Induced Parkinsonian Syndrome. Basic Neurochemistry: Molecular, V6th

Simunovic F, 2010, PLOS ONE, V5, DOI 10.1371/journal.pone.0008856

Simunovic F, 2009, BRAIN, V132, P1795, DOI 10.1093/brain/awn323

Soreq L, 2008, J NEUROIMMUNOL, V201, P227, DOI 10.1016/j.jneuroim.2008.06.019

Soreq L, 2014, PLOS COMPUT BIOL, V10, DOI 10.1371/journal.pcbi.1003517

Soreq L, 2013, BMC GENOMICS, V14, DOI 10.1186/1471-2164-14-478

Soreq L, 2013, FRONT MOL NEUROSCI, V6, DOI 10.3389/fnmol.2013.00010

Soreq L, 2012, NEURODEGENER DIS, V10, P203, DOI 10.1159/000332598

Soreq L, 2012, J CELL MOL MED, V16, P1496, DOI 10.1111/j.1582-4934.2011.01444.x

Soto-Ortolaza AI, 2016, PARKINSONISM RELAT D, V22, pS7, DOI 10.1016/j.parkreldis.2015.09.011

Spillantini MG, 1997, NATURE, V388, P839, DOI 10.1038/42166

Stamper C, 2008, MOVEMENT DISORD, V23, P1588, DOI 10.1002/mds.22184

Sun AG, 2016, INT J NEUROSCI, V126, P318, DOI 10.3109/00207454.2014.986673

Sun AG, 2014, NEUROENDOCRINOL LETT, V35, P398

Sutherland GT, 2009, PLOS ONE, V4, DOI 10.1371/journal.pone.0004955

Tang FC, 2010, NAT PROTOC, V5, P516, DOI 10.1038/nprot.2009.236

Vallelunga A, 2014, FRONT CELL NEUROSCI, V8, DOI 10.3389/fncel.2014.00156

Viswambharan V, 2017, BIOMARK MED, V11, P151, DOI 10.2217/bmm-2016-0242

Vogt IR, 2006, EXP NEUROL, V199, P465, DOI 10.1016/j.expneurol.2006.01.008

Wake C, 2016, BMC GENOMICS, V17, DOI 10.1186/s12864-016-3114-3

Wang Q, 2017, J ALZHEIMERS DIS, V56, P1525, DOI 10.3233/JAD-161032

Wang QQ, 2015, TRANSL NEURODEGENER, V4, DOI 10.1186/s40035-015-0042-0

Xia RP, 2012, NEUROSCI BULL, V28, P39, DOI 10.1007/s12264-012-1050-z

Zhang B, 2012, MOL BIOL REP, V39, P10881, DOI 10.1007/s11033-012-1985-1

Zhang Y, 2014, BIOMED ENVIRON SCI, V27, P462, DOI 10.3967/bes2014.074

Zhang YL, 2005, AM J MED GENET B, V137B, P5, DOI 10.1002/ajmg.b.30195

Zheng B, 2010, SCI TRANSL MED, V2, DOI 10.1126/scitranslmed.3001059

Zou FG, 2012, PLOS GENET, V8, DOI 10.1371/journal.pgen.1002707

NR 127

TC 72

Z9 79

U1 1

U2 23

PU WILEY

PI HOBOKEN

PA 111 RIVER ST, HOBOKEN 07030-5774, NJ USA

SN 0953-816X

EI 1460-9568

J9 EUR J NEUROSCI

JI Eur. J. Neurosci.

PD JAN

PY 2018

VL 47

IS 1

BP 1

EP 16

DI 10.1111/ejn.13760

PG 16

WC Neurosciences

WE Science Citation Index Expanded (SCI-EXPANDED)

SC Neurosciences & Neurology

GA FS0SZ

UT WOS:000419485300001

PM 29068110

DA 2024-08-12

ER

PT J

AU Zang, CX

Wang, L

Yang, HY

Shang, JM

Liu, H

Zhang, ZH

Ju, C

Yuan, FY

Li, FY

Bao, XQ

Zhang, D

AF Zang, Cai-Xia

Wang, Lu

Yang, Han-Yu

Shang, Jun-Mei

Liu, Hui

Zhang, Zi-Hong

Ju, Cheng

Yuan, Fang-Yu

Li, Fang-Yuan

Bao, Xiu-Qi

Zhang, Dan

TI HACE1 negatively regulates neuroinflammation through ubiquitylating and

degrading Rac1 in Parkinson's disease models

SO ACTA PHARMACOLOGICA SINICA

LA English

DT Article

DE HACE1; Rac1 activity; neuroinflammation; LPS; BV2 microglial cells;

MPTP-induced acute PD mouse model; alpha-synuclein transgenic mice;

Parkinson's disease

ID NADPH; ROS; MICROGLIA

AB Neuroinflammation plays an important role in neurodegenerative disease, such as Parkinson's disease (PD) and Alzheimer's disease. HACE1 (HECT domain and Ankyrin repeat Containing E3 ubiquitin-protein ligase 1) is a tumor suppressor. Recent evidence suggests that HACE1 may be involved in oxidative stress responses. Due to the critical role of ROS in neuroinflammation, we speculated that HACE1 might participate in neuroinflammation and related neurodegenerative disease, such as PD. In this study, we investigated the role of HACE1 in neuroinflammation of PD models. We showed that HACE1 knockdown exacerbated LPS-induced neuroinflammation in BV2 microglial cells in vitro through suppressing ubiquitination and degradation of activated Rac1, an NADPH oxidase subunit. Furthermore, we showed that HACE1 exerted vital neuronal protection through increasing Rac1 activity and stability in LPS-treated SH-SY5Y cells, as HACE1 knockdown leading to lower tolerance to LPS challenge. In MPTP-induced acute PD mouse model, HACE1 knockdown exacerbated motor deficits by activating Rac1. Finally, mutant alpha-synuclein (A53T)-overexpressing mice, a chronic PD mouse model, exhibited age-dependent reduction of HACE1 levels in the midbrain and striatum, implicating that HACE1 participated in PD pathological progression. This study for the first time demonstrates that HACE1 is a negative regulator of neuroinflammation and involved in the PD pathogenesis by regulating Rac1 activity. The data support HACE1 as a potential target for PD and other neurodegenerative disease.

C1 [Zang, Cai-Xia; Wang, Lu; Yang, Han-Yu; Shang, Jun-Mei; Liu, Hui; Zhang, Zi-Hong; Ju, Cheng; Yuan, Fang-Yu; Li, Fang-Yuan; Bao, Xiu-Qi; Zhang, Dan] Chinese Acad Med Sci & Peking Union Med Coll, Inst Mat Med, Dept Pharmacol, State Key Lab Bioact Subst & Funct Nat Med, Beijing 100050, China.

C3 Chinese Academy of Medical Sciences - Peking Union Medical College;

Peking Union Medical College

RP Zhang, D (corresponding author), Chinese Acad Med Sci & Peking Union Med Coll, Inst Mat Med, Dept Pharmacol, State Key Lab Bioact Subst & Funct Nat Med, Beijing 100050, China.

EM danzhang@imm.ac.cn

FU Chinese Academy of Medical Sciences Innovation Fund for Medical Sciences

[2016-I2M-3-011]; Chinese Academy of Medical Sciences Fundamental

Research Funds for the Central Universities [2018RC350002]; National

Natural Science Foundation of China [81630097, 81773718, 21772235]

FX The study was supported by grants from Chinese Academy of Medical

Sciences Innovation Fund for Medical Sciences (No. 2016-I2M-3-011),

Chinese Academy of Medical Sciences Fundamental Research Funds for the

Central Universities (No. 2018RC350002), National Natural Science

Foundation of China (No. 81630097, 81773718, 21772235).

CR Acosta MI, 2018, SCI REP-UK, V8, DOI 10.1038/s41598-018-19471-2

Bedard K, 2007, PHYSIOL REV, V87, P245, DOI 10.1152/physrev.00044.2005

Bischoff LJM, 2019, ARCH TOXICOL, V93, P435, DOI 10.1007/s00204-018-2353-2

Bos JL, 2007, CELL, V129, P865, DOI 10.1016/j.cell.2007.05.018

Cetinbas N, 2015, ONCOGENE, V34, P4005, DOI 10.1038/onc.2014.316

Chiurchiù V, 2011, ANTIOXID REDOX SIGN, V15, P2605, DOI 10.1089/ars.2010.3547

DAMIER P, 1993, NEUROSCIENCE, V52, P1, DOI 10.1016/0306-4522(93)90175-F

Daugaard M, 2013, NAT COMMUN, V4, DOI 10.1038/ncomms3180

Fernandez-Marcos PJ, 2016, ONCOTARGET, V7, P50814, DOI 10.18632/oncotarget.10744

Goka ET, 2015, ONCOGENE, V34, P5395, DOI 10.1038/onc.2014.468

Ha Duy, 2012, Parkinsonism Relat Disord, V18 Suppl 1, pS218, DOI 10.1016/S1353-8020(11)70067-0

Hirsch EC, 2009, LANCET NEUROL, V8, P382, DOI 10.1016/S1474-4422(09)70062-6

Hurley LL, 2013, NEUROTOX RES, V23, P131, DOI 10.1007/s12640-012-9348-1

Imamura K, 2003, ACTA NEUROPATHOL, V106, P518, DOI 10.1007/s00401-003-0766-2

Kempuraj D, 2016, J Neurol Neurosurg Spine, V1

Kempuraj D, 2017, FRONT CELL NEUROSCI, V11, DOI 10.3389/fncel.2017.00216

Kumar B, 2019, J VIROL, V93, DOI 10.1128/JVI.01812-18

Lam GY, 2010, SEMIN IMMUNOPATHOL, V32, P415, DOI 10.1007/s00281-010-0221-0

Lawrimore CJ, 2019, BRAIN SCI, V9, DOI 10.3390/brainsci9090228

Lee IT, 2012, BIOCHEM PHARMACOL, V84, P581, DOI 10.1016/j.bcp.2012.05.005

Linnerbauer M, 2020, NEURON, V108, P608, DOI 10.1016/j.neuron.2020.08.012

Mettouchi Amel, 2012, Small GTPases, V3, P102, DOI 10.4161/sgtp.19221

Milton VJ, 2012, DEV NEUROBIOL, V72, P100, DOI 10.1002/dneu.20957

Ouchi Y, 2005, ANN NEUROL, V57, P168, DOI 10.1002/ana.20338

Park J, 2015, MOL PAIN, V11, DOI 10.1186/s12990-015-0026-1

Perry VH, 2013, SEMIN IMMUNOPATHOL, V35, P601, DOI 10.1007/s00281-013-0382-8

Qin HW, 2016, J NEUROSCI, V36, P5144, DOI 10.1523/JNEUROSCI.4658-15.2016

Qin LY, 2004, J BIOL CHEM, V279, P1415, DOI 10.1074/jbc.M307657200

Rathinam R, 2011, FRONT BIOSCI-LANDMRK, V16, P2561, DOI 10.2741/3872

Razaghi B, 2018, DEV DYNAM, V247, P289, DOI [10.1002/DVDY.24600, 10.1002/dvdy.24600]

Samii A, 2004, LANCET, V363, P1783, DOI 10.1016/S0140-6736(04)16305-8

Schain M, 2017, CURR NEUROL NEUROSCI, V17, DOI 10.1007/s11910-017-0733-2

Tansey MG, 2010, NEUROBIOL DIS, V37, P510, DOI 10.1016/j.nbd.2009.11.004

Yan ZQ, 2018, CLIN IMMUNOL, V189, P4, DOI 10.1016/j.clim.2016.09.014

Yang QQ, 2019, GLIA, V67, P1017, DOI 10.1002/glia.23571

Yauger YJ, 2019, J NEUROINFLAMM, V16, DOI 10.1186/s12974-019-1430-7

Zhang LY, 2014, NAT COMMUN, V5, DOI 10.1038/ncomms4430

NR 37

TC 9

Z9 10

U1 4

U2 17

PU NATURE PUBL GROUP

PI LONDON

PA MACMILLAN BUILDING, 4 CRINAN ST, LONDON N1 9XW, United Kingdom

SN 1671-4083

EI 1745-7254

J9 ACTA PHARMACOL SIN

JI Acta Pharmacol. Sin.

PD FEB

PY 2022

VL 43

IS 2

BP 285

EP 294

DI 10.1038/s41401-021-00778-2

EA SEP 2021

PG 10

WC Chemistry, Multidisciplinary; Pharmacology & Pharmacy

WE Science Citation Index Expanded (SCI-EXPANDED)

SC Chemistry; Pharmacology & Pharmacy

GA YN4TG

UT WOS:000702175000001

PM 34593974

OA Green Published, Bronze

DA 2024-08-12

ER

PT J

AU De Chirico, F

Poeta, E

Babini, G

Piccolino, I

Monti, B

Massenzio, F

AF De Chirico, Francesca

Poeta, Eleonora

Babini, Giorgia

Piccolino, Iliana

Monti, Barbara

Massenzio, Francesca

TI New models of Parkinson's like neuroinflammation in human microglia

clone 3: Activation profiles induced by INF-γ plus high glucose and

mitochondrial inhibitors

SO FRONTIERS IN CELLULAR NEUROSCIENCE

LA English

DT Article

DE human microglia; HMC3; neuroinflammation; Parkinson's disease;

IFN-gamma; glucose; 6-OHDA; MPTP

ID NF-KAPPA-B; MOUSE MODEL; INTERFERON-GAMMA; DISEASE; CELLS; MPTP;

EXPRESSION; 6-HYDROXYDOPAMINE; POLARIZATION; PROTECTS

AB Microglia activation and neuroinflammation have been extensively studied in murine models of neurodegenerative disease; however, to overcome the genetic differences between species, a human cell model of microglia able to recapitulate the activation profiles described in patients is needed. Here we developed human models of Parkinson's like neuroinflammation by using the human microglia clone 3 (HMC3) cells, whose activation profile in response to classic inflammatory stimuli has been controversial and reported only at mRNA levels so far. In fact, we showed the increased expression of the pro-inflammatory markers iNOS, Caspase 1, IL-1 beta, in response to IFN-gamma plus high glucose, a non-specific disease stimulus that emphasized the dynamic polarization and heterogenicity of the microglial population. More specifically, we demonstrated the polarization of HMC3 cells through the upregulation of iNOS expression and nitrite production in response to the Parkinson's like stimuli, 6-hydroxidopamine (6-OHDA) and 1-methyl-4-phenyl-1,2,3,6-tetrahydropyridine (MPTP), the latter depending on the NF-kappa B pathway. Furthermore, we identified inflammatory mediators that promote the pro-inflammatory activation of human microglia as function of different pathways that can simulate the phenotypic transition according to the stage of the pathology. In conclusion, we established and characterized different systems of HMC3 cells activation as in vitro models of Parkinson's like neuroinflammation.

C1 [De Chirico, Francesca; Poeta, Eleonora; Babini, Giorgia; Piccolino, Iliana; Monti, Barbara; Massenzio, Francesca] Univ Bologna, Dept Pharm & Biotechnol, Bologna, Italy.

C3 University of Bologna

RP Massenzio, F (corresponding author), Univ Bologna, Dept Pharm & Biotechnol, Bologna, Italy.

EM francesca.massenzio2@unibo.it

RI Massenzio, Francesca/AEL-3075-2022

OI Massenzio, Francesca/0000-0002-5189-7546; Poeta,

Eleonora/0009-0005-9479-1230; Monti, Barbara/0000-0003-0330-482X

FU University of Bologna

FX This research was supported by RFO fund and ALMArie CURIE supplied by

the University of Bologna.

CR Akhter R, 2021, MOL IMMUNOL, V131, P171, DOI 10.1016/j.molimm.2020.12.035

Alexandrov PN, 2013, J INORG BIOCHEM, V128, P267, DOI 10.1016/j.jinorgbio.2013.05.010

Augusto-Oliveira M, 2019, CELLS-BASEL, V8, DOI 10.3390/cells8101293

Bachstetter AD, 2015, ACTA NEUROPATHOL COM, V3, DOI 10.1186/s40478-015-0209-z

Bennett ML, 2016, P NATL ACAD SCI USA, V113, pE1738, DOI 10.1073/pnas.1525528113

Bhurtel S, 2019, NEUROTOXICOLOGY, V71, P113, DOI 10.1016/j.neuro.2018.12.009

Brough D, 2007, J CELL SCI, V120, P772, DOI 10.1242/jcs.03377

Buccitelli C, 2020, NAT REV GENET, V21, P630, DOI 10.1038/s41576-020-0258-4

Butovsky O, 2018, NAT REV NEUROSCI, V19, P622, DOI 10.1038/s41583-018-0057-5

Cai MY, 2022, NEUROCHEM INT, V152, DOI 10.1016/j.neuint.2021.105221

Carpentier PA, 2008, BRAIN BEHAV IMMUN, V22, P140, DOI 10.1016/j.bbi.2007.08.011

Cavaliere C, 2020, FRONT CELL NEUROSCI, V14, DOI 10.3389/fncel.2020.00075

Chauhan P, 2021, BRAIN SCI, V11, DOI 10.3390/brainsci11111482

Chen CD, 2019, AGING-US, V11, P697, DOI 10.18632/aging.101770

Chen WY, 2018, INT J MOL SCI, V19, DOI 10.3390/ijms19113678

Cheng SC, 2014, SCIENCE, V345, P1579, DOI 10.1126/science.1250684

Chiu YJ, 2021, AGING-US, V13, P15620, DOI 10.18632/aging.203125

Chiu YJ, 2020, AGING-US, V12, P23619, DOI 10.18632/aging.103700

da Silva TP, 2016, NEUROSCI LETT, V622, P83, DOI 10.1016/j.neulet.2016.04.049

Davis BM, 2017, SCI REP-UK, V7, DOI 10.1038/s41598-017-01747-8

Deczkowska A, 2018, CELL, V173, P1073, DOI 10.1016/j.cell.2018.05.003

Dehmer T, 2004, J NEUROCHEM, V88, P494, DOI 10.1046/j.1471-4159.2003.02210.x

Dehmer T, 2000, J NEUROCHEM, V74, P2213, DOI 10.1046/j.1471-4159.2000.0742213.x

Dello Russo C, 2018, J NEUROINFLAMM, V15, DOI 10.1186/s12974-018-1288-0

Dosch M, 2019, ELIFE, V8, DOI 10.7554/eLife.42670

Fathi M, 2022, FRONT AGING NEUROSCI, V14, DOI 10.3389/fnagi.2022.855776

Machado MMF, 2019, PHARMACOL REP, V71, P556, DOI 10.1016/j.pharep.2018.11.005

Fu RY, 2014, MOL NEUROBIOL, V49, P1422, DOI 10.1007/s12035-013-8620-6

Galatro TF, 2017, NAT NEUROSCI, V20, P1162, DOI 10.1038/nn.4597

Gao F, 2013, PLOS ONE, V8, DOI 10.1371/journal.pone.0072046

Gasparotto J, 2017, SCI REP-UK, V7, DOI 10.1038/s41598-017-09257-3

Gerhard A, 2006, NEUROBIOL DIS, V21, P404, DOI 10.1016/j.nbd.2005.08.002

Ghosh N, 2018, NEUROSCI RES, V137, P36, DOI 10.1016/j.neures.2018.02.007

Gibbons HA, 2007, J NEUROSCI METH, V166, P89, DOI 10.1016/j.jneumeth.2007.07.005

Gimeno-Bayón J, 2014, J NEUROSCI RES, V92, P723, DOI 10.1002/jnr.23356

Giordano S, 2012, PLOS ONE, V7, DOI 10.1371/journal.pone.0044610

Guo Y, 2019, FASEB J, V33, P12164, DOI 10.1096/fj.201900992R

Haas SJP, 2016, FRONT MOL NEUROSCI, V9, DOI 10.3389/fnmol.2016.00007

He Y, 2001, BRAIN RES, V909, P187, DOI 10.1016/S0006-8993(01)02681-6

Hjorth Erik, 2010, Int J Alzheimers Dis, V2010, DOI 10.4061/2010/798424

Hung HC, 2022, IMMUN INFLAMM DIS, V10, DOI 10.1002/iid3.610

Jadhav VS, 2014, J NEUROCHEM, V131, P803, DOI 10.1111/jnc.12933

Janabi N, 2002, J IMMUNOL, V168, P4747, DOI 10.4049/jimmunol.168.9.4747

JANABI N, 1995, NEUROSCI LETT, V195, P105, DOI 10.1016/0304-3940(94)11792-H

Jiang X, 2022, FRONT CELL NEUROSCI, V15, DOI 10.3389/fncel.2021.811061

Jurga AM, 2020, FRONT CELL NEUROSCI, V14, DOI 10.3389/fncel.2020.00198

Kenkhuis B, 2022, STEM CELL REP, V17, P1351, DOI 10.1016/j.stemcr.2022.04.006

Landry RP, 2012, EXP NEUROL, V234, P340, DOI 10.1016/j.expneurol.2011.11.006

Lee E, 2019, CELL DEATH DIFFER, V26, P213, DOI 10.1038/s41418-018-0124-5

Leyh J, 2021, FRONT CELL NEUROSCI, V15, DOI 10.3389/fncel.2021.701673

Li B, 2009, J INNATE IMMUN, V1, P570, DOI 10.1159/000235563

Li CX, 2019, CELL BIOL INT, V43, P360, DOI 10.1002/cbin.10975

Li M, 2012, J MOL NEUROSCI, V48, P225, DOI 10.1007/s12031-012-9814-5

Li T, 2013, BRAIN, V136, P3578, DOI 10.1093/brain/awt287

Li Y, 2021, BRAIN SCI, V11, DOI 10.3390/brainsci11070896

Liang YR, 2020, TOXICOL IN VITRO, V63, DOI 10.1016/j.tiv.2019.104690

Liang YR, 2015, BRAIN RES, V1622, P43, DOI 10.1016/j.brainres.2015.06.008

Liu B, 2003, ENVIRON HEALTH PERSP, V111, P1065, DOI 10.1289/ehp.6361

Liu SY, 2022, J NEUROINFLAMM, V19, DOI 10.1186/s12974-022-02574-z

Lively S, 2018, FRONT CELL NEUROSCI, V12, DOI 10.3389/fncel.2018.00215

LOWRY OH, 1951, J BIOL CHEM, V193, P265

Lv QK, 2021, J NEUROCHEM, V157, P599, DOI 10.1111/jnc.15299

Maegawa H, 2021, METHODS MOL BIOL, V2322, P95, DOI 10.1007/978-1-0716-1495-2_10

Massenzio F, 2018, BBA-MOL BASIS DIS, V1864, P3771, DOI 10.1016/j.bbadis.2018.10.013

Mildner A, 2017, GLIA, V65, P375, DOI 10.1002/glia.23097

Monzón-Sandoval J, 2022, DIS MODEL MECH, V15, DOI 10.1242/dmm.049349

Mount MP, 2007, J NEUROSCI, V27, P3328, DOI 10.1523/JNEUROSCI.5321-06.2007

Nareika A, 2009, ATHEROSCLEROSIS, V202, P363, DOI 10.1016/j.atherosclerosis.2008.05.043

Niranjan R, 2010, FREE RADICAL RES, V44, P1304, DOI 10.3109/10715762.2010.501080

Orihuela R, 2016, BRIT J PHARMACOL, V173, P649, DOI 10.1111/bph.13139

Pandya H, 2017, NAT NEUROSCI, V20, P753, DOI 10.1038/nn.4534

Parajuli B, 2012, J NEUROINFLAMM, V9, DOI 10.1186/1742-2094-9-268

Payne SH, 2015, TRENDS BIOCHEM SCI, V40, P1, DOI 10.1016/j.tibs.2014.10.010

Prati F, 2015, ANGEW CHEM INT EDIT, V54, P1578, DOI 10.1002/anie.201410456

Przedborski S, 2000, RESTOR NEUROL NEUROS, V16, P135

Puntambekar SS, 2022, MOL NEURODEGENER, V17, DOI 10.1186/s13024-022-00545-9

Qiao C, 2017, MOL NEUROBIOL, V54, P4292, DOI 10.1007/s12035-016-9980-5

Rai MA, 2020, RETROVIROLOGY, V17, DOI 10.1186/s12977-020-00544-y

Rangaraju S, 2018, FRONT IMMUNOL, V9, DOI 10.3389/fimmu.2018.00405

Ren MR, 2018, EXP NEUROL, V302, P205, DOI 10.1016/j.expneurol.2018.01.016

Ruan CS, 2022, FRONT CELL NEUROSCI, V16, DOI 10.3389/fncel.2022.902372

Rui WJ, 2020, INT J NEUROPSYCHOPH, V23, P762, DOI 10.1093/ijnp/pyaa060

Sáez JC, 2020, BBA-MOL CELL RES, V1867, DOI 10.1016/j.bbamcr.2020.118720

Saha RN, 2006, ANTIOXID REDOX SIGN, V8, P929, DOI 10.1089/ars.2006.8.929

Sakurai H, 2003, J BIOL CHEM, V278, P36916, DOI 10.1074/jbc.M301598200

Scalzo P, 2009, J NEUROIMMUNOL, V216, P122, DOI 10.1016/j.jneuroim.2009.08.001

Seok J, 2013, P NATL ACAD SCI USA, V110, P3507, DOI 10.1073/pnas.1222878110

Shen YG, 2017, NAT COMMUN, V8, DOI 10.1038/s41467-017-00707-0

Sierra A, 2014, PLOS ONE, V9, DOI 10.1371/journal.pone.0106048

Smith AM, 2014, TRENDS NEUROSCI, V37, P125, DOI 10.1016/j.tins.2013.12.001

Smith AM, 2013, J NEUROINFLAMM, V10, DOI 10.1186/1742-2094-10-85

Smith AM, 2013, GLIA, V61, P929, DOI 10.1002/glia.22486

Sousa C, 2017, FRONT IMMUNOL, V8, DOI 10.3389/fimmu.2017.00198

Staal J, 2011, CELL RES, V21, P40, DOI 10.1038/cr.2010.168

Suzuki M, 2003, INFECT IMMUN, V71, P3503, DOI 10.1128/IAI.71.6.3503-3511.2003

Tanaka S, 2013, J NEUROINFLAMM, V10, DOI 10.1186/1742-2094-10-143

Tarabin V, 2004, N-S ARCH PHARMACOL, V369, P563, DOI 10.1007/s00210-004-0938-1

Thawkar BS, 2019, J NEUROIMMUNOL, V326, P62, DOI 10.1016/j.jneuroim.2018.11.010

Ta TT, 2019, P NATL ACAD SCI USA, V116, P4637, DOI 10.1073/pnas.1813562116

Vargas-Maya NI, 2021, J MICROBIOL METH, V187, DOI 10.1016/j.mimet.2021.106260

Wang Y, 2022, CELL DEATH DIS, V13, DOI 10.1038/s41419-022-05193-x

Wang YJ, 2021, SCI REP-UK, V11, DOI 10.1038/s41598-021-85702-8

Watanabe M, 2016, SCI REP-UK, V6, DOI 10.1038/srep38387

Wu DC, 2002, J NEUROSCI, V22, P1763, DOI 10.1523/JNEUROSCI.22-05-01763.2002

Wu F, 2015, NEUROSCIENCE, V284, P900, DOI 10.1016/j.neuroscience.2014.11.004

Xu Y, 2015, BRAIN BEHAV IMMUN, V50, P298, DOI 10.1016/j.bbi.2015.07.015

Yan JQ, 2015, INT J CLIN EXP MED, V8, P9030

Yang L, 2020, NEUROREPORT, V31, P139, DOI 10.1097/WNR.0000000000001385

Yao K, 2018, EXP GERONTOL, V111, P86, DOI 10.1016/j.exger.2018.07.010

Ye XX, 2020, J NEUROINFLAMM, V17, DOI 10.1186/s12974-019-1644-8

Zhang CY, 2021, REDOX BIOL, V47, DOI 10.1016/j.redox.2021.102134

Zhang DD, 2021, J NEUROINFLAMM, V18, DOI 10.1186/s12974-020-02065-z

Zhang JQ, 2020, GLIA, V68, P2674, DOI 10.1002/glia.23878

Zhang P, 2017, NEUROCHEM RES, V42, P1524, DOI 10.1007/s11064-017-2209-9

Zhang Q, 2020, MOLECULES, V25, DOI 10.3390/molecules25194359

Zhang X, 2015, CELL PHYSIOL BIOCHEM, V35, P1571, DOI 10.1159/000373972

Zhang YW, 2018, BIOCHEM BIOPH RES CO, V499, P797, DOI 10.1016/j.bbrc.2018.03.226

Zhou T, 2017, FRONT NEUROANAT, V11, DOI 10.3389/fnana.2017.00077

NR 118

TC 4

Z9 4

U1 0

U2 7

PU FRONTIERS MEDIA SA

PI LAUSANNE

PA AVENUE DU TRIBUNAL FEDERAL 34, LAUSANNE, CH-1015, SWITZERLAND

EI 1662-5102

J9 FRONT CELL NEUROSCI

JI Front. Cell. Neurosci.

PD NOV 29

PY 2022

VL 16

AR 1038721

DI 10.3389/fncel.2022.1038721

PG 13

WC Neurosciences

WE Science Citation Index Expanded (SCI-EXPANDED)

SC Neurosciences & Neurology

GA 7A1VY

UT WOS:000898253000001

PM 36523814

OA gold, Green Published

DA 2024-08-12

ER

PT J

AU Nolan, YM

Sullivan, AM

Toulouse, A

AF Nolan, Yvonne M.

Sullivan, Aideen M.

Toulouse, Andre

TI Parkinson's disease in the nuclear age of neuroinflammation

SO TRENDS IN MOLECULAR MEDICINE

LA English

DT Review

DE Parkinson's disease; inflammation; nuclear receptor; Nurr1; PPAR;

retinoic acid receptor; glucocorticoid receptor

ID PROLIFERATOR-ACTIVATED RECEPTORS; PPAR-GAMMA AGONIST; RETINOIC ACID

RECEPTORS; NF-KAPPA-B; DOPAMINERGIC-NEURONS; SUBSTANTIA-NIGRA;

TNF-ALPHA; MOUSE MODEL; MESSENGER-RNA; RAT-BRAIN

AB Chronic neuroinflammation is associated with the pathophysiology of Parkinson's disease, a movement disorder characterised by deterioration of the nigrostriatal system of the brain. Recent studies have yielded important insights into the regulation of inflammation by nuclear receptors, a superfamily of ligand-activated transcription factors. Certain nuclear receptors are also emerging as regulators of neurodegeneration, including the degeneration of dopaminergic neurons in Parkinson's disease, and the importance of transcriptional control in this process is becoming increasingly apparent. Here, we discuss the role of Nurr1, peroxisome proliferator-activated receptors (PPARs), retinoic acid receptors, and glucocorticoid receptors in neuroinflammatory processes that contribute to dopaminergic neuronal degeneration. We examine current evidence providing insight into the potential of these important players as therapeutic targets for Parkinson's disease.

C1 [Nolan, Yvonne M.; Sullivan, Aideen M.; Toulouse, Andre] Natl Univ Ireland Univ Coll Cork, Dept Anat & Neurosci, Cork, Ireland.

C3 University College Cork

RP Nolan, YM (corresponding author), Natl Univ Ireland Univ Coll Cork, Dept Anat & Neurosci, Cork, Ireland.

EM y.nolan@ucc.ie

RI Toulouse, Andre/A-3159-2009

OI Sullivan, Aideen/0000-0001-9692-6438; Nolan, Yvonne/0000-0003-2426-234X;

Toulouse, Andre/0000-0002-4045-8265

CR AKIYAMA H, 1989, BRAIN RES, V489, P247, DOI 10.1016/0006-8993(89)90857-3

Andersen JK, 2004, NAT MED, V10, pS18, DOI 10.1038/nrn1434

Baba Y, 2005, PARKINSONISM RELAT D, V11, P493, DOI 10.1016/j.parkreldis.2005.07.005

Bartels AL, 2007, MOVEMENT DISORD, V22, P1852, DOI 10.1002/mds.21552

Bas J, 2001, J NEUROIMMUNOL, V113, P146, DOI 10.1016/S0165-5728(00)00422-7

Blanquart C, 2003, J STEROID BIOCHEM, V85, P267, DOI 10.1016/S0960-0760(03)00214-0

Bossers K, 2010, BRAIN, V133, P3699, DOI 10.1093/brain/awq258

Braak H, 2003, NEUROBIOL AGING, V24, P197, DOI 10.1016/S0197-4580(02)00065-9

Brochard V, 2009, J CLIN INVEST, V119, P182, DOI 10.1172/JCI36470

Brodacki B, 2008, NEUROSCI LETT, V441, P158, DOI 10.1016/j.neulet.2008.06.040

Carta AR, 2011, NEUROSCIENCE, V194, P250, DOI 10.1016/j.neuroscience.2011.07.046

Carta AR, 2011, PARKINSONS DIS-US, V2011, DOI 10.4061/2011/689181

Castaño A, 2002, J NEUROCHEM, V81, P150, DOI 10.1046/j.1471-4159.2002.00799.x

Chattopadhyay N, 2001, MOL BRAIN RES, V92, P172, DOI 10.1016/S0169-328X(01)00155-3

Chen H, 2008, AM J EPIDEMIOL, V167, P90, DOI 10.1093/aje/kwm260

Choi WH, 2005, BIOCHEM BIOPH RES CO, V329, P125, DOI 10.1016/j.bbrc.2005.01.110

Ferrari CC, 2006, NEUROBIOL DIS, V24, P183, DOI 10.1016/j.nbd.2006.06.013

Clark RB, 2000, J IMMUNOL, V164, P1364, DOI 10.4049/jimmunol.164.3.1364

Collins LM, 2012, NEUROPHARMACOLOGY, V62, P2154, DOI 10.1016/j.neuropharm.2012.01.028

Cristiano L, 2001, J NEUROCYTOL, V30, P671, DOI 10.1023/A:1016525716209

Crotty S, 2008, EUR J NEUROSCI, V27, P294, DOI 10.1111/j.1460-9568.2007.06018.x

Cullingford TE, 1998, J NEUROCHEM, V70, P1366

Dehmer T, 2004, J NEUROCHEM, V88, P494, DOI 10.1046/j.1471-4159.2003.02210.x

DEXTER DT, 1989, J NEUROCHEM, V52, P1830, DOI 10.1111/j.1471-4159.1989.tb07264.x

Dheen ST, 2005, GLIA, V50, P21, DOI 10.1002/glia.20153

Dobbs RJ, 1999, ACTA NEUROL SCAND, V100, P34, DOI 10.1111/j.1600-0404.1999.tb00721.x

Fan XL, 2009, NEUROIMMUNOMODULAT, V16, P162, DOI 10.1159/000204229

FISZER U, 1994, J NEUROL SCI, V121, P39, DOI 10.1016/0022-510X(94)90154-6

Galter D, 2003, NEUROBIOL DIS, V14, P637, DOI 10.1016/j.nbd.2003.09.001

Gao XA, 2011, NEUROLOGY, V76, P863, DOI 10.1212/WNL.0b013e31820f2d79

Garrido-Gil P, 2012, J NEUROINFLAMM, V9, DOI 10.1186/1742-2094-9-38

Gelb DJ, 1999, ARCH NEUROL-CHICAGO, V56, P33, DOI 10.1001/archneur.56.1.33

Gilbert F, 2006, BIOL PSYCHIAT, V60, P538, DOI 10.1016/j.biopsych.2006.04.023

Hermanson E, 2003, EXP CELL RES, V288, P324, DOI 10.1016/S0014-4827(03)00216-7

Hirsch EC, 2009, LANCET NEUROL, V8, P382, DOI 10.1016/S1474-4422(09)70062-6

Hucke S, 2012, BRAIN, V135, P1586, DOI 10.1093/brain/aws058

Hunter RL, 2008, NEUROSCI LETT, V432, P198, DOI 10.1016/j.neulet.2007.12.019

Hutson CB, 2011, J NEUROTRAUM, V28, P1783, DOI 10.1089/neu.2010.1723

Iwashita A, 2007, J PHARMACOL EXP THER, V320, P1087, DOI 10.1124/jpet.106.115758

Jacobs FMJ, 2007, DEVELOPMENT, V134, P2673, DOI 10.1242/dev.02865

Jacobs FMJ, 2011, DEVELOPMENT, V138, P5213, DOI 10.1242/dev.071704

Jankovic J, 2012, CURR OPIN NEUROL, V25, P433, DOI 10.1097/WCO.0b013e3283542fc2

Jenner P, 2003, ANN NEUROL, V53, pS26, DOI 10.1002/ana.10483

Katsuki H, 2009, J NEUROCHEM, V110, P707, DOI 10.1111/j.1471-4159.2009.06171.x

Kim HJ, 2011, BBA-MOL BASIS DIS, V1812, P1, DOI 10.1016/j.bbadis.2010.08.006

Kim JH, 2002, NATURE, V418, P50, DOI 10.1038/nature00900

Kim WG, 2000, J NEUROSCI, V20, P6309, DOI 10.1523/JNEUROSCI.20-16-06309.2000

Klotz L, 2007, J NEUROIMMUNOL, V190, P34, DOI 10.1016/j.jneuroim.2007.07.017

Knott C, 2000, MOL CELL NEUROSCI, V16, P724, DOI 10.1006/mcne.2000.0914

Kreisler A, 2007, BRAIN RES, V1135, P77, DOI 10.1016/j.brainres.2006.12.011

Krey G, 1997, MOL ENDOCRINOL, V11, P779, DOI 10.1210/me.11.6.779

Kurkowska-Jastrzebska I, 2004, INT IMMUNOPHARMACOL, V4, P1307, DOI 10.1016/j.intimp.2004.05.006

Laloux C, 2012, PHARMACOL RES, V65, P514, DOI 10.1016/j.phrs.2012.02.008

LAW SW, 1992, MOL ENDOCRINOL, V6, P2129, DOI 10.1210/me.6.12.2129

Le W, 1999, J NEUROCHEM, V73, P2218

Le WD, 2003, NAT GENET, V33, P85, DOI 10.1038/ng1066

Le WD, 2008, J NEUROL SCI, V273, P29, DOI 10.1016/j.jns.2008.06.007

Lee HS, 2010, STEM CELLS, V28, P501, DOI 10.1002/stem.294

Lehmann JM, 1997, J BIOL CHEM, V272, P3406, DOI 10.1074/jbc.272.6.3406

Lévesque D, 2007, TRENDS NEUROSCI, V30, P22, DOI 10.1016/j.tins.2006.11.006

Long-Smith CM, 2010, J NEUROIMMUNOL, V226, P20, DOI 10.1016/j.jneuroim.2010.05.030

Luo XG, 2010, NEUROCHEM RES, V35, P540, DOI 10.1007/s11064-009-0094-6

Ma YL, 2011, BRIT MED BULL, V100, P173, DOI 10.1093/bmb/ldr040

Maden M, 2007, NAT REV NEUROSCI, V8, P755, DOI 10.1038/nrn2212

Mahmoudi S, 2009, NEUROBIOL DIS, V36, P213, DOI 10.1016/j.nbd.2009.07.017

MCCAFFERY P, 1994, P NATL ACAD SCI USA, V91, P7772, DOI 10.1073/pnas.91.16.7772

Mccoy MK, 2006, J NEUROSCI, V26, P9365, DOI 10.1523/JNEUROSCI.1504-06.2006

McGeer PL, 2003, ANN NEUROL, V54, P599, DOI 10.1002/ana.10728

MCGEER PL, 1988, NEUROLOGY, V38, P1285, DOI 10.1212/WNL.38.8.1285

McMorrow JP, 2011, BIOCHEM SOC T, V39, P688, DOI 10.1042/BST0390688

Michalik L, 2006, PHARMACOL REV, V58, P726, DOI 10.1124/pr.58.4.5

Miller AM, 2011, neurodegenerative disease - PROCESSES, PREVENTION, PROTECTION AND MONITORING, P323

Moreno S, 2004, NEUROSCIENCE, V123, P131, DOI 10.1016/j.neuroscience.2003.08.064

Morimoto M, 1996, NEUROSCI RES, V26, P235, DOI 10.1016/S0168-0102(96)01105-4

Morris J G, 1978, Clin Exp Neurol, V15, P24

Nadeau S, 2003, J NEUROSCI, V23, P5536, DOI 10.1523/jneurosci.23-13-05536.2003

No H, 2010, NEUROCHEM INT, V56, P620, DOI 10.1016/j.neuint.2010.01.005

Olanow CW, 2009, ANN NEUROL, V66, P591, DOI 10.1002/ana.21778

Godoy MCP, 2008, BRAIN, V131, P1880, DOI 10.1093/brain/awn101

Reale M, 2009, BRAIN BEHAV IMMUN, V23, P55, DOI 10.1016/j.bbi.2008.07.003

Rentzos M, 2007, ACTA NEUROL SCAND, V116, P374, DOI 10.1111/j.1600-0404.2007.00894.x

Ros-Bernal F, 2011, P NATL ACAD SCI USA, V108, P6632, DOI 10.1073/pnas.1017820108

RUBERTE E, 1993, DEVELOPMENT, V118, P267

Sacchetti P, 2001, J NEUROCHEM, V76, P1565, DOI 10.1046/j.1471-4159.2001.00181.x

Sadeghian M, 2012, J NEUROIMMUNOL, V246, P69, DOI 10.1016/j.jneuroim.2012.03.010

Saijo K, 2009, CELL, V137, P47, DOI 10.1016/j.cell.2009.01.038

Sakurada K, 1999, DEVELOPMENT, V126, P4017

Saucedo-Cardenas O, 1998, P NATL ACAD SCI USA, V95, P4013, DOI 10.1073/pnas.95.7.4013

SCHEINMAN RI, 1995, MOL CELL BIOL, V15, P943

Schintu N, 2009, EUR J NEUROSCI, V29, P954, DOI 10.1111/j.1460-9568.2009.06657.x

Schweingruber N, 2012, J NEUROENDOCRINOL, V24, P174, DOI 10.1111/j.1365-2826.2011.02161.x

SIAN J, 1994, ANN NEUROL, V36, P348, DOI 10.1002/ana.410360305

Smith LK, 2008, EUR J NEUROSCI, V27, P2133, DOI 10.1111/j.1460-9568.2008.06177.x

Stone DK, 2009, ANTIOXID REDOX SIGN, V11, P2151, DOI [10.1089/ars.2009.2460, 10.1089/ARS.2009.2460]

Swanson CR, 2011, J NEUROINFLAMM, V8, DOI 10.1186/1742-2094-8-91

Toulouse A, 2008, PROG NEUROBIOL, V85, P376, DOI 10.1016/j.pneurobio.2008.05.003

Ulusoy GK, 2011, BRAIN RES BULL, V85, P380, DOI 10.1016/j.brainresbull.2011.05.001

van Neerven S, 2010, J NEUROIMMUNOL, V229, P169, DOI 10.1016/j.jneuroim.2010.08.005

Villar-Cheda B, 2012, NEUROBIOL AGING, V33, DOI 10.1016/j.neurobiolaging.2010.08.006

Wagner J, 1999, NAT BIOTECHNOL, V17, P653, DOI 10.1038/10862

Wang GM, 2011, NEUROSCI RES, V70, P230, DOI 10.1016/j.neures.2011.02.001

Wirdefeldt K, 2011, EUR J EPIDEMIOL, V26, pS1, DOI 10.1007/s10654-011-9581-6

Woster AP, 2007, J NEUROCHEM, V103, P67, DOI 10.1111/j.1471-4159.2007.04706.x

Xu HH, 2006, J NEUROIMMUNOL, V171, P135, DOI 10.1016/j.jneuroim.2005.10.004

Xu JH, 2005, J NEUROSCI RES, V81, P403, DOI 10.1002/jnr.20518

Zetterstrom RH, 1996, MOL BRAIN RES, V41, P111, DOI 10.1016/0169-328X(96)00074-5

Zetterstrom RH, 1997, SCIENCE, V276, P248, DOI 10.1126/science.276.5310.248

Zhang LF, 2012, NEUROBIOL AGING, V33, DOI 10.1016/j.neurobiolaging.2011.03.022

Zhang S, 2011, J NEUROINFLAMM, V8, DOI 10.1186/1742-2094-8-154

Zhou F, 2007, NEUROPSYCHOPHARMACOL, V32, P2570, DOI 10.1038/sj.npp.1301381

NR 110

TC 94

Z9 104

U1 0

U2 21

PU ELSEVIER SCI LTD

PI OXFORD

PA THE BOULEVARD, LANGFORD LANE, KIDLINGTON, OXFORD OX5 1GB, OXON, United Kingdom

SN 1471-4914

EI 1471-499X

J9 TRENDS MOL MED

JI Trends Mol. Med

PD MAR

PY 2013

VL 19

IS 3

BP 187

EP 196

DI 10.1016/j.molmed.2012.12.003

PG 10

WC Biochemistry & Molecular Biology; Cell Biology; Medicine, Research &

Experimental

WE Science Citation Index Expanded (SCI-EXPANDED)

SC Biochemistry & Molecular Biology; Cell Biology; Research & Experimental

Medicine

GA 114YE

UT WOS:000316778100006

PM 23318001

DA 2024-08-12

ER

PT J

AU Abd Wahab, DYA

Gau, CH

Zakaria, R

Karuppan, MKM

A-rahbi, BS

Abdullah, Z

Alrafiah, A

Abdullah, JM

Muthuraju, S

AF Abd Wahab, Dayang Yasmin Abg

Gau, Chuang Huei

Zakaria, Rahimah

Karuppan, Mohan Kumar Muthu

A-rahbi, Badriya S.

Abdullah, Zuraidah

Alrafiah, Aziza

Abdullah, Jafri Malin

Muthuraju, Sangu

TI Review on Cross Talk between Neurotransmitters and Neuroinflammation in

Striatum and Cerebellum in the Mediation of Motor Behaviour

SO BIOMED RESEARCH INTERNATIONAL

LA English

DT Review

ID TUMOR-NECROSIS-FACTOR; THERAPEUTIC ROLE; INFLAMMATION; RECEPTORS; GABA;

INVOLVEMENT; BUSPIRONE; SEROTONIN; DEFICITS; AGONIST

AB Neurological diseases particularly Alzheimer's disease (AD), Parkinson's disease (PD), stroke, and epilepsy are on the rise all around the world causing morbidity and mortality globally with a common symptom of gradual loss or impairment of motor behaviour. Striatum, which is a component of the basal ganglia, is involved in facilitating voluntary movement while the cerebellum is involved in the maintenance of balance and coordination of voluntary movements. Dopamine, serotonin, gamma-aminobutyric acid (GABA), and glutamate, to name a few, interact in regulating the excitation and inhibition of motor neurons. In another hand, interestingly, the motor loss associated with neurological diseases is possibly resulted from neuroinflammation induced by the neuroimmune system. Toll-like receptors (TLRs) are present in the central nervous system (CNS), specifically and primarily expressed in microglia and are also found on neurons and astrocytes, functioning mainly in the regulation of proinflammatory cytokine production. TLRs are always found to be associated or involved in the induction of neuroinflammation in neurodegenerative disease. Activation of toll-like receptor 4 (TLR4) through TLR4 agonist, lipopolysaccharide (LPS), stimulation initiate a signaling cascade whereby the TLR4-LPS interaction has been found to result in physiological and behavioural changes including retardation of motor activity in the mouse model. TLR4 inhibitor TAK-242 was reflected in the reduction of the spinal cord pathology along with the motor improvement in ALS mouse. There is cross talk with neuroinflammation and neurochemicals. For example, TLR4 activation by LPS is noted to release proinflammatory cytokines, IL-1 beta, from microglia that subsequently suppresses GABA receptor activities at the postsynaptic site and reduces GABA synthesis at the presynaptic site. Glial glutamate transporter activities are also found to be suppressed, showing the association between TLR4 activation and the related neurotransmitters and corresponding receptors and transporters in the event of neuroinflammation. This review is helpful to understand the connection between neurotransmitter and neuroinflammation in striatum- and cerebellum-mediated motor behaviour.

C1 [Abd Wahab, Dayang Yasmin Abg; Gau, Chuang Huei; Abdullah, Jafri Malin; Muthuraju, Sangu] Univ Sains Malaysia, Jalan Hosp, Dept Neurosci & Brain & Behav Cluster, Sch Med Sci, Kota Baharu 16150, Kelantan, Malaysia.

[Zakaria, Rahimah] Univ Sains Malaysia, Jalan Hosp, Dept Physiol, Sch Med Sci, Kota Baharu 16150, Kelantan, Malaysia.

[Karuppan, Mohan Kumar Muthu] Florida Int Univ, Herbert Wertheim Coll Med, Dept Immunol & Nanomed, 11200 SW 8th St, Miami, FL 33199 USA.

[A-rahbi, Badriya S.] Oman Coll Hlth Sci, Muscat, Oman.

[Abdullah, Zuraidah] Univ Sains Malaysia, Sch Hlth Sci, Dept Biomed Sci, Jalan Hosp, Kota Baharu 16150, Kelantan, Malaysia.

[Alrafiah, Aziza] King Abdulaziz Univ, Fac Appl Med Sci, Dept Med Lab Technol, Jeddah, Saudi Arabia.

[Muthuraju, Sangu] Univ Houston, Coll Pharm, Dept Pharmacol & Pharmaceut Sci, Houston, TX 77240 USA.

C3 Universiti Sains Malaysia; Universiti Sains Malaysia; State University

System of Florida; Florida International University; Universiti Sains

Malaysia; King Abdulaziz University; University of Houston System;

University of Houston

RP Muthuraju, S (corresponding author), Univ Sains Malaysia, Jalan Hosp, Dept Neurosci & Brain & Behav Cluster, Sch Med Sci, Kota Baharu 16150, Kelantan, Malaysia.; Muthuraju, S (corresponding author), Univ Houston, Coll Pharm, Dept Pharmacol & Pharmaceut Sci, Houston, TX 77240 USA.

EM dyg_yasmin@hotmail.com; gauchuang0310@hotmail.com; rahimah@usm.my;

mmuthuka@fiu.edu; drbadriya.bsc.ihs@gmail.com; zuraidahabdullah@usm.my;

aalrafiah@kau.edu.sa; brainsciences@gmail.com; muthuraju67@gmail.com

RI Zakaria, Rahimah/C-8660-2016; SANGU, MUTHURAJU/C-3200-2011; SANGU,

MUTHURAJU/AAY-8060-2020; Alrafiah, Aziza/CAI-2526-2022; Abdullah, Jafri

Malin/C-2828-2011; ALRAFIAH, AZIZA/R-2504-2018

OI Zakaria, Rahimah/0000-0002-2459-3213; SANGU,

MUTHURAJU/0000-0002-7949-2110; Abdullah, Jafri

Malin/0000-0002-0258-7410; ALRAFIAH, AZIZA/0000-0003-0978-5775

FU School of Medical Sciences, Pusat Pengajian Sains Perubatan (PPSP),

Universiti Sains Malaysia [304/PPSP/61313158]

FX The authors thank the School of Medical Sciences, Pusat Pengajian Sains

Perubatan (PPSP), Universiti Sains Malaysia. This work was supported by

project no. 304/PPSP/61313158 from the short term grant. Also, the

authors thank the Director of the Hospital Universiti Sains Malaysia

(USM), Kubang Kerian, Kelantan, for granting permission to the

investigators to use the space and instruments belonging to the USM for

conducting the research.

CR Bak LK, 2006, J NEUROCHEM, V98, P641, DOI 10.1111/j.1471-4159.2006.03913.x

Benarroch EE, 2010, NEUROLOGY, V74, P259, DOI 10.1212/WNL.0b013e3181cc89e3

Bezard E, 2013, MOVEMENT DISORD, V28, P1088, DOI 10.1002/mds.25366

BOKA G, 1994, NEUROSCI LETT, V172, P151, DOI 10.1016/0304-3940(94)90684-X

Chaudhry FA, 2002, J CELL BIOL, V157, P349, DOI 10.1083/jcb.200201070

Chen WW, 2016, MOL MED REP, V13, P3391, DOI 10.3892/mmr.2016.4948

Chen XY, 2013, NEUROSCI BULL, V29, P701, DOI 10.1007/s12264-013-1351-x

Chiu CS, 2005, J NEUROSCI, V25, P3234, DOI 10.1523/JNEUROSCI.3364-04.2005

Ciranna L, 2006, CURR NEUROPHARMACOL, V4, P101, DOI 10.2174/157015906776359540

Costa JP, 2014, BRAIN RES, V1547, P34, DOI 10.1016/j.brainres.2013.12.003

Crews FT, 2016, PSYCHOPHARMACOLOGY, V233, P1543, DOI 10.1007/s00213-015-3906-1

Dadsetan S, 2016, J NEUROINFLAMM, V13, DOI 10.1186/s12974-016-0710-8

Danbolt NC, 2001, PROG NEUROBIOL, V65, P1, DOI 10.1016/S0301-0082(00)00067-8

Dürk T, 2005, INT IMMUNOL, V17, P599, DOI 10.1093/intimm/dxh242

Fan LW, 2011, BRAIN BEHAV IMMUN, V25, P286, DOI 10.1016/j.bbi.2010.09.020

Feigin VL, 2017, LANCET NEUROL, V16, P877, DOI 10.1016/S1474-4422(17)30299-5

Feng Y, 2017, CELL MOL NEUROBIOL, V37, P155, DOI 10.1007/s10571-016-0356-1

Glass CK, 2010, CELL, V140, P918, DOI 10.1016/j.cell.2010.02.016

Hanchar HJ, 2005, NAT NEUROSCI, V8, P339, DOI 10.1038/nn1398

Hernandez-Rabaza V, 2016, J NEUROINFLAMM, V13, DOI 10.1186/s12974-016-0549-z

Hines DJ, 2013, PLOS ONE, V8, DOI 10.1371/journal.pone.0060388

Hoxha Eriola, 2016, Front Synaptic Neurosci, V8, P35

Iyer SS, 2012, CRIT REV IMMUNOL, V32, P23, DOI 10.1615/CritRevImmunol.v32.i1.30

Karamanolis GP, 2016, ARTHRITIS RES THER, V18, DOI 10.1186/s13075-016-1094-y

Kawasaki T, 2014, FRONT IMMUNOL, V5, DOI 10.3389/fimmu.2014.00461

Kolodziejczak M, 2015, ACS CHEM NEUROSCI, V6, P1219, DOI 10.1021/cn5003489

Kravitz AV, 2012, NAT NEUROSCI, V15, P816, DOI 10.1038/nn.3100

Krzyszton CP, 2008, AM J PHYSIOL-REG I, V295, pR1109, DOI 10.1152/ajpregu.90302.2008

Le Marec N, 2001, PHARMACOL BIOCHEM BE, V69, P333, DOI 10.1016/S0091-3057(01)00536-6

Leisman G, 2013, REV NEUROSCIENCE, V24, P9, DOI 10.1515/revneuro-2012-0067

Leite AZ, 2017, FRONT IMMUNOL, V8, DOI 10.3389/fimmu.2017.01107

Lim JE, 2012, BEHAV BRAIN RES, V227, P36, DOI 10.1016/j.bbr.2011.10.027

Miller AH, 2014, PLOS ONE, V9, DOI 10.1371/journal.pone.0098156

Molteni M, 2016, MEDIAT INFLAMM, V2016, DOI 10.1155/2016/6978936

Montoliu C., 2015, Neuroimmunology and Neuroinflammation, V2, P138, DOI DOI 10.4103/2347-8659.160845

Mormina E, 2017, WORLD J RADIOL, V9, P371, DOI 10.4329/wjr.v9.i10.371

Nakamura T, 2014, FRONT INTEGR NEUROSC, V8, DOI 10.3389/fnint.2014.00056

Nau F, 2013, PLOS ONE, V8, DOI 10.1371/journal.pone.0075426

Ohno Y, 2015, PROG NEUROBIOL, V134, P104, DOI 10.1016/j.pneurobio.2015.09.005

Ohno Y, 2011, CNS NEUROSCI THER, V17, P58, DOI 10.1111/j.1755-5949.2010.00211.x

Owens DF, 2002, NAT REV NEUROSCI, V3, P715, DOI 10.1038/nrn919

Perea G, 2009, TRENDS NEUROSCI, V32, P421, DOI 10.1016/j.tins.2009.05.001

Qin LY, 2007, GLIA, V55, P453, DOI 10.1002/glia.20467

Rietdijk CD., 2016, Neuroimmunol. Neuroinflamm, V3, P27, DOI [10.20517/2347-8659.2015.28, DOI 10.20517/2347-8659.2015.28]

Salman MS, 2016, NEUROIMAG CLIN N AM, V26, P317, DOI 10.1016/j.nic.2016.03.003

Santos M, 2010, NEUROSCIENCE, V170, P453, DOI 10.1016/j.neuroscience.2010.07.010

Serafini G, 2013, EUR NEUROPSYCHOPHARM, V23, P1672, DOI 10.1016/j.euroneuro.2013.06.002

Shabab T, 2017, INT J NEUROSCI, V127, P624, DOI 10.1080/00207454.2016.1212854

Shmuel-Galia L, 2017, J BIOL CHEM, V292, P13415, DOI 10.1074/jbc.M117.784983

Sominsky L, 2015, FRONT NEUROSCI-SWITZ, V9, DOI 10.3389/fnins.2015.00201

Subramaniam SR, 2017, FRONT AGING NEUROSCI, V9, DOI 10.3389/fnagi.2017.00176

Tan RH, 2014, PLOS ONE, V9, DOI 10.1371/journal.pone.0105632

Tarr AJ, 2012, BEHAV BRAIN RES, V235, P334, DOI 10.1016/j.bbr.2012.07.038

Triarhou LC., 2000, Madame Curie Bioscience Database

Trouillas P, 1997, PROG BRAIN RES, V114, P589

Tsai MC, 2012, J NEUROSCI, V32, P1528, DOI 10.1523/JNEUROSCI.5232-11.2012

Vanover KE, 2008, PHARMACOL BIOCHEM BE, V90, P540, DOI 10.1016/j.pbb.2008.04.010

Venezia S, 2017, MOL NEURODEGENER, V12, DOI 10.1186/s13024-017-0195-7

Wagner MJ, 2017, NATURE, V544, P96, DOI 10.1038/nature21726

Wirths O, 2008, GENES BRAIN BEHAV, V7, P1, DOI 10.1111/j.1601-183X.2007.00373.x

Wu T, 2013, BRAIN, V136, P696, DOI 10.1093/brain/aws360

YAN X, 2015, J NEUROINFLAMM, V12, P1, DOI DOI 10.1186/s12974-014-0222-3

Yang HM, 2017, FRONT AGING NEUROSCI, V9, DOI 10.3389/fnagi.2017.00193

Yu BN, 2008, J PHARMACOL EXP THER, V327, P316, DOI 10.1124/jpet.108.143461

Zhang YL, 2016, NEURAL PLAST, V2016, DOI 10.1155/2016/8941327

Zhu JW, 2016, FRONT NEUROSCI-SWITZ, V10, DOI 10.3389/fnins.2016.00033

NR 66

TC 52

Z9 55

U1 1

U2 12

PU HINDAWI LTD

PI LONDON

PA ADAM HOUSE, 3RD FLR, 1 FITZROY SQ, LONDON, W1T 5HF, United Kingdom

SN 2314-6133

EI 2314-6141

J9 BIOMED RES INT-UK

JI Biomed Res. Int.

PD NOV 14

PY 2019

VL 2019

AR 1767203

DI 10.1155/2019/1767203

PG 10

WC Biotechnology & Applied Microbiology; Medicine, Research & Experimental

WE Science Citation Index Expanded (SCI-EXPANDED)

SC Biotechnology & Applied Microbiology; Research & Experimental Medicine

GA JQ7VZ

UT WOS:000499149600006

PM 31815123

OA Green Published, gold

DA 2024-08-12

ER

PT J

AU Shen, S

Zhang, C

Xu, YM

Shi, CH

AF Shen, Si

Zhang, Chan

Xu, Yu-Ming

Shi, Chang-He

TI The Role of Pathogens and Anti-Infective Agents in Parkinson's Disease,

from Etiology to Therapeutic Implications

SO JOURNAL OF PARKINSONS DISEASE

LA English

DT Review

DE Parkinson's disease; infection; gut microbiota; neuroinflammation; drug

repurposing

ID HEPATITIS-C VIRUS; JAPANESE ENCEPHALITIS PATIENTS; HELICOBACTER-PYLORI

INFECTION; ALPHA-SYNUCLEIN EXPRESSION; CENTRAL-NERVOUS-SYSTEM; H5N1

INFLUENZA-VIRUS; RAT MODEL; MICROGLIAL ACTIVATION; RISK-FACTOR; L-DOPA

AB Parkinson's disease is a debilitating neurodegenerative disorder whose etiology is still unclear, hampering the development of effective treatments. There is an urgent need to identify the etiology and provide further effective treatments. Recently, accumulating evidence has indicated that infection may play a role in the etiology of Parkinson's disease. The infective pathogens may act as a trigger for Parkinson's disease, the most common of which are hepatitis C virus, influenza virus, and Helicobacter pylori. In addition, gut microbiota is increasingly recognized to influence brain function through the gut-brain axis, showing an important role in the pathogenesis of Parkinson's disease. Furthermore, a series of anti-infective agents exhibit surprising neuroprotective effects via various mechanisms, such as interfering with alpha-synuclein aggregation, inhibiting neuroinflammation, attenuating oxidative stress, and preventing from cell death, independent of their antimicrobial effects. The pleiotropic agents affect important events in the pathogenesis of Parkinson's disease. Moreover, most of them are less toxic, clinically safe and have good blood-brain penetrability, making them hopeful candidates for the treatment of Parkinson's disease. However, the use of antibiotics and subsequent gut dysbiosis may also play a role in Parkinson's disease, making the long-term effects of anti-infective drugs worthy of further consideration and exploration. This review summarizes the current evidence for the association between infective pathogens and Parkinson's disease and subsequently explores the application prospects of anti-infective drugs in Parkinson's disease treatment, providing novel insights into the pathogenesis and treatment of Parkinson's disease.

C1 [Shen, Si; Zhang, Chan; Xu, Yu-Ming; Shi, Chang-He] Zhengzhou Univ, Affiliated Hosp 1, Dept Neurol, 1 Jian She East Rd, Zhengzhou 450000, Henan, China.

[Zhang, Chan; Xu, Yu-Ming; Shi, Chang-He] Zhengzhou Univ, Affiliated Hosp 1, Henan Key Lab Cerebrovasc Dis, Zhengzhou, Henan, China.

[Xu, Yu-Ming; Shi, Chang-He] Zhengzhou Univ, Inst Neurosci, Zhengzhou, Henan, China.

C3 Zhengzhou University; Zhengzhou University; Zhengzhou University

RP Xu, YM; Shi, CH (corresponding author), Zhengzhou Univ, Affiliated Hosp 1, Dept Neurol, 1 Jian She East Rd, Zhengzhou 450000, Henan, China.

EM xuyuming@zzu.edu.cn; shichanghe@gmail.com

RI xu, yuming/KRP-2493-2024; Liu, Donghua/KEJ-1974-2024; Li,

Wenjuan/KDN-8450-2024

OI Liu, Donghua/0000-0002-5830-9540;

FU National Natural Science Foundation of China [81974211, 81771290,

82171247, U1904207, 91849115]; National Key R&D Program of China

[2017YFA0105000]; Non-profit Central Research Institute Fund of Chinese

Academy of Medical Sciences [2020-PT310-01]

FX This work was supported by the National Natural Science Foundation of

China to Dr. Chang-he Shi [grant number 81974211, 81771290, 82171247];

the National Key R&D Program of China to Dr. Yu-ming Xu

[2017YFA0105000]; the National Natural Science Foundation of China to

Dr. Yu-ming Xu [grant numbers U1904207, 91849115] and the Non-profit

Central Research Institute Fund of Chinese Academy of Medical Sciences

[2020-PT310-01].

CR Acuña L, 2019, CELLS-BASEL, V8, DOI 10.3390/cells8080776

Aho VTE, 2019, EBIOMEDICINE, V44, P691, DOI 10.1016/j.ebiom.2019.05.064

Appelmelk BJ, 1997, TRENDS MICROBIOL, V5, P70, DOI 10.1016/S0966-842X(96)10084-6

Ascherio A, 2016, LANCET NEUROL, V15, P1255, DOI 10.1016/S1474-4422(16)30230-7

Auluck PK, 2002, NAT MED, V8, P1185, DOI 10.1038/nm1102-1185

BAILEY EV, 1975, ARCH INT PHARMACOD T, V216, P246

Bantle CM, 2021, EXP NEUROL, V346, DOI 10.1016/j.expneurol.2021.113845

Bantle CM, 2019, NPJ PARKINSON DIS, V5, DOI 10.1038/s41531-019-0090-8

Barini E, 2018, CHEMBIOCHEM, V19, P425, DOI 10.1002/cbic.201700500

Beatman EL, 2016, J VIROL, V90, P2767, DOI 10.1128/JVI.02949-15

Bennett BA, 1995, BRAIN RES, V705, P168, DOI 10.1016/0006-8993(95)01166-8

Bi W, 2011, BRAIN RES, V1395, P12, DOI 10.1016/j.brainres.2011.04.019

Bisht R, 2014, NEUROTOXICOLOGY, V44, P71, DOI 10.1016/j.neuro.2014.05.009

Blanco-Palmero VA, 2021, J NEUROL, V268, P3116, DOI 10.1007/s00415-021-10444-6

BREW BJ, 1995, ANN NEUROL, V38, P563, DOI 10.1002/ana.410380404

Cain MD, 2019, NEURON, V103, P771, DOI 10.1016/j.neuron.2019.07.015

Cermelli C, 2003, EUR J EPIDEMIOL, V18, P123, DOI 10.1023/A:1023067728557

Cersosimo MG, 2013, J NEUROL, V260, P1332, DOI 10.1007/s00415-012-6801-2

Chan HYE, 2000, HUM MOL GENET, V9, P2811, DOI 10.1093/hmg/9.19.2811

Charlett A, 1999, ACTA NEUROL SCAND, V99, P26

Chase TN, 2003, NEUROTOX RES, V5, P139, DOI 10.1007/BF03033378

Chen CK, 2018, INT J ENV RES PUB HE, V15, DOI 10.3390/ijerph15081587

Chen CK, 2017, PEERJ, V5, DOI 10.7717/peerj.3647

Chen SW, 2010, NEURAL REGEN RES, V5, P251, DOI 10.3969/j.issn.1673-5374.2010.04.002

Cheng CM, 2020, CNS SPECTRUMS, V25, P797, DOI 10.1017/S1092852919001664

Chhajed PN, 2006, RESPIRATION, V73, P367, DOI 10.1159/000087945

Cho Y, 2009, NEUROTOX RES, V16, P361, DOI 10.1007/s12640-009-9078-1

Choi HY, 2020, J VIRAL HEPATITIS, V27, P1171, DOI 10.1111/jvh.13346

Chotibut T, 2017, MOVEMENT DISORD, V32, P1547, DOI 10.1002/mds.27077

Chotibut T, 2014, MOL NEUROBIOL, V49, P1282, DOI 10.1007/s12035-013-8598-0

Cilia R, 2020, MOVEMENT DISORD, V35, P1287, DOI 10.1002/mds.28170

Cohen ME, 2020, LANCET NEUROL, V19, P804, DOI 10.1016/S1474-4422(20)30305-7

Crosbie EJ, 2013, LANCET, V382, P889, DOI 10.1016/S0140-6736(13)60022-7

Cummings CJ, 1998, NAT GENET, V19, P148, DOI 10.1038/502

Dardiotis E, 2018, CLIN NEUROL NEUROSUR, V175, P16, DOI 10.1016/j.clineuro.2018.09.039

Dauer W, 2003, NEURON, V39, P889, DOI 10.1016/S0896-6273(03)00568-3

Deretzi G, 2011, CURR MOL MED, V11, P696, DOI 10.2174/156652411797536660

Di Caprio R, 2015, MEDIAT INFLAMM, V2015, DOI 10.1155/2015/329418

Diguet E, 2004, EUR J NEUROSCI, V19, P3266, DOI 10.1111/j.0953-816X.2004.03372.x

Du YS, 2001, P NATL ACAD SCI USA, V98, P14669, DOI 10.1073/pnas.251341998

Einarsdóttir ER, 2009, SPEC CARE DENT, V29, P123, DOI 10.1111/j.1754-4505.2009.00075.x

Elfil M, 2020, MOVEMENT DISORD, V35, P921, DOI 10.1002/mds.28004

Elmer LW, 2018, CNS DRUGS, V32, P387, DOI 10.1007/s40263-018-0498-4

Eriksen JL, 2005, ARCH NEUROL-CHICAGO, V62, P353, DOI 10.1001/archneur.62.3.353

Faber I, 2020, MOVEMENT DISORD, V35, P1721, DOI 10.1002/mds.28277

Feng YH, 2019, PLOS ONE, V14, DOI 10.1371/journal.pone.0218384

Fletcher NF, 2012, GASTROENTEROLOGY, V142, P634, DOI 10.1053/j.gastro.2011.11.028

Fox SH, 2018, MOVEMENT DISORD, V33, P1248, DOI 10.1002/mds.27372

Gane SB, 2020, RHINOLOGY, V58, P299, DOI 10.4193/Rhin20.114

Gill K, 2016, HEPATOL INT, V10, P415, DOI 10.1007/s12072-015-9684-3

Golabi P, 2017, ANN HEPATOL, V16, P342, DOI 10.5604/16652681.1235476

Goldstein L, 2019, J NEUROL SCI, V398, P138, DOI 10.1016/j.jns.2019.01.012

González-Lizárraga F, 2017, SCI REP-UK, V7, DOI 10.1038/srep41755

Hamaue N, 2006, NEUROCHEM RES, V31, P1451, DOI 10.1007/s11064-006-9197-5

Hanaoka A, 2009, J CLIN NEUROSCI, V16, P1279, DOI 10.1016/j.jocn.2008.12.027

Harris MA, 2012, MOVEMENT DISORD, V27, P1111, DOI 10.1002/mds.25077

Hashim H, 2014, PLOS ONE, V9, DOI 10.1371/journal.pone.0112330

Hawkes CH, 2007, NEUROPATH APPL NEURO, V33, P599, DOI 10.1111/j.1365-2990.2007.00874.x

Heintz-Buschart A, 2018, MOVEMENT DISORD, V33, P88, DOI 10.1002/mds.27105

Ho SC, 2014, BEHAV BRAIN RES, V268, P177, DOI 10.1016/j.bbr.2014.04.022

Hsieh MH, 2017, BRAIN RES BULL, V132, P129, DOI 10.1016/j.brainresbull.2017.05.015

Hubsher G, 2012, NEUROLOGY, V78, P1096, DOI 10.1212/WNL.0b013e31824e8f0d

Jang H, 2012, J NEUROSCI, V32, P1545, DOI 10.1523/JNEUROSCI.5123-11.2012

Jang HM, 2009, P NATL ACAD SCI USA, V106, P14063, DOI 10.1073/pnas.0900096106

Jankovic J, 2020, J NEUROL NEUROSUR PS, V91, P795, DOI 10.1136/jnnp-2019-322338

Jiang JH, 2013, INT J MOL MED, V31, P825, DOI 10.3892/ijmm.2013.1280

Johnston O, 2008, J AM SOC NEPHROL, V19, P1411, DOI 10.1681/ASN.2007111202

Kalia LV, 2015, LANCET, V386, P896, DOI 10.1016/S0140-6736(14)61393-3

Kaur Baninder, 2017, Pathophysiology, V24, P71, DOI 10.1016/j.pathophys.2017.02.001

Kelly LP, 2014, MOVEMENT DISORD, V29, P999, DOI 10.1002/mds.25736

Khanlou N, 2009, J NEUROVIROL, V15, P131, DOI 10.1080/13550280802578075

Kilic Ü, 2004, ACTA NEUROPATHOL, V108, P65, DOI 10.1007/s00401-004-0867-6

Kim JM, 2016, MOVEMENT DISORD, V31, P1584, DOI 10.1002/mds.26755

Kountouras J, 2012, EUR J NEUROL, V19, pe56, DOI 10.1111/j.1468-1331.2012.03695.x

Koutsilieri E, 2002, J NEURAL TRANSM, V109, P767, DOI 10.1007/s007020200063

Lahner E, 2009, ALIMENT PHARM THER, V29, P379, DOI 10.1111/j.1365-2036.2008.03906.x

Lai SW, 2017, MEDICINE, V96, DOI [10.1097/MD.0000000000006075, 10.1097/md.0000000000006075]

Lazzarini M, 2013, GLIA, V61, P1084, DOI 10.1002/glia.22496

Li J, 2004, CHEM BIOL, V11, P1513, DOI 10.1016/j.chembiol.2004.08.025

Liang YR, 2020, TOXICOL IN VITRO, V63, DOI 10.1016/j.tiv.2019.104690

Liang YR, 2017, NEUROTOXICOLOGY, V63, P137, DOI 10.1016/j.neuro.2017.09.015

Limphaibool N, 2019, FRONT NEUROL, V10, DOI 10.3389/fneur.2019.00652

Lin CH, 2019, J NEUROINFLAMM, V16, DOI 10.1186/s12974-019-1528-y

Lin WY, 2019, JAMA NEUROL, V76, P1019, DOI 10.1001/jamaneurol.2019.1368

Liu KY, 2013, NEUROCHEM RES, V38, P201, DOI 10.1007/s11064-012-0909-8

Lo KC, 2003, VIRCHOWS ARCH, V442, P591, DOI 10.1007/s00428-003-0795-1

Lotharius J, 2002, NAT REV NEUROSCI, V3, P932, DOI 10.1038/nrn983

Malagelada C, 2010, J NEUROSCI, V30, P1166, DOI 10.1523/JNEUROSCI.3944-09.2010

Mao L, 2020, JAMA NEUROL, V77, P683, DOI 10.1001/jamaneurol.2020.1127

Marino BLB, 2020, MINI-REV MED CHEM, V20, P754, DOI 10.2174/1389557519666191104110908

Marreiros R, 2020, P NATL ACAD SCI USA, V117, P6741, DOI 10.1073/pnas.1906466117

Matheoud D, 2019, NATURE, V571, P565, DOI 10.1038/s41586-019-1405-y

McFarland NR, 2014, PLOS ONE, V9, DOI 10.1371/journal.pone.0086048

Mcgee DJ, 2018, J PARKINSON DIS, V8, P367, DOI 10.3233/JPD-181327

McLean PJ, 2004, BIOCHEM BIOPH RES CO, V321, P665, DOI 10.1016/j.bbrc.2004.07.021

Méndez-Guerrero A, 2020, NEUROLOGY, V95, pE2109, DOI 10.1212/WNL.0000000000010282

Mertsalmi TH, 2020, MOVEMENT DISORD, V35, P431, DOI 10.1002/mds.27924

Misra UK, 2005, NEUROCHEM RES, V30, P1075, DOI 10.1007/s11064-005-7414-2

Misra UK, 2002, POSTGRAD MED J, V78, P238, DOI 10.1136/pmj.78.918.238

Mlakar J, 2016, NEW ENGL J MED, V374, P951, DOI 10.1056/NEJMoa1600651

Müller T, 2019, EXPERT OPIN PHARMACO, V20, P1181, DOI 10.1080/14656566.2019.1612365

Müller T, 2011, SPEC CARE DENT, V31, P178, DOI 10.1111/j.1754-4505.2011.00205.x

NASTUK WL, 1976, NATURE, V264, P76, DOI 10.1038/264076a0

Negro F, 2015, GASTROENTEROLOGY, V149, P1345, DOI 10.1053/j.gastro.2015.08.035

Niehues M, 2009, J PHARM PHARMACOL, V61, P1303, DOI 10.1211/jpp/61.10.0005

Noto JM, 2017, PLOS PATHOG, V13, DOI 10.1371/journal.ppat.1006573

Nuzum Nathan D, 2020, Neurosci Biobehav Rev, V112, P227, DOI 10.1016/j.neubiorev.2020.02.003

Ogata A, 1997, J NEUROVIROL, V3, P141, DOI 10.3109/13550289709015803

Oida Y, 2006, BRAIN RES, V1082, P196, DOI 10.1016/j.brainres.2006.01.116

Pakpoor J, 2017, NEUROLOGY, V88, P1630, DOI [10.1212/WNL.0000000000003848, 10.1212/wnl.0000000000003848]

Panza F, 2019, BRAIN, V142, P2905, DOI 10.1093/brain/awz244

Pereira PAB, 2017, PARKINSONISM RELAT D, V38, P61, DOI 10.1016/j.parkreldis.2017.02.026

Pierantozzi M, 2006, NEUROLOGY, V66, P1824, DOI 10.1212/01.wnl.0000221672.01272.ba

Pierantozzi M, 2001, ANN NEUROL, V50, P686, DOI 10.1002/ana.1267

Puccini JM, 2015, J NEUROSCI, V35, P5271, DOI 10.1523/JNEUROSCI.0650-14.2015

Qian YW, 2018, BRAIN BEHAV IMMUN, V70, P194, DOI 10.1016/j.bbi.2018.02.016

Quintero EM, 2006, BRAIN RES, V1093, P198, DOI 10.1016/j.brainres.2006.03.104

Radkowski M, 2002, J VIROL, V76, P600, DOI 10.1128/JVI.76.2.600-608.2002

RAIL D, 1981, J NEUROL NEUROSUR PS, V44, P670, DOI 10.1136/jnnp.44.8.670

RAVENHOLT RT, 1982, LANCET, V2, P860

Reglodi D, 2017, PROG NEUROBIOL, V155, P120, DOI 10.1016/j.pneurobio.2015.10.004

Reichard RR, 2020, ACTA NEUROPATHOL, V140, P1, DOI 10.1007/s00401-020-02166-2

Rekdal VM, 2019, SCIENCE, V364, P1055, DOI 10.1126/science.aau6323

Rezak M, 2007, DM-DIS MON, V53, P259, DOI 10.1016/j.disamonth.2007.02.003

Robinson RL, 2003, AM J MED, V115, P252, DOI 10.1016/S0002-9343(03)00291-2

Roy U, 2015, FRONT MICROBIOL, V6, DOI 10.3389/fmicb.2015.00749

Ruzza P, 2014, ACS CHEM NEUROSCI, V5, P30, DOI 10.1021/cn400149k

Sadasivan S, 2017, NPJ PARKINSONS DIS, V3, DOI 10.1038/s41531-017-0019-z

Sampson TR, 2016, CELL, V167, P1469, DOI 10.1016/j.cell.2016.11.018

Santa-Cecília FV, 2016, NEUROTOX RES, V29, P447, DOI 10.1007/s12640-015-9592-2

Sardar AM, 1996, NEUROREPORT, V7, P910, DOI 10.1097/00001756-199603220-00015

Sawada H, 2010, PLOS ONE, V5, DOI 10.1371/journal.pone.0015298

SCHULTZ DR, 1977, NEUROLOGY, V27, P1095, DOI 10.1212/WNL.27.11.1095

SCHWAB R S, 1969, Transactions of the American Neurological Association, V94, P85

SCHWAB RS, 1969, J AMER MED ASSOC, V208, P1168, DOI 10.1001/jama.208.7.1168

Sejvar JJ, 2014, VIRUSES-BASEL, V6, P606, DOI 10.3390/v6020606

Sejvar JJ, 2003, JAMA-J AM MED ASSOC, V290, P511, DOI 10.1001/jama.290.4.511

Shen XL, 2017, HELICOBACTER, V22, DOI 10.1111/hel.12398

Socias SB, 2018, PROG NEUROBIOL, V162, P17, DOI 10.1016/j.pneurobio.2017.12.002

Song ER, 2021, J EXP MED, V218, DOI 10.1084/jem.20202135

Sriram K, 2006, J NEUROCHEM, V96, P706, DOI 10.1111/j.1471-4159.2005.03566.x

Stolzenberg E, 2017, J INNATE IMMUN, V9, P456, DOI 10.1159/000477990

Stott SRW, 2021, FRONT NEUROSCI-SWITZ, V15, DOI 10.3389/fnins.2021.653377

STRANG R R, 1965, Med J Aust, V1, P842

Su TH, 2019, MOVEMENT DISORD, V34, P1882, DOI 10.1002/mds.27848

Sun GL, 2015, J NEUROL SCI, V349, P190, DOI 10.1016/j.jns.2015.01.017

Suwarnalata G, 2016, PLOS ONE, V11, DOI 10.1371/journal.pone.0153725

Tai CH, 2019, BEHAV BRAIN RES, V364, P149, DOI 10.1016/j.bbr.2019.02.020

Tan AH, 2015, PARKINSONISM RELAT D, V21, P221, DOI 10.1016/j.parkreldis.2014.12.009

Taubenberger JK, 2006, P AM PHILOS SOC, V150, P86

Tikka TM, 2001, J IMMUNOL, V166, P7527, DOI 10.4049/jimmunol.166.12.7527

Tomiyama T, 1996, J BIOL CHEM, V271, P6839, DOI 10.1074/jbc.271.12.6839

Toovey S, 2011, INFLUENZA OTHER RESP, V5, P328, DOI 10.1111/j.1750-2659.2011.00232.x

Tsai HH, 2016, NEUROLOGY, V86, P840, DOI 10.1212/WNL.0000000000002307

Tsao SW, 2017, PHILOS T R SOC B, V372, DOI 10.1098/rstb.2016.0270

Tu T, 2017, BIOL CHEM, V398, P817, DOI 10.1515/hsz-2017-0118

Unger MM, 2016, PARKINSONISM RELAT D, V32, P66, DOI 10.1016/j.parkreldis.2016.08.019

van Kessel SP, 2019, NAT COMMUN, V10, DOI 10.1038/s41467-019-08294-y

Vlajinac H, 2013, INT J NEUROSCI, V123, P329, DOI 10.3109/00207454.2012.760560

Wangensteen KJ, 2016, CLIN NEUROPHARMACOL, V39, P1, DOI 10.1097/WNF.0000000000000120

Weissenborn K, 2006, GUT, V55, P1624, DOI 10.1136/gut.2005.080267

Wijarnpreecha K, 2018, EUR J GASTROEN HEPAT, V30, P9, DOI 10.1097/MEG.0000000000000991

Wroblewski LE, 2010, CLIN MICROBIOL REV, V23, P713, DOI 10.1128/CMR.00011-10

Wu DC, 2002, J NEUROSCI, V22, P1763, DOI 10.1523/JNEUROSCI.22-05-01763.2002

Wu WYY, 2015, J VIRAL HEPATITIS, V22, P784, DOI 10.1111/jvh.12392

Xu J, 2007, BRAIN RES, V1139, P220, DOI 10.1016/j.brainres.2006.12.074

Xue YC, 2018, FRONT MOL NEUROSCI, V11, DOI 10.3389/fnmol.2018.00063

Yang LC, 2003, J NEUROSCI RES, V74, P278, DOI 10.1002/jnr.10709

Zauli G, 2000, J BIOL CHEM, V275, P4159, DOI 10.1074/jbc.275.6.4159

Zhang YL, 2017, CELL DEATH DIS, V8, DOI 10.1038/cddis.2016.491

Zhang YL, 2015, MOL NEUROBIOL, V52, P78, DOI 10.1007/s12035-014-8845-z

de Rosso ALZ, 2009, ARQ NEURO-PSIQUIAT, V67, P827, DOI 10.1590/S0004-282X2009000500009

NR 172

TC 4

Z9 4

U1 0

U2 40

PU IOS PRESS

PI AMSTERDAM

PA NIEUWE HEMWEG 6B, 1013 BG AMSTERDAM, NETHERLANDS

SN 1877-7171

EI 1877-718X

J9 J PARKINSON DIS

JI J. Parkinsons Dis.

PY 2022

VL 12

IS 1

BP 27

EP 44

DI 10.3233/JPD-212929

PG 18

WC Neurosciences

WE Science Citation Index Expanded (SCI-EXPANDED)

SC Neurosciences & Neurology

GA YN2DC

UT WOS:000747072700002

PM 34719435

OA Green Published, hybrid

DA 2024-08-12

ER

PT J

AU Zhang, PF

Gao, F

AF Zhang, Peng-Fei

Gao, Fan

TI Neuroinflammation in Parkinson's disease: a meta-analysis of PET imaging

studies

SO JOURNAL OF NEUROLOGY

LA English

DT Review

DE Parkinson's disease; Inflammation; Neuroinflammation; Positron-emission

tomography; TSPO

ID PROTEIN 18 KDA; MICROGLIAL ACTIVATION; TRANSLOCATOR PROTEIN;

ALPHA-SYNUCLEIN; BENZODIAZEPINE-RECEPTOR; MULTIPLE-SCLEROSIS;

alzheimer's disease; NEURONAL FUNCTION; BINDING; BRAIN

AB Increasingly, evidence implicates an important role of neuroinflammation in neurodegeneration progression. Yet, brain imaging has not reached a consistent conclusion that neuroinflammation is involved in the pathogenesis of Parkinson's disease (PD). We aimed to review the evidence to quantitatively assess the existence and spatial distribution of neuroinflammation in the brain of PD patients. We systematically searched literature databases for case-control studies which used positron emission tomography to detect neuroinflammation represented by translocator protein (TSPO) levels in PD patients compared with healthy controls (HC). Standardized mean differences (SMD) were selected as effect sizes and random-effects models were used to combine effect sizes. Subgroup analyses for separate brain regions were conducted. Fifteen studies comprising 455 (HC = 198, PD = 238) participants and 19 brain regions were included. Compared to HC, PD patients had elevated TSPO levels in midbrain, putamen, anterior cingulate, posterior cingulate, thalamus, striatum, frontal, temporal, parietal, occipital, cortex, hippocampus, substantia nigra, pons, cerebellum, and caudate when using 1st-generation ligands. TSPO levels were elevated in the midbrain of PD patients when 2nd-generation ligands were used. We discussed the possible explanations of contrasting difference between these outcomes.

C1 [Zhang, Peng-Fei] Qingdao Univ, Qingdao Municipal Hosp, Sch Med, Dept Neurol, 5 Donghai Middle Rd, Qingdao, China.

[Gao, Fan] Affiliated Hosp Qingdao Univ, Dept Neurol, Qingdao, China.

C3 Qingdao Municipal Hospital; Qingdao University; Qingdao University

RP Zhang, PF (corresponding author), Qingdao Univ, Qingdao Municipal Hosp, Sch Med, Dept Neurol, 5 Donghai Middle Rd, Qingdao, China.

EM 2019020844@qdu.edu.cn

RI Zhang, Pengfei/GPC-5146-2022

OI Zhang, Pengfei/0000-0002-7090-0325

FU National Natural Science Foundation of China [81771148]

FX This study was supported by grants from the National Natural Science

Foundation of China (81771148).

CR Banati RB, 2002, GLIA, V40, P206, DOI 10.1002/glia.10144

Banati RB, 2000, BRAIN, V123, P2321, DOI 10.1093/brain/123.11.2321

Barkholt P, 2012, NEUROSCIENCE, V208, P85, DOI 10.1016/j.neuroscience.2012.02.004

Bartels AL, 2010, PARKINSONISM RELAT D, V16, P57, DOI 10.1016/j.parkreldis.2009.05.005

Belloli S, 2020, FRONT AGING NEUROSCI, V12, DOI 10.3389/fnagi.2020.00152

Chen X, 2018, FRONT IMMUNOL, V9, DOI 10.3389/fimmu.2018.02122

Choi YR, 2015, NEUROBIOL DIS, V83, P90, DOI 10.1016/j.nbd.2015.08.025

Costa B, 2009, ENDOCRINOLOGY, V150, P5438, DOI 10.1210/en.2009-0752

Costa B, 2009, PSYCHIAT GENET, V19, P110, DOI 10.1097/YPG.0b013e32832080f6

Debruyne JC, 2003, EUR J NEUROL, V10, P257, DOI 10.1046/j.1468-1331.2003.00571.x

Doorn KJ, 2014, ACTA NEUROPATHOL COM, V2, DOI 10.1186/s40478-014-0090-1

Dorsey ER, 2018, JAMA NEUROL, V75, P9, DOI 10.1001/jamaneurol.2017.3299

Edison P, 2013, NEUROPSYCHOPHARMACOL, V38, P938, DOI 10.1038/npp.2012.255

Fan Z, 2015, ALZHEIMERS DEMENT, V11, P608, DOI 10.1016/j.jalz.2014.06.016

Femminella GD, 2016, J ALZHEIMERS DIS, V51, P1275, DOI 10.3233/JAD-150827

Gao HM, 2011, ENVIRON HEALTH PERSP, V119, P807, DOI 10.1289/ehp.1003013

Gerhard A, 2006, NEUROBIOL DIS, V21, P404, DOI 10.1016/j.nbd.2005.08.002

Ghadery C, 2020, MOL NEUROBIOL, V57, P492, DOI 10.1007/s12035-019-01714-6

Ghadery C, 2017, J NEUROINFLAMM, V14, DOI 10.1186/s12974-016-0778-1

Gordon R, 2018, SCI TRANSL MED, V10, DOI 10.1126/scitranslmed.aah4066

Guo Q, 2012, NEUROIMAGE, V60, P902, DOI 10.1016/j.neuroimage.2011.12.078

Högel H, 2018, MULT SCLER J, V24, P1399, DOI 10.1177/1352458518791680

Iannaccone S, 2013, PARKINSONISM RELAT D, V19, P47, DOI 10.1016/j.parkreldis.2012.07.002

Joers V, 2017, PROG NEUROBIOL, V155, P57, DOI 10.1016/j.pneurobio.2016.04.006

Kang YN, 2018, J NEUROIMAGING, V28, P496, DOI 10.1111/jon.12519

Kobylecki C, 2013, PARKINSONISM RELAT D, V19, P527, DOI 10.1016/j.parkreldis.2013.01.017

Koshimori Y, 2015, PLOS ONE, V10, DOI 10.1371/journal.pone.0138721

Kouli A, 2020, ACTA NEUROPATHOL COM, V8, DOI 10.1186/s40478-020-01083-5

Kreisl WC, 2013, J CEREBR BLOOD F MET, V33, P53, DOI 10.1038/jcbfm.2012.131

Lagarde J, 2018, J NEURAL TRANSM, V125, P847, DOI 10.1007/s00702-017-1731-x

Lavisse S, 2021, PARKINSONISM RELAT D, V82, P29, DOI 10.1016/j.parkreldis.2020.11.011

Leal MC, 2013, FRONT CELL NEUROSCI, V7, DOI 10.3389/fncel.2013.00053

Lee HJ, 2008, BIOCHEM BIOPH RES CO, V372, P423, DOI 10.1016/j.bbrc.2008.05.045

Lin LF, 2021, STAT MED, V40, P403, DOI 10.1002/sim.8781

Martin I, 2011, ANNU REV GENOM HUM G, V12, P301, DOI 10.1146/annurev-genom-082410-101440

Masi A, 2015, MOL PSYCHIATR, V20, P440, DOI 10.1038/mp.2014.59

Ouchi Y, 2005, ANN NEUROL, V57, P168, DOI 10.1002/ana.20338

Owen DR, 2012, J CEREBR BLOOD F MET, V32, P1, DOI 10.1038/jcbfm.2011.147

Owen DRJ, 2011, INT REV NEUROBIOL, V101, P19, DOI 10.1016/B978-0-12-387718-5.00002-X

Page MJ, 2021, BMJ-BRIT MED J, V372, DOI [10.1016/j.ijsu.2021.105906, 10.1136/bmj.n71, 10.1136/bmj.n160]

Papadopoulos V, 2006, TRENDS PHARMACOL SCI, V27, P402, DOI 10.1016/j.tips.2006.06.005

POEWE W, 2017, NAT REV DIS PRIMERS, V3, DOI DOI 10.1038/NRDP.2017.13

Pont-Sunyer C, 2015, MOVEMENT DISORD, V30, P229, DOI 10.1002/mds.26077

Ransohoff RM, 2016, SCIENCE, V353, P777, DOI 10.1126/science.aag2590

Sanchez-Guajardo V, 2013, ASN NEURO, V5, P113, DOI 10.1042/AN20120066

Sanchez-Guajardo V, 2010, PLOS ONE, V5, DOI 10.1371/journal.pone.0008784

Schuiternaker A, 2007, J CEREBR BLOOD F MET, V27, P1603, DOI 10.1038/sj.jcbfm.9600459

Terada T, 2016, ANN NUCL MED, V30, P579, DOI 10.1007/s12149-016-1099-2

Varnäs K, 2019, EUR J NUCL MED MOL I, V46, P367, DOI 10.1007/s00259-018-4161-6

Wang SJ, 2015, P NATL ACAD SCI USA, V112, pE1926, DOI 10.1073/pnas.1417883112

Yasuno F, 2008, BIOL PSYCHIAT, V64, P835, DOI 10.1016/j.biopsych.2008.04.021

Yokokura M, 2017, J CEREBR BLOOD F MET, V37, P877, DOI 10.1177/0271678X16646788

NR 52

TC 18

Z9 19

U1 0

U2 16

PU SPRINGER HEIDELBERG

PI HEIDELBERG

PA TIERGARTENSTRASSE 17, D-69121 HEIDELBERG, GERMANY

SN 0340-5354

EI 1432-1459

J9 J NEUROL

JI J. Neurol.

PD MAY

PY 2022

VL 269

IS 5

BP 2304

EP 2314

DI 10.1007/s00415-021-10877-z

EA NOV 2021

PG 11

WC Clinical Neurology

WE Science Citation Index Expanded (SCI-EXPANDED)

SC Neurosciences & Neurology

GA 0Q6DR

UT WOS:000713544900001

PM 34724571

DA 2024-08-12

ER

PT J

AU Amo-Aparicio, J

Daly, J

Hojen, JF

Dinarello, CA

AF Amo-Aparicio, Jesus

Daly, Jonathan

Hojen, Jesper Falkesgaard

Dinarello, Charles A.

TI Pharmacologic inhibition of NLRP3 reduces the levels of a-synuclein and

protects dopaminergic neurons in a model of Parkinson's disease

SO JOURNAL OF NEUROINFLAMMATION

LA English

DT Article

DE Inflammasome; NLRP3; alpha-synuclein; OLT1177; Parkinson's disease

ID MPTP MOUSE MODEL; ALPHA-SYNUCLEIN; SUBSTANTIA-NIGRA; INFLAMMASOME

INHIBITOR; RECEPTOR 2; BRAIN; ACTIVATION; MICROGLIA; OLT1177; TREM2

AB Background Parkinson's disease (PD) is characterized by a progressive degeneration of dopaminergic neurons, which leads to irreversible loss of peripheral motor functions. Death of dopaminergic neurons induces an inflammatory response in microglial cells, which further exacerbates neuronal loss. Reducing inflammation is expected to ameliorate neuronal loss and arrest motor dysfunctions. Because of the contribution of the NLRP3 inflammasome to the inflammatory response in PD, we targeted NLRP3 using the specific inhibitor OLT1177 (R).

Methods We evaluated the effectiveness of OLT1177 (R) in reducing the inflammatory response in an MPTP neurotoxic model of PD. Using a combination of in vitro and in vivo studies, we analyzed the effects of NLRP3 inhibition on pro-inflammatory markers in the brain, alpha-synuclein aggregation, and dopaminergic neuron survival. We also determined the effects of OLT1177 (R) on locomotor deficits associated with MPTP and brain penetrance.

Results Treatment with OLT1177 (R) prevented the loss of motor function, reduced the levels of alpha-synuclein, modulated pro-inflammatory markers in the nigrostriatal areas of the brain, and protected dopaminergic neurons from degeneration in the MPTP model of PD. We also demonstrated that OLT1177 (R) crosses the blood-brain barrier and reaches therapeutic concentrations in the brain.

Conclusions These data suggest that targeting the NLRP3 inflammasome by OLT1177 (R) may be a safe and novel therapeutic approach to arrest neuroinflammation and protect against neurological deficits of Parkinson's disease in humans.

C1 [Amo-Aparicio, Jesus; Daly, Jonathan; Hojen, Jesper Falkesgaard; Dinarello, Charles A.] Univ Colorado, Dept Med, 12700 E 19th Ave, Aurora, CO 80045 USA.

[Hojen, Jesper Falkesgaard] Aarhus Univ, Dept Clin Med, DK-8200 Aarhus, Denmark.

C3 University of Colorado System; University of Colorado Anschutz Medical

Campus; Aarhus University

RP Amo-Aparicio, J (corresponding author), Univ Colorado, Dept Med, 12700 E 19th Ave, Aurora, CO 80045 USA.

EM jesus.amoaparicio@cuanschutz.edu

OI Hojen, Jesper Falkesgaard/0000-0002-2629-5391

FU NIH [AI-15614]; Interleukin Foundation; Olatec Therapeutics LLC;

Lundbeck Foundation

FX These studies are supported by NIH Grant AI-15614 from CAD, the

Interleukin Foundation, and Olatec Therapeutics LLC. JHF is supported by

the Lundbeck Foundation.

CR Ahn S, 2019, FRONT AGING NEUROSCI, V11, DOI 10.3389/fnagi.2019.00230

Amo-Aparicio J, 2022, EXP NEUROL, V347, DOI 10.1016/j.expneurol.2021.113889

Broz P, 2016, NAT REV IMMUNOL, V16, P407, DOI 10.1038/nri.2016.58

CRITCHLEY E, 1975, POSTGRAD MED J, V51, P619, DOI 10.1136/pgmj.51.599.619

Crowther RA, 2000, NEUROSCI LETT, V292, P128, DOI 10.1016/S0304-3940(00)01440-3

Dinarello CA, 2019, NAT REV RHEUMATOL, V15, P612, DOI 10.1038/s41584-019-0277-8

Dorsey ER, 2018, J PARKINSON DIS, V8, pS3, DOI 10.3233/JPD-181474

Dorsey ER, 2018, JAMA NEUROL, V75, P9, DOI 10.1001/jamaneurol.2017.3299

Dzamko N, 2017, ACTA NEUROPATHOL, V133, P303, DOI 10.1007/s00401-016-1648-8

Feigin VL, 2017, LANCET NEUROL, V16, P877, DOI 10.1016/S1474-4422(17)30299-5

Freeman D, 2013, PLOS ONE, V8, DOI 10.1371/journal.pone.0062143

García-Domínguez I, 2018, FRONT CELL NEUROSCI, V12, DOI 10.3389/fncel.2018.00398

George S, 2019, MOL NEURODEGENER, V14, DOI 10.1186/s13024-019-0335-3

Gordon R, 2018, SCI TRANSL MED, V10, DOI 10.1126/scitranslmed.aah4066

Guo M, 2020, BRAIN, V143, P1476, DOI 10.1093/brain/awaa090

Gustin A, 2015, PLOS ONE, V10, DOI 10.1371/journal.pone.0130624

Heneka MT, 2018, NAT REV NEUROSCI, V19, P610, DOI 10.1038/s41583-018-0055-7

Hornykiewicz O, 2010, J NEUROL, V257, pS249, DOI 10.1007/s00415-010-5741-y

Hu SS, 2020, NEUROPSYCH DIS TREAT, V16, P651, DOI 10.2147/NDT.S235562

Huang DP, 2017, PARKINSONS DIS-US, V2017, DOI 10.1155/2017/9349487

Jackson-Lewis V, 2007, NAT PROTOC, V2, P141, DOI 10.1038/nprot.2006.342

Jewell S, 2022, J PARKINSON DIS, V12, pS113, DOI 10.3233/JPD-223338

Keshet GI, 2007, J COMP NEUROL, V504, P690, DOI 10.1002/cne.21483

Kim C, 2015, CELL REP, V13, P771, DOI 10.1016/j.celrep.2015.09.044

Kim YS, 2006, EXP MOL MED, V38, P333, DOI 10.1038/emm.2006.40

Kluck V, 2020, LANCET RHEUMATOL, V2, pE270, DOI [10.1016/s2665-9913(20)30065-5, 10.1016/S2665-9913(20)30065-5]

Kouli A., 2018, Park Dis Pathog Clin Asp Internet

Lecht Shimon, 2007, Ther Clin Risk Manag, V3, P467

Lee E, 2019, CELL DEATH DIFFER, V26, P213, DOI 10.1038/s41418-018-0124-5

Lee HJ, 2008, BIOCHEM BIOPH RES CO, V372, P423, DOI 10.1016/j.bbrc.2008.05.045

Leem YH, 2020, BIOMED PHARMACOTHER, V130, DOI 10.1016/j.biopha.2020.110576

Li SS, 2022, CELL REP, V41, DOI 10.1016/j.celrep.2022.111532

Li XX, 2021, FRONT IMMUNOL, V12, DOI 10.3389/fimmu.2021.795036

Li YN, 2021, FRONT IMMUNOL, V12, DOI 10.3389/fimmu.2021.719807

Lian Hong, 2016, Bio Protoc, V6, DOI 10.21769/BioProtoc.1989

Liu GY, 2016, MOL NEUROBIOL, V53, P4931, DOI 10.1007/s12035-015-9416-7

Liu Z, 2019, BRAIN BEHAV IMMUN, V81, P630, DOI 10.1016/j.bbi.2019.07.026

Liu Z, 2017, MOL NEUROBIOL, V54, P7762, DOI 10.1007/s12035-016-0249-9

Lonnemann N, 2020, P NATL ACAD SCI USA, V117, P32145, DOI 10.1073/pnas.2009680117

Mantovani A, 2019, IMMUNITY, V50, P778, DOI 10.1016/j.immuni.2019.03.012

Marchetti C, 2019, J CARDIOVASC PHARM, V74, P285, DOI 10.1097/FJC.0000000000000718

Marchetti C, 2018, ARTHRITIS RES THER, V20, DOI 10.1186/s13075-018-1664-2

Marchetti C, 2018, P NATL ACAD SCI USA, V115, pE1530, DOI 10.1073/pnas.1716095115

Martinon F, 2002, MOL CELL, V10, P417, DOI 10.1016/S1097-2765(02)00599-3

MCGEER PL, 1988, NEUROLOGY, V38, P1285, DOI 10.1212/WNL.38.8.1285

Michel PP, 2016, NEURON, V90, P675, DOI 10.1016/j.neuron.2016.03.038

MOGI M, 1994, NEUROSCI LETT, V180, P147, DOI 10.1016/0304-3940(94)90508-8

MOGI M, 1994, NEUROSCI LETT, V165, P208, DOI 10.1016/0304-3940(94)90746-3

Muñoz-Planillo R, 2013, IMMUNITY, V38, P1142, DOI 10.1016/j.immuni.2013.05.016

Ou Z, 2021, MOL NEUROBIOL, V58, P1303, DOI 10.1007/s12035-020-02198-5

Park JH, 2020, MOL NEURODEGENER, V15, DOI 10.1186/s13024-019-0349-x

Park JS, 2017, FRONT NEUROSCI-SWITZ, V10, DOI 10.3389/fnins.2016.00618

Poewe W, 2017, NAT REV DIS PRIMERS, V3, DOI 10.1038/nrdp.2017.13

Prasad EM, 2021, PHARMACEUTICALS-BASE, V14, DOI 10.3390/ph14080717

Rabaneda-Lombarte N, 2021, J NEUROINFLAMM, V18, DOI 10.1186/s12974-021-02132-z

Rayaprolu S, 2013, MOL NEURODEGENER, V8, DOI 10.1186/1750-1326-8-19

Ren MR, 2018, EXP NEUROL, V302, P205, DOI 10.1016/j.expneurol.2018.01.016

Ross Owen A, 2013, Curr Genet Med Rep, V1, P52

Rui WJ, 2020, INT J NEUROPSYCHOPH, V23, P762, DOI 10.1093/ijnp/pyaa060

Sánchez-Fernández A, 2019, FRONT IMMUNOL, V10, DOI 10.3389/fimmu.2019.02578

Schwarz TC., 2023, P NATL ACAD SCI US, V120, DOI [10.1073/pnas.2201910120, DOI 10.1073/PNAS.2201910120]

Spillantini MG, 1997, NATURE, V388, P839, DOI 10.1038/42166

Stoiljkovic M, 2022, J ALZHEIMERS DIS, V88, P837, DOI 10.3233/JAD-210041

Stoker TB, 2018, FRONT NEUROSCI-SWITZ, V12, DOI 10.3389/fnins.2018.00693

Tengesdal IW, 2021, P NATL ACAD SCI USA, V118, DOI 10.1073/pnas.2000915118

Tyson T, 2016, J NEUROCHEM, V139, P275, DOI 10.1111/jnc.13449

Ugalde-Muñiz P, 2020, OXID MED CELL LONGEV, V2020, DOI 10.1155/2020/4807179

Vila M, 2000, J NEUROCHEM, V74, P721, DOI 10.1046/j.1471-4159.2000.740721.x

von Herrmann KM, 2018, NPJ PARKINSON DIS, V4, DOI 10.1038/s41531-018-0061-5

Walsh JG, 2014, NAT REV NEUROSCI, V15, P84, DOI 10.1038/nrn3638

Wohlford GF, 2021, J CARDIOVASC PHARM, V77, P49, DOI 10.1097/FJC.0000000000000931

Yu QY, 2021, FRONT PHARMACOL, V12, DOI 10.3389/fphar.2021.707696

Zhang QS, 2017, ACTA PHARMACOL SIN, V38, P1317, DOI 10.1038/aps.2017.49

Zhang YW, 2018, BIOCHEM BIOPH RES CO, V499, P797, DOI 10.1016/j.bbrc.2018.03.226

NR 74

TC 6

Z9 6

U1 3

U2 8

PU BMC

PI LONDON

PA CAMPUS, 4 CRINAN ST, LONDON N1 9XW, United Kingdom

EI 1742-2094

J9 J NEUROINFLAMM

JI J. Neuroinflamm.

PD JUN 22

PY 2023

VL 20

IS 1

AR 147

DI 10.1186/s12974-023-02830-w

PG 14

WC Immunology; Neurosciences

WE Science Citation Index Expanded (SCI-EXPANDED)

SC Immunology; Neurosciences & Neurology

GA K7UX7

UT WOS:001018464900001

PM 37349821

OA Green Published, gold

DA 2024-08-12

ER

PT J

AU Su, YC

Liu, YT

He, DW

Hu, GQ

Wang, HF

Ye, BJ

He, Y

Gao, XY

Liu, DF

AF Su, Yingchun

Liu, Yanting

He, Dewei

Hu, Guiqiu

Wang, Hefei

Ye, Bojian

He, Yuan

Gao, Xiyu

Liu, Dianfeng

TI Hordenine inhibits neuroinflammation and exerts neuroprotective effects

via inhibiting NF-κB and MAPK signaling pathways in vivo and in vitro

SO INTERNATIONAL IMMUNOPHARMACOLOGY

LA English

DT Article

DE Hordenine; Parkinson's disease; Neuroinflammation; NF-kappa B; MAPK

ID TYROSINE-HYDROXYLASE; parkinson's disease; INFLAMMATION; PROTECTS; MODEL

AB Parkinson's disease (PD) is a usual disease caused by degeneration of the central nervous system, which features the denaturation and death of dopaminergic neurons in the substantia nigra compact (SNc) of the midbrain. Neuroinflammation casts a consequential role in its pathogenesis, and the excessive activation of microglia as a major part of neuroinflammation cannot be ignored. Studies have indicated that Hordenine (HOR) functioned widely as an anti-oxidant and anti-inflammatory substance, but there are no reports on neuroinflammation effects. Therefore, this study is devoted to exploring the effect of HOR on neuroinflammation and its specific mechanism. In vivo, results revealed that HOR depressed the activation of microglia in SNc and protected dopaminergic neurons in the 6-hydroxydopamine (6-OHDA)-induced PD rat model, which terminally reduced movement disorders and weight loss. In vitro, studies have shown that HOR can inhibit inflammatory responses triggered by lipopolysaccharide (LPS) in BV-2 cells. More profound studies have discovered that the specific antiinflammatory mechanism is intimately associated with the NF-kappa B and MAPK signaling pathways. All in it together, HOR acts as a significant role in preserving dopaminergic neurons by restraining neuroinflammation mediated by activation of microglia. This may provide a potential drug for Parkinson's treatment.

C1 [He, Dewei; Gao, Xiyu; Liu, Dianfeng] Jilin Univ, Coll Anim Sci, Changchun, China.

[Su, Yingchun; Hu, Guiqiu; Wang, Hefei; Ye, Bojian; He, Yuan] Jilin Univ, Coll Vet Med, Changchun, China.

[Liu, Yanting] Catholic Univ Korea, Coll Med, Dept Neurosurg, Seoul St Marys Hosp, Seoul, South Korea.

C3 Jilin University; Jilin University; Catholic University of Korea; Seoul

St. Mary's Hospital

RP Liu, DF (corresponding author), Jilin Univ, Coll Anim Sci, Changchun, China.

EM suyc9918@mails.jlu.edu.cn; yantingliu02@gmail.com; m13144303829@163.com;

guiqiuhu@jlu.edu.cn; Wanghf9919@mails.jlu.edu.cn; yebj871781602@163.com;

heyuan9919@mails.jlu.edu.cn; gaoxy9916@mails.jlu.edu.cn; ccldf@163.com

RI Wang, Hefei/GOE-3868-2022

OI su, yingchun/0000-0003-0080-9523

FU National Natural Science Foundation of China [31772547]; Fundamental

Research Funds for the Central Universities [2021JCKT-06]; Jilin

Scientific and Technological Development Program [20200703011ZP]

FX The National Natural Science Foundation of China (project No.31772547),

Fundamental Research Funds for the Central Universities under Grant

(project No.2021JCKT-06) and Jilin Scientific and Technological

Development Program (project No.20200703011ZP) funded our study.

CR Badimon A, 2020, NATURE, V586, P417, DOI 10.1038/s41586-020-2777-8

Baker RG, 2011, CELL METAB, V13, P11, DOI 10.1016/j.cmet.2010.12.008

Cacabelos R, 2017, INT J MOL SCI, V18, DOI 10.3390/ijms18030551

Fang JY, 2005, LANCET ONCOL, V6, P322, DOI 10.1016/S1470-2045(05)70168-6

Fu SP, 2015, J NEUROINFLAMM, V12, DOI 10.1186/s12974-014-0230-3

Gao G, 2020, FRONT IMMUNOL, V11, DOI 10.3389/fimmu.2020.00161

Gao XY, 2020, IMMUNOBIOLOGY, V225, DOI 10.1016/j.imbio.2020.151965

Gelders G, 2018, J IMMUNOL RES, V2018, DOI 10.1155/2018/4784268

Guimaraes J, 2012, MOVEMENT DISORD, V27, P1078, DOI 10.1002/mds.25063

Haavik J, 1998, MOL NEUROBIOL, V16, P285, DOI 10.1007/BF02741387

Haque ME, 2020, MOVEMENT DISORD, V35, P20, DOI 10.1002/mds.27874

He DW, 2018, INT J MOL SCI, V19, DOI 10.3390/ijms19082242

Huang BX, 2018, FRONT IMMUNOL, V9, DOI 10.3389/fimmu.2018.02527

Huang BX, 2017, INT J MOL SCI, V18, DOI 10.3390/ijms18102043

Jeong YH, 2014, PLOS ONE, V9, DOI 10.1371/journal.pone.0087030

Jin X, 2019, CNS NEUROSCI THER, V25, P575, DOI 10.1111/cns.13086

Joers V, 2020, NEUROBIOL DIS, V144, DOI 10.1016/j.nbd.2020.105027

Kalia LV, 2015, LANCET, V386, P896, DOI 10.1016/S0140-6736(14)61393-3

Kim SC, 2013, FOOD CHEM, V141, P174, DOI 10.1016/j.foodchem.2013.03.017

Lawrence T, 2009, CSH PERSPECT BIOL, V1, DOI 10.1101/cshperspect.a001651

Lee YM, 2019, INT J MOL SCI, V20, DOI 10.3390/ijms20235907

LENARD L, 1991, BRAIN RES BULL, V27, P359, DOI 10.1016/0361-9230(91)90125-4

Liu ZY, 2020, J NEUROINFLAMM, V17, DOI 10.1186/s12974-020-01751-2

Mecha M, 2016, PHARMACOL THERAPEUT, V166, P40, DOI 10.1016/j.pharmthera.2016.06.011

Catorce MN, 2016, CURR NEUROPHARMACOL, V14, P155, DOI 10.2174/1570159X14666151204122017

Nayak D, 2014, ANNU REV IMMUNOL, V32, P367, DOI 10.1146/annurev-immunol-032713-120240

Pajares M, 2020, CELLS-BASEL, V9, DOI 10.3390/cells9071687

Poewe W, 2017, NAT REV DIS PRIMERS, V3, DOI 10.1038/nrdp.2017.13

Prinz M, 2019, CELL, V179, P292, DOI 10.1016/j.cell.2019.08.053

Sarkar S, 2020, J CLIN INVEST, V130, P4195, DOI 10.1172/JCI136174

Simola N, 2007, NEUROTOX RES, V11, P151, DOI 10.1007/BF03033565

Sommer T, 2020, J AGR FOOD CHEM, V68, P1998, DOI 10.1021/acs.jafc.9b06029

Stoddard-Bennett T, 2019, CELLS-BASEL, V8, DOI 10.3390/cells8010026

Su SH, 2018, BIOMED PHARMACOTHER, V104, P315, DOI 10.1016/j.biopha.2018.05.036

Su Yu-Sung, 2024, CRAN

Sun SC, 2017, NAT REV IMMUNOL, V17, P545, DOI 10.1038/nri.2017.52

Tolleson C, 2012, CNS NEUROL DISORD-DR, V11, P381, DOI 10.2174/187152712800792794

Vivekanantham S, 2015, INT J NEUROSCI, V125, P717, DOI 10.3109/00207454.2014.982795

Wang HH, 2018, CELL BIOSCI, V8, DOI 10.1186/s13578-018-0258-7

Wang QQ, 2015, TRANSL NEURODEGENER, V4, DOI 10.1186/s40035-015-0042-0

Wang X, 2020, EVID-BASED COMPL ALT, V2020, DOI 10.1155/2020/3107290

Whitton PS, 2007, BRIT J PHARMACOL, V150, P963, DOI 10.1038/sj.bjp.0707167

Yan X, 2017, INT J MOL SCI, V18, DOI 10.3390/ijms18020389

Yao ZQ, 2020, AGING-US, V12, P26121, DOI 10.18632/aging.202349

Zhou JW, 2018, J AGR FOOD CHEM, V66, P1620, DOI 10.1021/acs.jafc.7b05035

Zusso M, 2019, J NEUROINFLAMM, V16, DOI 10.1186/s12974-019-1538-9

NR 46

TC 10

Z9 12

U1 0

U2 7

PU ELSEVIER

PI AMSTERDAM

PA RADARWEG 29, 1043 NX AMSTERDAM, NETHERLANDS

SN 1567-5769

EI 1878-1705

J9 INT IMMUNOPHARMACOL

JI Int. Immunopharmacol.

PD JUL

PY 2022

VL 108

AR 108694

DI 10.1016/j.intimp.2022.108694

PG 12

WC Immunology; Pharmacology & Pharmacy

WE Science Citation Index Expanded (SCI-EXPANDED)

SC Immunology; Pharmacology & Pharmacy

GA 7V9AN

UT WOS:000913104200002

PM 35349959

DA 2024-08-12

ER

PT J

AU Peng, QY

Zhang, GX

Guo, XD

Dai, LJ

Xiong, M

Zhang, ZH

Chen, L

Zhang, ZT

AF Peng, Qinyu

Zhang, Guoxin

Guo, Xiaodi

Dai, Lijun

Xiong, Min

Zhang, Zhaohui

Chen, Liam

Zhang, Zhentao

TI Galectin-9/Tim-3 pathway mediates dopaminergic neurodegeneration in

MPTP-induced mouse model of Parkinson's disease

SO FRONTIERS IN MOLECULAR NEUROSCIENCE

LA English

DT Article

DE neurodegenerative disease; Galectin-9; neuroinflammation; mitochondrial

dysfunction; Parkinson's disease

ID ALPHA-SYNUCLEIN; NEUROINFLAMMATION; MICROGLIA; GALECTINS; CELLS; TAU

AB Galectin-9 (Gal-9) is a crucial immunoregulatory mediator in the central nervous system. Microglial activation and neuroinflammation play a key role in the degeneration of dopaminergic neurons in the substantia nigra (SN) in Parkinson's disease (PD). However, it remains unknown whether Gal-9 is involved in the pathogenesis of PD. We found that MPP+ treatment promoted the expression of Gal-9 and pro-inflammatory cytokines (IL-6, IL-1 beta, TNF-alpha, and MIP-1 alpha) in a concentration-dependent manner in BV2 cells. Gal-9 enhanced neurodegeneration and oxidative stress induced by MPP+ in SH-SY5Y cells and primary neurons. Importantly, deletion of Gal-9 or blockade of Tim-3 ameliorated microglial activation, reduced dopaminergic neuronal loss, and improved motor performance in an MPTP-induced mouse model of PD. These observations demonstrate a pathogenic role of the Gal-9/Tim-3 pathway in exacerbating microglial activation, neuroinflammation, oxidative stress, and dopaminergic neurodegeneration in the pathogenesis of PD.

C1 [Peng, Qinyu; Zhang, Guoxin; Guo, Xiaodi; Dai, Lijun; Xiong, Min; Zhang, Zhaohui; Zhang, Zhentao] Wuhan Univ, Renmin Hosp, Dept Neurol, Wuhan, China.

[Chen, Liam] Univ Minnesota, Med Sch, Dept Lab Med & Pathol, Minneapolis, MN 55455 USA.

C3 Wuhan University; University of Minnesota System; University of

Minnesota Twin Cities

RP Zhang, ZT (corresponding author), Wuhan Univ, Renmin Hosp, Dept Neurol, Wuhan, China.; Chen, L (corresponding author), Univ Minnesota, Med Sch, Dept Lab Med & Pathol, Minneapolis, MN 55455 USA.

EM llchen@umn.edu.cn; zhentaozhang@whu.edu.cn

RI Dai, Lijun/HPE-6038-2023; Zhang, Guoxin/S-7345-2017; Zhang,

Zhentao/JQV-7389-2023; zhao, weiwei/JUU-6585-2023

OI Zhang, Guoxin/0000-0002-2124-8502;

FU National Key Research & Development Program of China; National Natural

Science Foundation of China; Medical Science Advancement Program of

Wuhan University; [2019YFE0115900]; [81771382]; [81822016];

[TFLC2018001]

FX Funding This work was supported by grants from the National Key Research

& Development Program of China (2019YFE0115900), the National Natural

Science Foundation of China (nos. 81771382 and 81822016), and the

Medical Science Advancement Program of Wuhan University, grant

(TFLC2018001).

CR Aanhane E, 2018, ANGIOGENESIS, V21, P545, DOI 10.1007/s10456-018-9607-8

Anderson AC, 2007, SCIENCE, V318, P1141, DOI 10.1126/science.1148536

Barake F, 2020, CURR OPIN NEUROL, V33, P381, DOI 10.1097/WCO.0000000000000812

Bertino P, 2019, ONCOIMMUNOLOGY, V8, DOI 10.1080/2162402X.2019.1601482

Boza-Serrano A, 2019, ACTA NEUROPATHOL, V138, P251, DOI 10.1007/s00401-019-02013-z

Burman J, 2016, J NEUROIMMUNOL, V292, P40, DOI 10.1016/j.jneuroim.2016.01.008

Chen ZQ, 2019, CNS NEUROSCI THER, V25, P674, DOI 10.1111/cns.13100

Chia SJ, 2020, INT J MOL SCI, V21, DOI 10.3390/ijms21072464

da Rosa MM, 2021, EUR J PHARMACOL, V909, DOI 10.1016/j.ejphar.2021.174437

Dixon KO, 2021, NATURE, V595, P101, DOI 10.1038/s41586-021-03626-9

Eckardt V, 2020, EMBO REP, V21, DOI 10.15252/embr.201947852

Elahi S, 2012, BLOOD, V119, P4192, DOI 10.1182/blood-2011-11-389585

Falcon B, 2018, J BIOL CHEM, V293, P2438, DOI 10.1074/jbc.M117.809293

Fan JY, 2018, STEM CELL RES THER, V9, DOI 10.1186/s13287-018-0979-x

Forno LS, 1996, J NEUROPATH EXP NEUR, V55, P259, DOI 10.1097/00005072-199603000-00001

Gao L, 2015, J EXP MED, V212, P469, DOI 10.1084/jem.20132423

Ge MM, 2022, CURR NEUROPHARMACOL, V20, P2066, DOI 10.2174/1570159X20666220201094547

Golden-Mason L, 2017, HEPATOLOGY, V66, P271, DOI 10.1002/hep.29106

Harry GJ, 2008, EXPERT OPIN DRUG MET, V4, P1265, DOI 10.1517/17425255.4.10.1265

Imamura K, 2003, ACTA NEUROPATHOL, V106, P518, DOI 10.1007/s00401-003-0766-2

Iqbal AJ, 2022, FASEB J, V36, DOI 10.1096/fj.202100832R

Jia JY, 2020, DEV CELL, V52, P69, DOI 10.1016/j.devcel.2019.10.025

John S, 2016, FRONT MOL NEUROSCI, V9, DOI 10.3389/fnmol.2016.00139

Klibi J, 2009, BLOOD, V113, P1957, DOI 10.1182/blood-2008-02-142596

Koyama S, 2016, NAT COMMUN, V7, DOI 10.1038/ncomms10501

Krautter F, 2021, FRONT CELL DEV BIOL, V9, DOI 10.3389/fcell.2021.624082

Lee E, 2019, CELL DEATH DIFFER, V26, P213, DOI 10.1038/s41418-018-0124-5

Lee M, 2022, NAT COMMUN, V13, DOI 10.1038/s41467-022-28839-y

Lee VMY, 2006, NEURON, V52, P33, DOI 10.1016/j.neuron.2006.09.026

Lee Y, 2019, ARCH PHARM RES, V42, P416, DOI 10.1007/s12272-019-01133-0

Li Y, 2020, BRAIN BEHAV IMMUN, V83, P214, DOI 10.1016/j.bbi.2019.10.015

Liang TY, 2021, NEUROMOL MED, V23, P267, DOI 10.1007/s12017-020-08611-5

Liberal R, 2012, HEPATOLOGY, V56, P677, DOI 10.1002/hep.25682

Liu HB, 2022, EUR REV MED PHARMACO, V26, P623, DOI 10.26355/eurrev_202201_27889

Liu WW, 2020, FASEB J, V34, P6570, DOI 10.1096/fj.201901565RR

Lv Y, 2023, GENES DIS, V10, P2366, DOI 10.1016/j.gendis.2022.05.020

Mansour AA, 2022, BIOMED PHARMACOTHER, V151, DOI 10.1016/j.biopha.2022.113171

Martí Y, 2017, MOL CELL NEUROSCI, V83, P37, DOI 10.1016/j.mcn.2017.06.009

Miyakawa K, 2022, NAT COMMUN, V13, DOI 10.1038/s41467-022-28171-5

Nagatsu T, 2006, J NEURAL TRANSM-SUPP, P53

Hill CN, 2021, CANCER RES, V81

Pally D, 2022, ACS CHEM BIOL, V17, P1376, DOI 10.1021/acschembio.1c00902

Panda SK, 2018, J CLIN INVEST, V128, P1873, DOI 10.1172/JCI97333

Pardo E, 2019, MOL NEUROBIOL, V56, P7774, DOI 10.1007/s12035-019-1621-3

Polymeropoulos MH, 1997, SCIENCE, V276, P2045, DOI 10.1126/science.276.5321.2045

Premeaux TA, 2019, J NEUROVIROL, V25, P150, DOI 10.1007/s13365-018-0696-3

Rahimian R, 2021, MED RES REV, V41, P2582, DOI 10.1002/med.21784

Rahimian R, 2019, MOL NEUROBIOL, V56, P6371, DOI 10.1007/s12035-019-1527-0

Ren ZL, 2019, ACTA PHARMACOL SIN, V40, P441, DOI 10.1038/s41401-018-0077-8

Shen ZW, 2021, CNS NEUROSCI THER, V27, P842, DOI 10.1111/cns.13645

Siew JJ, 2019, NAT COMMUN, V10, DOI 10.1038/s41467-019-11441-0

Siew JJ, 2018, FRONT MOL NEUROSCI, V11, DOI 10.3389/fnmol.2018.00158

Steelman AJ, 2014, J NEUROINFLAMM, V11, DOI 10.1186/s12974-014-0144-0

Steelman AJ, 2013, J BIOL CHEM, V288, P23776, DOI 10.1074/jbc.M113.451658

Subramaniam SR, 2013, PROG NEUROBIOL, V106, P17, DOI 10.1016/j.pneurobio.2013.04.004

Surguchov A, 2022, NEUROMETHODS, V173, P155, DOI 10.1007/978-1-0716-1712-0_7

Tan YY, 2021, CELL BIOSCI, V11, DOI 10.1186/s13578-021-00592-7

Tao CC, 2020, CELL DEATH DIFFER, V27, P192, DOI 10.1038/s41418-019-0348-z

Wang X, 2019, J PSYCHIATR RES, V119, P102, DOI 10.1016/j.jpsychires.2019.10.002

Wdowiak K, 2018, INT J MOL SCI, V19, DOI 10.3390/ijms19010210

Wiersma VR, 2019, INT J MOL SCI, V20, DOI 10.3390/ijms20164046

Xu WD, 2021, AUTOIMMUN REV, V20, DOI 10.1016/j.autrev.2021.102847

Yang Ri-Yao, 2008, Expert Reviews in Molecular Medicine, V10, P1, DOI 10.1017/S1462399408000719

Yang RY, 2022, J BIOL CHEM, V298, DOI 10.1016/j.jbc.2022.101821

Yang RY, 2021, NAT COMMUN, V12, DOI 10.1038/s41467-021-21099-2

Zhang CX, 2020, ONCOGENESIS, V9, DOI 10.1038/s41389-020-00248-0

Zhang GX, 2022, MOL NEURODEGENER, V17, DOI 10.1186/s13024-022-00518-y

Zhang Y, 2020, LIFE SCI, V253, DOI 10.1016/j.lfs.2020.117745

Zhang ZT, 2014, NAT MED, V20, P1254, DOI 10.1038/nm.3700

Zheng R, 2021, J INFLAMM RES, V14, P3063, DOI 10.2147/JIR.S317672

NR 70

TC 1

Z9 1

U1 2

U2 16

PU FRONTIERS MEDIA SA

PI LAUSANNE

PA AVENUE DU TRIBUNAL FEDERAL 34, LAUSANNE, CH-1015, SWITZERLAND

SN 1662-5099

J9 FRONT MOL NEUROSCI

JI Front. Molec. Neurosci.

PD NOV 21

PY 2022

VL 15

AR 1046992

DI 10.3389/fnmol.2022.1046992

PG 14

WC Neurosciences

WE Science Citation Index Expanded (SCI-EXPANDED)

SC Neurosciences & Neurology

GA 6S9HR

UT WOS:000893294600001

PM 36479526

OA gold, Green Published

DA 2024-08-12

ER

PT J

AU Walsh, S

Finn, DP

Dowd, E

AF Walsh, S.

Finn, D. P.

Dowd, E.

TI TIME-COURSE OF NIGROSTRIATAL NEURODEGENERATION AND NEUROINFLAMMATION IN

THE 6-HYDROXYDOPAMINE-INDUCED AXONAL AND TERMINAL LESION MODELS OF

PARKINSON'S DISEASE IN THE RAT

SO NEUROSCIENCE

LA English

DT Article

DE Parkinson's disease; neuroinflammation; 6-hydroxydopamine; striatum;

substantia nigra

ID DEEP BRAIN-STIMULATION; MICROGLIAL ACTIVATION; SUBSTANTIA-NIGRA;

DOPAMINERGIC-NEURONS; SUBTHALAMIC NUCLEUS; POSTMORTEM ANALYSIS;

alzheimer's disease; STRIATAL INJECTION; REACTIVE MICROGLIA;

ALPHA-SYNUCLEIN

AB The pathogenesis of Parkinson's disease is thought to involve a self-sustaining cycle of neuroinflammation and neurodegeneration. In order to develop novel anti-inflammatory therapies to break this cycle, it is crucial that the temporal relationship between neurodegeneration and neuroinflammation is characterised in pre-clinical models to maximise their predictive validity. Thus, this study aimed to investigate the progression of neuroinflammation relative to nigrostriatal neurodegeneration in the two most commonly-used rat models of Parkinson's disease. Male Sprague Dawley rats were lesioned by terminal or axonal administration of 6-hydroxydopamine, and were sacrificed for quantitative immunohistochemistry (to assess nigrostriatal integrity (anti-tyrosine hydroxylase), microgliosis (anti-OX42) and astrocytosis (anti-GFAP)) at 6 h 24 h 72 h or 2 weeks post-lesion. Following terminal lesion, dopaminergic deafferentation of the striatum was evident from 6 h post-lesion and was accompanied by microglial and astroglial activation. Dopamine neuron loss from the substantia nigra did not occur until 2 weeks after terminal lesion, and this was preceded by microglial, but not astroglial, activation. Following axonal lesion, retraction of nigrostriatal terminals from the striatum was not observed until the 72 h time-point, and this was associated with a slight astrocytosis, but not microgliosis. Degeneration of dopaminergic neurons from the substantia nigra was also evident from 72 h after axonal lesion, and was accompanied by nigral microgliosis and astrocytosis by 2 weeks. This study highlights the temporal relationship between neurodegeneration and neuroinflammation in models of Parkinson's disease, and should facilitate use of these models in the development of anti-inflammatory therapies for the human condition. (C) 2011 IBRO. Published by Elsevier Ltd. All rights reserved.

C1 [Walsh, S.; Finn, D. P.; Dowd, E.] Natl Univ Ireland, Dept Pharmacol & Therapeut, Galway, Ireland.

[Walsh, S.; Finn, D. P.; Dowd, E.] Natl Univ Ireland, Natl Ctr Biomed Engn Sci, Galway, Ireland.

C3 Ollscoil na Gaillimhe-University of Galway; Ollscoil na

Gaillimhe-University of Galway

RP Dowd, E (corresponding author), Natl Univ Ireland, Dept Pharmacol & Therapeut, Galway, Ireland.

EM eilis.dowd@nuigalway.ie

OI Finn, David/0000-0001-6186-621X; Dowd, Eilis/0000-0002-2668-539X

FU Irish Research Council for Science, Engineering and Technology

FX S. Walsh is a recipient of an EMBARK PhD studentship from the Irish

Research Council for Science, Engineering and Technology.

CR AKIYAMA H, 1989, BRAIN RES, V489, P247, DOI 10.1016/0006-8993(89)90857-3

Ambrosi G, 2010, BRAIN RES BULL, V82, P29, DOI 10.1016/j.brainresbull.2010.01.011

Blandini F, 2007, EUR J NEUROSCI, V25, P397, DOI 10.1111/j.1460-9568.2006.05285.x

Butovsky O, 2005, MOL CELL NEUROSCI, V29, P381, DOI 10.1016/j.mcn.2005.03.005

Chung YC, 2010, BMB REP, V43, P225, DOI 10.5483/BMBRep.2010.43.4.225

Cicchetti F, 2002, EUR J NEUROSCI, V15, P991, DOI 10.1046/j.1460-9568.2002.01938.x

Counelis GJ, 2003, MOVEMENT DISORD, V18, P1062, DOI 10.1002/mds.10489

Crotty S, 2008, EUR J NEUROSCI, V27, P294, DOI 10.1111/j.1460-9568.2007.06018.x

Depino AM, 2003, EUR J NEUROSCI, V18, P2731, DOI 10.1111/j.1460-9568.2003.03014.x

DiLorenzo DJ, 2010, MOVEMENT DISORD, V25, P232, DOI 10.1002/mds.22935

Floden AM, 2005, J NEUROSCI, V25, P2566, DOI 10.1523/JNEUROSCI.4998-04.2005

Gaig C, 2009, MOVEMENT DISORD, V24, pS656, DOI 10.1002/mds.22672

Gao HM, 2002, J NEUROCHEM, V81, P1285, DOI 10.1046/j.1471-4159.2002.00928.x

Gao HM, 2008, TRENDS IMMUNOL, V29, P357, DOI 10.1016/j.it.2008.05.002

Garden GA, 2006, J NEUROIMMUNE PHARM, V1, P127, DOI 10.1007/s11481-006-9015-5

GRAEBER MB, 1988, J NEUROSCI RES, V21, P18, DOI 10.1002/jnr.490210104

Grealish S, 2008, BRAIN RES BULL, V77, P312, DOI 10.1016/j.brainresbull.2008.08.018

He Y, 2001, BRAIN RES, V909, P187, DOI 10.1016/S0006-8993(01)02681-6

Henderson JM, 2002, MOVEMENT DISORD, V17, P133, DOI 10.1002/mds.1261

Henning J, 2008, NEUROSCI RES, V62, P246, DOI 10.1016/j.neures.2008.09.001

Hirsch EC, 2009, LANCET NEUROL, V8, P382, DOI 10.1016/S1474-4422(09)70062-6

Iczkiewicz J, 2007, EXP NEUROL, V207, P95, DOI 10.1016/j.expneurol.2007.05.030

Jarraya B, 2003, MOVEMENT DISORD, V18, P1517, DOI 10.1002/mds.10607

Kim YS, 2007, FASEB J, V21, P179, DOI 10.1096/fj.06-5865com

Koprich JB, 2008, J NEUROINFLAMM, V5, DOI 10.1186/1742-2094-5-8

Langston JW, 1999, ANN NEUROL, V46, P598, DOI 10.1002/1531-8249(199910)46:4<598::AID-ANA7>3.0.CO;2-F

Lu L, 2010, CNS NEUROL DISORD-DR, V9, P232, DOI 10.2174/187152710791012035

Marinova-Mutafchieva L, 2009, J NEUROCHEM, V110, P966, DOI 10.1111/j.1471-4159.2009.06189.x

McGeer PL, 2003, ANN NEUROL, V54, P599, DOI 10.1002/ana.10728

MCGEER PL, 1988, NEUROLOGY, V38, P1285, DOI 10.1212/WNL.38.8.1285

Nussbaum RL, 2003, NEW ENGL J MED, V348, P1356, DOI 10.1056/NEJM2003ra020003

Rodrigues RWP, 2004, INT J NEUROSCI, V114, P197, DOI 10.1080/00207450490249338

Rodrigues RWP, 2001, INT J NEUROSCI, V109, P91, DOI 10.3109/00207450108986528

Rodriguez-Pallares J, 2007, J NEUROCHEM, V103, P145, DOI 10.1111/j.1471-4159.2007.04699.x

Rosenblad C, 2000, EXP NEUROL, V161, P503, DOI 10.1006/exnr.1999.7296

Sánchez-Pernaute R, 2004, J NEUROINFLAMM, V1, DOI 10.1186/1742-2094-1-6

Sun DA, 2008, J NEUROSURG, V109, P325, DOI 10.3171/JNS/2008/109/8/0325

Tansey MG, 2008, FRONT BIOSCI-LANDMRK, V13, P709, DOI 10.2741/2713

Whitton PS, 2010, CURR OPIN INVEST DR, V11, P788

Wu DC, 2002, J NEUROSCI, V22, P1763, DOI 10.1523/JNEUROSCI.22-05-01763.2002

Zecca L, 2003, TRENDS NEUROSCI, V26, P578, DOI 10.1016/j.tins.2003.08.009

Zhang W, 2004, FASEB J, V18, P589, DOI 10.1096/fj.03-0983fje

Zhang W, 2005, FASEB J, V19, P533, DOI 10.1096/fj.04-2751com

Zhang W, 2007, GLIA, V55, P1178, DOI 10.1002/glia.20532

NR 44

TC 106

Z9 116

U1 1

U2 6

PU PERGAMON-ELSEVIER SCIENCE LTD

PI OXFORD

PA THE BOULEVARD, LANGFORD LANE, KIDLINGTON, OXFORD OX5 1GB, United Kingdom

SN 0306-4522

EI 1873-7544

J9 NEUROSCIENCE

JI Neuroscience

PD FEB 17

PY 2011

VL 175

BP 251

EP 261

DI 10.1016/j.neuroscience.2010.12.005

PG 11

WC Neurosciences

WE Science Citation Index Expanded (SCI-EXPANDED)

SC Neurosciences & Neurology

GA 718FK

UT WOS:000287106300024

PM 21145947

DA 2024-08-12

ER

PT J

AU Chen, Y

Dai, JC

Tang, LF

Mikhailova, T

Liang, QM

Li, M

Zhou, JQ

Kopp, RF

Weickert, C

Chen, C

Liu, CY

AF Chen, Yu

Dai, Jiacheng

Tang, Longfei

Mikhailova, Tatiana

Liang, Qiuman

Li, Miao

Zhou, Jiaqi

Kopp, Richard F.

Weickert, Cynthia

Chen, Chao

Liu, Chunyu

TI Neuroimmune transcriptome changes in patient brains of psychiatric and

neurological disorders

SO MOLECULAR PSYCHIATRY

LA English

DT Article

ID CORTICOTROPIN-RELEASING HORMONE; PRENATAL IMMUNE ACTIVATION;

alzheimer's disease; GENE-EXPRESSION; BIPOLAR DISORDER; SUBSTANCE-P; MICE

LEADS; NEUROINFLAMMATION; SCHIZOPHRENIA; INFLAMMATION

AB Neuroinflammation has been implicated in multiple brain disorders but the extent and the magnitude of change in immune-related genes (IRGs) across distinct brain disorders has not been directly compared. In this study, 1275 IRGs were curated and their expression changes investigated in 2467 postmortem brains of controls and patients with six major brain disorders, including schizophrenia (SCZ), bipolar disorder (BD), autism spectrum disorder (ASD), major depressive disorder (MDD), Alzheimer's disease (AD), and Parkinson's disease (PD). There were 865 IRGs present across all microarray and RNA-seq datasets. More than 60% of the IRGs had significantly altered expression in at least one of the six disorders. The differentially expressed immune-related genes (dIRGs) shared across disorders were mainly related to innate immunity. Moreover, sex, tissue, and putative cell type were systematically evaluated for immune alterations in different neuropsychiatric disorders. Co-expression networks revealed that transcripts of the neuroimmune systems interacted with neuronal-systems, both of which contribute to the pathology of brain disorders. However, only a few genes with expression changes were also identified as containing risk variants in genome-wide association studies. The transcriptome alterations at gene and network levels may clarify the immune-related pathophysiology and help to better define neuropsychiatric and neurological disorders.

C1 [Chen, Yu; Dai, Jiacheng; Tang, Longfei; Liang, Qiuman; Li, Miao; Zhou, Jiaqi; Chen, Chao; Liu, Chunyu] Cent South Univ, Xiangya Hosp 2, Sch Life Sci, Ctr Med Genet, Changsha, China.

[Chen, Yu; Dai, Jiacheng; Tang, Longfei; Liang, Qiuman; Li, Miao; Zhou, Jiaqi; Chen, Chao; Liu, Chunyu] Cent South Univ, Xiangya Hosp 2, Sch Life Sci, Hunan Key Lab Med Genet, Changsha, China.

[Chen, Yu; Dai, Jiacheng; Tang, Longfei; Liang, Qiuman; Li, Miao; Zhou, Jiaqi; Chen, Chao; Liu, Chunyu] Cent South Univ, Xiangya Hosp 2, Dept Psychiat, Changsha, China.

[Chen, Yu] Broad Inst MIT & Harvard, Cambridge, MA 02142 USA.

[Dai, Jiacheng] Fudan Univ, State Key Lab Genet Engn, Human Phenome Inst, Shanghai, China.

[Dai, Jiacheng] Fudan Univ, Sch Life Sci, Shanghai, China.

[Mikhailova, Tatiana; Kopp, Richard F.; Liu, Chunyu] SUNY Upstate Med Univ, Dept Psychiat, Syracuse, NY USA.

[Weickert, Cynthia] SUNY Upstate Med Univ, Dept Neurosci & Physiol, Syracuse, NY 13210 USA.

[Weickert, Cynthia] UNSW, Sch Psychiat, Sydney, NSW, Australia.

[Chen, Chao] Cent South Univ, Hunan Key Lab Anim Models Human Dis, Changsha, China.

C3 Central South University; Central South University; Central South

University; Harvard University; Massachusetts Institute of Technology

(MIT); Broad Institute; Fudan University; Fudan University; State

University of New York (SUNY) System; State University of New York

(SUNY) Upstate Medical Center; State University of New York (SUNY)

System; State University of New York (SUNY) Upstate Medical Center;

University of New South United Kingdom Sydney; Central South University

RP Chen, C; Liu, CY (corresponding author), Cent South Univ, Xiangya Hosp 2, Sch Life Sci, Ctr Med Genet, Changsha, China.; Chen, C; Liu, CY (corresponding author), Cent South Univ, Xiangya Hosp 2, Sch Life Sci, Hunan Key Lab Med Genet, Changsha, China.; Chen, C; Liu, CY (corresponding author), Cent South Univ, Xiangya Hosp 2, Dept Psychiat, Changsha, China.; Chen, C (corresponding author), Cent South Univ, Hunan Key Lab Anim Models Human Dis, Changsha, China.

EM Chaochen@sklmg.edu.cn; LiuCh@upstate.edu

RI Tang, Longfei/O-9251-2017; Liu, Chunyu/G-7561-2012

OI Liu, Chunyu/0000-0002-5986-4415; Chen, Chao/0000-0002-5114-2282;

Mikhailova, Tatiana/0000-0003-4169-1322; Zhou,

Jiaqi/0009-0005-4139-9606; Li, Miao/0009-0008-9903-9111

FU National Natural Science Foundation of China [82022024, 31970572,

31871276]; National Key R&D Project of China [2016YFC1306000,

2017YFC0908701]; Innovation-driven Project of Central South University

[2020CX003]; NIH [U01MH122591, U01MH116489, R01MH110920]

FX National Natural Science Foundation of China Nos. 82022024. National

Natural Science Foundation of China Nos. 31970572. National Natural

Science Foundation of China Nos. 31871276. the National Key R&D Project

of China Grants No. 2016YFC1306000. the National Key R&D Project of

China Grants No. 2017YFC0908701. Innovation-driven Project of Central

South University Grant Nos. 2020CX003. NIH grants U01MH122591. NIH

grants U01MH116489. NIH grants R01MH110920.

CR Akbarian S, 2015, NAT NEUROSCI, V18, P1707, DOI 10.1038/nn.4156

Alganem K, 2020, BIORXIV, DOI 10.1101/2020.05.01.070805

[Anonymous], 2018, PIC TOOLK

Anttila V, 2018, SCIENCE, V360, P1313, DOI 10.1126/science.aap8757

Asadi S, 2012, J NEUROINFLAMM, V9, DOI 10.1186/1742-2094-9-85

Benedetti F, 2020, FRONT PSYCHIATRY, V11, DOI 10.3389/fpsyt.2020.00071

Bhattacharya S, 2018, SCI DATA, V5, DOI 10.1038/sdata.2018.15

Birnbaum R, 2018, MOL PSYCHIATR, V23, P1251, DOI 10.1038/mp.2017.89

Bitanihirwe BKY, 2010, NEUROPSYCHOPHARMACOL, V35, P2462, DOI 10.1038/npp.2010.129

Blalock EM, 2011, J CHEM NEUROANAT, V42, P118, DOI 10.1016/j.jchemneu.2011.06.007

Breuer K, 2013, NUCLEIC ACIDS RES, V41, pD1228, DOI 10.1093/nar/gks1147

Caccamo A, 2014, J NEUROSCI, V34, P7988, DOI 10.1523/JNEUROSCI.0777-14.2014

Cai HQ, 2020, MOL PSYCHIATR, V25, P761, DOI 10.1038/s41380-018-0235-x

Carbon S, 2019, NUCLEIC ACIDS RES, V47, pD330, DOI 10.1093/nar/gky1055

Díaz-Ramos MC, 2011, IMMUNOL LETT, V134, P183, DOI 10.1016/j.imlet.2010.09.016

Chang LC, 2014, PLOS ONE, V9, DOI 10.1371/journal.pone.0090980

Chen C, 2018, SCI TRANSL MED, V10, DOI 10.1126/scitranslmed.aat8178

Chow ML, 2011, BMC GENOMICS, V12, DOI 10.1186/1471-2164-12-449

Claes SJ, 2004, ANN MED, V36, P50, DOI 10.1080/07853890310017044

Connors EJ, 2014, BRAIN BEHAV IMMUN, V42, P178, DOI 10.1016/j.bbi.2014.06.020

Costa-Pinto FA, 2010, NEUROIMMUNOMODULAT, V17, P196, DOI 10.1159/000258722

D'Mello C, 2017, CURR TOP BEHAV NEURO, V31, P73, DOI 10.1007/7854_2016_37

Dantzer R, 2018, PHYSIOL REV, V98, P477, DOI 10.1152/physrev.00039.2016

Davis AP, 2019, NUCLEIC ACIDS RES, V47, pD948, DOI 10.1093/nar/gky868

de Leeuw CA, 2015, PLOS COMPUT BIOL, V11, DOI 10.1371/journal.pcbi.1004219

De Virgilio A, 2016, AUTOIMMUN REV, V15, P1005, DOI 10.1016/j.autrev.2016.07.022

Dougherty JD, 2010, NUCLEIC ACIDS RES, V38, P4218, DOI 10.1093/nar/gkq130

Dumitriu A, 2016, BMC MED GENOMICS, V9, DOI 10.1186/s12920-016-0164-y

Durieux AMS, 2015, FRONT BEHAV NEUROSCI, V9, DOI 10.3389/fnbeh.2015.00343

Fillman SG, 2014, TRANSL PSYCHIAT, V4, DOI 10.1038/tp.2014.8

Gandal MJ, 2018, SCIENCE, V362, P1265, DOI 10.1126/science.aat8127

Gandal MJ, 2018, SCIENCE, V359, P693, DOI 10.1126/science.aad6469

Garbett K, 2008, NEUROBIOL DIS, V30, P303, DOI 10.1016/j.nbd.2008.01.012

Gendelman HE, 2002, J NEUROVIROL, V8, P474, DOI 10.1080/13550280290168631

Godec J, 2016, IMMUNITY, V44, P194, DOI 10.1016/j.immuni.2015.12.006

Goines P, 2010, CURR OPIN NEUROL, V23, P111, DOI 10.1097/WCO.0b013e3283373514

Haddick PCG, 2017, J ALZHEIMERS DIS, V56, P1037, DOI 10.3233/JAD-160524

Harris N, 2018, NEURON, V100, P1163, DOI 10.1016/j.neuron.2018.09.048

He ZX, 2020, CELL REP, V33, DOI 10.1016/j.celrep.2020.108343

Hoffman GE, 2019, SCI DATA, V6, DOI 10.1038/s41597-019-0183-6

Horiuchi F, 2021, J NEUROINFLAMM, V18, DOI 10.1186/s12974-021-02154-7

Iliff Jeffrey J, 2012, Sci Transl Med, V4, p147ra111, DOI 10.1126/scitranslmed.3003748

Iwamoto K, 2005, HUM MOL GENET, V14, P241, DOI 10.1093/hmg/ddi022

Jansen IE, 2019, NAT GENET, V51, P404, DOI 10.1038/s41588-018-0311-9

Kelley KW, 2003, BRAIN BEHAV IMMUN, V17, pS112

Khan SA, 2016, CNS NEUROL DISORD-DR, V15, P390, DOI 10.2174/1871527315666160321104303

Kim SY, 2012, EPILEPSIA, V53, P37, DOI 10.1111/j.1528-1167.2012.03701.x

Lammert CR, 2019, METHODS MOL BIOL, V1960, P227, DOI 10.1007/978-1-4939-9167-9_20

Langfelder P, 2008, BMC BIOINFORMATICS, V9, DOI 10.1186/1471-2105-9-559

Lanz TA, 2019, TRANSL PSYCHIAT, V9, DOI 10.1038/s41398-019-0492-8

Lee JW, 2008, J NEUROINFLAMM, V5, DOI 10.1186/1742-2094-5-37

Leek JT, 2012, BIOINFORMATICS, V28, P882, DOI 10.1093/bioinformatics/bts034

Lenz KM, 2018, FRONT IMMUNOL, V9, DOI 10.3389/fimmu.2018.00698

Li FR, 2012, J NEUROSCI RES, V90, P2237, DOI 10.1002/jnr.23121

Liang WS, 2007, PHYSIOL GENOMICS, V28, P311, DOI 10.1152/physiolgenomics.00208.2006

Liberzon A, 2011, BIOINFORMATICS, V27, P1739, DOI 10.1093/bioinformatics/btr260

Lotz M, 2005, J NEUROCHEM, V94, P289, DOI 10.1111/j.1471-4159.2005.03188.x

Louveau A, 2015, NATURE, V523, P337, DOI 10.1038/nature14432

Lu YJ, 2021, FRONT IMMUNOL, V12, DOI 10.3389/fimmu.2021.768966

Lyman M, 2014, NEUROSCI RES, V79, P1, DOI 10.1016/j.neures.2013.10.004

Mashaghi A, 2016, CELL MOL LIFE SCI, V73, P4249, DOI 10.1007/s00018-016-2293-z

Maycox PR, 2009, MOL PSYCHIATR, V14, P1083, DOI 10.1038/mp.2009.18

Meltzer A, 2017, NEUROPSYCHOPHARMACOL, V42, P284, DOI 10.1038/npp.2016.158

Mitra S, 2015, NEUROSCIENCE, V292, P46, DOI 10.1016/j.neuroscience.2014.12.052

Morey JN, 2015, CURR OPIN PSYCHOL, V5, P13, DOI 10.1016/j.copsyc.2015.03.007

Morimoto K, 2019, FRONT NEUROSCI-SWITZ, V13, DOI 10.3389/fnins.2019.00916

Mukherjee S, 2021, HELIYON, V7, DOI 10.1016/j.heliyon.2021.e08518

Narayan S, 2008, BRAIN RES, V1239, P235, DOI 10.1016/j.brainres.2008.08.023

Nasr IW, 2019, EXP NEUROL, V320, DOI 10.1016/j.expneurol.2019.112957

Nisticò R, 2017, MOL BRAIN, V10, DOI 10.1186/s13041-017-0308-9

Novellino F, 2020, INT J MOL SCI, V21, DOI 10.3390/ijms21031115

O'Dushlaine C, 2015, NAT NEUROSCI, V18, P199, DOI 10.1038/nn.3922

O'Kane M, 2006, EXP DERMATOL, V15, P143

Ogata H, 1999, NUCLEIC ACIDS RES, V27, P29, DOI 10.1093/nar/27.1.29

Owens T, 2001, NAT MED, V7, P161, DOI 10.1038/84603

Ozawa K, 2006, BIOL PSYCHIAT, V59, P546, DOI 10.1016/j.biopsych.2005.07.031

Pais TF, 2019, FRONT IMMUNOL, V9, DOI 10.3389/fimmu.2018.03100

Pan PY, 2020, BEHAV GENET, V50, P233, DOI 10.1007/s10519-019-09986-3

Pape K, 2019, NAT REV NEUROL, V15, P317, DOI 10.1038/s41582-019-0174-4

Parab S, 2021, ELIFE, V10, DOI 10.7554/eLife.64295

Parboosing R, 2013, JAMA PSYCHIAT, V70, P677, DOI 10.1001/jamapsychiatry.2013.896

Parshukova D, 2019, J MOL RECOGNIT, V32, DOI 10.1002/jmr.2759

Purves-Tyson TD, 2020, FRONT IMMUNOL, V11, DOI 10.3389/fimmu.2020.02002

Raudvere U, 2019, NUCLEIC ACIDS RES, V47, pW191, DOI 10.1093/nar/gkz369

Reddy PH, 2005, J ALZHEIMERS DIS, V7, P103, DOI 10.3233/jad-2005-7203

Resende R, 2020, BBA-MOL BASIS DIS, V1866, DOI 10.1016/j.bbadis.2020.165752

Riley BE, 2014, PLOS ONE, V9, DOI 10.1371/journal.pone.0102909

Russo AJ, 2014, J CENT NERV SYST DIS, V6, P79, DOI [10.4137/JCNSNSD.S13767, 10.4137/JCNSD.S13767]

Saetre P, 2007, BMC PSYCHIATRY, V7, DOI 10.1186/1471-244X-7-46

Sasada T, 2016, FRONT PHARMACOL, V7, DOI 10.3389/fphar.2016.00405

Simanek AM, 2015, CURR PROB PEDIATR AD, V45, P325, DOI 10.1016/j.cppeds.2015.06.008

Sinclair D, 2013, SCI REP-UK, V3, DOI 10.1038/srep03539

Sinclair D, 2012, BMC PSYCHIATRY, V12, DOI 10.1186/1471-244X-12-84

Sinclair D, 2012, PLOS ONE, V7, DOI 10.1371/journal.pone.0031720

Sinclair D, 2011, NEUROPSYCHOPHARMACOL, V36, P2698, DOI 10.1038/npp.2011.160

Sparkman NL, 2008, NEUROIMMUNOMODULAT, V15, P323, DOI 10.1159/000156474

Spitsin S, 2013, J NEUROL SCI, V334, P18, DOI 10.1016/j.jns.2013.07.008

Starkstein S, 2015, J NEURODEV DISORD, V7, DOI 10.1186/s11689-015-9125-6

't Hart BA, 2013, J NEUROIMMUNE PHARM, V8, P757, DOI 10.1007/s11481-013-9486-0

Tanabe S, 2018, INT IMMUNOL, V30, P437, DOI 10.1093/intimm/dxy041

Tsagarakis Stylianos, 1994, Neuroimmunomodulation, V1, P329, DOI 10.1159/000097184

Tylee DS, 2018, AM J MED GENET B, V177, P641, DOI 10.1002/ajmg.b.32652

Voineagu I, 2011, NATURE, V474, P380, DOI 10.1038/nature10110

Volk DW, 2017, NEUROBIOL DIS, V99, P58, DOI 10.1016/j.nbd.2016.12.019

Volk DW, 2015, AM J PSYCHIAT, V172, P1112, DOI 10.1176/appi.ajp.2015.15010019

Vuillermot S, 2010, J NEUROSCI, V30, P1270, DOI 10.1523/JNEUROSCI.5408-09.2010

Webers A, 2020, IMMUNOL CELL BIOL, V98, P28, DOI 10.1111/imcb.12301

Wu XJ, 2021, MOL PSYCHIATR, V26, P7699, DOI 10.1038/s41380-021-01205-y

Xiong J, 2019, FRONT NEUROSCI-SWITZ, V13, DOI 10.3389/fnins.2019.00349

Yang H, 2020, MEDICINE, V99, DOI 10.1097/MD.0000000000019561

Yu GC, 2012, OMICS, V16, P284, DOI 10.1089/omi.2011.0118

Yuan N, 2019, TRANSL PSYCHIAT, V9, DOI 10.1038/s41398-019-0570-y

Zambon AC, 2012, BIOINFORMATICS, V28, P2209, DOI 10.1093/bioinformatics/bts366

Zhang B, 2013, CELL, V153, P707, DOI 10.1016/j.cell.2013.03.030

Zhang MW, 2020, FRONT GENET, V11, DOI 10.3389/fgene.2020.00363

Zhang X, 2021, FRONT AGING NEUROSCI, V13, DOI 10.3389/fnagi.2021.605970

Zhang Y, 2016, TRANSL PSYCHIAT, V6, DOI 10.1038/tp.2016.238

Zhang Y, 2016, NEURON, V89, P37, DOI 10.1016/j.neuron.2015.11.013

Zhang YL, 2005, AM J MED GENET B, V137B, P5, DOI 10.1002/ajmg.b.30195

Zheng B, 2010, SCI TRANSL MED, V2, DOI 10.1126/scitranslmed.3001059

NR 120

TC 16

Z9 18

U1 3

U2 28

PU SPRINGERNATURE

PI LONDON

PA CAMPUS, 4 CRINAN ST, LONDON, N1 9XW, United Kingdom

SN 1359-4184

EI 1476-5578

J9 MOL PSYCHIATR

JI Mol. Psychiatr.

PD FEB

PY 2023

VL 28

IS 2

BP 710

EP 721

DI 10.1038/s41380-022-01854-7

EA NOV 2022

PG 12

WC Biochemistry & Molecular Biology; Neurosciences; Psychiatry

WE Science Citation Index Expanded (SCI-EXPANDED)

SC Biochemistry & Molecular Biology; Neurosciences & Neurology; Psychiatry

GA A6AE4

UT WOS:000887859400001

PM 36424395

OA Green Submitted, Green Accepted

DA 2024-08-12

ER

PT J

AU Mishra, A

Bandopadhyay, R

Singh, PK

Mishra, PS

Sharma, N

Khurana, N

AF Mishra, Awanish

Bandopadhyay, Ritam

Singh, Prabhakar Kumar

Mishra, Pragya Shakti

Sharma, Neha

Khurana, Navneet

TI Neuroinflammation in neurological disorders: pharmacotherapeutic targets

from bench to bedside

SO METABOLIC BRAIN DISEASE

LA English

DT Review

DE Neuroinflammation; Neurological disorders; Alzheimer's disease;

Parkinson's disease; Epilepsy

ID TRAUMATIC BRAIN-INJURY; TUMOR-NECROSIS-FACTOR; NONSTEROIDAL

ANTIINFLAMMATORY DRUGS; CYCLOOXYGENASE-2 COX-2 INHIBITOR; INDUCED

COGNITIVE IMPAIRMENT; INNATE IMMUNE-RESPONSES; PPAR-GAMMA AGONIST;

INDUCED RAT MODEL; alzheimer's disease; MOUSE MODEL

AB Neuroinflammation is one of the host defensive mechanisms through which the nervous system protects itself from pathogenic and or infectious insults. Moreover, neuroinflammation occurs as one of the most common pathological outcomes in various neurological disorders, makes it the promising target. The present review focuses on elaborating the recent advancement in understanding molecular mechanisms of neuroinflammation and its role in the etiopathogenesis of various neurological disorders, especially Alzheimer's disease (AD), Parkinson's disease (PD), and Epilepsy. Furthermore, the current status of anti-inflammatory agents in neurological diseases has been summarized in light of different preclinical and clinical studies. Finally, possible limitations and future directions for the effective use of anti-inflammatory agents in neurological disorders have been discussed.

C1 [Mishra, Awanish; Bandopadhyay, Ritam; Singh, Prabhakar Kumar; Sharma, Neha; Khurana, Navneet] Lovely Profess Univ, Sch Pharmaceut Sci, Dept Pharmacol, Phagwara 144411, India.

[Mishra, Pragya Shakti] Sanjay Gandhi Post Grad Inst Med Sci SGPGIMS, Dept Nucl Med, Raebareli Rd, Lucknow 226014, Uttar Pradesh, India.

[Mishra, Awanish] Natl Inst Pharmaceut Educ & Res NIPER, Dept Pharmacol & Toxicol, Gauhati 781101, Assam, India.

C3 Lovely Professional University; Sanjay Gandhi Postgraduate Institute of

Medical Sciences; National Institute of Pharmaceutical Education &

Research, S.A.S. Nagar (Mohali)

RP Mishra, A (corresponding author), Lovely Profess Univ, Sch Pharmaceut Sci, Dept Pharmacol, Phagwara 144411, India.; Mishra, A (corresponding author), Natl Inst Pharmaceut Educ & Res NIPER, Dept Pharmacol & Toxicol, Gauhati 781101, Assam, India.

EM awanish1985@gmail.com

RI Sharma, Neha/AAG-5292-2022; Mishra, Awanish/neurodegenerative disease-9537-2020; sharma,

neha/HLV-7039-2023; Sharma, Neha/AFK-6955-2022; Khurana,

Navneet/AGJ-1666-2022; Sharma, Neha/HPH-6624-2023

OI Sharma, Neha/0000-0003-2786-1874; Mishra, Awanish/0000-0001-7863-5581;

Sharma, Neha/0000-0003-2786-1874; Khurana, Navneet/0000-0001-9899-4856;

Bandopadhyay, Ritam/0000-0001-9339-3285

CR Abd Wahab DYA, 2019, BIOMED RES INT-UK, V2019, DOI 10.1155/2019/1767203

Abuelezz SA, 2021, LIFE SCI, V273, DOI 10.1016/j.lfs.2021.119310

Acar G, 2015, TURK NEUROSURG, V25, P749, DOI 10.5137/1019-5149.JTN.10738-14.0

Aguirre-Vidal Y, 2020, J NEUROIMMUNOL, V349, DOI 10.1016/j.jneuroim.2020.577426

Ahuja M, 2016, J NEUROSCI, V36, P6332, DOI 10.1523/JNEUROSCI.0426-16.2016

Aisen PS, 2003, JAMA-J AM MED ASSOC, V289, P2819, DOI 10.1001/jama.289.21.2819

Ajami B, 2011, NAT NEUROSCI, V14, P1142, DOI 10.1038/nn.2887

Akyuz E, 2021, LIFE SCI, V265, DOI 10.1016/j.lfs.2020.118826

Alavian SM, 2011, J VIRAL HEPATITIS, V18, P821, DOI 10.1111/j.1365-2893.2011.01530.x

Alcoreza O, 2019, EPILEPSIA, V60, P1365, DOI 10.1111/epi.16073

Ali MM, 2019, CUREUS J MED SCIENCE, V11, DOI 10.7759/cureus.4620

Alyu F, 2017, ACTA NEUROPSYCHIATR, V29, P1, DOI 10.1017/neu.2016.47

Angelopoulou E, 2021, J BIOCHEM MOL TOXIC, V35, DOI 10.1002/jbt.22720

Ardestani PM, 2017, NEUROPHARMACOLOGY, V116, P371, DOI 10.1016/j.neuropharm.2017.01.010

Atlashkin V, 2003, MOL CELL BIOL, V23, P5198, DOI 10.1128/MCB.23.15.5198-5207.2003

Attia GM, 2019, FOLIA NEUROPATHOL, V57, P146, DOI 10.5114/fn.2019.84423

Aucott H, 2018, MOL MED, V24, DOI 10.1186/s10020-018-0021-x

Auriel Eitan, 2014, Handb Clin Neurol, V119, P577, DOI 10.1016/B978-0-7020-4086-3.00038-2

Azmand MJ, 2021, AVICENNA J PHYTOMEDI, V11, P79, DOI 10.22038/ajp.2020.16490

Babaei P, 2021, NEUROCHEM RES, V46, P1480, DOI 10.1007/s11064-021-03289-y

Bailey JNC, 2016, NAT GENET, V48, P189, DOI 10.1038/ng.3482

Bak LK, 2006, J NEUROCHEM, V98, P641, DOI 10.1111/j.1471-4159.2006.03913.x

Barbiero JK, 2014, PROG NEURO-PSYCHOPH, V53, P35, DOI 10.1016/j.pnpbp.2014.02.009

Barger SW, 2001, J NEUROCHEM, V76, P846, DOI 10.1046/j.1471-4159.2001.00075.x

Bartels AL, 2010, PARKINSONISM RELAT D, V16, P57, DOI 10.1016/j.parkreldis.2009.05.005

Bassani TB, 2017, BEHAV BRAIN RES, V335, P41, DOI 10.1016/j.bbr.2017.08.014

Becher B, 2002, J CLIN INVEST, V110, P493, DOI 10.1172/JCI200215751

Becher B, 2017, NAT REV IMMUNOL, V17, P49, DOI 10.1038/nri.2016.123

Begum AN, 2008, J PHARMACOL EXP THER, V326, P196, DOI 10.1124/jpet.108.137455

Bianchi ME, 2007, IMMUNOL REV, V220, P35, DOI 10.1111/j.1600-065X.2007.00574.x

Boiangiu RS, 2020, ANTIOXIDANTS-BASEL, V9, DOI 10.3390/antiox9080768

BOKA G, 1994, NEUROSCI LETT, V172, P151, DOI 10.1016/0304-3940(94)90684-X

Brambilla R, 2019, ACTA NEUROPATHOL, V137, P689, DOI 10.1007/s00401-019-02009-9

Brás IC, 2021, CELLS-BASEL, V10, DOI 10.3390/cells10020375

Breitner J, 2020, NEUROLOGY, V94, P594, DOI 10.1212/WNL.0000000000009185

Bronisz E, 2016, MEDIAT INFLAMM, V2016, DOI 10.1155/2016/7369020

Broz P, 2016, NAT REV IMMUNOL, V16, P407, DOI 10.1038/nri.2016.58

Cai ZY, 2013, NEUROMOL MED, V15, P25, DOI 10.1007/s12017-012-8207-9

Calsolaro V, 2016, ALZHEIMERS DEMENT, V12, P719, DOI 10.1016/j.jalz.2016.02.010

Cao JW, 2020, J ETHNOPHARMACOL, V257, DOI 10.1016/j.jep.2020.112884

Cao WH, 2019, J CHEM NEUROANAT, V98, P63, DOI 10.1016/j.jchemneu.2019.04.003

Carpentier PA, 2008, BRAIN BEHAV IMMUN, V22, P140, DOI 10.1016/j.bbi.2007.08.011

Castro AA, 2013, BRAIN RES, V1513, P103, DOI 10.1016/j.brainres.2013.03.029

Chaudhry FA, 2002, J CELL BIOL, V157, P349, DOI 10.1083/jcb.200201070

Chen Y, 2019, J NEUROIMMUNOL, V334, DOI 10.1016/j.jneuroim.2019.576978

Cheng CY, 2019, LIFE SCI, V223, P120, DOI 10.1016/j.lfs.2019.02.037

Chidlow G, 2017, FRONT NEUROSCI-SWITZ, V11, DOI 10.3389/fnins.2017.00478

Cho S, 2005, J NEUROSCI, V25, P2504, DOI 10.1523/JNEUROSCI.0035-05.2005

Chrysostomou V, 2013, CURR OPIN PHARMACOL, V13, P12, DOI 10.1016/j.coph.2012.09.008

Chung Y, 2009, IMMUNITY, V30, P576, DOI 10.1016/j.immuni.2009.02.007

Ferrari CC, 2006, NEUROBIOL DIS, V24, P183, DOI 10.1016/j.nbd.2006.06.013

Citraro R, 2015, BRAIN RES BULL, V113, P1, DOI 10.1016/j.brainresbull.2015.02.004

Condello C, 2015, NAT COMMUN, V6, DOI 10.1038/ncomms7176

Côté M, 2015, BRAIN BEHAV IMMUN, V46, P154, DOI 10.1016/j.bbi.2015.01.009

Cummings J, 2021, MOL NEURODEGENER, V16, DOI 10.1186/s13024-021-00424-9

Dadsetan S, 2016, J NEUROINFLAMM, V13, DOI 10.1186/s12974-016-0710-8

Danbolt NC, 2001, PROG NEUROBIOL, V65, P1, DOI 10.1016/S0301-0082(00)00067-8

Daniels MJD, 2016, NAT COMMUN, V7, DOI 10.1038/ncomms12504

Das B, 2021, ADV EXP MED BIOL, V1286, P65, DOI 10.1007/978-3-030-55035-6_4

Davalos D, 2005, NAT NEUROSCI, V8, P752, DOI 10.1038/nn1472

Degan D, 2018, CURR PHARM DESIGN, V24, P1485, DOI 10.2174/1381612824666180327170632

Denney L, 2012, J IMMUNOL, V189, P551, DOI 10.4049/jimmunol.1103608

Dhir Ashish, 2006, Indian Journal of Experimental Biology, V44, P286

Dhir A, 2006, PROG NEURO-PSYCHOPH, V30, P1478, DOI 10.1016/j.pnpbp.2006.06.003

Di SN, 2021, BIOMED PHARMACOTHER, V137, DOI 10.1016/j.biopha.2021.111369

Duarte JN, 2021, J OPHTHALMOL, V2021, DOI 10.1155/2021/4581909

Dürk T, 2005, INT IMMUNOL, V17, P599, DOI 10.1093/intimm/dxh242

Edwards FA, 2019, TRENDS NEUROSCI, V42, P310, DOI 10.1016/j.tins.2019.03.003

Ehrenreich H, 2007, BRAIN, V130, P2577, DOI 10.1093/brain/awm203

Elçioglu HK, 2015, ACTA HISTOCHEM, V117, P176, DOI 10.1016/j.acthis.2014.12.002

Elfakhri KH, 2019, NEUROBIOL DIS, V125, P123, DOI 10.1016/j.nbd.2019.01.020

Erdogan MA, 2022, DRUG CHEM TOXICOL, V45, P2160, DOI 10.1080/01480545.2021.1914464

Eugster HP, 1998, EUR J IMMUNOL, V28, P2178, DOI 10.1002/(SICI)1521-4141(199807)28:07<2178::AID-IMMU2178>3.0.CO;2-D

Fang P, 2012, MOL NEUROBIOL, V45, P499, DOI 10.1007/s12035-012-8264-y

Farfel-Becker T, 2011, HUM MOL GENET, V20, P1375, DOI 10.1093/hmg/ddr019

Fassbender K, 2004, FASEB J, V18, P203, DOI 10.1096/fj.03-0364fje

Feigin VL, 2020, LANCET NEUROL, V19, P255, DOI 10.1016/S1474-4422(19)30411-9

Fernandes GL, 2017, CLIN IMMUNOL, V180, P58, DOI 10.1016/j.clim.2017.04.004

Gagne JJ, 2010, NEUROLOGY, V74, P995, DOI 10.1212/WNL.0b013e3181d5a4a3

Galic MA, 2012, FRONT NEUROENDOCRIN, V33, P116, DOI 10.1016/j.yfrne.2011.12.002

Garcez ML, 2017, PROG NEURO-PSYCHOPH, V77, P23, DOI 10.1016/j.pnpbp.2017.03.010

García E, 2014, FREE RADICAL RES, V48, P159, DOI 10.3109/10715762.2013.857019

Garcia-Curran MM, 2019, ENEURO, V6, DOI 10.1523/ENEURO.0430-19.2019

Garden GA, 2013, NEUROTHERAPEUTICS, V10, P782, DOI 10.1007/s13311-013-0207-4

Ghavami S, 2014, PROG NEUROBIOL, V112, P24, DOI 10.1016/j.pneurobio.2013.10.004

Ghavami S, 2012, BBA-MOL CELL RES, V1823, P2274, DOI 10.1016/j.bbamcr.2012.09.008

Gilhus NE, 2019, NAT REV NEUROL, V15, P429, DOI 10.1038/s41582-019-0227-8

Glass CK, 2010, CELL, V140, P918, DOI 10.1016/j.cell.2010.02.016

Glick D, 2010, J PATHOL, V221, P3, DOI 10.1002/path.2697

Gobbo OL, 2004, NEUROSCIENCE, V125, P317, DOI 10.1016/j.neuroscience.2004.01.045

Godfred RM, 2013, EPILEPSY BEHAV, V27, P115, DOI 10.1016/j.yebeh.2012.12.031

Gorter JA, 2006, J NEUROSCI, V26, P11083, DOI 10.1523/JNEUROSCI.2766-06.2006

Goudarzi R, 2020, EPILEPSY BEHAV, V104, DOI 10.1016/j.yebeh.2019.106500

Gu XJ, 2014, CHIN J TRAUMATOL, V17, P1, DOI 10.3760/cma.j.issn.1008-1275.2014.01.001

Guan PP, 2019, FASEB J, V33, P13, DOI 10.1096/fj.201800355RRRR

Guan Y, 2018, LIFE SCI, V202, P1, DOI 10.1016/j.lfs.2018.03.031

Gugliandolo A, 2018, INFLAMMATION, V41, P93, DOI 10.1007/s10753-017-0667-5

Guo DD, 2013, INT J BIOCHEM CELL B, V45, P1849, DOI 10.1016/j.biocel.2013.06.002

Gupta A, 2010, INDIAN J EXP BIOL, V48, P577

Gupta A, 2009, PHARMACOL BIOCHEM BE, V94, P219, DOI 10.1016/j.pbb.2009.07.018

Gurung P, 2015, TRENDS MOL MED, V21, P193, DOI 10.1016/j.molmed.2014.11.008

Gyengesi E, 2020, NAT REV NEUROL, V16, P131, DOI 10.1038/s41582-019-0307-9

Hammond JW, 2016, FRONT CELL NEUROSCI, V9, DOI 10.3389/fncel.2015.00505

Harms AS, 2018, EXP NEUROL, V300, P179, DOI 10.1016/j.expneurol.2017.11.010

Hauser Stephen L, 2008, N Engl J Med, V358, P676, DOI 10.1056/NEJMoa0706383

Hawker K, 2009, ANN NEUROL, V66, P460, DOI 10.1002/ana.21867

He DW, 2021, FRONT IMMUNOL, V12, DOI 10.3389/fimmu.2021.619761

He P, 2013, PLOS ONE, V8, DOI 10.1371/journal.pone.0055091

Henkel JS, 2009, J NEUROIMMUNE PHARM, V4, P389, DOI 10.1007/s11481-009-9171-5

Himmerich H, 2019, FRONT PSYCHIATRY, V10, DOI 10.3389/fpsyt.2019.00030

Hirsch EC, 2021, MOVEMENT DISORD, V36, P16, DOI 10.1002/mds.28075

Holbrook JA, 2021, FRONT PHARMACOL, V12, DOI 10.3389/fphar.2021.643254

Howard R, 2020, JAMA NEUROL, V77, P164, DOI 10.1001/jamaneurol.2019.3762

Hu QS, 2016, TRANSL NEURODEGENER, V5, DOI 10.1186/s40035-016-0060-6

Hwang CJ, 2016, ONCOTARGET, V7, P46943, DOI 10.18632/oncotarget.10504

Iori V, 2017, NEUROBIOL DIS, V99, P12, DOI 10.1016/j.nbd.2016.12.007

Jaiswal P, 2020, J BIOCHEM MOL TOXIC, V34, DOI 10.1002/jbt.22575

Janda E, 2018, FRONT MOL NEUROSCI, V11, DOI 10.3389/fnmol.2018.00144

Ji J, 2018, AGING CELL, V17, DOI 10.1111/acel.12774

Ji Y, 2020, MOLECULES, V25, DOI 10.3390/molecules25215087

Jiang LL, 2016, J NEUROINFLAMM, V13, DOI 10.1186/s12974-016-0573-z

Jimenez S, 2008, J NEUROSCI, V28, P11650, DOI 10.1523/JNEUROSCI.3024-08.2008

Jones KA, 2018, BRAIN BEHAV IMMUN, V67, P299, DOI 10.1016/j.bbi.2017.09.006

Kalra J, 2016, PHARMACOL BIOCHEM BE, V146, P1, DOI 10.1016/j.pbb.2016.04.002

Kamata H, 2005, CELL, V120, P649, DOI 10.1016/j.cell.2004.12.041

Kanamori A, 2010, BRAIN, V133, P2612, DOI 10.1093/brain/awq105

Kang R, 2014, MOL ASPECTS MED, V40, P1, DOI 10.1016/j.mam.2014.05.001

Kappos L, 2014, LANCET NEUROL, V13, P353, DOI 10.1016/S1474-4422(14)70028-6

Karlmark KR, 2012, EUR J MICROBIOL IMMU, V2, P97, DOI 10.1556/EuJMI.2.2012.2.1

Karson A, 2021, EPILEPSY BEHAV, V115, DOI 10.1016/j.yebeh.2020.107532

Karumuri SB, 2019, TOXICOL REP, V6, P1295, DOI 10.1016/j.toxrep.2019.11.005

Katsumoto A, 2014, J IMMUNOL, V193, P2615, DOI 10.4049/jimmunol.1400716

Katyal J, 2015, EPILEPSY BEHAV, V44, P98, DOI 10.1016/j.yebeh.2014.12.032

Kaur D, 2019, INFLAMMOPHARMACOLOGY, V27, P663, DOI 10.1007/s10787-019-00580-x

Kim BW, 2015, NEUROPHARMACOLOGY, V97, P46, DOI 10.1016/j.neuropharm.2015.04.037

Kim HG, 2010, BASIC CLIN PHARMACOL, V107, P958, DOI 10.1111/j.1742-7843.2010.00612.x

Kim YE, 2017, NEUROPHARMACOLOGY, V117, P21, DOI 10.1016/j.neuropharm.2017.01.025

Kimizu T, 2020, EPILEPSY BEHAV, V109, DOI 10.1016/j.yebeh.2020.107116

Kolodziejczak M, 2015, ACS CHEM NEUROSCI, V6, P1219, DOI 10.1021/cn5003489

Kong ZH, 2017, J MOL NEUROSCI, V63, P385, DOI 10.1007/s12031-017-0989-7

Kostoula C, 2019, BRAIN BEHAV IMMUN, V81, P598, DOI 10.1016/j.bbi.2019.07.021

Kunnanayaka V, 2018, NEUROL INDIA, V66, P385, DOI 10.4103/0028-3886.227281

Kurt S, 2021, FUND CLIN PHARMACOL, V35, P861, DOI 10.1111/fcp.12651

Lamkanfi M, 2014, CELL, V157, P1013, DOI 10.1016/j.cell.2014.04.007

Lampron A, 2013, NEURON, V78, P214, DOI 10.1016/j.neuron.2013.04.005

Laurent C, 2018, BIOMED J, V41, P21, DOI 10.1016/j.bj.2018.01.003

Lecca D, 2015, NEUROSCIENCE, V302, P23, DOI 10.1016/j.neuroscience.2015.04.026

Lee EY, 2012, TOXICOL LETT, V213, P332, DOI 10.1016/j.toxlet.2012.07.016

Lee HK, 2007, SCIENCE, V315, P1398, DOI 10.1126/science.1136880

Lee KM, 2016, J NEUROSCI RES, V94, P865, DOI 10.1002/jnr.23764

Leng FD, 2021, NAT REV NEUROL, V17, P157, DOI 10.1038/s41582-020-00435-y

Leo A, 2020, NEUROTHERAPEUTICS, V17, P2004, DOI 10.1007/s13311-020-00893-8

Li L, 2021, BRAIN BEHAV IMMUN, V91, P740, DOI 10.1016/j.bbi.2020.10.007

Li S, 2019, EPILEPSY RES, V151, P1, DOI 10.1016/j.eplepsyres.2019.01.010

Li YH, 2007, BRAIN, V130, P490, DOI 10.1093/brain/awl273

Liang JF, 2008, BRAIN RES, V1210, P11, DOI 10.1016/j.brainres.2008.03.012

Lim HW, 2015, BRAIN RES BULL, V112, P25, DOI 10.1016/j.brainresbull.2015.01.004

Lin WJ, 2014, AUTOPHAGY, V10, P1692, DOI 10.4161/auto.36076

Liu D, 2013, NEUROBIOL AGING, V34, P1564, DOI 10.1016/j.neurobiolaging.2012.11.020

Liu DH, 2019, NEUROCHEM RES, V44, P2556, DOI 10.1007/s11064-019-02874-6

Liu QY, 2018, MOL IMMUNOL, V103, P115, DOI 10.1016/j.molimm.2018.09.010

Liu R, 2020, NEUROCHEM RES, V45, P2516, DOI 10.1007/s11064-020-03109-9

Loane DJ, 2016, EXP NEUROL, V275, P316, DOI 10.1016/j.expneurol.2015.08.018

Löscher W, 2020, PHARMACOL REV, V72, P606, DOI 10.1124/pr.120.019539

Lonnemann N, 2020, P NATL ACAD SCI USA, V117, P32145, DOI 10.1073/pnas.2009680117

Luft JG, 2019, N-S ARCH PHARMACOL, V392, P1347, DOI 10.1007/s00210-019-01675-6

Maan G, 2020, CURR TOP MED CHEM, V20, P1169, DOI 10.2174/1568026620666200416085330

de Moraes RCM, 2020, EXP GERONTOL, V141, DOI 10.1016/j.exger.2020.111097

Maier A, 2015, PLOS ONE, V10, DOI 10.1371/journal.pone.0139684

Mandal M, 2020, J DRUG DELIV SCI TEC, V56, DOI 10.1016/j.jddst.2020.101559

Mandal M, 2020, J BIOCHEM MOL TOXIC, V34, DOI 10.1002/jbt.22478

Mantovani A, 2002, TRENDS IMMUNOL, V23, P549, DOI 10.1016/S1471-4906(02)02302-5

Marques NF, 2018, NEUROTOX RES, V33, P549, DOI 10.1007/s12640-017-9840-8

Massa MG, 2017, AM J PATHOL, V187, P1613, DOI 10.1016/j.ajpath.2017.03.006

Mei M, 2019, NEUROPHARMACOLOGY, V160, DOI 10.1016/j.neuropharm.2019.107758

Mhillaj E, 2020, FRONT CELL DEV BIOL, V8, DOI 10.3389/fcell.2020.561179

Mhillaj E, 2018, NEUROSCIENCE, V372, P58, DOI 10.1016/j.neuroscience.2017.12.046

Miguel-Alvarez M, 2015, DRUG AGING, V32, P139, DOI 10.1007/s40266-015-0239-z

Mishra Awanish, 2019, J Epilepsy Res, V9, P119, DOI 10.14581/jer.19012

Mishra A, 2016, NEUROSCIENCE, V339, P319, DOI 10.1016/j.neuroscience.2016.10.010

Miyazaki I, 2020, CELLS-BASEL, V9, DOI 10.3390/cells9122623

Montoliu C., 2015, Neuroimmunology and Neuroinflammation, V2, P138, DOI DOI 10.4103/2347-8659.160845

Morales-Sosa M, 2018, PHARMACOL BIOCHEM BE, V170, P79, DOI 10.1016/j.pbb.2018.05.007

Morihara T, 2005, NEUROPSYCHOPHARMACOL, V30, P1111, DOI 10.1038/sj.npp.1300668

Moussa C, 2017, J NEUROINFLAMM, V14, DOI 10.1186/s12974-016-0779-0

Mouton-Liger F, 2018, GLIA, V66, P1736, DOI 10.1002/glia.23337

Nakanishi H, 2009, BEHAV BRAIN RES, V201, P1, DOI 10.1016/j.bbr.2009.02.001

Nimmerjahn A, 2005, SCIENCE, V308, P1314, DOI 10.1126/science.1110647

Niranjan R, 2016, INDIAN J GASTROENTER, V35, P72, DOI 10.1007/s12664-016-0629-0

Ota M, 2019, NEUROSCI LETT, V690, P232, DOI 10.1016/j.neulet.2018.10.048

Panicker N, 2021, J CELL BIOL, V220, DOI 10.1083/jcb.202012095

Park G, 2013, ACTA PHARMACOL SIN, V34, P1131, DOI 10.1038/aps.2013.57

Park J, 2019, CYTOTHERAPY, V21, P671, DOI 10.1016/j.jcyt.2019.04.054

Parsons CG, 2007, NEUROPHARMACOLOGY, V53, P699, DOI 10.1016/j.neuropharm.2007.07.013

Pasqualetti P, 2009, AGING CLIN EXP RES, V21, P102, DOI 10.1007/BF03325217

Paudel YN, 2018, FRONT NEUROSCI-SWITZ, V12, DOI 10.3389/fnins.2018.00628

Pauletti A., 2019, BRAIN, V142, pe39, DOI 10.1093/brain/awz130

Peng JT, 2019, NEUROCHEM RES, V44, P2566, DOI 10.1007/s11064-019-02875-5

Perea G, 2009, TRENDS NEUROSCI, V32, P421, DOI 10.1016/j.tins.2009.05.001

Piccioni G, 2021, INT J MOL SCI, V22, DOI 10.3390/ijms22052342

Poewe W, 2017, NAT REV DIS PRIMERS, V3, DOI 10.1038/nrdp.2017.13

Polascheck N, 2010, EXP NEUROL, V224, P219, DOI 10.1016/j.expneurol.2010.03.014

Poly TN, 2019, EUR J CLIN PHARMACOL, V75, P99, DOI 10.1007/s00228-018-2561-y

Prabowo AS, 2013, BRAIN PATHOL, V23, P45, DOI 10.1111/j.1750-3639.2012.00616.x

Qi YJ, 2018, NEUROBIOL DIS, V114, P24, DOI 10.1016/j.nbd.2018.02.016

Qian X, 2019, BRAIN RES, V1714, P111, DOI 10.1016/j.brainres.2019.02.028

Quamar S., 2019, Toxicol. Res. Appl, V3, DOI [10.1177/2397847319844782, DOI 10.1177/2397847319844782]

Radu BM, 2017, EPILEPSY RES, V131, P15, DOI 10.1016/j.eplepsyres.2017.02.003

Rana A, 2018, J NEUROINFLAMM, V15, DOI 10.1186/s12974-018-1192-7

Ransohoff RM, 2016, NAT NEUROSCI, V19, P987, DOI 10.1038/nn.4338

Ravikumar B, 2010, PHYSIOL REV, V90, P1383, DOI 10.1152/physrev.00030.2009

Ravizza T, 2008, NEUROBIOL DIS, V29, P142, DOI 10.1016/j.nbd.2007.08.012

Ravizza T, 2018, BRAIN BEHAV IMMUN, V72, P14, DOI 10.1016/j.bbi.2017.10.008

Reines SA, 2004, NEUROLOGY, V62, P66, DOI 10.1212/WNL.62.1.66

Reksidler AB, 2007, EUR J PHARMACOL, V560, P163, DOI 10.1016/j.ejphar.2006.12.032

Ren B, 2015, J NEUROL SCI, V348, P142, DOI 10.1016/j.jns.2014.11.026

Reynolds AD, 2010, J IMMUNOL, V184, P2261, DOI 10.4049/jimmunol.0901852

Richard S A., 2017, Journal of Behavioral and Brain Science, V7, DOI [DOI 10.4236/JBBS.2017.72006, 10.4236/JBBS.2017.72006]

Rivers-Auty J, 2020, BRAIN COMMUN, V2, DOI 10.1093/braincomms/fcaa109

Rocha EM, 2018, NEUROBIOL DIS, V109, P249, DOI 10.1016/j.nbd.2017.04.004

Rock RB, 2004, CLIN MICROBIOL REV, V17, P942, DOI 10.1128/CMR.17.4.942-964.2004

Roseti C, 2015, NEUROBIOL DIS, V82, P311, DOI 10.1016/j.nbd.2015.07.003

Russo MV, 2016, SCIENCE, V353, P783, DOI 10.1126/science.aaf6260

Sachdeva AK, 2015, J NUTR BIOCHEM, V26, P736, DOI 10.1016/j.jnutbio.2015.01.012

Saini N, 2020, BRAIN BEHAV IMMUN, V90, P108, DOI 10.1016/j.bbi.2020.08.001

Sánchez-Sarasúa S, 2020, INT J MOL SCI, V21, DOI 10.3390/ijms21228751

Saresella M, 2016, MOL NEURODEGENER, V11, DOI 10.1186/s13024-016-0088-1

Sawada M, 2009, PARKINSONISM RELAT D, V15, pS39, DOI 10.1016/S1353-8020(09)70011-2

Sawikr Y, 2017, ADV PROTEIN CHEM STR, V108, P33, DOI 10.1016/bs.apcsb.2017.02.001

Scaffidi P, 2002, NATURE, V418, P191, DOI 10.1038/nature00858

Schlichtiger J, 2010, BRIT J PHARMACOL, V160, P1062, DOI 10.1111/j.1476-5381.2010.00765.x

Schmitt C, 2012, J NEUROINFLAMM, V9, DOI 10.1186/1742-2094-9-187

Segal BM, 1996, J EXP MED, V184, P771, DOI 10.1084/jem.184.2.771

Shabab T, 2017, INT J NEUROSCI, V127, P624, DOI 10.1080/00207454.2016.1212854

Sharma R, 2019, BRAIN SCI, V9, DOI 10.3390/brainsci9110318

Shi JJ, 2017, TRENDS BIOCHEM SCI, V42, P245, DOI 10.1016/j.tibs.2016.10.004

Shi XJ, 2016, MOL MED REP, V14, P2397, DOI 10.3892/mmr.2016.5573

Shimada T, 2014, MEDIAT INFLAMM, V2014, DOI 10.1155/2014/901902

Sil S, 2014, NEUROIMMUNOMODULAT, V21, P304, DOI 10.1159/000357735

Silk M, 2018, NEUROLOGY, V90

Simard AR, 2006, NEURON, V49, P489, DOI 10.1016/j.neuron.2006.01.022

Simpson DSA, 2020, ANTIOXIDANTS-BASEL, V9, DOI 10.3390/antiox9080743

Solito E, 2012, FRONT PHARMACOL, V3, DOI 10.3389/fphar.2012.00014

Sonderegger I, 2008, J EXP MED, V205, P2281, DOI 10.1084/jem.20071119

Stuve O, 2020, THER ADV NEUROL DISO, V13, DOI 10.1177/1756286420935676

Sung S, 2004, AM J PATHOL, V165, P2197, DOI 10.1016/S0002-9440(10)63269-5

Takaki J, 2012, J NEUROINFLAMM, V9, DOI 10.1186/1742-2094-9-275

Takeuchi H, 2008, EXP NEUROL, V214, P144, DOI 10.1016/j.expneurol.2008.08.001

Takeuchi H, 2006, J BIOL CHEM, V281, P21362, DOI 10.1074/jbc.M600504200

Temp FR, 2017, EUR J PHARMACOL, V810, P15, DOI 10.1016/j.ejphar.2017.05.013

Terrone G, 2020, NEUROPHARMACOLOGY, V167, DOI 10.1016/j.neuropharm.2019.107742

Tezel G, 2000, J NEUROSCI, V20, P8693

Thal LJ, 2005, NEUROPSYCHOPHARMACOL, V30, P1204, DOI 10.1038/sj.npp.1300690

Tiwari S, 2019, INT J NANOMED, V14, P5541, DOI 10.2147/IJN.S200490

Trandafir CC, 2015, NEUROSCIENCE, V284, P601, DOI 10.1016/j.neuroscience.2014.10.021

Trempe JF, 2013, FRONT NEUROL, V4, DOI 10.3389/fneur.2013.00038

Tsai MC, 2012, J NEUROSCI, V32, P1528, DOI 10.1523/JNEUROSCI.5232-11.2012

Turrin NP, 2004, NEUROBIOL DIS, V16, P321, DOI 10.1016/j.nbd.2004.03.010

Vaccari JPDR, 2008, J NEUROSCI, V28, P3404, DOI 10.1523/JNEUROSCI.0157-08.2008

Van Bergen NJ, 2015, PLOS ONE, V10, DOI 10.1371/journal.pone.0140919

Van Dam D, 2010, J PSYCHOPHARMACOL, V24, P383, DOI 10.1177/0269881108097630

van Stuijvenberg M, 1998, PEDIATRICS, V102, DOI 10.1542/peds.102.5.e51

Vanover KE, 2008, PHARMACOL BIOCHEM BE, V90, P540, DOI 10.1016/j.pbb.2008.04.010

Varvel NH, 2009, J CLIN INVEST, V119, P3692, DOI 10.1172/JCI39716

Verma V, 2020, BRAIN SCI, V10, DOI 10.3390/brainsci10120923

Vezzani A, 2020, EPILEPSY CURR, V20, p40S, DOI 10.1177/1535759720948900

Vezzani A, 2019, NAT REV NEUROL, V15, P459, DOI 10.1038/s41582-019-0217-x

Vezzani A, 2011, BIOMARK MED, V5, P607, DOI [10.2217/BMM.11.61, 10.2217/bmm.11.61]

Viviani B, 2003, J NEUROSCI, V23, P8692

Voet S, 2019, EMBO MOL MED, V11, DOI 10.15252/emmm.201810248

Wallisch JS, 2017, NEUROCRIT CARE, V27, P44, DOI 10.1007/s12028-017-0378-7

Walsh JG, 2014, NAT REV NEUROSCI, V15, P84, DOI 10.1038/nrn3638

Wattananit S, 2016, J NEUROSCI, V36, P4182, DOI 10.1523/JNEUROSCI.4317-15.2016

Webers A, 2020, IMMUNOL CELL BIOL, V98, P28, DOI 10.1111/imcb.12301

Webster KM, 2019, EXP NEUROL, V320, DOI 10.1016/j.expneurol.2019.112979

Webster KM, 2017, J NEUROINFLAMM, V14, DOI 10.1186/s12974-016-0786-1

West AP, 2017, NAT REV IMMUNOL, V17, P363, DOI 10.1038/nri.2017.21

Wilcock GK, 2008, LANCET NEUROL, V7, P483, DOI 10.1016/S1474-4422(08)70090-5

Wilkins HM, 2017, FRONT IMMUNOL, V8, DOI 10.3389/fimmu.2017.00508

Wu WW, 2015, PLOS ONE, V10, DOI 10.1371/journal.pone.0132499

Yabuki Y, 2014, NEUROSCIENCE, V259, P126, DOI 10.1016/j.neuroscience.2013.11.051

Yang C, 2019, INT IMMUNOPHARMACOL, V75, DOI 10.1016/j.intimp.2019.05.036

Yang JS, 2017, INFLAMMOPHARMACOLOGY, V25, P471, DOI 10.1007/s10787-017-0348-x

Yang XJ, 2011, INVEST OPHTH VIS SCI, V52, P8442, DOI 10.1167/iovs.11-8152

Yatsiv I, 2002, J CEREBR BLOOD F MET, V22, P971, DOI 10.1097/00004647-200208000-00008

Yeo IJ, 2020, CELL DEATH DIS, V11, DOI 10.1038/s41419-020-2619-5

Yin J, 2018, MOL NEUROBIOL, V55, P1977, DOI 10.1007/s12035-017-0467-9

You SJ, 2008, EUR J PAEDIATR NEURO, V12, P314, DOI 10.1016/j.ejpn.2007.09.003

Yu L, 2006, P NATL ACAD SCI USA, V103, P4952, DOI 10.1073/pnas.0511288103

Yu Y, 2020, MOL NEUROBIOL, V57, P2830, DOI 10.1007/s12035-020-01923-4

Yuan C, 2021, OXID MED CELL LONGEV, V2021, DOI 10.1155/2021/8887716

Yue J, 2020, J NEUROINFLAMM, V17, DOI 10.1186/s12974-020-1718-7

Zhang CX, 2018, PATHOL RES PRACT, V214, P1765, DOI 10.1016/j.prp.2018.08.011

Zhang LY, 2021, NEURAL REGEN RES, V16, P1660, DOI 10.4103/1673-5374.303045

Zhang SH, 2019, EPILEPSY RES, V156, DOI 10.1016/j.eplepsyres.2019.106183

Zhang W, 2006, FASEB J, V20, P2496, DOI 10.1096/fj.06-6006com

Zhang ZJ, 2014, PHARMACOL BIOCHEM BE, V122, P191, DOI 10.1016/j.pbb.2014.03.028

Zhou TT, 2018, MOL MED REP, V17, P4883, DOI 10.3892/mmr.2018.8470

Zhu XB, 2019, INFLAMMOPHARMACOLOGY, V27, P1143, DOI 10.1007/s10787-019-00592-7

NR 299

TC 62

Z9 65

U1 13

U2 41

PU SPRINGER/PLENUM PUBLISHERS

PI NEW YORK

PA 233 SPRING ST, NEW YORK, NY 10013 USA

SN 0885-7490

EI 1573-7365

J9 METAB BRAIN DIS

JI Metab. Brain Dis.

PD OCT

PY 2021

VL 36

IS 7

BP 1591

EP 1626

DI 10.1007/s11011-021-00806-4

EA AUG 2021

PG 36

WC Endocrinology & Metabolism; Neurosciences

WE Science Citation Index Expanded (SCI-EXPANDED)

SC Endocrinology & Metabolism; Neurosciences & Neurology

GA UT8VL

UT WOS:000684770300001

PM 34387831

DA 2024-08-12

ER

PT J

AU Lin, QY

Hou, S

Dai, YY

Jiang, N

Lin, YJ

AF Lin, Qiuyu

Hou, Sen

Dai, Yuyin

Jiang, Nan

Lin, Yingjie

TI Monascin exhibits neuroprotective effects in rotenone model of

Parkinson's disease via antioxidation and anti-neuroinflammation

SO NEUROREPORT

LA English

DT Article

DE monascin; neuroinflammation; oxidative stress; Parkinson's disease

ID PPAR-GAMMA; OXIDATIVE STRESS; MITOCHONDRIAL DYSFUNCTION; INTRACEREBRAL

HEMORRHAGE; INFLAMMATION; EXPRESSION; NRF2

AB Increasing evidence verified that oxidative stress and neuroinflammatory response exacerbates motor deficits and increases neuronal loss in several rodent models of Parkinson's disease. In the present study, we explore the neuroprotective effects of monascin in a rotenone-induced Parkinson's disease model as well as the underlying mechanisms. Our results showed that monascin remarkedly attenuated behavioral impairments and the depletion of dopaminergic neurons induced by rotenone in the rats. Besides, monascin decreased the levels of pro-inflammatory factors such as interleukin-1 beta, interleukin-6, tumor necrosis factor-alpha and oxidative stress marker malondialdehyde while promoted the expression of superoxide dismutase, glutathione peroxidase and other antioxidant factors. Further detection of the expression of related proteins showed that monascin significantly promoted the expression of proliferator-activated receptor-gamma, F-E2-related factor 2 and heme oxygenase-1, but inhibited the expression of NF-kappa B. What's more, levels of growth factors that are essential for neuronal and synaptic function were increased under the effects of monascin. All in all, our results revealed that monascin exerted neuroprotective effects in rotenone model of Parkinson's disease via antioxidation and anti-neuroinflammation.

C1 [Lin, Qiuyu; Lin, Yingjie] Jilin Univ, Coll Chem, Dept Organ Chem, 2699 Qianjin St, Changchun 130021, Jilin, China.

[Lin, Qiuyu; Hou, Sen; Dai, Yuyin; Jiang, Nan] First Hosp Jilin Univ, Dept Nucl Med, Changchun 130021, Jilin, China.

C3 Jilin University; Jilin University

RP Lin, YJ (corresponding author), Jilin Univ, Coll Chem, Dept Organ Chem, 2699 Qianjin St, Changchun 130021, Jilin, China.

EM yingjie5253@sina.com

RI yang, le/KFB-5420-2024; QIU, LI/JPK-7397-2023

OI Lin, Yingjie/0000-0001-8492-6609

CR Campolo M, 2017, ANTIOXID REDOX SIGN, V27, P453, DOI 10.1089/ars.2016.6800

Carta AR, 2013, CURR DRUG TARGETS, V14, P743, DOI 10.2174/1389450111314070004

Ding YQ, 2018, FRONT AGING NEUROSCI, V10, DOI 10.3389/fnagi.2018.00246

Dursun E, 2015, J NEUROIMMUNOL, V283, P50, DOI 10.1016/j.jneuroim.2015.04.014

Gelders G, 2018, J IMMUNOL RES, V2018, DOI 10.1155/2018/4784268

Gratwicke J, 2015, BRAIN, V138, P1454, DOI 10.1093/brain/awv104

Hauser DN, 2013, NEUROBIOL DIS, V51, P35, DOI 10.1016/j.nbd.2012.10.011

Hsu WH, 2014, FOOD FUNCT, V5, P1334, DOI 10.1039/c3fo60575b

Hsu WH, 2014, J AGR FOOD CHEM, V62, P5337, DOI 10.1021/jf501373a

Hsu WH, 2012, FOOD CHEM TOXICOL, V50, P1178, DOI 10.1016/j.fct.2012.02.029

Islam MT, 2017, NEUROL RES, V39, P73, DOI 10.1080/01616412.2016.1251711

Jing HR, 2017, PLOS ONE, V12, DOI 10.1371/journal.pone.0169560

Joers V, 2017, PROG NEUROBIOL, V155, P57, DOI 10.1016/j.pneurobio.2016.04.006

Kang SY, 2017, NEUROSCI LETT, V642, P20, DOI 10.1016/j.neulet.2017.01.063

Nicholatos JW, 2018, ACTA NEUROPATHOL COM, V6, DOI 10.1186/s40478-018-0625-y

Patakova P, 2013, J IND MICROBIOL BIOT, V40, P169, DOI 10.1007/s10295-012-1216-8

Picillo M, 2019, PLOS ONE, V14, DOI 10.1371/journal.pone.0223621

Puspita L, 2017, MOL BRAIN, V10, DOI 10.1186/s13041-017-0340-9

Ramsey CP, 2007, J NEUROPATH EXP NEUR, V66, P75, DOI 10.1097/nen.0b013e31802d6da9

Sarkar S, 2016, INT J MOL SCI, V17, DOI 10.3390/ijms17060904

Sveinbjornsdottir S, 2016, J NEUROCHEM, V139, P318, DOI 10.1111/jnc.13691

Szanto A, 2010, IMMUNITY, V33, P699, DOI 10.1016/j.immuni.2010.11.009

Vivekanantham S, 2015, INT J NEUROSCI, V125, P717, DOI 10.3109/00207454.2014.982795

Wang GQ, 2018, BEHAV NEUROL, V2018, DOI 10.1155/2018/7646104

Wang J, 2017, BRAIN RES BULL, V134, P24, DOI 10.1016/j.brainresbull.2017.06.018

Xu W, 2017, CELL PHYSIOL BIOCHEM, V44, P2091, DOI 10.1159/000485947

Zhang X, 2017, SCI REP-UK, V7, DOI 10.1038/s41598-017-07442-y

Zhao XR, 2015, CNS NEUROSCI THER, V21, P357, DOI 10.1111/cns.12350

Zheng G, 2018, FOOD FUNCT, V9, P1454, DOI 10.1039/c7fo01892d

NR 29

TC 10

Z9 11

U1 2

U2 34

PU LIPPINCOTT WILLIAMS & WILKINS

PI PHILADELPHIA

PA TWO COMMERCE SQ, 2001 MARKET ST, PHILADELPHIA, PA 19103 USA

SN 0959-4965

EI 1473-558X

J9 NEUROREPORT

JI Neuroreport

PD JUN 7

PY 2020

VL 31

IS 9

BP 637

EP 643

DI 10.1097/WNR.0000000000001467

PG 7

WC Neurosciences

WE Science Citation Index Expanded (SCI-EXPANDED)

SC Neurosciences & Neurology

GA LT0RN

UT WOS:000536784200002

PM 32427711

DA 2024-08-12

ER

PT J

AU Wakade, C

Giri, B

Malik, A

Khodadadi, H

Morgan, JC

Chong, RK

Baban, B

AF Wakade, Chandramohan

Giri, Banabihari

Malik, Aneeq

Khodadadi, Hesam

Morgan, John C.

Chong, Raymond K.

Baban, Babak

TI Niacin modulates macrophage polarization in Parkinson's disease

SO JOURNAL OF NEUROIMMUNOLOGY

LA English

DT Article

DE Niacin; Macrophage polarization; GPR109A; Parkinson's disease

ID ACTIVATION; RECEPTORS

AB Neuroinflammation remains a central piece in Parkinson's disease (PD) pathophysiology. However, mechanisms by which PD links to the neuroinflammation remain elusive.

Here, for the first time, we report that lower dose of niacin in PD patients may affect macrophage polarization from M1 (pro-inflammatory) to M2 (counter-inflammatory) profile through the niacin receptor GPR109A. Skew in the peripheral macrophages were accompanied by improved quality of life assessments in patients. Low dose niacin supplementation may be beneficial in PD, boosting anti-inflammatory processes and suppressing inflammation. Varied niacin dosages for longer durations may further reveal the potential role of anti-inflammatory interventions in PD progression.

C1 [Wakade, Chandramohan] Charlie Norwood Veteran Affairs Med Ctr, Augusta, GA 30904 USA.

[Wakade, Chandramohan; Giri, Banabihari; Chong, Raymond K.] Augusta Univ, Coll Allied Hlth Sci, Dept Phys Therapy, Augusta, GA USA.

[Malik, Aneeq; Khodadadi, Hesam; Baban, Babak] Augusta Univ, Dent Coll Georgia, Dept Oral Biol, Augusta, GA 30904 USA.

[Morgan, John C.] Augusta Univ, Med Coll Georgia, Dept Neurol,Program, Parkinsons Fdn Ctr Excellence,Movement Disorders, Augusta, GA USA.

[Baban, Babak] Augusta Univ, Med Coll Georgia, Dept Neurol, Augusta, GA USA.

C3 US Department of Veterans Affairs; Veterans Health Administration (VHA);

University System of Georgia; Augusta University; University System of

Georgia; Augusta University; University System of Georgia; Augusta

University; University System of Georgia; Augusta University

RP Wakade, C (corresponding author), Charlie Norwood Veteran Affairs Med Ctr, Augusta, GA 30904 USA.; Baban, B (corresponding author), Augusta Univ, Dent Coll Georgia, Dept Oral Biol, Augusta, GA 30904 USA.

EM cwakade@augusta.edu; bbaban@augusta.edu

OI Chong, Raymond/0000-0002-2706-1300; Khodadadi, Hesam/0000-0003-3651-4305

FU Department of Veterans Affairs, Rehabilitation Research and Development

[I01RX001613]; Augusta University (AU) [PSRP00075]

FX This study was partly supported by the Merit Review AwardI01RX001613

issued by the Department of Veterans Affairs, Rehabilitation Research

and Development, and the PSRP00075 intramural funding by Augusta

University (AU).

CR Bartels AL, 2010, PARKINSONISM RELAT D, V16, P57, DOI 10.1016/j.parkreldis.2009.05.005

Blad CC, 2012, NAT REV DRUG DISCOV, V11, P603, DOI 10.1038/nrd3777

Chaudhuri KR, 2002, J NEUROL NEUROSUR PS, V73, P629, DOI 10.1136/jnnp.73.6.629

Chong RKY, 2011, EUR J NEUROL, V18, P260, DOI 10.1111/j.1468-1331.2010.03115.x

Feingold KR, 2014, J LIPID RES, V55, P2501, DOI 10.1194/jlr.M050955

Ganapathy V, 2013, CURR OPIN PHARMACOL, V13, P869, DOI 10.1016/j.coph.2013.08.006

Giri B, 2007, EUR J IMMUNOL, V37, P2104, DOI 10.1002/eji.200636680

Jenkinson C, 2007, PARKINSONISM RELAT D, V13, P22, DOI 10.1016/j.parkreldis.2006.06.006

Lee H, 2017, BIOCHEM SOC T, V45, P131, DOI 10.1042/BST20160262

Moehle MS, 2015, NEUROSCIENCE, V302, P59, DOI 10.1016/j.neuroscience.2014.11.018

Perry V.H., 2012, COLD SPRING HARB PER

Salem HA, 2017, SCI REP-UK, V7, DOI 10.1038/s41598-017-07280-y

Singh N, 2014, IMMUNITY, V40, P128, DOI 10.1016/j.immuni.2013.12.007

Stranahan AM, 2016, J CEREBR BLOOD F MET, V36, P2108, DOI 10.1177/0271678X16642233

Wakade C, 2014, J NEUROL SCI, V347, P34, DOI 10.1016/j.jns.2014.10.024

Wakade C, 2014, PLOS ONE, V9, DOI 10.1371/journal.pone.0109818

Zhao YL, 2014, PLOS ONE, V9, DOI 10.1371/journal.pone.0106867

NR 17

TC 48

Z9 54

U1 5

U2 23

PU ELSEVIER SCIENCE BV

PI AMSTERDAM

PA PO BOX 211, 1000 AE AMSTERDAM, NETHERLANDS

SN 0165-5728

EI 1872-8421

J9 J NEUROIMMUNOL

JI J. Neuroimmunol.

PD JUL 15

PY 2018

VL 320

BP 76

EP 79

DI 10.1016/j.jneuroim.2018.05.002

PG 4

WC Immunology; Neurosciences

WE Science Citation Index Expanded (SCI-EXPANDED)

SC Immunology; Neurosciences & Neurology

GA GI8CH

UT WOS:000434748900010

PM 29759143

OA hybrid

DA 2024-08-12

ER

PT J

AU Kiçik, A

Tüzün, E

Erdogdu, E

Bilgiç, B

Tüfekçioglu, Z

Öztürk-Idik, E

Hanagasi, H

Gürvit, H

AF Kicik, Ani

Tuzun, Erdem

Erdogdu, Emel

Bilgic, Basar

Tufekcioglu, Zeynep

Ozturk-Idik, Esin

Hanagasi, Hasmet

Gurvit, Hakan

TI Neuroinflammation Mediators are Reduced in Sera of Parkinson's Disease

Patients with Mild Cognitive Impairment

SO NOROPSIKIYATRI ARSIVI-ARCHIVES OF NEUROPSYCHIATRY

LA English

DT Article

DE Parkinson's disease; neuroinflammation; mild cognitive impairment;

inflammasome; IL-18

ID DIAGNOSTIC-CRITERIA

AB Introduction: Cognitive impairment is common in Parkinson's disease (PD) and PD patients with mild cognitive impairment (PD-MCI) are at increased risk of developing Parkinson's disease dementia (PDD). Reliable biomarkers are required for objective identification of cognitive decline in PD. In this pilot study, serum levels of well-known mediators of neuroinflammation were measured in PD patients with or without MCI to find out the involvement of neuroinflammation and microglial activation in PD-MCI.

Methods: 36 PD-MCI, 25 PD patients with normal cognition (PD-NC) and 19 healthy controls were recruited. Serum levels of NLR family pyrin domain containing 1 (NLRP1), NLRP3, caspase-1, NF-kB, IL-1b and IL-18 were measured by ELISA and a panel of neuropsychological tests was administered.

Results: PD-MCI patients showed significantly reduced levels of NF-kB, IL-lb and IL-18, whereas NLRP1, NLRP3 and caspase-1 levels were comparable among PD-NC and PD-MCI patients. IL-18 levels were positively correlated with Addenbrooke's Cognitive Examination-Revised and Symbol Digit Modalities Test scores.

Conclusion: Levels of several microglial activation mediators are reduced in PD-MCI patients inferring a protective role to certain inflammation factors against cognitive decline in PD.

C1 [Kicik, Ani] Demiroglu Bilim Univ, Fac Med, Dept Physiol, Istanbul, Turkey.

[Tuzun, Erdem] Istanbul Univ, Aziz Sancar Inst Expt Med, Dept Neurosci, Istanbul, Turkey.

[Erdogdu, Emel] Istanbul Univ, Neuroimaging Unit, Hulusi Behcet Life Sci Res Lab, Istanbul, Turkey.

[Bilgic, Basar; Tufekcioglu, Zeynep; Hanagasi, Hasmet; Gurvit, Hakan] Istanbul Univ, Istanbul Fac Med, Dept Neurol, Istanbul, Turkey.

[Ozturk-Idik, Esin] Bogazici Univ, Biomed Engn Inst, Istanbul, Turkey.

C3 Demiroglu Bilim University; Istanbul University; Istanbul University;

Istanbul University; Bogazici University

RP Kiçik, A (corresponding author), Demiroglu Bilim Univ, Fac Med, Dept Physiol, Istanbul, Turkey.

EM anikicik@gmail.com

RI Gurvit, Hakan/A-7646-2019; Tuzun, Erdem/AAH-9212-2019; Ozturk-Isik,

Esin/K-9771-2018; Erdogdu, Emel/ABH-5980-2020; Hanagasi, Hasmet

A./AAT-9501-2020; tufekcioglu, zeynep/AAO-8826-2021; Bilgic,

Basar/E-4821-2012; tufekcioglu, zeynep/IZD-9782-2023; Kicik,

Ani/AAU-9661-2021

OI Gurvit, Hakan/0000-0003-2908-8475; Ozturk-Isik,

Esin/0000-0002-8997-878X; Erdogdu, Emel/0000-0002-2817-5596; Bilgic,

Basar/0000-0001-6032-0856; Kicik, Ani/0000-0001-6625-6787

FU Scientific Research Projects Coordination Unit of Istanbul University

[21336, 31190]; TUBITAK [115S219]

FX This work was supported by Scientific Research Projects Coordination

Unit of Istanbul University (Project No: 21336 and Project No: 31190)

and TUBITAK (Project No: 115S219).

CR Codolo G, 2013, PLOS ONE, V8, DOI 10.1371/journal.pone.0055375

Dursun E, 2015, J NEUROIMMUNOL, V283, P50, DOI 10.1016/j.jneuroim.2015.04.014

HUGHES AJ, 1992, J NEUROL NEUROSUR PS, V55, P181, DOI 10.1136/jnnp.55.3.181

Karpenko MN, 2018, CELL IMMUNOL, V327, P77, DOI 10.1016/j.cellimm.2018.02.011

Litvan I, 2012, MOVEMENT DISORD, V27, P349, DOI 10.1002/mds.24893

Pickering M, 2007, PROG BRAIN RES, V163, P339, DOI 10.1016/S0079-6123(07)63020-9

Uysal-Cantürk P, 2018, EUR J NEUROL, V25, P148, DOI 10.1111/ene.13467

NR 7

TC 6

Z9 6

U1 1

U2 3

PU TURKISH NEUROPSYCHIATRY ASSOC-TURK NOROPSIKIYATRI DERNEGI

PI ISTANBUL

PA MESRUTIYET MAH HALASKARGAZI CAD SITE APT NO 128-1 SISLI, ISTANBUL,

TURKEY

SN 1300-0667

EI 1309-4866

J9 NOROPSIKIYATRI ARS

JI Noropsikiyatri Ars.

PD MAR

PY 2020

VL 57

IS 1

BP 15

EP 17

DI 10.29399/npa.23624

PG 3

WC Clinical Neurology

WE Science Citation Index Expanded (SCI-EXPANDED)

SC Neurosciences & Neurology

GA LL8OD

UT WOS:000531813000004

PM 32110144

OA Green Published

DA 2024-08-12

ER

PT J

AU Ponce, J

Ulu, A

Hanson, C

Cameron-Smith, E

Bertoni, J

Wuebker, J

Fisher, A

Siu, KC

Marmelat, V

Adamec, J

Bhatti, D

AF Ponce, Jana

Ulu, Arzu

Hanson, Corrine

Cameron-Smith, Erin

Bertoni, John

Wuebker, Jenna

Fisher, Alfred

Siu, Ka-Chun

Marmelat, Vivien

Adamec, Jiri

Bhatti, Danish

TI Role of Specialized Pro-resolving Mediators in Reducing

Neuroinflammation in Neurodegenerative Disorders

SO FRONTIERS IN AGING NEUROSCIENCE

LA English

DT Review

DE neurodegenerative disorder; Parkinson's disease; Alzheimer's disease;

neuroinflammation; specialized pro-resolving lipid mediator (SPM);

omega-3 fatty acids; omega-6 fatty acid; polyunsaturated fatty acids

ID POLYUNSATURATED FATTY-ACIDS; SYNUCLEIN ACTIVATES MICROGLIA; PRORESOLVING

LIPID MEDIATORS; AMYLOID-BETA PHAGOCYTOSIS; parkinson's disease;

DOCOSAHEXAENOIC ACID; ACUTE-INFLAMMATION; ALPHA-SYNUCLEIN;

SUBSTANTIA-NIGRA; URSOLIC ACID

AB Alzheimer's disease (AD) and Parkinson's disease (PD) are neurodegenerative disorders that affect millions of individuals worldwide. As incidence of these conditions increases with age, there will undoubtedly be an increased prevalence of cases in the near future. Neuroinflammation is a hallmark in the development and progression of neurodegenerative disease and prevention or resolution of chronic neuroinflammation may represent a novel approach to treatment. The present review highlights the potential of the anti-inflammatory and pro-resolving effects of polyunsaturated fatty acid (PUFA)-derived mediators (Specialized Pro-resolving Mediators-SPM) in neurodegenerative disorders. PUFA-derived SPM are biosynthesized in response to chemicals produced from acute inflammatory responses. Preclinical studies from both AD and PD models suggest a dysregulation of SPM and their receptors in neurological disorders. Decreased SPM may be due to inadequate substrate, an imbalance between SPM and pro-inflammatory mediators or a disruption in SPM synthesis. SPMs hold great promise for neuroprotection in AD by altering expression of pro-inflammatory genes, modulating macrophage function, serving as a biomarker for AD status, and promoting resolution of neuroinflammation. In PD, data suggest SPM are able to cross the blood-brain barrier, inhibit microglial activation and decrease induced markers of inflammation, possibly as a result of their ability to downregulate NF kappa B signaling pathways. Several in vivo and in vitro studies suggest a benefit from administration of SPMs in both neurodegenerative disorders. However, extrapolation of these outcomes to humans is difficult as no models are able to replicate all features of AD or PD. Minimal data evaluating these PUFA-derived metabolites in humans with neurodegenerative disorders are available and a gap in knowledge exists regarding behavior of SPM and their receptors in patients with these conditions. There is also large gap in our knowledge regarding which lipid mediator would be most effective in which model of AD or PD and how dietary intake or supplementation can impact SPM levels. Future direction should include focused, translational efforts to investigate SPM as an add-on (in addition to standard treatment) or as standalone agents in patients with neurodegenerative disorders.

C1 [Ponce, Jana; Hanson, Corrine; Siu, Ka-Chun] Univ Nebraska, Coll Allied Hlth Profess, Div Med Nutr Educ, Med Ctr, Omaha, NE 68588 USA.

[Ulu, Arzu] Univ Calif Riverside, Sch Med, Div Biomed Sci, Riverside, CA 92521 USA.

[Cameron-Smith, Erin; Bertoni, John; Bhatti, Danish] Univ Nebraska Med Ctr, Dept Neurol Sci, Coll Med, Omaha, NE USA.

[Wuebker, Jenna] Nebraska Med, Dept Pharmaceut & Nutr Care, Omaha, NE USA.

[Fisher, Alfred] Univ Nebraska Med Ctr, Coll Med, Dept Internal Med, Omaha, NE USA.

[Marmelat, Vivien] Univ Nebraska, Dept Biomech, Coll Educ Hlth & Human Sci, Omaha, NE USA.

[Adamec, Jiri] Univ Nebraska, Coll Arts & Sci, Dept Biochem, Lincoln, NE USA.

C3 University of Nebraska System; University of Nebraska Medical Center;

University of California System; University of California Riverside;

University of Nebraska System; University of Nebraska Medical Center;

University of Nebraska System; University of Nebraska Medical Center;

University of Nebraska System; University of Nebraska System; University

of Nebraska Lincoln

RP Ponce, J (corresponding author), Univ Nebraska, Coll Allied Hlth Profess, Div Med Nutr Educ, Med Ctr, Omaha, NE 68588 USA.

EM jana.ponce@unmc.edu

RI Siu, Joseph/N-9940-2016

OI Siu, Joseph/0000-0002-6968-5760; Ponce, Jana/0000-0002-1040-8750

CR Ådén E, 2011, NUTR NEUROSCI, V14, P25, DOI 10.1179/174313211X12966635733312

Alessandri JM, 2004, REPROD NUTR DEV, V44, P509, DOI 10.1051/rnd:2004063

Astorg P, 2004, CANCER CAUSE CONTROL, V15, P367, DOI 10.1023/B:CACO.0000027498.94238.a3

Bannenberg G, 2010, BBA-MOL CELL BIOL L, V1801, P1260, DOI 10.1016/j.bbalip.2010.08.002

Barage SH, 2015, NEUROPEPTIDES, V52, P1, DOI 10.1016/j.npep.2015.06.008

Barcia C, 2003, NEUROTOX RES, V5, P411, DOI 10.1007/BF03033170

Basselin M, 2012, BRAIN RES BULL, V87, P154, DOI 10.1016/j.brainresbull.2011.12.001

BEADLE JB, 1965, J AM OIL CHEM SOC, V42, P90, DOI 10.1007/BF02545036

Benner EJ, 2008, PLOS ONE, V3, DOI 10.1371/journal.pone.0001376

Béraud D, 2013, J NEUROIMMUNE PHARM, V8, P94, DOI 10.1007/s11481-012-9401-0

Birla H, 2019, NEUROMOL MED, V21, P42, DOI 10.1007/s12017-018-08521-7

Buckley CD, 2014, IMMUNITY, V40, P315, DOI 10.1016/j.immuni.2014.02.009

Cardoso HD, 2014, BBA-GEN SUBJECTS, V1840, P1902, DOI 10.1016/j.bbagen.2013.12.023

Carson MJ, 2006, CLIN NEUROSCI RES, V6, P237, DOI 10.1016/j.cnr.2006.09.004

Checkoway Harvey, 2011, IARC Sci Publ, P407

Chen WW, 2016, MOL MED REP, V13, P3391, DOI 10.3892/mmr.2016.4948

Choi SH, 2016, MOL NEURODEGENER, V11, DOI 10.1186/s13024-016-0139-7

Cova I, 2017, J NEUROL SCI, V379, P259, DOI 10.1016/j.jns.2017.06.030

Croisier E, 2005, J NEUROINFLAMM, V2, DOI 10.1186/1742-2094-2-14

de Lau LML, 2006, ARCH NEUROL-CHICAGO, V63, P362, DOI 10.1001/archneur.63.3.noc50312

Dinarello CA, 2000, CHEST, V118, P503, DOI 10.1378/chest.118.2.503

DiSabato DJ, 2016, J NEUROCHEM, V139, P136, DOI 10.1111/jnc.13607

Emre C, 2020, BRAIN PATHOL, V30, P614, DOI 10.1111/bpa.12812

Fiala M, 2015, FASEB J, V29, P2681, DOI 10.1096/fj.14-264218

Franceschi C, 2007, MECH AGEING DEV, V128, P92, DOI 10.1016/j.mad.2006.11.016

Gyengesi E, 2019, FRONT NEUROSCI-SWITZ, V13, DOI 10.3389/fnins.2019.00303

Harris WS, 2009, J NUTR, V139, p804S, DOI 10.3945/jn.108.101329

Hopperton KE, 2018, BRAIN BEHAV IMMUN, V69, P74, DOI 10.1016/j.bbi.2017.11.002

Hou YJ, 2019, NAT REV NEUROL, V15, P565, DOI 10.1038/s41582-019-0244-7

HUGHES AJ, 1992, J NEUROL NEUROSUR PS, V55, P181, DOI 10.1136/jnnp.55.3.181

Iqbal K, 2010, CURR ALZHEIMER RES, V7, P656, DOI 10.2174/156720510793611592

Koivisto H, 2014, J NUTR BIOCHEM, V25, P157, DOI 10.1016/j.jnutbio.2013.09.015

Konnova EA., 2018, Parkinsons disease: pathogenesis and clinical aspects

Kowal SL, 2013, MOVEMENT DISORD, V28, P311, DOI 10.1002/mds.25292

Krashia P, 2019, NAT COMMUN, V10, DOI 10.1038/s41467-019-11928-w

Lee JY, 2020, NAT COMMUN, V11, DOI 10.1038/s41467-020-16080-4

Lee VMY, 2006, NEURON, V52, P33, DOI 10.1016/j.neuron.2006.09.026

Leng FD, 2021, NAT REV NEUROL, V17, P157, DOI 10.1038/s41582-020-00435-y

Levy BD, 2001, NAT IMMUNOL, V2, P612, DOI 10.1038/89759

Levy BD, 2014, ANNU REV PHYSIOL, V76, P467, DOI 10.1146/annurev-physiol-021113-170408

Levy OA, 2009, APOPTOSIS, V14, P478, DOI 10.1007/s10495-008-0309-3

Litteljohn D, 2011, PARKINSONS DIS-US, V2011, DOI 10.4061/2011/713517

Marras C, 2018, NPJ PARKINSONS DIS, V4, DOI 10.1038/s41531-018-0058-0

MCGEER PL, 1988, NEUROLOGY, V38, P1285, DOI 10.1212/WNL.38.8.1285

Menegaz D, 2011, MOL ENDOCRINOL, V25, P1289, DOI 10.1210/me.2010-0442

Mizwicki MT, 2013, J ALZHEIMERS DIS, V34, P155, DOI 10.3233/JAD-121735

Mouchlis VD, 2018, J AM CHEM SOC, V140, P3285, DOI 10.1021/jacs.7b12045

Niranjan R, 2018, NEUROCHEM INT, V120, P13, DOI 10.1016/j.neuint.2018.07.003

Nordgren TM, 2019, NUTRIENTS, V11, DOI 10.3390/nu11010098

Oh Y, 2019, BMB REP, V52, P349, DOI 10.5483/BMBRep.2019.52.6.142

Orr SK, 2008, CURR OPIN INVEST DR, V9, P735

Orr SK, 2013, J NEUROCHEM, V127, P378, DOI 10.1111/jnc.12392

Papanikolaou Yanni, 2014, Nutr J, V13, P31, DOI 10.1186/1475-2891-13-31

Peng Y, 2021, FRONT PHARMACOL, V12, DOI 10.3389/fphar.2021.671734

Phani Sudarshan, 2012, Parkinsonism Relat Disord, V18 Suppl 1, pS207, DOI 10.1016/S1353-8020(11)70064-5

Plourde M, 2007, APPL PHYSIOL NUTR ME, V32, P619, DOI 10.1139/H07-034

Rai SN, 2017, FRONT AGING NEUROSCI, V9, DOI 10.3389/fnagi.2017.00421

Rai SN, 2019, NEUROTOX RES, V36, P452, DOI 10.1007/s12640-019-00038-6

Rai SN, 2017, J CHEM NEUROANAT, V85, P27, DOI 10.1016/j.jchemneu.2017.06.005

Rai SN, 2016, J CHEM NEUROANAT, V71, P41, DOI 10.1016/j.jchemneu.2015.12.002

Ryan AD, 2010, CURR OPIN PHARMACOL, V10, P166, DOI 10.1016/j.coph.2010.02.005

Sawada M, 2006, J NEURAL TRANSM-SUPP, P373

Serhan CN, 2015, SEMIN IMMUNOL, V27, P200, DOI 10.1016/j.smim.2015.03.004

Serhan CN, 2014, NATURE, V510, P92, DOI 10.1038/nature13479

Serhan CN, 2011, CURR TOP MED CHEM, V11, P629

Serhan CN, 2004, PROSTAG OTH LIPID M, V73, P155, DOI 10.1016/j.prostaglandins.2004.03.005

Serhan CN, 2000, J EXP MED, V192, P1197, DOI 10.1084/jem.192.8.1197

Simopoulos AP, 2002, BIOMED PHARMACOTHER, V56, P365, DOI 10.1016/S0753-3322(02)00253-6

Singh SS, 2018, FRONT PHARMACOL, V9, DOI 10.3389/fphar.2018.00757

Singh SS, 2020, OXID MED CELL LONGEV, V2020, DOI 10.1155/2020/6571484

Stoll G, 2000, J NEURAL TRANSM-SUPP, P81

Strike SC, 2016, J GERONTOL A-BIOL, V71, P236, DOI 10.1093/gerona/glv109

Su XM, 2008, NEUROBIOL AGING, V29, P1690, DOI 10.1016/j.neurobiolaging.2007.04.006

Tian YG, 2015, BIOCHEM BIOPH RES CO, V460, P799, DOI 10.1016/j.bbrc.2015.03.109

Tu DZ, 2019, J NEUROINFLAMM, V16, DOI 10.1186/s12974-019-1659-1

Tufekci KU, 2011, PARKINSONS DIS-US, V2011, DOI 10.4061/2011/487450

Virtanen JK, 2013, J AM HEART ASSOC, V2, DOI 10.1161/JAHA.113.000305

Wang QQ, 2015, TRANSL NEURODEGENER, V4, DOI 10.1186/s40035-015-0042-0

Wang XZ, 2015, J LIPID RES, V56, P674, DOI 10.1194/jlr.P055418

Wang XZ, 2015, ALZHEIMERS DEMENT, V11, P40, DOI 10.1016/j.jalz.2013.12.024

Wypijewska A, 2010, PARKINSONISM RELAT D, V16, P329, DOI 10.1016/j.parkreldis.2010.02.007

Xu JY, 2017, MED SCI MONITOR, V23, P2684, DOI 10.12659/MSM.901995

Xu MX, 2013, CNS NEUROSCI THER, V19, P235, DOI 10.1111/cns.12069

Yang B, 2019, FRONT NEUROL, V10, DOI 10.3389/fneur.2019.00642

Yin P, 2020, J ALZHEIMERS DIS, V73, P503, DOI 10.3233/JAD-190682

Yin P, 2019, FRONT CELL NEUROSCI, V13, DOI 10.3389/fncel.2019.00466

Zahra W, 2020, CNS NEUROL DISORD-DR, V19, P527, DOI 10.2174/1871527319666200812224457

Zhang W, 2005, FASEB J, V19, P533, DOI 10.1096/fj.04-2751com

Zhang Y, 2016, AM J CLIN NUTR, V103, P330, DOI 10.3945/ajcn.115.124081

Zhao W, 2012, PLOS ONE, V7, DOI 10.1371/journal.pone.0049191

Zhu MQ, 2016, MOL NEUROBIOL, V53, P2733, DOI 10.1007/s12035-015-9544-0

NR 91

TC 14

Z9 14

U1 1

U2 10

PU FRONTIERS MEDIA SA

PI LAUSANNE

PA AVENUE DU TRIBUNAL FEDERAL 34, LAUSANNE, CH-1015, SWITZERLAND

SN 1663-4365

J9 FRONT AGING NEUROSCI

JI Front. Aging Neurosci.

PD FEB 17

PY 2022

VL 14

AR 780811

DI 10.3389/fnagi.2022.780811

PG 11

WC Geriatrics & Gerontology; Neurosciences

WE Science Citation Index Expanded (SCI-EXPANDED)

SC Geriatrics & Gerontology; Neurosciences & Neurology

GA ZM7LK

UT WOS:000764533800001

PM 35250536

OA gold, Green Published

DA 2024-08-12

ER

PT J

AU Runtsch, MC

Ferrara, G

Angiari, S

AF Runtsch, Marah C.

Ferrara, Giovanni

Angiari, Stefano

TI Metabolic determinants of leukocyte pathogenicity in neurological

diseases

SO JOURNAL OF NEUROCHEMISTRY

LA English

DT Review

DE immune cells; immunometabolism; leukocytes; neurodegeneration;

neuroinflammation

ID BLOOD MONONUCLEAR-CELLS; NITRIC-OXIDE SYNTHASE; CENTRAL-NERVOUS-SYSTEM;

REMITTING MULTIPLE-SCLEROSIS; CHAIN ENZYME-ACTIVITIES;

GLUTAMATE-DEHYDROGENASE ACTIVITY; MITOCHONDRIAL COMPLEX-I;

alzheimer's disease; parkinson's disease; OXIDATIVE STRESS

AB Neuroinflammatory and neurodegenerative disease are characterized by the recruitment of circulating blood-borne innate and adaptive immune cells into the central nervous system (CNS). These leukocytes sustain the detrimental response in the CNS by releasing pro-inflammatory mediators that induce activation of local glial cells, blood-brain barrier (BBB) dysfunction, and neural cell death. However, infiltrating peripheral immune cells could also dampen CNS inflammation and support tissue repair. Recent advances in the field of immunometabolism demonstrate the importance of metabolic reprogramming for the activation and functionality of such innate and adaptive immune cell populations. In particular, an increasing body of evidence suggests that the activity of metabolites and metabolic enzymes could influence the pathogenic potential of immune cells during neuroinflammatory and neurodegenerative disorders. In this review, we discuss the role of intracellular metabolic cues in regulating leukocyte-mediated CNS damage in Alzheimer's and Parkinson's disease, multiple sclerosis and stroke, highlighting the therapeutic potential of drugs targeting metabolic pathways for the treatment of neurological diseases.

C1 [Runtsch, Marah C.; Angiari, Stefano] Trinity Coll Dublin, Sch Biochem & Immunol, 152-160 Pearse St, Dublin 2, Dublin, Ireland.

[Ferrara, Giovanni] IRCCS Osped Policlin San Martino, Genoa, Italy.

C3 Trinity College Dublin

RP Angiari, S (corresponding author), Trinity Coll Dublin, Sch Biochem & Immunol, 152-160 Pearse St, Dublin 2, Dublin, Ireland.

EM angiaris@tcd.ie

RI Ferrara, Giovanni/D-8550-2015; Runtsch, Marah C/JBS-3990-2023

OI Ferrara, Giovanni/0000-0002-4442-8864; Runtsch, Marah

C/0000-0002-9059-9269; Angiari, Stefano/0000-0003-4434-2224

CR Adiele RC, 2019, MITOCHONDRION, V44, P7, DOI 10.1016/j.mito.2017.12.005

Albanese F, 2019, FRONT NEUROSCI-SWITZ, V13, DOI 10.3389/fnins.2019.01352

Alexandrova ML, 2001, LUMINESCENCE, V16, P357

Amini P, 2018, NAT COMMUN, V9, DOI 10.1038/s41467-018-05387-y

Anandhan A, 2017, BRAIN RES BULL, V133, P12, DOI 10.1016/j.brainresbull.2017.03.009

Angiari S, 2020, CELL METAB, V31, P391, DOI 10.1016/j.cmet.2019.10.015

Angiari S, 2018, CELL RES, V28, P613, DOI 10.1038/s41422-018-0045-3

ANTUONO PG, 1995, DEMENTIA, V6, P200, DOI 10.1159/000106947

Arce-Varas N, 2017, CURR ALZHEIMER RES, V14, P112, DOI 10.2174/1567205013666161010125413

Arlehamn CSL, 2019, CURR OPIN IMMUNOL, V59, P115, DOI 10.1016/j.coi.2019.07.004

Armentero MT, 2011, NEUROBIOL AGING, V32, P2142, DOI 10.1016/j.neurobiolaging.2010.01.004

Armon-Omer A, 2020, MULT SCLER RELAT DIS, V41, DOI 10.1016/j.msard.2020.102025

Atashrazm F, 2019, MOVEMENT DISORD, V34, P406, DOI 10.1002/mds.27601

Atashrazm F, 2018, SCI REP-UK, V8, DOI 10.1038/s41598-018-33921-x

AUBBY D, 1988, J NEUROL NEUROSUR PS, V51, P893, DOI 10.1136/jnnp.51.7.893

Baird JK, 2019, PARKINSONISM RELAT D, V60, P25, DOI 10.1016/j.parkreldis.2018.10.029

Bakker NV, 2020, IMMUNOL REV, V295, P54, DOI 10.1111/imr.12848

Bantug GR, 2018, NAT REV IMMUNOL, V18, P19, DOI 10.1038/nri.2017.99

Barnas JL, 2019, CURR OPIN IMMUNOL, V61, P92, DOI 10.1016/j.coi.2019.09.004

BARROSO N, 1993, CLIN CHEM, V39, P667

Barthwal MK, 1999, ACTA NEUROL SCAND, V100, P300

Baufeld C, 2018, J NEURAL TRANSM, V125, P809, DOI 10.1007/s00702-017-1795-7

Bellomo G, 2020, MOVEMENT DISORD, V35, P34, DOI 10.1002/mds.27895

Berod L, 2014, NAT MED, V20, P1327, DOI 10.1038/nm.3704

Biragyn A, 2017, SEMIN IMMUNOPATHOL, V39, P283, DOI 10.1007/s00281-016-0615-8

Bjelobaba I, 2018, J NEUROSCI RES, V96, P1021, DOI 10.1002/jnr.24224

Bogie JFJ, 2020, ADV DRUG DELIVER REV, V159, P198, DOI 10.1016/j.addr.2020.01.004

Bomprezzi R, 2015, THER ADV NEUROL DISO, V8, P20, DOI 10.1177/1756285614564152

Bonaventura A, 2019, CARDIOVASC RES, V115, P1266, DOI 10.1093/cvr/cvz084

Bossù P, 2015, FRONT IMMUNOL, V6, DOI 10.3389/fimmu.2015.00632

Boukouris AE, 2016, TRENDS BIOCHEM SCI, V41, P712, DOI 10.1016/j.tibs.2016.05.013

Busse M, 2018, EUR ARCH PSY CLIN N, V268, P493, DOI 10.1007/s00406-017-0785-y

Butterfield DA, 2019, NAT REV NEUROSCI, V20, P148, DOI 10.1038/s41583-019-0132-6

Calabrese V, 2006, ANTIOXID REDOX SIGN, V8, P1975, DOI 10.1089/ars.2006.8.1975

Camandola S, 2017, EMBO J, V36, P1474, DOI 10.15252/embj.201695810

Caorsi R, 2017, ANN RHEUM DIS, V76, P1648, DOI 10.1136/annrheumdis-2016-210802

Caronti B, 1999, NEUROREPORT, V10, P2907, DOI 10.1097/00001756-199909290-00006

Cataldo AM, 2000, AM J PATHOL, V157, P277, DOI 10.1016/S0002-9440(10)64538-5

Cervenka I, 2017, SCIENCE, V357, DOI 10.1126/science.aaf9794

Chen C, 2019, BIOMED PHARMACOTHER, V109, P547, DOI 10.1016/j.biopha.2018.10.103

Chen CM, 2009, NEUROBIOL DIS, V33, P429, DOI 10.1016/j.nbd.2008.11.011

Chen KD, 2011, NEUROBIOL DIS, V43, P698, DOI 10.1016/j.nbd.2011.05.023

Cheng CI, 2014, BIOMED RES INT, V2014, DOI 10.1155/2014/214587

Chiang PL, 2018, J NEUROINFLAMM, V15, DOI 10.1186/s12974-018-1317-z

Ciccocioppo F, 2008, INT J IMMUNOPATH PH, V21, P23, DOI 10.1177/039463200802100104

Clarke AJ, 2019, NAT REV IMMUNOL, V19, P170, DOI 10.1038/s41577-018-0095-2

Codoni V, 2016, G3-GENES GENOM GENET, V6, P3361, DOI 10.1534/g3.116.033894

COLLINS JM, 1991, MECH AGEING DEV, V59, P153, DOI 10.1016/0047-6374(91)90081-A

Colonna M, 2017, ANNU REV IMMUNOL, V35, P441, DOI 10.1146/annurev-immunol-051116-052358

Colpo GD, 2019, FRONT NEUROL, V10, DOI 10.3389/fneur.2019.00778

Cook DA, 2017, NPJ PARKINSONS DIS, V3, DOI 10.1038/s41531-017-0010-8

Corlier F, 2015, TRANSL PSYCHIAT, V5, DOI 10.1038/tp.2015.87

Cragnolini AB, 2020, NEURAL REGEN RES, V15, P838, DOI 10.4103/1673-5374.268897

Cramer JV, 2019, J NEUROIMMUNOL, V326, P33, DOI 10.1016/j.jneuroim.2018.11.006

Cryan JF, 2019, PHYSIOL REV, V99, P1877, DOI 10.1152/physrev.00018.2018

Daniels BP, 2019, IMMUNITY, V50, P64, DOI 10.1016/j.immuni.2018.11.017

De Biasi S, 2019, EUR J IMMUNOL, V49, P2204, DOI 10.1002/eji.201948223

De Leo ME, 1998, NEUROSCI LETT, V250, P173, DOI 10.1016/S0304-3940(98)00469-8

De Rasmo D, 2020, BIOMEDICINES, V8, DOI 10.3390/biomedicines8040085

De Riccardis L, 2016, BBA CLIN, V6, P131, DOI 10.1016/j.bbacli.2016.10.004

De Riccardis L, 2015, J BIOTECHNOL, V202, P31, DOI 10.1016/j.jbiotec.2015.02.015

De Rosa V, 2015, NAT IMMUNOL, V16, P1174, DOI 10.1038/ni.3269

De Servi B, 2002, EXP GERONTOL, V37, P813, DOI 10.1016/S0531-5565(02)00018-9

DeBerardinis RJ, 2020, NAT METAB, V2, P127, DOI 10.1038/s42255-020-0172-2

Dendrou CA, 2015, NAT REV IMMUNOL, V15, P545, DOI 10.1038/nri3871

Devanney NA, 2020, EXP NEUROL, V329, DOI 10.1016/j.expneurol.2020.113310

Di WC, 2018, CURR ISSUES MOL BIOL, V28, P29, DOI 10.21775/cimb.028.029

Dionisio-Santos DA, 2019, J NEUROINFLAMM, V16, DOI 10.1186/s12974-019-1453-0

Domingues C, 2017, CURR ALZHEIMER RES, V14, P870, DOI 10.2174/1567205014666170317113606

Eckert A, 2001, J NEUROSCI RES, V64, P183, DOI 10.1002/jnr.1064

El Haddad S, 2020, GENE, V738, DOI 10.1016/j.gene.2020.144454

Emelyanov A, 2018, J NEUROL SCI, V395, P135, DOI 10.1016/j.jns.2018.10.002

Evans FL, 2019, FRONT IMMUNOL, V10, DOI 10.3389/fimmu.2019.02171

Fan LY, 2020, FRONT NEUROL, V10, DOI 10.3389/fneur.2019.01312

Fehlbaum-Beurdeley P, 2010, ALZHEIMERS DEMENT, V6, P25, DOI 10.1016/j.jalz.2009.07.001

Feigin VL, 2017, CIRC RES, V120, P439, DOI 10.1161/CIRCRESAHA.116.308413

Feldhaus P, 2011, METAB BRAIN DIS, V26, P229, DOI 10.1007/s11011-011-9253-y

Felk S, 2010, J NEUROL SCI, V295, P46, DOI 10.1016/j.jns.2010.05.013

Fertan E, 2019, FRONT PHARMACOL, V10, DOI 10.3389/fphar.2019.01044

Feske SK, 2009, BRAIN RES, V1257, P89, DOI 10.1016/j.brainres.2008.12.045

Forster C, 1999, ACTA NEUROPATHOL, V97, P215, DOI 10.1007/s004010050977

Fujii T, 2017, J PHARMACOL SCI, V134, P1, DOI 10.1016/j.jphs.2017.05.002

Garcia-Bonilla L, 2014, J IMMUNOL, V193, P2531, DOI 10.4049/jimmunol.1400918

Gate D, 2020, NATURE, V577, P399, DOI 10.1038/s41586-019-1895-7

Gatta L, 2009, NEUROSCI LETT, V453, P36, DOI 10.1016/j.neulet.2009.02.003

Gatto EM, 1996, MOVEMENT DISORD, V11, P261, DOI 10.1002/mds.870110308

Gatto EM, 1999, J NEUROL SCI, V165, P66, DOI 10.1016/S0022-510X(99)00079-9

Gatto EM, 2000, NITRIC OXIDE-BIOL CH, V4, P534, DOI 10.1006/niox.2000.0288

Gelders G, 2018, J IMMUNOL RES, V2018, DOI 10.1155/2018/4784268

Geltink RIK, 2018, ANNU REV IMMUNOL, V36, P461, DOI 10.1146/annurev-immunol-042617-053019

Gerriets VA, 2015, J CLIN INVEST, V125, P194, DOI 10.1172/JCI76012

Giubilei F, 2004, DEMENT GERIATR COGN, V18, P338, DOI 10.1159/000080128

Gold R, 2006, BRAIN, V129, P1953, DOI 10.1093/brain/awl075

González-Mundo I, 2020, EXP GERONTOL, V136, DOI 10.1016/j.exger.2020.110951

Gonzalo H, 2019, FRONT NEUROSCI-SWITZ, V13, DOI 10.3389/fnins.2019.00938

Grau AJ, 2001, J NEUROL SCI, V192, P41, DOI 10.1016/S0022-510X(01)00590-1

Greenhalgh AD, 2020, NAT REV NEUROSCI, V21, P139, DOI 10.1038/s41583-020-0263-9

Griffin JWD, 2017, OXID MED CELL LONGEV, V2017, DOI 10.1155/2017/5472792

Haghikia A, 2015, MULT SCLER J, V21, P1262, DOI 10.1177/1352458514561909

Helgadottir A, 2004, NAT GENET, V36, P233, DOI 10.1038/ng1311

Hewett JA, 2009, J NEUROCHEM, V110, P1717, DOI 10.1111/j.1471-4159.2009.06288.x

Hoepken HH, 2007, NEUROBIOL DIS, V25, P401, DOI 10.1016/j.nbd.2006.10.007

Hong S, 2016, CURR OPIN NEUROBIOL, V36, P128, DOI 10.1016/j.conb.2015.12.004

Hooftman A, 2019, TRENDS IMMUNOL, V40, P687, DOI 10.1016/j.it.2019.05.007

Huang SF, 2020, SCI REP-UK, V10, DOI 10.1038/s41598-020-64722-w

Huang YH, 2018, PLOS ONE, V13, DOI 10.1371/journal.pone.0193175

Hye A, 2005, NEUROSCI LETT, V373, P1, DOI 10.1016/j.neulet.2004.10.031

Iadecola C, 1996, STROKE, V27, P1373, DOI 10.1161/01.STR.27.8.1373

Iadecolar C, 2020, J CLIN INVEST, V130, P2777, DOI 10.1172/JCI135530

Ide K, 2015, NUTRITION, V31, P406, DOI 10.1016/j.nut.2014.08.001

Ik M., 2017, BIOMED PHARMACOTHER, V87, P561

Ik M., 2019, ARCH PHYSIOL BIOCHEM, P1

Injarabian L, 2020, INT J MOL SCI, V21, DOI 10.3390/ijms21010287

Ishizuka K, 2002, ANN NY ACAD SCI, V977, P173, DOI 10.1111/j.1749-6632.2002.tb04814.x

IWATSUJI K, 1989, GERONTOLOGY, V35, P218, DOI 10.1159/000213026

Jellusova J, 2020, IMMUNOL REV, V295, P39, DOI 10.1111/imr.12855

Jellusova J, 2017, NAT IMMUNOL, V18, P303, DOI 10.1038/ni.3664

Jickling GC, 2015, J CEREBR BLOOD F MET, V35, P888, DOI 10.1038/jcbfm.2015.45

Jickling GC, 2013, ANN NEUROL, V74, P232, DOI 10.1002/ana.23883

Jung J, 2019, NAT CELL BIOL, V21, P85, DOI 10.1038/s41556-018-0217-x

Kajikawa M, 2015, HYPERTENSION, V66, P892, DOI 10.1161/HYPERTENSIONAHA.115.05587

Kajikawa M, 2014, HYPERTENSION, V63, P856, DOI 10.1161/HYPERTENSIONAHA.113.02296

Kalanj-Bognar S, 2002, J GERONTOL A-BIOL, V57, pB16, DOI 10.1093/gerona/57.1.B16

Kalia LV, 2015, LANCET, V386, P896, DOI 10.1016/S0140-6736(14)61393-3

KALRA J, 1992, MOL CELL BIOCHEM, V112, P181

Kang JH, 2012, ENDOCR REV, V33, P715, DOI 10.1210/er.2011-1049

KARLSSON JO, 1995, NEUROBIOL AGING, V16, P901, DOI 10.1016/0197-4580(95)02004-7

Kaushik DK, 2019, J CLIN INVEST, V129, P3277, DOI 10.1172/JCI124012

Keppel MP, 2015, J IMMUNOL, V194, P1954, DOI 10.4049/jimmunol.1402099

Khakh BS, 2019, ANNU REV NEUROSCI, V42, P187, DOI 10.1146/annurev-neuro-070918-050443

Kierdorf K, 2019, NAT REV NEUROSCI, V20, P547, DOI 10.1038/s41583-019-0201-x

Kim HJ, 2016, PARKINSONISM RELAT D, V23, P99, DOI 10.1016/j.parkreldis.2015.12.002

Kim S, 2004, FASEB J, V18, P1615, DOI 10.1096/fj.04-1917fje

Kinney Jefferson W, 2018, Alzheimers Dement (N Y), V4, P575, DOI 10.1016/j.trci.2018.06.014

Kishore M, 2018, IMMUNITY, V48, P831, DOI 10.1016/j.immuni.2018.03.034

Klotz L, 2019, SCI TRANSL MED, V11, DOI 10.1126/scitranslmed.aao5563

Kluss JH, 2019, BIOCHEM SOC T, V47, P651, DOI 10.1042/BST20180462

Kornberg MD, 2020, WIRES SYST BIOL MED, V12, DOI 10.1002/wsbm.1486

Kornberg MD, 2018, SCIENCE, V360, P449, DOI 10.1126/science.aan4665

Kunkl M, 2019, CELLS-BASEL, V8, DOI 10.3390/cells8060575

Kuo PC, 2020, J NEUROINFLAMM, V17, DOI 10.1186/s12974-020-01768-7

Kuwahara T, 2020, FRONT NEUROSCI-SWITZ, V14, DOI 10.3389/fnins.2020.00227

La Rocca C, 2017, METABOLISM, V77, P39, DOI 10.1016/j.metabol.2017.08.011

Lafay-Chebassier C, 2005, J NEUROCHEM, V94, P215, DOI 10.1111/j.1471-4159.2005.03187.x

Lassmann H, 2019, CURR OPIN NEUROL, V32, P313, DOI 10.1097/WCO.0000000000000685

Lassmann H, 2018, CSH PERSPECT MED, V8, DOI 10.1101/cshperspect.a028936

Lazdon E, 2020, J NEURAL TRANSM, V127, P149, DOI 10.1007/s00702-020-02154-6

Le Page A, 2018, EXP GERONTOL, V107, P59, DOI 10.1016/j.exger.2017.12.019

Leuner K, 2012, MOL NEUROBIOL, V46, P194, DOI 10.1007/s12035-012-8300-y

Leutner S, 2005, PHARMACOPSYCHIATRY, V38, P312, DOI 10.1055/s-2005-916186

Levecque C, 2003, HUM MOL GENET, V12, P79, DOI 10.1093/hmg/ddg009

Levite M, 2016, ACTA PHYSIOL, V216, P42, DOI 10.1111/apha.12476

Li HD, 2017, SCI REP-UK, V7, DOI 10.1038/s41598-017-13700-w

Liao ST, 2019, NAT COMMUN, V10, DOI 10.1038/s41467-019-13078-5

Lien LM, 2017, J AM HEART ASSOC, V6, DOI 10.1161/JAHA.117.006157

Liu DD, 2018, NEUROCHEM INT, V114, P42, DOI 10.1016/j.neuint.2018.01.002

Lu ZM, 2018, TRENDS BIOCHEM SCI, V43, P301, DOI 10.1016/j.tibs.2018.01.006

Lynch MA, 2020, PROG NEUROBIOL, V184, DOI 10.1016/j.pneurobio.2019.101719

Maeda K, 2005, HYPERTENS RES, V28, P755, DOI 10.1291/hypres.28.755

Magistretti PJ, 2018, NAT REV NEUROSCI, V19, P235, DOI 10.1038/nrn.2018.19

Manda-Handzlik A, 2019, CELLS-BASEL, V8, DOI 10.3390/cells8121477

Mandas A, 2012, LIPIDS HEALTH DIS, V11, DOI 10.1186/1476-511X-11-39

Mangialasche F, 2015, J ALZHEIMERS DIS, V44, P649, DOI 10.3233/JAD-142052

Marksteiner J, 2009, EXP GERONTOL, V44, P370, DOI 10.1016/j.exger.2009.02.007

Martin MA, 1996, NEUROLOGY, V46, P1343, DOI 10.1212/WNL.46.5.1343

Maté I, 2015, J ALZHEIMERS DIS, V43, P213, DOI 10.3233/JAD-140861

Mathur D, 2014, FRONT NEUROL, V5, DOI 10.3389/fneur.2014.00250

Matias I, 2019, FRONT AGING NEUROSCI, V11, DOI 10.3389/fnagi.2019.00059

Maynard S, 2015, AGING-US, V7, P793, DOI 10.18632/aging.100810

Migliore L, 2002, NEUROLOGY, V58, P1809, DOI 10.1212/WNL.58.12.1809

Migliore L, 2001, INT J HYG ENVIR HEAL, V204, P61, DOI 10.1078/1438-4639-00074

Miki Y, 2018, NEUROBIOL AGING, V63, P33, DOI 10.1016/j.neurobiolaging.2017.11.006

Mimpen M, 2020, IMMUNOL LETT, V222, P1, DOI 10.1016/j.imlet.2020.02.012

Morel M, 2009, J CELL MOL MED, V13, P1476, DOI 10.1111/j.1582-4934.2009.00849.x

Moskowitz MA, 2010, NEURON, V67, P181, DOI 10.1016/j.neuron.2010.07.002

Müftüoglu M, 2004, MOVEMENT DISORD, V19, P544, DOI 10.1002/mds.10695

Mutez E, 2011, NEUROBIOL AGING, V32, P1839, DOI 10.1016/j.neurobiolaging.2009.10.016

Nakagawa Y, 2015, PHARMACOL THERAPEUT, V154, P21, DOI 10.1016/j.pharmthera.2015.06.010

Newcombe EA, 2018, J NEUROINFLAMM, V15, DOI 10.1186/s12974-018-1313-3

Ng XL, 2019, INT J MOL SCI, V20, DOI 10.3390/ijms20204975

Nguyen M, 2019, TRENDS NEUROSCI, V42, P140, DOI 10.1016/j.tins.2018.11.001

Ni Chasaide Caitlin, 2020, Brain Neurosci Adv, V4, p2398212819901082, DOI 10.1177/2398212819901082

O'Brien KL, 2019, NAT REV IMMUNOL, V19, P282, DOI 10.1038/s41577-019-0139-2

O'Neill LAJ, 2016, NAT REV IMMUNOL, V16, P553, DOI 10.1038/nri.2016.70

O'Neill LAJ, 2013, NATURE, V493, P346, DOI 10.1038/nature11862

Orihuela R, 2016, BRIT J PHARMACOL, V173, P649, DOI 10.1111/bph.13139

Ortega RA, 2016, J CLIN NEUROSCI, V28, P185, DOI 10.1016/j.jocn.2015.12.004

Paccalin M, 2005, ARCH INTERN MED, V165, P2428, DOI 10.1001/archinte.165.20.2428

Paccalin M, 2006, DEMENT GERIATR COGN, V22, P320, DOI 10.1159/000095562

Pålsson-McDermott EM, 2020, CELL RES, V30, P300, DOI 10.1038/s41422-020-0291-z

Pani A, 2009, BMC MED, V7, DOI 10.1186/1741-7015-7-66

Paolicelli RC, 2019, SEMIN CELL DEV BIOL, V94, P129, DOI 10.1016/j.semcdb.2019.03.012

Papagiannakis N, 2019, NEUROSCI LETT, V704, P112, DOI 10.1016/j.neulet.2019.04.003

Papagiannakis N, 2015, MOVEMENT DISORD, V30, P1830, DOI 10.1002/mds.26433

Patel CH, 2019, NAT REV DRUG DISCOV, V18, P669, DOI 10.1038/s41573-019-0032-5

Pearce EJ, 2018, NAT REV IMMUNOL, V18, P81, DOI 10.1038/nri.2017.139

Pearce EL, 2013, IMMUNITY, V38, P633, DOI 10.1016/j.immuni.2013.04.005

Pekny M, 2014, PHYSIOL REV, V94, P1077, DOI 10.1152/physrev.00041.2013

Peruzzotti-Jametti L, 2018, TRENDS MOL MED, V24, P838, DOI 10.1016/j.molmed.2018.07.007

Petrillo S, 2020, MOVEMENT DISORD, V35, P180, DOI 10.1002/mds.27878

Petrone AB, 2016, TRANSL STROKE RES, V7, P103, DOI 10.1007/s12975-015-0431-9

Petrozzi L, 2001, NEUROL SCI, V22, P83, DOI 10.1007/s100720170058

Pickrell AM, 2015, NEURON, V85, P257, DOI 10.1016/j.neuron.2014.12.007

Prigione A, 2006, NEUROBIOL DIS, V23, P36, DOI 10.1016/j.nbd.2006.01.013

Prigione A, 2010, NEUROSCI LETT, V477, P6, DOI 10.1016/j.neulet.2010.04.022

Prigione A, 2009, PARKINSONISM RELAT D, V15, P327, DOI 10.1016/j.parkreldis.2008.05.013

Prinz M, 2017, NAT NEUROSCI, V20, P136, DOI 10.1038/nn.4475

Puleston DJ, 2017, CELL METAB, V26, P131, DOI 10.1016/j.cmet.2017.06.019

Qadri R, 2018, RESTOR NEUROL NEUROS, V36, P719, DOI 10.3233/RNN-180852

Qin W, 2019, NAT CHEM BIOL, V15, P983, DOI 10.1038/s41589-019-0323-5

Rangachari M, 2013, J AUTOIMMUN, V45, P31, DOI 10.1016/j.jaut.2013.06.008

Ricker Edd, 2016, F1000Res, V5

Robb JL, 2020, NEUROSCIENCE, V447, P167, DOI 10.1016/j.neuroscience.2019.10.021

Robinson RAS, 2013, J ALZHEIMERS DIS, V37, P661, DOI 10.3233/JAD-130665

Rodríguez-Espinosa O, 2015, IMMUNOLOGY, V145, P213, DOI 10.1111/imm.12437

Rossi B, 2020, IMMUNOBIOLOGY, V225, DOI 10.1016/j.imbio.2019.10.014

Rossi B, 2011, J LEUKOCYTE BIOL, V89, P539, DOI 10.1189/jlb.0710432

Rothhammer V, 2015, SEMIN IMMUNOPATHOL, V37, P625, DOI 10.1007/s00281-015-0515-3

Ruhnau J, 2014, STROKE, V45, P794, DOI 10.1161/STROKEAHA.113.003342

Russell DG, 2019, NAT REV IMMUNOL, V19, P291, DOI 10.1038/s41577-019-0124-9

Ryan DG, 2020, ANNU REV IMMUNOL, V38, P289, DOI 10.1146/annurev-immunol-081619-104850

Ryan DG, 2019, NAT METAB, V1, P16, DOI 10.1038/s42255-018-0014-7

Sabatino JJ, 2019, NAT REV NEUROSCI, V20, P728, DOI 10.1038/s41583-019-0233-2

Sala G, 2014, BRAIN RES, V1546, P46, DOI 10.1016/j.brainres.2013.12.017

Santorelli FM, 1997, BIOCHEM BIOPH RES CO, V238, P326, DOI 10.1006/bbrc.1997.7167

Sapieha P, 2008, NAT MED, V14, P1067, DOI 10.1038/nm.1873

Saravia J, 2020, CELL RES, V30, P328, DOI 10.1038/s41422-020-0301-1

Sato Y, 2011, CLIN EXP HYPERTENS, V33, P397, DOI 10.3109/10641963.2010.549268

Sävman K, 2013, TRANSL STROKE RES, V4, P228, DOI 10.1007/s12975-012-0216-3

Saz-Leal P, 2018, CELL REP, V25, P1118, DOI 10.1016/j.celrep.2018.09.092

Schuessel K, 2006, FREE RADICAL BIO MED, V40, P850, DOI 10.1016/j.freeradbiomed.2005.10.041

Schwartz M, 2019, DIALOGUES CLIN NEURO, V21, P21

Seifert HA, 2018, IMMUNOLOGY, V154, P169, DOI 10.1111/imm.12887

Seki SM, 2017, FRONT IMMUNOL, V8, DOI 10.3389/fimmu.2017.01549

Seki SM, 2017, J IMMUNOL, V198, P4607, DOI 10.4049/jimmunol.1600825

Seki Y, 2010, J CLIN NEUROSCI, V17, P187, DOI 10.1016/j.jocn.2009.04.020

Shi LZ, 2011, J EXP MED, V208, P1367, DOI 10.1084/jem.20110278

Shim R, 2016, INT J MOL SCI, V17, DOI 10.3390/ijms17010064

Shinde S, 2006, NEUROL INDIA, V54, P390, DOI 10.4103/0028-3886.28112

Sie C, 2017, SEMIN IMMUNOPATHOL, V39, P99, DOI 10.1007/s00281-016-0608-7

Simons M, 2016, CSH PERSPECT BIOL, V8, DOI 10.1101/cshperspect.a020479

Sims NR, 2010, BBA-MOL BASIS DIS, V1802, P80, DOI 10.1016/j.bbadis.2009.09.003

Sippel TR, 2015, J CEREBR BLOOD F MET, V35, P1657, DOI 10.1038/jcbfm.2015.103

Smith AM, 2018, MOVEMENT DISORD, V33, P1580, DOI 10.1002/mds.104

Solana Corona, 2018, Int J Alzheimers Dis, V2018, P3128758, DOI 10.1155/2018/3128758

SORBI S, 1990, NEUROSCI LETT, V117, P165, DOI 10.1016/0304-3940(90)90138-Y

Sorgdrager FJH, 2019, FRONT IMMUNOL, V10, DOI 10.3389/fimmu.2019.02565

Srivastava A, 2019, NEUROCHEM INT, V129, DOI 10.1016/j.neuint.2019.104480

Straface E, 2005, FEBS LETT, V579, P2759, DOI 10.1016/j.febslet.2005.03.094

Sultana R, 2013, FREE RADICAL BIO MED, V65, P595, DOI 10.1016/j.freeradbiomed.2013.08.001

Sulzer D, 2017, NATURE, V546, P656, DOI 10.1038/nature22815

Sweeney MD, 2018, NAT REV NEUROL, V14, P133, DOI 10.1038/nrneurol.2017.188

Takemori K, 2011, BRAIN RES, V1417, P137, DOI 10.1016/j.brainres.2011.08.042

Tang B. L., 2020, J CELLULAR PHYSL, V2020

Tang Y, 2016, MOL NEUROBIOL, V53, P1181, DOI 10.1007/s12035-014-9070-5

Terzioglu G, 2017, MECH AGEING DEV, V167, P24, DOI 10.1016/j.mad.2017.09.003

Thériault P, 2015, ALZHEIMERS RES THER, V7, DOI 10.1186/s13195-015-0125-2

Thyagarajan D, 2000, ANN NEUROL, V48, P730, DOI 10.1002/1531-8249(200011)48:5<730::AID-ANA6>3.3.CO;2-S

Tian L, 2014, J ALZHEIMERS DIS, V42, P511, DOI 10.3233/JAD-132192

Tiribuzi R, 2014, NEUROBIOL AGING, V35, P345, DOI 10.1016/j.neurobiolaging.2013.08.003

Toffoli M, 2020, NEUROPHARMACOLOGY, V170, DOI 10.1016/j.neuropharm.2020.108022

Tramutola A, 2018, FREE RADICAL BIO MED, V129, P430, DOI 10.1016/j.freeradbiomed.2018.10.414

Tranah GJ, 2018, SCI REP-UK, V8, DOI 10.1038/s41598-018-30255-6

Troncoso-Escudero P, 2018, FRONT NEUROL, V9, DOI 10.3389/fneur.2018.00860

Vaibhav K, 2018, J EXP MED, V215, P2636, DOI 10.1084/jem.20171905

Valla J, 2006, MITOCHONDRION, V6, P323, DOI 10.1016/j.mito.2006.10.004

van Raam BJ, 2006, INT J HEMATOL, V84, P199, DOI 10.1532/IJH97.06131

Vergara D, 2015, BMC NEUROSCI, V16, DOI 10.1186/s12868-015-0183-1

Verkhratsky A, 2019, ADV EXP MED BIOL, V1175, P1, DOI 10.1007/978-981-13-9913-8_1

Vida C, 2019, INT J MOL SCI, V20, DOI 10.3390/ijms20030771

Viola A, 2019, FRONT IMMUNOL, V10, DOI 10.3389/fimmu.2019.01462

Vitte J, 2004, J CLIN IMMUNOL, V24, P683, DOI 10.1007/s10875-004-6243-4

Voet S, 2019, TRENDS MOL MED, V25, P112, DOI 10.1016/j.molmed.2018.11.005

Vogler S, 2005, J MOL MED, V83, P806, DOI 10.1007/s00109-005-0661-5

von Bernhardi R, 2005, DEMENT GERIATR COGN, V19, P204, DOI 10.1159/000083500

Walder CE, 1997, STROKE, V28, P2252, DOI 10.1161/01.STR.28.11.2252

Wang JY, 2019, FRONT PHARMACOL, V10, DOI 10.3389/fphar.2019.00286

Wang S, 2012, EUR J NEUROL, V19, P1015, DOI 10.1111/j.1468-1331.2012.03670.x

Wang S, 2014, J CLIN NEUROSCI, V21, P810, DOI 10.1016/j.jocn.2013.08.017

Wang X, 2019, STROKE, V50, P1869, DOI 10.1161/STROKEAHA.119.024564

Wang YJ, 2019, FRONT IMMUNOL, V10, DOI 10.3389/fimmu.2019.02781

Waters LR, 2018, ISCIENCE, V5, P99, DOI 10.1016/j.isci.2018.07.005

Wojsiat J, 2015, J ALZHEIMERS DIS, V46, P329, DOI 10.3233/JAD-141977

Wolfe H, 2019, NEUROSCI RES, V148, P54, DOI 10.1016/j.neures.2019.01.001

Wu GH, 2008, J NEUROL SCI, V271, P168, DOI 10.1016/j.jns.2008.04.011

Wu GH, 2011, BRAIN RES, V1394, P105, DOI 10.1016/j.brainres.2011.04.013

Xiong XG, 2009, BIOSCI BIOTECH BIOCH, V73, P657, DOI 10.1271/bbb.80717

Yang XD, 2018, PARKINSONISM RELAT D, V51, P30, DOI 10.1016/j.parkreldis.2018.02.037

Yates Sharon C, 2013, Acta Neuropathol Commun, V1, P3, DOI 10.1186/2051-5960-1-3

Yoo H, 2019, MOL NEUROBIOL, V56, P3211, DOI 10.1007/s12035-018-1295-2

YOSHINO H, 1992, J NEURAL TRANSM-PARK, V4, P27, DOI 10.1007/BF02257619

Yoshino Y, 2017, J ALZHEIMERS DIS, V58, P687, DOI 10.3233/JAD-161211

Youssef S, 2002, NATURE, V420, P78, DOI 10.1038/nature01158

Yu D, 2015, J ALZHEIMERS DIS, V43, P291, DOI 10.3233/JAD-140414

Yuan M, 2019, BMC NEUROL, V19, DOI 10.1186/s12883-019-1481-5

Zádori D, 2018, J ALZHEIMERS DIS, V62, P523, DOI 10.3233/JAD-170929

Zaslona Z, 2020, MOL CELL, V78, P814, DOI 10.1016/j.molcel.2020.04.002

Zhou Q, 2014, NEW ENGL J MED, V370, P911, DOI 10.1056/NEJMoa1307361

NR 298

TC 12

Z9 12

U1 2

U2 10

PU WILEY

PI HOBOKEN

PA 111 RIVER ST, HOBOKEN 07030-5774, NJ USA

SN 0022-3042

EI 1471-4159

J9 J NEUROCHEM

JI J. Neurochem.

PD JUL

PY 2021

VL 158

IS 1

SI SI

BP 36

EP 58

DI 10.1111/jnc.15169

EA SEP 2020

PG 23

WC Biochemistry & Molecular Biology; Neurosciences

WE Science Citation Index Expanded (SCI-EXPANDED)

SC Biochemistry & Molecular Biology; Neurosciences & Neurology

GA TJ1WY

UT WOS:000571587200001

PM 32880969

OA Bronze

DA 2024-08-12

ER

PT J

AU Paudel, YN

Angelopoulou, E

Semple, B

Piperi, C

Othman, I

Shaikh, MF

AF Paudel, Yam Nath

Angelopoulou, Efthalia

Semple, Bridgette

Piperi, Christina

Othman, Iekhsan

Shaikh, Mohd Farooq

TI Potential Neuroprotective Effect of the HMGB1 Inhibitor Glycyrrhizin in

Neurological Disorders

SO ACS CHEMICAL NEUROSCIENCE

LA English

DT Review

DE Glycyrrhizin; HMGB1; pharmacological inhibitor; neuroprotection;

neurological disorders; inflammation

ID GROUP BOX 1; MOBILITY GROUP BOX-1; TRAUMATIC BRAIN-INJURY; COGNITIVE

IMPAIRMENT; parkinson's disease; THERAPEUTIC TARGET; alzheimer's disease;

CYTOKINE ACTIVITY; RECEPTOR 4; RAT MODEL

AB Glycyrrhizin (glycyrrhizic acid), a bioactive triterpenoid saponin constituent of Glycyrrhiza glabra, is a traditional medicine possessing a plethora of pharmacological anti-inflammatory, antioxidant, antimicrobial, and antiaging properties. It is a known pharmacological inhibitor of high mobility group box 1 (HMGB1), a ubiquitous protein with proinflammatory cytokinelike activity. HMGB1 has been implicated in an array of inflammatory diseases when released extracellularly, mainly by activating intracellular signaling upon binding to the receptor for advanced glycation end products (RAGE) and toll-like receptor 4 (TLR4). HMGB1 neutralization strategies have demonstrated disease-modifying outcomes in several preclinical models of neurological disorders. Herein, we reveal the potential neuroprotective effects of glycyrrhizin against several neurological disorders. Emerging findings demonstrate the therapeutic potential of glycyrrhizin against several HMGB1-mediated pathological conditions including traumatic brain injury, neuroinflammation and associated conditions, epileptic seizures, Alzheimer's disease, Parkinson's disease, and multiple sclerosis. Glycyrrhizin's effects in neurological disorders are mainly attributed to the attenuation of neuronal damage by inhibiting HMGB1 expression and translocation as well as by downregulating the expression of inflammatory cytokines. A large number of preclinical findings supports the notion that glycyrrhizin might be a promising therapeutic alternative to overcome the shortcomings of the mainstream therapeutic strategies against neurological disorders, mainly by halting disease progression. However, future research is warranted for a deeper exploration of the precise underlying molecular mechanism as well as for clinical translation.

C1 [Paudel, Yam Nath; Othman, Iekhsan; Shaikh, Mohd Farooq] Monash Univ Malaysia, Jeffrey Cheah Sch Med & Hlth Sci, Neuropharmacol Res Lab, Bandar Sunway 46150, Selangor, Malaysia.

[Angelopoulou, Efthalia; Piperi, Christina] Natl & Kapodistrian Univ Athens, Med Sch, Dept Biol Chem, Athens 11527, Greece.

[Semple, Bridgette] Monash Univ, Alfred Hosp, Cent Clin Sch, Dept Neurosci, Melbourne, Vic 3800, Australia.

[Semple, Bridgette] Univ Melbourne, Dept Med, Royal Melbourne Hosp, Parkville, Vic 3010, Australia.

C3 Monash University; Monash University Malaysia; National & Kapodistrian

University of Athens; Florey Institute of Neuroscience & Mental Health;

Monash University; Melbourne Health; Royal Melbourne Hospital;

University of Melbourne

RP Paudel, YN; Shaikh, MF (corresponding author), Monash Univ Malaysia, Jeffrey Cheah Sch Med & Hlth Sci, Neuropharmacol Res Lab, Bandar Sunway 46150, Selangor, Malaysia.; Piperi, C (corresponding author), Natl & Kapodistrian Univ Athens, Med Sch, Dept Biol Chem, Athens 11527, Greece.

EM yam.paudel@monash.edu; cpiperi@med.uoa.gr; farooq.shaikh@monash.edu

RI Shaikh, Mohd. Farooq/H-6029-2019; Angelopoulou, Efthalia/ABB-8167-2020;

Piperi, Christina/AAF-2009-2020

OI Shaikh, Mohd. Farooq/0000-0001-9865-6224; Angelopoulou,

Efthalia/0000-0002-1426-3384; Piperi, Christina/0000-0002-2701-0618

FU Monash University Malaysia (MUM); HDR scholarship

FX Y.N.P. acknowledges Monash University Malaysia (MUM) for support with a

HDR scholarship.

CR Abudayyak M, 2015, TURK J MED SCI, V45, P496, DOI 10.3906/sag-1401-153

Al-Snafi A. E., 2019, INT J CURR PHARM RES, V8, P1

Andersson Å, 2008, J LEUKOCYTE BIOL, V84, P1248, DOI 10.1189/jlb.1207844

Andersson U, 2018, SEMIN IMMUNOL, V38, P40, DOI 10.1016/j.smim.2018.02.011

Andersson U, 2018, EXPERT OPIN THER TAR, V22, P263, DOI 10.1080/14728222.2018.1439924

Angelopoulou E, 2019, PHARMACOL RES, V150, DOI 10.1016/j.phrs.2019.104515

Angelopoulou E, 2018, J NEUROCHEM, V146, P211, DOI 10.1111/jnc.14450

Angelopoulou E, 2016, J MOL MED, V94, P867, DOI 10.1007/s00109-016-1435-y

Anwar F., 2015, ARCH MED, V1, P1, DOI DOI 10.21767/2472-0151.10001

Aucott H, 2018, J INNATE IMMUN, V10, P215, DOI 10.1159/000487056

Bar FMA, 2019, NAT PROD RES, V33, P2591, DOI [10.1080/14786419.2018.1462177, 10.1080/14786419.2018.1503658]

Baran A, 2019, NEUROL SCI, V40, P167, DOI 10.1007/s10072-018-3611-z

Barman A, 2016, INDIAN J PSYCHOL MED, V38, P172, DOI 10.4103/0253-7176.183086

BEATON JM, 1956, J CHEM SOC, P2417, DOI 10.1039/jr9560002417

Bianchi ME, 2007, IMMUNOL REV, V220, P35, DOI 10.1111/j.1600-065X.2007.00574.x

Brambilla R, 2019, ACTA NEUROPATHOL, V137, P757, DOI 10.1007/s00401-019-01980-7

Cai ZY, 2016, CELL MOL NEUROBIOL, V36, P483, DOI 10.1007/s10571-015-0233-3

Catena R, 2009, BIOL REPROD, V80, P358, DOI 10.1095/biolreprod.108.070243

Chang HC, 2019, NUTRIENTS, V11, DOI 10.3390/nu11020252

Chen H., 2019, J THERM SCI, V1, P1, DOI DOI 10.1016/j.biopha.2019.109277

Costello DA, 2011, J NEUROSCI, V31, P3871, DOI 10.1523/JNEUROSCI.6676-10.2011

D'Ambrosio A, 2020, MULT SCLER J, V26, P476, DOI 10.1177/1352458519837707

Damle M., 2014, International Journal of Herbal Medicine, V2, P132

Dendrou CA, 2015, NAT REV IMMUNOL, V15, P545, DOI 10.1038/nri3871

Dhingra D, 2004, J ETHNOPHARMACOL, V91, P361, DOI 10.1016/j.jep.2004.01.016

Du YS, 2001, P NATL ACAD SCI USA, V98, P14669, DOI 10.1073/pnas.251341998

Elger CE, 2004, LANCET NEUROL, V3, P663, DOI 10.1016/S1474-4422(04)00906-8

Fang P, 2012, MOL NEUROBIOL, V45, P499, DOI 10.1007/s12035-012-8264-y

Fleminger S, 2003, J NEUROL NEUROSUR PS, V74, P857, DOI 10.1136/jnnp.74.7.857

Frischer JM, 2009, BRAIN, V132, P1175, DOI 10.1093/brain/awp070

Fu LL, 2017, SCI REP-UK, V7, DOI 10.1038/srep45291

Fujita K, 2016, SCI REP-UK, V6, DOI 10.1038/srep31895

Gao TL, 2018, BIOCHEM BIOPH RES CO, V497, P430, DOI 10.1016/j.bbrc.2018.02.102

Gibbs RM, 2018, CELL STEM CELL, V23, P21, DOI 10.1016/j.stem.2018.05.019

Girard JP, 2007, CHEM BIOL, V14, P345, DOI 10.1016/j.chembiol.2007.04.001

González-Reyes S, 2016, EPILEPSY RES, V126, P126, DOI 10.1016/j.eplepsyres.2016.07.007

Gu XJ, 2014, CHIN J TRAUMATOL, V17, P1, DOI 10.3760/cma.j.issn.1008-1275.2014.01.001

Guo J, 2016, CNS NEUROL DISORD-DR, V15, P1001, DOI 10.2174/1871527315666160527163526

Harris HE, 2012, NAT REV RHEUMATOL, V8, P195, DOI 10.1038/nrrheum.2011.222

Hay J, 2016, ANNU REV PATHOL-MECH, V11, P21, DOI 10.1146/annurev-pathol-012615-044116

Hei Y, 2018, NEUROSCIENCE, V383, P150, DOI 10.1016/j.neuroscience.2018.05.010

Heneka MT, 2015, LANCET NEUROL, V14, P388, DOI 10.1016/S1474-4422(15)70016-5

Hirsch EC, 2009, LANCET NEUROL, V8, P382, DOI 10.1016/S1474-4422(09)70062-6

Hughes CD, 2019, ACTA NEUROPATHOL, V137, P103, DOI 10.1007/s00401-018-1907-y

Ieong CC, 2018, J CLIN NEUROSCI, V47, P278, DOI 10.1016/j.jocn.2017.10.034

Isbrucker RA, 2006, REGUL TOXICOL PHARM, V46, P167, DOI 10.1016/j.yrtph.2006.06.002

ISHIDATE M, 1984, FOOD CHEM TOXICOL, V22, P623, DOI 10.1016/0278-6915(84)90271-0

Jang A, 2013, CURR ALZHEIMER RES, V10, P829

Jung KM, 2012, NEUROBIOL AGING, V33, P1522, DOI 10.1016/j.neurobiolaging.2011.03.012

Kalia LV, 2018, PARKINSONISM RELAT D, V46, pS19, DOI 10.1016/j.parkreldis.2017.07.023

Kazama H, 2008, IMMUNITY, V29, P21, DOI 10.1016/j.immuni.2008.05.013

Keezer MR, 2016, LANCET NEUROL, V15, P106, DOI 10.1016/S1474-4422(15)00225-2

Kim SW, 2012, NEUROBIOL DIS, V46, P147, DOI 10.1016/j.nbd.2011.12.056

Kim YJ, 2008, KOREAN J PHYSIOL PHA, V12, P65, DOI 10.4196/kjpp.2008.12.2.65

King E, 2019, INT PSYCHOGERIATR, V31, P551, DOI 10.1017/S1041610218001126

Kirkeby A, 2019, NAT REV NEUROL, V15, P312, DOI 10.1038/s41582-019-0180-6

Kochanek PM, 2015, SEMIN NEUROL, V35, P83, DOI 10.1055/s-0035-1544237

Kong DL, 2019, J CELL PHYSIOL, V234, P4597, DOI 10.1002/jcp.27242

Kong ZH, 2017, J MOL NEUROSCI, V63, P385, DOI 10.1007/s12031-017-0989-7

Kumar A, 2012, BRAIN BEHAV IMMUN, V26, P1191, DOI 10.1016/j.bbi.2012.06.008

Lee CH, 2007, BIOL PHARM BULL, V30, P1898, DOI 10.1248/bpb.30.1898

Li CY, 2017, THYROID, V27, P722, DOI 10.1089/thy.2016.0432

Li JH, 2003, MOL MED, V9, P37, DOI 10.1007/BF03402105

Li J, 2018, DNA CELL BIOL, V37, P941, DOI 10.1089/dna.2018.4444

Li M, 2017, NEUROSCI LETT, V639, P114, DOI 10.1016/j.neulet.2016.12.046

Li YJ, 2019, J PHARM PHARMACOL, V71, P390, DOI 10.1111/jphp.13040

Liu JJ, 2018, MOLECULES, V23, DOI 10.3390/molecules23051213

Liu WF, 2019, FOOD NUTR RES, V63, DOI 10.29219/fnr.v63.1516

Luo L, 2014, CELL MOL NEUROBIOL, V34, P987, DOI 10.1007/s10571-014-0075-4

Luo Lidan, 2013, Exp Neurobiol, V22, P107, DOI 10.5607/en.2013.22.2.107

Lv RX, 2019, LIFE SCI, V223, P158, DOI 10.1016/j.lfs.2019.03.030

Maas AIR, 2008, LANCET NEUROL, V7, P728, DOI 10.1016/S1474-4422(08)70164-9

Malhotra S, 2015, J NEUROINFLAMM, V12, DOI 10.1186/s12974-015-0269-9

Mamedov NA., 2019, Plant Hum Heal Pharmacol Ther Uses, V3, P1, DOI [DOI 10.1007/978-3-030-04408-4_1, DOI 10.1007/978-3-030-04408-41]

Maroso M, 2010, NAT MED, V16, P413, DOI 10.1038/nm.2127

Mazarati A, 2011, EXP NEUROL, V232, P143, DOI 10.1016/j.expneurol.2011.08.012

McAfoose J, 2009, NEUROSCI BIOBEHAV R, V33, P355, DOI 10.1016/j.neubiorev.2008.10.005

McGeer EG, 2003, PROG NEURO-PSYCHOPH, V27, P741, DOI 10.1016/S0278-5846(03)00124-6

Mollica L, 2007, CHEM BIOL, V14, P431, DOI 10.1016/j.chembiol.2007.03.007

Musumeci D, 2014, PHARMACOL THERAPEUT, V141, P347, DOI 10.1016/j.pharmthera.2013.11.001

Nazari S, 2017, PHYTOTHER RES, V31, P1635, DOI 10.1002/ptr.5893

Nose M, 2017, J NAT MED-TOKYO, V71, P711, DOI 10.1007/s11418-017-1101-x

Ohnishi M, 2011, NEUROPHARMACOLOGY, V61, P975, DOI 10.1016/j.neuropharm.2011.06.026

Ojha S, 2016, NEUROTOX RES, V29, P275, DOI 10.1007/s12640-015-9579-z

Okuma Y, 2019, WORLD NEUROSURG, V122, pE864, DOI 10.1016/j.wneu.2018.10.164

Okuma Y, 2014, NEUROPHARMACOLOGY, V85, P18, DOI 10.1016/j.neuropharm.2014.05.007

Okuma Y, 2012, ANN NEUROL, V72, P373, DOI 10.1002/ana.23602

Pandey A, 2018, CYTOKINE, V102, P211, DOI 10.1016/j.cyto.2017.10.019

Pang HG, 2016, MEDIAT INFLAMM, V2016, DOI 10.1155/2016/4569521

Papaevgeniou N, 2016, ANTIOXID REDOX SIGN, V25, P855, DOI 10.1089/ars.2015.6494

Parker TM, 2017, BRAIN INJURY, V31, P2, DOI 10.1080/02699052.2016.1217045

Parvez MK, 2018, CURR DRUG METAB, V19, P424, DOI 10.2174/1389200218666170710190249

Paudel Y. N., 2020, PHARM RES, V152, P4

Paudel YN, 2019, LIFE SCI, V238, DOI 10.1016/j.lfs.2019.116924

Paudel YN, 2019, EUR J PHARMACOL, V858, DOI 10.1016/j.ejphar.2019.172487

Paudel YN, 2019, J NEUROCHEM, V151, P542, DOI 10.1111/jnc.14663

Paudel YN, 2018, FRONT NEUROSCI-SWITZ, V12, DOI 10.3389/fnins.2018.00628

Paudel YN, 2018, EUR J PHARMACOL, V837, P145, DOI 10.1016/j.ejphar.2018.08.020

Pradeep H., 2019, CURCUMIN NEUROL PSYC, P301

Ravizza T, 2018, BRAIN BEHAV IMMUN, V72, P14, DOI 10.1016/j.bbi.2017.10.008

Rehman MU, 2019, CURR NEUROPHARMACOL, V17, P247, DOI 10.2174/1570159X16666180911124605

Richard S A., 2017, Journal of Behavioral and Brain Science, V7, DOI [DOI 10.4236/JBBS.2017.72006, 10.4236/JBBS.2017.72006]

Robinson AP, 2013, J AUTOIMMUN, V43, P32, DOI 10.1016/j.jaut.2013.02.005

Rodriguez-Otormin F, 2019, WIRES NANOMED NANOBI, V11, DOI 10.1002/wnan.1532

Rosciszewski G, 2019, FRONT CELL NEUROSCI, V13, DOI 10.3389/fncel.2019.00380

Rosenberg GA, 2002, NEUROSCIENTIST, V8, P586, DOI 10.1177/1073858402238517

Santoro M, 2016, NEUROBIOL DIS, V91, P59, DOI 10.1016/j.nbd.2016.02.018

Sasaki T, 2016, EXP NEUROL, V275, P220, DOI 10.1016/j.expneurol.2015.11.003

Sathyamoorthy Y, 2020, NUTR NEUROSCI, V23, P955, DOI 10.1080/1028415X.2019.1580935

Semple BD, 2019, NEUROBIOL DIS, V123, P27, DOI 10.1016/j.nbd.2018.07.018

Sharifzadeh M, 2008, PLANTA MED, V74, P485, DOI 10.1055/s-2008-1074494

Song JH, 2013, MOLECULES, V18, P15788, DOI 10.3390/molecules181215788

Streit WJ, 2004, J NEUROINFLAMM, V1, DOI 10.1186/1742-2094-1-14

Stros M, 2010, BBA-GENE REGUL MECH, V1799, P101, DOI 10.1016/j.bbagrm.2009.09.008

Sun XJ, 2018, EXP CELL RES, V369, P112, DOI 10.1016/j.yexcr.2018.05.012

Tabuchi M, 2012, CELL MOL NEUROBIOL, V32, P1139, DOI 10.1007/s10571-012-9839-x

Tewari D, 2017, FRONT PHARMACOL, V8, DOI 10.3389/fphar.2017.00519

Thengane SR, 1998, IN VITRO CELL DEV-PL, V34, P331, DOI 10.1007/BF02822743

Trinka E, 2019, EPILEPSIA, V60, P7, DOI 10.1111/epi.14458

Tundis R, 2018, NAT PROD DRUG DISC, P63, DOI 10.1016/B978-0-12-809593-5.00003-3

Venereau E, 2016, PHARMACOL RES, V111, P534, DOI 10.1016/j.phrs.2016.06.031

Voet S, 2019, TRENDS MOL MED, V25, P112, DOI 10.1016/j.molmed.2018.11.005

Walker LE, 2017, J CLIN INVEST, V127, P2118, DOI 10.1172/JCI92001

Wan WB, 2016, J IMMUNOL RES, V2016, DOI 10.1155/2016/6430423

Wang H, 2004, J INTERN MED, V255, P320, DOI 10.1111/j.1365-2796.2003.01302.x

[王怀立 Wang Huaili], 2017, [中华实用儿科临床杂志, Chinese Journal of Applied Clinical Pediatrics], V32, P1111

Wang W, 2016, NEUROSCIENCE, V316, P328, DOI 10.1016/j.neuroscience.2015.11.001

Webster KM, 2019, EXP NEUROL, V320, DOI 10.1016/j.expneurol.2019.112979

Webster KM, 2017, J NEUROINFLAMM, V14, DOI 10.1186/s12974-016-0786-1

Woodcock T, 2013, FRONT NEUROL, V4, DOI 10.3389/fneur.2013.00018

Wu CX, 2015, SHOCK, V43, P412, DOI 10.1097/SHK.0000000000000309

Xiong XX, 2016, J NEUROINFLAMM, V13, DOI 10.1186/s12974-016-0705-5

Yamaguchi H, 2012, BIOINFORMATION, V8, P1147, DOI 10.6026/97320630081147

Yan S., 2018, WIRELESS PERS COMMUN, P1

Yang EJ, 2019, PHYTOMEDICINE, V54, P56, DOI 10.1016/j.phymed.2018.09.189

Yang H, 2005, J LEUKOCYTE BIOL, V78, P1, DOI 10.1189/jlb.1104648

Yang H, 2004, P NATL ACAD SCI USA, V101, P296, DOI 10.1073/pnas.2434651100

Yang Y, 2018, BRAIN BEHAV, V8, DOI 10.1002/brb3.948

Yasui S, 2011, DIGEST DIS SCI, V56, P3638, DOI 10.1007/s10620-011-1789-5

Yim SB, 2007, J PHARMACOL EXP THER, V321, P816, DOI 10.1124/jpet.107.119602

Zandarashvili L, 2013, J BIOL CHEM, V288, P11621, DOI 10.1074/jbc.M113.449942

Zhang WD, 2007, PLANTA MED, V73, P336, DOI 10.1055/s-2007-967136

Zhao H, 2013, CNS NEUROSCI THER, V19, P117, DOI 10.1111/cns.12043

Zhao JL, 2017, BRAIN BEHAV IMMUN, V64, P308, DOI 10.1016/j.bbi.2017.02.002

Zhu XL, 2012, PLOS ONE, V7, DOI 10.1371/journal.pone.0035823

NR 145

TC 50

Z9 58

U1 2

U2 35

PU AMER CHEMICAL SOC

PI WASHINGTON

PA 1155 16TH ST, NW, WASHINGTON, DC 20036 USA

SN 1948-7193

J9 ACS CHEM NEUROSCI

JI ACS Chem. Neurosci.

PD FEB 19

PY 2020

VL 11

IS 4

BP 485

EP 500

DI 10.1021/acschemneuro.9b00640

PG 31

WC Biochemistry & Molecular Biology; Chemistry, Medicinal; Neurosciences

WE Science Citation Index Expanded (SCI-EXPANDED)

SC Biochemistry & Molecular Biology; Pharmacology & Pharmacy; Neurosciences

& Neurology

GA KN9VQ

UT WOS:000515195800001

PM 31972087

DA 2024-08-12

ER

PT J

AU Ren, MR

Guo, Y

Wei, XB

Yan, SQ

Qin, Y

Zhang, XM

Jiang, F

Lou, HY

AF Ren, Manru

Guo, Ying

Wei, Xinbing

Yan, Shaoqi

Qin, Yue

Zhang, Xiumei

Jiang, Fan

Lou, Haiyan

TI TREM2 overexpression attenuates neuroinflammation and protects

dopaminergic neurons in experimental models of Parkinson's disease

SO EXPERIMENTAL NEUROLOGY

LA English

DT Article

DE Triggering receptor expressed on myeloid cells-2; Parkinson's disease;

Microglia; Neuroinflammation

ID MYELOID CELLS 2; MOUSE MODEL; UP-REGULATION; CUTTING EDGE; MICROGLIA;

INFLAMMATION; MPTP; DEFICIENCY; CLEARANCE; RESPONSES

AB Triggering receptor expressed on myeloid cells-2 (TREM2) was a newly identified receptor expressed on microglia. Several observations support the hypothesis that TREM2 variation may confer susceptibility to Parkinson's disease (PD). Therefore, in this paper, we explored the role of TREM2 in the 1-methyl-4-phenyl-1,2,3,6-tetrahydropyridine (MPTP) mouse model of PD. Our results revealed that overexpression of TREM2 remarkably reduced MPTP-induced neuropathology including the dopaminergic neurodegeneration and neuroinflammation in vivo. Further mechanistic study revealed that TREM2 inhibited neuroinflammation by negatively regulating the TRAF6/TLR4-mediated activation of the MAPK and NF-kappa B signaling pathways. Taken together, our data suggest that TREM2 may have important neuroprotective effects against PD by critically modulating neuroinflammatory responses. These findings provide insights into the role of TREM2 in PD pathogenesis, and highlight TREM2 as a potential therapeutic target for this kind of disease.

C1 [Ren, Manru; Guo, Ying; Wei, Xinbing; Yan, Shaoqi; Qin, Yue; Zhang, Xiumei; Lou, Haiyan] Shandong Univ, Dept Pharmacol, Sch Med, 44 Wenhua Xi Rd, Jinan 250012, Shandong, China.

[Jiang, Fan] Shandong Univ, Sch Med, Dept Pathophysiol, Jinan 250012, Shandong, China.

C3 Shandong University; Shandong University

RP Lou, HY (corresponding author), Shandong Univ, Dept Pharmacol, Sch Med, 44 Wenhua Xi Rd, Jinan 250012, Shandong, China.

EM louhaiyan@sdu.edu.cn

RI zhou, you/KBC-3567-2024; Jiang, Fan/B-7753-2013; jiang,

fan/KHV-8640-2024

OI Jiang, Fan/0000-0001-9466-2192;

FU Shandong Province Science and Technology Program [2016GSF201061,

2017GSF18187]; Foundation for Young Excellent Scientists of Shandong

Province [BS2013YY032]

FX This study was supported by grants from Shandong Province Science and

Technology Program (No. 2016GSF201061, No. 2017GSF18187) and Foundation

for Young Excellent Scientists of Shandong Province (BS2013YY032).

CR Belloli S, 2017, NEUROBIOL AGING, V53, P159, DOI 10.1016/j.neurobiolaging.2017.01.010

Block ML, 2007, NAT REV NEUROSCI, V8, P57, DOI 10.1038/nrn2038

Bouchon A, 2000, J IMMUNOL, V164, P4991, DOI 10.4049/jimmunol.164.10.4991

Bruno A, 2013, NEW ENGL J MED, V369, P1567

Chen ZH, 2016, J NEUROCHEM, V136, P10, DOI 10.1111/jnc.13062

Colonna M, 2003, NAT REV IMMUNOL, V3, P445, DOI 10.1038/nri1106

Glass CK, 2010, CELL, V140, P918, DOI 10.1016/j.cell.2010.02.016

Guerreiro R, 2013, NEW ENGL J MED, V368, P117, DOI 10.1056/NEJMoa1211851

Hickman SE, 2014, BIOCHEM PHARMACOL, V88, P495, DOI 10.1016/j.bcp.2013.11.021

Hsieh CL, 2009, J NEUROCHEM, V109, P1144, DOI 10.1111/j.1471-4159.2009.06042.x

Hu XM, 2015, NAT REV NEUROL, V11, P56, DOI 10.1038/nrneurol.2014.207

Ito H, 2012, EUR J IMMUNOL, V42, P176, DOI 10.1002/eji.201141679

Jackson-Lewis V, 2007, NAT PROTOC, V2, P141, DOI 10.1038/nprot.2006.342

Jha MK, 2016, BIOCHEM PHARMACOL, V103, P1, DOI 10.1016/j.bcp.2015.11.003

Jiang T, 2016, NEUROPHARMACOLOGY, V105, P196, DOI 10.1016/j.neuropharm.2016.01.028

Jiang T, 2014, NEUROPSYCHOPHARMACOL, V39, P2949, DOI 10.1038/npp.2014.164

Jiang T, 2014, NEUROBIOL AGING, V35, P1243, DOI 10.1016/j.neurobiolaging.2013.11.026

Kawabori M, 2015, J NEUROSCI, V35, P3384, DOI 10.1523/JNEUROSCI.2620-14.2015

Kurosaki R, 2004, PHARMACOL BIOCHEM BE, V78, P143, DOI 10.1016/j.pbb.2004.03.006

Liu GY, 2016, MOL NEUROBIOL, V53, P4931, DOI 10.1007/s12035-015-9416-7

Lue LF, 2015, BRAIN PATHOL, V25, P469, DOI 10.1111/bpa.12190

MCGEER PL, 1988, NEUROLOGY, V38, P1285, DOI 10.1212/WNL.38.8.1285

Noelker C, 2013, SCI REP-UK, V3, DOI 10.1038/srep01393

Paloneva J, 2003, J EXP MED, V198, P669, DOI 10.1084/jem.20030027

Pena-Altamira E, 2015, EXPERT OPINION THERA, P1

Piccio L, 2007, EUR J IMMUNOL, V37, P1290, DOI 10.1002/eji.200636837

Pisanu A, 2014, NEUROBIOL DIS, V71, P280, DOI 10.1016/j.nbd.2014.08.011

Pottier C, 2013, J ALZHEIMERS DIS, V35, P45, DOI 10.3233/JAD-122311

Przedborski S, 2001, J NEUROCHEM, V76, P1265, DOI 10.1046/j.1471-4159.2001.00183.x

Rayaprolu S, 2013, MOL NEURODEGENER, V8, DOI 10.1186/1750-1326-8-19

Takahashi K, 2005, J EXP MED, V201, P647, DOI 10.1084/jem.20041611

Takahashi K, 2007, PLOS MED, V4, P675, DOI 10.1371/journal.pmed.0040124

Turnbull IR, 2006, J IMMUNOL, V177, P3520, DOI 10.4049/jimmunol.177.6.3520

Walter J, 2016, J BIOL CHEM, V291, P4334, DOI 10.1074/jbc.R115.704981

Whitton PS, 2007, BRIT J PHARMACOL, V150, P963, DOI 10.1038/sj.bjp.0707167

Wu R, 2017, MOL BRAIN, V10, DOI 10.1186/s13041-017-0296-9

Yu JT, 2014, NEUROBIOL AGING, V35, DOI [10.1016/j.neurobiolaging.2013.11.019, 10.1016/j.neurobiolaging.2013.10.075]

Zhong L, 2015, J BIOL CHEM, V290, P15866, DOI 10.1074/jbc.M115.645986

Zhu KY, 2011, J CHROMATOGR B, V879, P737, DOI 10.1016/j.jchromb.2011.02.011

NR 39

TC 79

Z9 86

U1 0

U2 25

PU ACADEMIC PRESS INC ELSEVIER SCIENCE

PI SAN DIEGO

PA 525 B ST, STE 1900, SAN DIEGO, CA 92101-4495 USA

SN 0014-4886

EI 1090-2430

J9 EXP NEUROL

JI Exp. Neurol.

PD APR

PY 2018

VL 302

BP 205

EP 213

DI 10.1016/j.expneurol.2018.01.016

PG 9

WC Neurosciences

WE Science Citation Index Expanded (SCI-EXPANDED)

SC Neurosciences & Neurology

GA GD1MZ

UT WOS:000430265300020

PM 29407460

DA 2024-08-12

ER

PT J

AU Minchev, D

Kazakova, M

Sarafian, V

AF Minchev, Danail

Kazakova, Maria

Sarafian, Victoria

TI Neuroinflammation and Autophagy in Parkinson's Disease-Novel

Perspectives

SO INTERNATIONAL JOURNAL OF MOLECULAR SCIENCES

LA English

DT Review

DE Parkinson's disease (PD); neurodegeneration; neuroinflammation;

autophagy

ID CEREBROSPINAL-FLUID BIOMARKERS; NF-KAPPA-B; ALPHA-SYNUCLEIN;

NEUROTROPHIC FACTOR; LYSOSOMAL DEGRADATION; SIGNALING PATHWAYS;

SUBSTANTIA-NIGRA; DOPAMINE NEURONS; ESSENTIAL ROLES; ULK1 COMPLEX

AB Parkinson's disease (PD) is the second most prevalent neurodegenerative disorder. It is characterized by the accumulation of alpha-Synuclein aggregates and the degeneration of dopaminergic neurons in substantia nigra in the midbrain. Although the exact mechanisms of neuronal degeneration in PD remain largely elusive, various pathogenic factors, such as alpha-Synuclein cytotoxicity, mitochondrial dysfunction, oxidative stress, and pro-inflammatory factors, may significantly impair normal neuronal function and promote apoptosis. In this context, neuroinflammation and autophagy have emerged as crucial processes in PD that contribute to neuronal loss and disease development. They are regulated in a complex interconnected manner involving most of the known PD-associated genes. This review summarizes evidence of the implication of neuroinflammation and autophagy in PD and delineates the role of inflammatory factors and autophagy-related proteins in this complex condition. It also illustrates the particular significance of plasma and serum immune markers in PD and their potential to provide a personalized approach to diagnosis and treatment.

C1 [Minchev, Danail; Kazakova, Maria; Sarafian, Victoria] Med Univ Plovdiv, Dept Med Biol, Plovdiv 4000, Bulgaria.

[Minchev, Danail; Kazakova, Maria; Sarafian, Victoria] Med Univ Plovdiv, Res Inst, Plovdiv 4000, Bulgaria.

C3 Medical University Plovdiv; Medical University Plovdiv

RP Minchev, D (corresponding author), Med Univ Plovdiv, Dept Med Biol, Plovdiv 4000, Bulgaria.; Minchev, D (corresponding author), Med Univ Plovdiv, Res Inst, Plovdiv 4000, Bulgaria.

EM danail.minchev@mu-plovdiv.bg

RI Minchev, Danail/AAR-2012-2020

FU National Science Fund of Bulgaria [D01-395/18.12.2020]; Medical

University-Plovdiv [????-01/2021]

FX This work was financially supported by the National Science Fund of

Bulgaria, Grant NUCBAR-BBMRI.BG (H?K???/BBMRI-BG), Agreement No.

D01-395/18.12.2020 and by Medical University-Plovdiv, Grant No.

????-01/2021.

CR Abudu YP, 2019, DEV CELL, V49, P509, DOI 10.1016/j.devcel.2019.03.013

Akhmetzyanova E, 2019, FRONT SYST NEUROSCI, V13, DOI 10.3389/fnsys.2019.00037

Alegre-Abarrategui J, 2009, AUTOPHAGY, V5, P1208, DOI 10.4161/auto.5.8.9894

Alers S, 2012, MOL CELL BIOL, V32, P2, DOI 10.1128/MCB.06159-11

Anderson G, 2020, CURR TOP MED CHEM, V20, P524, DOI 10.2174/1568026620666200131094445

Anderson MA, 2016, NATURE, V532, P195, DOI 10.1038/nature17623

Armstrong MJ, 2020, JAMA-J AM MED ASSOC, V323, P548, DOI 10.1001/jama.2019.22360

Ates G, 2022, EXP BIOL MED, V247, P1080, DOI 10.1177/15353702221084933

Audesse AJ, 2019, PLOS GENET, V15, DOI 10.1371/journal.pgen.1008097

Bandhyopadhyay U, 2007, EXP GERONTOL, V42, P120, DOI 10.1016/j.exger.2006.05.019

Bandopadhyay R, 2004, BRAIN, V127, P420, DOI 10.1093/brain/awh054

Bantle CM, 2021, FRONT CELL DEV BIOL, V8, DOI 10.3389/fcell.2020.608026

Bereciartu JD, 2020, ANAT REC, V303, P1232, DOI 10.1002/ar.24266

Berglund R, 2020, SCI IMMUNOL, V5, DOI 10.1126/sciimmunol.abb5077

Bhat KP, 2008, FEBS LETT, V582, P3193, DOI 10.1016/j.febslet.2008.08.010

Bjorklund G, 2020, PHARMACOL RES, V161, DOI 10.1016/j.phrs.2020.105065

Blanchet J, 2008, PROG NEURO-PSYCHOPH, V32, P1243, DOI 10.1016/j.pnpbp.2008.03.024

Bolaños JP, 2016, J NEUROCHEM, V139, P115, DOI 10.1111/jnc.13486

Booth HDE, 2017, TRENDS NEUROSCI, V40, P358, DOI 10.1016/j.tins.2017.04.001

Boukhalfa A, 2020, NAT COMMUN, V11, DOI 10.1038/s41467-019-14086-1

Brier LW, 2019, MOL BIOL CELL, V30, P1098, DOI 10.1091/mbc.E18-11-0743

Cadwell K, 2008, NATURE, V456, P259, DOI 10.1038/nature07416

Cerri S, 2019, CURR MED CHEM, V26, P3702, DOI 10.2174/0929867325666180226094351

Chen A, 2013, MOL MED REP, V8, P1011, DOI 10.3892/mmr.2013.1628

Cheng JB, 2020, AUTOPHAGY, V16, P2193, DOI 10.1080/15548627.2020.1719723

Cheong H, 2011, P NATL ACAD SCI USA, V108, P11121, DOI 10.1073/pnas.1107969108

Chhatbar C, 2021, CELL MOL IMMUNOL, V18, P250, DOI 10.1038/s41423-020-00620-5

Choi JS, 2010, BIOCHEM BIOPH RES CO, V391, P147, DOI 10.1016/j.bbrc.2009.11.022

Chong ZZ, 2012, FUTUR NEUROL, V7, P733, DOI 10.2217/FNL.12.72

Chounchay S, 2020, EXCLI J, V19, P950, DOI 10.17179/excli2020-2249

Chu CT, 2013, NAT CELL BIOL, V15, P1197, DOI 10.1038/ncb2837

Chu YP, 2009, NEUROBIOL DIS, V35, P385, DOI 10.1016/j.nbd.2009.05.023

Clayton DF, 1999, J NEUROSCI RES, V58, P120, DOI 10.1002/(SICI)1097-4547(19991001)58:1<120::AID-JNR12>3.0.CO;2-E

Clayton DF, 1998, TRENDS NEUROSCI, V21, P249, DOI 10.1016/S0166-2236(97)01213-7

Cortes CJ, 2014, DRUG DISCOV TODAY, V19, P963, DOI 10.1016/j.drudis.2014.02.014

Curry DW, 2018, J PARKINSON DIS, V8, P161, DOI 10.3233/JPD-171296

Czlonkowska A, 1996, NEURODEGENERATION, V5, P137, DOI 10.1006/neur.1996.0020

Dehay B, 2010, J NEUROSCI, V30, P12535, DOI 10.1523/JNEUROSCI.1920-10.2010

Di Rita A, 2018, NAT COMMUN, V9, DOI 10.1038/s41467-018-05722-3

Dubbelaar ML, 2018, FRONT IMMUNOL, V9, DOI 10.3389/fimmu.2018.01753

Dufek M, 2009, PARKINSONISM RELAT D, V15, P318, DOI 10.1016/j.parkreldis.2008.05.014

Dumitriu A, 2012, PLOS GENET, V8, DOI 10.1371/journal.pgen.1002794

Dursun E, 2015, J NEUROIMMUNOL, V283, P50, DOI 10.1016/j.jneuroim.2015.04.014

Egan DF, 2011, AUTOPHAGY, V7, P645, DOI 10.4161/auto.7.6.15123

Esteves AR, 2020, SCI REP-UK, V10, DOI 10.1038/s41598-020-70174-z

Foffani G, 2018, NEURON, V99, P1116, DOI 10.1016/j.neuron.2018.07.028

Galluzzi L, 2019, CELL, V177, P1682, DOI 10.1016/j.cell.2019.05.026

Gan BY, 2006, J CELL BIOL, V175, P121, DOI 10.1083/jcb.200604129

Gao F, 2017, FRONT NEUROL, V8, DOI 10.3389/fneur.2017.00527

Guo YF, 2021, J CELL PHYSIOL, V236, P4152, DOI 10.1002/jcp.30111

Hall S, 2016, MOVEMENT DISORD, V31, P898, DOI 10.1002/mds.26578

Hara T, 2006, NATURE, V441, P885, DOI 10.1038/nature04724

Harrison DE, 2009, NATURE, V460, P392, DOI 10.1038/nature08221

Henríquez-Olguín C, 2015, BBA-MOL BASIS DIS, V1852, P1410, DOI 10.1016/j.bbadis.2015.03.012

Heras-Sandoval D, 2014, CELL SIGNAL, V26, P2694, DOI 10.1016/j.cellsig.2014.08.019

Hieke N, 2015, AUTOPHAGY, V11, P1471, DOI 10.1080/15548627.2015.1068488

Hirsch Etienne C, 2012, Parkinsonism Relat Disord, V18 Suppl 1, pS210, DOI 10.1016/S1353-8020(11)70065-7

Ho MS, 2019, ADV EXP MED BIOL, V1175, P335, DOI 10.1007/978-981-13-9913-8_13

Horvath TL, 2011, NEUROBIOL DIS, V43, P152, DOI 10.1016/j.nbd.2011.03.005

Hou YS, 2015, MOL CELL BIOL, V35, P2740, DOI 10.1128/MCB.00285-15

Hua J, 2019, CELL MOL NEUROBIOL, V39, P1017, DOI 10.1007/s10571-019-00696-2

Hung SY, 2015, NEUROPHARMACOLOGY, V93, P243, DOI 10.1016/j.neuropharm.2015.02.003

Hunot S, 1996, NEUROSCIENCE, V72, P355, DOI 10.1016/0306-4522(95)00578-1

Ichimiya T, 2020, INT J MOL SCI, V21, DOI 10.3390/ijms21238974

Imamura K, 2003, ACTA NEUROPATHOL, V106, P518, DOI 10.1007/s00401-003-0766-2

Itakura E, 2008, MOL BIOL CELL, V19, P5360, DOI 10.1091/mbc.E08-01-0080

Jaber N, 2013, ANN NY ACAD SCI, V1280, P48, DOI 10.1111/nyas.12026

Jankovic J, 2020, J NEUROL NEUROSUR PS, V91, P795, DOI 10.1136/jnnp-2019-322338

Jimenez-Moreno N, 2020, OXID MED CELL LONGEV, V2020, DOI 10.1155/2020/8865611

Jin F, 2008, EUR J PHARMACOL, V600, P78, DOI 10.1016/j.ejphar.2008.10.005

Jin MM, 2018, FRONT AGING NEUROSCI, V10, DOI 10.3389/fnagi.2018.00378

Jung CH, 2009, MOL BIOL CELL, V20, P1992, DOI 10.1091/mbc.E08-12-1249

Kaizuka T, 2016, MOL CELL BIOL, V36, P585, DOI 10.1128/MCB.01005-15

Kaminska B, 2005, BBA-PROTEINS PROTEOM, V1754, P253, DOI 10.1016/j.bbapap.2005.08.017

Katila N, 2017, NEUROPHARMACOLOGY, V125, P396, DOI 10.1016/j.neuropharm.2017.08.015

Kazakova Maria H, 2009, Folia Med (Plovdiv), V51, P5

Kazakova MH, 2021, ARCH TRAUMA RES, V10, P23, DOI 10.4103/atr.atr_43_20

Kim J, 2011, NAT CELL BIOL, V13, P132, DOI 10.1038/ncb2152

Kim R, 2018, J CLIN NEUROSCI, V58, P30, DOI 10.1016/j.jocn.2018.10.079

Kim SY, 2012, EPILEPSIA, V53, P37, DOI 10.1111/j.1528-1167.2012.03701.x

Kishi-Itakura C, 2014, J CELL SCI, V127, P4089, DOI 10.1242/jcs.156034

Klionsky DJ, 2008, AUTOPHAGY, V4, P740, DOI 10.4161/auto.6398

Knott C, 2000, MOL CELL NEUROSCI, V16, P724, DOI 10.1006/mcne.2000.0914

Komatsu M, 2006, NATURE, V441, P880, DOI 10.1038/nature04723

Kouchaki E, 2018, CLIN NEUROL NEUROSUR, V166, P76, DOI 10.1016/j.clineuro.2018.01.022

Kundu M, 2008, BLOOD, V112, P1493, DOI 10.1182/blood-2008-02-137398

Lauro C, 2020, FRONT IMMUNOL, V11, DOI 10.3389/fimmu.2020.00493

Le WD, 2016, FRONT MOL NEUROSCI, V9, DOI 10.3389/fnmol.2016.00089

Lee EJ, 2011, AUTOPHAGY, V7, P689, DOI 10.4161/auto.7.7.15450

Lee HJ, 2010, J BIOL CHEM, V285, P9262, DOI 10.1074/jbc.M109.081125

Li WW, 2012, CELL MOL LIFE SCI, V69, P1125, DOI 10.1007/s00018-011-0865-5

Liddelow SA, 2017, NATURE, V541, P481, DOI 10.1038/nature21029

Lindholm P, 2007, NATURE, V448, P73, DOI 10.1038/nature05957

Lindmo K, 2008, AUTOPHAGY, V4, P500, DOI 10.4161/auto.5829

Lindqvist D, 2012, PLOS ONE, V7, DOI 10.1371/journal.pone.0047387

Liu HY, 2018, ONCOTARGET, V9, P2887, DOI [10.18632/oncotarget.20812, 10.18632/oncotarget.23208]

Liu WW, 2020, FASEB J, V34, P6570, DOI 10.1096/fj.201901565RR

Liu Y, 2018, MOL CANCER, V17, DOI 10.1186/s12943-018-0856-3

Losmanová T, 2020, OXID MED CELL LONGEV, V2020, DOI 10.1155/2020/8506572

Lu J., 2015, AUTOPHAGY BIOL DIS, V2, P21

Lu M, 2016, INT J NEUROPSYCHOPH, V19, DOI 10.1093/ijnp/pyw047

Lynch-Day MA, 2012, CSH PERSPECT MED, V2, DOI 10.1101/cshperspect.a009357

Lystad AH, 2019, AUTOPHAGY, V15, P1485, DOI 10.1080/15548627.2019.1618100

Magdalinou NK, 2015, J NEUROL NEUROSUR PS, V86, P1240, DOI 10.1136/jnnp-2014-309562

Mak SK, 2010, J BIOL CHEM, V285, P13621, DOI 10.1074/jbc.M109.074617

Martinez-Vicente M, 2008, J CLIN INVEST, V118, P777, DOI 10.1172/JCI32806

Matsui T, 2018, J CELL BIOL, V217, P2633, DOI 10.1083/jcb.201712058

McNeill A, 2014, BRAIN, V137, P1481, DOI 10.1093/brain/awu020

Menza M, 2010, PSYCHOSOMATICS, V51, P474, DOI 10.1176/appi.psy.51.6.474

Miyazaki I, 2020, CELLS-BASEL, V9, DOI 10.3390/cells9122623

Moehle MS, 2015, NEUROSCIENCE, V302, P59, DOI 10.1016/j.neuroscience.2014.11.018

Morales I, 2020, GLIA, V68, P2277, DOI 10.1002/glia.23839

Nikoletopoulou V, 2017, CELL METAB, V26, P230, DOI 10.1016/j.cmet.2017.06.005

Obeso JA, 2017, MOVEMENT DISORD, V32, P1264, DOI 10.1002/mds.27115

Ohsumi Y, 1999, PHILOS T ROY SOC B, V354, P1577, DOI 10.1098/rstb.1999.0501

Olsson B, 2013, MOVEMENT DISORD, V28, P1882, DOI 10.1002/mds.25589

Osawa T, 2019, PROTEIN SCI, V28, P1005, DOI 10.1002/pro.3623

Osawa T, 2019, NAT STRUCT MOL BIOL, V26, P281, DOI 10.1038/s41594-019-0203-4

Ouyang L, 2018, J MED CHEM, V61, P2776, DOI 10.1021/acs.jmedchem.7b01575

Pajares M, 2020, CELLS-BASEL, V9, DOI 10.3390/cells9071687

Papinski D, 2016, J MOL BIOL, V428, P1725, DOI 10.1016/j.jmb.2016.03.030

Patil SP, 2014, NEUROSCIENCE, V277, P747, DOI 10.1016/j.neuroscience.2014.07.046

Pemberton Samantha, 2012, Commun Integr Biol, V5, P94

Petrova PS, 2003, J MOL NEUROSCI, V20, P173, DOI 10.1385/JMN:20:2:173

Pinteac R, 2021, NEUROL-NEUROIMMUNOL, V8, DOI 10.1212/NXI.0000000000000921

Porras MAG, 2018, PHYSIOLOGY, V33, P211, DOI 10.1152/physiol.00008.2018

Reale M, 2009, BRAIN BEHAV IMMUN, V23, P55, DOI 10.1016/j.bbi.2008.07.003

Rocha NP, 2018, MOL NEUROBIOL, V55, P1488, DOI 10.1007/s12035-017-0404-y

Rocha NP, 2014, MOVEMENT DISORD, V29, P527, DOI 10.1002/mds.25752

Sciarretta S, 2018, ANNU REV PHYSIOL, V80, P1, DOI 10.1146/annurev-physiol-021317-121427

Selikhova MV, 2002, B EXP BIOL MED+, V133, P81, DOI 10.1023/A:1015120930920

Shahad FA, 2022, J POPUL THER CLIN PH, V29, pE61, DOI 10.47750/jptcp.2022.932

Shaw RJ, 2009, ACTA PHYSIOL, V196, P65, DOI 10.1111/j.1748-1716.2009.01972.x

Shih RH, 2015, FRONT MOL NEUROSCI, V8, DOI 10.3389/fnmol.2015.00077

Simunovic F, 2009, BRAIN, V132, P1795, DOI 10.1093/brain/awn323

Smith ED, 2014, J BIOL CHEM, V289, P20615, DOI 10.1074/jbc.M114.568659

Snead David, 2014, Exp Neurobiol, V23, P292, DOI 10.5607/en.2014.23.4.292

Sonninen TM, 2020, SCI REP-UK, V10, DOI 10.1038/s41598-020-71329-8

Spencer B, 2009, J NEUROSCI, V29, P13578, DOI 10.1523/JNEUROSCI.4390-09.2009

Strappazzon F, 2015, CELL DEATH DIFFER, V22, P419, DOI 10.1038/cdd.2014.139

Stykel MG, 2021, CELL REP, V35, DOI 10.1016/j.celrep.2021.109099

Su P, 2016, NEUROSCIENCE, V319, P155, DOI 10.1016/j.neuroscience.2016.01.035

Suzuki H, 2015, NAT STRUCT MOL BIOL, V22, P572, DOI 10.1038/nsmb.3036

Takáts S, 2013, J CELL BIOL, V201, P531, DOI 10.1083/jcb.201211160

Tang QL, 2021, CELL DEATH DIS, V12, DOI 10.1038/s41419-021-04138-0

Tang Y, 2018, FRONT AGING NEUROSCI, V10, DOI 10.3389/fnagi.2018.00154

Nguyen TN, 2016, J CELL BIOL, V215, P857, DOI 10.1083/jcb.201607039

Tizaoui K, 2022, INT J BIOL SCI, V18, P3731, DOI 10.7150/ijbs.67587

Troncoso-Escudero P, 2018, FRONT NEUROL, V9, DOI 10.3389/fneur.2018.00860

Tysnes OB, 2017, J NEURAL TRANSM, V124, P901, DOI 10.1007/s00702-017-1686-y

Ullian EM, 2004, GLIA, V47, P209, DOI 10.1002/glia.20082

Väänänen T, 2017, PLOS ONE, V12, DOI 10.1371/journal.pone.0183294

Vahsen BF, 2021, NEURAL REGEN RES, V16, P1212, DOI 10.4103/1673-5374.300442

Vilalta A, 2014, J NEUROINFLAMM, V11, DOI 10.1186/1742-2094-11-58

Villar-Piqué A, 2019, J NEUROINFLAMM, V16, DOI 10.1186/s12974-019-1531-3

Voloboueva LA, 2013, FEBS LETT, V587, P756, DOI 10.1016/j.febslet.2013.01.067

von Stockum Sophia, 2018, Oncotarget, V9, P12550, DOI 10.18632/oncotarget.23799

Voutilainen MH, 2009, J NEUROSCI, V29, P9651, DOI 10.1523/JNEUROSCI.0833-09.2009

Wallot-Hieke N, 2018, AUTOPHAGY, V14, P743, DOI 10.1080/15548627.2017.1387342

Wang CJ, 2021, TRANSL NEURODEGENER, V10, DOI 10.1186/s40035-021-00265-y

Wang QS, 2015, CLIN SCI, V129, P757, DOI 10.1042/CS20150008

Wang QX, 2014, TOXICOL IN VITRO, V28, P1183, DOI 10.1016/j.tiv.2014.05.006

Wang WY, 2015, ANN TRANSL MED, V3, DOI 10.3978/j.issn.2305-5839.2015.03.49

Webb AE, 2014, TRENDS BIOCHEM SCI, V39, P159, DOI 10.1016/j.tibs.2014.02.003

Webster CP, 2016, EMBO J, V35, P1656, DOI 10.15252/embj.201694401

Wennström M, 2015, PLOS ONE, V10, DOI 10.1371/journal.pone.0135458

Wesselborg S, 2015, CELL MOL LIFE SCI, V72, P4721, DOI 10.1007/s00018-015-2034-8

Williams-Gray CH, 2016, MOVEMENT DISORD, V31, P995, DOI 10.1002/mds.26563

Wong PM, 2013, AUTOPHAGY, V9, P124, DOI 10.4161/auto.23323

Wu DC, 2002, J NEUROSCI, V22, P1763, DOI 10.1523/JNEUROSCI.22-05-01763.2002

Wu YC, 2011, NEUROSIGNALS, V19, P163, DOI 10.1159/000328516

Xilouri M, 2010, CNS NEUROL DISORD-DR, V9, P701, DOI 10.2174/187152710793237421

Yamada K, 2022, J MED INVESTIG, V69, P101, DOI 10.2152/jmi.69.101

Yan C, 2019, CELL DEATH DIS, V10, DOI 10.1038/s41419-019-2039-6

Yan QT, 2017, MOL PHARMACOL, V92, P640, DOI 10.1124/mol.117.109512

Yang S, 2015, AGING CELL, V14, P1024, DOI 10.1111/acel.12371

Yun CW, 2018, INT J MOL SCI, V19, DOI 10.3390/ijms19113466

Yun HR, 2020, INT J MOL SCI, V21, DOI 10.3390/ijms21093289

Zahoor I., 2018, Parkinson's Disease: Pathogenesis and Clinical Aspects

Zhang GL, 2018, INT J MOL SCI, V19, DOI 10.3390/ijms19071905

Zhang YL, 2005, AM J MED GENET B, V137B, P5, DOI 10.1002/ajmg.b.30195

Zhou JY, 2012, AUTOPHAGY, V8, P1712, DOI 10.4161/auto.21830

NR 182

TC 14

Z9 15

U1 2

U2 15

PU MDPI

PI BASEL

PA ST ALBAN-ANLAGE 66, CH-4052 BASEL, SWITZERLAND

EI 1422-0067

J9 INT J MOL SCI

JI Int. J. Mol. Sci.

PD DEC

PY 2022

VL 23

IS 23

AR 14997

DI 10.3390/ijms232314997

PG 20

WC Biochemistry & Molecular Biology; Chemistry, Multidisciplinary

WE Science Citation Index Expanded (SCI-EXPANDED)

SC Biochemistry & Molecular Biology; Chemistry

GA 6Z0WU

UT WOS:000897504600001

PM 36499325

OA gold, Green Published

DA 2024-08-12

ER

PT J

AU Pietrzak, D

Kasperek, K

Rekawek, P

Piatkowska-Chmiel, I

AF Pietrzak, Diana

Kasperek, Kamila

Rekawek, Pawel

Piatkowska-Chmiel, Iwona

TI The Therapeutic Role of Ketogenic Diet in Neurological Disorders

SO NUTRIENTS

LA English

DT Review

DE ketogenic diet; neurological disorders; epilepsy; depression; migraine;

Alzheimer's disease; Parkinson's disease

ID MODIFIED ATKINS DIET; DRUG-RESISTANT EPILEPSY; PARS RETICULATA NEURONS;

ALTERED GUT MICROBIOME; CENTRAL-NERVOUS-SYSTEM; parkinson's disease;

OXIDATIVE STRESS; K-ATP; BETA-HYDROXYBUTYRATE; alzheimer's disease

AB The ketogenic diet (KD) is a high-fat, low-carbohydrate and adequate-protein diet that has gained popularity in recent years in the context of neurological diseases (NDs). The complexity of the pathogenesis of these diseases means that effective forms of treatment are still lacking. Conventional therapy is often associated with increasing tolerance and/or drug resistance. Consequently, more effective therapeutic strategies are being sought to increase the effectiveness of available forms of therapy and improve the quality of life of patients. For the moment, it seems that KD can provide therapeutic benefits in patients with neurological problems by effectively controlling the balance between pro- and antioxidant processes and pro-excitatory and inhibitory neurotransmitters, and modulating inflammation or changing the composition of the gut microbiome. In this review we evaluated the potential therapeutic efficacy of KD in epilepsy, depression, migraine, Alzheimer's disease and Parkinson's disease. In our opinion, KD should be considered as an adjuvant therapeutic option for some neurological diseases.

C1 [Pietrzak, Diana; Kasperek, Kamila; Rekawek, Pawel; Piatkowska-Chmiel, Iwona] Med Univ Lublin, Fac Pharm, Chair & Dept Toxicol, Jaczewskiego 8b St, PL-20090 Lublin, Poland.

C3 Medical University of Lublin

RP Pietrzak, D; Piatkowska-Chmiel, I (corresponding author), Med Univ Lublin, Fac Pharm, Chair & Dept Toxicol, Jaczewskiego 8b St, PL-20090 Lublin, Poland.

EM dianapchb@gmail.com; kamila.kasperek.pharm@gmail.com;

pabloo0705011@gmail.com; iwona.piatkowska-chmiel@umlub.pl

OI Pietrzak, Diana/0009-0003-5060-534X; Piatkowska-Chmiel,

Iwona/0000-0003-1829-6981

CR Aarsland D, 2007, J NEUROL, V254, P38, DOI 10.1007/s00415-006-0234-8

Al-Karagholi MAM, 2021, BRAIN, V144, P2322, DOI 10.1093/brain/awab136

Albury CL, 2017, MOL GENET GENOMICS, V292, P729, DOI 10.1007/s00438-017-1317-1

Alfonsetti M, 2022, NUTRIENTS, V14, DOI 10.3390/nu14020380

Andoh A, 2016, DIGESTION, V93, P176, DOI 10.1159/000444066

Ang QY, 2020, CELL, V181, P1263, DOI 10.1016/j.cell.2020.04.027

Ansari F, 2020, CURR PHARM BIOTECHNO, V21, P555, DOI 10.2174/1389201021666200107113812

Apostolova Liana G, 2016, Continuum (Minneap Minn), V22, P419, DOI 10.1212/CON.0000000000000307

Ari C, 2016, FRONT MOL NEUROSCI, V9, DOI [10.3389/fnmal.2016.00137, 10.3389/fnmol.2016.00137]

Ashrafi M, 2017, ACTA NEUROL BELG, V117, P175, DOI 10.1007/s13760-016-0732-0

Aureli C, 2014, J NEUROSCI RES, V92, P347, DOI 10.1002/jnr.23318

Baby N, 2018, SEIZURE-EUR J EPILEP, V58, P41, DOI 10.1016/j.seizure.2018.03.020

Banks WA, 2012, PHARMACOL THERAPEUT, V136, P82, DOI 10.1016/j.pharmthera.2012.07.006

Barbanti P, 2017, NEUROL SCI, V38, pS111, DOI 10.1007/s10072-017-2889-6

Barrett E, 2012, J APPL MICROBIOL, V113, P411, DOI 10.1111/j.1365-2672.2012.05344.x

Bartolini E, 2020, NEUROL SCI, V41, P749, DOI 10.1007/s10072-019-04190-x

Basciani S, 2020, J CLIN ENDOCR METAB, V105, P2939, DOI 10.1210/clinem/dgaa336

Bellou V, 2016, PARKINSONISM RELAT D, V23, P1, DOI 10.1016/j.parkreldis.2015.12.008

Bentourkia M, 2009, AM J PHYSIOL-ENDOC M, V296, pE796, DOI 10.1152/ajpendo.90644.2008

Bjurulf B, 2020, DEV MED CHILD NEUROL, V62, P57, DOI 10.1111/dmcn.14393

Boden G, 2005, ANN INTERN MED, V142, P403, DOI 10.7326/0003-4819-142-6-200503150-00006

Bravo JA, 2011, P NATL ACAD SCI USA, V108, P16050, DOI 10.1073/pnas.1102999108

Bröer S, 2020, FRONT SYST NEUROSCI, V14, DOI 10.3389/fnsys.2020.581826

Brownlow ML, 2013, PLOS ONE, V8, DOI 10.1371/journal.pone.0075713

Caberlotto L, 2016, SCI REP-UK, V6, DOI 10.1038/srep32583

Cacabelos R, 2017, INT J MOL SCI, V18, DOI 10.3390/ijms18030551

Cai QY, 2017, WORLD J PEDIATR, V13, P528, DOI 10.1007/s12519-017-0053-2

Caputi V, 2018, INT J MOL SCI, V19, DOI 10.3390/ijms19061689

Chauhan A, 2020, NUTRIENTS, V12, DOI 10.3390/nu12020550

Cheng CM, 2003, ENDOCRINOLOGY, V144, P2676, DOI 10.1210/en.2002-0057

Chia SJ, 2020, INT J MOL SCI, V21, DOI 10.3390/ijms21072464

Cho NH, 2018, DIABETES RES CLIN PR, V138, P271, DOI 10.1016/j.diabres.2018.02.023

Choi A, 2021, NEUROTHERAPEUTICS, V18, P1637, DOI 10.1007/s13311-021-01067-w

Conner JM, 1997, J NEUROSCI, V17, P2295

Cooke MS, 2003, FASEB J, V17, P1195, DOI 10.1096/fj.02-0752rev

Cotter DG, 2013, AM J PHYSIOL-ENDOC M, V304, pE363, DOI 10.1152/ajpendo.00547.2012

Crosby L, 2021, FRONT NUTR, V8, DOI 10.3389/fnut.2021.702802

Cryan JF, 2020, LANCET NEUROL, V19, P179, DOI 10.1016/S1474-4422(19)30356-4

Cullingford TE, 2004, PROSTAG LEUKOTR ESS, V70, P253, DOI 10.1016/j.plefa.2003.09.008

Dahlin M, 2019, EBIOMEDICINE, V44, P741, DOI 10.1016/j.ebiom.2019.05.024

Danbolt NC, 2001, PROG NEUROBIOL, V65, P1, DOI 10.1016/S0301-0082(00)00067-8

Orti JED, 2021, FRONT NUTR, V8, DOI 10.3389/fnut.2021.744398

Delerive P, 1999, J BIOL CHEM, V274, P32048, DOI 10.1074/jbc.274.45.32048

Deransart C, 2003, EPILEPSIA, V44, P1513, DOI 10.1111/j.0013-9580.2003.26603.x

Di Lorenzo C, 2019, NUTRIENTS, V11, DOI 10.3390/nu11081742

Duelli R, 2001, NEWS PHYSIOL SCI, V16, P71

Duncan SH, 2009, ENVIRON MICROBIOL, V11, P2112, DOI 10.1111/j.1462-2920.2009.01931.x

Elamin M, 2017, FRONT MOL NEUROSCI, V10, DOI 10.3389/fnmol.2017.00377

Elesawy BH, 2021, BRAIN SCI, V11, DOI 10.3390/brainsci11020242

Emmanouilidou E, 2016, BRAIN, V139, P871, DOI 10.1093/brain/awv403

Engelking L.R., 2015, Textbook of Veterinary Physiological Chemistry, P450, DOI DOI 10.1016/B978-0-12-391909-0.50071-2

Eor JY, 2021, EPILEPSY RES, V174, DOI 10.1016/j.eplepsyres.2021.106668

Erecinska M, 1996, J NEUROCHEM, V67, P2325

Evans-Lacko S, 2018, PSYCHOL MED, V48, P1560, DOI 10.1017/S0033291717003336

Farris W, 2003, P NATL ACAD SCI USA, V100, P4162, DOI 10.1073/pnas.0230450100

Feinman RD, 2015, NUTRITION, V31, P1, DOI 10.1016/j.nut.2014.06.011

Fernandez AM, 2017, DIABETES, V66, P64, DOI 10.2337/db16-0861

Fernandez AM, 2012, NAT REV NEUROSCI, V13, P225, DOI 10.1038/nrn3209

Floyd RA, 1999, P SOC EXP BIOL MED, V222, P236, DOI 10.1046/j.1525-1373.1999.d01-140.x

Forsyth CB, 2011, PLOS ONE, V6, DOI 10.1371/journal.pone.0028032

Forsyth E, 1998, PHYS THER, V78, P1325, DOI 10.1093/ptj/78.12.1325

Fortier M, 2021, ALZHEIMERS DEMENT, V17, P543, DOI 10.1002/alz.12206

Freeman JM, 1998, PEDIATRICS, V102, P1358, DOI 10.1542/peds.102.6.1358

Fu SP, 2015, J NEUROINFLAMM, V12, DOI 10.1186/s12974-014-0230-3

Gano LB, 2014, J LIPID RES, V55, P2211, DOI 10.1194/jlr.R048975

Gao C, 2020, TRANSL NEURODEGENER, V9, DOI 10.1186/s40035-020-00191-5

Giladi N, 2008, MOVEMENT DISORD, V23, pS423, DOI 10.1002/mds.21927

Ginter E., 2013, PANDEMIC 21 CENTURY, V771, P50, DOI [10.1007/978-1-4614-5441-0_6, DOI 10.1007/978-1-4614-5441-0_6]

Glaubic-Latka Marta, 2004, Neurol Neurochir Pol, V38, P307

Gourbeyre P, 2011, J LEUKOCYTE BIOL, V89, P685, DOI 10.1189/jlb.1109753

Gross EC, 2019, NUTRIENTS, V11, DOI 10.3390/nu11040811

Guan YF, 2020, BRAIN BEHAV IMMUN, V88, P748, DOI 10.1016/j.bbi.2020.05.032

Gupta D, 2014, PHYSIOL BEHAV, V129, P73, DOI 10.1016/j.physbeh.2014.02.036

Gupta S, 2021, INDIAN PEDIATR, V58, P815, DOI 10.1007/s13312-021-2299-y

LeBlanc JG, 2013, CURR OPIN BIOTECH, V24, P160, DOI 10.1016/j.copbio.2012.08.005

Guzel O, 2019, EUR J PAEDIATR NEURO, V23, P143, DOI 10.1016/j.ejpn.2018.11.007

Hamer HM, 2008, ALIMENT PHARM THER, V27, P104, DOI 10.1111/j.1365-2036.2007.03562.x

Hammen C, 2018, ANNU REV CLIN PSYCHO, V14, P1, DOI [10.1146/annurev-clinpsy-050817084811, 10.1146/annurev-clinpsy-050817-084811]

Hasan-Olive MM, 2019, NEUROCHEM RES, V44, P22, DOI 10.1007/s11064-018-2588-6

Hertz L, 2007, J CEREBR BLOOD F MET, V27, P219, DOI 10.1038/sj.jcbfm.9600343

Hertz L, 2016, ADV NEUROBIOL, V13, P9, DOI 10.1007/978-3-319-45096-4_2

Hildebrand MS, 2014, EPILEPSIA, V55, pE18, DOI 10.1111/epi.12519

Hoffmann U, 2013, J CEREBR BLOOD F MET, V33, P191, DOI 10.1038/jcbfm.2012.132

Holscher HD, 2017, GUT MICROBES, V8, P172, DOI 10.1080/19490976.2017.1290756

Hsiao FJ, 2022, J HEADACHE PAIN, V23, DOI 10.1186/s10194-022-01392-1

Hu B, 2020, COGN NEURODYNAMICS, V14, P137, DOI 10.1007/s11571-019-09559-4

Huang C, 2018, GLIA, V66, P256, DOI 10.1002/glia.23241

Huang RX, 2016, NUTRIENTS, V8, DOI 10.3390/nu8080483

Hughes SD, 2014, J NEUROCHEM, V129, P426, DOI 10.1111/jnc.12646

HUTTENLOCHER PR, 1971, NEUROLOGY, V21, P1097, DOI 10.1212/WNL.21.11.1097

Hyde PN, 2017, Cancer Treat Res Commun, V12, P32, DOI DOI 10.1016/J.CTARC.2017.06.001

Ijff DM, 2016, EPILEPSY BEHAV, V60, P153, DOI 10.1016/j.yebeh.2016.04.033

Jankovic J, 2008, J NEUROL NEUROSUR PS, V79, P368, DOI 10.1136/jnnp.2007.131045

Jasarevic E, 2016, PHILOS T R SOC B, V371, DOI 10.1098/rstb.2015.0122

Jeong EA, 2011, EXP NEUROL, V232, P195, DOI 10.1016/j.expneurol.2011.09.001

Juge N, 2010, NEURON, V68, P99, DOI 10.1016/j.neuron.2010.09.002

Juge N, 2006, J BIOL CHEM, V281, P39499, DOI 10.1074/jbc.M607670200

Juszczyk G, 2021, ANTIOXIDANTS-BASEL, V10, DOI 10.3390/antiox10091439

Karschin C, 1997, FEBS LETT, V401, P59, DOI 10.1016/S0014-5793(96)01438-X

Kashiwaya Y, 2000, P NATL ACAD SCI USA, V97, P5440, DOI 10.1073/pnas.97.10.5440

Kellar D, 2020, LANCET NEUROL, V19, P758, DOI 10.1016/S1474-4422(20)30231-3

Kerti L, 2013, NEUROLOGY, V81, P1746, DOI 10.1212/01.wnl.0000435561.00234.ee

Kim DY, 2015, PLOS ONE, V10, DOI 10.1371/journal.pone.0119316

Kim JA, 2016, EPILEPSIA, V57, P51, DOI 10.1111/epi.13256

Kim N, 2018, J MICROBIOL, V56, P172, DOI 10.1007/s12275-018-8032-4

Kleinridders A, 2013, J CLIN INVEST, V123, P4667, DOI 10.1172/JCI67615

Klimova B, 2019, NEUROPSYCH DIS TREAT, V15, P3033, DOI 10.2147/NDT.S218238

Koch H, 2019, EPILEPSY BEHAV, V91, P90, DOI 10.1016/j.yebeh.2018.06.010

Koppel SJ, 2018, NEUROCHEM INT, V117, P114, DOI 10.1016/j.neuint.2017.05.019

Kossoff EH, 2006, EPILEPSIA, V47, P421, DOI 10.1111/j.1528-1167.2006.00438.x

Kossoff E, 2020, EPILEPSY CURR, V20, P125, DOI 10.1177/1535759719890337

Kossoff EH, 2008, EPILEPSIA, V49, P37, DOI 10.1111/j.1528-1167.2008.01831.x

Kossoff EH, 2008, EPILEPSIA, V49, P11, DOI 10.1111/j.1528-1167.2008.01823.x

Kossoff Eric H, 2018, Epilepsia Open, V3, P175, DOI 10.1002/epi4.12225

Kumar A., 2022, ALZHEIMER DIS

Kuwahara Atsukazu, 2020, Biomed Res, V41, P199, DOI 10.2220/biomedres.41.199

Kverneland M, 2018, EPILEPSIA, V59, P1567, DOI 10.1111/epi.14457

Kverneland M, 2015, EPILEPSY BEHAV, V53, P197, DOI 10.1016/j.yebeh.2015.10.021

Kwon HE, 2021, ACTA EPILEPTOL, V3, DOI 10.1186/s42494-021-00053-1

Kysel P, 2020, NUTRIENTS, V12, DOI 10.3390/nu12092832

Laffel L, 1999, DIABETES-METAB RES, V15, P412, DOI 10.1002/(SICI)1520-7560(199911/12)15:6<412::AID-DMRR72>3.0.CO;2-8

Lakshminarayanan K, 2021, EPILEPSY RES, V171, DOI 10.1016/j.eplepsyres.2021.106574

Lambrechts DAJE, 2017, ACTA NEUROL SCAND, V135, P231, DOI 10.1111/ane.12592

Lauretti E, 2020, AGING CELL, V19, DOI 10.1111/acel.13076

Lee GS, 2012, NATURE, V492, P123, DOI 10.1038/nature11588

Lee HK, 2009, MOL BIOL CELL, V20, P1533, DOI 10.1091/mbc.E08-07-0777

Leino RL, 2001, NEUROCHEM INT, V38, P519, DOI 10.1016/S0197-0186(00)00102-9

Li J, 2016, CIRCULATION, V133, P2434, DOI 10.1161/CIRCULATIONAHA.115.019645

Licha D, 2019, INT J MOL SCI, V20, DOI 10.3390/ijms20163873

Lin A, 2017, PEDIATR NEUROL, V68, P35, DOI 10.1016/j.pediatrneurol.2017.01.007

Ling H, 2012, BRAIN, V135, P1141, DOI 10.1093/brain/aws038

Liu RT, 2019, NEUROSCI BIOBEHAV R, V102, P13, DOI 10.1016/j.neubiorev.2019.03.023

Lotankar S, 2017, NEUROSCI BULL, V33, P585, DOI 10.1007/s12264-017-0183-5

Lu B, 2014, Handb Exp Pharmacol, V220, P223, DOI 10.1007/978-3-642-45106-5_9

Lubomski M, 2020, J NEUROL, V267, P2507, DOI 10.1007/s00415-019-09320-1

Lutas A, 2014, J NEUROSCI, V34, P16336, DOI 10.1523/JNEUROSCI.1357-14.2014

Ma D, 2018, SCI REP-UK, V8, DOI 10.1038/s41598-018-25190-5

Ma QQ, 2019, J NEUROINFLAMM, V16, DOI 10.1186/s12974-019-1434-3

Ma SH, 2019, SPORTS, V7, DOI 10.3390/sports7020040

Ma WY, 2007, J NEUROSCI, V27, P3618, DOI 10.1523/JNEUROSCI.0132-07.2007

Maalouf M, 2007, NEUROSCIENCE, V145, P256, DOI 10.1016/j.neuroscience.2006.11.065

Malhi GS, 2018, LANCET, V392, P2299, DOI 10.1016/S0140-6736(18)31948-2

Manoharan S, 2016, OXID MED CELL LONGEV, V2016, DOI 10.1155/2016/8590578

Martínez-François JR, 2018, ELIFE, V7, DOI 10.7554/eLife.32721

Masino S.A., 2012, JASPERS BASIC MECH E, P1157

Masters CL, 2015, NAT REV DIS PRIMERS, V1, DOI 10.1038/nrdp.2015.56

Matin N, 2015, HUM VACC IMMUNOTHER, V11, P2021, DOI 10.1080/21645515.2015.1034921

Mayeux R, 2012, CSH PERSPECT MED, V2, DOI 10.1101/cshperspect.a006239

Mazzoni P, 2012, CSH PERSPECT MED, V2, DOI 10.1101/cshperspect.a009282

McNally MA, 2009, PEDIATRICS, V124, pE300, DOI 10.1542/peds.2009-0217

Melo TM, 2006, NEUROCHEM INT, V48, P498, DOI 10.1016/j.neuint.2005.12.037

Menezes LFS, 2020, FRONT PHARMACOL, V11, DOI 10.3389/fphar.2020.01276

Milder JB, 2010, NEUROBIOL DIS, V40, P238, DOI 10.1016/j.nbd.2010.05.030

Miller TL, 1996, APPL ENVIRON MICROB, V62, P1589, DOI 10.1128/AEM.62.5.1589-1592.1996

Monsalve M, 2007, FRONT BIOSCI-LANDMRK, V12, P1131, DOI 10.2741/2132

Morrill SJ, 2019, DIABETES METAB SYND, V13, P1187, DOI 10.1016/j.dsx.2019.01.035

Mu CL, 2022, EBIOMEDICINE, V76, DOI 10.1016/j.ebiom.2022.103838

Mujica-Parodi LR, 2020, P NATL ACAD SCI USA, V117, P6170, DOI 10.1073/pnas.1913042117

Myette-Côté É, 2021, PROSTAG LEUKOTR ESS, V169, DOI 10.1016/j.plefa.2020.102236

Navab M, 2008, CURR OPIN CLIN NUTR, V11, P459, DOI 10.1097/MCO.0b013e32830460c2

Neal EG, 2008, LANCET NEUROL, V7, P500, DOI 10.1016/S1474-4422(08)70092-9

Niepotter P., 2019, HAPS Educator, V23, P426, DOI DOI 10.21692/HAPS.2019.002

Noh HS, 2006, J NEUROSCI RES, V83, P702, DOI 10.1002/jnr.20736

NORENBERG MD, 1979, BRAIN RES, V161, P303, DOI 10.1016/0006-8993(79)90071-4

Olesen J, 2018, CEPHALALGIA, V38, P1, DOI 10.1177/0333102417738202

Olson CA, 2018, CELL, V173, P1728, DOI [10.1016/j.cell.2018.04.027, 10.1016/j.cell.2018.06.051]

Osadchiy V, 2019, CLIN GASTROENTEROL H, V17, P322, DOI 10.1016/j.cgh.2018.10.002

Ota M, 2019, NEUROSCI LETT, V690, P232, DOI 10.1016/j.neulet.2018.10.048

OWEN OE, 1967, J CLIN INVEST, V46, P1589, DOI 10.1172/JCI105650

Paoli A, 2019, GENES-BASEL, V10, DOI 10.3390/genes10070534

Paoli A, 2014, BIOMED RES INT, V2014, DOI 10.1155/2014/474296

Patel M, 2016, TRENDS PHARMACOL SCI, V37, P768, DOI 10.1016/j.tips.2016.06.007

Pavón S, 2021, NUTR REV, V79, P802, DOI 10.1093/nutrit/nuaa113

Pearson-Smith JN, 2017, INT J MOL SCI, V18, DOI 10.3390/ijms18112365

Phillips MCL, 2021, ALZHEIMERS RES THER, V13, DOI 10.1186/s13195-021-00783-x

Phillips MCL, 2018, MOVEMENT DISORD, V33, P1306, DOI 10.1002/mds.27390

Piane Maria, 2007, J Headache Pain, V8, P334, DOI 10.1007/s10194-007-0427-2

Plum L, 2005, TRENDS ENDOCRIN MET, V16, P59, DOI 10.1016/j.tem.2005.01.008

Pondel Natalia, 2020, Postepy Biochem, V66, P270, DOI 10.18388/pb.2020_342

Poorshiri B, 2021, ACTA NEUROL BELG, V121, P483, DOI 10.1007/s13760-019-01225-0

Putananickal N, 2022, CEPHALALGIA, V42, P302, DOI 10.1177/03331024211043792

Qiao G, 2020, FISH SHELLFISH IMMUN, V107, P444, DOI 10.1016/j.fsi.2020.11.005

Quigley EMM, 2017, CURR NEUROL NEUROSCI, V17, DOI 10.1007/s11910-017-0802-6

Oliveira MDR, 2008, NEUROSCI LETT, V434, P66, DOI 10.1016/j.neulet.2008.01.032

Reigstad CS, 2015, FASEB J, V29, P1395, DOI 10.1096/fj.14-259598

Rusek M, 2019, INT J MOL SCI, V20, DOI 10.3390/ijms20163892

Sakai R, 2004, J NEUROCHEM, V88, P612, DOI 10.1111/j.1471-4159.2004.02179.x

Salcedo-Tello Pamela, 2011, Int J Alzheimers Dis, V2011, P189728, DOI 10.4061/2011/189728

Sampson TR, 2016, CELL, V167, P1469, DOI 10.1016/j.cell.2016.11.018

Saveanu RV, 2012, PSYCHIAT CLIN N AM, V35, P51, DOI 10.1016/j.psc.2011.12.001

Scheperjans F, 2018, J PARKINSON DIS, V8, pS31, DOI 10.3233/JPD-181477

Schürks M, 2012, J HEADACHE PAIN, V13, P1, DOI 10.1007/s10194-011-0399-0

Shao BZ, 2018, FRONT MOL NEUROSCI, V11, DOI 10.3389/fnmol.2018.00320

Sharma S, 2016, EPILEPSY RES, V127, P152, DOI 10.1016/j.eplepsyres.2016.09.002

Shimazu T, 2013, SCIENCE, V339, P211, DOI 10.1126/science.1227166

SOKOLOFF L, 1991, ADV EXP MED BIOL, V291, P21

Stafstrom Carl E., 2003, Epilepsy Curr, V3, P148, DOI 10.1046/j.1535-7597.2003.03415.x

Stoykovich S, 2019, ALZH DEMENT-DADM, V11, P264, DOI 10.1016/j.dadm.2019.01.009

Sullivan PG, 2004, ANN NEUROL, V55, P576, DOI 10.1002/ana.20062

Sveinbjornsdottir S, 2016, J NEUROCHEM, V139, P318, DOI 10.1111/jnc.13691

Taggart AKP, 2005, J BIOL CHEM, V280, P26649, DOI 10.1074/jbc.C500213200

Tagliabue A, 2017, CLIN NUTR ESPEN, V17, P33, DOI 10.1016/j.clnesp.2016.11.003

Tanner GR, 2011, J NEUROSCI, V31, P8689, DOI 10.1523/JNEUROSCI.5951-10.2011

Terrone G, 2020, NEUROPHARMACOLOGY, V167, DOI 10.1016/j.neuropharm.2019.107742

Tian CA, 2014, CHEM BIOL DRUG DES, V83, P1, DOI 10.1111/cbdd.12237

Tillery Erika E, 2021, Ment Health Clin, V11, P211, DOI 10.9740/mhc.2021.05.211

Tokarz VL, 2018, J CELL BIOL, V217, P2273, DOI 10.1083/jcb.201802095

Torosyan N, 2018, EXP GERONTOL, V111, P118, DOI 10.1016/j.exger.2018.07.009

Tschopp J, 2010, NAT REV IMMUNOL, V10, P210, DOI 10.1038/nri2725

Ulamek-Koziol M, 2020, NEURAL REGEN RES, V15, P857, DOI 10.4103/1673-5374.268900

Ulamek-Koziol M, 2019, NUTRIENTS, V11, DOI 10.3390/nu11102510

Unnikrishnan R, 2017, DIABETES, V66, P1432, DOI 10.2337/db16-0766

Van Der Louw E, 2020, DEV MED CHILD NEUROL, V62, P48, DOI 10.1111/dmcn.14306

VanItallie TB, 2005, NEUROLOGY, V64, P728, DOI 10.1212/01.WNL.0000152046.11390.45

Vega-López S, 2018, NUTRIENTS, V10, DOI 10.3390/nu10101361

Verdile G, 2015, MEDIAT INFLAMM, V2015, DOI 10.1155/2015/105828

Vining EPG, 1998, ARCH NEUROL-CHICAGO, V55, P1433, DOI 10.1001/archneur.55.11.1433

Weinstein G, 2015, NEUROLOGY, V84, P2329, DOI 10.1212/WNL.0000000000001655

Wells J, 2020, NUTRIENTS, V12, DOI 10.3390/nu12061809

Wheless JW, 2008, EPILEPSIA, V49, P3, DOI 10.1111/j.1528-1167.2008.01821.x

Winter G, 2018, REV NEUROSCIENCE, V29, P629, DOI 10.1515/revneuro-2017-0072

Wlodarczyk A, 2021, PROG NEURO-PSYCHOPH, V109, DOI 10.1016/j.pnpbp.2021.110257

Wlodarek D, 2019, NUTRIENTS, V11, DOI 10.3390/nu11010169

Xie G, 2017, WORLD J GASTROENTERO, V23, P6164, DOI 10.3748/wjg.v23.i33.6164

Xie J, 2010, J NEUROSCI RES, V88, P428, DOI 10.1002/jnr.22213

Xu WR, 2021, DIABET METAB SYND OB, V14, P4361, DOI [10.2147/DMSO.S333373DovePress, 10.2147/DMSO.S333373]

Yamada K, 2005, J MOL CELL CARDIOL, V38, P945, DOI 10.1016/j.yjmcc.2004.11.020

Yang XX, 2010, J MOL NEUROSCI, V42, P145, DOI 10.1007/s12031-010-9336-y

Yasuda T, 2013, MOL NEUROBIOL, V47, P466, DOI 10.1007/s12035-012-8327-0

Youm YH, 2015, NAT MED, V21, P263, DOI 10.1038/nm.3804

Yudkoff M, 2001, J NEUROSCI RES, V66, P272, DOI 10.1002/jnr.1221

Yuen AWC, 2018, EPILEPSY BEHAV, V78, P57, DOI 10.1016/j.yebeh.2017.10.010

Zandi-Nejad K, 2013, FASEB J, V27, P4366, DOI 10.1096/fj.12-223933

Zhang YF, 2013, J CEREBR BLOOD F MET, V33, P1307, DOI 10.1038/jcbfm.2013.87

Zhang YJ, 2018, EPILEPSY RES, V145, P163, DOI 10.1016/j.eplepsyres.2018.06.015

Zhao S, 2021, BRAIN RES BULL, V169, P1, DOI 10.1016/j.brainresbull.2021.01.003

Zhou FM, 2011, NEUROSCIENCE, V198, P69, DOI 10.1016/j.neuroscience.2011.07.061

Zhou WH, 2007, NUTR METAB, V4, DOI 10.1186/1743-7075-4-5

Zilberter Yuri, 2020, eNeuro, V7, DOI 10.1523/ENEURO.0303-20.2020

Zou XH, 2020, CELL PROLIFERAT, V53, DOI 10.1111/cpr.12806

NR 240

TC 34

Z9 34

U1 8

U2 22

PU MDPI

PI BASEL

PA ST ALBAN-ANLAGE 66, CH-4052 BASEL, SWITZERLAND

EI 2072-6643

J9 NUTRIENTS

JI Nutrients

PD MAY

PY 2022

VL 14

IS 9

AR 1952

DI 10.3390/nu14091952

PG 31

WC Nutrition & Dietetics

WE Science Citation Index Expanded (SCI-EXPANDED)

SC Nutrition & Dietetics

GA 1E4MU

UT WOS:000794465600001

PM 35565918

OA gold, Green Published

DA 2024-08-12

ER

PT J

AU Contaldi, E

Magistrelli, L

Comi, C

AF Contaldi, Elena

Magistrelli, Luca

Comi, Cristoforo

TI T Lymphocytes in Parkinson's Disease

SO JOURNAL OF PARKINSONS DISEASE

LA English

DT Review

DE Parkinson's disease; T cells; CD4+T cells; CD8+T cells;

neuroinflammation; peripheral immunity

ID CELL-DEVELOPMENT; ALPHA-SYNUCLEIN; MOUSE MODEL; NEURODEGENERATION;

SUBSETS; NEUROINFLAMMATION; INFILTRATION; DYSFUNCTION; BLOOD

AB T cells are key mediators of both humoral and cellular adaptive immune responses, and their role in Parkinson's disease (PD) is being increasingly recognized. Several lines of evidence have highlighted how T cells are involved in both the central nervous system and the periphery, leading to a profound imbalance in the immune network in PD patients. This review discusses the involvement of T cells in both preclinical and clinical studies, their importance as feasible biomarkers of motor and non-motor progression of the disease, and recent therapeutic strategies addressing the modulation of T cell response.

C1 [Contaldi, Elena; Magistrelli, Luca] Univ Piemonte Orientale, Univ Hosp Maggiore della Carita, Movement Disorders Ctr, Dept Translat Med, Novara, Italy.

[Contaldi, Elena] Univ Piemonte Orientale, Program Med Sci & Biotechnol, Novara, Italy.

[Magistrelli, Luca] Univ Insubria, Program Clin & Expt Med & Med Humanities, Varese, Italy.

[Comi, Cristoforo] Univ Piemonte Orientale, S Andrea Hosp, Dept Translat Med, Neurol Unit, Vercelli, Italy.

C3 University of Eastern Piedmont Amedeo Avogadro; University of Eastern

Piedmont Amedeo Avogadro; University of Insubria; University of Eastern

Piedmont Amedeo Avogadro

RP Comi, C (corresponding author), S Andrea Hosp, Neurol Unit, Corso Mario Abbiate 21, I-13100 Vercelli, Italy.

EM cristoforo.comi@med.uniupo.it

RI Contaldi, Elena/IQR-6622-2023; Comi, Cristoforo/A-7495-2014;

MAGISTRELLI, LUCA/AEX-0939-2022

OI Contaldi, Elena/0000-0002-0218-5251; Comi,

Cristoforo/0000-0002-6862-9468; MAGISTRELLI, LUCA/0000-0002-9546-8567

FU Fondazione CARIPLO [2011-0504]; AGING PROJECTDepartment of

Excellence-Universit`a del Piemonte Orientale

FX This research was supported by Fondazione CARIPLO

http://www.fondazionecariplo.it (Project 2011-0504), and by the AGING

PROJECTDepartment of Excellence-Universit`a del Piemonte Orientale. This

paper is part of the PhD program of Clinical and Experimental Medicine

and Medical Humanities (University of Insubria) for L.M. and Medical

Science and Biotechnology for E.C. These PhD programs and therefore this

paper constitute an important part of the project Recruiting and

training Physician-Scientists to empower translational research, funded

by Fondazione Cariplo.

CR Alvarez-Luquín DD, 2021, J TRANSL MED, V19, DOI 10.1186/s12967-021-03055-2

Alvarez-Luquín DD, 2019, J NEUROINFLAMM, V16, DOI 10.1186/s12974-019-1606-1

Arlehamn CSL, 2020, NAT COMMUN, V11, DOI 10.1038/s41467-020-15626-w

Baba Y, 2005, PARKINSONISM RELAT D, V11, P493, DOI 10.1016/j.parkreldis.2005.07.005

Bhatia D, 2021, J NEUROINFLAMM, V18, DOI 10.1186/s12974-021-02296-8

Boldrini VD, 2019, SCAND J IMMUNOL, V90, DOI 10.1111/sji.12793

Brochard V, 2009, J CLIN INVEST, V119, P182, DOI 10.1172/JCI36470

Broen JCA, 2020, NAT REV RHEUMATOL, V16, P167, DOI 10.1038/s41584-020-0374-8

Cebrián C, 2014, NAT COMMUN, V5, DOI 10.1038/ncomms4633

Cen L, 2017, NEUROSCI BULL, V33, P493, DOI 10.1007/s12264-017-0163-9

Chen QQ, 2019, FRONT MOL NEUROSCI, V12, DOI 10.3389/fnmol.2019.00218

Chen SY, 2017, NEUROSCI LETT, V648, P21, DOI 10.1016/j.neulet.2017.03.045

Chen XP, 2021, FRONT AGING NEUROSCI, V13, DOI 10.3389/fnagi.2021.617370

Chen YH, 2015, MOL MED REP, V12, P6105, DOI 10.3892/mmr.2015.4136

Chen YH, 2015, INT J CLIN EXP PATHO, V8, P2495

Churchill MJ, 2019, NEUROSCIENCE, V414, P8, DOI 10.1016/j.neuroscience.2019.06.006

Contaldi E, 2021, J PARKINSON DIS, V11, P507, DOI 10.3233/JPD-202417

de Azevedo MSP, 2013, J APPL MICROBIOL, V115, P319, DOI 10.1111/jam.12174

De Francesco E, 2021, MOVEMENT DISORD, V36, P225, DOI 10.1002/mds.28137

Duffy SS, 2018, J NEUROSCI RES, V96, P951, DOI 10.1002/jnr.24073

Ellis GI, 2013, EUR J IMMUNOL, V43, P3355, DOI 10.1002/eji.201343571

Ettle B, 2016, J NEUROINFLAMM, V13, DOI 10.1186/s12974-016-0694-4

Fang P, 2018, J HEMATOL ONCOL, V11, DOI 10.1186/s13045-018-0637-x

Galiano-Landeira J, 2020, BRAIN, V143, P3717, DOI 10.1093/brain/awaa269

Gendelman HE, 2017, NPJ PARKINSONS DIS, V3, DOI 10.1038/s41531-017-0013-5

Greenland JC, 2020, BMJ OPEN, V10, DOI 10.1136/bmjopen-2020-040527

Grossman WJ, 2004, IMMUNITY, V21, P589, DOI 10.1016/j.immuni.2004.09.002

Hamza TH, 2010, NAT GENET, V42, P781, DOI 10.1038/ng.642

Hill-Burns EM, 2017, MOVEMENT DISORD, V32, P739, DOI 10.1002/mds.26942

Jiang S, 2017, NEUROL SCI, V38, P1373, DOI 10.1007/s10072-017-2988-4

Kishimoto Y, 2019, NEUROMOL MED, V21, P239, DOI 10.1007/s12017-019-08539-5

Kortekaas R, 2005, ANN NEUROL, V57, P176, DOI 10.1002/ana.20369

Kosloski LM, 2013, J NEUROIMMUNOL, V265, P1, DOI 10.1016/j.jneuroim.2013.10.009

Kouli A, 2021, J NEUROINFLAMM, V18, DOI 10.1186/s12974-021-02287-9

Kustrimovic N, 2018, J NEUROINFLAMM, V15, DOI 10.1186/s12974-018-1248-8

Kustrimovic N, 2016, SCI REP-UK, V6, DOI 10.1038/srep33738

Larsen JM, 2017, IMMUNOLOGY, V151, P363, DOI 10.1111/imm.12760

Lee HG, 2020, EXP MOL MED, V52, P1255, DOI 10.1038/s12276-020-00486-7

Li WJ, 2021, J INT MED RES, V49, DOI 10.1177/0300060521998471

Li Z, 2021, FRONT IMMUNOL, V12, DOI 10.3389/fimmu.2021.632482

Liu Z, 2021, BRAIN BEHAV IMMUN, V98, P110, DOI 10.1016/j.bbi.2021.08.220

Liu Z, 2017, MOL NEUROBIOL, V54, P7762, DOI 10.1007/s12035-016-0249-9

Maasz G, 2017, DIS MODEL MECH, V10, P127, DOI 10.1242/dmm.027185

Copas ANM, 2021, FRONT NEUROL, V12, DOI 10.3389/fneur.2021.666737

Magistrelli L, 2020, BRAIN BEHAV IMMUN, V89, P668, DOI 10.1016/j.bbi.2020.07.005

Matheoud D, 2019, NATURE, V571, P565, DOI 10.1038/s41586-019-1405-y

Mosley RL, 2019, FRONT CELL NEUROSCI, V13, DOI 10.3389/fncel.2019.00421

Nalls MA, 2014, NAT GENET, V46, P989, DOI 10.1038/ng.3043

Nasrolahi A, 2019, REV NEUROSCIENCE, V30, P709, DOI 10.1515/revneuro-2018-0105

Olson KE, 2021, EBIOMEDICINE, V67, DOI 10.1016/j.ebiom.2021.103380

Park J, 2017, J LEUKOCYTE BIOL, V102, P1093, DOI 10.1189/jlb.1A0417-147RR

Peter I, 2018, JAMA NEUROL, V75, P939, DOI 10.1001/jamaneurol.2018.0605

Prots I, 2019, EXPERT OPIN THER TAR, V23, P309, DOI 10.1080/14728222.2019.1590336

Puschmann A, 2011, PARKINSONISM RELAT D, V17, P376, DOI 10.1016/j.parkreldis.2011.03.008

Reynolds AD, 2007, J LEUKOCYTE BIOL, V82, P1083, DOI 10.1189/jlb.0507296

Reynolds AD, 2010, J IMMUNOL, V184, P2261, DOI 10.4049/jimmunol.0901852

Rocha NP, 2018, MOL NEUROBIOL, V55, P1488, DOI 10.1007/s12035-017-0404-y

Sampson TR, 2016, CELL, V167, P1469, DOI 10.1016/j.cell.2016.11.018

Sanchez-Guajardo V, 2010, PLOS ONE, V5, DOI 10.1371/journal.pone.0008784

Saunders JAH, 2012, J NEUROIMMUNE PHARM, V7, P927, DOI 10.1007/s11481-012-9402-z

Schröder JB, 2018, FRONT NEUROL, V9, DOI 10.3389/fneur.2018.01081

Shameli A, 2016, IMMUNOBIOLOGY, V221, P333, DOI 10.1016/j.imbio.2015.10.002

Sommer A, 2018, CELL STEM CELL, V23, P123, DOI 10.1016/j.stem.2018.06.015

Sommer A, 2016, J NEUROINFLAMM, V13, DOI 10.1186/s12974-016-0632-5

Sulzer D, 2017, NATURE, V546, P656, DOI 10.1038/nature22815

Sun CC, 2019, ANN TRANSL MED, V7, DOI 10.21037/atm.2019.10.105

Thome AD, 2021, NPJ PARKINSONS DIS, V7, DOI 10.1038/s41531-021-00188-5

Thonhoff JR, 2018, NEUROL-NEUROIMMUNOL, V5, DOI 10.1212/NXI.0000000000000465

Trudler D, 2020, J NEUROCHEM, V152, P61, DOI 10.1111/jnc.14871

Walker JA, 2018, NAT REV IMMUNOL, V18, P121, DOI 10.1038/nri.2017.118

Wan QY, 2020, GUT, V69, P193, DOI 10.1136/gutjnl-2018-317103

Wang PP, 2021, CELL DISCOV, V7, DOI 10.1038/s41421-021-00280-3

Williams GP, 2021, BRAIN, V144, P2047, DOI 10.1093/brain/awab103

Yan ZQ, 2021, NEUROL-NEUROIMMUNOL, V8, DOI 10.1212/NXI.0000000000001036

Zhu F, 2019, DIGEST LIVER DIS, V51, P38, DOI 10.1016/j.dld.2018.09.017

NR 75

TC 17

Z9 17

U1 3

U2 9

PU IOS PRESS

PI AMSTERDAM

PA NIEUWE HEMWEG 6B, 1013 BG AMSTERDAM, NETHERLANDS

SN 1877-7171

EI 1877-718X

J9 J PARKINSON DIS

JI J. Parkinsons Dis.

PY 2022

VL 12

SU 1

BP S65

EP S74

DI 10.3233/JPD-223152

PG 10

WC Neurosciences

WE Science Citation Index Expanded (SCI-EXPANDED)

SC Neurosciences & Neurology

GA 5E2PZ

UT WOS:000865470900007

PM 35253782

OA hybrid, Green Published

DA 2024-08-12

ER

PT J

AU Grotemeyer, A

McFleder, RL

Wu, JJ

Wischhusen, J

Ip, CW

AF Grotemeyer, Alexander

McFleder, Rhonda Leah

Wu, Jingjing

Wischhusen, Joerg

Ip, Chi Wang

TI Neuroinflammation in Parkinson's Disease - Putative Pathomechanisms and

Targets for Disease-Modification

SO FRONTIERS IN IMMUNOLOGY

LA English

DT Review

DE Parkinson's disease; neuroinflammation; T cells; microglia;

neurodegeneration; animal models; inflammatory cascades

ID MPTP MOUSE MODEL; NONSTEROIDAL ANTIINFLAMMATORY DRUGS; NF-KAPPA-B;

PROTECTS DOPAMINERGIC-NEURONS; ADAPTIVE IMMUNE-RESPONSE;

RENIN-ANGIOTENSIN SYSTEM; CENTRAL-NERVOUS-SYSTEM; GROWTH-FACTOR-ALPHA;

REGULATORY T-CELLS; OXIDATIVE STRESS

AB Parkinson's disease (PD) is a progressive and debilitating chronic disease that affects more than six million people worldwide, with rising prevalence. The hallmarks of PD are motor deficits, the spreading of pathological alpha-synuclein clusters in the central nervous system, and neuroinflammatory processes. PD is treated symptomatically, as no causally-acting drug or procedure has been successfully established for clinical use. Various pathways contributing to dopaminergic neuron loss in PD have been investigated and described to interact with the innate and adaptive immune system. We discuss the possible contribution of interconnected pathways related to the immune response, focusing on the pathophysiology and neurodegeneration of PD. In addition, we provide an overview of clinical trials targeting neuroinflammation in PD.

C1 [Grotemeyer, Alexander; McFleder, Rhonda Leah; Wu, Jingjing; Ip, Chi Wang] Univ Hosp Wurzburg, Dept Neurol, Wurzburg, Germany.

[Wischhusen, Joerg] Univ Hosp Wurzburg, Dept Obstet & Gynecol, Sect Expt Tumor Immunol, Wurzburg, Germany.

C3 University of Wurzburg; University of Wurzburg

RP Ip, CW (corresponding author), Univ Hosp Wurzburg, Dept Neurol, Wurzburg, Germany.

RI Ip, Chi Wang/HCH-3822-2022

OI Ip, Chi Wang/0000-0003-0484-385X; Grotemeyer,

Alexander/0000-0002-9185-257X; McFleder, Rhonda/0000-0001-9986-4201

FU Interdisciplinary Center for Clinical Research (IZKF) at the University

of Wuerzburg [A-303, A-421, N-362, Z-2/79]; Deutsche Stiftung

Neurologie; ParkinsonFonds Germany; University Research Funds of the

State of Bavaria; Deutsche Forschungsgemeinschaft (DFG, German Research

Foundation) [424778381-TRR 295]; VERUM Foundation; Aeterna Zentaris; CRC

[TRR 295]; Alexander von Humboldt-Stiftung

FX CWI is supported by the Interdisciplinary Center for Clinical Research

(IZKF) at the University of Wuerzburg (A-303 to CWI, A-421 to CWI and

JWi, N-362 to CWI), by the Deutsche Stiftung Neurologie and the

ParkinsonFonds Germany and by the University Research Funds of the State

of Bavaria. Moreover, CWI is supported by the Deutsche

Forschungsgemeinschaft (DFG, German Research Foundation) Project-ID

424778381-TRR 295 (A06) and the VERUM Foundation. CWI and JWi are

supported by Aeterna Zentaris. AG is supported by Interdisciplinary

Center for Clinical Research (IZKF) at the University of Wuerzburg

(Z-2/79) and is a Gerok-Position holder funded by the CRC TRR 295

'Retune'. RM is funded by the Alexander von Humboldt-Stiftung.

CR Ahn JJ, 2021, CELLS-BASEL, V10, DOI 10.3390/cells10071605

Alam MM, 2022, CELL REP, V38, DOI 10.1016/j.celrep.2021.110090

Ambrosi G, 2017, NEUROTOX RES, V32, P71, DOI 10.1007/s12640-017-9712-2

Andersen JK, 2004, NAT MED, V10, pS18, DOI 10.1038/nrn1434

Andreasson K, 2010, PROSTAG OTH LIPID M, V91, P104, DOI 10.1016/j.prostaglandins.2009.04.003

Ansel H., 2019, J NUCL MED, V60, P488

Arlehamn CSL, 2020, NAT COMMUN, V11, DOI 10.1038/s41467-020-15626-w

Armstrong MJ, 2020, JAMA-J AM MED ASSOC, V323, P548, DOI 10.1001/jama.2019.22360

Asanuma M, 2001, J NEUROCHEM, V76, P1895, DOI 10.1046/j.1471-4159.2001.00205.x

Athauda D, 2016, PROG NEUROBIOL, V145, P98, DOI 10.1016/j.pneurobio.2016.10.001

Aubin N, 1998, J NEUROCHEM, V71, P1635, DOI 10.1046/j.1471-4159.1998.71041635.x

Aviles-Olmos I, 2013, BRAIN, V136, P374, DOI 10.1093/brain/aws009

Baba Y, 2005, PARKINSONISM RELAT D, V11, P493, DOI 10.1016/j.parkreldis.2005.07.005

Bandookwala M, 2020, INT J NEUROSCI, V130, P1047, DOI 10.1080/00207454.2020.1713776

Bansal V, 2009, J NEUROTRAUM, V26, P1353, DOI [10.1089/neu.2008.0858, 10.1089/neu.2008-0858]

Bas J, 2001, J NEUROIMMUNOL, V113, P146, DOI 10.1016/S0165-5728(00)00422-7

Bekkelund AK., 2018, HINDGUT MICROBIOME E

Belarbi K, 2017, MOL NEURODEGENER, V12, DOI 10.1186/s13024-017-0225-5

Bell L, 2020, FRONT IMMUNOL, V10, DOI 10.3389/fimmu.2019.03090

Bennett JL, 2009, ANN NEUROL, V66, P617, DOI 10.1002/ana.21802

Benoist CC, 2011, J PHARMACOL EXP THER, V339, P35, DOI 10.1124/jpet.111.182220

Beraud D, 2011, FRONT NEUROSCI-SWITZ, V5, DOI 10.3389/fnins.2011.00080

Berg D, 2015, MOVEMENT DISORD, V30, P1600, DOI 10.1002/mds.26431

Bertilsson G, 2008, J NEUROSCI RES, V86, P326, DOI 10.1002/jnr.21483

Bido S, 2021, NAT COMMUN, V12, DOI 10.1038/s41467-021-26519-x

Bienenstock J, 2015, NUTR REV, V73, P28, DOI 10.1093/nutrit/nuv019

BIRKMAYER W, 1961, Wien Klin Wochenschr, V73, P787

BJARNASON I, 1986, GUT, V27, P1292, DOI 10.1136/gut.27.11.1292

Bonifati V, 2003, SCIENCE, V299, P256, DOI 10.1126/science.1077209

Bower JH, 2006, NEUROLOGY, V67, P494, DOI 10.1212/01.wnl.0000227906.99570.cc

Braak H, 2000, J NEUROL, V247, P3

Braak H, 2006, NEUROSCI LETT, V396, P67, DOI 10.1016/j.neulet.2005.11.012

Braak H, 2004, CELL TISSUE RES, V318, P121, DOI 10.1007/s00441-004-0956-9

Braak H, 2003, NEUROBIOL AGING, V24, P197, DOI 10.1016/S0197-4580(02)00065-9

Braak H, 2007, ACTA NEUROPATHOL, V113, P421, DOI 10.1007/s00401-007-0193-x

Braczynski AK, 2017, J NEUROCHEM, V143, P467, DOI 10.1111/jnc.14207

Brochard V, 2009, J CLIN INVEST, V119, P182, DOI 10.1172/JCI36470

Brundin P, 2020, TRENDS NEUROSCI, V43, P931, DOI 10.1016/j.tins.2020.10.009

Camacho-Soto A, 2018, PARKINSONISM RELAT D, V50, P23, DOI 10.1016/j.parkreldis.2018.02.008

Cao SW, 2010, MOL NEURODEGENER, V5, DOI 10.1186/1750-1326-5-42

Carrasco E, 2008, NEUROSCI LETT, V441, P44, DOI 10.1016/j.neulet.2008.05.111

Casper D, 2000, NEUROSCI LETT, V289, P201, DOI 10.1016/S0304-3940(00)01294-5

Caughey GE, 2001, J IMMUNOL, V167, P2831, DOI 10.4049/jimmunol.167.5.2831

Chabrashvili T, 2003, AM J PHYSIOL-REG I, V285, pR117, DOI 10.1152/ajpregu.00476.2002

Chamberlain JL, 2021, J NEUROL, V268, P1643, DOI 10.1007/s00415-019-09498-4

Chandra G, 2017, J IMMUNOL, V198, P4312, DOI 10.4049/jimmunol.1700149

Chandra G, 2016, J BIOL CHEM, V291, P15267, DOI 10.1074/jbc.M116.714824

Chapman LM, 2012, J IMMUNOL, V189, P916, DOI 10.4049/jimmunol.1200580

Chartier-Harlin MC, 2004, LANCET, V364, P1167, DOI 10.1016/S0140-6736(04)17103-1

Chen HL, 2005, ANN NEUROL, V58, P963, DOI 10.1002/ana.20682

Chen SC, 2018, SCI REP-UK, V8, DOI 10.1038/s41598-018-28449-z

Chen SY, 2017, NEUROSCI LETT, V648, P21, DOI 10.1016/j.neulet.2017.03.045

Chen X, 2018, FRONT IMMUNOL, V9, DOI 10.3389/fimmu.2018.02122

Chen YH, 2015, MOL MED REP, V12, P6105, DOI 10.3892/mmr.2015.4136

Chen YH, 2015, INT J CLIN EXP PATHO, V8, P2495

Cheng JJ, 2018, ASN NEURO, V10, DOI 10.1177/1759091418812587

Cherry JD, 2016, ACTA NEUROPATHOL COM, V4, DOI 10.1186/s40478-016-0382-8

Chinta SJ, 2006, FREE RADICAL BIO MED, V41, P1442, DOI 10.1016/j.freeradbiomed.2006.08.002

clinicaltrials, 2019, NIAC PARK DIS

clinicaltrials, PHYS EFF NUTR SUPP P

Coles LD, 2018, J CLIN PHARMACOL, V58, P158, DOI 10.1002/jcph.1008

Colonna M, 2017, ANNU REV IMMUNOL, V35, P441, DOI 10.1146/annurev-immunol-051116-052358

Czech H, 2014, J HIST NEUROSCI, V23, P160, DOI 10.1080/0964704X.2013.865427

Daeron M, 1997, ANNU REV IMMUNOL, V15, P203, DOI 10.1146/annurev.immunol.15.1.203

Dalmay F, 2001, HYPERTENSION, V14, p56A, DOI DOI 10.1016/S0895-7061(01)01592-8

DAVIES NM, 1994, PHARM RES-DORDR, V11, P1652, DOI 10.1023/A:1018978308752

de Graaf MT, 2011, CYTOM PART B-CLIN CY, V80B, P43, DOI 10.1002/cyto.b.20542

Dhanwani R, 2022, NPJ PARKINSONS DIS, V8, DOI 10.1038/s41531-022-00282-2

Dias V, 2013, J PARKINSON DIS, V3, P461, DOI 10.3233/JPD-130230

Dijkstra AA, 2014, MOVEMENT DISORD, V29, P1244, DOI 10.1002/mds.25952

Dinarello CA, 2018, IMMUNOL REV, V281, P8, DOI 10.1111/imr.12621

Dunn L, 2014, NEUROBIOL AGING, V35, P1111, DOI 10.1016/j.neurobiolaging.2013.11.001

During MJ, 2003, NAT MED, V9, P1173, DOI 10.1038/nm919

Earls RH, 2019, J NEUROINFLAMM, V16, DOI 10.1186/s12974-019-1636-8

El-Arif G, 2021, MOLECULES, V26, DOI 10.3390/molecules26226945

Eyolfson E, 2020, J NEUROINFLAMM, V17, DOI 10.1186/s12974-020-01994-z

Fan J, 2019, PARKINSONISM RELAT D, V63, P149, DOI 10.1016/j.parkreldis.2019.02.013

Fan Z, 2020, J NEUROINFLAMM, V17, DOI 10.1186/s12974-019-1670-6

Fang L, 2020, LANCET RESP MED, V8, pE21, DOI 10.1016/S2213-2600(20)30116-8

FAZZINI E, 1992, MOVEMENT DISORD, V7, P153, DOI 10.1002/mds.870070210

Feng ZH, 2002, NEUROSCI LETT, V329, P354, DOI 10.1016/S0304-3940(02)00704-8

Ferreira SA, 2018, FRONT CELL NEUROSCI, V12, DOI 10.3389/fncel.2018.00247

Firbank MJ, 2017, J NEUROL NEUROSUR PS, V88, P310, DOI 10.1136/jnnp-2016-313918

Foltynie T, 2018, J PARKINSON DIS, V8, pS115, DOI 10.3233/JPD-181481

Forsyth CB, 2011, PLOS ONE, V6, DOI 10.1371/journal.pone.0028032

GALIANOLANDEIRA J, 2020, BRAIN, V143, P3717, DOI DOI 10.1093/BRAIN/AWAA269

Gao XA, 2011, NEUROLOGY, V76, P863, DOI 10.1212/WNL.0b013e31820f2d79

Garretti F, 2019, FRONT IMMUNOL, V10, DOI 10.3389/fimmu.2019.00303

Ghosh A, 2007, P NATL ACAD SCI USA, V104, P18754, DOI 10.1073/pnas.0704908104

Giasson BI, 2000, SCIENCE, V290, P985, DOI 10.1126/science.290.5493.985

Giri B, 2019, INT J MOL SCI, V20, DOI 10.3390/ijms20184559

Goedert M, 2018, NAT REV NEUROL, V14, P57, DOI 10.1038/nrneurol.2017.165

Goetz CG, 2011, CSH PERSPECT MED, V1, DOI 10.1101/cshperspect.a008862

Grammatopoulos TN, 2007, MOL NEURODEGENER, V2, DOI 10.1186/1750-1326-2-1

Groemping Y, 2005, BIOCHEM J, V386, P401, DOI 10.1042/BJ20041835

Guo M, 2017, FREE RADICAL BIO MED, V112, P16, DOI 10.1016/j.freeradbiomed.2017.10.369

Han M, 2012, PLOS ONE, V7, DOI 10.1371/journal.pone.0032383

Hancock DB, 2007, ARCH NEUROL-CHICAGO, V64, P576, DOI 10.1001/archneur.64.4.576

Haque ME, 2020, MOVEMENT DISORD, V35, P20, DOI 10.1002/mds.27874

Hardy TA, 2016, LANCET NEUROL, V15, P967, DOI 10.1016/S1474-4422(16)30043-6

Harms AS, 2017, ACTA NEUROPATHOL COM, V5, DOI 10.1186/s40478-017-0494-9

Harms AS, 2013, J NEUROSCI, V33, P9592, DOI 10.1523/JNEUROSCI.5610-12.2013

Hartung HP, 2020, LANCET NEUROL, V19, P280, DOI 10.1016/S1474-4422(20)30062-4

Heng Y, 2016, TOXICOL LETT, V243, P7, DOI 10.1016/j.toxlet.2015.12.005

Heuer A, 2013, EUR J NEUROSCI, V37, P294, DOI 10.1111/ejn.12036

Hijaz BA, 2020, MOL NEURODEGENER, V15, DOI 10.1186/s13024-020-00368-6

Hirsch EC, 2021, MOVEMENT DISORD, V36, P16, DOI 10.1002/mds.28075

HOEHN MM, 1967, NEUROLOGY, V17, P427, DOI 10.1212/WNL.17.5.427

HOFFMAN PM, 1981, ANN NEUROL, V10, P193, DOI 10.1002/ana.410100210

Hong S, 2018, IMMUNITY, V49, P695, DOI 10.1016/j.immuni.2018.08.012

Hopfner F, 2020, LANCET NEUROL, V19, P247, DOI 10.1016/S1474-4422(19)30400-4

Huang BH, 2018, NEUROSCIENCE, V379, P302, DOI 10.1016/j.neuroscience.2018.03.026

Hunot S, 1996, NEUROSCIENCE, V72, P355, DOI 10.1016/0306-4522(95)00578-1

Ip CW, 2017, ACTA NEUROPATHOL COM, V5, DOI 10.1186/s40478-017-0416-x

Ip CW, 2015, J NEURAL TRANSM, V122, P1633, DOI 10.1007/s00702-015-1444-y

Jeong SM, 2019, MOVEMENT DISORD, V34, P1014, DOI 10.1002/mds.27681

Jia HQ, 2008, J NEUROSCI RES, V86, P2083, DOI 10.1002/jnr.21650

Jiang S, 2017, NEUROL SCI, V38, P1373, DOI 10.1007/s10072-017-2988-4

Jin JJ, 2007, J NEUROINFLAMM, V4, DOI 10.1186/1742-2094-4-2

Joglar B, 2009, J NEUROCHEM, V109, P656, DOI 10.1111/j.1471-4159.2009.05999.x

Jowaed A, 2010, J NEUROSCI, V30, P6355, DOI 10.1523/JNEUROSCI.6119-09.2010

Kalia LV, 2015, LANCET, V386, P896, DOI 10.1016/S0140-6736(14)61393-3

KAO KJ, 1987, BLOOD, V70, P282

KAO KJ, 1986, BLOOD, V68, P627

Karikari AA, 2022, BRAIN BEHAV IMMUN, V101, P194, DOI 10.1016/j.bbi.2022.01.007

Katzenberger RJ, 2015, ELIFE, V4, DOI 10.7554/eLife.04790

Killinger BA, 2019, J NEUROCHEM, V150, P605, DOI 10.1111/jnc.14779

Kim S, 2019, NEURON, V103, P627, DOI 10.1016/j.neuron.2019.05.035

Kitada T, 1998, NATURE, V392, P605, DOI 10.1038/33416

Koprich JB, 2010, MOL NEURODEGENER, V5, DOI 10.1186/1750-1326-5-43

Kramar EA, 1997, REGUL PEPTIDES, V68, P131, DOI 10.1016/S0167-0115(96)02116-7

Kuan YC, 2017, PROG NEURO-PSYCHOPH, V79, P77, DOI 10.1016/j.pnpbp.2017.06.002

Kuhbandner K, 2020, J MOL NEUROSCI, V70, P1038, DOI 10.1007/s12031-020-01498-8

Kupsch A, 2014, J NEURAL TRANSM, V121, P1245, DOI 10.1007/s00702-014-1185-3

Kustrimovic N, 2018, J NEUROINFLAMM, V15, DOI 10.1186/s12974-018-1248-8

Labzin LI, 2018, ANNU REV MED, V69, P437, DOI 10.1146/annurev-med-050715-104343

Laghlam D, 2021, CELLS-BASEL, V10, DOI 10.3390/cells10071767

LANGSTON JW, 1985, TRENDS NEUROSCI, V8, P79

Lee CH, 2018, MOL NEUROBIOL, V55, P3007, DOI 10.1007/s12035-017-0550-2

Lee E, 2019, CELL DEATH DIFFER, V26, P213, DOI 10.1038/s41418-018-0124-5

Li R, 2022, NEUROL-NEUROIMMUNOL, V9, DOI 10.1212/NXI.0000000000001125

Li XY, 2019, ACTA NEUROPATHOL, V137, P825, DOI 10.1007/s00401-019-01974-5

Lingappan Krithika, 2018, Current Opinion in Toxicology, V7, P81, DOI 10.1016/j.cotox.2017.11.002

Liu JQ, 2020, ACTA PHARMACOL SIN, V41, P523, DOI 10.1038/s41401-020-0358-x

Liu WZ, 2015, EUR J PHARMACOL, V765, P284, DOI 10.1016/j.ejphar.2015.08.038

Liu Z, 2017, MOL NEUROBIOL, V54, P7762, DOI 10.1007/s12035-016-0249-9

Luchtman DW, 2009, PHYSIOL BEHAV, V98, P130, DOI 10.1016/j.physbeh.2009.04.021

Magistrelli L, 2020, J NEUROIMMUNE PHARM, V15, P74, DOI 10.1007/s11481-018-09831-0

Makhoul K, 2021, J NEUROL SCI, V422, DOI 10.1016/j.jns.2021.117331

Mao ZJ, 2017, NEUROCHEM RES, V42, P1104, DOI 10.1007/s11064-017-2185-0

Marques A, 2018, PARKINSONISM RELAT D, V55, P122, DOI 10.1016/j.parkreldis.2018.05.026

Marques CP, 2006, VIRUS RES, V121, P1, DOI 10.1016/j.virusres.2006.03.009

Martin HL, 2016, GLIA, V64, P386, DOI 10.1002/glia.22935

Matheoud D, 2019, NATURE, V571, P565, DOI 10.1038/s41586-019-1405-y

Matheoud D, 2016, CELL, V166, P314, DOI 10.1016/j.cell.2016.05.039

McGeer PL, 2015, EXPERT OPIN THER TAR, V19, P497, DOI 10.1517/14728222.2014.988707

McGeer PL, 2004, ANN NY ACAD SCI, V1035, P104, DOI 10.1196/annals.1332.007

MCGEER PL, 1988, ANN NEUROL, V24, P574, DOI 10.1002/ana.410240415

MCGEER PL, 1993, GLIA, V7, P84, DOI 10.1002/glia.440070114

MCGEER PL, 1988, NEUROLOGY, V38, P1285, DOI 10.1212/WNL.38.8.1285

Michell-Robinson MA, 2015, BRAIN, V138, P1138, DOI 10.1093/brain/awv066

Mischley LK, 2017, J PARKINSON DIS, V7, P289, DOI 10.3233/JPD-161040

Mogi M, 1996, NEUROSCI LETT, V211, P13, DOI 10.1016/0304-3940(96)12706-3

MOGI M, 1994, NEUROSCI LETT, V180, P147, DOI 10.1016/0304-3940(94)90508-8

MOGI M, 1994, NEUROSCI LETT, V165, P208, DOI 10.1016/0304-3940(94)90746-3

MONCADA S, 1982, ARTERIOSCLEROSIS, V2, P193, DOI 10.1161/01.ATV.2.3.193

Muñoz A, 2006, NEUROPHARMACOLOGY, V51, P112, DOI 10.1016/j.neuropharm.2006.03.004

National Library of Medicine (U.S.), 2018, GUT MICR PARK DIS

NAVERI L, 1994, J CEREBR BLOOD F MET, V14, P1096

Netland J, 2008, J VIROL, V82, P7264, DOI 10.1128/JVI.00737-08

Noelker C, 2013, SCI REP-UK, V3, DOI 10.1038/srep01393

Olanow CW, 2019, MOVEMENT DISORD, V34, P812, DOI 10.1002/mds.27690

Olszak T, 2012, SCIENCE, V336, P489, DOI 10.1126/science.1219328

Orr CF, 2005, BRAIN, V128, P2665, DOI 10.1093/brain/awh625

Orsini F, 2014, FRONT CELL NEUROSCI, V8, DOI 10.3389/fncel.2014.00380

Ouchi Y, 2005, ANN NEUROL, V57, P168, DOI 10.1002/ana.20338

Ouchi Y, 2009, PARKINSONISM RELAT D, V15, pS200, DOI 10.1016/S1353-8020(09)70814-4

Pagano G, 2018, NEUROLOGY, V90, pE1654, DOI 10.1212/WNL.0000000000005475

Panicker N, 2019, J EXP MED, V216, P1411, DOI 10.1084/jem.20182191

Parga JA, 2018, FREE RADICAL BIO MED, V129, P394, DOI 10.1016/j.freeradbiomed.2018.10.409

Parillaud VR, 2017, J NEUROINFLAMM, V14, DOI 10.1186/s12974-017-0830-9

Parkinson J, 2002, J NEUROPSYCH CLIN N, V14, P223, DOI 10.1176/appi.neuropsych.14.2.223

Ramos JMP, 2019, FRONT IMMUNOL, V10, DOI 10.3389/fimmu.2019.00080

Perez-Lloret S, 2017, EXPERT OPIN INV DRUG, V26, P1163, DOI 10.1080/13543784.2017.1371133

Peter I, 2018, JAMA NEUROL, V75, P939, DOI 10.1001/jamaneurol.2018.0605

Poly TN, 2017, NEUROEPIDEMIOLOGY, V49, P142, DOI 10.1159/000480401

Pouchieu C, 2018, INT J EPIDEMIOL, V47, P299, DOI 10.1093/ije/dyx225

Prinz M, 2017, NAT NEUROSCI, V20, P136, DOI 10.1038/nn.4475

Qiao WL, 2015, CARDIOLOGY, V131, P97, DOI 10.1159/000375362

Qin XY, 2016, JAMA NEUROL, V73, P1316, DOI 10.1001/jamaneurol.2016.2742

Qiu FF, 2017, ONCOTARGET, V8, P268, DOI 10.18632/oncotarget.13613

Quigley EMM, 2017, CURR NEUROL NEUROSCI, V17, DOI 10.1007/s11910-017-0802-6

Qureshi ST, 1999, J EXP MED, V189, P615, DOI 10.1084/jem.189.4.615

Racette BA, 2018, ANN CLIN TRANSL NEUR, V5, P870, DOI 10.1002/acn3.580

Raj T, 2014, SCIENCE, V344, P519, DOI 10.1126/science.1249547

Ransohoff RM, 2016, SCIENCE, V353, P777, DOI 10.1126/science.aag2590

Ray A, 2012, J IMMUNOL, V188, P3188, DOI 10.4049/jimmunol.1103354

Ren L, 2018, MEDICINE, V97, DOI 10.1097/MD.0000000000012172

Rey P, 2007, NEUROBIOL AGING, V28, P555, DOI 10.1016/j.neurobiolaging.2006.02.018

Reynolds AD, 2010, J IMMUNOL, V184, P2261, DOI 10.4049/jimmunol.0901852

Rodriguez-Pallares J, 2008, NEUROBIOL DIS, V31, P58, DOI 10.1016/j.nbd.2008.03.003

Rogers J, 1996, NEUROBIOL AGING, V17, P681, DOI 10.1016/0197-4580(96)00115-7

Rosenkranz D, 2007, J NEUROIMMUNOL, V188, P117, DOI 10.1016/j.jneuroim.2007.05.011

Rostami J, 2020, J NEUROINFLAMM, V17, DOI 10.1186/s12974-020-01776-7

Rowe DB, 1998, J NEUROSCI RES, V53, P551, DOI 10.1002/(SICI)1097-4547(19980901)53:5<551::AID-JNR5>3.0.CO;2-8

Roy A, 2015, NEUROSCIENCE, V302, P36, DOI 10.1016/j.neuroscience.2015.03.011

Roy J, 2017, FASEB J, V31, P3729, DOI 10.1096/fj.201700170R

Sabatino JJ, 2019, NAT REV NEUROSCI, V20, P728, DOI 10.1038/s41583-019-0233-2

Samii A, 2008, J CLIN NEUROSCI, V15, P1272, DOI 10.1016/j.jocn.2008.01.016

Sampson TR, 2016, CELL, V167, P1469, DOI 10.1016/j.cell.2016.11.018

Saunders JAH, 2012, J NEUROIMMUNE PHARM, V7, P927, DOI 10.1007/s11481-012-9402-z

Schonhoff AM, 2020, PROG BRAIN RES, V252, P169, DOI 10.1016/bs.pbr.2019.10.006

Seamon M., 2020, Clin. Exp. Neuroimmunol, V11, P47, DOI [10.1111/cen3.12553, DOI 10.1111/CEN3.12553]

Semerdzhiev SA, 2022, ACS CHEM NEUROSCI, V13, P143, DOI 10.1021/acschemneuro.1c00666

Shalash A, 2017, FRONT NEUROL, V8, DOI 10.3389/fneur.2017.00720

Shi Q, 2019, BMJ OPEN, V9, DOI 10.1136/bmjopen-2018-024954

Shukla AW, 2018, MOVEMENT DISORD, V33, P1560, DOI 10.1002/mds.27496

SIAN J, 1994, ANN NEUROL, V36, P348, DOI 10.1002/ana.410360305

Simola N, 2007, NEUROTOX RES, V11, P151, DOI 10.1007/BF03033565

Singh SS, 2020, NEUROTOX RES, V37, P491, DOI 10.1007/s12640-019-00147-2

Singleton AB, 2003, SCIENCE, V302, P841, DOI 10.1126/science.1090278

Smeyne M, 2013, FREE RADICAL BIO MED, V62, P13, DOI 10.1016/j.freeradbiomed.2013.05.001

Sommer A, 2018, CELL STEM CELL, V23, P123, DOI 10.1016/j.stem.2018.06.015

Sommer A, 2016, J NEUROINFLAMM, V13, DOI 10.1186/s12974-016-0632-5

Sopori M, 2002, NAT REV IMMUNOL, V2, P372, DOI 10.1038/nri803

Spielman LJ, 2018, NEUROCHEM INT, V120, P149, DOI 10.1016/j.neuint.2018.08.005

Srinivasan K, 2016, NAT COMMUN, V7, DOI 10.1038/ncomms11295

Stanton RC, 2012, IUBMB LIFE, V64, P362, DOI 10.1002/iub.1017

Stokholm MG, 2017, LANCET NEUROL, V16, P789, DOI 10.1016/S1474-4422(17)30173-4

Stone DK, 2009, ANTIOXID REDOX SIGN, V11, P2151, DOI [10.1089/ars.2009.2460, 10.1089/ARS.2009.2460]

Storelli E, 2019, FRONT NEUROL, V10, DOI 10.3389/fneur.2019.00013

Süss P, 2020, CELL REP, V30, P4082, DOI 10.1016/j.celrep.2020.02.109

Süss P, 2015, BRAIN BEHAV IMMUN, V49, P156, DOI 10.1016/j.bbi.2015.05.011

Sun CC, 2019, FRONT NEUROSCI-SWITZ, V13, DOI 10.3389/fnins.2019.01057

Sun MF, 2018, AGEING RES REV, V45, P53, DOI 10.1016/j.arr.2018.04.004

Surmeier DJ, 2017, NAT REV NEUROSCI, V18, P101, DOI 10.1038/nrn.2016.178

Tang Y, 2016, MOL NEUROBIOL, V53, P1181, DOI 10.1007/s12035-014-9070-5

te Riet L, 2015, CIRC RES, V116, P960, DOI 10.1161/CIRCRESAHA.116.303587

Teismann P, 2003, P NATL ACAD SCI USA, V100, P5473, DOI 10.1073/pnas.0837397100

Teismann P, 2012, BIOFACTORS, V38, P395, DOI 10.1002/biof.1035

Theodore S, 2008, J NEUROPATH EXP NEUR, V67, P1149, DOI 10.1097/NEN.0b013e31818e5e99

TOYKA KV, 1975, SCIENCE, V190, P397, DOI 10.1126/science.1179220

Tu DZ, 2019, J NEUROINFLAMM, V16, DOI 10.1186/s12974-019-1659-1

UNGERSTEDT U, 1968, EUR J PHARMACOL, V5, P107, DOI 10.1016/0014-2999(68)90164-7

UNGERSTEDT U, 1970, Brain Research, V24, P485, DOI 10.1016/0006-8993(70)90187-3

Valente EM, 2004, SCIENCE, V304, P1158, DOI 10.1126/science.1096284

Venkataramaiah S, 2019, J NEUROANAESTH CRIT, V6, P153, DOI 10.1055/s-0039-1689739

Visanji Naomi P, 2013, Acta Neuropathol Commun, V1, P2, DOI 10.1186/2051-5960-1-2

Wakade C, 2014, PLOS ONE, V9, DOI 10.1371/journal.pone.0109818

Walusinski O, 2018, REV NEUROL-FRANCE, V174, P491, DOI 10.1016/j.neurol.2017.08.005

Wang HX, 2016, CHINESE MED J-PEKING, V129, P2373, DOI 10.4103/0366-6999.190667

Wang P., 2022, FRONT IMMUNOL, V13, P814239, DOI [10.3389/fimmu.2022.814239, DOI 10.3389/FIMMU.2022.814239]

Wang SH, 2018, EXPERT REV NEUROTHER, V18, P573, DOI 10.1080/14737175.2018.1491310

Weindel CG, 2020, ELIFE, V9, DOI 10.7554/eLife.51071

Weiss R, 2015, INNATE IMMUN-LONDON, V21, P813, DOI 10.1177/1753425915602545

Wesley S, 2020, AUTOIMMUNE DISEASES, 6TH EDITION, P961, DOI 10.1016/B978-0-12-812102-3.00051-8

Wheeler C.J., 2014, Journal of neurology and neurophysiology, V5, P209, DOI [DOI 10.4172/2155-9562.1000209, 10.4172/2155-9562.1000209, 10.4172/2155-9562, DOI 10.4172/2155-9562]

Williams GP, 2021, BRAIN, V144, P2047, DOI 10.1093/brain/awab103

Wright John W, 2013, Front Endocrinol (Lausanne), V4, P158, DOI 10.3389/fendo.2013.00158

Wu DC, 2003, P NATL ACAD SCI USA, V100, P6145, DOI 10.1073/pnas.0937239100

Wu HY, 2017, J NEUROSCI, V37, P11701, DOI 10.1523/JNEUROSCI.1799-17.2017

YAMAGATA K, 1993, NEURON, V11, P371, DOI 10.1016/0896-6273(93)90192-T

Yang LY, 2018, FRONT NEUROSCI-SWITZ, V12, DOI 10.3389/fnins.2018.00073

Yun SP, 2018, NAT MED, V24, P931, DOI 10.1038/s41591-018-0051-5

Zawada WM, 2015, ACTA NEUROPATHOL COM, V3, DOI 10.1186/s40478-015-0189-z

Zhu XQ, 2017, ONCOTARGET, V8, P53829, DOI 10.18632/oncotarget.17754

ZIGMOND MJ, 1992, ANN NY ACAD SCI, V648, P71, DOI 10.1111/j.1749-6632.1992.tb24525.x

Zufferey A, 2014, J PROTEOMICS, V101, P130, DOI 10.1016/j.jprot.2014.02.008

NR 268

TC 44

Z9 44

U1 2

U2 8

PU FRONTIERS MEDIA SA

PI LAUSANNE

PA AVENUE DU TRIBUNAL FEDERAL 34, LAUSANNE, CH-1015, SWITZERLAND

SN 1664-3224

J9 FRONT IMMUNOL

JI Front. Immunol.

PD MAY 18

PY 2022

VL 13

AR 878771

DI 10.3389/fimmu.2022.878771

PG 18

WC Immunology

WE Science Citation Index Expanded (SCI-EXPANDED)

SC Immunology

GA 1V5IJ

UT WOS:000806122800001

PM 35663989

OA Green Published, gold

DA 2024-08-12

ER

PT J

AU Lee, JK

Tran, T

Tansey, MG

AF Lee, Jae-Kyung

Tran, Thi

Tansey, Malu G.

TI Neuroinflammation in Parkinson's Disease

SO JOURNAL OF NEUROIMMUNE PHARMACOLOGY

LA English

DT Review

DE microglia; Parkinson's disease; neuroinflammation; neurodegeneration;

peripheral immune cell; BBB

ID BLOOD-BRAIN-BARRIER; NONSTEROIDAL ANTIINFLAMMATORY DRUGS;

CENTRAL-NERVOUS-SYSTEM; SYNUCLEIN ACTIVATES MICROGLIA; ALPHA-SYNUCLEIN;

OXIDATIVE STRESS; CELL-DEATH; INTRANIGRAL INJECTION;

DOPAMINERGIC-NEURONS; ALZHEIMER-DISEASE

AB During the last two decades, a wealth of animal and human studies has implicated inflammation-derived oxidative stress and cytokine-dependent neurotoxicity in the progressive degeneration of the dopaminergic nigrostriatal pathway, the hallmark of Parkinson's disease (PD). In this review, we discuss the various hypotheses regarding the role of microglia and other immune cells in PD pathogenesis and progression, the inflammatory mechanisms implicated in disease progression from pre-clinical and clinical studies, the recent evidence that systemic inflammation can trigger microglia activation in PD-relevant central nervous system regions, the synergism between gene products linked to parkinsonian phenotypes (alpha-synuclein, parkin, Nurr1, and regulator of G-protein signaling-10) and neuroinflammation in promoting neurodegeneration of the nigrostriatal pathway, and the latest update on meta-analysis of epidemiological studies on the risk-lowering effects of anti-inflammatory drug regimens.

C1 [Tansey, Malu G.] Emory Univ, Sch Med, Dept Physiol, Atlanta, GA 30322 USA.

[Lee, Jae-Kyung; Tran, Thi; Tansey, Malu G.] Univ Texas SW Med Ctr Dallas, Dept Physiol, Dallas, TX 75390 USA.

C3 Emory University; University of Texas System; University of Texas

Southwestern Medical Center Dallas

RP Tansey, MG (corresponding author), Emory Univ, Sch Med, Dept Physiol, 615 Michael St, Atlanta, GA 30322 USA.

EM malu.tansey@emory.edu

OI LEE, JAE-KYUNG/0000-0003-0104-8623

FU NINDS NIH HHS [R01 NS049433-04, R01 NS049433] Funding Source: Medline

CR Aarnisalo P, 2002, J BIOL CHEM, V277, P35118, DOI 10.1074/jbc.M201707200

Abbott RD, 2003, J NEUROL, V250, P30, DOI 10.1007/s00415-003-1306-7

Astradsson A, 2009, NEUROBIOL DIS, V35, P348, DOI 10.1016/j.nbd.2009.05.018

Austin SA, 2006, J NEUROSCI, V26, P10558, DOI 10.1523/JNEUROSCI.1799-06.2006

Bartels AL, 2008, J NEURAL TRANSM, V115, P1001, DOI 10.1007/s00702-008-0030-y

Beal MF, 2005, ANN NEUROL, V58, P495, DOI 10.1002/ana.20624

Benner EJ, 2008, PLOS ONE, V3, DOI 10.1371/journal.pone.0001376

BENVENISTE EN, 1992, AM J PHYSIOL, V263, pC1, DOI 10.1152/ajpcell.1992.263.1.C1

Block ML, 2005, PROG NEUROBIOL, V76, P77, DOI 10.1016/j.pneurobio.2005.06.004

Brochard V, 2009, J CLIN INVEST, V119, P182, DOI 10.1172/JCI36470

Carvey PM, 2005, EUR J NEUROSCI, V22, P1158, DOI 10.1111/j.1460-9568.2005.04281.x

Carvey PM, 2003, FRONT BIOSCI-LANDMRK, V8, pS826, DOI 10.2741/1158

Castaño A, 1998, J NEUROCHEM, V70, P1584

Castaño A, 2002, J NEUROCHEM, V81, P150, DOI 10.1046/j.1471-4159.2002.00799.x

Chae SW, 2008, NEUROSCI LETT, V436, P205, DOI 10.1016/j.neulet.2008.03.031

Chen HL, 2005, ANN NEUROL, V58, P963, DOI 10.1002/ana.20682

Chen HL, 2003, ARCH NEUROL-CHICAGO, V60, P1059, DOI 10.1001/archneur.60.8.1059

Colton CA, 2009, J NEUROIMMUNE PHARM, V4, P399, DOI 10.1007/s11481-009-9164-4

De Pablos RM, 2004, FASEB J, V18, P407, DOI 10.1096/fj.04-2153fje

Engelhardt B, 2008, CURR PHARM DESIGN, V14, P1555, DOI 10.2174/138161208784705432

Esposito E, 2007, EXP NEUROL, V205, P295, DOI 10.1016/j.expneurol.2007.02.008

Fahn S, 2003, ANN NY ACAD SCI, V991, P1, DOI 10.1111/j.1749-6632.2003.tb07458.x

Farrer MJ, 2006, NAT REV GENET, V7, P306, DOI 10.1038/nrg1831

Floyd RA, 1999, P SOC EXP BIOL MED, V222, P236, DOI 10.1046/j.1525-1373.1999.d01-140.x

Frank-Cannon TC, 2008, J NEUROSCI, V28, P10825, DOI 10.1523/JNEUROSCI.3001-08.2008

Gao HM, 2002, J NEUROCHEM, V81, P1285, DOI 10.1046/j.1471-4159.2002.00928.x

Gao HM, 2008, J NEUROSCI, V28, P7687, DOI 10.1523/JNEUROSCI.0143-07.2008

Gerhard A, 2006, NEUROBIOL DIS, V21, P404, DOI 10.1016/j.nbd.2005.08.002

Ghosh A, 2007, P NATL ACAD SCI USA, V104, P18754, DOI 10.1073/pnas.0704908104

Goldberg MS, 2003, J BIOL CHEM, V278, P43628, DOI 10.1074/jbc.M308947200

Golde TE, 2009, MOL NEURODEGENER, V4, DOI 10.1186/1750-1326-4-8

Griffin WST, 2006, J NEUROINFLAMM, V3, DOI 10.1186/1742-2094-3-5

Hancock DB, 2007, ARCH NEUROL-CHICAGO, V64, P576, DOI 10.1001/archneur.64.4.576

Hernán MA, 2006, NEUROLOGY, V66, P1097, DOI 10.1212/01.wnl.0000204446.82823.28

Ito S, 2006, NEUROSCI RES, V56, P294, DOI 10.1016/j.neures.2006.07.009

Jang HM, 2009, P NATL ACAD SCI USA, V106, P14063, DOI 10.1073/pnas.0900096106

Jenner P, 1996, NEUROLOGY, V47, pS161, DOI 10.1212/WNL.47.6_Suppl_3.161S

Kim YS, 2006, EXP MOL MED, V38, P333, DOI 10.1038/emm.2006.40

KLEGERIS A, 2006, NEUROBIOL AGING, V20, P2000

Koprich JB, 2008, J NEUROINFLAMM, V5, DOI 10.1186/1742-2094-5-8

Kortekaas R, 2005, ANN NEUROL, V57, P176, DOI 10.1002/ana.20369

Le WD, 2003, NAT GENET, V33, P85, DOI 10.1038/ng1066

Lee JK, 2008, J NEUROSCI, V28, P8517, DOI 10.1523/JNEUROSCI.1806-08.2008

Liberatore GT, 1999, NAT MED, V5, P1403

Lin MT, 2006, NATURE, V443, P787, DOI 10.1038/nature05292

Liu YX, 2008, NEUROTOXICOLOGY, V29, P864, DOI 10.1016/j.neuro.2008.02.014

McGeer EG, 1998, EXP GERONTOL, V33, P371, DOI 10.1016/S0531-5565(98)00013-8

McGeer PL, 2008, MOVEMENT DISORD, V23, P474, DOI 10.1002/mds.21751

McGeer PL, 2007, NEUROBIOL AGING, V28, P639, DOI 10.1016/j.neurobiolaging.2006.03.013

McGeer PL, 2004, PARKINSONISM RELAT D, V10, pS3, DOI 10.1016/j.parkreldis.2004.01.005

MCGEER PL, 1988, ANN NEUROL, V24, P574, DOI 10.1002/ana.410240415

Minghetti L, 2005, BRAIN RES REV, V48, P251, DOI 10.1016/j.brainresrev.2004.12.015

Minghetti L, 2005, CURR OPIN NEUROL, V18, P315, DOI 10.1097/01.wco.0000169752.54191.97

Mizuno Y, 2001, ADV NEUROL, V86, P13

Moore DJ, 2005, ANNU REV NEUROSCI, V28, P57, DOI 10.1146/annurev.neuro.28.061604.135718

Mrak RE, 2005, NEUROBIOL AGING, V26, P349, DOI 10.1016/j.neurobiolaging.2004.05.010

Nagatsu T, 2006, CELL MOL NEUROBIOL, V26, P781, DOI 10.1007/s10571-006-9061-9

Nakamura Y, 2002, BIOL PHARM BULL, V25, P945, DOI 10.1248/bpb.25.945

Olanow CW, 2007, MOVEMENT DISORD, V22, pS335, DOI 10.1002/mds.21675

Orr CF, 2002, PROG NEUROBIOL, V68, P325, DOI 10.1016/S0301-0082(02)00127-2

Owen AD, 1996, ANN NY ACAD SCI, V786, P217, DOI 10.1111/j.1749-6632.1996.tb39064.x

Owen AD, 1997, J NEURAL TRANSM-SUPP, P167

Puntambekar Shweta S., 2008, P1, DOI 10.1007/978-0-387-73894-9_1

Purisai MG, 2007, NEUROBIOL DIS, V25, P392, DOI 10.1016/j.nbd.2006.10.008

Qin LY, 2007, GLIA, V55, P453, DOI 10.1002/glia.20467

Ransohoff RM, 2009, ANNU REV IMMUNOL, V27, P119, DOI 10.1146/annurev.immunol.021908.132528

Reynolds AD, 2009, J IMMUNOL, V182, P4137, DOI 10.4049/jimmunol.0803982

Rezai-Zadeh K, 2009, J NEUROIMMUNE PHARM, V4, P462, DOI 10.1007/s11481-009-9166-2

Saijo K, 2009, CELL, V137, P47, DOI 10.1016/j.cell.2009.01.038

Samii A, 2009, DRUG AGING, V26, P769, DOI 10.2165/11316780-000000000-00000

Sánchez-Pernaute R, 2004, J NEUROINFLAMM, V1, DOI 10.1186/1742-2094-1-6

Sawada M, 2006, J NEURAL TRANSM-SUPP, P373

Sedgwick JD, 2000, IMMUNOL TODAY, V21, P110, DOI 10.1016/S0167-5699(99)01573-X

Shimura H, 2000, NAT GENET, V25, P302, DOI 10.1038/77060

Su XM, 2008, NEUROBIOL AGING, V29, P1690, DOI 10.1016/j.neurobiolaging.2007.04.006

Sulzer D, 2007, TRENDS NEUROSCI, V30, P244, DOI 10.1016/j.tins.2007.03.009

Tanner CM, 2003, ADV NEUROL, V91, P133

Tansey MG, 2007, EXP NEUROL, V208, P1, DOI 10.1016/j.expneurol.2007.07.004

Tansey Malu G., 2008, P59

Teismann P, 2003, ANN NY ACAD SCI, V991, P272

Teismann P, 2003, P NATL ACAD SCI USA, V100, P5473, DOI 10.1073/pnas.0837397100

Tyurina YY, 2006, BRAIN RES, V1093, P71, DOI 10.1016/j.brainres.2005.10.105

UHL GR, 1985, NEUROLOGY, V35, P1215, DOI 10.1212/WNL.35.8.1215

Van Den Eeden SK, 2003, AM J EPIDEMIOL, V157, P1015, DOI 10.1093/aje/kwg068

Wang ZL, 2003, NATURE, V423, P555, DOI 10.1038/nature01645

Wersinger C, 2006, CURR MED CHEM, V13, P591, DOI 10.2174/092986706776055760

Westin JE, 2006, J NEUROSCI, V26, P9448, DOI 10.1523/JNEUROSCI.0944-06.2006

Whitton PS, 2007, BRIT J PHARMACOL, V150, P963, DOI 10.1038/sj.bjp.0707167

Wu DC, 2003, P NATL ACAD SCI USA, V100, P6145, DOI 10.1073/pnas.0937239100

Wyss-Coray T, 2006, NAT MED, V12, P1005, DOI 10.1038/nml1484

Zhang L, 2006, PHARMACOL THERAPEUT, V109, P33, DOI 10.1016/j.pharmthera.2005.05.007

Zhang W, 2005, FASEB J, V19, P533, DOI 10.1096/fj.04-2751com

NR 92

TC 124

Z9 141

U1 1

U2 33

PU SPRINGER

PI NEW YORK

PA ONE NEW YORK PLAZA, SUITE 4600, NEW YORK, NY, UNITED STATES

SN 1557-1890

EI 1557-1904

J9 J NEUROIMMUNE PHARM

JI J. Neuroimmune Pharm.

PD DEC

PY 2009

VL 4

IS 4

BP 419

EP 429

DI 10.1007/s11481-009-9176-0

PG 11

WC Neurosciences; Pharmacology & Pharmacy

WE Science Citation Index Expanded (SCI-EXPANDED)

SC Neurosciences & Neurology; Pharmacology & Pharmacy

GA 514TB

UT WOS:000271417400006

PM 19821032

OA Green Accepted

DA 2024-08-12

ER

PT J

AU He, SZ

Zhong, S

Liu, G

Yang, J

AF He, Songzhe

Zhong, Shan

Liu, Gang

Yang, Jun

TI Alpha-Synuclein: The Interplay of Pathology, Neuroinflammation, and

Environmental Factors in Parkinson's Disease

SO neurodegenerative disease

LA English

DT Review

DE alpha-Synuclein; Parkinson's disease; Protein aggregation;

Neuroinflammation; Environmental factors

ID LEWY BODY; ACTIVATES MICROGLIA; GAMMA-SYNUCLEIN; RISK-FACTORS; IN-VITRO;

MUTATION; BRAIN; MITOCHONDRIAL; DYSFUNCTION; FIBRILS

AB Background: Parkinson's disease (PD) is a multifactorial, chronic, and progressive neurodegenerative disease. alpha-Synuclein (alpha-syn), which is the main protein component of Lewy bodies, plays an important role in the pathological hallmarks of PD. However, the pathological function of alpha-syn and the molecular mechanisms responsible for the degeneration of dopaminergic neurons are still elusive. Summary: Cumulative evidence implicates that abnormal processing of alpha-syn will be predicted to lead to pathological changes in PD. Key Messages: In this review, we summarize the structure and physiological function of alpha-syn, and further discuss the interplay of pathology, neuroinflammation, and environmental factors in PD. Additionally, we suggest future directions for understanding the toxicity of alpha-syn to neurons, which may ultimately encourage us to better design disease-modifying therapeutic strategies for PD. (c) 2021 S. Karger AG, Basel

C1 [He, Songzhe; Yang, Jun] Guilin Med Univ, Affiliated Hosp, Clin Lab Dept, 15 Rd, Guilin 541001, Guangxi, China.

[He, Songzhe] Southern Med Univ, Nanfang Hosp, Dept Neurol, Guangzhou, China.

[Zhong, Shan; Liu, Gang] Guilin Hosp Tradit Chinese Med, Dept Acupuncture, Guilin, China.

C3 Guilin Medical University; Southern Medical University - China

RP Yang, J (corresponding author), Guilin Med Univ, Affiliated Hosp, Clin Lab Dept, 15 Rd, Guilin 541001, Guangxi, China.

EM yangjun977534@163.com

RI zhong, shan/HZJ-4647-2023

FU Young Teachers' Basic Ability Improvement Project of Guangxi

[2018KY0410]; Guangxi Medical and Health Appropriate Technology

Development and Promotion Application Project [S2018018]

FX This work was supported by the Young Teachers' Basic Ability Improvement

Project of Guangxi (No. 2018KY0410), Guangxi Medical and Health

Appropriate Technology Development and Promotion Application Project

(No. S2018018).

CR Abeliovich A, 2000, NEURON, V25, P239, DOI 10.1016/S0896-6273(00)80886-7

Agorogiannis EI, 2004, NEUROPATH APPL NEURO, V30, P215, DOI 10.1111/j.1365-2990.2004.00558.x

Akerman SC, 2019, J ALZHEIMERS DIS, V69, P463, DOI 10.3233/JAD-181191

Appel-Cresswell S, 2013, MOVEMENT DISORD, V28, P811, DOI 10.1002/mds.25421

Atik A, 2016, BRAIN PATHOL, V26, P410, DOI 10.1111/bpa.12370

Austin SA, 2006, J NEUROSCI, V26, P10558, DOI 10.1523/JNEUROSCI.1799-06.2006

Beal MF, 2001, NAT REV NEUROSCI, V2, P325, DOI 10.1038/35072550

Bennett MC, 2005, PHARMACOL THERAPEUT, V105, P311, DOI 10.1016/j.pharmthera.2004.10.010

Beraud D, 2011, FRONT NEUROSCI-SWITZ, V5, DOI 10.3389/fnins.2011.00080

Berg D, 2013, LANCET NEUROL, V12, P514, DOI 10.1016/S1474-4422(13)70047-4

Blauwendraat C, 2020, LANCET NEUROL, V19, P170, DOI 10.1016/S1474-4422(19)30287-X

Booth HDE, 2017, TRENDS NEUROSCI, V40, P358, DOI 10.1016/j.tins.2017.04.001

Braak H, 2003, J NEURAL TRANSM, V110, P517, DOI 10.1007/s00702-002-0808-2

Braak H, 2003, NEUROBIOL AGING, V24, P197, DOI 10.1016/S0197-4580(02)00065-9

Brochard V, 2009, J CLIN INVEST, V119, P182, DOI 10.1172/JCI36470

Burbulla LF, 2017, SCIENCE, V357, P1255, DOI 10.1126/science.aam9080

Cao SW, 2012, J NEUROINFLAMM, V9, DOI 10.1186/1742-2094-9-259

Cartier N, 2014, ACTA NEUROPATHOL, V128, P363, DOI 10.1007/s00401-014-1330-y

Cebrián C, 2014, NAT COMMUN, V5, DOI 10.1038/ncomms4633

Cervo PRD, 2017, NAT BIOTECHNOL, V35, P444, DOI 10.1038/nbt.3835

Chin-Chan M, 2015, FRONT CELL NEUROSCI, V9, DOI 10.3389/fncel.2015.00124

Chinta SJ, 2018, CELL REP, V22, P930, DOI 10.1016/j.celrep.2017.12.092

Comellas G, 2011, J MOL BIOL, V411, P881, DOI 10.1016/j.jmb.2011.06.026

Conway KA, 1998, NAT MED, V4, P1318, DOI 10.1038/3311

Coon S, 2006, ENVIRON HEALTH PERSP, V114, P1872, DOI 10.1289/ehp.9102

Dauer W, 2002, P NATL ACAD SCI USA, V99, P14524, DOI 10.1073/pnas.172514599

Dean RT, 1997, BIOCHEM J, V324, P1

Derkinderen P, 2014, MOVEMENT DISORD, V29, P976, DOI 10.1002/mds.25882

Ducas VC, 2012, J MOL BIOL, V423, P528, DOI 10.1016/j.jmb.2012.08.008

Emamzadeh FN, 2018, FRONT NEUROSCI-SWITZ, V12, DOI 10.3389/fnins.2018.00612

Engelender S, 2008, AUTOPHAGY, V4, P372, DOI 10.4161/auto.5604

Fellner L, 2013, GLIA, V61, P349, DOI 10.1002/glia.22437

Fu MH, 2018, BIOMED J, V41, P169, DOI 10.1016/j.bj.2018.02.005

Fu Y, 2018, J IMMUNOL RES, V2018, DOI 10.1155/2018/9348046

Galvin JE, 1999, P NATL ACAD SCI USA, V96, P13450, DOI 10.1073/pnas.96.23.13450

Gatto NM, 2009, ENVIRON HEALTH PERSP, V117, P1912, DOI 10.1289/ehp.0900852

George JM, 2002, GENOME BIOL, V3

Ghosh D, 2017, INT J BIOL MACROMOL, V100, P37, DOI 10.1016/j.ijbiomac.2016.10.021

Giannoccaro MP, 2017, MOVEMENT DISORD, V32, P346, DOI 10.1002/mds.26966

Giasson BI, 2000, SCIENCE, V290, P985, DOI 10.1126/science.290.5493.985

Gorell JM, 1997, NEUROLOGY, V48, P650, DOI 10.1212/WNL.48.3.650

Grabert K, 2016, NAT NEUROSCI, V19, P504, DOI 10.1038/nn.4222

Greenbaum EA, 2005, J BIOL CHEM, V280, P7800, DOI 10.1074/jbc.M411638200

Greten-Harrison B, 2010, P NATL ACAD SCI USA, V107, P19573, DOI 10.1073/pnas.1005005107

Grozdanov V, 2019, ANN NEUROL, V86, P593, DOI 10.1002/ana.25557

Hammond TR, 2019, IMMUNITY, V50, P253, DOI 10.1016/j.immuni.2018.11.004

Hou YJ, 2019, NAT REV NEUROL, V15, P565, DOI 10.1038/s41582-019-0244-7

Je G, 2018, MOL BRAIN, V11, DOI 10.1186/s13041-018-0371-x

Jia CY, 2019, FRONT NEUROSCI-SWITZ, V13, DOI 10.3389/fnins.2019.01124

Jiao ZG, 2019, ACS CHEM NEUROSCI, V10, P803, DOI 10.1021/acschemneuro.8b00373

Kalia LV, 2015, LANCET, V386, P896, DOI 10.1016/S0140-6736(14)61393-3

Kamp F, 2010, EMBO J, V29, P3571, DOI 10.1038/emboj.2010.223

Kaur S, 2012, CNS NEUROL DISORD-DR, V11, P387, DOI 10.2174/187152712800792811

Kelly R, 2020, MOLECULES, V25, DOI 10.3390/molecules25030453

Kiely AP, 2013, ACTA NEUROPATHOL, V125, P753, DOI 10.1007/s00401-013-1096-7

Kim S, 2019, NEURON, V103, P627, DOI 10.1016/j.neuron.2019.05.035

Klingelhoefer L, 2015, NAT REV NEUROL, V11, P625, DOI 10.1038/nrneurol.2015.197

Koprich JB, 2017, NAT REV NEUROSCI, V18, P515, DOI 10.1038/nrn.2017.75

Krüger R, 1998, NAT GENET, V18, P106, DOI 10.1038/ng0298-106

Lai BCL, 2002, PARKINSONISM RELAT D, V8, P297, DOI 10.1016/S1353-8020(01)00054-2

Lashuel HA, 2013, NAT REV NEUROSCI, V14, P38, DOI 10.1038/nrn3406

Lee EJ, 2010, J IMMUNOL, V185, P615, DOI 10.4049/jimmunol.0903480

Lee SB, 2009, BIOCHEM BIOPH RES CO, V381, P39, DOI 10.1016/j.bbrc.2009.02.002

Liddelow SA, 2017, NATURE, V541, P481, DOI 10.1038/nature21029

Liu YY, 2005, MOL NEUROBIOL, V31, P273, DOI 10.1385/MN:31:1-3:273

Ludtmann MHR, 2018, NAT COMMUN, V9, DOI 10.1038/s41467-018-04422-2

Luk KC, 2012, SCIENCE, V338, P949, DOI 10.1126/science.1227157

Luk KC, 2009, P NATL ACAD SCI USA, V106, P20051, DOI 10.1073/pnas.0908005106

McMillan KJ, 2017, MOL THER, V25, P2404, DOI 10.1016/j.ymthe.2017.08.017

Meier F, 2012, J AM CHEM SOC, V134, P5468, DOI 10.1021/ja300094r

Mohite GM, 2018, BIOCHEMISTRY-US, V57, P5014, DOI 10.1021/acs.biochem.8b00321

Mollenhauer B, 2011, LANCET NEUROL, V10, P230, DOI 10.1016/S1474-4422(11)70014-X

Neal M, 2018, BBA-MOL BASIS DIS, V1864, P432, DOI 10.1016/j.bbadis.2017.11.004

Ono K, 2011, NEUROBIOL DIS, V43, P715, DOI 10.1016/j.nbd.2011.05.025

Ouberai MM, 2013, J BIOL CHEM, V288, P20883, DOI 10.1074/jbc.M113.478297

Park JY, 2008, GLIA, V56, P1215, DOI 10.1002/glia.20691

Park J, 2013, J NEUROCHEM, V127, P221, DOI 10.1111/jnc.12361

Parmar M, 2020, NAT REV NEUROSCI, V21, P103, DOI 10.1038/s41583-019-0257-7

Pasanen P, 2014, NEUROBIOL AGING, V35, DOI 10.1016/j.neurobiolaging.2014.03.024

Patel D, 2018, HUM MOL GENET, V27, P1514, DOI 10.1093/hmg/ddy059

Peng C, 2020, NAT REV NEUROL, V16, P199, DOI 10.1038/s41582-020-0333-7

Polymeropoulos MH, 1997, SCIENCE, V276, P2045, DOI 10.1126/science.276.5321.2045

Prinz M, 2014, NAT REV NEUROSCI, V15, P300, DOI 10.1038/nrn3722

Prinz M, 2011, NAT NEUROSCI, V14, P1227, DOI 10.1038/nn.2923

Rocha EM, 2018, NEUROBIOL DIS, V109, P249, DOI 10.1016/j.nbd.2017.04.004

Santello M, 2019, NAT NEUROSCI, V22, P154, DOI 10.1038/s41593-018-0325-8

Saunders JAH, 2012, J NEUROIMMUNE PHARM, V7, P927, DOI 10.1007/s11481-012-9402-z

Saw G, 2020, GLIA, V68, P656, DOI 10.1002/glia.23748

Scheperjans F, 2015, J PARKINSON DIS, V5, P255, DOI 10.3233/JPD-150557

Schetters STT, 2018, FRONT IMMUNOL, V8, DOI 10.3389/fimmu.2017.01905

Shameli A, 2016, IMMUNOBIOLOGY, V221, P333, DOI 10.1016/j.imbio.2015.10.002

Shen JM, 2014, BRAIN RES, V1591, P14, DOI 10.1016/j.brainres.2014.09.046

Sofroniew MV, 2010, ACTA NEUROPATHOL, V119, P7, DOI 10.1007/s00401-009-0619-8

Souza JM, 2000, J BIOL CHEM, V275, P18344, DOI 10.1074/jbc.M000206200

Su XM, 2008, NEUROBIOL AGING, V29, P1690, DOI 10.1016/j.neurobiolaging.2007.04.006

Sui YT, 2014, PEPTIDES, V62, P197, DOI 10.1016/j.peptides.2014.09.018

Sulzer D, 2017, NATURE, V546, P656, DOI 10.1038/nature22815

Surguchov A, 2013, J NEUROSCI RES, V91, P161, DOI 10.1002/jnr.23149

Tanik SA, 2013, J BIOL CHEM, V288, P15194, DOI 10.1074/jbc.M113.457408

Tay TL, 2017, J PHYSIOL-LONDON, V595, P1929, DOI 10.1113/JP272134

Uversky VN, 2001, J BIOL CHEM, V276, P44284, DOI 10.1074/jbc.M105343200

Verkhratsky A, 2018, PHYSIOL REV, V98, P239, DOI 10.1152/physrev.00042.2016

Villar-Piqué A, 2016, J NEUROCHEM, V139, P240, DOI 10.1111/jnc.13249

Volpicelli-Daley LA, 2014, NAT PROTOC, V9, P2135, DOI 10.1038/nprot.2014.143

Volpicelli-Daley LA, 2011, NEURON, V72, P57, DOI 10.1016/j.neuron.2011.08.033

Wang CC, 2016, FRONT MOL NEUROSCI, V9, DOI 10.3389/fnmol.2016.00048

Wang JT, 2019, MOL NEUROBIOL, V56, P7664, DOI 10.1007/s12035-019-1567-5

Wang JT, 2018, EUR J NEUROSCI, V48, P1583, DOI 10.1111/ejn.13956

Wang SJ, 2016, J NEUROINFLAMM, V13, DOI 10.1186/s12974-016-0606-7

Wang ZP, 2019, BIOMED PHARMACOTHER, V115, DOI 10.1016/j.biopha.2019.108843

Yoshino H, 2017, NEUROBIOL AGING, V57, DOI 10.1016/j.neurobiolaging.2017.05.022

Yun SP, 2018, NAT MED, V24, P931, DOI 10.1038/s41591-018-0051-5

Yun SP, 2018, MOL NEURODEGENER, V13, DOI 10.1186/s13024-017-0233-5

Zarranz JJ, 2004, ANN NEUROL, V55, P164, DOI 10.1002/ana.10795

Zhang W, 2005, FASEB J, V19, P533, DOI 10.1096/fj.04-2751com

Zhang YL, 2017, CELL DEATH DIS, V8, DOI 10.1038/cddis.2016.491

Zheng W, 2012, PHARMACOL THERAPEUT, V133, P177, DOI 10.1016/j.pharmthera.2011.10.006

NR 117

TC 20

Z9 21

U1 3

U2 26

PU KARGER

PI BASEL

PA ALLSCHWILERSTRASSE 10, CH-4009 BASEL, SWITZERLAND

SN 1660-2854

EI 1660-2862

J9 NEURODEGENER DIS

JI Neurodegener. Dis.

PD FEB

PY 2021

VL 20

IS 2-3

BP 55

EP 64

DI 10.1159/000511083

EA JAN 2021

PG 10

WC Clinical Neurology; Neurosciences

WE Science Citation Index Expanded (SCI-EXPANDED)

SC Neurosciences & Neurology

GA QF6SR

UT WOS:000609317300001

PM 33465773

OA Bronze

DA 2024-08-12

ER

PT J

AU Zhao, YX

Liu, BH

Xu, L

Yu, SH

Fu, JY

Wang, J

Yan, XY

Su, J

AF Zhao, Yuanxin

Liu, Buhan

Xu, Long

Yu, Sihang

Fu, Jiaying

Wang, Jian

Yan, Xiaoyu

Su, Jing

TI ROS-Induced mtDNA Release: The Emerging Messenger for Communication

between Neurons and Innate Immune Cells during Neurodegenerative

Disorder Progression

SO ANTIOXIDANTS

LA English

DT Review

DE mtDNA; ROS; neurodegenerative disease; cGAS; STING; neuroinflammation

ID MITOCHONDRIAL-DNA; OXIDATIVE STRESS; alzheimer's disease;

HYDROGEN-SULFIDE; MODULATION; ACTIVATION; INJURY; CGAS; MACROPHAGES;

DYSFUNCTION

AB One of the most striking hallmarks shared by various neurodegenerative disease, including Parkinson's disease, Alzheimer's disease and amyotrophic lateral sclerosis, is microglia-mediated and astrocyte-mediated neuroinflammation. Although inhibitions of both harmful proteins and aggregation are major treatments for neurodegenerative disease, whether the phenomenon of non-normal protein or peptide aggregation is causally related to neuronal loss and synaptic damage is still controversial. Currently, excessive production of reactive oxygen species (ROS), which induces mitochondrial dysfunction in neurons that may play a key role in the regulation of immune cells, is proposed as a regulator in neurological disorders. In this review, we propose that mitochondrial DNA (mtDNA) release due to ROS may act on microglia and astrocytes adjacent to neurons to induce inflammation through activation of innate immune responses (such as cGAS/STING). Elucidating the relationship between mtDNA and the formation of a pro-inflammatory microenvironment could contribute to a better understanding of the mechanism of crosstalk between neuronal and peripheral immune cells and lead to the development of novel therapeutic approaches to neurodegenerative disease.

C1 [Zhao, Yuanxin; Liu, Buhan; Xu, Long; Yu, Sihang; Fu, Jiaying; Wang, Jian; Yan, Xiaoyu; Su, Jing] Jilin Univ, Coll Basic Med Sci, Dept Pathophysiol, Key Lab Pathobiol,Minist Educ, 126 Xinmin St, Changchun 130021, China.

C3 Jilin University

RP Yan, XY; Su, J (corresponding author), Jilin Univ, Coll Basic Med Sci, Dept Pathophysiol, Key Lab Pathobiol,Minist Educ, 126 Xinmin St, Changchun 130021, China.

EM yanxy@jlu.edu.cn; sujing@jlu.edu.cn

RI yan, xiaoyu/KQV-2239-2024; zhao, yuanxin/KDO-9377-2024; chen,

minghui/KFR-8832-2024; li, fangyu/KCY-0521-2024

OI li, fangyu/0009-0009-8303-9157; SU, JING/0000-0001-7636-2946

FU National Natural Science Foundation of China [82102733, 81772794]; Jilin

Provincial Research Foundation for the Development of Science and

Technology Projects [20200703009ZP, 20190201164JC, 20191008011TC]; Jilin

Provincial Health Technology Innovation Project [2021JC034, 2018SCZ021]

FX FundingThis research was funded by National Natural Science Foundation

of China (82102733 and 81772794); Jilin Provincial Research Foundation

for the Development of Science and Technology Projects (20200703009ZP,

20190201164JC and 20191008011TC); Jilin Provincial Health Technology

Innovation Project (2021JC034 and 2018SCZ021).

CR Aarreberg LD, 2019, MOL CELL, V74, P801, DOI 10.1016/j.molcel.2019.02.038

Alam M, 2021, CURR MOL PHARMACOL, V14, P263, DOI 10.2174/1566524020666200427214356

Ali T, 2018, MOL NEUROBIOL, V55, P6076, DOI 10.1007/s12035-017-0798-6

Angelova PR, 2018, FEBS LETT, V592, P692, DOI 10.1002/1873-3468.12964

Attia HN, 2018, BEHAV PHARMACOL, V29, P79, DOI 10.1097/FBP.0000000000000342

Bao DK, 2019, ONCOGENE, V38, P5007, DOI 10.1038/s41388-019-0772-z

Bargiela D, 2019, NEUROSCI LETT, V710, DOI 10.1016/j.neulet.2017.06.051

Belarbi K, 2020, MOL NEURODEGENER, V15, DOI 10.1186/s13024-020-00408-1

Bradley-Whitman MA, 2014, J NEUROCHEM, V128, P294, DOI 10.1111/jnc.12444

Brambilla G, 1987, Radiol Med, V74, P49

Campolo M, 2017, ANTIOXID REDOX SIGN, V27, P453, DOI 10.1089/ars.2016.6800

Cano A, 2019, J CONTROL RELEASE, V301, P62, DOI 10.1016/j.jconrel.2019.03.010

Cervera-Carles L, 2017, NEUROBIOL AGING, V53, DOI 10.1016/j.neurobiolaging.2016.12.009

Cheng AN, 2020, J IMMUNOTHER CANCER, V8, DOI 10.1136/jitc-2020-001372

Denzer I, 2016, PHARMACOL RES, V103, P80, DOI 10.1016/j.phrs.2015.11.019

Eftekharzadeh B, 2010, BIOCHIMIE, V92, P245, DOI 10.1016/j.biochi.2009.12.001

Enogieru AB, 2018, OXID MED CELL LONGEV, V2018, DOI 10.1155/2018/6241017

Erkkinen MG, 2018, CSH PERSPECT BIOL, V10, DOI 10.1101/cshperspect.a033118

Ettcheto M, 2020, MOL NEUROBIOL, V57, P1814, DOI 10.1007/s12035-019-01849-6

Gambardella S, 2019, FRONT IMMUNOL, V10, DOI 10.3389/fimmu.2019.01064

Gao YJ, 2020, DIGEST LIVER DIS, V52, P637, DOI 10.1016/j.dld.2020.03.021

Ghosh M, 2021, CANCER CELL, V39, P494, DOI 10.1016/j.ccell.2021.01.003

Giordano S, 2014, REDOX BIOL, V2, P82, DOI 10.1016/j.redox.2013.12.013

Giovinazzo D, 2021, P NATL ACAD SCI USA, V118, DOI 10.1073/pnas.2017225118

Giuliani D, 2013, NEUROBIOL LEARN MEM, V104, P82, DOI 10.1016/j.nlm.2013.05.006

Gutierrez-Merino C, 2011, CURR MED CHEM, V18, P1195, DOI 10.2174/092986711795029735

Han YL, 2020, J IMMUNOL, V204, P1437, DOI 10.4049/jimmunol.1900869

Heemels MT, 2016, NATURE, V539, P179, DOI 10.1038/539179a

Heneka MT, 2018, NAT REV NEUROSCI, V19, P610, DOI 10.1038/s41583-018-0055-7

Hirsch EC, 2021, MOVEMENT DISORD, V36, P16, DOI 10.1002/mds.28075

Hirsch Etienne C, 2012, Parkinsonism Relat Disord, V18 Suppl 1, pS210, DOI 10.1016/S1353-8020(11)70065-7

Hu J, 2019, J CLIN INVEST, V129, P5204, DOI 10.1172/JCI123835

Huang LS, 2020, IMMUNITY, V52, P475, DOI 10.1016/j.immuni.2020.02.002

Ignatenko O, 2018, NAT COMMUN, V9, DOI 10.1038/s41467-017-01859-9

Jauhari A, 2021, J CLIN INVEST, V131, DOI 10.1172/JCI150328

Johnson J, 2021, ARCH BIOCHEM BIOPHYS, V702, DOI 10.1016/j.abb.2020.108698

Kim J, 2019, SCIENCE, V366, P1531, DOI 10.1126/science.aav4011

Koh SH, 2006, NEUROSCI LETT, V395, P103, DOI 10.1016/j.neulet.2005.10.056

Koike H, 2021, MOLECULES, V26, DOI 10.3390/molecules26165091

Krysko DV, 2011, TRENDS IMMUNOL, V32, P157, DOI 10.1016/j.it.2011.01.005

Kwon HS, 2020, TRANSL NEURODEGENER, V9, DOI 10.1186/s40035-020-00221-2

Lee S, 2020, J NEUROINFLAMM, V17, DOI 10.1186/s12974-020-01963-6

Leurs CE, 2018, MULT SCLER J, V24, P472, DOI 10.1177/1352458517699874

Li TB, 2021, FRONT AGING NEUROSCI, V13, DOI 10.3389/fnagi.2021.765395

Liao YJ, 2020, THERANOSTICS, V10, P9644, DOI 10.7150/thno.47651

Liddelow SA, 2017, NATURE, V541, P481, DOI 10.1038/nature21029

Ma H, 2018, INT J MOL SCI, V19, DOI 10.3390/ijms19020461

Maher P, 2019, INT J MOL SCI, V20, DOI 10.3390/ijms20123056

Mathur V, 2017, NEURON, V96, P1290, DOI 10.1016/j.neuron.2017.11.032

Meng XY, 2020, OXID MED CELL LONGEV, V2020, DOI 10.1155/2020/8787156

Motwani M, 2019, NAT REV GENET, V20, P657, DOI 10.1038/s41576-019-0151-1

Nakahira K, 2011, NAT IMMUNOL, V12, P222, DOI 10.1038/ni.1980

Nakanishi H, 2011, NEURON GLIA BIOL, V7, P17, DOI 10.1017/S1740925X1100010X

Nasi M, 2020, MOL CELL NEUROSCI, V108, DOI 10.1016/j.mcn.2020.103538

Ozasa K, 2019, FRONT IMMUNOL, V10, DOI 10.3389/fimmu.2019.02212

Özdemir Z, 2021, CURR MED CHEM, V28, P6045, DOI 10.2174/0929867328666210203204710

Patel M, 2016, TRENDS PHARMACOL SCI, V37, P768, DOI 10.1016/j.tips.2016.06.007

Petrovic S, 2020, ANTIOXIDANTS-BASEL, V9, DOI 10.3390/antiox9111128

Pinti M, 2021, NEURAL REGEN RES, V16, P2393, DOI 10.4103/1673-5374.313034

Podlesniy P, 2013, ANN NEUROL, V74, P655, DOI 10.1002/ana.23955

Prasad A, 2019, FRONT MOL NEUROSCI, V12, DOI 10.3389/fnmol.2019.00025

Riley JS, 2018, EMBO J, V37, DOI 10.15252/embj.201899238

Salmina AB, 2015, FRONT PHYSIOL, V6, DOI 10.3389/fphys.2015.00361

Sansone P, 2017, P NATL ACAD SCI USA, V114, pE9066, DOI 10.1073/pnas.1704862114

Schepici G, 2020, INT J MOL SCI, V21, DOI 10.3390/ijms21228637

Scuderi SA, 2020, ANTIOXIDANTS-BASEL, V9, DOI 10.3390/antiox9070630

Sen T, 2020, P NATL ACAD SCI USA, V117, P4418, DOI 10.1073/pnas.1916895117

Simao F, 2012, EUR J NEUROSCI, V36, P2899, DOI 10.1111/j.1460-9568.2012.08229.x

Singh A, 2019, MOLECULES, V24, DOI 10.3390/molecules24081583

Singh SK, 2019, NEUROTHERAPEUTICS, V16, P666, DOI 10.1007/s13311-019-00767-8

Sliter DA, 2018, NATURE, V561, P258, DOI 10.1038/s41586-018-0448-9

Tabassum R, 2019, INT J MED SCI, V16, P1386, DOI 10.7150/ijms.36516

Tellone E, 2015, OXID MED CELL LONGEV, V2015, DOI 10.1155/2015/392169

Torralba D, 2018, NAT COMMUN, V9, DOI 10.1038/s41467-018-05077-9

Tsilioni I, 2018, J NEUROINFLAMM, V15, DOI 10.1186/s12974-018-1275-5

Tusi SK, 2010, J BIOTECHNOL, V150, pS455, DOI 10.1016/j.jbiotec.2010.09.667

Vargas MR, 2006, J NEUROCHEM, V97, P687, DOI 10.1111/j.1471-4159.2006.03742.x

Vellas B, 2012, LANCET NEUROL, V11, P851, DOI 10.1016/S1474-4422(12)70206-5

Vucic S, 2020, MEDICINE, V99, DOI 10.1097/MD.0000000000018904

Wang JY, 2006, CURR PHARM DESIGN, V12, P3521, DOI 10.2174/138161206778343109

Webers A, 2020, IMMUNOL CELL BIOL, V98, P28, DOI 10.1111/imcb.12301

Wolf SA, 2017, ANNU REV PHYSIOL, V79, P619, DOI 10.1146/annurev-physiol-022516-034406

Xu HD, 2021, CANCER BIOL MED, V18, P172, DOI 10.20892/j.issn.2095-3941.2020.0211

Xu HD, 2019, FRONT IMMUNOL, V10, DOI 10.3389/fimmu.2019.00127

Yamazaki H, 2015, PATHOL INT, V65, P210, DOI 10.1111/pin.12261

Yong HYF, 2019, CELL MOL IMMUNOL, V16, P540, DOI 10.1038/s41423-019-0223-3

Yu CH, 2020, CELL, V183, P636, DOI 10.1016/j.cell.2020.09.020

Yu K, 2019, NEUROPATH APPL NEURO, V45, P119, DOI 10.1111/nan.12489

Yun Y, 2020, INT J MOL SCI, V21, DOI 10.3390/ijms21113801

Zeng F, 2018, ANGEW CHEM INT EDIT, V57, P5808, DOI 10.1002/anie.201802309

Zhang XF, 2020, CELL DEATH DIS, V11, DOI 10.1038/s41419-020-03239-6

Zhao M, 2021, THERANOSTICS, V11, P1845, DOI 10.7150/thno.50905

Zhong L, 2020, CELL DISCOV, V6, DOI 10.1038/s41421-020-0162-2

Zhong X, 2019, MOL NUTR FOOD RES, V63, DOI 10.1002/mnfr.201801230

Zuo XX, 2021, NAT STRUCT MOL BIOL, V28, P132, DOI 10.1038/s41594-020-00537-7

NR 95

TC 34

Z9 36

U1 8

U2 68

PU MDPI

PI BASEL

PA ST ALBAN-ANLAGE 66, CH-4052 BASEL, SWITZERLAND

EI 2076-3921

J9 ANTIOXIDANTS-BASEL

JI Antioxidants

PD DEC

PY 2021

VL 10

IS 12

AR 1917

DI 10.3390/antiox10121917

PG 13

WC Biochemistry & Molecular Biology; Chemistry, Medicinal; Food Science &

Technology

WE Science Citation Index Expanded (SCI-EXPANDED)

SC Biochemistry & Molecular Biology; Pharmacology & Pharmacy; Food Science

& Technology

GA XW2WE

UT WOS:000735485300001

PM 34943020

OA gold, Green Published

DA 2024-08-12

ER

PT J

AU Singh, S

Sahu, K

Singh, C

Singh, A

AF Singh, Sukhdev

Sahu, Kuleshwar

Singh, Charan

Singh, Arti

TI Lipopolysaccharide induced altered signaling pathways in various

neurological disorders

SO NAUNYN-SCHMIEDEBERGS ARCHIVES OF PHARMACOLOGY

LA English

DT Review

DE Neuroinflammation; Lipopolysaccharide; Toll-like receptor;

MyD88-dependent; MyD88-independent

ID NF-KAPPA-B; DOUBLE-STRANDED-RNA; CYTOKINE PRODUCTION; GENE-EXPRESSION;

IN-VIVO; RECEPTOR; INNATE; RECOGNITION; LPS; INFLAMMATION

AB Neuroinflammation is defined as an inflammatory response within the brain or spinal cord, whereas the brain's innate immune system is triggered by various inflammatory challenges such as injury, infection, exposure to toxin (LPS) and ageing, which result in cognitive impairment and neurodegenerative disease including Alzheimer's disease (AD), Parkinson's disease (PD), amyotrophic lateral sclerosis (ALS) and multiple sclerosis (MS). Lipopolysaccharide (LPS) is a main structural component of the outer membrane of gram-negative bacteria, widely used systematically to stimulate the immune system and to generate profound physiological and behavioural changes. It consists of three parts: lipid A, a core oligosaccharide and an O side chain. It is reported by several scientists that, besides the systemic alteration, LPS also induces neurodegeneration by promoting neuroinflammation upon binding with the stimulation of Toll-like receptor-4 (TLR4) receptors present on glial cells. The mammalian Toll-like receptor (TLR) family consists of 13 membranes and TLR was discovered as a crucial pattern recognition receptor (PPR) involved in the recognition of pathogen-associated molecular patterns (PAMPs). Future studies will show that damage/danger-associated molecular patterns (DAMPs) are recognised by the involvement of PPRs, generated by the host itself. The stimulation of TLR4 by lipopolysaccharide phosphorylates two signalling pathways, namely the MyD88-dependent pathway and the MyD88-independent pathway. This activation subsequently triggers the release of various pro-inflammatory cytokines that are necessary to activate innate immune responses, and then promotes neuroinflammation. In this review, we critically demonstrated the epidemiology of neuroinflammation, types of TLRs, the molecular mechanism of TLR4 and management of neuroinflammation.

C1 [Singh, Sukhdev; Sahu, Kuleshwar; Singh, Arti] ISF Coll Pharm, Dept Pharmacol, Moga 142001, Punjab, India.

[Singh, Charan] ISF Coll Pharm, Dept Pharmaceut, Moga 142001, Punjab, India.

[Singh, Sukhdev; Sahu, Kuleshwar; Singh, Charan; Singh, Arti] IK Gujral Punjab Tech Univ, Jalandhar 144603, Punjab, India.

C3 ISF College of Pharmacy; ISF College of Pharmacy; I. K. Gujral Punjab

Technical University

RP Singh, A (corresponding author), ISF Coll Pharm, Dept Pharmacol, Moga 142001, Punjab, India.; Singh, A (corresponding author), IK Gujral Punjab Tech Univ, Jalandhar 144603, Punjab, India.

EM artiniper@gmail.com

RI Singh, Arti/HLX-4417-2023; Singh, Charan/GZL-3630-2022

OI Singh, Charan/0000-0002-1630-3183; , Arti/0000-0001-5168-4930

CR Abd Wahab DYA, 2019, BIOMED RES INT-UK, V2019, DOI 10.1155/2019/1767203

Akira S, 2006, CELL, V124, P783, DOI 10.1016/j.cell.2006.02.015

Alexopoulou L, 2001, NATURE, V413, P732, DOI 10.1038/35099560

Bao LH., 2018, J NEUROINFLAMMATION, V15, P1

Bauer S, 2001, P NATL ACAD SCI USA, V98, P9237, DOI 10.1073/pnas.161293498

Beutler B, 2003, NAT REV IMMUNOL, V3, P169, DOI 10.1038/nri1004

Botos I, 2011, STRUCTURE, V19, P447, DOI 10.1016/j.str.2011.02.004

Chang LF, 2001, NATURE, V410, P37, DOI 10.1038/35065000

Cherry S, 2006, NAT IMMUNOL, V7, P911, DOI 10.1038/ni1388

Chung ES, 2010, BRAIN RES, V1363, P143, DOI 10.1016/j.brainres.2010.09.049

Covert MW, 2005, SCIENCE, V309, P1854, DOI 10.1126/science.1112304

Dajas F, 2003, BRAZ J MED BIOL RES, V36, P1613, DOI 10.1590/S0100-879X2003001200002

Deng L, 2000, CELL, V103, P351, DOI 10.1016/S0092-8674(00)00126-4

Elkon KB, 2019, DUBOISLUPUS ERYTHEMA, P93, DOI [DOI 10.1016/B978-0-323-47927-1.00008-6, DOI 10.1074/jbc.M700017200]

Fitzgerald KA, 2003, NAT IMMUNOL, V4, P491, DOI 10.1038/ni921

Gao YT, 2020, METAB BRAIN DIS, V35, P661, DOI 10.1007/s11011-020-00533-2

Global Burden of Disease Study Collaborators, 2021, Lancet Neurol., V20, P795, DOI [10.1016/S1474-4422(20)30308-2, DOI 10.1016/S1474-4422(21)00252-0]

Guo BC, 2007, J BIOL CHEM, V282, P11817, DOI 10.1074/jbc.M700017200

Gupta M, 2018, NEUROMOL MED, V20, P343, DOI 10.1007/s12017-018-8497-7

Hayashi F, 2001, NATURE, V410, P1099, DOI 10.1038/35074106

He RL, 2009, BLOOD, V113, P429, DOI 10.1182/blood-2008-03-139923

Heil F, 2004, SCIENCE, V303, P1526, DOI 10.1126/science.1093620

Hemmi H, 2004, J EXP MED, V199, P1641, DOI 10.1084/jem.20040520

Hopfner F, 2020, LANCET NEUROL, V19, P247, DOI 10.1016/S1474-4422(19)30400-4

Hosoi T, 2014, NEUROSCI LETT, V569, P23, DOI 10.1016/j.neulet.2014.03.053

Hwang JH, 2016, CHEM-BIOL INTERACT, V248, P1, DOI 10.1016/j.cbi.2016.01.020

Iwasaki A, 2004, NAT IMMUNOL, V5, P987, DOI 10.1038/ni1112

Jin MS, 2007, CELL, V130, P1071, DOI 10.1016/j.cell.2007.09.008

Johnson GB, 2002, J IMMUNOL, V168, P5233, DOI 10.4049/jimmunol.168.10.5233

Jung JS, 2017, J AGR FOOD CHEM, V65, P373, DOI 10.1021/acs.jafc.6b05147

Kagan JC, 2012, TRENDS IMMUNOL, V33, P442, DOI 10.1016/j.it.2012.06.005

Kang JY, 2009, IMMUNITY, V31, P873, DOI 10.1016/j.immuni.2009.09.018

Kawai T, 1999, IMMUNITY, V11, P115, DOI 10.1016/S1074-7613(00)80086-2

Kawasaki T, 2014, FRONT IMMUNOL, V5, DOI 10.3389/fimmu.2014.00461

Khan A, 2018, FRONT PHARMACOL, V9, DOI 10.3389/fphar.2018.01383

Kumar V, 2019, J NEUROIMMUNOL, V332, P16, DOI 10.1016/j.jneuroim.2019.03.012

Li SY, 2002, P NATL ACAD SCI USA, V99, P5567, DOI 10.1073/pnas.082100399

Liwinski T, 2020, IMMUNOL REV, V297, P207, DOI 10.1111/imr.12901

LORD KA, 1990, ONCOGENE, V5, P1095

Lu YC, 2008, CYTOKINE, V42, P145, DOI 10.1016/j.cyto.2008.01.006

Maitra U, 2012, J IMMUNOL, V189, P1014, DOI 10.4049/jimmunol.1200857

Majdi F, 2019, MED HYPOTHESES, V133, DOI 10.1016/j.mehy.2019.109371

MATZINGER P, 1994, ANNU REV IMMUNOL, V12, P991, DOI 10.1146/annurev.iy.12.040194.005015

Meylan E, 2004, NAT IMMUNOL, V5, P503, DOI 10.1038/ni1061

Miller SI, 2005, NAT REV MICROBIOL, V3, P36, DOI 10.1038/nrmicro1068

Moradi-Marjaneh R, 2018, J CELL PHYSIOL, V233, P5613, DOI 10.1002/jcp.26273

Motoyama M, 2005, J BIOL CHEM, V280, P7444, DOI 10.1074/jbc.M412738200

Murthy M. R. Ven, 2010, Central Nervous System Agents in Medicinal Chemistry, V10, P238

Nicholas C, 2007, J IMMUNOL, V179, P7121, DOI 10.4049/jimmunol.179.10.7121

O'Neill LAJ, 2007, NAT REV IMMUNOL, V7, P353, DOI 10.1038/nri2079

Oganesyan G, 2006, NATURE, V439, P208, DOI 10.1038/nature04374

OHANIAN SH, 1967, J EXP MED, V125, P1137, DOI 10.1084/jem.125.6.1137

OITZL MS, 1993, BRAIN RES, V613, P160, DOI 10.1016/0006-8993(93)90468-3

Olanrewaju AA, 2020, J NEUROIMMUNE PHARM, V15, P473, DOI 10.1007/s11481-020-09916-9

Paludan SR, 2013, IMMUNITY, V38, P870, DOI 10.1016/j.immuni.2013.05.004

Plóciennikowska A, 2015, CELL MOL LIFE SCI, V72, P557, DOI 10.1007/s00018-014-1762-5

Poivre M, 2017, J ZHEJIANG UNIV-SC B, V18, P194, DOI [10.1631/jzus.B1600299, 10.1631/jzus.b1600299]

Poltorak A, 1998, BLOOD CELL MOL DIS, V24, P340, DOI 10.1006/bcmd.1998.0201

Raetz CRH, 2002, ANNU REV BIOCHEM, V71, P635, DOI 10.1146/annurev.biochem.71.110601.135414

Rosadini CV, 2017, CURR OPIN IMMUNOL, V44, P14, DOI 10.1016/j.coi.2016.10.005

Rose NR., 2006, The Autoimmune Diseases, V4, DOI DOI 10.1126/science.1087262

Sánchez-Paz A, 2020, REV AQUACULT, V12, P1818, DOI 10.1111/raq.12411

Sandri S, 2008, J LEUKOCYTE BIOL, V83, P1174, DOI 10.1189/jlb.0407203

Sato S, 2005, NAT IMMUNOL, V6, P1087, DOI 10.1038/ni1255

Scaffidi P, 2002, NATURE, V418, P191, DOI 10.1038/nature00858

Sinha K, 2015, BBA-GEN SUBJECTS, V1850, P769, DOI 10.1016/j.bbagen.2015.01.008

Slavich GM, 2014, PSYCHOL BULL, V140, P774, DOI 10.1037/a0035302

Smith NC, 2019, FRONT IMMUNOL, V10, DOI 10.3389/fimmu.2019.02292

Smolinski AT, 2003, FOOD CHEM TOXICOL, V41, P1381, DOI 10.1016/S0278-6915(03)00146-7

Suzuki N, 2002, NATURE, V416, P750, DOI 10.1038/nature736

Takaoka A, 2005, NATURE, V434, P243, DOI 10.1038/nature03308

Taylor KR, 2007, J BIOL CHEM, V282, P18265, DOI 10.1074/jbc.M606352200

Tiwari KK, 2014, BIOCHEM BIOPH RES CO, V450, P1345, DOI 10.1016/j.bbrc.2014.06.132

Vabulas RM, 2002, CURR TOP MICROBIOL, V270, P169

Vaure C, 2014, FRONT IMMUNOL, V5, DOI 10.3389/fimmu.2014.00316

Wang X, 2020, MED RES REV, V40, P2466, DOI 10.1002/med.21711

Xu L, 2020, J NEUROL, V267, P944, DOI 10.1007/s00415-019-09652-y

Yamamoto M, 2003, SCIENCE, V301, P640, DOI 10.1126/science.1087262

Yamamoto M, 2004, NATURE, V430, P218, DOI 10.1038/nature02738

Yu L, 2010, J CELL MOL MED, V14, P2592, DOI 10.1111/j.1582-4934.2010.01127.x

Zakaria R, 2017, PHYSIOL RES, V66, P553, DOI 10.33549/physiolres.933480

Zhang B, 2019, MOLECULES, V24, DOI 10.3390/molecules24112035

Zhang P, 2013, NEUROSCI LETT, V534, P123, DOI 10.1016/j.neulet.2012.11.052

Zhang TZ, 2014, INFLAMMATION, V37, P1976, DOI 10.1007/s10753-014-9930-1

Zhang ZJ, 2011, INT J MOL MED, V27, P195, DOI 10.3892/ijmm.2010.571

Zhao XX, 2020, NEUROL RES, V42, P291, DOI 10.1080/01616412.2020.1716467

NR 86

TC 15

Z9 15

U1 2

U2 24

PU SPRINGER

PI NEW YORK

PA ONE NEW YORK PLAZA, SUITE 4600, NEW YORK, NY, UNITED STATES

SN 0028-1298

EI 1432-1912

J9 N-S ARCH PHARMACOL

JI Naunyn-Schmiedebergs Arch. Pharmacol.

PD MAR

PY 2022

VL 395

IS 3

BP 285

EP 294

DI 10.1007/s00210-021-02198-9

EA JAN 2022

PG 10

WC Pharmacology & Pharmacy

WE Science Citation Index Expanded (SCI-EXPANDED)

SC Pharmacology & Pharmacy

GA YT4SH

UT WOS:000739769200001

PM 34989812

DA 2024-08-12

ER

PT J

AU Wallin, J

Svenningsson, P

AF Wallin, Johan

Svenningsson, Per

TI Potential Effects of Leukotriene Receptor Antagonist Montelukast in

Treatment of Neuroinflammation in Parkinson's Disease

SO INTERNATIONAL JOURNAL OF MOLECULAR SCIENCES

LA English

DT Review

DE montelukast; alpha-synuclein; leukotriene; microglia; neuroinflammation;

Parkinson's disease

ID MICROGLIAL ACTIVATION; CYSTEINYL LEUKOTRIENES; DOPAMINERGIC-NEURONS;

THERAPEUTIC TARGETS; alzheimer's disease; ALPHA-SYNUCLEIN; MOUSE MODEL;

BRAIN; 5-LIPOXYGENASE; INNATE

AB Parkinson's disease (PD) is a neurodegenerative disorder where misfolded alpha-synuclein-enriched aggregates called Lewy bodies are central in pathogenesis. No neuroprotective or disease-modifying treatments are currently available. Parkinson's disease is considered a multifactorial disease and evidence from multiple patient studies and animal models has shown a significant immune component during the course of the disease, highlighting immunomodulation as a potential treatment strategy. The immune changes occur centrally, involving microglia and astrocytes but also peripherally with changes to the innate and adaptive immune system. Here, we review current understanding of different components of the PD immune response with a particular emphasis on the leukotriene pathway. We will also describe evidence of montelukast, a leukotriene receptor antagonist, as a possible anti-inflammatory treatment for PD.

C1 [Wallin, Johan; Svenningsson, Per] Karolinska Inst, Dept Clin Neurosci, Lab Translat Neuropharmacol, S-17177 Stockholm, Sweden.

C3 Karolinska Institutet

RP Svenningsson, P (corresponding author), Karolinska Inst, Dept Clin Neurosci, Lab Translat Neuropharmacol, S-17177 Stockholm, Sweden.

EM svenningsson@ki.se; per.svenningsson@ki.se

RI Svenningsson, Per JL/K-5210-2014

OI Wallin, Johan/0000-0003-0490-9900; Svenningsson, Per/0000-0001-6727-3802

FU Axel and Margaret Ax:son Johnsons foundation

FX This work was supported by a grant from the Axel and Margaret Ax:son

Johnsons foundation.

CR Aigner L, 2020, FRONT MOL BIOSCI, V7, DOI 10.3389/fmolb.2020.610132

Alam Q, 2016, CURR PHARM DESIGN, V22, P541, DOI 10.2174/1381612822666151125000300

AlQasrawi D, 2020, MICROORGANISMS, V8, DOI 10.3390/microorganisms8050666

Arlehamn CSL, 2020, NAT COMMUN, V11, DOI 10.1038/s41467-020-15626-w

Bäck M, 2014, BRIT J PHARMACOL, V171, P3551, DOI 10.1111/bph.12665

Berg D, 2015, MOVEMENT DISORD, V30, P1600, DOI 10.1002/mds.26431

Best L, 2019, CURR NEUROL NEUROSCI, V19, DOI 10.1007/s11910-019-0934-y

Booth HDE, 2017, TRENDS NEUROSCI, V40, P358, DOI 10.1016/j.tins.2017.04.001

Brochard V, 2009, J CLIN INVEST, V119, P182, DOI 10.1172/JCI36470

Calabrese VP, 2007, NEUROLOGY, V69, P223, DOI 10.1212/01.wnl.0000271777.50910.73

Campolo M, 2019, BRAIN BEHAV IMMUN, V76, P236, DOI 10.1016/j.bbi.2018.12.003

Cerami C, 2017, INT J MOL SCI, V18, DOI 10.3390/ijms18050993

Chen F, 2020, BRAIN BEHAV IMMUN, V88, P844, DOI 10.1016/j.bbi.2020.03.022

Chen X, 2018, FRONT IMMUNOL, V9, DOI 10.3389/fimmu.2018.02122

Chen ZC, 2018, PROG NEUROBIOL, V169, P1, DOI 10.1016/j.pneurobio.2018.08.002

Chou VP, 2013, NEUROSCIENCE, V228, P73, DOI 10.1016/j.neuroscience.2012.10.009

Cumming P, 2018, J CEREBR BLOOD F MET, V38, P204, DOI 10.1177/0271678X17748786

Di Meco A, 2018, J ALZHEIMERS DIS, V62, P1337, DOI 10.3233/JAD-170700

Dzamko N, 2015, NEUROSCIENCE, V302, P89, DOI 10.1016/j.neuroscience.2014.10.028

Fang SH, 2006, NEUROSCIENCE, V140, P969, DOI 10.1016/j.neuroscience.2006.02.051

Fang SH, 2007, NEUROSCI LETT, V412, P78, DOI 10.1016/j.neulet.2006.10.065

Farias SE, 2007, J NEUROCHEM, V103, P1310, DOI 10.1111/j.1471-4159.2007.04830.x

Farmen K, 2021, P NATL ACAD SCI USA, V118, DOI 10.1073/pnas.2020858118

Fatima N., 2020, NEUROSURGERY, V67, DOI [10.1093/neuros/nyaa447_492, DOI 10.1093/NEUROS/NYAA447_492]

Fellner L, 2013, GLIA, V61, P349, DOI 10.1002/glia.22437

Fox SH, 2018, MOVEMENT DISORD, V33, P1248, DOI 10.1002/mds.27372

Gagliano SA, 2016, ANN CLIN TRANSL NEUR, V3, P924, DOI 10.1002/acn3.369

Gelosa P, 2017, MEDIAT INFLAMM, V2017, DOI 10.1155/2017/3454212

Ghosh A, 2016, CNS NEUROSCI THER, V22, P943, DOI 10.1111/cns.12596

Giri B, 2019, INT J MOL SCI, V20, DOI 10.3390/ijms20184559

Green HF, 2019, J PARKINSON DIS, V9, P705, DOI 10.3233/JPD-191699

Greenland JC, 2020, BMJ OPEN, V10, DOI 10.1136/bmjopen-2020-040527

Grinde B, 2021, INT J CLIN PHARM-NET, V43, P541, DOI 10.1007/s11096-020-01160-8

Haeggström JZ, 2011, CHEM REV, V111, P5866, DOI 10.1021/cr200246d

Hirsch EC, 2021, MOVEMENT DISORD, V36, P16, DOI 10.1002/mds.28075

Hirsch EC, 2009, LANCET NEUROL, V8, P382, DOI 10.1016/S1474-4422(09)70062-6

Ho MS, 2019, ADV EXP MED BIOL, V1175, P335, DOI 10.1007/978-981-13-9913-8_13

Trinh HKT, 2019, EXPERT REV RESP MED, V13, P1169, DOI 10.1080/17476348.2019.1670640

Jang H, 2017, NEUROREPORT, V28, P242, DOI 10.1097/WNR.0000000000000740

Jucaite A, 2015, BRAIN, V138, P2687, DOI 10.1093/brain/awv184

Kalia LV, 2015, LANCET, V386, P896, DOI 10.1016/S0140-6736(14)61393-3

Kang KH, 2013, NEUROPHARMACOLOGY, V73, P380, DOI 10.1016/j.neuropharm.2013.06.014

Kannarkat GT, 2013, J PARKINSON DIS, V3, P493, DOI 10.3233/JPD-130250

Kaur K, 2017, J NEUROL SCI, V381, P308, DOI 10.1016/j.jns.2017.08.3251

Kline EM, 2021, MOVEMENT DISORD, V36, P25, DOI 10.1002/mds.28411

Kouli A, 2019, BRAIN BEHAV IMMUN, V81, P41, DOI 10.1016/j.bbi.2019.06.042

Lai J, 2014, NEUROCHEM INT, V75, P26, DOI 10.1016/j.neuint.2014.05.006

Lee Y, 2019, ARCH PHARM RES, V42, P416, DOI 10.1007/s12272-019-01133-0

Liu CH, 2020, INT IMMUNOPHARMACOL, V78, DOI 10.1016/j.intimp.2019.105947

Lynch KR, 1999, NATURE, V399, P789

Malmstrom K, 1998, Am J Ther, V5, P189, DOI 10.1097/00045391-199805000-00010

Mansour RM, 2018, TOXICOL APPL PHARM, V358, P76, DOI 10.1016/j.taap.2018.09.012

Marschallinger J, 2020, NEUROTHERAPEUTICS, V17, P1061, DOI 10.1007/s13311-020-00836-3

Marschallinger J, 2015, NAT COMMUN, V6, DOI 10.1038/ncomms9466

Martino R, 2017, NEUROTOXICOLOGY, V61, P132, DOI 10.1016/j.neuro.2016.04.003

Michael J, 2019, DRUG DISCOV TODAY, V24, P505, DOI 10.1016/j.drudis.2018.09.008

Miller SJ, 2018, FRONT CELL NEUROSCI, V12, DOI 10.3389/fncel.2018.00401

Moran LB, 2008, NEUROGENETICS, V9, P1, DOI 10.1007/s10048-007-0116-y

Murray ME, 2014, ALZHEIMERS RES THER, V6, DOI 10.1186/alzrt231

Nagarajan VB, 2018, NEUROSCI LETT, V682, P100, DOI 10.1016/j.neulet.2018.05.052

O'Shea J.J., 2013, Clinical Immunology, V4th, P108

Offermanns S, 2006, TRENDS PHARMACOL SCI, V27, P384, DOI 10.1016/j.tips.2006.05.008

Peter I, 2018, JAMA NEUROL, V75, P939, DOI 10.1001/jamaneurol.2018.0605

Postuma RB, 2015, MOVEMENT DISORD, V30, P1591, DOI 10.1002/mds.26424

Racette BA, 2018, ANN CLIN TRANSL NEUR, V5, P870, DOI 10.1002/acn3.580

Rahman SO, 2019, EUR J PHARMACOL, V842, P208, DOI 10.1016/j.ejphar.2018.10.040

Ransohoff RM, 2011, NAT NEUROSCI, V14, P1098, DOI 10.1038/nn.2917

Rees K, 2011, COCHRANE DB SYST REV, DOI 10.1002/14651858.CD008454.pub2

Reiss TF, 1996, J ALLERGY CLIN IMMUN, V98, P528, DOI 10.1016/S0091-6749(96)70086-6

Rozin Spencer I, 2017, Open Neurol J, V11, P7, DOI 10.2174/1874205X01711010007

Saiki M, 2010, J NEUROL NEUROSUR PS, V81, P890, DOI 10.1136/jnnp.2008.162883

SAMUELSSON B, 1983, SCIENCE, V220, P568, DOI 10.1126/science.6301011

Sasaki F, 2019, INT IMMUNOL, V31, P607, DOI 10.1093/intimm/dxz044

Sherer TB, 2003, NEUROSCI LETT, V341, P87, DOI 10.1016/S0304-3940(03)00172-1

Sinha S, 2019, BIOORGAN MED CHEM, V27, P3745, DOI 10.1016/j.bmc.2019.06.040

Siracusa R, 2018, MOL NEUROBIOL, V55, P2403, DOI 10.1007/s12035-017-0496-4

Sommer A, 2018, CELL STEM CELL, V23, P123, DOI 10.1016/j.stem.2018.06.015

Stephenson J, 2018, IMMUNOLOGY, V154, P204, DOI 10.1111/imm.12922

Stokholm MG, 2017, LANCET NEUROL, V16, P789, DOI 10.1016/S1474-4422(17)30173-4

Stokholm MG, 2016, ANN NEUROL, V79, P940, DOI 10.1002/ana.24648

Svenningsson P, 2019, CURR OPIN NEUROL, V32, P597, DOI 10.1097/WCO.0000000000000707

Varnäs K, 2019, EUR J NUCL MED MOL I, V46, P367, DOI 10.1007/s00259-018-4161-6

Verkhratsky A, 2019, ADV EXP MED BIOL, V1175, P45, DOI 10.1007/978-981-13-9913-8_3

Verkhratsky A, 2019, ADV EXP MED BIOL, V1175, P149, DOI 10.1007/978-981-13-9913-8_7

Vivekanantham S, 2015, INT J NEUROSCI, V125, P717, DOI 10.3109/00207454.2014.982795

Wakade C, 2015, CLIN CASE REP, V3, P635, DOI 10.1002/ccr3.232

Wang Hao, 2013, Zhejiang Da Xue Xue Bao Yi Xue Ban, V42, P52

Wang YX, 2020, NEUROBIOL AGING, V87, P1, DOI 10.1016/j.neurobiolaging.2019.12.013

West PK, 2019, GLIA, V67, P1821, DOI 10.1002/glia.23634

Wijeyekoon RS, 2020, BRAIN BEHAV IMMUN, V87, P473, DOI 10.1016/j.bbi.2020.01.018

Witoelar A, 2017, JAMA NEUROL, V74, P780, DOI 10.1001/jamaneurol.2017.0469

Xilouri M, 2016, MOVEMENT DISORD, V31, P178, DOI 10.1002/mds.26477

Zhang DD, 2021, J NEUROINFLAMM, V18, DOI 10.1186/s12974-020-02065-z

Zhang P, 2019, NEUROL SCI, V40, P113, DOI 10.1007/s10072-018-3592-y

Zhang XY, 2014, BRAIN RES, V1572, P59, DOI 10.1016/j.brainres.2014.05.026

Zhang YS, 2018, EUR J PHARMACOL, V819, P30, DOI 10.1016/j.ejphar.2017.11.041

Zhao CZ, 2011, NEUROSCIENCE, V189, P1, DOI 10.1016/j.neuroscience.2011.05.066

Ziegler-Heitbrock L, 2015, FRONT IMMUNOL, V6, DOI 10.3389/fimmu.2015.00423

NR 98

TC 15

Z9 15

U1 1

U2 13

PU MDPI

PI BASEL

PA ST ALBAN-ANLAGE 66, CH-4052 BASEL, SWITZERLAND

EI 1422-0067

J9 INT J MOL SCI

JI Int. J. Mol. Sci.

PD JUN

PY 2021

VL 22

IS 11

AR 5606

DI 10.3390/ijms22115606

PG 13

WC Biochemistry & Molecular Biology; Chemistry, Multidisciplinary

WE Science Citation Index Expanded (SCI-EXPANDED)

SC Biochemistry & Molecular Biology; Chemistry

GA SQ2KJ

UT WOS:000660186300001

PM 34070609

OA Green Published, gold

DA 2024-08-12

ER

PT J

AU Mao, D

Zheng, Y

Xu, FF

Han, X

Zhao, HY

AF Mao, Di

Zheng, Yuan

Xu, Fenfen

Han, Xiao

Zhao, Hongyang

TI HMGB1 in nervous system diseases: A common biomarker and potential

therapeutic target

SO FRONTIERS IN NEUROLOGY

LA English

DT Review

DE HMGB1; biomarker; nervous system diseases; neuroinflammation;

therapeutic target

ID GROUP BOX 1; MOBILITY GROUP BOX-1; AUTISM SPECTRUM DISORDERS; NLRP3

INFLAMMASOME; DEPRESSIVE BEHAVIOR; KYNURENINE PATHWAY; GLYCYRRHIZIC

ACID; OXIDATIVE STRESS; PLASMA-LEVELS; RECEPTOR 4

AB High-mobility group box-1 (HMGB1) is a nuclear protein associated with early inflammatory changes upon extracellular secretion expressed in various cells, including neurons and microglia. With the progress of research, neuroinflammation is believed to be involved in the pathogenesis of neurological diseases such as Parkinson's, epilepsy, and autism. As a key promoter of neuroinflammation, HMGB1 is thought to be involved in the pathogenesis of Parkinson's disease, stroke, traumatic brain injury, epilepsy, autism, depression, multiple sclerosis, and amyotrophic lateral sclerosis. However, in the clinic, HMGB1 has not been described as a biomarker for the above-mentioned diseases. However, the current preclinical research results show that HMGB1 antagonists have positive significance in the treatment of Parkinson's disease, stroke, traumatic brain injury, epilepsy, and other diseases. This review discusses the possible mechanisms by which HMGB1 mediates Parkinson's disease, stroke, traumatic brain injury, epilepsy, autism, depression, multiple sclerosis, amyotrophic lateral sclerosis, and the potential of HMGB1 as a biomarker for these diseases. Future research needs to further explore the underlying molecular mechanisms and clinical translation.

C1 [Mao, Di] Shandong Univ, Jinan Cent Hosp, Dept Pediat, Jinan, China.

[Zheng, Yuan; Xu, Fenfen; Han, Xiao; Zhao, Hongyang] Shandong First Med Univ, Dept Pediat, Cent Hosp, Jinan, China.

C3 Shandong First Medical University & Shandong Academy of Medical

Sciences; Shandong University; Shandong First Medical University &

Shandong Academy of Medical Sciences

RP Zhao, HY (corresponding author), Shandong First Med Univ, Dept Pediat, Cent Hosp, Jinan, China.

EM drzhaohongyang@163.com

CR Anderson G, 2019, INT J MOL SCI, V20, DOI 10.3390/ijms20215500

Andersson Å, 2008, J LEUKOCYTE BIOL, V84, P1248, DOI 10.1189/jlb.1207844

Andersson U, 2018, SEMIN IMMUNOL, V38, P40, DOI 10.1016/j.smim.2018.02.011

Au AK, 2012, J NEUROTRAUM, V29, P2013, DOI 10.1089/neu.2011.2171

Babinská K, 2014, PHYSIOL RES, V63, pS613, DOI 10.33549/physiolres.932932

Banasr M, 2008, BIOL PSYCHIAT, V64, P863, DOI 10.1016/j.biopsych.2008.06.008

Baran A, 2019, NEUROL SCI, V40, P167, DOI 10.1007/s10072-018-3611-z

Barbosa IG, 2015, J NEUROIMMUNOL, V278, P69, DOI 10.1016/j.jneuroim.2014.11.021

Becker Katrin Anne, 2017, Neurosignals, V25, P88, DOI 10.1159/000484621

Bian HT, 2022, NEUROTOX RES, V40, P1393, DOI 10.1007/s12640-022-00553-z

Brambilla L, 2018, FRONT NEUROSCI-SWITZ, V12, DOI 10.3389/fnins.2018.00622

Bucova M, 2020, NEUROL SCI, V41, P599, DOI 10.1007/s10072-019-04136-3

Bustin M, 2001, TRENDS BIOCHEM SCI, V26, P152, DOI 10.1016/S0968-0004(00)01777-1

Cao ZY, 2020, J AFFECT DISORDERS, V265, P247, DOI 10.1016/j.jad.2020.01.048

Chatterjee M, 2020, BIOMOLECULES, V10, DOI 10.3390/biom10081177

Chen HS, 2020, TRANSL STROKE RES, V11, P967, DOI 10.1007/s12975-019-00772-1

Chen XR, 2018, J NEUROINFLAMM, V15, DOI [10.1186/s12974-018-1151-3, 10.1186/s12974-017-0917-3]

Chu YJ, 2021, J NEUROIMMUNOL, V352, DOI 10.1016/j.jneuroim.2021.577480

Claudia C, 2019, DIGEST LIVER DIS, V51, P1366, DOI 10.1016/j.dld.2019.06.006

Constantinescu CS, 2011, BRIT J PHARMACOL, V164, P1079, DOI 10.1111/j.1476-5381.2011.01302.x

Dipasquale V, 2017, INT J MOL CELL MED, V6, P148, DOI 10.22088/acadpub.BUMS.6.3.148

Emanuele E, 2010, PROG NEURO-PSYCHOPH, V34, P681, DOI 10.1016/j.pnpbp.2010.03.020

Fan CQ, 2018, FRONT IMMUNOL, V9, DOI 10.3389/fimmu.2018.02889

Frank MG, 2015, BRAIN BEHAV IMMUN, V48, P1, DOI 10.1016/j.bbi.2015.03.010

Franklin TC, 2018, BIOL PSYCHIAT, V83, P50, DOI 10.1016/j.biopsych.2017.06.034

Gan P, 2020, FRONT PHARMACOL, V11, DOI 10.3389/fphar.2020.00776

Gao HM, 2011, J NEUROSCI, V31, P1081, DOI 10.1523/JNEUROSCI.3732-10.2011

Gao TL, 2012, J TRAUMA ACUTE CARE, V72, P643, DOI 10.1097/TA.0b013e31823c54a6

Gao TL, 2018, BIOCHEM BIOPH RES CO, V497, P430, DOI 10.1016/j.bbrc.2018.02.102

Ghosh D, 2022, IMMUNOL RES, V70, P633, DOI 10.1007/s12026-022-09295-8

González-Reyes S, 2016, EPILEPSY RES, V126, P126, DOI 10.1016/j.eplepsyres.2016.07.007

GOODWIN GH, 1973, EUR J BIOCHEM, V38, P14, DOI 10.1111/j.1432-1033.1973.tb03026.x

Grad LI, 2017, CSH PERSPECT MED, V7, DOI 10.1101/cshperspect.a024117

Halder SK, 2018, J PHARMACOL EXP THER, V365, P27, DOI 10.1124/jpet.117.245340

Harms AS, 2018, EXP NEUROL, V300, P179, DOI 10.1016/j.expneurol.2017.11.010

Hisaoka-Nakashima K, 2019, PROG NEURO-PSYCHOPH, V92, P347, DOI 10.1016/j.pnpbp.2019.02.005

Huang QJ, 2020, CELL MOL BIOL, V66, P39, DOI 10.14715/cmb/2020.66.3.6

Huang Y, 2021, NEUROSCI BIOBEHAV R, V122, P18, DOI 10.1016/j.neubiorev.2020.12.028

Husarova VM, 2016, PSYCHIAT INVEST, V13, P174, DOI 10.4306/pi.2016.13.2.174

Hwang CS, 2013, NEUROBIOL DIS, V58, P13, DOI 10.1016/j.nbd.2013.04.013

Iori V, 2013, NEUROBIOL DIS, V58, P102, DOI 10.1016/j.nbd.2013.03.006

Kan MC, 2019, BRAZ J MED BIOL RES, V52, DOI [10.1590/1414-431X20197374, 10.1590/1414-431x20197374]

Karim MR, 2020, MOL NEURODEGENER, V15, DOI 10.1186/s13024-020-00364-w

Kikuchi K, 2009, J PHARMACOL EXP THER, V329, P865, DOI 10.1124/jpet.108.149484

Kikuchi K, 2009, BIOCHEM BIOPH RES CO, V385, P132, DOI 10.1016/j.bbrc.2009.04.041

Kim ID, 2018, CELL DEATH DIS, V9, DOI 10.1038/s41419-018-0438-8

Kim JB, 2006, J NEUROSCI, V26, P6413, DOI 10.1523/JNEUROSCI.3815-05.2006

Kim SJ, 2019, LAB INVEST, V99, P1389, DOI 10.1038/s41374-019-0254-5

Le K, 2018, NEUROL RES, V40, P907, DOI 10.1080/01616412.2018.1497254

Lee JY, 2015, J NEUROINFLAMM, V12, DOI 10.1186/s12974-015-0310-z

Lee JD, 2020, MOL NEUROBIOL, V57, P4143, DOI 10.1007/s12035-020-02019-9

Lee JD, 2019, J NEUROINFLAMM, V16, DOI 10.1186/s12974-019-1435-2

Lei C, 2022, NEUROSCIENCE, V481, P238, DOI 10.1016/j.neuroscience.2021.11.041

Lei C, 2013, NEUROSCIENCE, V228, P190, DOI 10.1016/j.neuroscience.2012.10.023

Li YJ, 2019, J PHARM PHARMACOL, V71, P390, DOI 10.1111/jphp.13040

Lian YJ, 2017, BRAIN BEHAV IMMUN, V59, P322, DOI 10.1016/j.bbi.2016.09.017

Lindersson EK, 2004, NEUROREPORT, V15, P2735

Liu K, 2007, FASEB J, V21, P3904, DOI 10.1096/fj.07-8770com

Liu L, 2019, MOLECULES, V24, DOI 10.3390/molecules24020326

Lo Coco D, 2007, NEUROSCI LETT, V412, P73, DOI 10.1016/j.neulet.2006.10.063

Maas AIR, 2017, LANCET NEUROL, V16, P987, DOI 10.1016/S1474-4422(17)30371-X

Maddison DC, 2015, SEMIN CELL DEV BIOL, V40, P134, DOI 10.1016/j.semcdb.2015.03.002

Makris G, 2021, CHILDREN-BASEL, V8, DOI 10.3390/children8060478

Malhotra S, 2015, J NEUROINFLAMM, V12, DOI 10.1186/s12974-015-0269-9

Manivannan S, 2020, BRAIN SCI, V10, DOI 10.3390/brainsci10100760

Maroso M, 2010, NAT MED, V16, P413, DOI 10.1038/nm.2127

Morimoto M, 2022, J AM HEART ASSOC, V11, DOI 10.1161/JAHA.121.024424

Myint AM, 2014, PROG NEURO-PSYCHOPH, V48, P304, DOI 10.1016/j.pnpbp.2013.08.008

Nadeem A, 2014, PULM PHARMACOL THER, V29, P31, DOI 10.1016/j.pupt.2014.06.001

Nadeem A, 2017, BRAIN BEHAV IMMUN, V61, P146, DOI 10.1016/j.bbi.2016.12.024

Nazari S, 2017, PHYTOTHER RES, V31, P1635, DOI 10.1002/ptr.5893

Nicaise AM, 2019, P NATL ACAD SCI USA, V116, P9030, DOI 10.1073/pnas.1818348116

Nishibori M, 2020, CELLS-BASEL, V9, DOI 10.3390/cells9122650

Nishibori M, 2019, J PHARMACOL SCI, V140, P94, DOI 10.1016/j.jphs.2019.04.006

Novellino F, 2020, INT J MOL SCI, V21, DOI 10.3390/ijms21031115

OKUDA Y, 1995, J NEUROIMMUNOL, V62, P103, DOI 10.1016/0165-5728(95)00114-H

Okuma Y, 2019, WORLD NEUROSURG, V122, pE864, DOI 10.1016/j.wneu.2018.10.164

Pang HG, 2016, MEDIAT INFLAMM, V2016, DOI 10.1155/2016/4569521

Paudel YN, 2021, ACS CHEM NEUROSCI, V12, P3288, DOI 10.1021/acschemneuro.0c00825

Paudel YN, 2020, INT J MOL SCI, V21, DOI 10.3390/ijms21134609

Paudel YN, 2020, PHARMACOL RES, V156, DOI 10.1016/j.phrs.2020.104792

Paudel YN, 2020, ACS CHEM NEUROSCI, V11, P485, DOI 10.1021/acschemneuro.9b00640

Paudel YN, 2018, FRONT NEUROSCI-SWITZ, V12, DOI 10.3389/fnins.2018.00628

Pauletti A, 2019, BRAIN, V142, pE38, DOI 10.1093/brain/awz128

Pedrazzi M, 2006, J NEUROCHEM, V99, P827, DOI 10.1111/j.1471-4159.2006.04120.x

Qiu J, 2008, J CEREBR BLOOD F MET, V28, P927, DOI 10.1038/sj.jcbfm.9600582

Qu YR, 2018, VIROLOGY, V525, P19, DOI 10.1016/j.virol.2018.09.001

Rana T, 2021, PHARMACOL REP, V73, P31, DOI 10.1007/s43440-020-00163-6

Ravizza Teresa, 2018, Epilepsia Open, V3, P133, DOI 10.1002/epi4.12242

Ren QY, 2022, J ETHNOPHARMACOL, V292, DOI 10.1016/j.jep.2022.115234

Riazi K, 2010, EPILEPSY RES, V89, P34, DOI 10.1016/j.eplepsyres.2009.09.004

Richard SA, 2017, BIOSCIENCE REP, V37, DOI 10.1042/BSR20171104

Rouillard ME, 2022, FRONT CELL NEUROSCI, V16, DOI 10.3389/fncel.2022.833186

Russo AJ, 2014, J CENT NERV SYST DIS, V6, P79, DOI [10.4137/JCNSNSD.S13767, 10.4137/JCNSD.S13767]

Russo AJ, 2013, BIOMARK INSIGHTS, V8, P35, DOI 10.4137/BMI.S11270

Safar MM, 2021, LIFE SCI, V287, DOI 10.1016/j.lfs.2021.120132

Santoro M, 2016, NEUROBIOL DIS, V91, P59, DOI 10.1016/j.nbd.2016.02.018

Sappington PL, 2002, GASTROENTEROLOGY, V123, P790, DOI 10.1053/gast.2002.35391

Sasaki T, 2016, EXP NEUROL, V275, P220, DOI 10.1016/j.expneurol.2015.11.003

Scharfman HE, 2012, EPILEPSY CURR, V12, P178, DOI 10.5698/1535-7511-12.5.178

Scheiblich H, 2017, J NEUROCHEM, V143, P534, DOI 10.1111/jnc.14225

Seo MS, 2019, J CLIN MED, V8, DOI 10.3390/jcm8050629

Shi YB, 2018, MOL MED REP, V17, P5125, DOI 10.3892/mmr.2018.8485

Singh V, 2016, ANTIOXID REDOX SIGN, V24, P635, DOI 10.1089/ars.2015.6397

Souery D, 2006, J CLIN PSYCHIAT, V67, P16

Sternberg Z, 2016, IMMUNOL RES, V64, P385, DOI 10.1007/s12026-015-8673-x

Su XF, 2011, MEDIAT INFLAMM, V2011, DOI 10.1155/2011/807142

Sun Y, 2018, FRONT IMMUNOL, V9, DOI 10.3389/fimmu.2018.01518

Tan SW, 2021, J NEUROINFLAMM, V18, DOI 10.1186/s12974-021-02274-0

Tang DL, 2010, J CELL BIOL, V190, P881, DOI 10.1083/jcb.200911078

Terrone G, 2020, NEUROPHARMACOLOGY, V167, DOI 10.1016/j.neuropharm.2019.107742

Tian XD, 2017, CURR DRUG DELIV, V14, P785, DOI 10.2174/1567201813666160808111933

Tian Y, 2020, CLIN SCI, V134, P2075, DOI 10.1042/CS20200553

Uzawa A, 2013, CLIN EXP IMMUNOL, V172, P37, DOI 10.1111/cei.12036

Wang B, 2020, BRAIN BEHAV IMMUN, V88, P132, DOI 10.1016/j.bbi.2020.06.019

Wang B, 2019, MOL MED REP, V20, P359, DOI 10.3892/mmr.2019.10225

Wang B, 2018, BEHAV BRAIN RES, V353, P250, DOI 10.1016/j.bbr.2018.01.024

Wang B, 2018, BRAIN BEHAV IMMUN, V72, P51, DOI 10.1016/j.bbi.2017.11.017

Wang HC, 1999, SCIENCE, V285, P248, DOI 10.1126/science.285.5425.248

Wang KH, 2016, DIAGN PATHOL, V11, DOI 10.1186/s13000-016-0459-5

Wang KY, 2012, CLIN CHIM ACTA, V413, P1737, DOI 10.1016/j.cca.2012.07.002

Wang XY, 2020, TRANSL NEUROSCI, V11, P227, DOI 10.1515/tnsci-2020-0129

Webster KM, 2019, EXP NEUROL, V320, DOI 10.1016/j.expneurol.2019.112979

Webster KM, 2019, J COMP NEUROL, V527, P1102, DOI 10.1002/cne.24589

Woodruff TM, 2011, MOL NEURODEGENER, V6, DOI 10.1186/1750-1326-6-11

Xiao YF, 2021, FRONT IMMUNOL, V12, DOI 10.3389/fimmu.2021.584097

Xie JF, 2021, BRAIN BEHAV IMMUN, V92, P67, DOI 10.1016/j.bbi.2020.11.029

Xu X, 2020, BRIT J PHARMACOL, V177, P5224, DOI 10.1111/bph.15261

Xue JM, 2021, J CELL PHYSIOL, V236, P3406, DOI 10.1002/jcp.30125

Yang F, 2020, BIOCHEM BIOPH RES CO, V526, P553, DOI 10.1016/j.bbrc.2020.02.149

Yang LJ, 2018, CELL PHYSIOL BIOCHEM, V46, P2532, DOI 10.1159/000489659

Ying Cui, 2020, Acta Histochem, V122, P151485, DOI 10.1016/j.acthis.2019.151485

Zhang HF, 2019, GEN PSYCHIAT, V32, DOI 10.1136/gpsych-2019-100084

Zhang JY, 2011, STROKE, V42, P1420, DOI 10.1161/STROKEAHA.110.598334

Zhang K, 2019, NANO LETT, V19, P2812, DOI 10.1021/acs.nanolett.8b04729

Zhen C, 2019, MULT SCLER RELAT DIS, V31, P87, DOI 10.1016/j.msard.2019.03.030

Zhu JR, 2018, ACTA PHARMACOL SIN, V39, P1706, DOI 10.1038/s41401-018-0160-1

Zhu M, 2018, J CHILD NEUROL, V33, P909, DOI 10.1177/0883073818801654

NR 138

TC 15

Z9 16

U1 1

U2 12

PU FRONTIERS MEDIA SA

PI LAUSANNE

PA AVENUE DU TRIBUNAL FEDERAL 34, LAUSANNE, CH-1015, SWITZERLAND

SN 1664-2295

J9 FRONT NEUROL

JI Front. Neurol.

PD OCT 31

PY 2022

VL 13

AR 1029891

DI 10.3389/fneur.2022.1029891

PG 15

WC Clinical Neurology; Neurosciences

WE Science Citation Index Expanded (SCI-EXPANDED)

SC Neurosciences & Neurology

GA 6G9CD

UT WOS:000885047600001

PM 36388178

OA Green Published, gold

DA 2024-08-12

ER

PT J

AU Zager, A

AF Zager, Adriano

TI Modulating the immune response with the wake-promoting drug modafinil: A

potential therapeutic approach for inflammatory disorders

SO BRAIN BEHAVIOR AND IMMUNITY

LA English

DT Review

DE Modafinil; Immunity; Inflammation; Dopamine; Microglia; T cells;

Macrophages

ID CONDITIONED PLACE PREFERENCE; NIGROSTRIATAL DOPAMINE NEURONS;

DOUBLE-BLIND; IN-VITRO; PREFRONTAL CORTEX; RAT MODEL; COCAINE;

TRANSPORTERS; AROUSAL; VIVO

AB Modafinil is a psychostimulant drug approved by the FDA primarily for the treatment of sleep disorders such as narcolepsy, excessive daytime sleepiness and sleep apnea. Several documented but not yet approved uses for modafinil have been described over the last 30 years, including alleviating fatigue in neurological and neuro-degenerative disorders. Recent evidence has suggested that modafinil may have an immunomodulatory effect. Here, we review the different effects of modafinil treatment in animal models of brain inflammation and peripheral immune function. We conclude that there is unequivocal evidence of an anti-inflammatory effect of modafinil in experimental animal models of brain inflammation and neurodegenerative disorders, including systemic inflammation and methamphetamine-induced neuroinflammation, Parkinson's disease, brain ischemia, and multiple sclerosis. Modafinil acts on resident glial cells and infiltrating immune cells, negatively affecting both innate and adaptive immune responses in the brain. We also review the outcomes of modafinil treatment on peripheral immune function. The results of studies on this subject are still controversial and far from conclusive, but point to a new avenue of research in relation to peripheral inflammation. The data reviewed here raise the possibility of modafinil being used as adjuvant treatment for neurological disorders in which inflammation plays an important role.

C1 [Zager, Adriano] Linkoping Univ, Fac Med & Hlth Sci, Dept Biomed & Clin Sci, Div Neurobiol, Linkoping, Sweden.

C3 Linkoping University

RP Zager, A (corresponding author), Linkoping Univ, Fac Med & Hlth Sci, Dept Biomed & Clin Sci, Div Neurobiol, Linkoping, Sweden.

EM adrianozager@hotmail.com

CR Abbasi Y, 2019, METAB BRAIN DIS, V34, P805, DOI 10.1007/s11011-018-0378-0

Aguirre JA, 1999, NEUROSCI LETT, V275, P215, DOI 10.1016/S0304-3940(99)00706-5

Andersen ML, 2010, PSYCHOPHARMACOLOGY, V210, P439, DOI 10.1007/s00213-010-1839-2

[Anonymous], 1993, EXP BRAIN RES

[Anonymous], 2017, NEUROPHARMACOLOGY, DOI DOI 10.1016/J.NEUROPHARM.2017.04.037

Antonelli T, 1998, NEUROREPORT, V9, P4209, DOI 10.1097/00001756-199812210-00038

Ballon JS, 2006, J CLIN PSYCHIAT, V67, P554, DOI 10.4088/JCP.v67n0406

BASTUJI H, 1988, PROG NEURO-PSYCHOPH, V12, P695, DOI 10.1016/0278-5846(88)90014-0

Battleday RM, 2015, EUR NEUROPSYCHOPHARM, V25, P1865, DOI 10.1016/j.euroneuro.2015.07.028

Beck P, 2008, SLEEP, V31, P1647, DOI 10.1093/sleep/31.12.1647

Ben-Shaanan TL, 2018, NAT COMMUN, V9, DOI 10.1038/s41467-018-05283-5

Ben-Shaanan TL, 2016, NAT MED, V22, P940, DOI 10.1038/nm.4133

Bernardi RE, 2015, DRUG ALCOHOL DEPEN, V155, P37, DOI 10.1016/j.drugalcdep.2015.08.016

Bonsall DR, 2015, PLOS ONE, V10, DOI 10.1371/journal.pone.0140678

Boutrel B, 2004, SLEEP, V27, P1181, DOI 10.1093/sleep/27.6.1181

Brandao WN, 2019, INT IMMUNOPHARMACOL, V75, DOI 10.1016/j.intimp.2019.105809

Brandt MD, 2014, NEUROSCIENCE, V278, P267, DOI 10.1016/j.neuroscience.2014.08.014

Buttarelli FR, 2011, CURR NEUROPHARMACOL, V9, P278, DOI 10.2174/157015911795596612

Cao JJ, 2011, ACS MED CHEM LETT, V2, P48, DOI 10.1021/ml1002025

Castellani G, 2019, BRAIN BEHAV IMMUN, V81, P138, DOI 10.1016/j.bbi.2019.06.006

Chen CR, 2013, PLOS ONE, V8, DOI 10.1371/journal.pone.0076102

Ciampoli M, 2017, SCI REP-UK, V7, DOI 10.1038/s41598-017-10112-8

Dackis CA, 2003, DRUG ALCOHOL DEPEN, V70, P29, DOI 10.1016/S0376-8716(02)00335-6

Dejban P, 2020, PHARMACOL REP, V72, P135, DOI 10.1007/s43440-019-00054-5

Deroche-Gamonet V, 2002, PSYCHOPHARMACOLOGY, V161, P387, DOI 10.1007/s00213-002-1080-8

Duchêne A, 2016, SLEEP, V39, P1283, DOI 10.5665/sleep.5854

Frakey LL, 2012, J CLIN PSYCHIAT, V73, P796, DOI 10.4088/JCP.10m06708

FUXE K, 1992, EXP BRAIN RES, V88, P117, DOI 10.1007/BF02259133

Gallopin T, 2004, SLEEP, V27, P19

Garcia-Rill E, 2007, SLEEP, V30, P1405, DOI 10.1093/sleep/30.11.1405

Gaskill PJ, 2012, J NEUROINFLAMM, V9, DOI 10.1186/1742-2094-9-203

Ghosh MC, 2003, INT IMMUNOPHARMACOL, V3, P1019, DOI 10.1016/S1567-5769(03)00100-0

Glass CK, 2010, CELL, V140, P918, DOI 10.1016/j.cell.2010.02.016

Gonzalez B., 2018, Prog. Neuro- Psychopharmacol. Biol. Psychiatry

González B, 2019, PROG NEURO-PSYCHOPH, V88, P222, DOI 10.1016/j.pnpbp.2018.07.019

González B, 2014, NEUROPHARMACOLOGY, V87, P188, DOI 10.1016/j.neuropharm.2014.02.002

Gopinath A, 2020, J IMMUNOL METHODS, V476, DOI 10.1016/j.jim.2019.112686

Han JX, 2018, INFLAMMOPHARMACOLOGY, V26, P385, DOI 10.1007/s10787-017-0387-3

Hart CL, 2008, NEUROPSYCHOPHARMACOL, V33, P761, DOI 10.1038/sj.npp.1301472

Hei SY, 2018, MULT SCLER RELAT DIS, V19, P85, DOI 10.1016/j.msard.2017.10.011

Hilaire ZD, 2001, NEUROREPORT, V12, P3533, DOI 10.1097/00001756-200111160-00032

Huang Y, 2016, NEUROENDOCRINOL LETT, V37, P491

Jiang W, 2016, SPINE, V41, pE330, DOI 10.1097/BRS.0000000000001287

Jung JC, 2012, MOLECULES, V17, P10446, DOI 10.3390/molecules170910446

Kuan YC, 2016, CLIN THER, V38, P874, DOI 10.1016/j.clinthera.2016.02.004

Kumar R, 2008, DRUGS, V68, P1803, DOI 10.2165/00003495-200868130-00003

LACOSTA S, 1994, BRAIN RES, V664, P225, DOI 10.1016/0006-8993(94)91976-3

Levite M, 2017, J NEURAL TRANSM, V124, P525, DOI 10.1007/s00702-016-1640-4

Liu XH, 2013, NEUROPHARMACOLOGY, V75, P533, DOI 10.1016/j.neuropharm.2013.04.019

Loland CJ, 2012, BIOL PSYCHIAT, V72, P405, DOI 10.1016/j.biopsych.2012.03.022

Mackie P, 2018, BRAIN BEHAV IMMUN, V70, P21, DOI 10.1016/j.bbi.2018.03.020

Madras BK, 2006, J PHARMACOL EXP THER, V319, P561, DOI 10.1124/jpet.106.106583

Malcolm R, 2006, AM J DRUG ALCOHOL AB, V32, P577, DOI 10.1080/00952990600920425

Mereu M., 2013, PSYCHOPHARMACOLOGY B

Mereu M, 2017, EUR J NEUROSCI, V45, P167, DOI 10.1111/ejn.13376

Minzenberg MJ, 2008, NEUROPSYCHOPHARMACOL, V33, P1477, DOI 10.1038/sj.npp.1301534

Murillo-Rodríguez E, 2007, BEHAV BRAIN RES, V176, P353, DOI 10.1016/j.bbr.2006.10.016

Pereira MR, 2016, J CELL PHYSIOL, V231, P1862, DOI 10.1002/jcp.25307

Qu WM, 2008, J NEUROSCI, V28, P8462, DOI 10.1523/JNEUROSCI.1819-08.2008

Quisenberry AJ, 2013, EXP CLIN PSYCHOPHARM, V21, P252, DOI 10.1037/a0031832

Raineri M, 2012, PLOS ONE, V7, DOI 10.1371/journal.pone.0046599

Reardon C, 2016, IMMUNOL LETT, V178, P92, DOI 10.1016/j.imlet.2016.08.006

Saha B, 2001, NEUROIMMUNOMODULAT, V9, P23, DOI 10.1159/000049004

Sahu S, 2013, EXP NEUROL, V248, P470, DOI 10.1016/j.expneurol.2013.07.021

Shuman T, 2012, BEHAV BRAIN RES, V235, P105, DOI 10.1016/j.bbr.2012.07.039

Soeiro AD, 2012, BEHAV BRAIN RES, V233, P367, DOI 10.1016/j.bbr.2012.05.023

Stone EA, 2002, SYNAPSE, V46, P269, DOI 10.1002/syn.10127

Szabo ST, 2019, SLEEP MED REV, V43, P23, DOI 10.1016/j.smrv.2018.09.006

UEKI A, 1993, EXP BRAIN RES, V96, P89, DOI 10.1007/BF00230442

Urbano FJ, 2007, P NATL ACAD SCI USA, V104, P12554, DOI 10.1073/pnas.0705087104

Urbano FJ, 2017, BEHAV BRAIN RES, V333, P276, DOI 10.1016/j.bbr.2017.07.013

Verrico CD, 2014, DRUG ALCOHOL DEPEN, V141, P72, DOI 10.1016/j.drugalcdep.2014.05.008

Volkow ND, 2009, JAMA-J AM MED ASSOC, V301, P1148, DOI 10.1001/jama.2009.351

Wadhwa M, 2018, FRONT CELL NEUROSCI, V12, DOI 10.3389/fncel.2018.00049

Wisor JP, 2001, J NEUROSCI, V21, P1787, DOI 10.1523/JNEUROSCI.21-05-01787.2001

Wuo-Silva R, 2019, FRONT PHARMACOL, V10, DOI 10.3389/fphar.2019.00211

Wuo-Silva R, 2016, FRONT PHARMACOL, V7, DOI 10.3389/fphar.2016.00420

Wuo-Silva R, 2011, ADDICT BIOL, V16, P565, DOI 10.1111/j.1369-1600.2011.00341.x

Xiao YL, 2004, ACTA PHARMACOL SIN, V25, P301

Yan YQ, 2015, CELL, V160, P62, DOI 10.1016/j.cell.2014.11.047

Yousefi-Manesh H., 2019, IMMUNOPHARMACOL IMMU, P1

Yousefi-Manesh H, 2019, EXP MOL PATHOL, V111, DOI 10.1016/j.yexmp.2019.104305

Zager A, 2018, PROG NEURO-PSYCHOPH, V81, P468, DOI 10.1016/j.pnpbp.2017.05.003

Zager A, 2018, PROG NEURO-PSYCHOPH, V81, P297, DOI 10.1016/j.pnpbp.2017.09.009

NR 84

TC 9

Z9 9

U1 0

U2 8

PU ACADEMIC PRESS INC ELSEVIER SCIENCE

PI SAN DIEGO

PA 525 B ST, STE 1900, SAN DIEGO, CA 92101-4495 USA

SN 0889-1591

EI 1090-2139

J9 BRAIN BEHAV IMMUN

JI Brain Behav. Immun.

PD AUG

PY 2020

VL 88

BP 878

EP 886

DI 10.1016/j.bbi.2020.04.038

PG 9

WC Immunology; Neurosciences; Psychiatry

WE Science Citation Index Expanded (SCI-EXPANDED)

SC Immunology; Neurosciences & Neurology; Psychiatry

GA MU5JL

UT WOS:000555709500099

PM 32311496

DA 2024-08-12

ER

PT J

AU Machado, V

Zöller, T

Attaai, A

Spittau, B

AF Machado, Venissa

Zoeller, Tanja

Attaai, Abdelraheim

Spittau, Bjoern

TI Microglia-Mediated Neuroinflammation and Neurotrophic Factor-Induced

Protection in the MPTP Mouse Model of Parkinson's Disease-Lessons from

Transgenic Mice

SO INTERNATIONAL JOURNAL OF MOLECULAR SCIENCES

LA English

DT Review

DE MPTP; neuroinflammation; microglia; Parkinson's disease; neurotrophic

factors; neuroprotection

ID COLONY-STIMULATING FACTOR; NIGROSTRIATAL DOPAMINERGIC SYSTEM;

GROWTH-FACTOR-BETA; FACTOR PROMOTES SURVIVAL; FACTOR G-CSF; TNF-ALPHA;

1-METHYL-4-PHENYL-1,2,3,6-TETRAHYDROPYRIDINE MPTP; DELIVERY PROTECTS;

MESSENGER-RNA; CDNF PROTECTS

AB Parkinson's disease (PD) is a neurodegenerative disease characterised by histopathological and biochemical manifestations such as loss of midbrain dopaminergic (DA) neurons and decrease in dopamine levels accompanied by a concomitant neuroinflammatory response in the affected brain regions. Over the past decades, the use of toxin-based animal models has been crucial to elucidate disease pathophysiology, and to develop therapeutic approaches aimed to alleviate its motor symptoms. Analyses of transgenic mice deficient for cytokines, chemokine as well as neurotrophic factors and their respective receptors in the 1-methyl-4-phenyl-1,2,3,6-tetrahydropyridine (MPTP) model of PD have broadened the current knowledge of neuroinflammation and neurotrophic support. Here, we provide a comprehensive review that summarises the contribution of microglia-mediated neuroinflammation in MPTP-induced neurodegeneration. Moreover, we highlight the contribution of neurotrophic factors as endogenous and/or exogenous molecules to slow the progression of midbrain dopaminergic (mDA) neurons and further discuss the potential of combined therapeutic approaches employing neuroinflammation modifying agents and neurotrophic factors.

C1 [Machado, Venissa; Zoeller, Tanja; Attaai, Abdelraheim; Spittau, Bjoern] Univ Freiburg, Dept Mol Embryol, Inst Anat & Cell Biol, Albertstr 17, D-79104 Freiburg, Germany.

[Machado, Venissa] Univ Freiburg, Spemann Grad Sch Biol & Med SGBM, Albertstr 19A, D-79104 Freiburg, Germany.

[Machado, Venissa; Zoeller, Tanja; Attaai, Abdelraheim] Univ Freiburg, Fac Biol, Schanzlestr 1, D-79104 Freiburg, Germany.

[Zoeller, Tanja] Univ Freiburg, Inst Anat & Cell Biol, Dept Neuroanat, Albertstr 17, D-79104 Freiburg, Germany.

[Attaai, Abdelraheim] Assiut Univ, Fac Vet Med, Dept Anat & Histol, Assiut 71526, Egypt.

C3 University of Freiburg; University of Freiburg; University of Freiburg;

University of Freiburg; Egyptian Knowledge Bank (EKB); Assiut University

RP Spittau, B (corresponding author), Univ Freiburg, Dept Mol Embryol, Inst Anat & Cell Biol, Albertstr 17, D-79104 Freiburg, Germany.

EM venissa.machado@sgbm.uni-freiburg.de;

tanja.zoeller@anat.uni-freiburg.de;

abdelraheim.attaai@anat.uni-freiburg.de;

bjoern.spittau@anat.uni-freiburg.de

RI Attaai, Abdelraheim/Q-4904-2018; Attaai, Abdelraheim/K-5073-2019;

Spittau, Björn/AGZ-8396-2022

OI Attaai, Abdelraheim/0000-0001-8481-2687; Attaai,

Abdelraheim/0000-0001-8481-2687; Spittau, Bjorn/0000-0001-5423-0571

FU Excellence Initiative of the German Research Foundation (GSC-4 Spemann

Graduate School); Intramural funding of the Medical Faculty of the

University of Freiburg (Forschungskommission); Egyptian Ministry of

Higher Education; Deutsche Forschungsgemeinschaft [SP 1555/2-1]

FX This study was supported by the Excellence Initiative of the German

Research Foundation (GSC-4 Spemann Graduate School, grant to Venissa

Machado), Intramural funding of the Medical Faculty of the University of

Freiburg (Forschungskommission, grant to Tanja Zoller), Egyptian

Ministry of Higher Education (grant to Abdelraheim Attaai) and grants

from the Deutsche Forschungsgemeinschaft (SP 1555/2-1 to Bjorn Spittau).

We apologise for studies that were not cited due to space constraints or

an oversight on our part.

CR Abdullah R, 2015, EXP GERONTOL, V68, P33, DOI 10.1016/j.exger.2014.09.014

Airavaara M, 2012, CELL TRANSPLANT, V21, P1213, DOI 10.3727/096368911X600948

Airavaara Mikko, 2012, Parkinsonism Relat Disord, V18 Suppl 1, pS143, DOI 10.1016/S1353-8020(11)70045-1

Alboni S, 2010, J NEUROINFLAMM, V7, DOI 10.1186/1742-2094-7-9

Allan SM, 2005, NAT REV IMMUNOL, V5, P629, DOI 10.1038/nri1664

Andrews ZB, 2006, NEUROBIOL DIS, V21, P568, DOI 10.1016/j.nbd.2005.09.001

Aron L, 2011, TRENDS NEUROSCI, V34, P88, DOI 10.1016/j.tins.2010.11.001

Barcia C, 2011, CELL DEATH DIS, V2, DOI 10.1038/cddis.2011.17

Barcia C, 2012, SCI REP-UK, V2, DOI 10.1038/srep00809

Baydyuk M, 2011, EXP NEUROL, V228, P118, DOI 10.1016/j.expneurol.2010.12.018

Berg D, 2014, MOVEMENT DISORD, V29, P454, DOI 10.1002/mds.25844

Bezard E, 1997, BRAIN RES, V766, P107, DOI 10.1016/S0006-8993(97)00531-3

Biju KC, 2013, NEUROSCI LETT, V535, P24, DOI 10.1016/j.neulet.2012.12.034

Biju KC, 2010, MOL THER, V18, P1536, DOI 10.1038/mt.2010.107

Block ML, 2007, BIOCHEM SOC T, V35, P1127, DOI 10.1042/BST0351127

Boger HA, 2008, BRAIN RES, V1241, P18, DOI 10.1016/j.brainres.2008.09.011

Bolin LM, 2002, J NEUROCHEM, V83, P167, DOI 10.1046/j.1471-4159.2002.01131.x

BOYCE S, 1984, BIOCHEM PHARMACOL, V33, P1747, DOI 10.1016/0006-2952(84)90344-7

Braak H, 2003, NEUROBIOL AGING, V24, P197, DOI 10.1016/S0197-4580(02)00065-9

Bradley JR, 2008, J PATHOL, V214, P149, DOI 10.1002/path.2287

Brochard V, 2009, J CLIN INVEST, V119, P182, DOI 10.1172/JCI36470

Buchman AS, 2012, ANN NEUROL, V71, P258, DOI 10.1002/ana.22588

Cao XQ, 2006, J NEUROCHEM, V99, P861, DOI 10.1111/j.1471-4159.2006.04125.x

Cardenas H, 2003, BRAIN RES, V985, P89, DOI 10.1016/S0006-8993(03)03172-X

Cardona AE, 2006, NAT NEUROSCI, V9, P917, DOI 10.1038/nn1715

CHADI G, 1993, EXP BRAIN RES, V97, P145

Chen LW, 2006, J COMP NEUROL, V497, P898, DOI 10.1002/cne.21014

Chen LW, 2004, NEUROSCIENCE, V126, P941, DOI 10.1016/j.neuroscience.2004.04.020

Cheng FC, 1998, NEUROSCI LETT, V252, P87, DOI 10.1016/S0304-3940(98)00554-0

CHIUEH CC, 1984, EUR J PHARMACOL, V100, P189, DOI 10.1016/0014-2999(84)90221-8

Choi DY, 2013, NEUROBIOL DIS, V49, P159, DOI 10.1016/j.nbd.2012.08.001

Ciesielska A, 2003, ACTA NEUROBIOL EXP, V63, P117, DOI 10.55782/ane-2003-1461

Codolo G, 2013, PLOS ONE, V8, DOI 10.1371/journal.pone.0055375

Czlonkowska A, 1996, NEURODEGENERATION, V5, P137, DOI 10.1006/neur.1996.0020

Date I, 1998, NEUROREPORT, V9, P2365, DOI 10.1097/00001756-199807130-00039

DATE I, 1990, BRAIN RES, V526, P156, DOI 10.1016/0006-8993(90)90264-C

Dauer W, 2003, NEURON, V39, P889, DOI 10.1016/S0896-6273(03)00568-3

DELZOMPO M, 1993, BRIT J PHARMACOL, V109, P411

Dinarello CA, 2007, EUR J IMMUNOL, V37, pS34, DOI 10.1002/eji.200737772

Ferger B, 2004, J NEUROCHEM, V89, P822, DOI 10.1111/j.1471-4159.2004.02399.x

de Yebenes JG, 2000, NEUROTOX RES, V2, P115, DOI 10.1007/BF03033789

GARCIA E, 1992, NEUROCHEM RES, V17, P979, DOI 10.1007/BF00966824

Gerecke KM, 2012, PLOS ONE, V7, DOI 10.1371/journal.pone.0043250

Goedert M, 2013, NAT REV NEUROL, V9, P13, DOI 10.1038/nrneurol.2012.242

Goldman JG, 2014, CURR OPIN NEUROL, V27, P434, DOI 10.1097/WCO.0000000000000112

Granholm AC, 2000, J NEUROSCI, V20, P3182, DOI 10.1523/JNEUROSCI.20-09-03182.2000

Gustin A, 2015, PLOS ONE, V10, DOI 10.1371/journal.pone.0130624

HADJICONSTANTINOU M, 1991, J NEUROCHEM, V57, P479, DOI 10.1111/j.1471-4159.1991.tb03776.x

HASEGAWA E, 1990, BIOCHEM BIOPH RES CO, V170, P1049, DOI 10.1016/0006-291X(90)90498-C

Hébert G, 2005, MOL BRAIN RES, V136, P267, DOI 10.1016/j.molbrainres.2005.01.009

Hegarty SV, 2014, NEURAL REGEN RES, V9, P1708, DOI 10.4103/1673-5374.143410

Hirsch EC, 2009, LANCET NEUROL, V8, P382, DOI 10.1016/S1474-4422(09)70062-6

HYMAN C, 1991, NATURE, V350, P230, DOI 10.1038/350230a0

JACKSONLEWIS V, 1995, NEURODEGENERATION, V4, P257, DOI 10.1016/1055-8330(95)90015-2

JAVITCH JA, 1985, P NATL ACAD SCI USA, V82, P2173, DOI 10.1073/pnas.82.7.2173

JAVITCH JA, 1984, EUR J PHARMACOL, V106, P455, DOI 10.1016/0014-2999(84)90740-4

Jellinger KA, 2012, MOVEMENT DISORD, V27, P8, DOI 10.1002/mds.23795

Kalkonde YV, 2007, BRAIN RES, V1128, P1, DOI 10.1016/j.brainres.2006.08.041

Kim NK, 2009, EUR J NEUROSCI, V29, P891, DOI 10.1111/j.1460-9568.2009.06653.x

KLAIDMAN LK, 1993, FREE RADICAL BIO MED, V15, P169, DOI 10.1016/0891-5849(93)90056-Z

Klevenyi P, 1999, NEUROREPORT, V10, P635, DOI 10.1097/00001756-199902250-00035

Kohutnicka M, 1998, IMMUNOPHARMACOLOGY, V39, P167, DOI 10.1016/S0162-3109(98)00022-8

Kosloski LM, 2013, J NEUROIMMUNOL, V265, P1, DOI 10.1016/j.jneuroim.2013.10.009

Kowsky S, 2007, P NATL ACAD SCI USA, V104, P20049, DOI 10.1073/pnas.0706177104

Krieglstein K, 1998, J NEUROSCI, V18, P9822

Krieglstein K, 1995, J NEURAL TRANSM-SUPP, P209

Kurkowska-Jastrzebska I, 1999, EXP NEUROL, V156, P50, DOI 10.1006/exnr.1998.6993

Lamkanfi M, 2012, ANNU REV CELL DEV BI, V28, P137, DOI 10.1146/annurev-cellbio-101011-155745

LANGSTON JW, 1983, SCIENCE, V219, P979, DOI 10.1126/science.6823561

Leng A, 2005, NEUROSCI LETT, V375, P107, DOI 10.1016/j.neulet.2004.10.077

LIN LFH, 1993, SCIENCE, V260, P1130, DOI 10.1126/science.8493557

Lindholm P, 2010, DEV NEUROBIOL, V70, P360, DOI 10.1002/dneu.20760

Lindholm P, 2007, NATURE, V448, P73, DOI 10.1038/nature05957

Lofrumento DD, 2011, NEUROIMMUNOMODULAT, V18, P79, DOI 10.1159/000320027

Luchtman DW, 2009, PHYSIOL BEHAV, V98, P130, DOI 10.1016/j.physbeh.2009.04.021

Martinon F, 2002, MOL CELL, V10, P417, DOI 10.1016/S1097-2765(02)00599-3

MAYER RA, 1986, J NEUROCHEM, V47, P1073

McGeer PL, 2008, MOVEMENT DISORD, V23, P474, DOI 10.1002/mds.21751

Meuer K, 2006, J NEUROCHEM, V97, P675, DOI 10.1111/j.1471-4159.2006.03727.x

MIZUNO Y, 1987, J NEUROCHEM, V48, P1787, DOI 10.1111/j.1471-4159.1987.tb05737.x

Morganti JM, 2012, J NEUROSCI, V32, P14592, DOI 10.1523/JNEUROSCI.0539-12.2012

Mount MP, 2007, J NEUROSCI, V27, P3328, DOI 10.1523/JNEUROSCI.5321-06.2007

Nadjar A, 2009, NEUROBIOL AGING, V30, P2021, DOI 10.1016/j.neurobiolaging.2008.02.009

NICKLAS WJ, 1987, LIFE SCI, V40, P721, DOI 10.1016/0024-3205(87)90299-2

NICKLAS WJ, 1985, LIFE SCI, V36, P2503, DOI 10.1016/0024-3205(85)90146-8

NIKKHAH G, 1993, EXP BRAIN RES, V92, P516

Oppenheim JJ, 2001, INT J HEMATOL, V74, P3, DOI 10.1007/BF02982543

OTHBERG A, 1995, EXP BRAIN RES, V105, P111

OTTO D, 1993, EUR J NEUROSCI, V5, P927, DOI 10.1111/j.1460-9568.1993.tb00943.x

OTTO D, 1994, GLIA, V11, P47, DOI 10.1002/glia.440110107

OTTO D, 1990, J NEUROSCI, V10, P1912

Owens T, 2014, GLIA, V62, P339, DOI 10.1002/glia.22608

Pattarini R, 2007, NEUROSCIENCE, V145, P654, DOI 10.1016/j.neuroscience.2006.12.030

Pestka S, 2007, J BIOL CHEM, V282, P20047, DOI 10.1074/jbc.R700004200

Pringsheim T, 2014, MOVEMENT DISORD, V29, P1583, DOI 10.1002/mds.25945

Przedborski S, 2001, CLIN NEUROSCI RES, V1, P407, DOI 10.1016/S1566-2772(01)00019-6

Przedborski S, 2001, J NEUROCHEM, V76, P1265, DOI 10.1046/j.1471-4159.2001.00183.x

Reynolds AD, 2007, J LEUKOCYTE BIOL, V82, P1083, DOI 10.1189/jlb.0507296

Root RK, 1999, J INFECT DIS, V179, pS342, DOI 10.1086/513857

ROSSETTI ZL, 1988, BIOCHEM PHARMACOL, V37, P4573, DOI 10.1016/0006-2952(88)90674-0

Rousselet E, 2002, EXP NEUROL, V177, P183, DOI 10.1006/exnr.2002.7960

Russo VC, 2005, ENDOCR REV, V26, P916, DOI 10.1210/er.2004-0024

Sánchez-Capelo A, 2003, MOL CELL NEUROSCI, V23, P614, DOI 10.1016/S1044-7431(03)00081-2

Schäbitz WR, 2003, STROKE, V34, P745, DOI 10.1161/01.STR.0000057814.70180.17

Schmidt N, 2001, J NEURAL TRANSM, V108, P1263, DOI 10.1007/s007020100004

Schneider A, 2005, J CLIN INVEST, V115, P2083, DOI 10.1172/JCI23559

Schober A, 2004, CELL TISSUE RES, V318, P215, DOI 10.1007/s00441-004-0938-y

Schober A, 1999, J NEUROSCI, V19, P2008

Schober A, 2007, NEUROBIOL DIS, V25, P378, DOI 10.1016/j.nbd.2006.10.005

Schroder K, 2010, CELL, V140, P821, DOI 10.1016/j.cell.2010.01.040

Sedelis M, 2000, NEUROREPORT, V11, P1093, DOI 10.1097/00001756-200004070-00037

Sedelis M, 2000, BEHAV GENET, V30, P171, DOI 10.1023/A:1001958023096

Skaper Stephen D, 2012, Methods Mol Biol, V846, P1, DOI 10.1007/978-1-61779-536-7_1

Song SJ, 2011, NEUROSCI LETT, V487, P153, DOI 10.1016/j.neulet.2010.10.012

Spillantini MG, 1997, NATURE, V388, P839, DOI 10.1038/42166

Sriram K, 2002, FASEB J, V16, P1474, DOI 10.1096/fj.02-0216fje

Sriram K, 2006, FASEB J, V20, P670, DOI 10.1096/fj.05-5106com

Sugama S, 2004, NEUROSCIENCE, V128, P451, DOI 10.1016/j.neuroscience.2004.07.020

Sulzer D, 2007, TRENDS NEUROSCI, V30, P244, DOI 10.1016/j.tins.2007.03.009

Tang Y, 2016, MOL NEUROBIOL, V53, P1181, DOI 10.1007/s12035-014-9070-5

Tatton NA, 1997, NEUROSCIENCE, V77, P1037, DOI 10.1016/S0306-4522(96)00545-3

TOMAC A, 1995, NATURE, V373, P335, DOI 10.1038/373335a0

Vila M, 2000, J NEUROCHEM, V74, P721, DOI 10.1046/j.1471-4159.2000.740721.x

Vroon A, 2007, EXP GERONTOL, V42, P762, DOI 10.1016/j.exger.2007.04.010

Walsh JG, 2014, NAT REV NEUROSCI, V15, P84, DOI 10.1038/nrn3638

Wang L, 2014, EXP NEUROL, V251, P22, DOI 10.1016/j.expneurol.2013.11.001

Weissmiller AM, 2012, TRANSL NEURODEGENER, V1, DOI 10.1186/2047-9158-1-14

Wirth SB, 1996, EXP NEUROL, V137, P191, DOI 10.1006/exnr.1996.0018

Wu DC, 2002, J NEUROSCI, V22, P1763, DOI 10.1523/JNEUROSCI.22-05-01763.2002

Yan YQ, 2015, CELL, V160, P62, DOI 10.1016/j.cell.2014.11.047

Yasuda Y, 2007, BRAIN RES, V1138, P196, DOI 10.1016/j.brainres.2006.12.054

Yasuda Y, 2008, J NEUROIMMUNOL, V204, P43, DOI 10.1016/j.jneuroim.2008.08.003

Zachrisson O, 2011, J PARKINSON DIS, V1, P49, DOI 10.3233/JPD-2011-0003

Zechel S, 2006, EUR J NEUROSCI, V23, P1671, DOI 10.1111/j.1460-9568.2006.04700.x

Zhao CH, 2007, NEUROBIOL DIS, V26, P36, DOI 10.1016/j.nbd.2006.11.012

NR 135

TC 53

Z9 60

U1 0

U2 15

PU MDPI

PI BASEL

PA ST ALBAN-ANLAGE 66, CH-4052 BASEL, SWITZERLAND

EI 1422-0067

J9 INT J MOL SCI

JI Int. J. Mol. Sci.

PD FEB

PY 2016

VL 17

IS 2

AR 151

DI 10.3390/ijms17020151

PG 24

WC Biochemistry & Molecular Biology; Chemistry, Multidisciplinary

WE Science Citation Index Expanded (SCI-EXPANDED)

SC Biochemistry & Molecular Biology; Chemistry

GA DG1LY

UT WOS:000371830800085

PM 26821015

OA gold, Green Published, Green Submitted

DA 2024-08-12

ER

PT J

AU Khan, MM

Raza, SS

Javed, H

Ahmad, A

Khan, A

Islam, F

Safhi, MM

Islam, F

AF Khan, Mohd Moshahid

Raza, Syed Shadab

Javed, Hayate

Ahmad, Ajmal

Khan, Andleeb

Islam, Farah

Safhi, Mohammed M.

Islam, Fakhrul

TI Rutin Protects Dopaminergic Neurons from Oxidative Stress in an Animal

Model of Parkinson's Disease

SO NEUROTOXICITY RESEARCH

LA English

DT Article

DE Rutin; 6-Hydroxydopamine; Behavioral activity; Oxidative stress;

Inflammation; Parkinson's disease

ID REPEATED CEREBRAL-ISCHEMIA; SPATIAL MEMORY IMPAIRMENT; RAT MODEL;

LIPID-PEROXIDATION; FREE-RADICALS; 6-HYDROXYDOPAMINE; GLUTATHIONE;

BRAIN; NEUROINFLAMMATION; LESIONS

AB This study was undertaken to investigate the neuroprotective effects of rutin (vitamin P) on 6-hydroxydopamine (6-OHDA)-induced Parkinson's disease (PD) in rats. Oxidative stress and inflammation is an important event, play a crucial role in neurodegenerative disease. Rutin has been shown to have antioxidant and anti-inflammatory actions, and thus was tested for its beneficial effects using 6-OHDA-induced PD rat model. Male Wistar rats were pre-treated with rutin (25 mg/kg bwt, orally) for 3 weeks and subjected to unilateral intrastriatal injection of 6-OHDA (10 mu g in 0.1% ascorbic acid in normal saline). Three weeks after 6-OHDA infusion, rats were tested for neurobehavioral activity, and were killed after 4 weeks of 6-OHDA infusion for the estimation of thiobarbituric acid reactive substances, glutathione, and its dependent enzymes (glutathione peroxidase and glutathione reductase), dopamine (DA) and its metabolite 3,4-dihydroxyphenyl acetic acid. The increase in 6-OHDA-induced rotations and deficits in locomotor activity and motor coordination and decrease in antioxidant level, DA content and its metabolite and increase in the number of dopaminergic D2 receptors in striatum were protected significantly with lesioned group pre-treated with rutin. These findings were further supported by the histopathological and immunohistochemical findings in the substantia nigra that showed that rutin protected neurons from deleterious effects of 6-OHDA. These results suggest that the consumption of rutin, which is novel vitamin, may have the possibility of protective effect against the neurological disorder such as PD.

C1 [Safhi, Mohammed M.; Islam, Fakhrul] Jazan Univ, Fac Pharm, Neurosci & Toxicol Unit, Gizan, Saudi Arabia.

[Khan, Mohd Moshahid; Raza, Syed Shadab; Javed, Hayate; Ahmad, Ajmal; Khan, Andleeb; Islam, Fakhrul] Jamia Hamdard, Dept Med Elementol & Toxicol, Neurotoxicol Lab, New Delhi 110062, India.

[Khan, Mohd Moshahid] Univ Iowa, Carver Coll Med, Dept Neurol, Iowa, IA USA.

[Ahmad, Ajmal] Georgia Hlth Sci Univ, Dept Neurol, Augusta, GA USA.

[Islam, Farah] Jamia Hamdard, Fac Pharm, Dept Biotechnol, New Delhi 110062, India.

C3 Jazan University; Jamia Hamdard University; University of Iowa;

University System of Georgia; Augusta University; Jamia Hamdard

University

RP Islam, F (corresponding author), Jazan Univ, Fac Pharm, Neurosci & Toxicol Unit, Gizan, Saudi Arabia.

EM fislam2001@yahoo.co.in; fislam2001@yahoo.co.in

RI Khan, Andleeb/AAE-4252-2020; Raza, Syed Shadab/AAE-5185-2019; Ahmad,

Ajmal/AAY-6018-2020; Khan, Andleeb/JZD-5974-2024

OI Khan, Andleeb/0000-0001-6125-3309; Raza, Syed

Shadab/0000-0002-6147-9399; Khan, Andleeb/0000-0001-6125-3309; Khan,

Mohammad Moshahid/0000-0003-4679-294X; Javed,

Hayate/0000-0003-0767-2830; ahmad, ajmal/0000-0003-2103-2944

FU DST; UGC; Department of Ayurveda, Yoga & Naturopathy, Unani, Siddha and

Homoeopathy (AYUSH), Ministry of Health and Family Welfare, Government

of India, New Delhi

FX Neurotoxicology Laboratory-Fund for the Improvement of Science and

Technology Sponsored by DST and Special Assistance Programme Sponsored

by UGC.; The authors thank the Department of Ayurveda, Yoga &

Naturopathy, Unani, Siddha and Homoeopathy (AYUSH), Ministry of Health

and Family Welfare, Government of India, New Delhi, for financial

assistance. We greatly acknowledge Ms. Lorie Leo, Department of Internal

Medicine, University of Iowa, for reviewing and editing this manuscript.

Technical assistance of Dharamvir Singh is gratefully appreciated.

CR AFANASEV IB, 1989, BIOCHEM PHARMACOL, V38, P1763, DOI 10.1016/0006-2952(89)90410-3

AGRAWAL AK, 1995, INT J DEV NEUROSCI, V13, P105, DOI 10.1016/0736-5748(95)00002-X

Ahmad M, 2005, J NEUROCHEM, V93, P94, DOI 10.1111/j.1471-4159.2005.03000.x

Allbutt HN, 2007, J NEUROSCI METH, V159, P195, DOI 10.1016/j.jneumeth.2006.07.006

Bishnoi M, 2007, FUND CLIN PHARMACOL, V21, P521, DOI 10.1111/j.1472-8206.2007.00512.x

Blandini Fabio, 2008, Parkinsonism Relat Disord, V14 Suppl 2, pS124, DOI 10.1016/j.parkreldis.2008.04.015

Blum D, 2001, PROG NEUROBIOL, V65, P135, DOI 10.1016/S0301-0082(01)00003-X

Cannon JR, 2005, NEUROSCI LETT, V373, P189, DOI 10.1016/j.neulet.2004.10.089

CARLBERG I, 1975, J BIOL CHEM, V250, P5475

Chaturvedi RK, 2006, NEUROBIOL DIS, V22, P421, DOI 10.1016/j.nbd.2005.12.008

Choi DK, 2005, J NEUROSCI, V25, P6594, DOI 10.1523/JNEUROSCI.0970-05.2005

Claiborne A., 1985, CRC HDB METHODS OXYG, P283

del Zoppo G, 2000, BRAIN PATHOL, V10, P95

Deumens R, 2002, EXP NEUROL, V175, P303, DOI 10.1006/exnr.2002.7891

Dringen R, 2000, PROG NEUROBIOL, V62, P649, DOI 10.1016/S0301-0082(99)00060-X

FREEMAN BA, 1982, LAB INVEST, V47, P412

Gao HM, 2008, J NEUROSCI, V28, P7687, DOI 10.1523/JNEUROSCI.0143-07.2008

Gupta R, 2003, PHARMACOL RES, V48, P209, DOI 10.1016/S1043-6618(03)00102-6

Hirsch EC, 2005, PARKINSONISM RELAT D, V11, pS9, DOI 10.1016/j.parkreldis.2004.10.013

Hirsch EC, 2003, ANN NY ACAD SCI, V991, P214

Hirsch EC, 2009, LANCET NEUROL, V8, P382, DOI 10.1016/S1474-4422(09)70062-6

Ishrat T, 2009, BRAIN RES, V1281, P117, DOI 10.1016/j.brainres.2009.04.010

Islam F, 2002, BIOL TRACE ELEM RES, V90, P203, DOI 10.1385/BTER:90:1-3:203

Jenner P, 2003, ANN NEUROL, V53, pS26, DOI 10.1002/ana.10483

Jenner Peter, 2007, Handb Clin Neurol, V83, P507, DOI 10.1016/S0072-9752(07)83024-7

JIANG ZY, 1992, ANAL BIOCHEM, V202, P384, DOI 10.1016/0003-2697(92)90122-N

Jin F, 2008, EUR J PHARMACOL, V600, P78, DOI 10.1016/j.ejphar.2008.10.005

Joglar B, 2009, J NEUROCHEM, V109, P656, DOI 10.1111/j.1471-4159.2009.05999.x

JOLLOW DJ, 1974, PHARMACOLOGY, V11, P151, DOI 10.1159/000136485

Kamalakkannan N, 2006, MOL CELL BIOCHEM, V293, P211, DOI 10.1007/s11010-006-9244-1

Khan MM, 2009, BRAIN RES, V1292, P123, DOI 10.1016/j.brainres.2009.07.026

Kirik D, 1998, EXP NEUROL, V152, P259, DOI 10.1006/exnr.1998.6848

Koda T, 2008, NUTR RES, V28, P629, DOI 10.1016/j.nutres.2008.06.004

Koda T, 2009, CELL MOL NEUROBIOL, V29, P523, DOI 10.1007/s10571-008-9344-4

Koprich JB, 2008, J NEUROINFLAMM, V5, DOI 10.1186/1742-2094-5-8

La Casa C, 2000, J ETHNOPHARMACOL, V71, P45, DOI 10.1016/S0378-8741(99)00174-9

LEVINE RL, 1990, METHOD ENZYMOL, V186, P464

Li YC, 2009, NEUROPHARMACOLOGY, V56, P580, DOI 10.1016/j.neuropharm.2008.10.016

Little JP, 2011, MINI-REV MED CHEM, V11, P582

LOWRY OH, 1951, J BIOL CHEM, V193, P265

MEISTER A, 1988, J BIOL CHEM, V263, P17205

MISKO TP, 1993, ANAL BIOCHEM, V214, P11, DOI 10.1006/abio.1993.1449

Mogi M, 1999, NEUROSCI LETT, V268, P101, DOI 10.1016/S0304-3940(99)00388-2

MOHANDAS J, 1984, CANCER RES, V44, P5086

Ogura T, 2005, NEUROSCI RES, V51, P299, DOI 10.1016/j.neures.2004.12.006

Paxinos G., 1997, RAT BRAIN STEROTAXIC, VThird

Pu FL, 2007, J PHARMACOL SCI, V104, P329, DOI 10.1254/jphs.FP0070247

Pu FL, 2004, J PHARMACOL SCI, V94, P393, DOI 10.1254/jphs.94.393

Rozas G, 1998, J NEUROSCI METH, V83, P165, DOI 10.1016/S0165-0270(98)00078-8

Sánchez-Iglesias S, 2009, J NEUROCHEM, V109, P879, DOI 10.1111/j.1471-4159.2009.06019.x

SAUER H, 1994, NEUROSCIENCE, V59, P401, DOI 10.1016/0306-4522(94)90605-X

Schwarting RKW, 1996, PROG NEUROBIOL, V50, P275, DOI 10.1016/S0301-0082(96)00040-8

Stevens MJ, 2000, DIABETES, V49, P1006, DOI 10.2337/diabetes.49.6.1006

SUN Y, 1990, FREE RADICAL BIO MED, V8, P583, DOI 10.1016/0891-5849(90)90156-D

Tansey MG, 2007, EXP NEUROL, V208, P1, DOI 10.1016/j.expneurol.2007.07.004

UTELY HC, 1967, ARCH BIOCHEM BIOPHYS, V260, P521

Xue YQ, 2007, NEUROSCIENCE, V146, P1245, DOI 10.1016/j.neuroscience.2007.02.004

Zafar KS, 2003, J NEUROCHEM, V84, P438, DOI 10.1046/j.1471-4159.2003.01531.x

NR 58

TC 128

Z9 135

U1 1

U2 21

PU SPRINGER

PI NEW YORK

PA 233 SPRING ST, NEW YORK, NY 10013 USA

SN 1029-8428

J9 NEUROTOX RES

JI Neurotox. Res.

PD JUL

PY 2012

VL 22

IS 1

BP 1

EP 15

DI 10.1007/s12640-011-9295-2

PG 15

WC Neurosciences

WE Science Citation Index Expanded (SCI-EXPANDED)

SC Neurosciences & Neurology

GA 943XN

UT WOS:000304160700001

PM 22194158

DA 2024-08-12

ER

PT J

AU Rohn, TT

Catlin, LW

AF Rohn, Troy T.

Catlin, Lindsey W.

TI Immunolocalization of Influenza A Virus and Markers of Inflammation in

the Human Parkinson's Disease Brain

SO PLOS ONE

LA English

DT Article

ID neurodegenerative disease; alzheimer's disease; ALPHA-SYNUCLEIN;

NEUROINFLAMMATION; NEUROPROTECTION; MODEL

AB Although much is known regarding the molecular mechanisms leading to neuronal cell loss in Parkinson's disease (PD), the initiating event has not been identified. Prevailing theories including a chemical insult or infectious agent have been postulated as possible triggers, leading to neuroinflammation. We present immunohistochemical data indicating the presence of influenza A virus within the substantia nigra pars compacta (SNpc) from postmortem PD brain sections. Influenza A virus labeling was identified within neuromelanin granules as well as on tissue macrophages in the SNpc. Further supporting a role for neuroinflammation in PD was the identification of T-lymphocytes that colocalized with an antibody to caspase-cleaved Beclin-1 within the SNpc. The presence of influenza A virus together with macrophages and T-lymphocytes may contribute to the neuroinflammation associated with this disease.

C1 [Rohn, Troy T.; Catlin, Lindsey W.] Boise State Univ, Dept Biol, Boise, ID 83725 USA.

C3 Idaho; Boise State University

RP Rohn, TT (corresponding author), Boise State Univ, Dept Biol, Boise, ID 83725 USA.

EM trohn@boisestate.edu

FU KO Dementia Foundation, Boise Idaho; NASA [NNX10AN29A]; NASA

[NNX10AN29A, 127883] Funding Source: Federal RePORTER

FX This work was funded by KO Dementia Foundation, Boise Idaho and a NASA

grant, NNX10AN29A. The funders had no role in study design, data

collection and analysis, decision to publish, or preparation of the

manuscript.

CR Amor S, 2010, IMMUNOLOGY, V129, P154, DOI 10.1111/j.1365-2567.2009.03225.x

Baumann Sven, 2002, Current Molecular Medicine (Hilversum), V2, P257, DOI 10.2174/1566524024605671

Beers DR, 2008, P NATL ACAD SCI USA, V105, P15558, DOI 10.1073/pnas.0807419105

Björklund A, 2010, PROG BRAIN RES, V184, P113, DOI 10.1016/S0079-6123(10)84006-3

Brochard V, 2009, J CLIN INVEST, V119, P182, DOI 10.1172/JCI36470

Dickson DW, 2001, CURR OPIN NEUROL, V14, P423, DOI 10.1097/00019052-200108000-00001

Goldsmith JR, 1997, ENVIRON RES, V73, P156, DOI 10.1006/enrs.1997.3696

Gordon S, 2007, EUR J IMMUNOL, V37, pS9, DOI 10.1002/eji.200737638

Jang HM, 2009, P NATL ACAD SCI USA, V106, P14063, DOI 10.1073/pnas.0900096106

Kaczmarek M, 2010, COMMUN DIS INTELL, V34, P8

Kokoulina P, 2010, NEURODEGENER DIS, V7, P243, DOI 10.1159/000287952

Kumar A, 2004, ARCH NEUROL-CHICAGO, V61, P1057, DOI 10.1001/archneur.61.7.1057

MCGEER PL, 1988, NEUROLOGY, V38, P1285, DOI 10.1212/WNL.38.8.1285

Miklossy J, 2006, EXP NEUROL, V197, P275, DOI 10.1016/j.expneurol.2005.10.034

Orr CF, 2005, BRAIN, V128, P2665, DOI 10.1093/brain/awh625

Qian L, 2010, J NEURAL TRANSM, V117, P971, DOI 10.1007/s00702-010-0428-1

RAVENHOLT RT, 1982, LANCET, V2, P860

Reichmann H, 2010, NEURODEGENER DIS, V7, P284, DOI 10.1159/000314478

Rezai-Zadeh K, 2009, J NEUROIMMUNE PHARM, V4, P462, DOI 10.1007/s11481-009-9166-2

Rohn T.T., 2009, INT J PHYSL PATH PHA, V1, P24

Rohn TT, 2008, BRAIN RES, V1228, P189, DOI 10.1016/j.brainres.2008.06.094

ROHN TT, 2010, NEUROBIOL DIS

SCHWARTZ J, 1979, ANN NEUROL, V6, P261, DOI 10.1002/ana.410060314

Schwartz M, 2005, J NEUROL SCI, V233, P163, DOI 10.1016/j.jns.2005.03.014

Tansey MG, 2007, EXP NEUROL, V208, P1, DOI 10.1016/j.expneurol.2007.07.004

Villoslada P, 2008, CLIN IMMUNOL, V128, P294, DOI 10.1016/j.clim.2008.04.003

WETMUR JG, 1979, ARCH NEUROL-CHICAGO, V36, P462, DOI 10.1001/archneur.1979.00500440032004

Winslow AR, 2010, J CELL BIOL, V190, P1023, DOI 10.1083/jcb.201003122

Wirawan E, 2010, CELL DEATH DIS, V1, DOI 10.1038/cddis.2009.16

Yamada T, 1996, Parkinsonism Relat Disord, V2, P113, DOI 10.1016/1353-8020(96)00006-5

Yang QA, 2010, NEUROSCIENTIST, V16, P335, DOI 10.1177/1073858409357118

2011, ANN INTERN MED, V154, P168

NR 32

TC 46

Z9 50

U1 0

U2 7

PU PUBLIC LIBRARY SCIENCE

PI SAN FRANCISCO

PA 1160 BATTERY STREET, STE 100, SAN FRANCISCO, CA 94111 USA

SN 1932-6203

J9 PLOS ONE

JI PLoS One

PD MAY 31

PY 2011

VL 6

IS 5

AR e20495

DI 10.1371/journal.pone.0020495

PG 10

WC Multidisciplinary Sciences

WE Science Citation Index Expanded (SCI-EXPANDED)

SC Science & Technology - Other Topics

GA 770OP

UT WOS:000291097600079

PM 21655265

OA Green Published, Green Submitted, gold

DA 2024-08-12

ER

PT J

AU Fakhri, S

Aneva, IY

Farzaei, MH

Sobarzo-Sánchez, E

AF Fakhri, Sajad

Aneva, Ina Yosifova

Farzaei, Mohammad Hosein

Sobarzo-Sanchez, Eduardo

TI The Neuroprotective Effects of Astaxanthin: Therapeutic Targets and

Clinical Perspective

SO MOLECULES

LA English

DT Review

DE neurodegenerative disease; astaxanthin; pharmacology; neuroprotective

agent; oxidative stress; neuroinflammation; apoptosis; drug delivery

system

ID NANOSTRUCTURED LIPID CARRIERS; SPINAL-CORD-INJURY; DEPRESSIVE-LIKE

BEHAVIOR; alzheimer's disease ROLE; EARLY BRAIN-INJURY; OXIDATIVE STRESS;

NEUROPATHIC PAIN; SUBARACHNOID HEMORRHAGE; SPREADING DEPRESSION; INDUCED

CYTOTOXICITY

AB As the leading causes of human disability and mortality, neurological diseases affect millions of people worldwide and are on the rise. Although the general roles of several signaling pathways in the pathogenesis of neurodegenerative disorders have so far been identified, the exact pathophysiology of neuronal disorders and their effective treatments have not yet been precisely elucidated. This requires multi-target treatments, which should simultaneously attenuate neuronal inflammation, oxidative stress, and apoptosis. In this regard, astaxanthin (AST) has gained growing interest as a multi-target pharmacological agent against neurological disorders including Parkinson's disease (PD), Alzheimer's disease (AD), brain and spinal cord injuries, neuropathic pain (NP), aging, depression, and autism. The present review highlights the neuroprotective effects of AST mainly based on its anti-inflammatory, antioxidative, and anti-apoptotic properties that underlies its pharmacological mechanisms of action to tackle neurodegeneration. The need to develop novel AST delivery systems, including nanoformulations, targeted therapy, and beyond, is also considered.

C1 [Fakhri, Sajad; Farzaei, Mohammad Hosein] Kermanshah Univ Med Sci, Hlth Inst, Pharmaceut Sci Res Ctr, Kermanshah 6718874414, Iran.

[Aneva, Ina Yosifova] Bulgarian Acad Sci, Inst Biodivers & Ecosyst Res, BU-1113 Sofia, Bulgaria.

[Sobarzo-Sanchez, Eduardo] Univ Santiago de Compostela, Fac Pharm, Dept Organ Chem, Lab Pharmaceut Chem, Santiago De Compostela 15782, Spain.

[Sobarzo-Sanchez, Eduardo] Univ Cent Chile, Fac Ciencias Salud, Inst Invest & Innovac Salud, Santiago 8330507, Chile.

C3 Kermanshah University of Medical Sciences; Bulgarian Academy of

Sciences; Universidade de Santiago de Compostela; Universidad Central de

Chile

RP Farzaei, MH (corresponding author), Kermanshah Univ Med Sci, Hlth Inst, Pharmaceut Sci Res Ctr, Kermanshah 6718874414, Iran.; Sobarzo-Sánchez, E (corresponding author), Univ Santiago de Compostela, Fac Pharm, Dept Organ Chem, Lab Pharmaceut Chem, Santiago De Compostela 15782, Spain.; Sobarzo-Sánchez, E (corresponding author), Univ Cent Chile, Fac Ciencias Salud, Inst Invest & Innovac Salud, Santiago 8330507, Chile.

EM mh.farzaei@gmail.com

RI Aneva, Ina Yosifova/AAI-4487-2020; Sobarzo-Sánchez, Eduardo/B-1507-2012;

Aneva, Ina/HNR-2928-2023

OI Sobarzo-Sánchez, Eduardo/0000-0002-2790-2544; Aneva,

Ina/0000-0002-6476-5438

CR Abadie-Guedes R, 2008, ALCOHOL CLIN EXP RES, V32, P1417, DOI 10.1111/j.1530-0277.2008.00710.x

Abadie-Guedes R, 2012, ALCOHOL CLIN EXP RES, V36, P1563, DOI 10.1111/j.1530-0277.2012.01766.x

Affandi M.M. M., 2011, ASIAN J PHARM CLIN R, V4, P142

Al-Amin MM, 2015, METAB BRAIN DIS, V30, P1237, DOI 10.1007/s11011-015-9699-4

Al-Amin MM, 2015, BEHAV BRAIN RES, V286, P112, DOI 10.1016/j.bbr.2015.02.041

Ambati RR, 2014, MAR DRUGS, V12, P128, DOI 10.3390/md12010128

Anarjan N, 2014, INT J FOOD PROP, V17, P937, DOI 10.1080/10942912.2012.685680

[Anonymous], STROKE S1

[Anonymous], FOOD STYLE 21

[Anonymous], CAROTENOIDS NUTR ANA

[Anonymous], 2001, FR J

[Anonymous], 2006, CYCLODEXTRINS THEIR

Archibald Neil, 2013, Handb Clin Neurol, V110, P435, DOI 10.1016/B978-0-444-52901-5.00037-X

Astray G, 2009, FOOD HYDROCOLLOID, V23, P1631, DOI 10.1016/j.foodhyd.2009.01.001

Bailey A, 1998, BRAIN, V121, P889, DOI 10.1093/brain/121.5.889

Bakunina N, 2015, IMMUNOLOGY, V144, P365, DOI 10.1111/imm.12443

Balietti M, 2016, J SCI FOOD AGR, V96, P615, DOI 10.1002/jsfa.7131

Barros MP, 2018, J AGR FOOD CHEM, V66, P5733, DOI 10.1021/acs.jafc.8b00866

Barros MP, 2014, NUTRIENTS, V6, P1293, DOI 10.3390/nu6031293

Berton O, 2006, NAT REV NEUROSCI, V7, P137, DOI 10.1038/nrn1846

Bharathiraja S, 2016, NANOMATERIALS-BASEL, V6, DOI 10.3390/nano6040078

Bhat AH, 2015, BIOMED PHARMACOTHER, V74, P101, DOI 10.1016/j.biopha.2015.07.025

Bhatt PC, 2016, RSC ADV, V6, P10001, DOI 10.1039/c5ra19113k

Bonomini F, 2015, AGING DIS, V6, P109, DOI 10.14336/AD.2014.0305

Boyle CA, 2011, PEDIATRICS, V127, P1034, DOI 10.1542/peds.2010-2989

Burns A, 2009, LANCET NEUROL, V8, P4, DOI 10.1016/S1474-4422(08)70271-0

Carter MJ, 2014, THER RECREAT J, V48, P275

Che HX, 2018, J AGR FOOD CHEM, V66, P4948, DOI 10.1021/acs.jafc.8b00988

Chez MG, 2007, PEDIATR NEUROL, V36, P361, DOI 10.1016/j.pediatrneurol.2007.01.012

Chiu CH, 2016, INT J MOL SCI, V17, DOI 10.3390/ijms17071128

Corrigan JD, 2010, J HEAD TRAUMA REHAB, V25, P72, DOI 10.1097/HTR.0b013e3181ccc8b4

Courchesne E, 2007, NEURON, V56, P399, DOI 10.1016/j.neuron.2007.10.016

D'Angelo R, 2013, EUR REV MED PHARMACO, V17, P3257

Date AA, 2004, CURR OPIN COLLOID IN, V9, P222, DOI 10.1016/j.cocis.2004.06.009

David BT, 2013, NEUROBIOL DIS, V54, P194, DOI 10.1016/j.nbd.2012.12.012

de Roos B, 2015, MOL NUTR FOOD RES, V59, P1229, DOI 10.1002/mnfr.201400568

Del Valle EMM, 2004, PROCESS BIOCHEM, V39, P1033, DOI 10.1016/S0032-9592(03)00258-9

Dong SZ, 2014, EUR J LIPID SCI TECH, V116, P978, DOI 10.1002/ejlt.201300261

Echeverría F, 2017, PROSTAG LEUKOTR ESS, V124, P1, DOI 10.1016/j.plefa.2017.08.001

Eren B, 2019, J COSMET DERMATOL-US, V18, P242, DOI 10.1111/jocd.12665

Evans T.A., 2008, Am. J. Biochem. Biotech, V4, P61

Fakhri S, 2019, EUR J PAIN, V23, P750, DOI 10.1002/ejp.1342

Fakhri S, 2018, BRAIN RES BULL, V143, P217, DOI 10.1016/j.brainresbull.2018.09.011

Fakhri S, 2018, PHARMACOL RES, V136, P1, DOI 10.1016/j.phrs.2018.08.012

Fernández MJF, 2019, CURR PHARM BIOTECHNO, V20, P100, DOI 10.2174/1389201019666180925120657

Finnerup NB, 2016, PAIN, V157, P1599, DOI 10.1097/j.pain.0000000000000492

Finnerup NB, 2005, PAIN, V118, P289, DOI 10.1016/j.pain.2005.08.013

Forner S, 2016, NEUROSCI LETT, V617, P14, DOI 10.1016/j.neulet.2016.02.005

Gabriel M, 2017, CAN MED ASSOC J, V189, pE747, DOI 10.1503/cmaj.160991

Galgano M, 2017, CELL TRANSPLANT, V26, P1118, DOI 10.1177/0963689717714102

Garcês A, 2018, EUR J PHARM SCI, V112, P159, DOI 10.1016/j.ejps.2017.11.023

Gong MY, 2016, BIOTECHNOL ADV, V34, P1396, DOI 10.1016/j.biotechadv.2016.10.005

Granot E, 2004, CLIN NUTR, V23, P3, DOI 10.1016/S0261-5614(03)00097-9

Grimmig Beth, 2018, Oncotarget, V9, P10388, DOI 10.18632/oncotarget.23737

Haenisch B, 2011, PHARMACOL THERAPEUT, V129, P352, DOI 10.1016/j.pharmthera.2010.12.002

Hama S, 2012, BIOL PHARM BULL, V35, P2238, DOI 10.1248/bpb.b12-00715

Hama S, 2012, J PHARM SCI-US, V101, P2909, DOI 10.1002/jps.23216

Higuera-Ciapara I, 2006, CRIT REV FOOD SCI, V46, P185, DOI 10.1080/10408690590957188

Chuyen HV, 2017, CRIT REV FOOD SCI, V57, P2600, DOI 10.1080/10408398.2015.1063477

Hussein G, 2006, J NAT PROD, V69, P443, DOI 10.1021/np050354+

Ikeda Y, 2008, J NEUROCHEM, V107, P1730, DOI 10.1111/j.1471-4159.2008.05743.x

Jiang TF, 2016, PROG NEUROBIOL, V147, P1, DOI 10.1016/j.pneurobio.2016.07.005

Jiang X, 2018, NEUROSCI LETT, V662, P36, DOI 10.1016/j.neulet.2017.09.064

Jiang X, 2017, ONCOTARGET, V8, P25552, DOI 10.18632/oncotarget.16069

Jiang X, 2016, BRAIN RES, V1649, P30, DOI 10.1016/j.brainres.2016.08.029

Jones DP, 2015, ANTIOXID REDOX SIGN, V23, P734, DOI 10.1089/ars.2015.6247

Kamezaki C, 2016, J CLIN BIOCHEM NUTR, V59, P100, DOI 10.3164/jcbn.15-153

Kansanen E, 2012, FREE RADICAL BIO MED, V52, P973, DOI 10.1016/j.freeradbiomed.2011.11.038

Karimi N, 2018, COLLOID INTERFAC SCI, V22, P18, DOI 10.1016/j.colcom.2017.11.006

Karnati HK, 2015, J ALZHEIMERS DIS, V48, P563, DOI 10.3233/JAD-150395

Kemper TL, 1998, J NEUROPATH EXP NEUR, V57, P645, DOI 10.1097/00005072-199807000-00001

Kern JK, 2013, TRANSL NEURODEGENER, V2, DOI 10.1186/2047-9158-2-17

Khalid N, 2017, FOOD RES INT, V102, P364, DOI 10.1016/j.foodres.2017.06.019

Khan I, 2018, SCI REP-UK, V8, DOI 10.1038/s41598-017-18644-9

Kidd P, 2011, ALTERN MED REV, V16, P355

Kim GH, 2015, EXP NEUROBIOL, V24, P325, DOI 10.5607/en.2015.24.4.325

Kim HA, 2012, N-S ARCH PHARMACOL, V385, P953, DOI 10.1007/s00210-012-0790-7

Kim S, 2010, J KOREAN SOC APPL BI, V53, P559, DOI 10.3839/jksabc.2010.086

Kittikaiwan P, 2007, CARBOHYD POLYM, V70, P378, DOI 10.1016/j.carbpol.2007.04.021

Komaki A, 2015, PHARMACOL BIOCHEM BE, V131, P98, DOI 10.1016/j.pbb.2015.02.008

Krajcovicova-Kudlackova M, 2009, BRATISL MED J, V110, P247

Kramer JLK, 2017, J NEUROSCI RES, V95, P1295, DOI 10.1002/jnr.23881

Krogh J, 2014, BRAIN BEHAV IMMUN, V35, P70, DOI 10.1016/j.bbi.2013.08.014

Kroning M, 2016, J CHRIST NURS, V33, P78, DOI 10.1097/CNJ.0000000000000254

Kuedo Z, 2016, MOLECULES, V21, DOI 10.3390/molecules21030382

Lampert A, 2006, EXP BRAIN RES, V174, P660, DOI 10.1007/s00221-006-0511-x

Lee DH, 2011, FOOD CHEM TOXICOL, V49, P271, DOI 10.1016/j.fct.2010.10.029

Lee DH, 2010, J CLIN BIOCHEM NUTR, V47, P121, DOI 10.3164/jcbn.10-29

Lee JS, 2011, INT J BIOL MACROMOL, V49, P268, DOI 10.1016/j.ijbiomac.2011.04.021

Lee KS, 2013, KOREAN J PHYSIOL PHA, V17, P89, DOI 10.4196/kjpp.2013.17.1.89

Lee M, 2002, BRAIN, V125, P1483, DOI 10.1093/brain/awf160

Li XH, 2009, J NEUROIMMUNOL, V207, P111, DOI 10.1016/j.jneuroim.2008.12.002

Liguori I, 2018, CLIN INTERV AGING, V13, P757, DOI 10.2147/CIA.S158513

Lin SF, 2016, PLOS ONE, V11, DOI 10.1371/journal.pone.0153685

Liu XJ, 2016, FOOD BIOPHYS, V11, P302, DOI 10.1007/s11483-016-9443-6

Liu XB, 2009, BRAIN RES, V1254, P18, DOI 10.1016/j.brainres.2008.11.076

Lu YP, 2010, BRAIN RES, V1360, P40, DOI 10.1016/j.brainres.2010.09.016

Mamik MK, 2017, BRAIN, V140, P2273, DOI 10.1093/brain/awx133

Manayi A, 2016, CRIT REV BIOTECHNOL, V36, P829, DOI 10.3109/07388551.2015.1049510

Masoudi A, 2017, BEHAV BRAIN RES, V329, P104, DOI 10.1016/j.bbr.2017.04.026

McClements DJ, 2012, SOFT MATTER, V8, P1719, DOI 10.1039/c2sm06903b

Miller AH, 2016, NAT REV IMMUNOL, V16, P22, DOI 10.1038/nri.2015.5

Montenegro L, 2016, J DRUG DELIV SCI TEC, V32, P100, DOI 10.1016/j.jddst.2015.10.003

Nalawade P, 2015, J INCL PHENOM MACRO, V83, P63, DOI 10.1007/s10847-015-0541-8

Naseri K, 2013, J MOL NEUROSCI, V49, P470, DOI 10.1007/s12031-012-9840-3

Ogawa M., 2007, Fujifilm research development, V52, P26

Ornoy A, 2019, INT J MOL SCI, V20, DOI 10.3390/ijms20051074

Ozturk B, 2016, CURR OPIN FOOD SCI, V7, P1, DOI 10.1016/j.cofs.2015.07.008

Padurariu M, 2013, PSYCHIAT DANUB, V25, P401

Palmen SJMC, 2004, BRAIN, V127, P2572, DOI 10.1093/brain/awh287

Pan L, 2018, MOLECULES, V23, DOI 10.3390/molecules23112822

Peng CH, 2010, EUR J PHARM BIOPHARM, V75, P154, DOI 10.1016/j.ejpb.2010.03.004

Pereira AC, 2017, MOL PSYCHIATR, V22, P296, DOI 10.1038/mp.2016.33

Perluigi M, 2009, PROTEOM CLIN APPL, V3, P682, DOI 10.1002/prca.200800161

Qiao JW, 2017, NEUROSCI LETT, V649, P55, DOI 10.1016/j.neulet.2017.03.048

Raikos V, 2017, INT J FOOD SCI TECH, V52, P68, DOI 10.1111/ijfs.13272

Riediger C, 2017, FRONT NEUROL, V8, DOI 10.3389/fneur.2017.00307

Rodrigo R, 2013, CLIN SCI, V124, P1, DOI 10.1042/CS20110663

Rodriguez-Ruiz V, 2018, MOLECULES, V23, DOI 10.3390/molecules23102601

Rolland AS, 2017, NEUROSCIENCE, V346, P126, DOI 10.1016/j.neuroscience.2017.01.014

Rubiano AM, 2015, NATURE, V527, pS193, DOI 10.1038/nature16035

Sadigh-Eteghad S, 2015, MED PRIN PRACT, V24, P1, DOI 10.1159/000369101

SAJDEL-SULKOWSKA E.M., 2008, Am.J.Biochem.Biotechnol, V4, P73

Sajdel-Sulkowska EM, 2011, CEREBELLUM, V10, P43, DOI 10.1007/s12311-010-0223-4

Sangsuriyawong A, 2019, FOOD SCI BIOTECHNOL, V28, P529

Sasso S, 2012, FEMS MICROBIOL REV, V36, P761, DOI 10.1111/j.1574-6976.2011.00304.x

Schumann CM, 2006, J NEUROSCI, V26, P7674, DOI 10.1523/JNEUROSCI.1285-06.2006

Shabab T, 2017, INT J NEUROSCI, V127, P624, DOI 10.1080/00207454.2016.1212854

Shanmugapriya K, 2019, INT J PHARMACEUT, V560, P334, DOI 10.1016/j.ijpharm.2019.02.015

Sharma K, 2018, NEUROSCI LETT, V674, P162, DOI 10.1016/j.neulet.2018.03.030

Shtilbans A, 2012, CURR OPIN NEUROL, V25, P460, DOI 10.1097/WCO.0b013e3283550c0d

Sies H, 2017, ANNU REV BIOCHEM, V86, P715, DOI 10.1146/annurev-biochem-061516-045037

Sotomayor-Gerding D, 2016, FOOD CHEM, V199, P463, DOI 10.1016/j.foodchem.2015.12.004

Stojkovska I, 2015, EXP BIOL MED, V240, P1387, DOI 10.1177/1535370215576313

Strickland D, 2004, MOVEMENT DISORD, V19, P318, DOI 10.1002/mds.10619

Takemoto Y, 2018, AURIS NASUS LARYNX, V45, P20, DOI 10.1016/j.anl.2017.02.001

Taksima T, 2015, LWT-FOOD SCI TECHNOL, V62, P431, DOI 10.1016/j.lwt.2015.01.011

Tamjidi F, 2018, J SCI FOOD AGR, V98, P511, DOI 10.1002/jsfa.8488

Tamjidi F, 2017, J FOOD SCI TECH MYS, V54, P3132, DOI 10.1007/s13197-017-2749-7

Tamjidi F, 2014, INNOV FOOD SCI EMERG, V26, P366, DOI 10.1016/j.ifset.2014.06.012

Tamjidi F, 2013, INNOV FOOD SCI EMERG, V19, P29, DOI 10.1016/j.ifset.2013.03.002

Thapa A, 2017, INT J MOL SCI, V18, DOI 10.3390/ijms18071583

Thomas J, 2015, BIOMED RES INT, V2015, DOI 10.1155/2015/172801

Thuret S, 2006, NAT REV NEUROSCI, V7, P628, DOI 10.1038/nrn1955

Tysnes OB, 2017, J NEURAL TRANSM, V124, P901, DOI 10.1007/s00702-017-1686-y

Varela JC, 2015, PHOTOSYNTH RES, V125, P423, DOI 10.1007/s11120-015-0149-2

Vargas DL, 2005, ANN NEUROL, V57, P67, DOI 10.1002/ana.20315

Wang HQ, 2010, BRAIN RES, V1360, P159, DOI 10.1016/j.brainres.2010.08.100

Wang Q, 2017, FOOD CHEM, V227, P9, DOI 10.1016/j.foodchem.2017.01.081

Wang XJ, 2018, CELL DEATH DISCOV, V4, DOI 10.1038/s41420-018-0114-x

Wang YB, 2019, ACTA HISTOCHEM, V121, P56, DOI 10.1016/j.acthis.2018.10.014

Weber S, 2014, EUR J PHARM BIOPHARM, V86, P7, DOI 10.1016/j.ejpb.2013.08.013

Wen X, 2015, NEUROSCIENCE, V303, P558, DOI 10.1016/j.neuroscience.2015.07.034

Wu HJ, 2015, MAR DRUGS, V13, P5750, DOI 10.3390/md13095750

Wu Q, 2014, MAR DRUGS, V12, P6125, DOI 10.3390/md12126125

Wu RP, 2010, P NATL ACAD SCI USA, V107, P7479, DOI 10.1073/pnas.1002890107

Wu WQ, 2014, FOOD FUNCT, V5, P158, DOI 10.1039/c3fo60400d

Wu YJ, 2018, MOLECULES, V23, DOI 10.3390/molecules23040726

Yamashita Eiji, 2015, PharmaNutrition, V3, P115, DOI 10.1016/j.phanu.2015.09.001

Ye QY, 2013, MAR DRUGS, V11, P1019, DOI 10.3390/md11041019

Ye QY, 2012, BMC NEUROSCI, V13, DOI 10.1186/1471-2202-13-156

Zajac G, 2018, PHYS CHEM CHEM PHYS, V20, P18038, DOI 10.1039/c8cp01742e

Zhang MK, 2016, BMC NEUROSCI, V17, DOI 10.1186/s12868-016-0295-2

Zhang XS, 2015, BRAIN RES, V1624, P113, DOI 10.1016/j.brainres.2015.07.020

Zhang XS, 2014, MAR DRUGS, V12, P4291, DOI 10.3390/md12084291

Zhang XS, 2014, J NEUROSURG, V121, P42, DOI 10.3171/2014.2.JNS13730

Zhang XS, 2019, FASEB J, V33, P722, DOI 10.1096/fj.201800642RR

Zhang XJ, 2017, CAN J CHEM ENG, V95, P412, DOI 10.1002/cjce.22712

Zhang YR, 2015, J FUNCT FOODS, V16, P137, DOI 10.1016/j.jff.2015.04.008

Zhou XY, 2017, BRAIN RES, V1657, P262, DOI 10.1016/j.brainres.2016.12.018

Zuluaga M, 2018, CHEM-BIOL INTERACT, V279, P145, DOI 10.1016/j.cbi.2017.11.012

NR 171

TC 89

Z9 91

U1 3

U2 37

PU MDPI

PI BASEL

PA ST ALBAN-ANLAGE 66, CH-4052 BASEL, SWITZERLAND

EI 1420-3049

J9 MOLECULES

JI Molecules

PD JUL 2

PY 2019

VL 24

IS 14

AR 2640

DI 10.3390/molecules24142640

PG 19

WC Biochemistry & Molecular Biology; Chemistry, Multidisciplinary

WE Science Citation Index Expanded (SCI-EXPANDED)

SC Biochemistry & Molecular Biology; Chemistry

GA IS7AS

UT WOS:000482303000123

PM 31330843

OA Green Published, Green Submitted, gold

DA 2024-08-12

ER

PT J

AU Terkelsen, MH

Klaestrup, IH

Hvingelby, V

Lauritsen, J

Pavese, N

Romero-Ramos, M

AF Terkelsen, Miriam Hojholt

Klaestrup, Ida H.

Hvingelby, Victor

Lauritsen, Johanne

Pavese, Nicola

Romero-Ramos, Marina

TI Neuroinflammation and Immune Changes in Prodromal Parkinson's Disease

and Other Synucleinopathies

SO JOURNAL OF PARKINSONS DISEASE

LA English

DT Review

DE Microglia; monocytes; T-cells; alpha-synuclein; Parkinson's disease;

LRRK2; GBA; prodromal

ID SLEEP BEHAVIOR DISORDER; ALPHA-SYNUCLEIN; MICROGLIAL ACTIVATION;

SYSTEMIC INFLAMMATION; alzheimer's disease; OXIDATIVE STRESS; CYTOKINE

RELEASE; GUT MICROBIOME; DOPAMINE; BLOOD

AB Multiple lines of clinical and pre-clinical research support a pathogenic role for neuroinflammation and peripheral immune system dysfunction in Parkinson's disease. In this paper, we have reviewed and summarised the published literature reporting evidence of neuroinflammation and peripheral immune changes in cohorts of patients with isolated REM sleep behaviour disorder and non-manifesting carriers of GBA or LRRK2 gene mutations, who have increased risk for Parkinsonism and synucleinopathies, and could be in the prodromal stage of these conditions. Taken together, the findings of these studies suggest that the early stages of pathology in Parkinsonism involve activation of both the central and peripheral immune systems with significant crosstalk. We consider these findings with respect to those found in patients with clinical Parkinson's disease and discuss their possible pathological roles. Moreover, those factors possibly associated with the immune response, such as the immunomodulatory role of the affected neurotransmitters and the changes in the gut-brain axis, are also considered.

C1 [Terkelsen, Miriam Hojholt; Hvingelby, Victor; Pavese, Nicola] Aarhus Univ, Dept Clin Med Nucl Med & PET, Aarhus, Denmark.

[Klaestrup, Ida H.; Lauritsen, Johanne; Romero-Ramos, Marina] Aarhus Univ, DANDRITE, Aarhus, Denmark.

[Klaestrup, Ida H.; Lauritsen, Johanne; Romero-Ramos, Marina] Aarhus Univ, Dept Biomed, Aarhus, Denmark.

[Pavese, Nicola] Newcastle Univ, Clin Ageing Res Unit, Newcastle Upon Tyne, Tyne & Wear, United Kingdom.

C3 Aarhus University; Aarhus University; Aarhus University; Newcastle

University - UK

RP Pavese, N (corresponding author), Dept Clin Med Nucl Med & PET, Palle Juul Jensens Blvd 165, DK-8200 Aarhus N, Denmark.

EM npavese@cfin.au.dk

OI Hojholt Terkelsen, Miriam/0000-0003-0971-3747; Pavese,

Nicola/0000-0002-6801-6194; Klaestrup, Ida/0000-0003-2605-3955

FU Health Faculty, Aarhus University; Independent Research Fund, Denmark

FX IHK is supported by a PhD Fellowship from the Health Faculty, Aarhus

University. MHT and VSH are supported by PhD Fellowships from the

Independent Research Fund, Denmark.

CR Ahern GP, 2011, CURR OPIN PHARMACOL, V11, P29, DOI 10.1016/j.coph.2011.02.004

Aho VTE, 2019, EBIOMEDICINE, V44, P691, DOI 10.1016/j.ebiom.2019.05.064

Alam MM, 2022, CELL REP, V38, DOI 10.1016/j.celrep.2021.110090

Andersen KB, 2020, PARKINSONISM RELAT D, V75, P63, DOI 10.1016/j.parkreldis.2020.05.013

Anheim M, 2012, NEUROLOGY, V78, P417, DOI 10.1212/WNL.0b013e318245f476

Arlehamn CSL, 2020, NAT COMMUN, V11, DOI 10.1038/s41467-020-15626-w

Avenali M, 2021, MOVEMENT DISORD, V36, P1267, DOI 10.1002/mds.28496

Banati RB, 2002, GLIA, V40, P206, DOI 10.1002/glia.10144

Berg D, 2021, NAT REV NEUROL, V17, P349, DOI 10.1038/s41582-021-00486-9

Berg D, 2015, MOVEMENT DISORD, V30, P1600, DOI 10.1002/mds.26431

Berg D, 2014, MOVEMENT DISORD, V29, P454, DOI 10.1002/mds.25844

Best L, 2019, CURR NEUROL NEUROSCI, V19, DOI 10.1007/s11910-019-0934-y

Blauwendraat C, 2020, LANCET NEUROL, V19, P170, DOI 10.1016/S1474-4422(19)30287-X

Borghammer P, 2019, J PARKINSON DIS, V9, pS281, DOI 10.3233/JPD-191721

Brochard V, 2009, J CLIN INVEST, V119, P182, DOI 10.1172/JCI36470

Brockmann K, 2017, EUR J NEUROL, V24, P427, DOI 10.1111/ene.13223

Brockmann K, 2016, J NEUROINFLAMM, V13, DOI 10.1186/s12974-016-0588-5

Butkovich LM, 2018, FRONT NEUROSCI-SWITZ, V12, DOI 10.3389/fnins.2018.00626

Campos-Acuña J, 2019, FRONT IMMUNOL, V10, DOI 10.3389/fimmu.2019.00239

Chahine LM, 2013, JAMA NEUROL, V70, P852, DOI 10.1001/jamaneurol.2013.1274

Chavan SS, 2017, J IMMUNOL, V198, P3389, DOI 10.4049/jimmunol.1601613

Cook DA, 2017, NPJ PARKINSONS DIS, V3, DOI 10.1038/s41531-017-0010-8

Craig DW, 2021, NATURE AGING, V1, P734, DOI 10.1038/s43587-021-00088-6

Croisier E, 2005, J NEUROINFLAMM, V2, DOI 10.1186/1742-2094-2-14

De Francesco E, 2021, MOVEMENT DISORD, V36, P225, DOI 10.1002/mds.28137

de Jonge WJ, 2005, NAT IMMUNOL, V6, P844, DOI 10.1038/ni1229

Devos D, 2013, NEUROBIOL DIS, V50, P42, DOI 10.1016/j.nbd.2012.09.007

Doorn KJ, 2014, ACTA NEUROPATHOL COM, V2, DOI 10.1186/s40478-014-0090-1

Drouin-Ouellet J, 2015, INT J NEUROPSYCHOPH, V18, DOI 10.1093/ijnp/pyu103

Dzamko N, 2016, MOVEMENT DISORD, V31, P889, DOI 10.1002/mds.26529

Earls RH, 2020, P NATL ACAD SCI USA, V117, P1762, DOI 10.1073/pnas.1909110117

Emelyanov A, 2021, MOVEMENT DISORD, V36, P1997, DOI 10.1002/mds.28688

Fagerholm SC, 2006, BLOOD, V108, P3379, DOI 10.1182/blood-2006-03-013557

Fan Z, 2015, ALZHEIMERS DEMENT, V11, P608, DOI 10.1016/j.jalz.2014.06.016

Fan Z, 2020, J NEUROINFLAMM, V17, DOI 10.1186/s12974-019-1670-6

Farmen K, 2021, P NATL ACAD SCI USA, V118, DOI 10.1073/pnas.2020858118

Ferreira SA, 2018, FRONT CELL NEUROSCI, V12, DOI 10.3389/fncel.2018.00247

Forsyth CB, 2011, PLOS ONE, V6, DOI 10.1371/journal.pone.0028032

Galiano-Landeira J, 2020, BRAIN, V143, P3717, DOI 10.1093/brain/awaa269

Galper J, 2021, MOVEMENT DISORD, V36, P1451, DOI 10.1002/mds.28525

Gatto EM, 1996, MOVEMENT DISORD, V11, P261, DOI 10.1002/mds.870110308

Geissmann F, 2003, IMMUNITY, V19, P71, DOI 10.1016/S1074-7613(03)00174-2

Gerhard A, 2006, NEUROBIOL DIS, V21, P404, DOI 10.1016/j.nbd.2005.08.002

Ghosh MC, 2003, INT IMMUNOPHARMACOL, V3, P1019, DOI 10.1016/S1567-5769(03)00100-0

Gold A, 2013, MOVEMENT DISORD, V28, P237, DOI 10.1002/mds.25298

Haghshomar M, 2019, NEUROSCIENCE, V403, P70, DOI 10.1016/j.neuroscience.2017.10.050

Hakimi M, 2011, J NEURAL TRANSM, V118, P795, DOI 10.1007/s00702-011-0653-2

Hall S, 2018, SCI REP-UK, V8, DOI 10.1038/s41598-018-31517-z

Hamelin L, 2016, BRAIN, V139, P1252, DOI 10.1093/brain/aww017

Hamza TH, 2010, NAT GENET, V42, P781, DOI 10.1038/ng.642

Harms AS, 2021, ACTA NEUROPATHOL, V141, P527, DOI 10.1007/s00401-021-02268-5

Healy DG, 2008, LANCET NEUROL, V7, P583, DOI 10.1016/S1474-4422(08)70117-0

Heintz-Buschart A, 2018, MOVEMENT DISORD, V33, P88, DOI 10.1002/mds.27105

Heinzel S, 2021, ANN NEUROL, V90, P329, DOI 10.1002/ana.26151

Heinzel S, 2019, MOVEMENT DISORD, V34, P1464, DOI 10.1002/mds.27802

Hilton D, 2014, ACTA NEUROPATHOL, V127, P235, DOI 10.1007/s00401-013-1214-6

Hodo TW, 2020, FRONT IMMUNOL, V11, DOI 10.3389/fimmu.2020.01869

Hoeffel G, 2018, CELL IMMUNOL, V330, P5, DOI 10.1016/j.cellimm.2018.01.001

Horsager J, 2020, BRAIN, V143, P3077, DOI 10.1093/brain/awaa238

Horvath S, 2015, AGING-US, V7, P1130, DOI 10.18632/aging.100859

Hu ZX, 2018, J BIOL REG HOMEOS AG, V32, P687

Hughes CD, 2019, ACTA NEUROPATHOL, V137, P103, DOI 10.1007/s00401-018-1907-y

Hughes LP, 2021, J PARKINSON DIS, V11, P1157, DOI 10.3233/JPD-202508

Hurny A, 2013, FOLIA NEUROPATHOL, V51, P127, DOI 10.5114/fn.2013.35955

Imamura K, 2003, ACTA NEUROPATHOL, V106, P518, DOI 10.1007/s00401-003-0766-2

Jensen MP, 2021, ANN NEUROL, V89, P803, DOI 10.1002/ana.26034

Jiang S, 2017, NEUROL SCI, V38, P1373, DOI 10.1007/s10072-017-2988-4

Jiménez-Jiménez F, 2021, J PERS MED, V11, DOI 10.3390/jpm11090880

Kalia LV, 2015, LANCET, V386, P896, DOI 10.1016/S0140-6736(14)61393-3

Kim C, 2013, NAT COMMUN, V4, DOI 10.1038/ncomms2534

King E, 2017, ALZ DIS ASSOC DIS, V31, P346, DOI 10.1097/WAD.0000000000000211

KLUTER H, 1995, J NEUROL SCI, V133, P134, DOI 10.1016/0022-510X(95)00180-A

Knudsen K, 2018, LANCET NEUROL, V17, P618, DOI 10.1016/S1474-4422(18)30162-5

Kouli A, 2021, J NEUROINFLAMM, V18, DOI 10.1186/s12974-021-02287-9

Kouli A, 2020, ACTA NEUROPATHOL COM, V8, DOI 10.1186/s40478-020-01083-5

Kressel AM, 2020, P NATL ACAD SCI USA, V117, P29803, DOI 10.1073/pnas.2008213117

Kustrimovic N, 2018, J NEUROINFLAMM, V15, DOI 10.1186/s12974-018-1248-8

Kustrimovic N, 2016, SCI REP-UK, V6, DOI 10.1038/srep33738

Lavisse S, 2012, J NEUROSCI, V32, P10809, DOI 10.1523/JNEUROSCI.1487-12.2012

Lebouvier T, 2014, J PARKINSON DIS, V4, P29, DOI 10.3233/JPD-130333

Lin KJ, 2019, INT J MOL SCI, V20, DOI 10.3390/ijms20215312

Liu H, 2016, STROKE VASC NEUROL, V1, P52, DOI 10.1136/svn-2016-000012

Liu SY, 2018, LANCET NEUROL, V17, P309, DOI 10.1016/S1474-4422(18)30032-2

Magistrelli L, 2021, INT J MOL SCI, V22, DOI 10.3390/ijms22073781

Magnusen AF, 2021, FRONT NEUROL, V12, DOI 10.3389/fneur.2021.636139

Mastroeni D, 2009, NEUROBIOL AGING, V30, P1805, DOI 10.1016/j.neurobiolaging.2008.01.001

Matt SM, 2020, J NEUROIMMUNE PHARM, V15, P114, DOI 10.1007/s11481-019-09851-4

Maurer SV, 2017, FRONT IMMUNOL, V8, DOI 10.3389/fimmu.2017.01489

MCGEER PL, 1988, NEUROLOGY, V38, P1285, DOI 10.1212/WNL.38.8.1285

MESULAM MM, 1988, J COMP NEUROL, V275, P216, DOI 10.1002/cne.902750205

Moghaddam HS, 2018, FRONT NEUROL, V9, DOI 10.3389/fneur.2018.00234

Mondello S, 2018, NEUROLOGY, V91, pE1710, DOI 10.1212/WNL.0000000000006439

Mueller B, 2022, NEUROL SCI, V43, P951, DOI 10.1007/s10072-021-05810-1

Mullin S, 2021, MOVEMENT DISORD, V36, P774, DOI 10.1002/mds.28375

Nissen SK, 2022, BRAIN BEHAV IMMUN, V101, P182, DOI 10.1016/j.bbi.2022.01.005

Ouchi Y, 2005, ANN NEUROL, V57, P168, DOI 10.1002/ana.20338

Panicker LM, 2014, STEM CELLS, V32, P2338, DOI 10.1002/stem.1732

Papadopoulos V, 2006, TRENDS PHARMACOL SCI, V27, P402, DOI 10.1016/j.tips.2006.06.005

Papandreou V, 2016, INFLAMMATION, V39, P1069, DOI 10.1007/s10753-016-0338-y

Pinoli M, 2017, J NEUROIMMUNE PHARM, V12, P602, DOI 10.1007/s11481-017-9749-2

Postuma RB, 2015, MOVEMENT DISORD, V30, P1591, DOI 10.1002/mds.26424

Prinz M, 2019, CELL, V179, P292, DOI 10.1016/j.cell.2019.08.053

Rastegar DA, 2019, NPJ PARKINSONS DIS, V5, DOI 10.1038/s41531-019-0086-4

Romano S, 2021, NPJ PARKINSONS DIS, V7, DOI 10.1038/s41531-021-00156-z

Sacramento PM, 2018, EUR J IMMUNOL, V48, P1376, DOI 10.1002/eji.201847525

San Luciano M, 2020, MOVEMENT DISORD, V35, P1755, DOI 10.1002/mds.28189

Sarkar C, 2010, BRAIN BEHAV IMMUN, V24, P525, DOI 10.1016/j.bbi.2009.10.015

Scanzano A, 2015, FRONT PHARMACOL, V6, DOI 10.3389/fphar.2015.00171

Scott KM, 2018, FRONT NEUROL, V9, DOI 10.3389/fneur.2018.00815

Shannon KM, 2012, MOVEMENT DISORD, V27, P716, DOI 10.1002/mds.25020

Simuni T, 2020, LANCET NEUROL, V19, P71, DOI 10.1016/S1474-4422(19)30319-9

Sommer A, 2018, CELL STEM CELL, V23, P123, DOI 10.1016/j.stem.2018.06.015

Staer K IA, 2020, MOV DISORD, V35, P283

Stær K, 2020, PARKINSONISM RELAT D, V81, P89, DOI 10.1016/j.parkreldis.2020.10.014

Stokholm MG, 2020, J NEUROL, V267, P2296, DOI 10.1007/s00415-020-09830-3

Stokholm MG, 2018, NEUROBIOL DIS, V115, P9, DOI 10.1016/j.nbd.2018.02.017

Stokholm MG, 2017, LANCET NEUROL, V16, P789, DOI 10.1016/S1474-4422(17)30173-4

Stolzenberg E, 2017, J INNATE IMMUN, V9, P456, DOI 10.1159/000477990

Sulzer D, 2017, NATURE, V546, P656, DOI 10.1038/nature22815

Thaler A, 2021, J PARKINSON DIS, V11, P1285, DOI 10.3233/JPD-212624

Thome AD, 2021, NPJ PARKINSONS DIS, V7, DOI 10.1038/s41531-021-00188-5

Trabold B, 2010, INJURY, V41, P1079, DOI 10.1016/j.injury.2010.05.034

Turkheimer FE, 2015, BIOCHEM SOC T, V43, P586, DOI 10.1042/BST20150058

Umehara T, 2020, J NEUROL SCI, V409, DOI 10.1016/j.jns.2019.116638

Usenko T, 2021, GENES-BASEL, V12, DOI 10.3390/genes12101545

Vida C, 2019, INT J MOL SCI, V20, DOI 10.3390/ijms20030771

Vitte J, 2004, J CLIN IMMUNOL, V24, P683, DOI 10.1007/s10875-004-6243-4

von Herrmann KM, 2018, NPJ PARKINSON DIS, V4, DOI 10.1038/s41531-018-0061-5

Wallings RL, 2020, FRONT NEUROSCI-SWITZ, V14, DOI 10.3389/fnins.2020.00443

Wang H, 2003, NATURE, V421, P384, DOI 10.1038/nature01339

Wang PP, 2021, CELL DISCOV, V7, DOI 10.1038/s41421-021-00280-3

Wijeyekoon RS, 2020, BRAIN BEHAV IMMUN, V87, P473, DOI 10.1016/j.bbi.2020.01.018

Wile DJ, 2017, LANCET NEUROL, V16, P351, DOI 10.1016/S1474-4422(17)30056-X

Williams-Gray CH, 2018, J NEUROINFLAMM, V15, DOI 10.1186/s12974-018-1206-5

Yan YQ, 2015, CELL, V160, P62, DOI 10.1016/j.cell.2014.11.047

Yan ZQ, 2021, NEUROL-NEUROIMMUNOL, V8, DOI 10.1212/NXI.0000000000001036

Yazdani S, 2019, ANN NEUROL, V86, P913, DOI 10.1002/ana.25614

Yoo BB, 2017, IMMUNITY, V46, P910, DOI 10.1016/j.immuni.2017.05.011

Yu BN, 2008, J PHARMACOL EXP THER, V327, P316, DOI 10.1124/jpet.108.143461

Zhang H, 2020, PARKINSONISM RELAT D, V78, P145, DOI 10.1016/j.parkreldis.2020.07.017

NR 140

TC 20

Z9 20

U1 3

U2 13

PU IOS PRESS

PI AMSTERDAM

PA NIEUWE HEMWEG 6B, 1013 BG AMSTERDAM, NETHERLANDS

SN 1877-7171

EI 1877-718X

J9 J PARKINSON DIS

JI J. Parkinsons Dis.

PY 2022

VL 12

SU 1

BP S149

EP S163

DI 10.3233/JPD-223245

PG 15

WC Neurosciences

WE Science Citation Index Expanded (SCI-EXPANDED)

SC Neurosciences & Neurology

GA 5E2PZ

UT WOS:000865470900015

PM 35723115

OA Green Published, hybrid

DA 2024-08-12

ER

PT J

AU Sandor, C

Millin, S

Dahl, A

Schalkamp, AK

Lawton, M

Hubbard, L

Rahman, N

Williams, N

Ben-Shlomo, Y

Grosset, DG

Hu, MT

Marchini, J

Webber, C

AF Sandor, Cynthia

Millin, Stephanie

Dahl, Andrew

Schalkamp, Ann-Kathrin

Lawton, Michael

Hubbard, Leon

Rahman, Nabila

Williams, Nigel

Ben-Shlomo, Yoav

Grosset, Donald G.

Hu, Michele T.

Marchini, Jonathan

Webber, Caleb

TI Universal clinical Parkinson's disease axes identify a major influence

of neuroinflammation

SO GENOME MEDICINE

LA English

DT Article

DE Parkinson's disease; Deeply phenotyped cohorts; Neuro-inflammation

ID NONMOTOR FEATURES; PROGRESSION; BIOMARKERS; MOTOR; DEMENTIA; SUBTYPES;

LEWY

AB Background: There is large individual variation in both clinical presentation and progression between Parkinson's disease patients. Generation of deeply and longitudinally phenotyped patient cohorts has enormous potential to identify disease subtypes for prognosis and therapeutic targeting.

Methods: Replicating across three large Parkinson's cohorts (Oxford Discovery cohort (n = 842)/Tracking UK Parkinson's study (n = 1807) and Parkinson's Progression Markers Initiative (n = 472)) with clinical observational measures collected longitudinally over 5-10 years, we developed a Bayesian multiple phenotypes mixed model incorporating genetic relationships between individuals able to explain many diverse clinical measurements as a smaller number of continuous underlying factors ("phenotypic axes").

Results: When applied to disease severity at diagnosis, the most influential of three phenotypic axes "Axis 1" was characterised by severe non-tremor motor phenotype, anxiety and depression at diagnosis, accompanied by faster progression in cognitive function measures. Axis 1 was associated with increased genetic risk of Alzheimer's disease and reduced CSF A beta 1-42 levels. As observed previously for Alzheimer's disease genetic risk, and in contrast to Parkinson's disease genetic risk, the loci influencing Axis 1 were associated with microglia-expressed genes implicating neuroinflammation. When applied to measures of disease progression for each individual, integration of Alzheimer's disease genetic loci haplotypes improved the accuracy of progression modelling, while integrating Parkinson's disease genetics did not.

Conclusions: We identify universal axes of Parkinson's disease phenotypic variation which reveal that Parkinson's patients with high concomitant genetic risk for Alzheimer's disease are more likely to present with severe motor and non-motor features at baseline and progress more rapidly to early dementia.

C1 [Sandor, Cynthia; Schalkamp, Ann-Kathrin; Rahman, Nabila; Webber, Caleb] Cardiff Univ, UK Dementia Res Inst, Cardiff CF24 4HQ, United Kingdom.

[Millin, Stephanie; Webber, Caleb] Univ Oxford, Dept Physiol Anat & Genet, Oxford OX1 3PT, United Kingdom.

[Dahl, Andrew; Marchini, Jonathan] Univ Oxford, Wellcome Ctr Human Genet, Oxford OX3 7BN, United Kingdom.

[Lawton, Michael; Ben-Shlomo, Yoav] Univ Bristol, Sch Social & Community Med, Bristol BS8 1TH, Avon, United Kingdom.

[Hubbard, Leon; Williams, Nigel] Cardiff Univ, MRC Ctr Neuropsychiat Genet & Genom, Sch Med, Inst Psychol Med & Clin Neurosci, Cardiff CF24 4HQ, United Kingdom.

[Grosset, Donald G.] Queen Elizabeth Univ Hosp, Dept Neurol, Inst Neurol Sci, Glasgow G51 4LB, Lanark, United Kingdom.

[Hu, Michele T.] Univ Oxford, Oxford Parkinsons Dis Ctr, Dept Physiol Anat & Genet, Le Gros Clark Bldg, Oxford OX1 3PT, United Kingdom.

[Hu, Michele T.] Univ Oxford, Nuffield Dept Clin Neurosci, Div Clin Neurol, Oxford OX3 7LF, United Kingdom.

[Marchini, Jonathan] Univ Oxford, Dept Stat, Oxford OX1, United Kingdom.

[Marchini, Jonathan] Regeneron Genet Ctr, Tarrytown, NY USA.

C3 Cardiff University; University of Oxford; University of Oxford; Wellcome

Centre for Human Genetics; University of Bristol; Cardiff University;

University of Glasgow; Queen Elizabeth University Hospital (QEUH);

University of Oxford; University of Oxford; University of Oxford;

Regeneron

RP Sandor, C; Webber, C (corresponding author), Cardiff Univ, UK Dementia Res Inst, Cardiff CF24 4HQ, United Kingdom.; Webber, C (corresponding author), Univ Oxford, Dept Physiol Anat & Genet, Oxford OX1 3PT, United Kingdom.

EM sandorc@cardiff.ac.uk; donaldgrosset@gmail.com;

michele.hu@ndcn.ox.ac.uk; webberc4@cardiff.ac.uk

RI Ben-Shlomo, Yoav/ABD-2004-2021

OI Ben-Shlomo, Yoav/0000-0001-6648-3007; Lawton,

Michael/0000-0002-3419-0354; Sandor, Cynthia/0000-0002-8905-1052

FU Monument Trust Discovery Award from Parkinson's UK; Oxford Genomics

Centre at the Wellcome Centre for Human Genetics; Wellcome Trust

[090532/Z/09/Z]; MRC Hub grant [G0900747 91070]; National Institute for

Health Research (NIHR) Oxford Biomedical Research Centre (BRC); UK

Dementia Research Institute, DRI Ltd - UK Medical Research Council;

Alzheimer's Society; Alzheimer's Research; Computational Science Program

- Michael J. Fox Foundation; UK Dementia Research Institute (UK DRI) -

Medical Research Council (MRC); Alzheimer's Research UK (AR-UK); Ser

Cymru II programme - Cardiff University; European Regional Development

Fund through the Welsh Government; Heath and Cares Research United Kingdom;

European Research Council (ERC) [617306]

FX This work was supported by the Monument Trust Discovery Award from

Parkinson's UK, the Oxford Genomics Centre at the Wellcome Centre for

Human Genetics, the Wellcome Trust (grant reference 090532/Z/09/Z) and

an MRC Hub grant G0900747 91070). Samples and associated clinical data

were supplied by the Oxford Parkinson's Disease Centre (OPDC) study,

funded by the Monument Trust Discovery Award from Parkinson's UK, with

the support of the National Institute for Health Research (NIHR) Oxford

Biomedical Research Centre (BRC). CW's lab is supported by the UK

Dementia Research Institute, which receives its funding from DRI Ltd,
[truncated: 36,309,708 more chars]
